# PINK1 deficiency rewires early immune responses in a mouse model of Parkinson’s disease triggered by intestinal infection

**DOI:** 10.1038/s41531-025-00945-w

**Published:** 2025-05-22

**Authors:** Sherilyn Junelle Recinto, Alexandra Kazanova, Lin Liu, Brendan Cordeiro, Shobina Premachandran, Hicham Bessaiah, Alexis Allot, Elia Afanasiev, Sriparna Mukherjee, Jessica Pei, Adam MacDonald, Moein Yaqubi, Heidi M. McBride, Diana Matheoud, Louis-Eric Trudeau, Samantha Gruenheid, Jo Anne Stratton

**Affiliations:** 1 Department of Neurology and Neurosurgery, Montreal Neurological Institute-Hospital, McGill University, Montréal, QC Canada; 2 Aligning Science Across Parkinson’s (ASAP) Collaborative Research Network, Chevy Chase, MD 20815 USA; 3 Department of Microbiology and Immunology, McGill University, Montréal, QC Canada; 4 Department of Neuroscience, Faculty of Medicine, Université de Montréal, Montréal, QC Canada; 5 Department of Pharmacology and Physiology, Faculty of Medicine, Université de Montréal, Montréal, QC Canada

**Keywords:** Inflammation, Parkinson's disease, Antigen processing and presentation

## Abstract

Parkinson’s disease is characterized by a period of non-motor symptoms, including gastrointestinal dysfunction, preceding motor deficits by several years to decades. This long prodrome is suggestive of peripheral immunity involvement in the initiation of disease. We previously developed a model system in PINK1 KO mice displaying PD-like motor symptoms at late stages following intestinal infections. Herein, we map the initiating immune events at the site of infection in this model. Using single-cell RNAseq, we demonstrate that peripheral myeloid cells are the earliest highly dysregulated immune cell type followed by an aberrant T cell response shortly after. We also demonstrate an increased propensity for antigen presentation and that activated myeloid cells acquire a proinflammatory profile capable of inducing cytotoxic T cell responses. Together, our study provides the first evidence that PINK1 is a key regulator of immune functions in the gut underlying early PD-related disease mechanisms.

## Introduction

Parkinson’s disease (PD) is a highly prevalent, age-associated progressive neurodegenerative disorder that is usually diagnosed upon the appearance of cardinal motor functional deficits such as bradykinesia, tremor, and rigidity. The motor symptoms are caused in part by the loss of dopamine (DA) neurons in the substantia nigra pars compacta of the midbrain and it is estimated that by the time of diagnosis, 60–80% of nigral DA neurons are already irrevocably lost, making treatment of PD challenging^1^. Nevertheless, it is now widely recognized that motor symptom development can be preceded by a long prodromal phase where non-motor symptoms, such as hyposmia, constipation, sleep disruption and anxiety, may be present^2^. The extensive prodromal period suggests that disease initiating mechanisms may occur early life and remain unnoticed until the appearance of motor symptoms in older age. Understanding the pathophysiology of the prodromal phase of PD offers promise for earlier detection and treatment.

Peripheral inflammation and immune dysfunction have been implicated in PD onset, including the emergence of various non-motor symptoms, such as gastrointestinal (GI) dysfunction^3^. Analyses of immune profiles in the stools of PD patients demonstrate higher levels of inflammatory mediators suggestive of GI inflammation^4–8^. Clinical studies further reveal that people with inflammatory bowel disease (IBD) and GI infections have an increased risk of developing PD later in life^4,9–13^. Other studies also underscore common genetic variants between patients with PD and immune disorders, such as IBD^14,15^. Intriguingly, evidence suggests that multiple genes implicated in familial forms of PD as well as several PD risk variants are expressed in immune cells and function in the regulation of immune responses.

Of interest, PTEN-induced kinase 1 (PINK1), a serine/threonine kinase, and Parkin (PRKN), an E3 ubiquitin ligase, both associated with familial forms of PD, have been shown to act as repressors of innate immune responses independently from their previously described roles in mitophagy. A loss of function of either of these two proteins disinhibits the formation of mitochondrial-derived vesicles (MDVs) in antigen-presenting cells leading to the presentation of mitochondrial peptides via major histocompatibility complex I (MHCI), a process referred to as mitochondrial antigen presentation (MitAP)^16^. MitAP leads to the establishment of mitochondrial antigen-specific T cells, implicating T cell autoimmunity in PD pathogenesis. Subsequently, others have also proposed that PINK1 modulates immune responses triggered by oxidative stress and viral infection^17–20^.

In accordance with this, intestinal infection with Gram-negative bacteria (Citrobacter rodentium) in PINK1 knockout (KO) mice was able to induce antigen-specific T-cell autoreactivity and increased entry of immune cells into the brain^21^. Notably, with aging and repeated infections, these mice developed L-DOPA reversible PD-like motor impairments. A similar phenomenon was also recently demonstrated using a major PD-associated human-relevant pathogen (Helicobacter pylori), where autoreactive CD8 + T-cell-dependent PD-like motor phenotypes developed following GI infection of PINK1 KO mice^22^. Additionally, adoptive transfer of mitochondrial antigen-specific CD8 + T cells was shown to be sufficient to induce motor impairments and DA neuron loss in both PINK1 KO and wild-type mice, supporting a critical and direct role for autoreactive CD8 + T cells in causing neuronal loss under such conditions^23^. Taken together, these data provide proof of concept that peripheral infections can lead to aberrant immunity engendering parkinsonism in genetically predisposed models of PD.

Critical questions remain, in particular regarding the mechanisms at play in the periphery at the earliest prodromal stages of the disease process. Understanding the immune mechanisms that initiate disease early in the periphery at the cell/molecular level will address a major knowledge gap that hinders the development of diagnostic and therapeutic approaches to stop the onset of impairment at early stages of disease before irreversible neuron death ensues. Here, we sought to provide an in-depth characterisation of the initiating immunological events in the gut mediated by PINK1 deficiency. We find that intestinal infection in PINK1 KO mice induces early immune dysregulation in the colon marked by the promotion of proinflammatory myeloid cell differentiation with an increased capacity to promote T-cell cytotoxic profiles. Further in-vitro findings demonstrate that PINK1 loss in myeloid cells following lipopolysaccharide stimulation can elicit more potent CD8 + T cell responses in both wild type and PINK1 KO CD8 + T cells. Collectively, our study deepens our knowledge of the role of PD-associated genes in the regulation of peripheral immunity, providing an opportunity for the development of biomarkers and therapeutic approaches.

## Results

### Single-cell RNAseq of the colon unveils a role for PINK1 within the immune cell compartment at the earliest stages following infection

We have previously described that repeated infection of PINK1 KO mice with mouse intestinal Gram-negative pathogen (C. rodentium) via gavage induces L-DOPA reversible motor symptoms 16-24 weeks later^21^. Despite clear evidence for the role of autoreactive CD8 + T cells in the neurodegenerative process within the brain^22,23^, it is unknown how this is instigated. The phases of C. rodentium infection subsequent to its establishment in the colon are well described, and include bacterial expansion (by 1 week), steady-state (1–2 weeks) and clearance (2+ weeks). This immune response is governed by several players of innate and adaptive immunity, including early myeloid cell responses driven by dendritic cells then ultimately the engagement of type 1 and interleukin 17-producing T helper cells (Th1 and Th17, respectively) for complete clearance of the infection^24^.

**Fig. 1:**
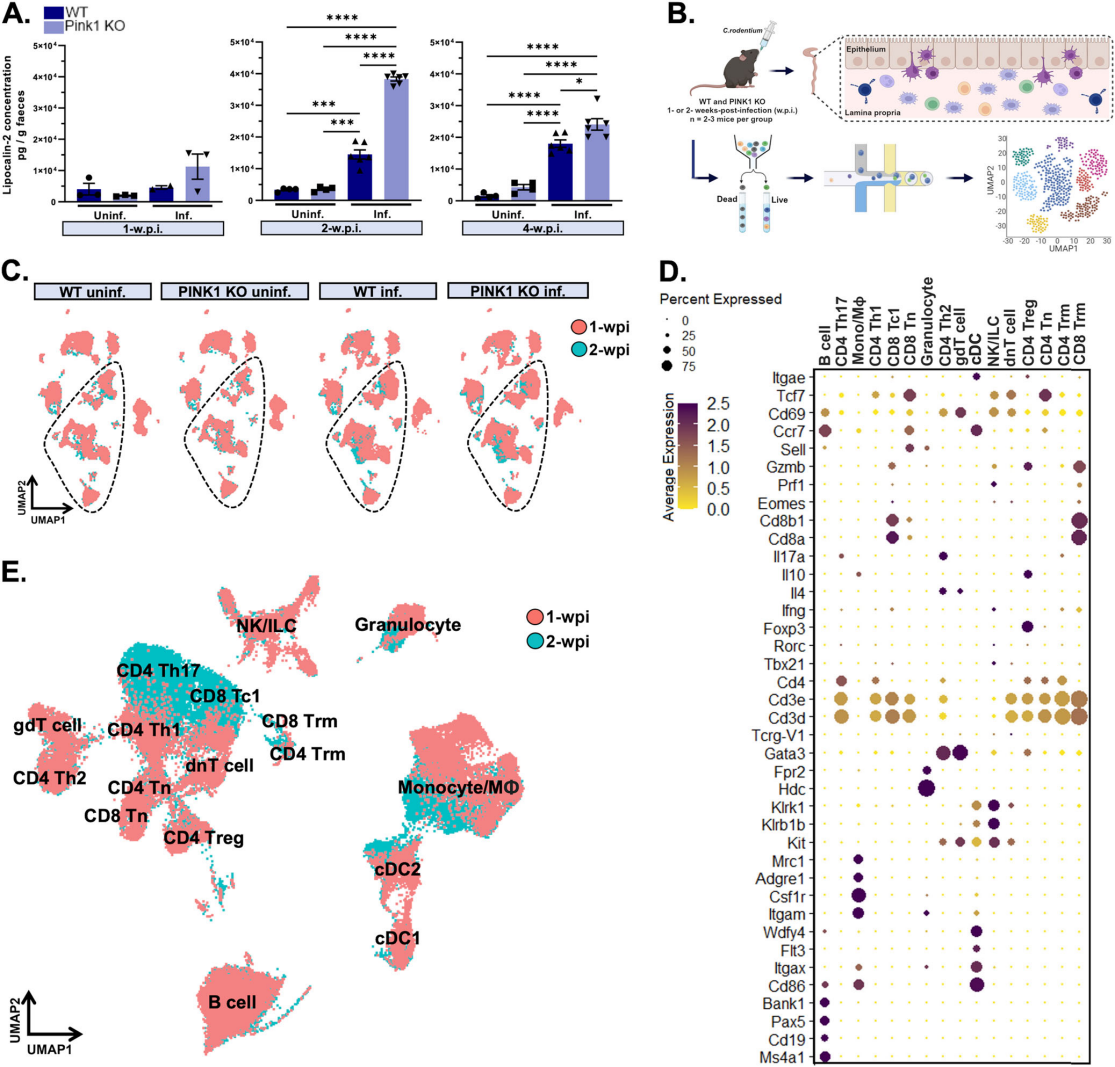
Single-cell transcriptomics of the colon in an infection-induced model of PD. **A** Lipocalin-2 levels were measured in faecal samples of WT and PINK1 KO mice at 1-, 2- and 4 w.p.i. and uninf. controls. Two-way ANOVA followed by Tukey’s multiple comparison test was applied. Mean ± SEM, *n* = 2-6 mice per group. **B** A schematic diagram depicts intestinal infection of WT and PINK1 KO mice via gavage at 1- and 2 w.p.i. followed by 3’scRNAseq of the colon from 2-3 mice per group. **C** Multiple cell types identified in the gut of WT and PINK1 KO inf. mice and uninf. control groups at 1- and 2 w.p.i. (in red and blue, respectively) are projected in 2D UMAP plots. Each scRNAseq dataset comprises ~40,000 cells and ~20,000 genes detected. **D** Dotplot indicates the cell identity marker genes curated from the literature used to annotate immune cells in the scRNAseq dataset. **E** Merged UMAP plots of immune cell types across all groups at both timepoints. Weeks post-infection, (w.p.i.), wild type (WT), knockout (KO), single-cell RNAsequencing (scRNAseq), infected (inf), uninfected (uninf), uniform manifold approximation and projection (UMAP).

We first employed an indirect, non-invasive approach using faecal analysis to evaluate whether there was any evidence for enhanced gut inflammation in PINK1 KO mice at different time points after infection. We used enzyme-linked immunosorbent assay (ELISA) to detect lipocalin-2 (LCN2) and calprotectin, which are highly expressed by activated innate immune/epithelial cells, and often used as indicators of intestinal inflammation^5,6,25,26^. We noted a progressive increase in the concentration of inflammatory markers over time which reached significance in wild type and PINK1 KO mice at 2- and 4 weeks post infection (w.p.i.) compared to uninfected mice. Importantly, there was significantly more LCN2 in PINK1 KO infected mice compared to wild type infected mice at these time points (Fig. 1A). Similar genotype-dependent increases were observed with calprotectin at 2 w.p.i. (Supplementary Fig. 1A). Additionally, we interrogated gut function in PINK1 KO mice after infection, as constipation is one of the earliest non-motor symptoms experienced by PD patients^2^. We discovered that PINK1 KO mice often exhibited lower faecal water content and/or reduced stool frequency at 2 w.p.i. compared to wild type infected mice, suggestive of exacerbated GI dysfunction (Supplementary Fig. 1B). It is noteworthy that the constipation phenotype is highly variable across cohorts albeit the reason for this is unclear.

**Fig. 2:**
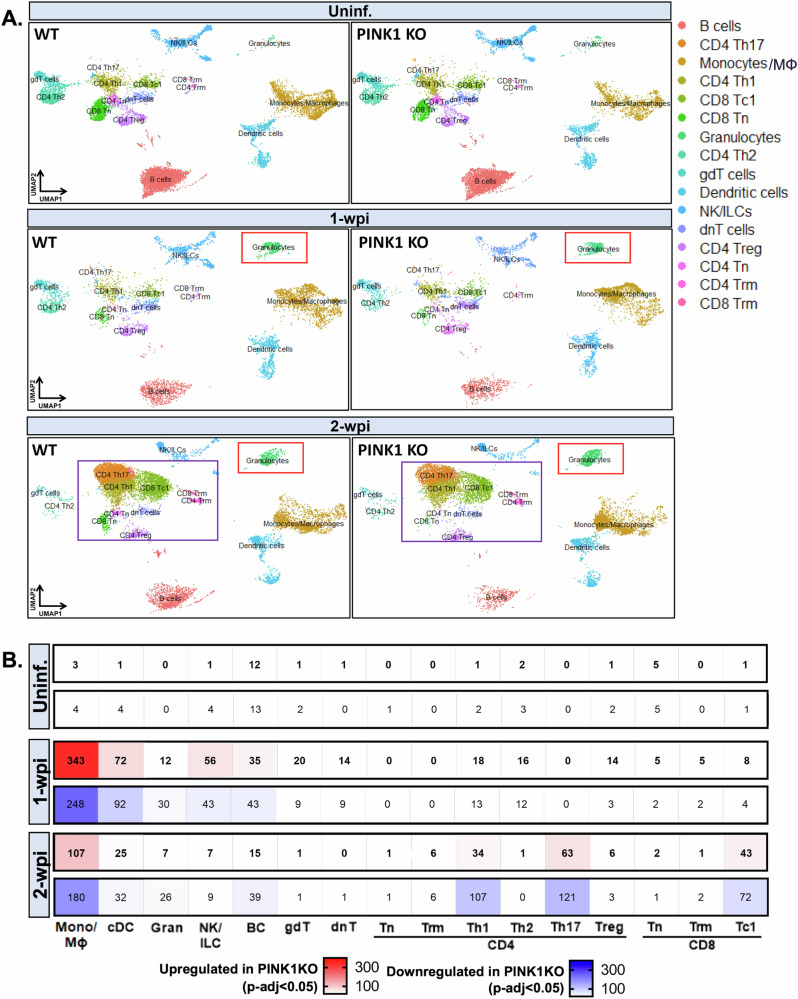
Differential gene expression analyses of the immune compartment in the colon of an infection-induced PD model. **A** UMAP plots of immune cell types in the gut segregated by genotype and timepoint. Note cell populations (in boxes) representing a clear expansion of immune cells in both genotypes following infection. **B** Each heatmap illustrates the number of significant DEGs in each immune cell types of PINK1 KO compared to WT mice at uninf., 1- and 2 w.p.i. (up-and down-regulated denoted as red and blue, respectively). To test for significance, Wilcoxon Rank Sum test was applied, with adjusted *p*-value < 0.05 using Bonferroni correction. Helper T cells (Th), cytotoxic 1 T cells (Tc1), naïve T cells (Tn), gamma delta T cells (gdT), natural killer (NK), innate lymphoid cell (ILC), double-negative T cells (dnT), regulatory T cells (Treg), tissue resident memory (Trm), differentially expressed genes (DEGs).

To gain insights into PINK1-mediated alterations in the intestinal immune landscape, we then performed single-cell RNAseq using 10X droplet-based microfluidics technology in FACS-sorted whole colonic lamina propria cells (Fig. 1B). Briefly, we prepared gene expression libraries from wild type and PINK1 KO infected mice along with uninfected control groups at 1- and 2 w.p.i. (Fig. 1C). Subsequent to quality control of our datasets employing standard parameters in Seurat^27^, we annotated all immune cell types using identity markers curated from the literature for each cell cluster (Fig. 1D, E). Cell populations were further defined by sets of genes displayed in Supplementary Table 1. By comparing wild type and PINK1 KO datasets in each distinct condition (uninfected control, 1 w.p.i. and 2 w.p.i.), we determined transcriptional regulation driven by PINK1 in specific immune cell types in response to intestinal infection. As shown in Fig. 2A, we did not observe obvious genotype-dependent alterations in cell cluster identities in each condition, although as expected, we noticed a larger population of granulocytes at 1- and 2 w.p.i. in both genotypes compared to the uninfected groups. Meanwhile, CD4+ and CD8 + T cell subtypes became more prominent at 2 w.p.i. in both genotypes. These changes are consistent with the involvement of innate and adaptive immunity in bacterial clearance at these timepoints, as expected^24^. Next, we performed differential gene expression analyses between PINK1 KO and wild type mice. The heatmap illustrates the number of genes that were either significantly upregulated or downregulated in each immune cell type of PINK1 KO mice compared to wild type (adjusted *p*-values < 0.05 using non-parametric Wilcoxon rank sum test with Bonferroni correction applied to evaluate significance) with the most substantial changes occurring in the myeloid cell compartment (Fig. 2B). We found extensive transcriptional regulation in monocytes/macrophages and dendritic cells at 1-w.p.i., followed by CD4 + Th1 and Th17 as well as CD8+ cytotoxic T cells (Tc1) at 2 w.p.i. Further exploration of this dataset can be performed here: https://singlocell.openscience.mcgill.ca/display?dataset=RNA_Ms_Gut_Immune_PD_2024.

### Higher proportions of effector T cells depicting proinflammatory and cytotoxic characteristics are present at 2-weeks post-infection in the colon of PINK1 KO mice

Effector CD4+ and CD8 + T cell subtypes that appear to be more abundant in peripheral blood, cerebrospinal fluid and brains of PD patients are now widely being investigated in PD due to their proinflammatory and cytotoxic profiles^28–36^. Previous mouse findings also point to the proliferation of a mitochondrial-antigen specific CD8 + T cell subset as a prerequisite for driving central nervous system (CNS)-related pathological processes via antigen presentation by dendritic cells in the spleen^16,21,22^. However, it is unknown if PINK1 loss also potentiates the recruitment or expansion of effector T cells and alters their expression profile in the gut at the earliest stages of disease.

**Fig. 3:**
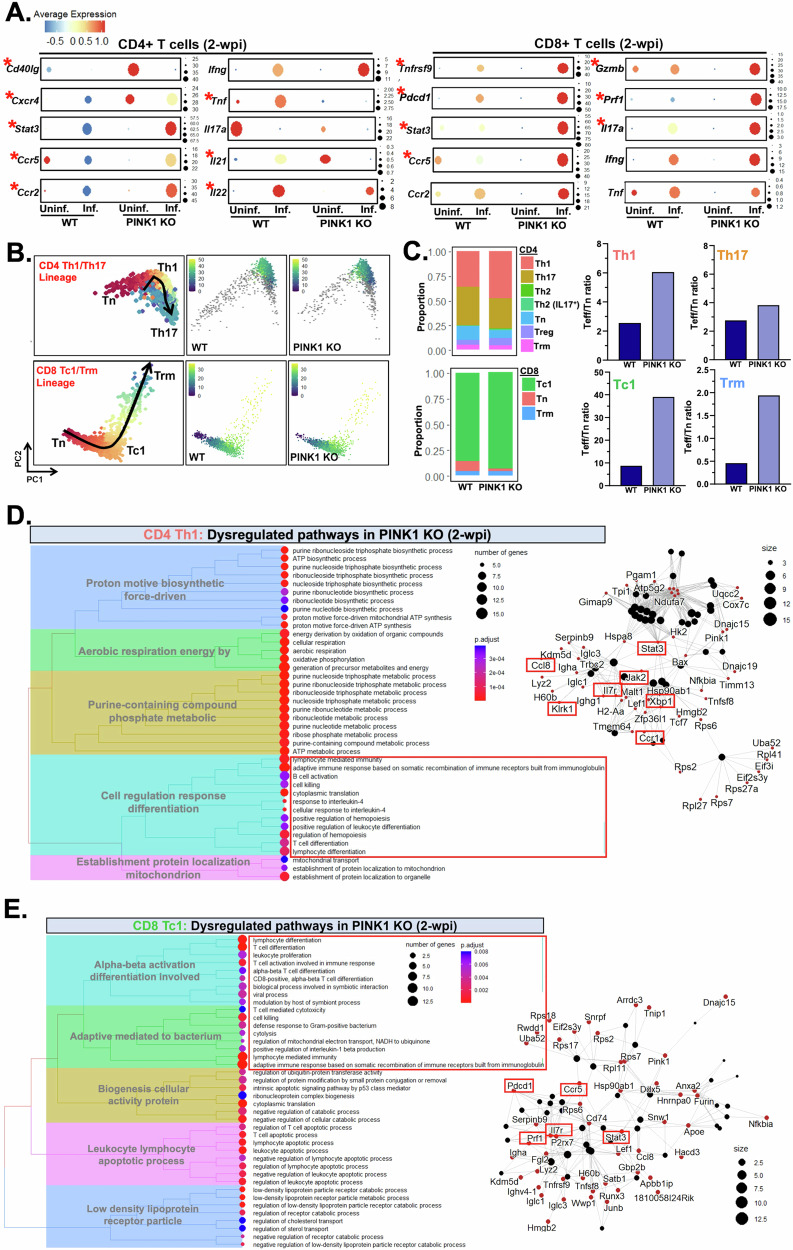
An expansion of proinflammatory and cytotoxic effector T cell subtypes in the gut of PINK1 KO mice following infection. **A** Dotplots indicate expression of known canonical T cell activation markers and T-cell cytokines as well as cytotoxicity-related genes across all conditions at 2 w.p.i. Significantly upregulated genes in T cells of PINK1 KO infected mice compared to WT are denoted with an asterisk. Kruskal-Wallis followed by Dunn’s multiple comparison test was applied. **B** Pseudotime analyses of CD4+ and CD8 + T cell subtypes revealed Th1 to Th17, and Tc1 to Trm lineage transitions from naive states. Both lineages are illustrated in principal component analysis (PCA) plots of intestinal T cell populations from WT and PINK1 KO infected mice. **C** The proportions of each CD4+ and CD8 + T cell subtypes were assessed and the ratios of effector-to-naïve T cells (Teff/Tn) in PINK1 KO compared to WT are presented. **D**, **E** Pathway enrichment analyses were run on significant DEGs in PINK1 KO CD4 + Th1 and CD8 + Tc1 cells using gene ontology terms of biological processes in ClusterProfiler. Dysregulated pathways are shown as treeplots while cnetplots display the genes involved, in which the biological processes and associated genes underlying the activation of adaptive immunity are highlighted in rectangles. Tn naive T cell, Tc1 cytotoxic T cells, Trm Resident memory cells, Cd40lg CD40 ligand, Cxcr4 C-X-C chemokine receptor type 4, Stat3 signal transducer activator of transcription 3, Ccr2/5 chemokine receptors 2 and 5, Tnfrsf9 TNF receptor superfamily member 9, Pdcd1 programmed cell death protein 1, Ifng interferon gamma, Tnf tumour necrosis factor, Il17a/Il21/Il22 interleukins, Gzmb granzyme B, Prf1 Perforin.

To address this, we examined the features of CD4+ and CD8 + T cells at peak expansion by interogating differentially expressed genes and performing trajectory analyses at 2 w.p.i (Fig. 3). Using known canonical markers of T cell activation^37–47^, we deciphered genotype-dependent transcriptomic alterations in specific chemokine receptors and proinflammatory proteins/cytokines. Notably, both CD4+ and CD8+ lymphocyte populations of infected PINK1 KO mice compared to WT mice have significant elevated expression of chemokine receptor CCR5 and signal transducer and activator of transcription 3 (STAT3), suggestive of more potent immune responses. Similarly, significantly higher levels of cytotoxicity-related genes, such as interleukin-17a (Il17a), granzyme B (Gzmb) and perforin (Prf1), as well as activation-induced markers including TNF receptor superfamily member 9 (Tnfrsf9) and programmed cell death protein 1 (Pdcd1) were detected in PINK1 KO CD8 + T cells at 2 w.p.i. Of note, we also observed a trend for increased expression of chemokine receptor CCR2 and interferon gamma (Ifng) in PINK1 KO CD8+ T cells. However, amongst proinflammatory cytokines assessed here, only Ifng expression showed an increasing trend in PINK1 KO CD4+ T cells while others were either unchanged or lower compared to wild type (Fig. 3A).

By employing Slingshot^48^, we further identified distinct T cell trajectories based on gene expression changes generated by unsupervised hierarchal clustering. Depending on the position in a given trajectory, a pseudotime value is assigned to a cell with increasing number. The higher the number, the further it is from the origin of the lineage which, in this case, was defined as naïve T cells (Tn). In line with previous reports underlining the plasticity of CD4 + T cells in transdifferentiating between subtypes modulated by extracellular cues and intrinsic signalling^49^, we found four lineages starting from naive T cells towards different effector T cell subsets: (1) Tn to T cell helper type 1 (Th1) to helper type 2 (Th2) and IL17-producing Th2 cells; (2) Tn to Th1 to resident memory cells (Trm); (3) Tn to Th1 to Th17 cells; and (4) Tn to Treg (data not shown). Considering that Th1 and Th17 gene expression was most regulated within the CD4 + T cell populations (Fig. 2B), we further probed this lineage transition to examine the frequency of these proinflammatory effector T cells (Fig. 3B, upper panel). PINK1 KO infected mice exhibited higher Tn to effector T cell transitions for both Th1 and Th17 compared to wild type. The genotype-dependent difference in the Th1 transition was most evident with a three-fold higher ratio of Th1/Tn (Fig. 3C, upper panel). Pseudotime analyses of CD8 + T cell population revealed a single trajectory from Tn to cytotoxic CD8 Tc1 to Trm cells (Fig. 3B, lower panel). Proportional analyses demonstrated that CD8 + T cell differentiation was greater in infected PINK1 KO with a four-fold increase in Tc1/Tn and Trm/Tn ratios compared to wild type (Fig. 3C, lower panel). We also performed flow cytometric staining of CD4+ and CD8 + T cells in the gut of wild type and PINK1 KO mice following infection. Naïve and effector T cells were identified based on high expression of cell surface markers, CD62L and CD44, respectively. We found a significantly higher ratio of overall effector/naïve CD4+ and CD8 + T cells in PINK1 KO mice at 2 w.p.i. compared to uninfected PINK1 KO mice, which was not apparent in wild type infected mice at 2 w.p.i (Supplementary Fig. 2A, B). Moreover, upon evaluating specific CD4 + T cell subpopulations, we also uncovered increases in Th1 and Th17 cells, defined based on the expression of transcription factors, T-bet and Rorγt, respectively (Supplementary Fig. 2C).

Given the high degree of gene regulation in effector T cell subpopulations (Th1, Th17 and Tc1) in infected PINK1 KO mice (Fig. 2B), we conducted pathway enrichment analyses using Gene Ontology (GO) terms to determine the top biological processes that were associated with significant differentially expressed genes in each of these T cell subtypes. A large number of GO terms were conserved across T cell types which were related to the activation of adaptive immunity (Fig. 3D, E). Amongst them were T cell differentiation, cellular response to interleukin-4 (which is imperative in shaping T cell responses), leucocyte cell-cell adhesion, T cell-mediated cytotoxicity and cell killing. Some DEGs that were driving these GO terms (Fig. 3D, E; Supplementary Table 2) include activation/suppressive markers such as Stat3, Xbp1, Cd40lg, Il7r, Pdcd1, as well as genes encoding tumour necrosis factor (TNF) ligand superfamilies (Tnfrsf4, Tnfsf8), C-C chemokine receptors and cytokines (Ccr1, Ccr2, Ccr5, Ccl8) and genes involved in cytotoxic T cell function (Klrk1, Klrc1, Prf1). This data suggests that PINK1-mediated immune dysregulation in the gut contributes to the expansion of proinflammatory and cytotoxic effector T cell subtypes early in disease.

### Proinflammatory myeloid cells with enhanced antigen presentation capacity are present at 1-week post-infection in the colon of PINK1 KO mice

Antigen-presenting cells (APCs) are pivotal for shaping T cell lineage commitment and instructing adaptive immunity. We speculated that PINK1 influences the capacity of myeloid cells to skew T-cells and present antigens at the earliest stages in our model. Importantly, understanding the role of myeloid cell populations early in the disease process has garnered interest due to studies demonstrating abnormal signatures of monocytes in prodromal PD, such as in REM-sleep behaviour disorder (RBD)^50–52^.

**Fig. 4:**
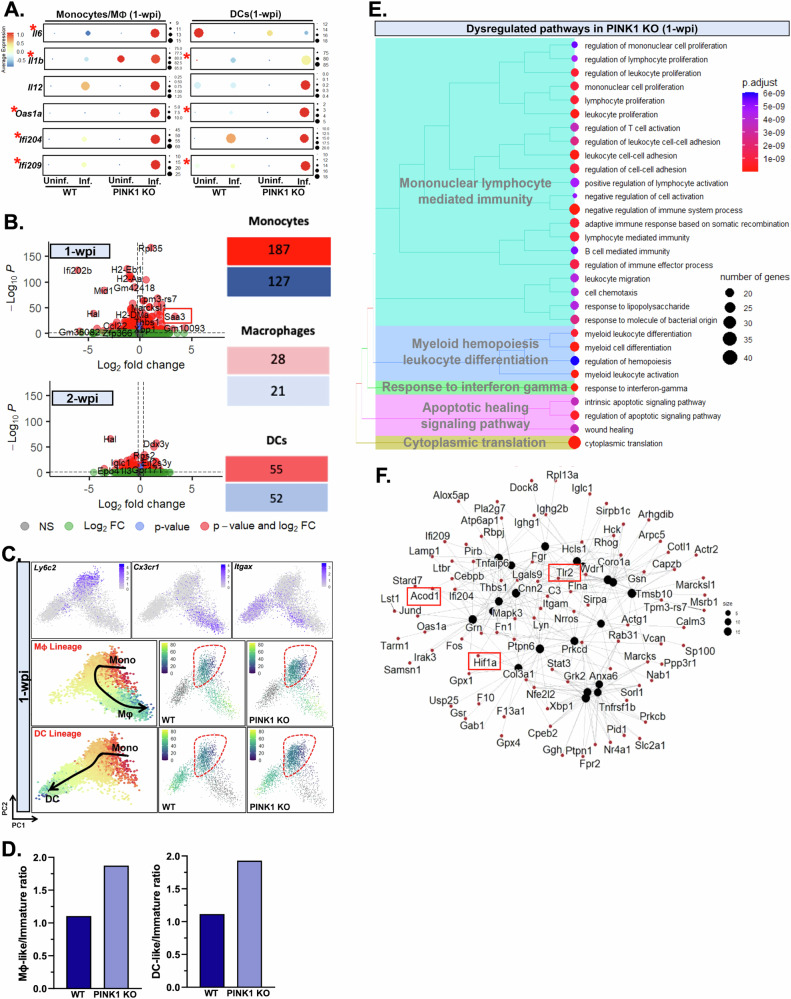
PINK1-driven dysregulation in the myeloid cell lineage is observed at the earliest phase of disease. **A** Dotplots indicate expression of T-cell-polarizing interleukin and interferon-related genes across all conditions in myeloid cell populations at 1 w.p.i. Significantly upregulated genes in myeloid cells of PINK1 KO infected mice compared to WT are denoted with an asterisk. Kruskal-Wallis followed by Dunn’s multiple comparison test was applied. **B** Volcano plots indicating DEGs in PINK1 KO compared to WT of monocytes/macrophages populations at 1- and 2 w.p.i. To test for significance, Wilcoxon Rank Sum test was applied using Bonferroni correction. Significant DEGs (p.adj < 0.5 with a log_2_ fold-change ≥ 0.25) are shown in red. Tables represent the number of DEGs per myeloid cell population whereby upregulated genes are shown in red while downregulated in blue. **C** Pseudotime analyses of myeloid cell populations at 1 w.p.i. revealed macrophage (Mϕ) and dendritic cell (DC) lineages originating from monocytes, defined by the expression of Cx3cr1, Itgax and Ly6c2, respectively. Both lineages are illustrated in PCA plots of intestinal myeloid cell populations from WT and PINK1 KO infected mice whereby the monocytes are encircled in red. **D** The cells within the monocyte population are classified to either immature or mature (Mϕ- or DC-like) subtypes with pseudotime values of low (0, 10) or high (10, 20). The proportions of each monocyte subtype was assessed and the ratios of Mϕ/DC-like-to-immature in PINK1 KO compared to WT were quantified. **E**, **F** Pathway enrichment analyses were run on significant DEGs in monocytes at 1 w.p.i. against Gene Ontological terms of biological processes database using ClusterProfiler. Dysregulated pathways are shown as a treeplot while a cnet plot is used to display the genes involved in driving major GO terms. Boxes represent genes that were subsequently validated using independent approaches. Interleukins, Il6/Il1b/Il12; 2’-5’-oligoadenylate synthetase 1, Oas1; interferon activated gene, Ifi204/9.

To elucidate a potential role of PINK1 in myeloid cells, we first assessed the expression of interleukin and interferon-related genes known to induce lymphocyte activation, including Il6, Il1b, Il12, Oas1a, Ifi204, Ifi209^53–55^. We denoted a striking upregulation of all T-cell-polarizing/activating cytokines evaluated here in the monocytes/macrophages population of PINK1 KO infected mice, while most were similarly elevated in PINK1 KO dendritic cells, with the exception of Il6, compared to wild type at 1 w.p.i. (Fig. 4A). Next, we delved deeper into the myeloid cell lineage and performed unsupervised clustering on these cells (generating higher resolution principal component analyses) to identify monocytes distinct from macrophages (Mϕs) and dendritic cells (DCs). The highest number of DEGs were found within intestinal monocytes (187 upregulated and 127 downregulated) (Fig. 4B). We then analyzed the differential trajectories in the monocytes using the aforementioned pseudotime methods, providing immature monocytes as origin given these are the precursor cells for Mϕs and DCs (Fig. 4C). This approach identified two distinct lineages in both wild type and PINK1 KO mice emerging from immature monocytes. One trajectory differentiated into macrophage-like mature monocytes and the other into DC-like monocytes. To determine whether genotype altered the propensity of intestinal monocytes to differentiate, we quantified the proportions of monocytes from wild type and PINK1 KO mice with low (0, 10) or high (10, 20) pseudotime values. This analysis suggested that PINK1 KO monocytes have a greater tendency to commit to becoming mature, antigen-presenting myeloid cells as evidenced by a higher ratio of Mϕ-like or DC-like monocytes in PINK1 KO infected mice compared to wild type (Fig. 4D). Most of the top GO terms associated with DEGs in the monocyte population in PINK1 KO infected mice compared to wild type represented functions related to dysregulated pathways of myeloid hemopoiesis leukocyte differentiation and mononuclear lymphocyte mediated immunity, which are congruent with an altered differentiation state and regulation of T cell activation (Fig. 4E; Supplementary Table 2). Key DEGs that were driving these GO terms and associated proinflammatory profiles include activation markers such as serum amyloid a3 (Saa3), aconitate decarboxylase 1 (Acod1), toll-like receptor 2 (Tlr2), and hypoxia-inducible factor 1 alpha (Hif1α) (Fig. 4B, F).

**Fig. 5:**
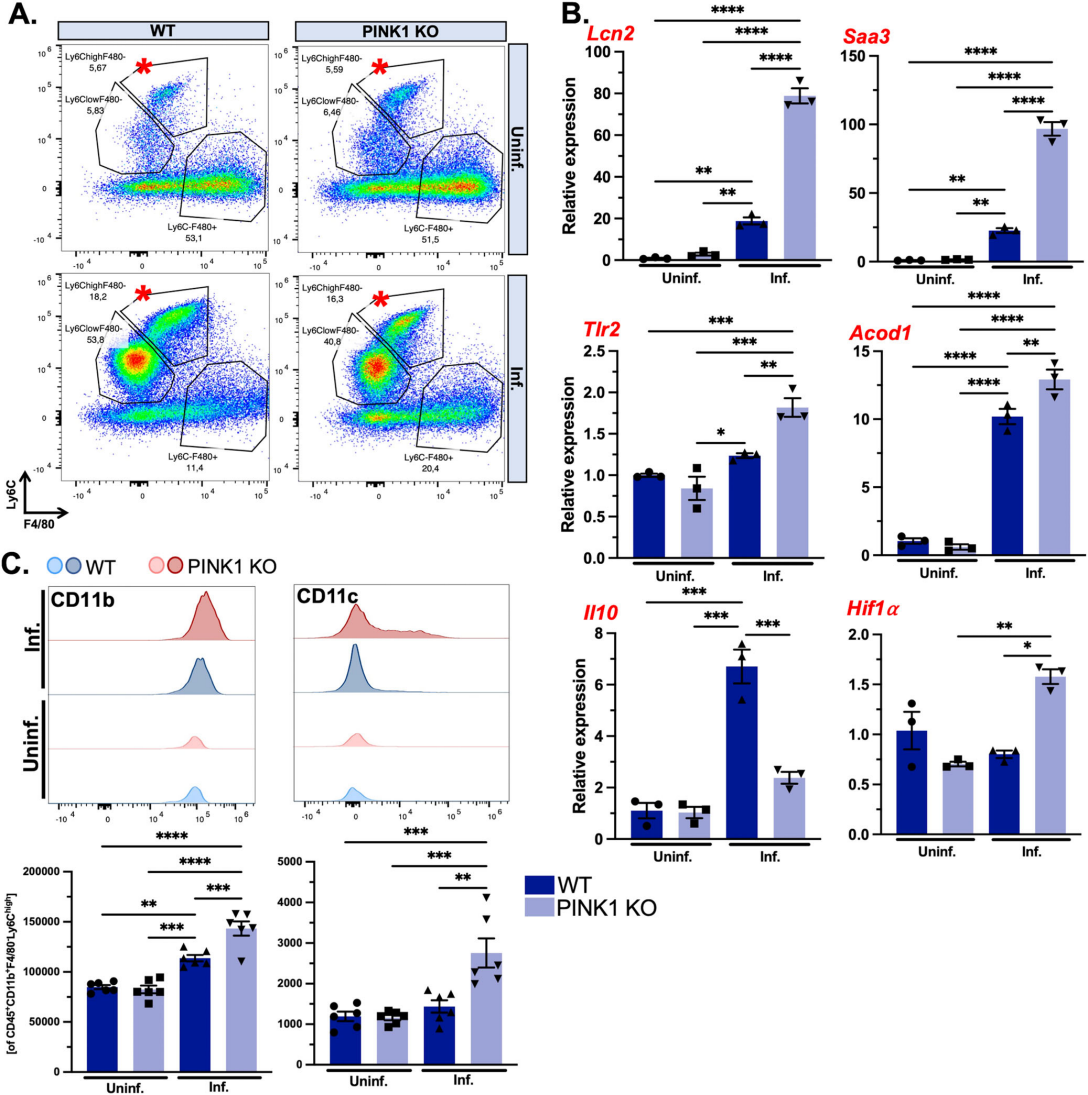
PINK1 KO monocytes acquire enhanced proinflammatory characteristics. **A** Representative flow cytometric graph of intestinal myeloid cells (CD45^+^CD11b^+^) from WT and PINK1 KO at 1-w.p.i. Ly6C^high^F4/80^-^ monocytes were collected by FACS for downstream gene expression analyses by qPCR. **B** qPCR of key DEGs involved in proinflammatory processes, previously identified in monocyte/macrophage clusters, were assessed from PINK1 KO infected mice compared to WT, including Lcn2, Saa3, Tlr2, Acod1, Il10 and Hif1a. Two-way ANOVA followed by Tukey’s multiple comparison test was applied. Mean ± SEM, *n* = 3 replicates of 6 pooled mice per group. **C** Flow cytometric analyses was performed on Ly6C^high^F4/80^-^ intestinal monocytes by evaluating geometric mean fluorescent intensity of CD11b and CD11c. Two-way ANOVA followed by Tukey’s multiple comparison test was applied. Mean ± SEM, *n* = 6 mice per group. Lipocalin-2, Lcn2; serum amyloid a 3, Saa3; toll-like receptor 2, Tlr2; aconitate decarboxylase 1, Acod1; interleukin 10, Il10; hypoxia-inducible 1 alpha, Hif1a; cluster of differentiation, CD11b/c.

To corroborate PINK1-driven myeloid cell changes, we isolated CD45^+^CD11b^+^F4/80^-^Ly6C^high^ monocytes from the colonic lamina propria of PINK1 KO and wild type mice at 1-w.p.i. by fluorescence-activated cell sorting (FACS) along with uninfected control groups (Fig. 5A). We validated significantly altered expression of key genes suggestive of enhanced proinflammatory responses in intestinal monocytes of PINK1 KO infected mice, such as Lcn2, Saa3, Acod1, Tlr2, Hif1α and a suppression of the anti-inflammatory cytokine, interleukin-10 (Il10) (Fig. 5B) which is analogous to critical observations made with single-cell RNAseq (Fig. 4B, F). We also noted elevated protein expression of CD11b and CD11c in intestinal Ly6C^high^ monocytes from PINK1 KO infected mice, further attesting to the increased differentiation state of PINK1 KO monocytes towards mature, antigen-presenting myeloid cells following infection (Fig. 5C).

### Myeloid and CD8 + T cells are poised for direct cell-cell contact mediated interactions in the colon of infected mice

**Fig. 6:**
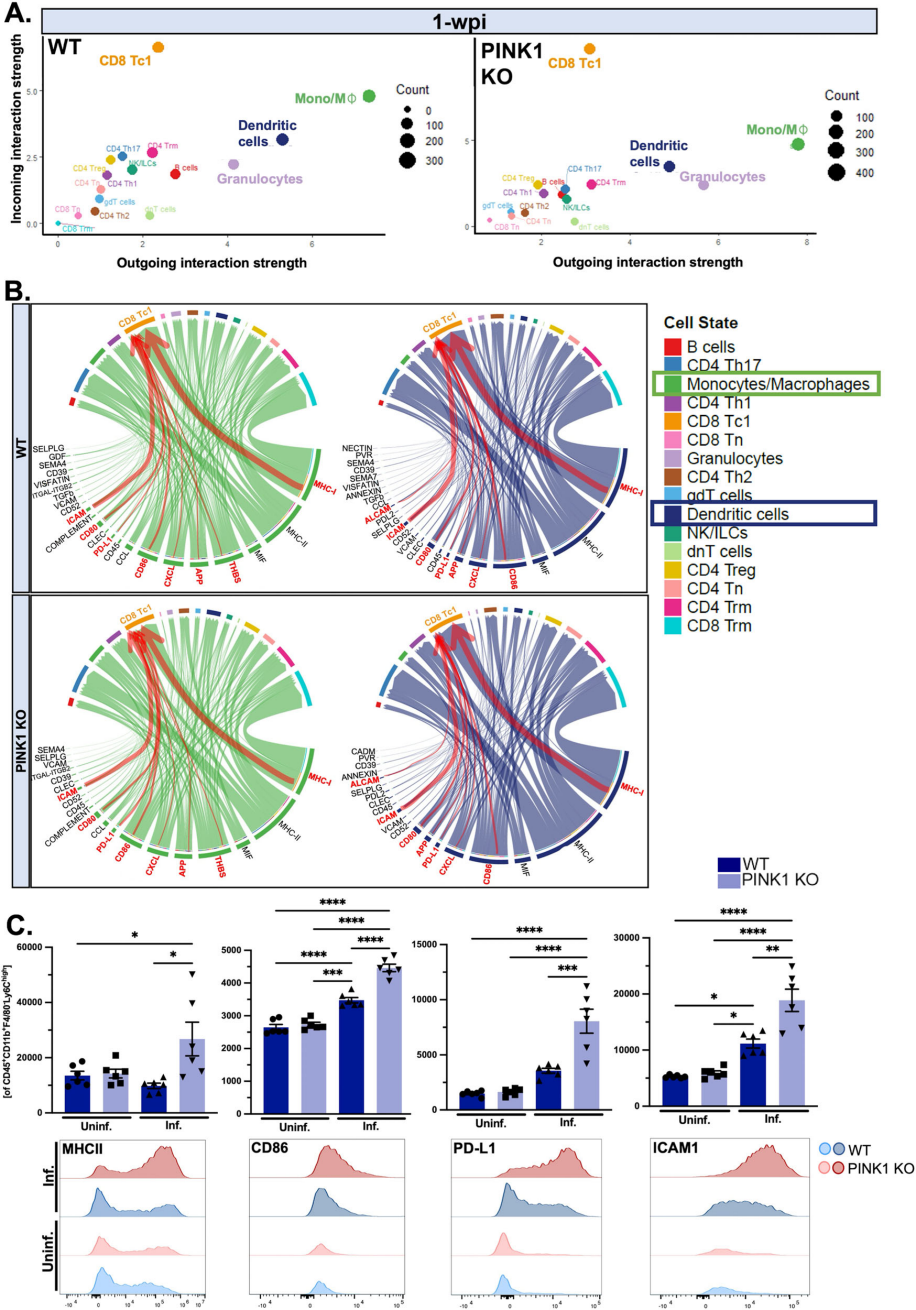
Intercellular communication analyses predict heightened myeloid-cytotoxic CD8 + T cell interactions regulating antigen presentation. **A** Unbiased cell-cell interaction analyses of intestinal immune cell populations in WT and PINK1 KO mice at 1- and 2-w.p.i. were conducted. Analysis of incoming and outgoing interaction strengths showed the putative strongest senders and receivers as the myeloid cells (Mono/Mϕ and DC) and effector CD8 + T cells (Tc1). Depicted is a scatter plot of immune cell interactions at 1-w.p.i. **B** Chord diagrams illustrate the associations in WT and PINK1 KO monocytes/macrophages (green) and dendritic cells (blue) to neighbouring immune cells following infection. Highlighted in red are pathways underpinning myeloid-CD8 + Tc1 cells interaction that are relevant to antigen presentation. The width of the arrows symbolizes the likelihood of interactions encompassing the specified pathway. **C** Flow cytometric analyses of Ly6C^high^F4/80^-^ intestinal monocytes were performed and geometric mean fluorescent intensity of proteins related to antigen presentation and implicated in myeloid-CD8 + T cells interaction were assessed, including MHCII, CD86, PD-L1 and ICAM1. Two-way ANOVA followed by Tukey’s multiple comparison test was applied. Mean ± SEM, *n* = 6 mice per group. Major histocompatibility complex II, MHCII; cluster of differentiation 86, CD86; programmed death-ligand 1, PD-L1; intercellular adhesion molecule 1, ICAM1.

Previous work has revealed that dysregulated antigen presentation in the absence of PINK1 in APCs can trigger CD8 + T cells to mount a cytotoxic response against self-cells^16,21,23^. Indeed, by conducting an unbiased predictive analysis of immune intercellular communication networks within the lamina propria of the gut using CellChat^56^, we uncovered that myeloid and CD8 + T cells were the dominant senders and receivers of signals, respectively, defined by the sum of all communication probabilities of outgoing/incoming signalling from/to a specific cell population (Fig. 6A). As such, we suspected that intestinal infection poises myeloid cells to interact preferentially with CD8 + T cells, eliciting a proinflammatory T cell response.

To decipher the outgoing signals from myeloid cells (monocytes/macrophages and dendritic cells) underpinning communication with CD8 + T cells, we assessed the overall cell-cell interactions between myeloid cells and all immune cell types identified at both 1- and 2-w.p.i. Accordingly, we revealed that CD8 + Tc1 cells were a major cell type receiving these signals (Fig. 6B) in both wild type and PINK1 KO mice. Of note, CD4 + Th17 cells are also receiving the majority of outgoing signals from myeloid cells although these cells were not identified as dominant receivers from the intercellular communication network analysis (Fig. 6A, B). Critically, wild type and PINK1 KO myeloid cells have an 80–100% overlap in outgoing signals sent to CD8 Tc1 cells which primarily pertain to direct cell-cell contact-mediated interactions related to antigen presentation^57–61^. These include major histocompatibility complexes (MHC), co-stimulatory molecules (CD86 and CD80), programmed death-ligand 1 (PD-L1) and intercellular adhesion molecule 1 (ICAM1) amongst others highlighted in Fig. 6B. Given the highest degree of PINK1 KO-related regulation within the myeloid lineage was in the monocyte population (Fig. 4A), we were particularly interested in the expression of direct cell-cell contact-mediated antigen presentation molecules in this population. Using flow cytometry of intestinal Ly6C^high^ monocytes in wild type and PINK1 KO mice at 1-w.p.i., we observed elevated levels of MHC, CD86, PD-L1 and ICAM1 in PINK1 KO monocytes compared to wild type, demonstrating that, on a protein level, there is the machinery for enhanced myeloid-T cell crosstalk relating to antigen presentation (Fig. 6C). Of note, intestinal Mϕs and DCs downregulated most of these proteins at this timepoint in the gut following infection with the exception of ICAM1, and no genotype-dependent differences were identified (data not shown).

Given our in vivo study focused only on gut tissue and only at defined time points, it remained unclear whether Mϕ and DCs could have similar propensities for antigen presentation, like monocytes. We differentiated primary Mϕs and DCs from mouse bone marrow progenitors and stimulating these cells with lipopolysaccharide (LPS) in vitro. Following challenge, PINK1 KO myeloid cells acquired elevated cell surface expression of MHCI/II as well as co-stimulatory molecules CD80 and CD86 (Supplementary Fig. 3A, B), which are required in signal 1 and 2 for direct T cell engagement. Previous findings from our group also corroborate that PINK1 modulates APC-T cell crosstalk wherein we report that PINK1 KO APCs are subject to enhanced mitochondrial antigen (OGDH) presentation, which leads to increased proportions of activated, OGDH-specific CD8 + T cells, indicating a cell-cell contact mechanism of T cell activation^21,22^. It is also unclear whether heightened CD8 + T cell activation is in part mediated by an aberrant signal 3 implicating cytokine secretion. We thus interrogated whether increased levels of T-cell-polarizing/activating cytokines (IL-6, IL-1β snd IL-12) were secreted by APCs lacking PINK1 in vitro. Congruent with our transcriptomics data (Fig. 4A), we demonstrated higher levels of IL-6, IL-1β and to some extent IL-12 secreted into the medium of LPS-treated PINK1 KO macrophages in vitro (Supplementary Fig. 3C).

We then utilized the conditioned medium (CM) from these macrophage cultures to study interactions with T cells (Supplementary Fig. 4A). Co-stimulation of either wild type or PINK1 KO CD8 + T cells with CM from LPS-treated PINK1 KO macrophages and anti-CD3/CD28 antibodies (for T cell receptor, TCR, and co-stimulatory molecules engagement) augmented T-cell mediated adaptive immune responses compared to challenges with CM from LPS-treated wild type macrophages. There was increased frequencies of wild type and PINK1 KO CD8 + T cells expressing cytolytic granules or proinflammatory cytokines, including granzyme B, perforin, IL-17a and IFN-γ, as well as activation-induced marker PD-1 (Supplementary Fig. 4B). We did not observe significant differences between wild type and PINK1 KO CD8 + T cells following activation with anti-CD3/CD28 antibodies alone (red bars). The sole T cell intrinsic effects that we could detect were unveiled when wild type and PINK1 KO CD8 + T cells were stimulated with CM from PINK1 KO APCs (light blue bars). In this context, there was higher frequency of CD8+ Prf1+ cells in PINK1 KO T cells compared to wild type (Supplementary Fig. 4B, 3^rd^ panel, light blue bars). Together, in addition to a direct cell-cell contact mediated dysregulation, our present findings allude to a strong cell-extrinsic effects driving robust cytotoxic T cell responses, perhaps partly mediated by excessive production of proinflammatory, T-cell-polarizing cytokines in PINK1-deficient myeloid cells.

## Discussion

Given the multifaceted nature of PD aetiology, it is important to consider multiple genetic and environmental triggers of this disease, including the impact of immune mechanisms that could drive pathophysiological processes in disease initiation and progression. In the present study, we further characterized a recently established infection model that is geared to study some of the earliest features of PD pathology driven by genetic-environmental interactions. This work unveiled dysregulated signalling pathways in myeloid cells at the initial stages of disease, potentially contributing to antigen presentation and cytotoxic T cell-mediated autoimmune mechanisms.

Clinical studies suggest intestinal immune dysfunction in PD wherein inflammatory mediators are reported to be more abundant in the stools of patients^4–8^. Exposure to microbial GI infections has been correlated with increased PD risk, where Gram-negative bacteria Helicobacter pylori is one of the most studied microbes associated with PD in humans^12^. Emerging preclinical models of PD instigated by GI infections may have far-reaching clinical significance in determining the underlying inflammatory-mediated mechanisms at play in the periphery driving pathology. To this end, we exploited our previously developed infection-induced PD model to study such mechanisms. Consistent with clinical observations indicating elevated inflammatory markers in stools of PD patients^4–7^, we found that PINK1 KO infected mice exhibit exacerbated intestinal inflammation as evidenced of higher faecal lipocalin-2 and calprotectin levels compared to wild type. Of note, we inoculated mice with higher C. rodentium (4 × 10^9^ CFU) contrary to our previous publication^21^ where genotype-dependent inflammatory intestinal markers following infection were not detectable. Interestingly, PINK1 KO mice at 2-w.p.i. tended to display more extensive gut dysmotility compared to wild type although these observations varied between cohorts. We suspect that differences between cohorts wherein some may have had high mouse Helicobacter positivity (which we did not track) could be underlying these PD-like phenotypes. Of note, in our related publication, we demonstrate that human Helicobacter pylori infection of PINK1 KO mice is sufficient to trigger PD-like motor deficits^22^. Future investigations are warranted to decipher the impact of a diversity of variables in our GI-targeted infection-induced PD models that may underlie the heterogeneity we observe across cohorts.

In line with a plethora of reports highlighting the role of other genes associated with familial PD in inflammation^62–68^, PINK1 has been shown to play pleiotropic effects in innate immunity^17–20,69^. However, whether PINK1-regulated inflammatory signalling perpetrates early disease processes in PD remains elusive. Here we discovered that the myeloid cell lineage exhibits the most substantial dysregulation at the initial stages of our infection-induced model. We further reveal an amplified proinflammatory response in monocytes of PINK1 KO infected mice, including upregulation of HIF1α. Consistent with this, a prior study demonstrated that deletion of PINK1 in non-immune cells resulted in glucose metabolism reprogramming, which requires increases in reactive oxygen species (ROS) production and the activation of HIF1α signalling^70^. Our data suggests the occurrence of early HIF1α-mediated metabolic alterations in activated PINK1 KO monocytes, which intriguingly is also in line with what has been observed in monocytes from peripheral blood of RBD patients who are considered at the prodromal period of the disease. These patients display elevated levels of mitochondrial ROS associated with a concomitant increase in glycolysis^71^. Other features of RBD monocytes include upregulation of human leucocyte antigen (HLA)-DR expression which correlates with worsened cognitive and motor performance^52^, while changes in TLR4 expression inversely correlate with the functionality of midbrain DA neurons, estimated by ^18^F-DOPA positron emission tomography (PET) imaging^50^. These findings are also recapitulated in our data demonstrating elevated expression of major histocompatibility complexes and toll-like receptors in intestinal monocytes of PINK1 KO mice at the earliest phase of infection.

Cellular roles of PINK1 seems to be context-dependent and operate in response to a distinct type of stressor. Previous studies elucidated conflicting results regarding the implication of PINK1 in mitigating inflammation^17–20,34^. Sliter et al., reported that PINK1 KO mice under mitochondrial stress display excessive systemic levels of the proinflammatory cytokines interleukin 6 and β-interferon via increased activation of the cyclic GMP–AMP synthase (cGAS)-stimulator of interferon genes (STING) signalling pathway^17^. A previous in vitro study of human skin cells following varicella zoster viral infection also suggested that PINK1 attenuates STING-mediated interferon responses^18^. In contrast, PINK1 was shown to induce retinoic-acid-inducible gene I (RIG-I)-like receptors-mediated antiviral immunity in peritoneal macrophages^19^. Here, we provide evidence for a negative regulation of myeloid cell responses by PINK1 in an intestinal microbial infection-induced PD model. It is still unclear if this dysregulation in myeloid cells triggered by PINK1 deficiency directly contributes to neuronal dysfunction or loss or whether effects are indirect, such as through other immune cells. Sun et al., previously reported that deletion of PINK1 in LPS/IFNγ-stimulated mixed-glia cultures elicits the generation of an inflammatory milieu conducive to neuronal death in vitro^20^. Intriguingly, our findings point to an upregulation of IL-6 and IL-1β secretion in activated PINK1 KO myeloid cells, which were formerly demonstrated to sufficiently induce dopaminergic neurodegeneration and aggravate synuclein-mediated neuronal pathology in iPSC models of PD^72–74^. Understanding the mechanisms for how PINK1 loss potentiates inflammatory responses in myeloid cells and the repercussions on DA neurons will be crucial to develop approaches to target immune-mediated pathology implicated in disease onset.

The contribution of PINK1 in inflammation has largely been attributed to its role in regulating mitophagy. During cellular stress, this serine/threonine kinase together with another PD-associated protein, the E3 ubiquitin ligase Parkin, is stabilized at the surface of damaged mitochondria, critical for the initiation of mitophagy. In the absence of PINK1/Parkin, impaired mitophagy occurs and is traditionally viewed as a putative neuronal cell-autonomous mechanism contributing to DA neuron loss^75–77^. Albeit recent work has suggested instead a role of PINK1 in modulating innate immunity induced by oxidative stress and viral infections^17–19^. Our group previously showed that PINK1 represses immune responses independent of mitophagy, through inhibition of the formation of MDVs, which carry proteins to late endosomes, where they are processed. PINK1 abrogation thus induces MDV transport to the endosomal and proteasomal compartments resulting in heightened MitAP in dendritic cells undergoing cellular stress^16^. With MitAP occurring in the periphery, it is likely that naïve T cells become educated against these self-peptides. Indeed, we have previously revealed that elevated MitAP in PINK1 KO dendritic cells leads to the establishment of mitochondrial antigen-specific T cells, which can later access the brain and cause degeneration of DA neurons^21–23^. The exact mechanisms linking such processes to neuronal loss are nonetheless unclear. Since DA neurons have the capacity to express MHCI at their cell surface in response to inflammatory signals^21,78^, a direct attack of DA neurons by cytotoxic CD8 + T cells is therefore plausible, as suggested by recent results demonstrating that adoptive transfer of mitochondria-specific CD8 + T cells in PINK1 KO and wild type mice is sufficient to cause death of midbrain DA neurons^23^. Importantly, our current study showcases that deregulated antigen presentation mediated by PINK1 KO occurs at the earliest stage of disease as evidenced by upregulation of MHC molecules and co-stimulatory ligands, including CD86 and ICAM1, in PINK1 KO APCs. We suggest that, in addition to perturbations in direct cell-cell contact mediated interactions as we have also previously noted^21,22^, a lack of PINK1 in myeloid cells may amplify cytotoxic T cell responses through excessive production of proinflammatory, T-cell-polarizing cytokines. Together, we posit that PINK1 may participate in a critical innate to adaptive immune transition in all aspects of APC-T cell cross talk via the three-signal paradigms, from TCR engagement, co-stimulation, and skewing.

The activation of intestinal CD8 + Tc1 cells in PINK1 KO mice at early stages after infection is illustrated by upregulation of Stat3 and Ccr5. A prior study demonstrated that CCR5 overexpression induces the activation of the JAK/STAT signalling pathway in human CD4 + T cells^39^. In addition, accumulation of CCR5 at the immunological synapse leads to more robust APC-T cell interactions^38^. Moreover, in this study, CD8 + Tc1 cells in PINK1 KO infected mice displayed significant elevated levels of cytotoxic mediator perforin (Prf1) concomitant with increased expression of Pdcd1 encoding PD-1, known to be upregulated early during acute infection^79^. We also described higher expression of other activation-induced markers (e.g., Tnfrsf9) as well as cytotoxicity-related genes (e.g., Gzmb, Ifng, Il17a) in intestinal CD8 + T cells of PINK1 KO infected mice. Our findings collectively suggest that effector CD8 + T cells are expanding in the gut of PINK1 KO mice at an early stage after intestinal infection, and it is likely that these T cells induce pronounced cytotoxic outcomes. Presently, it is still unclear if the appearance of more cytotoxic CD8 + T cells in the gut of PINK1 KO mice is a result of myeloid cell interactions with naïve or mitochondrial antigen-experienced T cells. Future work focused on identifying naturally occurring mitochondrial proteins processed and presented through the PINK1-controlled MDV pathway could inform plausible MHCI-TCR interactions in the gut and may open novel avenues for early detection and interventions in PD.

Ultimately, our study bridges critical knowledge gaps on the influence of genetic and environmental interplay on immune mechanisms that confer risk for PD. A deeper understanding of immunological events underlying the earliest pathological features may permit the development of effective therapies to delay or arrest the progression of disease from the periphery to the CNS.

## Methods

Protocols associated with this work can be found on protocols.io:

https://www.protocols.io/view/protocols-for-recinto-et-al-34-pink1-deficiency-re-kxygxy77ol8j/v3.

### Mice

DAT-Ires-Cre (RRID:IMSR_JAX:006660) mice were bred with Ai9 mice (RRID: IMSR_JAX:007909) to induce the expression of tdTomato in DAT-expressing cells (DAT- tdTomato) then were crossed with PINK1- deficient (PINK1 KO) mice in a mixed B6.129 background (RRID:IMSR**_**JAX:017946)^80^. Adult DAT-Cre-tdTomato wild type and PINK1 KO mice (both sexes) were utilized in the present study. Mice were housed under specific pathogen-free conditions, given water and food ad libitum. All animals experiments were performed in compliance to the guidelines and conditions specified by the Canadian Council on Animal Care and were approved by the animal care committee of Université de Montréal and McGill University (animal use protocol number MCGL-5009).

### Infection with C. rodentium

Mice were infected with chloramphenicol-resistant C. rodentium (4 × 10^9^ CFU) by oral gavage. To monitor burden of C. rodentium, faeces were collected from each mouse, weighed, dissociated in PBS, serial-diluted and plated on chloramphenicol-containing MacConkey agar Petri plates. Petri plates were incubated at 37 °C overnight to allow colony growth, which were counted the following day. C. rodentium was identified by its distinctive morphology on MacConkey agar. Final counts were measured as CFU g^-1^ of faeces. Faecal collection occurred on 1-, 2- and 4 weeks-post- infection (w.p.i.) to confirm ≥10^7^ CFU/g infection rates (~80% of infected mice achieved this rate and were included in the study).

### Colonic lamina propria cell preparation

Following mice euthanasia by CO_2_ asphyxiation and cervical dislocation, colons were dissected then faecal material was flushed with an 18 G needle and adipose tissue was removed. Colon was then cut longitudinally and then chopped into sections of 1-2 cm in length. To separate the mucosal epithelium, intestinal tissue sections were incubated in 2 mM EDTA at 37 °C for 15 min followed by vigorous manual shaking three times until cellular suspension appears dense and cloudy. Remaining intestinal tissue sections (consisting of the lamina propria (LP) cells) were then collected and enzymatically dissociated with 2:1 ratio of collagenase IV to DNAse I at 37 °C for 30 min followed by vigorous vortexing. The cellular suspension was then collected through a 70 μm cell strainer. A subsequent similar enzymatic digestion was necessary to completely dissociate intestinal tissue and release the whole lamina propria cells, which was then pooled with the first dissociation. Cells were spun down at 1500 rpm at 4 °C for 7 min at the end of each enzymatic digestion, then the pellet was resuspended in PBS supplemented with 1% foetal bovine serum (FBS). The percentage of viable cells was assessed by flow cytometry; the average was 60%. Cells were subject to downstream experiments, including cell surface and intracellular staining to acquire cell profile as well as cell sorting for qPCR and single-cell RNAseq (whereby only live single cells were collected by FACS).

Once separated, the mucosal epithelium was processed independently for single-cell RNAseq. Briefly, the cloudy cellular suspension containing the crypts was further dissociated into single cells by resuspending in TrypLe using 21 G needle followed by 10 mins incubation at 37 °C. The reaction was neutralized with basal media (Advanced DMEM F/12 supplemented with 10% FBS). Cellular suspension was then collected through 40 μm cell strainer and spun down at 900 rpm at 4 °C for 5 mins. The pellet was resuspended in PBS supplemented with 1% FBS. The percentage of viable cells was assessed by flow cytometry; the average was 40%. Finally, only live single cells were collected by FACS.

### Bone-marrow derived myeloid cell preparation

Sex-paired cohorts of male or female mice at 8–12 weeks of age were euthanized and bone marrow progenitor cells were flushed through a 25 G needle from both femurs and tibia. Bone marrow (BM) cells were further dissociated by passing through 18 G needle and collected through 70 μm cell strainer. Cellular suspensions were centrifuged at 300 x g for 10 min. The supernatants were discarded, and cells were resuspended in 2 ml of Ammonium Chloride-Potassium (ACK) red blood cells lysis buffer (ThermoFisher, Cat. No. A1049201) for 1 min. The reaction was stopped by adding 10 ml of basal medium (RPMI supplemented with 10% FBS, 1% antibiotic/antimycotic and GlutaMAX Supplement 1X). Then cells were centrifuged at 300 x g for 10 min. Supernatants were discarded and cells were resuspended in basal medium (RPMI supplemented with 1X glutamine, 1X penicillin/streptomycin and 10% FBS).

For BM-derived macrophage differentiation, progenitors were cultured in the presence of 20 ng/mL recombinant mouse M-CSF (PeproTech, Cat. No. 315-02). Cells were incubated at 37 °C, 5% CO2 for 7 days and media changes were completed every 2–3 days with fresh recombinant mouse M-CSF. Meanwhile, for BM-derived dendritic cell differentiation, progenitors were cultured in the presence of 20 ng/mL recombinant mouse GM-CSF (PeproTech, Cat. No. 315-03). Cells were incubated at 37 °C, 5% CO2 for 9 days and media change was completed on day 4 with fresh recombinant mouse GM-CSF.

Matured myeloid cells were harvested and supernatant collected after 24 h treatment with 500 ng/mL LPS-EB Ultrapure from E.coli O111:B4 (Invivogen, Cat. No. tlrl-3pelps).

### T cell stimulation assay

Spleens from wild type and PINK1 KO mice (8–12 weeks old) were collected and CD8 + T cells were purified using EasySep Mouse CD8 + T cell isolation kit (StemCell, Cat. No. 19853). Cells were counted and resuspended in RPMI medium supplemented with 1X glutamine, sodium pyruvate, non-essential amino-acids, β-mercaptoethanol, penicillin/streptomycin and 10% FBS. A density of 1 million cells per mL were plated in a 24-well suspension plate pre-coated with 10 μg/mL anti-mouse CD3 antibody (BioLegend, Cat. No. 100202). Cells were then immediately co-stimulated with 5 μg/mL anti-mouse CD28 antibody (BioLegend, Cat. No. 102102) alone or in combination with 1:1 ratio of conditioned medium from LPS-treated BMDM (added in two consecutive days) in the presence of 10 U/mL of IL-2. Following two-day challenge, CD8 + T cells were harvested and re-stimulated with 1X eBioscience Cell Stimulation Cocktail plus protein transport inhibitors consisting of phorbol 12-myristate 13-acetate (PMA), ionomycin, brefeldin A and monesin (ThermoFisher, Cat. No. 00-4975-93) for 5 h, necessary for intracellular cytokines evaluation. Finally, cells were analyzed by flow cytometry. The schematic diagram of the T cell stimulation assay is illustrated in Supplementary Fig. 4A.

### Enzyme-linked immunosorbent assay

For intestinal inflammatory markers (Lipocalin-2 and Calprotectin), faecal samples were suspended in PBS plus 0.1% Tween20 at a ratio of 100 mg of faeces per mL. To isolate the supernatants, samples were vortexed for 20 min before centrifugation at 13,000 rpm at 4 °C. For proinflammatory cytokines IL-6, IL-1β and IL-12p40 measurement in BMDM cultures, cells were treated with LPS (500 ng/ml) for 24 h. Supernatant was collected and briefly spun down to get rid of any debris or floating cells. ELISA was performed either using the mouse Lipocalin-2 DuoSet kit (R&D Systems, Cat. No. DY1857) or mouse S100A8/S100A9 Heterodimer (Calprotectin) DuoSet kit (R&D Systems, Cat. No. DY8596) or mouse IL-6 DuoSet kit (R&D Systems, Cat. No. DY406-05) or mouse IL-1 beta/IL-1F2 DuoSet kit (R&D Systems, Cat. No. DY401-05) or mouse IL-12/IL-23 p40 Non-Allele-specific DuoSet kit (R&D Systems, Cat. No. DY2398-05) as per manufacturer’s instructions. In brief, collected supernatants (either from faecal sample or BMDM supernatant) were applied to a 96-well plate pre-coated with corresponding capture antibody, washed three times with PBS plus 0.05% Tween20, treated with the detection antibody, washed three times, treated with streptavidin–HRP, washed three more times, and developed with a 1:1 ratio of hydrogen peroxide and tetramethylbenzidine. The optical density of the plates was read at 450 nm.

### Gastrointestinal motility assessment

Each mouse was placed separately in an empty sterile cage with access to water. For faecal water content measurement, 2-3 faecal pellets were collected immediately after output in a pre-weighted Eppendorf tube then weights were measured as “wet faeces”. Faecal pellets were then air-dried overnight at room temperature (or up to 48 h) and weights were again measured as “dry faeces”. To calculate the percent of faecal water content, we first determined the difference between weights of the “wet” and “dry” faeces, which is then divided by the weight of “wet” faeces and finally multiplied by 100. While the stool frequency was assessed by counting the number of faecal pellets found in each designated sterile cage for individual mouse over the course of 3 h then calculating the average number of faecal pellet output per hour.

### Flow cytometry

Following isolation, cells resuspended in PBS were stained using eBioscience fixable viability dye coupled with either Zombie Red (Invitrogen, Cat. No. 65-0866-14) or eFluor 506 (Invitrogen, Cat. No. 65-0866-14) for 20 min on ice, in the dark. Prior to washes with FACS buffer (PBS supplemented with 2% FBS and 1 mM EDTA), cells were spun down at 400 x g at 4 °C for 5 min. Fc receptors were then blocked with anti-mouse CD16/CD32 (Invitrogen, Cat. No. 14-01061-86) along with extracellular staining consisting of a mixture of conjugated antibodies in FACS buffer for 30 min on ice, in the dark. For intracellular transcription factor (TF) and cytokine staining (only T cells), cells were fixed in Foxp3/Transcription Factor Staining Buffer (eBiosciences, Cat. No. 00- 5523-00) for 60 min at RT, then washed in permeabilization buffer and stained with a mixture of conjugated antibodies in the same buffer. The extracellular and intracellular antibodies used are in Table 1–4. After washes, cells were resuspended in FACS buffer then acquired with either BD LSRFortessa or Attune NxT flow cytometer. For sorting live single cells, FACS Aria was used instead. Appropriate controls were applied such as single-stained UltraComp eBeads (Invitrogen, Cat. No. 01-2222-42) and fluorescence minus one (FMOs) using corresponding cells.

**Table 1:** Extracellular antibodies used for colonic myeloid cell staining

Antigen	Fluorophore	Clone	Dilution	Company	Cat. No.	RRID
CD45	Brilliant Violet 750	30-F11	1:250	BD	746947	AB_2871734
CD11b	APC Fire 810	M1/70	1:250	BioLegend	101288	AB_2910274
Ly6C	PerCP-Cy5.5	HK1.4	1:250	Invitrogen	45593282	AB_2723343
F4/80	APC	BM8	1:250	BioLegend	123116	AB_893481
CD11c	PE Fire 810	QA18A72	1:250	BioLegend	161106	AB_2904307
MHC II	APC/Cyanine7	M5/114.15.2	1:250	BioLegend	107628	AB_2069377
CD86	Alexa Fluor 700	GL-1	1:250	BioLegend	105024	AB_493721
PD-L1	Brilliant Violet 421	10 F.9G2	1:250	BioLegend	124315	AB_10897097
ICAM1	FITC	YN1/1.7.4	1:250	BioLegend	116105	AB_313696

**Table 2:** Extracellular and TF antibodies for colonic T cell staining

Antigen	Fluorophore	Clone	Concentrations	Company	Cat. No.	RRID
CD3	PerCP-Cy5.5	145-2C11	1:300	Invitrogen	45-0031-82	AB_1107000
CD4	APC-e780	RM4-5	1:300	Invitrogen	47-0042-82	AB_1272183
CD8	Alexa700	53-6.7	1:300	Invitrogen	56-0081-82	AB_494005
CD44	PE-Cy7	IM7	1:600	Invitrogen	25-0441-82	AB_469623
CD62L	APC	MEL-14	1:300	Invitrogen	17-0621	AB_469409
T-bet	BV711	O4-46	1:200	BD	563320	AB_2738136
RORγt	BV650	Q31-378	1:200	BD	564722	AB_2738915

**Table 3:** Extracellular antibodies for Bone marrow-derived myeloid cell staining

Antigen	Fluorophore	Clone	Concentrations	Company	Cat. No.	RRID
CD11b	PerCP-Cy5.5	M1/70	1:300	Invitrogen	45-0112-82	AB_953558
F4/80	PE-Texas Red	BM8	1:300	BioLegend	123146	AB_2564133
CD11c	APC e780	N418	1:200	Invitrogen	47-0114-82	AB_1548652
MHCII	PE-Cy7	M5/114.15.2	1:500	Invitrogen	25-5321-82	AB_10870792
MHCI	PE	M1/42	1:300	BioLegend	125506	AB_1227705
CD86	FITC	GL1	1:200	Invitrogen	11-0862-82	AB_465148
CD80	BV650	L307.4	1:200	BD	564158	AB_2738630

**Table 4:** Extracellular and intracellular antibodies for in vitro CD8 + T cell staining

Antigen	Fluorophore	Clone	Concentrations	Company	Cat. No.	RRID
CD8	APC-Cy7	53-.7	1:200	BioLegend	100714	AB_312752
PD-1	PE-e610	J43	1:200	Invitrogen	61-9985-82	AB_2574688
TNF-α	BV785	MP6-XT22	1:200	BioLegend	506341	AB_2565951
IFN-γ	FITC	XMG1.2	1:200	BioLegend	505806	AB_315399
Granzyme B	PE	NGZB	1:200	Invitrogen	12-8898-82	AB_10870787
Perforin	APC	S16009B	1:200	BioLegend	154404	AB_2721464
IL-17A	AF700	TC11-18H10.1	1:200	BioLegend	506914	AB_536016

### Quantitative reverse transcription PCR

RNA was extracted using the RNeasy mini kit (Qiagen, Cat. No. 74104) as instructed by the manufacturer. Applied Biosystems^TM^ High-Capacity cDNA Reverse Transcription Kit (FisherScientific, 4368814) was then used for reverse transcription to generate cDNA which was used for real-time quantitative PCR (qPCR) via TaqMan assays (as shown in Table 5) on a QuantStudio^TM^ 5 real-time PCR system. The 2^ −^ ^ΔΔCt^ method was used to analyse the data using Actb as control.

**Table 5:** TaqMan gene expression assays used in intestinal monocytes

Probes	Assay ID	Company	Cat. No.
Lcn2	Mm01324470_m1	ThermoFisher	4331182
Saa3	Mm00441203_m1	ThermoFisher	4331182
Tlr2	Mm00442346_m1	ThermoFisher	4331182
Acod1/Irg1	Mm01224532_m1	ThermoFisher	4331182
Il10	Mm01288386_m1	ThermoFisher	4331182
Hif1α	Mm00468869_m1	ThermoFisher	4331182
Actb	Mm02619580_g1	ThermoFisher	4331182

### Single-cell library preparation and analysis

Live cells were prepared for single-cell sequencing at final concentration of 1000 cells/μL. All cells were processed according to 10X Genomics Chromium Single Cell 3’ Reagent Guidelines (https://support.10xgenomics.com/single-cell-gene-expression). Briefly, cells were partitioned into nanoliter-scale Gel Bead-In-EMulsions (GEMs) using 10X GemCode Technology. Primers containing (i) an Illumina R1 sequence, (ii) a 16 bp 10x barcode, (iii) a 10 bp Unique Molecular Identifier (UMI) and (iv) a poly-dT primer sequence were incubated with partitioned cells resulting in barcoded, full-length cDNA from poly- adenylated mRNA. Silane magnetic beads were used to remove leftover biochemical reagents/primers, then cDNA was amplified by PCR. Enzymatic fragmentation and size selection was used to optimize cDNA amplicon size prior to library construction. R1 (read 1 primer sequence) were added during GEM incubation, whereas P5, P7, a sample index (i7), and R2 (read 2 primer sequence) were added during library construction via end repair, A-tailing, adaptor ligation and PCR. Quality control and quantification was performed using an Agilent Bioanalyzer High Sensitivity chip. Sequencing was performed using NovaSeq 6000 S4 PE 100 bp, which resulted in a read depth of ~20,000 reads/cell. Reads were processed using the 10X Genomics Cell Ranger Single Cell 2.0.0 pipeline (RRID:SCR_017344, https://support.10xgenomics.com/single-cell-gene-expression/software/pipelines/latest/what-is-cell-ranger) with default and recommended parameters, as previously described^81^. FASTQs generated from sequencing output were aligned to the mouse GRCm38 reference genome using the STAR algorithm 2.7.3a (RRID:SCR_004463, http://code.google.com/p/rna-star/)^82^. Next, gene-barcode matrices were generated for each individual sample by counting unique molecular identifiers (UMIs) and filtering non-cell associated barcodes. This output was then imported into the Seurat v4.9.9.9039 R toolkit v.4.2.1 (for quality control and downstream analysis of our single cell RNAseq experiment^83^). All functions were run with default parameters, unless specified otherwise. Low quality cells (<200 genes/cell and >5% of mitochondrial genes) were excluded from the overall experiment. Gene expression was log normalized to a scale factor of 10,000.

### Statistical analysis

Statistical analyses were performed using GraphPad Prism 5.0 software. Either a One-way or two-way ANOVA or non-parametric Kruskal-Wallis test were used to compare the mean of three or more groups of data, indicated in the Figure legends. *P*-values were adjusted using either Tukey’s or Dunn’s multiple comparison test. Mean and standard error of the mean (SEM) of biological replicates (n) are plotted in all graphs and indicated in the Figure legends. *P*-value < 0.05 was considered statistically significant.

### Illustrations

The graphical abstract and the schematic representation of the experimental designs were created with BioRender.com.

## Supplementary information

Supplementary Figures and legends

Supplementary Table 1 and 2

## Data Availability

The datasets, software/code, protocols, and lab materials used and/or generated in this study are listed in a Key Resource Table alongside their persistent identifiers at https://zenodo.org/records/15306350. Raw and processed single cell data available in GSE271210 and source code can be found at https://zenodo.org/records/15342597. Single cell RNAseq data is accessible through a user-interface website available for further analyses at https://singlocell.openscience.mcgill.ca/display?dataset=RNA_Ms_Gut_Immune_PD_2024. An earlier version of this manuscript was posted to BioRxiv on July 24, 2024 at 10.1101/2024.06.18.598931.
