# Supplementary material for: PINK1 deficiency rewires early immune responses in a mouse model of Parkinson’s disease triggered by intestinal infection
Source: NPJ Parkinsons Dis. 2025 May 22;11:133. doi: 10.1038/s41531-025-00945-w (PMC12098848; doi:10.1038/s41531-025-00945-w)
Supplement: Supplementary file 2 — Supplementary Table 1 and 2 [file 41531_2025_945_MOESM2_ESM.pdf]

Supplemetary Table 1: Full list of markers for each cell type annotated in Fig. 1D

B cells

Gene	p_val	avg_log2FC	pct.1	pct.2	p_val_adj
Ebf1	0	7.92801835	0.924	0.013	0
Cd79a	0	7.2290747	0.896	0.022	0
Mef2c	0	4.8693159	0.857	0.117	0
Scd1	0	7.00336497	0.705	0.023	0
Ighm	0	4.50830336	0.852	0.181	0
Ighd	0	7.89805272	0.595	0.007	0
Ms4a1	0	8.16673836	0.589	0.005	0
H2-Aa	0	0.57684414	0.992	0.44	0
H2-Ab1	0	0.29617774	0.983	0.445	0
H2-Ob	0	4.70811647	0.609	0.074	0
Pax5	0	8.00914354	0.501	0.004	0
Bank1	0	5.98546869	0.517	0.022	0
Ccr7	0	1.86600275	0.687	0.193	0
Ly6d	0	5.87277628	0.51	0.024	0
Cd79b	0	5.87225551	0.498	0.024	0
H2-DMb2	0	3.84939591	0.565	0.095	0
Cd74	0	0.8197178	0.999	0.543	0
Gm8369	0	2.66976994	0.603	0.182	0
Iglc2	0	2.21257334	0.729	0.316	0
Dmxl1	0	3.34370356	0.566	0.164	0
Cd83	0	1.17935424	0.748	0.352	0
Ralgps2	0	3.5036771	0.484	0.096	0
Iglc3	0	3.57733136	0.469	0.085	0
Fcmr	0	8.13287022	0.385	0.003	0
Cd19	0	7.41311682	0.387	0.005	0
S1pr1	0	2.68779212	0.488	0.109	0
Cd37	0	2.287651	0.596	0.233	0
Irs2	0	2.31530394	0.539	0.192	0
Fcrla	0	7.23660606	0.352	0.006	0
Satb1	0	1.26504113	0.822	0.479	0
Mzb1	0	3.40709264	0.4	0.063	0
Fcer2a	0	7.99332689	0.316	0.003	0
Stap1	0	2.34795768	0.535	0.227	0
Cd55	0	3.94266373	0.342	0.04	0
Vpreb3	0	6.85438144	0.299	0.006	0
Foxp1	0	1.67688484	0.71	0.424	0
B3gnt5	0	3.00967284	0.379	0.097	0
Txndc16	0	4.17542947	0.321	0.041	0
Nfatc3	0	1.79385406	0.514	0.244	0
Cd22	0	5.75035447	0.281	0.013	0
Pou2af1	0	4.80517305	0.283	0.018	0
Hvcn1	0	3.59611161	0.321	0.059	0
Dgkd	0	2.42671685	0.409	0.149	0
H2-Oa	0	2.93396789	0.344	0.088	0
Blnk	0	3.28184235	0.303	0.055	0

Cxcr4	0	1.31962157	0.668	0.424	0
Plac8	0	0.59450275	0.639	0.398	0
Ablim1	0	1.30465758	0.482	0.248	0
Camk2d	0	2.18550046	0.397	0.164	0
Cr2	0	7.70742287	0.232	0.002	0
Rasgef1b	0	2.79069373	0.323	0.093	0
Serp1	0	1.28151113	0.79	0.563	0
Tnfrsf13c	0	6.58209238	0.232	0.006	0
Tcf12	0	1.56315841	0.456	0.245	0
H2-DMa	0	0.77004353	0.498	0.287	0
Zfp318	0	3.98468289	0.242	0.032	0
Cd24a	0	1.83461498	0.301	0.092	0
Eif2ak3	0	2.76604323	0.278	0.071	0
Cd69	0	1.12464502	0.473	0.27	0
Bach2	0	2.79376751	0.265	0.063	0
Pkig	0	3.11646231	0.269	0.068	0
Cybb	0	0.62350808	0.457	0.256	0
Gm20696	0	4.04859743	0.223	0.022	0
H2-Eb2	0	5.78721243	0.21	0.009	0
Gnl3	0	1.82507838	0.418	0.219	0
Tcf4	0	2.08025972	0.357	0.158	0
Prkcb	0	2.03529999	0.351	0.153	0
Irf8	0	1.05844397	0.415	0.217	0
Snx5	0	1.77928179	0.486	0.289	0
Hmgn1	0	2.01454709	0.372	0.175	0
Blk	0	4.72737913	0.207	0.01	0
Dtx1	0	3.74858557	0.215	0.021	0
Cd72	0	2.25779103	0.29	0.097	0
Heg1	0	2.99454626	0.248	0.056	0
Chst3	0	7.71594199	0.193	0.002	0
Bcl11a	0	5.42132553	0.204	0.013	0
Slc12a6	0	2.57770515	0.261	0.074	0
Cmah	0	2.89652962	0.227	0.04	0
Retreg1	0	1.67527599	0.401	0.215	0
Cerk	0	2.37140526	0.294	0.108	0
Egr3	0	4.48580555	0.204	0.021	0
Sp100	0	1.54031318	0.407	0.225	0
Rhoh	0	1.25018895	0.426	0.246	0
Ero1lb	0	2.66740966	0.268	0.092	0
Fcrl1	0	5.83211847	0.181	0.005	0
Rasgrp3	0	5.62342156	0.184	0.008	0
Pold4	0	1.82722268	0.432	0.257	0
Iglc1	0	1.15990776	0.565	0.39	0
Stk17b	0	1.29696977	0.909	0.736	0
Zfp36l1	0	0.64533207	0.788	0.615	0
Spib	0	6.66704147	0.176	0.004	0
Syk	0	1.20426332	0.428	0.256	0
Pxk	0	2.50842993	0.262	0.092	0

B3gnt7	0	6.8016819	0.17	0.003	0
Mtss1	0	2.03367011	0.276	0.111	0
Btk	0	3.63705425	0.203	0.038	0
Irf4	0	1.78388295	0.291	0.126	0
Ppp3ca	0	1.47327119	0.455	0.291	0
Cxcr5	0	4.81855256	0.173	0.009	0
Myc	0	2.50061282	0.247	0.083	0
Trim7	0	5.73270083	0.169	0.008	0
Tsc22d3	0	1.22331628	0.437	0.277	0
Klhl24	0	1.35831099	0.428	0.27	0
Dennd5b	0	3.61504885	0.186	0.029	0
Bcar3	0	4.62906477	0.166	0.011	0
Snn	0	5.59286283	0.163	0.008	0
Cd38	0	2.04038756	0.259	0.106	0
Ciita	0	3.12419904	0.214	0.062	0
Plekha2	0	1.70296479	0.289	0.138	0
A630001G21R	0	2.9899194	0.19	0.042	0
Snx30	0	3.59053271	0.176	0.028	0
Smc6	0	1.72812361	0.38	0.233	0
Kras	0	1.30704507	0.558	0.414	0
Treml2	0	3.68211832	0.163	0.019	0
Tmem131l	0	2.15920442	0.233	0.091	0
Ilf3	0	1.93031574	0.27	0.128	0
Ripor2	0	1.58956959	0.249	0.109	0
Swap70	0	1.86317264	0.272	0.134	0
N4bp2	0	2.11224511	0.205	0.067	0
Parp1	0	2.16337413	0.233	0.097	0
Ptp4a3	0	2.83469358	0.183	0.047	0
Gga2	0	3.55515484	0.168	0.033	0
Tmem108	0	4.6639173	0.144	0.009	0
Ccr6	0	3.40817577	0.153	0.019	0
Sell	0	1.67930449	0.198	0.065	0
Strbp	0	2.08933668	0.219	0.086	0
Gm31243	0	7.16432972	0.135	0.002	0
Pik3ip1	0	2.89261215	0.159	0.03	0
Fam69a	0	3.36072163	0.156	0.028	0
Lrrk2	0	3.47224261	0.158	0.031	0
Fgfr1op	0	2.60475204	0.189	0.062	0
Pml	0	2.8552039	0.162	0.038	0
Cd180	0	3.41073971	0.161	0.038	0
Gm30211	0	5.44844251	0.127	0.005	0
Snx29	0	3.22873542	0.149	0.029	0
Aff3	0	3.49827979	0.136	0.017	0
Chchd10	0	2.17526643	0.197	0.078	0
Ly6e	0	0.78629304	0.874	0.757	0
Pgap1	0	2.73530785	0.154	0.038	0
Siglecg	0	2.9564049	0.151	0.037	0
Zbtb10	0	2.14460087	0.164	0.052	0

A530032D15R	0	3.27347988	0.142	0.03	0
Dok3	0	2.94059527	0.138	0.032	0
Cnr2	0	4.69371667	0.112	0.006	0
Fam43a	0	3.56164187	0.124	0.019	0
Manea	0	2.66684084	0.139	0.037	0
Hs3st1	0	1.00475281	0.138	0.037	0
Eef1g	0	0.94779952	0.699	0.601	0
Arhgap24	0	4.20321607	0.101	0.008	0
Bcl7a	0	3.53335949	0.102	0.012	0
A530040E14R	0	3.86420702	0.103	0.017	0
Ddx6	0	0.85339832	0.692	0.607	0
Rhobtb2	0	2.84674777	0.102	0.022	0
Rpl35	0	0.70734388	0.938	0.862	0
AC125149.3	0	3.79645228	0.087	0.014	0
Rpl36a	0	0.80956828	0.95	0.881	0
Hspe1	0	0.91395454	0.687	0.619	0
Rps17	0	0.61111374	0.92	0.852	0
Fam167a	0	4.23129977	0.073	0.006	0
Gpr137c	0	4.50730318	0.07	0.005	0
Srpk3	0	6.59225697	0.065	0.001	0
Rubcn	0	3.50493143	0.072	0.01	0
Rab30	0	4.57776713	0.066	0.006	0
Cecr2	0	6.6382914	0.061	0.001	0
Rpl10a	0	0.54907267	0.972	0.912	0
Rpl12	0	0.70338475	0.996	0.937	0
Sbk1	0	4.52832481	0.057	0.005	0
Rps18	0	0.43475392	0.981	0.929	0
H3f3a	0	0.58451134	0.921	0.871	0
Rps7	0	0.43721255	0.988	0.938	0
Bfsp2	0	5.34912372	0.052	0.003	0
Rps19	0	0.95282263	0.998	0.95	0
Rpl5	0	0.46297443	0.984	0.936	0
mt-Cytb	0	0.62912861	0.972	0.924	0
Rps4x	0	0.45038796	0.994	0.947	0
Jchain	0	0.30706855	0.8	0.753	0
Rps5	0	0.48637115	0.994	0.949	0
Rpl22	0	0.46747494	0.983	0.939	0
Rpl21	0	0.45963864	0.99	0.947	0
Rps2	0	0.40455507	0.984	0.941	0
Rpl32	0	0.32903141	0.993	0.951	0
Rps25	0	0.63667316	0.991	0.95	0
Rpl26	0	0.41334025	0.988	0.947	0
Rpl36	0	0.43911501	0.992	0.951	0
Rps20	0	0.97167987	0.998	0.958	0
Rpl6	0	0.33159522	0.988	0.948	0
Rpl7	0	0.41370006	0.979	0.94	0
Rps15	0	0.68421779	0.997	0.958	0
Rps26	0	0.69792153	0.997	0.959	0

Rpsa	0	0.39299884	0.996	0.959	0
Rps13	0	0.39337711	0.997	0.961	0
mt-Nd4	0	0.54795544	0.938	0.902	0
Rpl8	0	0.49119579	0.988	0.953	0
Ptma	0	0.42748806	0.971	0.936	0
Rpl39	0	0.4722454	0.997	0.963	0
Rpl18a	0	0.59212823	0.998	0.964	0
Rpl30	0	0.51439895	0.998	0.964	0
Rps3a1	0	0.49382233	0.998	0.965	0
Rpl34	0	0.33626857	0.997	0.964	0
Rpl13	0	0.53658088	0.999	0.966	0
Rpl19	0	0.29312091	0.998	0.965	0
Rps10	0	0.42703553	0.998	0.965	0
mt-Atp6	0	0.53252929	0.992	0.959	0
Rps28	0	0.64110852	0.998	0.966	0
Rpl17	0	0.32438062	0.996	0.964	0
Rpl27a	0	0.4738609	0.998	0.967	0
mt-Co1	0	0.39916686	0.996	0.966	0
mt-Co2	0	0.42272487	0.99	0.96	0
mt-Co3	0	0.40662482	0.992	0.963	0
Rpl37	0	0.50297507	0.997	0.969	0
Rps24	0	0.65994275	1	0.974	0
Rpl38	0	0.43572859	0.999	0.973	0
Rps21	0	0.66787099	1	0.975	0
Rps8	0	0.64380639	1	0.975	0
Rps12	0	0.62691311	0.999	0.977	0
Rps16	0	0.38333343	0.999	0.978	0
Rpl37a	0	0.5737194	1	0.98	0
Rplp2	0	0.4517206	0.999	0.98	0
Rplp1	0	0.30232103	0.999	0.98	0
Rpl23	0	0.4751326	0.999	0.98	0
Rpl35a	0	0.55274819	0.999	0.98	0
Rps27	0	0.95130862	0.999	0.981	0
Rps29	0	0.60924983	0.999	0.983	0
Rpl41	0	0.34038119	1	0.987	0
Rps9	0	0.35252083	0.999	0.989	0
Fau	0	0.5236543	1	0.993	0
Igkc	0	0.92892366	0.999	0.995	0
Igha	0	0.28944747	0.988	0.984	0
Napsa	4.391E-302	1.68312544	0.281	0.143	9.633E-298
Smim14	1.26E-296	1.66497444	0.329	0.193	2.763E-292
Phip	2.136E-296	1.36474301	0.393	0.247	4.686E-292
Bmp2k	3.183E-294	1.85409191	0.252	0.123	6.984E-290
Gm15991	9.455E-294	6.06308354	0.035	0.001	2.074E-289
Alkbh1	1.754E-290	1.50309937	0.335	0.193	3.848E-286
Stt3b	2.42E-288	1.85693583	0.266	0.136	5.309E-284
Malt1	2.726E-287	0.70555933	0.635	0.478	5.98E-283
mt-Nd2	8.138E-287	0.5598189	0.803	0.774	1.785E-282

Elmsan1	3.42E-282	1.48520608	0.318	0.178	7.502E-278
Btla	1.303E-279	2.32515016	0.161	0.059	2.859E-275
Nsf	1.429E-276	1.9712439	0.242	0.119	3.136E-272
Trp53i11	2.655E-275	2.22250833	0.13	0.04	5.824E-271
Pou2f2	1.374E-270	1.3737412	0.333	0.192	3.015E-266
Scaf11	1.533E-270	0.68574837	0.46	0.324	3.363E-266
Rpl31	2.891E-270	0.43181872	0.885	0.846	6.342E-266
Chst10	6.903E-268	3.87130831	0.05	0.005	1.514E-263
Rpl9	6.892E-267	0.29650917	0.989	0.954	1.512E-262
Cyth1	1.742E-266	1.27756137	0.425	0.3	3.822E-262
Fubp1	1.361E-263	1.15451164	0.447	0.322	2.986E-259
Klf2	2.491E-258	1.19343694	0.345	0.2	5.466E-254
Ffar1	7.012E-258	6.5761265	0.031	0.001	1.538E-253
Ublcp1	2.131E-256	2.21989456	0.117	0.035	4.676E-252
Cd86	4.426E-256	1.33114632	0.366	0.232	9.71E-252
Rel	1.608E-255	0.56320505	0.758	0.64	3.528E-251
Ezr	7.526E-254	1.00185581	0.531	0.421	1.651E-249
Zfp608	7.833E-250	2.76042807	0.096	0.025	1.718E-245
Sf3b3	7.416E-249	1.15444488	0.429	0.305	1.627E-244
Rpl4	3.141E-248	0.43999167	0.858	0.837	6.892E-244
Rps27rt	1.798E-247	1.03660841	0.51	0.411	3.944E-243
Nap1l1	6.526E-247	0.80084822	0.625	0.564	1.432E-242
Macf1	1.172E-246	1.06776192	0.479	0.366	2.57E-242
Ifi214	1.311E-246	2.4648856	0.098	0.026	2.876E-242
Inpp5d	1.48E-246	1.38486879	0.357	0.233	3.246E-242
Fam107b	9.877E-246	0.68217387	0.649	0.546	2.167E-241
Sypl	1.817E-245	1.88777302	0.239	0.123	3.986E-241
Rpl23a	5.158E-243	0.25403293	0.989	0.956	1.132E-238
Sp140	5.717E-243	1.37990933	0.333	0.21	1.254E-238
Sorl1	8.437E-243	1.15907239	0.359	0.228	1.851E-238
Pik3c2b	9.336E-243	4.65781674	0.035	0.002	2.048E-238
Ifi209	3.138E-240	1.86133199	0.206	0.096	6.885E-236
Otulin	4.162E-240	1.38185547	0.401	0.287	9.131E-236
mt-Nd5	9.729E-240	0.82390133	0.61	0.551	2.134E-235
Ash1l	2.261E-239	1.36646628	0.333	0.207	4.959E-235
Smg1	1.313E-238	1.22737252	0.413	0.296	2.881E-234
mt-Nd1	1.379E-232	0.39178369	0.927	0.91	3.025E-228
Rps3	2.543E-232	0.26673047	0.988	0.951	5.58E-228
Elf1	2.261E-230	0.78885245	0.559	0.446	4.961E-226
Myo1e	3.211E-229	1.49360019	0.271	0.151	7.044E-225
Rpl18	1.744E-227	0.25142554	0.991	0.954	3.826E-223
Lmo2	6.505E-224	2.63202595	0.144	0.056	1.427E-219
Plk2	3.698E-219	1.89971403	0.199	0.095	8.112E-215
Zfp507	1.249E-217	2.03837723	0.111	0.035	2.741E-213
Cacna1e	1.596E-217	4.63579914	0.037	0.003	3.501E-213
Sipa1	1.782E-216	1.94057196	0.169	0.073	3.91E-212
Lyl1	9.929E-214	3.39710902	0.056	0.01	2.178E-209
Itpr3	1.069E-213	1.90602935	0.146	0.057	2.345E-209

Lbh	1.498E-213	1.40980512	0.28	0.163	3.285E-209
Fchsd2	4.867E-213	1.71519316	0.211	0.105	1.068E-208
Kmt2e	3.231E-212	0.91358132	0.505	0.402	7.089E-208
Rbm25	1.825E-210	0.6661602	0.662	0.627	4.004E-206
Tmod3	4.233E-210	1.31323608	0.403	0.305	9.286E-206
Sema4b	4.611E-210	2.24724421	0.113	0.038	1.012E-205
Fcrl5	5.837E-207	5.77071472	0.025	0.001	1.281E-202
Rpl14	1.378E-206	0.3280092	0.934	0.902	3.023E-202
Ildr1	1.382E-206	3.12069151	0.08	0.021	3.032E-202
Rpl3	4.805E-206	0.30481035	0.956	0.923	1.054E-201
Trim11	8.466E-205	2.34517196	0.11	0.037	1.857E-200
Etnk1	6.129E-204	1.22722172	0.354	0.237	1.345E-199
Wdfy4	1.333E-203	1.74368547	0.187	0.089	2.923E-199
Prkce	2.193E-203	2.19597785	0.116	0.041	4.812E-199
March1	1.012E-200	2.46641383	0.126	0.048	2.221E-196
Ephx1	6.846E-198	2.55668183	0.078	0.02	1.502E-193
Cacna1i	1.888E-196	5.87019619	0.025	0.001	4.141E-192
Cenps	1.276E-193	3.590299	0.046	0.007	2.799E-189
Plekhm1	2.854E-193	2.25043359	0.098	0.031	6.26E-189
Gdi2	1.3E-190	0.67637288	0.646	0.594	2.852E-186
Pag1	5.057E-190	1.4779198	0.208	0.107	1.109E-185
Ssh2	1.297E-186	0.8890903	0.436	0.321	2.846E-182
Lynx1	2.803E-180	4.62808154	0.028	0.002	6.149E-176
Rpl28	1.068E-179	0.26677979	0.976	0.94	2.342E-175
Rps6	1.353E-178	0.27302891	0.953	0.926	2.968E-174
Pik3r4	2.834E-178	2.21159929	0.099	0.034	6.217E-174
Rnf145	1.538E-177	1.46435908	0.22	0.12	3.375E-173
Tmem55b	1.54E-177	1.56387596	0.233	0.134	3.378E-173
Srsf10	1.712E-177	1.3020701	0.306	0.203	3.756E-173
Ly86	2.062E-177	1.11656929	0.315	0.205	4.525E-173
Nxpe2	2.293E-176	8.21969376	0.019	0	5.032E-172
Coq8a	3.824E-175	1.62539527	0.145	0.063	8.39E-171
Eif4a2	1.153E-173	0.85035394	0.505	0.431	2.529E-169
Inpp5f	1.669E-173	2.43894837	0.081	0.024	3.661E-169
Ptpn6	6.493E-172	1.83090914	0.189	0.099	1.425E-167
Rpl27	1.291E-171	0.32838308	0.898	0.879	2.833E-167
Pkib	1.829E-171	1.50325332	0.192	0.098	4.012E-167
Farsb	1.358E-166	2.32018863	0.106	0.039	2.979E-162
Usp6nl	2.201E-166	1.9430513	0.141	0.063	4.829E-162
Bend5	2.283E-166	4.33778407	0.027	0.002	5.008E-162
Phtf2	2.686E-166	1.9923061	0.104	0.038	5.894E-162
Card6	2.29E-165	1.81586287	0.104	0.038	5.023E-161
Serinc3	4.171E-165	0.63057732	0.612	0.56	9.15E-161
Fmnl3	3.458E-163	2.22197154	0.08	0.025	7.587E-159
E130102H24R	8.048E-163	2.59264407	0.055	0.012	1.766E-158
Rapgef5	8.665E-163	3.48153659	0.034	0.004	1.901E-158
Arhgap25	9.84E-163	1.76933513	0.145	0.066	2.159E-158
Setbp1	1.214E-162	2.4490574	0.059	0.014	2.663E-158

2010309G21R	2.598E-162	7.55138142	0.018	0	5.701E-158
Pmf1	3.33E-162	2.08105327	0.123	0.052	7.306E-158
Cxxc5	4.596E-162	2.04633757	0.096	0.034	1.008E-157
Abcb10	1.842E-161	2.87407263	0.059	0.014	4.042E-157
Rere	1.903E-161	1.91563372	0.126	0.053	4.176E-157
Pnisr	1.099E-160	0.93901501	0.428	0.339	2.411E-156
Map4k1	6.31E-160	1.98987044	0.148	0.07	1.384E-155
Chst15	1.524E-159	2.60799625	0.062	0.016	3.343E-155
Matr3	1.548E-159	0.8520026	0.482	0.41	3.396E-155
Pold1	1.163E-158	2.71476698	0.065	0.017	2.551E-154
Slc9a7	5.64E-155	3.41738426	0.041	0.007	1.237E-150
Tia1	9.658E-155	1.29048236	0.253	0.156	2.119E-150
D1Ertd622e	6.788E-154	1.91030112	0.141	0.066	1.489E-149
Sp110	2.971E-150	1.3681043	0.266	0.173	6.518E-146
Bhlhe41	5.281E-150	2.58918381	0.05	0.011	1.159E-145
Gm20506	9.407E-150	4.37254394	0.033	0.004	2.064E-145
Cnot6l	1.296E-149	0.83643644	0.459	0.375	2.844E-145
AC168090.1	4.07E-148	6.05960454	0.018	0.001	8.929E-144
Neil1	5.402E-148	1.76417606	0.113	0.046	1.185E-143
4930426D05R	8.99E-148	6.56260376	0.017	0	1.972E-143
Luc7l2	1.866E-147	0.54528189	0.669	0.661	4.094E-143
Hist1h1e	2.655E-147	0.96591553	0.326	0.225	5.824E-143
Bri3	6.781E-147	0.78928964	0.492	0.419	1.488E-142
P2rx4	2.839E-146	1.52418296	0.248	0.16	6.23E-142
Rcc2	5.335E-146	1.38407567	0.237	0.146	1.17E-141
Tnfrsf13b	2.213E-145	2.03805333	0.119	0.051	4.854E-141
Bptf	6.359E-143	1.01275681	0.372	0.283	1.395E-138
Zfp106	9.073E-143	1.2012343	0.348	0.267	1.99E-138
Gm34215	1.55E-142	5.09396881	0.021	0.001	3.4E-138
Csnk1g3	2.422E-142	1.21586882	0.279	0.186	5.314E-138
Baz2b	3.915E-142	1.32251296	0.268	0.18	8.59E-138
Cdh17	4.789E-142	3.67169741	0.028	0.003	1.051E-137
Crebrf	6.366E-141	0.97683247	0.368	0.276	1.397E-136
Clk1	4.654E-140	0.45197774	0.702	0.679	1.021E-135
Snx2	1.843E-139	1.42040122	0.239	0.152	4.043E-135
Zfyve26	1.964E-138	1.82789908	0.102	0.041	4.309E-134
Sh3bp5	3.77E-138	1.41644787	0.159	0.081	8.272E-134
Gns	5.694E-138	1.26907259	0.27	0.182	1.249E-133
Srsf1	6.859E-137	1.18334849	0.264	0.172	1.505E-132
Shank1	1.101E-135	4.87573181	0.02	0.001	2.416E-131
Nr4a1	1.416E-135	0.79075952	0.438	0.345	3.107E-131
Apobec3	6.854E-135	1.39599816	0.237	0.15	1.504E-130
Kcnb1	1.233E-134	4.92145221	0.019	0.001	2.705E-130
2010007H06R	7.44E-134	5.64316878	0.018	0.001	1.632E-129
Tec	1.161E-133	1.56905976	0.163	0.086	2.547E-129
Ubap2l	3.938E-133	0.98620745	0.404	0.334	8.64E-129
Snx9	8.191E-132	1.67169175	0.185	0.107	1.797E-127
Slc44a2	8.327E-131	1.6851312	0.138	0.068	1.827E-126

Mef2b	7.235E-130	4.75729669	0.019	0.001	1.587E-125
Ncl	8.7E-129	0.5294454	0.775	0.81	1.909E-124
Rgs13	9.47E-129	5.18618297	0.016	0	2.078E-124
Slc38a9	1.156E-128	1.83451519	0.135	0.067	2.536E-124
Add3	2.333E-128	1.43070472	0.196	0.114	5.119E-124
Ttpal	2.659E-125	1.59937459	0.171	0.095	5.834E-121
Lars2	1.233E-123	0.42386891	0.728	0.734	2.706E-119
Atf7ip	1.04E-122	1.06432509	0.277	0.189	2.281E-118
Tmem245	1.041E-122	1.66283556	0.122	0.058	2.284E-118
Snrpg	1.531E-121	0.58141354	0.598	0.585	3.359E-117
Clec2i	1.676E-121	1.58948638	0.124	0.059	3.676E-117
Gm4610	2.991E-121	3.66501568	0.021	0.002	6.562E-117
Aicda	3.312E-121	6.31004586	0.014	0	7.267E-117
Ctso	7.218E-121	1.61989981	0.139	0.071	1.583E-116
Ppp1r16b	8.051E-119	0.6988016	0.404	0.304	1.766E-114
Hnrnpu	8.652E-119	0.65225834	0.552	0.529	1.898E-114
Plcg2	9.489E-119	1.83628698	0.153	0.084	2.082E-114
Zcchc7	1.181E-118	0.93169693	0.359	0.276	2.591E-114
Myo1c	7.787E-118	1.87020671	0.137	0.071	1.708E-113
Pten	1.263E-117	0.99275242	0.369	0.299	2.771E-113
Helz2	1.456E-117	1.49942378	0.153	0.082	3.193E-113
Sec24a	1.484E-117	1.60491677	0.177	0.103	3.256E-113
Snhg12	6.603E-117	0.90111901	0.355	0.274	1.449E-112
Cdk5r1	1.069E-115	2.01183836	0.062	0.02	2.345E-111
Hnrnpa3	4.688E-115	0.41151942	0.747	0.747	1.029E-110
Map3k1	2.001E-114	0.90688047	0.395	0.329	4.389E-110
Fam111a	1.33E-112	1.26156259	0.187	0.111	2.918E-108
Arhgap30	1.439E-112	0.96812832	0.362	0.288	3.158E-108
Rubcnl	3.624E-112	2.61118716	0.062	0.021	7.951E-108
Hnrnpa2b1	7.861E-112	0.38603314	0.753	0.773	1.725E-107
Slc15a4	4.25E-111	1.96509643	0.106	0.049	9.325E-107
Tmem163	5.099E-110	2.60127796	0.041	0.01	1.119E-105
Arhgef1	2.745E-109	0.887422	0.36	0.282	6.023E-105
Dhx9	4.773E-109	1.02943162	0.32	0.244	1.047E-104
Crisp3	5.582E-109	6.30714873	0.014	0	1.225E-104
Ppwd1	2.404E-108	1.23847909	0.225	0.148	5.274E-104
Whrn	4.233E-108	2.54169892	0.037	0.008	9.286E-104
Txnip	1.096E-107	0.7139425	0.311	0.221	2.405E-103
Gm15848	1.742E-107	7.38091141	0.012	0	3.821E-103
Cdt1	2.576E-107	1.5146039	0.141	0.076	5.651E-103
Gm30239	2.909E-107	8.7850008	0.012	0	6.381E-103
Ddx25	3.827E-107	3.049729	0.028	0.005	8.396E-103
Samd9l	2.475E-106	1.21745159	0.189	0.115	5.43E-102
Myl4	4.293E-106	2.58903734	0.043	0.011	9.418E-102
Klhl14	1.307E-105	7.39007596	0.012	0	2.867E-101
Il5ra	1.175E-104	5.20185654	0.015	0.001	2.578E-100
Gm13383	3.656E-104	4.90348875	0.017	0.001	8.022E-100
Polr1a	1.98E-102	1.75292788	0.103	0.048	4.3443E-98

Rapgef4	3.157E-102	2.66069791	0.028	0.005	6.9263E-98
Fam213a	5.619E-102	1.96266569	0.073	0.028	1.2329E-97
Ssbp1	1.341E-101	1.4247759	0.19	0.12	2.9418E-97
Scai	5.226E-101	1.67377904	0.081	0.034	1.1465E-96
Hist1h1c	1.027E-100	1.25423138	0.293	0.219	2.2526E-96
Irf2bp2	1.39E-100	0.75152405	0.468	0.415	3.0486E-96
Gm47230	1.49E-100	2.93795702	0.038	0.01	3.2691E-96
Ddx18	7.064E-100	1.21467079	0.248	0.174	1.5499E-95
Trim34a	1.052E-99	1.70079608	0.079	0.032	2.3087E-95
Arid3b	1.751E-99	1.90289132	0.067	0.025	3.8406E-95
Fam129c	4.089E-99	3.6193938	0.02	0.002	8.9703E-95
Tmed8	1.0801E-98	1.8921809	0.069	0.026	2.3695E-94
Cebpz	1.9331E-98	0.82756261	0.398	0.335	4.2411E-94
St6gal1	2.2438E-98	1.72401185	0.111	0.055	4.9227E-94
Set	2.2705E-98	0.68497214	0.525	0.508	4.9813E-94
Shisa5	2.0797E-97	0.51175266	0.609	0.587	4.5626E-93
Acox3	4.3431E-97	1.77622339	0.121	0.064	9.5283E-93
C130026I21Ri	4.6107E-96	2.21716195	0.079	0.034	1.0115E-91
Gm15987	1.7874E-95	2.32119355	0.071	0.029	3.9214E-91
Tra2b	2.5757E-95	0.54567201	0.594	0.594	5.6509E-91
Ktn1	7.0536E-95	0.98548896	0.284	0.211	1.5475E-90
Tardbp	1.0749E-92	0.91777372	0.344	0.28	2.3583E-88
Zfp280d	5.8888E-92	1.29057445	0.168	0.102	1.2919E-87
Pcmtd1	2.9289E-91	1.08444137	0.26	0.189	6.4257E-87
Eaf2	8.3969E-91	2.20964855	0.051	0.017	1.8422E-86
Cnn3	1.1374E-90	2.03345224	0.079	0.035	2.4952E-86
Mast4	1.7412E-90	0.98080102	0.239	0.164	3.82E-86
Ptprcap	6.614E-90	0.74766939	0.273	0.19	1.4511E-85
Sh2b3	3.4117E-89	1.57513441	0.152	0.091	7.485E-85
Pde7a	4.0656E-89	0.79775366	0.286	0.206	8.9196E-85
Rcsd1	4.5076E-89	1.14194308	0.15	0.087	9.8892E-85
Trim59	7.5107E-89	1.2231029	0.161	0.096	1.6478E-84
Sema5a	9.2374E-89	3.76190655	0.015	0.001	2.0266E-84
Ndufaf4	1.3695E-88	1.44811132	0.126	0.069	3.0047E-84
Stac2	2.2622E-87	3.84970282	0.018	0.002	4.963E-83
Gm15326	6.5302E-87	3.46400929	0.022	0.004	1.4327E-82
Cfap43	8.6417E-86	1.48922445	0.115	0.061	1.8959E-81
Slc30a5	1.224E-85	1.42032343	0.142	0.083	2.6853E-81
Fam208b	1.3542E-85	1.2692547	0.161	0.099	2.971E-81
Vars	3.3217E-85	1.41774395	0.167	0.105	7.2874E-81
Gm43388	1.3522E-84	5.9404662	0.012	0.001	2.9667E-80
Prpf38b	3.0715E-84	0.65238881	0.498	0.475	6.7385E-80
2700038G22R	1.4485E-83	2.29647503	0.042	0.013	3.1779E-79
Ilf2	2.7749E-83	1.09535335	0.235	0.168	6.0879E-79
Nucks1	3.0064E-83	0.65621463	0.465	0.427	6.5957E-79
Man1a	1.3967E-82	0.82692558	0.325	0.26	3.0643E-78
Smyd5	1.5112E-82	2.18401372	0.049	0.017	3.3154E-78
N4bp2l2	6.1079E-82	0.67687068	0.439	0.394	1.34E-77

Ung	1.1704E-81	2.44028314	0.033	0.008	2.5677E-77
Slc38a1	1.0315E-80	0.67626703	0.443	0.4	2.263E-76
Plekho1	1.5387E-80	1.45591533	0.177	0.117	3.3758E-76
Lamb3	1.5534E-80	1.39584149	0.081	0.038	3.4079E-76
Nol8	2.5016E-80	1.44131651	0.128	0.073	5.4882E-76
Taz	4.2966E-80	1.50740092	0.131	0.076	9.4262E-76
Iqcb1	5.7158E-79	2.00863287	0.06	0.024	1.254E-74
Fam214a	7.3184E-79	1.43759962	0.108	0.058	1.6056E-74
Tcof1	1.0144E-78	1.18372324	0.184	0.122	2.2254E-74
Trmt1	4.0008E-77	1.53951439	0.126	0.073	8.7773E-73
Timm9	7.0076E-77	1.59579743	0.086	0.042	1.5374E-72
Twsg1	2.1466E-76	1.38131495	0.107	0.058	4.7095E-72
R3hdm1	2.3184E-76	0.94926632	0.29	0.229	5.0863E-72
Fam169b	3.139E-76	1.07363723	0.143	0.085	6.8867E-72
Acsl3	1.1638E-75	1.87833316	0.07	0.031	2.5533E-71
Trim25	1.2276E-75	0.97100849	0.277	0.216	2.6933E-71
Bmyc	2.6869E-75	1.59065089	0.079	0.037	5.8947E-71
Gatad2b	6.0915E-75	0.95965565	0.303	0.246	1.3364E-70
Snx8	3.4975E-74	1.77656502	0.101	0.054	7.6731E-70
Eya1	1.1222E-73	2.51578277	0.028	0.007	2.462E-69
1110059E24R	1.4948E-73	1.76342324	0.077	0.037	3.2794E-69
9430038I01Ril	5.6655E-73	1.79533155	0.095	0.05	1.243E-68
Rabep2	6.8872E-73	1.75041646	0.07	0.032	1.511E-68
Tfam	8.7875E-73	1.32298897	0.14	0.085	1.9279E-68
Tk2	1.3081E-72	1.57700351	0.077	0.037	2.8698E-68
Mirt1	1.4339E-72	0.93500598	0.125	0.071	3.1458E-68
Rasgrp2	1.6435E-72	1.21410952	0.155	0.098	3.6057E-68
Cd2ap	5.8885E-72	1.29102523	0.212	0.155	1.2919E-67
Rpl22l1	6.8885E-72	0.3462612	0.716	0.746	1.5113E-67
Sh3pxd2a	2.3835E-71	1.93543296	0.077	0.037	5.2293E-67
Cse1l	3.4356E-71	1.3713373	0.146	0.092	7.5374E-67
Haao	4.7009E-71	3.25810522	0.029	0.008	1.0313E-66
Xylt1	3.3244E-70	1.19115876	0.133	0.08	7.2935E-66
Plekhm3	3.8369E-70	1.794896	0.097	0.052	8.4178E-66
Itsn2	3.9922E-70	0.91862636	0.314	0.262	8.7585E-66
Arid4b	8.8862E-70	0.80792837	0.359	0.31	1.9495E-65
AC133103.1	2.2717E-69	4.11522922	0.012	0.001	4.984E-65
Utp14b	2.6619E-69	1.91007655	0.06	0.026	5.84E-65
Crb3	2.9051E-69	3.20973631	0.018	0.003	6.3735E-65
Lrmp	4.6964E-69	1.52623941	0.133	0.082	1.0303E-64
Galnt11	1.874E-68	1.12856936	0.195	0.137	4.1113E-64
Tsr1	3.7973E-68	1.66407454	0.082	0.041	8.3309E-64
Hdac9	5.9264E-68	2.38721176	0.038	0.013	1.3002E-63
Slc20a2	6.7342E-68	1.88812973	0.057	0.024	1.4774E-63
Ms4a4c	9.4501E-68	1.3008107	0.156	0.103	2.0733E-63
Csprs	1.9773E-66	2.88257023	0.02	0.004	4.338E-62
Mybbp1a	2.4226E-65	1.18149628	0.221	0.167	5.3149E-61
Arhgap17	4.635E-65	0.94182841	0.269	0.214	1.0169E-60

Dnajc7	1.9123E-64	1.10185926	0.236	0.182	4.1953E-60
Rnf144a	5.8794E-64	1.36292835	0.065	0.03	1.2899E-59
Mreg	6.2531E-64	2.40648613	0.029	0.008	1.3719E-59
Gripap1	6.8368E-64	1.26326717	0.182	0.129	1.4999E-59
Prpf39	6.9746E-64	0.95681335	0.266	0.212	1.5302E-59
Nfkbid	9.0738E-64	1.06440958	0.183	0.126	1.9907E-59
Avl9	2.2061E-63	0.83941718	0.279	0.222	4.84E-59
Gm11738	2.7451E-63	4.33775426	0.01	0.001	6.0224E-59
Ftx	6.2732E-63	2.61149242	0.022	0.005	1.3763E-58
Kcnq1ot1	6.4443E-63	0.97775958	0.182	0.124	1.4138E-58
Rasal1	8.2065E-63	3.4348583	0.012	0.001	1.8004E-58
Bclaf1	2.577E-62	0.5569383	0.505	0.497	5.6536E-58
Mbd4	2.6214E-62	2.08515792	0.037	0.013	5.751E-58
Arhgef18	4.935E-62	1.43805181	0.068	0.033	1.0827E-57
Gm15675	1.1267E-61	3.69025843	0.012	0.001	2.4718E-57
Mga	1.2957E-61	1.04724935	0.202	0.146	2.8426E-57
Grap	1.3753E-61	0.93947692	0.179	0.122	3.0174E-57
Rnase6	1.5767E-61	1.90719459	0.099	0.057	3.4591E-57
Trim12a	1.703E-61	0.9535058	0.179	0.123	3.7363E-57
1700017B05R	7.4921E-61	1.33108407	0.122	0.075	1.6437E-56
Rnf122	1.0618E-60	2.22377606	0.034	0.011	2.3296E-56
Slc15a2	2.3951E-60	2.07676814	0.047	0.019	5.2546E-56
AC139671.1	2.7024E-60	2.0527392	0.027	0.008	5.9289E-56
Gpr18	2.8784E-60	1.08108504	0.132	0.082	6.3149E-56
Ppp3cc	3.0751E-60	1.3383335	0.089	0.048	6.7465E-56
Atp2a3	3.5864E-60	1.11708627	0.141	0.09	7.8683E-56
Cbx7	8.7373E-60	1.31568528	0.079	0.041	1.9169E-55
Mgrn1	1.5917E-59	1.55806099	0.104	0.061	3.4921E-55
Trmt10a	2.3918E-59	1.88367222	0.056	0.025	5.2474E-55
Traf5	3.1476E-59	1.78895746	0.072	0.036	6.9055E-55
Gm39321	1.7469E-58	1.84167554	0.018	0.004	3.8326E-54
Strada	2.0776E-58	1.7845457	0.059	0.027	4.5579E-54
Gm10138	3.0265E-58	1.74686275	0.067	0.033	6.6399E-54
Mdn1	4.1681E-58	1.21665239	0.163	0.112	9.1443E-54
Gadd45a	9.3376E-58	1.04489756	0.104	0.06	2.0486E-53
Sash3	9.8762E-58	1.23960305	0.098	0.056	2.1667E-53
Lncpint	1.0015E-57	0.9879845	0.211	0.157	2.1973E-53
Uba52	2.5239E-57	0.51639523	0.514	0.521	5.5371E-53
Mycbp2	5.4371E-57	0.6227256	0.477	0.475	1.1928E-52
Lpgat1	1.1958E-56	1.57192543	0.113	0.07	2.6235E-52
Ift140	1.6826E-56	2.43691993	0.032	0.011	3.6914E-52
Slamf6	1.436E-55	1.02135522	0.13	0.082	3.1504E-51
Ago2	2.177E-55	0.69937902	0.45	0.44	4.7761E-51
Pikfyve	5.617E-55	1.4735812	0.098	0.057	1.2323E-50
Depdc5	4.2128E-54	1.70909034	0.051	0.023	9.2425E-50
Zc3h12d	4.4469E-54	1.25719239	0.081	0.043	9.7561E-50
Ppan	5.3404E-54	1.4523581	0.083	0.046	1.1716E-49
Bicral	7.3727E-54	1.63930098	0.067	0.034	1.6175E-49

Uvrag	9.359E-54	1.08987736	0.244	0.2	2.0533E-49
Sgms1	1.3688E-53	1.07168657	0.176	0.126	3.003E-49
Pigr	2.3639E-53	2.60303515	0.012	0.002	5.1862E-49
Spns2	2.49E-53	3.34332265	0.013	0.002	5.4629E-49
Ppm1g	3.2534E-53	0.97843259	0.244	0.195	7.1376E-49
Gm26917	4.9936E-53	0.37118159	0.393	0.345	1.0956E-48
Prmt5	6.4879E-53	1.58169165	0.068	0.035	1.4234E-48
Dkc1	7.9903E-53	1.33234209	0.081	0.045	1.753E-48
Ctsh	9.4411E-53	0.63614397	0.312	0.257	2.0713E-48
Pdzd2	9.6086E-53	3.21147661	0.012	0.002	2.108E-48
Cdc7	1.9134E-52	1.70775956	0.037	0.014	4.1979E-48
Ap1s3	4.1431E-52	1.54958131	0.084	0.048	9.0896E-48
Rasd1	5.8174E-52	2.70967194	0.017	0.004	1.2763E-47
Aff4	9.0755E-52	0.69537054	0.374	0.343	1.9911E-47
Arhgap15	9.1723E-52	0.81896185	0.282	0.234	2.0123E-47
Xkrx	9.8265E-52	3.52929618	0.01	0.001	2.1558E-47
Gdap10	9.9672E-52	1.15755116	0.132	0.087	2.1867E-47
Zbtb20	1.873E-51	0.80416211	0.244	0.19	4.1093E-47
Rrm2b	2.5723E-51	1.23877047	0.099	0.059	5.6434E-47
Otud7b	3.0347E-51	1.32014653	0.073	0.039	6.6578E-47
Sfn	4.0161E-51	1.63314078	0.046	0.02	8.8109E-47
Zfp169	4.2469E-51	1.87393221	0.039	0.015	9.3173E-47
Ighg3	5.0612E-51	3.90476632	0.012	0.002	1.1104E-46
Apex1	6.7994E-51	1.55452069	0.092	0.054	1.4917E-46
Ceacam16	7.3061E-51	2.29790958	0.021	0.006	1.6029E-46
Irf2	3.5406E-50	0.85288888	0.296	0.254	7.7677E-46
Cpsf6	9.6937E-50	0.8193596	0.267	0.22	2.1267E-45
Cyfip2	9.9651E-50	0.81167171	0.265	0.216	2.1862E-45
9930111J21Ril	1.3688E-49	0.90883225	0.166	0.116	3.003E-45
Rnf216	2.0453E-49	1.44593879	0.11	0.07	4.4872E-45
Ttc14	2.5918E-49	0.63410646	0.382	0.349	5.6862E-45
Foxo1	3.0208E-49	0.53636371	0.352	0.302	6.6273E-45
Trim44	3.0478E-49	1.08731526	0.163	0.116	6.6865E-45
Cab39l	3.7936E-49	1.51564451	0.088	0.051	8.3227E-45
Rassf2	2.1159E-48	1.18015	0.166	0.121	4.642E-44
Fry	2.2607E-48	1.78992247	0.047	0.021	4.9597E-44
Mef2d	3.3843E-48	0.79778871	0.313	0.276	7.4249E-44
Slf1	3.9591E-48	1.62030961	0.055	0.027	8.686E-44
Vav2	5.27E-48	1.95503133	0.061	0.031	1.1562E-43
Msn	5.6262E-48	0.35415576	0.601	0.624	1.2343E-43
Son	1.6329E-47	0.30287956	0.683	0.729	3.5825E-43
Jade1	1.7897E-47	1.15284896	0.091	0.054	3.9265E-43
Mapk11	2.8199E-47	3.07444696	0.014	0.003	6.1866E-43
Hook1	2.8979E-47	1.57665241	0.051	0.024	6.3577E-43
Tra2a	3.2337E-47	0.44188239	0.528	0.537	7.0944E-43
Srebf2	3.7997E-47	0.78009961	0.336	0.306	8.3362E-43
2810004N23R	4.5854E-47	1.38672915	0.101	0.063	1.006E-42
Ddx21	5.4056E-47	0.71419677	0.437	0.437	1.1859E-42

Hsph1	1.5344E-46	0.93602563	0.214	0.164	3.3662E-42
Xndc1	1.9645E-46	1.70295125	0.036	0.014	4.31E-42
C1galt1	1.9794E-46	1.1587114	0.114	0.074	4.3425E-42
Mettl16	2.242E-46	1.30175927	0.098	0.06	4.9188E-42
Dusp4	2.6413E-46	0.80305825	0.174	0.125	5.7946E-42
Slco4a1	7.35E-46	2.74336412	0.015	0.003	1.6125E-41
Abhd17b	9.6087E-46	1.01850698	0.204	0.158	2.108E-41
Gm16152	1.149E-45	3.08158577	0.012	0.002	2.5208E-41
Zdhhc13	1.8412E-45	1.73136571	0.05	0.024	4.0393E-41
Nfatc1	3.1847E-45	0.71501909	0.217	0.166	6.987E-41
Zyg11b	3.3528E-45	1.11987422	0.152	0.108	7.3557E-41
Mtdh	4.067E-45	0.58476488	0.45	0.448	8.9225E-41
Nrm	7.0931E-45	1.70074746	0.071	0.04	1.5562E-40
Map3k9	1.0582E-44	3.15821214	0.015	0.003	2.3216E-40
Lmbrd1	1.3003E-44	1.10419335	0.198	0.156	2.8527E-40
Hhex	1.4316E-44	1.88466845	0.047	0.022	3.1409E-40
Zfp1	1.5381E-44	1.41452308	0.064	0.034	3.3745E-40
Ifi213	1.7486E-44	1.33129885	0.068	0.037	3.8364E-40
Arpc5l	2.9616E-44	1.3148791	0.167	0.126	6.4974E-40
Tcp11l2	3.4641E-44	0.76024132	0.258	0.212	7.6E-40
Prkd2	3.6818E-44	1.38343053	0.062	0.032	8.0775E-40
Abce1	3.6942E-44	1.10547411	0.187	0.143	8.1048E-40
Hnrnpa1	4.4653E-44	0.42970644	0.56	0.591	9.7965E-40
Iqsec1	4.8483E-44	1.18384635	0.118	0.078	1.0637E-39
Rock2	1.0607E-43	0.89557095	0.253	0.212	2.3271E-39
Invs	1.7544E-43	2.0440292	0.029	0.011	3.849E-39
Tcf3	2.6295E-43	1.28036999	0.085	0.05	5.7689E-39
Hnrnpf	4.1139E-43	0.41519987	0.563	0.601	9.0254E-39
Bcl9	8.7886E-43	2.18796005	0.036	0.015	1.9281E-38
BC051142	1.9161E-42	3.67693103	0.01	0.002	4.2038E-38
Lgals8	2.4138E-42	1.21413817	0.131	0.092	5.2957E-38
Pou6f1	2.8676E-42	1.38438352	0.05	0.025	6.2913E-38
Nup133	4.5938E-42	1.88215246	0.046	0.022	1.0078E-37
Gpd1l	4.8285E-42	1.58596504	0.062	0.034	1.0593E-37
Stoml1	5.3871E-42	2.05415844	0.033	0.014	1.1819E-37
3300005D01R	5.5662E-42	2.66322942	0.019	0.006	1.2212E-37
Csk	6.4921E-42	0.98460457	0.217	0.177	1.4243E-37
Prmt3	8.5302E-42	1.45331871	0.069	0.039	1.8714E-37
Hsh2d	1.4029E-41	2.11408573	0.021	0.006	3.0777E-37
Ciart	1.9229E-41	2.08159218	0.029	0.011	4.2186E-37
Smchd1	2.6046E-41	0.50349161	0.457	0.442	5.7143E-37
Ighg1	2.8085E-41	0.93041486	0.16	0.118	6.1616E-37
1500015A07R	4.0044E-41	1.39285172	0.082	0.049	8.7853E-37
Trim5	4.2741E-41	1.64941392	0.039	0.018	9.377E-37
Gm15706	5.2853E-41	2.57849366	0.02	0.006	1.1595E-36
Mtf2	5.5906E-41	0.96571083	0.175	0.132	1.2265E-36
Rfx7	8.5919E-41	1.09211459	0.133	0.093	1.885E-36
Gigyf1	1.2587E-40	0.94247006	0.166	0.124	2.7614E-36

Hist1h2bc	2.0629E-40	1.55083304	0.119	0.081	4.5258E-36
Hip1r	2.2629E-40	0.80941127	0.171	0.127	4.9646E-36
Zfp644	1.0658E-39	0.81477763	0.247	0.206	2.3383E-35
Mybl1	1.1771E-39	2.46085316	0.014	0.003	2.5825E-35
C1qbp	1.4919E-39	1.11444585	0.199	0.161	3.2732E-35
Dclk2	1.8545E-39	2.15515197	0.024	0.009	4.0686E-35
Mettl1	2.115E-39	1.74595833	0.077	0.047	4.6401E-35
Upf3b	2.8246E-39	1.10593508	0.117	0.079	6.1968E-35
Prr5	2.9431E-39	1.61171328	0.04	0.019	6.4568E-35
Fpgs	3.0969E-39	1.9749659	0.034	0.015	6.7944E-35
Polr1b	4.132E-39	2.06080215	0.024	0.009	9.0651E-35
Adgre5	5.8124E-39	0.65308979	0.366	0.343	1.2752E-34
Med7	7.4406E-39	1.217535	0.113	0.077	1.6324E-34
Rbm26	7.9448E-39	0.91671789	0.226	0.187	1.743E-34
Fam168b	8.1819E-39	0.85223243	0.255	0.22	1.795E-34
Ptbp3	1.0489E-38	0.34237662	0.612	0.655	2.3011E-34
Mapk12	1.1466E-38	2.80708376	0.012	0.003	2.5156E-34
Ptk2b	1.7143E-38	0.75738247	0.267	0.229	3.7609E-34
Unc93b1	1.9145E-38	0.45128655	0.315	0.267	4.2001E-34
Rev1	2.0393E-38	1.42709303	0.073	0.043	4.4739E-34
Tfeb	2.4458E-38	1.73938133	0.046	0.023	5.3659E-34
Tsc22d2	3.1655E-38	0.95831438	0.183	0.142	6.9449E-34
Bicra	3.265E-38	1.38660426	0.053	0.028	7.163E-34
Rpia	3.3112E-38	1.31554547	0.088	0.056	7.2645E-34
Hmgb1	4.1115E-38	0.29268978	0.638	0.67	9.0201E-34
Map4k5	4.358E-38	1.84064518	0.045	0.022	9.561E-34
Kmo	5.2807E-38	2.15931302	0.05	0.026	1.1585E-33
Tmem209	6.2178E-38	1.417414	0.062	0.034	1.3641E-33
Tex10	1.3395E-37	1.12656792	0.138	0.1	2.9387E-33
Atp6v0a1	1.7942E-37	1.46694659	0.073	0.044	3.9362E-33
BC017643	2.6787E-37	1.2663137	0.118	0.083	5.8768E-33
Kdm5a	2.7642E-37	0.65042381	0.341	0.313	6.0644E-33
Immp2l	3.2413E-37	2.29233846	0.031	0.013	7.1112E-33
Brwd1	4.5304E-37	1.15639763	0.134	0.097	9.9393E-33
Ahctf1	5.0959E-37	0.91471141	0.202	0.162	1.118E-32
Brd8	5.3711E-37	0.82921628	0.242	0.204	1.1784E-32
St8sia4	6.4544E-37	0.62592028	0.351	0.319	1.416E-32
Kcnc3	9.4249E-37	2.95129315	0.01	0.002	2.0677E-32
Erlin1	1.1476E-36	1.61115925	0.103	0.07	2.5177E-32
Jmjd1c	1.658E-36	0.50804087	0.436	0.427	3.6374E-32
Znhit6	1.8006E-36	1.37057438	0.063	0.036	3.9503E-32
Smc5	2.1492E-36	1.28985629	0.111	0.076	4.7152E-32
Nop10	2.1877E-36	0.74886897	0.339	0.322	4.7997E-32
Ighg2b	5.7783E-36	1.37035898	0.106	0.072	1.2677E-31
Clasp1	6.4101E-36	1.33436924	0.092	0.06	1.4063E-31
Uchl3	7.2287E-36	1.33394251	0.102	0.069	1.5859E-31
Nuggc	8.5676E-36	2.60186247	0.011	0.002	1.8796E-31
Smad7	1.5142E-35	0.61506047	0.342	0.312	3.3221E-31

Zfp870	1.7871E-35	2.84763991	0.011	0.002	3.9208E-31
Ankrd33b	2.14E-35	0.62105956	0.103	0.068	4.6949E-31
Atf7	3.3303E-35	1.27530411	0.1	0.067	7.3064E-31
Pus7	3.3574E-35	1.62420939	0.046	0.024	7.3658E-31
Oard1	7.2936E-35	1.1406234	0.095	0.062	1.6001E-30
Kctd14	1.0105E-34	2.73019652	0.019	0.006	2.217E-30
Zfp831	1.0136E-34	1.51523039	0.031	0.013	2.2238E-30
Ubqln1	1.4369E-34	1.01918429	0.188	0.151	3.1523E-30
Xpr1	2.0795E-34	1.03976327	0.161	0.124	4.5623E-30
Senp7	2.2498E-34	1.15961784	0.09	0.058	4.9359E-30
Hps3	2.3149E-34	1.15398392	0.117	0.082	5.0786E-30
Setd2	2.8607E-34	0.77234084	0.256	0.221	6.276E-30
Dcaf17	4.6044E-34	1.30678171	0.053	0.029	1.0102E-29
Pkn1	5.992E-34	0.8849899	0.246	0.215	1.3146E-29
Bmf	8.3082E-34	2.03214964	0.023	0.009	1.8227E-29
Srrm2	8.9439E-34	0.25210111	0.685	0.736	1.9622E-29
1700030K09R	8.9823E-34	2.60150629	0.013	0.004	1.9706E-29
Rbm5	9.3102E-34	0.64759163	0.37	0.359	2.0426E-29
Eml4	1.5351E-33	0.84290008	0.236	0.201	3.3678E-29
Dhx30	1.8519E-33	1.09048392	0.126	0.091	4.0629E-29
Emid1	2.0216E-33	1.55650648	0.024	0.009	4.4353E-29
Gopc	2.6298E-33	1.29836687	0.07	0.042	5.7696E-29
Kansl1	3.5274E-33	0.62466926	0.327	0.3	7.7389E-29
Thumpd3	3.8307E-33	1.31735171	0.083	0.053	8.4041E-29
Susd1	4.6463E-33	1.87838412	0.027	0.011	1.0194E-28
Ccnt2	6.2527E-33	0.81843022	0.227	0.192	1.3718E-28
Fam208a	7.0555E-33	0.99014269	0.163	0.126	1.5479E-28
Ctcf	7.9089E-33	0.66042199	0.304	0.274	1.7351E-28
Hspd1	9.7161E-33	0.76332736	0.347	0.332	2.1316E-28
Ubr1	1.2645E-32	1.07602501	0.094	0.062	2.7742E-28
Slc25a36	1.4284E-32	0.74913282	0.225	0.187	3.1339E-28
Trim26	1.5315E-32	0.90073294	0.167	0.13	3.36E-28
Filip1l	1.5885E-32	1.28088162	0.142	0.109	3.4849E-28
Cwf19l2	1.9534E-32	1.0051769	0.149	0.113	4.2855E-28
Zfp157	2.8029E-32	1.53504567	0.038	0.019	6.1492E-28
Pip5k1b	5.7179E-32	1.74941814	0.019	0.007	1.2544E-27
Washc4	8.8662E-32	1.04835072	0.154	0.119	1.9452E-27
Gart	1.769E-31	1.40487908	0.049	0.027	3.881E-27
Xk	1.8082E-31	2.28662843	0.011	0.003	3.9669E-27
Rasa3	2.807E-31	1.00521771	0.138	0.103	6.1583E-27
Patz1	3.938E-31	1.4917285	0.039	0.02	8.6395E-27
Nsa2	5.4706E-31	0.28600229	0.651	0.698	1.2002E-26
4930480K23R	5.5559E-31	2.024196	0.018	0.006	1.2189E-26
Rufy2	6.0283E-31	1.17374468	0.077	0.049	1.3225E-26
Ncf1	6.0285E-31	0.3958761	0.209	0.171	1.3226E-26
Zufsp	6.5983E-31	0.80937653	0.253	0.22	1.4476E-26
Dph5	7.9707E-31	1.04997238	0.122	0.088	1.7487E-26
Abi3	9.9215E-31	1.45040937	0.068	0.042	2.1767E-26

Acss1	9.9579E-31	1.52220996	0.063	0.038	2.1847E-26
Arid1a	1.0625E-30	0.75805085	0.228	0.193	2.331E-26
Icosl	1.5573E-30	1.55567207	0.067	0.042	3.4165E-26
Dennd6a	2.8047E-30	1.3235333	0.09	0.061	6.1532E-26
4933433G15R	4.639E-30	1.88855169	0.028	0.013	1.0177E-25
Lat2	5.1638E-30	1.21734971	0.13	0.098	1.1329E-25
Pwp2	5.9341E-30	1.73267508	0.034	0.016	1.3019E-25
Grpel2	6.9344E-30	1.33816065	0.066	0.04	1.5213E-25
Nolc1	9.2217E-30	1.00422292	0.178	0.145	2.0231E-25
Sp1	1.0692E-29	0.93293864	0.185	0.152	2.3457E-25
Jakmip1	1.1067E-29	0.6855479	0.175	0.136	2.4279E-25
Snap29	1.2882E-29	1.18934945	0.11	0.079	2.8262E-25
Mical3	1.3483E-29	1.76254381	0.022	0.009	2.958E-25
Rras2	1.4092E-29	0.96897971	0.094	0.064	3.0916E-25
Gpatch4	1.6839E-29	1.65951236	0.043	0.023	3.6942E-25
Lztfl1	1.707E-29	1.07016202	0.201	0.171	3.7451E-25
Serpini1	1.7733E-29	1.91907917	0.021	0.008	3.8904E-25
Mettl8	1.8622E-29	1.96374928	0.019	0.007	4.0854E-25
Rrn3	2.11E-29	1.36366694	0.074	0.048	4.6291E-25
Insig2	2.7612E-29	1.15466383	0.077	0.049	6.0578E-25
Akap2	2.9618E-29	2.08573869	0.012	0.003	6.4979E-25
Ddx50	4.6788E-29	0.68429323	0.325	0.309	1.0265E-24
Dicer1	4.9026E-29	0.85588401	0.164	0.129	1.0756E-24
Stk11ip	5.5559E-29	1.5106368	0.052	0.03	1.2189E-24
Helq	5.9969E-29	1.77756452	0.02	0.007	1.3157E-24
Mndal	6.2795E-29	0.77890115	0.289	0.263	1.3777E-24
L3mbtl3	6.7843E-29	1.35464486	0.049	0.027	1.4884E-24
Asxl2	8.6993E-29	0.76702857	0.203	0.168	1.9085E-24
Dnase2a	1.2128E-28	1.2998852	0.112	0.082	2.6608E-24
Rrs1	1.4802E-28	1.35732392	0.063	0.039	3.2473E-24
Mdm4	2.7807E-28	0.74663722	0.215	0.181	6.1006E-24
Tspan32	6.5736E-28	1.11984585	0.061	0.037	1.4422E-23
Kctd3	8.8644E-28	1.54461677	0.042	0.023	1.9448E-23
Camkk1	1.0766E-27	1.88096952	0.018	0.007	2.362E-23
Casp2	1.2246E-27	1.15798748	0.064	0.04	2.6866E-23
Lrif1	1.2628E-27	0.95213351	0.117	0.085	2.7705E-23
Utp20	1.6174E-27	1.38942504	0.056	0.034	3.5484E-23
Pou2f1	1.8954E-27	1.12040137	0.117	0.087	4.1584E-23
Armt1	2.1181E-27	1.37957948	0.044	0.024	4.6469E-23
2510046G10R	2.4158E-27	1.63357768	0.032	0.016	5.3E-23
Atm	2.8779E-27	1.0630648	0.062	0.038	6.3139E-23
Hnrnph3	3.3867E-27	0.92255291	0.158	0.125	7.4301E-23
Map7	3.6459E-27	1.59272848	0.021	0.008	7.9987E-23
Pes1	4.7322E-27	1.18658136	0.121	0.091	1.0382E-22
Bin1	5.1494E-27	1.10873502	0.159	0.128	1.1297E-22
Dnajc27	5.2489E-27	1.53692071	0.034	0.017	1.1516E-22
Adi1	5.3047E-27	1.39400033	0.044	0.025	1.1638E-22
Pabpn1	6.2164E-27	0.85446304	0.176	0.144	1.3638E-22

Tpst1	1.5628E-26	1.89070409	0.039	0.021	3.4287E-22
Gm26669	1.8438E-26	0.83905981	0.145	0.112	4.0452E-22
Nsun6	1.9119E-26	1.07466361	0.086	0.059	4.1944E-22
Phc2	2.3342E-26	0.98005049	0.166	0.135	5.1209E-22
Rmnd5b	2.3774E-26	1.18248985	0.052	0.03	5.2158E-22
Neu1	2.685E-26	0.95815054	0.149	0.118	5.8907E-22
Phf14	3.1726E-26	0.93664238	0.165	0.133	6.9604E-22
Letm2	4.4858E-26	1.04747951	0.069	0.044	9.8414E-22
Mrps18b	5.3153E-26	1.33586861	0.076	0.051	1.1661E-21
Mindy2	5.9635E-26	0.94627241	0.129	0.098	1.3083E-21
Wbp2	6.3217E-26	0.81720284	0.233	0.207	1.3869E-21
Wdr43	8.191E-26	0.83136647	0.225	0.197	1.797E-21
Gramd4	8.5495E-26	1.20215776	0.089	0.062	1.8757E-21
Slc25a25	1.3231E-25	1.19835272	0.075	0.05	2.9027E-21
Gcn1l1	1.4003E-25	1.00100284	0.117	0.087	3.0722E-21
Snhg18	2.2242E-25	1.74998219	0.016	0.006	4.8797E-21
Ankrd49	2.2505E-25	1.1962111	0.105	0.077	4.9373E-21
Dcaf8	2.3068E-25	0.80959821	0.215	0.187	5.0609E-21
Cpm	3.1973E-25	0.68776699	0.088	0.06	7.0146E-21
Eif3a	3.5718E-25	0.32235048	0.593	0.645	7.8362E-21
Adal	5.6246E-25	1.87883999	0.018	0.007	1.234E-20
Gfi1	6.2446E-25	1.15359758	0.05	0.03	1.37E-20
Trmt13	6.6413E-25	1.0098537	0.084	0.057	1.457E-20
Esf1	6.9745E-25	0.87669239	0.169	0.138	1.5301E-20
Cyp39a1	1.5996E-24	2.63284957	0.01	0.003	3.5093E-20
Cdca7	1.7711E-24	1.5623806	0.026	0.012	3.8857E-20
Usp11	2.4355E-24	1.75477389	0.022	0.01	5.3433E-20
Retreg3	2.5146E-24	0.949045	0.108	0.079	5.5167E-20
A430078G23R	2.5923E-24	1.24480156	0.033	0.017	5.6872E-20
Timm8a1	2.7948E-24	1.30892405	0.079	0.054	6.1316E-20
Naa15	3.275E-24	0.75585111	0.242	0.218	7.185E-20
Anapc15	4.0137E-24	1.37062395	0.093	0.067	8.8056E-20
Rpgrip1	4.715E-24	1.24142806	0.065	0.042	1.0344E-19
Arhgap45	5.0732E-24	0.58679043	0.295	0.271	1.113E-19
Grk6	6.6771E-24	0.7804419	0.161	0.129	1.4649E-19
Dip2b	7.3994E-24	0.74715886	0.216	0.188	1.6233E-19
Larp4	8.0927E-24	0.81842563	0.238	0.215	1.7754E-19
Mcm2	1.0736E-23	1.30286691	0.054	0.034	2.3554E-19
Zfp691	1.4154E-23	1.9860283	0.024	0.011	3.1053E-19
Ercc6l2	3.0038E-23	1.22323159	0.054	0.034	6.59E-19
Fam49b	3.2939E-23	0.43685178	0.428	0.44	7.2264E-19
Zfp932	4.6231E-23	1.35262404	0.036	0.019	1.0143E-18
Anp32e	5.5236E-23	0.60668355	0.313	0.299	1.2118E-18
Lax1	6.832E-23	1.04815813	0.053	0.033	1.4989E-18
Trmt61a	6.9718E-23	2.0109052	0.018	0.007	1.5296E-18
Timm10	1.0085E-22	1.29645301	0.102	0.076	2.2125E-18
Tspan13	1.3104E-22	0.40968706	0.43	0.427	2.875E-18
Wdr75	2.0794E-22	1.28560378	0.072	0.049	4.562E-18

Zbtb37	2.5216E-22	1.14587027	0.052	0.032	5.5322E-18
Zc3h6	2.539E-22	1.27279277	0.031	0.016	5.5704E-18
Pigw	2.6826E-22	2.13444535	0.013	0.005	5.8854E-18
Ddx55	2.6838E-22	1.16342453	0.059	0.038	5.888E-18
0610030E20R	6.2815E-22	0.92390541	0.109	0.082	1.3781E-17
Srek1	6.5582E-22	0.83291706	0.187	0.16	1.4388E-17
Mrpl38	7.6509E-22	1.17567003	0.099	0.074	1.6785E-17
Sesn1	8.0856E-22	0.82655408	0.073	0.049	1.7739E-17
Il12a	1.0689E-21	1.7047295	0.012	0.004	2.345E-17
Mdc1	1.097E-21	1.28616383	0.036	0.02	2.4066E-17
Tnrc6c	1.1306E-21	0.67561588	0.225	0.198	2.4804E-17
Ice2	1.2991E-21	1.60771077	0.026	0.013	2.8501E-17
Cbx5	1.3529E-21	1.0270712	0.08	0.056	2.9682E-17
Edaradd	1.3866E-21	1.84917424	0.016	0.006	3.0421E-17
Ifi206	1.5897E-21	1.25546107	0.05	0.031	3.4876E-17
Twnk	1.7964E-21	1.49657661	0.028	0.014	3.9411E-17
Gar1	1.9996E-21	1.15024186	0.098	0.073	4.387E-17
Zfp638	2.0297E-21	0.73627939	0.221	0.197	4.453E-17
Wdhd1	2.1973E-21	1.4045566	0.041	0.024	4.8207E-17
Ctse	2.482E-21	1.36279415	0.023	0.011	5.4454E-17
Zfp512	2.7385E-21	1.15602396	0.045	0.027	6.0081E-17
Fam76b	2.9633E-21	0.91146671	0.146	0.118	6.5013E-17
Cstf2	3.1973E-21	1.14496314	0.07	0.048	7.0146E-17
Eif5b	3.4389E-21	0.60595476	0.383	0.39	7.5446E-17
Pde3b	5.5386E-21	0.63021405	0.286	0.266	1.2151E-16
Pick1	5.7388E-21	1.33344499	0.03	0.016	1.259E-16
Nsmce4a	5.8942E-21	0.74214636	0.195	0.168	1.2931E-16
Casz1	6.6727E-21	1.81838168	0.026	0.013	1.4639E-16
Tctn1	7.096E-21	2.28825676	0.014	0.005	1.5568E-16
Gm3448	7.7662E-21	1.24233541	0.054	0.035	1.7038E-16
Miga1	8.2435E-21	1.40165608	0.027	0.014	1.8085E-16
Pds5a	8.4951E-21	0.68172817	0.227	0.202	1.8637E-16
Cep57	8.658E-21	0.8056077	0.119	0.091	1.8995E-16
Chd6	8.8982E-21	0.80044809	0.185	0.158	1.9522E-16
Dmwd	9.8141E-21	2.03450307	0.01	0.003	2.1531E-16
Nop56	1.0116E-20	0.66906556	0.226	0.2	2.2193E-16
Carns1	1.1644E-20	1.68585396	0.016	0.007	2.5546E-16
Usp24	1.213E-20	0.90800422	0.121	0.094	2.6612E-16
Entpd6	1.3791E-20	1.12098712	0.059	0.039	3.0257E-16
Pds5b	1.5981E-20	1.03754171	0.083	0.059	3.5061E-16
Cnp	1.9789E-20	1.00348425	0.088	0.064	4.3416E-16
Cspp1	2.0573E-20	0.89918497	0.126	0.099	4.5136E-16
Supt20	2.2348E-20	0.66185741	0.223	0.196	4.903E-16
Alkbh8	2.325E-20	1.19812176	0.046	0.028	5.1008E-16
Hnrnph1	2.6357E-20	0.34030259	0.478	0.495	5.7825E-16
2610037D02R	3.0648E-20	1.11116778	0.082	0.059	6.7239E-16
Dph2	3.366E-20	1.9643127	0.014	0.005	7.3846E-16
Rhoa	3.9071E-20	0.2710685	0.656	0.705	8.5719E-16

Kdm5d	4.3721E-20	1.28741612	0.041	0.025	9.592E-16
Igkv12-44	4.3838E-20	1.85999837	0.012	0.004	9.6177E-16
Pfas	4.483E-20	1.6132317	0.021	0.01	9.8352E-16
Zc3h8	4.9556E-20	1.58047054	0.019	0.009	1.0872E-15
Gpr174	5.2332E-20	0.78347034	0.112	0.085	1.1481E-15
Mphosph10	5.7376E-20	0.90619532	0.132	0.106	1.2588E-15
Swt1	6.529E-20	0.78793105	0.173	0.145	1.4324E-15
Rpf2	6.8171E-20	1.3408195	0.065	0.044	1.4956E-15
Arhgap4	8.6977E-20	1.02300133	0.095	0.071	1.9082E-15
Amigo2	9.7172E-20	1.88856888	0.01	0.003	2.1319E-15
Arhgap27os3	1.0228E-19	1.48808932	0.016	0.007	2.244E-15
Gm32856	1.0841E-19	1.69279886	0.012	0.004	2.3785E-15
Nom1	1.0859E-19	0.968848	0.114	0.089	2.3824E-15
Srsf11	1.1635E-19	0.39554368	0.471	0.504	2.5526E-15
Ifi203	1.2036E-19	0.8273182	0.246	0.225	2.6405E-15
Exosc1	1.9493E-19	1.18853657	0.058	0.039	4.2765E-15
Ppm1k	2.1807E-19	1.03741043	0.061	0.041	4.7843E-15
Prorsd1	2.7467E-19	0.98376912	0.115	0.09	6.0259E-15
Pdcd11	2.9064E-19	0.9173927	0.134	0.108	6.3762E-15
Tigd2	3.1013E-19	0.80303699	0.128	0.102	6.804E-15
Chd8	3.1083E-19	0.7382623	0.19	0.164	6.8193E-15
Zfp146	3.3122E-19	1.14366039	0.061	0.041	7.2666E-15
C530050E15R	3.6691E-19	2.0044138	0.01	0.003	8.0496E-15
Cirbp	3.9361E-19	0.49273056	0.315	0.299	8.6354E-15
Chchd4	3.9812E-19	1.26093834	0.061	0.041	8.7343E-15
Tmc8	4.0253E-19	1.53460373	0.021	0.01	8.8312E-15
Kdm3b	4.6222E-19	0.87470785	0.138	0.112	1.0141E-14
Lrrc57	5.0179E-19	1.3920441	0.036	0.021	1.1009E-14
P2ry10b	5.2291E-19	1.6385455	0.037	0.022	1.1472E-14
Slf2	6.02E-19	1.05076991	0.117	0.093	1.3207E-14
Diexf	6.6591E-19	1.33438483	0.042	0.026	1.4609E-14
Gne	6.9454E-19	1.61417713	0.018	0.008	1.5237E-14
Zfp39	7.0399E-19	1.65186271	0.018	0.008	1.5445E-14
Dnajb1	7.6114E-19	0.32276311	0.141	0.19	1.6699E-14
Tle4	8.7525E-19	0.57414258	0.257	0.235	1.9202E-14
Zmat1	9.4303E-19	1.66175218	0.018	0.008	2.0689E-14
Lta	1.0286E-18	1.29772593	0.023	0.012	2.2566E-14
Kcnq5	1.1462E-18	1.82791815	0.01	0.003	2.5147E-14
Sdad1	1.4283E-18	1.21358266	0.083	0.061	3.1334E-14
Ppm1l	1.5222E-18	1.30028308	0.022	0.011	3.3395E-14
Gtf3c2	1.6399E-18	0.87018476	0.135	0.11	3.5978E-14
Ikzf1	1.7778E-18	0.57251432	0.241	0.216	3.9003E-14
Gm15446	1.888E-18	1.86192272	0.011	0.004	4.1422E-14
Pitpnm2	2.0383E-18	1.07400074	0.032	0.018	4.4717E-14
Gemin6	2.0585E-18	1.90791487	0.017	0.007	4.5162E-14
Surf6	2.2747E-18	1.0372164	0.071	0.05	4.9905E-14
Svbp	2.6638E-18	1.02097016	0.158	0.135	5.844E-14
Lpp	2.8124E-18	0.25125586	0.113	0.158	6.1701E-14

Ifrd2	3.0968E-18	1.68654541	0.019	0.009	6.7941E-14
Nop16	3.3347E-18	1.38945417	0.058	0.039	7.3159E-14
Abraxas1	3.5163E-18	1.59442065	0.019	0.009	7.7145E-14
Cep295	3.738E-18	1.00945613	0.09	0.068	8.2008E-14
Zfp397	5.2691E-18	0.91466883	0.103	0.079	1.156E-13
Fancm	5.27E-18	1.23973446	0.032	0.019	1.1562E-13
0610040B10R	6.1762E-18	1.12360965	0.055	0.037	1.355E-13
Gm26853	6.5112E-18	1.01118142	0.085	0.063	1.4285E-13
Slc26a11	6.8437E-18	1.40408466	0.035	0.021	1.5014E-13
Dcaf1	6.9267E-18	0.80963332	0.099	0.075	1.5196E-13
Srm	9.4976E-18	1.42828006	0.053	0.035	2.0837E-13
Rbm38	1.0495E-17	0.79731992	0.077	0.055	2.3024E-13
Card11	1.2312E-17	0.90719332	0.101	0.078	2.7011E-13
Adamts6	1.2364E-17	1.14943126	0.026	0.014	2.7125E-13
Ftsj3	1.252E-17	1.05941053	0.091	0.069	2.7468E-13
Pck2	1.5656E-17	1.34476554	0.05	0.033	3.4348E-13
Dis3	2.0637E-17	1.29257581	0.04	0.025	4.5276E-13
Zkscan8	2.1967E-17	1.46262525	0.02	0.01	4.8193E-13
Mrm3	2.3086E-17	1.60366713	0.02	0.01	5.0648E-13
Noc3l	2.3758E-17	1.18246558	0.052	0.035	5.2122E-13
Wdr89	2.8335E-17	0.52270157	0.37	0.383	6.2163E-13
Efcab2	3.1699E-17	1.96441718	0.014	0.006	6.9544E-13
Gm16094	3.3742E-17	2.302226	0.013	0.005	7.4026E-13
Mttp	3.4951E-17	1.71834559	0.018	0.009	7.6679E-13
Rfx5	3.6464E-17	1.32500295	0.038	0.023	7.9997E-13
Haus5	3.716E-17	1.59821017	0.022	0.011	8.1524E-13
Cd200	4.6245E-17	0.89932164	0.075	0.054	1.0146E-12
Nvl	4.6355E-17	1.01730402	0.086	0.064	1.017E-12
Otud1	4.7571E-17	1.20913228	0.047	0.031	1.0437E-12
Coq5	4.9926E-17	1.01971831	0.069	0.049	1.0953E-12
Akt3	5.8644E-17	1.29476499	0.065	0.046	1.2866E-12
Mars2	6.0196E-17	1.55704796	0.018	0.009	1.3206E-12
Gigyf2	6.7275E-17	0.91903582	0.129	0.106	1.4759E-12
Zfp553	6.8594E-17	1.21657064	0.036	0.022	1.5049E-12
Atic	7.2311E-17	1.00290842	0.084	0.063	1.5864E-12
Bms1	7.9848E-17	0.86193702	0.129	0.105	1.7518E-12
Yae1d1	1.1672E-16	1.09224507	0.044	0.028	2.5607E-12
Stau2	1.4138E-16	1.71141992	0.015	0.006	3.1017E-12
Rnf41	1.4456E-16	1.18464645	0.052	0.035	3.1714E-12
Far1	1.6308E-16	0.78869473	0.199	0.179	3.5779E-12
Sdhaf1	2.1979E-16	0.7933284	0.16	0.137	4.822E-12
Parp11	2.2798E-16	1.24607833	0.042	0.027	5.0016E-12
Cep68	2.5595E-16	1.46842884	0.025	0.014	5.6153E-12
Clcn6	2.6248E-16	1.33736745	0.037	0.023	5.7586E-12
Lysmd1	3.294E-16	1.17778316	0.025	0.013	7.2266E-12
Mphosph8	3.364E-16	0.85107512	0.116	0.093	7.3802E-12
Mllt10	3.3863E-16	0.83275882	0.136	0.113	7.4292E-12
Ptbp2	3.5171E-16	0.84656339	0.152	0.13	7.7161E-12

Hgh1	4.4918E-16	1.69738936	0.016	0.007	9.8547E-12
Dbt	5.2729E-16	1.1370603	0.05	0.033	1.1568E-11
Kri1	5.3653E-16	1.03471592	0.076	0.057	1.1771E-11
Terf2	5.4804E-16	1.01972483	0.079	0.059	1.2024E-11
Klhl6	5.5702E-16	0.76064448	0.112	0.089	1.222E-11
Safb2	6.0156E-16	0.64227286	0.296	0.293	1.3198E-11
Aasdh	6.3507E-16	1.4300523	0.021	0.011	1.3933E-11
Mylip	6.6953E-16	0.90174351	0.105	0.084	1.4689E-11
Rpl10-ps3	7.9154E-16	0.28602912	0.128	0.173	1.7366E-11
Mlh3	8.1582E-16	1.47538238	0.017	0.008	1.7898E-11
Hist1h2ae	8.4492E-16	0.52989068	0.055	0.037	1.8537E-11
Mettl2	9.1608E-16	1.36023808	0.035	0.022	2.0098E-11
Lrpprc	1.0698E-15	1.11605077	0.064	0.046	2.3471E-11
Hdac10	1.1411E-15	1.97614178	0.014	0.006	2.5034E-11
Dennd6b	1.3279E-15	1.93269197	0.011	0.004	2.9133E-11
Rtn4ip1	1.3288E-15	1.41198055	0.02	0.01	2.9152E-11
Zmym6	1.4075E-15	1.45847915	0.021	0.011	3.0879E-11
Mettl3	1.6052E-15	1.09363156	0.05	0.034	3.5217E-11
Upf2	1.729E-15	0.66605106	0.175	0.151	3.7932E-11
C87436	1.7784E-15	0.94406357	0.071	0.052	3.9015E-11
Irf3	2.2386E-15	0.84038547	0.099	0.077	4.9113E-11
Nat10	2.2414E-15	1.33917733	0.036	0.022	4.9174E-11
Hist1h1d	2.3955E-15	0.83976601	0.085	0.065	5.2555E-11
Nop2	2.6362E-15	1.51164519	0.031	0.018	5.7836E-11
Gtpbp4	2.6515E-15	0.73394444	0.231	0.218	5.8171E-11
Fdxacb1	2.831E-15	1.48538913	0.042	0.028	6.2108E-11
Dhx57	2.9642E-15	1.18059068	0.034	0.021	6.5032E-11
Mnt	3.0428E-15	1.00328027	0.13	0.11	6.6755E-11
Uvssa	3.6608E-15	1.04260557	0.041	0.026	8.0314E-11
Scmh1	4.4284E-15	1.39426714	0.042	0.028	9.7155E-11
Ppcdc	5.017E-15	1.18784487	0.04	0.025	1.1007E-10
Itfg2	5.59E-15	1.07999874	0.042	0.028	1.2264E-10
Scd2	5.6112E-15	1.24726136	0.076	0.057	1.231E-10
Sarnp	6.3315E-15	0.30313324	0.188	0.249	1.3891E-10
Adat1	6.4922E-15	1.80504387	0.011	0.004	1.4243E-10
Ttc37	8.0507E-15	1.1496228	0.053	0.037	1.7662E-10
Rps6ka5	8.2298E-15	1.17448749	0.055	0.038	1.8055E-10
Gm26522	9.2649E-15	1.50603616	0.026	0.015	2.0326E-10
Mrpl23	9.938E-15	0.70114056	0.284	0.283	2.1803E-10
Gtf2a2	1.0035E-14	0.7742082	0.162	0.141	2.2015E-10
Dph6	1.0682E-14	1.2390126	0.053	0.037	2.3434E-10
Zcchc18	1.0921E-14	0.95143667	0.023	0.013	2.396E-10
Sptbn1	1.1954E-14	0.51000181	0.22	0.199	2.6227E-10
Hnrnpdl	1.2016E-14	0.44906417	0.427	0.457	2.6361E-10
Hpgds	1.2622E-14	0.71946237	0.107	0.085	2.7691E-10
Map4k2	1.2805E-14	0.66268568	0.122	0.099	2.8092E-10
2610035D17R	1.3695E-14	1.54709798	0.01	0.004	3.0046E-10
1300014J16Ril	1.4587E-14	1.97599244	0.01	0.004	3.2002E-10

Bid	1.4861E-14	1.42351559	0.023	0.013	3.2604E-10
Recql5	1.5107E-14	1.5548518	0.02	0.011	3.3143E-10
Ganc	1.5293E-14	1.23585724	0.044	0.029	3.3552E-10
Traf3ip3	1.6697E-14	0.77875077	0.088	0.068	3.663E-10
Mphosph9	1.86E-14	1.16208633	0.033	0.02	4.0806E-10
Prmt7	2.2116E-14	1.30725271	0.033	0.02	4.8521E-10
Alyref2	2.2427E-14	1.44133479	0.032	0.02	4.9202E-10
Zfp646	2.7205E-14	0.8551048	0.086	0.067	5.9685E-10
Ccnt1	3.1827E-14	0.55232346	0.288	0.28	6.9826E-10
Ctps	3.5434E-14	1.33958873	0.028	0.016	7.774E-10
Carf	4.148E-14	1.3671726	0.023	0.013	9.1003E-10
Hist1h2ap	4.3849E-14	0.89113536	0.04	0.026	9.6201E-10
Nop58	4.4156E-14	0.74145183	0.212	0.197	9.6874E-10
Phka1	4.697E-14	2.01686072	0.011	0.005	1.0305E-09
Sclt1	4.8196E-14	1.03998156	0.054	0.038	1.0574E-09
Polg	5.1349E-14	0.84913077	0.066	0.048	1.1265E-09
Thrap3	5.9573E-14	0.48685506	0.383	0.403	1.307E-09
Tmem186	6.2518E-14	1.41769921	0.02	0.01	1.3716E-09
Top3a	6.401E-14	1.34614576	0.026	0.015	1.4043E-09
2410002F23Ri	7.1119E-14	0.78869328	0.086	0.067	1.5603E-09
Psma1	7.7749E-14	0.26539958	0.204	0.266	1.7057E-09
Cables2	8.0034E-14	1.1360225	0.043	0.029	1.7559E-09
St8sia6	8.1434E-14	1.09884538	0.028	0.016	1.7866E-09
Pias4	8.7189E-14	0.95191448	0.103	0.083	1.9128E-09
Zdhhc17	9.7978E-14	1.07081084	0.058	0.042	2.1495E-09
Stambpl1	1.2463E-13	1.30138126	0.032	0.02	2.7342E-09
Foxk1	1.258E-13	1.1538778	0.05	0.035	2.7599E-09
4930570N18R	1.4424E-13	1.81736663	0.012	0.006	3.1645E-09
Ccar1	1.4768E-13	0.67904257	0.239	0.227	3.2399E-09
Ube3c	1.6723E-13	0.84926571	0.093	0.073	3.6688E-09
Ccdc50	1.8927E-13	0.7616914	0.257	0.253	4.1525E-09
Smyd4	2.1376E-13	1.32505593	0.019	0.01	4.6897E-09
Trim24	2.3415E-13	0.84199895	0.101	0.082	5.1369E-09
Igkv1-135	2.6566E-13	1.83109143	0.011	0.005	5.8284E-09
Git2	3.2323E-13	0.60792735	0.262	0.252	7.0913E-09
Vps13a	3.3741E-13	0.59450088	0.189	0.169	7.4023E-09
Hist3h2a	3.7945E-13	1.18456974	0.069	0.052	8.3247E-09
Luc7l3	3.8075E-13	0.68810167	0.214	0.199	8.3532E-09
Zfp592	3.9116E-13	0.84235121	0.107	0.087	8.5816E-09
Dido1	4.4296E-13	0.94576384	0.077	0.06	9.7181E-09
Lca5	4.6722E-13	1.50819929	0.011	0.005	1.025E-08
Nhlrc3	4.7184E-13	0.29194353	0.055	0.079	1.0352E-08
Dock11	4.9193E-13	0.7975114	0.166	0.148	1.0792E-08
Dvl2	5.2852E-13	1.33606792	0.037	0.025	1.1595E-08
Mettl13	5.3621E-13	1.16952372	0.029	0.018	1.1764E-08
Gm36839	6.0336E-13	1.73445137	0.017	0.009	1.3237E-08
Aldh2	6.0442E-13	1.00449883	0.135	0.118	1.326E-08
Snhg20	6.069E-13	0.89731859	0.062	0.045	1.3315E-08

Hist1h3d	6.092E-13	2.18476163	0.014	0.007	1.3365E-08
Sfxn1	6.6732E-13	1.04853207	0.075	0.058	1.464E-08
Slc4a1ap	6.8349E-13	0.84112386	0.112	0.092	1.4995E-08
AW549877	7.4887E-13	0.77654843	0.115	0.095	1.6429E-08
Ckap4	8.1339E-13	1.25587436	0.062	0.047	1.7845E-08
Patj	8.415E-13	0.92706551	0.027	0.017	1.8462E-08
Agbl3	8.7381E-13	1.80788812	0.013	0.006	1.917E-08
Grwd1	9.6042E-13	1.39953992	0.028	0.017	2.1071E-08
Ahsa1	1.0779E-12	0.92273025	0.132	0.113	2.3649E-08
Ptcd3	1.2391E-12	0.91012589	0.064	0.047	2.7185E-08
Icam2	1.3893E-12	1.08881018	0.037	0.025	3.048E-08
Ryr1	1.4009E-12	1.53133341	0.04	0.027	3.0735E-08
Hspa1b	1.4881E-12	1.25806002	0.181	0.161	3.2647E-08
Elk4	1.5435E-12	0.59824649	0.195	0.176	3.3864E-08
Nudt15	1.7494E-12	1.53680286	0.021	0.012	3.838E-08
Washc2	1.7991E-12	0.82871303	0.154	0.136	3.9471E-08
Sfpq	2.057E-12	0.29047546	0.504	0.551	4.5128E-08
Phf3	2.1324E-12	0.47561518	0.349	0.357	4.6783E-08
Hnrnpr	2.3161E-12	0.62705069	0.24	0.229	5.0813E-08
Kif18a	2.5183E-12	1.19755331	0.022	0.013	5.5248E-08
Phgdh	2.5947E-12	0.83883741	0.068	0.051	5.6924E-08
Hnrnpd	2.6864E-12	0.40264279	0.437	0.475	5.8936E-08
Brat1	3.1956E-12	1.29888806	0.021	0.012	7.0108E-08
Zc3h10	3.199E-12	1.16453299	0.063	0.047	7.0183E-08
Wdr73	3.2023E-12	1.33192488	0.026	0.016	7.0256E-08
Rftn1	3.2931E-12	0.84295074	0.119	0.101	7.2248E-08
Egr1	3.4767E-12	1.07431577	0.17	0.151	7.6275E-08
Wdr4	3.61E-12	1.51330284	0.021	0.012	7.92E-08
Dclre1c	3.697E-12	1.07458452	0.077	0.061	8.1108E-08
Rfc1	3.8062E-12	1.00459423	0.098	0.081	8.3505E-08
Herc4	3.982E-12	0.68945004	0.159	0.14	8.7361E-08
Sipa1l2	4.0121E-12	1.18152212	0.055	0.041	8.8022E-08
Kbtbd8	4.09E-12	1.51629609	0.012	0.005	8.9731E-08
Atad3a	4.1629E-12	1.313384	0.043	0.03	9.133E-08
Mrto4	4.3498E-12	0.28734494	0.079	0.107	9.5431E-08
Paics	4.6416E-12	0.25060363	0.095	0.125	1.0183E-07
Sag	5.0456E-12	1.27467323	0.017	0.009	1.107E-07
Cdc37l1	5.4318E-12	0.58834722	0.2	0.183	1.1917E-07
Setx	5.4671E-12	0.71360421	0.117	0.098	1.1994E-07
Gle1	5.483E-12	0.99478747	0.076	0.059	1.2029E-07
Qrsl1	7.2717E-12	1.10700203	0.041	0.028	1.5953E-07
Zfp950	7.3354E-12	0.99971519	0.055	0.04	1.6093E-07
Nemp1	7.727E-12	1.31065083	0.03	0.019	1.6952E-07
Heatr6	8.0821E-12	1.12039528	0.056	0.042	1.7731E-07
Lrch1	8.6386E-12	0.95479928	0.083	0.066	1.8952E-07
Trmt1l	8.7274E-12	0.77256008	0.102	0.084	1.9147E-07
Sumo3	8.9723E-12	0.27018838	0.082	0.111	1.9684E-07
Edrf1	9.3708E-12	0.82133195	0.066	0.05	2.0559E-07

Pus3	1.0154E-11	1.08443357	0.041	0.028	2.2278E-07
BC028528	1.1195E-11	0.59145535	0.053	0.075	2.4561E-07
D10Wsu102e	1.2289E-11	0.94499273	0.115	0.098	2.6961E-07
Pan3	1.2694E-11	0.56931491	0.265	0.258	2.7849E-07
Fam60a	1.2806E-11	0.9126282	0.083	0.066	2.8095E-07
Slc25a53	1.3199E-11	1.43439067	0.025	0.015	2.8958E-07
D6Wsu163e	1.3812E-11	1.59766006	0.014	0.007	3.0302E-07
Asrgl1	1.4537E-11	1.12179906	0.046	0.033	3.1894E-07
Hyls1	1.4647E-11	1.26106673	0.021	0.012	3.2133E-07
Blvrb	1.499E-11	0.76317009	0.152	0.135	3.2887E-07
Mir17hg	1.6876E-11	1.03488693	0.027	0.017	3.7024E-07
Elmod3	1.7499E-11	1.47553158	0.011	0.005	3.8392E-07
Akap8	1.8046E-11	0.78523229	0.12	0.102	3.9591E-07
6720427I07Ril	1.9772E-11	1.03207729	0.044	0.031	4.3379E-07
Ecd	1.9788E-11	0.98580818	0.076	0.059	4.3413E-07
Nfx1	2.0634E-11	0.78201023	0.133	0.115	4.5269E-07
Nudcd3	2.0891E-11	0.72400117	0.193	0.178	4.5834E-07
Mfsd4a	2.4241E-11	0.86255709	0.061	0.046	5.3183E-07
Rrp8	2.7053E-11	1.02365853	0.067	0.051	5.9351E-07
Ccdc15	2.7394E-11	1.46207651	0.012	0.006	6.01E-07
Lemd3	2.7793E-11	0.82730248	0.118	0.1	6.0976E-07
Snrpa	2.8979E-11	0.30381608	0.086	0.115	6.3577E-07
Ipo7	2.9832E-11	0.25779703	0.144	0.185	6.5448E-07
Tma16	3.0215E-11	1.01231036	0.086	0.07	6.6289E-07
Las1l	3.1075E-11	0.72714415	0.126	0.108	6.8175E-07
Cenpu	3.3477E-11	1.84547691	0.01	0.005	7.3445E-07
Zbed3	3.3803E-11	1.31820979	0.021	0.013	7.4161E-07
Mios	3.7928E-11	1.2664253	0.029	0.019	8.321E-07
Pibf1	3.9828E-11	0.93944241	0.068	0.052	8.7379E-07
Exosc10	4.1629E-11	0.99011211	0.084	0.068	9.1331E-07
Snrpd3	4.3587E-11	0.29364659	0.204	0.262	9.5625E-07
Gabpb2	4.462E-11	0.75007533	0.137	0.12	9.7891E-07
Eef1e1	5.2665E-11	0.90244878	0.117	0.1	1.1554E-06
Llph	5.3473E-11	0.47178274	0.411	0.446	1.1731E-06
Ptk2	5.4634E-11	1.57353605	0.017	0.01	1.1986E-06
Dennd3	6.5388E-11	1.00728729	0.035	0.023	1.4345E-06
Zmynd11	6.6166E-11	0.73787684	0.189	0.177	1.4516E-06
Eif1b	6.743E-11	0.26214585	0.112	0.145	1.4794E-06
Ercc8	6.9786E-11	1.318349	0.03	0.02	1.531E-06
Commd9	7.1122E-11	0.27638827	0.021	0.034	1.5603E-06
Prim2	7.1792E-11	1.41870094	0.03	0.02	1.575E-06
Trmt2a	7.5291E-11	1.03227407	0.049	0.036	1.6518E-06
Ddx54	7.8388E-11	0.72176643	0.234	0.229	1.7198E-06
Anp32b	7.8606E-11	0.41183903	0.431	0.467	1.7245E-06
Gfm2	8.0139E-11	1.01950856	0.047	0.034	1.7582E-06
Klhl42	8.2834E-11	1.26483208	0.013	0.006	1.8173E-06
Ddx23	8.7067E-11	0.73085416	0.166	0.151	1.9102E-06
Stxbp1	9.0142E-11	1.06354622	0.025	0.015	1.9776E-06

Gprasp1	1.0057E-10	0.97520624	0.038	0.026	2.2064E-06
Pprc1	1.0348E-10	1.27003297	0.04	0.028	2.2702E-06
Taf1c	1.4488E-10	1.07275301	0.03	0.02	3.1785E-06
Rad9a	1.4808E-10	1.2857633	0.022	0.014	3.2487E-06
Tbc1d5	1.4891E-10	0.96903336	0.102	0.086	3.267E-06
Slc16a6	1.5224E-10	0.5973602	0.208	0.195	3.3399E-06
Atr	1.5836E-10	0.773961	0.085	0.069	3.4743E-06
Dnpep	1.6606E-10	0.25904196	0.039	0.057	3.6431E-06
Lars	1.6856E-10	0.28063771	0.109	0.141	3.6979E-06
Ppdpf	1.7597E-10	0.76725118	0.181	0.168	3.8606E-06
Polr3d	1.8963E-10	1.01459682	0.084	0.068	4.1602E-06
Cib1	1.9051E-10	0.2713066	0.119	0.153	4.1796E-06
Aldh9a1	2.0525E-10	0.2961902	0.042	0.06	4.5029E-06
Tmem60	2.481E-10	0.96331548	0.082	0.066	5.443E-06
Rccd1	2.641E-10	1.08846145	0.022	0.013	5.7942E-06
Pcm1	2.6571E-10	0.6982618	0.167	0.152	5.8294E-06
Dhps	2.9009E-10	0.25059847	0.086	0.113	6.3642E-06
Abca5	3.0383E-10	1.53611114	0.014	0.007	6.6656E-06
Nle1	3.1215E-10	1.26684461	0.025	0.016	6.8483E-06
Jade2	3.1703E-10	0.73122994	0.06	0.046	6.9553E-06
Rnf115	3.1774E-10	0.28983796	0.105	0.136	6.9709E-06
Fbxo4	3.3821E-10	1.17613357	0.048	0.035	7.4199E-06
Zfp622	3.3877E-10	0.81688319	0.189	0.177	7.4324E-06
Gmip	3.7725E-10	0.85648306	0.106	0.09	8.2765E-06
Wdr12	3.7919E-10	1.04780904	0.046	0.034	8.3191E-06
Srcap	3.873E-10	0.81296245	0.112	0.095	8.4969E-06
Phc1	3.9052E-10	1.23016112	0.034	0.024	8.5677E-06
Harbi1	4.6739E-10	1.41659368	0.019	0.011	1.0254E-05
Nsd1	5.0852E-10	0.59319252	0.234	0.226	1.1157E-05
Zfp800	5.2443E-10	0.26499304	0.124	0.159	1.1506E-05
Usp37	5.6012E-10	0.25550858	0.077	0.101	1.2288E-05
Rraga	5.7046E-10	0.25611851	0.093	0.121	1.2515E-05
Ddx3y	6.0366E-10	0.51797233	0.213	0.199	1.3244E-05
Lrrk1	6.0523E-10	0.34276347	0.04	0.058	1.3278E-05
Ccdc125	6.2819E-10	1.23833457	0.013	0.007	1.3782E-05
Hirip3	6.9483E-10	0.97981116	0.042	0.03	1.5244E-05
Cfap97	7.0301E-10	0.84904221	0.06	0.046	1.5423E-05
Tango6	7.211E-10	1.30602245	0.017	0.009	1.582E-05
Atp6v1h	7.2975E-10	0.75885634	0.186	0.176	1.601E-05
Brd3	7.4377E-10	0.74899772	0.101	0.084	1.6318E-05
Phf20	7.7223E-10	0.9784117	0.091	0.076	1.6942E-05
Ccdc77	7.9726E-10	1.27000126	0.02	0.012	1.7491E-05
Atraid	8.1176E-10	0.42112588	0.061	0.083	1.7809E-05
AI987944	8.119E-10	0.97888487	0.043	0.031	1.7812E-05
Arfgap2	1.0073E-09	0.25467435	0.109	0.139	2.2098E-05
2900093K20R	1.0279E-09	1.06304849	0.048	0.036	2.2551E-05
Ap1m1	1.0953E-09	0.96341174	0.085	0.07	2.4029E-05
Gdf11	1.1394E-09	1.18738586	0.018	0.011	2.4996E-05

Mthfd1l	1.1445E-09	1.03287635	0.034	0.023	2.5109E-05
Vps33a	1.1646E-09	0.25132428	0.037	0.053	2.555E-05
Pdhx	1.1822E-09	1.09212023	0.037	0.026	2.5936E-05
Ighv1-9	1.3866E-09	1.40141695	0.01	0.005	3.0421E-05
Zmynd8	1.4168E-09	0.69912636	0.119	0.103	3.1083E-05
Hmgcs1	1.4259E-09	0.26779334	0.108	0.137	3.1282E-05
Qtrt1	1.4981E-09	1.24039982	0.033	0.022	3.2867E-05
Cdkl3	1.5334E-09	1.419593	0.013	0.007	3.3641E-05
Rbm33	1.6548E-09	0.57537999	0.214	0.203	3.6305E-05
Adpgk	1.7439E-09	1.02004395	0.068	0.054	3.8259E-05
E130307A14R	1.7669E-09	1.32378947	0.02	0.012	3.8764E-05
Dnajc9	1.8884E-09	0.63555577	0.129	0.112	4.1429E-05
Trim30b	1.9605E-09	0.67432469	0.028	0.018	4.3011E-05
Spopl	2.0348E-09	0.80651955	0.098	0.082	4.4641E-05
Zfp207	2.1029E-09	0.50583024	0.315	0.323	4.6135E-05
1700037C18R	2.2961E-09	1.43985968	0.022	0.014	5.0374E-05
E230001N04R	2.406E-09	1.14153402	0.017	0.01	5.2786E-05
Rbbp6	2.6372E-09	0.54675251	0.297	0.303	5.7856E-05
Sft2d3	2.6837E-09	0.84568008	0.058	0.044	5.8878E-05
Dmac2	2.6876E-09	0.8315113	0.088	0.073	5.8964E-05
Egln2	2.7789E-09	0.90226861	0.106	0.091	6.0967E-05
C920021L13R	2.8781E-09	1.35057739	0.011	0.005	6.3142E-05
Phlpp2	3.1178E-09	1.29663757	0.021	0.013	6.8402E-05
Gm10762	3.1523E-09	1.37038718	0.024	0.016	6.9159E-05
Zfp277	3.164E-09	0.31532329	0.054	0.073	6.9414E-05
Ly6k	3.695E-09	1.19991655	0.011	0.006	8.1065E-05
Ahcyl1	4.1729E-09	0.2585333	0.097	0.124	9.1549E-05
Pinx1	4.1939E-09	1.16723296	0.047	0.035	9.2011E-05
Scaf8	4.5163E-09	0.66420864	0.142	0.127	9.9082E-05
Rad17	4.5295E-09	0.89842379	0.076	0.062	9.9373E-05
Slc17a9	4.5387E-09	1.19374863	0.017	0.01	9.9574E-05
Pcgf6	4.5432E-09	1.10661102	0.023	0.014	9.9673E-05
Ankle2	4.7585E-09	0.90957618	0.081	0.067	0.0001044
Ccar2	5.172E-09	1.25924989	0.02	0.012	0.00011347
Atpaf1	5.2493E-09	1.147561	0.02	0.013	0.00011517
Glyr1	5.2931E-09	0.75086644	0.188	0.179	0.00011613
Polr3h	5.6846E-09	1.34053024	0.021	0.013	0.00012471
Hook2	5.7491E-09	1.17228625	0.042	0.031	0.00012613
Zmym5	6.1203E-09	0.62237975	0.171	0.158	0.00013427
Gm26857	6.3844E-09	1.77893294	0.013	0.007	0.00014007
Telo2	6.5973E-09	1.45033124	0.02	0.012	0.00014474
Rbm48	6.8288E-09	0.77367301	0.071	0.057	0.00014982
Wdr61	7.109E-09	0.29805336	0.09	0.115	0.00015596
Pura	7.5002E-09	0.58903834	0.186	0.173	0.00016455
Slc37a2	7.6132E-09	0.47156784	0.028	0.042	0.00016702
Det1	8.0788E-09	1.43712692	0.012	0.007	0.00017724
Zfp422	8.6386E-09	1.169585	0.021	0.014	0.00018952
Cenpo	9.0087E-09	1.33653425	0.015	0.009	0.00019764

Nup205	9.1081E-09	0.9704589	0.051	0.039	0.00019982
Mlh1	9.3273E-09	1.12681873	0.017	0.01	0.00020463
Papolg	1.0117E-08	0.91067636	0.062	0.049	0.00022195
Prmt6	1.0904E-08	1.18348943	0.038	0.028	0.00023922
Rtn3	1.1268E-08	0.76230583	0.224	0.223	0.00024721
Setdb2	1.1462E-08	1.04338961	0.041	0.03	0.00025146
Ly9	1.1672E-08	0.73984262	0.086	0.071	0.00025607
AB124611	1.1813E-08	0.97200482	0.099	0.085	0.00025916
Ibtk	1.2222E-08	0.81605341	0.103	0.088	0.00026815
Tbl1xr1	1.2429E-08	0.66567817	0.166	0.153	0.00027268
Ranbp2	1.2725E-08	0.41285161	0.368	0.392	0.00027917
D130040H23F	1.282E-08	1.20782751	0.016	0.009	0.00028126
Fam219b	1.369E-08	1.21393828	0.027	0.018	0.00030035
Stoml2	1.3791E-08	0.25524552	0.043	0.059	0.00030255
Ercc6	1.4144E-08	0.90388942	0.067	0.054	0.0003103
Cnot4	1.456E-08	0.25374288	0.177	0.22	0.00031942
Tcaim	1.5015E-08	1.01364049	0.025	0.016	0.00032941
Urb2	1.5141E-08	1.22745895	0.02	0.013	0.00033217
Gm10076	1.5306E-08	0.27964686	0.141	0.176	0.00033579
Nol11	1.5438E-08	0.81607722	0.111	0.097	0.00033869
Rlim	1.5596E-08	0.60332727	0.275	0.282	0.00034216
Polr3e	1.5619E-08	1.13464396	0.033	0.024	0.00034267
Khsrp	1.6696E-08	0.26295697	0.11	0.138	0.0003663
Nifk	1.6769E-08	0.8441085	0.093	0.079	0.00036789
Hdac7	1.7326E-08	0.62483088	0.095	0.08	0.00038012
Parn	1.7338E-08	1.0043097	0.04	0.029	0.00038039
Shmt1	1.7927E-08	1.63469849	0.01	0.005	0.00039331
Polr3g	1.799E-08	1.17959965	0.016	0.01	0.00039469
Gm13012	1.8185E-08	1.04131032	0.017	0.011	0.00039897
Nagk	1.8566E-08	0.27092501	0.023	0.035	0.00040733
Phf7	1.8872E-08	1.35408235	0.023	0.015	0.00041403
Bend3	1.9716E-08	1.22919814	0.023	0.015	0.00043256
Camta1	2.2906E-08	0.37760056	0.063	0.082	0.00050254
Urb1	2.3772E-08	1.24295927	0.024	0.016	0.00052154
D3Ertd751e	2.4054E-08	0.85197359	0.026	0.017	0.00052773
Dnajc21	2.4497E-08	0.26875381	0.154	0.192	0.00053744
Usf3	2.5067E-08	1.04488955	0.068	0.055	0.00054995
Bnip1	2.5216E-08	1.14044558	0.04	0.029	0.00055321
Rara	2.6272E-08	0.3456143	0.076	0.099	0.00057637
Aldh7a1	2.7749E-08	1.29441839	0.022	0.015	0.00060878
Taf15	2.8175E-08	0.63725237	0.197	0.188	0.00061812
Rsrc2	2.8244E-08	0.25870171	0.522	0.578	0.00061964
Huwe1	2.9471E-08	0.46608158	0.323	0.336	0.00064656
Snrpf	3.0615E-08	0.29473819	0.509	0.572	0.00067166
Tubgcp5	3.1734E-08	0.9886353	0.036	0.026	0.00069622
Nup214	3.1753E-08	0.28063216	0.076	0.098	0.00069664
Mtcp1	3.2187E-08	1.15154822	0.019	0.012	0.00070614
C330018D20F	3.2833E-08	1.56065295	0.01	0.005	0.00072033

Asb13	3.2951E-08	0.88690689	0.04	0.029	0.00072291
Gm15232	3.4548E-08	1.59005432	0.015	0.009	0.00075796
Mtmr4	3.545E-08	1.33208455	0.035	0.025	0.00077773
Txnrd3	3.5671E-08	1.87145545	0.01	0.005	0.00078258
C920006O11F	3.9747E-08	1.28800976	0.011	0.006	0.000872
Strn3	4.0394E-08	0.67158801	0.163	0.151	0.00088621
Gm26890	4.2748E-08	1.22312391	0.015	0.009	0.00093786
Mapk8	4.3854E-08	0.26639295	0.064	0.083	0.00096212
Heatr1	4.3919E-08	0.99649374	0.07	0.057	0.00096355
Txlna	4.603E-08	0.84450697	0.108	0.094	0.00100984
Banp	4.8978E-08	1.04845832	0.035	0.025	0.00107453
Zfp839	5.3797E-08	1.63772547	0.014	0.008	0.00118026
5730455P16R	5.7322E-08	1.10364259	0.032	0.023	0.00125758
Odf2l	5.7887E-08	1.19164428	0.022	0.015	0.00126998
Ints7	6.1777E-08	0.75275971	0.069	0.056	0.00135532
Numa1	6.4825E-08	0.47707059	0.208	0.196	0.00142219
2700049A03R	6.6399E-08	1.13974532	0.016	0.01	0.00145673
Phykpl	6.7655E-08	0.99145229	0.049	0.038	0.00148428
Dopey1	6.7801E-08	0.78887803	0.087	0.073	0.00148748
Knl1	6.9176E-08	1.12920145	0.019	0.012	0.00151766
1700096K18R	7.027E-08	1.16836351	0.035	0.026	0.00154165
Taok3	7.4098E-08	0.31051332	0.14	0.174	0.00162564
Oxa1l	7.7573E-08	0.27472959	0.09	0.113	0.00170187
Prpf4b	7.8785E-08	0.36408013	0.385	0.406	0.00172846
Nol9	8.0425E-08	0.9626836	0.048	0.037	0.00176445
Dnajb4	8.0994E-08	0.72395436	0.106	0.092	0.00177694
Pdcd2l	8.2013E-08	0.28973006	0.039	0.053	0.00179927
Nhp2	8.3617E-08	0.43847802	0.148	0.187	0.00183446
2310057M21F	8.7902E-08	1.1030905	0.032	0.023	0.00192849
Tut1	8.8705E-08	0.99809119	0.036	0.027	0.0019461
Kat14	9.1761E-08	1.14917749	0.027	0.019	0.00201315
Fam35a	9.7711E-08	1.05523256	0.022	0.015	0.00214367
Naa50	9.7819E-08	0.26271425	0.171	0.212	0.00214605
Mettl18	1.0057E-07	1.26052922	0.012	0.007	0.00220649
Ipo5	1.015E-07	0.39300763	0.073	0.094	0.00222679
C2cd3	1.0277E-07	0.84996419	0.061	0.049	0.00225459
Tnfaip1	1.0383E-07	0.26098794	0.031	0.044	0.00227787
Nudcd1	1.0394E-07	1.13502727	0.024	0.016	0.00228041
Naglu	1.1024E-07	0.33764561	0.01	0.017	0.00241855
Xrcc5	1.1621E-07	0.94371458	0.022	0.015	0.00254948
Prdx3	1.2055E-07	0.31309654	0.064	0.083	0.0026447
Zfp961	1.2228E-07	1.06257183	0.016	0.01	0.00268263
Gm20045	1.3301E-07	1.23881151	0.02	0.013	0.00291809
Zmiz2	1.3491E-07	0.33195141	0.129	0.161	0.00295981
Cenpk	1.3551E-07	1.19616016	0.024	0.017	0.00297288
Snrpa1	1.3631E-07	0.28366798	0.117	0.145	0.00299044
Slc10a7	1.392E-07	1.13085788	0.034	0.025	0.00305397
AI606181	1.4572E-07	1.15504403	0.028	0.02	0.00319703

BC004004	1.4756E-07	0.31739012	0.085	0.108	0.00323731
Baz1b	1.4803E-07	0.57421175	0.194	0.185	0.00324768
Zfp472	1.6015E-07	0.90007806	0.035	0.025	0.00351356
Fam117a	1.6975E-07	0.90634366	0.031	0.022	0.00372416
Rpp14	1.7203E-07	1.057348	0.037	0.028	0.0037742
Zfp595	1.7327E-07	1.05322159	0.023	0.016	0.00380126
Fam136a	1.7715E-07	1.04046444	0.043	0.033	0.00388654
Gm28935	1.8772E-07	1.12353903	0.019	0.013	0.00411836
Chtf8	1.9494E-07	0.28124878	0.07	0.09	0.0042767
Pnrc2	2.0875E-07	0.68326332	0.153	0.142	0.00457985
Syce2	2.0942E-07	0.2849904	0.02	0.03	0.00459457
Lcorl	2.1266E-07	0.94226896	0.072	0.06	0.00466551
Urm1	2.1666E-07	0.32962791	0.056	0.073	0.00475323
Ccdc9	2.225E-07	0.37566539	0.112	0.139	0.00488142
Lsm6	2.4277E-07	0.63635589	0.213	0.21	0.00532609
Rnf2	2.6349E-07	0.68752181	0.112	0.099	0.00578068
Ift22	2.6991E-07	0.40421865	0.027	0.038	0.0059216
Hmgn2	2.7446E-07	0.61675395	0.165	0.155	0.00602139
Arfgef2	2.7869E-07	0.91471939	0.064	0.052	0.00611415
Hsdl1	2.9955E-07	0.87614241	0.053	0.042	0.00657191
Hnrnph2	3.0107E-07	0.51327122	0.314	0.333	0.0066051
Zfp688	3.3184E-07	0.90999074	0.034	0.025	0.00728017
Abhd6	3.3232E-07	1.31612012	0.015	0.01	0.0072907
Krit1	3.419E-07	0.42843552	0.292	0.294	0.00750086
Yrdc	3.7967E-07	0.29053095	0.095	0.119	0.00832968
Rhebl1	4.0091E-07	1.14222924	0.016	0.01	0.00879546
Egr2	4.1509E-07	1.23222669	0.043	0.033	0.00910657
Parp6	4.1609E-07	0.92986095	0.037	0.028	0.00912865
Zfp956	4.3015E-07	1.00430296	0.017	0.011	0.00943698
Mast3	4.4497E-07	0.74737505	0.067	0.055	0.00976223
Ripor1	4.5382E-07	1.08629303	0.02	0.013	0.0099564
Ogfod3	4.5456E-07	1.35735467	0.02	0.014	0.0099726
Tle1	4.6368E-07	0.39209061	0.022	0.032	0.01017262
Eif2a	4.7826E-07	0.3199578	0.125	0.154	0.01049255
Sharpin	4.9056E-07	0.36611003	0.06	0.078	0.01076247
Emc6	5.0244E-07	0.27656792	0.192	0.236	0.01102312
Cbx1	5.1232E-07	0.67892045	0.169	0.16	0.01123985
Gemin5	5.1984E-07	1.2057167	0.021	0.014	0.0114048
Ythdf2	5.2305E-07	0.30024619	0.102	0.127	0.01147526
Mrpl3	5.3171E-07	0.27906693	0.072	0.091	0.0116652
Zfp292	5.4816E-07	0.44448673	0.253	0.248	0.01202599
Epn1	6.0093E-07	0.36798062	0.161	0.2	0.01318379
Srp54a	6.0494E-07	0.28455915	0.076	0.096	0.01327188
Ppp3cb	6.2449E-07	0.6381402	0.172	0.163	0.01370068
Ncaph2	6.3319E-07	0.27170533	0.084	0.105	0.01389163
Kat7	6.3397E-07	0.78514681	0.101	0.089	0.01390865
Bcap29	6.4514E-07	0.3891954	0.058	0.075	0.01415378
Plpbp	6.666E-07	0.34752686	0.135	0.167	0.0146245

Ddx41	6.7129E-07	0.86538164	0.109	0.097	0.01472752
2810405F17Ri	6.983E-07	1.07016529	0.021	0.014	0.01532005
Rbm12b1	7.0344E-07	1.07406362	0.013	0.008	0.01543281
Ebag9	7.062E-07	0.88814575	0.055	0.045	0.01549327
Rttn	7.4914E-07	1.21633262	0.015	0.009	0.01643529
Hars2	7.6153E-07	0.88060646	0.032	0.024	0.01670715
Dync1li2	8.4611E-07	0.25040275	0.039	0.052	0.01856271
Ube2g2	8.6836E-07	0.38051379	0.082	0.103	0.01905101
Nol6	8.9292E-07	1.00826492	0.042	0.033	0.01958978
Arhgap26	9.0981E-07	0.25741505	0.087	0.108	0.01996034
Ndc1	9.6096E-07	0.89225119	0.019	0.012	0.02108246
Usp28	9.7101E-07	1.02902288	0.02	0.013	0.02130292
Irf9	1.003E-06	0.31673985	0.121	0.149	0.02200378
Drg1	1.0105E-06	0.27655274	0.069	0.087	0.02217045
Mto1	1.0114E-06	1.18687573	0.023	0.016	0.02218809
Rfc2	1.0287E-06	0.89889364	0.088	0.077	0.02256958
Fnbp4	1.0633E-06	0.69088483	0.138	0.127	0.02332799
Angel2	1.0663E-06	0.59889849	0.163	0.152	0.02339386
Eefsec	1.102E-06	1.12029471	0.035	0.026	0.02417676
Tex9	1.1413E-06	0.71712161	0.02	0.013	0.0250383
Thumpd2	1.1556E-06	1.21905775	0.014	0.008	0.02535293
Pex1	1.2691E-06	1.18009106	0.025	0.018	0.0278437
Selenoh	1.2847E-06	0.26280156	0.123	0.15	0.02818599
Eif4g1	1.319E-06	0.28789289	0.204	0.251	0.02893708
Lman2l	1.3522E-06	1.06052402	0.05	0.04	0.02966574
Sigmar1	1.3781E-06	0.29227979	0.014	0.022	0.03023393
Adar	1.3946E-06	0.80562861	0.08	0.068	0.03059611
Ints4	1.4584E-06	1.11630668	0.029	0.021	0.0319948
Psmd11	1.4779E-06	0.29475399	0.213	0.262	0.03242278
Smarcc2	1.4929E-06	0.26337064	0.064	0.081	0.03275272
Hist2h2be	1.4994E-06	1.43677512	0.01	0.006	0.03289497
AU041133	1.5267E-06	0.93339573	0.022	0.015	0.03349397
Ube2q1	1.5883E-06	0.32659748	0.123	0.151	0.03484515
Akap8l	1.5941E-06	0.68335878	0.105	0.093	0.03497255
Brca2	1.5985E-06	1.11187255	0.013	0.008	0.03506983
Them6	1.6075E-06	1.19846632	0.018	0.012	0.03526632
Tfb2m	1.611E-06	0.79040016	0.055	0.045	0.03534416
Ddx51	1.6112E-06	1.03354026	0.021	0.015	0.03534742
A930029G22R	1.6662E-06	1.36867735	0.011	0.006	0.03655538
Myo18a	1.761E-06	0.46599109	0.032	0.044	0.03863561
Taf5l	1.7881E-06	0.75628732	0.1	0.088	0.03922865
Clk4	1.7994E-06	0.4979803	0.278	0.285	0.03947723
Zfp710	1.8138E-06	0.3475349	0.018	0.026	0.03979246
Rexo4	1.8394E-06	0.3259339	0.072	0.091	0.04035392
Nob1	1.8511E-06	0.89093782	0.071	0.06	0.04061159
Snrpd1	1.895E-06	0.26225384	0.281	0.35	0.04157438
Necap1	1.9392E-06	0.79005716	0.1	0.088	0.04254391
Thoc6	1.9401E-06	0.36682287	0.036	0.048	0.04256398

Rassf3	1.9721E-06	0.52859631	0.207	0.202	0.04326528
Cops5	1.9731E-06	0.32355345	0.137	0.167	0.04328835
5430427O19R	1.9801E-06	1.12073239	0.03	0.023	0.04344198
Snx25	1.9957E-06	0.97340984	0.028	0.021	0.04378437
Pbdc1	2.0277E-06	0.39862638	0.134	0.164	0.04448628
Pafah1b3	2.0501E-06	0.61748448	0.051	0.067	0.04497716
Ccni	2.0667E-06	0.51880921	0.248	0.25	0.04534096
Jun	2.0982E-06	0.30424326	0.283	0.28	0.04603325
Cux1	2.2026E-06	0.32550578	0.174	0.214	0.04832347
Natd1	2.2284E-06	1.15540311	0.02	0.014	0.04888843
Mrpl12	2.3749E-06	0.34220882	0.119	0.146	0.05210382
Zfp639	2.5901E-06	0.27976359	0.071	0.088	0.05682398
Taf1d	2.6608E-06	0.44605438	0.358	0.386	0.0583758
Ppp1r13b	2.8473E-06	0.78033851	0.053	0.043	0.06246622
Ube2d1	2.9074E-06	0.82622023	0.095	0.084	0.06378586
Rrp9	2.9652E-06	0.99379034	0.043	0.034	0.0650546
Hypk	3.066E-06	0.36610101	0.139	0.171	0.06726546
Hspa9	3.1415E-06	0.27127883	0.236	0.29	0.06892094
Mcm7	3.1773E-06	0.80052558	0.057	0.047	0.06970577
Top3b	3.2363E-06	0.87330926	0.052	0.042	0.07100083
Slc35a2	3.247E-06	0.25793325	0.036	0.048	0.07123512
Phb2	3.3398E-06	0.35063579	0.16	0.195	0.07327281
Tbcel	3.3439E-06	0.64950669	0.075	0.064	0.07336225
Kif20b	3.3641E-06	0.70920783	0.028	0.02	0.07380433
Tsc2	3.6241E-06	0.25883288	0.023	0.032	0.07950818
Snrnp40	3.8211E-06	0.28177641	0.083	0.102	0.08383126
Lnpk	3.8352E-06	0.61012392	0.094	0.082	0.08413983
Igkv1-117	4.1916E-06	0.95118167	0.013	0.008	0.09195984
Nsun5	4.5149E-06	1.17236221	0.021	0.015	0.09905271
Zbtb4	4.6272E-06	0.71054669	0.053	0.043	0.10151658
Nfu1	4.6754E-06	0.48959486	0.078	0.097	0.10257428
Pold3	4.6818E-06	0.28424261	0.047	0.06	0.10271366
Abcg1	4.7222E-06	0.76704174	0.114	0.103	0.10360139
Wdr77	4.7707E-06	0.85316596	0.028	0.021	0.10466538
Xpc	4.86E-06	0.93355324	0.027	0.02	0.10662273
Slx4	4.8752E-06	1.15735358	0.02	0.014	0.10695761
Hmg20a	4.9452E-06	0.8210374	0.049	0.039	0.10849361
Pcyt2	4.9604E-06	0.39430986	0.03	0.04	0.10882557
Bmi1	4.9824E-06	0.87002529	0.049	0.039	0.10930879
Nid1	5.0703E-06	0.89182839	0.012	0.007	0.11123749
Rab3gap1	5.2132E-06	0.6097175	0.176	0.169	0.1143713
Tsen15	5.3386E-06	0.94707069	0.025	0.018	0.11712404
Osgepl1	5.3651E-06	1.2133787	0.012	0.007	0.11770449
Bcl7b	5.54E-06	0.32530852	0.124	0.151	0.12154125
Rfx3	5.5841E-06	0.83242277	0.029	0.022	0.12251002
Mpv17l	5.6201E-06	1.03232687	0.034	0.026	0.12329897
Foxn3	5.7376E-06	0.53036791	0.282	0.294	0.12587683
Zfp605	5.8317E-06	1.40639978	0.011	0.007	0.12794163

Mccc1	5.9121E-06	1.32796932	0.016	0.01	0.12970559
Trim65	5.9875E-06	1.21878229	0.016	0.011	0.1313599
Nup107	6.0935E-06	0.87358444	0.028	0.02	0.13368554
Wdr76	6.1252E-06	0.93418119	0.025	0.018	0.13438066
Fam117b	6.4068E-06	0.31012656	0.221	0.27	0.14055946
Zfp740	6.5214E-06	0.70100381	0.083	0.072	0.14307312
Jpx	6.7486E-06	1.22080764	0.017	0.011	0.14805759
Dzip3	6.8444E-06	1.06482131	0.018	0.012	0.15015982
Ylpm1	6.8517E-06	0.71645504	0.083	0.072	0.15031989
Bri3bp	7.0908E-06	0.44774363	0.064	0.08	0.15556463
Vipas39	7.4159E-06	0.37897444	0.026	0.035	0.16269736
Cct7	7.5764E-06	0.26195099	0.253	0.31	0.16621873
Htt	7.5877E-06	0.32724761	0.088	0.108	0.16646703
H2-Ke6	7.6873E-06	0.26773109	0.075	0.093	0.16865072
Fbxo11	7.7227E-06	0.32333043	0.171	0.207	0.16942921
Grk2	7.8549E-06	0.5220876	0.292	0.308	0.17232883
Gps2	8.1082E-06	0.30160433	0.125	0.152	0.17788473
Dhodh	8.4954E-06	1.24501761	0.013	0.008	0.18637988
Fbxo5	8.5169E-06	0.90164126	0.017	0.012	0.18685189
2010315B03R	8.5274E-06	1.12334223	0.032	0.025	0.18708345
5730480H06R	8.6258E-06	0.9195542	0.025	0.018	0.18924167
Rtcb	8.8683E-06	0.75544506	0.171	0.167	0.19456086
Klhl12	9.4356E-06	0.61169681	0.057	0.047	0.20700795
Snapc4	9.7889E-06	1.10134492	0.019	0.013	0.21475888
Tlk1	9.8499E-06	0.54065351	0.184	0.177	0.21609691
Atg16l1	9.9854E-06	0.73424034	0.078	0.068	0.21906996
Gpr171	1.005E-05	0.25177745	0.206	0.247	0.22048228
Rrp12	1.0441E-05	1.1458407	0.034	0.026	0.22906709
Slc39a10	1.0673E-05	1.09560165	0.033	0.026	0.23415364
Sc5d	1.0782E-05	0.31776774	0.069	0.086	0.23654593
Thap2	1.0887E-05	0.96310484	0.065	0.056	0.23884188
Eri3	1.1326E-05	0.25247256	0.02	0.028	0.24848141
Gm37494	1.1388E-05	0.78275467	0.047	0.038	0.24984956
Bccip	1.1759E-05	0.37826035	0.097	0.119	0.25798164
Adm	1.1762E-05	0.92356849	0.01	0.006	0.25804514
St13	1.254E-05	0.2643515	0.293	0.361	0.27511694
Shprh	1.2556E-05	0.76434499	0.048	0.039	0.27546635
Tsn	1.2579E-05	0.28638072	0.209	0.252	0.27597123
Hsf1	1.3031E-05	0.74548527	0.073	0.063	0.28589334
Sirt3	1.3801E-05	0.3054556	0.016	0.023	0.30277175
Arfgef1	1.3827E-05	0.52896407	0.214	0.212	0.30335416
Dnajc2	1.4008E-05	0.3521524	0.155	0.187	0.30732457
Nars2	1.4126E-05	1.17248662	0.016	0.011	0.30991279
Ttf2	1.4238E-05	0.86315322	0.027	0.02	0.31237246
Ube2v2	1.4803E-05	0.74951614	0.124	0.114	0.32475775
Arid1b	1.5254E-05	0.71764137	0.146	0.138	0.33464883
Sec16a	1.5314E-05	0.32594608	0.065	0.081	0.33597008
Trp53bp1	1.6799E-05	0.71634592	0.058	0.048	0.36855239

Med9	1.7616E-05	0.36728204	0.029	0.038	0.38647624
Irak3	1.7933E-05	0.28795874	0.103	0.091	0.39343925
Rnf187	1.8169E-05	0.30498053	0.119	0.143	0.3986179
Camkk2	1.8182E-05	0.32297734	0.043	0.055	0.39890211
Psme2b	1.8445E-05	0.71751032	0.04	0.032	0.40466773
Patl1	1.9306E-05	0.31619957	0.079	0.096	0.42355036
Rab11fip2	1.9372E-05	0.72459801	0.087	0.077	0.42499201
Qtrt2	1.9462E-05	1.01533943	0.024	0.018	0.42697494
Fam50a	2.026E-05	0.46110775	0.113	0.138	0.44449115
Mak16	2.0419E-05	0.65211908	0.159	0.152	0.4479626
Ino80e	2.0436E-05	0.27647838	0.063	0.078	0.44835243
Cpsf2	2.114E-05	0.82817614	0.113	0.105	0.46378134
Foxk2	2.131E-05	0.27947741	0.029	0.038	0.46752002
Dmac1	2.1826E-05	0.46473638	0.096	0.117	0.47884012
Crlf3	2.2114E-05	0.25832703	0.35	0.363	0.48516492
Fgd2	2.2335E-05	0.7024112	0.06	0.075	0.49001068
Plekhg2	2.2572E-05	0.39686644	0.026	0.035	0.49521543
Ccdc134	2.34E-05	0.5207977	0.03	0.04	0.51336418
3110062M04F	2.3621E-05	1.07479888	0.036	0.028	0.51821884
Mrpl19	2.4457E-05	0.2713364	0.075	0.091	0.53656865
Tmem69	2.5015E-05	1.29765527	0.01	0.006	0.54879758
Tsr2	2.5063E-05	0.9404357	0.037	0.03	0.5498604
Tsc1	2.5748E-05	0.75207498	0.092	0.082	0.56489423
Ccdc137	2.6334E-05	0.96968361	0.024	0.018	0.5777494
Lamtor1	2.6451E-05	0.42552935	0.136	0.165	0.58030203
Gtf2ird2	2.6686E-05	1.11331908	0.014	0.009	0.58545491
Fam45a	2.6939E-05	0.27703293	0.037	0.048	0.59101245
Tmem199	2.7046E-05	1.09959425	0.028	0.022	0.59335202
Slc4a2	2.7336E-05	0.27274885	0.034	0.044	0.59972145
Ctdp1	2.8062E-05	0.26876452	0.057	0.071	0.61564693
Nup85	2.9792E-05	0.28783242	0.023	0.032	0.65360513
Acap1	2.9968E-05	0.56171245	0.112	0.101	0.65747573
Mpp1	3.0097E-05	0.31985204	0.056	0.069	0.66030347
Dpp4	3.1129E-05	0.59524492	0.056	0.07	0.68293176
Nop14	3.1548E-05	0.75680573	0.136	0.129	0.69212776
Diablo	3.1559E-05	0.31913486	0.056	0.07	0.69237332
Inafm1	3.2043E-05	1.26103188	0.02	0.014	0.70300201
Mbtps2	3.2271E-05	0.90002643	0.039	0.031	0.7079979
Stradb	3.2897E-05	1.085414	0.021	0.016	0.72172134
Gucd1	3.3248E-05	1.16512012	0.055	0.047	0.72942926
Cc2d1b	3.3258E-05	0.78419726	0.068	0.059	0.72965635
Msh3	3.3943E-05	0.83944164	0.045	0.036	0.74468115
Kmt2b	3.4161E-05	0.32762976	0.084	0.102	0.74946395
Gm42715	3.5823E-05	1.16429282	0.011	0.007	0.78591888
Mipol1	3.5855E-05	0.98538806	0.012	0.008	0.78661298
Uba6	3.588E-05	0.39880205	0.087	0.106	0.78718075
Pbxip1	3.6335E-05	0.26470179	0.169	0.2	0.79714984
Gm45051	3.7234E-05	0.87816189	0.02	0.015	0.81688529

Fip1l1	3.8606E-05	0.43046883	0.282	0.29	0.84697554
Lrba	4.003E-05	0.59874651	0.107	0.097	0.87821141
Wdr3	4.0448E-05	0.85382058	0.059	0.051	0.88738025
Apip	4.0921E-05	0.40824153	0.035	0.046	0.89776889
Tef	4.0978E-05	0.80646622	0.031	0.024	0.89901931
Gm48960	4.1379E-05	1.30966067	0.011	0.007	0.90780934
Ifi208	4.1716E-05	0.89995767	0.018	0.013	0.91520883
0610009O20R	4.1928E-05	1.04246834	0.023	0.017	0.91986777
Atg2b	4.1943E-05	0.83727077	0.035	0.028	0.92019806
Xpnpep3	4.2087E-05	0.97097895	0.022	0.016	0.92335122
Ddx17	4.2719E-05	0.44078868	0.325	0.347	0.93720857
Pmpca	4.2945E-05	0.3072519	0.057	0.071	0.94216854
Lss	4.3924E-05	1.18875586	0.012	0.008	0.96364039
Wwp2	4.3963E-05	0.28417522	0.102	0.123	0.96450871
Trmo	4.4133E-05	1.0161217	0.021	0.015	0.9682257
Arl2	4.4607E-05	0.28201878	0.02	0.028	0.97863966
Ptpa	4.487E-05	0.34625853	0.104	0.125	0.98439546
Kctd5	4.5157E-05	0.29581055	0.034	0.044	0.99068872
Glcci1	4.664E-05	0.82542461	0.032	0.025	1
Tlr1	4.6845E-05	0.33204277	0.019	0.026	1
Jagn1	4.7182E-05	0.28410331	0.03	0.039	1
Naa35	4.7954E-05	0.27844141	0.065	0.079	1
Atxn7l3	4.8689E-05	0.27476362	0.066	0.08	1
Rrp15	4.8695E-05	0.94028762	0.061	0.053	1
Gtf2e1	4.8873E-05	1.02881137	0.029	0.022	1
Gtpbp10	4.9094E-05	0.8174752	0.029	0.022	1
Poc1b	5.133E-05	0.9709323	0.025	0.019	1
Nfrkb	5.2668E-05	0.59496906	0.089	0.079	1
Casc4	5.2875E-05	0.59880258	0.023	0.017	1
Fam126b	5.4008E-05	0.76779651	0.045	0.037	1
Rpl27-ps3	5.4462E-05	0.37949024	0.084	0.101	1
Ddx1	5.5264E-05	0.27546315	0.069	0.084	1
Gnl3l	5.886E-05	0.75600994	0.082	0.072	1
Fastkd1	6.0509E-05	1.20096588	0.021	0.016	1
Pigg	6.157E-05	1.40204594	0.01	0.006	1
Vps41	6.2056E-05	0.4482882	0.077	0.094	1
Dhx58	6.2104E-05	0.46795994	0.017	0.023	1
Med1	6.2306E-05	0.31133764	0.128	0.153	1
Ogdh	6.3397E-05	0.31940363	0.158	0.188	1
Tmem230	6.4258E-05	0.33773334	0.053	0.066	1
Mgat1	6.4419E-05	0.91047016	0.052	0.044	1
Neurl4	6.4995E-05	1.09162136	0.025	0.019	1
Tsnax	6.5527E-05	0.81802827	0.055	0.046	1
Xpo1	6.6174E-05	0.70980139	0.112	0.103	1
Fbxl12	6.6352E-05	0.48286103	0.128	0.118	1
Zfp619	6.6702E-05	1.30897352	0.011	0.007	1
Ino80	6.8286E-05	0.37919117	0.082	0.099	1
Togaram1	6.8308E-05	0.31864754	0.06	0.074	1

Zxdb	6.8955E-05	0.76557198	0.047	0.039	1
Ambra1	7.1266E-05	0.28361253	0.038	0.049	1
Txndc11	7.3251E-05	0.38935416	0.124	0.147	1
Tug1	7.3359E-05	0.58867731	0.166	0.161	1
Ncor2	7.3583E-05	0.34494119	0.064	0.078	1
Cul5	7.3896E-05	0.37198453	0.086	0.104	1
Phf11a	7.4126E-05	0.32440014	0.007	0.012	1
Morc2a	7.6902E-05	0.86571478	0.045	0.037	1
Ehmt1	7.7833E-05	0.7099184	0.064	0.055	1
Prpf4	8.0866E-05	0.64462815	0.1	0.09	1
Lin9	8.136E-05	0.8927093	0.035	0.028	1
A530017D24R	8.2273E-05	1.17062416	0.013	0.009	1
Dffa	8.2616E-05	0.95185205	0.024	0.018	1
Ttc32	8.3907E-05	0.90347677	0.031	0.024	1
Rnf169	8.5644E-05	0.30294476	0.031	0.04	1
Ssh1	8.9891E-05	0.31440125	0.038	0.049	1
Ifi44	9.367E-05	0.34399435	0.006	0.011	1
Dmtf1	9.5236E-05	0.28205336	0.107	0.127	1
Suclg1	9.5243E-05	0.41093023	0.083	0.099	1
Zc3h18	9.8578E-05	0.310669	0.12	0.142	1
Sf3b4	9.9073E-05	0.35061924	0.093	0.112	1
Rcc1l	9.9685E-05	1.10649733	0.021	0.016	1
Atp8a1	0.00010089	0.81441212	0.125	0.119	1
Smarcad1	0.00010231	0.68740379	0.126	0.119	1
Tvp23b	0.00010643	0.31008315	0.034	0.043	1
Fto	0.00010656	0.75652062	0.07	0.062	1
Hist1h2ac	0.00010843	1.12916125	0.022	0.016	1
Ankrd26	0.00010856	0.91854248	0.016	0.011	1
Pacsin1	0.00010908	0.78453405	0.017	0.012	1
Trmt2b	0.00011032	0.83772355	0.031	0.024	1
Fbl	0.00011187	0.69092867	0.23	0.24	1
Mrpl11	0.00011188	0.4942699	0.085	0.102	1
Tomm40	0.00011309	0.91741553	0.075	0.066	1
Slc35c2	0.00011396	0.57092748	0.046	0.057	1
Ino80b	0.00011525	0.37060664	0.065	0.079	1
Ddx31	0.00012249	1.02513596	0.017	0.012	1
2610301B20R	0.00012342	1.07018352	0.01	0.006	1
Opa1	0.00012609	0.31037081	0.042	0.052	1
Wdr18	0.0001261	0.40169062	0.048	0.06	1
Dguok	0.00012683	0.29791241	0.074	0.089	1
Mtmr3	0.0001306	0.59579302	0.118	0.109	1
Cog7	0.00013068	0.27236786	0.017	0.024	1
Armc10	0.00013136	0.31227689	0.046	0.057	1
Stx16	0.00013256	0.67645979	0.131	0.124	1
Zmym4	0.00013545	0.83678719	0.057	0.049	1
Cwf19l1	0.00013707	1.03201542	0.015	0.01	1
Zbtb40	0.00013769	1.00376995	0.02	0.015	1
Clasrp	0.00014068	1.00570823	0.029	0.023	1

Tdp1	0.00014069	1.25590077	0.017	0.012	1
Igkv1-110	0.00014378	0.51035052	0.014	0.009	1
Map2k1	0.00014483	0.45288622	0.075	0.091	1
Cic	0.00014494	0.42226256	0.086	0.103	1
Cmtm8	0.00014524	0.67974182	0.007	0.011	1
Qser1	0.0001458	1.30508677	0.021	0.016	1
Trak1	0.00015212	0.41819962	0.059	0.072	1
Trim41	0.00015537	0.29159096	0.033	0.043	1
Klc2	0.00015807	1.22083513	0.012	0.008	1
Marveld2	0.00015878	1.2272734	0.011	0.007	1
Pcnt	0.00016139	0.25266148	0.092	0.109	1
Rnpc3	0.00016263	0.31351558	0.087	0.104	1
Smagp	0.00016603	1.24936355	0.03	0.024	1
Nmral1	0.00016719	0.37738355	0.011	0.017	1
Mlxip	0.00016829	0.61500535	0.104	0.095	1
Zfp395	0.00016889	0.73669837	0.022	0.016	1
Rfk	0.00016944	0.32137261	0.086	0.103	1
Iglv1	0.00017267	0.57813879	0.033	0.027	1
Hist1h3e	0.00017308	0.59618086	0.075	0.066	1
Nucb2	0.00017334	0.60955172	0.062	0.076	1
Brpf1	0.00017855	0.36907727	0.074	0.089	1
Pcyox1	0.00017932	0.3051222	0.015	0.021	1
Styx	0.00017979	0.84058268	0.035	0.028	1
Ankhd1	0.00017988	0.61651597	0.14	0.134	1
Cwc22	0.00018007	0.32605917	0.089	0.106	1
Usp33	0.0001806	0.86893601	0.05	0.043	1
Sav1	0.00018258	0.36347375	0.055	0.067	1
Cstf3	0.00018326	0.70040771	0.084	0.076	1
Mcmbp	0.00018788	0.68614173	0.103	0.095	1
Nubpl	0.00018995	0.31876884	0.009	0.014	1
Smg9	0.00019109	0.32831023	0.053	0.065	1
Unk	0.00019212	1.04306012	0.033	0.027	1
Hspa14	0.00019281	0.4121414	0.11	0.131	1
Nup160	0.00019535	0.83151045	0.042	0.035	1
Cntrob	0.00019573	0.82341928	0.021	0.016	1
Atp6v0d1	0.00021451	0.37624319	0.227	0.276	1
Fuca1	0.00021618	0.49631356	0.17	0.205	1
Sars	0.00021748	0.2624508	0.214	0.251	1
Rft1	0.00022548	1.18705595	0.019	0.014	1
Smurf2	0.00022718	0.41412237	0.059	0.072	1
Fam185a	0.00022723	0.8695594	0.014	0.01	1
Bola1	0.00022763	0.2674365	0.035	0.044	1
Bcl9l	0.00023084	0.85037489	0.035	0.028	1
Phrf1	0.00023537	0.32889474	0.095	0.113	1
Cobll1	0.00024504	0.50255626	0.057	0.049	1
Pecam1	0.0002451	0.48650284	0.148	0.14	1
Odf2	0.0002498	0.71331511	0.08	0.072	1
Tesk1	0.00025808	0.35882074	0.041	0.051	1

Erg28	0.00026106	0.29762697	0.065	0.078	1
Ep300	0.00026178	0.54471361	0.196	0.195	1
Ulk3	0.00026195	0.95765102	0.015	0.011	1
Trim12c	0.00026343	0.51595748	0.108	0.099	1
Tubgcp6	0.00026465	1.0127671	0.022	0.017	1
Tatdn1	0.00026528	0.31288897	0.029	0.037	1
Vps9d1	0.00026748	0.31340192	0.025	0.033	1
Isg20l2	0.00027315	0.25366044	0.064	0.077	1
Idua	0.00027783	1.07268224	0.026	0.021	1
4931406P16R	0.00027998	0.31638858	0.073	0.087	1
Cdc23	0.00028229	0.74969176	0.033	0.027	1
Ppie	0.00028238	0.45165368	0.055	0.067	1
Cdv3	0.00028352	0.44542181	0.375	0.421	1
Wdr26	0.00028668	0.51428793	0.326	0.357	1
Ankrd44	0.00029001	0.41052206	0.305	0.322	1
Ppp1r7	0.00029185	0.36813129	0.063	0.076	1
Mitd1	0.0002923	0.65547915	0.058	0.05	1
Nsmce1	0.00029907	0.38765395	0.081	0.096	1
Ccdc86	0.00030575	0.99561222	0.05	0.043	1
Gm39469	0.00031077	0.30594394	0.007	0.012	1
Sufu	0.00031123	0.90307755	0.025	0.019	1
Tsix	0.00031242	0.34397501	0.113	0.133	1
Bloc1s6os	0.00032075	1.18173436	0.01	0.007	1
Zbtb39	0.00032977	0.97530849	0.015	0.011	1
Zfp68	0.00033019	0.63563323	0.079	0.071	1
Trp53rkb	0.000336	1.03537076	0.024	0.019	1
Gcfc2	0.00033798	0.88967359	0.016	0.011	1
Htatsf1	0.00034069	0.34132414	0.093	0.11	1
Cntrl	0.00034093	0.27358911	0.143	0.167	1
Per1	0.0003438	0.25966198	0.199	0.234	1
Sun2	0.00034458	0.46994101	0.215	0.216	1
Dstyk	0.00035549	0.32674317	0.024	0.031	1
Zfp729a	0.00036914	0.7984412	0.051	0.043	1
Mre11a	0.00036955	0.80673719	0.044	0.037	1
Zfp952	0.0003697	0.73333062	0.031	0.024	1
Parvg	0.0003701	0.29668148	0.066	0.079	1
Zfp110	0.00037515	0.73560875	0.071	0.063	1
Naa25	0.00038018	0.94656368	0.055	0.047	1
C2cd5	0.00038235	0.67235442	0.054	0.047	1
Tmem138	0.0003877	1.03483874	0.014	0.01	1
Rgs14	0.00039122	0.35467305	0.022	0.029	1
Chd9	0.00040083	0.73274488	0.095	0.087	1
Gm9493	0.00040646	0.36753942	0.068	0.082	1
Cdk4	0.00041038	0.48750556	0.075	0.09	1
Csnk2a1	0.00041217	0.62762487	0.178	0.177	1
Utp18	0.00041452	0.84716177	0.111	0.105	1
Kti12	0.00041933	0.88881061	0.038	0.031	1
Uspl1	0.00042533	0.61513677	0.096	0.088	1

Tbc1d14	0.00042935	0.82059483	0.061	0.053	1
Fastk	0.00042996	0.27184672	0.028	0.036	1
Gstz1	0.00043194	1.23213354	0.013	0.009	1
Usp36	0.00044368	0.65690805	0.098	0.09	1
Mrpl1	0.00045285	0.52340726	0.036	0.045	1
Wbp4	0.0004531	0.38424539	0.163	0.192	1
Zfp866	0.00045946	0.81055616	0.029	0.023	1
Ilvbl	0.00046649	0.3308962	0.034	0.042	1
Med12	0.00046848	0.32124648	0.062	0.074	1
Hmbox1	0.00047262	0.27831858	0.046	0.057	1
Gm17275	0.00047488	0.93104801	0.021	0.016	1
Vps18	0.00047499	0.53443652	0.075	0.09	1
Gm43672	0.00047931	0.98715787	0.017	0.012	1
Bod1l	0.00047932	0.47842831	0.218	0.219	1
Cops3	0.00048035	0.7024263	0.072	0.064	1
Plcl2	0.00048204	0.73330838	0.09	0.082	1
Tmem41b	0.00049302	0.35362	0.086	0.102	1
Pank3	0.0005053	0.39015648	0.097	0.114	1
Alg1	0.00051316	0.32807259	0.021	0.027	1
Hus1	0.00051336	1.03687753	0.024	0.019	1
Bfar	0.00052039	0.77936139	0.122	0.116	1
Nsun4	0.00052686	0.62520502	0.052	0.045	1
Tbc1d1	0.00052888	0.37740864	0.112	0.131	1
Zcchc4	0.00053543	0.9202838	0.032	0.026	1
Msi2	0.00054475	0.34970515	0.226	0.223	1
Zfp871	0.00055121	0.27265447	0.154	0.179	1
Stip1	0.00056598	0.38646205	0.133	0.157	1
Tspan2	0.00056841	0.93219768	0.011	0.008	1
Rpl36a-ps1	0.000583	0.77301469	0.106	0.1	1
Rsf1	0.00058331	0.49407837	0.204	0.204	1
Zfp706	0.00059888	0.45152587	0.272	0.286	1
Ubtf	0.00060702	0.5305429	0.167	0.164	1
Xpo6	0.0006106	0.36145238	0.108	0.127	1
Ttc4	0.00061324	0.74908931	0.058	0.051	1
Tmem168	0.00061732	0.40411684	0.08	0.095	1
Gss	0.0006349	0.28669755	0.013	0.019	1
Elp3	0.00065333	0.8732474	0.024	0.019	1
Foxj2	0.00069455	0.66112155	0.071	0.063	1
Ado	0.00069754	0.26318877	0.026	0.033	1
Dpm1	0.00069971	0.2752615	0.096	0.112	1
Tcea1	0.00070632	0.28632268	0.28	0.336	1
Rabgap1l	0.00070647	0.37366747	0.192	0.186	1
Plekhf2	0.00072696	0.36677311	0.088	0.103	1
Vezf1	0.00073318	0.52884419	0.169	0.165	1
Exoc1	0.00073575	0.32422268	0.051	0.061	1
Tgfb3	0.00075458	0.93786651	0.02	0.015	1
Dus2	0.00075796	0.73620038	0.04	0.034	1
D730003I15Ri	0.00075887	0.32624092	0.03	0.037	1

Eif2b4	0.00076165	0.84434582	0.049	0.042	1
Impact	0.00077671	0.54502643	0.153	0.148	1
C330007P06R	0.0007798	0.27552667	0.069	0.082	1
Aar2	0.00079897	0.25100746	0.041	0.05	1
Ndufs1	0.00080924	0.36876438	0.078	0.092	1
Naa40	0.00081557	0.88200796	0.038	0.032	1
Zfp60	0.00084072	0.96455092	0.013	0.009	1
Ikbkb	0.00085618	0.26235416	0.263	0.313	1
Elac2	0.00085651	0.30122474	0.023	0.029	1
Agk	0.00085709	0.70435992	0.041	0.034	1
Aldh1b1	0.00085854	0.58398317	0.007	0.011	1
Zfp62	0.00086175	0.53769839	0.153	0.148	1
Ammecr1	0.0008628	1.08545116	0.018	0.013	1
Nt5c2	0.00086315	0.41466116	0.05	0.06	1
Calhm2	0.00088794	1.16977784	0.016	0.012	1
Sf3b2	0.00089339	0.38203772	0.371	0.412	1
Eif2b3	0.00090279	0.906004	0.025	0.019	1
Tceanc	0.00093755	1.00435005	0.013	0.009	1
Orc5	0.0009672	1.06455733	0.013	0.009	1
Brcc3	0.00098585	0.35904997	0.058	0.069	1
Mta2	0.00099958	0.32184098	0.115	0.134	1
Neurl2	0.00100799	1.5666786	0.011	0.008	1
Tapt1	0.00100995	0.63834673	0.057	0.05	1
Gpn1	0.00101093	0.81715014	0.044	0.038	1
Nme1	0.00101909	0.32095331	0.282	0.341	1
Insig1	0.00102412	0.44119029	0.127	0.15	1
1600002K03R	0.00105549	0.33675441	0.045	0.054	1
Rnf220	0.00105647	0.59575754	0.131	0.126	1
Sae1	0.00105649	0.26540637	0.058	0.068	1
Pus7l	0.00106109	1.23577377	0.011	0.008	1
Smarcb1	0.00106472	0.44736128	0.069	0.082	1
Polr3b	0.0010764	0.77632421	0.033	0.027	1
Clspn	0.0010952	0.6241947	0.025	0.02	1
Ubxn2b	0.00109874	1.19470049	0.01	0.007	1
Ccz1	0.00110586	0.36827647	0.114	0.134	1
Prkdc	0.00110617	0.74205012	0.04	0.034	1
Ago1	0.00110878	0.84014095	0.047	0.04	1
Mdm1	0.00112182	0.88083715	0.012	0.009	1
Dnal4	0.00114902	0.30994174	0.008	0.011	1
Caprin1	0.0011517	0.53655999	0.268	0.286	1
Trmt10c	0.0011692	0.72426251	0.057	0.05	1
Rabgap1	0.0011842	0.69763682	0.073	0.066	1
Pign	0.00119867	0.29842331	0.028	0.035	1
Surf2	0.00121818	0.8669967	0.037	0.031	1
Uri1	0.00124776	0.62923828	0.09	0.083	1
Tbrg4	0.0012613	0.43761797	0.038	0.046	1
Rnf214	0.00126354	0.35407295	0.03	0.038	1
Add1	0.00126727	0.32253442	0.103	0.121	1

Dcp1a	0.00128672	0.28839116	0.052	0.062	1
Wdr81	0.0013017	1.02172318	0.033	0.027	1
Smad5	0.00130544	0.73131857	0.047	0.041	1
Mtmr2	0.00131409	0.33293579	0.05	0.059	1
Cryzl1	0.00134948	0.28730329	0.032	0.039	1
Tubgcp4	0.00135321	0.67716629	0.069	0.062	1
Acin1	0.00135601	0.36134274	0.343	0.371	1
Nelfa	0.001358	0.79606659	0.051	0.045	1
Acox1	0.00137885	0.52405445	0.034	0.041	1
Psmg3	0.00140124	0.31154068	0.044	0.053	1
Nfatc2ip	0.00140277	0.69117565	0.05	0.043	1
Rpa3	0.00142389	0.32271038	0.047	0.056	1
Spc25	0.00143984	1.32495642	0.013	0.009	1
Mtm1	0.00148329	0.43178434	0.028	0.035	1
2310061I04Ril	0.00148825	0.28740602	0.022	0.028	1
Ermard	0.00149671	0.91957035	0.025	0.02	1
E130309D02R	0.00151782	0.2834528	0.044	0.052	1
1110038F14Ri	0.00152081	0.33250643	0.094	0.109	1
Elf2	0.00155934	0.28608732	0.186	0.217	1
Poglut1	0.00156383	0.47308007	0.027	0.034	1
Sssca1	0.00157204	0.31272708	0.038	0.046	1
Pum1	0.001588	0.4695742	0.237	0.245	1
1190007I07Ril	0.00159137	0.26626161	0.027	0.034	1
Limd1	0.00159682	0.68510539	0.17	0.171	1
Prpf6	0.00161495	0.62232455	0.127	0.122	1
Polm	0.00170864	0.87751261	0.02	0.016	1
Adnp2	0.00172708	0.26042142	0.03	0.037	1
Klhl18	0.0017439	0.79842591	0.033	0.028	1
Tgfbrap1	0.00175042	0.3621251	0.024	0.031	1
Riox2	0.0017608	0.39875308	0.039	0.048	1
Zfp113	0.00177033	0.71167818	0.017	0.013	1
Dbnl	0.00177136	0.45447078	0.131	0.153	1
Rabl3	0.00178424	0.95538172	0.028	0.023	1
Nup54	0.00179989	0.34345919	0.079	0.092	1
Ankrd17	0.00180547	0.28430159	0.208	0.242	1
Ap1ar	0.00181261	0.29407137	0.056	0.066	1
Rpa1	0.0018156	0.59114482	0.066	0.058	1
Irak1	0.00185625	0.71744556	0.153	0.152	1
Orc3	0.00186757	0.78122581	0.049	0.043	1
Nipbl	0.00187175	0.33015525	0.38	0.418	1
Flad1	0.00187617	0.27409792	0.014	0.018	1
Smim8	0.00187736	0.32989654	0.047	0.056	1
Phf12	0.00191546	0.71142925	0.074	0.067	1
Pogz	0.00193262	0.77865932	0.051	0.045	1
Gstcd	0.0019534	0.74787019	0.023	0.019	1
Oma1	0.00195466	0.2784694	0.012	0.016	1
Mtif2	0.00196458	0.73603251	0.04	0.034	1
Dennd1b	0.00196793	0.25416323	0.185	0.214	1

Ints13	0.00197304	0.3221919	0.056	0.066	1
Srrt	0.0019777	0.40272192	0.121	0.141	1
Rad1	0.00198255	0.8048633	0.019	0.015	1
Nol12	0.00201734	0.5066996	0.06	0.071	1
Ivd	0.00205463	0.27586876	0.023	0.029	1
Champ1	0.00208067	0.77636602	0.041	0.035	1
Rpap2	0.00209045	0.73040683	0.03	0.025	1
Snx16	0.00212716	0.27678349	0.016	0.021	1
Zfp943	0.00216187	0.8481534	0.029	0.024	1
Cabin1	0.00217928	0.33676558	0.088	0.102	1
Foxred1	0.00220464	0.86703124	0.029	0.024	1
Btf3l4	0.00222646	0.97923268	0.029	0.024	1
Iws1	0.00226715	0.57045831	0.167	0.167	1
Galnt1	0.00226984	0.30513886	0.177	0.205	1
Jrkl	0.00227539	0.38201165	0.037	0.044	1
Hcfc1	0.00228153	0.72730777	0.083	0.076	1
Hcfc2	0.00230186	0.70410414	0.036	0.03	1
Cinp	0.00232382	0.32007259	0.021	0.027	1
Ate1	0.0023514	0.39227735	0.073	0.086	1
Tmx2	0.00235674	0.79314975	0.034	0.029	1
Fbxw5	0.00236384	0.37512257	0.024	0.031	1
0610037L13Ri	0.00237729	0.52326908	0.056	0.067	1
Stag2	0.00238594	0.26930388	0.219	0.255	1
Sympk	0.00238927	0.38740873	0.053	0.063	1
Clk2	0.00239558	0.74265692	0.054	0.048	1
Alkbh6	0.00242549	0.32110994	0.052	0.062	1
Rnf146	0.00243684	0.31072101	0.115	0.133	1
Fam126a	0.00243894	0.3827269	0.063	0.074	1
Mrgbp	0.00246448	0.68929026	0.042	0.036	1
Samd1	0.00249168	0.38969381	0.02	0.025	1
Scaf4	0.00250571	0.84697419	0.044	0.038	1
Arhgap11a	0.00255566	0.4264358	0.029	0.035	1
Fam20b	0.00259262	0.33273543	0.032	0.039	1
Lsg1	0.00259284	0.70632626	0.092	0.086	1
Rab2b	0.00260369	0.57810913	0.048	0.041	1
Ubn2	0.00261289	0.56397299	0.19	0.192	1
B130006D01R	0.00267683	0.87405017	0.013	0.01	1
Vrk2	0.00268273	0.38834576	0.056	0.066	1
Usp7	0.00271121	0.31007382	0.235	0.276	1
Pex6	0.0027191	0.3083331	0.024	0.03	1
Lbr	0.00276471	0.36923351	0.243	0.249	1
Rbmx2	0.00281643	0.26450852	0.044	0.052	1
Cep128	0.00283055	1.00557295	0.021	0.016	1
Ppip5k2	0.0028306	0.66373792	0.06	0.054	1
Rnf113a2	0.00283438	0.35805214	0.049	0.058	1
Ppp4r3a	0.00285721	0.28966973	0.197	0.228	1
Fam53a	0.00288364	0.68008606	0.041	0.036	1
Mrps31	0.00291265	0.25647488	0.032	0.039	1

Zfp251	0.00294317	0.70822633	0.012	0.009	1
Ankrd10	0.0030361	0.50492562	0.072	0.065	1
Itpr2	0.00307187	0.32849322	0.209	0.207	1
Rbm12	0.00308506	0.32595147	0.055	0.064	1
Zfp317	0.00308544	0.72916654	0.025	0.02	1
Zbtb44	0.00309496	0.47320639	0.109	0.102	1
Dhrs7b	0.00312323	0.35246847	0.025	0.031	1
Itfg1	0.00314355	0.42588464	0.046	0.055	1
Tpcn1	0.00317861	0.51154443	0.027	0.034	1
Arfgap1	0.00320033	0.40623162	0.044	0.052	1
Cacybp	0.00322339	0.60405468	0.18	0.181	1
Tbccd1	0.00322657	1.043479	0.015	0.012	1
Dnmt3b	0.00322689	1.12995639	0.01	0.007	1
6230400D17R	0.00326774	0.92974199	0.015	0.011	1
Cul4b	0.0033123	0.66278521	0.054	0.048	1
Gdi1	0.00332099	0.57938076	0.117	0.112	1
Kcnk5	0.00333915	0.66515198	0.015	0.011	1
Abcf3	0.00335123	0.25571449	0.045	0.054	1
Zkscan3	0.00341926	0.67107149	0.072	0.066	1
Thoc5	0.00341997	0.35619721	0.023	0.028	1
Usp5	0.00350195	0.36538512	0.037	0.045	1
Mapk8ip3	0.00353418	0.26965815	0.067	0.078	1
Rp9	0.00356336	0.308385	0.236	0.276	1
Atl3	0.00359156	0.29901114	0.1	0.115	1
Nisch	0.0035946	0.25650981	0.216	0.251	1
Nlrc4	0.00360634	1.3168026	0.018	0.014	1
Vps52	0.00364149	0.42267432	0.034	0.042	1
Rpl7l1	0.00365636	0.47536519	0.088	0.102	1
Gm26882	0.00366895	0.87765816	0.028	0.024	1
Fam98a	0.00372439	0.69513779	0.033	0.028	1
Papd5	0.00373815	0.27083434	0.072	0.083	1
Mrpl10	0.00374584	0.32813131	0.039	0.046	1
Ptrh2	0.00375238	0.28027433	0.041	0.049	1
Ogfod2	0.00375904	0.26601216	0.02	0.026	1
Lrrc42	0.00378545	0.32039302	0.02	0.025	1
G6pc3	0.00383646	0.48646736	0.027	0.034	1
Trim30a	0.00384642	0.41525497	0.14	0.161	1
E2f2	0.00386803	0.33939607	0.015	0.02	1
Ireb2	0.00386941	0.66864173	0.117	0.112	1
Pi4ka	0.00395862	0.35148566	0.072	0.083	1
A330069E16R	0.00399441	0.81510547	0.011	0.008	1
Hdac1	0.0040099	0.3374989	0.101	0.116	1
Gm17259	0.00404187	1.31704828	0.01	0.007	1
Farsa	0.00407671	0.77779122	0.067	0.061	1
Nelfb	0.00409571	0.29142742	0.074	0.086	1
Srfbp1	0.00409678	0.32992769	0.03	0.036	1
Szt2	0.00410563	0.81732233	0.016	0.013	1
Thoc1	0.00413878	0.38975483	0.035	0.042	1

Rbsn	0.00415011	0.35470928	0.034	0.041	1
2510002D24R	0.00417768	0.49603842	0.084	0.078	1
Msh6	0.00419741	0.8183906	0.027	0.022	1
Mtx3	0.00420126	0.85204242	0.013	0.01	1
Rnf167	0.00421563	0.30248522	0.097	0.111	1
Rrnad1	0.00426343	0.79995734	0.041	0.036	1
Cep192	0.0042675	0.56496486	0.083	0.076	1
Nomo1	0.00436264	0.37582525	0.028	0.034	1
BC005561	0.00437025	0.37284891	0.095	0.11	1
Tmem185a	0.00437075	0.27522674	0.014	0.018	1
Hmgxb3	0.00437884	0.318288	0.027	0.033	1
Sltm	0.00439285	0.28432608	0.274	0.32	1
Dlst	0.0044106	0.63648542	0.177	0.18	1
Thap4	0.00443938	0.25026055	0.014	0.019	1
Itpr1	0.00444387	0.25553435	0.177	0.203	1
Cdc25b	0.00446595	0.5270978	0.03	0.025	1
Mkl2	0.00448327	0.28260393	0.015	0.02	1
Haus7	0.00458655	0.34720946	0.018	0.023	1
Mettl25	0.00465985	1.11649778	0.015	0.011	1
Gtf3c1	0.0046821	0.29000916	0.06	0.07	1
Dpp9	0.00469064	0.49407112	0.041	0.049	1
Bdp1	0.00469795	0.55198188	0.169	0.169	1
Tspyl2	0.00478228	0.81201064	0.036	0.031	1
Pms1	0.00485301	0.92482293	0.014	0.01	1
Ap5m1	0.00496529	0.29080805	0.039	0.046	1
Eif2b1	0.00498435	0.28962929	0.025	0.031	1
Ints2	0.00499457	0.89566921	0.023	0.019	1
Taf1a	0.00506416	0.89394809	0.019	0.015	1
Gm8186	0.00506645	0.32770585	0.068	0.078	1
Cenpc1	0.00508347	0.2923425	0.054	0.063	1
Agl	0.00509909	0.26860246	0.011	0.015	1
Dus1l	0.00515136	0.35517516	0.025	0.03	1
Lats2	0.0052955	0.74167976	0.136	0.135	1
Inpp5k	0.005306	0.3173784	0.053	0.062	1
Pdxk	0.00533138	0.47404601	0.081	0.094	1
Zzef1	0.00538548	0.293907	0.106	0.121	1
Mrm1	0.0053884	0.97032375	0.015	0.011	1
Klf16	0.00540251	0.25230914	0.022	0.027	1
Nrf1	0.00540839	0.65505224	0.086	0.08	1
Med4	0.00541592	0.33527327	0.089	0.102	1
Actr6	0.00541594	0.71140471	0.025	0.02	1
Poll	0.00554338	0.88885563	0.012	0.009	1
Rps6kb1	0.00557994	0.56707385	0.169	0.169	1
Anapc16	0.00563669	0.40578524	0.108	0.124	1
Pelp1	0.00567002	0.32803441	0.033	0.039	1
Dhx33	0.00569288	0.95655646	0.024	0.02	1
Epb41l4aos	0.00570578	0.37063898	0.162	0.188	1
Tmem63a	0.00575815	0.30522851	0.027	0.033	1

Dis3l2	0.00578219	0.32818013	0.037	0.044	1
Snx14	0.00579179	0.34349048	0.035	0.041	1
Tmem68	0.0057934	0.75225417	0.048	0.043	1
Ppp1r11	0.0058114	0.26005543	0.24	0.279	1
9430015G10R	0.00581854	1.04901319	0.01	0.007	1
Glmn	0.0058998	0.80539543	0.019	0.015	1
Kansl2	0.00596337	0.71981301	0.063	0.058	1
Ncoa6	0.00599478	0.38647931	0.066	0.077	1
Ftsj1	0.00599631	0.82669823	0.016	0.013	1
Zfp148	0.00611772	0.52642306	0.175	0.176	1
Rmi1	0.00624539	0.61214685	0.056	0.05	1
Gmfb	0.00625889	0.35825442	0.17	0.196	1
5430403G16R	0.00632673	0.84670817	0.01	0.008	1
Gm20300	0.00633272	0.89658139	0.015	0.012	1
Pom121	0.00636109	0.31510326	0.104	0.119	1
Gnpat	0.00639292	0.75896591	0.042	0.037	1
Nae1	0.00648926	0.40080964	0.089	0.102	1
Clpp	0.00658626	0.39141297	0.04	0.048	1
Dnttip2	0.00659604	0.31718162	0.182	0.21	1
Rdm1	0.00661535	0.28855967	0.014	0.018	1
Jmjd6	0.00675643	0.34147986	0.074	0.086	1
Cisd2	0.00682861	0.32623759	0.251	0.297	1
Csnk1g2	0.00688258	0.5387122	0.158	0.157	1
Tacc1	0.00688526	0.26908816	0.296	0.351	1
Prr3	0.00700482	0.88914348	0.033	0.029	1
Fbxl4	0.00703524	1.10628554	0.01	0.008	1
Zfp407	0.00710965	0.72611736	0.066	0.061	1
Nemf	0.0071354	0.56204327	0.15	0.148	1
Yars2	0.00716307	0.84536377	0.019	0.015	1
Dscr3	0.00716553	0.40416802	0.019	0.024	1
Synj2bp	0.00720376	0.26799613	0.089	0.102	1
Utp14a	0.00720422	0.74456757	0.119	0.116	1
Sp3os	0.0072139	0.28634774	0.034	0.041	1
Rgp1	0.00722191	0.88444682	0.023	0.019	1
Pms2	0.00729034	0.54489354	0.038	0.033	1
Fgd6	0.00729108	0.92225889	0.023	0.019	1
Ahsa2	0.0073336	0.3499592	0.041	0.048	1
Micall1	0.00737908	0.54521909	0.035	0.042	1
C730034F03R	0.00739405	0.56298793	0.025	0.021	1
Ints9	0.00739425	0.37614097	0.03	0.036	1
Chordc1	0.00740429	0.69890598	0.118	0.115	1
Pde2a	0.0074987	0.99272845	0.032	0.028	1
Fra10ac1	0.0075091	0.8077966	0.021	0.017	1
Clns1a	0.00756106	0.44938173	0.082	0.095	1
Cnot10	0.0076248	0.38713425	0.054	0.063	1
Fkbp15	0.00767849	0.38212542	0.091	0.104	1
Zer1	0.00772196	0.55450383	0.05	0.044	1
Snx27	0.00779516	0.33716669	0.032	0.038	1

Zfp346	0.00783137	0.75369993	0.015	0.011	1
Ndst2	0.00783889	0.44940767	0.015	0.019	1
Wdr46	0.00786877	1.0045394	0.028	0.024	1
BC035044	0.00789924	0.73635479	0.02	0.025	1
Tbc1d25	0.00790574	0.83884562	0.011	0.009	1
Trim37	0.00794809	0.25660565	0.016	0.02	1
Ttll5	0.00799089	0.85751877	0.021	0.017	1
Ptpn4	0.00803049	0.28161166	0.032	0.038	1
Scaf1	0.00810293	0.34674974	0.055	0.064	1
Ripk2	0.00814671	0.3587087	0.07	0.081	1
Tsc22d1	0.00827325	0.71083918	0.047	0.055	1
Prps1	0.0083229	0.29836412	0.077	0.087	1
6030458C11R	0.00835393	0.75105196	0.027	0.023	1
Smg5	0.00836882	0.61154348	0.107	0.103	1
Rreb1	0.00839326	0.87765835	0.052	0.047	1
Nbr1	0.00847459	0.33324312	0.081	0.092	1
Mbtps1	0.00850591	0.57146621	0.136	0.133	1
Naf1	0.00853411	0.41283566	0.036	0.043	1
Prdm11	0.00860246	0.72242938	0.021	0.017	1
Ttc5	0.00863752	0.59769999	0.072	0.066	1
Adck2	0.00868442	0.27446219	0.014	0.018	1
Slc41a2	0.00876856	0.30146338	0.017	0.021	1
Ankrd13c	0.00883765	0.29327049	0.113	0.129	1
Mrps35	0.00884911	0.36904226	0.049	0.057	1
Supv3l1	0.00887696	0.34365442	0.026	0.031	1
Pla2g12a	0.00892697	0.99091269	0.025	0.021	1
U2af2	0.00892915	0.56153247	0.211	0.221	1
mt-Atp8	0.00895644	0.50696918	0.193	0.197	1
Bcdin3d	0.00904492	0.75939279	0.019	0.016	1
Brms1l	0.00920047	0.7062055	0.021	0.017	1
Mta3	0.00963882	0.67501824	0.086	0.082	1
Gpd2	0.00968933	0.72451258	0.151	0.153	1
St14	0.00986476	1.01409261	0.013	0.01	1
Dtymk	0.00991933	0.73981135	0.063	0.057	1
Tbc1d17	0.01006156	0.72010954	0.059	0.054	1
Pot1a	0.01010524	0.53413455	0.046	0.04	1
Pa2g4	0.01014072	0.45805925	0.145	0.167	1
Tcf20	0.01019657	0.31573968	0.116	0.131	1
Haus2	0.01035671	0.30863861	0.036	0.042	1
Utp15	0.01043421	0.69983283	0.068	0.063	1
Zdhhc6	0.01067612	0.80413417	0.046	0.041	1
Msmo1	0.01074745	0.90442582	0.117	0.115	1
Xrcc4	0.01075637	0.33323124	0.027	0.032	1
Xab2	0.01098987	0.40883653	0.05	0.058	1
Sh3bp2	0.01100266	0.81938919	0.042	0.049	1
Pex3	0.01107684	0.29879434	0.044	0.051	1
Zfp958	0.01129464	0.74475507	0.035	0.031	1
Akt1s1	0.01136873	0.34397726	0.022	0.027	1

Ints6	0.01152107	0.29434263	0.08	0.091	1
Zfp869	0.01158968	0.69953033	0.02	0.017	1
Ankfy1	0.01169109	0.49827398	0.091	0.104	1
Gm5914	0.01175545	0.56663177	0.026	0.022	1
Strn4	0.01179752	0.45185121	0.061	0.071	1
Lsm4	0.01182899	0.45325598	0.303	0.333	1
Gm30054	0.01184425	0.5037252	0.034	0.029	1
Gm26518	0.01185738	0.65385563	0.071	0.066	1
Akap10	0.01187589	0.56919338	0.073	0.068	1
Coq6	0.01187611	0.31028497	0.011	0.015	1
Ctc1	0.01188057	0.76240331	0.026	0.022	1
Tmem57	0.01203538	0.74051672	0.1	0.097	1
Trappc12	0.01214925	0.64263156	0.054	0.048	1
Zdhhc8	0.01215241	0.27740139	0.02	0.024	1
Nip7	0.0122512	0.76369077	0.11	0.107	1
Lrrc61	0.01230166	0.97189519	0.018	0.015	1
Zcchc10	0.01233118	0.26179769	0.064	0.073	1
9030025P20R	0.01238353	1.16252649	0.01	0.007	1
Actr5	0.01243634	0.25436602	0.042	0.048	1
Ifngr2	0.01254066	0.48348939	0.14	0.161	1
Ttc27	0.01288202	0.84225702	0.018	0.014	1
Shq1	0.01295795	1.05727269	0.011	0.008	1
Cdk2	0.01296865	0.87379995	0.029	0.025	1
Fbxw9	0.01298671	0.38601393	0.007	0.01	1
Zfp65	0.01298866	0.33515372	0.02	0.024	1
Prdm15	0.01300038	0.94932556	0.015	0.012	1
Jpt2	0.01303803	0.58881558	0.02	0.024	1
Gpank1	0.01304669	0.30592439	0.059	0.068	1
Ggact	0.01331853	0.83053226	0.011	0.008	1
Tnrc6a	0.01335149	0.46968695	0.2	0.204	1
Usp10	0.01340008	0.73006512	0.069	0.064	1
N4bp3	0.01342658	0.9354226	0.011	0.008	1
Wdr6	0.013442	0.72175309	0.019	0.015	1
Dync2h1	0.0134708	0.91615948	0.013	0.01	1
Blzf1	0.01354347	0.40423297	0.036	0.042	1
Senp1	0.0136723	0.34957802	0.07	0.079	1
Carmil2	0.01371497	0.55800711	0.028	0.024	1
Rnaseh1	0.01373214	0.32931647	0.022	0.027	1
Arhgap27	0.01376445	0.82115628	0.03	0.026	1
Aaed1	0.01383799	0.48999809	0.049	0.056	1
Zfp868	0.01389667	0.32172369	0.048	0.055	1
Usp18	0.01392	0.30694641	0.014	0.018	1
Gcc2	0.01397417	0.31253871	0.112	0.127	1
Abcd3	0.01404676	0.38590253	0.012	0.016	1
Polr3a	0.01408556	0.8251403	0.042	0.037	1
Naa30	0.01410511	0.27208932	0.063	0.072	1
Cystm1	0.01410717	0.75056358	0.087	0.083	1
Lig3	0.01435126	0.83350833	0.047	0.042	1

Cct3	0.01446127	0.60292375	0.21	0.222	1
H2-T24	0.0145276	0.91834961	0.01	0.007	1
Dtwd2	0.01490974	1.11535584	0.012	0.009	1
Fam120b	0.01491137	0.6553831	0.062	0.057	1
Birc3	0.01503657	0.30419932	0.387	0.426	1
Nbas	0.01506736	0.35160461	0.025	0.03	1
Lrp10	0.01508379	0.3799399	0.203	0.235	1
Cmss1	0.01509501	0.37504285	0.029	0.034	1
Lsm3	0.01514211	0.35051159	0.143	0.163	1
Camsap2	0.01522398	0.83161022	0.048	0.043	1
Slx4ip	0.01526872	0.72308019	0.025	0.022	1
Gm44777	0.01536449	0.80395658	0.013	0.011	1
Hltf	0.01537232	0.29546509	0.086	0.097	1
Zfp90	0.01538361	0.30635406	0.011	0.014	1
Borcs7	0.01574343	0.56352375	0.058	0.066	1
Lsm14a	0.01599876	0.50867263	0.181	0.184	1
Zc3h7b	0.01617976	0.69949216	0.032	0.028	1
Scnm1	0.01650183	0.41500099	0.026	0.031	1
Exosc9	0.01654898	0.39630823	0.042	0.049	1
Pspc1	0.01660609	0.33906601	0.11	0.124	1
Rnf26	0.0166402	0.28307879	0.015	0.019	1
Med17	0.01668385	0.32536191	0.045	0.051	1
Mecp2	0.0166915	0.62391879	0.131	0.129	1
Trit1	0.01670766	0.86396816	0.028	0.024	1
Zcwpw1	0.01682082	1.09321516	0.015	0.012	1
Cnot2	0.01685318	0.59166916	0.135	0.134	1
Polr2e	0.01688342	0.39749301	0.135	0.154	1
Zfp984	0.0169331	0.31389491	0.025	0.03	1
Maea	0.016939	0.35602928	0.097	0.11	1
Msl3	0.01694595	0.38161277	0.052	0.06	1
Entpd5	0.01695013	0.80888167	0.024	0.021	1
Sf3a3	0.01702674	0.41150356	0.088	0.1	1
Cpsf3	0.01705005	0.90353905	0.053	0.049	1
Fbxo22	0.01716735	0.34984261	0.043	0.05	1
Fam8a1	0.01725168	0.65407086	0.049	0.045	1
4921524J17Ril	0.01732134	0.27091522	0.097	0.108	1
Smarcc1	0.01746231	0.4266892	0.09	0.103	1
Nsun2	0.01748348	0.75454327	0.091	0.088	1
Cdc73	0.01757652	0.31444626	0.147	0.166	1
Puf60	0.01764286	0.33981117	0.217	0.251	1
Zfp236	0.01765307	0.37108484	0.044	0.051	1
Kmt2d	0.01783896	0.30444685	0.11	0.124	1
Prr12	0.01802714	0.68027356	0.018	0.014	1
Alg6	0.01803326	0.32373841	0.008	0.01	1
Cenpq	0.01812331	0.44642167	0.092	0.087	1
Rpp38	0.01819392	0.87626019	0.027	0.024	1
Nfs1	0.01825282	0.28171721	0.027	0.032	1
Klhl20	0.01831377	0.62018183	0.038	0.033	1

Rnpepl1	0.01838286	0.33664623	0.054	0.062	1
Prdm2	0.018466	0.25548284	0.125	0.141	1
Aatf	0.01860423	0.75433496	0.058	0.053	1
Timmdc1	0.01865728	0.39849792	0.013	0.017	1
Gm45902	0.01880079	0.86778545	0.025	0.022	1
Cog4	0.01880856	0.38295876	0.045	0.052	1
Nudt6	0.01891599	0.75764275	0.011	0.009	1
Rbm15b	0.01892612	0.34176307	0.038	0.044	1
Mgat4a	0.01903853	0.54174044	0.129	0.127	1
Sf3a1	0.01912967	0.37190672	0.092	0.104	1
Tfdp2	0.01926916	0.25405877	0.03	0.035	1
Setd1b	0.01941696	0.27179241	0.067	0.076	1
Rgl2	0.01949842	0.52480182	0.033	0.039	1
Mcoln1	0.01951637	0.41373898	0.023	0.027	1
Zfp568	0.01961036	0.92064071	0.014	0.011	1
Cars2	0.01961813	0.29846983	0.036	0.042	1
Ints6l	0.01995218	0.29878315	0.04	0.046	1
Inip	0.0200582	0.64593267	0.074	0.069	1
Polr2b	0.02009255	0.33015719	0.062	0.071	1
Prpf19	0.0202478	0.44797632	0.066	0.075	1
Alg3	0.02032617	0.40821432	0.012	0.016	1
Ikbkap	0.0206499	0.66780352	0.036	0.031	1
Pi4kb	0.02070224	0.70916336	0.027	0.023	1
Cherp	0.0207285	0.35613421	0.063	0.072	1
Crtc1	0.02085408	0.97720788	0.011	0.008	1
Tmem107	0.02108061	0.46193376	0.009	0.011	1
Cdk12	0.02108289	0.28775703	0.249	0.288	1
Hlcs	0.02117572	0.80327651	0.036	0.032	1
Chml	0.02120322	0.96076089	0.016	0.013	1
Zdhhc4	0.02120601	0.43849845	0.03	0.035	1
Usp22	0.02130139	0.47986753	0.058	0.066	1
Ofd1	0.02132403	0.37259934	0.027	0.032	1
Rnf141	0.02151307	0.84508919	0.049	0.044	1
Zfp780b	0.02172578	0.76001916	0.025	0.022	1
Atp5s	0.02185219	0.84507766	0.012	0.01	1
C030034I22Ri	0.02205257	0.60594199	0.053	0.048	1
Atad1	0.02223365	0.49978813	0.094	0.107	1
1110032A03R	0.02230262	0.96460789	0.034	0.03	1
Phka2	0.02236793	0.6606092	0.02	0.024	1
Cstf2t	0.02246249	0.40549716	0.038	0.044	1
Phkb	0.02246714	0.72319961	0.035	0.031	1
Hist1h4i	0.02269351	0.40162866	0.067	0.076	1
Cox18	0.02274544	0.32639952	0.027	0.032	1
Parp2	0.02278476	0.80017522	0.069	0.065	1
Zfp652	0.02281132	0.34660273	0.076	0.086	1
Gm28707	0.02283935	0.46807138	0.011	0.014	1
Zfp369	0.02302811	0.70804032	0.029	0.025	1
Ccdc191	0.02307848	0.98270025	0.012	0.009	1

Ralgapa1	0.02319165	0.48286402	0.099	0.095	1
Mfn1	0.02323194	0.48292498	0.027	0.032	1
Cep44	0.02328402	0.46124532	0.064	0.059	1
Mlst8	0.02339144	0.88233994	0.019	0.015	1
Snrnp48	0.02342732	0.4555302	0.194	0.199	1
Taf6	0.02343217	0.79241014	0.037	0.033	1
9930104L06Ri	0.02348366	0.90322624	0.01	0.008	1
Zfp282	0.02370205	0.28316471	0.015	0.018	1
4930524J08Ril	0.02390251	0.32254364	0.009	0.011	1
Atp2c1	0.02392414	0.49290291	0.08	0.091	1
Zfp180	0.023963	0.76295525	0.046	0.042	1
Rmdn3	0.02415637	0.4378808	0.024	0.029	1
Nkrf	0.02421258	0.84446072	0.018	0.015	1
Zfp729b	0.02427029	0.66043117	0.043	0.039	1
Zdhhc16	0.02429527	0.3146676	0.016	0.019	1
Supt7l	0.02471371	0.71469467	0.032	0.028	1
Lin52	0.02477301	0.73791182	0.023	0.02	1
Trp53rka	0.02515466	0.35058818	0.029	0.034	1
Igip	0.02527716	0.79363109	0.014	0.011	1
Gm29361	0.0254154	0.78738191	0.013	0.011	1
Mtch2	0.02563371	0.46576488	0.161	0.185	1
Atad2b	0.02569473	0.61175296	0.121	0.119	1
Col27a1	0.02571237	0.41364047	0.008	0.01	1
Dhrs11	0.02579029	0.33497542	0.008	0.01	1
Tada1	0.02579411	0.29893092	0.042	0.048	1
Primpol	0.02604903	0.71121349	0.022	0.018	1
Pdik1l	0.02609104	0.72975485	0.028	0.024	1
Cklf	0.0263751	0.72714855	0.118	0.117	1
Gm26759	0.02667537	0.48999655	0.028	0.024	1
Apobec1	0.02677265	0.49363815	0.052	0.059	1
Rcbtb1	0.02677322	0.33841004	0.032	0.037	1
Zkscan14	0.02680831	0.65199541	0.051	0.047	1
Rbm12b2	0.02709206	0.70470901	0.016	0.013	1
Mrpl45	0.02720701	0.49325131	0.068	0.077	1
Ndufaf6	0.02736912	0.34869227	0.012	0.015	1
Gpn2	0.02743504	0.4174147	0.016	0.02	1
Trmt44	0.02751594	0.91151506	0.021	0.018	1
Fam204a	0.02764095	0.52101493	0.216	0.226	1
Adck1	0.02783216	0.41123839	0.016	0.019	1
Exosc7	0.02789435	0.59829161	0.044	0.04	1
Gid4	0.02825776	0.39140751	0.048	0.055	1
Prps2	0.02870552	0.25804295	0.051	0.057	1
Mon2	0.02874415	0.40068615	0.109	0.123	1
Smarcd1	0.02912026	0.31417308	0.066	0.075	1
Dph7	0.02912751	0.70835227	0.015	0.012	1
Phb	0.02917399	0.35281962	0.023	0.027	1
Poc5	0.02933436	0.73026389	0.028	0.024	1
Gm4707	0.02949929	0.89475909	0.043	0.039	1

Nrbp1	0.02951029	0.44641474	0.189	0.219	1
Luc7l	0.02989931	0.54190825	0.129	0.127	1
Tti1	0.02991974	1.07686662	0.011	0.009	1
Osbpl7	0.02992132	0.68732329	0.026	0.022	1
Spata24	0.02992177	0.6768823	0.019	0.016	1
Msto1	0.03010796	0.35372301	0.014	0.017	1
Zzz3	0.03027887	0.62500171	0.116	0.114	1
Zfp593	0.03040386	0.55658988	0.098	0.111	1
2610008E11R	0.03045939	0.45817314	0.032	0.028	1
Exd2	0.03048833	0.27802991	0.014	0.017	1
Mif4gd	0.03072057	0.43563121	0.212	0.219	1
Kantr	0.03121353	0.65387214	0.019	0.016	1
Capn15	0.03126519	0.26723019	0.038	0.043	1
Rhot2	0.03135773	0.28621283	0.021	0.025	1
Dcp1b	0.03146849	0.81159513	0.015	0.013	1
Bud23	0.03155025	0.52022894	0.047	0.054	1
Brd7	0.03166076	0.45505383	0.135	0.153	1
Stard5	0.03171254	0.40708415	0.022	0.026	1
Rfc4	0.03201665	0.28523446	0.023	0.027	1
Pithd1	0.0321252	0.69776122	0.044	0.04	1
Riok2	0.03225997	0.69357131	0.049	0.044	1
L3mbtl2	0.03240288	0.69065012	0.02	0.017	1
Tssc4	0.03251086	0.42077531	0.034	0.039	1
Dnajb2	0.03259025	0.59726266	0.099	0.097	1
Usp40	0.03259552	0.81451018	0.028	0.025	1
Gfm1	0.03289584	0.48046275	0.024	0.028	1
Ddx19a	0.03290942	0.42494603	0.06	0.068	1
Arl5b	0.03304185	0.47009785	0.149	0.17	1
Zfp738	0.03312472	0.31031694	0.029	0.034	1
Ppp2r3d	0.03353299	0.58437012	0.037	0.042	1
Actl6a	0.03363011	0.43125044	0.099	0.111	1
Snrnp200	0.03364814	0.39221806	0.153	0.174	1
Npat	0.03365513	0.31123048	0.048	0.054	1
Armc8	0.03406109	0.64345919	0.052	0.047	1
BC005624	0.03413003	0.34650425	0.207	0.239	1
Yeats4	0.0342037	0.52649001	0.207	0.217	1
Nudt13	0.03432345	0.95111173	0.025	0.022	1
Katnbl1	0.03437189	0.43992857	0.07	0.079	1
Mipep	0.03465414	0.45565784	0.014	0.017	1
Zc3h4	0.03504691	0.3454159	0.053	0.06	1
Mx1	0.03515752	0.70687261	0.016	0.019	1
Xpo7	0.03539284	0.56739285	0.069	0.064	1
Mtrr	0.03539469	0.67685719	0.029	0.025	1
Gtf2h1	0.03548541	0.40282385	0.083	0.094	1
Tecpr1	0.03557181	0.34197655	0.221	0.225	1
Asnsd1	0.03557927	0.78409786	0.078	0.074	1
Prickle3	0.0359239	0.65271943	0.021	0.018	1
Scly	0.03595307	0.35131463	0.023	0.027	1

Ube2w	0.03635911	0.78165873	0.066	0.062	1
Msl1	0.03686329	0.35990249	0.082	0.092	1
Zswim8	0.0373432	0.46664278	0.099	0.111	1
Kdm4c	0.03735329	0.31212557	0.043	0.049	1
Ahdc1	0.03769843	0.75255836	0.017	0.014	1
Men1	0.03781322	0.83627835	0.015	0.013	1
Ppp6r2	0.03815017	0.61705081	0.032	0.029	1
Ing5	0.03816106	0.6364282	0.122	0.121	1
Ercc4	0.03819361	0.33015251	0.025	0.029	1
Syvn1	0.03861131	0.48489303	0.08	0.09	1
Ncoa5	0.0386799	0.41442745	0.03	0.034	1
Rprd2	0.03881102	0.54135424	0.12	0.118	1
Bag4	0.03884326	0.25080534	0.044	0.05	1
Coq7	0.03915199	0.44494298	0.022	0.026	1
Cetn3	0.03991094	0.49997599	0.158	0.181	1
Pcf11	0.04003447	0.53687517	0.137	0.137	1
Immt	0.04005438	0.46925588	0.127	0.143	1
Rpap1	0.04011068	0.58507492	0.023	0.02	1
Aldh4a1	0.04019928	0.31649719	0.011	0.013	1
Prr14l	0.0402423	0.6570707	0.05	0.046	1
Rbm4	0.04030177	0.29827414	0.016	0.019	1
Eif5a2	0.04032529	0.29800296	0.011	0.013	1
Cdk5rap1	0.04037603	0.25682315	0.009	0.012	1
Grhpr	0.04040089	0.29026966	0.018	0.022	1
Metap1d	0.04047571	0.74636644	0.03	0.027	1
Il16	0.04050936	0.25737356	0.11	0.105	1
Zfp319	0.04071839	0.31099228	0.018	0.021	1
Mum1	0.04081994	0.60282899	0.045	0.042	1
Phlpp1	0.04118658	0.68236729	0.123	0.123	1
Txndc5	0.04186498	0.61799757	0.09	0.087	1
Gtpbp2	0.04225463	0.40394391	0.123	0.138	1
Capn7	0.04243631	0.35810364	0.075	0.084	1
Rtel1	0.0425351	0.6591076	0.013	0.011	1
Hbp1	0.04296147	0.48111327	0.23	0.244	1
Sap130	0.04342864	0.33790652	0.016	0.019	1
Edc4	0.04363503	0.30464357	0.061	0.069	1
Nek1	0.04366241	0.36221683	0.012	0.015	1
Acad9	0.04397089	0.79184855	0.021	0.018	1
Cdk7	0.04420175	0.43317014	0.038	0.044	1
Marf1	0.04432227	0.49676053	0.121	0.119	1
Dus3l	0.0443748	0.90274546	0.028	0.025	1
Gse1	0.04461947	0.63324586	0.063	0.059	1
Zmym2	0.04476514	0.39698385	0.073	0.082	1
Yipf6	0.04542286	0.43503941	0.059	0.066	1
Map1s	0.04556925	0.43268551	0.048	0.054	1
Rint1	0.0456391	0.29755383	0.027	0.031	1
Rhot1	0.0456666	0.44552972	0.05	0.056	1
Nt5dc3	0.0457422	0.74639045	0.041	0.038	1

Pdcd1lg2	0.04586166	0.47684623	0.126	0.124	1
Alg12	0.0463101	0.27081768	0.01	0.013	1
Sgk3	0.04631695	0.7301442	0.059	0.067	1
Pole4	0.04646941	0.49021214	0.239	0.256	1
Rgs19	0.04681899	0.49934303	0.061	0.069	1
Dhx34	0.04733716	0.9193388	0.023	0.021	1
Atrn	0.04734329	0.33017683	0.03	0.034	1
Zfp24	0.04744133	0.3970665	0.056	0.063	1
Hnrnpul1	0.04752956	0.33195786	0.258	0.301	1
Ceacam1	0.04808438	0.43476931	0.016	0.02	1
Ddi2	0.04826682	0.36887614	0.069	0.077	1
Foxj3	0.048445	0.31252585	0.088	0.098	1
Nup210	0.04855673	0.30948515	0.094	0.104	1
Parg	0.04863936	0.38494468	0.085	0.094	1
Zfp810	0.048901	0.88655489	0.013	0.011	1
Fbxo46	0.0492687	0.29194425	0.015	0.018	1
Cluap1	0.04929676	0.37822038	0.018	0.022	1
C330011M18F	0.04943838	0.82968988	0.011	0.009	1
C030006K11R	0.04950258	0.76905669	0.01	0.008	1
Ccdc117	0.04951356	0.45176079	0.063	0.059	1
Fig4	0.04957598	0.91833806	0.015	0.013	1
Sart3	0.04964439	0.41799832	0.066	0.074	1
Gmeb1	0.05019673	0.51824013	0.041	0.047	1
Tpp2	0.050271	0.44837685	0.259	0.279	1
Cluh	0.05051546	0.77710239	0.036	0.032	1
Mrpl50	0.05082237	0.46715378	0.057	0.064	1
Usp42	0.05108645	0.34069832	0.039	0.044	1
Yeats2	0.05129607	0.27118574	0.037	0.042	1
Usp39	0.05150994	0.44950523	0.055	0.062	1
Tmem87a	0.05175924	0.3889823	0.077	0.086	1
Carmil1	0.05175966	1.29896003	0.018	0.016	1
Nup88	0.05197621	0.44051978	0.08	0.089	1
Rprd1b	0.05230971	0.34150265	0.083	0.092	1
Tysnd1	0.05253722	0.3669825	0.014	0.017	1
Ddx20	0.05273573	0.67970859	0.032	0.028	1
Camsap1	0.05275052	0.57590684	0.031	0.027	1
Gstt2	0.05276648	0.68786107	0.044	0.04	1
Prim1	0.05279455	0.3596777	0.016	0.019	1
Ncoa1	0.05282093	0.59928914	0.081	0.077	1
Rnf213	0.05341644	0.35652911	0.116	0.129	1
Cyb5b	0.05420464	0.50031889	0.103	0.116	1
Dcun1d1	0.05424691	0.61294288	0.115	0.114	1
Fbxo9	0.05673463	0.32007289	0.052	0.058	1
Txlng	0.05693466	0.5089706	0.051	0.048	1
Gpatch2l	0.05697055	0.40005644	0.041	0.046	1
Ap2b1	0.05736215	0.57114566	0.196	0.208	1
Prpf3	0.0574626	0.70126281	0.049	0.045	1
Armcx2	0.05784418	0.7826111	0.012	0.01	1

Kctd13	0.05823692	0.31487686	0.019	0.022	1
Mettl5	0.05830701	0.3821016	0.026	0.03	1
Hmgxb4	0.05834649	0.48732022	0.042	0.047	1
Trub1	0.05835419	0.78393173	0.013	0.011	1
Dgcr8	0.05882428	0.63357499	0.024	0.021	1
Mtif3	0.05899745	0.36636433	0.023	0.027	1
Cacna1d	0.05926834	0.85086667	0.02	0.017	1
Drg2	0.05963608	0.44997873	0.026	0.03	1
Mau2	0.05967254	0.32116394	0.132	0.147	1
Ccdc69	0.05970986	0.88306432	0.018	0.015	1
Hectd3	0.06011144	0.35261622	0.02	0.023	1
Trim33	0.06013584	0.61761311	0.096	0.093	1
Pfkfb4	0.06013816	0.57147826	0.025	0.029	1
Cacfd1	0.0601596	0.49952082	0.033	0.038	1
H2-M3	0.06052283	0.72686809	0.132	0.135	1
Mynn	0.06105682	0.36565306	0.07	0.078	1
Hoxb4	0.06116127	0.75347121	0.012	0.01	1
Pdss2	0.06152017	0.88004356	0.013	0.011	1
Trrap	0.06152979	0.36285364	0.069	0.077	1
Susd3	0.06254933	0.3998365	0.053	0.06	1
Tti2	0.06317792	0.72131954	0.025	0.022	1
Vps13b	0.06365781	0.64882951	0.095	0.093	1
Zranb2	0.06430272	0.58042855	0.156	0.159	1
Arhgef11	0.06448678	0.53003306	0.023	0.027	1
Prps1l3	0.06457017	0.65874195	0.024	0.021	1
Ppm1a	0.06463282	0.59080617	0.179	0.187	1
Itga4	0.06516563	0.27271451	0.344	0.372	1
Mrps10	0.06523569	0.73298289	0.052	0.049	1
Tnpo1	0.06554265	0.38659523	0.161	0.182	1
Nsun3	0.06597771	0.70875264	0.031	0.028	1
Ntmt1	0.06607797	0.51037491	0.019	0.023	1
Lsm5	0.0664766	0.25998315	0.268	0.308	1
Zbtb22	0.06700253	0.51167934	0.027	0.031	1
Ago4	0.06813525	0.94422516	0.017	0.015	1
Usp14	0.0681649	0.46488125	0.121	0.135	1
Trim30c	0.06854259	0.33727116	0.009	0.011	1
Pgp	0.0685935	0.37472397	0.05	0.055	1
Cdan1	0.06984064	0.61437631	0.022	0.019	1
Ppp4r3b	0.07000666	0.45249208	0.209	0.219	1
Lyar	0.07128899	0.48733087	0.131	0.147	1
Mfap1b	0.07189686	0.50407611	0.074	0.07	1
Cbfa2t2	0.07203183	0.31477442	0.022	0.025	1
Rbm19	0.07259225	0.49315743	0.041	0.046	1
Rabep1	0.07264579	0.60938053	0.134	0.136	1
Pomgnt1	0.07288769	0.45891533	0.012	0.015	1
Smarcd2	0.07297041	0.33887538	0.114	0.126	1
Zfp637	0.07308532	0.65815176	0.015	0.013	1
Dpp8	0.07308962	0.42268359	0.09	0.1	1

Rhno1	0.07386124	0.52403536	0.05	0.046	1
Fance	0.07412235	0.30550182	0.01	0.012	1
Nr2c2ap	0.07518273	0.72798168	0.049	0.046	1
Hps4	0.07534267	0.43202177	0.057	0.064	1
Trappc8	0.07605265	0.35164106	0.079	0.088	1
Vamp2	0.07702236	0.53886181	0.145	0.147	1
Phc3	0.0770738	0.41679131	0.141	0.141	1
Pip4k2c	0.07728357	0.32356092	0.066	0.073	1
Chd1l	0.07788319	0.30670691	0.024	0.028	1
Cdk5rap2	0.07790423	0.34150298	0.043	0.048	1
Araf	0.07815285	0.37784428	0.108	0.119	1
Pycrl	0.0782607	0.46487844	0.01	0.012	1
Rbm28	0.07984843	0.40587667	0.134	0.149	1
Spata5	0.08008441	0.70850408	0.054	0.051	1
Ap3s2	0.08012286	0.52218329	0.019	0.022	1
Zfp748	0.08084518	0.65644313	0.027	0.025	1
Csrp2	0.08152245	0.60610847	0.027	0.031	1
Fam84b	0.0827165	0.26608725	0.011	0.014	1
Dhcr7	0.08274577	0.38462571	0.015	0.017	1
Zfp280c	0.08276821	0.68574186	0.025	0.022	1
Coq2	0.08314122	0.67649509	0.051	0.048	1
Cactin	0.08322509	0.35329167	0.031	0.035	1
Lrig2	0.08363675	0.63244975	0.033	0.03	1
Zscan29	0.08440011	0.25688168	0.022	0.025	1
Scaper	0.0844113	0.36398425	0.043	0.048	1
Ampd2	0.08525092	0.33033606	0.014	0.017	1
Ptgr2	0.08586668	0.36318527	0.017	0.02	1
Suclg2	0.08620283	0.65783372	0.063	0.06	1
AI314180	0.08641014	0.44195517	0.102	0.114	1
Banf1	0.08666492	0.39910222	0.201	0.23	1
Ap4e1	0.08781905	0.70103325	0.029	0.026	1
Rdh11	0.08783818	0.26623644	0.043	0.047	1
Lcor	0.08871672	0.64144707	0.058	0.055	1
Hmgn5	0.08959157	0.401605	0.107	0.118	1
Prkci	0.08989755	0.44390091	0.016	0.019	1
Nek9	0.08998407	0.39130772	0.061	0.068	1
Trp53inp2	0.09010416	0.78017995	0.1	0.1	1
Gm20513	0.09017405	1.54515089	0.014	0.012	1
Polr2a	0.09049692	0.30331498	0.3	0.325	1
Tnks1bp1	0.0914982	0.73074155	0.013	0.011	1
Lrrcc1	0.09171182	0.31976043	0.027	0.031	1
Zfp81	0.09180458	0.63985246	0.021	0.018	1
Fen1	0.09193723	0.50428206	0.035	0.04	1
Ttll3	0.09197872	0.70503353	0.013	0.011	1
Rbm6	0.09244659	0.48147846	0.168	0.172	1
Rbl1	0.09259719	0.27447781	0.029	0.033	1
Ddx39	0.09277407	0.72315057	0.087	0.085	1
Lclat1	0.0928041	0.32028744	0.015	0.018	1

Phf23	0.09344961	0.57856337	0.071	0.079	1
Ccdc97	0.09411218	0.4789364	0.044	0.049	1
Mtg2	0.09440843	0.31886376	0.016	0.019	1
Aqr	0.09464616	0.33065985	0.137	0.152	1
Spidr	0.09482235	0.52110758	0.021	0.024	1
Cops7b	0.09560422	0.72975716	0.022	0.02	1
Mief2	0.09609802	0.86296529	0.01	0.008	1
Themis2	0.09650617	0.26884342	0.064	0.061	1
Scpep1	0.09656739	0.48940893	0.172	0.197	1
Coro7	0.09660291	0.53470281	0.071	0.079	1
Ints10	0.09670786	0.40586289	0.016	0.019	1
Eef2kmt	0.09718908	0.59909857	0.029	0.033	1
Taf1	0.09757002	0.41165832	0.146	0.163	1
Polr2m	0.09757627	0.47103425	0.209	0.219	1
Lrrc40	0.09758643	0.38187057	0.017	0.02	1
Platr25	0.09771249	0.74587426	0.012	0.01	1
Atp11c	0.09771701	0.57412259	0.052	0.057	1
Xpo4	0.09865215	0.52816632	0.094	0.092	1
Fam53b	0.09873052	0.48437683	0.032	0.029	1
Rev3l	0.09928978	0.45971683	0.088	0.086	1
Acbd5	0.10025897	0.37230162	0.152	0.17	1
Rasal3	0.10039914	0.40796396	0.072	0.069	1
Fuz	0.10054625	0.34275472	0.014	0.016	1
R3hcc1	0.10076583	0.8017517	0.019	0.016	1
Usp4	0.10201042	0.43409622	0.093	0.103	1
Zfp560	0.10314872	0.3225604	0.068	0.075	1
Setd6	0.10373039	0.31136608	0.013	0.016	1
Ncoa3	0.10405563	0.46812066	0.149	0.151	1
Exosc2	0.10471951	0.27538427	0.066	0.062	1
Vezt	0.10487443	0.72654721	0.02	0.018	1
Cdk8	0.10493575	0.46031602	0.06	0.066	1
Cnot8	0.10507214	0.70836137	0.078	0.076	1
Thumpd1	0.10597979	0.57718549	0.089	0.087	1
Selenoo	0.1060386	0.48975605	0.023	0.026	1
Hnrnpl	0.1060495	0.30737361	0.255	0.292	1
Zbtb7a	0.10606032	0.50801362	0.201	0.212	1
Casp9	0.10607173	0.85597765	0.01	0.008	1
Gm26825	0.10614918	0.7607417	0.03	0.027	1
Pan2	0.1064185	0.5715917	0.038	0.035	1
Rabepk	0.10667756	0.59097761	0.021	0.025	1
Tbck	0.10723912	0.45016834	0.045	0.05	1
Tmem243	0.10828379	0.42381447	0.197	0.206	1
Cog5	0.10883277	0.65664123	0.062	0.06	1
Mkln1	0.10952777	0.35357274	0.215	0.244	1
Cdc26	0.1102294	0.64297255	0.121	0.122	1
Pex7	0.11024689	0.48149034	0.066	0.073	1
Rhbdd2	0.1103432	0.33801363	0.017	0.02	1
Trappc9	0.11104756	0.43876819	0.039	0.043	1

Hsd17b7	0.11109258	0.4402154	0.028	0.032	1
Ttc13	0.11112743	0.76834483	0.021	0.019	1
Etfrf1	0.11119538	0.50722786	0.031	0.035	1
Anapc1	0.11124324	0.55324246	0.102	0.1	1
Akr1b10	0.11124755	0.49694603	0.03	0.033	1
Ccdc171	0.11187154	0.65593015	0.015	0.013	1
Fam193b	0.11210441	0.2620254	0.046	0.051	1
Pus1	0.11298791	0.51782492	0.05	0.056	1
Mtrf1l	0.1131625	0.32567846	0.015	0.017	1
Lmbr1l	0.11351411	0.45482355	0.064	0.06	1
Tsfm	0.11390644	0.74097186	0.029	0.026	1
Usp31	0.11406231	0.56762332	0.034	0.031	1
Zc3h3	0.1147936	0.3439037	0.023	0.026	1
Aacs	0.11520495	0.79123618	0.017	0.015	1
Zfp942	0.11549918	0.32852485	0.047	0.051	1
Gtpbp8	0.11551845	0.3351932	0.017	0.019	1
Cstf1	0.11571837	0.85417313	0.036	0.033	1
Phactr4	0.11605695	0.82507433	0.049	0.046	1
AW554918	0.11675438	0.67842247	0.026	0.023	1
Hif1an	0.11741978	0.29345905	0.09	0.099	1
Tmem201	0.11771171	0.41812443	0.014	0.012	1
Khnyn	0.1178421	0.26510861	0.068	0.074	1
Mon1a	0.11816336	0.38391907	0.013	0.015	1
Maml3	0.11831977	1.10412285	0.046	0.044	1
Alg8	0.11883918	0.67036655	0.009	0.011	1
Amn1	0.11889091	0.49269896	0.108	0.107	1
Cutc	0.11903244	0.49038587	0.017	0.02	1
Gm11423	0.11940961	0.76788673	0.01	0.009	1
Lmtk2	0.12063915	0.58046785	0.053	0.05	1
Tmem129	0.12161877	1.07748398	0.01	0.009	1
Ubiad1	0.12162476	0.33604696	0.008	0.01	1
Zfp101	0.12174016	0.68631883	0.021	0.018	1
Pou5f2	0.12190448	0.66063942	0.012	0.01	1
Dclre1b	0.1229126	0.36114137	0.017	0.02	1
Tmem9	0.12309482	0.34704012	0.013	0.015	1
Ltbp4	0.12317235	0.32939337	0.021	0.024	1
Chmp7	0.12420233	0.74694118	0.032	0.029	1
Letmd1	0.12451582	0.40145229	0.026	0.029	1
Sugp2	0.1252306	0.60493064	0.042	0.04	1
Prep	0.12536092	0.58043093	0.033	0.037	1
Tmem229b	0.12565676	0.40526419	0.016	0.019	1
Snw1	0.12606059	0.37979644	0.295	0.324	1
Rpf1	0.12656789	0.58535755	0.102	0.101	1
Mcm3ap	0.12703878	0.53022618	0.043	0.04	1
Pnpla7	0.1271079	0.36452932	0.055	0.061	1
Nek4	0.12739596	0.56494989	0.008	0.01	1
Clock	0.12770251	0.54959008	0.093	0.092	1
Llgl1	0.12877319	0.27053862	0.02	0.023	1

Cog8	0.12878367	0.48917163	0.045	0.042	1
Zgrf1	0.12897926	0.59483123	0.014	0.012	1
Acvr1b	0.12919105	0.45100697	0.012	0.014	1
Gga3	0.12929287	0.54152701	0.027	0.03	1
Tmem161b	0.12949855	0.7989428	0.04	0.037	1
Pdpr	0.12950397	0.59682334	0.037	0.035	1
Klhl15	0.1298651	0.46974386	0.027	0.031	1
Ddx58	0.13069737	0.56328599	0.07	0.068	1
Hspa1a	0.13100125	1.27130423	0.132	0.133	1
Gm42722	0.13115577	0.59361624	0.012	0.01	1
Zfp445	0.13139537	0.32363874	0.135	0.149	1
Synrg	0.13245362	0.57256023	0.105	0.104	1
Grasp	0.13271119	0.63886736	0.1	0.099	1
Ercc5	0.13295426	0.41307097	0.04	0.044	1
Enpp1	0.13494901	0.49321306	0.032	0.03	1
Gm16973	0.13502186	0.58645491	0.015	0.013	1
Nup188	0.13509754	0.43427868	0.028	0.031	1
Nemp2	0.13639452	0.43114372	0.044	0.041	1
Rnf123	0.13693708	0.56149366	0.042	0.04	1
Kbtbd4	0.13720153	0.33084932	0.016	0.019	1
Tcerg1	0.13728654	0.4389049	0.151	0.169	1
Zdhhc20	0.1378258	0.32039477	0.172	0.191	1
Nedd1	0.13784702	0.48635849	0.022	0.025	1
Pias1	0.13809564	0.49826536	0.133	0.135	1
Rad23b	0.13811669	0.43196799	0.201	0.229	1
Pced1a	0.13859621	0.33533932	0.015	0.017	1
2210016L21Ri	0.13879508	0.4355759	0.062	0.068	1
4933434E20R	0.13895757	0.55476309	0.147	0.151	1
Terf1	0.14094219	0.51796798	0.08	0.077	1
Hibadh	0.14144626	0.51691483	0.082	0.091	1
Rab26os	0.14263679	0.28682152	0.03	0.033	1
Hepacam2	0.1426702	1.20994486	0.023	0.021	1
Cep162	0.14272395	0.6318025	0.028	0.025	1
Ppp2r2a	0.14297745	0.36198229	0.215	0.244	1
Snx11	0.14368096	0.40516046	0.027	0.03	1
Ifit3	0.14384643	0.46106161	0.018	0.016	1
Slc50a1	0.14394997	0.52810804	0.129	0.13	1
Rars2	0.14425433	0.83579542	0.011	0.009	1
Cnot3	0.14527059	0.52862847	0.167	0.173	1
Nprl3	0.14541598	0.66879807	0.02	0.017	1
Slc14a1	0.14614054	1.02304648	0.015	0.013	1
Trak2	0.14643303	0.37416117	0.07	0.076	1
Eif2ak4	0.1466495	0.89012704	0.022	0.02	1
Ppid	0.14678645	0.46982952	0.063	0.069	1
Acsf2	0.14811807	1.0942647	0.037	0.034	1
Zfp426	0.14856798	0.33918656	0.016	0.019	1
Sgsh	0.14885357	0.59861742	0.013	0.016	1
Tbc1d10c	0.15028458	0.33636369	0.071	0.068	1

Phospho2	0.15029472	0.37939704	0.045	0.049	1
Alg11	0.15093144	0.43246264	0.021	0.023	1
1810009A15R	0.1514849	0.83371478	0.01	0.009	1
Morc3	0.15183825	0.53016378	0.098	0.108	1
Noa1	0.15275217	0.35860369	0.019	0.021	1
Rapgef1	0.15385627	0.39759773	0.098	0.107	1
B4galt7	0.15615799	0.36440292	0.017	0.019	1
Usp53	0.15629416	0.65971733	0.041	0.038	1
Hdac6	0.15650221	0.51877824	0.012	0.014	1
Ift46	0.1568553	0.38110055	0.022	0.025	1
Zbtb11	0.15729046	0.35644647	0.113	0.123	1
Ube4a	0.15794052	0.80396873	0.048	0.046	1
Dtd2	0.15819311	0.90920913	0.012	0.01	1
Cep57l1	0.15845072	0.66526602	0.014	0.012	1
Fam122b	0.15859227	0.69997097	0.012	0.011	1
Arhgap12	0.15861338	0.54591346	0.039	0.037	1
Fam3c	0.15899217	0.50858499	0.082	0.08	1
Gm11808	0.16017029	0.45137794	0.247	0.27	1
Asb7	0.16043641	0.69341912	0.017	0.015	1
Mier3	0.16085722	0.48120674	0.039	0.043	1
Wdr44	0.16116128	0.41073214	0.064	0.061	1
Mapk1ip1	0.16136606	0.56426081	0.023	0.021	1
Hinfp	0.16221097	0.6004604	0.047	0.044	1
Tigar	0.16395281	0.43114394	0.016	0.018	1
4930532G15R	0.16398197	0.6910795	0.011	0.009	1
Tsga10	0.16441492	0.72657302	0.018	0.02	1
Ltn1	0.16461777	0.61056138	0.07	0.068	1
Arhgap39	0.16541388	0.37986801	0.02	0.023	1
Pde6d	0.16570877	0.48370678	0.014	0.016	1
Mplkip	0.1665498	0.63219544	0.111	0.112	1
Naa16	0.16679471	0.53722805	0.047	0.044	1
Gtpbp3	0.16727539	0.39016584	0.01	0.012	1
Fyttd1	0.16797585	0.41988664	0.122	0.134	1
Iffo1	0.1694851	0.25576745	0.022	0.024	1
Tmco4	0.1713142	0.30553752	0.014	0.016	1
Inpp5a	0.17183835	0.74539156	0.031	0.029	1
Wdr5	0.17210589	0.69621668	0.053	0.051	1
Abcb7	0.1726607	0.51217444	0.046	0.05	1
Tceanc2	0.17394563	0.82135122	0.034	0.032	1
Ttf1	0.1742573	0.5482205	0.071	0.069	1
Zfp143	0.17576494	0.61248664	0.027	0.03	1
Pcnx4	0.17604548	0.33819637	0.01	0.011	1
Socs4	0.17777058	0.5512377	0.117	0.118	1
Abca3	0.17779923	0.53603737	0.043	0.048	1
Dimt1	0.17840607	0.78321276	0.025	0.023	1
Ipp	0.17903559	0.66965156	0.012	0.01	1
Cep135	0.1791035	0.5646078	0.073	0.071	1
Itch	0.17930434	0.37571896	0.194	0.218	1

Dhx29	0.17953576	0.44149541	0.031	0.034	1
Srbd1	0.17993212	0.32746679	0.023	0.026	1
Ckap2l	0.18155431	0.66719598	0.013	0.012	1
Gvin1	0.18172896	0.40253618	0.009	0.01	1
Mcm5	0.18229825	0.42003366	0.036	0.04	1
Donson	0.18244226	0.36389483	0.077	0.084	1
Ndc80	0.18280496	0.28451058	0.012	0.014	1
Asb3	0.18432869	0.32502591	0.026	0.028	1
Cep120	0.18471947	0.51296168	0.112	0.112	1
Kbtbd3	0.18552352	0.4573952	0.01	0.012	1
Pigq	0.18656817	0.51147866	0.032	0.035	1
Hira	0.18722828	0.32663336	0.029	0.032	1
Zbtb14	0.18779581	0.6241864	0.011	0.01	1
Zfp410	0.18794537	0.3695944	0.057	0.062	1
Rps12-ps3	0.1885661	0.66365504	0.076	0.074	1
Wbp11	0.18949634	0.39377167	0.29	0.32	1
Pskh1	0.18960276	0.3710512	0.018	0.02	1
Map3k8	0.18962154	0.44664304	0.131	0.144	1
Slc37a1	0.19075161	0.28979268	0.011	0.012	1
Aen	0.19121085	0.73052761	0.032	0.03	1
Dlg1	0.19147806	0.59000027	0.093	0.093	1
Fbxl19	0.19202272	0.7907827	0.01	0.009	1
Zfp777	0.19350576	0.60877809	0.027	0.025	1
Gm17477	0.19378096	0.57838969	0.012	0.011	1
Hist2h2ac	0.19443077	0.3170688	0.066	0.072	1
Pnpt1	0.19542231	0.79189765	0.034	0.032	1
Snrnp35	0.19564083	0.82278646	0.028	0.031	1
Cdkn2aipnl	0.19651605	0.41604118	0.053	0.05	1
1810024B03R	0.19753999	0.92555407	0.012	0.011	1
Fam193a	0.19841893	0.44182665	0.106	0.116	1
Mterf1a	0.19865414	0.44800358	0.039	0.036	1
Mfsd8	0.19909787	0.54756838	0.022	0.021	1
Gm20234	0.19942235	0.44472643	0.011	0.01	1
Mettl14	0.19983863	0.37257713	0.046	0.05	1
Gm340	0.19989692	0.74692468	0.019	0.017	1
Fxr2	0.20093972	0.49543532	0.074	0.08	1
Gtf2h3	0.20096271	0.77244891	0.011	0.009	1
Mthfd1	0.20353049	0.67018286	0.025	0.023	1
Cdpf1	0.20499332	0.45008339	0.014	0.016	1
Ggnbp2	0.20507117	0.37847262	0.191	0.214	1
Ruvbl1	0.20530408	0.73621358	0.074	0.073	1
Bora	0.20716206	0.42537033	0.023	0.021	1
Zfp84	0.20723404	0.39106767	0.047	0.051	1
Trappc5	0.20742017	0.55661113	0.098	0.107	1
Ip6k2	0.20759203	0.48276009	0.042	0.039	1
Gtpbp6	0.20899583	0.51201571	0.034	0.037	1
Igf2bp3	0.20905858	0.35442929	0.014	0.016	1
Spin1	0.21069881	0.32969277	0.144	0.158	1

Rmnd1	0.21167804	0.90976943	0.015	0.013	1
Pcmtd2	0.21272437	0.26111042	0.093	0.1	1
Eml5	0.21403799	0.39699118	0.028	0.026	1
Kynu	0.21600581	1.25119344	0.018	0.02	1
Mief1	0.21631798	0.51540035	0.039	0.042	1
Dbp	0.21813312	0.28827946	0.013	0.015	1
Zbtb18	0.21826822	0.72844564	0.095	0.096	1
Dhx37	0.21871679	0.85713923	0.019	0.018	1
Sdhaf3	0.21923528	0.77046235	0.016	0.014	1
Elp4	0.22026904	0.28876784	0.01	0.012	1
Nelfcd	0.22294449	0.37088367	0.024	0.027	1
Ikbkg	0.22378536	0.60710032	0.033	0.031	1
Ints5.1	0.22422248	0.32410771	0.018	0.02	1
Mterf4	0.22490191	0.49189705	0.016	0.018	1
Limk2	0.22545957	0.57949012	0.025	0.023	1
Coil	0.22632583	0.73062999	0.02	0.018	1
Usp15	0.22651787	0.33152412	0.215	0.241	1
Zfp142	0.2271575	0.34297792	0.04	0.044	1
Kpna1	0.22845292	0.34880776	0.281	0.307	1
Tgds	0.22927627	0.38514466	0.011	0.013	1
Irgq	0.22951957	0.63794014	0.021	0.019	1
Taok2	0.23126363	0.48096646	0.062	0.061	1
Brpf3	0.23143105	0.28950441	0.02	0.022	1
Ppp1r12b	0.23296427	0.4022374	0.014	0.016	1
U2surp	0.23344316	0.44747566	0.252	0.275	1
Saal1	0.23378469	0.49395596	0.02	0.022	1
Zfp746	0.23398902	0.35630691	0.078	0.084	1
Usp30	0.23414803	0.76541822	0.014	0.013	1
Ppip5k1	0.23613838	0.31791764	0.013	0.014	1
Cd3eap	0.23639633	0.40791019	0.021	0.023	1
Mocs3	0.23759398	0.41950325	0.009	0.011	1
Slc25a32	0.23814564	0.44136058	0.025	0.027	1
Lrrc14	0.23824019	0.45681573	0.016	0.018	1
Supt16	0.23835951	0.39073809	0.196	0.219	1
Taf3	0.23877124	0.36820578	0.103	0.111	1
Asf1a	0.23929715	0.30645301	0.061	0.065	1
Ybey	0.239588	0.42183234	0.009	0.011	1
Narfl	0.24230456	0.62921523	0.023	0.025	1
Elp2	0.24361579	0.4418944	0.032	0.034	1
Tor3a	0.24407116	0.94513866	0.049	0.048	1
Tyw1	0.24412361	0.68057646	0.021	0.019	1
Zfp276	0.24421847	0.38739904	0.012	0.014	1
Tk1	0.2453463	0.48243661	0.011	0.013	1
Tspan14	0.24600961	0.42226711	0.092	0.1	1
Xpot	0.25034719	0.52011498	0.053	0.058	1
Dgke	0.25093466	0.64366123	0.04	0.038	1
Mcoln2	0.25158747	0.41832825	0.012	0.013	1
Bud13	0.25330091	0.47703588	0.037	0.04	1

Cep164	0.25417911	0.44751825	0.047	0.051	1
Cenpe	0.25453819	0.27927324	0.012	0.014	1
BC003965	0.2553547	0.63461653	0.058	0.056	1
Dedd2	0.2557457	0.44062079	0.066	0.065	1
Celf1	0.25587133	0.29657115	0.251	0.283	1
Wdr11	0.25587505	0.61083082	0.025	0.028	1
Senp2	0.25612485	0.39690083	0.196	0.22	1
Ppcs	0.25793995	0.50632647	0.016	0.014	1
Zmym1	0.25866278	0.33356617	0.033	0.036	1
Arap1	0.2606822	0.50671091	0.114	0.126	1
Nup93	0.26212264	0.4972878	0.023	0.025	1
Orc2	0.2627473	0.57373496	0.051	0.049	1
Ipo8	0.26343457	0.53435574	0.063	0.062	1
Ppif	0.26392745	0.45310515	0.028	0.031	1
Mcm3	0.26697873	0.45043819	0.066	0.071	1
Hmgn3	0.26723484	0.55027772	0.032	0.03	1
Insr	0.26785755	0.29075078	0.038	0.041	1
Sin3a	0.26828765	0.44481701	0.081	0.087	1
Hist1h3f	0.2695227	0.51076694	0.023	0.021	1
Tipin	0.26953651	0.36046503	0.028	0.03	1
Rxrb	0.27036806	0.52090307	0.058	0.063	1
Ttc33	0.27101901	0.38684488	0.053	0.057	1
Inpp5e	0.27110118	0.35111984	0.014	0.015	1
Gm6377	0.2725868	0.62507801	0.116	0.129	1
Zfp953	0.27305704	0.72738665	0.012	0.011	1
Pcid2	0.27786613	0.45970744	0.039	0.042	1
Carm1	0.27787272	0.70767555	0.029	0.028	1
Bcl2l12	0.27898033	0.58943668	0.017	0.018	1
Coasy	0.28201014	0.51234895	0.017	0.018	1
Zpr1	0.282182	0.47608158	0.069	0.075	1
Coa7	0.28224984	0.73763761	0.038	0.042	1
Ssrp1	0.28283289	0.49036534	0.179	0.188	1
Flcn	0.28342494	0.63640729	0.082	0.082	1
Trub2	0.28350107	0.51178379	0.024	0.022	1
Mrpl16	0.28363203	0.49831639	0.064	0.069	1
Pnpla6	0.28387262	0.59901373	0.019	0.017	1
1110008L16Ri	0.28471905	0.78939837	0.011	0.01	1
Sirt1	0.28722268	0.33967579	0.084	0.09	1
Gtf2h2	0.28846069	0.42492154	0.02	0.022	1
Mfap1a	0.28854152	0.41268035	0.086	0.085	1
Sft2d1	0.28896215	0.56146326	0.145	0.161	1
Poli	0.28991733	0.57792703	0.012	0.011	1
Smcr8	0.29181439	0.55521835	0.055	0.059	1
Trim35	0.29473589	0.59055797	0.117	0.129	1
Gm19951	0.29683204	0.67996867	0.008	0.01	1
Tpk1	0.29687118	0.94286346	0.012	0.011	1
Zfp653	0.29873013	0.48415025	0.015	0.016	1
Setdb1	0.29896489	0.52760235	0.038	0.041	1

Sf3a2	0.29943205	0.63217846	0.064	0.063	1
AC165953.2	0.30008654	0.33662236	0.009	0.01	1
Spg11	0.3004533	0.63507738	0.018	0.02	1
Dennd4b	0.30128831	0.77835709	0.072	0.072	1
Ppp5c	0.30158584	0.74104362	0.044	0.042	1
AU022252	0.30209468	0.79680459	0.02	0.019	1
Ddx27	0.30233922	0.60006051	0.107	0.108	1
Tmub1	0.30258599	0.42352914	0.033	0.036	1
Hist2h4	0.30270222	0.59591357	0.017	0.015	1
Rab4b	0.30287668	0.89170814	0.013	0.012	1
Mtbp	0.30322408	0.29565789	0.014	0.015	1
Ubr3	0.30352326	0.67473907	0.089	0.09	1
Apex2	0.3038616	0.39385542	0.014	0.016	1
Tnpo3	0.30697532	0.49581539	0.162	0.17	1
Ubqln4	0.30732494	0.65323964	0.031	0.03	1
Ogfod1	0.30823009	0.40999648	0.016	0.018	1
Vps45	0.3087477	0.41968061	0.018	0.019	1
Wdr47	0.31008631	0.33187386	0.02	0.021	1
Orai3	0.31180187	0.56178672	0.02	0.022	1
Fam206a	0.31197697	0.48120674	0.018	0.02	1
Gm26782	0.31214148	0.47400237	0.015	0.017	1
Zfp626	0.31270812	0.69816951	0.027	0.025	1
Dcaf11	0.3133902	0.48257751	0.087	0.094	1
Tprkb	0.3135981	0.42423465	0.033	0.035	1
Aste1	0.31478721	0.30748038	0.014	0.016	1
Taf8	0.31531597	0.69932864	0.025	0.023	1
Prr14	0.31547397	0.45504643	0.092	0.099	1
Gtf3c4	0.31683516	0.42391622	0.018	0.02	1
Adsl	0.318235	0.61957266	0.041	0.039	1
Rictor	0.31984693	0.30375078	0.289	0.33	1
Cad	0.32186389	0.41651856	0.021	0.023	1
Rnf111	0.32210357	0.51739578	0.183	0.195	1
E130208F15Ri	0.32296519	0.63859239	0.014	0.013	1
Zscan26	0.32459403	0.59853969	0.044	0.043	1
Ercc2	0.32608969	0.29283788	0.02	0.022	1
Gm42670	0.32790303	0.5917776	0.016	0.018	1
Zfp51	0.32817965	0.35054877	0.025	0.027	1
Btrc	0.32829708	0.39885849	0.025	0.027	1
Mettl22	0.32924338	0.52769316	0.009	0.01	1
Vps11	0.33085504	0.55160483	0.061	0.066	1
Acot2	0.333732	0.38430848	0.038	0.036	1
Chfr	0.33502934	0.40051827	0.132	0.143	1
Snapc3	0.33604332	0.59080583	0.043	0.042	1
Bag5	0.338764	0.47561021	0.048	0.052	1
Uqcc1	0.33890979	0.68413492	0.028	0.026	1
Akap11	0.34302753	0.5182673	0.068	0.073	1
Zkscan5	0.343275	0.78522866	0.01	0.009	1
D2hgdh	0.34482077	0.62348255	0.011	0.01	1

Topbp1	0.34485516	0.53545336	0.094	0.094	1
Brwd3	0.3449097	0.61929788	0.047	0.046	1
Cdk13	0.34646111	0.50066918	0.144	0.149	1
Trnau1ap	0.34654348	0.66138439	0.084	0.085	1
Usp21	0.3493587	0.45212451	0.019	0.02	1
Ppat	0.35116617	0.53565231	0.047	0.045	1
Ap3m2	0.35568463	0.36321587	0.022	0.024	1
Ecsit	0.35915939	0.53725642	0.036	0.034	1
Adprhl2	0.35960432	0.65369279	0.035	0.034	1
Trim27	0.35981981	0.48957631	0.102	0.111	1
Chkb	0.35988017	0.67341272	0.063	0.063	1
Krt10	0.36030742	0.63175915	0.011	0.01	1
Mbd5	0.36191495	0.34782363	0.03	0.032	1
Pik3cg	0.36256988	0.79618088	0.07	0.071	1
Mbip	0.36268108	0.51601343	0.04	0.039	1
Fasn	0.3635088	0.41685705	0.05	0.054	1
Cwc27	0.3671279	0.55842584	0.041	0.044	1
Gm43848	0.36769422	0.39374435	0.02	0.022	1
Gm20707	0.37254606	0.58115275	0.015	0.016	1
Mllt1	0.37431511	0.64619688	0.018	0.017	1
Tacstd2	0.37549923	0.75782918	0.01	0.009	1
Arel1	0.37605849	0.32679024	0.044	0.046	1
2310035C23R	0.37707169	0.38682465	0.125	0.134	1
Zfp120	0.38145571	0.43094956	0.043	0.046	1
Uck1	0.38173865	0.53426529	0.026	0.028	1
Bckdha	0.38182255	0.53379084	0.054	0.058	1
Ss18l2	0.38336533	0.33965867	0.055	0.058	1
Ankrd52	0.38522664	0.57150006	0.033	0.031	1
Trim8	0.38577794	0.56346961	0.065	0.07	1
Rbm41	0.38790858	0.54868574	0.021	0.023	1
Gtf3c3	0.3880818	0.41853045	0.036	0.039	1
Vps33b	0.39282627	0.39489766	0.016	0.018	1
Micu3	0.39407383	0.60012278	0.027	0.025	1
Tada2b	0.39440893	0.65419597	0.026	0.025	1
9430060I03Ri	0.39509777	0.44550865	0.014	0.013	1
2810006K23R	0.39594955	0.88585652	0.01	0.009	1
Utp4	0.39659598	0.4491672	0.033	0.035	1
Zbtb48	0.39668023	0.28666441	0.01	0.011	1
Mob4	0.39756408	0.48942046	0.195	0.209	1
Gm26699	0.40246945	0.61808932	0.047	0.046	1
Ankzf1	0.40357823	0.53907946	0.016	0.018	1
Gm49359	0.40529484	0.46399027	0.018	0.019	1
Twistnb	0.40674063	0.47131411	0.194	0.207	1
2810402E24R	0.4067701	0.59280944	0.059	0.058	1
Uhmk1	0.40747181	0.49005722	0.141	0.146	1
Tchp	0.40782763	0.36785987	0.015	0.016	1
Guf1	0.40973793	0.57976328	0.03	0.029	1
Xrn2	0.41002505	0.33406733	0.278	0.317	1

Tmem39b	0.41062073	0.59499387	0.017	0.016	1
Otud6b	0.41260907	0.50505774	0.042	0.045	1
Dffb	0.41508985	0.39561629	0.018	0.017	1
Bmt2	0.41568671	0.52811178	0.087	0.093	1
Zbtb5	0.4165271	0.53156918	0.029	0.027	1
Ap4m1	0.41676777	0.56545237	0.035	0.037	1
Timm29	0.41678073	0.32200921	0.023	0.025	1
Cop1	0.41678947	0.5403746	0.134	0.147	1
Arglu1	0.41810002	0.36903946	0.283	0.312	1
Mrrf	0.42094908	0.9181163	0.025	0.024	1
Zw10	0.42204868	0.59459253	0.029	0.028	1
Zfp322a	0.42273201	0.61350374	0.069	0.069	1
Eif4enif1	0.42628834	0.54655316	0.064	0.063	1
Tmem106b	0.42840146	0.53281551	0.077	0.077	1
4732440D04R	0.42842633	0.36823452	0.04	0.042	1
Sars2	0.42988071	0.54486917	0.013	0.014	1
Tcp1	0.43066597	0.4667213	0.275	0.31	1
Syncrip	0.43154493	0.30602333	0.305	0.35	1
Ube3a	0.43169937	0.41595483	0.195	0.206	1
Rnf44	0.43245689	0.49527326	0.11	0.112	1
Calcoco1	0.43267967	0.32308769	0.029	0.031	1
Xrcc1	0.43335164	0.55989637	0.026	0.025	1
Csnk1g1	0.43461068	0.45076011	0.078	0.083	1
Pum3	0.43696904	0.59251149	0.081	0.087	1
Casd1	0.4387911	0.52238582	0.034	0.033	1
Eif2ak1	0.43904528	0.44654595	0.08	0.086	1
Dnajb5	0.43930892	0.83689338	0.015	0.016	1
Rnf25	0.43989017	0.43600601	0.026	0.028	1
Shmt2	0.44064242	0.43680151	0.04	0.042	1
S100pbp	0.44243829	0.42710647	0.051	0.054	1
Phf20l1	0.44556367	0.27993644	0.291	0.317	1
Ghitm	0.44970531	0.29473995	0.373	0.44	1
Faap24	0.45129851	0.39507337	0.017	0.019	1
Pigu	0.45406266	0.87254741	0.031	0.03	1
Flywch1	0.45621245	0.80125258	0.017	0.016	1
Pex12	0.45625319	0.70875706	0.011	0.01	1
D430042O09R	0.45828304	0.553537	0.062	0.066	1
Wdr59	0.46270586	0.59148117	0.012	0.013	1
Pycr2	0.46360106	0.30179606	0.044	0.047	1
Ttc28	0.46378555	0.25923142	0.034	0.036	1
B3gnt8	0.46391062	0.9706799	0.019	0.021	1
Hist1h3i	0.46397657	0.2797378	0.018	0.019	1
Qrich1	0.46601013	0.56197749	0.121	0.125	1
1810032O08R	0.46702657	0.60169702	0.058	0.058	1
Nuak2	0.46926869	0.93671073	0.081	0.089	1
Nr2c2	0.46930799	0.45890083	0.158	0.166	1
Ctps2	0.47047687	0.52631092	0.042	0.044	1
Ghdc	0.47305714	0.93206694	0.013	0.014	1

Taf2	0.47314632	0.4780398	0.045	0.048	1
Esco1	0.47555687	0.43624635	0.13	0.134	1
Zbtb2	0.47588572	0.57205708	0.037	0.036	1
Znhit3	0.47729845	0.62176801	0.034	0.033	1
Gnl2	0.47790073	0.60140929	0.083	0.089	1
Slc43a3	0.48006769	0.47356447	0.014	0.013	1
Bysl	0.48035257	0.58170453	0.025	0.024	1
Gon4l	0.48064411	0.42742648	0.066	0.07	1
Alg14	0.48345396	0.64183416	0.009	0.01	1
Gm38394	0.48601998	0.47358938	0.013	0.013	1
Dhx35	0.48684773	0.40853852	0.026	0.027	1
Nmd3	0.48965279	0.58353944	0.091	0.092	1
Acaca	0.49093229	0.73962586	0.034	0.034	1
Dnajc11	0.491533	0.57409099	0.046	0.049	1
Rcc1	0.49197873	0.62626408	0.018	0.017	1
Slc25a22	0.49605393	0.78654879	0.016	0.015	1
Eftud2	0.49871178	0.50662283	0.091	0.097	1
Ubr7	0.4992074	0.51427904	0.063	0.062	1
Dnaja3	0.49930191	0.80837372	0.018	0.017	1
Anks3	0.50093577	0.43501297	0.036	0.038	1
Atpaf2	0.50269962	0.50555615	0.027	0.028	1
Mcm9	0.50381088	0.54574814	0.04	0.039	1
Derl3	0.5057155	0.49484676	0.019	0.018	1
Dars	0.50572901	0.65756707	0.085	0.087	1
Tbp	0.50598775	0.49464384	0.034	0.036	1
Pggt1b	0.50772825	0.47184204	0.026	0.028	1
Yipf2	0.50932654	0.48733501	0.017	0.016	1
Med13	0.5096262	0.32505874	0.266	0.301	1
Spata7	0.50977825	0.5900236	0.01	0.009	1
Tagap1	0.51092184	0.47849613	0.025	0.024	1
Kansl1l	0.51441099	0.48806056	0.035	0.034	1
Ccdc115	0.51720202	0.50941474	0.052	0.055	1
Csad	0.51853496	0.78324594	0.013	0.014	1
Yme1l1	0.51870088	0.35542558	0.192	0.211	1
Kat8	0.51927638	0.71397997	0.018	0.018	1
Cipc	0.5197611	0.33817281	0.099	0.099	1
Rps19bp1	0.52035316	0.64856625	0.074	0.078	1
Cdc16	0.52061009	0.60533453	0.037	0.036	1
Larp1	0.52146932	0.39489235	0.219	0.244	1
Mis18bp1	0.52407041	0.33067503	0.009	0.01	1
Abraxas2	0.52505168	0.56880682	0.091	0.097	1
Akap9	0.52694821	0.46393256	0.18	0.191	1
Pigh	0.52708652	0.45489518	0.01	0.01	1
Mfng	0.52802398	0.49085205	0.03	0.032	1
Slc25a16	0.52916046	0.36029232	0.028	0.03	1
Polr3f	0.5298953	0.53719217	0.051	0.054	1
Coq4	0.53044174	0.72688397	0.012	0.012	1
Poldip2	0.53055976	0.63324863	0.029	0.031	1

Ruvbl2	0.53164201	0.60443081	0.025	0.026	1
Krr1	0.53263469	0.50343048	0.074	0.074	1
Gapvd1	0.53345331	0.49804866	0.146	0.159	1
Pradc1	0.53347994	0.68655778	0.014	0.013	1
Rida	0.53400324	0.6554116	0.022	0.022	1
Dnajc16	0.53551097	0.56558092	0.012	0.013	1
Edc3	0.53625555	0.45862297	0.024	0.025	1
Ppp1r21	0.53850558	0.59970129	0.041	0.043	1
Slc7a6	0.53937262	0.41235212	0.019	0.02	1
Prrc1	0.53980619	0.69491259	0.055	0.055	1
Sepsecs	0.54035037	0.78580319	0.018	0.018	1
Tbl3	0.54250959	0.79204469	0.025	0.025	1
Slc35d2	0.54308957	0.54359468	0.011	0.012	1
Skiv2l2	0.54584592	0.56255971	0.105	0.108	1
Eif2ak2	0.54596576	0.62548213	0.041	0.043	1
Evl	0.54665193	0.52485815	0.129	0.134	1
Lzic	0.54852958	0.58561742	0.043	0.045	1
Nxpe3	0.54975847	0.29975707	0.028	0.027	1
D930016D06F	0.55058473	0.67474648	0.02	0.019	1
Pqbp1	0.5515874	0.42295206	0.113	0.121	1
Mgme1	0.55642808	0.56930318	0.012	0.013	1
Atg16l2	0.55648107	0.4807788	0.051	0.051	1
Umad1	0.55651212	0.84095075	0.018	0.018	1
Knop1	0.55664969	0.50181243	0.087	0.093	1
Apeh	0.55759781	0.71425515	0.038	0.037	1
Fem1a	0.55854906	0.61083739	0.036	0.036	1
Mrps27	0.56157499	0.74119652	0.017	0.018	1
Ncbp3	0.56168497	0.43880032	0.092	0.097	1
Mtap	0.56221569	0.4346443	0.028	0.029	1
Pkd1	0.563647	0.52633424	0.049	0.048	1
Ankrd28	0.56393413	0.52568763	0.045	0.048	1
Sema7a	0.56550636	0.64100678	0.054	0.054	1
Dtwd1	0.56559157	0.54240527	0.011	0.012	1
Als2	0.56629804	0.69725234	0.023	0.022	1
Cpsf7	0.5674316	0.54808676	0.134	0.145	1
Zdhhc14	0.56778483	1.07529071	0.022	0.021	1
Tmed1	0.57007701	0.71583385	0.009	0.01	1
S1pr3	0.57057269	0.79324087	0.012	0.011	1
Eif2s3y	0.57074542	0.55487875	0.084	0.086	1
Trim56	0.57156361	0.28999356	0.08	0.084	1
Rpain	0.57193161	0.66132293	0.028	0.029	1
Pank4	0.57307511	0.4841005	0.024	0.025	1
Kctd18	0.57516365	0.50962594	0.049	0.048	1
Msh2	0.57529727	0.4107019	0.034	0.033	1
Tmcc1	0.57737475	0.46685128	0.097	0.099	1
Vwa8	0.58157249	0.41382339	0.015	0.015	1
Cdkal1	0.58527257	0.57435152	0.044	0.044	1
Sfi1	0.58704079	0.39269881	0.033	0.032	1

Gba2	0.58723872	0.43542805	0.012	0.012	1
Pgam5	0.58753554	0.60052654	0.04	0.039	1
Fam118b	0.5882288	0.64839075	0.015	0.014	1
Med16	0.58875652	0.59593705	0.044	0.046	1
Zmynd19	0.58966023	0.57094272	0.026	0.025	1
Etfbkmt	0.59326022	0.44622904	0.011	0.01	1
Ubxn7	0.59965645	0.31312444	0.083	0.087	1
Cct2	0.60085555	0.32229585	0.373	0.437	1
B3galnt2	0.60117063	0.38475056	0.029	0.03	1
Psme3	0.60172459	0.57119639	0.113	0.121	1
Recql	0.60310309	0.56153097	0.023	0.023	1
AC129328.1	0.60333751	0.53994382	0.012	0.011	1
Ears2	0.60352622	0.92636692	0.019	0.019	1
Hspbap1	0.60430873	0.64796196	0.017	0.018	1
Hdac8	0.6044241	0.38284959	0.019	0.02	1
Bbs9	0.60556831	0.84515551	0.015	0.014	1
Alg9	0.60645573	0.77553484	0.023	0.025	1
Fam122a	0.60672499	0.40655603	0.059	0.059	1
Trmt5	0.60991152	0.72782192	0.01	0.009	1
Dynlt1a	0.61308683	0.57149018	0.01	0.011	1
Fiz1	0.61438738	0.67016942	0.067	0.068	1
Gtf2h4	0.61797056	0.58219012	0.016	0.017	1
Pop1	0.61862666	0.63392433	0.01	0.01	1
Rad51d	0.62003492	0.586635	0.024	0.023	1
Zfp266	0.62030099	0.42066241	0.09	0.091	1
Ythdf3	0.62047556	0.41944267	0.203	0.225	1
Fgd3	0.62200425	0.46477391	0.073	0.077	1
Brix1	0.62321232	0.52346601	0.096	0.103	1
Scrib	0.62377619	0.50198339	0.033	0.035	1
Gm34961	0.62421019	0.67144836	0.01	0.009	1
Cenpj	0.62431477	0.57685625	0.012	0.011	1
Ddx56	0.62567267	0.71677079	0.038	0.037	1
Fam199x	0.62707118	0.29253408	0.027	0.029	1
Caap1	0.62725995	0.44198055	0.055	0.058	1
Gm26533	0.62824464	0.74796621	0.01	0.01	1
Usp34	0.62851553	0.33031154	0.249	0.277	1
Alkbh2	0.63309967	0.49402566	0.012	0.012	1
Babam2	0.63440439	0.55322761	0.1	0.106	1
Hist2h2aa1	0.63478288	0.47722479	0.036	0.036	1
Iqce	0.6382675	0.51788843	0.012	0.013	1
Zfp182	0.63980478	0.46751514	0.021	0.022	1
Stag1	0.64010586	0.46463974	0.167	0.178	1
Cmc4	0.64112867	0.49494112	0.015	0.016	1
Cep152	0.64195115	0.50529292	0.013	0.014	1
Ccp110	0.64264276	0.63748643	0.016	0.015	1
Gpatch1	0.64293572	0.7665491	0.015	0.014	1
Kat6a	0.64349986	0.43972314	0.198	0.213	1
Otud4	0.64407598	0.49668014	0.12	0.125	1

Zfp367	0.64589187	0.64061105	0.023	0.023	1
D830025C05F	0.64755631	0.65554142	0.025	0.024	1
Tmem184c	0.64901438	0.60337607	0.021	0.02	1
Ap4b1	0.65158372	0.64878783	0.01	0.011	1
Cdyl2	0.65229152	0.37442473	0.012	0.013	1
Hibch	0.65322409	0.37303112	0.015	0.016	1
Rae1	0.65387414	0.53916853	0.069	0.073	1
Itgb3bp	0.65472605	0.41691555	0.026	0.026	1
Nup155	0.65659651	0.57516618	0.03	0.03	1
Ap5z1	0.65729778	0.77996938	0.01	0.01	1
Fam133b	0.65838546	0.43130072	0.186	0.203	1
Zfp408	0.65919336	0.54548448	0.015	0.016	1
Wdr55	0.66043899	0.69280727	0.022	0.022	1
Scfd1	0.66086438	0.51669249	0.113	0.121	1
1600020E01R	0.66358811	0.64151597	0.055	0.058	1
BC037034	0.66537944	0.58568838	0.026	0.027	1
Rab5b	0.66599603	0.5583107	0.078	0.083	1
Ngrn	0.6703338	0.44720334	0.036	0.036	1
Gm28875	0.67147154	0.54473789	0.022	0.023	1
Cry2	0.67628487	0.59294722	0.023	0.023	1
Krtcap3	0.67850416	0.36408941	0.011	0.012	1
1700052K11R	0.67995021	0.36997045	0.01	0.01	1
Xrn1	0.68087019	0.52688404	0.121	0.126	1
Wars2	0.68101586	0.6664252	0.012	0.012	1
Nek8	0.68164387	0.28416745	0.016	0.016	1
Pik3ap1	0.68215415	0.52314485	0.066	0.07	1
Lias	0.68276665	0.48360028	0.059	0.062	1
Wdcp	0.68281385	0.4328619	0.015	0.016	1
Spout1	0.68357386	0.73705225	0.02	0.019	1
Brf2	0.68528637	0.67655988	0.018	0.017	1
Plgrkt	0.68950309	0.49353312	0.198	0.216	1
Arid2	0.69236475	0.56444265	0.054	0.056	1
Vps8	0.69666626	0.7332712	0.021	0.021	1
Haus6	0.70427587	0.40497918	0.035	0.036	1
Kpnb1	0.70428719	0.48988059	0.131	0.141	1
Tubb2b	0.70549263	0.37862252	0.011	0.012	1
BC002059	0.70566087	0.5157581	0.016	0.015	1
Daxx	0.7072549	0.55619311	0.054	0.056	1
Nup43	0.70729151	0.69777911	0.012	0.012	1
Abcb8	0.71292519	0.65841094	0.011	0.011	1
Aaas	0.71309204	0.67362353	0.018	0.017	1
Nrde2	0.71319577	0.50745069	0.049	0.051	1
Wdr92	0.71470519	0.29451248	0.013	0.013	1
Nrros	0.71622315	0.48873606	0.148	0.157	1
Vars2	0.71972523	0.65399299	0.01	0.011	1
Psip1	0.7225348	0.34076719	0.142	0.147	1
Rsbn1	0.72411401	0.29194475	0.15	0.156	1
Paxip1	0.72763421	0.55627892	0.04	0.042	1

Rufy1	0.72813602	0.81450211	0.033	0.034	1
N6amt1	0.72839899	0.59434448	0.058	0.061	1
Mus81	0.72913153	0.62821448	0.012	0.011	1
Acbd4	0.7336448	0.34074321	0.011	0.012	1
Smim1	0.73431582	0.60052492	0.013	0.012	1
Snx19	0.73524664	0.59297051	0.024	0.025	1
Hspb1	0.73531359	1.02692014	0.016	0.016	1
Nat2	0.73561694	0.46994106	0.012	0.013	1
Rnf31	0.73619503	0.5745923	0.048	0.049	1
Pdxdc1	0.73627549	0.62017141	0.05	0.052	1
Cbwd1	0.73873592	0.65298238	0.023	0.023	1
Gm26670	0.73886109	0.30523991	0.01	0.01	1
Cdk9	0.7418918	0.50441415	0.154	0.164	1
Smarcal1	0.74507091	0.59648826	0.018	0.017	1
Zfp398	0.74602482	0.35295651	0.049	0.051	1
Nckipsd	0.74604715	0.82004827	0.013	0.013	1
4930522L14Ri	0.74677869	0.36468789	0.018	0.019	1
Rsl24d1	0.74888683	0.55353669	0.115	0.12	1
Safb	0.74911891	0.40102765	0.185	0.202	1
Tsen54	0.74943179	0.36414218	0.012	0.013	1
Gemin2	0.74990174	0.37065253	0.021	0.022	1
Cfl2	0.74998832	0.52681006	0.096	0.099	1
Chsy1	0.7500775	0.49181683	0.063	0.066	1
Smpd4	0.75124654	0.60736609	0.031	0.031	1
Rmi2	0.75142119	0.34933922	0.011	0.011	1
Zfp930	0.75268529	0.40726534	0.015	0.015	1
Top2b	0.75316222	0.27417399	0.277	0.306	1
2610002M06F	0.75336393	0.52440332	0.03	0.031	1
Cops7a	0.75354616	0.6777994	0.041	0.041	1
Tirap	0.75500241	0.50794123	0.016	0.016	1
Pabpc4	0.75508905	0.43842425	0.081	0.084	1
Ciz1	0.75817292	0.61390737	0.047	0.049	1
Gm27010	0.7584696	0.67089581	0.01	0.011	1
Psmd5	0.7588476	0.58789366	0.034	0.034	1
Ncdn	0.7609539	0.4629554	0.026	0.027	1
Ccdc66	0.76278277	0.35534251	0.022	0.023	1
Bcs1l	0.76432961	0.66366581	0.013	0.012	1
Zbtb26	0.76659977	0.51511093	0.026	0.026	1
Pcnx3	0.76788434	0.63488891	0.033	0.033	1
Ccdc84	0.77065527	0.45422293	0.021	0.021	1
AI480526	0.77068904	0.48671465	0.021	0.02	1
Mrps2	0.7714687	0.52914304	0.017	0.017	1
Ralgps1	0.77373024	0.68895222	0.01	0.01	1
Krba1	0.77381862	0.32226982	0.013	0.012	1
Tdp2	0.77472359	0.37178301	0.07	0.071	1
Dcaf5	0.77536282	0.47452489	0.046	0.046	1
Asb1	0.77588913	0.54107633	0.015	0.015	1
Snapin	0.77593812	0.66777653	0.049	0.049	1

Tmem175	0.77970191	0.44937409	0.012	0.013	1
Ikzf5	0.78407126	0.37514227	0.093	0.097	1
Rfc3	0.78512316	0.62687801	0.04	0.042	1
Ddx11	0.78617042	0.34445346	0.011	0.011	1
Zfp821	0.78744543	0.78630985	0.033	0.034	1
Atxn2	0.78842759	0.59647311	0.07	0.073	1
Suv39h1	0.7909786	0.6065224	0.024	0.025	1
Setd1a	0.791249	0.5039573	0.054	0.056	1
mt-Nd4l	0.79234603	0.34367713	0.318	0.366	1
Map2k7	0.79691269	0.45293069	0.119	0.123	1
Ggps1	0.79852214	0.42064578	0.084	0.088	1
Phf21a	0.79942508	0.4354022	0.08	0.083	1
2610021A01R	0.80450315	0.4339632	0.013	0.013	1
Pias3	0.80550903	0.41402873	0.018	0.018	1
Xpo5	0.80780919	0.55601246	0.011	0.012	1
Mettl17	0.80914519	0.50118593	0.019	0.019	1
Atxn7l2	0.81092351	0.47780248	0.015	0.016	1
Klf9	0.81460824	0.44793095	0.047	0.048	1
Osbpl2	0.81495967	0.46229724	0.036	0.037	1
Rab23	0.81667025	0.27669436	0.009	0.01	1
Zbed4	0.81775432	0.69306342	0.026	0.026	1
Nup153	0.81862718	0.43754891	0.152	0.162	1
Rnps1	0.81932204	0.41746681	0.216	0.236	1
Arhgap35	0.81979273	0.47795645	0.011	0.011	1
Gm16124	0.82248282	0.38253794	0.01	0.01	1
Tmem62	0.82400432	0.49901367	0.014	0.014	1
Arhgap6	0.82606125	0.93822632	0.027	0.027	1
Gm2000	0.82620935	0.69164665	0.051	0.052	1
mt-Nd6	0.82680049	0.54632577	0.067	0.068	1
Wee1	0.83051802	0.36001518	0.018	0.019	1
Pigo	0.83061733	0.45488073	0.019	0.019	1
Ttll4	0.83143299	0.54951306	0.018	0.018	1
Pkmyt1	0.83366981	0.71642291	0.013	0.013	1
Ppp1r37	0.83501829	0.44972217	0.046	0.046	1
Atrip	0.83513234	0.58195211	0.022	0.023	1
Cep63	0.83587479	0.54415838	0.051	0.051	1
Ndufaf1	0.83642431	0.43185748	0.018	0.018	1
Stx17	0.83763733	0.71866561	0.029	0.03	1
Rnmt	0.83891505	0.5231912	0.111	0.118	1
Psmg2	0.84003061	0.49218647	0.039	0.04	1
Polr1e	0.84187963	0.56825674	0.011	0.012	1
Nbeal1	0.84205089	0.51477181	0.081	0.084	1
Sugp1	0.84241421	0.47852252	0.059	0.06	1
Zfp946	0.84261722	0.39681701	0.012	0.013	1
Terf2ip	0.84278759	0.29174222	0.038	0.038	1
Dvl3	0.84456138	0.50333376	0.039	0.04	1
Prpsap2	0.84587275	0.58802032	0.037	0.038	1
Med24	0.84698391	0.58653731	0.032	0.032	1

Mlycd	0.84744045	0.6736988	0.013	0.013	1
Cdk19	0.84748915	0.51837562	0.08	0.082	1
Mrpl47	0.84975445	0.77464552	0.024	0.024	1
Ctu2	0.85121423	0.58019193	0.041	0.043	1
Wiz	0.8512996	0.58708614	0.031	0.032	1
Trmt6	0.85246356	0.61615301	0.074	0.077	1
Ctbp1	0.85264632	0.44094989	0.185	0.201	1
Echdc1	0.85272623	0.61680587	0.017	0.017	1
Psenen	0.85364841	0.31193978	0.324	0.371	1
Ighmbp2	0.85706949	0.45465925	0.018	0.018	1
Zfp597	0.86028363	0.5325023	0.026	0.027	1
Triap1	0.86253083	0.52059134	0.058	0.059	1
Ints8	0.86268943	0.43796767	0.037	0.037	1
Sass6	0.8627505	0.58487542	0.015	0.015	1
Vps50	0.86287257	0.85759417	0.041	0.042	1
Fbxo8	0.86443821	0.37621003	0.058	0.059	1
Ints3	0.86891527	0.69630101	0.031	0.032	1
Rbm34	0.8795861	0.52430306	0.106	0.112	1
Sfswap	0.88058272	0.49336517	0.147	0.158	1
Zfp933	0.88144514	0.56480952	0.011	0.011	1
Gys1	0.88146499	0.67608891	0.01	0.01	1
Cep104	0.8841463	0.77302053	0.021	0.021	1
Exoc6	0.88476978	0.58872396	0.047	0.048	1
Zswim1	0.88618789	0.4048817	0.013	0.013	1
Ippk	0.88767621	0.44893581	0.01	0.01	1
Kmt2c	0.88992512	0.37534113	0.197	0.214	1
Lum	0.89312632	0.32838404	0.015	0.015	1
Tomm70a	0.89371642	0.44612591	0.153	0.164	1
Myef2	0.89472121	0.43245431	0.083	0.086	1
Ipo4	0.89489563	0.88312234	0.021	0.021	1
Ttc38	0.89556295	0.45818619	0.012	0.011	1
Stk38	0.89571297	0.34657475	0.134	0.141	1
Trmt10b	0.89778835	0.4912006	0.01	0.01	1
Zbtb24	0.89823286	0.59182042	0.044	0.045	1
Acd	0.89879954	0.59115476	0.059	0.06	1
Prpf31	0.90001276	0.59969996	0.055	0.056	1
1110059G10R	0.90076835	0.51290647	0.091	0.096	1
Nedd4l	0.90224769	0.7854005	0.037	0.039	1
Baz2a	0.90293691	0.53053759	0.123	0.131	1
Ythdc2	0.90302975	0.5250284	0.065	0.067	1
Eral1	0.90338407	0.5702939	0.015	0.015	1
Mtg1	0.90897007	0.7270564	0.011	0.011	1
Sergef	0.91050695	0.69934622	0.028	0.029	1
Rc3h2	0.91365643	0.35469894	0.175	0.186	1
Slc9a8	0.91589057	0.55206015	0.063	0.065	1
Hist1h4h	0.91592452	0.40357773	0.036	0.036	1
Gnpnat1	0.91776053	0.41352658	0.044	0.045	1
Lsm2	0.9178974	0.58575334	0.087	0.091	1

Rad50	0.91983284	0.25207863	0.081	0.082	1
Zbtb41	0.92125992	0.37727894	0.033	0.033	1
Tlr7	0.92149786	0.84067292	0.039	0.04	1
Pias2	0.92709887	0.43557041	0.049	0.049	1
Pik3cd	0.92754647	0.36081439	0.142	0.15	1
Ndor1	0.92755249	0.51290497	0.009	0.01	1
Wdr36	0.92825303	0.6498518	0.05	0.051	1
Tars2	0.92916576	0.36402426	0.028	0.029	1
Med23	0.92929371	0.46783696	0.047	0.048	1
Slc30a4	0.93034964	0.83880529	0.021	0.021	1
2700016F22Ri	0.93378883	0.67508962	0.012	0.012	1
Sec61a2	0.93476776	0.67835728	0.027	0.028	1
Cnpy3	0.93676712	0.86344619	0.078	0.083	1
Prpf8	0.93990913	0.44620959	0.193	0.21	1
Fastkd2	0.94043996	0.63977226	0.021	0.021	1
Rbbp7	0.94169856	0.40361642	0.174	0.186	1
Wdr74	0.94244945	0.82923854	0.022	0.023	1
Gpcpd1	0.94293448	0.36769182	0.223	0.245	1
Mavs	0.94422438	0.70805819	0.012	0.012	1
Smg6	0.94653567	0.37143461	0.13	0.137	1
Efl1	0.95261228	0.55699462	0.037	0.038	1
Dock9	0.9573217	0.27363913	0.018	0.018	1
Cdc25a	0.95780004	0.49674	0.033	0.033	1
Zfp26	0.95784153	0.47857831	0.047	0.048	1
Hpse	0.96065062	0.76964406	0.027	0.028	1
Atl2	0.96313062	0.43778297	0.092	0.095	1
Pyroxd1	0.96341716	0.47087246	0.047	0.048	1
Aida	0.96346451	0.63736194	0.073	0.076	1
Dgkq	0.9660811	0.5529314	0.017	0.017	1
Smug1	0.96628146	0.5146906	0.019	0.019	1
Kat2a	0.96657356	0.66621454	0.017	0.017	1
Nphs1	0.97175041	0.62481988	0.01	0.01	1
Pwwp2a	0.97318256	0.41515395	0.081	0.083	1
Zfp512b	0.97715383	0.51496149	0.011	0.011	1
Mad2l1	0.97788958	0.29217014	0.022	0.022	1
Rbmxl1	0.97972544	0.66916226	0.095	0.1	1
Ipo11	0.98062651	0.52578178	0.021	0.022	1
Myo5a	0.983745	0.62953076	0.1	0.106	1
Zfyve27	0.99072935	0.52282021	0.049	0.05	1
Slc28a2	0.99320454	0.32243165	0.045	0.046	1
Vegfb	0.99580366	0.71697063	0.024	0.024	1
Ddx39b	0.99747159	0.47351994	0.194	0.213	1
Tmem126b	0.99805018	0.47296669	0.025	0.025	1
Zfp513	0.99965637	0.76930366	0.041	0.042	1

CD4 Th1

Gene	p_val	avg_log2FC	pct.1	pct.2	p_val_adj
Icos	0	1.60143047	0.816	0.315	0
Rora	0	0.9668249	0.873	0.405	0
Cd28	0	1.73088691	0.706	0.275	0
Trbc2	0	1.07509217	0.748	0.355	0
Ctla4	0	1.32745329	0.647	0.259	0
Cd3g	0	1.18416807	0.663	0.285	0
Ptpn22	0	1.43679533	0.729	0.351	0
Ramp3	0	1.64005287	0.729	0.353	0
Hcst	0	1.35695669	0.808	0.439	0
Cd3d	0	1.1301308	0.65	0.287	0
Cd3e	0	1.25848877	0.608	0.265	0
Emb	0	0.99849468	0.927	0.593	0
Ifngr1	0	1.25666414	0.922	0.594	0
Gimap4	0	1.4953335	0.583	0.257	0
Gimap6	0	1.20641415	0.711	0.386	0
Ets1	0	1.00048156	0.956	0.641	0
Cxcr6	0	1.19821638	0.545	0.234	0
Dgat1	0	0.96841888	0.744	0.434	0
Gimap3	0	1.35556985	0.592	0.285	0
Maf	0	1.3873289	0.608	0.304	0
Gramd3	0	1.46235052	0.544	0.242	0
Nr3c1	0	1.22524449	0.714	0.412	0
Aebp2	0	1.26723855	0.798	0.501	0
Cd2	0	1.24097001	0.56	0.264	0
Sh2d2a	0	1.16802264	0.569	0.277	0
Lat	0	1.41715305	0.493	0.206	0
Il18r1	0	1.55558356	0.466	0.183	0
Trac	0	1.31564636	0.488	0.21	0
Itk	0	1.05226267	0.573	0.296	0
Rnf125	0	1.63354828	0.447	0.184	0
Cd6	0	1.93864498	0.392	0.129	0
Cd4	0	1.81685388	0.397	0.135	0
Slamf1	0	1.9638863	0.387	0.126	0
Kdm2b	0	1.14582591	0.597	0.336	0
Gimap7	0	1.60294494	0.419	0.16	0
Tnfrsf4	0	1.14760678	0.408	0.151	0
Cd5	0	1.9806529	0.371	0.117	0
Stk4	0	1.26175936	0.629	0.376	0
Zeb1	0	1.18322594	0.583	0.332	0
Odc1	0	1.07422453	0.848	0.601	0
Hif1a	0	1.09634029	0.798	0.552	0
Stat5b	0	1.33038764	0.507	0.262	0
Il7r	0	1.23290732	0.621	0.377	0
Ppp1cc	0	1.15486402	0.619	0.376	0
Ubald2	0	1.1785224	0.783	0.54	0
Zfp36l2	0	1.23979746	0.697	0.455	0

Cd40lg	0	2.17839855	0.314	0.076	0
Fyb	0	0.97796738	0.728	0.491	0
Tnfaip3	0	0.72209198	0.888	0.652	0
Grap2	0	1.68146995	0.389	0.162	0
Vps37b	0	0.7924079	0.873	0.646	0
Tcf7	0	1.22968296	0.384	0.159	0
Trat1	0	2.44969529	0.283	0.067	0
Akap13	0	1.09326146	0.94	0.737	0
Got1	0	1.24820221	0.643	0.442	0
Hmgb2	0	1.2389692	0.903	0.705	0
Clint1	0	1.10593371	0.661	0.466	0
Tesc	0	1.90001118	0.291	0.098	0
Tmsb10	0	0.66336829	0.982	0.884	0
Rps15a	0	0.42973552	0.99	0.955	0
Tpt1	0	0.28372208	0.999	0.995	0
Bcl11b	2.807E-303	1.3978556	0.359	0.151	6.159E-299
Pnrc1	1.368E-299	0.70150295	0.865	0.727	3.001E-295
Rps16	7.431E-297	0.33598845	0.996	0.98	1.63E-292
Bhlhe40	1.429E-287	0.76422728	0.801	0.586	3.135E-283
D16Ertd472e	1.738E-285	1.00598083	0.595	0.358	3.813E-281
Thy1	5.358E-281	1.16474202	0.456	0.228	1.176E-276
Gimap1	2.385E-275	1.15918784	0.484	0.259	5.233E-271
Rps14	2.553E-275	0.32410019	0.994	0.975	5.602E-271
Actn2	1.038E-271	1.38725028	0.303	0.119	2.277E-267
Ltb	2.032E-271	1.23167168	0.479	0.257	4.458E-267
Tnfsf8	2.947E-269	1.47334779	0.293	0.115	6.465E-265
Rps13	1.386E-267	0.35183541	0.991	0.966	3.041E-263
Rpl19	2.577E-262	0.35121162	0.996	0.968	5.654E-258
Rpl12	5.1E-261	0.38720188	0.991	0.944	1.119E-256
Spn	1.006E-257	1.31389681	0.396	0.196	2.208E-253
Pde4d	1.37E-252	1.49320141	0.332	0.15	3.005E-248
Lck	2.448E-249	1.23587186	0.384	0.185	5.372E-245
Ostf1	1.655E-237	0.91977403	0.667	0.514	3.632E-233
Abcb1a	1.435E-236	1.48131495	0.34	0.162	3.148E-232
Rpl18a	1.557E-232	0.33008729	0.995	0.968	3.416E-228
Gm26740	1.293E-229	1.19683535	0.4	0.205	2.836E-225
Dusp10	3.555E-228	1.76753702	0.256	0.104	7.8E-224
Tshz1	1.852E-225	1.61293236	0.278	0.119	4.063E-221
Plcxd2	3.555E-223	1.41569629	0.33	0.155	7.799E-219
Mbnl1	2.496E-221	0.60621669	0.862	0.758	5.476E-217
Orai1	1.283E-219	1.03734787	0.505	0.315	2.816E-215
Skap1	5.076E-219	1.12266886	0.403	0.211	1.114E-214
Cdkn1b	5.321E-218	0.99582918	0.565	0.385	1.167E-213
Rpl24	4.138E-217	0.38886525	0.982	0.946	9.078E-213
Rpl34	1.142E-215	0.31727667	0.994	0.968	2.506E-211
Rgs1	2.462E-214	0.69113984	0.763	0.548	5.401E-210
Ubb	3.385E-214	0.5587932	0.898	0.814	7.426E-210
Furin	8.517E-212	0.76680676	0.61	0.407	1.869E-207

Tnfrsf18	8.364E-211	0.91089895	0.444	0.24	1.835E-206
Eif1	2.157E-209	0.34739258	0.988	0.977	4.731E-205
Gna13	2.145E-204	0.7428135	0.778	0.669	4.706E-200
Rpl18	2.455E-204	0.35230324	0.989	0.958	5.387E-200
Ddx5	1.504E-203	0.41302025	0.974	0.951	3.299E-199
Riok1	1.606E-200	1.11506986	0.476	0.301	3.524E-196
H2afz	3.872E-199	0.53321338	0.859	0.714	8.496E-195
Themis	5.168E-199	1.55910472	0.226	0.09	1.134E-194
Fasl	1.829E-197	1.59603863	0.227	0.092	4.014E-193
Crot	7.646E-197	1.6051898	0.263	0.117	1.678E-192
Mbd2	1.668E-196	1.04403564	0.522	0.361	3.66E-192
Arap2	7.106E-196	0.87125481	0.582	0.389	1.559E-191
Zcchc11	2.395E-195	0.75500154	0.692	0.543	5.254E-191
Camk4	1.796E-191	1.365052	0.281	0.129	3.94E-187
AU020206	5.28E-187	1.12400695	0.423	0.252	1.158E-182
Sept1	5.196E-185	0.96936988	0.478	0.294	1.14E-180
P2ry10	1.494E-183	0.79882205	0.623	0.447	3.278E-179
Rnf138	4.123E-182	1.31571162	0.34	0.184	9.046E-178
Zc3hav1	9.093E-180	0.86103621	0.587	0.433	1.995E-175
Atxn1	1.018E-176	1.25151197	0.34	0.182	2.233E-172
Fryl	3.21E-176	1.26122541	0.368	0.21	7.042E-172
Rpsa	2.03E-175	0.30729554	0.994	0.963	4.453E-171
AW112010	1.331E-172	0.54723878	0.647	0.45	2.921E-168
Crbn	1.393E-171	1.35561467	0.307	0.159	3.055E-167
Rpl30	2.104E-170	0.25829612	0.994	0.968	4.616E-166
Rpl17	4.126E-169	0.27141956	0.994	0.968	9.052E-165
Zdhhc18	2.796E-168	0.90414585	0.517	0.35	6.134E-164
Mfsd6	3.898E-167	1.37768432	0.285	0.142	8.552E-163
H2-Q7	1.971E-166	0.59143999	0.75	0.614	4.325E-162
Pgm2l1	2.688E-166	1.5121063	0.224	0.098	5.896E-162
Cxcr3	4.402E-166	2.01610323	0.132	0.042	9.657E-162
Sipa1l1	2.003E-162	1.01164574	0.46	0.304	4.395E-158
Rps11	3.941E-162	0.31754025	0.99	0.957	8.645E-158
Cblb	3.272E-157	1.00652968	0.441	0.282	7.178E-153
Rpl11	4.615E-157	0.31686667	0.98	0.943	1.013E-152
H2-K1	2.216E-156	0.37355881	0.963	0.909	4.861E-152
Il12rb2	5.267E-156	1.70304223	0.167	0.063	1.155E-151
Neurl3	6.336E-155	0.70317515	0.648	0.512	1.39E-150
Tnfsf11	1.596E-154	1.13154587	0.227	0.1	3.502E-150
Prdx6	1.29E-153	0.71876661	0.636	0.505	2.83E-149
Satb1	1.817E-153	0.4838795	0.732	0.523	3.986E-149
Ctla2a	3.246E-152	0.97440344	0.308	0.157	7.121E-148
Gm12840	4.189E-148	0.95820891	0.371	0.216	9.19E-144
Ubl5	1.043E-147	0.60628544	0.763	0.688	2.288E-143
Cd53	2.718E-146	0.54267374	0.786	0.703	5.963E-142
Rack1	1.065E-145	0.31113771	0.973	0.934	2.337E-141
St8sia1	9.708E-145	2.28730425	0.085	0.022	2.13E-140
Ubash3a	3.291E-144	1.90241251	0.127	0.042	7.221E-140

Crem	2.035E-142	0.59197381	0.714	0.585	4.465E-138
Smco4	1.209E-139	1.3813395	0.217	0.101	2.653E-135
Ern1	1.317E-139	0.96696126	0.392	0.244	2.89E-135
Rpl36	2.607E-139	0.25926241	0.992	0.956	5.72E-135
Rinl	9.1E-138	1.15523785	0.292	0.159	1.997E-133
Tex2	5.264E-137	1.15080261	0.319	0.183	1.155E-132
Ptp4a2	8.454E-137	0.54418422	0.788	0.718	1.855E-132
Lmnb1	1.247E-135	0.56908465	0.578	0.431	2.737E-131
Itpkb	1.885E-135	0.95859689	0.509	0.378	4.136E-131
Ppia	6.453E-135	0.32640238	0.96	0.912	1.416E-130
Dnajb6	1.582E-134	0.67808525	0.666	0.571	3.471E-130
Abcb1b	2.187E-134	1.39573621	0.268	0.144	4.797E-130
Rpl9	2.772E-133	0.26070262	0.99	0.958	6.082E-129
Nebl	5.189E-133	1.59807602	0.163	0.066	1.138E-128
Trib2	1.129E-131	1.18930405	0.238	0.116	2.477E-127
Vcpkmt	1.93E-130	1.73198621	0.156	0.064	4.235E-126
Sh2d1a	6.096E-130	1.45644485	0.171	0.072	1.337E-125
Tmem64	8.646E-130	1.00333266	0.372	0.225	1.897E-125
Serbp1	2.674E-129	0.48487437	0.812	0.744	5.866E-125
Cd247	2.883E-127	1.22243094	0.24	0.12	6.324E-123
1190002N15R	1.03E-125	1.77019295	0.14	0.054	2.26E-121
Faah	1.882E-124	1.31246199	0.206	0.097	4.129E-120
Rnf19a	2.591E-121	1.17285009	0.275	0.153	5.685E-117
Saraf	8.077E-120	0.88324843	0.492	0.371	1.772E-115
Rasgrp1	2.377E-119	1.25732738	0.27	0.15	5.215E-115
Carnmt1	2.553E-119	1.3764092	0.246	0.132	5.6E-115
Rundc3b	5.59E-119	1.62040709	0.139	0.055	1.226E-114
Cytip	1.054E-118	0.41866907	0.841	0.732	2.313E-114
Rbm39	3.118E-118	0.37385185	0.898	0.858	6.84E-114
Sesn3	8.9E-118	1.02824257	0.34	0.209	1.953E-113
Trbc1	1.823E-117	0.69021957	0.407	0.251	3.999E-113
Gmfg	6.487E-117	0.64414702	0.625	0.534	1.423E-112
Prrc2c	2.221E-114	0.49746526	0.765	0.7	4.873E-110
Ms4a4b	2.4E-114	0.55181302	0.397	0.238	5.266E-110
Gpr132	2.497E-114	0.66232074	0.607	0.504	5.478E-110
Acsbg1	1.522E-113	1.9152586	0.101	0.034	3.339E-109
Gapdh	1.52E-111	0.29795558	0.845	0.736	3.334E-107
Uqcrh	1.61E-110	0.36672111	0.899	0.858	3.532E-106
Stk26	4.655E-110	1.36017863	0.186	0.089	1.021E-105
Arhgap31	1.793E-109	1.03261602	0.34	0.218	3.933E-105
Cnbp	1.852E-109	0.49944359	0.738	0.671	4.063E-105
Arpc2	4.349E-108	0.46989483	0.816	0.737	9.541E-104
Paxbp1	5.77E-108	1.09301731	0.336	0.217	1.266E-103
Ptpn18	5.944E-108	0.55294049	0.686	0.609	1.304E-103
Il21r	1.672E-107	0.90518315	0.436	0.313	3.669E-103
Serpinb1a	1.719E-107	1.21655363	0.25	0.136	3.77E-103
Kcnmb4	2.117E-107	1.89142759	0.102	0.036	4.643E-103
Dusp5	1.588E-106	0.58836351	0.651	0.531	3.483E-102

Sub1	1.983E-106	0.42478129	0.868	0.809	4.35E-102
Btg2	2.335E-106	0.51881365	0.644	0.535	5.122E-102
Tnrc6b	5.738E-106	0.77592939	0.465	0.338	1.259E-101
Esyt2	1.415E-105	0.88490621	0.368	0.243	3.105E-101
Stat4	8.437E-105	0.73705177	0.503	0.373	1.851E-100
Arid5a	3.92E-103	0.97406459	0.379	0.262	8.599E-99
Sntb1	8.495E-101	1.78767843	0.099	0.035	1.8637E-96
Ppp1r12a	1.154E-100	0.84441084	0.453	0.346	2.5313E-96
Rplp0	1.367E-100	0.28491767	0.969	0.932	2.9985E-96
Nfatc2	1.475E-100	1.68173688	0.151	0.068	3.2366E-96
Sytl2	7.359E-100	1.42495784	0.148	0.066	1.6145E-95
Mier1	1.232E-99	0.77471954	0.506	0.399	2.703E-95
Leprotl1	4.749E-99	0.8516061	0.421	0.308	1.0419E-94
Smap2	1.9332E-98	0.85771043	0.453	0.342	4.2412E-94
Gm47015	2.0544E-98	1.80119361	0.1	0.036	4.5072E-94
Ube2b	3.9183E-98	0.57087887	0.644	0.568	8.5963E-94
Dtx4	2.1628E-96	1.65291466	0.141	0.063	4.7449E-92
Il18rap	2.5548E-96	0.92698068	0.276	0.161	5.605E-92
Gtf2i	2.9848E-96	1.07773601	0.251	0.145	6.5483E-92
Chst2	3.1473E-96	2.06956272	0.072	0.022	6.9048E-92
4930523C07R	4.8764E-96	0.63372575	0.534	0.416	1.0698E-91
Clk1	5.3867E-96	0.46055245	0.742	0.678	1.1818E-91
Oaz1	8.8341E-96	0.34590812	0.885	0.828	1.9381E-91
Dgka	2.758E-95	1.15603863	0.2	0.104	6.0508E-91
B4galnt4	1.576E-94	2.53477054	0.045	0.01	3.4577E-90
Rab8b	4.2069E-93	0.47353376	0.682	0.579	9.2296E-89
Sit1	6.5905E-93	2.05188976	0.071	0.021	1.4459E-88
Utrn	2.9985E-92	0.91114073	0.349	0.234	6.5783E-88
Limd2	2.708E-90	0.79572812	0.421	0.309	5.9412E-86
Prr13	7.6525E-90	0.7045161	0.509	0.418	1.6789E-85
Rpl27	7.3859E-89	0.28529232	0.915	0.879	1.6204E-84
Lgals1	7.3422E-87	0.39903773	0.626	0.504	1.6108E-82
Eef2	2.4393E-86	0.30444494	0.909	0.875	5.3516E-82
Epb41	4.3826E-86	0.86471536	0.432	0.331	9.6149E-82
Grb7	5.6859E-86	1.83603458	0.074	0.024	1.2474E-81
Cox7c	9.049E-86	0.35321196	0.866	0.819	1.9853E-81
Gabpb1	5.5532E-85	1.0474471	0.262	0.161	1.2183E-80
Rwdd1	4.759E-84	0.9500514	0.4	0.309	1.0441E-79
Hells	2.8254E-83	1.58117711	0.129	0.058	6.1986E-79
Peli1	4.4479E-82	0.64607772	0.557	0.463	9.7582E-78
Sdf4	1.5797E-81	0.77514878	0.453	0.362	3.4657E-77
Man2a2	1.7113E-81	1.26017274	0.195	0.108	3.7545E-77
Atad2	3.2211E-81	0.94916239	0.268	0.166	7.0669E-77
Zap70	3.9364E-81	1.00866859	0.224	0.129	8.6361E-77
Ppp1r16b	4.0552E-81	0.62581136	0.437	0.311	8.8967E-77
Atp8a2	4.9832E-81	2.32676421	0.044	0.01	1.0933E-76
D8Ertd738e	8.0608E-81	0.58482885	0.601	0.539	1.7685E-76
Cir1	3.0877E-80	0.9168821	0.325	0.223	6.7742E-76

Eif3h	4.1358E-80	0.53017815	0.653	0.599	9.0736E-76
Hopx	4.3769E-80	0.45463637	0.342	0.23	9.6025E-76
Paip2	6.9451E-80	0.6538193	0.506	0.421	1.5237E-75
Uhrf2	4.4654E-79	0.6224343	0.446	0.337	9.7966E-75
Mat2b	5.5287E-79	0.88797302	0.34	0.239	1.2129E-74
Tecpr1	5.5689E-79	0.72744365	0.328	0.214	1.2218E-74
Nt5e	5.6094E-79	1.15339217	0.175	0.091	1.2306E-74
Slc3a2	1.0575E-78	0.51615393	0.677	0.608	2.3201E-74
Madd	3.8282E-78	0.96859949	0.326	0.228	8.3988E-74
Ipmk	1.1953E-77	0.7140014	0.238	0.14	2.6224E-73
Npm1	3.3619E-77	0.27732929	0.892	0.851	7.3756E-73
Sod1	4.3504E-77	0.6140343	0.583	0.516	9.5443E-73
Shisa5	5.4963E-77	0.40626823	0.661	0.584	1.2058E-72
Jakmip1	2.5766E-74	0.89170402	0.229	0.135	5.6529E-70
Fosl2	2.3711E-73	0.55093063	0.591	0.51	5.2019E-69
Usmg5	3.3223E-73	0.53500253	0.671	0.621	7.2887E-69
S100a10	3.3655E-73	0.37745574	0.658	0.55	7.3836E-69
Rpl13a	3.5402E-73	0.51322817	0.626	0.577	7.7668E-69
Pik3r1	3.6094E-73	0.74635624	0.45	0.358	7.9187E-69
Chd2	4.9357E-73	0.57909262	0.561	0.488	1.0828E-68
Ncor1	6.5585E-73	0.53387325	0.574	0.501	1.4389E-68
Ccr2	6.9376E-73	0.67251668	0.351	0.245	1.522E-68
1110034G24R	2.3417E-70	1.88337848	0.06	0.019	5.1374E-66
Rac2	3.5416E-69	0.45542513	0.636	0.575	7.7699E-65
Atp5h	5.6611E-69	0.38326263	0.776	0.738	1.242E-64
Abhd17c	1.1617E-68	1.33513002	0.14	0.071	2.5487E-64
Inpp1	1.714E-68	1.30807194	0.143	0.073	3.7603E-64
Ndfip1	3.819E-68	0.75726754	0.371	0.277	8.3786E-64
Lef1	5.8503E-68	0.65830855	0.159	0.082	1.2835E-63
Rnf157	8.294E-68	0.87346436	0.29	0.196	1.8196E-63
Nsd3	1.1919E-67	0.58603706	0.491	0.404	2.6149E-63
Atp2b4	2.5869E-67	0.92951453	0.28	0.19	5.6755E-63
Gm19585	1.5033E-66	0.82319017	0.226	0.136	3.2981E-62
S1pr4	1.8862E-66	1.4306379	0.113	0.053	4.1381E-62
Celf2	3.2058E-66	0.50406818	0.606	0.539	7.0332E-62
Gimap5	8.6215E-66	0.66071116	0.324	0.22	1.8915E-61
Aplp2	1.8362E-65	0.77356011	0.408	0.326	4.0285E-61
Igtp	4.4861E-65	0.92244276	0.203	0.12	9.842E-61
4932438A13R	7.2793E-64	0.66042299	0.462	0.38	1.597E-59
Mkrn1	1.3335E-63	0.66055624	0.474	0.408	2.9255E-59
Gimap9	5.9054E-63	0.87236827	0.214	0.129	1.2956E-58
Map1lc3b	1.0205E-62	0.43933352	0.632	0.583	2.2388E-58
Vgll4	4.6254E-62	0.64674366	0.411	0.312	1.0148E-57
Pdcd4	5.4669E-62	0.62335892	0.411	0.313	1.1994E-57
Nabp1	1.0849E-61	0.48762253	0.47	0.37	2.3801E-57
Pfdn5	4.3689E-61	0.34307227	0.801	0.77	9.585E-57
Naca	7.5885E-60	0.2930187	0.869	0.829	1.6649E-55
S100a13	2.2967E-59	0.62832043	0.46	0.389	5.0387E-55

Sla	4.0765E-59	0.60656739	0.476	0.399	8.9434E-55
Kbtbd11	5.6552E-59	0.97063533	0.191	0.114	1.2407E-54
Fus	7.0625E-59	0.37859924	0.763	0.749	1.5494E-54
Gm33104	2.8935E-58	1.51498156	0.094	0.043	6.348E-54
St6galnac1	5.1239E-58	2.83725627	0.022	0.004	1.1241E-53
Itgb3	5.5612E-58	0.90101186	0.181	0.106	1.2201E-53
Lamc1	1.7941E-57	1.00688118	0.164	0.093	3.936E-53
Tprgl	2.5562E-57	0.88575508	0.291	0.21	5.6079E-53
Polg2	4.5198E-57	1.17219505	0.129	0.067	9.9161E-53
Cd96	6.3454E-57	1.18290443	0.129	0.067	1.3921E-52
Phf6	1.6613E-56	1.21901505	0.16	0.092	3.6448E-52
Zdhhc2	5.1657E-56	1.49311966	0.086	0.038	1.1333E-51
Supt4a	1.2885E-55	0.59342896	0.506	0.45	2.8269E-51
Tgfbr2	2.3932E-55	0.78344916	0.312	0.229	5.2504E-51
Sgtb	1.7171E-54	1.82570054	0.053	0.018	3.7672E-50
Etaa1	1.8279E-54	1.27751361	0.113	0.057	4.0102E-50
Il2rg	7.3457E-54	0.47548925	0.54	0.47	1.6116E-49
Fyn	7.7394E-54	0.67060883	0.416	0.345	1.6979E-49
Klk8	9.4965E-54	1.03304009	0.161	0.094	2.0834E-49
Arpc3	9.9987E-54	0.34229366	0.81	0.77	2.1936E-49
Rpl29	2.1042E-53	0.29267005	0.894	0.853	4.6164E-49
Nr1d2	2.4327E-53	0.85622519	0.247	0.167	5.337E-49
Inpp4b	3.3411E-53	0.79047797	0.22	0.14	7.3301E-49
Rabac1	4.5995E-53	0.58832613	0.49	0.434	1.0091E-48
Pde3b	4.6147E-53	0.73164416	0.344	0.262	1.0124E-48
H2-Q4	1.1126E-52	0.77997817	0.33	0.251	2.441E-48
Rbm3	1.2923E-52	0.29367012	0.815	0.787	2.8351E-48
Gnas	2.0686E-52	0.42841858	0.718	0.706	4.5382E-48
Sumo2	2.3964E-52	0.49589155	0.571	0.534	5.2575E-48
Arpp19	2.4484E-52	0.51673295	0.527	0.478	5.3716E-48
Aes	2.5436E-52	0.59545116	0.465	0.402	5.5805E-48
Sp4	3.2825E-52	1.03397584	0.192	0.12	7.2014E-48
Ss18	3.6806E-52	0.93334758	0.237	0.161	8.0749E-48
Tnfrsf25	1.1139E-51	1.43884758	0.089	0.041	2.4438E-47
Cirbp	3.2173E-51	0.63533629	0.376	0.295	7.0585E-47
Gprin3	3.3697E-51	1.64731305	0.061	0.024	7.3928E-47
Arntl	4.998E-51	1.17073248	0.138	0.077	1.0965E-46
Myl12b	6.1342E-51	0.43969664	0.591	0.546	1.3458E-46
Ar	9.5153E-51	0.96901361	0.17	0.101	2.0876E-46
Cnst	1.3288E-50	1.6235033	0.07	0.03	2.9152E-46
Cd47	2.7079E-50	0.4780227	0.589	0.543	5.941E-46
Prkar1a	7.3428E-50	0.42225034	0.63	0.595	1.6109E-45
Klf3	2.4629E-49	0.90204112	0.282	0.207	5.4033E-45
Pmepa1	4.2421E-49	0.81126455	0.339	0.263	9.3068E-45
Mllt3	5.3856E-49	1.02215643	0.175	0.108	1.1816E-44
Grap	2.0124E-48	0.8131279	0.198	0.126	4.415E-44
Vps54	2.47E-48	0.9480019	0.197	0.129	5.419E-44
Podnl1	4.1151E-48	1.47766345	0.067	0.028	9.0282E-44

Slc4a7	1.9046E-47	0.80713003	0.328	0.258	4.1785E-43
Ankrd11	2.3556E-47	0.41630618	0.626	0.596	5.1678E-43
Raf1	2.6973E-47	0.99811102	0.221	0.15	5.9176E-43
Mgea5	8.7008E-47	0.65430105	0.404	0.339	1.9089E-42
Lysmd2	9.5486E-47	1.48351557	0.072	0.032	2.0949E-42
Lpxn	1.1288E-46	0.81740495	0.3	0.23	2.4764E-42
Ntrk3	4.2051E-46	1.70607291	0.052	0.02	9.2255E-42
Syf2	6.9135E-46	0.57410004	0.459	0.407	1.5167E-41
Pja1	3.4864E-45	1.15884235	0.125	0.07	7.6487E-41
Axin2	4.7702E-45	1.3021526	0.072	0.032	1.0465E-40
Eif3f	6.0286E-45	0.33684695	0.71	0.689	1.3226E-40
Cd82	6.6748E-45	0.5442408	0.425	0.359	1.4644E-40
Myl12a	1.9493E-44	0.4251502	0.599	0.561	4.2765E-40
Sh3kbp1	3.0981E-44	0.70964651	0.361	0.294	6.7968E-40
A630023P12R	4.062E-44	1.79505248	0.036	0.011	8.9116E-40
Ctsw	5.9054E-44	0.69172178	0.212	0.14	1.2956E-39
Rsbn1l	1.1928E-43	0.57701383	0.424	0.356	2.617E-39
Bzw2	1.2881E-43	0.80679411	0.243	0.173	2.8259E-39
Bnip3l	2.1071E-43	0.69197339	0.372	0.311	4.6227E-39
Il16	5.8317E-43	0.8810961	0.163	0.101	1.2794E-38
Dnajc15	7.5609E-43	1.02345936	0.251	0.189	1.6588E-38
Ybx3	1.6979E-42	0.80218153	0.35	0.29	3.725E-38
Psmb1	2.3101E-42	0.43469063	0.57	0.541	5.0682E-38
Bicdl1	2.3473E-42	1.64819772	0.044	0.016	5.1497E-38
Chd3	2.896E-42	0.59980262	0.361	0.287	6.3534E-38
Hmgb1	1.2628E-41	0.30963098	0.683	0.662	2.7704E-37
Foxo1	2.6559E-41	0.5938079	0.379	0.304	5.8268E-37
Nmrk1	4.0682E-41	0.74093636	0.184	0.119	8.9253E-37
Gypc	4.8157E-41	1.38780916	0.094	0.05	1.0565E-36
Ski	1.3961E-40	0.87501125	0.224	0.16	3.0629E-36
Crlf3	1.891E-40	0.57128293	0.415	0.355	4.1486E-36
Ikzf3	2.3182E-40	0.42695993	0.378	0.29	5.0859E-36
Slain1	8.4194E-40	1.00647992	0.116	0.066	1.8471E-35
Btf3	8.9501E-40	0.25972568	0.841	0.81	1.9636E-35
Gpr174	1.1336E-39	0.8975371	0.142	0.085	2.487E-35
Mxd4	2.5908E-39	0.80537401	0.24	0.175	5.6839E-35
Mxi1	1.0645E-38	0.69001291	0.357	0.296	2.3355E-34
Khdrbs1	1.8414E-38	0.55643074	0.475	0.438	4.0398E-34
Ccr4	1.8637E-38	0.89015605	0.093	0.049	4.0887E-34
H2afy	2.1298E-38	0.4540578	0.547	0.509	4.6726E-34
Idh3a	2.2232E-38	1.14473117	0.145	0.092	4.8775E-34
Ttc39b	5.9598E-38	0.82197073	0.26	0.197	1.3075E-33
Ogt	8.408E-38	0.56623789	0.377	0.312	1.8446E-33
Dennd4c	8.4767E-38	1.21647648	0.113	0.066	1.8597E-33
AI467606	1.1115E-37	1.04109234	0.129	0.077	2.4385E-33
Sgip1	1.2127E-37	1.49229244	0.051	0.021	2.6605E-33
Gm10827	1.2558E-37	1.06391455	0.084	0.043	2.7552E-33
Mepce	2.9629E-37	0.94114258	0.171	0.113	6.5004E-33

4833420G17R	3.6034E-37	0.84355901	0.222	0.159	7.9056E-33
Fam107b	4.2775E-37	0.31791885	0.612	0.56	9.3845E-33
Dbf4	4.3109E-37	1.11279707	0.149	0.096	9.4577E-33
2810001G20R	4.5031E-37	1.31485161	0.07	0.033	9.8793E-33
Eif2s2	1.4913E-36	0.31631341	0.703	0.675	3.2718E-32
Emg1	3.8621E-36	0.65788417	0.353	0.301	8.473E-32
Ddx24	4.6553E-36	0.4367817	0.537	0.505	1.0213E-31
Aak1	6.6239E-36	0.87224128	0.183	0.124	1.4532E-31
Prrc2b	3.3546E-35	0.74150211	0.242	0.18	7.3596E-31
Cwc15	5.7501E-35	0.58050662	0.429	0.393	1.2615E-30
Cnot6l	6.606E-35	0.5094704	0.441	0.386	1.4493E-30
St6galnac3	8.9473E-35	1.02987187	0.101	0.057	1.963E-30
Sec62	1.8861E-34	0.39067145	0.557	0.531	4.138E-30
Smap1	2.2013E-34	0.7391388	0.325	0.275	4.8293E-30
Slc25a40	3.4239E-34	1.25357073	0.075	0.038	7.5116E-30
Smad7	3.4735E-34	0.55529932	0.375	0.312	7.6204E-30
Cdk17	4.2252E-34	0.76634717	0.197	0.138	9.2698E-30
Runx2	1.5745E-33	0.86660809	0.219	0.163	3.4543E-29
Tigit	1.7465E-33	1.47761436	0.051	0.022	3.8316E-29
Tnfaip8	2.003E-33	0.59087588	0.428	0.385	4.3943E-29
Dek	2.2412E-33	0.4071221	0.525	0.487	4.917E-29
Crmp1	2.6503E-33	1.57240532	0.051	0.022	5.8145E-29
Kmt2a	3.3332E-33	0.52488147	0.384	0.324	7.3127E-29
Gpr68	3.4195E-33	1.16937421	0.122	0.075	7.5021E-29
Gm15655	3.497E-33	1.46537123	0.05	0.022	7.6721E-29
Fam102a	4.1577E-33	0.8074378	0.166	0.111	9.1216E-29
Gm29243	4.5754E-33	1.36085719	0.055	0.025	1.0038E-28
Ier5l	6.0864E-33	0.78345967	0.196	0.136	1.3353E-28
Avl9	1.3769E-32	0.66232268	0.288	0.227	3.0209E-28
Hnrnpa0	2.5023E-32	0.36755555	0.612	0.608	5.4898E-28
Gna15	2.716E-32	1.12939276	0.121	0.075	5.9586E-28
Zbtb1	3.1314E-32	0.8573691	0.189	0.133	6.8699E-28
Mpzl3	4.5193E-32	1.47199426	0.051	0.022	9.9148E-28
Rgs2	9.6244E-32	0.41604536	0.532	0.482	2.1115E-27
Ly6a	1.313E-31	0.37757178	0.385	0.314	2.8807E-27
Prrt1	1.4146E-31	1.17481752	0.081	0.044	3.1034E-27
Ubr2	1.4399E-31	0.87378458	0.221	0.166	3.159E-27
Polr1d	1.8141E-31	0.44405216	0.507	0.488	3.9799E-27
Plcg1	2.3555E-31	1.01599206	0.114	0.069	5.1677E-27
Ttc39c	2.6662E-31	1.0078544	0.116	0.071	5.8493E-27
Gm2245	2.7415E-31	1.69942248	0.035	0.014	6.0145E-27
Dnajc8	2.8838E-31	0.53237584	0.434	0.401	6.3268E-27
Diaph1	3.6333E-31	0.45130032	0.492	0.459	7.9711E-27
Myh9	5.3085E-31	0.35935695	0.6	0.581	1.1646E-26
Bik	5.3609E-31	1.52254191	0.042	0.018	1.1761E-26
Arl6ip1	5.6741E-31	0.38020751	0.552	0.528	1.2448E-26
Pym1	9.3561E-31	1.07763016	0.129	0.083	2.0526E-26
Zfp773	1.0213E-30	1.93479278	0.029	0.01	2.2407E-26

Ap3s1	1.1437E-30	0.7890084	0.259	0.209	2.5092E-26
Zbtb25	1.1553E-30	1.1272032	0.091	0.052	2.5347E-26
Ube2i	2.7407E-30	0.45166746	0.483	0.458	6.0128E-26
Fam169b	2.8547E-30	0.6809695	0.142	0.091	6.263E-26
Cdk2ap2	2.9928E-30	0.27499804	0.445	0.403	6.5659E-26
Cyld	3.288E-30	0.54368987	0.363	0.312	7.2136E-26
Slc35e2	3.5124E-30	1.21902792	0.081	0.045	7.7059E-26
Cbx4	5.4901E-30	0.78680697	0.167	0.115	1.2045E-25
Scrt1	5.8065E-30	1.43780474	0.049	0.022	1.2739E-25
Itpr1	5.9099E-30	0.72522221	0.249	0.193	1.2966E-25
Hipk1	6.8865E-30	0.62497976	0.342	0.294	1.5108E-25
Sri	7.1137E-30	0.56228748	0.411	0.378	1.5607E-25
Ube2s	1.1353E-29	0.40849381	0.494	0.466	2.4907E-25
D030056L22R	1.7015E-29	1.26457751	0.082	0.046	3.733E-25
Elob	1.8851E-29	0.34394578	0.595	0.578	4.1358E-25
Esm1	2.6156E-29	2.49702267	0.013	0.003	5.7384E-25
Xist	2.6444E-29	0.34226377	0.482	0.418	5.8015E-25
Zfp874b	2.6505E-29	1.29911671	0.065	0.033	5.8149E-25
Itgal	3.947E-29	0.49837417	0.37	0.316	8.6593E-25
Klf13	4.5135E-29	0.60777998	0.355	0.309	9.9022E-25
Sumo1	5.6612E-29	0.55018295	0.411	0.38	1.242E-24
Nipal3	8.4417E-29	1.20168303	0.075	0.041	1.852E-24
Ulk1	1.1098E-28	0.97173613	0.147	0.1	2.4348E-24
Tpr	1.273E-28	0.42522101	0.53	0.513	2.7927E-24
Sytl1	1.2952E-28	1.5190748	0.044	0.02	2.8416E-24
Ehd3	1.4621E-28	1.3557218	0.059	0.03	3.2076E-24
Srpk1	1.6409E-28	0.74818026	0.236	0.183	3.6E-24
Ldha	1.997E-28	0.39068637	0.52	0.49	4.3813E-24
Cenpl	2.5164E-28	1.11263717	0.129	0.085	5.5206E-24
H60b	3.241E-28	1.53618099	0.038	0.016	7.1103E-24
Mbp	3.3955E-28	0.8905292	0.199	0.15	7.4494E-24
Gls	3.9913E-28	0.46380045	0.501	0.479	8.7565E-24
Krtcap2	5.338E-28	0.51980327	0.431	0.401	1.1711E-23
Selplg	5.3904E-28	0.37519395	0.457	0.409	1.1826E-23
Stk39	1.5694E-27	1.12211771	0.084	0.049	3.4431E-23
Tmpo	2.5116E-27	0.71529055	0.221	0.168	5.5102E-23
Vax2	2.8569E-27	1.79076235	0.032	0.013	6.2677E-23
Znrf1	3.3997E-27	0.72822527	0.18	0.13	7.4586E-23
Plekha5	3.5492E-27	1.07141513	0.099	0.06	7.7865E-23
5830411N06R	5.0947E-27	2.0556957	0.014	0.003	1.1177E-22
Tnks2	7.6823E-27	0.67360137	0.305	0.26	1.6854E-22
Eif3e	1.1205E-26	0.32873437	0.585	0.574	2.4582E-22
Ypel3	1.187E-26	0.60394458	0.275	0.222	2.6042E-22
Stk10	1.3355E-26	0.71643042	0.249	0.198	2.93E-22
Lrig1	1.6339E-26	1.36065221	0.039	0.017	3.5846E-22
Ccdc88c	1.8936E-26	0.65053043	0.204	0.15	4.1544E-22
Hivep2	2.8237E-26	0.58787284	0.348	0.303	6.1949E-22
Ccm2	7.0088E-26	0.90059331	0.17	0.124	1.5377E-21

Brd1	9.5715E-26	0.6042562	0.328	0.284	2.0999E-21
Pon2	1.015E-25	0.87058979	0.19	0.144	2.2268E-21
Lsm8	1.0728E-25	0.72637313	0.222	0.174	2.3537E-21
Cited2	1.5031E-25	0.69288882	0.211	0.16	3.2976E-21
Higd1a	1.6711E-25	0.66897574	0.344	0.308	3.6662E-21
Cenpa	1.934E-25	0.42453539	0.437	0.391	4.243E-21
Eef1d	2.621E-25	0.31753992	0.586	0.578	5.7502E-21
Emsy	3.0277E-25	0.6736649	0.244	0.192	6.6424E-21
Top2b	3.0568E-25	0.5548588	0.343	0.297	6.7062E-21
Cox14	3.4685E-25	0.61354238	0.394	0.372	7.6095E-21
Crybg1	4.3938E-25	0.90747755	0.172	0.127	9.6396E-21
Camk2n1	4.7134E-25	0.74771335	0.135	0.091	1.0341E-20
Inf2	5.1497E-25	1.23353054	0.091	0.056	1.1298E-20
Gng2	5.8202E-25	0.60075512	0.35	0.313	1.2769E-20
Pkp3	8.5684E-25	0.93560141	0.125	0.083	1.8798E-20
Ctcf	1.2608E-24	0.57461084	0.322	0.275	2.766E-20
Pde7a	1.8957E-24	0.43434565	0.276	0.216	4.159E-20
Ubn1	1.9193E-24	0.6240994	0.303	0.262	4.2107E-20
Nemp2	2.4393E-24	1.19671226	0.07	0.039	5.3517E-20
Smc3	3.5827E-24	0.68587201	0.262	0.216	7.86E-20
Setd5	4.5591E-24	0.44807978	0.448	0.421	1.0002E-19
Dennd2d	6.5706E-24	1.04254509	0.074	0.042	1.4415E-19
Dkkl1	7.0688E-24	1.29486771	0.054	0.028	1.5508E-19
Smarce1	8.3273E-24	0.66830519	0.305	0.268	1.8269E-19
Purb	1.0387E-23	0.42305165	0.52	0.512	2.2788E-19
Fam103a1	1.4868E-23	0.73277923	0.233	0.188	3.2619E-19
Ndufv3	1.7587E-23	0.54137259	0.391	0.366	3.8585E-19
Isy1	2.4847E-23	0.40655919	0.456	0.422	5.4512E-19
Cldnd1	3.2985E-23	0.75017427	0.276	0.235	7.2365E-19
Sept6	3.9972E-23	0.63124321	0.247	0.199	8.7695E-19
Zdhhc23	7.9603E-23	1.18073198	0.079	0.047	1.7464E-18
B4galt1	8.9889E-23	0.58459858	0.285	0.239	1.9721E-18
Rab39b	9.2145E-23	1.67787751	0.031	0.013	2.0216E-18
Cpm	1.016E-22	0.76645145	0.099	0.062	2.229E-18
Cish	1.2045E-22	0.57403555	0.262	0.214	2.6426E-18
Elf1	1.2623E-22	0.25551075	0.511	0.462	2.7694E-18
Ifng	1.3917E-22	0.58528632	0.085	0.052	3.0533E-18
Mrpl9	1.6138E-22	0.78523939	0.219	0.176	3.5405E-18
Slfn1	1.9851E-22	0.45250962	0.106	0.068	4.3551E-18
Olfr60	2.5415E-22	1.41767447	0.039	0.018	5.5758E-18
2410089E03R	2.6183E-22	1.05089271	0.085	0.052	5.7442E-18
Dock2	3.4385E-22	0.42367855	0.446	0.42	7.5437E-18
Sdcbp2	3.5501E-22	1.26712031	0.066	0.038	7.7886E-18
Cd164	4.5994E-22	0.49274026	0.434	0.411	1.0091E-17
Birc2	5.8075E-22	0.4702045	0.37	0.328	1.2741E-17
Mrps5	8.4366E-22	1.0314512	0.119	0.082	1.8509E-17
Gm14718	8.6056E-22	1.32122569	0.03	0.013	1.888E-17
Rad18	1.7595E-21	0.88660098	0.108	0.072	3.8601E-17

Gpc1	1.7851E-21	1.31615838	0.067	0.039	3.9164E-17
Gabarapl2	2.0977E-21	0.53209646	0.377	0.352	4.6021E-17
Kcnmb4os2	3.2138E-21	1.44515054	0.033	0.014	7.0507E-17
Rtf1	3.9697E-21	0.50688083	0.362	0.329	8.7091E-17
5033430I15Ril	4.2995E-21	1.67863496	0.026	0.01	9.4328E-17
Uba52	5.3348E-21	0.33316214	0.526	0.519	1.1704E-16
Prr7	7.7519E-21	0.57889507	0.207	0.16	1.7007E-16
Gstp3	7.9287E-21	1.01842857	0.082	0.051	1.7395E-16
Nlk	1.3984E-20	0.97002807	0.095	0.061	3.0679E-16
Ncoa2	1.5874E-20	0.64559565	0.229	0.185	3.4827E-16
Gimap8	1.6633E-20	0.55405213	0.147	0.104	3.6492E-16
Tomm7	1.9106E-20	0.29239324	0.638	0.647	4.1916E-16
Nop53	2.2888E-20	0.35354904	0.54	0.54	5.0213E-16
Slc39a4	2.7255E-20	1.23273353	0.047	0.024	5.9794E-16
Zadh2	3.3355E-20	1.11371346	0.067	0.04	7.3177E-16
Fkbp3	7.2965E-20	0.56996547	0.296	0.257	1.6008E-15
2010111I01Ril	1.0548E-19	0.99701569	0.136	0.099	2.314E-15
Smad3	1.3696E-19	0.841053	0.131	0.093	3.0047E-15
Paip2b	1.6001E-19	0.79902264	0.123	0.086	3.5104E-15
Il17re	2.1047E-19	1.51920502	0.032	0.014	4.6176E-15
Tnik	2.1536E-19	0.67044769	0.127	0.089	4.7247E-15
Gm15283	2.5836E-19	1.53955079	0.037	0.018	5.6681E-15
March3	4.0825E-19	1.40975402	0.03	0.013	8.9566E-15
Ip6k1	6.6109E-19	0.6362038	0.203	0.162	1.4504E-14
Cep97	7.0443E-19	1.108285	0.049	0.027	1.5454E-14
Prkcq	7.5011E-19	0.76842114	0.077	0.047	1.6457E-14
Mosmo	8.3902E-19	1.01079812	0.098	0.066	1.8407E-14
Sh2b1	1.0049E-18	0.83043783	0.099	0.066	2.2046E-14
Dnaja4	1.3362E-18	1.0142693	0.064	0.038	2.9315E-14
Tob1	1.5582E-18	0.84665691	0.109	0.075	3.4186E-14
Acoxl	1.8265E-18	2.23169664	0.014	0.005	4.0072E-14
Nab1	2.041E-18	0.53812962	0.345	0.318	4.4778E-14
Galnt9	2.0511E-18	2.30410538	0.012	0.003	4.5E-14
Gm20275	2.1774E-18	2.11591878	0.011	0.003	4.7769E-14
Acot2	3.0079E-18	1.03723342	0.059	0.034	6.5991E-14
Il2	3.6687E-18	0.39465938	0.042	0.022	8.0488E-14
Gm15340	4.5373E-18	1.09060102	0.029	0.013	9.9545E-14
Prkca	4.6118E-18	0.76104186	0.099	0.066	1.0118E-13
Gpx4	7.5258E-18	0.2528431	0.554	0.535	1.6511E-13
Ppil1	8.8939E-18	1.15489468	0.064	0.038	1.9512E-13
BC031181	1.0621E-17	0.64308583	0.298	0.272	2.3301E-13
Fam118a	1.7651E-17	1.00642503	0.055	0.032	3.8724E-13
Virma	2.0218E-17	0.80127489	0.152	0.116	4.4357E-13
Phf20l1	2.1353E-17	0.44916677	0.342	0.309	4.6847E-13
Srp14	2.7529E-17	0.39353983	0.446	0.439	6.0397E-13
Rrad	2.7683E-17	0.77048687	0.124	0.09	6.0733E-13
Ube2j1	3.7463E-17	0.69062758	0.274	0.244	8.219E-13
Gt(ROSA)26Sor	6.559E-17	0.63525522	0.253	0.219	1.439E-12

Fancf	7.5641E-17	1.11488289	0.052	0.03	1.6595E-12
4932438H23R	8.1951E-17	1.41749915	0.03	0.014	1.7979E-12
Gpr15	8.9344E-17	1.99596003	0.012	0.004	1.9601E-12
Wasf2	1.1566E-16	0.44998033	0.388	0.373	2.5374E-12
Prkch	1.4656E-16	0.58985678	0.193	0.153	3.2154E-12
Tmem65	1.8562E-16	1.15904382	0.104	0.074	4.0724E-12
Tnk2	1.891E-16	0.91713053	0.064	0.039	4.1486E-12
Trbv12-2	1.9059E-16	1.93009701	0.016	0.006	4.1814E-12
H2afv	1.9719E-16	0.39262011	0.318	0.283	4.3262E-12
Rad21	2.2102E-16	0.4904529	0.295	0.263	4.8489E-12
Isca1	2.2228E-16	0.68653118	0.131	0.097	4.8766E-12
Nfkb2	2.2742E-16	0.52711456	0.325	0.301	4.9893E-12
Acvr2a	2.4064E-16	1.08662345	0.077	0.05	5.2793E-12
Hs3st3b1	2.835E-16	1.2568801	0.043	0.024	6.2198E-12
Tma7	4.398E-16	0.27578495	0.572	0.58	9.6488E-12
Tab2	5.8282E-16	0.35548441	0.435	0.418	1.2786E-11
Mknk2	6.3882E-16	0.50197927	0.305	0.275	1.4015E-11
Ormdl3	7.9884E-16	0.72371944	0.11	0.078	1.7526E-11
Ric1	8.079E-16	0.62951124	0.253	0.223	1.7724E-11
Frg1	8.1423E-16	0.59182655	0.264	0.233	1.7863E-11
Map4k2	1.016E-15	0.60971246	0.135	0.1	2.229E-11
L1cam	1.6609E-15	1.16821758	0.043	0.024	3.6439E-11
Rnf166	2.1455E-15	0.78339235	0.148	0.115	4.7069E-11
Nxpe3	2.2276E-15	0.98691865	0.045	0.026	4.887E-11
Adgrl1	2.3846E-15	0.9338551	0.063	0.039	5.2316E-11
Fnbp1	2.7198E-15	0.39733112	0.415	0.401	5.9671E-11
Ddb2	2.7977E-15	1.15808282	0.054	0.032	6.138E-11
Srp9	4.3952E-15	0.37116995	0.478	0.478	9.6426E-11
Heca	5.2712E-15	0.51067507	0.262	0.226	1.1565E-10
Il27ra	5.8315E-15	0.76256625	0.081	0.054	1.2794E-10
Prkcz	6.5807E-15	1.10698272	0.043	0.024	1.4437E-10
Zfp672	6.6761E-15	0.8227947	0.14	0.108	1.4647E-10
Rpa2	7.9801E-15	0.75137561	0.093	0.064	1.7507E-10
H2afj	7.9918E-15	0.36217896	0.503	0.506	1.7533E-10
Gm2682	8.2913E-15	0.7691457	0.045	0.025	1.819E-10
A930024E05R	8.7794E-15	0.77511403	0.079	0.052	1.9261E-10
Jak2	9.879E-15	0.45524182	0.457	0.45	2.1673E-10
Tomm20	1.1689E-14	0.34036492	0.496	0.5	2.5645E-10
Mien1	1.7308E-14	0.57890401	0.281	0.257	3.7971E-10
Fam96b	1.8012E-14	0.68967352	0.235	0.208	3.9517E-10
H2-T22	2.0825E-14	0.55436797	0.264	0.234	4.5688E-10
Slc20a1	2.7601E-14	0.55635654	0.208	0.174	6.0554E-10
Trim59	2.7825E-14	0.44785054	0.14	0.105	6.1046E-10
Mob2	2.9088E-14	0.75208329	0.15	0.117	6.3816E-10
Fam110a	2.9807E-14	0.61524208	0.173	0.139	6.5393E-10
Trbv2	3.2145E-14	1.8824313	0.014	0.005	7.0524E-10
Gm19557	3.3084E-14	1.4398426	0.024	0.011	7.2584E-10
Mettl23	3.3615E-14	0.5812845	0.254	0.226	7.3748E-10

Unc45a	4.8553E-14	0.79217901	0.107	0.078	1.0652E-09
Bdh1	5.5107E-14	1.47305942	0.025	0.012	1.209E-09
N4bp2l2	5.8195E-14	0.3545493	0.418	0.401	1.2767E-09
Abhd8	5.9933E-14	1.04564054	0.051	0.031	1.3149E-09
Zfp292	7.5356E-14	0.41620614	0.281	0.246	1.6532E-09
Tnfrsf26	9.0807E-14	0.77118375	0.047	0.028	1.9922E-09
Abhd2	9.2602E-14	0.64202065	0.16	0.127	2.0316E-09
Churc1	9.4249E-14	0.67400948	0.238	0.212	2.0677E-09
Pcmtd2	1.1263E-13	0.64791256	0.127	0.096	2.471E-09
Pak2	1.4741E-13	0.31218795	0.519	0.53	3.234E-09
Krt83	1.506E-13	0.72633221	0.054	0.033	3.304E-09
Bcl2l1	1.5832E-13	0.71702183	0.145	0.114	3.4733E-09
Lsm5	1.9111E-13	0.44744348	0.322	0.298	4.1927E-09
Terf1	2.2954E-13	0.73662247	0.103	0.075	5.0358E-09
Gsk3b	2.3358E-13	0.56772066	0.274	0.248	5.1245E-09
Top2a	2.7363E-13	0.30614957	0.119	0.088	6.0031E-09
Gm42372	2.7405E-13	1.48268995	0.019	0.008	6.0125E-09
Ssh2	2.8058E-13	0.2682807	0.376	0.339	6.1557E-09
Nif3l1	3.2594E-13	1.18251058	0.041	0.024	7.1507E-09
Spcs2	3.537E-13	0.39280378	0.436	0.434	7.7597E-09
Fbxl14	4.6803E-13	0.94917459	0.081	0.057	1.0268E-08
Tmem191c	4.9864E-13	1.173272	0.037	0.021	1.094E-08
Senp6	5.1804E-13	0.35043198	0.378	0.357	1.1365E-08
Sytl3	5.3602E-13	0.36627202	0.168	0.131	1.176E-08
Tmem154	5.4991E-13	1.01274669	0.049	0.03	1.2065E-08
Zc3h13	5.9501E-13	0.48182864	0.313	0.29	1.3054E-08
Tnfrsf10b	6.9095E-13	0.88718879	0.042	0.025	1.5159E-08
Ubap2	8.3497E-13	0.83597612	0.118	0.09	1.8318E-08
Zfp707	8.8247E-13	1.44367849	0.027	0.014	1.936E-08
Itpk1	9.8842E-13	0.88222359	0.099	0.073	2.1685E-08
Kdm5a	1.0666E-12	0.41711509	0.339	0.316	2.3399E-08
1500009L16Ri	1.1539E-12	1.74437146	0.019	0.008	2.5316E-08
Fam124b	1.1629E-12	1.36460405	0.021	0.01	2.5513E-08
Gnpda2	1.2388E-12	0.94051008	0.084	0.06	2.7178E-08
Sp3	1.2698E-12	0.57776776	0.233	0.204	2.7858E-08
Trp53inp1	1.2748E-12	0.45146635	0.24	0.203	2.7968E-08
Krit1	1.3093E-12	0.43571666	0.317	0.291	2.8725E-08
Tbc1d30	1.4356E-12	1.12328558	0.033	0.018	3.1495E-08
Prelid2	1.4373E-12	0.93242097	0.08	0.056	3.1533E-08
Mrpl33	1.6086E-12	0.33333406	0.442	0.439	3.5292E-08
Helz	2.0495E-12	0.62883846	0.156	0.125	4.4964E-08
Nucks1	2.0732E-12	0.31537312	0.442	0.434	4.5483E-08
Cds2	2.9351E-12	0.68846536	0.113	0.085	6.4393E-08
Gm20069	3.0071E-12	1.09205984	0.038	0.022	6.5972E-08
Cebpg	3.2021E-12	0.62923024	0.209	0.182	7.025E-08
Fam58b	3.6162E-12	1.12942601	0.048	0.03	7.9336E-08
Prelid1	3.6928E-12	0.53520549	0.315	0.303	8.1017E-08
Amz2	3.7706E-12	0.85073045	0.117	0.09	8.2722E-08

F2r	4.5434E-12	0.61337084	0.095	0.069	9.9678E-08
Ankrd44	4.6793E-12	0.3792832	0.34	0.317	1.0266E-07
Cdr2	5.3606E-12	1.01332593	0.048	0.03	1.1761E-07
Cnot6	5.6683E-12	0.55771093	0.269	0.245	1.2436E-07
Pced1b	6.1216E-12	1.05245458	0.044	0.027	1.343E-07
Kmt2e	7.2373E-12	0.26211747	0.438	0.419	1.5878E-07
1810058I24Ril	7.7709E-12	0.54820183	0.303	0.29	1.7049E-07
Nlrc5	1.0296E-11	0.74154147	0.11	0.084	2.2589E-07
Cox10	1.0704E-11	0.99979822	0.071	0.05	2.3482E-07
Fdx1	1.3897E-11	0.87006314	0.145	0.118	3.0489E-07
Ctdspl2	1.5421E-11	0.68948002	0.162	0.134	3.3831E-07
Mif4gd	1.7684E-11	0.58448253	0.241	0.216	3.8797E-07
Crebbp	1.8937E-11	0.44076651	0.363	0.355	4.1546E-07
Chic2	1.9374E-11	0.48719267	0.299	0.281	4.2505E-07
Rab2a	2.0631E-11	0.36863979	0.431	0.438	4.5263E-07
Fam160a2	2.2629E-11	1.01788193	0.032	0.018	4.9646E-07
Gpbp1l1	2.3661E-11	0.5695711	0.216	0.188	5.1909E-07
Wdyhv1	2.4612E-11	0.97932265	0.057	0.038	5.3996E-07
Arl2bp	2.8537E-11	0.62581915	0.187	0.16	6.2608E-07
Hccs	2.9599E-11	0.71359003	0.169	0.142	6.4938E-07
Gm44066	3.3107E-11	1.53487134	0.014	0.006	7.2633E-07
Tmem234	3.8742E-11	0.39333832	0.421	0.426	8.4997E-07
Tgif2	3.9527E-11	0.95045266	0.056	0.037	8.6717E-07
Acadl	4.0875E-11	0.89637215	0.181	0.158	8.9675E-07
Arl6ip5	4.3441E-11	0.59116745	0.236	0.213	9.5306E-07
Mrps18c	4.5527E-11	0.69596195	0.216	0.195	9.9882E-07
Hp1bp3	5.1945E-11	0.54402697	0.284	0.265	1.1396E-06
Nol10	5.4001E-11	0.87158392	0.108	0.084	1.1847E-06
Gm26551	6.5084E-11	1.04971748	0.029	0.016	1.4279E-06
Ctdsp2	6.564E-11	0.69424898	0.171	0.146	1.4401E-06
Epsti1	8.5363E-11	0.49603636	0.323	0.311	1.8728E-06
AC147041.1	8.6899E-11	1.48355993	0.015	0.006	1.9065E-06
Tspyl1	9.1102E-11	0.66334145	0.15	0.123	1.9987E-06
Ccnd2	9.5183E-11	0.41188045	0.333	0.307	2.0882E-06
Bag4	9.7749E-11	0.81703459	0.067	0.047	2.1445E-06
Xlr4b	9.8487E-11	0.78992416	0.083	0.06	2.1607E-06
Tspyl4	1.0018E-10	1.2314158	0.023	0.012	2.1978E-06
Ldb1	1.1409E-10	0.62695182	0.138	0.111	2.5031E-06
1700012B07R	1.2046E-10	1.18810905	0.029	0.016	2.6427E-06
Bcas2	1.3078E-10	0.51473914	0.275	0.257	2.8692E-06
Fbxl12	1.4462E-10	0.53436761	0.146	0.117	3.1728E-06
Fam160b1	1.5606E-10	0.71119953	0.101	0.076	3.4238E-06
Srpk2	1.6373E-10	0.43049259	0.229	0.2	3.592E-06
Wrnip1	1.7218E-10	0.8581463	0.065	0.045	3.7775E-06
Frat2	1.9767E-10	0.71449643	0.067	0.047	4.3367E-06
Gm11808	2.2984E-10	0.51440457	0.28	0.264	5.0426E-06
Dzip1	2.5458E-10	1.55714999	0.013	0.005	5.5853E-06
Mthfd2	2.727E-10	0.64993944	0.223	0.202	5.9827E-06

Sys1	3.1604E-10	0.44643952	0.335	0.325	6.9336E-06
Asxl1	3.1969E-10	0.71954762	0.105	0.081	7.0136E-06
Bex3	3.2561E-10	0.54716136	0.225	0.202	7.1435E-06
Rbl2	3.7199E-10	0.50046375	0.17	0.142	8.1611E-06
Hist1h4d	4.1171E-10	0.40244524	0.247	0.222	9.0325E-06
Trnt1	4.7222E-10	0.69971865	0.177	0.153	1.036E-05
Tgfbr3	4.7466E-10	0.93908426	0.029	0.016	1.0413E-05
Gm35082	5.1081E-10	0.78577806	0.062	0.043	1.1207E-05
Snrnp70	5.2828E-10	0.27469885	0.51	0.524	1.159E-05
Jmy	5.9548E-10	0.57033492	0.182	0.155	1.3064E-05
Celsr1	6.0773E-10	1.1322893	0.024	0.013	1.3333E-05
Slk	6.1675E-10	0.43144008	0.343	0.333	1.3531E-05
Mri1	6.4204E-10	0.8331516	0.098	0.076	1.4086E-05
Slc37a3	6.4523E-10	0.83910102	0.057	0.039	1.4156E-05
Snapc1	7.2496E-10	0.65841376	0.153	0.128	1.5905E-05
Ldlrad4	8.3034E-10	0.7383781	0.068	0.048	1.8217E-05
Zfp654	9.4107E-10	0.74165852	0.078	0.057	2.0646E-05
Hist3h2ba	9.5425E-10	1.17311784	0.034	0.02	2.0935E-05
Zufsp	9.7822E-10	0.41064254	0.249	0.224	2.1461E-05
Aars	1.0641E-09	0.59845786	0.204	0.182	2.3346E-05
Kif2a	1.0888E-09	0.69586216	0.147	0.123	2.3887E-05
Cript	1.1114E-09	0.39717034	0.359	0.355	2.4384E-05
Selenos	1.1244E-09	0.36640225	0.418	0.422	2.4668E-05
Tmem71	1.1346E-09	0.66071595	0.09	0.067	2.4891E-05
Dut	1.1627E-09	0.51518893	0.134	0.108	2.5507E-05
Hadhb	1.4757E-09	0.58486721	0.2	0.177	3.2375E-05
Ddx46	1.729E-09	0.44074112	0.314	0.303	3.7933E-05
Kdm5b	1.7495E-09	0.50194357	0.128	0.103	3.8382E-05
AC163354.1	1.7955E-09	0.89916779	0.065	0.046	3.9391E-05
Stim1	2.3055E-09	0.55326993	0.171	0.146	5.0581E-05
Rab35	2.4188E-09	0.72089726	0.143	0.12	5.3066E-05
Crebrf	2.5755E-09	0.32115834	0.313	0.291	5.6505E-05
Mapk14	2.6985E-09	0.77688677	0.131	0.108	5.9203E-05
Hdgfl3	2.7095E-09	0.91862314	0.044	0.029	5.9444E-05
Flt3l	2.8833E-09	0.77406195	0.06	0.042	6.3256E-05
Zwint	2.9923E-09	0.86761642	0.096	0.074	6.5649E-05
Cpe	3.2765E-09	1.62273806	0.013	0.006	7.1883E-05
Nol7	3.4082E-09	0.36184207	0.417	0.424	7.4772E-05
Zfand6	3.8239E-09	0.49626971	0.286	0.272	8.3892E-05
Vopp1	4.3086E-09	0.880637	0.096	0.075	9.4526E-05
Chd1	6.6527E-09	0.48383356	0.26	0.243	0.00014595
Relb	6.6731E-09	0.47615543	0.297	0.287	0.0001464
Carmil2	6.8306E-09	0.91345211	0.037	0.023	0.00014986
Taf4b	7.6822E-09	0.66883479	0.103	0.081	0.00016854
Fbxo21	7.704E-09	0.95910493	0.049	0.033	0.00016902
Eif2d	8.1009E-09	0.86162115	0.056	0.039	0.00017772
Cmas	8.2508E-09	0.64928494	0.194	0.174	0.00018101
Zrsr2	8.9888E-09	0.62665848	0.249	0.236	0.00019721

Hs2st1	9.2843E-09	0.94346418	0.082	0.062	0.00020369
Phc3	9.351E-09	0.52987985	0.163	0.139	0.00020515
Tsc22d4	9.6805E-09	0.38314975	0.298	0.279	0.00021238
Lgalsl	1.0847E-08	1.05753014	0.027	0.015	0.00023796
Cenpw	1.0895E-08	0.78065259	0.084	0.064	0.00023903
Cdadc1	1.1107E-08	0.84597929	0.076	0.057	0.00024369
Irgm2	1.1717E-08	0.71160468	0.06	0.042	0.00025707
Gpatch8	1.2715E-08	0.48139757	0.253	0.233	0.00027896
Sptssa	1.2753E-08	0.33698947	0.26	0.241	0.00027979
Tmx4	1.3136E-08	0.62189132	0.152	0.129	0.0002882
Selenow	1.4019E-08	0.25317111	0.505	0.524	0.00030756
Ifi27	1.7395E-08	0.5052506	0.217	0.196	0.00038163
Tlk1	1.9027E-08	0.46140736	0.2	0.177	0.00041743
E4f1	1.9935E-08	0.68146561	0.099	0.078	0.00043736
Tbrg1	1.9939E-08	0.54026599	0.229	0.21	0.00043745
Pfdn2	2.0465E-08	0.4352694	0.31	0.304	0.00044898
Supt20	2.1601E-08	0.47078491	0.222	0.199	0.0004739
Numa1	2.181E-08	0.39247821	0.221	0.196	0.00047848
Phtf1	2.6873E-08	0.8765272	0.056	0.04	0.00058957
Zhx2	2.7291E-08	0.53693865	0.135	0.111	0.00059874
Trbv3	3.0706E-08	1.61941847	0.01	0.004	0.00067367
Prkx	3.2773E-08	0.5742092	0.21	0.192	0.000719
Fli1	3.2992E-08	0.48912262	0.273	0.26	0.0007238
Spryd7	3.3522E-08	1.06757316	0.07	0.053	0.00073543
Pbrm1	3.5195E-08	0.4747584	0.289	0.278	0.00077215
Zfp677	3.9344E-08	1.07232026	0.025	0.015	0.00086316
Plekhf1	3.9397E-08	1.21423029	0.02	0.011	0.00086433
Alkbh4	4.0976E-08	1.0511127	0.027	0.016	0.00089898
Ly6g5b	4.5172E-08	1.29577124	0.022	0.012	0.00099104
Pot1a	4.8392E-08	0.80992869	0.056	0.04	0.00106167
Wasl	4.866E-08	0.74407987	0.111	0.09	0.00106756
Fbxo33	4.894E-08	0.64785393	0.138	0.116	0.0010737
Fam46c	5.6105E-08	0.48715219	0.232	0.215	0.00123088
Herc3	5.8775E-08	0.8106705	0.063	0.046	0.00128947
Zfp949	6.7757E-08	0.99285983	0.037	0.025	0.00148653
Snai3	8.2618E-08	1.42575689	0.01	0.004	0.00181256
Tcp11l2	8.5797E-08	0.34586194	0.242	0.219	0.0018823
Gm20139	8.9303E-08	1.41525461	0.01	0.004	0.00195922
C1qtnf4	9.5627E-08	1.38708227	0.016	0.008	0.00209795
Pcbp4	9.7204E-08	1.22682367	0.019	0.011	0.00213256
Sacs	1.0284E-07	0.72417001	0.077	0.059	0.00225611
Tnrc6c	1.0322E-07	0.43488856	0.222	0.201	0.00226459
Gm28053	1.2918E-07	1.2500233	0.013	0.006	0.00283409
Dctn4	1.3098E-07	0.4137928	0.335	0.333	0.00287367
Arl15	1.4422E-07	1.00267092	0.039	0.027	0.00316411
Thap12	1.4436E-07	0.66016787	0.139	0.119	0.00316713
Psmd4	1.5531E-07	0.45714642	0.319	0.319	0.00340743
Hdhd5	1.586E-07	0.92542357	0.03	0.019	0.00347963

Eprs	1.713E-07	0.27524602	0.408	0.407	0.00375812
Zfp874a	1.7342E-07	0.94723428	0.031	0.02	0.00380463
Tspan5	1.7594E-07	0.9185376	0.053	0.038	0.00386004
Rexo5	1.9238E-07	1.10410203	0.02	0.011	0.00422054
Cep120	1.9788E-07	0.56152965	0.131	0.11	0.00434136
Uqcr10	2.0897E-07	0.29401978	0.447	0.459	0.00458449
Hmgb3	2.1577E-07	0.99342935	0.036	0.024	0.00473375
Pkn2	2.2069E-07	0.40753465	0.318	0.312	0.00484169
Sap30	2.3563E-07	0.86739195	0.106	0.088	0.0051695
Aqp3	2.4889E-07	1.07252242	0.021	0.012	0.00546039
Il1r1	2.5136E-07	0.89752212	0.057	0.042	0.00551456
Ccdc59	2.5221E-07	0.51862641	0.285	0.282	0.00553316
Klhl12	2.5534E-07	0.5922652	0.064	0.048	0.005602
Hic1	2.6205E-07	1.04373905	0.054	0.04	0.00574917
Cggbp1	2.716E-07	0.37920013	0.371	0.378	0.00595871
Gmnn	3.3588E-07	0.7631436	0.075	0.058	0.00736897
9930021J03Ril	3.6592E-07	0.6377908	0.105	0.086	0.00802798
Fbxo8	3.6984E-07	0.61096909	0.075	0.057	0.00811385
Acap1	3.7266E-07	0.51148547	0.121	0.101	0.00817575
Lcn4	4.3648E-07	0.92564972	0.023	0.013	0.00957583
Arhgap45	4.6596E-07	0.32563494	0.29	0.274	0.01022279
Gm14029	4.8052E-07	1.01846925	0.025	0.015	0.01054206
Srsf2	5.0305E-07	0.28997524	0.477	0.501	0.01103649
Klrb1f	5.2282E-07	1.02922415	0.024	0.015	0.01147021
Kmt5b	5.51E-07	0.51437137	0.184	0.164	0.01208829
Tbx21	6.05E-07	0.69312745	0.056	0.041	0.01327317
Slc22a15	6.5321E-07	0.5850013	0.074	0.058	0.01433085
Sv2c	6.8165E-07	0.97703172	0.029	0.019	0.01495464
Mns1	7.1263E-07	0.84183281	0.03	0.019	0.01563445
Yy1	7.4271E-07	0.40253242	0.323	0.323	0.01629425
Rbmx	7.7562E-07	0.61246379	0.082	0.065	0.01701639
Snw1	7.8944E-07	0.38840302	0.322	0.319	0.01731944
Fam189b	7.9522E-07	0.63527336	0.056	0.041	0.01744639
2510002D24R	8.0263E-07	0.59041439	0.096	0.077	0.01760899
Zc3h15	8.1072E-07	0.32803113	0.381	0.385	0.01778632
Cd226	8.6415E-07	0.73951557	0.064	0.049	0.01895861
Gch1	9.1687E-07	0.42278647	0.279	0.27	0.02011517
Hectd1	9.4099E-07	0.37805473	0.318	0.316	0.02064439
Slc35f2	1.021E-06	1.20145226	0.012	0.006	0.02239965
Haus3	1.0374E-06	0.49892858	0.116	0.096	0.02276005
Atp10a	1.063E-06	0.71688796	0.075	0.059	0.02332152
Gramd1c	1.0742E-06	1.31481102	0.014	0.007	0.02356768
Bcorl1	1.128E-06	1.13438892	0.019	0.011	0.02474725
Prpf18	1.1601E-06	0.53852118	0.154	0.135	0.02545232
Ubtd2	1.1978E-06	1.3846661	0.024	0.015	0.02627756
Twistnb	1.2267E-06	0.52212008	0.217	0.203	0.02691327
Wac	1.2356E-06	0.34157355	0.345	0.345	0.02710727
Pdcd2	1.238E-06	0.74218635	0.084	0.068	0.02716031

Zbtb44	1.3131E-06	0.54931544	0.121	0.102	0.0288091
Maf1	1.3599E-06	0.51993141	0.159	0.141	0.02983416
Btbd11	1.3746E-06	0.9397385	0.025	0.016	0.03015683
Srebf2	1.4085E-06	0.42328964	0.313	0.312	0.0309018
Syt11	1.4176E-06	0.87339394	0.045	0.032	0.03110074
Leo1	1.4547E-06	0.74466202	0.07	0.054	0.0319151
Sec11a	1.4826E-06	0.4720775	0.263	0.255	0.03252748
Dcaf15	1.4998E-06	0.67911167	0.102	0.084	0.03290384
Spsb3	1.5312E-06	0.75760199	0.083	0.067	0.03359356
Abtb1	1.673E-06	0.75572907	0.062	0.047	0.03670461
1700020I14Ril	1.7143E-06	0.43771916	0.218	0.202	0.03760953
Igf2r	1.7423E-06	0.59109577	0.128	0.109	0.03822404
Prdm10	1.7796E-06	0.84334213	0.046	0.033	0.03904326
Tbl1x	1.7887E-06	0.55813034	0.199	0.184	0.03924169
Cd101	1.8449E-06	0.96958516	0.032	0.021	0.04047427
Adora2a	1.9036E-06	0.54367926	0.182	0.165	0.04176231
Sfpq	2.1036E-06	0.26302281	0.515	0.545	0.04615031
Arnt2	2.1612E-06	1.20107998	0.013	0.007	0.04741367
Rtn4rl1	2.3058E-06	0.98784695	0.018	0.01	0.05058666
Rabgap1l	2.3986E-06	0.39375352	0.205	0.186	0.05262383
Wdr12	2.4141E-06	0.81107352	0.048	0.035	0.05296215
Pdlim1	2.4588E-06	0.54322681	0.133	0.114	0.05394267
Crebzf	2.6245E-06	0.58876451	0.13	0.112	0.05757871
Mbnl3	2.8998E-06	0.75095783	0.057	0.043	0.06361775
Fip1l1	2.9201E-06	0.42469427	0.294	0.288	0.0640631
Fopnl	2.9243E-06	0.57629333	0.095	0.078	0.06415719
Rchy1	3.211E-06	0.4614942	0.209	0.194	0.07044572
Kif5c	3.2682E-06	1.42376664	0.014	0.008	0.071702
1110012L19Ril	3.3302E-06	0.90931982	0.047	0.034	0.07306131
Zfp212	3.3572E-06	0.72098531	0.052	0.039	0.07365359
Rab27a	3.5559E-06	0.69936556	0.061	0.047	0.0780128
Scand1	3.6171E-06	0.29745901	0.456	0.48	0.07935515
Agfg1	4.0126E-06	0.5343501	0.2	0.186	0.08803301
Asf1a	4.4302E-06	0.65071073	0.079	0.063	0.09719377
Kansl1	4.5822E-06	0.34437265	0.311	0.305	0.10052857
Eif3m	4.7641E-06	0.30969077	0.413	0.432	0.10451856
Pecam1	5.1845E-06	0.4808654	0.159	0.14	0.11374169
Gm39323	5.2637E-06	1.43494951	0.01	0.005	0.11548135
Tmed2	5.2794E-06	0.32032196	0.42	0.441	0.11582468
Ezh1	5.3784E-06	0.51684251	0.104	0.087	0.11799618
P2rx7	5.3906E-06	0.88361321	0.062	0.048	0.11826412
Pfkl	5.6227E-06	0.81439218	0.094	0.079	0.12335694
Tnfaip8l2	5.7849E-06	0.82237189	0.085	0.069	0.12691556
Rab11fip4	5.8755E-06	0.98332195	0.031	0.021	0.12890293
Chd6	6.5992E-06	0.39198574	0.18	0.161	0.14477977
Etaa1os	7.0191E-06	1.28671444	0.014	0.008	0.15399111
Tbc1d10c	7.7785E-06	0.44848723	0.084	0.067	0.17065281
Gm16286	7.9612E-06	0.45520564	0.265	0.26	0.17466126

Sms	8.2407E-06	0.46182266	0.229	0.216	0.18079353
Kat6b	8.2664E-06	0.60162587	0.124	0.107	0.18135624
Hist2h2bb	8.6729E-06	0.93088185	0.034	0.023	0.19027481
Cdk11b	8.743E-06	0.33504095	0.342	0.343	0.19181245
Alkbh5	8.981E-06	0.50092354	0.231	0.223	0.19703376
Mrpl18	9.1037E-06	0.55455754	0.18	0.166	0.19972553
Anxa6	9.547E-06	0.42598111	0.262	0.255	0.20945116
Donson	1.0135E-05	0.60484422	0.097	0.081	0.22236019
Gpn3	1.0499E-05	0.62451787	0.091	0.075	0.2303316
Ociad1	1.074E-05	0.48412475	0.245	0.238	0.23562654
Jpt1	1.1743E-05	0.33076141	0.375	0.387	0.25763466
Usp48	1.355E-05	0.61113598	0.11	0.094	0.29726968
Pdxp	1.3828E-05	1.31288493	0.013	0.007	0.30336505
Cnnm3	1.527E-05	0.64909642	0.043	0.031	0.33501646
Acbd5	1.5506E-05	0.54566352	0.18	0.165	0.34018029
Rest	1.6915E-05	0.4771949	0.249	0.241	0.37108811
Cep57	1.7283E-05	0.57018279	0.111	0.094	0.37917663
G3bp2	1.7777E-05	0.39080446	0.265	0.256	0.39000283
Nop56	1.7778E-05	0.43202727	0.218	0.204	0.39003602
Rsrp1	1.7867E-05	0.33163126	0.299	0.293	0.39198252
Rictor	1.7918E-05	0.31164223	0.327	0.322	0.39309535
Dhx36	1.8014E-05	0.42359159	0.248	0.238	0.39520218
Cdkn2aip	1.807E-05	0.59185184	0.098	0.082	0.39644704
Dubr	1.9678E-05	0.78449413	0.057	0.044	0.43171053
Olfr1369-ps1	2.2415E-05	1.01567632	0.03	0.021	0.49175457
Gpsm3	2.2579E-05	0.50473846	0.234	0.227	0.49536547
Mkrn2	2.306E-05	0.72975912	0.058	0.046	0.50592245
Stat5a	2.3238E-05	0.43870472	0.199	0.184	0.50980794
Uqcc3	2.3463E-05	0.55035545	0.165	0.151	0.51475434
Rsbn1	2.3538E-05	0.46371723	0.17	0.154	0.51639035
Cdc25b	2.3563E-05	0.68778764	0.036	0.025	0.51694961
Hexim1	2.3583E-05	0.50634329	0.193	0.179	0.5173877
Ms4a6b	2.4195E-05	0.26445301	0.267	0.255	0.53081556
Trim24	2.5029E-05	0.51995467	0.1	0.084	0.54910668
Adipor2	2.5494E-05	0.48430373	0.212	0.201	0.55930511
Klc3	2.5712E-05	1.36370692	0.01	0.005	0.56409478
Prex1	2.6679E-05	0.49534318	0.241	0.234	0.5853188
Eif3j2	2.7203E-05	0.27066894	0.077	0.101	0.5968094
Ppil4	2.7378E-05	0.50416997	0.171	0.156	0.60064453
Pdcd5	2.8791E-05	0.44697001	0.302	0.305	0.63163905
Rif1	2.9591E-05	0.39915442	0.15	0.133	0.64920368
Wdr45	3.06E-05	0.7163723	0.052	0.04	0.67133517
Cdc40	3.0641E-05	0.40411354	0.251	0.243	0.67223722
Gpbp1	3.1458E-05	0.29829358	0.363	0.37	0.69015873
Stk32c	3.3074E-05	1.12395046	0.03	0.021	0.72561296
Ttll12	3.3922E-05	1.09625924	0.023	0.015	0.74422332
Tmem263	3.4222E-05	0.61254222	0.059	0.047	0.7507992
Plpp6	3.645E-05	0.8871528	0.037	0.027	0.7996815

Nav2	3.6568E-05	0.34542447	0.064	0.05	0.80227406
Ddx42	3.6728E-05	0.44159409	0.254	0.249	0.80578276
Ppp2r2d	3.698E-05	0.52915746	0.222	0.216	0.81129834
Arid4a	3.7174E-05	0.43870862	0.219	0.208	0.81556469
Ndufa5	3.7485E-05	0.53035026	0.226	0.221	0.82237248
Ccnd3	4.0594E-05	0.62832716	0.121	0.106	0.8905853
Yes1	4.0854E-05	0.71018385	0.032	0.022	0.8963052
Elof1	4.2996E-05	0.27409124	0.083	0.107	0.94328741
Ttbk2	4.412E-05	0.82998725	0.028	0.019	0.96795732
Mphosph6	4.4508E-05	0.7032539	0.113	0.099	0.9764648
Mrps16	5.0688E-05	0.52096547	0.231	0.226	1
Thada	5.1236E-05	0.77541683	0.053	0.041	1
AC163032.1	5.2888E-05	0.72382628	0.031	0.022	1
Meis3	5.3786E-05	1.00236012	0.02	0.013	1
Izumo4	5.3876E-05	0.94775221	0.027	0.018	1
Armcx5	5.5301E-05	0.59865743	0.069	0.056	1
Rlf	5.5991E-05	0.3687755	0.244	0.236	1
Rab8a	5.7764E-05	0.44433133	0.256	0.253	1
Sugt1	5.7972E-05	0.49808103	0.251	0.249	1
Gxylt1	5.8957E-05	0.62358252	0.148	0.134	1
Ranbp6	6.0121E-05	0.72448823	0.036	0.027	1
Insl6	6.028E-05	0.80819392	0.06	0.048	1
Rb1cc1	6.2414E-05	0.34327591	0.266	0.258	1
Sept9	6.2446E-05	0.5690652	0.177	0.166	1
E030030I06Ril	6.2579E-05	0.66599825	0.025	0.017	1
Gm26799	6.3455E-05	0.89946681	0.017	0.011	1
Mrps24	6.4101E-05	0.53632606	0.227	0.224	1
Cep192	6.4203E-05	0.48814684	0.091	0.076	1
4732440D04R	6.47E-05	0.66981845	0.052	0.041	1
Narf	6.4924E-05	0.6345725	0.069	0.056	1
Rcan3	6.4975E-05	1.10441658	0.024	0.017	1
Gdf11	6.5756E-05	0.79439992	0.018	0.011	1
Slamf6	6.6844E-05	0.54381213	0.104	0.089	1
Sbno1	6.7533E-05	0.32769184	0.348	0.355	1
Atn1	6.9113E-05	0.6738439	0.061	0.049	1
Vps26b	7.1815E-05	0.82703986	0.054	0.042	1
Aff1	7.3395E-05	0.33816892	0.334	0.337	1
Yaf2	7.475E-05	0.63731339	0.085	0.071	1
Neu3	7.4951E-05	0.75691855	0.026	0.018	1
Card6	7.5088E-05	0.37631387	0.062	0.049	1
Usp3	7.9352E-05	0.50920013	0.194	0.183	1
Gpatch11	7.9366E-05	0.70478517	0.068	0.056	1
Ndc80	8.4566E-05	0.99267196	0.02	0.013	1
Gcc2	8.6966E-05	0.50095479	0.137	0.123	1
Sap30l	8.7424E-05	0.67019179	0.114	0.101	1
Elavl1	9.0004E-05	0.4606889	0.235	0.229	1
4930503L19Ri	9.6447E-05	0.61317044	0.094	0.08	1
Acpp	9.8137E-05	0.47420303	0.045	0.034	1

4921524J17Ril	9.8141E-05	0.45840242	0.12	0.105	1
Siah1a	0.0001025	0.69781139	0.062	0.05	1
Raly	0.00010476	0.35200735	0.335	0.345	1
Lsm10	0.00010485	0.29069928	0.047	0.063	1
Azi2	0.00011067	0.44652115	0.189	0.178	1
Zfp362	0.00011257	0.76280424	0.046	0.035	1
Gnptg	0.00011738	0.59545501	0.074	0.061	1
Sars	0.00012489	0.40668922	0.25	0.244	1
Wbp1	0.00013151	0.80944017	0.062	0.05	1
Rasgrp2	0.00013208	0.35988223	0.123	0.107	1
Herc1	0.00013439	0.27222122	0.256	0.245	1
Srp19	0.00013467	0.46697085	0.248	0.246	1
Zer1	0.00013761	0.51573769	0.056	0.044	1
Cysltr2	0.00013854	0.51931411	0.094	0.08	1
Gars	0.00014088	0.62840481	0.149	0.137	1
Gabbr1	0.000145	0.69052696	0.073	0.061	1
Msi2	0.00014894	0.25659872	0.237	0.222	1
Thap3	0.00014975	0.57247051	0.074	0.061	1
Eva1b	0.00017087	0.25193877	0.076	0.097	1
Chrne	0.00017216	0.7232273	0.036	0.027	1
Rab3gap1	0.00017801	0.44180298	0.181	0.169	1
Cers4	0.00017804	0.41427141	0.117	0.102	1
Kif23	0.00017825	0.44109454	0.067	0.055	1
Arhgap1	0.00018004	0.72110691	0.082	0.07	1
Cipc	0.00018244	0.49714291	0.112	0.098	1
Tmem97	0.00020009	0.9627761	0.039	0.029	1
Zfp287	0.00020494	0.89543659	0.016	0.01	1
Naa20	0.00020498	0.71021826	0.116	0.104	1
Pglyrp2	0.00021728	0.80514593	0.017	0.011	1
Kcnk7	0.0002338	1.13159625	0.011	0.006	1
Pitpnc1	0.0002506	0.40318148	0.18	0.168	1
Perp	0.00025298	1.07361987	0.014	0.009	1
Gm14305	0.00025709	1.14455768	0.018	0.012	1
Armc7	0.0002587	0.64452925	0.041	0.032	1
Slc35d1	0.00026037	0.69533583	0.051	0.041	1
Iqgap2	0.0002681	0.6311825	0.155	0.146	1
Mast4	0.00027023	0.31254366	0.192	0.176	1
Ncald	0.00027442	0.67283156	0.046	0.036	1
Rdx	0.00027923	0.3604164	0.28	0.282	1
Sla2	0.00029304	0.59067039	0.052	0.041	1
Zfpm1	0.00029531	0.6705894	0.045	0.036	1
Ccsap	0.00029784	0.78036291	0.027	0.019	1
Baiap2	0.00030309	0.63852785	0.15	0.14	1
Aplp1	0.00030929	1.03201321	0.015	0.01	1
Maml2	0.00031619	0.64309458	0.046	0.036	1
Cd84	0.00031629	0.40115977	0.213	0.205	1
Pacs1	0.00031931	0.6114758	0.069	0.058	1
Brd9	0.00032404	0.51599719	0.177	0.168	1

Abhd18	0.00033754	0.75135173	0.039	0.03	1
Mfap1a	0.00034031	0.51577252	0.097	0.084	1
Nr1d1	0.00036958	0.69539101	0.035	0.026	1
Ssbp2	0.00037168	0.59268098	0.06	0.049	1
Brap	0.00037525	0.53276278	0.146	0.135	1
Exosc8	0.00038089	0.62973219	0.122	0.11	1
Xkr6	0.00038123	0.89578989	0.014	0.009	1
Cdc37	0.00038378	0.36836848	0.335	0.35	1
Cdon	0.00039121	0.96159948	0.026	0.018	1
Synj2	0.00039564	0.88692138	0.047	0.037	1
Ank	0.00039893	0.25145402	0.039	0.052	1
Nars	0.00040711	0.40807885	0.339	0.354	1
Slc39a8	0.00041486	1.40849714	0.01	0.006	1
Nsmce4a	0.00041963	0.42324551	0.182	0.172	1
Taf5	0.00043568	0.70056942	0.047	0.037	1
Per1	0.00044714	0.36180342	0.234	0.227	1
Mctp2	0.00045538	0.44985132	0.056	0.045	1
Pde5a	0.000464	0.46874146	0.039	0.03	1
Mrpl57	0.00046458	0.49765626	0.221	0.219	1
Vamp1	0.00047687	0.77135339	0.03	0.022	1
Cyb5a	0.00047982	0.44464536	0.212	0.207	1
Naa30	0.00048223	0.55286355	0.082	0.07	1
Vamp4	0.00048479	0.51546907	0.197	0.191	1
Sf3b6	0.00048811	0.26987793	0.392	0.417	1
Pola2	0.00049075	0.29335943	0.013	0.02	1
Sidt1	0.00049682	0.394801	0.036	0.028	1
Ccdc117	0.00050587	0.48614967	0.07	0.058	1
Ube2o	0.00053807	0.70958887	0.052	0.042	1
Nkap	0.00056877	0.55265149	0.121	0.109	1
Gm10036	0.00057652	0.38717531	0.281	0.284	1
Ccno	0.00058148	0.97173989	0.012	0.008	1
Rhno1	0.00058856	0.64265238	0.057	0.046	1
Prc1	0.0005907	0.58426644	0.043	0.033	1
Deaf1	0.0006016	0.948936	0.02	0.014	1
Zfp266	0.0006396	0.48673708	0.102	0.09	1
Zfp799	0.00065286	0.95605167	0.017	0.012	1
Setd3	0.00069498	0.55734236	0.15	0.14	1
Atg12	0.00069798	0.49548899	0.168	0.158	1
Cops2	0.00069876	0.25010908	0.127	0.155	1
Cks2	0.00069914	0.34738295	0.171	0.161	1
Nbdy	0.00070104	0.2988032	0.057	0.073	1
Trir	0.00073261	0.32534415	0.34	0.354	1
Cdc37l1	0.00076545	0.34136261	0.196	0.185	1
Dnajc1	0.00077078	0.46036682	0.205	0.2	1
Irf2bpl	0.00077276	0.48566294	0.105	0.093	1
Fam92a	0.00078964	0.66225669	0.051	0.041	1
Ddhd2	0.00081203	0.63556799	0.087	0.076	1
Rnf167	0.00082236	0.48310842	0.119	0.107	1

Kpna1	0.00083241	0.35647239	0.299	0.303	1
Rpusd4	0.00084527	0.70724794	0.051	0.042	1
Rassf5	0.00085343	0.41082164	0.15	0.138	1
Dpm1	0.00087353	0.55041649	0.12	0.108	1
Dcun1d4	0.00087738	0.67523825	0.029	0.022	1
Fam78a	0.00088393	0.6048962	0.029	0.022	1
Wbp11	0.00089222	0.34397024	0.308	0.316	1
Mrpl4	0.0008937	0.27541673	0.09	0.112	1
Slc24a5	0.00091921	0.98166224	0.023	0.016	1
Fam216a	0.00094202	0.7188104	0.039	0.031	1
Lnpk	0.00096221	0.45723356	0.096	0.083	1
Eno1	0.00096779	0.46376645	0.234	0.234	1
Cbx8	0.00100557	0.89680593	0.018	0.012	1
Nfatc1	0.00102451	0.4177418	0.185	0.174	1
Hbb-bs	0.00103235	0.94502466	0.057	0.072	1
Snrnp48	0.00103528	0.41386638	0.205	0.197	1
2310035C23R	0.00103797	0.43774174	0.143	0.132	1
Lmbr1l	0.00103849	0.52058339	0.071	0.06	1
Gm17745	0.00105646	0.65019592	0.037	0.029	1
Vta1	0.00105727	0.29583285	0.076	0.095	1
Leng1	0.00109307	0.70728727	0.067	0.057	1
Tmem192	0.00113538	0.26176621	0.069	0.086	1
Gpr155	0.00116413	1.0263712	0.018	0.012	1
Hagh	0.00117751	0.26932452	0.042	0.055	1
Casp3	0.00119312	0.59248121	0.12	0.109	1
Rbm22	0.00124421	0.40752287	0.229	0.227	1
Rab23	0.00126777	0.86219686	0.014	0.009	1
E130311K13R	0.00129166	0.87396335	0.018	0.012	1
Psip1	0.00131703	0.38148623	0.157	0.145	1
Ankrd12	0.00142489	0.28571943	0.281	0.276	1
Rasal3	0.00145744	0.42299225	0.079	0.068	1
Adk	0.00147373	0.64137537	0.073	0.063	1
Rab19	0.00149573	0.65315761	0.042	0.034	1
Zmym1	0.00152005	0.62759811	0.044	0.035	1
Smim15	0.0015629	0.485488	0.153	0.144	1
Mbtps1	0.00156977	0.4445437	0.143	0.132	1
Itga3	0.00157732	0.94014819	0.02	0.014	1
Rprd2	0.00161992	0.46104398	0.128	0.117	1
Tbc1d9b	0.00169222	0.81808359	0.056	0.047	1
Ccdc92	0.0017178	1.05445301	0.012	0.008	1
Acss2	0.00172659	0.60270809	0.033	0.025	1
Plekha3	0.0017456	0.57185064	0.094	0.083	1
Cox11	0.00181378	0.88645727	0.039	0.031	1
Gm26670	0.00184894	0.91019107	0.014	0.01	1
Pcif1	0.00185606	0.43103228	0.234	0.234	1
Chfr	0.00193304	0.41006204	0.15	0.14	1
Zfp280b	0.00197484	0.69726803	0.026	0.02	1
Sec24d	0.00198189	0.25312215	0.041	0.053	1

Npat	0.00199583	0.50834899	0.062	0.052	1
Tgif1	0.00200248	0.25768444	0.36	0.369	1
Zfp775	0.00202702	0.84232827	0.011	0.007	1
Cdk6	0.00204639	0.60220574	0.135	0.126	1
Espn	0.00206946	0.61650526	0.029	0.022	1
Pdcd6	0.00208548	0.44054643	0.263	0.271	1
Alad	0.00208927	0.25619863	0.018	0.025	1
Zkscan14	0.00211089	0.58999954	0.056	0.047	1
Supt5	0.00216445	0.37284922	0.267	0.27	1
Xbp1	0.00220327	0.27216761	0.37	0.388	1
Uba5	0.00220522	0.26853937	0.08	0.098	1
Hid1	0.00220647	0.75100486	0.015	0.01	1
Enpp1	0.00225759	0.77150547	0.037	0.03	1
Tasp1	0.00226234	0.61701675	0.036	0.028	1
Xpa	0.00228186	0.65749337	0.116	0.107	1
Socs2	0.00240895	0.37409644	0.098	0.085	1
Vps28	0.00241964	0.38188138	0.261	0.266	1
Grk6	0.00248115	0.25850492	0.146	0.134	1
Las1l	0.00256959	0.38368775	0.121	0.11	1
Tstd3	0.00266444	0.69486353	0.039	0.031	1
Fam204a	0.00272313	0.36129785	0.226	0.224	1
Tmem201	0.00281405	0.88319944	0.017	0.012	1
Use1	0.00283518	0.25180731	0.415	0.445	1
Zgpat	0.00285309	0.61494245	0.047	0.039	1
Dap	0.00285914	0.57622477	0.152	0.146	1
Psmc5	0.00287958	0.26623114	0.145	0.174	1
Zfp53	0.00293077	0.49015755	0.046	0.038	1
Dph7	0.00300114	0.87552515	0.017	0.012	1
Kif5b	0.00302927	0.27121623	0.4	0.428	1
Ndufb11	0.00307495	0.25519527	0.427	0.458	1
Asxl2	0.00312108	0.37700734	0.182	0.173	1
Egln1	0.00313413	0.61872704	0.111	0.102	1
Tox2	0.00318101	0.42071224	0.05	0.041	1
Gab3	0.00318421	0.60699986	0.022	0.016	1
Ep400	0.0031944	0.43913686	0.206	0.203	1
Mrpl41	0.00322755	0.56828307	0.11	0.101	1
Eif4ebp2	0.00325128	0.38598057	0.184	0.177	1
Fam98b	0.00326455	0.55072238	0.163	0.157	1
F2rl1	0.00340921	1.00728258	0.012	0.008	1
4930453N24R	0.00342173	0.54000272	0.105	0.096	1
Abhd10	0.0036088	0.64889008	0.05	0.041	1
Whamm	0.00361947	0.60281707	0.081	0.072	1
Actr1a	0.00366098	0.25168707	0.079	0.096	1
Cab39	0.00371287	0.29414849	0.284	0.288	1
Elmo1	0.0037898	0.31537335	0.292	0.298	1
Nlrc3	0.00379167	0.37920464	0.051	0.043	1
Vamp7	0.00382572	0.2743164	0.047	0.059	1
Zfp68	0.00388477	0.55314163	0.081	0.071	1

Zfp574	0.00389672	0.59575961	0.036	0.029	1
Dnajc9	0.00396062	0.30718966	0.125	0.115	1
Nans	0.00397442	0.28959306	0.079	0.096	1
Unkl	0.00403611	0.56144645	0.039	0.031	1
Pno1	0.00403622	0.28266688	0.059	0.073	1
Zfp746	0.00407405	0.47773688	0.092	0.082	1
Apol7e	0.00408637	0.56455028	0.027	0.021	1
Exosc4	0.00412989	0.63312646	0.064	0.055	1
Ppp1r8	0.00416517	0.55966252	0.052	0.044	1
Ccdc127	0.00417279	0.59831267	0.098	0.089	1
Nt5dc1	0.00425531	0.59076257	0.046	0.038	1
Uqcc2	0.00435308	0.27542044	0.172	0.206	1
Adnp	0.00448823	0.71784378	0.039	0.032	1
Sgms1	0.00449731	0.35673076	0.145	0.134	1
U2af1	0.0045874	0.41566916	0.287	0.301	1
Snapc5	0.00459989	0.32330848	0.101	0.122	1
4932422M17F	0.00460568	0.69686875	0.024	0.018	1
Mrps12	0.00462474	0.26878832	0.092	0.111	1
Slc35g1	0.0046636	0.80357106	0.015	0.011	1
Irf2bp1	0.00467776	0.63128044	0.04	0.033	1
Orc4	0.00467985	0.50196526	0.107	0.097	1
Plcb4	0.00469349	0.51189576	0.051	0.042	1
Usp15	0.00472599	0.40931264	0.235	0.236	1
Mrpl44	0.00474166	0.63647368	0.048	0.04	1
Mrpl15	0.00474181	0.27215213	0.105	0.126	1
Ranbp10	0.00489381	0.54636304	0.068	0.059	1
Usp36	0.00496194	0.41932572	0.101	0.091	1
Abcb9	0.00498635	0.51346222	0.036	0.029	1
Zfp141	0.00499761	0.83500158	0.018	0.013	1
Alkbh3	0.00512166	0.25326084	0.03	0.039	1
Dbr1	0.00546064	0.66249648	0.052	0.044	1
A830010M20F	0.00546961	0.92400091	0.01	0.006	1
Hipk2	0.00550556	0.31207119	0.088	0.106	1
Slc35b3	0.00553262	0.56879374	0.065	0.056	1
Ston2	0.00555364	0.32875511	0.053	0.065	1
Thoc7	0.00562123	0.46554013	0.204	0.202	1
Pik3ca	0.00563029	0.42140631	0.111	0.101	1
Lrp8	0.00564382	0.29971324	0.01	0.015	1
Dusp12	0.00564831	0.76068051	0.035	0.028	1
Bola2	0.00568821	0.27746107	0.384	0.412	1
Blmh	0.00570106	0.25345891	0.107	0.128	1
Mrpl32	0.00580335	0.4360582	0.227	0.231	1
Hist2h4	0.00589173	0.73062605	0.02	0.015	1
Terf2ip	0.00607257	0.61762548	0.045	0.038	1
Mcm6	0.00618431	0.5284417	0.104	0.095	1
Snip1	0.00641063	0.59189524	0.082	0.073	1
Zmym5	0.00662103	0.35367479	0.168	0.159	1
Gpr55	0.00668396	0.63146994	0.086	0.077	1

Nckap1	0.00670015	0.80703991	0.015	0.01	1
Atxn2l	0.00686192	0.43154993	0.222	0.223	1
Gm527	0.00708729	0.81802446	0.016	0.012	1
Asns	0.00721642	0.70707203	0.06	0.052	1
Tpgs1	0.00725786	0.26518174	0.039	0.049	1
1110004F10Ri	0.00734986	0.33711686	0.303	0.316	1
Gadd45gip1	0.00736139	0.50287541	0.151	0.146	1
Mrpl19	0.00738434	0.25091885	0.074	0.089	1
Kmt2b	0.00743029	0.25077258	0.084	0.1	1
R3hdm4	0.0074412	0.4802314	0.149	0.143	1
Kctd10	0.00753035	0.49258253	0.099	0.09	1
Rbm27	0.00756049	0.42353536	0.228	0.228	1
Rab40c	0.0076789	0.72663963	0.026	0.021	1
Me2	0.00777196	0.57376111	0.101	0.093	1
Eea1	0.00777203	0.31708953	0.199	0.234	1
Fbxo34	0.00780667	0.27654964	0.085	0.101	1
Tdp2	0.00801608	0.4737818	0.079	0.07	1
Ufd1	0.00805983	0.29596671	0.081	0.097	1
Nr2c2	0.0082554	0.35375941	0.171	0.164	1
Pex16	0.00862285	0.61193527	0.086	0.078	1
Hscb	0.00862667	0.32917206	0.069	0.083	1
Ngrn	0.00886444	0.62269234	0.042	0.035	1
Mtor	0.00900401	0.35670725	0.051	0.062	1
Akirin2	0.00904606	0.46618139	0.172	0.169	1
Ahdc1	0.00912475	0.51154637	0.019	0.014	1
Sucla2	0.00913781	0.28819734	0.066	0.08	1
Thyn1	0.00951584	0.35673759	0.028	0.036	1
Rbm43	0.00972293	0.58995716	0.044	0.037	1
Phf3	0.0098843	0.2696418	0.342	0.357	1
Birc6	0.00996762	0.26135745	0.347	0.364	1
Zscan2	0.01028158	0.75836776	0.015	0.011	1
Pms2	0.01029468	0.53501751	0.04	0.033	1
Sin3b	0.01038064	0.34533837	0.314	0.334	1
Ing2	0.01041835	0.37655141	0.156	0.15	1
Kcnk5	0.01048382	0.60900512	0.016	0.012	1
Gm30054	0.01048436	0.48571876	0.036	0.03	1
Prdm4	0.01055277	0.26560097	0.038	0.048	1
Mzt2	0.01071069	0.27080714	0.02	0.026	1
Ssbp3	0.01084495	0.44450151	0.119	0.111	1
Ccnc	0.01097944	0.60148985	0.068	0.06	1
Spock2	0.01100508	0.69712759	0.022	0.017	1
Atg13	0.01105009	0.62298695	0.063	0.056	1
Btg3	0.01111818	0.57145279	0.065	0.058	1
Rorc	0.01121524	0.66417175	0.018	0.014	1
Pttg1	0.01127587	0.39071155	0.134	0.126	1
Zbtb11os1	0.01141664	0.85561017	0.019	0.015	1
Eid2	0.01145276	1.06628002	0.01	0.007	1
Suco	0.01161223	0.33894274	0.26	0.264	1

Zfp956	0.01171122	0.70101911	0.016	0.012	1
Eapp	0.01196659	0.31848309	0.233	0.234	1
Golm1	0.01207739	0.63141925	0.041	0.034	1
Fgfr1op2	0.01215173	0.31884865	0.295	0.309	1
Rpap3	0.01228612	0.56099414	0.049	0.042	1
Wipi2	0.01266785	0.47823352	0.117	0.11	1
Dock9	0.01267126	0.47898251	0.022	0.017	1
Rpp30	0.01281891	0.2909737	0.029	0.036	1
Sf3b5	0.01297102	0.32716421	0.303	0.32	1
Idnk	0.01321322	0.42708538	0.18	0.178	1
Rfxap	0.0133051	0.67784931	0.105	0.099	1
Eri1	0.01331439	0.26218403	0.126	0.147	1
Gemin7	0.01365514	0.53215856	0.12	0.113	1
Mtbp	0.01372399	0.70739358	0.019	0.015	1
Il23r	0.01393339	0.52910174	0.023	0.018	1
Ccs	0.01426537	0.30084517	0.045	0.055	1
Cxxc1	0.0145233	0.50951173	0.074	0.066	1
Zfp382	0.01463234	0.86103879	0.021	0.016	1
Borcs8	0.01465252	0.35687242	0.111	0.131	1
Nipal1	0.01482739	0.91271534	0.014	0.01	1
Clec2i	0.01517179	0.26054627	0.079	0.07	1
Snrnp25	0.01542594	0.33587636	0.049	0.06	1
Ccpg1	0.01551378	0.5049239	0.065	0.057	1
Lyrm9	0.01562681	0.65370921	0.034	0.028	1
Rnf220	0.01579697	0.41139439	0.133	0.126	1
Fbxw7	0.01589294	0.55447948	0.084	0.077	1
Zxdb	0.01662307	0.50191896	0.046	0.039	1
Usp1	0.01692504	0.48808196	0.131	0.125	1
Xlr4a	0.01722422	0.58740621	0.035	0.029	1
Pola1	0.01756799	0.67069962	0.048	0.041	1
Gzmk	0.01762129	0.5094965	0.019	0.014	1
Cstad	0.01762181	0.89351684	0.01	0.007	1
Tspan3	0.01764871	0.32579162	0.084	0.098	1
Golt1b	0.01785041	0.28595547	0.085	0.1	1
Ecsit	0.01855272	0.52925755	0.04	0.034	1
Frat1	0.0186881	0.71057084	0.025	0.02	1
Ubxn4	0.01871129	0.2532511	0.362	0.387	1
Afg3l1	0.01872695	0.27126824	0.08	0.094	1
Hjurp	0.01874412	0.43962705	0.092	0.084	1
Slc48a1	0.01884993	0.32730248	0.048	0.058	1
Glcci1	0.01893754	0.55752055	0.032	0.026	1
Gm9856	0.01897771	0.52849554	0.033	0.028	1
Ptpn3	0.01905573	0.79857038	0.025	0.02	1
Plpp5	0.01930906	0.34819878	0.028	0.035	1
Dap3	0.01955028	0.26863434	0.08	0.094	1
Dok2	0.01972903	0.29162483	0.073	0.086	1
Usp38	0.01984733	0.38265994	0.143	0.137	1
Mrps11	0.02017732	0.28800978	0.04	0.049	1

Tarsl2	0.02032562	1.01023525	0.012	0.009	1
Dpy30	0.0204156	0.32846586	0.073	0.086	1
Mcph1	0.0204521	0.86377834	0.032	0.027	1
Ppp2r5a	0.02067364	0.327671	0.278	0.289	1
Ccnl2	0.02078613	0.29821213	0.29	0.301	1
Apool	0.02088885	0.28061554	0.027	0.034	1
Spata2	0.02130975	0.25631638	0.064	0.076	1
1700097N02R	0.02131469	0.71423167	0.018	0.014	1
Emc4	0.02146199	0.41682298	0.079	0.093	1
Ttc5	0.02158693	0.49542741	0.074	0.067	1
Erich1	0.021784	0.60223657	0.062	0.056	1
Rpl39l	0.02187205	0.55995096	0.01	0.007	1
Tbl2	0.02238228	0.68663285	0.019	0.015	1
Helb	0.02247639	0.54496383	0.065	0.058	1
Tbc1d10b	0.0226595	0.59709585	0.079	0.072	1
Elac1	0.02274721	0.25057066	0.01	0.015	1
1700056E22R	0.02278923	0.66014596	0.02	0.016	1
Tbc1d22b	0.02280126	0.68914435	0.024	0.02	1
Zfp931	0.02297664	0.8465049	0.022	0.017	1
Il12rb1	0.02320752	0.64280447	0.025	0.02	1
1810032O08R	0.02329879	0.61455586	0.064	0.058	1
Txnl1	0.02332505	0.26023913	0.203	0.239	1
Gm11110	0.02387865	1.03192687	0.01	0.007	1
Gm32036	0.02390536	0.69453466	0.02	0.016	1
Shkbp1	0.02395668	0.298383	0.048	0.057	1
F8a	0.02396697	0.62000597	0.03	0.025	1
Tor1b	0.02411458	0.2626845	0.071	0.084	1
Tle4	0.02413788	0.31751389	0.238	0.239	1
Psma2	0.02417184	0.26339987	0.383	0.417	1
Pcmt1	0.02455579	0.25628436	0.148	0.171	1
Carf	0.02467076	0.4128533	0.019	0.015	1
Dnajc18	0.02496481	0.79883693	0.03	0.025	1
E230001N04R	0.02502548	0.45065391	0.014	0.011	1
Nfrkb	0.02508643	0.35078324	0.088	0.08	1
Ppfia1	0.02514881	0.40575303	0.141	0.136	1
Gm42722	0.02532875	0.43715111	0.014	0.01	1
Ino80	0.02546683	0.25728016	0.084	0.097	1
Asb8	0.02559508	0.25507165	0.028	0.035	1
Trp53	0.02570744	0.36579252	0.17	0.166	1
Slc16a1	0.02594269	0.25709187	0.044	0.053	1
Pink1	0.02594892	0.4521275	0.082	0.075	1
Mical1	0.02605364	0.26024821	0.115	0.133	1
Cdan1	0.02631736	0.5690576	0.024	0.019	1
Farp1	0.02661501	0.60545803	0.018	0.014	1
Tufm	0.02700386	0.27860281	0.046	0.055	1
Trim23	0.02732406	0.63000925	0.031	0.026	1
Hba-a1	0.02735732	1.04123576	0.019	0.025	1
Cbx7	0.02761407	0.28903969	0.054	0.047	1

Tbcc	0.02770607	0.26682868	0.033	0.041	1
Hacd3	0.02779764	0.32689651	0.116	0.108	1
Pptc7	0.02804676	0.53983743	0.103	0.097	1
Slc25a17	0.0281376	0.28275239	0.045	0.054	1
Mcm4	0.02857791	0.46691654	0.104	0.097	1
Zbtb49	0.0286034	0.73363923	0.016	0.013	1
Rnf14	0.02938401	0.59394573	0.073	0.067	1
Snrnp27	0.03001367	0.27052098	0.171	0.199	1
Ubxn6	0.03031435	0.31114664	0.106	0.122	1
Fmc1	0.03063619	0.29187925	0.074	0.087	1
Fpgt	0.03108587	0.68508831	0.033	0.028	1
Lancl2	0.03121446	0.28570666	0.016	0.021	1
Adrm1	0.03145271	0.32909776	0.114	0.133	1
Sh3bp5l	0.03150896	0.63281305	0.024	0.019	1
Nrbf2	0.03212129	0.27616055	0.094	0.109	1
Ezh2	0.03230401	0.65104111	0.098	0.093	1
Nt5c	0.03245774	0.29901229	0.155	0.181	1
Brd8	0.03252741	0.29332579	0.211	0.211	1
Prickle1	0.0328373	0.63733427	0.019	0.015	1
Jtb	0.03297201	0.32448538	0.151	0.175	1
Ing3	0.03331093	0.45332558	0.149	0.146	1
Ccnt1	0.03331187	0.27669403	0.274	0.282	1
Sephs1	0.03345256	0.2924734	0.017	0.022	1
Erc1	0.03364797	0.46373555	0.03	0.025	1
Lockd	0.03385326	0.52136821	0.046	0.04	1
Dguok	0.03387333	0.26737894	0.075	0.087	1
Rxra	0.03389978	0.4495359	0.014	0.019	1
Nup35	0.03415803	0.61201972	0.019	0.015	1
2310061I04Ril	0.03437341	0.521912	0.031	0.026	1
Tmem50a	0.03447974	0.27645405	0.344	0.366	1
2410004B18R	0.0352895	0.26589902	0.063	0.074	1
Foxj2	0.03543511	0.43230607	0.071	0.064	1
Suv39h2	0.03582638	0.81243742	0.016	0.013	1
Zfp329	0.03608678	0.47885695	0.058	0.051	1
Dnajc24	0.03620791	0.2723557	0.033	0.04	1
Cnot11	0.03631253	0.63582401	0.065	0.059	1
Simc1	0.03651833	0.57940935	0.025	0.021	1
Zfp809	0.0366082	0.63923578	0.038	0.032	1
Kxd1	0.03662635	0.3325306	0.153	0.178	1
Ubxn11	0.03677489	0.50744712	0.035	0.03	1
Hadh	0.03692741	0.27777865	0.051	0.06	1
Hace1	0.03736873	0.56110594	0.04	0.035	1
Csnk2b	0.03797765	0.34338179	0.28	0.297	1
Agk	0.03802228	0.56164657	0.04	0.035	1
Crls1	0.03824609	0.25866887	0.027	0.033	1
Atg5	0.03828486	0.28914275	0.078	0.09	1
Orc6	0.03846528	0.65877723	0.059	0.054	1
Susd3	0.03889246	0.27780334	0.05	0.059	1

Dtl	0.03901712	0.72151441	0.028	0.024	1
Bet1	0.03912512	0.30691211	0.071	0.083	1
Eloc	0.03934805	0.27336983	0.354	0.383	1
Dld	0.03979641	0.26441282	0.101	0.116	1
Ppil2	0.0398131	0.25455153	0.082	0.094	1
Tyk2	0.04011512	0.28781805	0.047	0.055	1
Ubl7	0.04020185	0.25859312	0.057	0.067	1
Gm20234	0.04086846	0.57241601	0.013	0.01	1
Ppm1h	0.04121437	0.52773003	0.092	0.087	1
Cd2bp2	0.04140899	0.47984211	0.124	0.12	1
Mzt1	0.04143804	0.54189839	0.094	0.089	1
Smad4	0.04173017	0.37543574	0.242	0.251	1
2610008E11R	0.04174712	0.5078799	0.033	0.028	1
Atg16l2	0.04176642	0.49445062	0.057	0.05	1
Pbxip1	0.04203505	0.32598394	0.195	0.194	1
Slc52a2	0.04212572	0.7571419	0.026	0.021	1
Alkbh7	0.04252426	0.28324846	0.034	0.041	1
H1f0	0.04262637	0.53141726	0.099	0.094	1
Diaph3	0.04299026	0.30463647	0.017	0.021	1
Elf4	0.04322354	0.47351404	0.101	0.096	1
B3gat2	0.04332377	0.68301711	0.018	0.014	1
Plaa	0.04338293	0.26983564	0.11	0.126	1
Tpd52l2	0.04357727	0.47135401	0.149	0.148	1
Lipo3	0.04419374	0.25606881	0.025	0.031	1
Gbp6	0.04429704	0.58627975	0.018	0.014	1
Msantd4	0.04441259	0.30834115	0.044	0.052	1
Smurf1	0.04511402	0.31987532	0.034	0.041	1
Map2k7	0.04513937	0.41560121	0.127	0.122	1
Zcchc8	0.04547994	0.61464247	0.068	0.062	1
Cbx3	0.04558635	0.27512421	0.277	0.285	1
Apopt1	0.04641195	0.30245737	0.075	0.086	1
Sltm	0.04725664	0.27871483	0.3	0.312	1
Kdm6a	0.0474845	0.37380402	0.175	0.173	1
Snhg6	0.0475439	0.28105768	0.177	0.204	1
Adprm	0.04792768	0.59910505	0.04	0.035	1
Ube2t	0.04850734	0.57331854	0.027	0.022	1
Atf6	0.04870979	0.25495075	0.157	0.18	1
Klhl11	0.04896526	0.68229635	0.019	0.016	1
Ahsa2	0.04897457	0.27585367	0.04	0.048	1
Cdk5	0.04920304	0.30914413	0.032	0.039	1
Zfp865	0.04992269	0.46772636	0.044	0.039	1
Zfp668	0.05049392	0.72973594	0.014	0.011	1
B3galt2	0.05077769	0.51313873	0.023	0.019	1
Smg6	0.05098023	0.31854795	0.14	0.135	1
Stau1	0.05118142	0.2527864	0.074	0.085	1
Zdhhc3	0.05190869	0.29705489	0.091	0.105	1
Dtd1	0.05202521	0.65974102	0.051	0.045	1
Rnf126	0.05210668	0.30520137	0.034	0.041	1

B4galnt1	0.0521848	0.32476411	0.208	0.209	1
2410131K14R	0.05246517	0.25697663	0.02	0.025	1
Pop5	0.05273421	0.3410946	0.122	0.117	1
Med17	0.05322344	0.28749356	0.043	0.051	1
Saysd1	0.05389262	0.25540925	0.027	0.033	1
Zfp235	0.05409155	0.67589383	0.011	0.008	1
Zfp664	0.05429629	0.53283168	0.07	0.064	1
Zfp46	0.054335	0.30639651	0.008	0.011	1
Ccdc25	0.05434552	0.48455959	0.108	0.103	1
Elk4	0.05478414	0.29196019	0.182	0.18	1
Aspscr1	0.05516892	0.29517881	0.043	0.051	1
Dgke	0.05580335	0.49741717	0.043	0.038	1
Fdft1	0.05629974	0.29459088	0.027	0.033	1
Slc35b1	0.05656345	0.32662369	0.113	0.13	1
Gm37387	0.05668663	0.72847328	0.011	0.008	1
Fam104a	0.05758145	0.33488659	0.269	0.283	1
Mdp1	0.05873402	0.4287115	0.118	0.114	1
Prkab1	0.05879062	0.28869499	0.031	0.037	1
Sec22b	0.05891571	0.26001536	0.121	0.138	1
Akap10	0.06041997	0.43802853	0.074	0.068	1
Gm17477	0.06094582	0.60284273	0.014	0.011	1
Rpl9-ps6	0.06097684	0.32419266	0.051	0.059	1
Tmem165	0.06116469	0.27563906	0.069	0.079	1
Acot7	0.06158934	0.58368667	0.124	0.121	1
Taf3	0.06190821	0.44407318	0.114	0.109	1
Cisd1	0.06248064	0.30347395	0.072	0.083	1
Cox19	0.06249366	0.30315317	0.041	0.048	1
1110008P14R	0.0626748	0.39719842	0.163	0.162	1
Tmem248	0.06268326	0.27236504	0.146	0.167	1
Trip11	0.06283627	0.30026358	0.224	0.227	1
Mon1a	0.06401358	0.69868911	0.018	0.015	1
Arhgap15	0.0645372	0.26221822	0.241	0.243	1
Ndufaf5	0.06471386	0.25657454	0.011	0.014	1
Usf1	0.06471452	0.56520748	0.064	0.058	1
Dnah8	0.06517824	0.49512005	0.011	0.008	1
Kansl3	0.06575274	0.4579403	0.137	0.134	1
Vps51	0.0663481	0.71145204	0.064	0.06	1
Vps4a	0.06710539	0.33941837	0.07	0.08	1
Tor1a	0.06715557	0.34428498	0.091	0.105	1
Oxa1l	0.06806099	0.32669921	0.096	0.11	1
Zfp87	0.06833052	0.55616546	0.067	0.062	1
Rmnd5a	0.06837444	0.40948288	0.131	0.128	1
Dyrk1b	0.06843173	0.69002208	0.029	0.025	1
Isca2	0.068458	0.32003123	0.067	0.077	1
Adam12	0.06860632	0.45586335	0.012	0.009	1
Tmed4	0.0687716	0.27448363	0.033	0.039	1
Cobll1	0.06906197	0.29500654	0.056	0.05	1
Fuom	0.06945328	0.46211279	0.024	0.029	1

Bcl6	0.06949497	0.49075833	0.137	0.134	1
Gm5914	0.06960917	0.54807939	0.027	0.023	1
Ino80e	0.06979483	0.31829761	0.066	0.076	1
Sympk	0.06993462	0.26318231	0.054	0.062	1
Mink1	0.07084297	0.65427912	0.074	0.069	1
Klhdc3	0.07113299	0.2540092	0.06	0.069	1
Snupn	0.07149124	0.59509947	0.021	0.018	1
Igf2bp3	0.07169284	0.69124934	0.018	0.015	1
Khnyn	0.07186739	0.44735307	0.078	0.073	1
Csrp1	0.07300646	0.29183649	0.121	0.138	1
Ints1	0.07359039	0.33982091	0.04	0.047	1
2510039O18R	0.07369375	0.4898794	0.09	0.085	1
Fbrsl1	0.07383038	0.26747855	0.076	0.086	1
Nmt2	0.07394119	0.25309486	0.101	0.113	1
Utp23	0.07587757	0.49131971	0.072	0.067	1
Trappc13	0.07647197	0.54980785	0.054	0.05	1
Mapk1ip1	0.07656568	0.43387335	0.024	0.021	1
Casp8ap2	0.07664154	0.37745246	0.152	0.149	1
Thg1l	0.07740977	0.27908537	0.017	0.021	1
Sike1	0.0776311	0.39277342	0.098	0.093	1
Zfp740	0.07775914	0.34865316	0.079	0.074	1
Desi1	0.07785622	0.4893933	0.071	0.066	1
Sc5d	0.07823134	0.46471088	0.088	0.083	1
Mex3c	0.07854308	0.43314424	0.108	0.104	1
Nf2	0.07924566	0.27767803	0.031	0.037	1
Ric8a	0.07939283	0.38567471	0.072	0.082	1
Lin37	0.07941879	0.29582733	0.03	0.035	1
Rnf219	0.0796445	0.68942316	0.023	0.02	1
Aqr	0.07967271	0.42735735	0.151	0.149	1
4930481A15R	0.07973206	0.50972435	0.015	0.012	1
Tada3	0.08016249	0.38652719	0.03	0.035	1
Fam193b	0.08049931	0.42678111	0.054	0.049	1
Serpina3f	0.08070966	0.65390943	0.023	0.019	1
Ncapd2	0.08135983	0.58885659	0.028	0.024	1
Prps1	0.0815359	0.47327697	0.09	0.085	1
Abca2	0.0822115	0.48932478	0.038	0.033	1
Gm13546	0.08241343	0.26974761	0.009	0.012	1
Med19	0.08258883	0.3008572	0.052	0.06	1
Ralgapb	0.08307634	0.51137977	0.085	0.081	1
Fastkd3	0.08311643	0.72207479	0.015	0.012	1
Sephs2	0.0833563	0.43671002	0.045	0.04	1
Casp1	0.08350884	0.2907571	0.051	0.058	1
Zfp846	0.08372373	0.69744028	0.016	0.013	1
Prelid3b	0.08480593	0.35912714	0.11	0.126	1
Dtwd2	0.08508851	0.74172684	0.012	0.009	1
Etfrf1	0.08547874	0.26278644	0.029	0.034	1
Cenpc1	0.08612873	0.27627028	0.054	0.062	1
Prpsap1	0.086194	0.25725315	0.064	0.073	1

Aggf1	0.08761377	0.47814445	0.118	0.115	1
Slc4a1ap	0.08762244	0.38571801	0.1	0.095	1
Rap1gds1	0.08853247	0.32859712	0.104	0.118	1
Sprtn	0.08856769	0.59688553	0.043	0.039	1
Frmd8	0.08930677	0.65886496	0.036	0.032	1
Rsf1	0.08958984	0.27650728	0.203	0.204	1
Aasdhppt	0.08972902	0.52758157	0.063	0.059	1
Palm	0.08987747	0.29511523	0.009	0.012	1
Rcbtb2	0.09124167	0.58747703	0.087	0.099	1
Rbm48	0.09158707	0.42243952	0.064	0.06	1
Acap3	0.09204882	0.64177431	0.018	0.015	1
Gatd1	0.09268178	0.29269184	0.047	0.054	1
Plagl1	0.09274722	0.89003855	0.021	0.018	1
Ccdc85b	0.09546795	0.84322178	0.011	0.008	1
Haus1	0.09684805	0.29362586	0.009	0.012	1
Cdipt	0.09713877	0.44005081	0.129	0.127	1
Ercc1	0.09786111	0.3428591	0.024	0.029	1
Ppp2r5c	0.09815595	0.36844697	0.262	0.279	1
Nsfl1c	0.09841053	0.27937963	0.094	0.106	1
Traf7	0.09893207	0.31263093	0.059	0.067	1
Fez2	0.09949716	0.2655859	0.024	0.028	1
Zfp692	0.09967189	0.74695371	0.017	0.014	1
Eif2b5	0.10030364	0.28109578	0.05	0.057	1
Mettl14	0.10035817	0.45072006	0.054	0.049	1
Spin1	0.1008175	0.3343572	0.157	0.155	1
Cox16	0.10124604	0.27558835	0.066	0.075	1
Rab4a	0.10137626	0.46415747	0.043	0.039	1
Angel2	0.10143087	0.39997563	0.155	0.154	1
Cry1	0.10146257	0.51789671	0.083	0.079	1
Xlr4c	0.10191483	0.4308031	0.025	0.021	1
Rab33b	0.10214036	0.51335346	0.047	0.043	1
Srek1ip1	0.10215102	0.29973875	0.043	0.049	1
Zfp260	0.10237711	0.39838401	0.072	0.067	1
Tapbpl	0.10255702	0.41631321	0.124	0.12	1
Kin	0.10256942	0.43668993	0.101	0.097	1
Brd3	0.10269901	0.39700397	0.091	0.087	1
Rpa3	0.10285639	0.27736602	0.048	0.055	1
R3hcc1	0.10315977	0.58265652	0.02	0.016	1
Epc2	0.10371606	0.36014461	0.177	0.178	1
0610010K14R	0.10488362	0.38851668	0.043	0.05	1
Lpcat4	0.10550913	0.29491564	0.019	0.022	1
Rrp36	0.10554279	0.29639606	0.034	0.04	1
Icmt	0.10554642	0.27855221	0.028	0.033	1
Endog	0.10555258	0.30873557	0.023	0.027	1
Thap11	0.10596953	0.26812842	0.019	0.023	1
Zfp560	0.10618482	0.44835042	0.078	0.073	1
Ick	0.10638795	0.84504737	0.012	0.009	1
Golga2	0.10711124	0.31626929	0.056	0.064	1

Gm13012	0.10804591	0.60678197	0.014	0.012	1
Ncaph	0.1084141	0.38876905	0.013	0.017	1
Dtx3	0.1084267	0.48115185	0.072	0.067	1
Med30	0.10846906	0.46679117	0.119	0.117	1
Ighmbp2	0.10851305	0.70292936	0.021	0.018	1
Srebf1	0.10883022	0.29578667	0.056	0.064	1
Acot9	0.10950679	0.38150412	0.062	0.07	1
1110059G10R	0.1097863	0.27061483	0.085	0.096	1
Kctd2	0.10979814	0.56516799	0.043	0.039	1
Scoc	0.10981123	0.27976171	0.037	0.042	1
Asb3	0.1102926	0.50628976	0.031	0.028	1
Lancl1	0.11062364	0.35053391	0.027	0.032	1
Csnk2a2	0.11082426	0.25157587	0.217	0.249	1
Fyco1	0.11105058	0.49728926	0.061	0.056	1
Rnf4	0.11134986	0.27546969	0.2	0.228	1
Hyls1	0.11161816	0.59399332	0.016	0.014	1
Btbd6	0.11170268	0.67603935	0.01	0.008	1
Gemin2	0.11174243	0.67257716	0.025	0.021	1
Foxk2	0.11211889	0.3965306	0.032	0.037	1
Mrpl43	0.11240377	0.48315781	0.166	0.17	1
Tcea1	0.11366773	0.26035782	0.307	0.328	1
Fam53b	0.11376066	0.44984937	0.033	0.029	1
Tmem9b	0.11402964	0.29337747	0.09	0.101	1
Haus2	0.11422282	0.25772062	0.036	0.042	1
Ppm1k	0.11424268	0.35314076	0.048	0.044	1
Gm26724	0.1145369	0.26796537	0.01	0.013	1
Rc3h2	0.11477947	0.37694412	0.182	0.185	1
Pcnp	0.11482668	0.40894691	0.234	0.246	1
Kptn	0.11549169	0.42835169	0.025	0.029	1
Atg4b	0.11631487	0.3158728	0.049	0.055	1
Tmem222	0.11668785	0.32520162	0.059	0.067	1
Slc16a6	0.11670216	0.31788885	0.196	0.197	1
1110051M20F	0.1170889	0.2896638	0.009	0.011	1
Foxo4	0.11715694	0.52695333	0.023	0.02	1
2810403A07R	0.11782708	0.37122494	0.147	0.145	1
Eef1akmt2	0.11802342	0.25316549	0.026	0.031	1
Tinf2	0.11808135	0.40274037	0.086	0.082	1
Ddx49	0.11813339	0.2935686	0.06	0.068	1
Tpgs2	0.1182797	0.61026683	0.035	0.031	1
Bora	0.11913992	0.51538255	0.024	0.021	1
Sbf1	0.11939784	0.41702214	0.109	0.106	1
Sgf29	0.11979227	0.58513	0.039	0.035	1
Zfp12	0.11996252	0.7068211	0.011	0.009	1
Hif1an	0.12041187	0.38119403	0.101	0.097	1
Pam16	0.12069396	0.48720989	0.146	0.147	1
Trim27	0.1208087	0.44952017	0.112	0.109	1
Ubxn7	0.12081869	0.36455981	0.091	0.086	1
Esco1	0.12119329	0.34852928	0.135	0.133	1

Fbxw2	0.12132388	0.28562576	0.221	0.226	1
Ahi1	0.12148186	0.36645435	0.083	0.079	1
Park7	0.12153651	0.34899477	0.294	0.315	1
Rnf6	0.12201455	0.27742457	0.153	0.173	1
Ntan1	0.12218484	0.48263462	0.147	0.148	1
Zfp868	0.12262659	0.51234712	0.057	0.053	1
Ddrgk1	0.12296579	0.27773086	0.2	0.23	1
Dmtf1	0.1233897	0.40853888	0.126	0.123	1
Mturn	0.12400964	0.48814594	0.014	0.012	1
Ubr5	0.1250031	0.3714501	0.215	0.223	1
Glmn	0.12531742	0.57738858	0.019	0.016	1
C1qtnf12	0.12537976	0.33459457	0.015	0.018	1
Lsm14b	0.12645002	0.25669116	0.028	0.033	1
Sqle	0.12653639	0.71571099	0.056	0.052	1
Ssb	0.12681104	0.26479716	0.355	0.388	1
Fam53a	0.12707749	0.34726694	0.04	0.036	1
Lrba	0.12783708	0.33909181	0.103	0.098	1
Kif20b	0.12838271	0.4086653	0.025	0.021	1
Zbtb42	0.12866517	1.09385932	0.022	0.019	1
Thumpd1	0.12913556	0.38925105	0.091	0.087	1
Usp31	0.12916363	0.26505776	0.028	0.032	1
Sco1	0.12916714	0.25027707	0.017	0.02	1
Xpot	0.12942617	0.25241614	0.051	0.057	1
Rfng	0.12984796	0.28459954	0.013	0.016	1
F2rl2	0.12993992	0.85622526	0.01	0.008	1
Ptar1	0.12997279	0.55219068	0.019	0.016	1
Tigd2	0.13043506	0.33842543	0.11	0.106	1
Aip	0.13046981	0.29741287	0.104	0.117	1
Tmem156	0.13090972	0.3119785	0.046	0.042	1
Tmem62	0.13155165	0.57740504	0.016	0.014	1
Dhx29	0.13163834	0.26212179	0.029	0.034	1
Zfp35	0.13248324	0.25970431	0.013	0.016	1
Drg1	0.13277053	0.48569181	0.087	0.083	1
Clpx	0.13353894	0.28370987	0.054	0.061	1
Zswim1	0.13458652	0.65386175	0.015	0.013	1
Slc37a1	0.13515451	0.54316813	0.014	0.012	1
Wipf2	0.13517908	0.25476552	0.121	0.136	1
Arfgap2	0.13520871	0.28225424	0.12	0.135	1
Tmub1	0.13656283	0.49046672	0.039	0.035	1
Ube3b	0.1366964	0.27028921	0.046	0.052	1
1810055G02R	0.13693613	0.39828443	0.009	0.012	1
Dicer1	0.1370829	0.28777969	0.138	0.135	1
Hras	0.13715039	0.46417049	0.066	0.074	1
Ilf2	0.13790738	0.30092293	0.18	0.18	1
Man1a2	0.13803609	0.39582294	0.145	0.145	1
Tusc2	0.13823159	0.25129992	0.033	0.038	1
9130401M01F	0.13874849	0.43427012	0.066	0.075	1
Ppm1d	0.13890736	0.45381918	0.058	0.054	1

Rnf139	0.13915078	0.45904367	0.077	0.073	1
Ankrd54	0.139299	0.48923621	0.038	0.034	1
Chchd3	0.13950791	0.29515627	0.128	0.144	1
C1d	0.14022291	0.32545265	0.087	0.098	1
Inafm2	0.14023723	0.55069516	0.024	0.021	1
Pdpr	0.14041229	0.42399943	0.039	0.035	1
Pcsk7	0.14156816	0.46926184	0.069	0.065	1
Mfap1b	0.14219056	0.32846077	0.075	0.071	1
Etfdh	0.14391013	0.25282333	0.047	0.053	1
Ddx28	0.14492662	0.78326666	0.029	0.026	1
Nmb	0.1454188	0.58680807	0.028	0.025	1
Nrde2	0.14666778	0.31194392	0.045	0.051	1
Nup153	0.1482911	0.35741253	0.16	0.16	1
Evi5l	0.148399	0.7705647	0.012	0.01	1
4933427D14R	0.14921039	0.62431513	0.015	0.013	1
Lnx2	0.14922392	0.6056683	0.047	0.044	1
Zfp995	0.14924345	0.30571986	0.008	0.011	1
Fbxo32	0.1497139	0.40781126	0.053	0.049	1
Pwwp2a	0.14977263	0.38563824	0.086	0.082	1
Tnfaip8l1	0.15003695	0.89322601	0.019	0.016	1
Ercc3	0.15015796	0.49770208	0.067	0.063	1
Bod1l	0.15162495	0.28441282	0.215	0.219	1
Mmgt2	0.15221752	0.29110692	0.023	0.027	1
Trmt10c	0.15233374	0.44059379	0.055	0.051	1
Letm2	0.15261433	0.33407948	0.052	0.048	1
Zfp146	0.15299171	0.32743193	0.048	0.044	1
Kctd18	0.15308277	0.3594621	0.052	0.048	1
Rnps1	0.15333698	0.34163097	0.224	0.233	1
Dnttip1	0.15443413	0.49073735	0.068	0.076	1
Ino80d	0.1546439	0.35048738	0.218	0.225	1
Cbll1	0.15558284	0.31339073	0.103	0.115	1
Ube4b	0.15630051	0.50187265	0.073	0.07	1
Cdc23	0.15651916	0.48853503	0.031	0.028	1
Txnl4a	0.1567881	0.37423701	0.087	0.098	1
Anp32e	0.15726733	0.25642174	0.288	0.303	1
Pdik1l	0.15738166	0.49329434	0.028	0.024	1
Coil	0.15760086	0.62991652	0.021	0.018	1
Dsel	0.15837852	0.8101231	0.01	0.008	1
Ostm1	0.15911358	0.26230793	0.069	0.076	1
Clns1a	0.15974352	0.38949335	0.095	0.092	1
Tef	0.15999303	0.56990169	0.028	0.025	1
Nbr1	0.16012247	0.2552775	0.082	0.091	1
Rce1	0.16115454	0.35814208	0.048	0.054	1
Yars	0.16157006	0.51899018	0.076	0.073	1
Strip1	0.16221941	0.28915143	0.056	0.063	1
Ciao1	0.16290352	0.33244082	0.045	0.051	1
Haus6	0.16293394	0.40299305	0.039	0.036	1
Thap7	0.16328788	0.41284301	0.037	0.042	1

Zbtb38	0.1637371	0.46081387	0.087	0.084	1
Zfp398	0.16375864	0.42937447	0.054	0.05	1
Pdp2	0.16407356	0.26348203	0.015	0.018	1
Agap2	0.16409162	0.4697903	0.022	0.02	1
Ppp1r16a	0.16469529	0.4017058	0.013	0.016	1
Mcrs1	0.16482811	0.27902199	0.079	0.088	1
Dolpp1	0.1650854	0.32955423	0.009	0.011	1
Cyhr1	0.16508564	0.4826201	0.076	0.073	1
Dcun1d5	0.16516345	0.33897821	0.262	0.282	1
Nipsnap1	0.16593985	0.67647173	0.02	0.018	1
2700097O09R	0.16609746	0.530331	0.032	0.029	1
Clk3	0.16641014	0.30588801	0.14	0.157	1
AI987944	0.16797281	0.34422564	0.037	0.033	1
Slco3a1	0.16823759	0.43937517	0.149	0.151	1
Smn1	0.1692354	0.41305709	0.077	0.086	1
Mrps26	0.1694707	0.51665521	0.097	0.095	1
Incenp	0.16995558	0.483151	0.041	0.038	1
Ccdc43	0.17019499	0.26270844	0.047	0.052	1
Pex11b	0.17044151	0.69221694	0.019	0.016	1
Mindy3	0.1712113	0.34898897	0.09	0.1	1
Zbtb45	0.17293196	0.81139717	0.014	0.012	1
Zfp91	0.17328132	0.28840537	0.35	0.384	1
Mrpl22	0.17345343	0.25783478	0.032	0.037	1
Bloc1s3	0.17350697	0.63844461	0.02	0.018	1
Pla2g16	0.17403638	0.35125991	0.184	0.189	1
AU041133	0.17441826	0.44374904	0.019	0.016	1
Cmtm4	0.17581133	0.40842473	0.013	0.016	1
Ptdss1	0.17588047	0.41401522	0.049	0.055	1
Cnot9	0.17607869	0.35237265	0.038	0.043	1
Ccnyl1	0.1762614	0.30987119	0.022	0.026	1
Rab11fip2	0.17703517	0.32920223	0.082	0.078	1
Cpd	0.17724145	0.26260824	0.093	0.103	1
Puf60	0.17726296	0.33530625	0.234	0.246	1
AI480526	0.17728013	0.45054869	0.023	0.02	1
Rbm10	0.17891332	0.48430907	0.055	0.051	1
Cep44	0.1791999	0.31047994	0.063	0.059	1
Pold2	0.1793223	0.34402244	0.021	0.024	1
Ankrd27	0.17980973	0.25347802	0.018	0.021	1
Cbr1	0.1814472	0.52526985	0.032	0.029	1
Zfp607a	0.18269337	0.67228104	0.018	0.016	1
0610012G03R	0.18290218	0.40752196	0.122	0.138	1
Mospd1	0.18318734	0.28356498	0.017	0.02	1
Cops7b	0.18343674	0.28673621	0.017	0.02	1
Mex3b	0.18523702	0.71784273	0.029	0.026	1
Ctu2	0.18606522	0.25369064	0.038	0.043	1
Pdcl	0.18630415	0.31615865	0.063	0.07	1
Rpp25l	0.18787314	0.47964266	0.101	0.1	1
Osbpl3	0.18815169	0.31475202	0.087	0.083	1

Elf2	0.18900715	0.3259467	0.205	0.212	1
Rnf121	0.19109561	0.3240115	0.043	0.048	1
Ube2a	0.19131093	0.36298392	0.226	0.239	1
Gm47662	0.19144506	0.68697665	0.021	0.018	1
Atpaf1	0.19184995	0.38597011	0.016	0.014	1
Cenpt	0.19316181	0.2715142	0.008	0.01	1
Rint1	0.19446169	0.396862	0.033	0.03	1
Nosip	0.19470467	0.49813853	0.059	0.056	1
Taf11	0.19681708	0.56306062	0.071	0.068	1
Clk4	0.1970904	0.26070053	0.27	0.285	1
Cops5	0.19873207	0.26012757	0.146	0.163	1
Ncbp1	0.19894448	0.4373226	0.08	0.077	1
Zfand1	0.1992167	0.32548438	0.017	0.02	1
Rbak	0.20041797	0.69394503	0.013	0.011	1
Mff	0.20062701	0.29587444	0.157	0.177	1
Dynll1	0.20147021	0.26741689	0.357	0.392	1
Mms19	0.20149361	0.30774542	0.057	0.063	1
Uggt1	0.20150782	0.29392163	0.095	0.105	1
Nin	0.20173384	0.35661536	0.133	0.132	1
Pear1	0.20192956	0.39583994	0.024	0.021	1
Slc1a5	0.20246581	0.28962344	0.115	0.128	1
Gm28935	0.20447356	0.41378525	0.016	0.014	1
Gm11423	0.20491403	0.59971206	0.01	0.009	1
Gpkow	0.20540339	0.41889013	0.055	0.051	1
Tor4a	0.20544623	0.73929047	0.011	0.009	1
Agap3	0.20671648	0.6079695	0.043	0.04	1
Ppwd1	0.2069499	0.25050933	0.163	0.162	1
Fbxl20	0.20797591	0.44093336	0.067	0.064	1
Tmem203	0.20862611	0.47405298	0.059	0.056	1
Cwc22	0.20880563	0.29955094	0.094	0.104	1
Akirin1	0.20930322	0.25755641	0.203	0.229	1
March2	0.21074928	0.4720984	0.096	0.094	1
Atp2a3	0.21118796	0.25066382	0.102	0.099	1
Mau2	0.21135679	0.30421149	0.145	0.144	1
Slc10a3	0.21213901	0.48750771	0.027	0.024	1
Hist1h4c	0.21280009	0.47895672	0.055	0.052	1
Ptrh2	0.21325964	0.35663709	0.043	0.048	1
Nudt22	0.21326015	0.40430486	0.01	0.012	1
Jak3	0.21369462	0.51888908	0.06	0.057	1
Surf1	0.21484242	0.36724951	0.082	0.091	1
Lsm1	0.21605458	0.46522529	0.081	0.078	1
Carhsp1	0.21621804	0.46211505	0.079	0.076	1
Snrpc	0.21650663	0.27101288	0.207	0.234	1
Ctla2b	0.21724357	0.30254206	0.009	0.011	1
Wdr20	0.21734956	0.44954262	0.09	0.088	1
Zfp142	0.2174837	0.27322837	0.039	0.044	1
Dus2	0.21804558	0.43846905	0.038	0.035	1
Kremen1	0.21827567	0.55703577	0.025	0.023	1

Kiz	0.21937254	0.30328934	0.017	0.02	1
Polr2j	0.22002985	0.54626825	0.099	0.098	1
2900076A07R	0.22060117	0.485629	0.016	0.014	1
Ube2j2	0.22107634	0.26055229	0.199	0.226	1
Ptrh1	0.22229971	0.3674796	0.01	0.012	1
Def6	0.22240343	0.40359747	0.057	0.054	1
1600002K03R	0.22247059	0.32133494	0.047	0.053	1
Cars2	0.22272672	0.27072371	0.037	0.041	1
Trabd	0.22286072	0.33473743	0.077	0.085	1
Slc35c1	0.22436592	0.27328154	0.013	0.015	1
Tgtp2	0.22493443	0.52026646	0.018	0.016	1
Stard10	0.22538614	0.60779023	0.025	0.022	1
Slc25a24	0.22600313	0.59974228	0.069	0.067	1
Smim27	0.22644178	0.4686391	0.085	0.083	1
Rnf7	0.22675571	0.43289991	0.2	0.212	1
1700123O20R	0.2269856	0.36251428	0.084	0.093	1
Zc3h3	0.22778428	0.27547853	0.023	0.026	1
Jade2	0.22802527	0.27200437	0.052	0.048	1
Tbce	0.22871217	0.27434738	0.072	0.08	1
Wdr53	0.23069844	0.32789815	0.022	0.025	1
Pdhb	0.23091248	0.25638487	0.146	0.162	1
Arl4a	0.23143318	0.38682287	0.117	0.116	1
Stxbp5	0.23158333	0.38773584	0.054	0.051	1
Golph3	0.23216703	0.28429522	0.215	0.244	1
Cdk1	0.23237662	0.42586343	0.011	0.013	1
Ndor1	0.23297107	0.72121466	0.011	0.009	1
Wdr48	0.23305929	0.31836864	0.086	0.094	1
Cep170b	0.23414079	0.71478381	0.013	0.011	1
Nsmf	0.23479345	0.35078881	0.02	0.023	1
Pstk	0.2348574	0.37074744	0.039	0.036	1
Foxj3	0.23593899	0.42329909	0.098	0.096	1
Zcchc17	0.23661357	0.26801342	0.094	0.103	1
Ccdc6	0.23705141	0.50794392	0.085	0.083	1
Zkscan1	0.23777801	0.29321047	0.046	0.051	1
Itga1	0.23784572	0.5135862	0.062	0.059	1
Cmc1	0.23860822	0.52011295	0.159	0.165	1
Slc4a8	0.23863355	0.74319624	0.018	0.016	1
Rp9	0.23891389	0.29249951	0.255	0.27	1
Rcl1	0.23937037	0.29683508	0.057	0.063	1
Tex264	0.23960558	0.36950233	0.05	0.055	1
Kpna6	0.23964195	0.25460731	0.046	0.051	1
Mvb12b	0.24003415	0.27187098	0.053	0.058	1
Alkbh8	0.24041846	0.38891998	0.034	0.031	1
Dda1	0.24063556	0.43370895	0.155	0.159	1
Oraov1	0.24295506	0.35900988	0.038	0.042	1
Eogt	0.24300611	0.40682297	0.01	0.012	1
Mad2l1bp	0.24303975	0.28528281	0.039	0.043	1
Lrwd1	0.24345285	0.39783391	0.028	0.031	1

Gins4	0.24368894	0.42749742	0.047	0.052	1
Tomm5	0.24452432	0.27580592	0.181	0.203	1
Zfp81	0.24496154	0.35869588	0.021	0.018	1
Kmt2d	0.24630448	0.39151744	0.122	0.121	1
Srfbp1	0.24709078	0.30397091	0.031	0.035	1
Kat7	0.24928822	0.26682832	0.084	0.092	1
Ermp1	0.2500554	0.50438147	0.045	0.042	1
Sap18	0.25155174	0.38120165	0.204	0.216	1
Dmap1	0.25186529	0.72399094	0.025	0.023	1
Rabggta	0.25212533	0.27635366	0.029	0.032	1
Edem1	0.25236542	0.38389884	0.262	0.284	1
Exosc7	0.25360096	0.33614123	0.037	0.041	1
Zbtb48	0.2537515	0.50036056	0.013	0.011	1
Rdh14	0.25400413	0.33880804	0.023	0.026	1
Cbx6	0.25523495	0.50231908	0.02	0.018	1
Cdk7	0.25570286	0.59156614	0.045	0.043	1
2610021A01R	0.25670598	0.38494396	0.015	0.013	1
Mrpl21	0.2570265	0.44758325	0.168	0.175	1
Capn15	0.25725436	0.48797649	0.045	0.042	1
Ppp3cc	0.25770689	0.25196209	0.059	0.055	1
Ankrd46	0.25801322	0.41357066	0.021	0.019	1
Trp53i13	0.26044961	0.59999801	0.024	0.021	1
Impad1	0.26065378	0.3102664	0.09	0.099	1
Scyl3	0.26277266	0.43222238	0.065	0.063	1
Mterf4	0.26277507	0.41620777	0.015	0.018	1
Dpy19l3	0.2631584	0.50872467	0.037	0.035	1
Tango2	0.26353637	0.3235719	0.021	0.024	1
Eif1ad	0.26376163	0.33329575	0.106	0.117	1
Pdpk1	0.26455009	0.41810017	0.126	0.127	1
Triap1	0.26504134	0.55699606	0.061	0.059	1
Nup188	0.26552978	0.31958416	0.027	0.031	1
Tmem101	0.26610247	0.39555484	0.015	0.017	1
Zbtb24	0.26630611	0.26862293	0.041	0.045	1
1700021F05Ri	0.26699952	0.4364415	0.018	0.021	1
Ift20	0.26700416	0.52229065	0.155	0.162	1
Mier2	0.26701049	0.32669706	0.011	0.013	1
Fam114a2	0.26726532	0.27302725	0.078	0.086	1
Prkrip1	0.26817833	0.33684971	0.077	0.085	1
Cyth2	0.2686989	0.3516625	0.105	0.116	1
Eed	0.26950621	0.42464815	0.117	0.116	1
Kdm1a	0.26961283	0.25415396	0.208	0.233	1
Arhgef10	0.26981095	0.59483656	0.018	0.021	1
1110038F14Ri	0.26989357	0.40933359	0.107	0.106	1
Gm26532	0.2705707	0.34217255	0.076	0.073	1
Kbtbd2	0.27058873	0.41236831	0.114	0.114	1
Lsm12	0.27085603	0.27952451	0.288	0.314	1
Grpel1	0.27105934	0.38609649	0.145	0.163	1
Nsmaf	0.27129265	0.27723481	0.113	0.124	1

Dffb	0.27285553	0.5341325	0.019	0.017	1
Grhpr	0.27347154	0.27118632	0.019	0.021	1
Snx15	0.27394275	0.31915231	0.022	0.025	1
Ccnk	0.27417229	0.46023287	0.114	0.114	1
D3Ertd751e	0.27427664	0.33765289	0.021	0.018	1
Cd200r4	0.275705	0.64809922	0.025	0.023	1
Prcc	0.27580185	0.51333761	0.047	0.045	1
D230025D16F	0.27586614	0.30860182	0.029	0.033	1
Med31	0.27665009	0.26759054	0.076	0.083	1
Sun1	0.27739533	0.57235264	0.043	0.04	1
Zfp169	0.27804774	0.26799876	0.022	0.02	1
Pgrmc1	0.27878406	0.37020691	0.079	0.087	1
Ncapd3	0.27912151	0.37688423	0.06	0.057	1
Arfrp1	0.27922354	0.3267536	0.109	0.12	1
Fdxr	0.27949764	0.26863882	0.024	0.027	1
Atxn7	0.27950281	0.28610754	0.134	0.133	1
Kctd9	0.28005895	0.25509997	0.023	0.026	1
Bbx	0.28017627	0.27138054	0.139	0.153	1
Mms22l	0.28082311	0.55978557	0.026	0.024	1
Slc7a6os	0.28145083	0.34500177	0.083	0.081	1
Knop1	0.28337738	0.34655818	0.084	0.092	1
Dmac2	0.28433195	0.35922633	0.078	0.075	1
Zfp748	0.28528275	0.38430075	0.027	0.025	1
Amd2	0.28556705	0.30733059	0.013	0.015	1
Armc1	0.28593571	0.35605958	0.075	0.073	1
Lrrc47	0.28603917	0.31834068	0.074	0.081	1
Kctd17	0.28680259	0.84536712	0.023	0.021	1
Rabggtb	0.28697691	0.34190839	0.127	0.14	1
Phf1	0.28806131	0.26900562	0.073	0.07	1
Hbb-bt	0.28855376	1.33762453	0.01	0.011	1
Stn1	0.28905083	0.48570197	0.044	0.042	1
Tsr3	0.28908986	0.59408511	0.041	0.039	1
Cul2	0.28917178	0.50440214	0.078	0.076	1
Zfpl1	0.28943991	0.4254586	0.033	0.037	1
Zfp84	0.28965495	0.43160653	0.053	0.05	1
Camk2b	0.28982415	0.57474908	0.02	0.018	1
Gpr107	0.29056335	0.29629741	0.094	0.103	1
Axin1	0.29091972	0.44992321	0.078	0.076	1
Cog8	0.29123566	0.26232857	0.039	0.043	1
Haus4	0.29148571	0.26802097	0.02	0.023	1
Zfp825	0.29205449	0.52729298	0.013	0.011	1
Nsun4	0.29225918	0.43385182	0.049	0.046	1
Nup50	0.2926678	0.38062552	0.159	0.164	1
Zc3hc1	0.29370944	0.37292113	0.043	0.047	1
Zfp777	0.29425406	0.51436661	0.028	0.025	1
Dnajc21	0.29469319	0.28988755	0.169	0.187	1
Gm26561	0.29587309	0.64362531	0.01	0.009	1
Ddx47	0.29617989	0.33314868	0.145	0.16	1

Runx2os1	0.29639613	0.65554999	0.02	0.018	1
Pitpnb	0.2968975	0.34106148	0.09	0.098	1
Dtnb	0.2975148	0.32566059	0.04	0.037	1
Fbxo3	0.29760142	0.47757035	0.078	0.077	1
Lig3	0.29886528	0.25783546	0.039	0.043	1
Atrip	0.30179082	0.41334475	0.02	0.023	1
Dnajc2	0.30267653	0.27304229	0.165	0.182	1
Tbc1d17	0.3029418	0.26925864	0.051	0.055	1
Reep4	0.3033101	0.30844131	0.04	0.044	1
Paxip1	0.30468246	0.26003582	0.038	0.042	1
Mtap	0.30501801	0.3542621	0.031	0.029	1
Abtb2	0.30526802	0.43681719	0.056	0.061	1
Ckap5	0.30580035	0.3585979	0.081	0.089	1
B230219D22R	0.30580544	0.26481108	0.237	0.268	1
Cutc	0.3089159	0.3459426	0.017	0.02	1
Orai2	0.30894691	0.40110406	0.053	0.05	1
Coa3	0.30999426	0.41851569	0.113	0.125	1
Nsd2	0.31042442	0.3543473	0.109	0.119	1
Imp3	0.31047496	0.37588122	0.177	0.183	1
Sart3	0.31056199	0.32057977	0.067	0.073	1
Mrpl40	0.31068978	0.33426668	0.062	0.067	1
Polr2b	0.31075926	0.31839722	0.064	0.07	1
Mea1	0.31123633	0.37035837	0.097	0.107	1
Ppp2cb	0.31146187	0.42152375	0.096	0.095	1
Zfp330	0.31265235	0.31880371	0.12	0.132	1
Cluap1	0.31283884	0.27107724	0.019	0.021	1
Umps	0.31342219	0.29463067	0.032	0.036	1
Gtf3c5	0.31425045	0.3044475	0.021	0.024	1
C730034F03R	0.31496615	0.31640471	0.024	0.021	1
Ubxn2a	0.3159657	0.33810357	0.179	0.185	1
Gtdc1	0.31775198	0.49352644	0.053	0.051	1
Zfp760	0.31968712	0.47803489	0.01	0.009	1
B3galt5	0.32011253	0.37540167	0.027	0.025	1
Abcf1	0.32070328	0.35579238	0.236	0.252	1
Ngly1	0.32316257	0.26741236	0.051	0.056	1
Dis3l	0.32432301	0.35463788	0.012	0.014	1
Scyl2	0.32531962	0.28977588	0.089	0.096	1
Rnf123	0.32548881	0.34344091	0.037	0.041	1
Als2cl	0.32571126	0.35644923	0.012	0.01	1
Pcnt	0.32621479	0.4272478	0.106	0.106	1
Cpox	0.32706361	0.39822128	0.045	0.042	1
Tmem59	0.32713068	0.3091127	0.24	0.256	1
Fbxo46	0.32892899	0.58593542	0.019	0.017	1
Pnkd	0.32962914	0.35990827	0.069	0.076	1
Bap1	0.32997492	0.27972293	0.051	0.056	1
Pstpip1	0.33071559	0.53826021	0.119	0.121	1
Rnf181	0.3314907	0.41134529	0.082	0.08	1
C030034I22Ri	0.33338951	0.27638094	0.051	0.049	1

Tubgcp4	0.3336926	0.36675672	0.066	0.063	1
Mrps23	0.33372406	0.44318039	0.134	0.137	1
Rad52	0.33393204	0.54330072	0.019	0.018	1
Nasp	0.33413762	0.33556979	0.186	0.192	1
Hdac8	0.33433496	0.3633545	0.022	0.02	1
Zbtb16	0.33441917	0.26665347	0.014	0.016	1
Nr1h2	0.33586761	0.38354636	0.115	0.126	1
Uqcrfs1	0.33603471	0.28460207	0.317	0.349	1
1500011B03R	0.3367515	0.69713031	0.023	0.021	1
Smyd3	0.33683431	0.42014131	0.029	0.027	1
Tipin	0.33750103	0.30727925	0.027	0.03	1
Mad2l1	0.33994375	0.29013383	0.02	0.022	1
Csnk1g2	0.34001593	0.34622998	0.155	0.157	1
Zcchc9	0.34046087	0.4191127	0.166	0.172	1
Mpst	0.34055215	0.59797467	0.053	0.051	1
E2f6	0.34260487	0.26022307	0.01	0.011	1
Dgcr2	0.34490503	0.34355984	0.052	0.056	1
Aurkaip1	0.3449671	0.29547402	0.228	0.259	1
Map3k3	0.34586416	0.29831641	0.057	0.062	1
Nelfb	0.34595983	0.2863923	0.078	0.084	1
Rad1	0.34643962	0.25555991	0.017	0.016	1
Arhgap10	0.34692381	0.33361474	0.037	0.04	1
Ccdc107	0.34694356	0.36601905	0.099	0.108	1
Ssna1	0.3470232	0.56530166	0.086	0.086	1
Mrpl13	0.34793271	0.46605301	0.136	0.139	1
Crnkl1	0.34820478	0.42455815	0.125	0.127	1
Calcoco1	0.3494095	0.43874619	0.033	0.031	1
3110040N11R	0.34991994	0.49085312	0.032	0.03	1
Ube2z	0.35340584	0.31881105	0.133	0.146	1
Upf2	0.35419067	0.2544924	0.155	0.156	1
2610044O15R	0.35553852	0.49010944	0.015	0.013	1
Topors	0.35589361	0.27407435	0.094	0.101	1
Cpped1	0.35670592	0.28653882	0.014	0.016	1
Gbp8	0.35684661	0.50118626	0.058	0.056	1
Sdr39u1	0.35733045	0.38926346	0.012	0.014	1
Adh5	0.36123989	0.5841529	0.131	0.136	1
Gm30025	0.36218981	0.41887206	0.011	0.013	1
Ngdn	0.36254577	0.40726186	0.15	0.155	1
Zfp518a	0.36386233	0.300891	0.045	0.049	1
Unc50	0.36452532	0.26514973	0.08	0.087	1
Gps2	0.36600621	0.41911571	0.143	0.147	1
Rgs10	0.3686152	0.32273051	0.279	0.302	1
Hsd17b7	0.36889636	0.38314685	0.029	0.032	1
Usp25	0.36950051	0.29498333	0.251	0.268	1
Srpr	0.37079874	0.30169918	0.262	0.282	1
Acat2	0.37240463	0.36963412	0.021	0.019	1
Naa60	0.37265099	0.49788692	0.048	0.046	1
Araf	0.3749884	0.25952557	0.109	0.118	1

Cep19	0.37560695	0.87562967	0.01	0.009	1
Lrch3	0.37567843	0.42515162	0.09	0.09	1
Cfap20	0.37686821	0.39397985	0.072	0.078	1
Zfhx2	0.37752953	0.39635469	0.032	0.03	1
Lsg1	0.37769807	0.30765297	0.081	0.088	1
Gm31597	0.37836418	0.35349296	0.02	0.022	1
Bet1l	0.3784598	0.35888603	0.104	0.114	1
Hmgn5	0.37903309	0.35001757	0.108	0.117	1
Rex1bd	0.37962491	0.36024977	0.155	0.173	1
Lpar6	0.37976818	0.32891825	0.087	0.085	1
Scaf8	0.3821178	0.30442141	0.12	0.13	1
AI837181	0.38288103	0.42272518	0.074	0.072	1
Tbpl1	0.38342262	0.34846847	0.08	0.086	1
Fam98a	0.38512573	0.25766704	0.026	0.029	1
Zdhhc7	0.3851302	0.34154836	0.043	0.046	1
Rbm18	0.38538157	0.40693057	0.083	0.082	1
D730003I15Ri	0.38717639	0.35844618	0.033	0.036	1
Cyb561d1	0.38727877	0.55581067	0.027	0.025	1
Lysmd3	0.38858899	0.31586376	0.103	0.102	1
Ryk	0.38888447	0.35001617	0.022	0.02	1
Cc2d1a	0.39009287	0.28920862	0.014	0.016	1
Nfyc	0.3902075	0.38611935	0.066	0.072	1
Rabif	0.39115227	0.46956205	0.052	0.05	1
Dennd1c	0.39152081	0.25776344	0.058	0.062	1
Ube2g1	0.39155032	0.39741329	0.081	0.08	1
Disp1	0.39164196	0.5702977	0.013	0.011	1
Rnf26	0.39218566	0.36029815	0.017	0.019	1
Bmt2	0.39268611	0.28188572	0.086	0.093	1
Klhl28	0.39285167	0.37281648	0.071	0.069	1
Mmgt1	0.39346855	0.26943073	0.029	0.031	1
3110043O21R	0.39477746	0.50506405	0.118	0.12	1
Maml1	0.39541883	0.45169481	0.073	0.072	1
Kdm1b	0.39552193	0.34879396	0.017	0.018	1
Sart1	0.39593617	0.2876045	0.121	0.132	1
Ndufs4	0.39613863	0.40562877	0.216	0.232	1
Hist1h3a	0.39680405	0.27330949	0.026	0.028	1
Hba-a2	0.39804848	1.01667943	0.018	0.02	1
Pkp4	0.39813619	0.31902064	0.039	0.042	1
Rpp21	0.39828935	0.36938693	0.149	0.153	1
Gm26699	0.39862204	0.3314127	0.043	0.047	1
Gbf1	0.3994448	0.32786533	0.071	0.076	1
Zxdc	0.39973061	0.38493305	0.031	0.033	1
Ric8b	0.39980463	0.33020012	0.018	0.02	1
Ufc1	0.40015555	0.32789963	0.212	0.242	1
Sec23b	0.40111443	0.25658186	0.116	0.126	1
Faap24	0.40247991	0.45458657	0.02	0.018	1
Mettl5	0.4030799	0.43403952	0.027	0.03	1
Galt	0.40505156	0.37937126	0.024	0.026	1

AC129328.1	0.40594124	0.47835253	0.012	0.011	1
Mrpl51	0.40937467	0.420822	0.131	0.134	1
Zranb1	0.40991459	0.31707434	0.182	0.188	1
Zfp180	0.41245151	0.40159164	0.044	0.042	1
Gm4673	0.41263532	0.51515796	0.017	0.019	1
Tex261	0.41407441	0.37472893	0.114	0.125	1
Abt1	0.41458137	0.26812027	0.058	0.063	1
Mrpl46	0.41620182	0.4216594	0.071	0.077	1
Exosc9	0.41676054	0.39050299	0.045	0.048	1
Tprkb	0.41775212	0.30036505	0.032	0.035	1
Zfp830	0.4185521	0.32786446	0.082	0.088	1
Mcu	0.41871085	0.38191278	0.034	0.032	1
Ndufaf8	0.41879463	0.28452083	0.174	0.192	1
Med24	0.4192694	0.32161507	0.03	0.032	1
Sap18b	0.41950231	0.28813905	0.012	0.013	1
Slc9a9	0.41976496	0.61341249	0.066	0.065	1
Abca7	0.42007635	0.32518759	0.057	0.062	1
Tfb2m	0.42046447	0.50111484	0.048	0.047	1
Derl2	0.42105408	0.48783676	0.134	0.139	1
Phax	0.42122708	0.55617158	0.105	0.107	1
Pik3c3	0.42232082	0.41491188	0.056	0.054	1
2610524H06R	0.42374178	0.45733924	0.038	0.041	1
Rufy2	0.42374589	0.2949322	0.056	0.054	1
Mfsd14a	0.42406657	0.29711914	0.111	0.12	1
Ing4	0.42528633	0.26899704	0.063	0.067	1
Gmps	0.4253644	0.30774898	0.129	0.141	1
Trak2	0.42538313	0.26211503	0.071	0.076	1
Uimc1	0.42668148	0.36358892	0.14	0.143	1
Mvk	0.42700529	0.42556786	0.024	0.026	1
Mppe1	0.42723932	0.31785215	0.014	0.013	1
Pank2	0.42861152	0.32878406	0.126	0.138	1
Bcas3	0.42870887	0.26088396	0.034	0.037	1
Cdkn2c	0.42920388	0.48325609	0.02	0.018	1
Mtmr2	0.4308943	0.40419803	0.054	0.058	1
Cyb5d2	0.43278252	0.41611307	0.018	0.016	1
Tprn	0.43350388	0.53388363	0.013	0.012	1
Cpsf4	0.43457435	0.48908699	0.037	0.035	1
Slc7a5	0.43575976	0.33485393	0.057	0.062	1
Sars2	0.43608468	0.43253429	0.012	0.014	1
Hectd4	0.4379028	0.47935764	0.029	0.028	1
Cox20	0.43817259	0.27594295	0.141	0.153	1
Eif4enif1	0.44098784	0.3412381	0.059	0.064	1
Commd7	0.44125761	0.38134761	0.102	0.111	1
Pdcd7	0.4412945	0.28380883	0.052	0.055	1
Dctn6	0.44333764	0.44693448	0.071	0.07	1
Rad17	0.44747809	0.36755421	0.066	0.064	1
Nit1	0.448751	0.26086441	0.049	0.052	1
Lin52	0.44968055	0.42993815	0.022	0.02	1

Xrcc4	0.44978401	0.25635875	0.029	0.032	1
Emc8	0.45009634	0.471521	0.047	0.051	1
Zfp930	0.45012444	0.25571547	0.013	0.015	1
Ing5	0.45056054	0.36703795	0.12	0.121	1
Tjap1	0.45152256	0.5040844	0.051	0.05	1
Pura	0.45170668	0.26267896	0.172	0.176	1
Tlcd1	0.45201449	0.49807998	0.015	0.017	1
Ddx10	0.45249378	0.27859835	0.098	0.097	1
B130006D01R	0.45251963	0.34427514	0.011	0.01	1
Mbip	0.4527723	0.37519416	0.037	0.039	1
Lypla1	0.45281373	0.3492294	0.098	0.106	1
Cchcr1	0.45286599	0.57352522	0.011	0.01	1
Ocel1	0.45390485	0.38147605	0.042	0.045	1
Klhl36	0.45506914	0.41404938	0.01	0.012	1
Gm10093	0.45561279	0.28176491	0.023	0.025	1
Fam120aos	0.45573121	0.65351154	0.01	0.009	1
Ppip5k1	0.45632404	0.5255899	0.015	0.014	1
Bloc1s4	0.45771776	0.34832005	0.081	0.088	1
Crkl	0.45809945	0.39601363	0.09	0.09	1
Kcnn4	0.45823617	0.50656203	0.091	0.091	1
Desi2	0.45992699	0.4518468	0.108	0.11	1
Ugp2	0.46053525	0.56506254	0.126	0.13	1
Rrp8	0.46095526	0.27807554	0.051	0.054	1
Phospho2	0.4616863	0.37103855	0.05	0.048	1
Rcor1	0.46253843	0.2569318	0.105	0.112	1
Hint3	0.46435261	0.48582695	0.11	0.112	1
Zpr1	0.46486866	0.30422114	0.069	0.074	1
Zfp280c	0.46611432	0.28671539	0.021	0.023	1
Upf3a	0.46649123	0.40259488	0.128	0.131	1
Acad8	0.46857311	0.50179707	0.011	0.012	1
Psmg2	0.46874704	0.46367447	0.041	0.039	1
Zfp101	0.46895948	0.4028724	0.02	0.019	1
Usp42	0.46941854	0.41885882	0.045	0.043	1
Clp1	0.46979686	0.38832242	0.07	0.069	1
Vps36	0.47104477	0.31651378	0.158	0.174	1
Lmtk2	0.47108056	0.39864998	0.052	0.051	1
Anapc4	0.47132374	0.38863676	0.04	0.043	1
Zfp553	0.47175049	0.35118722	0.025	0.024	1
Rab26os	0.47235532	0.36564111	0.03	0.033	1
Zfp952	0.47298246	0.34947732	0.027	0.025	1
Tmem18	0.47298453	0.61514262	0.015	0.014	1
Dnajc19	0.47320277	0.33538377	0.182	0.203	1
Intu	0.47392566	0.3449285	0.009	0.011	1
Ice1	0.47393818	0.40129438	0.074	0.074	1
Zfp959	0.47525437	0.34230344	0.031	0.029	1
Mtfmt	0.4752669	0.2815764	0.021	0.023	1
Osbpl7	0.475741	0.53342069	0.024	0.023	1
Hbs1l	0.47592033	0.41663695	0.087	0.087	1

Mapk8ip3	0.47735251	0.37915945	0.077	0.076	1
Dgcr14	0.47804348	0.45144185	0.037	0.039	1
Gbp4	0.47891471	0.31172441	0.122	0.122	1
Zfp580	0.48048766	0.65979267	0.01	0.009	1
BC002059	0.48074308	0.37347614	0.017	0.015	1
Zfp275	0.48131294	0.52697415	0.024	0.023	1
Tmem87a	0.48157065	0.40380693	0.085	0.085	1
Dusp14	0.48261496	0.39110005	0.013	0.014	1
Ppp2r2a	0.48290028	0.28102833	0.226	0.24	1
Med26	0.48326756	0.44968499	0.027	0.026	1
Nrf1	0.48354345	0.25318963	0.076	0.082	1
Trim39	0.48531473	0.26796731	0.055	0.053	1
Cbr4	0.48538406	0.34630409	0.011	0.012	1
Soat2	0.48582475	0.46509946	0.01	0.009	1
Fxn	0.48812357	0.58053279	0.037	0.036	1
Sik2	0.48829893	0.25712226	0.101	0.108	1
Gpank1	0.48846146	0.34350991	0.067	0.066	1
Zfp598	0.48861104	0.29556948	0.043	0.046	1
Ccdc30	0.491258	0.52200828	0.015	0.013	1
Fam173b	0.49233921	0.55125207	0.025	0.027	1
Chmp1b	0.4929348	0.25884704	0.143	0.155	1
Rexo2	0.49303063	0.41816311	0.194	0.207	1
Pskh1	0.49388373	0.27784666	0.018	0.02	1
Ppif	0.49453011	0.2621122	0.029	0.031	1
Gm9493	0.4958356	0.41299007	0.074	0.08	1
Nudt19	0.49634183	0.59734376	0.031	0.03	1
Tarbp2	0.49723592	0.3808735	0.041	0.044	1
Fahd2a	0.49855074	0.4171151	0.017	0.018	1
Dr1	0.4994026	0.43990472	0.082	0.082	1
Unc119b	0.50194779	0.39899246	0.091	0.091	1
Fnta	0.50234453	0.29385775	0.045	0.049	1
Wtap	0.50292647	0.27498053	0.182	0.2	1
Pigm	0.50332182	0.2772678	0.064	0.068	1
Nt5c3b	0.50379933	0.43962515	0.036	0.034	1
Sfi1	0.50630094	0.31277354	0.034	0.032	1
Bub3	0.50737838	0.30378987	0.146	0.158	1
Scaf1	0.50800788	0.29680259	0.059	0.063	1
Ppp2r1b	0.51009315	0.26317857	0.03	0.032	1
Emc7	0.51124433	0.25815068	0.249	0.282	1
Exoc8	0.5118906	0.41419934	0.014	0.015	1
Pcgf1	0.51314275	0.47945145	0.021	0.02	1
Sgpp1	0.51374959	0.32137601	0.057	0.056	1
Zfp160	0.51436366	0.37381003	0.032	0.034	1
Ptpn11	0.51514801	0.45323538	0.135	0.14	1
Coq3	0.51650305	0.64901299	0.013	0.012	1
Ino80b	0.51740582	0.34980688	0.072	0.077	1
Git2	0.51766511	0.25147266	0.243	0.255	1
Gm11131	0.51783844	0.44486244	0.012	0.011	1

Yod1	0.51800753	0.32415696	0.04	0.043	1
5031425E22R	0.51912635	0.2516873	0.113	0.12	1
Gtpbp1	0.52004191	0.37677022	0.066	0.065	1
Mrgbp	0.52008768	0.25828799	0.035	0.037	1
Tm2d3	0.52187476	0.55294804	0.068	0.068	1
Ppil3	0.52343468	0.63871261	0.054	0.054	1
Slc39a3	0.52394731	0.4949278	0.027	0.025	1
Notch2	0.52596861	0.28612555	0.156	0.169	1
Vamp2	0.52648969	0.31511165	0.143	0.147	1
Ccdc94	0.52717745	0.43562908	0.017	0.019	1
Erlec1	0.5281664	0.48843654	0.105	0.106	1
Ipp	0.52843746	0.43308476	0.01	0.011	1
Gatsl3	0.52924691	0.35933382	0.034	0.036	1
Trmt13	0.53010789	0.27313687	0.059	0.062	1
Wdr70	0.53020452	0.42144922	0.064	0.068	1
Toe1	0.53046896	0.30678473	0.033	0.035	1
Snrnp200	0.53068908	0.25419358	0.158	0.171	1
Safb	0.53071774	0.2995611	0.191	0.2	1
Phf2	0.53228081	0.42528371	0.07	0.07	1
Xpo4	0.53329083	0.36351684	0.093	0.093	1
Atf1	0.53386545	0.28359816	0.151	0.164	1
Rbm4b	0.53396392	0.4682204	0.079	0.079	1
Rsl1d1	0.53399975	0.35407875	0.196	0.207	1
Emc3	0.53458991	0.26185449	0.162	0.176	1
Tmem128	0.53463957	0.28243335	0.215	0.24	1
Fam172a	0.53469047	0.32372879	0.136	0.138	1
Pfkp	0.53496564	0.33262041	0.249	0.269	1
Tomm34	0.53505286	0.48928418	0.096	0.097	1
Mcm3ap	0.53532196	0.26561876	0.039	0.041	1
Maip1	0.53576189	0.52309086	0.067	0.073	1
Slc25a4	0.53743838	0.31837553	0.221	0.246	1
Lcmt2	0.53791028	0.52155852	0.015	0.014	1
Tm9sf1	0.53820773	0.27791124	0.019	0.02	1
Ppp6r1	0.54140918	0.28418465	0.158	0.172	1
Med11	0.54173353	0.38549738	0.11	0.112	1
Ncdn	0.54234422	0.29882469	0.025	0.027	1
Fam3c	0.54262752	0.37211845	0.081	0.08	1
Rusc1	0.54420005	0.32586493	0.054	0.058	1
Ppp4r3a	0.5459734	0.27625131	0.212	0.223	1
Ebpl	0.54597771	0.4400466	0.046	0.049	1
Tmem42	0.54830392	0.48795907	0.027	0.029	1
Man2c1	0.5491667	0.467754	0.021	0.023	1
2300009A05R	0.54931289	0.55369129	0.073	0.073	1
Rbm14	0.54964243	0.41898129	0.047	0.046	1
Smim26	0.54970004	0.48109324	0.072	0.071	1
Coa5	0.55214238	0.39007235	0.151	0.158	1
Tmem167b	0.55220828	0.3558396	0.064	0.069	1
Commd8	0.5522856	0.35900044	0.115	0.124	1

Zfp472	0.55272559	0.26678369	0.028	0.027	1
Chtop	0.55304152	0.27286603	0.188	0.206	1
Znhit3	0.55463393	0.47004032	0.034	0.033	1
Hltf	0.5555395	0.32459111	0.095	0.095	1
Rab10os	0.55743082	0.33726051	0.148	0.162	1
Nap1l4	0.56297537	0.34627856	0.18	0.189	1
Wdr55	0.56298132	0.35460716	0.02	0.022	1
Cep76	0.5639262	0.55563266	0.015	0.014	1
Zfp770	0.56520766	0.30397159	0.019	0.021	1
Polr2c	0.56584296	0.42920684	0.155	0.163	1
Acbd6	0.56632421	0.45144453	0.126	0.13	1
Slc52a3	0.56786692	0.3166494	0.013	0.015	1
B3galt6	0.57013217	0.63640389	0.013	0.012	1
H2-Ke6	0.5702356	0.31178254	0.085	0.09	1
Stk25	0.57149003	0.37691854	0.04	0.042	1
AI597479	0.57519604	0.31413542	0.017	0.018	1
Taf1b	0.57595297	0.29268144	0.036	0.038	1
Dcaf12	0.57650673	0.41500402	0.125	0.129	1
Dcp1a	0.57734573	0.47151638	0.061	0.06	1
Nprl2	0.57857388	0.26626412	0.015	0.016	1
Lin54	0.58025813	0.38020981	0.184	0.194	1
Smarcad1	0.58151595	0.34236175	0.118	0.12	1
Zfp51	0.58237685	0.36121696	0.025	0.027	1
Mettl13	0.58257735	0.25974422	0.021	0.02	1
Spryd3	0.58267801	0.52853559	0.027	0.029	1
Dazap1	0.58412218	0.30473939	0.157	0.17	1
Lpin1	0.5847764	0.42192754	0.056	0.055	1
Ing1	0.58636412	0.31675245	0.134	0.137	1
E130309D02R	0.58773936	0.38659276	0.048	0.051	1
Naxd	0.58889291	0.3621535	0.09	0.091	1
Klhl25	0.58933795	0.37202315	0.022	0.021	1
D17H6S53E	0.58979621	0.51321602	0.022	0.021	1
Rrm2	0.59054672	0.33235961	0.01	0.011	1
Arl13b	0.59113954	0.38603044	0.099	0.105	1
Snrnp40	0.59127373	0.25181769	0.093	0.099	1
Ctbp1	0.59160998	0.26652228	0.183	0.2	1
Med4	0.59323768	0.31672869	0.099	0.1	1
Med28	0.59572744	0.31053485	0.215	0.231	1
Setd6	0.59577973	0.36531668	0.014	0.015	1
Rgs3	0.59666232	0.42595466	0.033	0.032	1
Slc25a19	0.59821313	0.46150331	0.09	0.097	1
Chpf2	0.59852948	0.43122157	0.021	0.022	1
Bola1	0.60290007	0.37093516	0.04	0.042	1
Nek7	0.60344128	0.27629403	0.188	0.197	1
Rasa1	0.60644196	0.44903853	0.093	0.095	1
Ccne2	0.60674413	0.52148596	0.011	0.01	1
Ulk4	0.6093773	0.28623613	0.03	0.029	1
Setd1b	0.60963121	0.25921055	0.075	0.074	1

Hspbp1	0.61075025	0.3530343	0.051	0.054	1
Pip5k1a	0.6118689	0.29503005	0.164	0.17	1
Gtf2b	0.61213909	0.28203298	0.181	0.198	1
Cnot7	0.61431755	0.38198624	0.136	0.14	1
Nbeal2	0.61461054	0.4523822	0.029	0.028	1
Yipf4	0.61515441	0.37695556	0.162	0.171	1
Taf6l	0.61715505	0.33161252	0.176	0.193	1
Nck1	0.61871159	0.34628855	0.145	0.15	1
Oaz2	0.61889457	0.49933213	0.035	0.037	1
Sirt7	0.61911436	0.33759585	0.108	0.115	1
Dedd	0.62247143	0.46022804	0.046	0.045	1
Mapk9	0.62310508	0.35317021	0.049	0.052	1
Qrsl1	0.62390517	0.28814951	0.029	0.031	1
Dnm2	0.62488987	0.33689063	0.178	0.188	1
Zhx1	0.62508368	0.30992214	0.079	0.083	1
Bop1	0.62513858	0.43689999	0.05	0.053	1
Ccdc73	0.62515733	0.33044169	0.012	0.013	1
Nsmce3	0.62516799	0.36686922	0.071	0.075	1
Tnrc6a	0.62588322	0.27914491	0.195	0.204	1
Hnrnph3	0.62658154	0.29096458	0.129	0.132	1
C330027C09F	0.62716103	0.57465849	0.01	0.009	1
Zfp780b	0.62753519	0.33981487	0.021	0.022	1
Rrnad1	0.62828658	0.34087332	0.037	0.036	1
Ccng1	0.62956578	0.34595338	0.113	0.121	1
Psmg3	0.62980796	0.38035849	0.049	0.052	1
Panx1	0.62997741	0.34277867	0.065	0.064	1
G2e3	0.63087099	0.36410468	0.059	0.058	1
Hmgcr	0.63232384	0.27700549	0.194	0.212	1
Fcho1	0.6334393	0.43456871	0.035	0.035	1
Pramef8	0.63504679	0.27419772	0.033	0.035	1
Mettl17	0.63670058	0.51053831	0.02	0.019	1
Gcfc2	0.6379534	0.54306563	0.013	0.012	1
Pigo	0.64086693	0.51009788	0.02	0.019	1
Cops4	0.64156442	0.37520766	0.138	0.149	1
Nudcd2	0.64272722	0.43676252	0.07	0.074	1
Tmco6	0.64458158	0.43990784	0.019	0.018	1
Dnajc17	0.64628311	0.56693248	0.018	0.017	1
Atf5	0.64628738	0.46002399	0.02	0.021	1
Galnt6	0.64736821	0.43934553	0.082	0.083	1
Dcaf5	0.6476474	0.36353976	0.047	0.046	1
Cars	0.64802194	0.48534077	0.088	0.089	1
Golga1	0.64841104	0.37818882	0.015	0.016	1
Gm16973	0.64878343	0.42449486	0.014	0.014	1
Rnf20	0.64992321	0.35027023	0.156	0.163	1
Bcdin3d	0.6501881	0.3251324	0.017	0.016	1
Tsn	0.65055041	0.25002071	0.223	0.246	1
Edc4	0.65237296	0.41294177	0.067	0.067	1
Stim2	0.65273614	0.40989026	0.087	0.093	1

Mib2	0.65312645	0.39834906	0.01	0.011	1
Asb6	0.65319669	0.52808735	0.032	0.031	1
Camk2g	0.65365863	0.40453249	0.041	0.043	1
Zfp738	0.654057	0.41053863	0.034	0.033	1
Rab26	0.65424297	0.45485812	0.016	0.015	1
Cog4	0.65439754	0.32227176	0.049	0.051	1
Tigar	0.6567333	0.38310167	0.019	0.018	1
Chrac1	0.65766599	0.37199601	0.175	0.186	1
Zfp747	0.65862442	0.38966719	0.014	0.014	1
Ppat	0.65922133	0.40432426	0.043	0.046	1
Nmi	0.66145633	0.32092404	0.12	0.123	1
Zmynd19	0.66172861	0.60811395	0.026	0.025	1
Timm17a	0.66234595	0.38295958	0.156	0.171	1
Zc3h14	0.66324682	0.29019482	0.116	0.119	1
Cox15	0.66405951	0.5674348	0.019	0.018	1
Exoc6	0.66442771	0.30539264	0.046	0.048	1
Snrpa1	0.66461205	0.40122554	0.13	0.14	1
1110004E09R	0.66526223	0.37078521	0.031	0.032	1
Fbxw17	0.66529049	0.54657928	0.023	0.024	1
Rai1	0.66738167	0.29512425	0.055	0.057	1
Trib3	0.66823899	0.47109219	0.014	0.015	1
Map9	0.66850281	0.6553735	0.01	0.009	1
Otud3	0.66976888	0.51217704	0.014	0.015	1
Clec16a	0.67124868	0.43747205	0.016	0.017	1
Dtwd1	0.67173953	0.29493094	0.011	0.012	1
Ppm1b	0.67414411	0.27755881	0.128	0.131	1
Zmat3	0.67417379	0.39784647	0.015	0.016	1
Gdap2	0.67469933	0.58608628	0.045	0.045	1
Apaf1	0.67628137	0.32038827	0.164	0.172	1
Tradd	0.6769157	0.43421727	0.029	0.031	1
Ybey	0.67786803	0.42873747	0.01	0.01	1
Zfand2b	0.67928838	0.30303129	0.077	0.081	1
Eipr1	0.67949671	0.44538075	0.069	0.072	1
Mrps30	0.68212665	0.31630948	0.076	0.08	1
Cdkal1	0.6827327	0.3636492	0.044	0.044	1
Klhl21	0.68296515	0.35336702	0.03	0.032	1
Zfp869	0.6832983	0.38603543	0.018	0.017	1
Mrpl35	0.68582543	0.48310193	0.094	0.097	1
Zbtb5	0.68680992	0.30129135	0.027	0.028	1
Itfg2	0.6875019	0.36745582	0.029	0.031	1
Brms1	0.68984517	0.51398168	0.063	0.066	1
Iqcc	0.69013654	0.40643217	0.02	0.02	1
Cnot3	0.69071236	0.26890182	0.165	0.173	1
Sptan1	0.69128062	0.26547478	0.166	0.173	1
Tspan14	0.69168072	0.3667744	0.097	0.099	1
Cog2	0.69497405	0.47370269	0.073	0.074	1
Sgcb	0.69504733	0.5476377	0.021	0.021	1
Txlng	0.69585258	0.29613071	0.046	0.048	1

Zfp113	0.6962719	0.51283532	0.014	0.014	1
Smim13	0.69763076	0.40073231	0.03	0.031	1
Erg28	0.69774726	0.45546581	0.075	0.076	1
Golim4	0.69832064	0.42572674	0.057	0.059	1
Kat6a	0.69946539	0.29582862	0.201	0.212	1
Zfp369	0.70155699	0.26592814	0.025	0.026	1
Smug1	0.70185991	0.32844692	0.02	0.019	1
Iars	0.7024326	0.42226841	0.084	0.086	1
Adss	0.70306081	0.30606619	0.233	0.251	1
Aamp	0.70474221	0.28170316	0.265	0.292	1
Paxx	0.70613095	0.36631104	0.056	0.056	1
Zbtb17	0.70622235	0.47412587	0.047	0.049	1
Ccr9	0.70721668	0.58587054	0.025	0.025	1
Pcgf6	0.70922862	0.3127385	0.017	0.016	1
Gramd1b	0.71043616	0.26612919	0.08	0.084	1
Heatr5b	0.71312319	0.34208714	0.027	0.026	1
Topbp1	0.71412236	0.35814985	0.093	0.094	1
Il1rl2	0.71429661	0.37034547	0.009	0.01	1
Luc7l	0.71443784	0.33793394	0.124	0.128	1
Aktip	0.71670814	0.26654985	0.023	0.024	1
Qsox1	0.71702093	0.32700258	0.06	0.062	1
Pgrmc2	0.71792011	0.49947209	0.08	0.081	1
Polr2d	0.71803691	0.44744483	0.051	0.053	1
Rfx1	0.71813541	0.42294276	0.045	0.045	1
Slc7a6	0.71909008	0.39258024	0.019	0.02	1
Pelp1	0.71990351	0.45459279	0.037	0.038	1
Stk11	0.72066033	0.2662053	0.213	0.227	1
Mrpl58	0.7211597	0.48434859	0.113	0.121	1
Tmem126b	0.72431758	0.38996672	0.025	0.025	1
Stambp	0.72476605	0.48354904	0.043	0.045	1
Spsb1	0.72597249	0.59466394	0.026	0.025	1
Bcl2l13	0.72656953	0.37393043	0.048	0.05	1
Rnaseh1	0.7270756	0.35190999	0.025	0.026	1
Ubxn8	0.72769312	0.40425751	0.048	0.05	1
Fbxl6	0.7281407	0.45791539	0.044	0.046	1
Zfp444	0.72838541	0.39401358	0.016	0.017	1
Mtif3	0.73070051	0.47247021	0.025	0.027	1
Kdm3a	0.73114687	0.31499369	0.097	0.103	1
Lonp2	0.73189386	0.33697715	0.07	0.07	1
Pgam5	0.73227143	0.35074303	0.038	0.039	1
Adprhl2	0.73227711	0.39089729	0.034	0.034	1
Wrap73	0.73268546	0.53060756	0.021	0.02	1
Cherp	0.73545526	0.3609153	0.067	0.071	1
Ankrd50	0.73614288	0.39351892	0.046	0.046	1
Rbbp5	0.73659701	0.34400124	0.042	0.042	1
Hpf1	0.73673358	0.31611415	0.176	0.191	1
Tbc1d12	0.73768735	0.41050616	0.012	0.012	1
Gm6133	0.73802019	0.52256686	0.029	0.03	1

2810405F17Ri	0.73848872	0.35276844	0.016	0.015	1
Prpf4	0.73867304	0.33452913	0.091	0.092	1
Nol9	0.73936537	0.26837049	0.037	0.039	1
B230354K17R	0.73975331	0.36612928	0.014	0.014	1
Bud31	0.73991838	0.2694746	0.199	0.219	1
Ulbp1	0.74105403	0.33144899	0.009	0.01	1
Uspl1	0.74145644	0.2535421	0.089	0.09	1
Sap30bp	0.74206453	0.44691548	0.074	0.078	1
Polr3k	0.74333134	0.51218929	0.065	0.066	1
Aagab	0.74393453	0.26818591	0.066	0.069	1
Sntb2	0.74407573	0.47809798	0.049	0.048	1
Sec22a	0.74564875	0.44766179	0.026	0.027	1
Cmip	0.74577324	0.28478515	0.254	0.284	1
Slc35e1	0.74711708	0.4803256	0.077	0.079	1
Mtg2	0.74821109	0.45077417	0.019	0.018	1
Rit1	0.74926171	0.5088552	0.042	0.042	1
Ebna1bp2	0.75050666	0.34462631	0.129	0.139	1
Med10	0.75056268	0.46554858	0.145	0.153	1
Slc45a4	0.75127975	0.44239609	0.035	0.036	1
C2cd2l	0.75145012	0.60124939	0.018	0.018	1
Crip2	0.75245111	0.54010803	0.018	0.017	1
Sec23ip	0.75447484	0.42284484	0.058	0.058	1
Mark2	0.75594827	0.27371519	0.204	0.217	1
Yipf6	0.75631863	0.37101105	0.064	0.065	1
Ppp1r37	0.75645148	0.36545716	0.045	0.046	1
Hist4h4	0.75745736	0.3438084	0.017	0.018	1
Polr3c	0.75850893	0.49933444	0.081	0.083	1
Sft2d3	0.75985203	0.3228501	0.047	0.047	1
Prpf8	0.76086042	0.25830075	0.193	0.209	1
3830406C13R	0.76165156	0.43290335	0.086	0.088	1
Ankra2	0.76206526	0.27362233	0.064	0.067	1
Wdr47	0.76219378	0.48922976	0.022	0.021	1
Fmr1	0.7624134	0.25842409	0.168	0.175	1
Klhl15	0.76622909	0.26699123	0.029	0.03	1
Hic2	0.76811425	0.71213939	0.016	0.016	1
Csnk1d	0.76902846	0.28182572	0.229	0.249	1
Brpf3	0.76908264	0.26975028	0.02	0.021	1
Gm26982	0.77275684	0.44891605	0.026	0.026	1
Fam192a	0.77555679	0.38235221	0.104	0.111	1
Ubac1	0.77555894	0.27426682	0.034	0.035	1
Rnf10	0.77652588	0.31614569	0.201	0.221	1
Anapc2	0.77692843	0.4039123	0.055	0.057	1
Ttc33	0.77790399	0.37115564	0.054	0.056	1
Actr5	0.77955641	0.40192534	0.047	0.047	1
Rbm41	0.78008353	0.45485237	0.023	0.022	1
Usp19	0.78053674	0.2838299	0.179	0.193	1
Faap100	0.781664	0.45084181	0.05	0.052	1
Minpp1	0.78216212	0.34312505	0.025	0.026	1

Cdk5rap1	0.78294002	0.44823806	0.011	0.012	1
Atxn7l2	0.78399336	0.38968361	0.015	0.016	1
Tada2b	0.78444692	0.32286794	0.024	0.025	1
Klf16	0.78528302	0.45625526	0.026	0.026	1
Taf8	0.78528592	0.2831951	0.023	0.024	1
Wdr44	0.78629199	0.33644509	0.062	0.062	1
Brf2	0.78739584	0.43978816	0.018	0.017	1
Dctn3	0.78806109	0.36763368	0.248	0.276	1
Svil	0.78830108	0.39130028	0.098	0.1	1
Sh3glb2	0.78855257	0.56453963	0.042	0.042	1
Ogfod2	0.7909825	0.43576127	0.025	0.025	1
Cenpv	0.79198865	0.52921144	0.017	0.017	1
Asb1	0.7932728	0.48921943	0.014	0.015	1
Lias	0.79561332	0.37706457	0.061	0.061	1
Stx6	0.79647388	0.29273464	0.121	0.125	1
Sugp1	0.7984603	0.44415116	0.059	0.06	1
Gpr34	0.80214235	0.40989257	0.035	0.035	1
Ranbp9	0.80324907	0.3690679	0.11	0.113	1
Exoc6b	0.80339271	0.48092003	0.042	0.044	1
Tex30	0.80342132	0.34717421	0.053	0.053	1
Riox1	0.80429199	0.43028761	0.025	0.025	1
Rnf103	0.80514019	0.34475453	0.057	0.057	1
Rfwd3	0.80561442	0.32987998	0.036	0.038	1
L3mbtl2	0.80639925	0.27483143	0.017	0.018	1
Sra1	0.80655824	0.27813838	0.227	0.25	1
Inip	0.80747126	0.30719173	0.067	0.07	1
Dynlrb1	0.80805197	0.3276313	0.253	0.281	1
Cry2	0.8083238	0.27761627	0.022	0.023	1
E530011L22Ri	0.80858298	0.44698448	0.03	0.03	1
Slu7	0.809408	0.33515709	0.171	0.182	1
Commd2	0.81121096	0.43133191	0.077	0.079	1
Taf9b	0.81238585	0.26646441	0.016	0.017	1
Smg5	0.81439233	0.28306656	0.099	0.104	1
Clstn1	0.81539692	0.33883605	0.018	0.019	1
Cd3eap	0.81710775	0.35640684	0.022	0.023	1
Ppp1r14c	0.81734472	0.26259146	0.009	0.01	1
Ide	0.81784754	0.34317451	0.072	0.074	1
4930430F08Ri	0.81787763	0.41343294	0.01	0.01	1
Smg8	0.81789681	0.34839742	0.028	0.029	1
Rpl27-ps3	0.81799458	0.39119907	0.093	0.098	1
Plcl2	0.81975289	0.31463682	0.081	0.084	1
Zmat2	0.82116086	0.26229558	0.276	0.305	1
Smim10l1	0.82189397	0.50402334	0.084	0.089	1
Ttc38	0.82276595	0.35426212	0.011	0.012	1
Anks3	0.82341055	0.2724222	0.037	0.038	1
Zfp810	0.82561587	0.26111275	0.011	0.012	1
Malsu1	0.8257449	0.46476542	0.113	0.118	1
Blzf1	0.8264475	0.40898684	0.04	0.041	1

Gm20300	0.82726087	0.32897737	0.012	0.013	1
Dars2	0.82889084	0.47964544	0.027	0.027	1
Map3k10	0.82986131	0.31077658	0.012	0.013	1
Surf6	0.830531	0.26717452	0.054	0.054	1
Usp39	0.83184484	0.35721608	0.058	0.06	1
March5	0.83287371	0.40654154	0.167	0.182	1
Txndc11	0.83317271	0.32145677	0.135	0.144	1
Atg4d	0.83348614	0.34078265	0.044	0.046	1
Rnf113a2	0.83373585	0.32528347	0.055	0.057	1
Pop4	0.83374737	0.4316984	0.063	0.064	1
Naa16	0.83552689	0.34110761	0.043	0.045	1
Rtl8b	0.83715563	0.61107087	0.02	0.021	1
Sin3a	0.83726464	0.28251407	0.083	0.087	1
Rhebl1	0.84059835	0.37893533	0.011	0.011	1
Mcm9	0.84158006	0.37600974	0.04	0.04	1
Lzts1	0.84337764	0.55602236	0.009	0.01	1
Rwdd2b	0.84427755	0.47653047	0.011	0.011	1
Cabin1	0.84622009	0.31439113	0.097	0.099	1
Cdca7l	0.84633574	0.33770176	0.02	0.021	1
Pias3	0.84679228	0.3682376	0.018	0.018	1
Mrpl49	0.84685389	0.40893086	0.038	0.038	1
Peak1	0.8479461	0.26181403	0.144	0.152	1
Gtf3c1	0.84860992	0.33146876	0.066	0.068	1
Krtcap3	0.8489425	0.35620077	0.012	0.011	1
Zfp182	0.84987488	0.28983905	0.022	0.022	1
1700052K11R	0.85019384	0.30724699	0.01	0.01	1
Coq5	0.855111	0.30960215	0.051	0.053	1
Smarcd1	0.85729745	0.35310976	0.071	0.073	1
Kcnc1	0.85828725	0.45152541	0.011	0.011	1
Tars2	0.85883917	0.26565526	0.028	0.029	1
Cdyl2	0.86024656	0.37828039	0.013	0.013	1
Rpa1	0.86025702	0.27938325	0.058	0.06	1
Nsrp1	0.86079918	0.31301907	0.178	0.189	1
Uba2	0.86092234	0.37180448	0.104	0.109	1
Ints5.1	0.86154963	0.37206468	0.019	0.019	1
E2f4	0.86257716	0.45508891	0.075	0.077	1
Zcrb1	0.86272001	0.28235693	0.269	0.304	1
Bola3	0.86577199	0.32484636	0.139	0.147	1
Tmem115	0.86642653	0.5188266	0.032	0.032	1
Utf1	0.86673487	0.53386868	0.014	0.014	1
Wrap53	0.86757726	0.42090324	0.012	0.011	1
Pkd1	0.87060156	0.31005717	0.048	0.048	1
Zfp871	0.8709648	0.28071312	0.165	0.176	1
Cnep1r1	0.8713976	0.50307818	0.057	0.058	1
Smim8	0.87212897	0.39508117	0.054	0.054	1
Lyrm4	0.8722334	0.51627299	0.02	0.021	1
Gm9844	0.8723501	0.6258706	0.03	0.03	1
Bhlhb9	0.87419267	0.27922217	0.014	0.014	1

Phf13	0.87444647	0.40525151	0.027	0.027	1
Ccdc174	0.87643613	0.31597835	0.176	0.187	1
Dnajc30	0.87921159	0.55120061	0.045	0.045	1
Yipf3	0.88095198	0.42539301	0.147	0.158	1
Rnf38	0.88767194	0.31211796	0.073	0.076	1
Rab2b	0.88768483	0.37036622	0.042	0.042	1
Dolk	0.8880534	0.3511343	0.013	0.014	1
Tcaim	0.88861844	0.3634067	0.018	0.018	1
2310033P09R	0.89016149	0.40827358	0.088	0.091	1
Ints7	0.89096368	0.252075	0.057	0.058	1
Aimp2	0.89212523	0.51120544	0.028	0.029	1
Fbxo25	0.89345823	0.45426823	0.014	0.015	1
9330020H09R	0.8946202	0.33381834	0.021	0.021	1
Mrps31	0.89578347	0.35650028	0.038	0.038	1
Pard6a	0.89586693	0.46967463	0.027	0.028	1
Hes6	0.89651939	0.35844508	0.028	0.028	1
Orc2	0.89809137	0.33716798	0.048	0.049	1
Strn	0.89809281	0.39251044	0.059	0.061	1
Edrf1	0.89906146	0.34619275	0.052	0.053	1
Herpud2	0.89914138	0.29378292	0.106	0.111	1
Tor1aip2	0.89950726	0.3181793	0.121	0.126	1
Mir17hg	0.90008729	0.41511559	0.019	0.018	1
Nudt21	0.90180881	0.31957418	0.199	0.217	1
Ift27	0.90267019	0.30692755	0.028	0.029	1
Sec24c	0.90392469	0.35158645	0.102	0.107	1
Rbm15b	0.90532969	0.37368284	0.043	0.043	1
Smpdl3a	0.90640615	0.35481104	0.172	0.185	1
Ikzf5	0.90726453	0.30434613	0.093	0.097	1
Kcnab2	0.90806214	0.29108753	0.052	0.054	1
Maz	0.90816638	0.39333591	0.081	0.083	1
Trim21	0.90822	0.38025107	0.018	0.018	1
Adat2	0.90866042	0.50629507	0.023	0.023	1
Smarca2	0.91013833	0.28291602	0.138	0.144	1
Wdr82	0.91023595	0.33076221	0.099	0.102	1
Mrps22	0.91098414	0.55322681	0.027	0.028	1
Tysnd1	0.91286117	0.49422347	0.017	0.017	1
Katna1	0.91318143	0.3379574	0.135	0.143	1
Ctr9	0.91324467	0.32118819	0.087	0.09	1
Fam210a	0.91326309	0.36084437	0.031	0.031	1
Sos1	0.91411919	0.37628521	0.053	0.054	1
Mta1	0.91577386	0.43855556	0.073	0.076	1
Ccdc66	0.91631345	0.28283725	0.023	0.023	1
Zfp942	0.91655785	0.30942082	0.05	0.051	1
Spryd4	0.91688831	0.34960245	0.013	0.014	1
Mapre3	0.91795852	0.41685774	0.016	0.016	1
Mtmr12	0.91864615	0.35853231	0.086	0.089	1
Rrm1	0.91924582	0.26995069	0.058	0.058	1
Fam53c	0.92001974	0.37851135	0.059	0.061	1

Prdm2	0.92074719	0.30410842	0.133	0.139	1
Tubb4b	0.92295284	0.32764837	0.129	0.136	1
Ptger2	0.92480182	0.32519257	0.026	0.026	1
Pofut2	0.92489198	0.3978463	0.18	0.195	1
Mfsd8	0.92820246	0.45599496	0.021	0.021	1
Pin1	0.92848803	0.44053649	0.138	0.147	1
Ostc	0.93182886	0.30749292	0.264	0.293	1
Nop9	0.93232777	0.38867089	0.068	0.069	1
Faap20	0.93721263	0.44404482	0.045	0.046	1
Zfp958	0.93752943	0.43428236	0.032	0.032	1
Lsm3	0.93772411	0.32707266	0.151	0.16	1
Ncoa3	0.94009782	0.29644418	0.143	0.151	1
Osbpl2	0.94025513	0.3380378	0.037	0.037	1
Rnf168	0.94210197	0.43129606	0.048	0.049	1
Arl6ip4	0.94257293	0.44637746	0.099	0.104	1
Zfp788	0.94432524	0.38251123	0.019	0.018	1
Pdha1	0.94625752	0.38702093	0.138	0.147	1
Atrn	0.94683309	0.30653979	0.034	0.034	1
Rnpc3	0.94689012	0.39437462	0.097	0.101	1
AW549877	0.94803018	0.26540327	0.096	0.099	1
2700062C07R	0.94807528	0.40366288	0.029	0.029	1
Dhx8	0.94988761	0.32459159	0.13	0.136	1
Gcc1	0.95055597	0.33458191	0.041	0.041	1
Clip1	0.95115563	0.34583462	0.139	0.148	1
Nde1	0.95158747	0.51199641	0.037	0.038	1
Hars2	0.95198864	0.40361364	0.025	0.025	1
Alg10b	0.95238347	0.36721864	0.043	0.043	1
Magoh	0.95279338	0.33770719	0.221	0.244	1
Gm44777	0.9542188	0.31823901	0.011	0.011	1
Fkbp5	0.9550281	0.38518634	0.032	0.032	1
Fam8a1	0.95603284	0.3079227	0.045	0.046	1
Srp68	0.95784913	0.44381631	0.078	0.081	1
Eral1	0.95800502	0.60003464	0.015	0.015	1
Trim36	0.95905349	0.58852649	0.03	0.031	1
Abhd13	0.96234785	0.42153125	0.044	0.044	1
Faim	0.96342942	0.25540608	0.12	0.125	1
Pyroxd1	0.96344445	0.383975	0.047	0.048	1
Eif2b1	0.9638011	0.47254584	0.029	0.03	1
Adamts10	0.9646086	0.36760071	0.025	0.025	1
Timm21	0.96520967	0.45336149	0.024	0.024	1
Rbm42	0.96540671	0.38135652	0.185	0.2	1
Prps1l3	0.9659529	0.31530618	0.021	0.022	1
Zbtb11	0.96710665	0.28998898	0.117	0.122	1
Dynll2	0.96866372	0.42312215	0.125	0.132	1
Bin2	0.96934978	0.43464135	0.127	0.135	1
Rtel1	0.96935228	0.33224921	0.011	0.011	1
Cep295	0.96990273	0.31684067	0.07	0.072	1
Mettl7a1	0.97094187	0.72942449	0.015	0.015	1

Bod1	0.9712261	0.47579527	0.031	0.031	1
Atf2	0.97263954	0.30757644	0.133	0.14	1
Top3a	0.97290771	0.27138931	0.017	0.017	1
Tsg101	0.97293555	0.37619202	0.123	0.129	1
Npm3	0.97470479	0.33759469	0.171	0.183	1
Gtpbp8	0.97490298	0.3427435	0.019	0.019	1
Mut	0.97758005	0.31191347	0.025	0.025	1
Dusp19	0.97769119	0.48688825	0.019	0.019	1
Tagap1	0.97822111	0.3226676	0.024	0.024	1
Parg	0.98095464	0.28180106	0.09	0.093	1
Tbp	0.98120023	0.30664958	0.035	0.035	1
Mrs2	0.98186934	0.40699683	0.04	0.041	1
Wbp1l	0.98210081	0.30904042	0.083	0.085	1
Rexo1	0.9836228	0.32435319	0.137	0.144	1
Rngtt	0.98382279	0.37001273	0.081	0.083	1
Trappc8	0.98392979	0.33983454	0.084	0.086	1
Ddx20	0.9844992	0.38766063	0.029	0.029	1
Nsdhl	0.98477929	0.59315986	0.025	0.026	1
Unc13d	0.98905961	0.37120393	0.04	0.04	1
Zfp317	0.98952027	0.33972899	0.021	0.021	1
Rps6kb1	0.99000158	0.26421505	0.161	0.17	1
Ercc2	0.99161307	0.36044509	0.022	0.022	1
Nxt1	0.99375033	0.4668915	0.066	0.068	1
Dcun1d3	0.99416702	0.4654624	0.022	0.022	1
Pdp1	0.99426219	0.32892243	0.026	0.026	1
Zscan21	0.99457732	0.39480765	0.02	0.02	1
Zzef1	0.99484708	0.32846501	0.113	0.118	1
Vbp1	0.99601043	0.29277787	0.18	0.193	1
Capn10	0.99710099	0.36742991	0.013	0.014	1
Habp4	0.9971665	0.44662744	0.013	0.013	1
Cramp1l	0.99869996	0.43067837	0.053	0.055	1

CD4 Th2

Gene	p_val	avg_log2FC	pct.1	pct.2	p_val_adj
Ptpn13	0	4.98491727	0.802	0.058	0
Hlf	0	4.70641309	0.819	0.083	0
Gata3	0	3.37259504	0.813	0.123	0
Ipmk	0	3.45459141	0.703	0.127	0
Tnfsf8	0	3.78998292	0.661	0.11	0
Calca	0	4.74905582	0.559	0.041	0
Uhrf2	0	2.80661745	0.836	0.327	0
Il17rb	0	6.14826932	0.511	0.011	0
Tmem64	0	2.15543002	0.71	0.22	0
Nr3c1	0	2.10809075	0.888	0.421	0
Maf	0	1.69342433	0.773	0.313	0
Selenot	0	2.62058432	0.766	0.316	0
Nrip1	0	2.33177004	0.918	0.47	0
Rora	0	1.40599705	0.876	0.429	0
Il17a	0	5.06115787	0.462	0.036	0
AC149090.1	0	2.58762631	0.694	0.272	0
Epas1	0	3.03575726	0.491	0.083	0
Lmo4	0	2.30902711	0.699	0.301	0
Il17f	0	6.04036802	0.398	0.017	0
Il4	0	4.74225705	0.379	0.013	0
Ar	0	2.46524559	0.456	0.093	0
Emb	0	1.51972355	0.97	0.609	0
Furin	0	1.83248543	0.765	0.412	0
Tox	0	2.86210626	0.421	0.074	0
Socs2	0	3.00851792	0.42	0.074	0
Trps1	0	2.12778704	0.633	0.295	0
Il5	0	3.08922573	0.36	0.022	0
Il13	0	4.51404228	0.331	0.013	0
Fam46a	0	2.50489737	0.553	0.236	0
Actn2	0	1.88343071	0.435	0.123	0
Serp1	0	2.17696332	0.902	0.592	0
Areg	0	1.95371719	0.427	0.118	0
Siah2	0	2.89648376	0.406	0.12	0
Gcnt1	0	3.47219084	0.323	0.041	0
Pcsk1	0	6.18295684	0.283	0.005	0
Il1rl1	0	3.0574165	0.317	0.043	0
Rad50	0	2.90034339	0.333	0.072	0
Spry1	0	4.57891451	0.268	0.015	0
Dst	0	2.86558495	0.303	0.072	0
Ccr4	0	2.90216242	0.271	0.044	0
Lgals7	0	4.44520033	0.227	0.016	0
1700061F12Ri	0	7.46702062	0.204	0.002	0
Tox2	0	3.03677399	0.237	0.035	0
Ldlrad4	0	2.89802839	0.231	0.043	0
Egln3	0	2.84449213	0.217	0.035	0
Chdh	0	5.55206138	0.185	0.005	0

Stab2	0	5.04633173	0.169	0.008	0
Dkk3	0	4.36179469	0.153	0.01	0
Il9r	0	4.35576112	0.151	0.008	0
Edil3	0	4.8594475	0.15	0.007	0
9230102O04R	0	5.40330168	0.142	0.004	0
Tph1	0	6.62082274	0.138	0.002	0
Pde7b	0	3.40305542	0.15	0.018	0
Gm14718	0	4.47110286	0.139	0.01	0
Mras	0	4.78797374	0.127	0.009	0
Amph	0	5.54954201	0.082	0.002	0
Grp	0	8.69774036	0.053	0	0
mt-Atp6	0	0.67236346	0.998	0.964	0
mt-Co1	0	0.58534035	0.999	0.971	0
Apbb2	2.445E-288	4.13138478	0.083	0.006	5.365E-284
Ptgir	4.497E-284	3.51045687	0.104	0.01	9.867E-280
Cd40lg	1.546E-279	2.57481186	0.322	0.088	3.392E-275
Zeb2	3.045E-279	1.36328209	0.748	0.409	6.681E-275
mt-Co3	7.854E-278	0.57230167	0.997	0.967	1.723E-273
Elf1	2.715E-274	1.41061839	0.743	0.456	5.956E-270
Aebp2	1.012E-271	1.22298865	0.806	0.516	2.219E-267
Gm42418	6.656E-269	0.50487189	1	1	1.46E-264
Sik2	5.6E-266	2.37115443	0.334	0.098	1.229E-261
Rbpj	1.42E-265	1.55662937	0.71	0.429	3.114E-261
Spcs3	5.406E-261	2.18006948	0.44	0.172	1.186E-256
Cnnm2	3.627E-254	2.52944312	0.231	0.052	7.958E-250
Plcb4	4.608E-254	2.77291703	0.194	0.037	1.011E-249
Rpl37a	4.137E-252	0.46565953	1	0.983	9.076E-248
Ikzf3	1.273E-251	1.51732495	0.605	0.286	2.793E-247
Ahnak	1.525E-248	0.96210099	0.851	0.533	3.346E-244
Myo6	3.909E-248	3.57169996	0.123	0.016	8.576E-244
mt-Cytb	2.544E-244	0.68618872	0.989	0.93	5.582E-240
Gm28050	1.097E-241	6.34725845	0.035	0.001	2.406E-237
Tnfaip3	1.257E-238	1.02965333	0.899	0.664	2.757E-234
Palld	3.908E-235	3.43486444	0.146	0.023	8.574E-231
Tespa1	1.696E-234	2.05278938	0.311	0.091	3.721E-230
Msi2	2.188E-231	1.72956026	0.491	0.213	4.801E-227
Samd11	9.177E-222	2.40390034	0.285	0.083	2.013E-217
Zrsr1	3.924E-221	2.71248322	0.195	0.042	8.609E-217
Fosb	5.719E-221	1.38337362	0.609	0.31	1.255E-216
Kit	1.136E-219	1.80895816	0.384	0.139	2.492E-215
Bcl11b	2.411E-218	1.60852932	0.421	0.159	5.29E-214
Paxbp1	7.141E-215	1.74515691	0.481	0.217	1.567E-210
Atxn1	2.833E-212	1.66546547	0.448	0.186	6.216E-208
Znrf1	3.905E-211	2.05859996	0.353	0.126	8.568E-207
Adgrg7	7.912E-210	6.51192139	0.025	0	1.736E-205
Kmt2a	5.692E-208	1.5898286	0.59	0.319	1.249E-203
Dennd4a	8.327E-208	1.02243343	0.87	0.72	1.827E-203
Tagln2	1.423E-204	1.18831364	0.766	0.511	3.123E-200

4930503L19Ri	6.585E-202	2.20642876	0.26	0.075	1.445E-197
mt-Nd4	4.572E-201	0.62010239	0.976	0.906	1.003E-196
Ero1l	5.813E-201	2.1324575	0.408	0.171	1.275E-196
mt-Co2	3.077E-199	0.46395719	0.995	0.964	6.75E-195
Malat1	8.894E-193	0.55485182	1	0.999	1.951E-188
Ncor1	3.442E-192	1.14368557	0.727	0.499	7.551E-188
Acvr2a	5.402E-188	2.50214501	0.194	0.047	1.185E-183
Akap13	1.845E-187	0.85293854	0.896	0.75	4.047E-183
Chn2	3.461E-187	2.64990444	0.166	0.036	7.593E-183
Rasgrp1	2.688E-185	1.72861621	0.383	0.152	5.898E-181
Bhlhe40	2.089E-184	1.08434726	0.801	0.597	4.582E-180
Tet2	2.445E-184	1.72418854	0.502	0.265	5.364E-180
Cd3g	6.623E-182	1.18758489	0.605	0.307	1.453E-177
Nsg2	2.163E-179	2.69850791	0.123	0.021	4.746E-175
Sept11	6.603E-178	1.65562237	0.451	0.213	1.449E-173
Nlrc3	2.171E-175	2.47351267	0.169	0.038	4.763E-171
Samsn1	7.711E-174	1.04666301	0.748	0.517	1.692E-169
Ddx5	3.748E-166	0.56755438	0.983	0.952	8.222E-162
Lgals1	6.092E-164	0.94952212	0.745	0.506	1.336E-159
Mns1	4.799E-163	3.09959402	0.105	0.017	1.053E-158
Itk	2.408E-161	1.18301169	0.576	0.31	5.283E-157
Pxdc1	5.127E-161	1.38823928	0.373	0.153	1.125E-156
Thy1	6.258E-161	1.28943398	0.494	0.238	1.373E-156
Itm2b	5.531E-159	0.706236	0.892	0.718	1.213E-154
Itga2	1.413E-155	3.43588135	0.069	0.008	3.101E-151
Ern1	8.512E-150	1.32851464	0.482	0.249	1.867E-145
Gadd45b	8.729E-149	1.42242185	0.667	0.464	1.915E-144
Phldb3	1.186E-148	3.14940502	0.079	0.011	2.602E-144
Cd44	1.995E-148	0.71462698	0.781	0.53	4.376E-144
Jak2	3.828E-147	1.19221281	0.648	0.443	8.397E-143
Sh3bgrl3	6.333E-146	0.88500884	0.871	0.706	1.389E-141
mt-Nd1	1.097E-145	0.53151738	0.984	0.91	2.406E-141
Cpm	1.516E-145	2.00541604	0.204	0.06	3.325E-141
Pgm2l1	2.326E-144	1.80603188	0.279	0.102	5.102E-140
Rplp1	2.015E-143	0.38409029	1	0.982	4.42E-139
Sec11c	2.782E-143	1.35299577	0.582	0.388	6.104E-139
Cd200r1	7.858E-141	2.19080074	0.162	0.042	1.724E-136
Tmem30a	8.784E-140	1.37930477	0.516	0.308	1.927E-135
Tpbgl	5.642E-139	3.11565229	0.059	0.006	1.238E-134
Skap1	5.244E-138	1.27427476	0.45	0.219	1.151E-133
Dennd5a	2.427E-137	1.51685577	0.445	0.237	5.324E-133
Rb1	3.218E-137	1.77529568	0.328	0.141	7.06E-133
mt-Nd2	1.257E-135	0.64799862	0.89	0.775	2.758E-131
Rnf128	2.408E-135	2.84050221	0.076	0.011	5.283E-131
Hspa2	2.709E-135	2.63301602	0.134	0.031	5.944E-131
Eif4g2	2.711E-135	0.89028419	0.795	0.667	5.948E-131
Ttc3	5.081E-135	1.88312938	0.269	0.101	1.115E-130
Ccdc88c	8.359E-135	1.47723826	0.345	0.147	1.834E-130

Trgv2	4.208E-134	3.13117614	0.073	0.01	9.232E-130
Tmsb4x	2.548E-133	0.36906968	0.999	0.997	5.59E-129
Caly	5.293E-133	6.71530109	0.018	0	1.161E-128
Dusp10	1.144E-131	1.59113078	0.287	0.111	2.51E-127
Atp2b1	1.58E-130	0.93420525	0.768	0.595	3.466E-126
Tmem45a	4.14E-129	5.07693202	0.024	0.001	9.082E-125
Senp6	1.16E-128	1.20682998	0.558	0.352	2.546E-124
Hivep2	4.415E-128	1.30697464	0.503	0.299	9.686E-124
Gpx4	1.805E-127	1.11678601	0.68	0.531	3.959E-123
Hacd3	9.009E-127	1.66055809	0.269	0.103	1.976E-122
Inpp4b	1.81E-125	1.44948398	0.328	0.14	3.97E-121
Nr4a1	6.581E-124	0.98910007	0.576	0.353	1.444E-119
Tlk1	1.061E-123	1.63436996	0.358	0.172	2.328E-119
Bmpr2	7.723E-123	2.44002926	0.197	0.065	1.694E-118
D430036J16Ri	3.951E-118	4.59633553	0.024	0.001	8.669E-114
Cd28	5.806E-118	1.33154054	0.509	0.305	1.274E-113
Rgs2	1.536E-117	0.97424172	0.676	0.479	3.371E-113
Zdhhc23	3.995E-115	2.18810846	0.157	0.045	8.764E-111
Mlf1	9.289E-115	3.589094	0.049	0.005	2.038E-110
Nek7	1.166E-114	1.568396	0.371	0.189	2.559E-110
Irf2bp2	4.559E-114	1.0264949	0.614	0.417	1E-109
Spock2	2.469E-113	2.50641881	0.082	0.014	5.416E-109
Nfkbiz	5.512E-113	0.85373447	0.653	0.452	1.209E-108
Fam107b	1.053E-112	0.8716442	0.724	0.558	2.311E-108
C030034L19R	1.211E-112	3.76732292	0.035	0.003	2.658E-108
Myo1b	6.148E-112	3.6179332	0.064	0.009	1.349E-107
Galc	3.588E-111	2.62796636	0.139	0.038	7.871E-107
Htr1b	1.541E-110	3.9006211	0.033	0.002	3.38E-106
Grin2d	2.846E-110	4.80992872	0.023	0.001	6.244E-106
Smco4	1.35E-107	1.59896561	0.259	0.106	2.962E-103
Gpr45	2.707E-107	3.95121264	0.032	0.002	5.939E-103
Nav2	3.798E-107	1.74638708	0.157	0.047	8.332E-103
Robo1	7.169E-106	4.72923104	0.02	0.001	1.573E-101
Ramp3	1.146E-105	1.00407929	0.583	0.378	2.513E-101
Aff4	6.304E-105	1.16254182	0.522	0.342	1.383E-100
Ppp2r3a	2.793E-103	2.58945996	0.105	0.025	6.128E-99
Ldha	1.546E-102	1.24979457	0.624	0.487	3.3927E-98
Ccdc184	6.164E-102	1.62019178	0.132	0.036	1.3524E-97
Nfkb1	9.764E-102	0.83645277	0.761	0.639	2.1421E-97
Prc1	1.1592E-97	2.51175099	0.118	0.031	2.5431E-93
Nabp1	1.1416E-96	0.85981239	0.569	0.372	2.5046E-92
Herc1	1.7901E-96	1.22573593	0.418	0.239	3.9272E-92
Ccnd2	4.1063E-96	1.24708459	0.487	0.302	9.0088E-92
Hs3st1	2.7162E-95	1.43401174	0.16	0.051	5.959E-91
Prickle1	3.2965E-95	2.80167259	0.072	0.013	7.2322E-91
Mbnl1	1.1467E-93	0.5434456	0.871	0.763	2.5157E-89
Icos	1.3523E-93	0.59075647	0.598	0.349	2.9667E-89
Zap70	1.5231E-93	1.34998767	0.289	0.131	3.3416E-89

Gm42921	2.8934E-93	4.40068345	0.025	0.002	6.3479E-89
1700113H08R	3.1594E-93	4.56031266	0.019	0.001	6.9314E-89
Gm26870	1.7146E-91	2.76074733	0.087	0.019	3.7617E-87
Cep85l	2.3457E-91	2.13004545	0.162	0.055	5.1462E-87
Platr3	3.9351E-91	4.72918737	0.02	0.001	8.6333E-87
Runx3	9.6673E-91	1.05534829	0.612	0.467	2.1209E-86
Gls	1.0272E-90	0.98768081	0.616	0.476	2.2537E-86
Rpl23a	1.7779E-90	0.30655821	0.994	0.961	3.9005E-86
Itgb7	1.995E-90	1.27993184	0.412	0.244	4.3768E-86
Trbc1	2.4201E-89	0.68915765	0.473	0.256	5.3095E-85
Itpkb	2.5796E-89	0.9604077	0.55	0.383	5.6593E-85
B3galt2	3.5335E-89	2.56590258	0.079	0.017	7.7521E-85
Nmt2	4.3793E-88	1.72566591	0.242	0.107	9.6078E-84
Ubb	1.7759E-85	0.61694354	0.904	0.818	3.8961E-81
Rcor1	5.3919E-85	1.6383405	0.24	0.107	1.1829E-80
Tnfsf11	6.8106E-85	1.17822232	0.246	0.106	1.4942E-80
Lars2	1.0884E-83	0.59831125	0.817	0.73	2.3879E-79
Zfyve16	8.0449E-83	1.99925993	0.183	0.072	1.765E-78
Gm973	3.1349E-82	5.24941113	0.015	0.001	6.8776E-78
Ptp4a2	1.3157E-81	0.56532125	0.816	0.721	2.8865E-77
Ets1	1.5221E-81	0.46791836	0.903	0.659	3.3394E-77
Rapgef2	2.7518E-81	1.22714642	0.373	0.214	6.0372E-77
Gprasp2	4.792E-81	3.88104236	0.022	0.002	1.0513E-76
Coro2b	9.2107E-80	2.85375886	0.053	0.009	2.0207E-75
Adgrl1	1.2377E-79	1.98153199	0.124	0.038	2.7153E-75
Klrg1	1.9871E-79	1.46441368	0.112	0.032	4.3595E-75
Esyt2	5.9619E-79	1.13645622	0.412	0.247	1.308E-74
Rbfa	8.2682E-79	1.94573991	0.213	0.093	1.814E-74
Birc2	8.8419E-79	1.13240869	0.484	0.326	1.9398E-74
Nmur1	1.4247E-78	5.22502265	0.014	0.001	3.1257E-74
Mvb12b	7.6373E-78	2.06584588	0.151	0.054	1.6755E-73
Insr	1.7395E-77	2.06375495	0.121	0.037	3.8163E-73
Osbpl8	2.0057E-77	1.20629127	0.394	0.24	4.4003E-73
Cobll1	5.6224E-77	1.63619489	0.14	0.047	1.2335E-72
Faah	2.4143E-76	1.49796597	0.228	0.102	5.2968E-72
Frmd4b	7.4058E-76	1.67826403	0.255	0.127	1.6248E-71
Itm2c	1.3968E-75	1.07994197	0.437	0.287	3.0644E-71
Fbxl21	2.9836E-75	2.71993585	0.041	0.006	6.5457E-71
Zfp536	1.2653E-74	4.63031763	0.017	0.001	2.7759E-70
Rab4a	4.0753E-74	1.91374969	0.117	0.036	8.9408E-70
Fgl2	5.1594E-74	1.11808613	0.413	0.257	1.1319E-69
Polg2	6.5124E-74	1.73764486	0.174	0.069	1.4287E-69
Kif3a	3.185E-72	2.85801684	0.068	0.015	6.9875E-68
Itpr1	6.6198E-72	1.20948356	0.339	0.193	1.4523E-67
Vipr2	1.0283E-71	3.93537188	0.022	0.002	2.2559E-67
Nr4a3	3.967E-71	0.99587373	0.527	0.371	8.7032E-67
Dscaml1	4.6446E-71	5.07896113	0.015	0.001	1.019E-66
Slc25a3	6.8028E-71	0.6010033	0.847	0.774	1.4925E-66

Tob2	7.9228E-71	1.08791377	0.477	0.329	1.7382E-66
Asb2	9.3958E-71	1.90559144	0.133	0.046	2.0614E-66
Pnn	5.2845E-70	1.17649559	0.394	0.248	1.1594E-65
Tiam1	1.4978E-69	1.74708336	0.193	0.085	3.2861E-65
Pnrc1	7.6472E-69	0.47814587	0.831	0.736	1.6777E-64
Il7r	1.5951E-68	0.48076864	0.598	0.391	3.4995E-64
Tsc22d2	2.1408E-68	1.40574109	0.276	0.145	4.6966E-64
Rsbn1	8.9071E-68	1.36910972	0.283	0.15	1.9541E-63
Ppfibp2	2.5303E-67	2.50638858	0.142	0.054	5.5512E-63
Trp53inp1	3.7053E-67	1.08817483	0.35	0.2	8.1291E-63
Selenow	9.1421E-67	0.7093897	0.637	0.518	2.0057E-62
Cd52	1.2862E-66	0.5792632	0.891	0.816	2.8218E-62
Il6	2.425E-66	1.78966653	0.115	0.038	5.3202E-62
Ide	3.7471E-66	1.69988583	0.17	0.071	8.2207E-62
Sik3	3.8881E-66	1.24553716	0.323	0.188	8.5301E-62
Eef2	6.4284E-66	0.38419359	0.916	0.876	1.4103E-61
Arg1	6.8517E-66	2.3224617	0.056	0.011	1.5032E-61
mt-Nd3	1.2661E-65	0.77866315	0.588	0.463	2.7776E-61
Spty2d1	4.8713E-65	1.25860156	0.43	0.298	1.0687E-60
Cd3e	5.3813E-65	0.77545079	0.465	0.288	1.1806E-60
Cldnd1	6.4029E-65	1.31186403	0.368	0.233	1.4047E-60
Gm14275	1.6847E-64	4.72729948	0.013	0.001	3.696E-60
Rxrg	1.7047E-64	4.76827762	0.013	0.001	3.74E-60
Dcbld2	1.7251E-64	3.01804972	0.042	0.007	3.7847E-60
Myadm	4.0372E-64	1.66209728	0.245	0.128	8.8572E-60
Specc1	6.5645E-64	1.9693823	0.158	0.066	1.4402E-59
Adam12	1.7959E-63	2.8076621	0.044	0.008	3.9399E-59
Slc9a1	7.4759E-63	1.85448358	0.131	0.048	1.6401E-58
Clvs1	1.3433E-62	5.808476	0.01	0	2.9471E-58
Gng2	2.1317E-62	0.98474023	0.445	0.311	4.6766E-58
Fosl2	3.1499E-62	0.69101142	0.636	0.512	6.9106E-58
Cers4	8.0769E-62	1.23788853	0.213	0.099	1.772E-57
Plod2	2.9878E-61	2.03299506	0.078	0.021	6.555E-57
Lrrn2	4.4168E-61	3.41562625	0.024	0.002	9.69E-57
Raph1	4.8136E-61	1.56039269	0.255	0.137	1.056E-56
Serinc3	1.5091E-60	0.5977672	0.682	0.565	3.3109E-56
Cd4	2.3008E-60	0.98883337	0.287	0.153	5.0477E-56
Rinl	2.9153E-60	1.26001074	0.295	0.165	6.396E-56
Nt5e	2.0064E-59	1.19188857	0.204	0.094	4.4019E-55
Abhd17c	2.6999E-59	1.65209631	0.169	0.073	5.9233E-55
Reps1	6.2933E-59	1.56925745	0.195	0.092	1.3807E-54
Bmper	1.4806E-58	4.82367549	0.011	0	3.2483E-54
Fam69c	2.2078E-58	4.09248004	0.014	0.001	4.8436E-54
Podnl1	2.3797E-58	2.06570224	0.094	0.029	5.2209E-54
Fus	2.718E-58	0.51532213	0.799	0.749	5.963E-54
Pln	4.8709E-58	5.60426943	0.012	0.001	1.0686E-53
Atrx	4.8922E-58	0.85816463	0.556	0.442	1.0733E-53
Lat	5.4479E-58	0.76890766	0.379	0.225	1.1952E-53

Tcrg-C2	7.7484E-58	2.50376434	0.049	0.01	1.6999E-53
Sptssa	1.2337E-57	1.0345283	0.367	0.238	2.7066E-53
Fsd1l	3.3979E-57	4.01713306	0.021	0.002	7.4547E-53
Ythdc1	5.7073E-57	0.73569947	0.612	0.506	1.2521E-52
Elovl5	9.7091E-57	1.03011012	0.364	0.233	2.1301E-52
Cst7	1.1233E-56	1.73086788	0.141	0.057	2.4644E-52
Cdkn2a	2.4063E-56	2.94757821	0.037	0.006	5.2792E-52
Sod1	3.2948E-56	0.67811039	0.629	0.518	7.2284E-52
Gclc	3.5187E-56	1.22293309	0.226	0.115	7.7197E-52
Nedd4	8.334E-55	1.61903204	0.138	0.056	1.8284E-50
Tor1aip2	1.5799E-54	1.56715904	0.229	0.122	3.4662E-50
AU020206	2.1879E-54	0.91659309	0.402	0.262	4.8E-50
Neu3	9.3791E-54	2.15098101	0.064	0.017	2.0577E-49
Itpr2	1.1443E-53	0.96906062	0.334	0.203	2.5104E-49
Gm10605	1.4704E-53	3.33740061	0.02	0.002	3.2258E-49
Man2a2	3.1661E-53	1.26070347	0.218	0.111	6.9462E-49
Anp32a	5.9398E-53	0.7944814	0.515	0.398	1.3031E-48
S100a11	9.4525E-53	0.3379785	0.719	0.562	2.0738E-48
Sfi1	9.8527E-53	1.99019255	0.091	0.03	2.1616E-48
Arl14ep	1.0763E-52	2.05114173	0.109	0.04	2.3612E-48
Penk	1.3799E-52	2.13026971	0.053	0.012	3.0273E-48
Nedd9	1.6488E-52	1.24687362	0.253	0.138	3.6174E-48
Ttn	3.181E-52	1.88311975	0.072	0.02	6.9788E-48
Celf5	3.2797E-52	4.00412454	0.014	0.001	7.1952E-48
AC109617.1	5.1297E-52	3.61231902	0.02	0.002	1.1254E-47
Npy	5.5793E-52	5.07140833	0.016	0.001	1.224E-47
Fos	6.334E-52	1.12790786	0.447	0.314	1.3896E-47
Dlg4	1.1406E-51	2.18541615	0.08	0.025	2.5024E-47
Ncoa2	2.445E-51	1.02388323	0.308	0.184	5.3642E-47
Arhgap20	2.4671E-51	3.45926425	0.02	0.002	5.4126E-47
Itgb3	2.8238E-51	1.11286464	0.216	0.108	6.1951E-47
Zfhx2	3.2901E-51	2.08720696	0.086	0.028	7.2182E-47
Ell2	3.3795E-51	1.06278008	0.402	0.283	7.4144E-47
D16Ertd472e	3.7611E-51	0.68875526	0.517	0.373	8.2514E-47
Plagl1	5.7916E-51	2.13990927	0.062	0.016	1.2706E-46
Plk3	6.2153E-51	1.52949334	0.134	0.055	1.3636E-46
Pigl	9.9258E-51	2.72070916	0.04	0.008	2.1776E-46
Akap12	1.0304E-50	2.84270809	0.06	0.015	2.2605E-46
Ormdl3	1.7136E-50	1.38510939	0.169	0.078	3.7594E-46
St6galnac2	2.5805E-50	3.28480827	0.029	0.004	5.6614E-46
Mgat5	3.8422E-50	1.45023972	0.177	0.085	8.4295E-46
Col12a1	9.7797E-50	2.47017068	0.044	0.009	2.1456E-45
Zbtb1	3.239E-49	1.15255956	0.244	0.134	7.106E-45
Hipk1	2.1641E-48	0.87628638	0.415	0.294	4.7479E-44
Serpinb6a	2.5665E-48	1.28842249	0.183	0.09	5.6307E-44
Ppp1r15a	3.017E-48	0.76413332	0.519	0.406	6.619E-44
Dyrk3	4.1654E-48	2.43629544	0.046	0.01	9.1386E-44
Irak3	5.8578E-48	1.12854953	0.184	0.09	1.2851E-43

mt-Nd4l	5.9838E-48	0.89197853	0.463	0.354	1.3128E-43
Sh2b1	2.2303E-47	1.59778309	0.147	0.066	4.8931E-43
Aldoa	2.4868E-47	0.72413398	0.56	0.458	5.4557E-43
Rgs12	3.246E-47	3.03490695	0.044	0.01	7.1213E-43
Far1	4.3236E-47	1.20649753	0.288	0.178	9.4856E-43
Cd69	5.6517E-47	0.75816791	0.439	0.302	1.2399E-42
Klf6	5.9785E-47	0.71620536	0.653	0.562	1.3116E-42
Lamc1	1.9436E-46	1.10453092	0.192	0.096	4.2641E-42
Jmjd1c	3.4161E-46	0.69012085	0.537	0.425	7.4946E-42
Chd3	5.2667E-46	0.74902185	0.418	0.289	1.1555E-41
Senp1	1.9798E-45	1.59475917	0.157	0.075	4.3435E-41
Crbn	8.1985E-45	0.92452744	0.283	0.167	1.7987E-40
Abhd2	2.0825E-44	1.10551787	0.228	0.126	4.5687E-40
Picalm	2.3038E-44	0.66451639	0.616	0.523	5.0544E-40
Bmp2	2.3079E-44	1.74808153	0.08	0.027	5.0633E-40
Aes	3.8088E-44	0.70938433	0.511	0.403	8.3562E-40
Nfil3	5.6394E-44	0.87748585	0.406	0.291	1.2372E-39
Itgal	1.9317E-43	0.73904356	0.436	0.316	4.238E-39
Ywhaz	2.3325E-43	0.44524321	0.763	0.71	5.1173E-39
Wls	5.2474E-43	1.4420544	0.17	0.085	1.1512E-38
Ankrd12	5.7666E-43	0.85742268	0.391	0.272	1.2651E-38
Fmr1	7.9734E-43	1.11862433	0.278	0.171	1.7493E-38
Osbpl3	9.6196E-43	1.4512742	0.164	0.08	2.1104E-38
Galnt12	1.0982E-42	2.12399496	0.077	0.026	2.4093E-38
Clk1	1.4435E-42	0.45861044	0.746	0.681	3.1668E-38
Pitpnc1	1.9484E-42	0.94318316	0.274	0.165	4.2747E-38
Rexo2	2.8275E-42	1.16990902	0.303	0.202	6.2032E-38
Sprn	3.327E-42	4.59740252	0.01	0.001	7.2991E-38
Ccser2	4.0186E-42	1.42367439	0.211	0.117	8.8165E-38
Crebl2	4.8674E-42	1.68684245	0.09	0.033	1.0679E-37
Chic1	5.8981E-42	2.41965328	0.037	0.008	1.294E-37
Sub1	7.3463E-42	0.66287552	0.784	0.816	1.6117E-37
AI504432	7.7415E-42	1.45561651	0.203	0.112	1.6984E-37
Apaf1	1.8547E-41	1.14981659	0.269	0.168	4.069E-37
Hif1a	2.4753E-41	0.51724314	0.662	0.57	5.4306E-37
Rbm39	2.5106E-41	0.34340755	0.88	0.861	5.508E-37
Runx1	2.7447E-41	1.04205261	0.448	0.348	6.0216E-37
mt-Nd5	5.1717E-41	0.53758455	0.639	0.559	1.1346E-36
Fkbp5	6.9725E-41	1.78252323	0.084	0.03	1.5297E-36
Vps37b	2.4878E-40	0.40912771	0.764	0.662	5.4579E-36
Pvt1	6.1283E-40	1.89505592	0.083	0.03	1.3445E-35
B630019A10R	8.6623E-40	2.72843333	0.024	0.004	1.9004E-35
Tnk2	9.1573E-40	1.46743192	0.099	0.039	2.009E-35
Ssbp3	1.1855E-39	1.18665509	0.197	0.108	2.601E-35
Cdk17	1.2576E-39	0.96383593	0.24	0.139	2.7591E-35
Sept9	1.2618E-39	1.15857123	0.261	0.163	2.7682E-35
Trpm1	1.5785E-39	3.79222593	0.014	0.001	3.463E-35
Dxo	4.6381E-39	1.78174782	0.094	0.037	1.0176E-34

Gm38405	6.8103E-39	3.05050768	0.021	0.003	1.4941E-34
Lncpint	8.8269E-39	1.12048597	0.262	0.164	1.9365E-34
Prrc2c	9.6494E-39	0.42715311	0.758	0.704	2.117E-34
Lpar6	1.052E-38	1.51776789	0.161	0.083	2.3079E-34
Selenok	1.2089E-38	0.51741193	0.701	0.635	2.6522E-34
Slc7a8	1.3683E-38	1.80926458	0.128	0.06	3.0018E-34
Hcst	1.6867E-38	0.28383451	0.633	0.465	3.7005E-34
Gm2a	1.8481E-38	0.30225686	0.515	0.367	4.0546E-34
Cenpf	2.0882E-38	2.04767024	0.051	0.014	4.5812E-34
Pkdcc	2.3637E-38	2.67805535	0.042	0.011	5.1856E-34
Fyb	2.4275E-38	0.41474876	0.638	0.507	5.3257E-34
mt-Atp8	2.5129E-38	1.01838561	0.293	0.193	5.5131E-34
Gm48236	3.4376E-38	2.13075852	0.044	0.011	7.5418E-34
Ccl1	8.93E-38	1.31135576	0.044	0.011	1.9591E-33
Sik1	9.02E-38	0.79062841	0.392	0.283	1.9789E-33
Fgf16	1.9602E-37	3.53985112	0.012	0.001	4.3005E-33
Ptpn7	2.1488E-37	1.52087905	0.127	0.059	4.7143E-33
2700097O09R	4.5972E-37	1.66004873	0.077	0.028	1.0086E-32
Mtmr10	6.9709E-37	1.77619181	0.102	0.043	1.5293E-32
Ankrd6	8.584E-37	2.80698634	0.023	0.004	1.8832E-32
Mast3	1.4149E-36	1.49381083	0.12	0.055	3.1042E-32
Klk8	1.6796E-36	1.10222141	0.18	0.096	3.6849E-32
Grik5	3.4534E-36	2.50789614	0.031	0.006	7.5764E-32
S100a10	3.7605E-36	0.28390997	0.693	0.554	8.2502E-32
Pde7a	4.5156E-36	0.83294912	0.325	0.217	9.9067E-32
Junb	4.6043E-36	0.40280381	0.881	0.831	1.0101E-31
Arglu1	6.5791E-36	0.88231615	0.4	0.303	1.4434E-31
Gnb1	1.0538E-35	0.59418797	0.572	0.5	2.3119E-31
Cep290	1.3877E-35	2.21731872	0.063	0.021	3.0444E-31
Gm12214	2.2911E-35	3.83836158	0.01	0.001	5.0264E-31
Fam102a	2.9877E-35	1.06540459	0.199	0.112	6.5547E-31
Dach2	3.1423E-35	3.12174083	0.016	0.002	6.8938E-31
Tcrg-C4	6.9248E-35	1.84876923	0.054	0.017	1.5192E-30
Map4	7.4448E-35	1.29029416	0.205	0.121	1.6333E-30
Nr1d2	7.5264E-35	0.88781596	0.27	0.17	1.6512E-30
Acot11	1.0297E-34	2.36994879	0.037	0.009	2.2591E-30
L1cam	2.4339E-34	1.89499176	0.068	0.024	5.3397E-30
Map1b	2.4777E-34	2.51166305	0.029	0.006	5.4359E-30
S100a13	2.6277E-34	0.68361996	0.48	0.392	5.765E-30
Crip1	3.3593E-34	0.31824712	0.777	0.647	7.37E-30
Hnrnph1	3.9332E-34	0.52276102	0.573	0.489	8.6291E-30
Hmgn3	6.4986E-34	1.94681188	0.075	0.028	1.4257E-29
Zfp36l1	6.5386E-34	0.53670597	0.712	0.644	1.4345E-29
4932438H23R	8.3429E-34	1.92142766	0.049	0.014	1.8303E-29
Gm16286	1.224E-33	0.86767143	0.348	0.257	2.6853E-29
Zfp773	3.0667E-33	2.09718799	0.04	0.01	6.7279E-29
Aplp1	3.5693E-33	2.42181405	0.037	0.009	7.8307E-29
C77080	4.6195E-33	3.62946309	0.014	0.002	1.0135E-28

Chrne	5.2157E-33	1.65573244	0.071	0.026	1.1443E-28
Hspa4l	5.4275E-33	1.33243218	0.135	0.068	1.1907E-28
Tomm7	6.8166E-33	0.45364678	0.707	0.644	1.4955E-28
E130308A19R	8.5519E-33	1.5676661	0.068	0.025	1.8762E-28
Creb3l2	2.1787E-32	1.45140582	0.095	0.04	4.7798E-28
Adgrg6	2.8151E-32	2.74862269	0.044	0.013	6.1761E-28
Lockd	3.5874E-32	1.56015259	0.092	0.039	7.8703E-28
Npnt	1.4072E-31	1.24798342	0.069	0.025	3.0873E-27
Ndfip1	2.0347E-31	0.68279499	0.383	0.281	4.4639E-27
Gm5547	4.0358E-31	1.47966449	0.158	0.088	8.8541E-27
Slk	4.6615E-31	0.78746778	0.416	0.331	1.0227E-26
Tmod2	8.998E-31	3.69519806	0.013	0.002	1.9741E-26
Dtx3	9.7067E-31	1.47545587	0.129	0.065	2.1295E-26
Sept8	1.303E-30	2.02288335	0.063	0.023	2.8587E-26
Acsl4	1.3962E-30	1.02586403	0.257	0.171	3.0631E-26
Sesn3	1.5163E-30	0.71648476	0.318	0.217	3.3266E-26
C2cd5	1.782E-30	1.45748008	0.101	0.046	3.9094E-26
Kmt2c	2.0957E-30	0.92411975	0.297	0.207	4.5978E-26
Sfpq	2.4308E-30	0.50314119	0.606	0.54	5.3329E-26
Prkd3	2.614E-30	1.03694581	0.195	0.115	5.7349E-26
Cyb5a	3.9899E-30	1.01868639	0.289	0.204	8.7534E-26
Gnas	4.6808E-30	0.3712051	0.759	0.705	1.0269E-25
Tub	5.5787E-30	2.91279037	0.013	0.002	1.2239E-25
Tgfbr3	8.5211E-30	1.70675486	0.05	0.016	1.8694E-25
St6galnac3	8.7363E-30	1.32753712	0.12	0.059	1.9167E-25
Utrn	1.1159E-29	0.6412303	0.341	0.24	2.4481E-25
Oaz1	1.359E-29	0.25023179	0.885	0.831	2.9815E-25
Cdc42ep3	1.5081E-29	0.86780275	0.138	0.071	3.3085E-25
Gm12840	2.0333E-29	0.69591412	0.325	0.225	4.4608E-25
Slain1	2.9597E-29	1.2634908	0.132	0.068	6.4934E-25
Cox6c	3.3057E-29	0.37760106	0.768	0.704	7.2524E-25
Sntb1	6.1614E-29	1.31965221	0.089	0.039	1.3518E-24
Klf12	8.1093E-29	2.93564379	0.015	0.002	1.7791E-24
Ggt7	1.5239E-28	3.39475755	0.013	0.002	3.3433E-24
Etnk1	2.6435E-28	0.68782617	0.35	0.255	5.7995E-24
Atp5h	3.1568E-28	0.36010634	0.801	0.739	6.9258E-24
Snapc1	4.4928E-28	1.16295556	0.203	0.127	9.8567E-24
Arhgap31	6.2631E-28	0.78977506	0.314	0.225	1.3741E-23
Fam122a	6.989E-28	1.57733516	0.114	0.057	1.5333E-23
Rem2	9.9363E-28	3.09157933	0.015	0.002	2.1799E-23
Snrnp70	1.532E-27	0.51932358	0.583	0.52	3.361E-23
Elmo1	1.7041E-27	0.76627124	0.376	0.295	3.7387E-23
Lnpep	1.8134E-27	0.57525525	0.471	0.387	3.9785E-23
Saraf	2.1676E-27	0.55866763	0.465	0.378	4.7554E-23
Scrt1	2.8311E-27	1.57407194	0.061	0.023	6.2112E-23
Ccr8	3.1683E-27	2.16493984	0.028	0.006	6.951E-23
Prr13	4.2767E-27	0.53123392	0.503	0.423	9.3825E-23
Rgs1	4.8991E-27	0.35567228	0.686	0.562	1.0748E-22

Rps6ka3	5.0676E-27	1.06922575	0.209	0.133	1.1118E-22
Baz1a	6.1657E-27	0.92352494	0.406	0.334	1.3527E-22
Il27ra	7.5764E-27	1.27042633	0.11	0.054	1.6622E-22
Fam110a	7.8819E-27	0.92831016	0.218	0.139	1.7292E-22
Gm43254	1.1329E-26	2.5819271	0.017	0.003	2.4854E-22
Stxbp4	1.4612E-26	2.94289298	0.016	0.003	3.2058E-22
Olfr1259	1.6856E-26	2.6475352	0.018	0.003	3.6979E-22
Mfge8	1.9911E-26	1.45268914	0.072	0.03	4.3682E-22
Smarca2	2.0064E-26	0.92324788	0.22	0.141	4.4019E-22
Tnfaip8	2.5148E-26	0.7437736	0.455	0.386	5.5171E-22
Ppme1	3.0357E-26	1.07334529	0.172	0.102	6.6601E-22
Tppp	3.2882E-26	2.55377514	0.024	0.005	7.214E-22
Vps13a	5.7289E-26	0.86383759	0.25	0.169	1.2569E-21
C2	6.9793E-26	2.83080283	0.02	0.004	1.5312E-21
Tspan13	7.1333E-26	0.49295834	0.506	0.424	1.565E-21
Clstn1	1.132E-25	2.08328862	0.05	0.018	2.4836E-21
Mllt3	1.7197E-25	0.88220004	0.184	0.111	3.7729E-21
Tnik	3.3784E-25	0.93562016	0.157	0.089	7.4118E-21
Tra2b	3.5839E-25	0.48090575	0.633	0.592	7.8628E-21
Ddb1	3.6485E-25	1.19270175	0.206	0.135	8.0045E-21
Eml2	4.0681E-25	1.79998695	0.065	0.027	8.925E-21
Arhgap32	4.9588E-25	2.73040896	0.027	0.006	1.0879E-20
Cpne7	6.67E-25	2.75382582	0.022	0.005	1.4633E-20
Zhx1	6.6914E-25	1.29765327	0.142	0.081	1.468E-20
Tnfsf10	6.841E-25	1.72373716	0.051	0.018	1.5008E-20
Rnf43	7.1043E-25	2.10868203	0.036	0.011	1.5586E-20
Srrm2	8.0502E-25	0.37401619	0.758	0.725	1.7661E-20
Cpsf6	1.3784E-24	0.75980645	0.309	0.226	3.0241E-20
Taf3	1.7311E-24	1.10468377	0.174	0.107	3.7978E-20
Rbl2	2.4565E-24	0.95636387	0.216	0.142	5.3893E-20
Slc38a2	2.5359E-24	0.53095127	0.631	0.583	5.5636E-20
Slc35g1	2.9431E-24	2.11619098	0.034	0.01	6.4569E-20
Nck2	3.2111E-24	1.34380589	0.081	0.037	7.0447E-20
Uhrf1bp1l	3.2439E-24	0.92479391	0.231	0.156	7.1167E-20
St3gal2	4.4384E-24	2.16995836	0.041	0.013	9.7373E-20
Enpp2	5.0662E-24	3.3766022	0.019	0.004	1.1115E-19
Tmtc2	7.7124E-24	2.32995708	0.048	0.017	1.692E-19
Rassf5	2.4171E-23	0.94377448	0.208	0.137	5.3029E-19
Hccs	2.5126E-23	0.96749301	0.214	0.141	5.5123E-19
Ctgf	2.9004E-23	3.28816482	0.024	0.006	6.3632E-19
Iglc1	3.063E-23	0.35425993	0.491	0.419	6.7199E-19
Bzw2	3.2885E-23	0.85286065	0.253	0.176	7.2147E-19
Impg1	3.3464E-23	3.18289893	0.011	0.001	7.3417E-19
Galnt1	3.6131E-23	0.84087207	0.273	0.197	7.9268E-19
Nup98	3.6427E-23	0.58663405	0.453	0.39	7.9918E-19
Zfp704	3.6589E-23	2.59144494	0.032	0.009	8.0272E-19
Casc4	3.8785E-23	1.55298588	0.047	0.017	8.509E-19
Gm26532	4.5221E-23	1.28175749	0.128	0.071	9.921E-19

Clgn	6.3547E-23	3.68097621	0.011	0.001	1.3942E-18
Slco2b1	8.6294E-23	2.10347964	0.044	0.016	1.8932E-18
Nfkbid	9.8692E-23	0.85207668	0.206	0.134	2.1652E-18
Il12rb1	1.8621E-22	1.45450747	0.051	0.019	4.0854E-18
Tbca	2.7183E-22	0.52725442	0.514	0.459	5.9638E-18
Per3	3.0565E-22	2.28184977	0.026	0.007	6.7057E-18
Gypc	3.3677E-22	1.21958044	0.101	0.052	7.3884E-18
Wee1	4.6835E-22	1.97020156	0.048	0.018	1.0275E-17
Nktr	6.1159E-22	0.48135818	0.614	0.581	1.3418E-17
Nectin3	1.2708E-21	3.02047748	0.014	0.002	2.7881E-17
Smpdl3a	1.2768E-21	0.87545464	0.25	0.181	2.8011E-17
Calm3	1.2933E-21	0.72879231	0.39	0.329	2.8373E-17
Pde4a	1.9157E-21	1.74226207	0.042	0.015	4.2029E-17
Crmp1	2.0167E-21	1.7562628	0.057	0.024	4.4245E-17
Gkap1	2.2914E-21	2.0473904	0.044	0.016	5.0271E-17
Tra2a	2.6385E-21	0.49212483	0.574	0.534	5.7886E-17
Rai1	3.2949E-21	1.23255723	0.104	0.055	7.2287E-17
Camk2n1	3.9333E-21	0.90780347	0.154	0.093	8.6292E-17
Ypel2	4.5312E-21	1.5307096	0.044	0.016	9.9409E-17
Stat4	6.9875E-21	0.55695105	0.459	0.382	1.533E-16
Hes1	7.6278E-21	1.2976165	0.158	0.099	1.6735E-16
Birc6	1.1894E-20	0.63405222	0.42	0.36	2.6094E-16
Mapk8ip1	1.216E-20	3.16479874	0.012	0.002	2.6678E-16
Il2rg	1.3967E-20	0.46867336	0.534	0.474	3.0643E-16
Zmat3	1.476E-20	1.90757331	0.041	0.015	3.2381E-16
C030037D09F	2.265E-20	3.19916354	0.011	0.002	4.9692E-16
Epm2aip1	2.3992E-20	1.3225198	0.106	0.058	5.2636E-16
B3gnt2	2.6442E-20	1.50213489	0.049	0.019	5.8011E-16
Ube2d3	2.6692E-20	0.36237704	0.784	0.774	5.856E-16
Xkr6	3.1175E-20	2.16642931	0.029	0.008	6.8396E-16
Gng3	3.5532E-20	3.20591918	0.011	0.002	7.7953E-16
Ptp4a1	3.6735E-20	0.56002784	0.51	0.464	8.0592E-16
Snhg12	7.377E-20	0.63582554	0.359	0.286	1.6184E-15
Gm20186	7.5497E-20	0.85699873	0.159	0.099	1.6563E-15
Anks6	7.9048E-20	3.23215027	0.01	0.001	1.7342E-15
Cast	8.9397E-20	0.67006095	0.351	0.283	1.9613E-15
Crem	9.4204E-20	0.42877869	0.633	0.594	2.0667E-15
Atxn7	9.5796E-20	0.8674822	0.196	0.131	2.1017E-15
Mdm4	9.7326E-20	0.73223658	0.256	0.185	2.1352E-15
Cox7a2	1.0932E-19	0.43803936	0.612	0.582	2.3983E-15
Prkacb	1.2724E-19	0.95553957	0.215	0.151	2.7916E-15
Calcrl	1.7523E-19	1.21861571	0.063	0.028	3.8445E-15
Pecam1	2.0498E-19	0.82528559	0.206	0.139	4.4971E-15
Chst2	2.3212E-19	1.38673477	0.058	0.025	5.0924E-15
Gm15655	2.4168E-19	1.30472311	0.055	0.023	5.3023E-15
Abhd14b	2.5763E-19	2.07460178	0.033	0.011	5.6522E-15
1700020I14Ril	5.104E-19	0.7510942	0.269	0.201	1.1198E-14
Zbtb38	5.7162E-19	1.07168457	0.135	0.082	1.2541E-14

Slc35e2	5.7176E-19	1.07568351	0.09	0.046	1.2544E-14
Myl12a	5.7965E-19	0.35955745	0.611	0.563	1.2717E-14
Acbd7	6.6015E-19	2.42478807	0.014	0.003	1.4483E-14
Zfp831	6.6278E-19	1.2582709	0.042	0.016	1.4541E-14
AW011738	6.6638E-19	1.13763225	0.126	0.075	1.462E-14
Sv2c	9.5311E-19	1.47826603	0.047	0.019	2.091E-14
Leng8	1.1598E-18	0.77472706	0.241	0.175	2.5444E-14
Cbarp	1.2215E-18	1.27324624	0.098	0.053	2.6799E-14
AL590144.2	1.7287E-18	1.38360462	0.096	0.052	3.7925E-14
Jcad	1.741E-18	1.72873582	0.025	0.007	3.8197E-14
Ccng1	1.8621E-18	1.06575874	0.176	0.118	4.0853E-14
Cnbp	2.1085E-18	0.28404677	0.707	0.675	4.6259E-14
Stxbp5	2.5181E-18	1.19890016	0.093	0.05	5.5244E-14
Raver2	2.6131E-18	2.80868017	0.011	0.002	5.7329E-14
Flt3l	2.6625E-18	1.24145301	0.082	0.042	5.8413E-14
Cd6	2.7348E-18	0.61460006	0.22	0.149	5.9999E-14
Lif	2.8572E-18	1.58544371	0.048	0.02	6.2685E-14
Zbtb4	3.5258E-18	1.29387615	0.084	0.043	7.7353E-14
Cchcr1	3.781E-18	2.10148497	0.03	0.01	8.2952E-14
Bclaf1	4.5945E-18	0.48864219	0.538	0.497	1.008E-13
Ddx17	6.6581E-18	0.56483323	0.4	0.341	1.4607E-13
Gm13012	8.5892E-18	2.03621175	0.032	0.011	1.8844E-13
Arhgef6	1.0163E-17	0.93423364	0.206	0.146	2.2296E-13
Erc1	1.1634E-17	1.34089175	0.055	0.024	2.5524E-13
Ermp1	1.2265E-17	1.49831765	0.08	0.041	2.6908E-13
Setd5	1.486E-17	0.47542653	0.475	0.421	3.2601E-13
Mbnl3	1.8173E-17	1.50197586	0.082	0.043	3.9869E-13
Dtnb	1.8198E-17	1.19739878	0.073	0.036	3.9924E-13
Cntln	2.4007E-17	1.92794817	0.051	0.022	5.267E-13
Coq10b	2.5013E-17	0.80451467	0.321	0.263	5.4875E-13
Pan3	3.7258E-17	0.61298957	0.32	0.257	8.1741E-13
Gm26759	3.9973E-17	1.50356031	0.053	0.024	8.7697E-13
Kmt2d	4.0671E-17	0.88193558	0.177	0.119	8.9229E-13
Rnf166	5.0052E-17	0.89905397	0.172	0.116	1.0981E-12
Rsrc2	5.2733E-17	0.41593083	0.589	0.567	1.1569E-12
Zfp280b	5.7298E-17	1.53867689	0.046	0.019	1.2571E-12
Kdsr	6.0024E-17	1.17448239	0.094	0.052	1.3169E-12
Stk19	6.0826E-17	1.17810358	0.162	0.11	1.3345E-12
Zbtb20	6.3681E-17	0.75286185	0.264	0.197	1.3971E-12
2900060B14R	7.0914E-17	1.35301854	0.089	0.049	1.5558E-12
Ttc14	7.2903E-17	0.55252744	0.41	0.353	1.5994E-12
Pdlim1	8.7733E-17	0.91871896	0.17	0.114	1.9248E-12
Adrb2	9.0314E-17	1.287232	0.085	0.046	1.9814E-12
Vcl	1.007E-16	1.44358722	0.091	0.051	2.2093E-12
Raly	1.0255E-16	0.58674295	0.393	0.342	2.2499E-12
Gm26614	1.08E-16	3.09736538	0.01	0.002	2.3694E-12
Gm28187	1.1612E-16	2.27858876	0.019	0.005	2.5476E-12
Ralbp1	2.1205E-16	0.76465701	0.29	0.23	4.6521E-12

Suco	2.2478E-16	0.70717401	0.322	0.261	4.9315E-12
Ppp1cc	2.298E-16	0.31276876	0.475	0.394	5.0415E-12
Skil	3.5034E-16	0.39645834	0.539	0.491	7.6861E-12
Ermn	3.7086E-16	0.63920442	0.119	0.07	8.1363E-12
Ryk	4.0004E-16	1.51934232	0.046	0.02	8.7765E-12
Cdip1	4.5E-16	0.79559602	0.213	0.153	9.8726E-12
Fam105a	4.6481E-16	1.2456356	0.156	0.106	1.0197E-11
Cdc37l1	5.4707E-16	0.69486097	0.246	0.184	1.2002E-11
Tcrg-C1	5.6522E-16	1.38984219	0.051	0.023	1.24E-11
AY036118	5.9021E-16	0.47541134	0.537	0.488	1.2949E-11
Hip1	7.0854E-16	1.35728026	0.101	0.059	1.5545E-11
Pard3b	7.4025E-16	1.97379598	0.017	0.004	1.624E-11
Paqr8	7.975E-16	2.14133236	0.018	0.005	1.7496E-11
Crebzf	1.0821E-15	0.87114986	0.166	0.111	2.3741E-11
Il2	1.3197E-15	1.75339114	0.05	0.022	2.8953E-11
Plcl1	1.3732E-15	1.91232704	0.046	0.02	3.0127E-11
Ikbip	1.4522E-15	1.88103586	0.035	0.013	3.1861E-11
Purb	1.5283E-15	0.39610727	0.55	0.511	3.353E-11
Rmdn2	1.6325E-15	1.97059982	0.025	0.008	3.5814E-11
Casp4	1.7527E-15	0.82897834	0.287	0.231	3.8453E-11
Lonrf3	1.972E-15	1.94321175	0.026	0.008	4.3263E-11
Prr7	2.2737E-15	0.67569486	0.224	0.162	4.9883E-11
Srsf5	2.3869E-15	0.28733102	0.689	0.67	5.2366E-11
Plxdc2	2.9384E-15	1.4038921	0.073	0.038	6.4466E-11
Arntl	3.0965E-15	0.82465457	0.129	0.081	6.7935E-11
Kcnq5	3.1645E-15	2.26168013	0.016	0.004	6.9425E-11
Mbd2	3.56E-15	0.44201765	0.43	0.373	7.8104E-11
Mfsd14a	3.7329E-15	0.88879724	0.17	0.117	8.1897E-11
Zfp445	4.3091E-15	0.78014634	0.201	0.144	9.4538E-11
Zdhhc2	4.4646E-15	1.1100237	0.077	0.041	9.7948E-11
Cpd	4.7174E-15	0.90987642	0.15	0.1	1.035E-10
Sms	4.9118E-15	0.73652975	0.273	0.215	1.0776E-10
Meis3	4.9192E-15	1.6804128	0.033	0.013	1.0792E-10
Ptpn9	5.8256E-15	1.30645142	0.104	0.062	1.2781E-10
Aprt	7.7373E-15	0.54709103	0.379	0.331	1.6975E-10
Clip3	9.4506E-15	2.70381676	0.01	0.002	2.0734E-10
Zcchc10	1.1974E-14	1.05735607	0.113	0.07	2.6271E-10
Rsbn1l	1.2854E-14	0.42660133	0.422	0.36	2.82E-10
Plec	1.2924E-14	1.00739651	0.162	0.113	2.8354E-10
Rccd1	1.3173E-14	1.49023517	0.035	0.014	2.8899E-10
Tex9	1.3354E-14	1.65501374	0.035	0.014	2.9298E-10
Map6	1.3489E-14	2.35562986	0.013	0.003	2.9594E-10
Rbmx	1.5211E-14	0.94479477	0.107	0.065	3.3372E-10
Dennd1c	1.5489E-14	1.05443538	0.101	0.06	3.3981E-10
Eif4a2	1.7578E-14	0.38648395	0.491	0.443	3.8565E-10
Ikzf1	2.2012E-14	0.60725781	0.278	0.218	4.8293E-10
Rab37	2.3445E-14	1.1688472	0.068	0.036	5.1436E-10
Ogt	2.6731E-14	0.51198861	0.377	0.315	5.8644E-10

Mien1	3.0124E-14	0.62387851	0.312	0.257	6.6089E-10
Irs2	3.322E-14	0.48242377	0.32	0.253	7.2882E-10
Pafah1b1	4.3031E-14	0.31850216	0.608	0.587	9.4407E-10
Cysltr1	4.3279E-14	1.44692612	0.086	0.05	9.495E-10
Emsy	4.59E-14	0.64733761	0.254	0.195	1.007E-09
Cbx6	5.146E-14	1.49383593	0.04	0.017	1.129E-09
Irf4	6.0974E-14	0.79670098	0.21	0.154	1.3377E-09
Tbl1xr1	6.743E-14	0.81075899	0.205	0.153	1.4793E-09
1300002E11R	7.1745E-14	1.16049666	0.071	0.038	1.574E-09
Cops9	7.8594E-14	0.5066805	0.443	0.407	1.7243E-09
Isy1	8.5556E-14	0.54545228	0.459	0.423	1.877E-09
Tnpo2	1.0611E-13	1.14586061	0.088	0.051	2.328E-09
Ivns1abp	1.1443E-13	0.61996592	0.317	0.263	2.5106E-09
Cntn1	1.266E-13	1.70185921	0.024	0.008	2.7775E-09
Spn	1.2884E-13	0.48470719	0.275	0.211	2.8267E-09
Tnfrsf25	1.439E-13	0.92622446	0.079	0.044	3.157E-09
Rasal3	2.105E-13	0.83663882	0.109	0.068	4.6181E-09
Abi2	2.2838E-13	1.55782992	0.054	0.027	5.0104E-09
Il18rap	2.6758E-13	0.5377889	0.228	0.169	5.8705E-09
Impact	2.9606E-13	0.81848791	0.198	0.147	6.4954E-09
Gm27166	3.1218E-13	2.49906461	0.018	0.005	6.8489E-09
Fam213a	3.2441E-13	1.13378949	0.066	0.035	7.1172E-09
Fam185a	4.2547E-13	1.61105818	0.027	0.01	9.3344E-09
Adcy7	4.396E-13	1.18098002	0.123	0.081	9.6444E-09
Ehmt2	4.4039E-13	0.95512325	0.142	0.097	9.6618E-09
Eprs	4.6611E-13	0.61983956	0.441	0.406	1.0226E-08
Kif5c	4.7964E-13	1.93165182	0.023	0.008	1.0523E-08
Abhd8	5.1802E-13	1.2754684	0.06	0.031	1.1365E-08
Mir155hg	5.3506E-13	1.14546024	0.132	0.089	1.1739E-08
Xlr4a	6.04E-13	1.21185077	0.057	0.029	1.3251E-08
Pbx4	6.182E-13	1.94625066	0.019	0.006	1.3563E-08
Gm20342	6.8248E-13	1.26581119	0.046	0.022	1.4973E-08
Exoc3	7.0521E-13	0.91161385	0.203	0.154	1.5472E-08
Ppp3ca	7.0527E-13	0.40467655	0.371	0.318	1.5473E-08
Camk2g	7.086E-13	1.14065203	0.075	0.042	1.5546E-08
Herpud2	7.4656E-13	0.87026356	0.155	0.109	1.6379E-08
Rbm43	8.2564E-13	1.26693812	0.066	0.036	1.8114E-08
Lgals4	9.7032E-13	1.77377034	0.012	0.003	2.1288E-08
H2afj	1.0696E-12	0.40076679	0.533	0.505	2.3465E-08
Ccdc50	1.0802E-12	0.75339514	0.297	0.252	2.3698E-08
Mxi1	1.1023E-12	0.58736933	0.348	0.3	2.4183E-08
Luc7l2	1.1515E-12	0.27613389	0.675	0.662	2.5263E-08
Trdv4	1.4623E-12	0.66796278	0.057	0.029	3.2082E-08
Tmem14a	1.6284E-12	2.57200943	0.013	0.003	3.5726E-08
Dync1h1	2.0333E-12	0.64856957	0.27	0.22	4.4608E-08
Pard3	2.0366E-12	1.06688925	0.03	0.012	4.468E-08
Bhlhb9	2.5715E-12	1.53188447	0.032	0.013	5.6415E-08
Nckap1	2.9086E-12	1.54478232	0.027	0.01	6.3812E-08

Rab26os	3.137E-12	1.16281179	0.059	0.031	6.8822E-08
St6gal1	3.1813E-12	1.0328728	0.102	0.064	6.9796E-08
Klf4	4.5386E-12	0.43595754	0.304	0.243	9.9571E-08
Gt(ROSA)26Sor	4.7751E-12	0.52943205	0.272	0.22	1.0476E-07
Ephb6	5.5785E-12	1.97413335	0.011	0.002	1.2239E-07
Zfp40	6.0145E-12	2.13277106	0.018	0.006	1.3195E-07
Cela1	6.3111E-12	1.82393125	0.029	0.011	1.3846E-07
Gm44851	6.8475E-12	1.84985486	0.019	0.006	1.5023E-07
Wbp1	7.6135E-12	1.2662077	0.083	0.05	1.6703E-07
Serinc5	8.2822E-12	1.32317381	0.049	0.024	1.817E-07
B230307C23R	8.4571E-12	1.10791125	0.082	0.049	1.8554E-07
Wwtr1	1.0252E-11	1.98684296	0.02	0.007	2.2492E-07
Vgll4	1.0403E-11	0.33563976	0.382	0.318	2.2824E-07
Nbea	1.1835E-11	2.18971143	0.01	0.002	2.5965E-07
Gdi1	1.3005E-11	0.83266349	0.155	0.111	2.8532E-07
Mrpl33	1.3414E-11	0.37402736	0.47	0.438	2.9429E-07
Pdia3	1.3462E-11	0.31758969	0.582	0.545	2.9534E-07
Pawr	1.3933E-11	2.48092028	0.011	0.003	3.0568E-07
Pwwp2a	1.5348E-11	0.89029048	0.121	0.081	3.3671E-07
Rpl13a	1.548E-11	0.30169245	0.598	0.581	3.3961E-07
Carnmt1	1.6415E-11	0.64818216	0.19	0.14	3.6012E-07
Atp2a3	1.665E-11	0.60982206	0.142	0.097	3.6528E-07
Polg	1.8756E-11	0.88777006	0.084	0.05	4.115E-07
Herc4	1.9012E-11	0.67615204	0.19	0.142	4.171E-07
Pigp	1.9811E-11	1.07502762	0.107	0.07	4.3464E-07
Dnajb6	2.304E-11	0.33027901	0.591	0.579	5.0548E-07
Acot7	2.6186E-11	0.92243558	0.163	0.12	5.7449E-07
1190002N15R	2.6521E-11	0.900942	0.096	0.06	5.8184E-07
Gm26799	2.7067E-11	1.46516494	0.027	0.011	5.9381E-07
Fam117a	2.9574E-11	1.24994668	0.046	0.023	6.4883E-07
4932438A13R	3.338E-11	0.34382639	0.436	0.385	7.3233E-07
Apbb1	3.8976E-11	2.21279457	0.01	0.002	8.5509E-07
Pde1c	3.9829E-11	2.16567942	0.013	0.004	8.7381E-07
Tiparp	4.3962E-11	0.33595716	0.366	0.31	9.6448E-07
Iffo2	4.4554E-11	1.53619008	0.053	0.028	9.7747E-07
Aven	4.5038E-11	1.21031727	0.075	0.045	9.8809E-07
Epb41l1	4.6343E-11	1.65765768	0.018	0.006	1.0167E-06
Gm42372	4.689E-11	1.91453007	0.023	0.009	1.0287E-06
Gm45051	4.9754E-11	1.28136485	0.034	0.015	1.0915E-06
Cnn2	5.0439E-11	0.6155371	0.316	0.273	1.1066E-06
Srpk1	5.2537E-11	0.66265709	0.234	0.186	1.1526E-06
Trim36	5.5191E-11	1.32432489	0.056	0.03	1.2108E-06
Grb7	5.7688E-11	1.29775096	0.053	0.028	1.2656E-06
Ankrd16	5.9264E-11	1.31359153	0.053	0.028	1.3002E-06
5430416N02R	6.699E-11	0.83961489	0.126	0.086	1.4697E-06
Gm42047	6.8474E-11	1.85435626	0.022	0.008	1.5022E-06
Ifnar1	7.737E-11	0.53783698	0.386	0.353	1.6974E-06
1700097N02R	7.8016E-11	1.29889533	0.032	0.014	1.7116E-06

Nudt3	8.0669E-11	0.85836908	0.155	0.113	1.7698E-06
Ccdc171	8.0986E-11	1.54405779	0.03	0.013	1.7768E-06
Golm1	8.5525E-11	1.34421027	0.06	0.034	1.8763E-06
A630072M18F	8.7518E-11	0.87600801	0.092	0.058	1.92E-06
Mia2	1.026E-10	0.57772589	0.313	0.272	2.2509E-06
9930021J03Ril	1.0271E-10	0.89507448	0.125	0.086	2.2534E-06
Igsf5	1.0272E-10	1.32366337	0.027	0.011	2.2535E-06
Prkcz	1.2822E-10	1.13331338	0.049	0.025	2.8129E-06
Rnf125	1.2911E-10	0.36739285	0.264	0.205	2.8325E-06
Kcnc1	1.3347E-10	1.6215104	0.026	0.01	2.9283E-06
Prpf4b	1.3536E-10	0.45079397	0.435	0.401	2.9697E-06
Tasp1	1.4318E-10	1.19040528	0.052	0.028	3.1412E-06
Ucp2	1.827E-10	0.28731137	0.57	0.534	4.0084E-06
Phip	1.9596E-10	0.56091637	0.318	0.272	4.2992E-06
Plscr4	1.9658E-10	2.056013	0.014	0.004	4.3127E-06
Uqcrq	2.1125E-10	0.40035583	0.581	0.574	4.6347E-06
Rnf157	2.1936E-10	0.5169781	0.254	0.202	4.8126E-06
Asxl2	2.2979E-10	0.67433646	0.217	0.172	5.0414E-06
Dnaja4	2.6182E-10	1.02843819	0.067	0.039	5.744E-06
Ccdc69	2.6382E-10	1.45743758	0.033	0.015	5.7879E-06
Lppos	2.6619E-10	1.50949035	0.042	0.021	5.8399E-06
Phf1	2.9797E-10	0.7363382	0.105	0.069	6.5371E-06
Anxa11	3.0961E-10	0.82153028	0.185	0.144	6.7926E-06
2700081O15R	3.4243E-10	2.10031037	0.015	0.005	7.5126E-06
2510009E07R	3.8146E-10	1.45849885	0.063	0.037	8.3689E-06
Csnk1g3	3.8599E-10	0.48723987	0.249	0.201	8.4681E-06
Ppp1r18	4.5712E-10	0.67297302	0.237	0.195	1.0029E-05
Ttc39b	5.2005E-10	0.58840428	0.246	0.201	1.1409E-05
Eef1d	5.3592E-10	0.29308765	0.584	0.578	1.1758E-05
Gpx7	5.8003E-10	2.0082773	0.014	0.004	1.2725E-05
Tnp2	6.9614E-10	2.45855653	0.012	0.003	1.5273E-05
Cd5	7.7805E-10	0.60225499	0.182	0.137	1.707E-05
Ercc5	8.2893E-10	1.11539846	0.07	0.042	1.8186E-05
Rcbtb2	8.3713E-10	1.17071246	0.133	0.096	1.8366E-05
Usp38	8.9429E-10	0.59632894	0.18	0.136	1.962E-05
Tubb5	9.1627E-10	0.29124869	0.423	0.385	2.0102E-05
Abhd14a	9.8138E-10	1.81436816	0.029	0.013	2.153E-05
Rnf152	1.0948E-09	1.87378398	0.012	0.004	2.4018E-05
Bcorl1	1.2027E-09	1.44024433	0.027	0.011	2.6386E-05
Nxpe3	1.4073E-09	0.92610291	0.049	0.026	3.0874E-05
Mum1	1.4266E-09	1.08627897	0.068	0.041	3.1297E-05
Ntng2	1.4807E-09	1.82943796	0.014	0.004	3.2486E-05
Psat1	1.7619E-09	1.09199767	0.064	0.038	3.8655E-05
B930036N10R	1.8009E-09	0.69916341	0.125	0.088	3.9509E-05
Hsf1	1.8092E-09	0.97511224	0.096	0.064	3.9692E-05
Top2a	1.872E-09	0.43717178	0.128	0.089	4.1071E-05
Ccdc82	2.1488E-09	0.76273534	0.13	0.093	4.7143E-05
Nudt4	2.3154E-09	0.2929677	0.232	0.186	5.0798E-05

Mapre3	2.4417E-09	1.41548034	0.032	0.015	5.357E-05
Dpy19l3	2.7781E-09	1.02123418	0.059	0.034	6.0949E-05
Cpox	3.0167E-09	1.04296267	0.068	0.042	6.6183E-05
Fastkd3	3.0572E-09	1.35271734	0.027	0.012	6.7072E-05
Helz	3.1371E-09	0.62239423	0.167	0.126	6.8826E-05
Espn	3.384E-09	1.23011219	0.042	0.022	7.4242E-05
Nisch	3.5054E-09	0.57369015	0.283	0.243	7.6906E-05
Ube2e2	3.651E-09	2.06997251	0.018	0.007	8.0099E-05
Nxpe4	3.934E-09	2.48014287	0.015	0.005	8.6308E-05
Fbxl12	4.1422E-09	0.55577823	0.16	0.118	9.0876E-05
Klf8	4.3768E-09	2.28200048	0.011	0.003	9.6023E-05
Syne1	4.3905E-09	0.90597574	0.097	0.065	9.6322E-05
Gm5127	4.7415E-09	1.72989946	0.013	0.004	0.00010402
Pkd2	5.0043E-09	1.71925898	0.023	0.01	0.00010979
Tshz3	5.647E-09	1.70767444	0.016	0.006	0.00012389
Mxd4	5.7737E-09	0.52017025	0.223	0.179	0.00012667
Brd1	5.9582E-09	0.49480272	0.322	0.286	0.00013072
Sdccag8	6.2587E-09	1.26823788	0.066	0.041	0.00013731
Dcp1b	6.4608E-09	1.24738324	0.028	0.012	0.00014174
Stk24	6.534E-09	0.39728915	0.376	0.335	0.00014335
Pds5a	6.8535E-09	0.54402021	0.246	0.205	0.00015036
Gm47015	6.9363E-09	0.74877635	0.067	0.041	0.00015218
Nebl	7.0498E-09	0.62448386	0.108	0.074	0.00015467
Cdk11b	7.3882E-09	0.60192945	0.368	0.342	0.00016209
Adck5	7.6058E-09	1.804789	0.026	0.011	0.00016686
Creb1	8.8392E-09	0.60297901	0.202	0.161	0.00019392
Chd9	9.9226E-09	0.78703719	0.122	0.087	0.00021769
Gm17745	1.0074E-08	1.13786574	0.051	0.029	0.00022102
Zcchc7	1.1317E-08	0.43810324	0.33	0.29	0.00024828
Dnajc6	1.2626E-08	1.90822496	0.01	0.003	0.00027701
Hexim1	1.3025E-08	0.46298815	0.221	0.178	0.00028576
Nap1l3	1.436E-08	2.14674673	0.012	0.004	0.00031505
Pls3	1.4574E-08	2.13844289	0.016	0.006	0.00031974
Hist3h2a	1.7488E-08	0.90145923	0.083	0.054	0.00038368
Taf1d	1.868E-08	0.42201615	0.408	0.38	0.00040981
Pdrg1	1.9915E-08	0.98068903	0.096	0.066	0.00043692
Fbxo33	2.0296E-08	0.67856762	0.154	0.117	0.00044526
Prrt1	2.0884E-08	0.89608649	0.073	0.046	0.00045817
Ino80d	2.2245E-08	0.56332146	0.261	0.223	0.00048803
Nop53	2.2309E-08	0.33480645	0.543	0.54	0.00048944
Gm44066	2.4406E-08	1.73841647	0.016	0.006	0.00053544
Orc4	2.5432E-08	0.80348444	0.131	0.097	0.00055796
1700056E22R	2.5874E-08	1.4020036	0.031	0.015	0.00056764
P4ha2	2.6357E-08	1.55817004	0.015	0.006	0.00057825
Cd84	2.6796E-08	0.63514665	0.242	0.204	0.00058788
Rif1	2.8615E-08	0.66228445	0.171	0.133	0.00062777
Sesn1	3.4525E-08	0.69044703	0.081	0.052	0.00075745
Hnrnpll	3.4689E-08	0.82154994	0.199	0.165	0.00076105

Adora2a	3.7913E-08	0.43587035	0.207	0.165	0.00083177
Xist	4.2352E-08	0.78101061	0.411	0.424	0.00092917
Vps13c	4.2507E-08	0.97147654	0.105	0.075	0.00093257
Rab39b	4.8345E-08	1.31590777	0.029	0.014	0.00106063
Gpm6b	5.1373E-08	1.28255678	0.018	0.007	0.00112706
Rasgrp4	5.5016E-08	1.14963964	0.076	0.05	0.00120699
Ubash3a	5.648E-08	0.71177838	0.076	0.049	0.00123912
Zbtb25	5.6797E-08	0.7834107	0.082	0.054	0.00124607
Parp3	5.7091E-08	1.14496201	0.064	0.04	0.00125252
Nufip2	5.7676E-08	0.47985833	0.261	0.222	0.00126536
Kitl	6.4292E-08	1.30603816	0.026	0.012	0.00141051
Cish	9.5677E-08	0.51318124	0.255	0.217	0.00209906
Stk11	1.0154E-07	0.50276433	0.261	0.225	0.00222775
Wisp1	1.0561E-07	1.65156379	0.014	0.005	0.00231705
Kctd17	1.4392E-07	1.53558613	0.037	0.02	0.00315738
Dnah8	1.4717E-07	1.18304781	0.019	0.008	0.00322883
Herc2	1.5761E-07	0.69130339	0.169	0.134	0.00345786
Atn1	1.6819E-07	0.83248459	0.074	0.049	0.0036899
Pomc	1.6932E-07	1.24969736	0.025	0.012	0.00371466
Zfp747	1.7962E-07	1.24911965	0.027	0.013	0.00394074
Ptbp1	1.8342E-07	0.43055742	0.351	0.325	0.0040241
Gdf11	1.8795E-07	1.1623859	0.025	0.011	0.00412345
Ddx24	1.8838E-07	0.43923884	0.498	0.508	0.00413292
Tsix	1.9511E-07	0.90848537	0.16	0.128	0.00428054
Nlgn2	1.9801E-07	2.06583122	0.012	0.004	0.00434404
Btbd19	2.0208E-07	1.4968407	0.026	0.012	0.00443339
Mib1	2.2091E-07	0.88056092	0.153	0.121	0.00484662
Cdkl2	2.2351E-07	1.85352221	0.015	0.006	0.00490359
Cenpv	2.2602E-07	1.48247251	0.032	0.016	0.00495874
Ppm1b	2.5239E-07	0.57155231	0.165	0.129	0.0055371
Pip5k1b	2.6619E-07	1.03622308	0.019	0.008	0.00584
Ppm1l	2.9366E-07	1.06344196	0.026	0.013	0.00644267
Unc5b	3.1191E-07	1.85681045	0.01	0.003	0.0068429
Mipol1	3.3231E-07	1.52968162	0.019	0.008	0.00729045
Fdx1	3.708E-07	0.72808235	0.152	0.119	0.00813501
Arl4a	3.982E-07	0.72357903	0.148	0.115	0.00873602
Cdca7l	4.1189E-07	1.27022564	0.036	0.02	0.00903639
Stn1	4.186E-07	0.8429626	0.064	0.041	0.00918356
Bbx	4.5809E-07	0.56919946	0.185	0.15	0.01004993
Pip4k2b	4.6629E-07	1.15081808	0.049	0.029	0.01023004
Fam199x	4.8262E-07	1.16952323	0.046	0.028	0.01058816
Setd3	5.1924E-07	0.70876028	0.172	0.14	0.01139169
Cep170	5.2E-07	0.91107527	0.133	0.104	0.01140818
0610040B10R	5.2653E-07	0.89131847	0.061	0.039	0.01155146
Pabpn1	5.4164E-07	0.63769212	0.182	0.148	0.01188308
Sema6d	5.7501E-07	1.47856584	0.061	0.039	0.01261524
Prps2	5.8501E-07	0.87209957	0.08	0.055	0.01283462
Ppp6r2	6.2088E-07	1.11672113	0.048	0.029	0.01362139

Ahr	6.2935E-07	0.43632896	0.339	0.307	0.01380734
Arl13b	6.8232E-07	0.7384253	0.134	0.104	0.01496938
Socs5	6.9214E-07	0.77818221	0.059	0.038	0.01518482
Rab11a	7.35E-07	0.61225871	0.245	0.216	0.01612509
Simc1	7.6288E-07	1.05386501	0.037	0.021	0.01673685
Myo1d	7.9707E-07	1.34713001	0.012	0.004	0.01748697
Glud1	8.0411E-07	0.42907193	0.401	0.391	0.01764143
5031425E22R	8.1299E-07	0.55861946	0.151	0.118	0.01783619
Dsel	8.1792E-07	1.63002698	0.018	0.008	0.01794426
Kat2b	8.2265E-07	0.95805431	0.091	0.065	0.01804802
Pmaip1	9.096E-07	0.80620524	0.171	0.14	0.01995569
D3Ertd751e	9.6969E-07	1.19681781	0.033	0.018	0.02127401
Kremen1	9.9544E-07	1.23585533	0.039	0.022	0.02183893
Cdk8	1.0209E-06	0.82301365	0.09	0.064	0.0223965
Srsf11	1.1885E-06	0.33155984	0.503	0.498	0.02607511
Cdc73	1.2132E-06	0.53281154	0.195	0.161	0.02661695
Nf2	1.2743E-06	1.16355607	0.056	0.036	0.02795715
Ltk	1.3244E-06	1.83118683	0.01	0.003	0.02905632
Cep85	1.3531E-06	1.2199192	0.043	0.026	0.02968617
Tmem260	1.3633E-06	1.00670849	0.05	0.031	0.02990944
Tmem106c	1.4461E-06	1.35468771	0.035	0.019	0.0317255
Pkn2	1.46E-06	0.41825808	0.337	0.312	0.03203048
2810454H06R	1.5339E-06	0.89846554	0.06	0.039	0.03365246
Gnb4	1.5498E-06	1.02592376	0.064	0.043	0.03400098
Chd2	1.5852E-06	0.25262986	0.504	0.494	0.03477859
Ppp1r14b	1.7048E-06	0.67827489	0.2	0.171	0.03740101
Ahrr	1.7424E-06	2.06999549	0.012	0.005	0.03822648
Ppp2r1b	1.7897E-06	1.04644066	0.05	0.031	0.03926335
Ndrg1	1.8256E-06	0.80960603	0.194	0.162	0.04005188
Kcnk5	1.8379E-06	1.02900723	0.024	0.012	0.04032168
Zfand6	1.9065E-06	0.51274699	0.298	0.273	0.04182655
Dubr	1.9274E-06	1.0130231	0.066	0.045	0.04228567
Dalrd3	2.0915E-06	0.90796266	0.088	0.063	0.04588649
Tmem154	2.1407E-06	0.73837361	0.05	0.031	0.04696403
Rfx1	2.1829E-06	0.94080892	0.066	0.044	0.04789021
Clspn	2.2359E-06	1.06706326	0.035	0.02	0.04905433
Itgb3bp	2.4261E-06	1.04821506	0.042	0.025	0.05322625
Srsf2	2.6237E-06	0.31591509	0.501	0.499	0.05756125
Zbtb12	2.7678E-06	1.84578271	0.011	0.004	0.06072232
Thra	2.9666E-06	1.18204278	0.035	0.02	0.06508328
Cnih1	2.9869E-06	0.84043595	0.126	0.098	0.06552988
Elf4	3.004E-06	0.59253719	0.124	0.095	0.06590494
Dip2a	3.085E-06	1.77371146	0.021	0.01	0.06768166
Gm17477	3.106E-06	1.45996417	0.022	0.011	0.06814169
Pparg	3.1111E-06	1.71902255	0.019	0.009	0.0682554
Pnisr	3.2157E-06	0.36251592	0.379	0.354	0.07054829
Stim1	3.2576E-06	0.47716081	0.18	0.147	0.07146783
Hdac7	3.3661E-06	0.59778707	0.109	0.082	0.07384849

Rabac1	3.4104E-06	0.28752175	0.454	0.438	0.07481982
Mtmr1	3.4427E-06	0.87024221	0.101	0.075	0.07552924
Jmy	3.53E-06	0.45985183	0.19	0.156	0.07744556
Agbl3	3.5327E-06	1.3959195	0.016	0.007	0.07750347
Paqr3	3.5634E-06	1.69586867	0.011	0.004	0.0781774
Kidins220	3.5672E-06	0.75978968	0.126	0.098	0.07826126
Tmx4	3.7469E-06	0.53396408	0.161	0.13	0.08220275
Lipo3	3.7614E-06	1.15056986	0.048	0.03	0.08252077
Plekha7	3.9457E-06	1.90372664	0.01	0.004	0.08656563
Stk39	4.0112E-06	0.78046757	0.074	0.051	0.08800195
Bola2	4.144E-06	0.33773357	0.421	0.409	0.09091446
Tfdp2	4.2422E-06	1.01246605	0.052	0.033	0.09306856
Rab21	4.7039E-06	0.38960957	0.332	0.308	0.10319811
Noc2l	4.7662E-06	0.56044244	0.196	0.166	0.10456615
D130040H23F	5.0778E-06	1.26150784	0.02	0.01	0.11140175
Cdk6	5.1104E-06	0.66872292	0.155	0.126	0.11211703
Mapre2	5.2805E-06	0.66391217	0.164	0.135	0.11584817
Ormdl1	5.2889E-06	0.92903095	0.055	0.036	0.11603378
Adcy3	5.3469E-06	1.11716234	0.026	0.013	0.1173066
Pfn2	5.6342E-06	1.54010592	0.012	0.005	0.12360833
Akap17b	5.9582E-06	1.6973377	0.015	0.007	0.13071731
Hopx	5.992E-06	0.49316838	0.273	0.239	0.13145848
Rab26	6.3311E-06	1.1437402	0.027	0.015	0.13889904
Cbx5	6.34E-06	0.78343788	0.083	0.059	0.13909235
Xlr4c	6.6524E-06	0.90029433	0.036	0.021	0.14594682
Zfpm1	6.6848E-06	0.90775615	0.055	0.036	0.14665837
Fbxo34	6.7148E-06	0.88588123	0.126	0.098	0.14731594
Cox14	6.8015E-06	0.3733897	0.386	0.373	0.14921806
Fryl	6.8037E-06	0.3219576	0.259	0.222	0.14926568
A230050P20R	7.0248E-06	1.1983747	0.029	0.016	0.15411789
Tbl1x	7.4262E-06	0.50979593	0.215	0.184	0.16292448
Mcu	7.5481E-06	0.79206899	0.05	0.032	0.16559679
Lrig1	7.8612E-06	0.60226858	0.032	0.018	0.17246745
Dcaf15	7.9065E-06	0.65629563	0.111	0.085	0.17346027
Atg16l2	7.94E-06	0.79350017	0.072	0.05	0.17419477
Gimap8	8.3492E-06	0.41517022	0.137	0.106	0.18317216
Rftn1	8.6427E-06	0.70401088	0.13	0.103	0.18961125
Gstcd	8.7899E-06	0.90064322	0.033	0.019	0.19284154
Zfp946	8.8204E-06	1.15812794	0.024	0.012	0.19350982
Trim14	8.8277E-06	1.3632751	0.018	0.009	0.19367174
Ldb1	8.8982E-06	0.62984624	0.141	0.113	0.19521691
Ndrg3	9.2476E-06	0.66013096	0.087	0.064	0.20288364
Sc5d	9.6896E-06	0.71765687	0.108	0.082	0.21258055
Bmi1	1.0254E-05	0.94746193	0.06	0.04	0.22495513
Ankrd44	1.1056E-05	0.34026574	0.345	0.318	0.24255426
Zfp871	1.1872E-05	0.48858216	0.203	0.174	0.26046891
Cdc37	1.2119E-05	0.40797759	0.36	0.348	0.26587227
Qsox1	1.2788E-05	0.6909037	0.084	0.061	0.28054879

Pear1	1.3177E-05	0.98483095	0.035	0.021	0.2890991
Fkbp3	1.3826E-05	0.45170557	0.287	0.259	0.30332713
Rundc1	1.4451E-05	0.99977074	0.038	0.023	0.31704869
Rps6ka5	1.4803E-05	0.79617718	0.06	0.041	0.32476155
Rsrp1	1.5481E-05	0.45492995	0.317	0.293	0.33964303
Elk4	1.576E-05	0.42315704	0.211	0.179	0.34576718
Tmem67	1.6156E-05	1.81427247	0.016	0.007	0.35445695
Pi4ka	1.7286E-05	0.69506355	0.105	0.08	0.37924217
Sacs	1.7929E-05	0.65196977	0.082	0.06	0.39335037
Itpr3	1.7993E-05	0.53902356	0.098	0.072	0.3947459
Nxnl2	1.8806E-05	1.10722431	0.01	0.004	0.41259132
Ypel4	1.9769E-05	1.22172515	0.011	0.004	0.43371654
Axin2	1.9778E-05	0.77259415	0.053	0.035	0.43389975
Fut8	2.0024E-05	0.94333223	0.069	0.049	0.43930682
Zfp950	2.0066E-05	0.73218115	0.061	0.042	0.4402312
Ppp1r9b	2.0248E-05	0.8218825	0.119	0.095	0.44422025
Spcs2	2.0547E-05	0.30288711	0.441	0.434	0.45078987
Tspan32	2.0753E-05	0.71195334	0.06	0.041	0.45530155
Dnmbp	2.1065E-05	1.12316473	0.024	0.013	0.46214917
Aak1	2.1901E-05	0.48812248	0.157	0.128	0.48049091
Srsf6	2.3732E-05	0.59422924	0.273	0.255	0.52064688
Ppp2r5e	2.4722E-05	0.70345538	0.119	0.094	0.54238166
Mthfd2l	2.568E-05	1.47262891	0.032	0.019	0.56339984
Mark3	2.6491E-05	0.70234052	0.196	0.172	0.58117961
Rapgef6	2.6902E-05	0.26461528	0.318	0.287	0.59020565
Slc52a3	2.7087E-05	1.27114462	0.026	0.014	0.59426143
Churc1	2.7508E-05	0.59307962	0.235	0.214	0.60349314
Nsmf	2.7852E-05	1.08620168	0.036	0.022	0.61105002
mt-Nd6	2.803E-05	0.81758017	0.089	0.067	0.6149595
Huwe1	2.8373E-05	0.37897433	0.351	0.333	0.62248429
Ccnt2	2.8589E-05	0.52798186	0.222	0.197	0.62721288
Dnajc1	2.8595E-05	0.4723379	0.225	0.199	0.62734847
Sit1	2.9654E-05	0.89122225	0.04	0.025	0.6505838
Mtr	2.9917E-05	0.80803981	0.069	0.049	0.65635397
Pkd1	3.1508E-05	0.84031524	0.067	0.048	0.69125528
Abcb1a	3.1724E-05	0.34542926	0.208	0.176	0.69598623
Slc25a23	3.3513E-05	1.34919252	0.021	0.011	0.7352331
Sfmbt2	3.3567E-05	1.38822276	0.016	0.007	0.73642694
Hsbp1l1	3.6655E-05	1.12435968	0.015	0.007	0.8041718
Wdr44	3.7988E-05	0.77028937	0.082	0.061	0.83342631
Axin1	3.8844E-05	0.61258298	0.099	0.076	0.85219424
Tyms	3.96E-05	1.38922768	0.034	0.02	0.86878439
Trip12	3.964E-05	0.40966956	0.322	0.305	0.8696552
Tbck	4.0066E-05	0.85171703	0.068	0.048	0.87901162
Foxo3	4.112E-05	0.620185	0.078	0.057	0.90213787
Suv39h2	4.2084E-05	1.19589964	0.023	0.012	0.92328675
Gnptg	4.3864E-05	0.80609692	0.082	0.061	0.96232245
Zfp652	4.4003E-05	0.72449112	0.107	0.083	0.96537847

Mex3c	4.4139E-05	0.78413236	0.128	0.104	0.96836661
Zfp945	4.4192E-05	0.89607733	0.057	0.039	0.96952807
Slc39a4	4.5745E-05	0.80867058	0.04	0.026	1
Fyco1	4.6065E-05	0.75584867	0.077	0.056	1
Gm19557	5.0457E-05	0.79681069	0.022	0.012	1
Bivm	5.0958E-05	2.09884752	0.011	0.004	1
Pon3	5.1819E-05	1.43152394	0.028	0.016	1
Trim44	5.2422E-05	0.60813846	0.149	0.123	1
Tnrc6a	5.284E-05	0.47368365	0.227	0.202	1
Atxn7l1	5.7512E-05	0.76781624	0.118	0.094	1
Intu	5.8481E-05	1.40175431	0.019	0.01	1
Pld1	5.943E-05	1.69880752	0.016	0.008	1
Zbtb33	6.0088E-05	0.82005467	0.07	0.051	1
Qprt	6.1845E-05	1.38958806	0.01	0.004	1
Pianp	6.4261E-05	1.96891019	0.02	0.011	1
Pla2g16	6.4432E-05	0.52963861	0.212	0.187	1
Gprasp1	6.4904E-05	0.76674865	0.042	0.027	1
Rb1cc1	6.7628E-05	0.35999181	0.282	0.257	1
Smg1	6.8222E-05	0.32789144	0.336	0.316	1
Tmem259	6.8666E-05	0.67967302	0.144	0.12	1
Diaph3	6.9351E-05	0.99696791	0.033	0.02	1
Atp6v0a2	7.0926E-05	0.77379233	0.085	0.064	1
Macf1	7.1496E-05	0.29995851	0.402	0.386	1
Ino80dos	7.1519E-05	1.51027825	0.012	0.005	1
Ehbp1l1	7.5321E-05	0.68371893	0.101	0.079	1
Flot2	7.6208E-05	0.98213241	0.057	0.04	1
Irf6	7.7256E-05	1.83192175	0.01	0.004	1
Ighg1	8.0215E-05	0.25596096	0.152	0.124	1
Zbtb21	8.4945E-05	0.71776251	0.128	0.105	1
Scai	8.5308E-05	0.72832806	0.059	0.042	1
Cep57l1	8.5641E-05	1.11187914	0.022	0.012	1
Dzip3	8.6352E-05	1.24839774	0.023	0.013	1
Bora	8.8222E-05	0.99209792	0.033	0.02	1
Mettl8	8.9739E-05	0.93926269	0.017	0.009	1
Dis3l2	9.1742E-05	0.88916741	0.059	0.042	1
Epc2	9.3931E-05	0.47220328	0.202	0.177	1
Ankrd27	9.4099E-05	1.2080514	0.033	0.02	1
Traf3ip2	9.4217E-05	0.74750474	0.05	0.034	1
Arhgef12	9.7933E-05	0.76966112	0.069	0.051	1
Susd2	9.8672E-05	1.36588421	0.012	0.005	1
Susd6	9.8692E-05	0.43858076	0.332	0.316	1
Tm9sf3	9.8736E-05	0.34580948	0.389	0.384	1
Abi3bp	9.9701E-05	0.81695506	0.025	0.014	1
Thap3	0.00010017	0.69887864	0.082	0.061	1
Kmo	0.00010111	1.13359976	0.045	0.03	1
Fam92a	0.00011211	0.7448496	0.058	0.041	1
Dapp1	0.00011285	0.50436897	0.217	0.193	1
Rabgap1l	0.00011867	0.26703994	0.216	0.186	1

Zfp219	0.00011932	1.25676064	0.036	0.023	1
St7	0.00012178	1.11655807	0.051	0.035	1
Zc3h7b	0.00012217	1.06674337	0.042	0.028	1
Pqlc1	0.00012718	0.71556948	0.11	0.088	1
Cbfb	0.0001284	0.68422124	0.156	0.134	1
Zfp329	0.00012878	0.72492355	0.07	0.051	1
Fcho1	0.00013615	0.96165488	0.05	0.034	1
Atl3	0.00013972	0.51627485	0.135	0.111	1
Zbtb37	0.0001452	0.88248022	0.051	0.035	1
Naip5	0.0001467	1.25211612	0.039	0.025	1
Ost4	0.00015028	0.25639222	0.471	0.478	1
Zfp664	0.00015113	0.71878249	0.084	0.064	1
Fra10ac1	0.00015314	1.03640149	0.029	0.017	1
Ints6l	0.00015644	0.83290391	0.062	0.045	1
Gm27010	0.00015944	1.23689434	0.019	0.01	1
Fam216a	0.00016169	0.83653684	0.045	0.031	1
Ubxn11	0.00016574	0.88711712	0.044	0.03	1
Atp6v1g2	0.00016737	1.59913198	0.011	0.005	1
Ptar1	0.00016863	0.91934741	0.027	0.016	1
Dicer1	0.00017019	0.42344583	0.161	0.135	1
Nsun6	0.00017151	0.68995874	0.082	0.063	1
Rcsd1	0.00017331	0.45982759	0.122	0.098	1
Dynll2	0.00017441	0.64224568	0.153	0.13	1
Ttc21b	0.00019507	1.40848593	0.014	0.007	1
Miga1	0.00020239	1.21697098	0.027	0.016	1
Mmp25	0.00020358	1.41773292	0.027	0.016	1
Gsk3b	0.00021413	0.40802827	0.268	0.25	1
Rtel1	0.00021487	1.05161368	0.02	0.011	1
Tgfbr2	0.00021666	0.36573431	0.261	0.236	1
Zc3h6	0.00021927	0.84745409	0.03	0.018	1
Heca	0.00022458	0.30392459	0.254	0.228	1
Pqlc3	0.00022968	1.09837768	0.053	0.037	1
Sytl2	0.00023312	0.47318928	0.094	0.072	1
Clip1	0.00023319	0.5453777	0.169	0.146	1
Pdxp	0.00024851	1.20999544	0.015	0.007	1
4930402H24R	0.00025514	1.03562317	0.038	0.025	1
Reep5	0.00025609	0.30616262	0.409	0.406	1
Cnot10	0.00025799	0.84497355	0.079	0.061	1
Maged1	0.00026246	1.21702934	0.02	0.011	1
Elmo2	0.00026339	0.99424809	0.046	0.032	1
Man2a1	0.00026761	0.45280469	0.214	0.192	1
C2cd2	0.00026864	1.15959375	0.024	0.014	1
1700019D03R	0.00027627	1.08725003	0.012	0.005	1
Ccdc107	0.00027718	0.70969767	0.127	0.107	1
Poli	0.000278	1.11412097	0.02	0.011	1
Lxn	0.00028051	1.29645634	0.038	0.025	1
Unc45a	0.00028096	0.5986126	0.101	0.08	1
Plekhb2	0.00028509	0.60698155	0.154	0.133	1

Rcor3	0.00029639	1.07272607	0.031	0.019	1
Vopp1	0.00029704	0.68985668	0.096	0.076	1
Sf3b3	0.00029891	0.30071398	0.342	0.327	1
Pitpnm1	0.00030149	0.57485469	0.115	0.094	1
Akr1c13	0.00030177	1.40615174	0.021	0.012	1
Gdap2	0.00031939	0.80284961	0.061	0.045	1
1300014J16Ril	0.00033145	1.43693365	0.011	0.005	1
Cars2	0.00033164	0.86783709	0.056	0.04	1
Tdrkh	0.0003376	1.26800587	0.02	0.012	1
Hadh	0.0003424	0.84275579	0.077	0.059	1
Fnip1	0.00034576	0.52132883	0.234	0.215	1
Wac	0.0003478	0.36169467	0.353	0.344	1
Zdhhc20	0.00034825	0.50120888	0.208	0.187	1
Galnt11	0.00035134	0.55049882	0.169	0.147	1
Tspoap1	0.00037728	1.31120108	0.019	0.011	1
Galnt3	0.00038261	1.42958418	0.017	0.009	1
Cd96	0.00038484	0.49793489	0.092	0.072	1
Rbm12b1	0.00039416	0.90424979	0.016	0.009	1
Hnrnph3	0.00039723	0.5089457	0.153	0.131	1
Gm8797	0.00041075	0.84152537	0.104	0.085	1
Ankrd17	0.00042977	0.40634319	0.255	0.235	1
Krba1	0.00044201	0.86130621	0.021	0.012	1
Selenoi	0.00044308	0.75214138	0.083	0.065	1
Vars2	0.00045257	1.01658102	0.018	0.01	1
Gm26669	0.00048537	0.49645285	0.141	0.118	1
Gm14305	0.00050256	1.15890191	0.02	0.012	1
Xpr1	0.00055627	0.53261598	0.151	0.13	1
Snap47	0.00056502	1.26737209	0.015	0.008	1
Kif3c	0.00057948	1.50380914	0.015	0.008	1
Rictor	0.00059649	0.35804431	0.335	0.322	1
Gpbp1	0.0006048	0.29511433	0.378	0.369	1
Pkp3	0.00062065	0.54871635	0.106	0.086	1
Focad	0.00062234	1.17927453	0.018	0.01	1
Mettl7a1	0.00062656	1.36979052	0.024	0.015	1
Arrb1	0.00062673	0.71335562	0.1	0.082	1
Izumo4	0.0006444	0.89010049	0.029	0.019	1
Setdb2	0.00066104	0.69502012	0.045	0.032	1
Gpsm1	0.00066531	1.07171853	0.016	0.009	1
Ranbp6	0.00066737	0.92182628	0.039	0.027	1
Per1	0.00066848	0.38338062	0.248	0.227	1
Gramd1c	0.0006724	1.18529548	0.014	0.007	1
Gabbr1	0.00071492	0.61544922	0.079	0.061	1
Smad4	0.00072005	0.56571792	0.263	0.25	1
Phf6	0.0007247	0.37864653	0.119	0.097	1
Ptges3l	0.00073103	1.7068081	0.013	0.006	1
Ankib1	0.00075279	0.45623115	0.117	0.096	1
Akap1	0.00076511	1.54728701	0.01	0.004	1
Megf9	0.0007905	1.0419578	0.019	0.011	1

Agfg2	0.00079659	1.15487677	0.029	0.019	1
Uri1	0.00080546	0.54406374	0.103	0.084	1
Tfpi	0.00081204	1.36389029	0.016	0.009	1
Baz1b	0.00085757	0.45612006	0.206	0.186	1
Ppp4r3a	0.00087272	0.42266969	0.241	0.221	1
5033430I15Ril	0.00087853	1.07085335	0.019	0.011	1
Vmac	0.0009258	1.40503491	0.012	0.006	1
Gm11110	0.00093738	1.36923113	0.013	0.007	1
Cyp51	0.00093764	0.53899377	0.173	0.153	1
Uspl1	0.00095169	0.54377882	0.109	0.089	1
Zdhhc15	0.00096172	1.47397998	0.01	0.005	1
Dcn	0.00098023	0.3424984	0.111	0.091	1
Eif4e3	0.00098877	0.85712397	0.077	0.061	1
E2f6	0.00098878	1.08806752	0.019	0.011	1
Ccnd3	0.00105345	0.60541684	0.126	0.106	1
2810021J22Ril	0.00107364	1.54928586	0.01	0.005	1
Gnai3	0.00107832	0.37247076	0.283	0.268	1
Tmem170b	0.00108506	0.9400185	0.05	0.036	1
Hmgn5	0.00110637	0.40772819	0.136	0.115	1
Acap3	0.00112724	0.89815195	0.024	0.015	1
Dnajb13	0.00113106	1.16757238	0.012	0.006	1
Htatsf1	0.00113263	0.58448295	0.126	0.106	1
Grk5	0.00113515	1.10750483	0.035	0.024	1
Pgrmc1	0.00117935	0.62782746	0.104	0.086	1
Slc25a36	0.0011986	0.37565363	0.216	0.193	1
Hsf2	0.00120211	0.81271666	0.054	0.04	1
Hectd2	0.00121454	1.31877694	0.012	0.006	1
Fam122b	0.0012439	1.22206319	0.018	0.011	1
Trip11	0.00130344	0.36638392	0.245	0.226	1
Rbm12b2	0.00135563	0.85731612	0.022	0.013	1
Tmem86b	0.00138118	1.25027066	0.011	0.005	1
Egln1	0.00142895	0.60011567	0.121	0.102	1
Pcbp4	0.00146212	1.12460464	0.019	0.011	1
Enpp4	0.00150853	0.67751785	0.038	0.027	1
Cep350	0.00155407	0.60241164	0.194	0.179	1
Miip	0.00156173	0.91429516	0.038	0.026	1
B3gntl1	0.0016283	1.10642492	0.01	0.005	1
A630089N07R	0.00163908	1.48040228	0.011	0.005	1
Actr8	0.00164423	0.63379492	0.045	0.032	1
Nbeal1	0.00166087	0.67410578	0.1	0.083	1
Os9	0.00166195	0.49442133	0.222	0.208	1
Lss	0.00166513	1.1140426	0.015	0.008	1
Dlg3	0.00167544	1.09750931	0.011	0.005	1
Nfic	0.00171119	1.07850789	0.047	0.035	1
Mbd5	0.00173527	0.73034352	0.043	0.031	1
Ski	0.00184246	0.31328941	0.186	0.165	1
Zfp512b	0.00186941	1.04248831	0.018	0.011	1
Dpm3	0.00192582	0.38374032	0.263	0.251	1

Tulp4	0.00193596	0.48958547	0.192	0.175	1
Bmp7	0.00194952	1.16512699	0.013	0.007	1
Zfp579	0.00195061	1.18460042	0.017	0.01	1
Rcl1	0.00199306	0.64556152	0.078	0.062	1
Trub1	0.00201754	1.28713933	0.019	0.011	1
Acap1	0.00205424	0.44922202	0.122	0.102	1
Bpgm	0.00212461	0.93019418	0.03	0.02	1
Ubn2	0.00212644	0.48018635	0.207	0.191	1
Mtrr	0.00218223	0.73315148	0.037	0.026	1
Zfp938	0.00227919	1.02942966	0.034	0.023	1
Mboat1	0.00246611	1.35061139	0.014	0.008	1
Lrrcc1	0.00248263	1.05188086	0.041	0.03	1
Spryd4	0.00254321	0.90406794	0.021	0.013	1
Sla	0.00259031	0.32992421	0.404	0.405	1
Exoc6b	0.00259458	0.82289128	0.056	0.043	1
Hmgcr	0.00262433	0.42573604	0.225	0.21	1
Prrc2a	0.00263684	0.34988681	0.249	0.234	1
Pou2f2	0.00265926	0.35330171	0.237	0.217	1
Lcor	0.00282361	0.70120936	0.069	0.055	1
Deptor	0.00286192	1.28752353	0.028	0.019	1
Phf20l1	0.00288249	0.25101079	0.327	0.311	1
Rpe	0.0029923	0.66486555	0.088	0.073	1
Pank4	0.00308964	0.71331213	0.035	0.024	1
Praf2	0.00313639	1.1000914	0.034	0.024	1
Gm48882	0.00314093	1.29358493	0.01	0.005	1
Zfp692	0.00326888	0.9676776	0.021	0.014	1
B3gat2	0.00327857	1.10631497	0.022	0.014	1
Pias3	0.00329375	0.85252703	0.027	0.018	1
Katnbl1	0.00333773	0.75675373	0.091	0.076	1
Tmem156	0.00357678	0.56969074	0.055	0.042	1
Pigm	0.00357776	0.68979634	0.082	0.067	1
Fbxl18	0.00358513	1.19216789	0.016	0.01	1
Phtf2	0.00358623	0.51821401	0.063	0.049	1
4933406P04R	0.00363952	1.20834838	0.014	0.008	1
Tecr	0.00372754	0.57244791	0.185	0.171	1
Phlda1	0.00375677	0.50304531	0.153	0.135	1
Trove2	0.00378347	0.98849265	0.025	0.017	1
Klf9	0.00384282	0.51664098	0.061	0.048	1
Gm26809	0.00395019	0.72025386	0.03	0.02	1
Sod3	0.00400464	1.36867205	0.011	0.006	1
Scly	0.00405001	0.80526369	0.036	0.026	1
Ercc6l2	0.00405682	0.81722166	0.049	0.037	1
Ankrd10	0.00410518	0.53334836	0.081	0.066	1
Tmem237	0.00423637	1.19230123	0.019	0.012	1
Rhbdd2	0.00431581	0.74989221	0.028	0.019	1
Mfsd4a	0.00435526	0.57334782	0.062	0.048	1
Ankmy2	0.00436403	0.83216711	0.032	0.022	1
Ctif	0.0044592	1.20703683	0.011	0.006	1

Trp53bp1	0.00452948	0.53808846	0.063	0.05	1
2310035C23R	0.00455281	0.47087125	0.149	0.132	1
Ighv1-9	0.00458297	1.12595048	0.01	0.005	1
Arl2bp	0.00458899	0.39563311	0.179	0.162	1
Dynll1	0.00460965	0.3140681	0.381	0.389	1
Galnt7	0.00471257	0.58332509	0.109	0.094	1
Ndufa1	0.00471492	0.30206115	0.408	0.423	1
Prr12	0.00473745	0.82149934	0.022	0.015	1
Hectd4	0.004818	0.82337597	0.038	0.027	1
Snx13	0.00484639	0.56897583	0.069	0.056	1
Sh3kbp1	0.0048823	0.26715708	0.312	0.299	1
AI314180	0.00492547	0.55582953	0.127	0.111	1
Fbxl17	0.00492618	0.83652909	0.049	0.037	1
Gm47283	0.00492728	0.43846317	0.181	0.166	1
Ywhab	0.00493928	0.3720269	0.347	0.352	1
Bpnt1	0.00497785	0.49267761	0.094	0.078	1
Ube3c	0.00498068	0.54330416	0.092	0.076	1
Fam53b	0.00518395	0.69801688	0.039	0.029	1
Ikzf5	0.00518997	0.44097311	0.112	0.096	1
Cdc42se2	0.00534199	0.38250813	0.299	0.295	1
Xpc	0.00539152	0.71156648	0.03	0.021	1
Zfp827	0.00556045	0.91415406	0.011	0.006	1
Guf1	0.00562248	0.93394279	0.039	0.029	1
Cbx7	0.00562475	0.43168419	0.06	0.047	1
Hmbox1	0.00567003	0.6979917	0.067	0.054	1
Mlec	0.00590863	0.48185423	0.16	0.145	1
Rps6kb2	0.00592456	0.98054122	0.057	0.045	1
Phtf1os	0.00596481	0.95974994	0.013	0.007	1
Rab3gap2	0.00598641	0.75727903	0.069	0.056	1
Psen2	0.00618572	0.44786882	0.134	0.118	1
Snrk	0.00620945	0.38284097	0.095	0.079	1
Dusp19	0.0062998	0.97882938	0.027	0.018	1
Nfatc2	0.00638277	0.56361728	0.09	0.075	1
Rps6ka1	0.00649479	0.81128594	0.088	0.075	1
Cdon	0.00660932	0.96031949	0.027	0.019	1
Uty	0.00662324	0.53529119	0.064	0.051	1
Tmco1	0.00671258	0.57228817	0.191	0.18	1
Ap1s2	0.00678304	0.6130374	0.139	0.125	1
Anxa6	0.00697976	0.35271784	0.265	0.255	1
Elk3	0.00709535	0.61656876	0.102	0.087	1
Tnfrsf11a	0.00718213	1.32603814	0.054	0.043	1
Kat6a	0.00719874	0.34357403	0.226	0.21	1
Polm	0.00740604	0.77730955	0.024	0.016	1
Themis	0.00747096	0.28893936	0.119	0.101	1
Rrm2b	0.00759212	0.57834297	0.079	0.065	1
Dopey1	0.00766998	0.42666751	0.09	0.075	1
2900097C17R	0.00792138	0.35314953	0.248	0.239	1
Ahi1	0.00793034	0.42186689	0.094	0.078	1

Phc3	0.00794905	0.38367023	0.157	0.14	1
Slc25a4	0.00809362	0.44439255	0.25	0.244	1
D030056L22R	0.00815061	0.58095949	0.061	0.049	1
Zdhhc21	0.00833317	0.61488887	0.115	0.101	1
Fam208a	0.00860428	0.49571234	0.148	0.133	1
Slco3a1	0.00861648	0.58950213	0.163	0.151	1
Gm26882	0.00876978	0.63690765	0.033	0.024	1
Rab2b	0.00886123	0.59141385	0.054	0.042	1
Asf1a	0.00894882	0.59600664	0.077	0.064	1
Dusp6	0.00903801	0.33947344	0.073	0.059	1
Pnpla6	0.00923175	0.85986744	0.025	0.017	1
Alad	0.009243	0.94372779	0.033	0.024	1
Pla2g6	0.0093615	1.05719897	0.011	0.006	1
Chchd3	0.00942208	0.60902915	0.155	0.142	1
Ppp1r3f	0.00959602	0.71799111	0.012	0.007	1
Cntrob	0.00970096	0.85724605	0.024	0.016	1
Krt10	0.00973082	0.94002674	0.016	0.01	1
Dtl	0.0097818	0.7504388	0.033	0.024	1
Bscl2	0.01023587	0.70982843	0.113	0.1	1
Eif4g3	0.0102662	0.50405599	0.209	0.201	1
Thap12	0.01026954	0.48611467	0.135	0.12	1
Tcta	0.01031345	1.20775042	0.015	0.009	1
Cyth3	0.01048155	0.69186569	0.142	0.13	1
Xndc1	0.0105936	0.71636114	0.026	0.018	1
Gm32856	0.01061534	1.0849008	0.01	0.006	1
Agap2	0.01080448	0.63174606	0.028	0.019	1
Zfp609	0.01082555	0.8979475	0.031	0.023	1
Kif1b	0.01094958	0.36789517	0.15	0.135	1
Dpysl3	0.01100796	1.10608514	0.013	0.008	1
Rap1gds1	0.01108036	0.552486	0.13	0.117	1
Tspyl2	0.01121542	0.8230873	0.041	0.032	1
Tor1aip1	0.01125628	0.4152418	0.327	0.333	1
Kcnmb4	0.01129024	0.4920337	0.053	0.041	1
Haus3	0.01178124	0.38532485	0.113	0.098	1
Nucb1	0.01200005	0.57272578	0.176	0.165	1
Rev3l	0.012203	0.45906271	0.1	0.086	1
Chka	0.01226184	0.34568972	0.127	0.112	1
Agk	0.01272412	0.51086482	0.045	0.035	1
Esco1	0.01277794	0.34993634	0.148	0.133	1
Arid3b	0.01295608	0.56967431	0.042	0.032	1
Cetn2	0.01296709	0.42869564	0.179	0.165	1
Tmx2	0.01303813	0.81173596	0.039	0.03	1
Spin1	0.01306323	0.29872728	0.171	0.155	1
Etfb	0.01309078	0.71413912	0.17	0.162	1
Ccno	0.0131334	0.73804822	0.013	0.008	1
Otud4	0.01320661	0.38887966	0.138	0.123	1
Rab6b	0.01324234	0.76656514	0.02	0.013	1
Gm46430	0.01341573	1.09131364	0.012	0.007	1

Fxyd1	0.01362415	1.09168429	0.01	0.005	1
Alg10b	0.01389179	0.64670465	0.054	0.043	1
Iqce	0.01425399	0.84542521	0.018	0.012	1
Slc35d2	0.01432191	1.01716243	0.018	0.012	1
Hp1bp3	0.01453468	0.35521148	0.272	0.266	1
Fam124b	0.01473213	1.03283488	0.016	0.011	1
Gab3	0.01488872	0.90249133	0.024	0.016	1
Zmat4	0.01522685	0.74168265	0.01	0.006	1
Hells	0.01526973	0.46509466	0.077	0.064	1
Creb3l1	0.0153126	0.78153664	0.02	0.014	1
Immp1l	0.01532653	0.77909744	0.088	0.076	1
Slc17a9	0.01567935	0.77576927	0.017	0.011	1
Cc2d1a	0.01595814	1.0358892	0.022	0.015	1
Rhot2	0.01599869	0.7757344	0.033	0.024	1
Gba2	0.01615571	0.8925807	0.017	0.011	1
Akap9	0.01637724	0.45974154	0.2	0.189	1
Kpna1	0.01645226	0.33637656	0.307	0.302	1
Dnmt1	0.01657864	0.48152075	0.083	0.07	1
Aebp1	0.01660907	0.84212858	0.012	0.007	1
Ccdc92	0.01675893	0.80751428	0.013	0.008	1
Fam160a2	0.01684443	0.65818425	0.027	0.019	1
Dctn4	0.01687007	0.30939457	0.333	0.334	1
Zfp410	0.01694826	0.45251092	0.073	0.061	1
Nkiras1	0.01698559	0.65081241	0.032	0.024	1
Zfp275	0.01706252	0.55621038	0.031	0.023	1
Gm11713	0.01744704	1.14766321	0.017	0.012	1
Ppp1r3e	0.01793861	1.43362894	0.01	0.006	1
C2cd3	0.01795581	0.60711261	0.062	0.051	1
Abca7	0.01804214	0.52121687	0.073	0.061	1
Ddx11	0.01808417	0.85162191	0.017	0.011	1
Hba-a1	0.01815237	0.33496226	0.032	0.024	1
Plscr1	0.01848417	0.43452153	0.146	0.132	1
Ppp2r5b	0.01928049	1.24004419	0.014	0.009	1
Smyd4	0.01972005	0.90756099	0.017	0.011	1
Phactr2	0.01977569	0.26905291	0.057	0.073	1
Ift27	0.02041423	0.97645303	0.037	0.028	1
Pigh	0.02042323	1.10345874	0.015	0.01	1
Pdha1	0.02049348	0.4782231	0.157	0.146	1
Cyp4f16	0.02061909	0.3531578	0.026	0.036	1
Gdpd5	0.0207044	0.99744125	0.021	0.015	1
Prpf39	0.02080916	0.35022267	0.23	0.222	1
4930532G15R	0.02094826	1.20230194	0.014	0.009	1
Ksr1	0.02098412	0.79753889	0.03	0.022	1
Farp1	0.02104544	0.76125571	0.02	0.014	1
Zfp398	0.02108419	0.56948718	0.061	0.05	1
Chmp1b	0.02111687	0.39011834	0.166	0.153	1
Dusp23	0.02133619	1.37892616	0.018	0.012	1
Med14	0.02165983	0.51386409	0.101	0.088	1

Xrcc2	0.02203672	0.92304618	0.01	0.006	1
Tuft1	0.02212715	1.01102895	0.014	0.009	1
Adpgk	0.02215121	0.56908698	0.067	0.056	1
Arhgef11	0.02227228	1.03929588	0.034	0.026	1
Vwa8	0.0222856	0.71121116	0.021	0.015	1
Mkln1	0.02311451	0.3130538	0.246	0.238	1
Klhl26	0.02316416	1.50767504	0.01	0.006	1
Ppargc1b	0.02352995	0.99839333	0.013	0.008	1
Cited2	0.02376816	0.3975524	0.177	0.164	1
Cep250	0.02400334	0.58797129	0.061	0.05	1
Cuedc1	0.02400648	0.76823448	0.018	0.013	1
BC031181	0.02402148	0.29116376	0.277	0.274	1
Gprin3	0.02439147	0.41219594	0.035	0.026	1
Cdr2	0.02456021	0.46436862	0.04	0.031	1
Snrnp48	0.0247707	0.38018347	0.208	0.198	1
Sirt5	0.02536874	0.96260607	0.01	0.006	1
Lnpk	0.02538081	0.40295206	0.097	0.084	1
Slx4	0.02562994	0.73228348	0.021	0.015	1
Zbtb40	0.02611271	0.8401138	0.021	0.015	1
Zfp638	0.02616529	0.35591065	0.211	0.201	1
Pitrm1	0.02617951	0.75909251	0.044	0.035	1
Wrnip1	0.02652119	0.54308967	0.057	0.047	1
Slain2	0.02671971	0.44738648	0.147	0.135	1
Bphl	0.02700122	0.27213476	0.008	0.014	1
Dhcr24	0.0273508	0.81025184	0.034	0.026	1
Edf1	0.02769723	0.33367674	0.385	0.404	1
Ppp1r12b	0.02776999	0.76157326	0.021	0.015	1
Celf1	0.02796377	0.27813596	0.282	0.277	1
Prkaca	0.02829267	0.6843583	0.051	0.042	1
B4galt4	0.02847419	1.55726868	0.01	0.006	1
Bcap31	0.02854927	0.3659979	0.241	0.238	1
Vamp1	0.02874991	0.42629941	0.03	0.022	1
Fam210a	0.02917576	0.74044783	0.039	0.031	1
Fbxo9	0.02917755	0.38403198	0.067	0.056	1
Eed	0.02947424	0.40136669	0.128	0.116	1
Mthfsd	0.02949542	0.82797242	0.022	0.016	1
Cox10	0.02990025	0.65882948	0.061	0.051	1
Faap20	0.03024653	0.64425286	0.056	0.046	1
Fam53c	0.03035269	0.39201721	0.072	0.06	1
Zfp677	0.03052085	0.69223092	0.021	0.015	1
Ndufb9	0.03054424	0.29581553	0.369	0.382	1
Inpp5e	0.03061306	0.82530456	0.021	0.015	1
Itfg1	0.03068214	0.54520971	0.063	0.053	1
R3hdm1	0.03087039	0.32146332	0.247	0.239	1
Insl6	0.03102518	0.62171985	0.058	0.048	1
Eef1akmt2	0.03109102	0.63703648	0.038	0.03	1
Nfat5	0.03156982	0.32208233	0.274	0.27	1
Xxylt1	0.03198991	0.89428103	0.028	0.021	1

B3galnt2	0.03216758	0.75229559	0.038	0.03	1
Psip1	0.03333719	0.35136165	0.158	0.145	1
Kcnq1ot1	0.03339435	0.38490216	0.147	0.134	1
Hbb-bs	0.03356612	0.57331896	0.081	0.07	1
Uvssa	0.03371452	0.71456937	0.036	0.028	1
Smyd2	0.03373476	0.9427524	0.021	0.015	1
Naip6	0.03382301	1.15503434	0.024	0.018	1
Tmem184c	0.03396462	0.66365943	0.027	0.02	1
Fam3c	0.03396566	0.40697613	0.092	0.08	1
Ccdc91	0.0343896	1.28493218	0.015	0.01	1
Tmpo	0.03489837	0.33578064	0.184	0.172	1
Dclk2	0.0349569	0.57997691	0.016	0.011	1
Pcgf2	0.03566924	1.05044643	0.01	0.006	1
Ndufaf7	0.03657835	0.42340663	0.041	0.033	1
Irak1bp1	0.03665611	1.05055326	0.012	0.008	1
Cops4	0.03712735	0.39150313	0.157	0.148	1
Nadk2	0.03796353	0.65601071	0.041	0.033	1
BC017158	0.03814287	1.17589659	0.01	0.006	1
Pcbp3	0.03827674	1.17294507	0.01	0.006	1
Rbbp5	0.03884527	0.47950645	0.051	0.041	1
Mettl4	0.03968401	0.78030714	0.016	0.011	1
Dlc1	0.04003382	0.90704549	0.01	0.006	1
Trbv12-2	0.04007776	0.75878827	0.01	0.006	1
Mtfmt	0.04028903	0.70380058	0.03	0.023	1
Pard6a	0.04052134	0.83332124	0.035	0.027	1
Sugp2	0.04082309	0.6157004	0.049	0.04	1
Yeats2	0.04107185	0.63776895	0.049	0.04	1
Zfp148	0.04108398	0.39126781	0.185	0.175	1
Adipor2	0.04120577	0.34057088	0.21	0.202	1
Csrnp2	0.04145697	0.66580363	0.012	0.008	1
Zfp990	0.04211364	0.76186641	0.01	0.006	1
Myef2	0.04220486	0.47463804	0.096	0.085	1
Wdr5b	0.04277198	1.08878347	0.01	0.006	1
Mical3	0.04315487	0.92093568	0.016	0.011	1
Rgs14	0.04328993	0.78759503	0.034	0.027	1
Pi4k2a	0.04331014	0.30356331	0.136	0.163	1
Decr1	0.04373738	0.90715032	0.028	0.021	1
Taok2	0.0438434	0.50174413	0.071	0.061	1
Ppic	0.04445883	0.75886161	0.014	0.01	1
Cep41	0.04500497	0.91244296	0.018	0.013	1
Fam234a	0.04514522	0.2826944	0.027	0.036	1
Fbxl20	0.045325	0.62238705	0.074	0.064	1
Lysmd2	0.04634995	0.5735974	0.043	0.035	1
Smim15	0.04686983	0.44016981	0.154	0.145	1
Sdf2	0.04733794	0.35673421	0.226	0.221	1
Uckl1	0.04765806	0.2829104	0.03	0.04	1
Fancg	0.04833982	0.89646215	0.01	0.007	1
Enpp5	0.04919821	1.47896874	0.01	0.006	1

Asrgl1	0.04974202	0.64399748	0.043	0.035	1
Kiz	0.05039181	1.04985539	0.025	0.019	1
Tm7sf3	0.05066737	0.88404632	0.033	0.026	1
Upb1	0.05119453	1.50085931	0.01	0.006	1
Mlh1	0.05235418	0.7628067	0.016	0.012	1
Map2k6	0.05235649	0.99389462	0.01	0.007	1
Mta1	0.05264778	0.57028626	0.085	0.075	1
Wasl	0.05305979	0.47887965	0.102	0.092	1
Apol7e	0.05329026	0.60772199	0.027	0.021	1
Fasn	0.05344265	0.50778861	0.062	0.053	1
Plpp5	0.05360941	0.78719393	0.041	0.034	1
Bcap29	0.05388369	0.26577687	0.059	0.073	1
Ndufa3	0.05398106	0.27616715	0.389	0.411	1
Bcl2l13	0.05456352	0.59032528	0.058	0.05	1
Fam83d	0.05470258	0.94227605	0.01	0.007	1
Calhm2	0.05522543	0.82446539	0.017	0.012	1
Gm9856	0.05534404	0.69643345	0.035	0.028	1
3110062M04F	0.05581853	0.56462463	0.037	0.03	1
Ccnb1ip1	0.05596389	0.8077951	0.01	0.007	1
Gsk3a	0.05612019	0.44020022	0.155	0.147	1
Zfp235	0.05632096	0.91346929	0.012	0.008	1
Proscos	0.05718322	0.36232558	0.013	0.019	1
Zhx3	0.05742647	0.89000327	0.016	0.011	1
Ssh1	0.05774269	0.77368935	0.055	0.047	1
Clock	0.05809885	0.64823206	0.1	0.092	1
Msh2	0.05893016	0.5733408	0.04	0.033	1
Gstp3	0.05929092	0.34830561	0.062	0.053	1
Cdkn2aip	0.05948099	0.32274254	0.094	0.083	1
Cbx3	0.06096355	0.30581267	0.284	0.284	1
Arhgef3	0.06098452	0.32111113	0.18	0.172	1
Sqle	0.06131125	0.62236467	0.06	0.052	1
Lrrc58	0.06182886	0.7555727	0.202	0.203	1
D2hgdh	0.06196792	0.79072267	0.015	0.01	1
Prdm11	0.06265662	0.50980266	0.024	0.018	1
Sergef	0.06290546	0.26955161	0.021	0.029	1
Padi2	0.06389548	0.54059509	0.059	0.05	1
Rfx7	0.06396039	0.53507179	0.109	0.1	1
Arid1a	0.06406505	0.27358478	0.208	0.199	1
Cep95	0.0648026	0.86248631	0.014	0.01	1
S100pbp	0.06508496	0.41012362	0.062	0.054	1
Carns1	0.06519566	0.47180943	0.012	0.008	1
Agap3	0.06524072	0.64309102	0.048	0.04	1
Rad52	0.06539968	0.54213512	0.023	0.017	1
Plcg1	0.06568923	0.330214	0.083	0.073	1
Ano8	0.06599775	0.89214487	0.013	0.009	1
Alms1	0.06612225	1.14186186	0.015	0.011	1
Rmi1	0.06655474	0.52349624	0.059	0.051	1
Ripk3	0.06696447	0.26763698	0.029	0.037	1

Upf3b	0.06732189	0.45387229	0.095	0.085	1
Cdk19	0.06782845	0.57497412	0.09	0.081	1
Kif23	0.06926261	0.42498435	0.064	0.055	1
Ccdc66	0.06938468	0.71256364	0.029	0.023	1
Stard4	0.06964532	0.41282715	0.115	0.105	1
Btrc	0.07017822	0.6985005	0.033	0.026	1
Sft2d3	0.07053369	0.45611095	0.055	0.046	1
Tmem191c	0.07106157	0.65964565	0.028	0.022	1
Hic1	0.07208738	0.46105379	0.049	0.041	1
Trim24	0.07276335	0.40443182	0.095	0.085	1
Gm26847	0.07280324	0.69433574	0.01	0.006	1
Fam173a	0.07397393	0.26168945	0.135	0.158	1
9130401M01F	0.07439184	0.63907459	0.082	0.074	1
Ezh1	0.07466111	0.29507297	0.098	0.088	1
Acyp1	0.07471596	0.70747246	0.051	0.044	1
Unc13d	0.07491859	0.63214036	0.048	0.04	1
Ankrd39	0.07601075	0.56894721	0.031	0.025	1
Mtap	0.07642938	0.60225211	0.035	0.029	1
Atp11a	0.077262	0.80608717	0.041	0.034	1
Sh3bp4	0.07753171	1.11329038	0.028	0.022	1
Vps33b	0.07844496	0.74005307	0.022	0.017	1
Polr3h	0.0785021	0.83676549	0.019	0.014	1
Ube2j1	0.07881529	0.39769718	0.244	0.247	1
Tbc1d8b	0.0789374	0.86037861	0.027	0.021	1
Cdpf1	0.08003065	0.88011648	0.02	0.016	1
Sirt2	0.08091728	0.44182549	0.185	0.182	1
Sacm1l	0.08163593	0.68589628	0.079	0.071	1
Ints6	0.08233818	0.5374186	0.098	0.089	1
Smg6	0.08238046	0.26481073	0.145	0.135	1
Alkbh2	0.08272898	0.95010228	0.016	0.012	1
Rbbp4	0.08340822	0.310723	0.266	0.266	1
Cisd3	0.08356597	0.73967165	0.055	0.047	1
Exoc4	0.08437367	0.44319238	0.105	0.096	1
Rnf170	0.08501941	0.77952478	0.033	0.027	1
Uqcr11	0.08527282	0.42876216	0.289	0.302	1
Fam102b	0.0854608	0.3116907	0.068	0.081	1
Hmcn2	0.08636898	0.51357316	0.013	0.009	1
Ttc39c	0.08731407	0.27171596	0.084	0.075	1
Kif16b	0.08752803	0.51723428	0.041	0.034	1
Homer1	0.08816079	0.42864032	0.098	0.089	1
Txlng	0.08838369	0.56594931	0.056	0.048	1
Mllt10	0.0893578	0.42444218	0.125	0.117	1
Tbc1d9b	0.08937617	0.49820654	0.055	0.048	1
Cxcl12	0.08979893	0.57486205	0.025	0.019	1
Ints8	0.08998395	0.58295172	0.044	0.037	1
Mtx2	0.09021713	0.26550718	0.032	0.04	1
Btbd9	0.09024535	0.43832189	0.09	0.082	1
Ankrd50	0.09049294	0.53854616	0.053	0.046	1

Nudcd3	0.09063175	0.39507079	0.186	0.181	1
Iffo1	0.09095099	0.64345454	0.029	0.023	1
Ccdc28a	0.0910157	0.62701654	0.032	0.026	1
Rtn4rl1	0.09129294	0.66004619	0.015	0.011	1
Sirt1	0.09158121	0.34176191	0.099	0.089	1
Unkl	0.09187786	0.3707774	0.038	0.032	1
Kmt5c	0.09200475	0.73643911	0.024	0.019	1
Pdpr	0.09266524	0.36869156	0.042	0.035	1
Lrrc45	0.09278372	0.99653738	0.02	0.015	1
Copz2	0.09284787	0.37021183	0.006	0.01	1
Kdm3b	0.09325449	0.34592935	0.125	0.116	1
Dnm1l	0.09339343	0.44251358	0.133	0.126	1
Ppp6r1	0.09357109	0.46421407	0.175	0.171	1
Zfp317	0.09367137	0.47077382	0.026	0.021	1
Zfp397	0.09425477	0.29178563	0.092	0.083	1
Ptger4	0.09436493	0.39322494	0.17	0.163	1
Zmat1	0.09443404	0.49247915	0.013	0.01	1
Mdh1	0.09569297	0.40872907	0.267	0.274	1
P2rx7	0.09590228	0.6835046	0.056	0.049	1
Ric8b	0.09664748	0.78108567	0.025	0.019	1
Mast2	0.096991	0.68755374	0.03	0.024	1
Fam35a	0.09718049	0.67918785	0.02	0.016	1
Limk1	0.09724999	0.96798958	0.019	0.015	1
Rpn2	0.09831727	0.25654342	0.19	0.221	1
Sh3rf1	0.09883091	0.57915173	0.018	0.014	1
Gbp8	0.09883794	0.59880062	0.063	0.056	1
Camk2b	0.09918889	0.53917944	0.022	0.018	1
Jag1	0.09950229	0.31609553	0.017	0.023	1
Selenoo	0.09965551	0.38978913	0.019	0.026	1
Cdkl3	0.10020682	0.36555579	0.011	0.008	1
Aagab	0.10052459	0.50382156	0.076	0.068	1
Rps6kb1	0.10084027	0.29300673	0.176	0.169	1
Mcee	0.10133406	0.33806716	0.037	0.046	1
Micu3	0.10149787	0.65327628	0.031	0.025	1
Armc6	0.10220129	1.07576749	0.01	0.007	1
Usp20	0.10239066	0.76351254	0.016	0.012	1
Dpysl2	0.10243044	0.29504529	0.151	0.175	1
Mtmr3	0.10306171	0.34195104	0.119	0.11	1
Scmh1	0.10320477	0.6944528	0.036	0.03	1
Klhl11	0.10329203	0.61501705	0.02	0.016	1
Akap11	0.10393115	0.545515	0.079	0.071	1
Snx25	0.10529813	0.61604409	0.027	0.022	1
Pomgnt1	0.10533477	0.88166981	0.018	0.014	1
1810043G02R	0.10621762	0.56042109	0.014	0.019	1
Gpr108	0.10630414	0.3450545	0.054	0.064	1
Eif2d	0.10633434	0.47278688	0.048	0.041	1
1700052K11R	0.10681287	0.36572194	0.013	0.01	1
Azi2	0.10719175	0.28208591	0.185	0.179	1

Ahcy	0.10733929	0.5536599	0.013	0.009	1
Parg	0.10842448	0.41204453	0.101	0.092	1
Luc7l	0.10849765	0.37290329	0.135	0.127	1
Kif13a	0.10923425	0.27643558	0.011	0.016	1
Mturn	0.10936973	0.64913432	0.016	0.012	1
Nsun3	0.10993926	0.46779733	0.034	0.028	1
Cyb5d2	0.11012664	0.61137546	0.021	0.016	1
Nr2f6	0.11022185	0.28512051	0.013	0.018	1
Aig1	0.11029923	0.51977377	0.12	0.113	1
Fam219b	0.11046827	0.60832194	0.025	0.02	1
Otud3	0.11131115	0.86075094	0.019	0.015	1
Tnfsf4	0.11153492	0.6636162	0.016	0.012	1
Herc3	0.11209311	0.25583627	0.055	0.047	1
Pcmtd2	0.11278923	0.28885721	0.107	0.098	1
Ubxn7	0.11279859	0.37022835	0.095	0.086	1
Slc4a8	0.11301151	0.86383045	0.02	0.016	1
Kcnab2	0.11464708	0.4681415	0.061	0.054	1
Wdr13	0.11542689	0.91306576	0.029	0.024	1
Plpp6	0.11573243	0.50326281	0.034	0.028	1
Atp1b2	0.11585674	0.7103769	0.072	0.063	1
Dip2b	0.11617117	0.29986786	0.199	0.193	1
Tbc1d12	0.11690039	0.76122512	0.016	0.012	1
Dgcr6	0.1183823	0.84369147	0.029	0.024	1
Unc119	0.11871671	0.34031174	0.066	0.078	1
Naxe	0.11955262	0.45684882	0.066	0.078	1
Iars2	0.11978177	0.38569519	0.079	0.071	1
Brpf3	0.11991871	0.51969405	0.026	0.021	1
Ifi27	0.12041777	0.28706433	0.203	0.198	1
Spg20	0.12185605	0.63317055	0.03	0.025	1
Ap3s1	0.12215664	0.31457745	0.215	0.213	1
Rufy2	0.12224741	0.42422238	0.061	0.054	1
Smim11	0.12260916	0.37468303	0.143	0.137	1
Tdrp	0.1232759	0.54266091	0.014	0.011	1
Ncor2	0.12329124	0.52842938	0.082	0.075	1
Mfsd8	0.1240325	0.65086038	0.026	0.021	1
Recql	0.1245456	0.67478074	0.028	0.023	1
Gipc1	0.12551732	0.35370504	0.01	0.015	1
Prmt2	0.12591524	0.8030321	0.013	0.01	1
Fam168a	0.12616322	0.48159646	0.086	0.079	1
Rab11fip2	0.12772854	0.38376264	0.086	0.078	1
1700012B07R	0.12802381	0.59727587	0.022	0.017	1
Piezo1	0.12852136	0.29091692	0.064	0.076	1
B3gat3	0.12915299	0.5511374	0.048	0.042	1
Aqp3	0.12946684	0.56718509	0.016	0.012	1
Stat1	0.13025269	0.28804245	0.347	0.353	1
Tmem201	0.13027754	0.7358471	0.016	0.012	1
Pygb	0.13090442	0.69732076	0.04	0.034	1
Sil1	0.13093199	0.27430764	0.021	0.027	1

Trrap	0.13211913	0.46692777	0.082	0.075	1
Tgfb3	0.13276173	0.55712384	0.02	0.016	1
Zbtb7b	0.13296593	0.7270174	0.038	0.032	1
Sclt1	0.1333208	0.54371098	0.046	0.04	1
Ncoa3	0.13427242	0.29943965	0.157	0.15	1
Ttll3	0.13478459	0.59106623	0.015	0.011	1
Selenof	0.13510822	0.29422652	0.316	0.331	1
Ppfia1	0.13529013	0.29970254	0.144	0.136	1
Phf21a	0.13560969	0.38428601	0.09	0.082	1
Sp3os	0.13690806	0.29348502	0.045	0.039	1
Fbxw5	0.13695225	0.54228686	0.035	0.029	1
Srsf1	0.13748954	0.28812691	0.194	0.189	1
Maz	0.13826747	0.59230568	0.089	0.083	1
Mapk1ip1	0.13910351	0.37582009	0.026	0.021	1
Zfp740	0.13941724	0.39298309	0.081	0.074	1
Gfpt2	0.14012605	0.4296018	0.014	0.011	1
Luzp1	0.14285625	0.26700256	0.05	0.059	1
Serhl	0.14286003	0.29774892	0.025	0.031	1
Flvcr1	0.14348616	0.27938716	0.038	0.046	1
Wdfy2	0.14391786	0.5880345	0.069	0.063	1
Gtf3c1	0.14488551	0.29127272	0.075	0.067	1
Clasp1	0.14652969	0.44045552	0.072	0.065	1
Vdac1	0.14721313	0.32171799	0.161	0.187	1
Slc35a1	0.14849759	0.80603957	0.026	0.021	1
Xpo6	0.14891121	0.33588312	0.13	0.123	1
Gfi1	0.1490189	0.27427011	0.039	0.033	1
Mocs2	0.1501419	0.30795232	0.072	0.083	1
Uros	0.15184999	0.81129577	0.01	0.007	1
Rusc1	0.15252979	0.50961608	0.064	0.057	1
Fes	0.15422373	0.32627663	0.056	0.066	1
Cdc7	0.1546053	0.62522782	0.022	0.018	1
Pde8a	0.1547004	0.27860265	0.03	0.037	1
Snhg20	0.15512779	0.53228601	0.054	0.048	1
Tmem164	0.15679516	0.55598486	0.042	0.037	1
9930104L06Ri	0.1571513	0.80447133	0.011	0.008	1
Agpat1	0.15744076	0.63412403	0.046	0.04	1
Mettl13	0.15766064	0.43846799	0.024	0.02	1
Tmem55a	0.15832669	0.731543	0.021	0.017	1
Zfp868	0.15833548	0.57026684	0.06	0.053	1
Tmed8	0.15845594	0.42382024	0.039	0.034	1
Atm	0.15885827	0.332358	0.048	0.042	1
Tmem181a	0.16038379	0.87281676	0.033	0.028	1
Purg	0.16097904	1.05510382	0.011	0.008	1
Pms1	0.16124103	0.58758138	0.014	0.011	1
Rcn1	0.16253228	0.55310923	0.022	0.018	1
Ankrd40	0.16378731	0.5079028	0.074	0.068	1
Platr25	0.16415087	0.92320842	0.014	0.011	1
Tpgs2	0.16589873	0.55205184	0.036	0.031	1

Ipo7	0.16725004	0.38829563	0.18	0.178	1
Gm42722	0.16766677	0.66899457	0.014	0.011	1
Arl6ip5	0.16777931	0.30329246	0.216	0.215	1
Cenpx	0.1685028	0.34888153	0.125	0.144	1
Tmem80	0.16886574	0.4528136	0.035	0.03	1
Adck2	0.16943124	0.71948521	0.021	0.017	1
Chd6	0.17008679	0.25656601	0.169	0.162	1
Slc52a2	0.1703326	0.49296695	0.026	0.022	1
Zmym6	0.1713247	0.86840823	0.016	0.013	1
Ift43	0.17147707	1.13970066	0.012	0.009	1
Fpgt	0.17168701	0.48385515	0.033	0.028	1
Akr1b3	0.1717685	0.28917827	0.175	0.201	1
Ap3m2	0.17335435	0.51817592	0.028	0.024	1
2810402E24R	0.17499816	0.51879103	0.064	0.058	1
Sh3bp1	0.17517042	0.3244894	0.072	0.084	1
Atxn10	0.17565439	0.60079938	0.106	0.101	1
Slc5a3	0.17584023	0.33325552	0.089	0.082	1
Decr2	0.180677	0.82910641	0.018	0.014	1
Zfp280c	0.18311756	0.53635423	0.027	0.023	1
Pofut1	0.18675623	0.55840906	0.036	0.031	1
Sdr39u1	0.18773035	0.59527006	0.017	0.014	1
Fam20b	0.18776753	0.65849248	0.043	0.038	1
Hmbs	0.18849152	0.28283651	0.019	0.024	1
Stard3nl	0.18866211	0.31277201	0.086	0.099	1
Bub1b	0.18952241	0.61815036	0.017	0.014	1
Ttll4	0.19020609	0.59208694	0.022	0.018	1
Aga	0.19134848	0.73139851	0.045	0.04	1
Gtpbp2	0.19166196	0.37949335	0.14	0.135	1
Gls2	0.19308388	0.75673686	0.01	0.007	1
Lpcat4	0.1942686	0.64654929	0.026	0.022	1
Mocos	0.19502598	0.35351871	0.007	0.01	1
Gid4	0.19650953	0.26316764	0.046	0.054	1
Ppfibp1	0.19658355	0.76836029	0.045	0.04	1
Tbc1d31	0.19717809	0.84701243	0.024	0.02	1
Slc41a3	0.19746752	0.36860609	0.012	0.016	1
Inpp5k	0.20213143	0.37792226	0.066	0.061	1
Inpp4a	0.20222305	0.51463014	0.066	0.06	1
Ipo11	0.20247686	0.60822434	0.026	0.021	1
Rbm4	0.20267023	0.4918367	0.022	0.019	1
AW549877	0.20286928	0.29239793	0.105	0.098	1
Rdx	0.20461368	0.35727478	0.275	0.282	1
Pgam5	0.20508895	0.57543946	0.044	0.039	1
Mzt1	0.20686818	0.42165277	0.095	0.089	1
Atf5	0.20730698	0.56284472	0.025	0.021	1
Slc6a13	0.20740096	0.3193993	0.01	0.008	1
Dcaf6	0.20744382	0.56198261	0.024	0.02	1
Pskh1	0.20776293	0.65922575	0.024	0.02	1
Gm16973	0.21065682	0.45599239	0.017	0.014	1

Zfp523	0.21230861	0.70022984	0.011	0.008	1
Scfd2	0.21301476	0.47139084	0.026	0.021	1
Fktn	0.21311627	1.0348809	0.011	0.009	1
Kctd9	0.21388394	0.45736925	0.03	0.026	1
Tcea1	0.21439952	0.27811535	0.313	0.327	1
Plekha5	0.21465613	0.31633689	0.069	0.063	1
Frs2	0.21624901	0.36651393	0.057	0.051	1
Clec16a	0.2173023	0.59228759	0.02	0.017	1
Ppp6r3	0.21784898	0.29400722	0.222	0.223	1
Lypla2	0.2185171	0.26858805	0.107	0.121	1
Zfp251	0.21953456	0.74417787	0.012	0.009	1
Tdrd3	0.22015207	0.57245337	0.025	0.021	1
Pdss2	0.22049604	0.67159796	0.014	0.011	1
Inppl1	0.22192405	0.77313857	0.03	0.026	1
Lrrc28	0.22205686	0.55333506	0.021	0.018	1
St7l	0.22263903	0.28234949	0.029	0.035	1
Helq	0.22406339	0.5636819	0.012	0.01	1
Fars2	0.22437431	0.34900503	0.033	0.039	1
Fance	0.2246105	0.27193634	0.009	0.012	1
Tsg101	0.22828723	0.40152229	0.132	0.128	1
Akap8l	0.23054661	0.35652891	0.1	0.095	1
Zfp467	0.23055831	0.45982058	0.018	0.023	1
Mpv17l2	0.23069822	0.508164	0.035	0.042	1
Rprd1a	0.23174895	1.01040106	0.01	0.007	1
Arf3	0.23320013	0.27161235	0.124	0.14	1
Mettl5	0.23390425	0.32209054	0.025	0.029	1
Casc3	0.23449569	0.42283908	0.169	0.167	1
Inpp5b	0.23621218	0.64124275	0.035	0.03	1
Nudt19	0.23716272	0.77956416	0.034	0.03	1
R3hdm4	0.23930461	0.34157007	0.146	0.143	1
Tchp	0.24021505	0.5208525	0.019	0.016	1
Rcn2	0.24027705	0.29398108	0.083	0.094	1
Dmtf1	0.24287966	0.27984777	0.129	0.123	1
Zc3h11a	0.24339673	0.64066824	0.024	0.02	1
Wrn	0.24587025	0.43551466	0.068	0.063	1
Arl5b	0.24587138	0.25535919	0.169	0.166	1
Aasdh	0.24606114	0.69142467	0.016	0.013	1
Hs6st1	0.24734243	0.48715464	0.103	0.099	1
Cmc4	0.24736782	0.85812105	0.019	0.016	1
Aaas	0.24876031	0.47609147	0.02	0.017	1
Ahdc1	0.25106296	0.46658095	0.017	0.014	1
Nlrc5	0.25222588	0.32016512	0.091	0.086	1
Kdm6a	0.25432152	0.34189114	0.174	0.173	1
Dpy19l4	0.25571421	0.83168005	0.034	0.03	1
Kdm4c	0.25812081	0.51220134	0.053	0.048	1
Cad	0.25914063	0.49064935	0.026	0.022	1
Ece1	0.25918135	0.50797307	0.183	0.209	1
Ptch1	0.26147598	0.61033262	0.031	0.027	1

Psma3	0.26330738	0.25212017	0.428	0.466	1
Pbx3	0.26340266	0.76815156	0.02	0.017	1
Ccdc191	0.26355444	0.79928647	0.012	0.01	1
2510002D24R	0.26432427	0.28267759	0.084	0.079	1
Klhl25	0.26440068	0.45394331	0.024	0.02	1
Ube4a	0.26441777	0.28005537	0.04	0.047	1
Isoc2b	0.26740415	0.34256257	0.011	0.014	1
Cd99l2	0.26741963	0.61891459	0.019	0.016	1
Elp4	0.26775466	0.45754866	0.009	0.011	1
Zkscan3	0.26799373	0.40841113	0.072	0.067	1
Dcaf5	0.26838477	0.39720056	0.051	0.046	1
Ssbp4	0.26950797	0.32658461	0.102	0.115	1
Wdr11	0.27076988	0.36142668	0.023	0.027	1
Cbwd1	0.27106464	0.70233239	0.026	0.023	1
Rbm14	0.27123003	0.37535161	0.051	0.046	1
Map4k5	0.27277332	0.3090466	0.022	0.026	1
Rbak	0.27300319	0.57888275	0.013	0.011	1
Armcx2	0.27335149	0.53592414	0.012	0.01	1
Marf1	0.27339088	0.26896804	0.124	0.119	1
Nprl3	0.27427247	0.51766924	0.021	0.018	1
Yod1	0.27680157	0.46727684	0.047	0.043	1
Cnst	0.27802949	0.32274051	0.037	0.033	1
Alg12	0.27909882	0.37511297	0.01	0.013	1
Scin	0.27930513	0.41641928	0.009	0.011	1
Tmem167b	0.27955292	0.49236779	0.073	0.068	1
Lin54	0.28143756	0.38029739	0.192	0.194	1
Arhgap27os3	0.28542068	0.56705644	0.011	0.008	1
Usp36	0.28734986	0.25596619	0.097	0.092	1
Ccny	0.28783433	0.4347925	0.108	0.104	1
4833403J16Ri	0.28977692	0.4334523	0.017	0.014	1
Rheb	0.2898433	0.37012153	0.261	0.276	1
Pbx2	0.28985741	0.31325726	0.035	0.04	1
Gstp1	0.29023844	0.51167711	0.025	0.022	1
Akt1	0.29032103	0.27481708	0.098	0.11	1
Kif13b	0.29044657	0.46734934	0.057	0.053	1
Pwwp2b	0.29050062	0.79141454	0.013	0.01	1
Ammecr1	0.29317884	0.72621176	0.017	0.014	1
Ppa2	0.293254	0.61456195	0.034	0.03	1
St13	0.29382214	0.28445072	0.33	0.349	1
Slc9a9	0.29426189	0.36918099	0.058	0.066	1
Ubr4	0.29565591	0.38678571	0.158	0.157	1
Cmtm4	0.29594493	0.39851608	0.013	0.016	1
Fdxr	0.2970275	0.44166215	0.03	0.026	1
Msl1	0.29767541	0.41123637	0.095	0.09	1
Gm20234	0.29847386	0.51714865	0.012	0.01	1
Lrrc8d	0.29944679	0.26391386	0.248	0.255	1
Ugdh	0.30138857	0.50698304	0.064	0.06	1
2810405F17Ri	0.3018848	0.55041981	0.018	0.015	1

Tbc1d10c	0.30221561	0.38256626	0.073	0.068	1
Dcun1d2	0.30241575	0.26080312	0.008	0.01	1
Sgsm2	0.30302496	0.84760681	0.012	0.01	1
Strn3	0.30541046	0.29669497	0.156	0.153	1
Tomm34	0.30639048	0.34124572	0.101	0.097	1
Gm38394	0.3076008	0.54617062	0.016	0.013	1
Exoc7	0.30761418	0.36999245	0.028	0.032	1
Mipep	0.30916514	0.26608125	0.013	0.016	1
Keap1	0.30980383	0.59219067	0.053	0.049	1
Abcc4	0.30995472	0.28334789	0.019	0.023	1
Mccc1	0.3111498	0.77518695	0.014	0.011	1
Efcab14	0.31212097	0.49437331	0.085	0.081	1
Stx16	0.31342682	0.40833231	0.127	0.125	1
Zfp932	0.31456183	0.52025492	0.026	0.022	1
Ubtf	0.31702059	0.25698867	0.167	0.164	1
Wwox	0.31909747	0.67463151	0.026	0.023	1
Man2b2	0.31953064	0.25655964	0.017	0.021	1
Coro2a	0.32209456	0.50024863	0.076	0.072	1
Vps13d	0.32220217	0.34219957	0.095	0.09	1
Gm340	0.32362928	0.48646711	0.02	0.018	1
Synj2	0.32435057	0.49432362	0.042	0.038	1
Ext2	0.32717603	0.27860551	0.035	0.04	1
Hnrnpl	0.32740921	0.29388126	0.276	0.286	1
Ptdss2	0.32787382	0.32434138	0.011	0.014	1
B230219D22R	0.3296081	0.30465311	0.255	0.266	1
Cep76	0.33280223	0.63323944	0.016	0.014	1
Abca5	0.33539606	0.73741602	0.01	0.008	1
Zfp619	0.33576003	0.50912181	0.01	0.008	1
Mtg2	0.33591808	0.70329782	0.021	0.018	1
Vill	0.33592016	0.65483021	0.016	0.019	1
4732440D04R	0.33755298	0.30492908	0.037	0.042	1
Gm17018	0.33888127	0.37046943	0.058	0.065	1
Smad1	0.33948041	0.28368596	0.035	0.04	1
Flywch1	0.34273826	0.53458089	0.019	0.016	1
Serf1	0.34331487	0.26550087	0.013	0.016	1
Zfp758	0.34385319	0.47185558	0.033	0.03	1
Col4a3bp	0.34396357	0.33390773	0.164	0.164	1
Cradd	0.34523077	0.78762777	0.02	0.017	1
Tial1	0.34589779	0.44425459	0.134	0.134	1
Ank	0.34741148	0.3241228	0.045	0.051	1
D5Ertd579e	0.34787881	0.37719682	0.084	0.08	1
Zfp956	0.34797604	0.32949465	0.014	0.012	1
Gtf3c3	0.35043333	0.25432221	0.034	0.038	1
Palm	0.35048622	0.36225115	0.01	0.012	1
Ipo8	0.35190942	0.5601381	0.065	0.062	1
Pop1	0.35309317	0.25985017	0.012	0.01	1
Spg7	0.35468477	0.56267886	0.082	0.079	1
Cnpy4	0.35490366	0.29107967	0.018	0.021	1

Ggps1	0.3553055	0.30489442	0.091	0.087	1
Casp1	0.3555323	0.49051006	0.061	0.058	1
Phf12	0.36118318	0.42518667	0.072	0.068	1
Usp2	0.36203696	0.67183203	0.019	0.017	1
Zfp358	0.36211359	0.36775869	0.008	0.01	1
Rnf4	0.36275306	0.30304794	0.22	0.226	1
Ebpl	0.36426627	0.49408627	0.052	0.048	1
Ddx19b	0.36507989	0.25816669	0.031	0.035	1
H2-Ke6	0.36557342	0.31508046	0.082	0.09	1
Chst11	0.36899483	0.39140034	0.09	0.087	1
Tpx2	0.37040693	0.56701796	0.016	0.014	1
Klhdc1	0.37048411	0.47872823	0.018	0.016	1
Babam1	0.37052986	0.30795098	0.066	0.073	1
Akr1b10	0.37194211	0.32684319	0.029	0.033	1
Atg4a	0.37262564	0.53962299	0.018	0.015	1
Cep83	0.37330696	0.25322861	0.111	0.122	1
Ankrd26	0.37346893	0.54549274	0.014	0.012	1
Rarres2	0.37406613	0.39931405	0.024	0.021	1
Usp47	0.37407488	0.32283125	0.146	0.145	1
Slmap	0.37535066	0.35914865	0.074	0.07	1
G6pc3	0.37748598	0.36448765	0.029	0.033	1
3010003L21Ri	0.37754958	0.60549092	0.012	0.014	1
Kctd13	0.37763579	0.46495174	0.025	0.022	1
Wdr20	0.37892099	0.30359772	0.091	0.088	1
Utp11	0.37911981	0.26517544	0.118	0.13	1
Ubr5	0.38229807	0.31064874	0.217	0.223	1
Sirt6	0.38262953	0.3488739	0.009	0.011	1
Zfp607a	0.38294828	0.32199503	0.018	0.016	1
Clpb	0.38382627	0.38983123	0.018	0.021	1
Riox2	0.38532202	0.33476528	0.041	0.046	1
Mlh3	0.3855189	0.73566387	0.011	0.009	1
Zfp236	0.38583818	0.36336485	0.053	0.05	1
Tbccd1	0.38819541	0.30299139	0.01	0.012	1
Nsd1	0.38847036	0.29475949	0.221	0.227	1
Mapk8ip3	0.39057764	0.41595758	0.079	0.076	1
Ercc4	0.39184245	0.30010372	0.025	0.029	1
Ccdc77	0.39237497	0.53007379	0.015	0.013	1
Slc1a5	0.39782789	0.35833009	0.129	0.127	1
Txndc12	0.40253397	0.35384987	0.03	0.034	1
Hps1	0.40253715	0.31274614	0.03	0.034	1
Mbd3	0.40329933	0.54086622	0.084	0.082	1
Med15	0.40527824	0.28696717	0.157	0.157	1
Hemk1	0.40535966	0.30244062	0.014	0.012	1
Zkscan17	0.40540237	0.28649115	0.027	0.03	1
1500011K16R	0.40543803	0.48846868	0.098	0.109	1
Stambpl1	0.40590761	0.26142117	0.025	0.022	1
Zscan29	0.4097169	0.34550467	0.028	0.025	1
Zfp511	0.41140715	0.31723636	0.072	0.079	1

Ttc13	0.41179209	0.53154907	0.021	0.019	1
Wdr47	0.41372316	0.58886494	0.024	0.021	1
Zfp846	0.41534563	0.28489879	0.011	0.013	1
Sae1	0.41589788	0.4234718	0.069	0.066	1
Cd226	0.41801011	0.35167191	0.054	0.05	1
Poc1b	0.41949393	0.35229487	0.022	0.02	1
Rfng	0.42007322	0.27812765	0.013	0.016	1
Bag3	0.42156271	0.2780145	0.058	0.055	1
Ddx51	0.42316745	0.51287894	0.018	0.016	1
Smarcal1	0.42479812	0.36590253	0.015	0.017	1
Med23	0.42486312	0.45492965	0.051	0.047	1
Slc25a40	0.42548041	0.33270441	0.044	0.041	1
Trp53i13	0.42561558	0.49358722	0.024	0.022	1
Ptdss1	0.4265995	0.40522336	0.057	0.054	1
Lcorl	0.42982241	0.37193426	0.057	0.063	1
Ptgr2	0.42986519	0.67317677	0.022	0.02	1
Rab11fip5	0.43090668	0.77226488	0.019	0.017	1
Trip10	0.43116541	0.25866699	0.013	0.015	1
Tmem9	0.43146868	0.54971163	0.017	0.015	1
Ndfip2	0.43157172	0.33562622	0.178	0.181	1
Glb1	0.43418885	0.59142341	0.031	0.029	1
Amd1	0.4342249	0.34127343	0.069	0.066	1
Luc7l3	0.43529029	0.31780292	0.196	0.202	1
Mfsd13a	0.43561911	0.91470142	0.012	0.01	1
1110051M20F	0.43573194	0.57912951	0.009	0.011	1
Gga1	0.43602002	0.26076895	0.058	0.064	1
Foxj3	0.43666018	0.31694398	0.099	0.096	1
Zkscan8	0.43808107	0.57730106	0.014	0.012	1
Epc1	0.44031013	0.26000402	0.203	0.205	1
Rdh10	0.44053571	0.50967127	0.015	0.013	1
Rgs18	0.44054362	0.28220649	0.01	0.012	1
Anks3	0.4407575	0.42377382	0.041	0.038	1
Mbd1	0.44080265	0.56328203	0.073	0.07	1
Stk25	0.4422877	0.37778928	0.045	0.042	1
Slc6a8	0.4437358	0.79807382	0.012	0.01	1
Cand1	0.44904475	0.51450782	0.113	0.112	1
Nipsnap1	0.45014363	0.77040308	0.02	0.018	1
Nbeal2	0.4501872	0.29272946	0.025	0.028	1
Gfm2	0.45100406	0.42803449	0.039	0.036	1
Acvr1	0.45118203	0.29277011	0.009	0.01	1
Zfp182	0.45146281	0.35941985	0.025	0.022	1
1810032O08R	0.45234031	0.30634935	0.053	0.058	1
Klhl7	0.45375119	0.39022989	0.024	0.027	1
Heatr5b	0.45583389	0.36417597	0.029	0.026	1
Akr1e1	0.45694863	0.53538693	0.01	0.012	1
Haghl	0.45694971	0.32362925	0.018	0.021	1
Col6a1	0.4575738	0.2981479	0.025	0.023	1
Slc9a6	0.45895786	0.51566042	0.017	0.015	1

Snhg9	0.45938096	0.46940093	0.065	0.062	1
BC002059	0.45984851	0.35556925	0.013	0.015	1
Tbc1d32	0.46108204	0.34848349	0.009	0.01	1
Ttbk2	0.46188194	0.35495019	0.022	0.02	1
Zfp386	0.46225971	0.5049173	0.035	0.039	1
Ss18l1	0.46348637	0.40611858	0.011	0.009	1
Mllt1	0.46399851	0.39824514	0.019	0.017	1
Wdr6	0.46505469	0.32767725	0.018	0.016	1
Lgalsl	0.46558589	0.45729229	0.018	0.016	1
Tubg1	0.46583175	0.83602269	0.008	0.01	1
Gcdh	0.4667616	0.60950156	0.015	0.013	1
Hist4h4	0.46774154	0.27069504	0.02	0.018	1
Znhit6	0.47071469	0.30716829	0.043	0.041	1
Dffa	0.47071679	0.36073867	0.021	0.019	1
Rnf11	0.47094155	0.29325213	0.111	0.11	1
Rnf215	0.4720977	0.30120092	0.032	0.036	1
Scrib	0.47426031	0.4933417	0.037	0.034	1
Mettl3	0.47466053	0.32873474	0.039	0.037	1
Zfp429	0.47481735	0.42801448	0.009	0.01	1
Ago3	0.47509481	0.31364211	0.065	0.072	1
Taf5	0.47523421	0.28568077	0.04	0.038	1
Entpd6	0.47718884	0.28049125	0.045	0.042	1
Rab23	0.47723385	0.6303599	0.011	0.01	1
Slc25a22	0.4773721	0.3113911	0.017	0.015	1
Gm26782	0.47760521	0.52057063	0.018	0.016	1
Gin1	0.47898155	0.74187436	0.02	0.018	1
Zscan26	0.48241504	0.41758598	0.045	0.043	1
Ehmt1	0.48253781	0.26617621	0.052	0.057	1
Dcaf17	0.48350371	0.43222649	0.036	0.033	1
Acads	0.48423117	0.47052751	0.047	0.044	1
Hira	0.48539094	0.47911981	0.034	0.031	1
Bad	0.48573667	0.52213186	0.038	0.042	1
Zdhhc13	0.48603666	0.47793496	0.031	0.029	1
Pias2	0.48658886	0.45863644	0.052	0.049	1
Dcaf4	0.48677682	0.53205581	0.017	0.015	1
Taf1b	0.48690287	0.31185833	0.035	0.038	1
Tmco3	0.48745556	0.28022936	0.024	0.026	1
Slc25a24	0.48854432	0.48505434	0.069	0.067	1
1110008L16Ri	0.49015778	0.64679964	0.008	0.01	1
Ppp1r13b	0.49109632	0.27275151	0.048	0.045	1
Ppp5c	0.49309149	0.5305651	0.045	0.043	1
Actr1b	0.49361734	0.35497164	0.078	0.085	1
Pigk	0.49367154	0.32536066	0.056	0.061	1
Rgp1	0.49397962	0.45872699	0.021	0.019	1
Tmem245	0.49429019	0.30187111	0.072	0.069	1
Spon2	0.49557408	0.36154975	0.017	0.015	1
Gm31597	0.49602549	0.46971186	0.019	0.022	1
Npepl1	0.49681242	0.55995786	0.068	0.066	1

Zmym4	0.49930343	0.44554974	0.053	0.051	1
Araf	0.50009486	0.37759649	0.118	0.117	1
Mex3b	0.50163553	0.29141524	0.024	0.026	1
Mterf1a	0.50172103	0.30414313	0.039	0.037	1
Hba-a2	0.50177402	0.43248105	0.018	0.02	1
Rad54l2	0.50254222	0.2869728	0.07	0.067	1
Mfn1	0.50287396	0.30383415	0.029	0.032	1
Letmd1	0.50344925	0.26697005	0.026	0.028	1
Ak3	0.50418254	0.37191263	0.032	0.035	1
Endov	0.5041907	0.58718688	0.014	0.012	1
Man2c1	0.50437514	0.36085236	0.02	0.023	1
Slc2a9	0.50464202	0.46590718	0.012	0.01	1
Tbc1d22b	0.50818488	0.45550672	0.022	0.02	1
Fancm	0.50952008	0.49416383	0.023	0.021	1
Traf7	0.51007204	0.42042738	0.069	0.067	1
Osbpl9	0.51062726	0.3564537	0.217	0.225	1
Micall1	0.51226114	0.31970573	0.037	0.041	1
Eps15l1	0.51240706	0.29707141	0.063	0.069	1
Gm32036	0.51278413	0.40510848	0.018	0.016	1
Polr2g	0.51500711	0.40885202	0.123	0.124	1
Fhl2	0.51541463	0.51028426	0.017	0.015	1
Ipo9	0.5165683	0.33314481	0.032	0.029	1
Asb1	0.51695628	0.38544479	0.016	0.015	1
Umad1	0.51716529	0.52342615	0.016	0.018	1
Snrnp25	0.52046916	0.44293626	0.061	0.059	1
Ulk4	0.52156648	0.28092583	0.032	0.029	1
Pcsk7	0.52180755	0.33253318	0.068	0.066	1
Sppl3	0.5228646	0.38573486	0.088	0.086	1
Uxs1	0.52293854	0.25148215	0.028	0.031	1
Nt5c3b	0.52341886	0.37729963	0.037	0.034	1
Rnf214	0.52504018	0.27211927	0.033	0.036	1
Thap4	0.52620967	0.58174995	0.016	0.018	1
Hic2	0.52671879	0.52980155	0.017	0.016	1
Trappc6a	0.52745967	0.57279954	0.107	0.108	1
Pi4kb	0.52759055	0.36430125	0.026	0.024	1
Arfrp1	0.52827642	0.34400969	0.12	0.119	1
Ccl11	0.52896411	0.41119124	0.014	0.013	1
Ubr3	0.52923952	0.38856337	0.091	0.09	1
Mtx3	0.52962056	0.66506249	0.012	0.01	1
Ago1	0.5336075	0.27462597	0.038	0.042	1
4932422M17R	0.53429406	0.28952178	0.02	0.019	1
Metap1d	0.53676251	0.27623832	0.025	0.028	1
3110001I22Ril	0.53688713	0.36077555	0.018	0.016	1
Shoc2	0.53876434	0.30294733	0.13	0.13	1
Armcx5	0.53889413	0.36877744	0.06	0.057	1
Them4	0.53915894	0.57086248	0.02	0.018	1
Slc39a11	0.54142044	0.34496867	0.018	0.02	1
1810044D09R	0.54234088	0.26425568	0.015	0.017	1

Rfk	0.54466931	0.28121887	0.094	0.1	1
Hist2h2bb	0.54501314	0.32187373	0.026	0.024	1
Bcl2l2	0.54520525	0.73790541	0.015	0.014	1
Tmem18	0.5474235	0.70102542	0.016	0.014	1
Mad2l1	0.54819389	0.32654284	0.024	0.022	1
Tpbg	0.54911774	0.6158952	0.01	0.008	1
2610507B11R	0.55209475	0.34224719	0.119	0.118	1
B230354K17R	0.55562256	0.65170326	0.015	0.014	1
Abcd4	0.56337852	0.6296739	0.022	0.021	1
Emc7	0.56479448	0.31438129	0.263	0.279	1
Tomm40l	0.56539404	0.62738803	0.021	0.02	1
Zfp653	0.56867656	0.4083894	0.014	0.016	1
Neurl4	0.56926235	0.43397639	0.022	0.02	1
Tusc3	0.57003005	0.41963884	0.073	0.071	1
Tmem62	0.57094047	0.43967696	0.015	0.014	1
Kars	0.57287137	0.34053711	0.09	0.098	1
E530011L22Ri	0.57595379	0.30290074	0.032	0.03	1
2410089E03R	0.57778698	0.31374307	0.057	0.055	1
Stx4a	0.57921054	0.45616505	0.154	0.16	1
Mospd3	0.57944801	0.36426684	0.044	0.048	1
Ube2o	0.58006326	0.38536858	0.045	0.043	1
BC029722	0.58102332	0.48476675	0.05	0.048	1
Arsk	0.58196549	0.66443526	0.013	0.014	1
Ints3	0.58242149	0.65604931	0.034	0.032	1
Sdccag3	0.58277637	0.52444934	0.082	0.082	1
Ralgapa2	0.58313743	0.40602871	0.045	0.043	1
Auts2	0.58651788	0.34164914	0.01	0.009	1
Naa30	0.58657878	0.32759013	0.073	0.071	1
Alg6	0.58779443	0.37768102	0.009	0.01	1
Metrn	0.58920895	0.59033098	0.031	0.029	1
Pcgf6	0.5909834	0.37217158	0.017	0.016	1
Med20	0.59338809	0.29288063	0.046	0.044	1
Tab3	0.59373313	0.30597231	0.034	0.037	1
Tsnax	0.5941929	0.43602179	0.05	0.048	1
Zc3h8	0.5942342	0.36864582	0.009	0.01	1
Asb3	0.59463086	0.39001169	0.03	0.028	1
Dynlt1a	0.5965763	0.28781764	0.009	0.01	1
Mob1b	0.59715999	0.30441079	0.114	0.123	1
Cracr2a	0.60062959	0.49748844	0.013	0.012	1
Hdac6	0.60091381	0.37677182	0.012	0.014	1
Ankrd54	0.60204768	0.31316612	0.036	0.034	1
Lcmt1	0.60350884	0.55881749	0.042	0.041	1
Zgpat	0.6039175	0.34434559	0.041	0.04	1
C330011M18F	0.60404482	0.41184768	0.011	0.01	1
Asb7	0.60564499	0.2808038	0.014	0.015	1
Sec31a	0.60659922	0.3548496	0.15	0.153	1
Mkks	0.60754337	0.54986103	0.01	0.009	1
Arl15	0.60867297	0.37572626	0.026	0.028	1

Lsm14b	0.61080253	0.27952171	0.03	0.033	1
Lrrc1	0.61245634	0.40296587	0.012	0.013	1
Gpatch2	0.61376006	0.44323096	0.051	0.049	1
Phtf1	0.61522153	0.29476824	0.043	0.041	1
Coq4	0.61553935	0.60561868	0.013	0.012	1
Zfp81	0.61809915	0.51017387	0.02	0.018	1
Fbxo25	0.61978279	0.29777928	0.013	0.015	1
Olfr60	0.6203113	0.3198052	0.018	0.02	1
Ppip5k2	0.62442622	0.30808746	0.057	0.055	1
Dusp14	0.62588554	0.27622628	0.015	0.014	1
Mfng	0.62648714	0.33926394	0.03	0.032	1
Lrrfip2	0.62670783	0.3467553	0.136	0.149	1
Srprb	0.62680017	0.36720872	0.056	0.06	1
Strn	0.62763446	0.35028025	0.062	0.061	1
1110008P14R	0.6310449	0.34096707	0.159	0.163	1
Ikbkg	0.63173506	0.3775902	0.033	0.031	1
Cuta	0.63523079	0.33950661	0.19	0.211	1
Gtf3a	0.6357102	0.31529653	0.055	0.058	1
Clec3b	0.63702751	0.40617675	0.014	0.013	1
Map9	0.64040943	0.57327911	0.01	0.009	1
Pcnx3	0.64215986	0.40312455	0.035	0.033	1
Gm42726	0.64326129	0.3342393	0.01	0.009	1
Gm43672	0.64381998	0.31425189	0.014	0.013	1
Atad5	0.64657891	0.2887121	0.027	0.029	1
Cpsf1	0.65196908	0.29704347	0.044	0.042	1
Fbxw4	0.65319053	0.43824153	0.025	0.027	1
Oaz2	0.65495126	0.30917991	0.034	0.037	1
Rxra	0.65645012	0.39869419	0.017	0.018	1
Zfp580	0.65973523	0.51196819	0.01	0.009	1
Pigv	0.65993888	0.35848629	0.017	0.016	1
Crtap	0.66011561	0.34443186	0.013	0.012	1
Nt5dc1	0.66054076	0.33909609	0.04	0.039	1
Zfp426	0.66182425	0.3155162	0.017	0.018	1
Zer1	0.6619401	0.3145993	0.047	0.045	1
Rnf14	0.66486125	0.32377202	0.063	0.067	1
Fam210b	0.66537859	0.48118029	0.029	0.028	1
Slc39a13	0.667836	0.50703579	0.014	0.016	1
Polb	0.66788765	0.49837061	0.105	0.114	1
Nt5m	0.67031331	0.46831341	0.012	0.013	1
Rexo1	0.67033864	0.31822401	0.141	0.144	1
Cds2	0.67058429	0.29574943	0.088	0.087	1
Cenpc1	0.67114004	0.25132268	0.063	0.062	1
Ppm1h	0.67158856	0.38665883	0.087	0.087	1
Whamm	0.67164286	0.27931239	0.069	0.073	1
Sirt3	0.67172465	0.60140178	0.02	0.022	1
Synrg	0.67225746	0.33402223	0.098	0.105	1
Med30	0.67255375	0.27252474	0.11	0.117	1
AW554918	0.67339105	0.33160446	0.025	0.024	1

Trpm4	0.67492999	0.47043212	0.011	0.012	1
8030462N17R	0.67713143	0.31273856	0.025	0.024	1
Gm49359	0.67721769	0.46909363	0.02	0.019	1
Zbtb14	0.6797184	0.71137334	0.011	0.01	1
Cep19	0.68012669	0.47904728	0.01	0.009	1
Btbd7	0.68088898	0.28395236	0.169	0.183	1
Vegfb	0.68134309	0.44415634	0.022	0.024	1
Wdr59	0.68136432	0.55037703	0.014	0.013	1
Mcoln1	0.68234162	0.37312637	0.025	0.026	1
Trmt5	0.68359856	0.7739484	0.01	0.009	1
Mnat1	0.68421639	0.25754416	0.072	0.076	1
Rfesd	0.68624413	0.60900057	0.019	0.018	1
Dazap1	0.68681897	0.2610999	0.158	0.17	1
Desi1	0.68942606	0.32048966	0.067	0.066	1
Rgl2	0.69157749	0.44981693	0.035	0.038	1
Pik3r2	0.69243503	0.90500695	0.012	0.011	1
9930022D16R	0.69250904	0.43426817	0.019	0.02	1
Tmem185a	0.69294149	0.41882584	0.016	0.018	1
Caap1	0.69367217	0.35012928	0.058	0.057	1
Krcc1	0.69399574	0.50544378	0.102	0.11	1
Nudcd1	0.69655164	0.25290035	0.016	0.018	1
Map3k4	0.6997362	0.32172525	0.049	0.048	1
Mms22l	0.70456241	0.62991607	0.025	0.024	1
Klhl15	0.70636983	0.27897729	0.031	0.03	1
March2	0.70777227	0.3346202	0.094	0.094	1
Cdyl	0.70806932	0.3436592	0.05	0.053	1
Srgap2	0.7082101	0.42569498	0.1	0.101	1
Ubqln4	0.7094678	0.34738333	0.031	0.03	1
Ash2l	0.71209359	0.37004528	0.063	0.062	1
Prpsap1	0.71358737	0.35883282	0.068	0.072	1
Gtf2ird2	0.71461498	0.37589939	0.011	0.01	1
Pmm2	0.71499079	0.36086991	0.074	0.074	1
Cdk10	0.71655366	0.48788717	0.019	0.018	1
Nek9	0.71827405	0.27330909	0.063	0.067	1
B3galt6	0.71862001	0.41272972	0.013	0.012	1
Fto	0.71917948	0.42958516	0.064	0.063	1
Ice2	0.72241314	0.49993935	0.016	0.015	1
Trim39	0.72245657	0.26686467	0.054	0.053	1
Sh3glb2	0.72344833	0.31819558	0.04	0.043	1
Golim4	0.72572146	0.2788918	0.056	0.059	1
Mtfr1l	0.72685439	0.46363674	0.067	0.067	1
Rpl36a-ps1	0.72711186	0.25878669	0.096	0.101	1
Evi5l	0.72761919	0.33767949	0.011	0.01	1
Klhl5	0.72821889	0.44425299	0.031	0.033	1
Banp	0.72933864	0.45351943	0.026	0.027	1
Slc25a46	0.73033924	0.3879329	0.053	0.052	1
Abcb8	0.73153439	0.34446916	0.01	0.011	1
Cep63	0.73246647	0.34742192	0.052	0.051	1

Atg4d	0.73601059	0.31795863	0.046	0.045	1
Nsd2	0.7368197	0.30453106	0.111	0.118	1
Ccdc73	0.73691439	0.79135267	0.014	0.013	1
Nudt5	0.73739302	0.31779691	0.051	0.053	1
0610009O20R	0.73786213	0.33196466	0.017	0.018	1
Plcb3	0.73880055	0.34902746	0.01	0.011	1
AI606181	0.74437754	0.27513457	0.022	0.022	1
Trio	0.74588867	0.56950913	0.082	0.083	1
Car5b	0.7474164	0.50009408	0.011	0.012	1
Cep170b	0.7477747	0.41665316	0.012	0.012	1
Smim1	0.74936902	0.353346	0.013	0.013	1
Psph	0.74974146	0.43153451	0.034	0.035	1
Hjurp	0.74999136	0.33128213	0.081	0.085	1
Zfp444	0.75018641	0.59964604	0.017	0.017	1
Lancl1	0.75216505	0.61746765	0.03	0.031	1
Pick1	0.75340652	0.49482972	0.019	0.018	1
Dhx57	0.75353019	0.35594492	0.024	0.023	1
Src	0.7550434	0.34426655	0.028	0.027	1
Phb	0.75595097	0.4403208	0.027	0.026	1
Mcph1	0.75744012	0.41275639	0.026	0.028	1
4632415L05Ri	0.75825954	0.3488402	0.013	0.013	1
Arhgap12	0.76234853	0.36024177	0.038	0.037	1
Icmt	0.76443172	0.37728822	0.031	0.032	1
Rnf6	0.76506075	0.26443431	0.16	0.171	1
Nsdhl	0.76542982	0.57663613	0.027	0.026	1
Abcc5	0.76628617	0.26060914	0.029	0.03	1
Pros1	0.76679947	0.44919499	0.015	0.016	1
Kdm1a	0.76715925	0.26629036	0.221	0.231	1
Ampd2	0.76728718	0.71938591	0.017	0.016	1
Naa60	0.7694357	0.42231815	0.046	0.046	1
Hcfc2	0.76979319	0.32694822	0.032	0.031	1
Mbp	0.77223843	0.26418396	0.15	0.154	1
Lrch3	0.77245992	0.31323287	0.089	0.09	1
Ppm1k	0.77474998	0.33556989	0.042	0.044	1
Slc37a1	0.77476841	0.29517971	0.013	0.012	1
Smim10l1	0.77528651	0.39877217	0.083	0.088	1
Abca2	0.77658144	0.30270338	0.035	0.034	1
Tacc2	0.77662019	0.59957605	0.009	0.01	1
BC024978	0.7781374	0.38215696	0.011	0.012	1
Ctla2a	0.77990981	0.26630138	0.165	0.17	1
Atat1	0.78125641	0.5171404	0.015	0.014	1
Coq2	0.7822504	0.3432746	0.049	0.048	1
Ndst1	0.78375511	0.33764315	0.04	0.039	1
Zfp931	0.78378429	0.4757473	0.018	0.018	1
Acta2	0.7839936	0.29287034	0.015	0.016	1
Wdyhv1	0.78441636	0.31569018	0.038	0.04	1
Slc35a2	0.78478577	0.34156261	0.046	0.046	1
Marveld1	0.78498095	0.49706532	0.021	0.022	1

Kifap3	0.78504233	0.64325666	0.018	0.019	1
Nomo1	0.7852087	0.31884924	0.032	0.033	1
Ppip5k1	0.78585828	0.49402275	0.013	0.014	1
Zfp994	0.7865226	0.48713091	0.025	0.024	1
Ndufaf4	0.78660162	0.33819914	0.079	0.079	1
Pstpip1	0.78918127	0.46852402	0.117	0.121	1
Ascc1	0.79003079	0.56596333	0.042	0.042	1
Tmbim4	0.79308897	0.28348705	0.167	0.182	1
Ncoa1	0.79315378	0.38263904	0.078	0.078	1
Camkk2	0.79376879	0.32160098	0.051	0.053	1
Secisbp2l	0.79491694	0.37663896	0.121	0.125	1
Eif5a2	0.7956129	0.71434903	0.013	0.013	1
Skiv2l	0.79585252	0.31330544	0.076	0.076	1
Galns	0.79623279	0.53329651	0.021	0.022	1
Pdgfra	0.79898233	0.34599296	0.01	0.01	1
Fam126b	0.79956857	0.31079171	0.039	0.038	1
Mib2	0.80003441	0.58525509	0.011	0.011	1
Ccsap	0.80296258	0.37751946	0.02	0.02	1
Gnpat	0.80781991	0.36830777	0.039	0.038	1
Tmem230	0.81071952	0.31568951	0.061	0.063	1
Prickle3	0.81212125	0.74234859	0.019	0.018	1
Lta4h	0.8121481	0.25519875	0.076	0.079	1
Exoc5	0.81262251	0.30229049	0.19	0.199	1
Nf1	0.81266368	0.51161604	0.073	0.074	1
Rint1	0.8127019	0.28620989	0.031	0.03	1
Pde6d	0.81324061	0.37498808	0.015	0.016	1
Aamdc	0.81392991	0.33943326	0.024	0.025	1
Wdcp	0.81435145	0.39143735	0.016	0.016	1
Zbtb49	0.8171742	0.31207201	0.012	0.013	1
Meaf6	0.81750442	0.48950266	0.059	0.06	1
Abhd16a	0.81806562	0.37151301	0.068	0.072	1
Mlycd	0.81899889	0.52754124	0.012	0.013	1
Arhgap35	0.81937994	0.42897787	0.011	0.011	1
Dclre1a	0.82034162	0.4463267	0.01	0.011	1
Pign	0.8219072	0.33439888	0.032	0.034	1
Selenom	0.82320009	0.55043101	0.061	0.061	1
Rab3d	0.82651263	0.75826167	0.016	0.016	1
Tspyl4	0.82786776	0.59512248	0.012	0.013	1
Rgs3	0.82985758	0.29203393	0.033	0.032	1
Gtpbp3	0.83043411	0.52089175	0.011	0.011	1
Glipr2	0.83322301	0.28285709	0.074	0.074	1
Cep152	0.83342441	0.33845808	0.014	0.014	1
Tmem159	0.83480562	0.86526254	0.016	0.015	1
Ube2v1	0.83486312	0.44446348	0.021	0.02	1
Zcwpw1	0.83655453	0.38861227	0.012	0.013	1
Rpap2	0.83671793	0.39623578	0.026	0.026	1
Hddc3	0.83849251	0.64178239	0.009	0.01	1
Cmc1	0.83961536	0.3398559	0.157	0.164	1

C1qtnf12	0.83975043	0.43317281	0.017	0.018	1
Map2k5	0.84224298	0.40415163	0.031	0.031	1
Carf	0.84226468	0.29655142	0.014	0.015	1
Zfp709	0.84228684	0.74486249	0.012	0.011	1
Ttf2	0.84344296	0.31985136	0.021	0.022	1
Sigirr	0.8444586	0.26627598	0.029	0.03	1
Hk1	0.84535677	0.34981297	0.07	0.073	1
Erich1	0.84747941	0.33002037	0.054	0.056	1
Tmem263	0.84961775	0.35151831	0.048	0.048	1
Dvl1	0.85012896	0.29940731	0.037	0.037	1
Snx11	0.85022789	0.30172442	0.029	0.03	1
Kpnb1	0.85113069	0.27799064	0.135	0.139	1
Acbd4	0.85192803	0.3474254	0.012	0.012	1
Ulbp1	0.85202318	0.37938837	0.009	0.01	1
Zdhhc17	0.85268994	0.32249805	0.045	0.045	1
Zfp995	0.85435612	0.52527151	0.011	0.01	1
Slc24a5	0.85505754	0.45073962	0.016	0.017	1
Ly6g5b	0.85636467	0.33029442	0.013	0.013	1
Tsc2	0.85727453	0.42832295	0.031	0.03	1
Apc	0.85831871	0.31069421	0.105	0.11	1
Slc50a1	0.85845103	0.26353276	0.127	0.13	1
D730003I15Ri	0.86012717	0.2583521	0.035	0.036	1
Mrs2	0.8621654	0.43067172	0.041	0.041	1
Tyw1	0.86264716	0.51824183	0.019	0.02	1
Ugp2	0.86385701	0.26344985	0.126	0.13	1
Chd1l	0.86399563	0.30452078	0.028	0.027	1
Pogz	0.86401539	0.30476613	0.046	0.046	1
Cyhr1	0.86422452	0.39065078	0.071	0.073	1
Mppe1	0.86691581	0.39124979	0.013	0.013	1
Exoc2	0.86911039	0.27339694	0.075	0.077	1
Nudt22	0.87111833	0.52038187	0.012	0.012	1
Accs	0.87397738	0.64504927	0.015	0.014	1
Meg3	0.87432001	0.52964655	0.01	0.011	1
Slc25a12	0.87670871	0.31235862	0.033	0.034	1
Ap1s3	0.87700828	0.36206389	0.053	0.054	1
Llgl1	0.87705229	0.45682029	0.022	0.022	1
Vezt	0.87722493	0.55562155	0.018	0.018	1
Hspbp1	0.8787469	0.37774838	0.054	0.054	1
Oxsr1	0.88114956	0.3657651	0.059	0.061	1
Ctps2	0.88153757	0.32392941	0.044	0.044	1
Klhl36	0.88541854	0.4094694	0.011	0.012	1
Plekhg3	0.88702475	0.51208542	0.049	0.051	1
Cfap36	0.88709077	0.34940049	0.086	0.089	1
Ccdc163	0.88863346	0.34126672	0.016	0.016	1
Cfdp1	0.89019097	0.40918038	0.134	0.143	1
Phpt1	0.89560462	0.53701067	0.035	0.035	1
Zranb2	0.89648948	0.35382865	0.152	0.159	1
Ogfod1	0.89664537	0.46503099	0.018	0.018	1

Ogn	0.89844958	0.4577099	0.014	0.014	1
Dido1	0.90111526	0.26061577	0.062	0.063	1
Pctp	0.90317053	0.47222627	0.01	0.01	1
Samd1	0.90388246	0.46086845	0.025	0.024	1
Zcchc4	0.90509786	0.29830875	0.028	0.027	1
Tubgcp4	0.90511743	0.30827492	0.063	0.063	1
Arrdc2	0.90783011	0.46225357	0.012	0.012	1
Rtn4ip1	0.90901318	0.36572212	0.012	0.012	1
Dynlrb1	0.90908118	0.29884351	0.255	0.279	1
Habp4	0.90916419	0.37006657	0.013	0.013	1
Men1	0.90979136	0.27579601	0.013	0.013	1
Enc1	0.91356016	0.64434582	0.018	0.019	1
Arhgap39	0.91430525	0.54363724	0.022	0.023	1
Rnf38	0.91703921	0.35251465	0.073	0.075	1
Spout1	0.92231077	0.40320143	0.019	0.019	1
Tmem158	0.92314	0.27743875	0.011	0.011	1
Tmem35b	0.92501043	0.80515878	0.01	0.01	1
Phkb	0.9262813	0.4559621	0.032	0.032	1
Capn10	0.9282867	0.27067527	0.013	0.014	1
Pcmt1	0.93205829	0.33386853	0.16	0.17	1
Pik3cg	0.93380868	0.45062934	0.069	0.071	1
Zfp287	0.9350932	0.33342966	0.011	0.011	1
Sec61a1	0.93518105	0.2832669	0.213	0.229	1
Polk	0.93673207	0.32049934	0.027	0.028	1
Anapc4	0.93811947	0.37074247	0.042	0.043	1
Ankra2	0.93830948	0.36858894	0.065	0.067	1
Tmed4	0.93866344	0.39587281	0.038	0.039	1
Sdhaf3	0.94016286	0.51131251	0.014	0.015	1
Gstk1	0.94151492	0.84234065	0.011	0.011	1
Dhrs11	0.94171465	0.37839542	0.01	0.01	1
Ago4	0.94458168	0.71819513	0.015	0.015	1
Ralgps1	0.94752857	0.36651282	0.01	0.01	1
Tmem63b	0.94822345	0.48324937	0.046	0.047	1
Gm12216	0.94893541	0.39491691	0.024	0.024	1
Shprh	0.95071164	0.34959168	0.04	0.041	1
Arid2	0.95111974	0.39592446	0.054	0.056	1
Taf1a	0.95269552	0.36526607	0.015	0.016	1
BC037034	0.95278303	0.33243255	0.027	0.027	1
6230400D17R	0.9546662	0.32555089	0.012	0.012	1
Gtf2h2	0.95540475	0.37201735	0.022	0.022	1
Ccdc90b	0.95632022	0.38482776	0.02	0.02	1
Chml	0.95967883	0.36734093	0.013	0.013	1
Brwd3	0.96033716	0.35453743	0.045	0.046	1
Slc16a1	0.961093	0.27709303	0.051	0.052	1
Bahd1	0.96124479	0.30558486	0.043	0.043	1
Kif1bp	0.96189974	0.35131905	0.027	0.027	1
Endod1	0.96323783	0.62418108	0.051	0.052	1
Psmf1	0.96480208	0.4034884	0.056	0.058	1

Tprkb	0.96602745	0.27662206	0.034	0.035	1
Gmcl1	0.96622257	0.30274592	0.043	0.044	1
Rmi2	0.96972031	0.40240283	0.011	0.011	1
Tubgcp3	0.97063664	0.30684718	0.063	0.064	1
Ring1	0.97088453	0.4266135	0.045	0.046	1
Fastk	0.97206078	0.25648937	0.034	0.034	1
Dctn6	0.97220107	0.34463655	0.069	0.071	1
Mpv17	0.97249419	0.62435374	0.018	0.018	1
Nup155	0.97584589	0.27121157	0.03	0.03	1
Gas2	0.97862491	0.3175824	0.01	0.01	1
Pja2	0.9789372	0.39851449	0.044	0.045	1
Gm30025	0.98047588	0.34407265	0.012	0.012	1
Zfp276	0.98065067	0.32168235	0.013	0.013	1
Rnf187	0.98142602	0.32632528	0.133	0.139	1
Hltf	0.98271225	0.3105585	0.092	0.095	1
Ddx55	0.98274517	0.26213349	0.042	0.042	1
Vps52	0.98275219	0.33513114	0.04	0.04	1
Zfp825	0.98606631	0.32990573	0.011	0.011	1
Nsun5	0.98650051	0.42786857	0.016	0.016	1
Atp5s	0.99102812	0.29033819	0.01	0.01	1
Elac1	0.99211177	0.3056162	0.014	0.014	1
Taf12	0.99351459	0.38324829	0.059	0.06	1
Gm13212	0.99401379	0.43464142	0.036	0.036	1
Fuz	0.99543752	0.64051839	0.016	0.016	1
Zranb1	0.99609015	0.26702308	0.179	0.188	1
Man1b1	0.9965269	0.4001681	0.066	0.067	1
Fbxo18	0.99674579	0.37931367	0.023	0.023	1
Hdac2	0.99731491	0.33361177	0.063	0.065	1
Cep44	0.99778374	0.52100201	0.059	0.06	1
Rab28	0.99833447	0.31473744	0.041	0.041	1
AC165953.2	0.99947294	0.28719659	0.01	0.01	1
Leng9	0.99959067	0.27211859	0.031	0.031	1
Bcl2l12	0.99990349	0.63231718	0.018	0.018	1

CD4 Th17

Gene	p_val	avg_log2FC	pct.1	pct.2	p_val_adj
Cxcr6	0	2.61710001	0.908	0.205	0
Icos	0	1.99798973	0.946	0.307	0
Cd3g	0	2.00384815	0.887	0.268	0
Ctla4	0	1.96709859	0.857	0.244	0
Tnfrsf4	0	2.37498289	0.717	0.126	0
Rora	0	1.29056121	0.978	0.399	0
Actn2	0	3.25149825	0.657	0.09	0
Ermn	0	5.10710655	0.593	0.027	0
Cd3d	0	1.58584504	0.829	0.274	0
Trbc2	0	1.60209844	0.888	0.345	0
Furin	0	1.80013234	0.905	0.383	0
Lat	0	2.0929253	0.702	0.19	0
Sh2d2a	0	1.60014322	0.772	0.261	0
Ramp3	0	1.86291589	0.855	0.345	0
Nmrk1	0	3.24099293	0.581	0.085	0
Il18r1	0	1.95667192	0.662	0.168	0
Cd3e	0	1.40063108	0.746	0.255	0
Ppp1cc	0	1.93040852	0.847	0.358	0
Dgat1	0	1.51191063	0.91	0.422	0
Ostf1	0	2.38041256	0.964	0.489	0
Cd4	0	2.27793226	0.591	0.12	0
Hcst	0	1.38124428	0.892	0.435	0
Cd2	0	1.55743363	0.703	0.254	0
Ltb	0	1.80547882	0.68	0.241	0
D16Ertd472e	0	1.49966264	0.78	0.344	0
Trac	0	1.58942162	0.634	0.199	0
Ptpn22	0	1.03757802	0.765	0.35	0
S100a4	0	1.143195	0.742	0.327	0
Thy1	0	1.48853842	0.623	0.215	0
Spn	0	1.78036938	0.588	0.18	0
Cd40lg	0	2.70616586	0.471	0.064	0
AW112010	0	1.23920681	0.84	0.434	0
Tnfrsf18	0	1.44575075	0.623	0.226	0
Orai1	0	1.54241571	0.696	0.299	0
Cd6	0	1.96354828	0.516	0.12	0
Emb	0	1.19646341	0.985	0.591	0
Cd5	0	2.18806013	0.501	0.107	0
Slamf1	0	2.14301882	0.51	0.117	0
Aebp2	0	1.15193922	0.889	0.496	0
Ly6a	0	1.59903239	0.681	0.289	0
Il2rb	0	0.77370684	0.658	0.268	0
Sept1	0	1.38535713	0.668	0.279	0
Hif1a	0	1.39040872	0.926	0.542	0
Cd28	0	0.901364	0.662	0.282	0
Mier1	0	1.54508267	0.758	0.378	0
Ern1	0	1.55000632	0.606	0.227	0

Ifngr1	0	1.08846785	0.97	0.592	0
Lck	0	1.60650814	0.544	0.172	0
Lgals1	0	1.23879341	0.854	0.485	0
Satb1	0	0.57524963	0.87	0.512	0
Fyb	0	1.09287747	0.84	0.483	0
Tesc	0	2.37696806	0.442	0.086	0
Odc1	0	1.17852174	0.95	0.594	0
Gmfg	0	1.3598597	0.868	0.514	0
Kdm2b	0	0.79013119	0.682	0.331	0
Itk	0	0.8001784	0.643	0.292	0
Bhlhe40	0	0.85261436	0.926	0.576	0
Zeb1	0	1.20426082	0.676	0.326	0
Mbd2	0	1.35884134	0.696	0.347	0
Ets1	0	0.8528266	0.987	0.64	0
Sipa1l1	0	1.39135156	0.631	0.291	0
Nr3c1	0	0.78695485	0.747	0.411	0
Got1	0	1.23145028	0.763	0.433	0
Stat5b	0	1.18090827	0.587	0.257	0
Clint1	0	1.20314345	0.784	0.457	0
Cdkn1b	0	1.07674166	0.698	0.374	0
Serpinb1a	0	2.23878821	0.442	0.12	0
Stk4	0	1.1277568	0.694	0.373	0
Smap2	0	1.34878056	0.641	0.327	0
Arap2	0	0.92164433	0.695	0.381	0
Ppp1r16b	0	0.8787459	0.607	0.297	0
Ece1	0	2.2349869	0.493	0.183	0
Ndfip1	0	1.36188977	0.569	0.26	0
Carnmt1	0	2.08377561	0.426	0.118	0
Rwdd1	0	1.49657341	0.599	0.292	0
Camk4	0	1.76176338	0.424	0.118	0
Lmnb1	0	0.7682	0.725	0.419	0
Sri	0	1.29805362	0.656	0.357	0
Ybx3	0	1.50639682	0.57	0.271	0
Stat4	0	0.98030626	0.658	0.361	0
Myl12b	0	1.21241667	0.824	0.527	0
Zdhhc18	0	0.9414596	0.637	0.341	0
S100a10	0	0.81606679	0.831	0.535	0
Rnf125	0	1.23068468	0.478	0.183	0
Tnfsf8	0	1.47824642	0.401	0.107	0
Ccr2	0	1.05305053	0.523	0.23	0
Grap2	0	1.49532756	0.452	0.159	0
Vgll4	0	0.99624915	0.59	0.297	0
Prr13	0	1.10932689	0.695	0.402	0
Mxi1	0	1.2605798	0.571	0.278	0
Id2	0	0.62330612	0.869	0.579	0
Ldha	0	1.1597903	0.758	0.469	0
Nebl	0	2.81028211	0.339	0.052	0
Tpi1	0	1.43480458	0.517	0.23	0

S100a13	0	1.13062706	0.656	0.372	0
Sdf4	0	1.10718415	0.629	0.347	0
Skap1	0	1.08217962	0.487	0.205	0
Myl12a	0	1.13201392	0.822	0.542	0
Rinl	0	1.52265569	0.426	0.148	0
Pde4d	0	1.48258494	0.416	0.144	0
Spcs2	0	1.12327576	0.683	0.413	0
Higd1a	0	1.38314821	0.559	0.289	0
AU020206	0	1.08013286	0.516	0.246	0
Tnfsf11	0	1.43999104	0.358	0.09	0
Cd164	0	1.36307906	0.658	0.391	0
Cd47	0	0.95125393	0.793	0.526	0
Sumo2	0	0.96353137	0.781	0.516	0
Gpx4	0	1.02433303	0.779	0.516	0
Eif3h	0	1.01189229	0.845	0.583	0
Hmgb2	0	1.20936286	0.962	0.701	0
Il18rap	0	1.27815029	0.41	0.151	0
Atxn1	0	1.32769832	0.434	0.175	0
Paxbp1	0	1.46099542	0.466	0.207	0
Anp32a	0	1.23286123	0.639	0.382	0
Prelid1	0	1.3112318	0.537	0.283	0
Ubl5	0	1.12851032	0.927	0.674	0
Elob	0	0.87654615	0.812	0.56	0
Mif	0	0.99234191	0.711	0.461	0
Themis	0	1.88603325	0.33	0.082	0
Cyb5a	0	1.59628209	0.435	0.188	0
Trat1	0	2.06682266	0.31	0.067	0
Gm19585	0	1.3165756	0.367	0.124	0
Tshz1	0	1.64319049	0.356	0.114	0
Plcxd2	0	1.19623619	0.392	0.151	0
H2afz	0	0.76732719	0.948	0.707	0
Akap13	0	1.03582472	0.975	0.736	0
Fdx1	0	2.17790782	0.338	0.101	0
Gapdh	0	0.99351491	0.963	0.727	0
Cytip	0	0.84097858	0.956	0.723	0
Itpr1	0	1.3084666	0.412	0.179	0
Usmg5	0	0.8090367	0.837	0.607	0
Runx2	0	1.45625443	0.378	0.149	0
Lamc1	0	1.78289302	0.303	0.082	0
Itm2b	0	0.56128239	0.926	0.707	0
Zap70	0	1.33572626	0.334	0.12	0
Cd247	0	1.33237517	0.322	0.114	0
Arpc2	0	0.79466294	0.935	0.728	0
Kcnmb4	0	3.19536217	0.231	0.025	0
Il17a	0	2.42098049	0.238	0.035	0
Smco4	0	1.51200486	0.296	0.095	0
Ttc39c	0	2.24437644	0.257	0.059	0
AI467606	0	1.89069105	0.253	0.067	0

Arpc3	0	0.78823802	0.943	0.759	0
Sytl2	0	1.84768979	0.233	0.059	0
Pkp3	0	1.86592946	0.247	0.073	0
Myl6	0	0.67583692	0.945	0.774	0
Prelid2	0	2.53344735	0.212	0.045	0
Ubash3a	0	2.37052603	0.202	0.037	0
Oaz1	0	0.7975319	0.979	0.82	0
Ubb	0	0.68931385	0.968	0.809	0
Slc22a15	0	2.12625667	0.203	0.046	0
Sub1	0	0.6719592	0.958	0.802	0
Tnfrsf25	0	2.20199825	0.177	0.034	0
Il1r1	0	2.4521414	0.163	0.033	0
Tmsb10	0	0.76981089	0.996	0.884	0
Sgip1	0	3.00703221	0.122	0.015	0
Pde5a	0	2.54902541	0.126	0.022	0
1700012B07R	0	3.61301204	0.112	0.009	0
Tff1	0	4.35385417	0.104	0.006	0
Il17re	0	3.61010863	0.103	0.008	0
Vax2	0	3.79435523	0.098	0.007	0
Ppia	0	0.57720249	0.996	0.909	0
H2-K1	0	0.52399789	0.993	0.907	0
H2-D1	0	0.52537189	0.993	0.908	0
Kcnmb4os2	0	3.36943791	0.091	0.009	0
Srgn	0	0.34041247	0.999	0.928	0
Rplp0	0	0.60754267	0.997	0.93	0
Rpl11	0	0.43919647	0.997	0.942	0
Rpl24	0	0.59613019	0.997	0.945	0
Rpl21	0	0.41123472	0.999	0.951	0
Rps15a	0	0.59390476	1	0.954	0
Rps11	0	0.5051302	0.999	0.956	0
Rpl18	0	0.44942366	1	0.957	0
Rps13	0	0.37706383	1	0.965	0
Rpl34	0	0.41658203	1	0.968	0
Rpl19	0	0.43483748	1	0.968	0
Birc2	6.851E-307	1.04445785	0.576	0.311	1.503E-302
Gimap7	6.761E-306	1.13280699	0.403	0.163	1.483E-301
Acsbg1	6.959E-306	2.17741877	0.148	0.03	1.527E-301
Gna15	7.482E-306	1.7908503	0.227	0.066	1.642E-301
Madd	2.666E-300	1.21345318	0.457	0.217	5.85E-296
Eno1	2.757E-300	1.3360556	0.446	0.216	6.049E-296
Rac2	3.12E-300	0.75734128	0.791	0.562	6.845E-296
Arl6ip1	3.546E-299	0.86875781	0.748	0.511	7.78E-295
Sh3bgrl3	2.261E-295	0.67249145	0.894	0.696	4.961E-291
Ubald2	4.525E-295	0.67503433	0.806	0.54	9.927E-291
Ndufv3	3.287E-294	1.06329946	0.591	0.348	7.211E-290
Abcb1b	8.817E-294	1.41196439	0.345	0.138	1.934E-289
Uqcrh	1.6E-293	0.52633649	0.967	0.852	3.51E-289
Gm12840	5.11E-292	0.92781232	0.458	0.209	1.121E-287

Rack1	5.664E-292	0.41930481	0.996	0.932	1.243E-287
Bik	6.631E-290	2.82497213	0.095	0.013	1.455E-285
Maf	9.319E-288	0.36286305	0.615	0.305	2.044E-283
Rgs1	6.283E-287	0.62048011	0.84	0.543	1.379E-282
Aldoa	1.477E-285	0.91802096	0.679	0.442	3.241E-281
Ier5l	1.295E-284	1.33619585	0.328	0.125	2.84E-280
Limd2	6.478E-284	1.01659498	0.556	0.299	1.421E-279
Pecam1	1.839E-283	1.39052346	0.325	0.126	4.035E-279
Grb7	1.997E-283	2.48660179	0.117	0.021	4.381E-279
Cmip	9.147E-283	1.22445879	0.493	0.263	2.007E-278
Atp5h	2.138E-281	0.64327425	0.902	0.727	4.691E-277
BC031181	4.211E-281	1.18384209	0.486	0.256	9.238E-277
Hlf	7.524E-279	0.71525662	0.283	0.095	1.651E-274
Gng2	5.482E-278	1.10427773	0.532	0.297	1.203E-273
Tpt1	8.75E-277	0.25422092	1	0.995	1.92E-272
Prdx6	1.852E-272	0.76016415	0.749	0.496	4.063E-268
Cd82	4.1E-270	0.87925659	0.597	0.345	8.994E-266
Prkar1a	1.14E-269	0.7244725	0.8	0.581	2.5E-265
Ctsw	4.352E-269	1.13306865	0.335	0.13	9.548E-265
Srp14	2.296E-268	0.8888626	0.657	0.421	5.038E-264
Gimap4	1.741E-267	0.88177623	0.533	0.264	3.819E-263
Atp2b4	1.464E-265	1.16415639	0.398	0.18	3.211E-261
Rpl15	1.245E-263	0.42145424	0.993	0.91	2.732E-259
Sec61g	1.047E-260	0.67456347	0.859	0.666	2.297E-256
Cd52	2.419E-260	0.52142978	0.959	0.807	5.308E-256
Eif3f	3.374E-260	0.63825611	0.864	0.676	7.401E-256
Rpl18a	8.899E-259	0.33083312	1	0.968	1.952E-254
Bcl2a1b	1.593E-258	0.4122747	0.749	0.445	3.496E-254
Leprotl1	1.483E-255	0.93600085	0.536	0.299	3.253E-251
Il2rg	3.656E-253	0.78387797	0.7	0.457	8.021E-249
Olfr60	3.703E-253	2.47941365	0.09	0.014	8.123E-249
Rps7	5.124E-253	0.35839059	0.998	0.943	1.124E-248
Hells	9.611E-253	1.78659211	0.187	0.054	2.109E-248
Thap12	1.381E-252	1.72231496	0.278	0.107	3.031E-248
Gimap1	4.652E-252	0.84336768	0.518	0.258	1.021E-247
Rab8b	5.465E-249	0.61926001	0.816	0.568	1.199E-244
Fryl	1.064E-248	1.06951573	0.43	0.206	2.335E-244
Selenos	1.692E-248	0.90899846	0.631	0.403	3.711E-244
Ms4a4b	1.926E-248	0.7455952	0.485	0.231	4.225E-244
Map1lc3b	2.302E-247	0.66270567	0.79	0.569	5.049E-243
Tprgl	1.206E-244	1.15033915	0.412	0.2	2.645E-240
Krtcap2	7.413E-243	0.92770004	0.609	0.386	1.626E-238
Gabarapl2	1.427E-242	0.98478669	0.558	0.336	3.13E-238
Gstp3	8.206E-242	1.77792372	0.164	0.044	1.8E-237
Ntrk3	1.658E-241	2.49386584	0.096	0.016	3.636E-237
Cox7c	1.862E-241	0.52906189	0.946	0.813	4.084E-237
Crot	4.104E-240	1.14796046	0.296	0.115	9.003E-236
Fau	1.549E-239	0.25382769	1	0.994	3.398E-235

Epb41	2.019E-239	0.96558464	0.551	0.322	4.428E-235
Gnpda2	1.926E-238	1.93607445	0.178	0.052	4.225E-234
Ap3s1	5.061E-237	1.2249685	0.396	0.197	1.11E-232
Manf	5.426E-236	0.85363711	0.678	0.446	1.19E-231
Mkrn1	6.944E-234	0.85448855	0.622	0.396	1.523E-229
Il1r2	8.632E-233	0.57484846	0.449	0.222	1.894E-228
Psmb1	1.006E-232	0.74761679	0.739	0.527	2.207E-228
Aes	2.072E-232	0.86279666	0.613	0.39	4.547E-228
Ncor1	1.837E-231	0.72224895	0.72	0.488	4.029E-227
Arhgap31	1.253E-229	1.01519723	0.424	0.211	2.75E-225
Cd96	1.661E-229	1.4738652	0.198	0.062	3.643E-225
Tex2	2.876E-229	1.03271452	0.385	0.179	6.31E-225
Bcl2a1d	1.095E-228	0.75946032	0.558	0.317	2.402E-224
Cd53	4.681E-228	0.58772892	0.871	0.696	1.027E-223
Gtf2i	5.476E-228	1.21132443	0.326	0.139	1.201E-223
Vcpkmt	1.437E-227	1.6458746	0.194	0.061	3.153E-223
Diaph1	5.921E-227	0.77178954	0.671	0.443	1.299E-222
Rnf19a	4.519E-225	1.05434521	0.339	0.148	9.915E-221
Naca	9.355E-225	0.48070865	0.95	0.822	2.052E-220
Btf3	2.859E-224	0.4966992	0.939	0.802	6.273E-220
Smarce1	8.665E-224	1.12685318	0.458	0.255	1.901E-219
Rbpj	7.093E-222	0.83774734	0.641	0.422	1.556E-217
Txn1	7.701E-222	0.54716178	0.778	0.539	1.69E-217
Hopx	9.43E-222	0.77043422	0.444	0.222	2.069E-217
Gabpb1	1.007E-220	1.13874802	0.344	0.155	2.21E-216
Bnip3l	1.971E-220	0.91536396	0.518	0.299	4.324E-216
Ikzf3	4.966E-219	0.71634348	0.53	0.278	1.089E-214
Gramd3	2.429E-218	0.71823465	0.491	0.249	5.329E-214
Stk26	4.428E-218	1.27835924	0.237	0.085	9.714E-214
Dnajb6	6.778E-216	0.68559362	0.766	0.563	1.487E-211
Fasl	2.322E-215	1.12102517	0.248	0.091	5.094E-211
Rps14	4.908E-214	0.26955806	1	0.974	1.077E-209
Ppib	4.964E-214	0.73680726	0.721	0.51	1.089E-209
Ddit4	1.277E-213	1.15988558	0.363	0.173	2.801E-209
Ctla2a	2.842E-213	0.88944204	0.349	0.154	6.235E-209
Mif4gd	8.166E-213	1.04021917	0.401	0.202	1.792E-208
Crem	1.378E-212	0.51694486	0.815	0.577	3.024E-208
Rbm3	8.296E-212	0.51245401	0.924	0.778	1.82E-207
Atp5d	1.728E-211	0.79625084	0.675	0.474	3.791E-207
Fancf	6.081E-211	2.14977974	0.112	0.025	1.334E-206
Pnrc1	1.643E-210	0.50852267	0.899	0.725	3.605E-206
D8Ertd738e	9.489E-210	0.70725743	0.729	0.528	2.082E-205
Mien1	2.417E-209	1.07959521	0.439	0.243	5.302E-205
Gch1	1.199E-208	1.00030797	0.458	0.254	2.63E-204
Tecpr1	2.443E-208	0.8713097	0.422	0.207	5.359E-204
Supt4a	3.202E-208	0.75994742	0.651	0.438	7.025E-204
Plcb4	3.883E-207	1.86554844	0.135	0.035	8.519E-203
Ypel3	6.28E-205	0.98981712	0.411	0.211	1.378E-200

Sh2d1a	3.877E-203	1.2173677	0.205	0.069	8.506E-199
Nfatc2	2.845E-202	1.65451541	0.193	0.065	6.242E-198
H2afy	3.402E-202	0.71313227	0.708	0.496	7.463E-198
Ly6g5b	5.034E-202	2.84212618	0.064	0.009	1.104E-197
Lysmd2	9.131E-201	1.95816067	0.117	0.028	2.003E-196
Ube2b	1.09E-198	0.60872259	0.758	0.559	2.392E-194
Slc25a3	2.299E-198	0.55275818	0.909	0.765	5.044E-194
Mat2b	2.861E-198	0.89600687	0.437	0.232	6.277E-194
Fam102a	3.777E-197	1.1601376	0.259	0.103	8.285E-193
Cox14	8.084E-197	0.89703915	0.555	0.358	1.774E-192
Rgs10	1.275E-196	1.00421884	0.481	0.284	2.798E-192
Trbc1	8.299E-196	0.85866321	0.464	0.247	1.821E-191
Tsc22d4	1.687E-195	0.92682889	0.469	0.264	3.702E-191
Cnbp	3.947E-194	0.55671255	0.845	0.662	8.66E-190
Pgam1	1.896E-193	1.09789544	0.417	0.233	4.159E-189
Mrpl33	3.501E-193	0.72500321	0.63	0.423	7.68E-189
Riok1	5.908E-193	0.79381055	0.517	0.299	1.296E-188
Prrc2c	5.798E-192	0.52618701	0.859	0.692	1.272E-187
Camk2n1	5.887E-192	1.22528735	0.225	0.084	1.292E-187
Fnbp1	3.106E-191	0.79919907	0.592	0.386	6.814E-187
Tmpo	1.086E-190	1.06661021	0.336	0.158	2.383E-186
Tspo	3.952E-190	0.65248143	0.669	0.447	8.671E-186
Sytl1	3.029E-188	2.25730858	0.085	0.016	6.645E-184
Ndufa13	9.42E-188	0.68753474	0.704	0.51	2.067E-183
Sla	1.561E-187	0.70219864	0.603	0.388	3.424E-183
Gm10827	4.544E-186	1.79060081	0.137	0.039	9.969E-182
Pfdn5	3.48E-185	0.47642823	0.905	0.762	7.635E-181
Reep5	1.583E-183	0.80585725	0.579	0.391	3.472E-179
Atp10a	2.029E-183	1.57828203	0.161	0.051	4.451E-179
Gimap3	3.461E-183	0.58024235	0.536	0.292	7.593E-179
AC163354.1	6.303E-183	1.82883545	0.138	0.04	1.383E-178
Tox2	1.293E-182	1.62525683	0.128	0.035	2.836E-178
Rpsa	7.95E-182	0.30404102	1	0.963	1.744E-177
Serf2	2.475E-181	0.40915736	0.958	0.84	5.43E-177
Fam110a	2.564E-181	1.06914957	0.289	0.129	5.625E-177
Klk8	1.782E-180	1.18903806	0.228	0.088	3.909E-176
Bcl11b	3.397E-180	0.75292482	0.338	0.154	7.452E-176
Ube2i	5.204E-179	0.7116246	0.634	0.445	1.142E-174
Sh3kbp1	8.846E-179	0.86021185	0.483	0.284	1.941E-174
Pon2	1.206E-178	1.23515893	0.292	0.135	2.646E-174
Cox6c	2.165E-178	0.51731731	0.864	0.693	4.75E-174
St6galnac3	2.303E-178	1.41495079	0.163	0.052	5.052E-174
Xlr4b	4.554E-178	1.53342334	0.164	0.054	9.991E-174
Crbn	9.731E-178	0.94687004	0.332	0.158	2.135E-173
Stat3	4.884E-177	0.5901957	0.765	0.596	1.071E-172
Rab2a	2.426E-176	0.74348004	0.611	0.422	5.323E-172
Krt83	4.371E-176	1.77082876	0.111	0.028	9.59E-172
Fxyd5	6.24E-176	0.46455853	0.848	0.644	1.369E-171

Prrt1	8.052E-176	1.6198837	0.136	0.04	1.767E-171
Paip2	1.205E-175	0.68942098	0.613	0.412	2.645E-171
Arf5	1.997E-175	0.68456804	0.653	0.467	4.381E-171
Inf2	7.313E-175	1.82277929	0.156	0.051	1.604E-170
Mfsd6	2.462E-173	1.03737288	0.305	0.142	5.401E-169
Osbpl3	4.429E-173	1.38418691	0.197	0.073	9.717E-169
Cenpa	6.529E-173	0.83408732	0.577	0.379	1.432E-168
Fam96b	1.177E-171	1.10979867	0.364	0.197	2.583E-167
Rpl9	3.09E-171	0.27209513	0.999	0.957	6.779E-167
Hmgb1	1.305E-170	0.51057747	0.819	0.651	2.863E-166
Isy1	3.271E-169	0.63677655	0.614	0.408	7.175E-165
Man2a2	3.549E-168	1.2397452	0.245	0.104	7.787E-164
1110008P14R	2.013E-167	1.12832543	0.306	0.15	4.417E-163
Ube2s	2.914E-167	0.65283339	0.647	0.453	6.392E-163
Rpl6	8.327E-167	0.28000223	0.998	0.952	1.827E-162
H2-Q7	1.166E-166	0.42130834	0.826	0.609	2.558E-162
Ablim3	1.797E-166	3.83218738	0.031	0.002	3.943E-162
Apaf1	3.557E-166	1.14169448	0.316	0.159	7.803E-162
Tnfrsf9	1.116E-165	0.55316634	0.284	0.125	2.448E-161
Cox5a	5.75E-165	0.64585816	0.687	0.499	1.262E-160
Psmb3	6.107E-165	0.85112801	0.504	0.325	1.34E-160
Cysltr2	8.264E-165	1.37114101	0.192	0.072	1.813E-160
Gm27166	1.47E-164	3.32754581	0.036	0.003	3.226E-160
Emsy	4.49E-164	0.94730369	0.356	0.183	9.85E-160
Rpl17	4.737E-164	0.25512667	1	0.967	1.039E-159
Cblb	3.613E-163	0.725447	0.484	0.28	7.928E-159
Rpl7a	3.013E-162	0.36814681	0.975	0.895	6.61E-158
Tma7	3.051E-162	0.59703419	0.736	0.566	6.693E-158
Ckb	1.646E-161	0.9947864	0.329	0.168	3.611E-157
Rest	2.289E-161	0.98543091	0.4	0.228	5.021E-157
Lsp1	1.772E-160	0.53866138	0.678	0.449	3.887E-156
Pgk1	2.796E-160	0.78598914	0.507	0.326	6.134E-156
Cwc15	3.772E-160	0.75463047	0.562	0.381	8.275E-156
Hccs	4.975E-160	1.1676379	0.28	0.132	1.091E-155
Sec11c	1.474E-159	0.75237685	0.563	0.381	3.234E-155
Tgfbr2	2.091E-159	0.88568534	0.402	0.222	4.587E-155
Fam129a	3.82E-159	0.86086045	0.419	0.243	8.38E-155
H2afv	4.946E-159	0.74609015	0.46	0.27	1.085E-154
Eif1	8.383E-159	0.25963617	0.998	0.976	1.839E-154
Rpl32	1.825E-158	0.26766927	0.999	0.955	4.004E-154
Scrt1	1.644E-157	1.94271864	0.085	0.019	3.608E-153
Sdcbp2	2.526E-157	1.76639098	0.117	0.033	5.542E-153
Park7	9.453E-157	0.86265296	0.474	0.299	2.074E-152
Emg1	9.931E-157	0.82923892	0.47	0.291	2.179E-152
Ncald	6.963E-156	1.75272982	0.111	0.03	1.528E-151
Atp5j2	9.764E-156	0.6079936	0.701	0.523	2.142E-151
Cox7b	7.249E-155	0.60016053	0.695	0.499	1.59E-150
Arpp19	4.917E-154	0.61402127	0.652	0.467	1.079E-149

Crmp1	6.048E-152	1.8450838	0.085	0.02	1.327E-147
Lsm8	1.951E-151	0.96832997	0.322	0.165	4.28E-147
Slc25a4	3.705E-151	1.00066201	0.394	0.231	8.127E-147
Fkbp3	4.145E-151	0.82302007	0.424	0.246	9.093E-147
Lgals7	7.467E-151	1.88172875	0.082	0.019	1.638E-146
Itgal	7.611E-151	0.62342372	0.502	0.305	1.67E-146
Celf2	8.121E-150	0.54643008	0.716	0.53	1.782E-145
Polr1d	1.186E-149	0.64647131	0.65	0.476	2.601E-145
Rpl29	2.418E-149	0.42567927	0.949	0.849	5.304E-145
Cish	6.574E-149	0.76636616	0.376	0.204	1.442E-144
Kbtbd11	8.629E-147	0.96945273	0.246	0.109	1.893E-142
Slc2a3	1.109E-146	1.18862798	0.148	0.051	2.433E-142
Gypc	1.95E-146	1.66860388	0.138	0.046	4.277E-142
Gpr183	2.595E-146	0.45787559	0.651	0.434	5.693E-142
Rabac1	3.277E-146	0.66636283	0.602	0.424	7.189E-142
Gxylt1	4.801E-146	1.13920138	0.262	0.124	1.053E-141
Ltb4r1	3.981E-145	1.76879868	0.126	0.04	8.735E-141
Il7r	5.944E-145	0.63711222	0.581	0.382	1.304E-140
H2afj	1.11E-144	0.61216737	0.672	0.492	2.436E-140
Ucp2	3.043E-144	0.61057704	0.706	0.52	6.676E-140
Pfkp	3.483E-144	0.86631575	0.42	0.254	7.641E-140
Gabbr1	1.485E-143	1.40769176	0.153	0.054	3.257E-139
Arid5a	2.453E-143	0.73784992	0.436	0.258	5.382E-139
Stk24	2.928E-143	0.71554315	0.506	0.321	6.424E-139
Rps3	1.268E-142	0.25224145	0.999	0.954	2.782E-138
Ncoa2	2.589E-142	0.90557291	0.333	0.176	5.681E-138
Ostc	3.228E-142	0.88200774	0.439	0.278	7.081E-138
Ccr4	1.481E-141	1.18510528	0.137	0.045	3.249E-137
Xlr4a	8.293E-141	1.85416919	0.093	0.024	1.819E-136
Sys1	1.149E-140	0.77683728	0.483	0.312	2.521E-136
Vps28	4.409E-140	0.87391572	0.415	0.253	9.673E-136
Cir1	4.687E-140	0.79184723	0.388	0.219	1.028E-135
4833420G17R	9.803E-140	0.92753633	0.302	0.153	2.151E-135
Notch2	1.122E-139	1.12859783	0.298	0.157	2.461E-135
Dut	2.106E-139	1.12509943	0.226	0.1	4.62E-135
Inpp1	7.444E-139	1.23667003	0.18	0.071	1.633E-134
Ifng	1.161E-138	1.03543934	0.14	0.047	2.546E-134
Cirbp	2.091E-138	0.72693891	0.47	0.287	4.588E-134
Dkkl1	8.321E-138	1.81491763	0.094	0.025	1.826E-133
Ybx1	3.876E-137	0.47998404	0.876	0.724	8.503E-133
Nipal3	4.129E-137	1.61536158	0.119	0.037	9.058E-133
Mrpl18	2.532E-136	1.02083959	0.298	0.155	5.556E-132
S1pr4	6.621E-136	1.36652398	0.144	0.05	1.453E-131
Atp5g1	8.215E-136	0.67282847	0.55	0.378	1.802E-131
Itpk1	1.671E-135	1.37689732	0.17	0.067	3.665E-131
Csnk2b	3.586E-135	0.90426111	0.437	0.283	7.867E-131
Cnot6	7.254E-135	0.82812216	0.397	0.234	1.591E-130
Ndufb11	1.434E-134	0.6288998	0.612	0.442	3.145E-130

Psma2	2.921E-134	0.6779788	0.565	0.401	6.408E-130
Mrps16	5.13E-134	0.9326245	0.367	0.214	1.126E-129
Epas1	2.73E-133	0.96910035	0.208	0.089	5.99E-129
Sin3b	5.283E-133	0.77518889	0.479	0.319	1.159E-128
F2r	1.78E-132	1.06588479	0.166	0.063	3.904E-128
Faah	1.867E-132	0.9179746	0.222	0.097	4.096E-128
Egln1	2.539E-132	1.22362689	0.21	0.094	5.57E-128
Edf1	2.618E-131	0.68194869	0.553	0.39	5.743E-127
Ifi27	3.354E-131	0.8987378	0.336	0.186	7.359E-127
Sv2c	5.614E-131	2.03530663	0.07	0.015	1.232E-126
Dap	6.77E-131	1.19447062	0.265	0.136	1.485E-126
Atp5j	1.664E-130	0.61627193	0.615	0.45	3.651E-126
Cox4i1	3.399E-130	0.3541094	0.953	0.839	7.456E-126
Gm26740	4.658E-130	0.54525379	0.387	0.208	1.022E-125
Wasf2	1.035E-129	0.6745468	0.53	0.361	2.271E-125
Arhgdib	1.895E-129	0.53280638	0.736	0.588	4.158E-125
Ankrd37	3.746E-129	1.65543697	0.112	0.035	8.218E-125
Prr29	7.047E-129	1.82545614	0.061	0.012	1.546E-124
Khdrbs1	7.737E-129	0.66143599	0.588	0.428	1.698E-124
Mrpl57	8.215E-129	0.93874141	0.354	0.207	1.802E-124
Ndufa4	1.026E-127	0.61770641	0.624	0.456	2.251E-123
Znrf1	1.147E-127	0.92994015	0.255	0.124	2.516E-123
Srp19	2.028E-127	0.87828118	0.386	0.234	4.448E-123
Ptpn3	2.233E-127	2.36047104	0.07	0.016	4.9E-123
Il12rb2	2.645E-127	1.09555208	0.165	0.064	5.803E-123
Prr7	2.799E-127	0.80292001	0.299	0.153	6.141E-123
Ube2j1	1.18E-126	0.89114442	0.384	0.235	2.588E-122
Btg2	1.452E-126	0.43552342	0.71	0.53	3.185E-122
Gm47015	4.422E-126	1.32864501	0.113	0.036	9.701E-122
Pla2g16	1.978E-125	0.94272241	0.319	0.177	4.339E-121
Pdcd6	3.418E-125	0.85250125	0.409	0.258	7.498E-121
Chchd2	5.072E-125	0.37331845	0.906	0.769	1.113E-120
Hsbp1l1	6.264E-125	2.88423937	0.037	0.005	1.374E-120
Rrad	7.775E-125	1.32213686	0.193	0.084	1.706E-120
Cpd	1.124E-124	1.21167084	0.205	0.093	2.467E-120
Sesn3	1.196E-124	0.68601274	0.373	0.208	2.625E-120
Pak2	1.926E-124	0.54192678	0.672	0.516	4.226E-120
Syf2	1.94E-124	0.6057948	0.566	0.399	4.256E-120
Gt(ROSA)26Sor	2.829E-124	0.83512777	0.36	0.21	6.207E-120
1190002N15R	2.87E-124	1.26035711	0.146	0.054	6.297E-120
Il23r	3.164E-124	2.03324213	0.065	0.014	6.942E-120
Rbm43	3.828E-124	1.65674082	0.103	0.031	8.397E-120
Abhd18	3.003E-123	1.6910744	0.091	0.026	6.588E-119
Rpl10	4.092E-123	0.30151804	0.989	0.921	8.977E-119
Atad2	4.572E-123	0.8183419	0.309	0.163	1.003E-118
Ndufs5	4.906E-123	0.90337224	0.391	0.248	1.076E-118
Mcm6	1.035E-122	1.14531841	0.197	0.087	2.271E-118
Ndufb9	3.411E-122	0.65691884	0.527	0.369	7.483E-118

B4galnt4	5.313E-122	2.30641154	0.052	0.009	1.166E-117
Gpsm3	8.614E-122	0.89189088	0.36	0.216	1.89E-117
Rpl14	1.335E-121	0.2817258	0.981	0.901	2.929E-117
Ppme1	2.554E-121	1.21131511	0.207	0.095	5.604E-117
Ptp4a2	1.658E-120	0.38341948	0.847	0.713	3.637E-116
Higd2a	3.392E-120	0.86481345	0.386	0.242	7.441E-116
Selenow	3.54E-120	0.52190718	0.674	0.51	7.766E-116
Psme2	3.863E-120	0.551479	0.598	0.43	8.474E-116
Gmnn	1.316E-119	1.40381748	0.14	0.053	2.888E-115
Rbx1	2.862E-119	0.61377581	0.591	0.435	6.28E-115
Pgm2l1	8.536E-119	0.89690893	0.218	0.099	1.873E-114
Brd1	1.309E-118	0.71987134	0.433	0.275	2.873E-114
Jakmip1	1.458E-118	0.75908217	0.269	0.132	3.2E-114
Top2b	1.073E-117	0.70403316	0.451	0.288	2.354E-113
Tmem64	1.529E-117	0.44547518	0.398	0.224	3.355E-113
Plekhf1	2.387E-117	2.48606555	0.048	0.008	5.237E-113
Sra1	3.445E-117	0.83315323	0.381	0.237	7.558E-113
Sec61b	8.863E-117	0.43037598	0.781	0.614	1.944E-112
Cox5b	9.166E-117	0.4750248	0.733	0.552	2.011E-112
D030056L22R	1.374E-116	1.52658157	0.122	0.043	3.014E-112
Pfkl	1.998E-116	1.36077523	0.17	0.073	4.383E-112
Sh2b1	3.619E-115	1.21740105	0.155	0.062	7.94E-111
March2	4.772E-115	1.27988667	0.19	0.086	1.047E-110
Zeb2	1.88E-114	0.29446161	0.623	0.404	4.124E-110
Cpe	4.388E-114	3.17443597	0.034	0.004	9.628E-110
Pdcd5	7.046E-114	0.72531616	0.443	0.293	1.546E-109
Dusp5	7.51E-114	0.36881351	0.722	0.526	1.648E-109
Tbrg1	1.209E-113	0.86785351	0.342	0.201	2.652E-109
Pin1	1.25E-113	1.1264231	0.257	0.137	2.743E-109
Tmem50a	1.688E-113	0.6799985	0.5	0.352	3.703E-109
Pfn1	1.917E-113	0.39094674	0.886	0.743	4.207E-109
Bzw2	2.3E-113	0.89293525	0.304	0.168	5.047E-109
Zc3hav1	3.449E-113	0.45296498	0.611	0.432	7.566E-109
Fis1	9.203E-113	0.66818889	0.512	0.363	2.019E-108
Hnrnpd	1.071E-112	0.53739721	0.614	0.455	2.35E-108
Tspan5	2.158E-112	1.62984453	0.104	0.034	4.735E-108
Sugt1	2.704E-112	0.84668829	0.378	0.238	5.932E-108
Ddx46	3.119E-112	0.69791839	0.444	0.292	6.842E-108
Eif3k	5.574E-112	0.5045529	0.716	0.558	1.223E-107
Smc3	1.861E-111	0.81114634	0.352	0.208	4.083E-107
Cox8a	2.377E-111	0.31441369	0.933	0.8	5.216E-107
Zfp292	3.603E-111	0.62752322	0.395	0.236	7.904E-107
St6galnac1	5.918E-111	3.10196414	0.029	0.003	1.298E-106
Dnajc8	7.83E-111	0.62457313	0.545	0.392	1.718E-106
Mettl23	8.501E-111	0.75221927	0.361	0.217	1.865E-106
Vps54	1.288E-110	0.81254598	0.248	0.125	2.826E-106
Dnajc15	2.089E-110	0.9795949	0.315	0.184	4.583E-106
Psma7	2.138E-110	0.49298375	0.678	0.508	4.691E-106

Dad1	2.847E-110	0.40406917	0.684	0.53	6.246E-106
Slc35d1	3.107E-110	1.55581195	0.107	0.036	6.816E-106
Mrpl43	3.711E-110	0.99364949	0.285	0.16	8.141E-106
Idh3a	3.037E-109	1.17179453	0.191	0.088	6.663E-105
Tomm20	5.34E-109	0.51502517	0.64	0.488	1.172E-104
Ptpn18	6.094E-109	0.43598206	0.756	0.603	1.337E-104
Prickle1	1.407E-108	1.99778005	0.056	0.012	3.087E-104
Srp9	2.233E-108	0.52636069	0.618	0.466	4.898E-104
Pdia3	4.818E-108	0.50040503	0.699	0.533	1.057E-103
Dctn3	7.251E-108	0.78088695	0.4	0.263	1.591E-103
Rfxap	1.716E-107	1.21903518	0.193	0.091	3.765E-103
Serbp1	4.243E-107	0.35001539	0.863	0.74	9.308E-103
Rac1	5.717E-107	0.49633392	0.673	0.519	1.254E-102
Krit1	1.065E-105	0.61507756	0.437	0.281	2.337E-101
Ero1l	1.447E-105	0.74865193	0.301	0.169	3.175E-101
Gng5	1.522E-105	0.39291633	0.801	0.635	3.339E-101
Mthfd2	1.621E-105	0.95756883	0.322	0.193	3.555E-101
Fbxo21	1.708E-105	1.6679485	0.093	0.03	3.747E-101
Sumo1	1.712E-105	0.60195625	0.519	0.371	3.756E-101
Acot7	2.041E-105	1.12555448	0.222	0.113	4.478E-101
Uqcr10	3.519E-105	0.5476369	0.597	0.446	7.719E-101
Ctcf	8.885E-105	0.63227171	0.423	0.267	1.949E-100
Cript	1.474E-104	0.59943492	0.495	0.343	3.234E-100
Tpr	3.142E-104	0.47015979	0.655	0.503	6.894E-100
Arntl	3.506E-104	1.05793338	0.171	0.075	7.692E-100
Sap30l	6.306E-104	1.1764517	0.195	0.094	1.383E-99
Ppp1r12a	1.743E-103	0.59095482	0.499	0.343	3.823E-99
Etaa1	3.109E-103	1.20088532	0.138	0.055	6.821E-99
Ndufa11	4.938E-103	0.76139388	0.414	0.279	1.0834E-98
Tomm7	7.856E-103	0.41779241	0.78	0.635	1.7236E-98
Tnrc6b	1.065E-102	0.44849217	0.512	0.334	2.336E-98
Uqcrfs1	1.392E-102	0.69055744	0.474	0.335	3.0547E-98
Ppp1r11	3.274E-102	0.68497784	0.404	0.26	7.1832E-98
Fkbp1a	4.852E-102	0.79466661	0.415	0.281	1.0644E-97
Pik3r1	8.321E-102	0.48662107	0.516	0.353	1.8255E-97
Kif2a	3.584E-101	1.06284372	0.226	0.117	7.862E-97
Dennd5a	3.611E-101	0.77256684	0.37	0.234	7.9218E-97
Ndufb8	5.258E-101	0.70794959	0.444	0.306	1.1536E-96
Cxcr3	6.604E-101	1.27060048	0.118	0.043	1.4488E-96
Eif3c	8.027E-101	0.54250652	0.658	0.525	1.7611E-96
Rab39b	1.184E-100	2.08656228	0.052	0.011	2.598E-96
Sf3b6	1.203E-100	0.54387931	0.55	0.403	2.6383E-96
Tigit	2.11E-100	1.63425006	0.073	0.02	4.6284E-96
Tnfaip8l2	6.438E-100	1.38133553	0.149	0.064	1.4125E-95
Chst2	7.103E-100	1.56206713	0.076	0.022	1.5583E-95
Ssr4	1.245E-99	0.50417081	0.614	0.447	2.7315E-95
Ndufa1	2.724E-99	0.58582177	0.553	0.411	5.9763E-95
Foxo1	4.064E-99	0.46343825	0.466	0.297	8.9152E-95

Frg1	4.527E-99	0.71806915	0.361	0.225	9.9315E-95
Psmd4	5.768E-99	0.67052293	0.447	0.308	1.2654E-94
Ndufs4	5.806E-99	0.83061796	0.348	0.221	1.2739E-94
Cenpw	1.4267E-98	1.27202905	0.142	0.059	3.13E-94
Akirin1	1.5908E-98	0.79966075	0.347	0.217	3.4901E-94
Nudcd2	1.9546E-97	1.34660349	0.152	0.067	4.2882E-93
Lilr4b	2.4727E-97	0.25844772	0.589	0.41	5.4248E-93
Pja1	3.5617E-97	1.09842037	0.157	0.068	7.8141E-93
Kmt2a	3.7651E-97	0.45917234	0.481	0.316	8.2602E-93
Tctex1d1	6.1943E-97	3.13624142	0.025	0.003	1.359E-92
Pqlc1	1.4555E-96	1.3218456	0.173	0.082	3.1933E-92
Ech1	1.5422E-96	1.26185642	0.192	0.095	3.3833E-92
Tmem234	2.0832E-96	0.5559551	0.555	0.414	4.5702E-92
Ndufb7	2.7243E-96	0.5832247	0.507	0.366	5.9769E-92
Scand1	6.2304E-96	0.55394037	0.612	0.466	1.3669E-91
Mrps24	8.0952E-96	0.82901707	0.339	0.214	1.776E-91
Rpl13a	1.0786E-95	0.43009118	0.712	0.57	2.3664E-91
Atp5l	1.2056E-95	0.33516659	0.885	0.754	2.6449E-91
Prrc2b	1.2198E-95	0.7387238	0.304	0.175	2.6762E-91
Abcb1a	1.6671E-95	0.54551272	0.299	0.167	3.6574E-91
Hs2st1	2.0755E-95	1.44332435	0.137	0.058	4.5535E-91
Acpp	2.1523E-95	1.60746624	0.092	0.03	4.7219E-91
Ptprcap	2.2927E-95	0.58870094	0.338	0.194	5.0299E-91
Top2a	2.8368E-95	0.64249449	0.179	0.083	6.2236E-91
Cox6b1	4.5894E-95	0.40379933	0.771	0.612	1.0069E-90
Zdhhc2	8.9824E-95	1.34202019	0.104	0.037	1.9707E-90
Hint1	1.1465E-94	0.45486448	0.712	0.564	2.5154E-90
Il21r	1.583E-94	0.45393255	0.469	0.311	3.473E-90
Cenpl	1.081E-93	1.20810306	0.173	0.082	2.3716E-89
Gpr68	1.8876E-93	1.11717986	0.16	0.072	4.1412E-89
Gm16286	1.891E-93	0.74215588	0.381	0.25	4.1486E-89
Ppp1ca	1.9249E-93	0.5807398	0.556	0.426	4.2231E-89
Selplg	3.2519E-93	0.42872864	0.561	0.401	7.1344E-89
Podnl1	3.3146E-93	1.37120618	0.085	0.027	7.2718E-89
Capg	4.3535E-93	0.84862079	0.312	0.187	9.5512E-89
Uqcrq	5.5379E-93	0.40876263	0.722	0.562	1.215E-88
Mrps18c	7.5524E-93	0.86466045	0.307	0.187	1.6569E-88
Nfkb2	2.3308E-92	0.61607082	0.431	0.292	5.1136E-88
Gnas	3.9763E-92	0.36732709	0.813	0.698	8.7236E-88
Tnik	1.533E-91	0.84733243	0.181	0.084	3.3632E-87
Sec62	2.4798E-91	0.41919986	0.668	0.522	5.4405E-87
Smpdl3a	2.9074E-91	0.81505106	0.292	0.174	6.3786E-87
Cks2	4.6716E-91	0.70636518	0.268	0.152	1.0249E-86
Sla2	6.9951E-91	1.28599797	0.102	0.037	1.5347E-86
Dek	9.7943E-91	0.4353714	0.631	0.478	2.1488E-86
Chd1	1.6666E-90	0.72190202	0.364	0.234	3.6563E-86
Sms	3.0588E-90	0.76050514	0.334	0.207	6.7108E-86
Gpc1	5.8119E-90	1.49839175	0.1	0.036	1.2751E-85

Olfr1369-ps1	7.3588E-90	2.08001351	0.065	0.018	1.6144E-85
Pex16	1.037E-89	1.22843158	0.157	0.072	2.275E-85
B3galt2	4.7515E-89	1.77087205	0.059	0.015	1.0424E-84
Zcchc11	2.6088E-88	0.34265258	0.709	0.543	5.7234E-84
Tmem160	7.2571E-88	0.70817341	0.42	0.297	1.5921E-83
Tmem167	1.1082E-87	0.65798202	0.418	0.29	2.4313E-83
Srpr	1.6577E-87	0.6845832	0.398	0.27	3.6369E-83
Ppp1r14c	2.5304E-87	2.39567291	0.038	0.007	5.5514E-83
Smdt1	6.8361E-87	0.47693124	0.601	0.459	1.4998E-82
Rtraf	4.2575E-86	0.56403441	0.504	0.37	9.3405E-82
Tm2d3	5.0094E-86	1.21395429	0.14	0.061	1.099E-81
Ptger2	5.3562E-86	1.63468414	0.072	0.022	1.1751E-81
Psma4	5.4263E-86	0.74416187	0.381	0.259	1.1905E-81
Mbnl3	6.4789E-86	1.37599403	0.104	0.039	1.4214E-81
Abhd17c	7.631E-86	0.87552501	0.155	0.07	1.6742E-81
Nars	9.7592E-86	0.67413028	0.469	0.343	2.1411E-81
Gimap6	1.2322E-85	0.26356797	0.599	0.398	2.7034E-81
Nif3l1	1.2566E-85	1.76483985	0.071	0.021	2.7568E-81
Lpxn	1.3688E-85	0.69148532	0.352	0.226	3.0031E-81
Kcnk7	2.0846E-85	2.63060091	0.03	0.004	4.5733E-81
Srpk1	2.7372E-85	0.72838126	0.298	0.178	6.005E-81
Cox7a2	3.3227E-85	0.38528627	0.712	0.572	7.2898E-81
Mast4	5.3341E-85	0.67838191	0.289	0.168	1.1702E-80
Rundc3b	1.8336E-84	1.07972127	0.132	0.056	4.0228E-80
H1f0	1.933E-84	1.19003398	0.175	0.087	4.2409E-80
Leo1	1.6122E-83	1.30608495	0.12	0.05	3.5371E-79
Alkbh5	1.6751E-83	0.75017052	0.334	0.214	3.6751E-79
Spsb3	1.7227E-83	1.13506799	0.14	0.062	3.7794E-79
Eef2	1.8678E-83	0.25517623	0.956	0.871	4.0977E-79
Fam160b1	1.8176E-82	1.03831694	0.155	0.072	3.9875E-78
Shisa5	1.9984E-82	0.30263433	0.734	0.579	4.3842E-78
Unc119b	2.0861E-82	1.04646163	0.172	0.084	4.5766E-78
Atp5g3	4.9279E-82	0.5233303	0.548	0.416	1.0811E-77
Plekha5	4.9508E-82	1.1249833	0.132	0.057	1.0862E-77
Aprt	5.6266E-82	0.63629957	0.446	0.322	1.2344E-77
Zadh2	7.9677E-82	1.26845894	0.099	0.037	1.748E-77
Zcrb1	1.1201E-81	0.66017466	0.413	0.291	2.4575E-77
Sept6	1.5848E-81	0.68499038	0.317	0.194	3.4768E-77
Klf13	3.3662E-81	0.5721984	0.437	0.302	7.385E-77
Dgka	8.6988E-81	0.69637079	0.204	0.104	1.9084E-76
Gars	9.3997E-81	0.99514641	0.23	0.13	2.0622E-76
Ss18	1.2547E-80	0.72480636	0.272	0.159	2.7526E-76
Rtf1	1.5406E-80	0.56238838	0.454	0.321	3.3799E-76
Zfand6	2.6632E-80	0.63562285	0.39	0.263	5.8427E-76
Txndc17	4.9008E-80	0.68220789	0.406	0.289	1.0752E-75
Sptssa	1.0014E-79	0.51097277	0.356	0.233	2.1971E-75
Dubr	1.9002E-79	1.25812485	0.103	0.04	4.1688E-75
Mphosph6	2.4298E-79	1.05104843	0.182	0.093	5.3308E-75

Rnf157	3.3715E-79	0.57281131	0.318	0.194	7.3968E-75
Atp5c1	3.5884E-79	0.48734363	0.575	0.441	7.8727E-75
Rnf138	6.2867E-79	0.58864109	0.31	0.188	1.3792E-74
Ormdl3	1.0934E-78	0.91686125	0.157	0.074	2.3989E-74
Slc39a4	1.1846E-78	1.48894431	0.07	0.022	2.5989E-74
Arl15	4.8358E-78	1.58046047	0.073	0.024	1.0609E-73
Asns	6.163E-78	1.38049499	0.114	0.048	1.3521E-73
Gm29243	9.777E-78	1.46689491	0.072	0.023	2.145E-73
Tsg101	1.0033E-77	0.96172725	0.216	0.121	2.2012E-73
Tmem258	1.5293E-77	0.56281261	0.496	0.375	3.3551E-73
Pfdn2	1.646E-77	0.59965443	0.42	0.294	3.6111E-73
Cox7a2l	1.679E-77	0.37955419	0.716	0.587	3.6836E-73
Chd2	2.5715E-77	0.40390807	0.624	0.483	5.6416E-73
Fam124b	2.9311E-77	2.084851	0.039	0.008	6.4305E-73
Calm2	6.9686E-77	0.36051946	0.745	0.609	1.5288E-72
Serpinb6a	7.4763E-77	0.80119917	0.174	0.086	1.6402E-72
Gtdc1	8.5552E-77	1.16960867	0.111	0.046	1.8769E-72
Tmem263	8.791E-77	1.17977905	0.106	0.043	1.9287E-72
Psmb6	2.03E-76	0.51877845	0.484	0.357	4.4536E-72
Polr2c	3.9108E-76	0.88774178	0.255	0.154	8.58E-72
Polg2	4.1831E-76	0.91287837	0.144	0.067	9.1774E-72
Jpt1	5.8955E-76	0.51128206	0.506	0.376	1.2934E-71
Grpel1	2.0259E-75	0.89396246	0.252	0.153	4.4447E-71
Pik3ca	2.0621E-75	0.9091553	0.184	0.095	4.5241E-71
Rasgrp1	3.2118E-75	0.56160719	0.264	0.151	7.0463E-71
Nol7	4.8455E-75	0.50298945	0.537	0.414	1.0631E-70
St8sia1	4.9699E-75	1.38608012	0.071	0.023	1.0903E-70
Hp1bp3	5.7429E-75	0.60959147	0.378	0.257	1.2599E-70
Gm20069	8.4432E-75	1.52781587	0.065	0.02	1.8524E-70
Gm17745	1.146E-74	1.47640801	0.075	0.026	2.5143E-70
Fam103a1	2.0505E-74	0.72482675	0.293	0.183	4.4985E-70
Selenok	2.502E-74	0.32113685	0.767	0.627	5.4891E-70
Bola3	3.159E-74	0.85192466	0.237	0.139	6.9305E-70
Trib2	3.8521E-74	0.4889174	0.221	0.119	8.4512E-70
Trbv2	9.9702E-74	2.37269353	0.026	0.004	2.1874E-69
Rbfa	1.5557E-73	1.09081288	0.174	0.091	3.4131E-69
Hip1r	1.864E-73	0.62334823	0.23	0.126	4.0894E-69
Zufsp	1.8758E-73	0.5359334	0.339	0.216	4.1154E-69
Impa2	2.1845E-73	1.40069435	0.11	0.047	4.7925E-69
Smap1	2.3925E-73	0.63037572	0.387	0.27	5.249E-69
Ssna1	8.9482E-73	1.0362532	0.159	0.08	1.9631E-68
Psmb5	9.5243E-73	0.67402867	0.366	0.254	2.0895E-68
Surf4	1.1638E-72	0.6319064	0.38	0.266	2.5532E-68
Polr2j	5.6635E-72	1.08267373	0.174	0.092	1.2425E-67
Rorc	7.9093E-72	1.90384575	0.045	0.011	1.7352E-67
Tmem123	9.0462E-72	0.36844048	0.59	0.452	1.9847E-67
Malsu1	9.2009E-72	1.01994109	0.198	0.111	2.0186E-67
Spcs1	1.5263E-71	0.64259948	0.388	0.277	3.3484E-67

Dock2	1.7388E-71	0.43139316	0.546	0.411	3.8147E-67
Gna13	2.2975E-71	0.33266716	0.794	0.668	5.0406E-67
Clic1	1.3288E-70	0.45064173	0.564	0.434	2.9153E-66
Sap18	1.3788E-70	0.69402401	0.313	0.206	3.0248E-66
Tbl1x	1.437E-70	0.73665911	0.282	0.177	3.1527E-66
Nktr	1.6027E-70	0.35001345	0.689	0.573	3.5162E-66
Stk10	1.6222E-70	0.61851036	0.308	0.193	3.5589E-66
Cdon	1.8298E-70	1.72857882	0.055	0.016	4.0143E-66
Tmed2	3.0016E-70	0.47117003	0.548	0.43	6.5851E-66
Sars	3.8148E-70	0.62130578	0.351	0.235	8.3693E-66
Taf10	3.9417E-70	0.50514558	0.504	0.386	8.6478E-66
Purb	6.1146E-70	0.42861687	0.627	0.503	1.3415E-65
2410015M20F	6.761E-70	0.60816001	0.409	0.296	1.4833E-65
Eprs	7.1244E-70	0.42165292	0.52	0.397	1.563E-65
Cmc2	7.4255E-70	1.20345506	0.145	0.072	1.6291E-65
Dctn4	1.096E-69	0.51266438	0.445	0.324	2.4046E-65
Iltifb	1.2208E-69	3.10063172	0.017	0.002	2.6783E-65
Il17f	1.2736E-69	0.68557559	0.077	0.027	2.7941E-65
Ppp1r3b	1.8107E-69	0.64607033	0.135	0.062	3.9725E-65
Raf1	1.8309E-69	0.64652596	0.251	0.148	4.0168E-65
Snapc5	1.9431E-69	1.04034763	0.199	0.113	4.263E-65
Gadd45gip1	2.0137E-69	0.83677134	0.234	0.138	4.4178E-65
1110012L19Ri	2.3315E-69	1.42071411	0.083	0.031	5.1151E-65
2410089E03R	3.4013E-69	1.14510031	0.114	0.05	7.4622E-65
Stk32c	4.6938E-69	1.6728498	0.059	0.018	1.0298E-64
Rlf	6.6056E-69	0.59376246	0.34	0.227	1.4492E-64
Pym1	2.2283E-68	1.03186835	0.159	0.081	4.8886E-64
Klrc1	2.5581E-68	0.33261228	0.189	0.098	5.6122E-64
Tmem191c	3.5847E-68	1.67986928	0.059	0.019	7.8646E-64
U2af1	3.8197E-68	0.59577273	0.403	0.291	8.38E-64
4932438H23R	3.8691E-68	1.73269457	0.047	0.013	8.4884E-64
Fam92a	4.3362E-68	1.2525493	0.093	0.037	9.5132E-64
Snrpd2	5.9311E-68	0.47496866	0.501	0.38	1.3012E-63
Minos1	6.4425E-68	0.43566467	0.586	0.468	1.4134E-63
Trir	6.5311E-68	0.51426824	0.462	0.344	1.4328E-63
Cd226	6.8118E-68	1.04711632	0.107	0.045	1.4944E-63
Psma5	8.0754E-68	0.70401407	0.352	0.248	1.7717E-63
Stk11	8.5E-68	0.67350243	0.326	0.217	1.8648E-63
Ndufa8	9.144E-68	0.80041849	0.299	0.2	2.0061E-63
3110043O21R	1.0008E-67	0.99527604	0.199	0.113	2.1957E-63
Fam98b	1.099E-67	0.74201878	0.248	0.15	2.4111E-63
Naa20	1.322E-67	0.94324232	0.181	0.098	2.9004E-63
Sod1	2.938E-67	0.3981035	0.634	0.513	6.4456E-63
Bcl2l1	2.9555E-67	0.69763187	0.198	0.109	6.4841E-63
Tcf25	3.5502E-67	0.40996551	0.623	0.507	7.7888E-63
Rsbn1l	3.7701E-67	0.38212765	0.49	0.351	8.2712E-63
Sike1	4.2806E-67	0.91036481	0.167	0.087	9.3913E-63
Mri1	5.2692E-67	1.08262378	0.145	0.072	1.156E-62

Zfp266	6.1383E-67	0.80673369	0.165	0.084	1.3467E-62
Sit1	9.0494E-67	1.30301188	0.066	0.022	1.9853E-62
Lsm12	1.2306E-66	0.6115371	0.412	0.303	2.6998E-62
Rbl2	1.2388E-66	0.6271891	0.236	0.137	2.7178E-62
Stk39	1.5435E-66	1.04278715	0.108	0.047	3.3864E-62
Gm15283	1.9889E-66	1.70923423	0.054	0.016	4.3634E-62
Sf3b5	2.3171E-66	0.54676319	0.422	0.31	5.0834E-62
Wasl	4.1848E-66	0.97681597	0.164	0.086	9.181E-62
Itga3	4.7075E-66	2.12520248	0.046	0.012	1.0328E-61
Plcg1	4.9518E-66	0.84472767	0.14	0.067	1.0864E-61
Tmem59	8.5338E-66	0.62644654	0.354	0.246	1.8722E-61
Nsd3	1.1134E-65	0.36005506	0.536	0.401	2.4427E-61
Psmb2	1.1934E-65	0.71400314	0.346	0.245	2.6181E-61
Pkm	1.5968E-65	0.35093734	0.563	0.432	3.5031E-61
Mapkapk3	2.1383E-65	0.99675411	0.223	0.135	4.6912E-61
Mapk14	2.4745E-65	0.96566617	0.186	0.104	5.4288E-61
Gpi1	2.5786E-65	0.4847354	0.515	0.4	5.6572E-61
Mrpl20	2.9054E-65	0.73815608	0.303	0.205	6.3742E-61
Ubn1	4.7958E-65	0.57877844	0.368	0.257	1.0522E-60
Arl2bp	5.01E-65	0.67423334	0.252	0.154	1.0992E-60
Ndufa5	5.542E-65	0.71019789	0.315	0.213	1.2159E-60
Neurl3	6.3107E-65	0.27632381	0.652	0.513	1.3845E-60
Eif2s2	7.1429E-65	0.30142155	0.796	0.668	1.5671E-60
1700020I14Ril	7.8647E-65	0.59584738	0.301	0.195	1.7254E-60
Tbc1d10b	8.2989E-65	1.08493655	0.137	0.068	1.8207E-60
Ldlrad4	8.3781E-65	1.1324803	0.104	0.045	1.8381E-60
Hsp90b1	8.9956E-65	0.25597267	0.842	0.713	1.9736E-60
Itgb3	9.2871E-65	0.53908077	0.196	0.105	2.0375E-60
Ciapin1	9.4695E-65	0.95531738	0.165	0.088	2.0775E-60
Aqp3	1.1477E-64	1.77321637	0.041	0.01	2.5179E-60
Bcas2	1.9645E-64	0.57615328	0.362	0.25	4.3099E-60
Zc3h13	2.0856E-64	0.52649335	0.399	0.282	4.5756E-60
Zwint	2.3771E-64	1.08122527	0.141	0.07	5.215E-60
Synj2	2.4975E-64	1.41859088	0.086	0.034	5.4792E-60
Uqcc3	2.5777E-64	0.72108735	0.239	0.145	5.6553E-60
Zfp706	2.8367E-64	0.55124291	0.388	0.274	6.2233E-60
Slc3a2	3.5024E-64	0.31041051	0.744	0.603	7.6839E-60
Snapc1	1.119E-63	0.76350832	0.212	0.123	2.4549E-59
Slc25a40	1.1652E-63	1.12773543	0.091	0.037	2.5563E-59
Dda1	1.7684E-63	0.76876628	0.244	0.151	3.8796E-59
Utrn	2.8826E-63	0.43168541	0.356	0.234	6.3241E-59
Dcun1d5	3.0213E-63	0.58902321	0.378	0.271	6.6283E-59
Cdc37	3.9358E-63	0.50025401	0.452	0.339	8.6348E-59
Avpi1	8.4606E-63	1.02070126	0.216	0.131	1.8562E-58
Eif3m	9.3683E-63	0.40759769	0.539	0.421	2.0553E-58
Pam16	1.3532E-62	0.84392399	0.229	0.14	2.9688E-58
Myh9	1.6237E-62	0.35818582	0.686	0.574	3.5622E-58
Chmp2a	2.2301E-62	0.4998201	0.47	0.356	4.8927E-58

Stx11	2.6232E-62	0.58995255	0.273	0.174	5.755E-58
Nudt21	5.7222E-62	0.67434689	0.306	0.207	1.2554E-57
Mea1	1.1936E-61	1.00417758	0.177	0.1	2.6187E-57
Ly75	1.2682E-61	1.29619291	0.105	0.047	2.7823E-57
Ube2j2	1.6318E-61	0.70418969	0.314	0.216	3.5799E-57
P2rx7	1.6555E-61	1.15407906	0.102	0.045	3.632E-57
Atp5o.1	2.2592E-61	0.49058177	0.466	0.358	4.9565E-57
Phax	2.9877E-61	0.95339607	0.178	0.1	6.5547E-57
4932438A13R	4.0781E-61	0.33737449	0.508	0.376	8.9469E-57
Zc3h15	4.5365E-61	0.45091079	0.491	0.376	9.9526E-57
Calm3	5.0949E-61	0.49963085	0.429	0.323	1.1178E-56
Tpm3	9.4675E-61	0.32875231	0.743	0.626	2.0771E-56
Csnk1a1	2.7977E-60	0.33607027	0.676	0.566	6.1379E-56
Ndufa3	2.8608E-60	0.43253044	0.513	0.401	6.2763E-56
Thoc7	3.1034E-60	0.6848994	0.292	0.195	6.8086E-56
Pop5	3.8253E-60	0.82999504	0.193	0.111	8.3924E-56
4930523C07R	7.4922E-60	0.28430887	0.559	0.415	1.6437E-55
Senp6	1.0285E-59	0.39606796	0.474	0.349	2.2563E-55
Narf	1.6492E-59	1.01307925	0.112	0.052	3.6183E-55
9930021J03Ril	1.965E-59	0.93334882	0.155	0.082	4.3109E-55
Eloc	2.0483E-59	0.44783691	0.481	0.372	4.4938E-55
Golim4	3.0519E-59	1.27932217	0.114	0.054	6.6956E-55
Tmem176a	3.4847E-59	0.44973207	0.366	0.249	7.6451E-55
Dusp4	3.6386E-59	0.63997122	0.216	0.127	7.9828E-55
9930111J21Ril	4.5328E-59	0.52875061	0.207	0.118	9.9445E-55
Fam104a	5.9617E-59	0.53270597	0.379	0.274	1.3079E-54
Dnajb11	6.0385E-59	0.7322634	0.312	0.218	1.3248E-54
1810058I24Ril	1.198E-58	0.5342655	0.386	0.283	2.6283E-54
Lsm5	1.4328E-58	0.50138978	0.402	0.292	3.1435E-54
Nol10	1.8695E-58	0.94937835	0.151	0.08	4.1015E-54
Chrac1	1.9319E-58	0.74760989	0.27	0.178	4.2384E-54
Rpp25l	2.4984E-58	0.85834375	0.17	0.094	5.4813E-54
Crlf3	2.899E-58	0.3699131	0.473	0.351	6.3601E-54
Smim15	3.2143E-58	0.74268309	0.226	0.138	7.0519E-54
Wisp1	4.526E-58	2.1993416	0.022	0.004	9.9296E-54
Saraf	4.5823E-58	0.31620084	0.5	0.371	1.0053E-53
Morf4l1	5.4957E-58	0.27814879	0.81	0.706	1.2057E-53
1810037I17Ril	9.4711E-58	0.35808746	0.572	0.451	2.0779E-53
Erg28	1.1068E-57	0.99816058	0.136	0.07	2.4283E-53
Maf1	1.3079E-57	0.66659692	0.224	0.136	2.8694E-53
H60b	1.3141E-57	1.55380855	0.048	0.015	2.883E-53
Derl2	2.1886E-57	0.79621984	0.216	0.132	4.8015E-53
Mrpl44	2.6153E-57	1.20382769	0.087	0.037	5.7378E-53
Nkap	5.1533E-57	0.82384287	0.182	0.104	1.1306E-52
Wbp1	6.0576E-57	1.19628055	0.102	0.047	1.329E-52
Pbrm1	7.4448E-57	0.52524213	0.378	0.27	1.6333E-52
Ppdpf	8.8352E-57	0.69600212	0.254	0.163	1.9384E-52
Timm17a	1.4559E-56	0.7464215	0.251	0.163	3.194E-52

Slain1	1.7188E-56	0.74465755	0.131	0.065	3.7708E-52
Arpc5	1.7415E-56	0.26311772	0.774	0.649	3.8206E-52
Sec11a	2.1926E-56	0.57420009	0.348	0.248	4.8104E-52
Med10	2.2615E-56	0.80595097	0.23	0.146	4.9616E-52
Il27ra	2.2752E-56	0.81613708	0.111	0.052	4.9915E-52
Ston2	2.814E-56	1.25586718	0.119	0.059	6.1736E-52
Mgea5	3.3803E-56	0.41100671	0.453	0.335	7.4161E-52
Psmb8	6.2978E-56	0.34710524	0.559	0.434	1.3817E-51
Eif3i	6.6607E-56	0.5301122	0.416	0.316	1.4613E-51
Coro1a	8.4253E-56	0.33722269	0.71	0.601	1.8484E-51
Ccdc88c	9.1348E-56	0.40739131	0.245	0.146	2.0041E-51
Arl6ip4	1.1196E-55	0.95742709	0.171	0.098	2.4564E-51
Gimap5	1.1619E-55	0.27193616	0.342	0.219	2.5492E-51
Exosc4	1.3695E-55	1.11005538	0.108	0.051	3.0046E-51
Ddx24	1.5081E-55	0.31983428	0.615	0.499	3.3086E-51
Ssb	1.5617E-55	0.41582805	0.485	0.376	3.4263E-51
Rab4a	2.1241E-55	0.98381028	0.084	0.035	4.66E-51
Aak1	4.1308E-55	0.56891512	0.209	0.123	9.0627E-51
Tiprl	4.7179E-55	0.4430145	0.279	0.178	1.0351E-50
Tbcb	8.6758E-55	0.7533527	0.288	0.201	1.9034E-50
Churc1	9.6383E-55	0.64613481	0.301	0.207	2.1146E-50
Farp1	1.0709E-54	1.65943397	0.042	0.012	2.3494E-50
Exosc8	2.1577E-54	0.82847102	0.181	0.105	4.7337E-50
Ost4	2.4013E-54	0.35173551	0.582	0.468	5.2683E-50
Atp5g2	2.5809E-54	0.27246587	0.806	0.684	5.6623E-50
Slc25a19	3.4758E-54	0.91014979	0.161	0.09	7.6256E-50
Mrpl41	4.0949E-54	0.81672427	0.169	0.096	8.9838E-50
Creb3l2	4.2959E-54	1.1599669	0.088	0.038	9.4247E-50
Bet1	4.572E-54	1.03743708	0.142	0.076	1.003E-49
Ezh1	7.9047E-54	0.77693469	0.153	0.083	1.7342E-49
Cox10	8.3757E-54	1.12759847	0.102	0.047	1.8375E-49
Batf	9.2322E-54	0.57885284	0.267	0.174	2.0255E-49
0610012G03R	9.3207E-54	0.85463579	0.208	0.13	2.0449E-49
Mrpl9	9.8613E-54	0.64309384	0.264	0.173	2.1635E-49
Fgfr1op2	1.3645E-53	0.48267066	0.403	0.299	2.9935E-49
Igtp	1.6085E-53	0.58217142	0.205	0.121	3.5289E-49
Timm8b	2.1135E-53	0.71503992	0.267	0.181	4.6367E-49
Ift20	2.137E-53	0.74897211	0.238	0.154	4.6884E-49
Fbxl14	2.4863E-53	0.92061283	0.112	0.054	5.4547E-49
Med30	2.9621E-53	0.83366956	0.187	0.111	6.4986E-49
Dynlrb1	3.0399E-53	0.57865282	0.364	0.271	6.6693E-49
Bex3	4.5303E-53	0.5894208	0.291	0.196	9.9389E-49
Gm42372	6.2778E-53	1.83555716	0.031	0.007	1.3773E-48
Trp53	9.3211E-53	0.675491	0.246	0.159	2.045E-48
Cds2	9.7492E-53	0.7511488	0.152	0.082	2.1389E-48
Cox6a1	1.0948E-52	0.34271601	0.62	0.508	2.402E-48
Gemin7	1.7852E-52	0.82111569	0.182	0.108	3.9165E-48
G3bp2	2.3467E-52	0.42448703	0.355	0.248	5.1484E-48

Rnf166	2.4643E-52	0.75449422	0.189	0.112	5.4065E-48
Cd2bp2	3.4639E-52	0.72665328	0.191	0.114	7.5993E-48
Zbtb38	5.633E-52	0.87536226	0.145	0.079	1.2358E-47
Trbv3	7.3295E-52	2.30094293	0.02	0.003	1.608E-47
Ccdc12	8.3094E-52	0.44699446	0.422	0.32	1.823E-47
Pnkd	9.3957E-52	1.02209637	0.132	0.07	2.0613E-47
Dhx36	1.5423E-51	0.50667241	0.331	0.231	3.3836E-47
Map4k2	1.5873E-51	0.61160565	0.173	0.097	3.4823E-47
Ctdsp2	2.0562E-51	0.70662487	0.223	0.142	4.511E-47
Eef1d	2.8061E-51	0.29855809	0.677	0.57	6.1562E-47
Gm20139	2.9637E-51	2.20358333	0.021	0.004	6.502E-47
Cisd1	3.1104E-51	0.97503641	0.141	0.077	6.824E-47
Aip	3.2131E-51	0.77348988	0.185	0.11	7.0493E-47
Aars	3.2663E-51	0.65075044	0.265	0.177	7.166E-47
Ap2s1	3.8151E-51	0.62080373	0.349	0.263	8.37E-47
Med28	5.1697E-51	0.5829764	0.313	0.222	1.1342E-46
Setd5	6.0701E-51	0.34028508	0.525	0.415	1.3317E-46
Uqcc2	6.3768E-51	0.68020987	0.282	0.196	1.399E-46
Gm33104	6.6037E-51	0.94537028	0.094	0.043	1.4488E-46
Gpr132	7.1286E-51	0.25711378	0.625	0.503	1.5639E-46
Snu13	8.0934E-51	0.3746368	0.495	0.389	1.7756E-46
Rnf7	8.8292E-51	0.64909068	0.291	0.204	1.937E-46
Xpa	1.1818E-50	0.81611324	0.174	0.102	2.5927E-46
Runx1	1.3689E-50	0.39502193	0.451	0.343	3.0032E-46
Hsbp1	1.6113E-50	0.65862924	0.297	0.211	3.5351E-46
Isca1	1.6628E-50	0.66417871	0.166	0.094	3.6481E-46
Nmb	2.5411E-50	1.21896779	0.06	0.022	5.5749E-46
1110034G24R	2.9026E-50	1.38615737	0.055	0.02	6.3681E-46
Cd200r1	3.0161E-50	1.05527334	0.092	0.042	6.6171E-46
Txnl4a	3.191E-50	0.97881389	0.159	0.092	7.0008E-46
Ass1	4.0768E-50	0.64817478	0.212	0.133	8.9441E-46
Mxd4	4.21E-50	0.49899661	0.266	0.173	9.2362E-46
Apol7e	5.7332E-50	1.33798897	0.053	0.018	1.2578E-45
2610524H06R	7.3829E-50	1.38766863	0.083	0.037	1.6197E-45
Vps36	8.2406E-50	0.71974504	0.248	0.166	1.8079E-45
Ndufa7	9.992E-50	0.36850966	0.537	0.435	2.1921E-45
Mdh1	1.1531E-49	0.52934353	0.36	0.266	2.5298E-45
Neu3	1.5463E-49	1.37133848	0.048	0.016	3.3925E-45
Aplp2	1.6736E-49	0.38156841	0.433	0.325	3.6718E-45
Ddx42	3.6512E-49	0.51983839	0.337	0.242	8.0103E-45
Pdcd4	4.4309E-49	0.27436836	0.437	0.312	9.721E-45
Areg	4.4704E-49	1.18624865	0.201	0.123	9.8076E-45
Ntan1	6.0682E-49	0.74565754	0.22	0.142	1.3313E-44
Sik2	6.15E-49	0.75085376	0.173	0.101	1.3492E-44
Mrps5	6.2119E-49	0.89832289	0.144	0.08	1.3628E-44
Avl9	6.793E-49	0.39766509	0.327	0.224	1.4903E-44
Pmepa1	1.0802E-48	0.4799011	0.363	0.261	2.3699E-44
Eif2d	1.6405E-48	1.16056191	0.083	0.037	3.5991E-44

Tomm22	1.6463E-48	0.52515203	0.39	0.3	3.6119E-44
Galnt9	2.0312E-48	2.38830223	0.018	0.003	4.4561E-44
Ubxn1	2.3678E-48	0.42372784	0.46	0.364	5.1946E-44
4833439L19Ri	3.1738E-48	0.93518451	0.151	0.086	6.963E-44
Zbtb25	3.3323E-48	0.91916006	0.105	0.051	7.3107E-44
Tsn	3.6974E-48	0.50458819	0.33	0.237	8.1118E-44
Ndufb6	4.6443E-48	0.63697177	0.301	0.217	1.0189E-43
Rad21	4.6471E-48	0.41620462	0.36	0.257	1.0195E-43
Ar	5.1971E-48	0.77383387	0.174	0.101	1.1402E-43
Mrps23	7.3058E-48	0.72823732	0.207	0.131	1.6028E-43
Rad18	7.4261E-48	0.77054739	0.132	0.07	1.6292E-43
Zfp87	7.5263E-48	0.91571103	0.113	0.058	1.6512E-43
Txnl1	8.2785E-48	0.61434447	0.314	0.229	1.8162E-43
Trbv12-2	9.1275E-48	2.03625787	0.024	0.005	2.0025E-43
Gnai2	1.0901E-47	0.26817735	0.788	0.661	2.3915E-43
Gpatch11	1.1457E-47	0.98122658	0.106	0.053	2.5136E-43
Hnrnpa0	1.1601E-47	0.28983786	0.7	0.6	2.5451E-43
Bub3	1.4717E-47	0.6231754	0.231	0.151	3.2288E-43
Ccdc107	1.8962E-47	0.93174007	0.17	0.102	4.1602E-43
Sf3b2	1.8994E-47	0.36501285	0.497	0.396	4.167E-43
Mdp1	2.3961E-47	0.74922275	0.18	0.108	5.2568E-43
Zfp672	2.5878E-47	0.75038594	0.177	0.105	5.6773E-43
Selenof	2.833E-47	0.50358234	0.413	0.323	6.2153E-43
Prkca	3.2924E-47	0.64587619	0.125	0.064	7.2233E-43
Commd2	3.6079E-47	0.93734239	0.134	0.074	7.9153E-43
Rab27a	3.777E-47	0.99398224	0.093	0.044	8.2864E-43
Tab2	4.0652E-47	0.29151928	0.522	0.41	8.9186E-43
Pebp1	4.5796E-47	0.55368486	0.337	0.251	1.0047E-42
Susd3	5.8381E-47	1.06684637	0.107	0.054	1.2808E-42
Acot9	6.2856E-47	1.0621585	0.122	0.065	1.379E-42
Rdx	7.6992E-47	0.43080247	0.37	0.274	1.6891E-42
Hint3	9.6352E-47	0.85870394	0.175	0.106	2.1139E-42
Chic2	1.7111E-46	0.45582262	0.369	0.275	3.754E-42
Nedd8	1.8293E-46	0.39468949	0.496	0.396	4.0133E-42
Brap	1.8956E-46	0.68146353	0.206	0.13	4.1588E-42
2210011C24R	2.7332E-46	2.34969792	0.018	0.003	5.9963E-42
Unc50	3.6718E-46	0.84854731	0.144	0.081	8.0555E-42
Nsmce4a	4.1492E-46	0.50731549	0.252	0.166	9.103E-42
Vps51	4.3613E-46	1.13968629	0.109	0.056	9.5683E-42
Zhx2	4.4001E-46	0.6011027	0.181	0.108	9.6534E-42
St6galnac2	4.5247E-46	2.16282035	0.02	0.004	9.9266E-42
Yaf2	4.655E-46	0.89885346	0.126	0.068	1.0213E-41
Sdf2	5.8757E-46	0.56864137	0.3	0.215	1.2891E-41
Atp5k	7.0311E-46	0.35779279	0.518	0.418	1.5425E-41
Pcif1	7.2293E-46	0.51845436	0.316	0.227	1.586E-41
Mrpl51	1.6824E-45	0.74111468	0.201	0.128	3.691E-41
Ccdc59	2.3278E-45	0.50254105	0.364	0.275	5.1069E-41
Edem1	2.4124E-45	0.44377754	0.366	0.275	5.2925E-41

Sntb1	2.9565E-45	0.91365733	0.082	0.037	6.4862E-41
Slco3a1	3.2561E-45	0.70872066	0.22	0.145	7.1435E-41
Rab8a	3.9818E-45	0.5191659	0.334	0.246	8.7357E-41
Atp6v1f	4.2385E-45	0.25327961	0.686	0.559	9.2988E-41
Ubap2	4.9302E-45	0.75488726	0.152	0.087	1.0816E-40
Ube2a	5.3932E-45	0.58323716	0.315	0.231	1.1832E-40
Gpbp1l1	5.7735E-45	0.51450194	0.271	0.184	1.2667E-40
Adh5	6.2006E-45	0.82292731	0.2	0.13	1.3604E-40
Crybg1	7.5958E-45	0.65522956	0.198	0.125	1.6664E-40
1700097N02R	1.0917E-44	1.59960536	0.039	0.012	2.3951E-40
Wrnip1	1.1106E-44	0.93713754	0.091	0.043	2.4365E-40
Rab21	1.1798E-44	0.36020335	0.404	0.3	2.5883E-40
Gprin3	1.2348E-44	1.01981061	0.06	0.024	2.7091E-40
Polr2d	1.3895E-44	1.07715754	0.098	0.049	3.0485E-40
Atp5f1	1.6875E-44	0.37750361	0.528	0.434	3.7023E-40
Ociad1	1.7925E-44	0.49101951	0.321	0.232	3.9326E-40
Slc24a5	2.2212E-44	1.71351311	0.043	0.015	4.873E-40
Aurkaip1	2.4149E-44	0.56497012	0.332	0.249	5.298E-40
Sap30	3.5259E-44	0.97821601	0.146	0.085	7.7355E-40
Smarcd1	4.3897E-44	0.89552578	0.126	0.069	9.6305E-40
Snrpb	4.7372E-44	0.35235906	0.508	0.411	1.0393E-39
Acadl	8.4099E-44	0.77696095	0.228	0.154	1.845E-39
Stat5a	8.8877E-44	0.55827083	0.262	0.179	1.9499E-39
Psmc5	1.1509E-43	0.72382106	0.24	0.166	2.525E-39
Chd4	1.4945E-43	0.29783229	0.548	0.444	3.2787E-39
Insl6	1.5308E-43	1.16767333	0.092	0.045	3.3584E-39
Sacs	1.6317E-43	0.76641347	0.11	0.056	3.5798E-39
Nxt1	1.6382E-43	0.97770499	0.118	0.063	3.5941E-39
Uba52	1.8296E-43	0.30941718	0.607	0.512	4.014E-39
Snrnp27	3.3616E-43	0.62009104	0.269	0.19	7.3751E-39
4930550C14R	3.4074E-43	3.23972857	0.012	0.001	7.4755E-39
Cdc34	3.8731E-43	0.60152521	0.231	0.154	8.4973E-39
Atxn7l3b	4.7193E-43	0.40731863	0.43	0.34	1.0354E-38
Cdc40	5.0466E-43	0.4406674	0.328	0.236	1.1072E-38
Cyld	5.2228E-43	0.36920692	0.408	0.309	1.1458E-38
Abhd2	5.5352E-43	0.60725747	0.198	0.124	1.2144E-38
Supt20	1.0444E-42	0.44506745	0.283	0.194	2.2912E-38
Abhd8	1.5687E-42	1.05867758	0.068	0.029	3.4415E-38
Hist2h2ac	1.872E-42	0.7880807	0.122	0.066	4.107E-38
Ttc39b	2.4171E-42	0.43879407	0.284	0.196	5.303E-38
Ufc1	2.7272E-42	0.57368573	0.312	0.233	5.9832E-38
Zfp362	3.9068E-42	1.13892077	0.074	0.033	8.5711E-38
Gcc2	4.8831E-42	0.60387723	0.19	0.118	1.0713E-37
Ndufv2	6.007E-42	0.64714456	0.264	0.188	1.3179E-37
Tmed9	6.0275E-42	0.39802359	0.431	0.34	1.3224E-37
Tmem9b	7.1456E-42	0.82301467	0.157	0.095	1.5677E-37
Vamp4	7.7592E-42	0.58781698	0.263	0.185	1.7023E-37
Tmem203	8.3056E-42	1.0313169	0.102	0.052	1.8222E-37

Hadhb	8.9466E-42	0.49279673	0.254	0.172	1.9628E-37
Fli1	1.4435E-41	0.41476026	0.345	0.254	3.1668E-37
Lsm1	1.5339E-41	0.86447392	0.131	0.074	3.3653E-37
Ankrd12	1.5721E-41	0.3334849	0.368	0.268	3.4491E-37
Erlec1	2.0583E-41	0.73731151	0.166	0.101	4.5158E-37
Dynll1	2.3211E-41	0.34404937	0.478	0.381	5.0923E-37
Wdyhv1	2.5301E-41	1.13459948	0.078	0.036	5.5507E-37
Med31	2.727E-41	0.75865055	0.136	0.078	5.9827E-37
Nmi	3.9524E-41	0.67523355	0.185	0.117	8.6711E-37
Atp6v0e	5.3601E-41	0.25348628	0.598	0.483	1.1759E-36
Mdh2	7.6435E-41	0.3824029	0.392	0.302	1.6769E-36
G2e3	8.4083E-41	0.84985756	0.105	0.054	1.8447E-36
Cdadc1	8.9564E-41	0.91707393	0.105	0.055	1.9649E-36
Trim59	1.272E-40	0.35244386	0.173	0.103	2.7906E-36
Fam169b	2.1349E-40	0.36057966	0.157	0.09	4.6837E-36
Msi2	2.2565E-40	0.34223155	0.311	0.216	4.9506E-36
Rnf10	2.4987E-40	0.54738986	0.292	0.213	5.4819E-36
Gm34794	3.0026E-40	2.53863064	0.014	0.002	6.5874E-36
Ulk1	3.2389E-40	0.63840083	0.164	0.099	7.1058E-36
4921524J17Ril	3.77E-40	0.59298587	0.167	0.101	8.2711E-36
Gm39323	4.2396E-40	2.21721932	0.02	0.004	9.3014E-36
Srek1ip1	4.9175E-40	1.03997863	0.09	0.045	1.0789E-35
Cdk2ap1	5.0081E-40	0.78316821	0.172	0.108	1.0987E-35
Srrm1	5.1042E-40	0.33923676	0.55	0.461	1.1198E-35
Atf6	6.8139E-40	0.70422494	0.245	0.173	1.4949E-35
Slfn1	7.4191E-40	0.25694853	0.123	0.067	1.6277E-35
Snrpc	8.3703E-40	0.52376448	0.305	0.226	1.8364E-35
Atp8a2	1.3201E-39	1.36615479	0.035	0.011	2.8962E-35
Zbtb1	1.3787E-39	0.51460974	0.206	0.132	3.0248E-35
Ip6k1	1.6732E-39	0.52209014	0.237	0.16	3.6708E-35
Dnajc9	1.7503E-39	0.47096831	0.179	0.11	3.8399E-35
Atp5b	1.9801E-39	0.27490168	0.674	0.576	4.3441E-35
Cdk12	2.4969E-39	0.44520962	0.36	0.274	5.4779E-35
Espn	3.1544E-39	1.1619283	0.051	0.02	6.9205E-35
Sbno1	5.0269E-39	0.37665514	0.437	0.347	1.1029E-34
Prpf18	6.2354E-39	0.5267973	0.202	0.131	1.368E-34
Plekha3	7.0445E-39	0.76223815	0.137	0.08	1.5455E-34
Ppp2ca	9.9675E-39	0.32256036	0.575	0.486	2.1868E-34
Ndufs3	1.4534E-38	0.70210446	0.22	0.151	3.1887E-34
Ubxn11	1.5211E-38	1.07389141	0.063	0.027	3.3372E-34
1110008F13Ri	2.1515E-38	0.56640379	0.277	0.203	4.7202E-34
Gpr107	2.1587E-38	0.70708757	0.158	0.097	4.7359E-34
Gm2245	2.2861E-38	1.44923788	0.039	0.013	5.0154E-34
Sp3	2.318E-38	0.4475372	0.282	0.2	5.0854E-34
2810001G20R	2.3395E-38	0.85856187	0.073	0.033	5.1327E-34
Hist3h2ba	3.1201E-38	1.17109406	0.049	0.019	6.8452E-34
1110004F10Ri	3.5135E-38	0.3865219	0.395	0.308	7.7083E-34
Aimp1	3.9503E-38	0.47379218	0.378	0.297	8.6667E-34

Cd84	3.9855E-38	0.48936378	0.28	0.199	8.7438E-34
Utp23	4.054E-38	0.8388447	0.115	0.064	8.894E-34
Hnrnpll	4.0984E-38	0.62746726	0.231	0.161	8.9915E-34
Ndufb10	4.631E-38	0.43401622	0.406	0.326	1.016E-33
Ogt	4.819E-38	0.27077176	0.413	0.309	1.0572E-33
1300002E11R	4.9374E-38	0.9026701	0.076	0.036	1.0832E-33
Xlr4c	5.4381E-38	1.24368054	0.05	0.019	1.1931E-33
Cbx3	6.7473E-38	0.33890292	0.371	0.277	1.4803E-33
Ctdspl2	7.0168E-38	0.5086947	0.201	0.131	1.5394E-33
Sar1a	9.5193E-38	0.37552618	0.42	0.336	2.0884E-33
Rp9	1.4921E-37	0.39785899	0.347	0.262	3.2734E-33
Ralbp1	1.6071E-37	0.44710886	0.307	0.226	3.5259E-33
Scyl2	1.6493E-37	0.73260258	0.15	0.091	3.6185E-33
Metap2	1.6696E-37	0.44984744	0.39	0.31	3.6629E-33
Akirin2	1.7447E-37	0.58898136	0.236	0.163	3.8277E-33
Dnajc19	1.759E-37	0.59977597	0.268	0.195	3.859E-33
Gm44851	2.2407E-37	1.96649684	0.022	0.006	4.9159E-33
H2-T22	2.8534E-37	0.46872083	0.312	0.23	6.2601E-33
Pced1b	3.0344E-37	0.94454736	0.059	0.025	6.6572E-33
Gm10036	4.3758E-37	0.43925983	0.361	0.277	9.6E-33
Mrpl58	4.4304E-37	0.81478241	0.177	0.115	9.72E-33
Cmas	4.8855E-37	0.53762673	0.243	0.17	1.0718E-32
Pink1	4.981E-37	0.72995412	0.125	0.071	1.0928E-32
Rbm33	5.4076E-37	0.40789175	0.281	0.198	1.1864E-32
Cdk6	5.5887E-37	0.60638617	0.187	0.122	1.2261E-32
Cnot7	6.0266E-37	0.56257911	0.203	0.135	1.3222E-32
Rex1bd	6.8838E-37	0.71675313	0.233	0.166	1.5102E-32
Aggf1	7.4212E-37	0.71084916	0.173	0.11	1.6281E-32
Hist1h4d	9.2138E-37	0.30710004	0.304	0.218	2.0214E-32
Zfp329	9.2531E-37	0.80406036	0.093	0.048	2.03E-32
Dpy19l3	9.4184E-37	1.00365422	0.069	0.032	2.0663E-32
Tcrg-C1	9.8857E-37	0.9293599	0.053	0.021	2.1688E-32
Mob2	9.8904E-37	0.55420472	0.181	0.115	2.1699E-32
Hdac1	9.993E-37	0.65729447	0.171	0.109	2.1924E-32
Rap1a	1.1754E-36	0.34010647	0.454	0.366	2.5788E-32
Rassf5	1.1837E-36	0.49622881	0.205	0.134	2.597E-32
Terf1	1.2724E-36	0.68665331	0.128	0.073	2.7914E-32
Hnrnpl	1.2906E-36	0.41465269	0.363	0.279	2.8314E-32
Calr	1.3193E-36	0.25184715	0.789	0.681	2.8944E-32
Ttc14	1.3199E-36	0.25641308	0.445	0.347	2.8958E-32
Gab3	1.4418E-36	1.35625978	0.041	0.015	3.1632E-32
Gm30054	2.1596E-36	0.81757511	0.063	0.028	4.7379E-32
Nab1	2.1713E-36	0.32643357	0.401	0.313	4.7636E-32
Nlk	2.5921E-36	0.77276349	0.11	0.06	5.6868E-32
Helz	3.2019E-36	0.5506917	0.189	0.122	7.0246E-32
Gimap9	3.367E-36	0.2905043	0.207	0.131	7.3869E-32
AC147041.1	3.5874E-36	1.84309718	0.022	0.006	7.8704E-32
Mrpl13	3.6978E-36	0.69777817	0.199	0.134	8.1125E-32

Rhof	3.7961E-36	0.69355564	0.146	0.089	8.3283E-32
Bsg	4.4178E-36	0.50603119	0.349	0.276	9.6922E-32
Ramp1	5.926E-36	1.05475492	0.131	0.079	1.3001E-31
Vdac2	6.3856E-36	0.40260665	0.396	0.316	1.4009E-31
Angel2	6.8727E-36	0.47830982	0.22	0.148	1.5078E-31
Cope	8.4168E-36	0.58068664	0.264	0.195	1.8466E-31
Krt18	9.6989E-36	2.18884782	0.015	0.003	2.1278E-31
Slc4a7	1.246E-35	0.36460137	0.342	0.257	2.7337E-31
Taf6l	1.4531E-35	0.55678937	0.257	0.185	3.1879E-31
Ccdc127	1.6727E-35	0.71209408	0.141	0.085	3.6697E-31
Rhno1	2.4219E-35	0.78855203	0.086	0.044	5.3135E-31
Emc4	2.5639E-35	0.90734358	0.141	0.087	5.6249E-31
Timm13	2.7121E-35	0.3906277	0.415	0.336	5.9501E-31
Ing1	2.7486E-35	0.5910616	0.199	0.132	6.0302E-31
Hivep2	2.8965E-35	0.33546389	0.39	0.3	6.3546E-31
Pde3b	3.4206E-35	0.25872872	0.355	0.262	7.5044E-31
Unc45a	4.0115E-35	0.68801615	0.13	0.076	8.8009E-31
Cmc1	4.7873E-35	0.66103551	0.225	0.159	1.0503E-30
Ddrgk1	5.4232E-35	0.51307366	0.294	0.221	1.1898E-30
Siva1	5.5257E-35	0.76379758	0.201	0.139	1.2123E-30
Cd48	6.5182E-35	0.75319448	0.197	0.135	1.43E-30
Rnf181	7.6758E-35	0.69308265	0.129	0.076	1.684E-30
Ppil1	8.5564E-35	0.9425806	0.076	0.037	1.8772E-30
R3hdm4	9.4639E-35	0.59037211	0.203	0.138	2.0763E-30
Lman2	1.3991E-34	0.47176047	0.35	0.276	3.0694E-30
Tmem248	1.6657E-34	0.62028819	0.226	0.16	3.6545E-30
Slc52a3	1.7334E-34	1.39717233	0.036	0.013	3.8029E-30
Magoh	1.8842E-34	0.45646955	0.312	0.236	4.1337E-30
Cdk17	1.9002E-34	0.36281042	0.209	0.137	4.1689E-30
Relb	2.2819E-34	0.38771816	0.362	0.282	5.0062E-30
Cetn2	2.2869E-34	0.51924616	0.23	0.16	5.0171E-30
Nt5c	2.5114E-34	0.6225423	0.24	0.173	5.5097E-30
Upf3a	2.9845E-34	0.73498246	0.187	0.126	6.5477E-30
Tmem128	3.1537E-34	0.5091627	0.303	0.232	6.9189E-30
Ccdc25	3.3802E-34	0.67261232	0.157	0.099	7.4158E-30
Cited2	4.1989E-34	0.27733621	0.234	0.158	9.2119E-30
Denr	4.5865E-34	0.38977594	0.318	0.24	1.0062E-29
Fundc2	5.0634E-34	0.51179703	0.283	0.21	1.1109E-29
Uqcrc2	5.2272E-34	0.57158135	0.274	0.205	1.1468E-29
Srp72	6.4261E-34	0.46105276	0.336	0.262	1.4098E-29
2310033P09R	6.571E-34	0.70766947	0.142	0.087	1.4416E-29
Apoa2	8.8145E-34	2.59546243	0.014	0.003	1.9338E-29
Tgfb1	9.2137E-34	0.3559342	0.544	0.454	2.0214E-29
Acap1	9.5567E-34	0.48795785	0.158	0.098	2.0966E-29
Mknk2	1.2659E-33	0.29100504	0.356	0.271	2.7772E-29
Armc1	1.5076E-33	0.72279752	0.119	0.069	3.3075E-29
Lsm10	1.6329E-33	1.06604432	0.102	0.058	3.5825E-29
Hprt	1.8504E-33	0.51056457	0.279	0.209	4.0595E-29

Tmed10	1.9486E-33	0.28756706	0.546	0.457	4.2751E-29
Trappc4	2.0025E-33	0.57402128	0.249	0.183	4.3933E-29
Rrp1	2.4936E-33	0.47615199	0.318	0.246	5.4706E-29
Anp32e	2.5372E-33	0.33060922	0.38	0.295	5.5664E-29
Klrb1f	2.5617E-33	1.18682674	0.038	0.014	5.6201E-29
Gm13546	3.4163E-33	1.95169685	0.03	0.01	7.495E-29
Helb	4.3061E-33	0.68688109	0.1	0.055	9.4471E-29
Nsmce3	4.4813E-33	0.85336702	0.12	0.071	9.8315E-29
Sept9	5.4197E-33	0.53877729	0.229	0.162	1.189E-28
Gpr174	6.0083E-33	0.37980206	0.143	0.085	1.3182E-28
Ice1	6.128E-33	0.7700456	0.119	0.07	1.3444E-28
Ran	6.4652E-33	0.27442172	0.508	0.42	1.4184E-28
Cbx4	8.1278E-33	0.44331004	0.179	0.115	1.7831E-28
Grap	8.5994E-33	0.34431015	0.196	0.127	1.8866E-28
Akna	1.074E-32	0.34954761	0.29	0.211	2.3562E-28
Elavl1	1.2668E-32	0.45421692	0.298	0.224	2.7793E-28
Guk1	1.5888E-32	0.72326756	0.151	0.096	3.4856E-28
2010111I01Ril	2.3362E-32	0.63388143	0.154	0.098	5.1254E-28
Rab35	2.8416E-32	0.68043631	0.176	0.117	6.2341E-28
Cab39	3.2399E-32	0.33361479	0.363	0.281	7.1079E-28
Yipf4	3.3247E-32	0.57611846	0.231	0.165	7.2941E-28
Gm32036	4.0276E-32	1.24276762	0.038	0.014	8.8362E-28
Glrx5	4.1652E-32	0.66587046	0.187	0.127	9.138E-28
Cdipt	5.1109E-32	0.54737901	0.183	0.122	1.1213E-27
Mrpl40	5.1656E-32	0.85717583	0.109	0.063	1.1333E-27
Tpd52l2	5.5103E-32	0.60391253	0.205	0.143	1.2089E-27
Igf2r	5.8085E-32	0.45992068	0.166	0.106	1.2743E-27
Drap1	7.1754E-32	0.38804592	0.394	0.321	1.5742E-27
Ccdc174	8.8639E-32	0.49818576	0.249	0.18	1.9447E-27
Pcmtd2	9.5331E-32	0.45556884	0.152	0.094	2.0915E-27
Ccm2	1.002E-31	0.48891644	0.186	0.123	2.1983E-27
Sdhb	1.0341E-31	0.61152657	0.242	0.18	2.2688E-27
Igf2bp3	1.2063E-31	1.4729974	0.036	0.013	2.6466E-27
Pdcl3	1.2691E-31	0.7410543	0.173	0.117	2.7844E-27
Asf1a	1.3797E-31	0.82333249	0.106	0.061	3.0269E-27
Rock1	1.3879E-31	0.27451075	0.475	0.393	3.0449E-27
Ube2m	1.457E-31	0.60447913	0.247	0.183	3.1965E-27
4930453N24R	1.7058E-31	0.67274641	0.146	0.092	3.7423E-27
Vps37a	1.7799E-31	0.62266879	0.17	0.113	3.9048E-27
Casp8ap2	1.8521E-31	0.42456202	0.211	0.144	4.0634E-27
Dnajc1	1.9419E-31	0.48071331	0.265	0.195	4.2603E-27
Mrpl4	2.0035E-31	0.75150647	0.16	0.105	4.3955E-27
Ubac1	2.3147E-31	1.09616554	0.066	0.032	5.0783E-27
Mrpl32	2.3708E-31	0.47987079	0.294	0.225	5.2013E-27
Rabgap1l	2.4295E-31	0.27451571	0.26	0.181	5.33E-27
Iqcc	4.187E-31	1.29437286	0.043	0.018	9.1859E-27
Pot1a	4.4869E-31	0.75262771	0.076	0.038	9.8438E-27
Ccnc	5.425E-31	0.77133128	0.101	0.057	1.1902E-26

Cfap36	5.5253E-31	0.69663868	0.136	0.085	1.2122E-26
Virma	5.5393E-31	0.57040387	0.173	0.114	1.2153E-26
Rgs11	6.8251E-31	2.41507744	0.012	0.002	1.4974E-26
Psmd8	7.4905E-31	0.40565192	0.359	0.288	1.6433E-26
Fbxl12	8.552E-31	0.41713389	0.177	0.115	1.8762E-26
H2afx	8.6561E-31	0.64224344	0.121	0.072	1.8991E-26
Dctn6	1.1735E-30	0.73131519	0.113	0.067	2.5745E-26
Rabif	1.2423E-30	0.83088006	0.087	0.047	2.7254E-26
Zfp830	1.2511E-30	0.69120793	0.135	0.084	2.7447E-26
Ube2o	1.5969E-30	0.94683628	0.077	0.04	3.5035E-26
Slc25a24	1.7849E-30	0.8008089	0.108	0.063	3.9159E-26
Lias	1.9374E-30	0.7403388	0.102	0.058	4.2504E-26
Fam118a	2.7979E-30	0.80397423	0.064	0.031	6.1383E-26
A630023P12R	2.8282E-30	1.12527871	0.033	0.012	6.2049E-26
Tmx4	2.9487E-30	0.46277155	0.187	0.126	6.4691E-26
Ranbp6	3.4441E-30	1.07934315	0.055	0.025	7.5561E-26
Ccdc124	4.251E-30	0.64920401	0.203	0.145	9.3262E-26
Cnn2	4.2643E-30	0.35220694	0.345	0.269	9.3554E-26
Abracl	4.6561E-30	0.263755	0.564	0.482	1.0215E-25
Mlec	4.9006E-30	0.56849421	0.201	0.141	1.0752E-25
Rsl1d1	4.946E-30	0.48132276	0.268	0.201	1.0851E-25
Pcnp	5.002E-30	0.4190902	0.31	0.239	1.0974E-25
Imp3	5.0403E-30	0.5001663	0.243	0.177	1.1058E-25
Tomm5	6.4905E-30	0.55125436	0.262	0.196	1.4239E-25
Irf2bp1	6.5462E-30	0.87393986	0.064	0.031	1.4362E-25
Susd2	7.6881E-30	1.93104801	0.018	0.004	1.6867E-25
Eif4ebp2	8.0729E-30	0.38930229	0.24	0.172	1.7711E-25
Prdx2	8.1576E-30	0.46261613	0.353	0.285	1.7897E-25
Gm14718	8.2232E-30	0.85832893	0.034	0.013	1.8041E-25
Hpf1	8.7892E-30	0.45865002	0.251	0.184	1.9283E-25
Tspyl4	1.5366E-29	1.32111856	0.032	0.011	3.3711E-25
Pofut2	1.6322E-29	0.53616483	0.251	0.188	3.5809E-25
Med21	1.7335E-29	0.60132252	0.163	0.108	3.8032E-25
Il17rc	1.8934E-29	2.64653021	0.011	0.002	4.1539E-25
Zfp382	2.2564E-29	1.32671077	0.038	0.015	4.9503E-25
Ppil4	2.2984E-29	0.44317371	0.216	0.152	5.0425E-25
Snrnp40	2.4184E-29	0.68664131	0.146	0.094	5.3058E-25
Mff	2.7034E-29	0.53121209	0.231	0.17	5.9309E-25
Psma1	2.7251E-29	0.40715825	0.318	0.249	5.9786E-25
Golm1	2.9467E-29	0.93106613	0.065	0.032	6.4649E-25
Ndufs6	3.2821E-29	0.43041687	0.335	0.268	7.2005E-25
Prorsd1	3.3478E-29	0.55132327	0.142	0.09	7.3448E-25
Zfp941	3.5969E-29	2.49466132	0.012	0.002	7.8913E-25
Rchy1	3.8418E-29	0.41362359	0.258	0.19	8.4284E-25
Ccni	4.4602E-29	0.34082971	0.316	0.243	9.7852E-25
Crebzf	4.5074E-29	0.54969066	0.165	0.109	9.8888E-25
Ubxn4	4.5775E-29	0.31742821	0.453	0.379	1.0043E-24
Anxa6	4.7614E-29	0.38427171	0.322	0.25	1.0446E-24

Donson	4.9231E-29	0.61614029	0.128	0.079	1.0801E-24
Mpp7	5.3369E-29	0.6548429	0.158	0.105	1.1709E-24
Alg5	5.8583E-29	0.80555001	0.127	0.08	1.2852E-24
Lockd	6.6702E-29	0.71348301	0.074	0.038	1.4634E-24
Phf5a	6.9098E-29	0.46339746	0.28	0.215	1.5159E-24
Rab19	7.5177E-29	0.91352794	0.064	0.032	1.6493E-24
Gm35082	1.1549E-28	0.82873623	0.078	0.041	2.5337E-24
Psip1	1.1908E-28	0.39326034	0.205	0.141	2.6124E-24
Ppp1r35	1.2625E-28	0.95330837	0.075	0.039	2.7699E-24
1700123O20R	1.3815E-28	0.67565917	0.137	0.088	3.0309E-24
Ndufb5	1.4257E-28	0.35091195	0.424	0.356	3.1278E-24
Ndufb4	1.5279E-28	0.51218695	0.288	0.226	3.352E-24
Casc1	1.9719E-28	2.20573901	0.011	0.002	4.3262E-24
Atf1	2.0257E-28	0.47977486	0.219	0.158	4.4441E-24
Setd3	2.7836E-28	0.53576141	0.195	0.136	6.107E-24
Yipf3	2.8621E-28	0.53221651	0.212	0.152	6.2791E-24
Syt11	2.8807E-28	0.83695601	0.063	0.031	6.32E-24
Dnaja4	2.9278E-28	0.48610939	0.072	0.037	6.4234E-24
Slc35b1	2.9596E-28	0.74965187	0.177	0.124	6.493E-24
Abtb1	3.1313E-28	0.69584312	0.084	0.045	6.8697E-24
Usp1	3.3492E-28	0.50014676	0.179	0.121	7.3478E-24
Mical1	3.381E-28	0.5866938	0.183	0.127	7.4176E-24
Ncaph	3.5082E-28	1.41801582	0.037	0.015	7.6966E-24
Gtf2h5	3.6956E-28	0.37951168	0.324	0.255	8.1079E-24
Stx6	3.7602E-28	0.46249695	0.177	0.12	8.2495E-24
Ckap5	3.9722E-28	0.65936154	0.132	0.084	8.7147E-24
Pdia6	4.5743E-28	0.30660923	0.436	0.358	1.0036E-23
Nemp2	4.6796E-28	0.73593406	0.074	0.039	1.0267E-23
Iars2	5.4091E-28	0.77037315	0.112	0.068	1.1867E-23
Bzw1	5.5352E-28	0.26488416	0.503	0.43	1.2144E-23
Terf2ip	5.9411E-28	0.8108118	0.069	0.036	1.3034E-23
Baiap2	5.9862E-28	0.5321859	0.194	0.136	1.3133E-23
Use1	6.0036E-28	0.28276757	0.509	0.436	1.3171E-23
Ocel1	6.0814E-28	1.00491522	0.077	0.042	1.3342E-23
Insig2	6.5508E-28	0.81057617	0.09	0.051	1.4372E-23
H2-Q4	9.3359E-28	0.30207554	0.329	0.251	2.0482E-23
Gpatch8	1.1307E-27	0.35199686	0.3	0.229	2.4806E-23
Cyhr1	1.1969E-27	0.70427538	0.114	0.07	2.626E-23
Eapp	1.3215E-27	0.35755875	0.297	0.229	2.8992E-23
Tmem110	1.3265E-27	1.28522821	0.049	0.023	2.9101E-23
3830406C13R	1.3807E-27	0.7031032	0.132	0.084	3.0292E-23
Pgls	1.5136E-27	0.37696278	0.375	0.307	3.3206E-23
Tnks2	1.6783E-27	0.31225007	0.331	0.258	3.6821E-23
Ssr1	1.8648E-27	0.39996776	0.318	0.253	4.0913E-23
Yy1	1.936E-27	0.31121967	0.391	0.317	4.2474E-23
Ube4b	2.0171E-27	0.71588461	0.11	0.066	4.4253E-23
C1qtnf4	2.9052E-27	1.50783706	0.024	0.008	6.3737E-23
Cdkn2aip	3.0358E-27	0.52304438	0.128	0.079	6.6602E-23

Pdcd7	3.0424E-27	0.75635231	0.091	0.052	6.6748E-23
Kat6b	3.3285E-27	0.50442735	0.158	0.104	7.3024E-23
Cep192	3.5743E-27	0.48316376	0.121	0.074	7.8417E-23
Il16	3.6085E-27	0.37068726	0.157	0.102	7.9166E-23
1110038F14Ri	3.9222E-27	0.59538652	0.154	0.102	8.6048E-23
Blm	4.3898E-27	0.93247997	0.069	0.036	9.6308E-23
Trnt1	4.475E-27	0.54230123	0.208	0.15	9.8177E-23
2510002D24R	4.5606E-27	0.54840743	0.123	0.075	1.0005E-22
Crnkl1	4.7548E-27	0.56424002	0.177	0.122	1.0432E-22
Fam216a	5.016E-27	0.91581789	0.059	0.029	1.1005E-22
Runx2os1	5.0874E-27	1.3561899	0.04	0.017	1.1161E-22
Frmd8	5.7473E-27	0.92267071	0.06	0.03	1.2609E-22
Faim	6.0627E-27	0.38274413	0.177	0.12	1.3301E-22
Raly	6.2302E-27	0.30697128	0.41	0.339	1.3668E-22
Ptprk	6.4388E-27	1.58007604	0.015	0.004	1.4126E-22
Abcf1	7.3247E-27	0.42747222	0.31	0.246	1.607E-22
Nsd2	7.799E-27	0.62337466	0.167	0.114	1.711E-22
0610010K14R	7.8367E-27	0.9705635	0.082	0.046	1.7193E-22
Flt3l	8.2697E-27	0.77082189	0.076	0.04	1.8143E-22
Fbxw2	8.6792E-27	0.27226703	0.291	0.22	1.9041E-22
Txndc11	8.7319E-27	0.4589729	0.197	0.138	1.9157E-22
Pigx	8.8375E-27	0.81724733	0.118	0.074	1.9389E-22
Arl6ip5	9.1453E-27	0.41177569	0.275	0.21	2.0064E-22
Tfrc	1.3309E-26	0.39194081	0.148	0.097	2.9199E-22
Fam114a2	1.6027E-26	0.65115675	0.128	0.081	3.5162E-22
Ncbp1	1.6048E-26	0.60773958	0.119	0.074	3.5208E-22
Hjurp	1.9605E-26	0.61207679	0.128	0.081	4.3011E-22
Ssr2	2.131E-26	0.59891771	0.225	0.17	4.6753E-22
Hectd1	2.24E-26	0.25787921	0.385	0.31	4.9144E-22
Cebpzos	2.2646E-26	0.86966262	0.092	0.053	4.9683E-22
Phtf1	2.425E-26	0.77933137	0.072	0.038	5.3202E-22
Coa3	2.6104E-26	0.6837569	0.171	0.12	5.7269E-22
Chrne	2.9577E-26	0.98693092	0.053	0.026	6.4889E-22
Tspyl1	3.2797E-26	0.50134544	0.176	0.121	7.1953E-22
Cdc25b	3.2969E-26	0.8923407	0.051	0.024	7.233E-22
Zfp664	3.7032E-26	0.69492213	0.103	0.061	8.1244E-22
Nras	4.9941E-26	0.41668526	0.273	0.211	1.0956E-21
Ski	5.031E-26	0.37904162	0.223	0.161	1.1037E-21
Ankib1	5.0551E-26	0.55019436	0.142	0.093	1.109E-21
Ing2	6.3742E-26	0.47226272	0.203	0.146	1.3984E-21
Prkcz	6.4365E-26	0.85234905	0.051	0.024	1.4121E-21
Bag1	6.7918E-26	0.34717034	0.348	0.284	1.4901E-21
Ttc5	7.3367E-26	0.59891602	0.106	0.064	1.6096E-21
Timm17b	7.5763E-26	0.67069141	0.165	0.115	1.6622E-21
Vamp2	7.6441E-26	0.41088135	0.2	0.142	1.677E-21
Acbd6	8.8046E-26	0.60073834	0.178	0.126	1.9316E-21
Ahi1	9.2891E-26	0.48932427	0.121	0.075	2.0379E-21
Snw1	9.4189E-26	0.29387355	0.385	0.313	2.0664E-21

Zbtb42	1.0209E-25	1.4498906	0.04	0.017	2.2397E-21
Pfdn1	1.0284E-25	0.61458276	0.196	0.143	2.2561E-21
Mbp	1.3879E-25	0.44772595	0.208	0.149	3.0449E-21
Tmem71	1.4555E-25	0.43772093	0.11	0.066	3.1933E-21
Sec22b	1.5151E-25	0.47962867	0.186	0.132	3.324E-21
Ngdn	1.7493E-25	0.54893215	0.205	0.15	3.8377E-21
Alkbh7	1.862E-25	1.03244999	0.07	0.038	4.0851E-21
Idnk	2.0632E-25	0.42475306	0.234	0.173	4.5265E-21
Bod1l	2.0982E-25	0.29481944	0.283	0.213	4.6033E-21
9130401M01F	2.191E-25	0.79357414	0.113	0.071	4.8069E-21
Mrps12	2.2527E-25	0.66447442	0.154	0.105	4.9422E-21
Cpped1	2.4574E-25	1.37152672	0.035	0.014	5.3912E-21
Mgat2	2.464E-25	0.63037024	0.165	0.115	5.4058E-21
Ubxn2a	2.6583E-25	0.37115333	0.242	0.179	5.832E-21
Sae1	2.7439E-25	0.61604581	0.104	0.063	6.0198E-21
Dusp12	2.8881E-25	1.04523167	0.054	0.026	6.3362E-21
Pdk1	3.3094E-25	0.66075126	0.08	0.045	7.2606E-21
Cdc42se1	3.5205E-25	0.38020764	0.255	0.193	7.7236E-21
Gpr171	3.5622E-25	0.33012319	0.304	0.234	7.815E-21
Taf11	3.6146E-25	0.6918249	0.106	0.065	7.9301E-21
Cep57	4.4718E-25	0.52638539	0.141	0.092	9.8106E-21
Trp53i13	4.9678E-25	1.19061023	0.044	0.02	1.0899E-20
Usf1	5.7638E-25	0.7293823	0.094	0.056	1.2645E-20
Rif1	6.0343E-25	0.29230068	0.189	0.13	1.3239E-20
Ube2d2a	6.5368E-25	0.27389338	0.427	0.362	1.4341E-20
Cox11	7.1861E-25	1.00220727	0.058	0.029	1.5766E-20
Incenp	7.2413E-25	0.844331	0.067	0.035	1.5887E-20
Cebpg	8.0993E-25	0.43204245	0.239	0.18	1.7769E-20
Desi1	9.0466E-25	0.80497563	0.102	0.063	1.9847E-20
Cdkn2c	9.0913E-25	1.29306369	0.039	0.017	1.9945E-20
Kxd1	9.6355E-25	0.54592569	0.225	0.171	2.1139E-20
Lamtor5	9.7049E-25	0.52690664	0.225	0.17	2.1292E-20
Emc7	1.1487E-24	0.38098274	0.334	0.274	2.5201E-20
Mrps14	1.3454E-24	0.42463725	0.312	0.253	2.9518E-20
3110040N11R	1.5715E-24	1.02556559	0.056	0.028	3.4476E-20
Yeats4	1.93E-24	0.38676504	0.271	0.21	4.2342E-20
Ddx50	1.9636E-24	0.25967977	0.379	0.306	4.3079E-20
Lonp2	2.0254E-24	0.65327429	0.108	0.067	4.4435E-20
Bud31	2.1013E-24	0.41360283	0.271	0.212	4.61E-20
Tbc1d9b	2.2558E-24	0.91756419	0.079	0.045	4.9491E-20
Mcm4	2.6578E-24	0.58917656	0.141	0.094	5.831E-20
Impa1	3.0403E-24	0.53838083	0.162	0.112	6.67E-20
Jmjd6	3.107E-24	0.59262914	0.124	0.08	6.8165E-20
Tufm	3.4168E-24	0.84065278	0.088	0.052	7.4962E-20
L1cam	3.5258E-24	0.90895593	0.049	0.024	7.7352E-20
Emc8	4.0334E-24	0.86857036	0.082	0.048	8.8488E-20
Etaa1os	4.7419E-24	1.45782397	0.022	0.007	1.0403E-19
Trbv13-2	5.5401E-24	2.01526582	0.011	0.002	1.2154E-19

Atp2a3	5.681E-24	0.35537384	0.145	0.095	1.2464E-19
Fopnl	6.57E-24	0.50832539	0.12	0.076	1.4414E-19
Med4	6.9149E-24	0.49999363	0.144	0.096	1.5171E-19
Nxpe3	8.068E-24	0.72565926	0.052	0.025	1.77E-19
Smim10l1	8.2259E-24	0.66333254	0.128	0.085	1.8047E-19
Mad2l1bp	9.6284E-24	0.82817977	0.073	0.04	2.1124E-19
Dhx8	9.9344E-24	0.44659792	0.185	0.132	2.1795E-19
Hmgb3	1.1973E-23	0.91381368	0.048	0.023	2.6268E-19
Aamp	1.2479E-23	0.33309283	0.345	0.284	2.7377E-19
Ssbp4	1.3858E-23	0.65051175	0.158	0.11	3.0403E-19
Lzts1	1.4165E-23	1.57004243	0.024	0.008	3.1077E-19
Pcsk7	1.729E-23	0.58977989	0.102	0.062	3.7933E-19
Mpc2	1.7378E-23	0.38619841	0.307	0.249	3.8126E-19
Afg3l1	1.9421E-23	0.66007197	0.133	0.089	4.2607E-19
Ankmy1	1.9644E-23	1.78123724	0.015	0.004	4.3098E-19
Cops4	2.0274E-23	0.46322348	0.197	0.144	4.4479E-19
Mosmo	2.0899E-23	0.57738986	0.106	0.066	4.5851E-19
Myef2	2.4403E-23	0.57397107	0.126	0.082	5.3537E-19
Ufl1	2.5392E-23	0.71564843	0.11	0.069	5.5707E-19
Usp48	2.7507E-23	0.53592425	0.138	0.092	6.0347E-19
Smim26	3.112E-23	0.73627648	0.108	0.068	6.8275E-19
Trbv15	3.3927E-23	2.03192276	0.011	0.002	7.4432E-19
Gatad1	3.5679E-23	0.53919845	0.16	0.112	7.8277E-19
Rap2c	4.0812E-23	0.39770431	0.209	0.154	8.9537E-19
Ier3ip1	4.1991E-23	0.32580124	0.357	0.297	9.2125E-19
Uqcrc1	4.2481E-23	0.54441453	0.247	0.195	9.32E-19
March5	4.6433E-23	0.47862294	0.231	0.177	1.0187E-18
Gtf3c1	5.3641E-23	0.58470826	0.104	0.065	1.1768E-18
Abhd17a	6.4154E-23	0.59792577	0.162	0.114	1.4075E-18
Gmps	7.0248E-23	0.51583049	0.186	0.135	1.5412E-18
Ppm1d	7.5887E-23	0.77547896	0.087	0.052	1.6649E-18
Isg20	7.6072E-23	0.29508299	0.094	0.056	1.669E-18
Ppp2r1b	7.965E-23	0.99087848	0.057	0.029	1.7474E-18
Gm11808	8.3809E-23	0.32888883	0.324	0.261	1.8387E-18
Tnfaip8l1	8.832E-23	1.38235212	0.034	0.015	1.9377E-18
Rbm42	9.0248E-23	0.39194778	0.252	0.195	1.98E-18
1110059G10R	9.0739E-23	0.6151751	0.136	0.091	1.9907E-18
Gldc	9.2362E-23	1.58188357	0.019	0.006	2.0263E-18
Actr10	9.4589E-23	0.5567929	0.175	0.126	2.0752E-18
Dtd1	1.015E-22	0.86822123	0.076	0.043	2.2267E-18
Gatsl3	1.043E-22	0.91503957	0.062	0.033	2.2883E-18
Armcx5	1.3661E-22	0.68115107	0.091	0.055	2.9971E-18
Capza1	1.5009E-22	0.28455905	0.403	0.342	3.2929E-18
Hnrnpul1	1.6486E-22	0.29160199	0.351	0.288	3.6169E-18
Gm19557	1.6549E-22	1.25410593	0.028	0.011	3.6306E-18
Stag2	1.6559E-22	0.30347851	0.306	0.243	3.633E-18
Zcchc9	1.7705E-22	0.51913127	0.22	0.168	3.8842E-18
Mkrn2	1.8964E-22	0.7443262	0.077	0.044	4.1605E-18

Rbm24	1.9851E-22	1.77124956	0.017	0.005	4.3552E-18
Edaradd	2.0762E-22	1.21159297	0.021	0.007	4.5551E-18
Fam3c	2.193E-22	0.46064683	0.12	0.077	4.8112E-18
Zfp560	2.2921E-22	0.58727084	0.111	0.07	5.0286E-18
Cstad	2.5264E-22	1.57729615	0.019	0.006	5.5426E-18
E4f1	2.6021E-22	0.52598605	0.119	0.076	5.7087E-18
Mrps30	2.6237E-22	0.67625944	0.117	0.076	5.7561E-18
Phc3	3.1909E-22	0.3075176	0.193	0.136	7.0006E-18
Uqcrb	3.441E-22	0.27864679	0.436	0.372	7.5492E-18
Epsti1	3.5031E-22	0.29269518	0.37	0.307	7.6855E-18
Polr3k	4.1321E-22	0.71736364	0.1	0.062	9.0653E-18
Mrpl54	4.2015E-22	0.366622	0.359	0.302	9.2178E-18
Wbp11	4.2056E-22	0.2580482	0.376	0.31	9.2266E-18
Lsm3	4.4144E-22	0.43105956	0.208	0.155	9.6847E-18
Gm26699	4.8806E-22	0.77607174	0.076	0.044	1.0707E-17
Znhit2	5.8547E-22	1.11254486	0.054	0.028	1.2845E-17
Ing5	5.9035E-22	0.48567151	0.166	0.117	1.2952E-17
Pgrmc2	6.0082E-22	0.578506	0.119	0.078	1.3181E-17
Wdr45	6.5105E-22	0.73573992	0.069	0.039	1.4283E-17
Stim1	7.5304E-22	0.30973364	0.2	0.143	1.6521E-17
Cfap20	7.7587E-22	0.6418143	0.114	0.074	1.7022E-17
Cdr2	8.4952E-22	0.90247066	0.056	0.029	1.8638E-17
Cxxc1	8.8843E-22	0.62350918	0.102	0.064	1.9491E-17
Ndufaf8	9.4249E-22	0.4190655	0.241	0.186	2.0677E-17
Ate1	9.6851E-22	0.61889549	0.122	0.08	2.1248E-17
Zrsr2	9.8912E-22	0.37438272	0.29	0.232	2.17E-17
Znrd1	9.9916E-22	0.34914418	0.244	0.188	2.1921E-17
Vps26b	1.0204E-21	0.80108213	0.072	0.041	2.2386E-17
Acp1	1.1974E-21	0.58450191	0.181	0.134	2.6271E-17
Suds3	1.2394E-21	0.47465841	0.158	0.111	2.7191E-17
Ric1	1.2469E-21	0.31679317	0.28	0.221	2.7355E-17
Ehd3	1.3188E-21	0.65898564	0.057	0.03	2.8933E-17
Ube2l3	1.3909E-21	0.2813914	0.424	0.365	3.0515E-17
Tstd3	1.4008E-21	0.84954752	0.057	0.03	3.0732E-17
Bet1l	1.4888E-21	0.54955051	0.155	0.109	3.2664E-17
Zbtb45	1.4937E-21	1.3384929	0.027	0.011	3.2771E-17
Dcaf12	1.5356E-21	0.50962451	0.173	0.125	3.3689E-17
Mynn	1.6049E-21	0.54279207	0.113	0.073	3.521E-17
Snf8	1.7073E-21	0.48414965	0.23	0.18	3.7456E-17
Psmc4	1.7367E-21	0.65419483	0.17	0.125	3.8102E-17
Pms2	2.1634E-21	0.73814516	0.059	0.032	4.7464E-17
Erich1	2.2615E-21	0.74791417	0.088	0.053	4.9615E-17
Dpm3	2.5327E-21	0.36120553	0.305	0.247	5.5565E-17
Plpp6	2.6342E-21	0.90582222	0.051	0.026	5.7792E-17
Ssbp3	2.7501E-21	0.36860705	0.156	0.108	6.0335E-17
Kctd2	2.7794E-21	0.7980129	0.067	0.037	6.0976E-17
Adipor2	2.9703E-21	0.32686624	0.255	0.197	6.5166E-17
Rexo2	2.9889E-21	0.47793264	0.254	0.202	6.5574E-17

Mllt3	3.1772E-21	0.32030026	0.16	0.11	6.9706E-17
Nrbf2	3.5008E-21	0.57249062	0.149	0.104	7.6805E-17
Grhpr	3.8368E-21	1.14226362	0.041	0.019	8.4175E-17
Pank2	3.866E-21	0.52597286	0.181	0.133	8.4816E-17
Snrpa1	3.88E-21	0.47681531	0.185	0.136	8.5123E-17
Zc3h14	3.9248E-21	0.45428951	0.163	0.115	8.6107E-17
Zfp91	4.085E-21	0.27202793	0.436	0.376	8.9621E-17
Actl6a	4.2174E-21	0.49590175	0.151	0.106	9.2525E-17
Ebp	4.2581E-21	0.68358682	0.157	0.113	9.3419E-17
Zfp322a	5.2094E-21	0.58996232	0.104	0.066	1.1429E-16
Pfdn6	5.8411E-21	0.5152039	0.2	0.151	1.2815E-16
Micu2	6.002E-21	0.64131774	0.117	0.077	1.3168E-16
Mrps26	7.6551E-21	0.54069095	0.134	0.091	1.6794E-16
Arrb1	8.1537E-21	0.6973769	0.118	0.079	1.7888E-16
Med8	9.0545E-21	0.71976711	0.123	0.084	1.9865E-16
Uspl1	9.5604E-21	0.48249825	0.129	0.086	2.0975E-16
Brd3	1.1084E-20	0.4399776	0.126	0.084	2.4318E-16
Lyrm9	1.2397E-20	0.88331417	0.051	0.027	2.7198E-16
Gsk3b	1.2748E-20	0.2630734	0.308	0.245	2.7967E-16
Coa5	1.291E-20	0.46897704	0.203	0.154	2.8324E-16
Tsr3	1.3765E-20	0.87506774	0.065	0.037	3.0199E-16
Ccng1	1.5672E-20	0.46514449	0.163	0.116	3.4383E-16
Npat	1.7151E-20	0.58447246	0.084	0.05	3.7629E-16
Fnta	1.7263E-20	0.64382163	0.077	0.046	3.7874E-16
Stambp	1.8699E-20	0.89709746	0.073	0.043	4.1024E-16
Ebna1bp2	1.8973E-20	0.49310113	0.182	0.134	4.1624E-16
Agfg1	1.961E-20	0.29394036	0.24	0.183	4.3023E-16
Zfp553	1.9841E-20	0.79611467	0.045	0.022	4.353E-16
Ubr2	2.1265E-20	0.2978789	0.222	0.167	4.6653E-16
Ndufb3	2.2805E-20	0.33081882	0.328	0.274	5.0032E-16
2310035C23R	2.3836E-20	0.37530032	0.179	0.129	5.2295E-16
Traf3ip3	2.4433E-20	0.4234508	0.107	0.068	5.3604E-16
Trappc6b	2.8732E-20	0.41205469	0.24	0.188	6.3035E-16
Copb2	3.4491E-20	0.43736999	0.241	0.191	7.5669E-16
Azi2	3.4705E-20	0.38217165	0.229	0.175	7.614E-16
Hdac8	3.5825E-20	0.94417884	0.039	0.018	7.8596E-16
Ubtd2	3.7619E-20	1.19057451	0.032	0.014	8.2533E-16
Cnnm3	4.5882E-20	0.73582023	0.056	0.03	1.0066E-15
Specc1l	4.5901E-20	0.34625202	0.334	0.278	1.007E-15
Dennd4c	5.2373E-20	0.5392871	0.104	0.067	1.149E-15
Zfp949	5.3164E-20	0.71497567	0.047	0.024	1.1664E-15
Prpf40a	5.9531E-20	0.27999586	0.389	0.331	1.306E-15
Upf2	6.5154E-20	0.27824769	0.205	0.151	1.4294E-15
Zkscan14	6.5941E-20	0.66541765	0.076	0.045	1.4467E-15
Pdap1	6.9803E-20	0.31371389	0.334	0.278	1.5314E-15
Arfrp1	9.0446E-20	0.49250679	0.161	0.115	1.9843E-15
Mrpl21	9.2632E-20	0.46955067	0.218	0.17	2.0323E-15
Bag4	9.5734E-20	0.6163424	0.077	0.046	2.1003E-15

Pdcd10	9.9666E-20	0.26708925	0.37	0.314	2.1866E-15
Thap3	1.0662E-19	0.47126744	0.095	0.059	2.3391E-15
Rbck1	1.1094E-19	0.48926075	0.172	0.126	2.4339E-15
D5Ertd579e	1.1396E-19	0.56517379	0.116	0.077	2.5001E-15
Tmem29	1.14E-19	0.8094488	0.083	0.051	2.5011E-15
E530011L22Ri	1.1956E-19	0.86339785	0.052	0.028	2.6229E-15
Zmat2	1.2212E-19	0.27971559	0.356	0.298	2.6792E-15
Nans	1.3185E-19	0.65748263	0.131	0.091	2.8927E-15
Nelfb	1.3485E-19	0.48546257	0.12	0.081	2.9585E-15
Alkbh4	1.453E-19	0.86373639	0.035	0.016	3.1878E-15
Tdg	1.5926E-19	0.46107212	0.15	0.106	3.494E-15
Cfap97	1.6606E-19	0.58144155	0.077	0.046	3.6433E-15
Sbf1	1.6851E-19	0.49101285	0.146	0.103	3.6969E-15
Dbf4	1.7538E-19	0.51289551	0.139	0.097	3.8477E-15
Gm28053	1.8687E-19	1.47365545	0.018	0.006	4.0998E-15
Naa10	1.8747E-19	0.67938051	0.147	0.106	4.1129E-15
Prelid3b	1.936E-19	0.63963971	0.164	0.121	4.2474E-15
Trabd	1.9411E-19	0.49365956	0.121	0.081	4.2586E-15
Ubr5	1.9742E-19	0.35507436	0.273	0.218	4.3313E-15
Surf1	2.2576E-19	0.58718944	0.126	0.087	4.953E-15
Prex1	2.4026E-19	0.35298378	0.284	0.23	5.2711E-15
Eif4a3	2.5072E-19	0.40235646	0.215	0.166	5.5005E-15
H13	2.5915E-19	0.31869238	0.279	0.226	5.6854E-15
Hnrnpr	2.6389E-19	0.30390498	0.284	0.227	5.7894E-15
F8a	2.6411E-19	0.85427875	0.046	0.023	5.7943E-15
Mmadhc	2.6863E-19	0.63837942	0.14	0.099	5.8935E-15
Pacs1	2.7044E-19	0.54412759	0.09	0.056	5.9331E-15
Ppp2r5c	3.0113E-19	0.28688726	0.329	0.273	6.6065E-15
Pfdn4	3.1973E-19	0.61229736	0.135	0.095	7.0146E-15
Gm28187	3.5596E-19	1.63506108	0.015	0.005	7.8095E-15
Nap1l4	3.7105E-19	0.37901272	0.236	0.184	8.1405E-15
Os9	4.1919E-19	0.42659809	0.254	0.205	9.1967E-15
Ccdc92	4.2211E-19	1.19688694	0.02	0.007	9.2607E-15
Wapl	4.5363E-19	0.260906	0.401	0.343	9.9522E-15
Fam58b	4.6465E-19	0.80409964	0.055	0.03	1.0194E-14
Slc37a3	4.6888E-19	0.74267879	0.066	0.038	1.0287E-14
Rer1	5.0323E-19	0.39154681	0.257	0.208	1.104E-14
Inafm2	5.1368E-19	0.97519293	0.04	0.019	1.127E-14
B9d2	5.7612E-19	0.56583712	0.157	0.115	1.2639E-14
Bcap31	5.8035E-19	0.34598228	0.286	0.234	1.2732E-14
Tubb4b	5.9741E-19	0.36617313	0.179	0.131	1.3107E-14
Kdm5b	6.1252E-19	0.29801218	0.147	0.101	1.3438E-14
Rgs9	7.2473E-19	0.95534565	0.044	0.022	1.59E-14
Eif1ax	8.8788E-19	0.4396367	0.26	0.212	1.9479E-14
Slc1a5	9.5641E-19	0.50723602	0.168	0.123	2.0983E-14
Tpgs2	9.9269E-19	1.00293601	0.054	0.029	2.1779E-14
Snrpd1	1.007E-18	0.25178761	0.389	0.333	2.2092E-14
Acbd5	1.0858E-18	0.30535967	0.214	0.162	2.3821E-14

Rnf14	1.1254E-18	0.65010877	0.099	0.064	2.469E-14
Gm30025	1.1314E-18	1.41812106	0.027	0.011	2.4822E-14
Ecsit	1.1331E-18	0.73300128	0.058	0.032	2.486E-14
Polk	1.1404E-18	0.79431896	0.049	0.026	2.5019E-14
Eif3g	1.207E-18	0.40043969	0.254	0.205	2.648E-14
Sar1b	1.211E-18	0.54133326	0.172	0.129	2.6567E-14
Ing4	1.2414E-18	0.53626937	0.099	0.064	2.7236E-14
Kdm1a	1.2606E-18	0.36023373	0.279	0.227	2.7656E-14
Pdik1l	1.2698E-18	0.9268992	0.045	0.023	2.7858E-14
Uap1	1.3197E-18	0.35572301	0.204	0.157	2.8954E-14
Lsm4	1.3379E-18	0.2653151	0.379	0.323	2.9353E-14
Ppp2r2d	1.3426E-18	0.35101519	0.264	0.212	2.9456E-14
Pld3	1.3935E-18	0.43573758	0.192	0.144	3.0573E-14
Ubl4a	1.4379E-18	0.77143867	0.117	0.08	3.1546E-14
Rnps1	1.4705E-18	0.30041805	0.283	0.228	3.2262E-14
2300009A05R	1.5068E-18	0.70264005	0.106	0.07	3.3057E-14
Fam173a	1.8147E-18	0.46376356	0.2	0.154	3.9813E-14
Strap	1.8398E-18	0.39759667	0.229	0.181	4.0364E-14
Cep97	2.0441E-18	0.64528435	0.05	0.027	4.4845E-14
Zfand2b	2.2181E-18	0.62966338	0.114	0.078	4.8664E-14
Cbx8	2.229E-18	1.04681181	0.027	0.011	4.8903E-14
Arl1	2.2334E-18	0.5001742	0.204	0.159	4.8998E-14
A930024E05R	2.235E-18	0.42370153	0.085	0.052	4.9033E-14
Xpo6	2.3318E-18	0.48286482	0.164	0.12	5.1157E-14
Kctd18	2.5253E-18	0.58205104	0.076	0.046	5.5402E-14
Smarcad1	2.554E-18	0.41053275	0.161	0.116	5.6031E-14
Kctd17	2.736E-18	1.20393408	0.039	0.019	6.0026E-14
Dnajc5	2.7495E-18	0.27020086	0.302	0.247	6.0321E-14
Sh3glb2	2.7672E-18	0.78166942	0.068	0.04	6.0711E-14
Dnajc3	3.1048E-18	0.33236393	0.32	0.267	6.8116E-14
Rab3gap1	3.131E-18	0.33095764	0.218	0.166	6.8692E-14
Ciao1	3.3395E-18	0.74173281	0.078	0.048	7.3266E-14
Rnf167	3.4505E-18	0.4016799	0.148	0.105	7.57E-14
Hras	3.5102E-18	0.71301732	0.105	0.071	7.7009E-14
Jak3	3.9098E-18	0.55774462	0.087	0.054	8.5777E-14
5033430I15Ril	3.9586E-18	1.121812	0.025	0.01	8.6847E-14
Idh3b	4.0832E-18	0.41536338	0.22	0.173	8.958E-14
Nudt16	4.2195E-18	0.70418228	0.058	0.033	9.2572E-14
Whamm	4.7648E-18	0.53448098	0.106	0.07	1.0453E-13
Mpzl3	4.7976E-18	0.73425274	0.045	0.023	1.0525E-13
Atp2a2	4.9306E-18	0.35680737	0.3	0.249	1.0817E-13
Med19	5.3311E-18	0.75638956	0.088	0.057	1.1696E-13
Dpm1	5.7392E-18	0.44315256	0.148	0.106	1.2591E-13
Usp3	5.8623E-18	0.35919699	0.231	0.18	1.2861E-13
Prc1	6.2412E-18	0.62704676	0.057	0.032	1.3693E-13
Sart1	6.4496E-18	0.43246217	0.172	0.127	1.415E-13
Nfkbib	6.9657E-18	0.27479908	0.372	0.315	1.5282E-13
Tmf1	7.5815E-18	0.32087719	0.238	0.187	1.6633E-13

Eva1b	7.7643E-18	0.66565873	0.13	0.092	1.7034E-13
Per1	7.9786E-18	0.2565929	0.278	0.223	1.7504E-13
Nfyb	9.5235E-18	0.40755596	0.154	0.112	2.0894E-13
Aplp1	9.5699E-18	1.11576328	0.023	0.009	2.0995E-13
Man1a2	1.0514E-17	0.43434142	0.187	0.141	2.3066E-13
Pttg1	1.066E-17	0.34650677	0.169	0.123	2.3386E-13
Mrpl15	1.233E-17	0.491707	0.162	0.121	2.7052E-13
Kansl3	1.2662E-17	0.44354028	0.175	0.13	2.7778E-13
Rexo1	1.3343E-17	0.42089268	0.185	0.14	2.9274E-13
Cry1	1.3698E-17	0.50188345	0.112	0.076	3.0053E-13
Cr1l	1.3923E-17	0.31305061	0.231	0.18	3.0545E-13
Ubxn6	1.4814E-17	0.50046085	0.159	0.118	3.2501E-13
Wdr78	1.5831E-17	1.62002882	0.014	0.004	3.4732E-13
Tmub1	1.6068E-17	0.78540394	0.058	0.033	3.5253E-13
Axin2	1.6103E-17	0.47489192	0.059	0.034	3.5328E-13
Csnk1g2	1.697E-17	0.27405807	0.202	0.153	3.723E-13
Mrps7	1.7771E-17	0.57766768	0.175	0.134	3.8988E-13
Tor1a	1.783E-17	0.61664355	0.139	0.101	3.9117E-13
Bdh1	1.8698E-17	0.91687862	0.027	0.012	4.1021E-13
Psma6	2.0233E-17	0.35334333	0.281	0.234	4.439E-13
Ppp1r8	2.1884E-17	0.6285722	0.071	0.043	4.8012E-13
Uba5	2.2676E-17	0.56248976	0.131	0.093	4.9749E-13
Rnf168	2.5285E-17	0.74206027	0.076	0.047	5.5473E-13
Nkiras1	2.6258E-17	0.7896257	0.043	0.023	5.7608E-13
March3	2.8614E-17	1.00178999	0.03	0.014	6.2775E-13
Borcs8	3.1345E-17	0.53813844	0.167	0.126	6.8767E-13
Gm5914	3.5054E-17	0.74796643	0.042	0.021	7.6905E-13
Ufd1	3.9777E-17	0.6143607	0.13	0.093	8.7266E-13
Bora	4.0696E-17	0.92151865	0.039	0.019	8.9282E-13
Cct5	4.3498E-17	0.27225598	0.346	0.295	9.5431E-13
Sgf29	4.8848E-17	0.72912292	0.058	0.033	1.0717E-12
Wbp1l	5.0077E-17	0.48798512	0.119	0.082	1.0986E-12
E2f4	5.8208E-17	0.54122543	0.108	0.074	1.277E-12
Golt1b	5.9596E-17	0.58887532	0.133	0.096	1.3075E-12
Rnf11	6.4853E-17	0.38830153	0.147	0.107	1.4228E-12
Sdhaf1	6.5766E-17	0.29079044	0.184	0.137	1.4429E-12
Polr2b	6.7724E-17	0.57498039	0.1	0.066	1.4858E-12
Hadha	6.8739E-17	0.42502705	0.24	0.194	1.5081E-12
Psmd9	6.9896E-17	0.5294896	0.126	0.089	1.5334E-12
Ndufs7	6.9918E-17	0.32793946	0.248	0.199	1.5339E-12
Adgrl1	7.8538E-17	0.51635517	0.066	0.039	1.7231E-12
C1qtnf12	8.8926E-17	1.19321536	0.034	0.016	1.951E-12
Fam160a2	9.0009E-17	0.74761349	0.036	0.018	1.9747E-12
Psmc2	9.0841E-17	0.51651808	0.163	0.122	1.993E-12
Zgpat	9.6295E-17	0.68960226	0.064	0.038	2.1126E-12
Eef1akmt1	1.0231E-16	1.07494297	0.051	0.029	2.2446E-12
Cep120	1.0243E-16	0.29657417	0.151	0.108	2.2472E-12
Slc35b3	1.0319E-16	0.61198869	0.085	0.054	2.2638E-12

2310061I04Ril	1.0684E-16	0.74153866	0.047	0.025	2.3439E-12
Gm10093	1.0692E-16	0.91271732	0.044	0.023	2.3458E-12
Rad52	1.0751E-16	0.97391681	0.034	0.016	2.3586E-12
Thap7	1.0784E-16	0.91461484	0.065	0.039	2.3658E-12
Ccdc30	1.151E-16	1.19618028	0.028	0.012	2.5251E-12
Mtfr1l	1.1981E-16	0.68649281	0.096	0.064	2.6284E-12
Atg13	1.2457E-16	0.64975307	0.084	0.054	2.733E-12
Abca2	1.2522E-16	0.69478313	0.056	0.032	2.7471E-12
Sec61a1	1.4067E-16	0.35040312	0.271	0.224	3.0861E-12
Tipin	1.4429E-16	0.88581147	0.05	0.028	3.1655E-12
Ube2t	1.867E-16	0.97246263	0.041	0.021	4.096E-12
Fmnl1	1.9282E-16	0.26011121	0.261	0.212	4.2302E-12
Kif20b	1.9462E-16	0.90438388	0.039	0.02	4.2697E-12
Prpf8	2.118E-16	0.32859353	0.252	0.203	4.6467E-12
Fuom	2.4169E-16	1.13345021	0.049	0.027	5.3024E-12
Tnk2	2.4227E-16	0.49275758	0.065	0.039	5.3152E-12
Rpp21	2.4306E-16	0.42321828	0.193	0.149	5.3325E-12
Cnep1r1	2.4601E-16	0.63210548	0.085	0.055	5.3972E-12
Dcp2	2.4608E-16	0.47846299	0.158	0.118	5.3987E-12
Gm10369	2.668E-16	2.03907618	0.01	0.003	5.8532E-12
Ppil3	2.7746E-16	0.83620373	0.08	0.051	6.0871E-12
Kmt5b	3.1211E-16	0.27605059	0.21	0.162	6.8474E-12
Mrpl22	3.2177E-16	0.89004647	0.058	0.034	7.0593E-12
Knop1	3.4662E-16	0.42991829	0.126	0.089	7.6046E-12
Mrps31	3.471E-16	0.82413288	0.06	0.036	7.6151E-12
Wipi2	3.6124E-16	0.36217814	0.148	0.107	7.9252E-12
Dapk3	3.6841E-16	0.51918432	0.117	0.082	8.0826E-12
Mex3c	3.6967E-16	0.41508253	0.14	0.101	8.1101E-12
Arid4a	3.7282E-16	0.25402246	0.256	0.204	8.1793E-12
Trim27	4.1121E-16	0.47224429	0.145	0.106	9.0215E-12
Smim11	4.3961E-16	0.39306179	0.176	0.134	9.6445E-12
Dazap1	4.7393E-16	0.36458841	0.211	0.166	1.0398E-11
Fam204a	4.8734E-16	0.27534481	0.271	0.22	1.0692E-11
Atg101	5.0637E-16	0.52197718	0.113	0.079	1.1109E-11
Pdxp	6.019E-16	1.22677555	0.018	0.007	1.3205E-11
Brk1	7.1282E-16	0.2559766	0.413	0.365	1.5639E-11
Hddc2	8.0529E-16	0.77951941	0.063	0.038	1.7667E-11
Slc39a3	8.598E-16	0.9468145	0.044	0.024	1.8863E-11
Nbdy	9.214E-16	0.74927587	0.1	0.069	2.0215E-11
Gm47689	9.2288E-16	1.91305332	0.01	0.003	2.0247E-11
Arpc5l	9.3621E-16	0.36259542	0.171	0.13	2.054E-11
Rufy2	9.6886E-16	0.40532736	0.081	0.051	2.1256E-11
Tbc1d1	9.773E-16	0.34564618	0.166	0.124	2.1441E-11
Plekhb2	1.0207E-15	0.44276842	0.171	0.131	2.2392E-11
Zfp746	1.0319E-15	0.48763287	0.115	0.08	2.2638E-11
Sigirr	1.1066E-15	0.79087819	0.05	0.028	2.4277E-11
Wbp4	1.1378E-15	0.3526127	0.228	0.183	2.4962E-11
Umps	1.1973E-15	0.69506789	0.057	0.033	2.6267E-11

Ngrn	1.2385E-15	0.5900735	0.058	0.034	2.717E-11
Herc3	1.4238E-15	0.49836119	0.073	0.045	3.1237E-11
Rbm22	1.4659E-15	0.28448744	0.272	0.223	3.216E-11
Smarca2	1.4776E-15	0.38793157	0.183	0.14	3.2416E-11
Polr2k	1.5894E-15	0.30402341	0.304	0.258	3.4869E-11
Nudt16l1	1.7482E-15	0.60338428	0.071	0.045	3.8353E-11
Dnttip1	1.7657E-15	0.60367176	0.105	0.073	3.8738E-11
Zfp788	2.2966E-15	0.90502198	0.034	0.017	5.0386E-11
Rcan3	2.4009E-15	1.0775252	0.032	0.016	5.2672E-11
Slc25a51	2.427E-15	0.38151666	0.212	0.169	5.3246E-11
Rab33b	2.6728E-15	0.53720426	0.067	0.041	5.8638E-11
Gtf2b	2.7077E-15	0.33291228	0.237	0.192	5.9404E-11
Nsfl1c	2.9004E-15	0.46456221	0.139	0.102	6.3631E-11
Pdha1	2.9762E-15	0.45443185	0.184	0.143	6.5294E-11
Fam120aos	3.2878E-15	1.33408929	0.019	0.008	7.2131E-11
Gm9844	3.3763E-15	0.90922508	0.049	0.028	7.4073E-11
Lxn	3.4828E-15	1.0537157	0.044	0.024	7.641E-11
Maml2	3.5285E-15	0.62206161	0.059	0.035	7.7411E-11
Rbm4b	3.5645E-15	0.50695186	0.11	0.076	7.8202E-11
Rab11fip4	3.7235E-15	0.82787767	0.039	0.02	8.169E-11
Rbmx	3.7923E-15	0.42375929	0.095	0.064	8.3199E-11
Tmem165	3.9278E-15	0.49159489	0.109	0.076	8.6172E-11
Abt1	4.2592E-15	0.53066696	0.09	0.06	9.3442E-11
Chfr	4.27E-15	0.31601788	0.18	0.137	9.3679E-11
Adprm	4.4891E-15	0.66903209	0.056	0.033	9.8487E-11
Uba2	4.5301E-15	0.41621329	0.144	0.106	9.9387E-11
Nudt19	4.5474E-15	0.83204591	0.049	0.028	9.9765E-11
Eif1ad	4.5808E-15	0.47315676	0.151	0.113	1.005E-10
Cdc25a	5.1006E-15	0.91435008	0.053	0.031	1.119E-10
Emc3	5.4473E-15	0.36482161	0.215	0.172	1.1951E-10
Mrps33	5.8125E-15	0.40025458	0.264	0.224	1.2752E-10
Mtr	6.0178E-15	0.63462539	0.074	0.048	1.3202E-10
Carmil2	6.0245E-15	0.61623845	0.042	0.023	1.3217E-10
Gskip	6.3406E-15	0.57414474	0.099	0.068	1.3911E-10
Gm20275	6.6131E-15	1.52096414	0.01	0.003	1.4508E-10
Haus3	7.4851E-15	0.28211972	0.134	0.095	1.6422E-10
Bloc1s4	7.8288E-15	0.53736522	0.118	0.084	1.7176E-10
Zfp770	8.4763E-15	0.87549566	0.037	0.019	1.8596E-10
Nde1	8.5512E-15	0.70978995	0.06	0.036	1.876E-10
Zbtb11os1	9.0997E-15	1.01660399	0.029	0.014	1.9964E-10
Lcn4	9.761E-15	0.78784261	0.028	0.013	2.1415E-10
Trmt10c	1E-14	0.4596781	0.077	0.05	2.1939E-10
Def6	1.0105E-14	0.49392848	0.08	0.052	2.2169E-10
Eny2	1.1051E-14	0.28462502	0.268	0.222	2.4245E-10
Ttc33	1.1056E-14	0.55568831	0.082	0.054	2.4257E-10
Hypk	1.1228E-14	0.37273868	0.203	0.162	2.4632E-10
Zfp646	1.2036E-14	0.4016616	0.099	0.068	2.6406E-10
Trappc13	1.2338E-14	0.62266015	0.074	0.048	2.7069E-10

Cdc5l	1.3246E-14	0.28636492	0.293	0.247	2.906E-10
Nlrc5	1.339E-14	0.31552257	0.118	0.083	2.9377E-10
Sec22a	1.4428E-14	0.87024131	0.045	0.025	3.1653E-10
Ptpn11	1.4586E-14	0.44843901	0.176	0.137	3.2E-10
Mpst	1.4647E-14	0.7852445	0.075	0.049	3.2135E-10
Phgdh	1.5171E-14	0.51363227	0.08	0.052	3.3284E-10
Tradd	1.541E-14	0.88528724	0.05	0.029	3.3808E-10
Commd7	1.5941E-14	0.48576187	0.144	0.108	3.4972E-10
Arhgap1	1.6181E-14	0.59607001	0.099	0.068	3.5499E-10
Jtb	1.6215E-14	0.40389384	0.21	0.17	3.5575E-10
Col4a3bp	1.6675E-14	0.29597475	0.204	0.16	3.6583E-10
Rpa2	2.0049E-14	0.29171359	0.096	0.064	4.3985E-10
6330403K07R	2.1954E-14	1.31556772	0.014	0.005	4.8165E-10
Mrpl35	2.2169E-14	0.57500426	0.128	0.094	4.8636E-10
Stard3nl	2.2319E-14	0.71574493	0.128	0.096	4.8966E-10
Ormdl1	2.4747E-14	0.57912686	0.057	0.034	5.4293E-10
Zer1	2.6104E-14	0.38753651	0.069	0.043	5.7271E-10
Tmem60	2.6635E-14	0.45376155	0.097	0.067	5.8433E-10
Thoc3	2.8744E-14	0.72685255	0.082	0.055	6.3061E-10
Gm26765	2.9002E-14	1.38559785	0.013	0.004	6.3626E-10
Brd9	3.0118E-14	0.31554963	0.208	0.165	6.6075E-10
Fam60a	3.0578E-14	0.48991175	0.097	0.067	6.7085E-10
Dnajc21	3.0893E-14	0.31287436	0.225	0.182	6.7776E-10
Bax	3.1825E-14	0.32193929	0.308	0.266	6.982E-10
Map1lc3a	3.2791E-14	0.48059647	0.165	0.129	7.1939E-10
Wdfy1	3.3016E-14	0.60134586	0.115	0.083	7.2433E-10
Klc1	3.3733E-14	0.58791966	0.093	0.063	7.4007E-10
Med11	3.4771E-14	0.40815101	0.145	0.108	7.6285E-10
Pno1	3.5222E-14	0.72456255	0.099	0.069	7.7273E-10
Gm26724	3.6769E-14	1.25010181	0.025	0.012	8.0667E-10
Dpy30	3.7045E-14	0.60517778	0.114	0.082	8.1274E-10
Lpar6	3.7375E-14	0.40558616	0.116	0.083	8.1997E-10
Tpgs1	3.9301E-14	0.8218544	0.071	0.046	8.6222E-10
Ccdc66	3.9516E-14	0.72886	0.039	0.021	8.6695E-10
Rce1	4.0539E-14	0.65026169	0.078	0.051	8.8939E-10
Lmbr1l	4.4101E-14	0.36393937	0.088	0.059	9.6753E-10
Gid8	4.5245E-14	0.49438359	0.104	0.073	9.9264E-10
Aup1	4.7022E-14	0.32662054	0.275	0.233	1.0316E-09
Akr1b3	4.8589E-14	0.34247843	0.238	0.197	1.066E-09
Selenoo	4.8677E-14	0.70663595	0.043	0.024	1.0679E-09
Smim20	5.0127E-14	0.58015539	0.098	0.069	1.0997E-09
Gnptg	5.0456E-14	0.42198871	0.089	0.06	1.107E-09
Snip1	5.0931E-14	0.48093081	0.102	0.071	1.1174E-09
Btbd1	5.4418E-14	0.50801362	0.146	0.111	1.1939E-09
C1d	5.501E-14	0.57774883	0.127	0.094	1.2069E-09
E130311K13R	5.5384E-14	1.00426289	0.025	0.012	1.2151E-09
Rc3h2	5.5756E-14	0.25164029	0.226	0.181	1.2232E-09
Map2k2	5.6176E-14	0.34633121	0.263	0.222	1.2324E-09

Syap1	5.7705E-14	0.53399088	0.14	0.106	1.266E-09
Cul2	5.8994E-14	0.4658256	0.105	0.073	1.2943E-09
4732440D04R	6.3511E-14	0.49483224	0.064	0.04	1.3934E-09
Cep44	6.3914E-14	0.37678508	0.086	0.057	1.4022E-09
Dnajb13	6.4961E-14	1.386657	0.015	0.006	1.4252E-09
Cyth2	6.4995E-14	0.5036133	0.147	0.112	1.4259E-09
Phf6	7.0595E-14	0.32615527	0.131	0.095	1.5488E-09
Btg3	7.5654E-14	0.55823206	0.084	0.056	1.6598E-09
Cox19	7.7724E-14	0.74809986	0.07	0.045	1.7052E-09
Cbx1	7.8806E-14	0.26458308	0.201	0.158	1.7289E-09
Snx17	7.9646E-14	0.40569882	0.176	0.138	1.7474E-09
Pcmt1	8.2182E-14	0.36782246	0.206	0.166	1.803E-09
Ggps1	8.2467E-14	0.36182926	0.118	0.084	1.8092E-09
Gm9856	8.4233E-14	0.63425405	0.046	0.026	1.848E-09
Prpf4	8.5108E-14	0.39389499	0.123	0.089	1.8672E-09
Tex261	8.563E-14	0.42478934	0.157	0.121	1.8786E-09
Cox20	8.9391E-14	0.34229849	0.189	0.149	1.9612E-09
Sucla2	9.8239E-14	0.55324222	0.107	0.076	2.1553E-09
Pcbp4	1.0073E-13	0.91026714	0.023	0.01	2.21E-09
Pdcl	1.0533E-13	0.47525341	0.097	0.067	2.3109E-09
Ccng2	1.0971E-13	0.30833791	0.13	0.095	2.407E-09
Kif5c	1.1026E-13	1.01022446	0.018	0.008	2.419E-09
Faf2	1.147E-13	0.49848703	0.118	0.085	2.5164E-09
Parl	1.2084E-13	0.52054584	0.138	0.105	2.6512E-09
Cdan1	1.2844E-13	0.74363502	0.035	0.018	2.8178E-09
Sgtb	1.4234E-13	0.74033452	0.037	0.02	3.1228E-09
Zfpl1	1.7155E-13	0.85096705	0.056	0.035	3.7637E-09
Pak4	1.7782E-13	1.15639097	0.04	0.022	3.9011E-09
Abcf2	1.8595E-13	0.6343616	0.078	0.052	4.0795E-09
Nt5c3b	1.8626E-13	0.6565092	0.054	0.033	4.0864E-09
Mrps6	1.8733E-13	0.31613561	0.169	0.13	4.1099E-09
Mia2	1.8939E-13	0.28389729	0.313	0.27	4.155E-09
Cd3eap	2.0542E-13	0.96458624	0.039	0.021	4.5067E-09
Atl3	2.0986E-13	0.3621421	0.145	0.109	4.6042E-09
Eif4e3	2.1524E-13	0.60162436	0.087	0.059	4.722E-09
Dennd2d	2.1658E-13	0.28628493	0.068	0.043	4.7516E-09
Cbll1	2.1999E-13	0.33971462	0.147	0.111	4.8263E-09
Emc6	2.3781E-13	0.28637716	0.267	0.225	5.2172E-09
Gstm5	2.7137E-13	0.6485612	0.044	0.025	5.9535E-09
Ddt	2.7264E-13	0.34290866	0.208	0.17	5.9815E-09
Capn10	2.9517E-13	1.09530131	0.026	0.012	6.4757E-09
Asxl1	3.014E-13	0.44935273	0.112	0.08	6.6124E-09
Mau2	3.1036E-13	0.26530767	0.182	0.141	6.8089E-09
AI837181	3.3974E-13	0.45375255	0.1	0.07	7.4536E-09
St3gal2	3.4155E-13	0.98179013	0.027	0.013	7.4932E-09
Zbtb24	3.5153E-13	0.57971713	0.067	0.043	7.7122E-09
Mrpl12	3.6083E-13	0.42757943	0.174	0.138	7.9163E-09
Nit1	3.6231E-13	0.58503563	0.075	0.05	7.9487E-09

Eed	3.7889E-13	0.35394184	0.15	0.113	8.3124E-09
4933427D14R	4.5763E-13	0.88353005	0.025	0.012	1.004E-08
Map3k10	4.6263E-13	0.96196697	0.025	0.012	1.015E-08
Ube2g1	4.7067E-13	0.440499	0.108	0.077	1.0326E-08
Banf1	4.9538E-13	0.27652616	0.264	0.221	1.0868E-08
Elf4	5.1912E-13	0.43507547	0.127	0.094	1.1389E-08
Zfp654	5.2266E-13	0.37611879	0.084	0.057	1.1467E-08
Elof1	5.2437E-13	0.59528794	0.134	0.103	1.1504E-08
Sap30bp	5.3208E-13	0.58135965	0.105	0.076	1.1673E-08
Mrps21	5.4415E-13	0.31734942	0.273	0.235	1.1938E-08
Commd4	5.9891E-13	0.50336703	0.173	0.139	1.3139E-08
Anapc2	6.1421E-13	0.61721606	0.081	0.055	1.3475E-08
2310011J03Ril	6.1559E-13	0.52890734	0.113	0.083	1.3505E-08
Tmem14c	6.2556E-13	0.31185042	0.301	0.262	1.3724E-08
Sirt7	6.348E-13	0.34661052	0.148	0.112	1.3927E-08
Mrpl34	6.5607E-13	0.56074404	0.133	0.101	1.4394E-08
Gtf3a	6.722E-13	0.53796773	0.083	0.056	1.4747E-08
Sirt2	6.7932E-13	0.36875784	0.217	0.18	1.4904E-08
Txndc9	7.3129E-13	0.3497565	0.22	0.183	1.6044E-08
Esco1	7.6697E-13	0.25965526	0.169	0.13	1.6827E-08
Gmds	7.9697E-13	0.79030812	0.059	0.038	1.7485E-08
2700062C07R	8.0292E-13	0.79111013	0.047	0.028	1.7615E-08
Nudc	8.0667E-13	0.33730184	0.21	0.172	1.7698E-08
Ctr9	8.3767E-13	0.32935953	0.12	0.087	1.8378E-08
Rpa3	8.6696E-13	0.54234844	0.078	0.052	1.902E-08
Casp1	9.0107E-13	0.63395431	0.081	0.056	1.9769E-08
Fxn	9.1956E-13	0.75732125	0.055	0.034	2.0174E-08
Eif4e2	9.4271E-13	0.29410312	0.274	0.235	2.0682E-08
Kctd9	9.6047E-13	0.81455631	0.042	0.024	2.1072E-08
Ndufs8	9.811E-13	0.39290345	0.207	0.17	2.1524E-08
Bcl7c	9.8185E-13	0.46866854	0.165	0.131	2.1541E-08
Usp42	9.8509E-13	0.64548952	0.064	0.041	2.1612E-08
Adam12	1.0047E-12	0.91653246	0.019	0.008	2.2042E-08
Orc6	1.0681E-12	0.56979396	0.077	0.052	2.3433E-08
Fbxo3	1.0775E-12	0.46631076	0.104	0.074	2.3639E-08
Lgalsl	1.0882E-12	0.93055502	0.03	0.015	2.3875E-08
Copb1	1.0888E-12	0.36521802	0.238	0.2	2.3886E-08
Mis18a	1.1424E-12	0.56770539	0.081	0.055	2.5063E-08
Myo1g	1.3405E-12	0.35526575	0.251	0.213	2.941E-08
AI987944	1.3906E-12	0.51241534	0.052	0.032	3.0507E-08
Fyco1	1.4295E-12	0.51367834	0.081	0.055	3.1361E-08
Ccser2	1.4632E-12	0.29632449	0.154	0.118	3.2101E-08
Dnajc17	1.4929E-12	0.9104773	0.031	0.016	3.2754E-08
Tinf2	1.5063E-12	0.38700826	0.111	0.08	3.3046E-08
Tigar	1.5371E-12	0.67840178	0.032	0.017	3.3723E-08
Nop9	1.6455E-12	0.50339707	0.095	0.067	3.61E-08
Magee1	1.76E-12	1.14000035	0.013	0.005	3.8613E-08
Fbxo8	1.7692E-12	0.35009611	0.084	0.057	3.8815E-08

Dynll2	1.8672E-12	0.42531408	0.163	0.128	4.0964E-08
Capn15	1.9216E-12	0.56846561	0.063	0.04	4.2157E-08
Nsdhl	1.9774E-12	0.97298931	0.042	0.024	4.3383E-08
Gm14029	2.0427E-12	0.85249432	0.029	0.015	4.4814E-08
Fam76b	2.0591E-12	0.25074228	0.157	0.12	4.5174E-08
Ash2l	2.0698E-12	0.51106837	0.086	0.06	4.5408E-08
Xpo4	2.1145E-12	0.33219087	0.123	0.09	4.6389E-08
Snrnp25	2.1565E-12	0.59674388	0.082	0.057	4.7311E-08
Mink1	2.2075E-12	0.57182973	0.095	0.067	4.8429E-08
Ebpl	2.2515E-12	0.68824858	0.07	0.047	4.9396E-08
Cwc22	2.2623E-12	0.40401804	0.133	0.1	4.9632E-08
Ppp1r3f	2.4575E-12	0.99134765	0.016	0.006	5.3914E-08
Rwdd4a	2.6898E-12	0.70064031	0.091	0.064	5.9012E-08
Sik3	2.6931E-12	0.2723174	0.23	0.189	5.9085E-08
Zfp212	2.6946E-12	0.56369897	0.06	0.038	5.9117E-08
Pygo2	2.8078E-12	0.74964171	0.039	0.022	6.16E-08
Hdgfl3	2.8169E-12	0.55211389	0.048	0.028	6.18E-08
Acat1	2.8266E-12	0.53817899	0.125	0.095	6.2013E-08
Zfp777	3.0931E-12	0.61836823	0.042	0.024	6.786E-08
Zfp707	3.2169E-12	0.76494109	0.027	0.014	7.0574E-08
Itpripl1	3.3466E-12	0.96276783	0.037	0.021	7.3422E-08
Mak16	3.7549E-12	0.31528536	0.188	0.15	8.2378E-08
Rngtt	3.7699E-12	0.43516494	0.111	0.081	8.2708E-08
Mrpl46	3.8314E-12	0.52302299	0.102	0.074	8.4058E-08
Traf4	3.8507E-12	0.2825188	0.124	0.091	8.448E-08
Scamp3	3.9471E-12	0.34970195	0.142	0.108	8.6596E-08
Zfp113	4.0063E-12	0.79842044	0.026	0.013	8.7895E-08
Ndufc2	4.0079E-12	0.25045692	0.34	0.301	8.7929E-08
Suox	4.0376E-12	1.11981163	0.025	0.013	8.858E-08
Creld2	4.218E-12	0.50746503	0.158	0.126	9.2538E-08
Kin	4.8015E-12	0.44992245	0.126	0.095	1.0534E-07
Polr2f	4.8814E-12	0.39226267	0.2	0.165	1.0709E-07
Zfp287	4.96E-12	0.83331091	0.021	0.01	1.0882E-07
Otud7b	5.0307E-12	0.35013879	0.067	0.043	1.1037E-07
Mtap	5.1592E-12	0.59023446	0.046	0.027	1.1319E-07
Fkbp8	5.2908E-12	0.34257955	0.234	0.197	1.1607E-07
E030030I06Ril	5.3406E-12	0.50876067	0.031	0.017	1.1717E-07
Cdc26	5.562E-12	0.34598798	0.153	0.119	1.2202E-07
Fbxw17	5.7851E-12	0.91139702	0.04	0.023	1.2692E-07
Mrpl24	5.8095E-12	0.28877644	0.263	0.225	1.2745E-07
Atg4d	5.9282E-12	0.49393055	0.067	0.044	1.3006E-07
Uhrf1bp1l	6.5555E-12	0.28712372	0.194	0.156	1.4382E-07
Phospho2	6.6481E-12	0.54839593	0.07	0.046	1.4585E-07
Zfp677	6.7257E-12	0.7623009	0.028	0.014	1.4756E-07
Pdpk1	7.0983E-12	0.33914548	0.159	0.124	1.5573E-07
Mplkip	7.3709E-12	0.37082086	0.143	0.11	1.6171E-07
Npm3	7.3745E-12	0.31758151	0.216	0.179	1.6179E-07
Gstt2	7.4944E-12	0.67814071	0.06	0.039	1.6442E-07

Arhgap26	7.6842E-12	0.36961666	0.134	0.101	1.6858E-07
Zfp874b	8.0021E-12	0.43643533	0.055	0.034	1.7556E-07
Vti1b	8.868E-12	0.46468655	0.163	0.131	1.9456E-07
Dctn2	8.9937E-12	0.47698651	0.143	0.112	1.9731E-07
Zfp280b	9.2554E-12	0.83566019	0.034	0.019	2.0305E-07
Hscb	9.6835E-12	0.55753099	0.108	0.08	2.1245E-07
Tusc3	9.8496E-12	0.40705218	0.097	0.069	2.1609E-07
Prcc	1.1014E-11	0.67082955	0.065	0.043	2.4163E-07
Zdhhc7	1.1506E-11	0.64002586	0.067	0.044	2.5243E-07
Tg	1.2112E-11	1.11444775	0.016	0.007	2.6572E-07
Mrps17	1.2464E-11	0.50652895	0.141	0.11	2.7345E-07
Tex264	1.2705E-11	0.65408929	0.076	0.053	2.7873E-07
Srprb	1.3699E-11	0.63121999	0.082	0.057	3.0055E-07
Rae1	1.4054E-11	0.42578855	0.097	0.07	3.0833E-07
Ccndbp1	1.5216E-11	0.36933883	0.143	0.111	3.3383E-07
Sugp1	1.5612E-11	0.44224405	0.083	0.058	3.4251E-07
Mms22l	1.5691E-11	0.73015863	0.039	0.023	3.4425E-07
Mrps34	1.5708E-11	0.48698408	0.126	0.096	3.4463E-07
Chchd1	1.5796E-11	0.36460203	0.235	0.2	3.4654E-07
Mcrs1	1.6043E-11	0.40637585	0.114	0.085	3.5197E-07
Strn	1.6226E-11	0.44153646	0.084	0.059	3.5599E-07
Pitpnb	1.7103E-11	0.41980084	0.125	0.095	3.7521E-07
Adnp	1.7127E-11	0.64340795	0.05	0.031	3.7574E-07
Ankra2	1.7158E-11	0.41568178	0.091	0.064	3.7643E-07
Zfp146	1.7245E-11	0.41056359	0.065	0.043	3.7834E-07
Hif1an	1.7446E-11	0.28990397	0.127	0.095	3.8275E-07
Pard6a	1.7969E-11	0.78129152	0.044	0.026	3.9422E-07
Mrps11	1.9982E-11	0.71767583	0.069	0.046	4.3838E-07
Pop4	2.1333E-11	0.57793509	0.087	0.062	4.6802E-07
Gm15655	2.1532E-11	0.52789564	0.039	0.023	4.7239E-07
2510039O18R	2.2353E-11	0.35172672	0.113	0.083	4.904E-07
Etfrf1	2.3737E-11	0.59935421	0.052	0.032	5.2077E-07
Zfp930	2.444E-11	0.81742757	0.026	0.014	5.3619E-07
Ndufaf3	2.4756E-11	0.73060076	0.07	0.048	5.4312E-07
Chmp5	2.5289E-11	0.29405531	0.203	0.169	5.5482E-07
Vopp1	2.5474E-11	0.4466263	0.102	0.074	5.5888E-07
Zmpste24	2.6547E-11	0.4867757	0.114	0.086	5.8242E-07
Sh3bp5l	2.8023E-11	0.79834946	0.034	0.019	6.148E-07
Gm4673	2.8416E-11	0.72167845	0.032	0.017	6.2341E-07
Traf3	2.8721E-11	0.3277972	0.176	0.142	6.3011E-07
Wdr95	2.9034E-11	0.94050048	0.017	0.008	6.3697E-07
Agpat3	3.0684E-11	0.3929155	0.124	0.094	6.7317E-07
Letm1	3.1054E-11	0.46000023	0.123	0.093	6.813E-07
Drg1	3.1505E-11	0.43669936	0.11	0.081	6.9118E-07
Vps72	3.3382E-11	0.53334662	0.075	0.052	7.3237E-07
Gps1	3.5635E-11	0.56068753	0.092	0.066	7.818E-07
Dnajc18	3.7215E-11	0.74987377	0.041	0.024	8.1646E-07
Tfg	3.8053E-11	0.59697328	0.096	0.07	8.3485E-07

Adrm1	3.9644E-11	0.43548444	0.16	0.129	8.6975E-07
Klhl28	3.9811E-11	0.31772475	0.094	0.067	8.7342E-07
Caap1	3.9966E-11	0.4575851	0.08	0.055	8.7681E-07
Fam193b	4.1184E-11	0.32454383	0.071	0.048	9.0353E-07
Rab11fip2	4.3407E-11	0.36745194	0.104	0.076	9.523E-07
Prkrip1	4.4807E-11	0.44583328	0.11	0.082	9.8303E-07
Rdh11	4.7839E-11	0.38580328	0.067	0.045	1.0495E-06
C030034I22Ri	4.9952E-11	0.40421476	0.07	0.047	1.0959E-06
Isca2	5.007E-11	0.55397608	0.1	0.074	1.0985E-06
Ttc9c	5.1025E-11	0.56020467	0.083	0.059	1.1194E-06
Lzic	5.3451E-11	0.60646624	0.064	0.043	1.1727E-06
Iars	5.5524E-11	0.41130137	0.112	0.083	1.2181E-06
Dph7	5.5948E-11	0.72895079	0.023	0.011	1.2274E-06
Zfp235	6.4472E-11	0.94940868	0.017	0.008	1.4145E-06
Uchl5	6.475E-11	0.42067892	0.113	0.085	1.4205E-06
Smim8	7.0708E-11	0.56136106	0.076	0.052	1.5513E-06
Pgp	7.2164E-11	0.54970748	0.076	0.052	1.5832E-06
Pdcd2	7.5292E-11	0.47449024	0.093	0.067	1.6518E-06
Zdhhc3	8.0014E-11	0.44321053	0.13	0.101	1.7554E-06
Heatr3	8.0409E-11	0.75701028	0.052	0.033	1.7641E-06
Hsd17b7	8.0793E-11	0.51170526	0.048	0.03	1.7725E-06
Rtf2	8.0885E-11	0.36429485	0.239	0.208	1.7745E-06
Cops5	8.1993E-11	0.31669911	0.193	0.159	1.7988E-06
Sephs2	8.2136E-11	0.40705535	0.059	0.039	1.802E-06
Armc7	8.3465E-11	0.55731106	0.05	0.031	1.8311E-06
Actr8	8.3803E-11	0.55976077	0.05	0.031	1.8386E-06
Apopt1	8.5663E-11	0.55265373	0.11	0.083	1.8794E-06
Tbpl1	8.8501E-11	0.38220891	0.112	0.084	1.9416E-06
Rab3ip	8.8826E-11	0.56869037	0.061	0.04	1.9488E-06
Zmat5	8.8922E-11	0.63474687	0.092	0.068	1.9509E-06
Slu7	9.1292E-11	0.26056223	0.215	0.179	2.0029E-06
1500011B03R	9.6069E-11	0.83466683	0.034	0.02	2.1077E-06
Brcc3	1.0078E-10	0.48491566	0.09	0.065	2.211E-06
Cutc	1.0441E-10	1.01251715	0.033	0.018	2.2907E-06
Ilkap	1.0468E-10	0.35899878	0.143	0.112	2.2965E-06
Eif6	1.0476E-10	0.35241552	0.246	0.215	2.2982E-06
Dgcr14	1.1386E-10	0.60848739	0.057	0.038	2.4981E-06
Fam162a	1.2259E-10	0.55664793	0.117	0.09	2.6896E-06
Proser1	1.232E-10	0.37746948	0.116	0.087	2.7028E-06
Gm527	1.239E-10	0.82917553	0.023	0.011	2.7183E-06
Psmb7	1.2483E-10	0.33856389	0.265	0.234	2.7387E-06
Gatad2a	1.2781E-10	0.27421843	0.221	0.185	2.804E-06
Secisbp2l	1.2952E-10	0.33195548	0.154	0.122	2.8415E-06
Gm27010	1.3226E-10	0.9037829	0.02	0.01	2.9016E-06
Nipal1	1.3722E-10	1.11831847	0.02	0.009	3.0104E-06
Magi2	1.4754E-10	0.9645832	0.014	0.006	3.237E-06
Dmap1	1.6583E-10	0.75348966	0.037	0.022	3.6382E-06
Psmd12	1.684E-10	0.36357248	0.211	0.179	3.6944E-06

Dtwd1	1.7212E-10	0.78673039	0.022	0.011	3.7762E-06
Tbce	1.8173E-10	0.43788627	0.103	0.077	3.987E-06
Mlx	1.8675E-10	0.58738091	0.077	0.054	4.097E-06
Sdr39u1	1.883E-10	0.7879172	0.025	0.013	4.1312E-06
Slc35e2	1.9249E-10	0.37778856	0.068	0.046	4.2231E-06
Erh	1.9656E-10	0.52462794	0.128	0.1	4.3123E-06
Slc4a1ap	2.0735E-10	0.25931223	0.124	0.093	4.549E-06
Asb3	2.1168E-10	0.46024417	0.043	0.027	4.6441E-06
Galnt2	2.1613E-10	1.17909472	0.015	0.006	4.7416E-06
Dgke	2.199E-10	0.57301597	0.056	0.037	4.8243E-06
Cabin1	2.2237E-10	0.37870274	0.126	0.097	4.8786E-06
Cyc1	2.2419E-10	0.39662888	0.183	0.153	4.9186E-06
Dnajc2	2.2734E-10	0.26085514	0.213	0.178	4.9877E-06
Dedd	2.2935E-10	0.56009687	0.065	0.044	5.0318E-06
Rsrc1	2.3817E-10	0.40970423	0.148	0.118	5.2253E-06
Dnajb12	2.3909E-10	0.58852396	0.075	0.053	5.2454E-06
Selenom	2.4254E-10	0.71180217	0.082	0.06	5.3211E-06
Exosc9	2.4473E-10	0.55691654	0.068	0.046	5.3692E-06
2610008E11R	2.4869E-10	0.51087424	0.044	0.027	5.456E-06
AI480526	2.7395E-10	0.53678257	0.034	0.019	6.0102E-06
Psmd13	2.8388E-10	0.34540491	0.189	0.158	6.2281E-06
Ankrd50	2.871E-10	0.44344292	0.065	0.044	6.2987E-06
Gpank1	2.9639E-10	0.33677766	0.089	0.064	6.5024E-06
Kdelr2	2.9853E-10	0.40013572	0.154	0.124	6.5495E-06
Reep4	2.9893E-10	0.59548137	0.063	0.042	6.5583E-06
Exosc7	3.076E-10	0.60378034	0.058	0.039	6.7484E-06
Leng1	3.1871E-10	0.4986635	0.079	0.056	6.9922E-06
Klhl12	3.2613E-10	0.26276984	0.07	0.047	7.1551E-06
Trim24	3.3586E-10	0.26143844	0.111	0.083	7.3684E-06
Rbm15b	3.469E-10	0.50013751	0.062	0.042	7.6106E-06
Blmh	3.4892E-10	0.32211006	0.155	0.123	7.655E-06
Rint1	3.5216E-10	0.58198808	0.046	0.029	7.7259E-06
Zcchc17	3.5532E-10	0.40326908	0.129	0.1	7.7953E-06
Lrba	3.5629E-10	0.27598698	0.127	0.096	7.8166E-06
Mfhas1	3.6057E-10	0.71581132	0.079	0.056	7.9106E-06
Tmem115	3.8635E-10	0.65746881	0.048	0.031	8.4762E-06
Rmnd5a	3.89E-10	0.33193702	0.157	0.126	8.5342E-06
Paxx	4.0552E-10	0.45881476	0.077	0.054	8.8967E-06
Ryk	4.0623E-10	0.51186901	0.034	0.019	8.9122E-06
Ddb2	4.1798E-10	0.38539618	0.051	0.033	9.1702E-06
Zdhhc12	4.4484E-10	1.08991434	0.023	0.012	9.7593E-06
Zfp53	4.5378E-10	0.33463847	0.056	0.037	9.9555E-06
Htatip2	4.5632E-10	0.6649621	0.077	0.055	1.0011E-05
Gpr34	4.6334E-10	0.63934693	0.051	0.033	1.0165E-05
Ankrd16	4.6364E-10	0.60654318	0.045	0.028	1.0172E-05
Gins4	4.6375E-10	0.55213737	0.072	0.05	1.0174E-05
Kctd13	4.6842E-10	0.67187895	0.035	0.021	1.0277E-05
Sun1	4.7873E-10	0.48534597	0.058	0.039	1.0503E-05

F2rl2	4.9214E-10	1.20379038	0.016	0.007	1.0797E-05
Foxj2	5.3267E-10	0.31866364	0.087	0.062	1.1686E-05
Pias3	5.4199E-10	0.61188073	0.03	0.017	1.1891E-05
Fam53a	5.4717E-10	0.4110876	0.054	0.035	1.2004E-05
Mettl21a	5.5225E-10	1.17704089	0.017	0.008	1.2116E-05
Naa16	5.5353E-10	0.49156537	0.063	0.043	1.2144E-05
Acoxl	5.8023E-10	1.14394972	0.012	0.005	1.273E-05
Cenpc1	5.8548E-10	0.4029308	0.083	0.06	1.2845E-05
Tecr	5.875E-10	0.32652468	0.201	0.169	1.2889E-05
Trim23	5.9207E-10	0.54769486	0.041	0.025	1.2989E-05
Arl4a	5.963E-10	0.32319053	0.144	0.114	1.3082E-05
Rraga	5.9944E-10	0.32125707	0.144	0.114	1.3151E-05
Gm14154	6.0625E-10	1.4349834	0.014	0.006	1.3301E-05
Ino80e	6.2125E-10	0.45410065	0.098	0.073	1.363E-05
Gmpr2	6.5226E-10	0.80018863	0.045	0.029	1.431E-05
Ppp2cb	6.6382E-10	0.34126848	0.121	0.093	1.4564E-05
Nr1h2	6.7171E-10	0.39109253	0.153	0.123	1.4737E-05
Dsel	6.7998E-10	1.02632663	0.017	0.008	1.4918E-05
Izumo4	7.1516E-10	0.60331067	0.031	0.018	1.569E-05
Fan1	7.5106E-10	0.95654301	0.015	0.007	1.6477E-05
4632415L05Ri	7.6826E-10	0.85577002	0.023	0.012	1.6855E-05
Klhl3	7.785E-10	1.14026032	0.011	0.004	1.708E-05
Dera	7.9239E-10	0.52589648	0.084	0.061	1.7384E-05
Mtor	8.021E-10	0.66768022	0.081	0.059	1.7597E-05
Xrcc4	8.9767E-10	0.55237451	0.047	0.03	1.9694E-05
AI597479	9.5336E-10	0.84569162	0.03	0.017	2.0916E-05
Cmtm4	9.6975E-10	0.97666934	0.027	0.015	2.1275E-05
Ccz1	9.8813E-10	0.34938112	0.158	0.128	2.1679E-05
Tarsl2	1.0308E-09	1.06580038	0.018	0.009	2.2615E-05
Ap1s2	1.0517E-09	0.43081213	0.152	0.123	2.3073E-05
Zmynd19	1.0639E-09	0.66467463	0.039	0.024	2.3341E-05
Tnfsf10	1.2138E-09	0.58929046	0.032	0.019	2.6629E-05
Tbc1d30	1.2586E-09	0.5551579	0.032	0.018	2.7613E-05
Bclaf3	1.3247E-09	0.55784599	0.09	0.067	2.9063E-05
Rrp36	1.3568E-09	0.65127608	0.056	0.038	2.9766E-05
Zfp64	1.359E-09	0.76764149	0.039	0.024	2.9816E-05
Endod1	1.3734E-09	0.65335524	0.071	0.05	3.013E-05
Zdhhc23	1.4495E-09	0.55011801	0.069	0.048	3.18E-05
Tdp2	1.4886E-09	0.25460255	0.094	0.069	3.2658E-05
Cops6	1.4888E-09	0.43664583	0.162	0.134	3.2662E-05
Rpusd3	1.5531E-09	0.92767659	0.013	0.005	3.4074E-05
Mypopos	1.8037E-09	0.86670818	0.016	0.007	3.9571E-05
Igbp1	1.8113E-09	0.33653363	0.197	0.166	3.9738E-05
Ubp1	1.917E-09	0.32205329	0.144	0.115	4.2057E-05
Sgcb	1.9543E-09	0.66613618	0.033	0.02	4.2875E-05
Mapk9	1.9813E-09	0.5135276	0.071	0.05	4.3469E-05
Cdk5	2.1068E-09	0.69740752	0.055	0.037	4.6222E-05
Paip1	2.2325E-09	0.41381843	0.088	0.065	4.898E-05

Pdzd8	2.2534E-09	0.29492569	0.121	0.094	4.9438E-05
Hemk1	2.4275E-09	0.94881241	0.022	0.011	5.3256E-05
Wdr44	2.4573E-09	0.30979128	0.083	0.06	5.391E-05
Fam83d	2.5009E-09	1.12697971	0.014	0.006	5.4867E-05
Aktip	2.5506E-09	0.58179078	0.038	0.023	5.5958E-05
Aimp2	2.582E-09	0.7355811	0.043	0.027	5.6646E-05
Sec24c	2.6708E-09	0.35858346	0.132	0.104	5.8595E-05
Appl2	2.6883E-09	0.78077328	0.04	0.025	5.8979E-05
Nrgn	2.761E-09	0.45675829	0.057	0.038	6.0574E-05
Zbtb5	2.7778E-09	0.6500924	0.042	0.026	6.0941E-05
Arnt2	2.7793E-09	0.94595153	0.015	0.007	6.0975E-05
Aasdhppt	2.8671E-09	0.44034521	0.079	0.057	6.2901E-05
Ccnk	2.9006E-09	0.38020794	0.14	0.112	6.3637E-05
Acsl4	2.9277E-09	0.27384926	0.203	0.171	6.4231E-05
Thumpd1	2.966E-09	0.35712119	0.112	0.085	6.5071E-05
Rtn4rl1	3.1647E-09	0.7422375	0.02	0.01	6.9429E-05
Ino80b	3.2342E-09	0.43338482	0.098	0.074	7.0956E-05
Dnlz	3.2409E-09	0.54493784	0.078	0.056	7.1102E-05
Stn1	3.413E-09	0.62065659	0.059	0.04	7.4877E-05
Slc35a1	3.4591E-09	0.70277996	0.034	0.021	7.589E-05
Sec23b	3.4819E-09	0.26160178	0.153	0.123	7.639E-05
B3galt4	3.5008E-09	1.1212949	0.017	0.008	7.6804E-05
Arel1	3.6084E-09	0.4397296	0.064	0.044	7.9165E-05
Zfp688	3.6253E-09	0.45010661	0.041	0.026	7.9536E-05
Nosip	3.7067E-09	0.48787906	0.075	0.054	8.1322E-05
Rrnad1	4.0007E-09	0.45707843	0.053	0.035	8.7772E-05
Zfp330	4.0407E-09	0.36671908	0.157	0.129	8.865E-05
Xkr6	4.1314E-09	0.79243699	0.017	0.008	9.0638E-05
Trmt13	4.1562E-09	0.32955848	0.083	0.06	9.1184E-05
Pcgf2	4.1888E-09	1.11468205	0.013	0.006	9.1898E-05
Smn1	4.1931E-09	0.43198068	0.108	0.083	9.1993E-05
Crlf2	4.4228E-09	0.25258139	0.262	0.231	9.7032E-05
Rprd1b	4.4345E-09	0.29118511	0.115	0.088	9.7288E-05
Mrpl50	4.5315E-09	0.38307784	0.084	0.061	9.9417E-05
Gm17477	4.564E-09	0.6461775	0.02	0.01	0.00010013
Zzef1	4.5876E-09	0.26327942	0.145	0.116	0.00010065
Rnf187	4.669E-09	0.30595662	0.166	0.136	0.00010243
Gbf1	4.7303E-09	0.4131818	0.098	0.074	0.00010378
Erc1	4.8296E-09	0.4361961	0.039	0.024	0.00010596
Zfc3h1	4.8977E-09	0.42323811	0.121	0.095	0.00010745
Spast	5.0035E-09	0.4991969	0.087	0.065	0.00010977
Rnf219	5.0286E-09	0.56089213	0.032	0.019	0.00011032
Gtf2f1	5.4318E-09	0.34930486	0.164	0.135	0.00011917
Cnot11	5.5772E-09	0.44023245	0.079	0.058	0.00012236
Slc52a2	5.5987E-09	0.72606318	0.034	0.021	0.00012283
Ino80	5.6914E-09	0.41356759	0.12	0.094	0.00012486
Cog4	5.7046E-09	0.43776063	0.069	0.049	0.00012515
Zcchc8	5.9226E-09	0.48696457	0.083	0.061	0.00012994

Trib3	6.071E-09	0.89886612	0.025	0.014	0.00013319
Fam78a	6.0779E-09	0.35872356	0.035	0.021	0.00013334
Pigf	6.3252E-09	0.77082634	0.041	0.026	0.00013877
St6galnac6	6.4605E-09	0.74993257	0.051	0.034	0.00014174
Golga3	6.7098E-09	0.41876467	0.088	0.065	0.00014721
Actr1a	6.7305E-09	0.3793103	0.118	0.093	0.00014766
Zfp414	7.0976E-09	0.83997847	0.034	0.021	0.00015571
Acot2	7.1457E-09	0.35279985	0.053	0.035	0.00015677
Chst12	7.286E-09	0.42160925	0.061	0.042	0.00015985
Zfp574	7.5132E-09	0.54712556	0.044	0.028	0.00016483
Uggt1	7.5865E-09	0.3671117	0.129	0.102	0.00016644
Tmem106b	7.8213E-09	0.32074648	0.099	0.075	0.00017159
Uxt	7.9808E-09	0.63394271	0.086	0.064	0.00017509
Mtrr	8.4141E-09	0.55168884	0.04	0.025	0.0001846
Gm5547	8.509E-09	0.47108374	0.113	0.089	0.00018668
Josd2	8.7169E-09	0.51667419	0.081	0.06	0.00019124
Rbm10	9.657E-09	0.45554949	0.07	0.05	0.00021186
Ak2	9.7023E-09	0.31227538	0.219	0.19	0.00021286
Ppm1k	1.0163E-08	0.25169799	0.062	0.043	0.00022296
Sec13	1.0317E-08	0.39090927	0.135	0.109	0.00022634
Mfap3	1.0358E-08	0.5636268	0.062	0.043	0.00022725
Tbcc	1.0665E-08	0.75515724	0.056	0.039	0.00023399
Kptn	1.0845E-08	0.69977316	0.042	0.027	0.00023794
Dnajc4	1.0879E-08	0.83103009	0.054	0.037	0.00023869
Mllt11	1.1264E-08	0.89056745	0.022	0.012	0.00024711
Rit1	1.1274E-08	0.60299766	0.058	0.04	0.00024735
Tbc1d10a	1.1276E-08	0.36557901	0.138	0.112	0.00024739
Pfn2	1.1596E-08	1.09646335	0.011	0.005	0.00025441
Med14	1.1607E-08	0.35429673	0.112	0.087	0.00025464
2010015M23F	1.1641E-08	1.33128819	0.013	0.006	0.0002554
Dxo	1.1773E-08	0.60499712	0.055	0.038	0.00025829
Tmem42	1.1782E-08	0.59784303	0.043	0.028	0.00025849
Zfp637	1.2577E-08	0.78757344	0.023	0.013	0.00027594
Mrpl19	1.2882E-08	0.45473596	0.11	0.086	0.00028262
Mcm9	1.2957E-08	0.33821903	0.056	0.038	0.00028426
Gm48855	1.3883E-08	1.16716402	0.01	0.004	0.00030458
Cdc123	1.4067E-08	0.41426671	0.1	0.077	0.00030861
Prdm10	1.4322E-08	0.53130699	0.05	0.033	0.0003142
Otub1	1.502E-08	0.30684011	0.197	0.169	0.00032951
Snx25	1.5062E-08	0.55974516	0.034	0.021	0.00033045
Krr1	1.517E-08	0.2724216	0.095	0.072	0.00033282
Rnf113a1	1.5606E-08	1.29534098	0.01	0.004	0.00034238
Pop7	1.6074E-08	0.48016846	0.12	0.096	0.00035264
Kctd10	1.6602E-08	0.31978064	0.114	0.089	0.00036422
Golga7	1.6713E-08	0.36130645	0.177	0.15	0.00036667
Gps2	1.7109E-08	0.29110689	0.174	0.145	0.00037536
Rtl8b	1.7338E-08	0.90553109	0.033	0.02	0.00038038
Ppp1r14b	1.7379E-08	0.32465391	0.199	0.17	0.00038127

Ide	1.7452E-08	0.33179564	0.096	0.072	0.00038287
Kdm3a	1.7777E-08	0.25366569	0.127	0.1	0.00039001
Zfp931	1.8475E-08	0.8004331	0.029	0.017	0.00040533
Ccl25	1.9321E-08	0.50707449	0.041	0.026	0.00042389
Eci2	1.9741E-08	0.62123077	0.087	0.067	0.0004331
Srp68	1.99E-08	0.42995565	0.102	0.079	0.00043658
Mzt1	2.0131E-08	0.33570117	0.112	0.087	0.00044166
Zfp141	2.0174E-08	0.78832894	0.023	0.013	0.00044261
Rnf6	2.0223E-08	0.27910829	0.198	0.168	0.00044368
Zbtb8os	2.0269E-08	0.53172731	0.111	0.088	0.00044469
Eid2	2.1297E-08	1.05679806	0.014	0.006	0.00046722
Rpap3	2.1819E-08	0.41654249	0.059	0.041	0.00047869
Nfkbil1	2.2034E-08	0.55207425	0.051	0.034	0.0004834
Agap3	2.2428E-08	0.61104412	0.056	0.039	0.00049205
Krba1	2.2463E-08	0.70930709	0.021	0.012	0.00049282
Nsmce2	2.3055E-08	0.50975691	0.1	0.078	0.0005058
Psme2b	2.3669E-08	0.2599765	0.048	0.032	0.00051928
Snapc3	2.4433E-08	0.37660636	0.059	0.041	0.00053603
Mettl4	2.444E-08	0.77759884	0.02	0.011	0.00053618
Trappc5	2.6137E-08	0.32467747	0.129	0.104	0.00057341
Cnot8	2.6846E-08	0.36093854	0.098	0.075	0.00058897
Secisbp2	2.6972E-08	0.32144422	0.099	0.076	0.00059175
Ddhd2	2.7684E-08	0.35710756	0.098	0.075	0.00060735
Hdhd5	2.7688E-08	0.45047663	0.031	0.019	0.00060745
Thyn1	2.7858E-08	0.76031191	0.05	0.034	0.00061118
Mcm3ap	2.8849E-08	0.36165734	0.057	0.039	0.00063292
Atg5	3.072E-08	0.32270035	0.111	0.087	0.00067397
Zswim1	3.1385E-08	0.70912296	0.022	0.012	0.00068855
Cherp	3.1981E-08	0.4148624	0.09	0.068	0.00070164
Cnst	3.2473E-08	0.33471377	0.048	0.032	0.00071241
Ccdc166	3.2585E-08	0.99721065	0.01	0.004	0.00071488
Jazf1	3.2827E-08	1.28089634	0.012	0.005	0.00072019
Ddx10	3.3427E-08	0.29242387	0.121	0.095	0.00073336
Smug1	3.9042E-08	0.6240033	0.03	0.018	0.00085654
Gpr55	3.9482E-08	0.27396298	0.1	0.076	0.0008662
Fam207a	4.2404E-08	0.4090824	0.094	0.072	0.00093031
Dusp14	4.2559E-08	0.6982498	0.024	0.013	0.00093371
Ei24	4.4609E-08	0.43260587	0.079	0.059	0.00097867
Ppcs	4.4781E-08	0.67403199	0.024	0.014	0.00098244
Hist2h2bb	4.5261E-08	0.43789709	0.037	0.023	0.00099299
Rfwd3	4.5267E-08	0.49179087	0.053	0.036	0.00099311
Adarb1	4.6434E-08	0.95858804	0.017	0.008	0.00101871
Wrap53	4.7062E-08	0.91623499	0.02	0.011	0.0010325
Klhl20	5.4963E-08	0.47950747	0.048	0.033	0.00120582
Wdr83os	5.6198E-08	0.27353307	0.234	0.207	0.00123292
Nr1d1	5.656E-08	0.5004218	0.04	0.026	0.00124088
Rcn1	5.6676E-08	0.42694583	0.029	0.017	0.00124341
Dpm2	5.6859E-08	0.43580398	0.117	0.094	0.00124742

B230217O12R	5.7147E-08	0.88805978	0.012	0.005	0.00125375
Ankrd52	5.8597E-08	0.49471201	0.045	0.03	0.00128557
Lypla1	6.0851E-08	0.31360167	0.128	0.103	0.001335
Rad54l2	6.1802E-08	0.28361005	0.087	0.065	0.00135587
Dus1l	6.3094E-08	0.66754333	0.043	0.028	0.00138421
Commd3	6.468E-08	0.42959728	0.126	0.102	0.00141901
Leng9	6.6627E-08	0.44994623	0.045	0.03	0.00146173
Sqle	6.8512E-08	0.5380007	0.069	0.051	0.00150307
Hsd17b10	7.1321E-08	0.4446397	0.132	0.109	0.00156471
Ccsap	7.1778E-08	0.56786221	0.031	0.019	0.00157475
Dtx3	7.221E-08	0.39651698	0.087	0.066	0.00158421
Trim21	7.2236E-08	0.55617402	0.029	0.017	0.00158478
Ddx25	7.5652E-08	0.47112361	0.017	0.009	0.00165972
Nelfa	7.5882E-08	0.33641246	0.063	0.045	0.00166477
Dtnb	7.6512E-08	0.33525915	0.053	0.036	0.00167859
Slc35e1	7.957E-08	0.36057011	0.099	0.077	0.00174569
BC003965	8.038E-08	0.35745233	0.074	0.055	0.00176347
Rabep2	8.0516E-08	0.30548489	0.054	0.037	0.00176645
Asb6	8.1797E-08	0.6004802	0.045	0.03	0.00179454
Coa6	8.3385E-08	0.48768696	0.072	0.054	0.00182938
Ddx28	8.4188E-08	0.66916757	0.039	0.025	0.00184699
Mrps35	8.4769E-08	0.4636211	0.073	0.054	0.00185975
Rassf7	8.7692E-08	1.06764886	0.012	0.005	0.00192387
Ube2q1	8.8025E-08	0.26120449	0.17	0.144	0.00193117
Fbxl6	8.808E-08	0.56436671	0.062	0.044	0.00193239
Ttc4	8.8462E-08	0.31326494	0.07	0.051	0.00194077
Zfp61	8.9742E-08	1.12858933	0.011	0.005	0.00196884
Rad17	9.3292E-08	0.34302415	0.084	0.063	0.00204673
Ddx1	9.4321E-08	0.40382301	0.101	0.079	0.0020693
Selenoi	9.8032E-08	0.37808241	0.085	0.064	0.00215072
Srebf1	9.8359E-08	0.41438433	0.082	0.062	0.0021579
Bcdin3d	9.8791E-08	0.46989043	0.026	0.015	0.00216737
Foxj3	9.899E-08	0.2599329	0.119	0.094	0.00217175
Yrdc	1.0258E-07	0.31911133	0.137	0.112	0.00225058
E130308A19R	1.0312E-07	0.42569392	0.039	0.025	0.00226226
Hinfp	1.0551E-07	0.36499758	0.061	0.044	0.0023147
Rpl9-ps6	1.0739E-07	0.56195502	0.076	0.057	0.00235598
Gabarapl1	1.0832E-07	0.33540987	0.194	0.169	0.00237654
B3galt5	1.0917E-07	0.26956506	0.038	0.024	0.00239507
Ttbk2	1.1375E-07	0.4623894	0.031	0.019	0.00249547
Inip	1.165E-07	0.36400205	0.089	0.068	0.00255583
Glrx2	1.175E-07	0.36943408	0.146	0.122	0.00257774
Mrpl55	1.184E-07	0.65394989	0.074	0.056	0.00259768
Psmf1	1.1946E-07	0.47415643	0.075	0.056	0.00262082
Thap4	1.2198E-07	0.88379606	0.028	0.017	0.00267619
Gosr1	1.2204E-07	0.46880127	0.09	0.069	0.00267744
Orc3	1.2826E-07	0.51282592	0.06	0.043	0.0028139
Tdrkh	1.3165E-07	0.90649893	0.02	0.011	0.00288829

Cdk7	1.3595E-07	0.42808459	0.058	0.041	0.00298255
Eif2ak1	1.3918E-07	0.36062884	0.105	0.083	0.00305341
Triap1	1.3922E-07	0.41142463	0.077	0.058	0.00305432
Zfp68	1.4006E-07	0.28583981	0.092	0.07	0.0030727
Fez2	1.4279E-07	0.74028192	0.04	0.027	0.00313259
Mrps22	1.4376E-07	0.73208713	0.04	0.027	0.00315387
Ndufs1	1.4449E-07	0.27186165	0.111	0.088	0.00316989
Smpd1	1.5418E-07	0.57725584	0.042	0.028	0.00338252
Il12rb1	1.5438E-07	0.54946327	0.031	0.019	0.00338704
Tarbp2	1.6351E-07	0.52004565	0.059	0.042	0.00358734
Ttll12	1.6458E-07	0.84831124	0.025	0.015	0.00361062
Tfpt	1.6923E-07	0.56281551	0.043	0.029	0.00371275
Mapk8ip3	1.696E-07	0.28109359	0.096	0.074	0.00372084
Sf3a3	1.7372E-07	0.36201591	0.119	0.096	0.00381135
Orai2	1.7478E-07	0.36993471	0.067	0.049	0.00383459
Snai3	1.7894E-07	1.03070717	0.01	0.004	0.00392584
Pdzd11	1.7942E-07	0.64370057	0.075	0.057	0.00393634
Pef1	1.8284E-07	0.52181977	0.056	0.039	0.00401126
Ttc1	1.8486E-07	0.33899661	0.138	0.114	0.00405561
Scfd1	1.8823E-07	0.29662156	0.143	0.118	0.00412956
1700066M21F	1.9094E-07	0.89911532	0.012	0.006	0.004189
Cdkn2a	1.922E-07	0.98499345	0.014	0.007	0.0042166
Klc3	1.9595E-07	1.04073145	0.012	0.005	0.00429891
Dnajc30	1.9992E-07	0.58992105	0.061	0.044	0.00438605
Clp1	2.0023E-07	0.26145945	0.088	0.067	0.00439285
Tmem101	2.0951E-07	0.83387327	0.026	0.016	0.00459636
Phb2	2.1432E-07	0.2681864	0.212	0.187	0.00470191
Gcfc2	2.1616E-07	0.73735261	0.021	0.011	0.00474233
Ola1	2.1662E-07	0.34500743	0.18	0.155	0.00475244
Clns1a	2.1913E-07	0.26559151	0.114	0.091	0.00480748
Slc35g1	2.2349E-07	0.62640376	0.019	0.01	0.00490319
Zfp869	2.333E-07	0.56354734	0.027	0.017	0.00511832
Taf12	2.4561E-07	0.40879078	0.078	0.059	0.00538834
Pak1ip1	2.4846E-07	0.25503996	0.212	0.185	0.00545106
Cib1	2.5352E-07	0.27864824	0.17	0.145	0.0055619
Agap2	2.5486E-07	0.3928645	0.03	0.019	0.00559134
Rnf34	2.6079E-07	0.39935253	0.079	0.06	0.00572146
Slc25a16	2.6324E-07	0.45643737	0.042	0.028	0.00577514
Lrch3	2.6758E-07	0.27589412	0.111	0.088	0.00587035
Rnf40	2.7539E-07	0.3189091	0.078	0.059	0.00604187
Naxe	2.7667E-07	0.58245959	0.096	0.076	0.00606992
Myo10	2.7808E-07	0.94066616	0.022	0.013	0.00610091
Pex11b	2.9505E-07	0.67902786	0.026	0.016	0.00647305
Tube1	3.0726E-07	1.1403693	0.012	0.005	0.006741
Lnx2	3.1314E-07	0.51176279	0.059	0.043	0.00687008
Zw10	3.1679E-07	0.52681903	0.041	0.027	0.00695012
Fam8a1	3.2929E-07	0.38494528	0.061	0.044	0.00722432
Stk16	3.3199E-07	0.52450777	0.078	0.06	0.00728362

Minpp1	3.3248E-07	0.45138285	0.038	0.025	0.00729435
Itgb1bp1	3.39E-07	0.44478354	0.088	0.068	0.00743725
Gstt1	3.5507E-07	1.2947783	0.01	0.004	0.00778989
Anln	3.5825E-07	1.33178927	0.01	0.004	0.00785956
Rnft1	3.7649E-07	0.45374269	0.078	0.059	0.0082599
Zfp84	3.7715E-07	0.36217665	0.067	0.049	0.00827425
Mtif3	3.8631E-07	0.47449588	0.038	0.025	0.00847527
Rbbp5	3.8652E-07	0.28663443	0.057	0.04	0.00847989
Ndufa10	3.9732E-07	0.31338333	0.205	0.181	0.00871672
Alkbh6	4.0192E-07	0.34424777	0.077	0.058	0.00881774
Csrp1	4.028E-07	0.28761996	0.159	0.134	0.00883695
Trappc6a	4.1093E-07	0.48174529	0.127	0.106	0.00901537
Spag7	4.1529E-07	0.42478866	0.132	0.11	0.00911111
Naxd	4.1609E-07	0.29681011	0.111	0.089	0.00912864
Stk25	4.182E-07	0.39690805	0.057	0.041	0.00917499
Nup35	4.1844E-07	0.60358179	0.025	0.015	0.00918018
Seh1l	4.1903E-07	0.32893761	0.123	0.101	0.00919312
Lcmt2	4.3032E-07	0.8222526	0.023	0.014	0.00944085
Chm	4.416E-07	0.26882394	0.097	0.076	0.0096883
Mrpl27	4.6187E-07	0.5351768	0.1	0.08	0.01013304
Lysmd1	4.8478E-07	0.56556443	0.025	0.015	0.01063559
Cnp	4.8891E-07	0.30892951	0.087	0.067	0.01072613
Iqgap2	5.0543E-07	0.3068037	0.169	0.145	0.01108861
Ubxn8	5.1645E-07	0.461026	0.066	0.049	0.01133046
Hcfc1r1	5.3945E-07	0.34379984	0.188	0.166	0.01183499
Pstk	5.4046E-07	0.33504572	0.051	0.035	0.01185708
Shkbp1	5.461E-07	0.48627903	0.073	0.055	0.01198078
Mettl26	5.5E-07	0.4824898	0.078	0.059	0.01206655
Ficd	5.6169E-07	1.07186392	0.02	0.011	0.01232298
Hid1	5.6548E-07	0.75728547	0.018	0.01	0.01240613
Fmc1	6.1161E-07	0.46047603	0.104	0.084	0.01341811
Mlf2	6.2137E-07	0.36510331	0.174	0.152	0.01363234
Mtfmt	6.2672E-07	0.55778339	0.034	0.022	0.01374969
Abcc1	6.2767E-07	0.33883496	0.112	0.091	0.01377036
Ndufb2	6.5054E-07	0.29228539	0.237	0.214	0.01427223
Hspa14	6.5832E-07	0.29968754	0.149	0.125	0.01444284
Gm45606	6.7669E-07	1.1267271	0.01	0.004	0.01484585
Mettl14	6.9213E-07	0.27946619	0.065	0.048	0.01518461
Mbip	6.9375E-07	0.52120718	0.053	0.038	0.01522015
H2-Ke6	7.0581E-07	0.32871858	0.11	0.088	0.0154848
Ccdc47	7.2113E-07	0.33593515	0.122	0.099	0.01582089
Zfp687	7.6544E-07	0.38986207	0.042	0.028	0.01679306
Pear1	7.7012E-07	0.38077489	0.032	0.021	0.01689561
Pphln1	7.7015E-07	0.46215284	0.078	0.06	0.01689636
Bod1	7.711E-07	0.58586929	0.043	0.03	0.01691715
Tada3	7.8008E-07	0.55577382	0.048	0.033	0.01711415
Kbtbd4	7.9063E-07	0.52730806	0.028	0.017	0.01734573
Asb8	8.4179E-07	0.48940006	0.047	0.033	0.01846806

Ccr9	8.6225E-07	0.99341739	0.036	0.024	0.01891701
Zfp160	8.7092E-07	0.36949895	0.047	0.033	0.01910712
A830010M20F	8.8511E-07	0.92887536	0.013	0.006	0.0194185
Dok2	8.9491E-07	0.34546355	0.104	0.083	0.01963346
Dnmt1	8.956E-07	0.26776354	0.089	0.069	0.01964853
Mthfd2l	9.2003E-07	0.59865342	0.029	0.018	0.02018446
Ppil2	9.2806E-07	0.29418984	0.113	0.091	0.02036074
Cdc23	9.4644E-07	0.34343818	0.04	0.027	0.02076393
Rab18	1.011E-06	0.27119363	0.171	0.147	0.02217957
Dbr1	1.0248E-06	0.41001743	0.059	0.043	0.02248412
Svil	1.0662E-06	0.34321273	0.12	0.098	0.02339111
Bpnt1	1.1112E-06	0.29593603	0.097	0.076	0.02437952
Lin37	1.1362E-06	0.53135387	0.048	0.034	0.02492689
Atpaf1	1.1638E-06	0.49748159	0.022	0.013	0.02553183
Exoc8	1.1907E-06	0.73718132	0.024	0.014	0.02612304
Ap4e1	1.192E-06	0.41172379	0.038	0.026	0.02615209
Dhx32	1.266E-06	0.65421528	0.025	0.015	0.0277741
Lrrc41	1.2835E-06	0.3881606	0.082	0.064	0.02815951
Cul5	1.3E-06	0.39420654	0.12	0.099	0.02852074
Taf9b	1.3515E-06	0.64686544	0.026	0.016	0.02965043
Zbtb17	1.4326E-06	0.48673322	0.064	0.048	0.03142954
Btbd10	1.4505E-06	0.41715619	0.061	0.045	0.03182326
Coq3	1.4602E-06	0.9179848	0.02	0.011	0.03203479
Tm9sf1	1.5292E-06	0.56444231	0.03	0.019	0.03354821
Eef1akmt2	1.6524E-06	0.45117565	0.042	0.029	0.03625281
Gtf3c6	1.7265E-06	0.38240256	0.12	0.099	0.03787816
Med25	1.7326E-06	0.40538286	0.074	0.057	0.03801131
Rfc3	1.7427E-06	0.38678544	0.055	0.04	0.03823411
Slc45a4	1.8223E-06	0.48657209	0.049	0.035	0.03998031
Plk4	1.8776E-06	0.28889215	0.073	0.056	0.04119165
Hadh	1.8843E-06	0.36792831	0.076	0.058	0.04133889
Fbxo34	1.9174E-06	0.32067142	0.119	0.098	0.04206495
Zfp51	1.9231E-06	0.49607313	0.038	0.026	0.04219155
Kantr	1.9397E-06	0.5002634	0.026	0.016	0.0425556
Tyk2	2.014E-06	0.49108455	0.069	0.053	0.04418512
Smim27	2.0223E-06	0.33967373	0.102	0.082	0.04436626
Maz	2.0795E-06	0.34560283	0.102	0.082	0.04562303
Exoc2	2.0811E-06	0.26971652	0.096	0.076	0.04565617
3830403N18R	2.102E-06	1.03120751	0.012	0.006	0.04611564
Tbl2	2.1324E-06	0.54965703	0.023	0.014	0.04678363
Bag6	2.1509E-06	0.35910571	0.144	0.123	0.04718869
Cnnm2	2.1693E-06	0.26308683	0.075	0.057	0.04759218
Haus6	2.1776E-06	0.44899505	0.049	0.035	0.04777476
Dars2	2.1892E-06	0.55644097	0.038	0.026	0.0480287
Zfp780b	2.2065E-06	0.4813491	0.033	0.021	0.0484083
Ntmt1	2.2093E-06	0.65215088	0.032	0.021	0.04847036
Mrs2	2.2166E-06	0.45192243	0.055	0.04	0.04862981
Rab2b	2.2786E-06	0.3361732	0.056	0.041	0.04999016

Lax1	2.2907E-06	0.30181846	0.049	0.035	0.05025649
Gm31597	2.3202E-06	0.67139744	0.032	0.021	0.05090263
Fastk	2.3263E-06	0.35833616	0.047	0.033	0.05103656
Fam192a	2.3348E-06	0.2963663	0.13	0.108	0.05122232
Hat1	2.4254E-06	0.41763064	0.131	0.111	0.05321034
Wdr92	2.4547E-06	0.66646701	0.021	0.012	0.05385267
Dnajc24	2.5361E-06	0.54087553	0.053	0.039	0.05563844
Ankmy2	2.5471E-06	0.45904274	0.033	0.022	0.05588039
Gm6133	2.5735E-06	0.5768327	0.041	0.029	0.05646088
Ubl7	2.6613E-06	0.30619156	0.082	0.064	0.05838699
B230354K17R	2.6713E-06	0.68838278	0.022	0.013	0.05860502
Clpp	2.6946E-06	0.44547163	0.06	0.045	0.05911756
Rab23	2.8809E-06	0.49463015	0.017	0.009	0.06320396
Nipsnap3b	2.9345E-06	0.43159606	0.113	0.094	0.06438077
Polr2i	2.9533E-06	0.35557954	0.148	0.128	0.06479188
Wdr53	2.9619E-06	0.5659869	0.035	0.024	0.06498184
Alkbh8	2.9646E-06	0.33390591	0.044	0.03	0.06503969
CAAA0111838	3.0599E-06	0.38460111	0.114	0.095	0.06713029
Mtbp	3.2096E-06	0.54402827	0.024	0.014	0.07041446
Gm11831	3.2838E-06	0.94953273	0.011	0.005	0.07204343
Tmem192	3.316E-06	0.45013319	0.102	0.083	0.07275054
Zfp846	3.3325E-06	0.63242236	0.021	0.013	0.07311238
Dyrk1b	3.3846E-06	0.46025691	0.036	0.024	0.07425446
Platr25	3.3923E-06	0.71479045	0.018	0.01	0.07442302
Rnf5	3.4544E-06	0.57167232	0.06	0.046	0.07578677
Ints11	3.4823E-06	0.5053796	0.046	0.033	0.07639782
Rnaseh1	3.4997E-06	0.51596722	0.037	0.025	0.07678023
Cwc27	3.5432E-06	0.40637666	0.057	0.043	0.07773495
Abhd10	3.6669E-06	0.40145436	0.056	0.041	0.0804481
Cd101	3.7485E-06	0.48248367	0.032	0.021	0.08223824
Gatd1	3.8138E-06	0.38971089	0.068	0.052	0.08367008
Zfp335os	3.817E-06	0.86138868	0.012	0.006	0.08374154
Mapk1ip1	3.8674E-06	0.43233773	0.031	0.02	0.08484637
Bloc1s6	3.8806E-06	0.47242548	0.062	0.047	0.0851362
Zfp580	4.0582E-06	0.90023514	0.016	0.009	0.08903384
Asb7	4.0674E-06	0.52329677	0.024	0.015	0.08923408
Wdr61	4.1305E-06	0.33898492	0.129	0.108	0.0906184
Mrpl14	4.1608E-06	0.38372467	0.155	0.136	0.09128404
Tmem80	4.1918E-06	0.54747297	0.042	0.029	0.09196407
D17H6S53E	4.2146E-06	0.60187176	0.03	0.02	0.09246459
Tmsb15b1	4.2215E-06	0.43469838	0.031	0.02	0.09261588
Psmd14	4.3064E-06	0.31252175	0.196	0.175	0.09447721
Gas2	4.3678E-06	0.74917065	0.016	0.009	0.09582471
Setdb2	4.643E-06	0.33850151	0.044	0.031	0.10186295
Rrp8	4.701E-06	0.28208766	0.069	0.053	0.10313553
Chmp7	4.711E-06	0.40475586	0.041	0.029	0.10335398
Rnf113a2	4.8148E-06	0.29953432	0.072	0.055	0.10563159
2610001J05Ril	4.8483E-06	0.30716149	0.135	0.114	0.10636743

Intu	4.8602E-06	0.97471462	0.017	0.01	0.10662819
Ndc80	4.9702E-06	0.66277327	0.021	0.013	0.10904112
Aldh7a1	5.0235E-06	0.47362045	0.024	0.015	0.11021161
Tgds	5.0359E-06	0.68005041	0.02	0.012	0.11048283
Zfp825	5.2198E-06	0.70572318	0.018	0.011	0.11451717
Pglyrp2	5.2427E-06	0.47652562	0.019	0.011	0.11501939
Washc3	5.3307E-06	0.41794436	0.103	0.085	0.1169501
Zfp738	5.35E-06	0.42572142	0.045	0.032	0.11737256
Mocs2	5.3692E-06	0.35610001	0.1	0.081	0.11779505
Mrpl49	5.387E-06	0.49185278	0.051	0.037	0.11818542
Gm4924	5.4565E-06	0.75089945	0.011	0.005	0.11970935
Cog6	5.4574E-06	0.34165088	0.045	0.032	0.11972968
3110002H16R	5.5718E-06	0.48788616	0.041	0.029	0.12223914
Eipr1	5.6207E-06	0.25888195	0.089	0.071	0.12331165
2700097O09R	5.7407E-06	0.26455921	0.041	0.028	0.12594589
Celsr1	5.9262E-06	0.54176919	0.021	0.013	0.13001533
Snx15	6.0458E-06	0.64725137	0.034	0.023	0.13263952
Psmg3	6.1322E-06	0.38697815	0.066	0.051	0.13453519
Alg2	6.2617E-06	0.71135319	0.026	0.016	0.13737472
Brip1os	6.3481E-06	0.52772871	0.042	0.03	0.13927007
Gna11	6.5332E-06	0.51521438	0.031	0.021	0.14333147
Sart3	6.8526E-06	0.29782661	0.089	0.071	0.15033893
Snx19	6.8739E-06	0.5197297	0.035	0.024	0.15080674
Plpp5	6.8882E-06	0.61083644	0.046	0.033	0.15112061
Polr3c	7.222E-06	0.29078382	0.1	0.081	0.15844443
Ginm1	7.3074E-06	0.32855521	0.098	0.08	0.16031674
Arhgef10	7.4432E-06	0.70657243	0.03	0.02	0.16329545
Gbp6	7.508E-06	0.48201312	0.022	0.014	0.16471843
Faap24	7.5678E-06	0.61871505	0.027	0.018	0.16603084
Perp	7.6995E-06	0.57418543	0.016	0.009	0.16891978
Zfpm1	7.7109E-06	0.31868574	0.049	0.035	0.16916997
Smim19	7.9265E-06	0.38486211	0.08	0.063	0.17390036
Gm20045	8.1444E-06	0.44888438	0.022	0.013	0.1786806
Zfp729a	8.1546E-06	0.29056845	0.058	0.043	0.17890328
Ercc3	8.1697E-06	0.29908515	0.079	0.062	0.17923509
Nckap1	8.1773E-06	0.55041823	0.017	0.01	0.17940267
Hace1	8.5427E-06	0.36000387	0.047	0.034	0.18741854
Coa4	8.7921E-06	0.80948396	0.033	0.022	0.19288919
Msantd4	8.8144E-06	0.39652211	0.065	0.05	0.1933798
Rbm41	9.0433E-06	0.48989576	0.032	0.022	0.19840116
Rmdn2	9.067E-06	0.82054961	0.014	0.008	0.19892099
Zkscan1	9.1009E-06	0.43808366	0.064	0.049	0.19966521
Pigc	9.6667E-06	0.3734211	0.044	0.032	0.21207751
Trafd1	1.0284E-05	0.2782959	0.162	0.142	0.22561967
Tex30	1.0782E-05	0.2893012	0.068	0.052	0.23654898
Wdcp	1.0889E-05	0.53649223	0.024	0.015	0.23889655
6030458C11R	1.1135E-05	0.44340955	0.033	0.022	0.24428497
Pex19	1.1543E-05	0.38473022	0.058	0.044	0.25323877

Zfp39	1.157E-05	0.57781335	0.016	0.009	0.25383548
Mad2l1	1.1601E-05	0.5476095	0.032	0.021	0.2545151
CAAA0114733	1.1855E-05	1.40898518	0.011	0.005	0.26008684
Ostm1	1.1893E-05	0.31604089	0.092	0.074	0.26091712
Gm14305	1.1931E-05	0.70037903	0.019	0.011	0.26175701
Gm26563	1.1941E-05	0.87891182	0.011	0.005	0.26198063
D730003I15Ri	1.1998E-05	0.38239622	0.048	0.035	0.26322204
Hagh	1.2109E-05	0.59574687	0.068	0.053	0.2656604
Adprhl2	1.2361E-05	0.3320015	0.046	0.033	0.27117815
Dtx4	1.2691E-05	0.35511154	0.086	0.069	0.2784276
Ppt2	1.2924E-05	0.51684949	0.073	0.058	0.28353716
Nfs1	1.3162E-05	0.48063097	0.042	0.03	0.28876038
Usp50	1.3327E-05	0.44282701	0.05	0.037	0.29237492
Naa38	1.3525E-05	0.36493413	0.146	0.127	0.29672278
Sp3os	1.3587E-05	0.343717	0.052	0.038	0.29807663
Ric8a	1.3605E-05	0.3635407	0.097	0.08	0.29848436
Haghl	1.3704E-05	0.62081774	0.03	0.02	0.30064439
Chchd4	1.4249E-05	0.50490139	0.057	0.044	0.31260178
Exo5	1.4304E-05	0.7071733	0.017	0.01	0.31380871
Mrps18a	1.5041E-05	0.45006668	0.13	0.113	0.32998884
Foxo4	1.5273E-05	0.43563654	0.029	0.019	0.33507851
Pstpip1	1.5474E-05	0.28200058	0.139	0.119	0.33948226
2410131K14R	1.568E-05	0.54944629	0.034	0.024	0.34400541
Dzip1	1.5684E-05	0.7564074	0.011	0.006	0.3441001
Bloc1s3	1.5731E-05	0.6922878	0.026	0.017	0.34511577
Arid3a	1.5746E-05	0.40128488	0.06	0.046	0.34545286
Ehbp1l1	1.737E-05	0.3047629	0.095	0.078	0.38107442
Yars	1.7615E-05	0.37349817	0.089	0.072	0.38645469
Micu3	1.8528E-05	0.38927772	0.036	0.025	0.40647935
Timeless	1.8558E-05	0.86233681	0.022	0.014	0.40714324
Osbp	1.8617E-05	0.2567641	0.104	0.085	0.40843532
Uty	1.8707E-05	0.35273223	0.065	0.05	0.41041124
Pmm1	1.8779E-05	0.59793527	0.043	0.031	0.41200308
Gm28935	1.8958E-05	0.43151888	0.021	0.013	0.41592715
Mettl18	1.9638E-05	0.61259972	0.013	0.007	0.43084078
Itpa	1.9782E-05	0.43373363	0.062	0.048	0.43400594
Sertad3	2.0085E-05	0.52866177	0.033	0.023	0.44064607
Yipf6	2.0207E-05	0.29084103	0.08	0.063	0.44332441
Zfp444	2.114E-05	0.70777289	0.025	0.016	0.46378091
Zfp692	2.1213E-05	0.53688885	0.021	0.013	0.46539566
Cog2	2.1265E-05	0.36140077	0.089	0.073	0.46652718
Rnf26	2.1555E-05	0.62161249	0.027	0.018	0.47288706
Bbip1	2.1654E-05	0.26400293	0.14	0.121	0.47506753
Trappc2	2.2832E-05	0.5738972	0.031	0.022	0.50090107
9330160F10Ri	2.3013E-05	0.5076367	0.021	0.013	0.50489135
Aagab	2.4118E-05	0.28437265	0.084	0.067	0.52912448
Tmem18	2.5017E-05	0.58000281	0.022	0.014	0.54884968
Bola1	2.6171E-05	0.46059693	0.054	0.041	0.57415577

Cyth3	2.6281E-05	0.31670356	0.148	0.129	0.57657689
Nsmf	2.688E-05	0.47632012	0.032	0.022	0.58972077
Cramp1l	2.7114E-05	0.30097818	0.068	0.053	0.59486298
Ccr8	2.7292E-05	0.63793028	0.013	0.007	0.59876529
Map3k5	2.7727E-05	0.29217265	0.076	0.06	0.60831054
Nlrx1	2.8015E-05	1.18853295	0.011	0.006	0.61462291
Rad51d	2.8039E-05	0.38037695	0.032	0.022	0.61514094
Gstp1	2.8061E-05	0.57209442	0.031	0.021	0.61562673
Bcas3	2.8714E-05	0.42127077	0.048	0.036	0.62995571
Sec23ip	2.8818E-05	0.33442021	0.072	0.057	0.63223383
Tor4a	2.91E-05	0.68470173	0.015	0.009	0.638423
Nubp2	3.0616E-05	0.47754962	0.079	0.064	0.6716804
Auh	3.1746E-05	0.30495428	0.149	0.13	0.69647105
Glo1	3.2187E-05	0.36639902	0.091	0.075	0.70615325
Atf5	3.2378E-05	0.67374441	0.029	0.02	0.71033067
Mbd3	3.2391E-05	0.43367302	0.097	0.081	0.7106261
Ubr7	3.2584E-05	0.36302775	0.076	0.061	0.71486283
Hars	3.2623E-05	0.25948488	0.087	0.071	0.71570547
Guf1	3.2841E-05	0.376861	0.039	0.028	0.72050083
Cyb561d2	3.2869E-05	0.57435188	0.049	0.036	0.72110551
Gm11110	3.3688E-05	0.89775533	0.012	0.007	0.73907155
Ccdc137	3.3938E-05	0.3592051	0.027	0.018	0.74455549
Nabp2	3.4095E-05	0.40794844	0.079	0.064	0.74800944
Cbx6	3.4316E-05	0.27202841	0.026	0.017	0.75285942
Rpl27-ps3	3.442E-05	0.29319654	0.115	0.097	0.75513634
Fbxl18	3.4427E-05	0.71692421	0.016	0.009	0.75530209
Apool	3.6347E-05	0.5778045	0.044	0.033	0.79742484
Sirt6	3.7125E-05	0.64500656	0.018	0.011	0.81448583
Srsf9	3.7196E-05	0.27719968	0.17	0.152	0.81603532
Pdlim2	3.7394E-05	0.3190409	0.057	0.043	0.82038217
Yipf1	3.7764E-05	0.42877382	0.101	0.085	0.82851283
Txnrd2	3.9298E-05	0.63003072	0.048	0.036	0.86216178
Trappc3	3.9331E-05	0.43506993	0.1	0.084	0.86287528
Mtf1	3.953E-05	0.36969633	0.071	0.057	0.86725533
Glipr1	4.2061E-05	0.32138052	0.144	0.126	0.92278018
Dcps	4.3717E-05	0.45673975	0.064	0.051	0.95910541
Tor1b	4.3769E-05	0.40295154	0.098	0.081	0.96023844
Tmem126b	4.6672E-05	0.39081896	0.034	0.024	1
Gtpbp1	4.6845E-05	0.2772133	0.079	0.064	1
Slc35c1	4.7019E-05	0.70627675	0.022	0.014	1
Cep170b	4.8329E-05	0.59017086	0.018	0.011	1
Tcaim	4.895E-05	0.42389506	0.026	0.017	1
Nup37	4.8963E-05	0.78457323	0.015	0.009	1
Gm17018	4.9069E-05	0.35927978	0.078	0.063	1
4930430F08Ri	5.0581E-05	0.6933373	0.016	0.009	1
Dhps	5.0652E-05	0.26095434	0.125	0.107	1
Dhrs7	5.1242E-05	0.33506203	0.095	0.079	1
4930402H24R	5.1518E-05	0.55381878	0.035	0.025	1

Ctu2	5.33E-05	0.28744191	0.054	0.041	1
Cnih1	5.3519E-05	0.31316146	0.115	0.098	1
1700021F05Ri	5.4157E-05	0.71721746	0.029	0.02	1
Slc48a1	5.4393E-05	0.50223707	0.07	0.056	1
Nanos1	5.5417E-05	0.66355321	0.011	0.006	1
Wdr5b	5.6089E-05	0.64560647	0.011	0.006	1
Stim2	5.7705E-05	0.31428935	0.108	0.091	1
Nudt18	5.8488E-05	0.38565072	0.047	0.035	1
Lsm2	5.8612E-05	0.28252135	0.107	0.089	1
Scaf1	5.8797E-05	0.36133008	0.076	0.061	1
5730480H06R	5.9079E-05	0.47889142	0.028	0.019	1
Phf13	5.9616E-05	0.54499404	0.037	0.027	1
Aldh18a1	6.064E-05	0.53265283	0.036	0.026	1
Fastkd3	6.0672E-05	0.68521177	0.019	0.012	1
Pdpr	6.1092E-05	0.25464658	0.046	0.034	1
Psph	6.1312E-05	0.45057513	0.046	0.034	1
Cep19	6.3565E-05	0.69228794	0.014	0.008	1
Mark4	6.4415E-05	0.5354682	0.043	0.032	1
Mrps9	6.4418E-05	0.2544605	0.122	0.104	1
Gin1	6.5111E-05	0.57805755	0.026	0.017	1
Caml	6.7541E-05	0.39755381	0.047	0.035	1
Trim36	6.8593E-05	0.46589454	0.041	0.03	1
Nubp1	6.9812E-05	0.31048852	0.135	0.118	1
Marveld1	7.1E-05	0.74735875	0.03	0.021	1
Actr5	7.763E-05	0.29179748	0.059	0.046	1
Abhd16a	7.8643E-05	0.37918732	0.085	0.071	1
Jkamp	7.9064E-05	0.3951918	0.06	0.047	1
Diablo	8.0908E-05	0.31727432	0.081	0.066	1
Ap2a1	8.2887E-05	0.50369897	0.056	0.044	1
Ccne2	8.3079E-05	0.65616494	0.016	0.01	1
Gnpda1	8.3583E-05	0.49145943	0.062	0.049	1
Anapc4	8.4017E-05	0.30982515	0.054	0.042	1
Tyw5	8.4649E-05	0.66996736	0.026	0.018	1
Bpgm	8.689E-05	0.40853896	0.028	0.019	1
Zfp689	8.9032E-05	0.67124358	0.013	0.007	1
2310039H08R	9.0328E-05	0.44910165	0.078	0.064	1
Wdr83	9.3449E-05	0.5879018	0.032	0.023	1
Arl10	9.432E-05	0.83336652	0.021	0.014	1
Sfi1	9.8416E-05	0.33865445	0.042	0.031	1
Mta1	9.9216E-05	0.30287217	0.09	0.074	1
Edc3	9.949E-05	0.41644428	0.034	0.024	1
Timm22	9.983E-05	0.30321684	0.078	0.063	1
Rab26	0.00010194	0.44494809	0.022	0.014	1
Lin9	0.00010443	0.40248945	0.039	0.029	1
Zfp775	0.00010984	0.82649844	0.012	0.007	1
Znhit3	0.00011018	0.39297691	0.043	0.032	1
Nt5dc1	0.00011318	0.34929462	0.05	0.038	1
Zc3h3	0.00011358	0.39913168	0.034	0.025	1

Faap20	0.00011828	0.28290347	0.058	0.045	1
Gm49359	0.00012016	0.52625934	0.026	0.018	1
Tmed4	0.00012167	0.48942723	0.049	0.038	1
Gdf11	0.00012268	0.33431317	0.018	0.011	1
Ppp1r16a	0.00012319	0.54106063	0.023	0.015	1
Cars	0.00012334	0.29731568	0.104	0.088	1
Galnt12	0.00012337	0.2931411	0.037	0.027	1
Hspbp1	0.00012442	0.37011359	0.066	0.053	1
Ccdc9	0.00012457	0.25151236	0.151	0.133	1
Vps4a	0.00012518	0.34372225	0.093	0.078	1
Polr3f	0.00012559	0.2573759	0.066	0.052	1
2310057M21F	0.00012594	0.29740743	0.033	0.024	1
Gm9493	0.00012896	0.38134899	0.093	0.078	1
Zfp952	0.00012973	0.29627822	0.034	0.025	1
2010109A12R	0.00012996	0.32191298	0.013	0.007	1
Rbmx2	0.00013095	0.3149024	0.063	0.05	1
Eid2b	0.00013917	0.78019043	0.017	0.011	1
Nfatc2ip	0.00014105	0.30883142	0.056	0.043	1
Pyurf	0.00014113	0.50826352	0.059	0.047	1
Pop1	0.00014175	0.73346654	0.016	0.01	1
Gm27003	0.00014445	0.42936269	0.012	0.007	1
Memo1	0.0001483	0.3994852	0.056	0.044	1
Trim14	0.00015072	0.46899356	0.014	0.009	1
Srfbp1	0.00015464	0.37431416	0.045	0.034	1
Timm21	0.00015539	0.46940169	0.033	0.024	1
Bcl2l13	0.00015647	0.26966241	0.062	0.049	1
Tspyl3	0.00015743	0.75275387	0.012	0.007	1
Golga1	0.00016007	0.53975269	0.023	0.016	1
Dcun1d4	0.00016179	0.29198964	0.03	0.022	1
Jagn1	0.00016485	0.48652983	0.048	0.037	1
Brms1	0.00016486	0.38867808	0.079	0.065	1
Rbak	0.00016625	0.70679672	0.017	0.01	1
Zfp760	0.00017067	0.48359479	0.014	0.008	1
Cbfa2t2	0.00017235	0.42896032	0.033	0.024	1
Polr3g	0.00017241	0.64609044	0.017	0.01	1
Iffo1	0.00017287	0.29627869	0.032	0.023	1
Zbtb48	0.00018206	0.49108437	0.017	0.011	1
Usp39	0.00018286	0.29052454	0.073	0.059	1
Cacfd1	0.00018678	0.38849697	0.047	0.036	1
Gpr146	0.00018687	0.27221979	0.081	0.066	1
Kdm4c	0.00018769	0.25273879	0.06	0.047	1
Med18	0.00019328	0.69390951	0.012	0.007	1
Mrpl11	0.00019449	0.3924394	0.113	0.098	1
Hmbs	0.00019513	0.72819854	0.032	0.023	1
Rassf1	0.00019675	0.27601915	0.152	0.135	1
Rad1	0.00019763	0.42881905	0.023	0.015	1
Gm37387	0.00020339	0.54953111	0.013	0.008	1
Cpsf4	0.00020983	0.45661258	0.045	0.035	1

Mrpl36	0.00021267	0.31144436	0.142	0.126	1
Gramd1c	0.00021562	0.54334212	0.013	0.007	1
Nelfcd	0.00021816	0.36871787	0.035	0.025	1
Coq10a	0.00022157	0.45902516	0.036	0.026	1
Gtf2f2	0.00022367	0.32358417	0.116	0.101	1
Socs7	0.00022461	0.33085169	0.059	0.047	1
2610044O15R	0.00022684	0.47470176	0.02	0.013	1
Ccs	0.00022847	0.519701	0.065	0.053	1
Zbtb41	0.00023098	0.26655112	0.043	0.032	1
Fkbpl	0.00023117	0.93651978	0.012	0.007	1
Prkab1	0.00023186	0.2938376	0.047	0.036	1
Dohh	0.000232	0.34215324	0.085	0.071	1
Timm44	0.00023962	0.26262936	0.113	0.097	1
B3galt6	0.0002401	0.67484324	0.018	0.012	1
Rbm14	0.00024035	0.35604432	0.058	0.046	1
Ptrhd1	0.00024138	0.47309996	0.11	0.096	1
Mcur1	0.00024877	0.30788888	0.118	0.103	1
Dexi	0.00025101	0.34987281	0.04	0.03	1
Mturn	0.00025339	0.33623251	0.018	0.012	1
Gm26514	0.00026207	0.648225	0.011	0.006	1
Krtcap3	0.00026291	0.61477601	0.017	0.011	1
Hibch	0.00026646	0.33842288	0.023	0.015	1
Capn1	0.00026651	0.27444476	0.103	0.088	1
Rabepk	0.00026807	0.44741869	0.032	0.023	1
Sap18b	0.00027114	0.75223989	0.019	0.012	1
L3mbtl2	0.00028676	0.37037151	0.025	0.017	1
Zfp865	0.00028881	0.28318818	0.05	0.038	1
Rpap2	0.00029019	0.33470656	0.034	0.025	1
Gm45509	0.00029532	0.69822142	0.013	0.007	1
Ints5.1	0.00029584	0.4917168	0.026	0.019	1
Stx8	0.00030248	0.37484898	0.127	0.112	1
Hikeshi	0.00030843	0.3918574	0.085	0.072	1
Tprn	0.00031768	0.58348477	0.018	0.012	1
Zfp511	0.00031978	0.34098364	0.092	0.078	1
Copg1	0.00032131	0.26165473	0.102	0.087	1
Tmem97	0.00032276	0.48218987	0.039	0.029	1
Rusc1	0.00032279	0.25623299	0.07	0.056	1
Disp1	0.00032453	0.65894575	0.017	0.011	1
Zfp874a	0.00033223	0.35778388	0.029	0.02	1
Cox18	0.00033503	0.36777051	0.04	0.03	1
Mrpl2	0.00034024	0.39837027	0.073	0.061	1
Zscan29	0.00036044	0.36477837	0.033	0.024	1
Tysnd1	0.00036751	0.50883165	0.023	0.016	1
Fchsd1	0.00037309	0.52114367	0.017	0.011	1
Meaf6	0.00037602	0.41222234	0.071	0.059	1
Tmem208	0.0003808	0.2704496	0.134	0.119	1
Map3k3	0.00039807	0.25689477	0.074	0.061	1
Ilk	0.0004101	0.36258479	0.109	0.095	1

Zfp810	0.00041749	0.54086129	0.017	0.011	1
Mcph1	0.00042475	0.40667415	0.036	0.027	1
Gemin2	0.0004311	0.43550273	0.029	0.021	1
E230001N04R	0.00043622	0.34380139	0.017	0.011	1
Gm44066	0.0004416	0.47285114	0.011	0.006	1
Cep152	0.00044756	0.54498067	0.02	0.013	1
Lysmd4	0.00044905	0.78634788	0.012	0.007	1
Scnm1	0.00045046	0.38515942	0.039	0.03	1
Rab10os	0.00045092	0.265662	0.176	0.16	1
Gtf3c3	0.00045244	0.32732124	0.048	0.037	1
Ngly1	0.00046032	0.2510601	0.067	0.054	1
Desi2	0.00047176	0.28106602	0.124	0.109	1
Prkcsh	0.00047275	0.27572399	0.16	0.146	1
Haus2	0.00047951	0.30082938	0.051	0.04	1
Pip4k2b	0.00048149	0.29787637	0.039	0.029	1
Tubg1	0.00048194	0.71963406	0.015	0.009	1
Nucb1	0.00048287	0.25250626	0.179	0.164	1
Pold3	0.0004847	0.36850917	0.069	0.056	1
C730034F03R	0.00049206	0.43169132	0.029	0.021	1
Wrap73	0.00049358	0.3665421	0.028	0.02	1
Rexo5	0.00050882	0.31599523	0.018	0.012	1
Traf3ip1	0.00050973	0.55574906	0.013	0.008	1
Acd	0.00050996	0.36666052	0.072	0.059	1
Nprl2	0.00051035	0.59077898	0.022	0.015	1
Pola1	0.00052023	0.37424284	0.052	0.041	1
Rdh14	0.00052376	0.34459141	0.034	0.025	1
Mmgt2	0.00053051	0.38732336	0.035	0.026	1
Adamts10	0.00053078	0.34268811	0.033	0.024	1
Gpatch2	0.00054171	0.28888515	0.06	0.048	1
Ccdc130	0.00054966	0.53231236	0.024	0.017	1
Gm26847	0.00054996	0.465014	0.011	0.006	1
Dcakd	0.00055156	0.60806109	0.016	0.01	1
Acot13	0.0005808	0.37179919	0.058	0.047	1
Acads	0.00060383	0.37720986	0.055	0.044	1
Taf1b	0.00060781	0.31358388	0.048	0.037	1
Gm21781	0.00063264	0.63575049	0.011	0.006	1
Med20	0.00063323	0.25669889	0.054	0.043	1
Smim12	0.00063762	0.37096361	0.066	0.054	1
Rnf121	0.00064631	0.28923586	0.058	0.047	1
Cyb5d2	0.00064994	0.46582239	0.023	0.016	1
Rexo4	0.00065455	0.31331847	0.1	0.086	1
Hira	0.00068897	0.36973878	0.04	0.031	1
Oxld1	0.00070512	0.81468721	0.013	0.008	1
Pdp1	0.00071656	0.42383613	0.034	0.025	1
Fbxo45	0.00072975	0.31777704	0.038	0.029	1
Abcf3	0.00075171	0.25316064	0.063	0.051	1
Slc25a42	0.00077767	0.58928766	0.011	0.006	1
Mvb12a	0.00079642	0.35152989	0.141	0.128	1

Hes6	0.00080354	0.38185687	0.036	0.028	1
Phpt1	0.00080618	0.46011729	0.043	0.034	1
Snapc2	0.00080739	0.60407366	0.03	0.022	1
Mrps28	0.00082353	0.28489806	0.124	0.11	1
Fcf1	0.00082778	0.25807961	0.131	0.117	1
Nat2	0.00082966	0.51672256	0.018	0.012	1
Gm20721	0.00084856	0.46436298	0.012	0.008	1
Wdfy2	0.00088812	0.35596833	0.074	0.062	1
Kdm4b	0.00090855	0.26504364	0.033	0.025	1
Mpg	0.00092764	0.52818929	0.051	0.041	1
Arhgap11a	0.0009766	0.47951991	0.043	0.033	1
Gm12166	0.00100558	0.66243225	0.013	0.009	1
Etfdh	0.0010245	0.26904792	0.063	0.052	1
Foxk2	0.00102772	0.35357346	0.046	0.036	1
Vamp7	0.0010357	0.35563457	0.069	0.057	1
Osgepl1	0.00103742	0.49223142	0.012	0.008	1
Phf11b	0.00104107	0.26684431	0.079	0.067	1
Cradd	0.00105131	0.46395483	0.024	0.017	1
Qrsl1	0.00105819	0.28513096	0.039	0.03	1
Adat2	0.00108263	0.57264071	0.03	0.023	1
Fars2	0.00108439	0.48341065	0.047	0.038	1
Bhlhb9	0.00108602	0.257755	0.019	0.013	1
Ndor1	0.00109459	0.61575178	0.014	0.009	1
Mvk	0.00109575	0.53278532	0.034	0.026	1
Dctpp1	0.00109637	0.50534451	0.072	0.061	1
Kremen1	0.00110908	0.38027946	0.03	0.022	1
Traf7	0.00114871	0.30028015	0.078	0.066	1
Mtrf1l	0.00115758	0.44983904	0.023	0.016	1
Mars	0.00116147	0.44742371	0.048	0.038	1
Ahcy	0.00120747	0.63410044	0.014	0.009	1
Gtf2h4	0.00120787	0.65069846	0.022	0.016	1
Zfp35	0.00123708	0.34553491	0.022	0.015	1
Smim13	0.0012484	0.46230237	0.039	0.03	1
Wdr18	0.0012856	0.34397734	0.068	0.057	1
Mul1	0.00128673	0.3535012	0.077	0.066	1
Vta1	0.00129014	0.34437314	0.105	0.092	1
Zfp668	0.0013027	0.48271262	0.017	0.011	1
Mmab	0.00133601	0.87423171	0.013	0.008	1
Zbtb26	0.001387	0.25447112	0.033	0.025	1
Wdr47	0.00139292	0.2858973	0.028	0.02	1
Fbxo46	0.00142758	0.380833	0.024	0.017	1
Rab40c	0.00143489	0.35322954	0.028	0.021	1
Hyou1	0.00143506	0.28301268	0.131	0.117	1
Zmym3	0.00145511	0.56125673	0.022	0.016	1
Aifm1	0.00147284	0.44937072	0.046	0.037	1
Zpr1	0.00150317	0.25968239	0.085	0.073	1
Lppos	0.00152548	0.35036396	0.029	0.022	1
Cox7a1	0.00153386	0.96449932	0.013	0.009	1

Zbtb49	0.00153935	0.3491312	0.018	0.012	1
Rpusd4	0.00168019	0.2500776	0.052	0.042	1
Tmco6	0.00171799	0.38694975	0.024	0.018	1
Fam149b	0.00172882	0.37604255	0.026	0.019	1
Gm16740	0.00173836	0.65613176	0.011	0.007	1
Gm26533	0.00174152	0.37839787	0.015	0.01	1
Slc4a2	0.00177113	0.26221547	0.051	0.041	1
Tm2d2	0.00179426	0.31971457	0.141	0.129	1
Deaf1	0.00179991	0.42653272	0.02	0.014	1
Dcaf10	0.00181067	0.31389038	0.057	0.047	1
Piga	0.00181583	0.28097905	0.053	0.043	1
BC029722	0.00183681	0.26730165	0.058	0.047	1
Nipsnap1	0.00188736	0.38159126	0.024	0.017	1
Zfp790	0.00193951	0.5585848	0.01	0.006	1
Med29	0.00198889	0.39234182	0.099	0.087	1
Hmg20b	0.00199689	0.3079217	0.119	0.107	1
Irgq	0.00201503	0.25997319	0.026	0.019	1
Thap1	0.00206359	0.41063678	0.036	0.028	1
Tmem70	0.00208561	0.34691582	0.056	0.046	1
Blzf1	0.00214246	0.32920018	0.05	0.04	1
Rfesd	0.0023281	0.53072683	0.024	0.018	1
Zbtb6	0.00238656	0.36593081	0.051	0.042	1
Zfp282	0.00239903	0.48589527	0.023	0.017	1
Ncapd2	0.00240484	0.3411457	0.031	0.024	1
Saysd1	0.00241552	0.42979946	0.04	0.032	1
Fzr1	0.00246368	0.29177421	0.062	0.051	1
Smpd5	0.00249891	0.41793001	0.012	0.007	1
Fam136a	0.00264726	0.32345922	0.043	0.034	1
Ino80c	0.00265837	0.35092327	0.067	0.057	1
Sec23a	0.00265973	0.31989595	0.04	0.032	1
Acot8	0.00272245	0.49762546	0.049	0.04	1
Metrn	0.00275306	0.34751112	0.037	0.029	1
Cenpx	0.00276427	0.30142032	0.153	0.142	1
A430005L14Ri	0.00279947	0.36838764	0.062	0.052	1
Tjap1	0.00285419	0.32342631	0.059	0.049	1
Psmd6	0.00299173	0.2537914	0.148	0.137	1
Nudt1	0.00303261	0.78380526	0.017	0.012	1
2310031A07R	0.00304552	0.79404008	0.031	0.024	1
Med27	0.00305087	0.37244838	0.045	0.037	1
Ndufa9	0.00305899	0.32298594	0.12	0.109	1
Pex13	0.00307211	0.32408112	0.061	0.051	1
Man2c1os	0.00318284	0.5614339	0.01	0.006	1
Slc35d2	0.00319802	0.41524959	0.017	0.012	1
Rmdn3	0.0032636	0.30169678	0.035	0.027	1
Mmp25	0.00326628	0.81222665	0.022	0.016	1
Ccdc94	0.00328371	0.46009917	0.024	0.018	1
Wdr12	0.00337412	0.26809354	0.044	0.036	1
Klhl42	0.0034248	0.31664549	0.011	0.007	1

Gdpd5	0.00344269	0.41291364	0.02	0.014	1
Trmt12	0.00348608	0.51807906	0.011	0.007	1
Fbxl15	0.00352062	0.50290684	0.029	0.022	1
Kcnc1	0.00352669	0.30844221	0.015	0.01	1
Alkbh3	0.00356512	0.42608666	0.046	0.038	1
Zfp60	0.00360832	0.47684207	0.014	0.01	1
Ppm1j	0.00363719	0.31520406	0.022	0.016	1
Ric8b	0.00373551	0.38482293	0.025	0.019	1
Stoml2	0.00397212	0.3905903	0.065	0.055	1
A530017D24R	0.00404593	0.40300553	0.013	0.009	1
Fdft1	0.00413962	0.41619767	0.04	0.032	1
Xpo5	0.00415679	0.62730821	0.016	0.011	1
Cox15	0.00416352	0.40986824	0.024	0.018	1
Penk	0.00426986	0.79107477	0.019	0.013	1
Mthfsd	0.00427812	0.53373918	0.021	0.016	1
Them4	0.00434869	0.33352259	0.024	0.018	1
Btbd6	0.00447132	0.25665718	0.012	0.008	1
Mtcp1	0.00451801	0.29836406	0.018	0.013	1
Anapc11	0.00456184	0.25252688	0.141	0.13	1
Thg1l	0.00457946	0.50468956	0.026	0.02	1
Ndufaf1	0.00460437	0.41801758	0.024	0.018	1
Zfp607a	0.00461062	0.38520031	0.021	0.016	1
Gm20234	0.00463372	0.27944263	0.014	0.01	1
Serhl	0.00467323	0.40408242	0.038	0.03	1
Eif2b5	0.00483441	0.29521786	0.066	0.056	1
Ptrh2	0.00488357	0.30600421	0.056	0.047	1
Tmem201	0.00491197	0.35956969	0.017	0.012	1
1500009L16Ri	0.00491593	0.62619851	0.013	0.009	1
Mlst8	0.00498419	0.26223714	0.021	0.016	1
Nup107	0.00501043	0.28496976	0.028	0.021	1
Spryd7	0.00521945	0.31188932	0.063	0.054	1
Zfp524	0.00522067	0.46169837	0.046	0.038	1
Camta1	0.00526728	0.33845102	0.088	0.078	1
Lmtk3	0.00529286	0.35178448	0.013	0.009	1
Isyna1	0.00532868	0.36780127	0.078	0.069	1
Cenpj	0.00540441	0.43195027	0.016	0.011	1
Tmem147	0.00543588	0.38402439	0.1	0.09	1
Rbm47	0.00545733	0.32995563	0.152	0.143	1
2700016F22Ri	0.00549818	0.32816281	0.016	0.011	1
B130006D01R	0.00554214	0.34544006	0.014	0.01	1
Crip2	0.00558157	0.55097255	0.023	0.017	1
Lage3	0.00592207	0.44415658	0.067	0.058	1
Cyb561d1	0.00609016	0.34156323	0.031	0.024	1
Oraov1	0.0062709	0.28455137	0.05	0.041	1
C330027C09F	0.00648931	0.67214324	0.013	0.009	1
Cenpb	0.00649765	0.29207473	0.113	0.102	1
Akt1	0.00677602	0.3461653	0.118	0.109	1
Cep41	0.00681747	0.4543127	0.018	0.013	1

Ercc1	0.00717378	0.52264123	0.034	0.028	1
Lig4	0.00743598	0.46536914	0.011	0.007	1
Zfp799	0.00755451	0.39619074	0.017	0.012	1
Myg1	0.00760286	0.28498154	0.05	0.042	1
AC129328.1	0.00761515	0.49281712	0.015	0.011	1
Naa60	0.00772726	0.25521592	0.054	0.045	1
Nbas	0.00776032	0.39298113	0.036	0.029	1
Mtmr9	0.00799712	0.34548097	0.027	0.021	1
Ermp1	0.00919762	0.37604587	0.05	0.042	1
Atxn7l2	0.00949729	0.38200081	0.02	0.015	1
Gm47662	0.0095557	0.87730969	0.023	0.018	1
Fahd2a	0.00994531	0.67635499	0.023	0.018	1
Slc2a8	0.01002652	0.43407072	0.019	0.014	1
Fam98a	0.01014154	0.28513947	0.035	0.028	1
Isoc2b	0.01016305	0.65143424	0.019	0.014	1
Med22	0.01016846	0.55651637	0.023	0.018	1
Rbm45	0.0106409	0.31018647	0.025	0.02	1
Adgrg1	0.01068097	0.92113015	0.011	0.007	1
AU022252	0.01078549	0.28027381	0.024	0.018	1
Gm14325	0.01089575	0.93860361	0.01	0.006	1
Akt1s1	0.01100736	0.43228951	0.032	0.026	1
Cd320	0.01103195	0.36450276	0.028	0.022	1
Cbr4	0.01112061	0.57284843	0.016	0.012	1
Prdx3	0.01122867	0.26660326	0.088	0.079	1
Rab29	0.01152529	0.40983767	0.021	0.016	1
Cnot9	0.01162618	0.33722423	0.049	0.042	1
Osgep	0.01177549	0.28864097	0.122	0.113	1
Plrg1	0.01231262	0.37424226	0.044	0.036	1
Trim41	0.01245552	0.28067185	0.048	0.04	1
Oxnad1	0.01252929	0.36486039	0.013	0.009	1
Tor2a	0.01265256	0.38055694	0.056	0.048	1
Dtwd2	0.01275036	0.30324613	0.013	0.009	1
Fam210b	0.01286126	0.32672717	0.034	0.027	1
Mastl	0.01327895	0.52614599	0.01	0.006	1
Chpf	0.01333399	0.25591991	0.027	0.022	1
Brf2	0.01339906	0.36936766	0.022	0.017	1
Gm43848	0.01352657	0.32188191	0.027	0.021	1
Apba3	0.01368852	0.46020388	0.025	0.019	1
Ncapg	0.01378425	0.55799485	0.013	0.009	1
Serpine2	0.01432475	0.46646023	0.011	0.008	1
Irak1bp1	0.01446626	0.80150499	0.011	0.007	1
Cchcr1	0.01465773	0.31318332	0.014	0.01	1
Mrpl28	0.01526336	0.27131533	0.082	0.074	1
Sdf2l1	0.01560277	0.37741648	0.153	0.147	1
Slc4a8	0.01597555	0.43115577	0.02	0.015	1
Rabl3	0.01599705	0.29587646	0.029	0.023	1
Trappc11	0.01607066	0.2575339	0.045	0.038	1
Sssca1	0.01655491	0.35391508	0.051	0.044	1

Mrto4	0.01709847	0.26191729	0.11	0.101	1
Gpn2	0.01721067	0.32968771	0.024	0.019	1
Sfmbt2	0.01729314	0.53384996	0.011	0.008	1
Rnf126	0.01745298	0.34999606	0.047	0.04	1
Tepsin	0.01752964	0.50103953	0.012	0.009	1
Poc5	0.01753691	0.34222881	0.03	0.024	1
Ppp2r5d	0.01767003	0.3670865	0.043	0.036	1
Gm14326	0.01796437	0.67907115	0.012	0.008	1
Glod4	0.01853368	0.30401956	0.088	0.08	1
Dffb	0.01907298	0.29568234	0.021	0.017	1
Serpina3f	0.01918495	0.34981363	0.025	0.019	1
Atrip	0.0198875	0.32792919	0.028	0.022	1
Ift52	0.02030477	0.29861796	0.065	0.057	1
Slc33a1	0.02068974	0.40986414	0.034	0.028	1
Gm11273	0.02090128	0.8611709	0.01	0.007	1
Saal1	0.02104317	0.340677	0.027	0.021	1
Dnajc12	0.02125755	0.34128049	0.046	0.039	1
Lmf2	0.02161369	0.29253803	0.047	0.04	1
Pms1	0.02197109	0.28736764	0.015	0.011	1
Pex12	0.02237321	0.30483595	0.014	0.01	1
Snx21	0.02245383	0.84433632	0.017	0.013	1
Trpm4	0.02409718	0.47443973	0.015	0.011	1
Pmpcb	0.02448934	0.30169077	0.075	0.067	1
Ipp	0.02449129	0.31066043	0.014	0.01	1
Pskh1	0.02460295	0.36584409	0.024	0.019	1
Fam35a	0.0247255	0.26570005	0.02	0.016	1
Bscl2	0.02564475	0.25939095	0.108	0.1	1
Zdhhc16	0.02675845	0.42967064	0.023	0.018	1
Mettl22	0.0277998	0.52348761	0.013	0.01	1
Map3k12	0.02923444	0.2628917	0.025	0.02	1
Habp4	0.02950092	0.39196135	0.017	0.013	1
Spata24	0.02954421	0.32266387	0.021	0.016	1
Nedd1	0.03014134	0.30824594	0.029	0.024	1
Gpt	0.03115539	0.57454464	0.012	0.009	1
Btd	0.0322461	0.35927702	0.022	0.017	1
Riox1	0.0332179	0.35829918	0.03	0.024	1
Mterf4	0.03383699	0.31886345	0.021	0.017	1
Gm26561	0.0341186	0.39670646	0.012	0.009	1
Fbxo42	0.03584077	0.293677	0.044	0.038	1
Urod	0.03643033	0.3903541	0.037	0.031	1
Sdhaf4	0.03643529	0.34464258	0.052	0.045	1
Tgtp2	0.03763613	0.25868178	0.02	0.015	1
0610009B22R	0.03773091	0.37326249	0.02	0.016	1
Phtf1os	0.03912684	0.25044131	0.01	0.007	1
Apex2	0.03984391	0.42801116	0.019	0.015	1
Klhl36	0.04012628	0.27136619	0.015	0.011	1
Acbd4	0.04027484	0.30878823	0.015	0.012	1
Psmd10	0.04034683	0.33562191	0.085	0.078	1

Spryd4	0.04036653	0.34333953	0.017	0.013	1
Pex14	0.04197683	0.35222604	0.033	0.027	1
Mycbp	0.04686041	0.29163717	0.05	0.044	1
Car5b	0.04741319	0.63584789	0.015	0.011	1
Cluap1	0.04754881	0.27275619	0.025	0.021	1
2310022B05R	0.04774158	0.36683929	0.024	0.02	1
Ilvbl	0.04999336	0.2551687	0.046	0.04	1
Zfp12	0.05019543	0.32016178	0.012	0.009	1
Vps25	0.05113943	0.60037183	0.011	0.008	1
9330020H09R	0.0527587	0.31881086	0.025	0.021	1
Atxn1l	0.05291559	0.29461693	0.048	0.043	1
Icmt	0.05447213	0.25662971	0.037	0.032	1
Thap11	0.05475056	0.28724637	0.027	0.023	1
Fbxo6	0.05504889	0.43464245	0.059	0.053	1
Aamdc	0.05530462	0.29144898	0.029	0.024	1
Rpl39l	0.05537817	0.30895839	0.01	0.007	1
Sars2	0.05567374	0.2935514	0.017	0.013	1
Acaa2	0.05588379	0.34255125	0.052	0.047	1
Pcgf1	0.05752119	0.31375786	0.024	0.02	1
Aptx	0.0577883	0.27967414	0.037	0.032	1
Acap3	0.05829824	0.2840397	0.019	0.015	1
Zfp933	0.05892494	0.34405286	0.014	0.011	1
Tstd2	0.05968574	0.30310056	0.032	0.027	1
Gm48960	0.06079505	0.41198641	0.01	0.007	1
Ccdc58	0.06529329	0.30520081	0.038	0.033	1
Zfp809	0.06689907	0.26354742	0.038	0.032	1
Dolpp1	0.07254238	0.63026219	0.013	0.011	1
Commd1	0.07277047	0.31926252	0.106	0.101	1
Paqr7	0.07383804	0.47588718	0.01	0.007	1
1810055G02R	0.07736785	0.4130318	0.014	0.011	1
Gm16124	0.07849985	0.26759072	0.012	0.01	1
Tada2a	0.08108428	0.27692509	0.01	0.008	1
Akip1	0.08213072	0.30131136	0.024	0.02	1
Mkks	0.08478149	0.52408774	0.012	0.009	1
Ick	0.0879553	0.3992205	0.012	0.009	1
Mettl7a1	0.09150009	0.41650916	0.018	0.015	1
Zfp46	0.0947773	0.37073624	0.013	0.011	1
Prune1	0.09531469	0.34874221	0.022	0.018	1
Gm11423	0.09576266	0.30289994	0.011	0.009	1
Atat1	0.09632047	0.3345251	0.017	0.014	1
Suv39h2	0.09813022	0.2574444	0.016	0.013	1
Cog1	0.09884231	0.2690565	0.035	0.031	1
Smyd2	0.10358048	0.38673037	0.019	0.015	1
4933406P04R	0.10430686	0.39691213	0.011	0.008	1
Soat2	0.10835455	0.39219589	0.012	0.009	1
Arl16	0.1100396	0.27408685	0.016	0.013	1
Mier2	0.11016993	0.27238705	0.016	0.013	1
Zfp653	0.11393697	0.25733136	0.019	0.016	1

Fam20a	0.11483113	0.4586924	0.011	0.009	1
Cwf19l1	0.11985366	0.32094232	0.013	0.011	1
Zfp628	0.12220096	0.31690878	0.029	0.025	1
Nudt14	0.12239007	0.43505902	0.044	0.04	1
Otud3	0.12415504	0.32804257	0.018	0.015	1
Uevld	0.12573557	0.42476766	0.012	0.009	1
Nelfe	0.12672017	0.3188093	0.074	0.069	1
Nudt2	0.12758101	0.47314906	0.026	0.022	1
Med9	0.13420028	0.39712288	0.04	0.036	1
Hps6	0.13425473	0.52950662	0.018	0.015	1
Slc35b4	0.13451697	0.264451	0.024	0.02	1
Dcaf4	0.1346226	0.28101948	0.018	0.015	1
Cinp	0.14029657	0.29188861	0.029	0.026	1
Pgm2	0.14051057	0.32088326	0.023	0.02	1
Zhx3	0.14157375	0.32670101	0.014	0.011	1
Arfip2	0.14904194	0.26099524	0.018	0.015	1
Rrp7a	0.15131345	0.27444813	0.07	0.065	1
Tubgcp2	0.15303461	0.26778348	0.046	0.042	1
Kif3c	0.15532574	0.26163261	0.01	0.008	1
Bloc1s5	0.16080497	0.30676855	0.011	0.009	1
Rwdd2b	0.16239811	0.33298906	0.013	0.011	1
Gmppb	0.16779585	0.34481147	0.047	0.043	1
Spr	0.17367173	0.2986572	0.048	0.044	1
Cdk5rap3	0.18001941	0.25941302	0.06	0.056	1
Ap1g2	0.18211999	0.25140687	0.039	0.036	1
Cysltr1	0.1864807	0.46741749	0.055	0.051	1
Gas8	0.18967136	0.50633253	0.011	0.009	1
Haus1	0.19561839	0.43612695	0.013	0.011	1
Flad1	0.19704825	0.34583468	0.02	0.017	1
Zfyve19	0.19924083	0.2757145	0.017	0.015	1
1700037H04R	0.20139601	0.2553717	0.031	0.028	1
Hax1	0.21312556	0.31805674	0.114	0.112	1
Cetn4	0.22207354	0.64988486	0.014	0.012	1
Tmem238	0.22284653	0.38950929	0.041	0.037	1
Tcta	0.22591272	0.33592674	0.011	0.009	1
Arv1	0.2285211	0.29382976	0.01	0.008	1
Usp30	0.22951502	0.30981191	0.015	0.013	1
Ndufaf2	0.22987605	0.29674642	0.05	0.047	1
Tbcd	0.24264202	0.27237496	0.034	0.031	1
Elp5	0.26486354	0.31509262	0.109	0.108	1
Msto1	0.26667752	0.27178854	0.018	0.016	1
Hsdl2	0.27519591	0.31649543	0.042	0.039	1
Cd151	0.2825828	0.4075012	0.011	0.01	1
Rtca	0.28409295	0.29239791	0.038	0.035	1
1110065P20R	0.28750816	0.29418173	0.045	0.042	1
Ydjc	0.28796341	0.58788957	0.013	0.011	1
Setd6	0.29481374	0.25792409	0.017	0.015	1
Tmem242	0.29650148	0.28481206	0.052	0.049	1

Crcp	0.30034043	0.26714105	0.046	0.043	1
3010003L21Ri	0.32632684	0.31044574	0.016	0.014	1
Igsf8	0.39506133	0.27599185	0.089	0.097	1
Limk1	0.40216832	0.31330687	0.017	0.015	1
Hddc3	0.40772176	0.36773086	0.011	0.01	1
Lancl2	0.41150107	0.34509611	0.022	0.02	1
Timm23	0.41921381	0.26870957	0.021	0.02	1
Lyrm1	0.42317752	0.37404131	0.017	0.015	1
Ormdl2	0.44142679	0.27403722	0.09	0.089	1
Alg12	0.5033802	0.3566121	0.013	0.012	1
Nqo2	0.51794802	0.39953933	0.019	0.017	1
Cnpy2	0.66113429	0.35657704	0.076	0.077	1
Btbd2	0.69080126	0.25700114	0.013	0.012	1
Pigyl	0.69526201	0.25080836	0.051	0.05	1
Mzt2	0.86121823	0.26744604	0.026	0.026	1
Slc6a8	0.91481635	0.2657017	0.01	0.01	1
Platr17	0.92235802	0.27747854	0.011	0.012	1
Akr1e1	0.92567078	0.34673814	0.011	0.012	1

CD4 Tn

Gene	p_val	avg_log2FC	pct.1	pct.2	p_val_adj
Izumo1r	0	5.57949696	0.66	0.023	0
Lrig1	0	4.2765643	0.229	0.016	0
Sostdc1	3.904E-283	7.8157171	0.045	0	8.565E-279
Tcf7	3.258E-261	2.69606723	0.684	0.173	7.149E-257
Myb	6.671E-256	3.29990064	0.342	0.043	1.464E-251
Il21	1.455E-243	6.01924092	0.066	0.001	3.193E-239
Nsg2	6.295E-241	3.67056148	0.237	0.022	1.381E-236
Sh2d1a	1.08E-235	3.28788726	0.435	0.076	2.369E-231
Tnfsf8	1.581E-233	2.32042716	0.579	0.125	3.468E-229
H2-Q2	7.096E-230	5.6131146	0.077	0.002	1.557E-225
Ikzf2	9.935E-176	1.98605589	0.62	0.177	2.18E-171
Dennd2d	3.184E-174	3.2854121	0.283	0.042	6.985E-170
Tbc1d4	1.074E-169	2.12744482	0.527	0.139	2.357E-165
A630023P12R	2.231E-168	3.83625663	0.147	0.012	4.895E-164
Trac	8.884E-154	2.06646731	0.656	0.229	1.949E-149
Ptprc	1.054E-153	1.20653075	0.983	0.893	2.313E-149
Scg2	8.815E-151	7.87450018	0.021	0	1.934E-146
Slamf6	1.491E-148	2.74591434	0.387	0.087	3.271E-144
Trib2	7.015E-148	2.42854599	0.468	0.123	1.539E-143
Bcl11b	1.379E-144	2.23798679	0.541	0.165	3.026E-140
Ces2c	1.026E-143	6.33469576	0.028	0	2.252E-139
Tox	2.481E-132	2.51921787	0.365	0.083	5.444E-128
Trbc2	1.316E-128	1.44080206	0.834	0.384	2.888E-124
Maf	1.625E-128	1.79873605	0.729	0.326	3.565E-124
Gimap3	9.557E-124	1.71065697	0.705	0.307	2.097E-119
Tox2	3.675E-121	3.10190356	0.234	0.04	8.062E-117
Ifi27l2a	9.462E-117	1.50143163	0.815	0.471	2.076E-112
Ntrk3	1.017E-116	3.44580842	0.164	0.021	2.232E-112
Tmem65	4.494E-116	3.00062551	0.316	0.074	9.859E-112
Cd3d	5.799E-116	1.3899161	0.749	0.314	1.272E-111
Gpm6b	1.114E-110	4.32205448	0.087	0.007	2.445E-106
Hif1a	3.884E-109	1.69177258	0.834	0.57	8.521E-105
Rflnb	7.614E-105	2.73275971	0.173	0.025	1.67E-100
Rnf19a	4.094E-100	2.25506397	0.462	0.16	8.9822E-96
Utrn	4.232E-100	1.92312564	0.574	0.24	9.2852E-96
Ipcef1	3.7563E-97	2.69742423	0.253	0.054	8.2409E-93
Sesn3	1.5003E-94	1.98169707	0.536	0.217	3.2915E-90
Gm12534	1.5989E-94	6.21144834	0.023	0	3.5077E-90
Inpp4b	2.8203E-94	2.06822502	0.431	0.144	6.1874E-90
Ctla4	5.4172E-92	1.3854737	0.653	0.289	1.1885E-87
Lef1	2.6066E-86	1.87425579	0.319	0.086	5.7186E-82
Esm1	4.8292E-86	5.0664195	0.052	0.003	1.0595E-81
Mbnl1	7.6763E-84	0.97091851	0.927	0.765	1.6841E-79
Fam84a	5.3577E-82	5.37790368	0.019	0	1.1754E-77
Fyb	8.1904E-79	1.1593293	0.805	0.508	1.7969E-74
Tespa1	3.4302E-78	2.35883205	0.321	0.097	7.5256E-74

B630019A10R	1.2754E-76	4.07628086	0.056	0.004	2.7982E-72
Tnfrsf4	1.5029E-76	1.51749929	0.457	0.17	3.2971E-72
Cd3e	4.9553E-76	1.2551475	0.635	0.291	1.0871E-71
Ift80	1.7324E-73	3.19402584	0.141	0.024	3.8007E-69
Malat1	3.4911E-73	0.63635934	1	0.999	7.6591E-69
2310001H17R	1.9179E-71	2.06590818	0.3	0.091	4.2077E-67
Gimap6	3.8405E-71	1.15780261	0.738	0.41	8.4257E-67
Cd4	1.0854E-69	1.66230186	0.414	0.155	2.3813E-65
Plcg1	9.7642E-69	2.24186202	0.257	0.071	2.1422E-64
Cd28	1.9797E-68	1.18905053	0.632	0.309	4.3432E-64
Lck	9.262E-67	1.54410764	0.471	0.199	2.032E-62
Il6st	2.4359E-66	2.25166216	0.314	0.106	5.3441E-62
Chd3	2.7195E-66	1.61959274	0.56	0.29	5.9662E-62
Smc4	7.317E-66	2.06671597	0.428	0.183	1.6053E-61
Shisa5	2.3497E-65	1.20029237	0.785	0.589	5.155E-61
Cd200	7.2327E-65	2.33354605	0.22	0.056	1.5868E-60
Prkca	8.2803E-65	2.21097002	0.244	0.067	1.8166E-60
Cd2	2.5396E-63	1.29165985	0.586	0.286	5.5717E-59
Skap1	6.5912E-63	1.48157819	0.496	0.224	1.446E-58
Smco4	1.1465E-62	2.12309214	0.318	0.109	2.5152E-58
Nav2	1.3146E-62	2.56361884	0.201	0.049	2.8841E-58
Prkcq	6.61E-62	2.44248227	0.197	0.048	1.4502E-57
Gm14718	3.3838E-61	2.86548365	0.096	0.014	7.4238E-57
Cd27	1.409E-60	1.68862893	0.318	0.107	3.0913E-56
Sh3gl3	3.148E-60	4.38158209	0.035	0.002	6.9064E-56
Igf1r	5.9519E-59	2.05215316	0.23	0.065	1.3058E-54
Raf1	4.4979E-58	1.93269114	0.375	0.154	9.8679E-54
Trp53inp1	4.836E-58	1.5950122	0.454	0.203	1.061E-53
Ttn	2.324E-57	3.34649414	0.119	0.021	5.0987E-53
Fam169b	2.3561E-56	1.89827933	0.283	0.093	5.169E-52
Ms4a6b	6.848E-56	1.63784684	0.492	0.253	1.5024E-51
St8sia1	7.6708E-56	2.65273769	0.133	0.026	1.6829E-51
Fyn	9.8601E-56	1.39775449	0.59	0.348	2.1632E-51
Rps27	1.0625E-55	0.42581597	0.998	0.984	2.3309E-51
Art2b	3.4617E-55	3.82713221	0.096	0.015	7.5947E-51
Rgs10	7.3406E-55	1.75634785	0.522	0.297	1.6105E-50
Eea1	1.0237E-54	1.96472248	0.452	0.229	2.2459E-50
Cd3g	1.2152E-54	0.97052406	0.625	0.315	2.6661E-50
Stat5b	2.0637E-54	1.40826493	0.536	0.281	4.5275E-50
Rhoh	2.3718E-54	1.42142481	0.536	0.275	5.2035E-50
Ttc3	4.7988E-54	2.06749989	0.295	0.105	1.0528E-49
Rplp1	6.9867E-54	0.38670399	0.998	0.983	1.5328E-49
Ddx24	2.9529E-52	1.20543665	0.693	0.506	6.4784E-48
Srpk2	4.2221E-52	1.79066292	0.426	0.2	9.2628E-48
Spock2	7.8183E-52	3.1067312	0.098	0.016	1.7153E-47
Lat	8.3889E-51	1.30926688	0.478	0.228	1.8404E-46
Susd4	1.055E-50	5.7135772	0.019	0.001	2.3146E-46
Arid5b	6.6105E-50	1.67378468	0.401	0.186	1.4503E-45

Adk	7.0716E-50	2.30863222	0.209	0.062	1.5514E-45
mt-Atp6	8.5878E-50	0.43990437	1	0.965	1.8841E-45
Zfp281	1.0302E-49	2.11826754	0.267	0.093	2.2603E-45
Mllt3	2.3423E-49	1.81024347	0.298	0.112	5.1387E-45
Elovl6	3.446E-49	2.70798779	0.141	0.032	7.5601E-45
Tspan5	5.2096E-48	2.55200768	0.155	0.038	1.1429E-43
Hey1	6.4908E-48	3.45711704	0.056	0.006	1.424E-43
Cd5	7.996E-48	1.63721337	0.339	0.137	1.7542E-43
Itpkb	8.5595E-48	1.25203176	0.614	0.387	1.8779E-43
Gpr83	1.1591E-47	5.03125878	0.023	0.001	2.543E-43
Cd247	1.301E-44	1.61123526	0.319	0.128	2.8542E-40
Zfp831	2.519E-44	2.62559811	0.091	0.016	5.5265E-40
Ptpn22	4.4432E-44	0.99701367	0.634	0.381	9.748E-40
Rpl12	6.2725E-44	0.4458354	0.995	0.947	1.3761E-39
Slc5a3	1.4239E-42	1.99116159	0.232	0.08	3.124E-38
Nt5e	1.599E-42	1.60583886	0.264	0.097	3.508E-38
Gm42418	1.6137E-42	0.26849399	1	1	3.5404E-38
Nxpe3	3.7597E-42	2.5202031	0.119	0.026	8.2485E-38
Gimap8	9.9376E-42	1.66389498	0.277	0.106	2.1802E-37
Cblb	1.376E-41	1.3871774	0.504	0.294	3.0188E-37
Gpr174	3.8795E-41	1.65476652	0.248	0.088	8.5112E-37
Trim59	1.1446E-40	2.04000486	0.269	0.106	2.5111E-36
Stk39	3.9774E-40	2.28748247	0.171	0.051	8.726E-36
Khdrbs1	9.7684E-39	1.27327391	0.6	0.44	2.1431E-34
Trbc1	2.0395E-38	1.22989173	0.482	0.262	4.4745E-34
Prrc2c	2.6498E-37	0.84046501	0.799	0.705	5.8133E-33
Gimap4	4.788E-37	1.05123972	0.51	0.283	1.0504E-32
Clk1	5.6885E-37	0.83284567	0.794	0.682	1.248E-32
AC119264.1	2.5208E-36	5.31182373	0.016	0.001	5.5304E-32
Fbn2	9.7348E-36	5.02458888	0.012	0	2.1357E-31
Gm9885	9.7778E-36	5.01342572	0.012	0	2.1452E-31
Rps29	1.2402E-35	0.31682321	1	0.986	2.7208E-31
Itgb8	2.6573E-35	2.61025405	0.061	0.009	5.8299E-31
Mrps6	5.843E-35	1.80735568	0.291	0.131	1.2819E-30
Pipox	4.0681E-34	5.8248767	0.01	0	8.925E-30
Ablim1	5.582E-34	1.03944999	0.497	0.288	1.2246E-29
Peli1	6.5838E-34	1.06917788	0.639	0.469	1.4444E-29
Icos	7.0498E-34	0.67000495	0.616	0.355	1.5467E-29
Dgka	1.185E-33	1.61897272	0.264	0.11	2.5997E-29
Faim	2.2096E-33	2.17003506	0.272	0.123	4.8477E-29
Ssh2	6.3543E-33	1.11293316	0.532	0.34	1.3941E-28
Hspa1b	6.5224E-33	1.49010219	0.335	0.162	1.4309E-28
Ets1	7.004E-33	0.56251869	0.902	0.666	1.5366E-28
Mgst2	5.8574E-32	2.85165291	0.079	0.016	1.2851E-27
Leprotl1	7.532E-32	1.11335125	0.496	0.316	1.6525E-27
Ptrh1	7.66E-32	3.01246455	0.065	0.011	1.6805E-27
Socs1	9.1348E-32	1.71222204	0.26	0.115	2.0041E-27
Ankrd12	5.0848E-31	1.24354054	0.455	0.274	1.1156E-26

mt-Co1	1.1561E-30	0.33725693	0.998	0.972	2.5363E-26
mt-Cytb	1.3293E-30	0.43964464	0.97	0.932	2.9163E-26
Ms4a4b	2.2522E-30	0.97072662	0.454	0.249	4.9411E-26
Prkch	2.5262E-30	1.37242311	0.318	0.155	5.5423E-26
Rasa1	3.4351E-30	1.80333111	0.225	0.093	7.5363E-26
Gtf2i	3.569E-30	1.3061905	0.314	0.152	7.8299E-26
Patj	7.4037E-30	2.31841106	0.082	0.018	1.6243E-25
Agfg1	7.4162E-30	1.50887371	0.347	0.186	1.627E-25
Ephx1	7.7627E-30	2.03422812	0.112	0.03	1.7031E-25
Gm37170	8.8939E-30	3.68794224	0.033	0.004	1.9512E-25
Tspan32	3.7372E-29	2.02271362	0.133	0.04	8.1992E-25
Rpsa	3.7452E-29	0.38949764	0.991	0.965	8.2165E-25
Klk1b27	8.5941E-29	3.52558886	0.023	0.002	1.8855E-24
Zfp36l2	1.5516E-28	0.87005513	0.651	0.474	3.404E-24
Pacsin1	2.0543E-28	2.68714198	0.065	0.012	4.507E-24
Batf	3.3962E-28	1.6073901	0.333	0.18	7.4508E-24
Ehd3	3.5157E-28	2.13562986	0.112	0.031	7.713E-24
Frat2	4.496E-28	2.04104238	0.145	0.048	9.8637E-24
Elf1	6.1747E-28	0.83644044	0.628	0.465	1.3547E-23
Ndrg3	6.1941E-28	2.27226256	0.171	0.063	1.3589E-23
Stk17b	1.0311E-26	0.53061239	0.887	0.766	2.2621E-22
Esyt2	1.7252E-26	1.08845417	0.421	0.252	3.7849E-22
Gm3739	1.7488E-26	4.25500131	0.012	0.001	3.8366E-22
Kbtbd11	5.0044E-26	1.35683176	0.257	0.119	1.0979E-21
Btla	5.7513E-26	1.67248887	0.19	0.076	1.2618E-21
Gm8369	6.4158E-26	0.66171774	0.454	0.256	1.4076E-21
Gimap9	7.8272E-26	1.33865798	0.279	0.135	1.7172E-21
Gm29114	9.5477E-26	4.61828736	0.012	0.001	2.0947E-21
Myh9	1.3717E-25	0.76716972	0.691	0.582	3.0093E-21
Cd6	1.5332E-25	1.16680895	0.302	0.15	3.3637E-21
Zc3h12d	1.964E-25	1.72447973	0.143	0.049	4.3089E-21
Tnik	3.7752E-25	1.39559867	0.213	0.091	8.2825E-21
Gm35363	4.0167E-25	3.61210183	0.024	0.002	8.8123E-21
Itk	4.0481E-25	0.77302222	0.508	0.318	8.881E-21
Cers4	1.1109E-24	1.56410328	0.227	0.102	2.4371E-20
Hspa1a	1.5144E-24	1.56507084	0.267	0.132	3.3225E-20
Saraf	1.5818E-24	1.04432203	0.52	0.38	3.4703E-20
5830468F06Ri	1.8573E-24	2.99349725	0.038	0.006	4.0747E-20
Sptb	1.9553E-24	4.63572981	0.014	0.001	4.2898E-20
Gimap1	3.6815E-24	0.99294791	0.447	0.277	8.0768E-20
Actn1	4.6956E-24	1.73837631	0.18	0.073	1.0302E-19
Ccdc126	9.1644E-24	2.85816172	0.044	0.007	2.0106E-19
Rps28	1.8093E-23	0.27821487	0.99	0.972	3.9695E-19
Ksr1	2.7728E-23	2.30593998	0.084	0.022	6.0832E-19
Gstp3	3.4591E-23	1.95042059	0.143	0.052	7.5889E-19
F2rl1	4.2435E-23	2.83647915	0.045	0.008	9.3097E-19
Camk4	5.7668E-23	1.28364517	0.277	0.141	1.2652E-18
Il16	2.9368E-22	1.41108338	0.225	0.105	6.443E-18

Dusp10	3.2475E-22	1.20127107	0.243	0.116	7.1247E-18
Ahi1	4.1701E-22	1.57106356	0.183	0.078	9.1488E-18
Osbpl6	4.3586E-22	4.73750068	0.01	0	9.5624E-18
Dgkz	1.0752E-21	1.88600889	0.183	0.08	2.359E-17
Gm26917	1.7119E-21	0.87561527	0.489	0.352	3.7556E-17
Msi2	1.7561E-21	1.17756174	0.365	0.222	3.8527E-17
Plagl1	1.8996E-21	2.72145848	0.07	0.017	4.1675E-17
mt-Nd4	2.0766E-21	0.37736407	0.955	0.908	4.5559E-17
Pptc7	2.0849E-21	1.70952676	0.206	0.096	4.5742E-17
Rabgap1l	2.1804E-21	1.056489	0.33	0.186	4.7837E-17
Crlf3	2.3226E-21	0.99752725	0.497	0.359	5.0955E-17
Rps16	3.3834E-21	0.25875951	0.997	0.981	7.4227E-17
Ikzf1	4.3211E-21	1.10884922	0.36	0.219	9.48E-17
Lbh	7.1809E-21	1.19794331	0.319	0.183	1.5754E-16
Arap2	1.7037E-20	0.8084358	0.569	0.404	3.7378E-16
Tnrc6b	1.7867E-20	0.750728	0.503	0.347	3.9199E-16
Arid4a	3.5353E-20	1.25939748	0.339	0.207	7.7561E-16
Znrf1	1.115E-19	1.34194452	0.251	0.133	2.4463E-15
Tnfrsf26	1.3281E-19	1.98867031	0.092	0.029	2.9138E-15
Bmp7	1.5389E-19	2.89248239	0.038	0.007	3.3761E-15
Rps15a	2.0742E-19	0.27879051	0.993	0.958	4.5505E-15
Ash1l	2.4921E-19	1.06014738	0.365	0.228	5.4674E-15
Socs3	2.7804E-19	1.06936626	0.45	0.312	6.0998E-15
Dtx1	3.8073E-19	1.42740007	0.141	0.055	8.3528E-15
Fus	5.7528E-19	0.55713442	0.794	0.75	1.2621E-14
Rapgef6	5.8951E-19	1.02036735	0.422	0.286	1.2933E-14
Celsr1	6.871E-19	2.48065781	0.056	0.013	1.5074E-14
Matk	7.4141E-19	2.28333385	0.11	0.039	1.6266E-14
Tmsb10	7.4763E-19	0.32687269	0.974	0.892	1.6402E-14
Cbx3	1.1342E-18	0.98441125	0.419	0.283	2.4882E-14
Themis	1.3579E-18	1.32138201	0.208	0.1	2.979E-14
Nek7	2.0807E-18	1.35862017	0.316	0.195	4.5648E-14
Limd2	3.7463E-18	0.84794241	0.452	0.318	8.219E-14
Traf3ip3	3.8128E-18	1.47882399	0.162	0.07	8.3649E-14
Cdkn1b	5.2337E-18	0.71291981	0.536	0.399	1.1482E-13
Stk26	5.9887E-18	1.37931401	0.199	0.096	1.3139E-13
Hsph1	8.9658E-18	1.37039134	0.293	0.172	1.967E-13
Acsl3	1.111E-17	2.00482821	0.105	0.037	2.4374E-13
1110034G24R	1.1379E-17	2.22520065	0.075	0.022	2.4965E-13
Foxp3	1.3902E-17	1.52049147	0.086	0.027	3.05E-13
Syt11	1.7872E-17	1.93097945	0.096	0.033	3.921E-13
Bzw2	1.9974E-17	1.27104066	0.297	0.177	4.3822E-13
mt-Co3	2.2535E-17	0.25773949	0.995	0.968	4.944E-13
Slc41a1	3.5089E-17	1.63448516	0.166	0.076	7.6981E-13
Tgfbr3	3.5881E-17	2.03403538	0.063	0.017	7.8719E-13
Trav14d-3-dv8	4.8992E-17	4.09398159	0.01	0.001	1.0748E-12
Pou6f1	4.9613E-17	1.80627877	0.087	0.028	1.0885E-12
Cxcr5	4.9732E-17	1.46097344	0.106	0.038	1.0911E-12

Nhsl1	5.5373E-17	4.16534269	0.014	0.001	1.2148E-12
Ldlrad4	5.7268E-17	1.59407786	0.124	0.049	1.2564E-12
Gypc	6.6452E-17	1.75525143	0.129	0.053	1.4579E-12
A630014C17R	6.7997E-17	3.73652742	0.012	0.001	1.4918E-12
Sbno2	8.0204E-17	1.2099664	0.361	0.247	1.7596E-12
Utp14b	1.738E-16	2.11538971	0.091	0.031	3.8131E-12
Fam189b	1.9677E-16	1.68741704	0.11	0.042	4.317E-12
Luc7l2	2.01E-16	0.57213856	0.729	0.662	4.4097E-12
Jun	2.1088E-16	0.88298306	0.408	0.279	4.6266E-12
Cbr1	2.8706E-16	1.95534935	0.086	0.029	6.2978E-12
Phf20l1	3.897E-16	0.95683556	0.431	0.311	8.5496E-12
Bicdl1	4.1303E-16	2.32066434	0.063	0.018	9.0614E-12
Zfp53	7.9372E-16	1.83761846	0.101	0.038	1.7413E-11
Fam78a	9.6698E-16	1.81447216	0.072	0.022	2.1215E-11
Arhgap45	9.6727E-16	0.89824862	0.393	0.274	2.1221E-11
Numa1	1.2656E-15	0.98629611	0.314	0.197	2.7767E-11
Psip1	1.3304E-15	1.11842744	0.253	0.145	2.9187E-11
Mycbp2	1.6159E-15	0.63381068	0.586	0.474	3.545E-11
Gpr146	1.6386E-15	1.93938678	0.145	0.067	3.595E-11
Rps17	1.644E-15	0.34607902	0.894	0.864	3.6068E-11
Ikzf4	1.807E-15	2.22410481	0.058	0.016	3.9644E-11
Rassf5	2.2542E-15	1.31352754	0.243	0.138	4.9454E-11
Satb1	2.9792E-15	0.55556482	0.682	0.54	6.536E-11
Lncpint	3.0277E-15	1.26469031	0.272	0.166	6.6424E-11
Slc9a9	3.4014E-15	1.85261725	0.141	0.065	7.4623E-11
Zcchc7	3.6458E-15	0.87581876	0.405	0.29	7.9985E-11
Sla	4.8631E-15	0.8222255	0.51	0.404	1.0669E-10
Setd4	5.1201E-15	2.45476181	0.045	0.011	1.1233E-10
Fip1l1	6.6601E-15	1.04838467	0.396	0.288	1.4612E-10
Fam118a	6.9244E-15	2.0133259	0.091	0.033	1.5191E-10
Lars2	8.7376E-15	0.45218657	0.787	0.733	1.9169E-10
Zap70	1.3375E-14	0.99308774	0.244	0.136	2.9343E-10
Gm42722	1.3455E-14	2.00011762	0.044	0.01	2.9519E-10
Gprasp1	1.4095E-14	1.77126784	0.08	0.027	3.0922E-10
Gm26551	1.4096E-14	2.06753498	0.058	0.016	3.0925E-10
Gm3468	1.4152E-14	3.1574735	0.012	0.001	3.1048E-10
mt-Nd2	1.4313E-14	0.4226138	0.815	0.779	3.1402E-10
1700019D03R	1.5749E-14	2.5120217	0.03	0.005	3.4552E-10
Chic1	1.6007E-14	2.56763074	0.038	0.008	3.5118E-10
Sept9	1.8502E-14	1.22478278	0.269	0.166	4.0593E-10
Atp11b	1.8579E-14	1.00458834	0.41	0.306	4.0761E-10
Cnst	1.8651E-14	1.8478712	0.089	0.033	4.0918E-10
Celf2	2.1814E-14	0.66432736	0.628	0.544	4.7858E-10
Ubac2	2.3054E-14	1.45487688	0.208	0.116	5.0578E-10
Nfatc1	2.3643E-14	1.08708619	0.281	0.174	5.1871E-10
Pitpnm2	2.5056E-14	1.85339766	0.065	0.02	5.497E-10
Itpr2	3.4616E-14	1.10908062	0.318	0.206	7.5944E-10
Inpp5f	3.6914E-14	1.74628075	0.091	0.034	8.0986E-10

Zfp292	3.8358E-14	0.93824987	0.36	0.248	8.4154E-10
St13	3.872E-14	0.92737837	0.438	0.348	8.4948E-10
Casc4	4.1265E-14	2.1030828	0.059	0.017	9.0532E-10
Stk4	4.581E-14	0.64762015	0.517	0.397	1.005E-09
Arhgap31	4.6695E-14	1.01073103	0.34	0.227	1.0244E-09
Tiam1	4.8939E-14	1.47235713	0.171	0.088	1.0737E-09
Gm3636	5.6176E-14	3.07418211	0.016	0.002	1.2324E-09
Kras	7.113E-14	0.76277788	0.527	0.439	1.5605E-09
Tspan3	7.3095E-14	1.79996015	0.18	0.096	1.6036E-09
Itga6	7.6654E-14	1.559131	0.117	0.05	1.6817E-09
Prg4	9.3396E-14	2.83094731	0.023	0.004	2.049E-09
Prpf38b	1.4034E-13	0.65600457	0.562	0.479	3.0789E-09
Bclaf1	1.475E-13	0.65788931	0.574	0.498	3.2361E-09
Gm2237	1.6271E-13	2.74818632	0.019	0.003	3.5696E-09
Tra2b	1.6612E-13	0.50053691	0.658	0.593	3.6445E-09
Gm14085	1.843E-13	2.73928408	0.026	0.005	4.0433E-09
Btg1	1.9566E-13	0.31060414	0.958	0.973	4.2925E-09
C030034L19R	2.0402E-13	2.6718526	0.023	0.004	4.4759E-09
Ifi208	2.0716E-13	2.1809716	0.049	0.013	4.5449E-09
Cbarp	2.0749E-13	1.70592591	0.122	0.054	4.5521E-09
Sigirr	2.5988E-13	1.80500877	0.08	0.029	5.7015E-09
Tra2a	3.331E-13	0.59808809	0.607	0.534	7.308E-09
Tasp1	3.7551E-13	1.81789028	0.079	0.028	8.2383E-09
Rundc3a	5.6907E-13	3.38766745	0.016	0.002	1.2485E-08
Kat6b	5.9361E-13	1.2939557	0.195	0.108	1.3023E-08
Il27ra	6.5057E-13	1.36308996	0.124	0.056	1.4273E-08
Hspe1	7.9576E-13	0.58368591	0.67	0.631	1.7458E-08
Smtn	9.7087E-13	3.20501916	0.023	0.004	2.13E-08
Aff3	9.7534E-13	1.18666316	0.096	0.038	2.1398E-08
Thada	1.0368E-12	1.54530146	0.101	0.042	2.2746E-08
Cbx6	1.0382E-12	1.88177122	0.058	0.018	2.2776E-08
Sh3rf1	1.0494E-12	2.20481408	0.049	0.014	2.3023E-08
Ccdc141	1.086E-12	2.96635926	0.016	0.002	2.3825E-08
Trbv3	1.0918E-12	2.98778483	0.024	0.004	2.3952E-08
Zfp612	1.1066E-12	2.72150201	0.016	0.002	2.4277E-08
Ap1s3	1.1783E-12	1.66398543	0.119	0.054	2.5852E-08
Dnaja1	1.2548E-12	0.47888009	0.649	0.57	2.7529E-08
Serbp1	1.3216E-12	0.42437763	0.794	0.749	2.8994E-08
Crbn	1.4198E-12	0.98048781	0.272	0.17	3.115E-08
Reps1	1.4459E-12	1.32540487	0.176	0.095	3.1722E-08
Arhgef1	1.8395E-12	0.84436007	0.4	0.295	4.0357E-08
Trbv20	3.0162E-12	3.18918222	0.017	0.002	6.6173E-08
Bcl6	3.2786E-12	1.23249785	0.222	0.134	7.1929E-08
Spsb1	3.3622E-12	1.89114959	0.07	0.025	7.3762E-08
Pear1	4.1198E-12	1.92619649	0.063	0.021	9.0385E-08
Smap1	4.2041E-12	1.01473143	0.372	0.279	9.2234E-08
Ptpn11	4.6093E-12	1.23196185	0.227	0.139	1.0112E-07
Eif4a2	4.6176E-12	0.74924842	0.515	0.444	1.0131E-07

Pdcd4	4.7307E-12	0.77100583	0.426	0.321	1.0379E-07
Git2	5.1342E-12	0.91140193	0.354	0.253	1.1264E-07
Cacna2d4	5.5017E-12	3.24969074	0.01	0.001	1.207E-07
Rasgef1a	5.9372E-12	2.38570449	0.031	0.007	1.3026E-07
Galnt9	5.952E-12	2.95479121	0.023	0.004	1.3058E-07
Tnrc6c	5.9833E-12	1.00914074	0.3	0.202	1.3127E-07
Spata13	6.6068E-12	0.9626768	0.218	0.128	1.4495E-07
Senp7	6.7195E-12	1.50365415	0.131	0.063	1.4742E-07
Tnfrsf14	6.9138E-12	2.22342846	0.049	0.014	1.5168E-07
Irf2bp2	7.754E-12	0.5920103	0.524	0.423	1.7012E-07
Arid5a	7.9936E-12	0.99316596	0.365	0.271	1.7537E-07
Rnf145	8.1214E-12	1.02895614	0.229	0.137	1.7818E-07
Atad2	8.2999E-12	0.95238401	0.272	0.174	1.8209E-07
Krba1	8.821E-12	1.93733481	0.044	0.012	1.9352E-07
Arhgap15	1.0686E-11	0.91360324	0.34	0.242	2.3444E-07
Tbcel	1.3785E-11	1.35984035	0.133	0.065	3.0243E-07
Kmt2a	1.5439E-11	0.70089021	0.429	0.328	3.3871E-07
Bmyc	1.5576E-11	1.59625292	0.101	0.044	3.4173E-07
Hopx	1.5924E-11	0.49586831	0.347	0.239	3.4936E-07
Zfp280d	1.707E-11	1.13929363	0.197	0.113	3.745E-07
Vps13a	1.9724E-11	1.00506133	0.264	0.171	4.3273E-07
Camk2d	2.0394E-11	0.92764301	0.304	0.205	4.4743E-07
Ltb	2.5634E-11	0.43679745	0.403	0.275	5.6239E-07
Kif1b	2.6153E-11	1.05816774	0.222	0.135	5.7378E-07
Fam210a	2.6251E-11	1.93374993	0.079	0.031	5.7592E-07
Ttc28	3.0308E-11	1.75628565	0.086	0.035	6.6492E-07
Asap1	3.3103E-11	1.47549197	0.194	0.115	7.2625E-07
Zfyve28	3.4097E-11	3.02707961	0.021	0.004	7.4806E-07
Matr3	3.51E-11	0.65577914	0.506	0.422	7.7005E-07
Zfpm1	4.108E-11	1.42159771	0.087	0.036	9.0126E-07
Coro2b	4.2756E-11	2.06293595	0.038	0.01	9.3802E-07
Dlg4	4.5256E-11	1.8589869	0.07	0.026	9.9286E-07
Gm49179	4.6302E-11	3.13869166	0.012	0.001	1.0158E-06
Jmjd1c	4.9205E-11	0.82960224	0.494	0.428	1.0795E-06
Clec2i	4.9854E-11	1.27647739	0.138	0.07	1.0937E-06
Ggt7	5.1885E-11	3.04343821	0.014	0.002	1.1383E-06
Swt1	5.2009E-11	1.14707012	0.237	0.149	1.141E-06
Srrm2	5.8453E-11	0.40339848	0.752	0.726	1.2824E-06
Tnrc6a	6.1933E-11	0.9521394	0.293	0.202	1.3587E-06
Rinl	6.4406E-11	0.89644247	0.262	0.169	1.413E-06
Itgal	6.6089E-11	0.73807981	0.415	0.32	1.4499E-06
Kdm5b	6.7992E-11	1.06173268	0.183	0.104	1.4917E-06
Mdn1	6.8927E-11	1.24065687	0.201	0.12	1.5122E-06
Tigd2	6.9093E-11	1.17735562	0.185	0.106	1.5158E-06
Tbc1d30	9.0386E-11	2.31870384	0.056	0.019	1.983E-06
Stim2	9.2444E-11	1.37797607	0.164	0.092	2.0281E-06
Lrrc32	1.0406E-10	2.42743605	0.058	0.02	2.2829E-06
Atrx	1.1904E-10	0.69941952	0.511	0.446	2.6117E-06

Fam53b	1.2385E-10	1.73275409	0.073	0.029	2.7171E-06
Ube2d3	1.243E-10	0.40459265	0.805	0.774	2.7269E-06
Ddx60	1.3904E-10	1.86596514	0.07	0.027	3.0503E-06
Tert	1.4229E-10	2.92844693	0.01	0.001	3.1216E-06
Gramd3	1.7846E-10	0.61427048	0.382	0.267	3.9153E-06
Gpr155	1.8938E-10	2.37120203	0.042	0.012	4.1548E-06
Bcl2	2.2503E-10	1.07627597	0.216	0.131	4.9369E-06
Ankmy2	2.291E-10	2.06642525	0.061	0.022	5.0261E-06
1190002N15R	2.4517E-10	1.54310666	0.122	0.061	5.3788E-06
Gm11579	2.5518E-10	3.10946942	0.014	0.002	5.5984E-06
Trappc12	2.5576E-10	1.49608138	0.105	0.049	5.6111E-06
Ext1	2.7009E-10	1.62065777	0.15	0.084	5.9255E-06
Atp9b	2.7465E-10	1.42580412	0.157	0.088	6.0256E-06
Grhl1	2.7788E-10	2.59416615	0.028	0.006	6.0965E-06
Cdh24	3.0335E-10	2.54239602	0.024	0.005	6.6552E-06
Atcay	3.225E-10	3.73381524	0.012	0.002	7.0753E-06
Rbm25	3.7593E-10	0.4805064	0.677	0.633	8.2475E-06
Rif1	4.4096E-10	1.06298704	0.215	0.133	9.6742E-06
Hdac7	4.7571E-10	1.07910675	0.152	0.082	1.0437E-05
Klhl3	4.8821E-10	2.61013108	0.023	0.005	1.0711E-05
Csnk1g3	4.8912E-10	0.75765031	0.293	0.201	1.0731E-05
Tmem64	5.2669E-10	0.9048051	0.335	0.237	1.1555E-05
Sec14l1	5.6956E-10	1.60772846	0.143	0.079	1.2495E-05
Prkacb	5.7795E-10	1.0077981	0.236	0.152	1.268E-05
S1pr1	5.8258E-10	0.48197812	0.277	0.176	1.2781E-05
Lancl3	6.5911E-10	3.83298132	0.01	0.001	1.446E-05
Map7	7.3672E-10	2.1680802	0.037	0.01	1.6163E-05
Rere	7.5416E-10	1.26698018	0.127	0.066	1.6545E-05
Adamts6	7.7184E-10	1.92249767	0.049	0.016	1.6933E-05
Rnf157	7.8163E-10	0.86837026	0.291	0.203	1.7148E-05
Fgf13	8.5087E-10	2.22095052	0.026	0.006	1.8667E-05
Nt5dc1	8.6107E-10	1.50017422	0.087	0.038	1.8891E-05
Fam43a	1.0887E-09	1.26795626	0.086	0.037	2.3884E-05
Adi1	1.0932E-09	1.54592756	0.07	0.028	2.3984E-05
Gm44148	1.3429E-09	3.4175449	0.01	0.001	2.9461E-05
Cep97	1.379E-09	1.81819685	0.07	0.028	3.0254E-05
Zscan12	1.4405E-09	2.37884523	0.028	0.007	3.1603E-05
Gimap7	1.4427E-09	0.61345257	0.279	0.181	3.1651E-05
Fas	1.5598E-09	1.04305259	0.192	0.118	3.422E-05
Slc43a1	1.9034E-09	3.2654409	0.01	0.001	4.1758E-05
Phip	1.9755E-09	0.8105627	0.358	0.273	4.3339E-05
Anp32a	1.9791E-09	0.53524841	0.483	0.401	4.342E-05
C2cd5	2.1482E-09	1.66602126	0.099	0.047	4.713E-05
4833420G17R	2.1737E-09	0.90615323	0.246	0.164	4.769E-05
Ptprcap	2.2277E-09	0.72827286	0.298	0.204	4.8873E-05
Sgip1	2.4604E-09	1.65761366	0.061	0.023	5.3979E-05
Fam102a	2.6046E-09	0.97990289	0.19	0.115	5.7142E-05
Dnajb1	2.936E-09	1.17407179	0.258	0.18	6.4413E-05

Grik5	3.1684E-09	2.26537504	0.028	0.007	6.9511E-05
Stat1	3.2286E-09	0.59890901	0.442	0.352	7.0833E-05
Nfatc3	3.4596E-09	0.58496801	0.393	0.292	7.5899E-05
Setd5	3.5422E-09	0.68220285	0.483	0.423	7.7712E-05
Cd40lg	3.5884E-09	0.72866082	0.169	0.096	7.8725E-05
Ptpn18	3.9552E-09	0.38533183	0.654	0.615	8.6774E-05
Igsf23	4.1157E-09	2.17447406	0.017	0.003	9.0294E-05
Mdm4	4.4676E-09	0.86767922	0.269	0.187	9.8014E-05
Fam168a	4.6E-09	1.50679014	0.14	0.078	0.00010092
Ppip5k2	5.5371E-09	1.41135695	0.108	0.054	0.00012148
B3gnt2	6.1097E-09	1.63131636	0.054	0.02	0.00013404
Arhgef12	6.581E-09	1.48829622	0.103	0.051	0.00014438
Rpl22l1	7.4177E-09	0.3634662	0.761	0.74	0.00016274
Nrn1	8.1054E-09	2.00019111	0.019	0.004	0.00017782
Afp	8.9277E-09	4.02719036	0.01	0.001	0.00019587
Kif21b	9.8259E-09	1.13961254	0.143	0.081	0.00021557
Rsbn1	9.8635E-09	0.9048503	0.232	0.154	0.00021639
Gm26759	1.0206E-08	1.56844983	0.061	0.024	0.0002239
Zeb1	1.0318E-08	0.5729484	0.447	0.353	0.00022636
Tlk1	1.1073E-08	0.87442032	0.257	0.178	0.00024293
Maz	1.1641E-08	1.15004118	0.145	0.083	0.00025539
Gprin3	1.1671E-08	1.61012836	0.065	0.026	0.00025606
6330403K07R	1.421E-08	2.29175502	0.023	0.005	0.00031175
Tspan13	1.4261E-08	0.67968903	0.489	0.427	0.00031287
Pkp4	1.4274E-08	1.62354484	0.087	0.041	0.00031315
Cnot6l	1.4804E-08	0.62009627	0.459	0.39	0.00032479
R3hdm1	1.5333E-08	0.77232552	0.323	0.239	0.00033639
Gm4841	1.5993E-08	3.3492998	0.012	0.002	0.00035087
Cyld	1.6093E-08	0.71863643	0.387	0.316	0.00035307
Mxd4	1.6105E-08	0.94837594	0.258	0.18	0.00035332
Dpp4	1.7918E-08	1.62911343	0.122	0.067	0.00039309
Jak2	1.8966E-08	0.43100715	0.527	0.45	0.00041608
Jak1	2.1083E-08	0.43311799	0.632	0.595	0.00046254
Dip2b	2.1374E-08	0.91490158	0.269	0.192	0.00046892
Nup153	2.1434E-08	1.00545727	0.232	0.159	0.00047025
Sept1	2.3109E-08	0.53058732	0.407	0.309	0.00050698
Adprm	2.47E-08	1.66700365	0.077	0.035	0.0005419
Rbm33	2.5812E-08	0.78336211	0.283	0.204	0.00056629
Hnrnpa0	2.6707E-08	0.43642709	0.63	0.608	0.00058592
Lrba	2.7183E-08	1.01535789	0.164	0.098	0.00059636
Prdm5	2.8101E-08	3.23781346	0.01	0.001	0.0006165
Supt20	2.932E-08	0.77363298	0.279	0.2	0.00064325
Son	3.2277E-08	0.37906605	0.743	0.72	0.00070813
Mlh3	3.6375E-08	2.33220791	0.031	0.009	0.00079802
Rpa1	3.8013E-08	1.35532799	0.112	0.059	0.00083397
Zbtb4	3.8236E-08	1.35714607	0.091	0.044	0.00083885
Ctdspl2	3.9235E-08	0.88129726	0.208	0.135	0.00086077
Bptf	4.3846E-08	0.66362953	0.379	0.299	0.00096194

Hbs1l	4.4229E-08	1.42202465	0.145	0.086	0.00097035
Asxl1	4.4735E-08	1.25959914	0.141	0.082	0.00098145
Hmgb1	4.735E-08	0.38154237	0.686	0.664	0.00103881
Ubald2	5.1534E-08	0.32610242	0.637	0.56	0.00113061
Tpr	5.2473E-08	0.6479537	0.551	0.514	0.00115121
Gm20069	5.3337E-08	1.54078477	0.058	0.023	0.00117017
Rpap3	5.5105E-08	1.46375227	0.087	0.042	0.00120895
Phactr2	6.633E-08	1.63117172	0.126	0.072	0.00145521
Pik3c2a	6.8087E-08	1.02797111	0.216	0.147	0.00149377
Carf	7.0002E-08	1.87552875	0.042	0.015	0.00153578
Tubb2a	7.0848E-08	1.81959558	0.12	0.068	0.00155433
Sh3kbp1	7.0995E-08	0.70883805	0.368	0.299	0.00155756
Ranbp9	7.1152E-08	1.16468225	0.176	0.112	0.001561
Phc3	7.2008E-08	0.97066433	0.211	0.14	0.00157979
Haus3	7.7082E-08	1.02379013	0.161	0.097	0.0016911
Selenow	8.1547E-08	0.45797006	0.569	0.522	0.00178906
2700097O09R	8.1995E-08	1.67282442	0.066	0.029	0.00179889
Vps54	8.4241E-08	0.94544096	0.202	0.134	0.00184816
Sntb1	8.515E-08	1.28707802	0.084	0.04	0.00186811
Ptp4a2	8.9089E-08	0.37104446	0.726	0.724	0.00195453
Gm15655	1.0061E-07	2.00146433	0.058	0.024	0.00220719
Chst2	1.0376E-07	1.45711521	0.061	0.026	0.00227643
Phf6	1.0393E-07	0.9099613	0.161	0.097	0.00228004
Gdpd3	1.1018E-07	2.29065016	0.068	0.031	0.00241723
Rac2	1.102E-07	0.39198248	0.621	0.58	0.00241762
Pde4d	1.1893E-07	0.82238831	0.239	0.165	0.00260919
A230050P20R	1.2546E-07	1.90931656	0.044	0.016	0.00275253
Ifi44	1.313E-07	2.03609622	0.031	0.01	0.00288054
Elk4	1.3137E-07	0.76869222	0.253	0.179	0.00288223
Dus2	1.3646E-07	1.24710358	0.075	0.035	0.00299384
Pxmp2	1.3744E-07	2.98315971	0.019	0.004	0.00301539
Rsrp1	1.4753E-07	0.60929298	0.366	0.293	0.0032366
Ctcf	1.5446E-07	0.61080014	0.358	0.278	0.00338872
Smchd1	1.8068E-07	0.64524803	0.485	0.444	0.0039639
Zfp827	1.8081E-07	2.3490807	0.023	0.006	0.00396682
Zfp141	2.0157E-07	1.81028226	0.038	0.013	0.00442235
Jund	2.1468E-07	0.36917621	0.707	0.687	0.00470977
Ewsr1	2.1646E-07	0.56907068	0.455	0.404	0.00474891
Rictor	2.1981E-07	0.60948941	0.389	0.321	0.00482248
Vamp1	2.257E-07	1.50849083	0.054	0.022	0.00495156
Ephb6	2.3056E-07	2.86786228	0.014	0.003	0.00505829
Gm9856	2.5589E-07	1.64793761	0.063	0.028	0.0056139
Hivep2	2.6409E-07	0.68329593	0.373	0.306	0.00579397
Itpk1	3.0978E-07	1.20293548	0.127	0.074	0.00679621
Rasgrp1	3.1857E-07	0.78719931	0.23	0.159	0.00698901
Naa16	3.1883E-07	1.21751242	0.087	0.044	0.00699472
Pgap1	3.2432E-07	1.08249489	0.108	0.059	0.00711529
Mettl13	3.6547E-07	1.4499829	0.049	0.019	0.00801803

Pycr2	3.6817E-07	1.38030653	0.089	0.046	0.00807717
Eml5	3.6895E-07	1.45633659	0.059	0.026	0.00809446
Frat1	3.8805E-07	1.75373406	0.051	0.021	0.0085135
Evl	3.9371E-07	1.16544995	0.194	0.132	0.0086375
Gucy1a1	3.9988E-07	2.90984049	0.012	0.002	0.00877302
Tnfrsf18	4.0273E-07	0.42410474	0.347	0.257	0.00883555
Sp3	4.2017E-07	0.81543552	0.276	0.205	0.00921817
Ankrd17	4.2031E-07	0.86125836	0.302	0.235	0.00922115
Fam241a	4.3486E-07	1.13950894	0.129	0.076	0.00954048
Adgrl1	4.4276E-07	1.32915234	0.082	0.041	0.00971375
Usp33	4.5622E-07	1.32843515	0.086	0.043	0.01000903
Rasal3	4.799E-07	1.08430762	0.12	0.069	0.01052851
Hnrnpa2b1	4.858E-07	0.29265749	0.792	0.77	0.01065793
Stim1	5.0403E-07	0.86001003	0.213	0.147	0.01105797
Rap1gds1	5.1393E-07	1.08877421	0.176	0.117	0.01127514
Rprd2	6.0923E-07	0.99624165	0.178	0.118	0.01336594
Setd7	6.1606E-07	1.03726494	0.117	0.067	0.01351566
Xaf1	6.1659E-07	1.21165621	0.101	0.055	0.01352744
Slc26a11	6.581E-07	1.54926123	0.054	0.023	0.01443802
Gm15340	6.7095E-07	1.62973196	0.038	0.014	0.01471998
Hnrnph1	6.947E-07	0.50254311	0.541	0.491	0.01524109
Slfn1	7.0112E-07	0.70670984	0.124	0.071	0.01538179
Lclat1	7.0322E-07	1.82959782	0.044	0.017	0.01542785
Zer1	7.3455E-07	1.21727447	0.087	0.045	0.01611521
Rbm39	7.4343E-07	0.26797684	0.867	0.862	0.01631013
Tcof1	7.8266E-07	0.88195952	0.197	0.133	0.01717074
Acin1	8.1152E-07	0.57465501	0.422	0.365	0.01780386
Tnfaip8	8.5202E-07	0.59745669	0.433	0.388	0.01869243
Thy1	8.8131E-07	0.45776082	0.332	0.247	0.01933504
Tcf12	8.8661E-07	0.46261858	0.361	0.282	0.01945136
Prps2	8.9548E-07	1.19933542	0.101	0.056	0.01964602
Zfp654	9.4799E-07	1.26226833	0.105	0.059	0.02079791
Mum1	9.5017E-07	1.36258719	0.082	0.042	0.0208457
Gm15283	1.0049E-06	1.62090089	0.047	0.019	0.02204748
Sdf4	1.0295E-06	0.57059971	0.421	0.369	0.02258596
Tmem154	1.0911E-06	1.25797039	0.066	0.031	0.02393725
Zfp946	1.1359E-06	2.0781691	0.035	0.012	0.0249199
Prpf18	1.1578E-06	0.82079247	0.199	0.136	0.02540103
Ifi213	1.1714E-06	1.43129614	0.082	0.042	0.02569995
Apobec3	1.2033E-06	0.83959723	0.23	0.165	0.02639999
Nfia	1.2061E-06	1.91976287	0.051	0.021	0.026461
Kcnn4	1.22E-06	1.13238744	0.145	0.091	0.02676504
Emsy	1.2346E-06	0.78828643	0.264	0.196	0.02708616
Cdk2ap2	1.293E-06	0.34949474	0.461	0.406	0.02836611
Rc3h2	1.2974E-06	0.81587764	0.25	0.184	0.02846439
Akna	1.3108E-06	0.74942551	0.283	0.217	0.02875686
Kcnq1ot1	1.4608E-06	1.09393049	0.195	0.134	0.03204781
Sema4f	1.5382E-06	2.14289434	0.028	0.009	0.03374653

Por	1.5845E-06	1.09430557	0.272	0.215	0.03476306
Stat5a	1.5872E-06	0.94865776	0.246	0.185	0.03482181
Rnf139	1.6411E-06	1.28348498	0.122	0.073	0.03600416
Zc3h6	1.6519E-06	1.56428082	0.045	0.018	0.03624
4933427D14R	1.7366E-06	1.96598435	0.035	0.013	0.03810011
Rbl1	1.7407E-06	1.44961729	0.066	0.032	0.03818848
Angptl2	1.7418E-06	3.09798294	0.021	0.006	0.03821357
Pfn2	1.7459E-06	2.54161474	0.019	0.005	0.03830389
Rab37	1.7612E-06	1.2844872	0.073	0.036	0.03863981
Rundc3b	1.7705E-06	1.13811804	0.108	0.062	0.03884336
Ldb1	2.1207E-06	0.88874621	0.171	0.113	0.04652602
Hdhd5	2.2232E-06	1.47589945	0.047	0.02	0.04877436
Gemin8	2.2966E-06	2.04096688	0.023	0.007	0.05038533
Hmg20a	2.336E-06	1.59171037	0.079	0.041	0.05124849
Ifi209	2.4471E-06	0.92580848	0.173	0.115	0.05368694
Aebp2	2.4627E-06	0.27730858	0.597	0.526	0.05402913
Ly9	2.4742E-06	1.02321116	0.122	0.073	0.05428051
Trim56	2.5033E-06	0.89971931	0.134	0.082	0.05491915
Trim34a	2.5897E-06	1.17679196	0.079	0.04	0.05681479
Cep192	2.6621E-06	1.00804451	0.127	0.077	0.05840433
Pcmtd2	2.6865E-06	0.95213439	0.154	0.098	0.05893914
Ypel2	2.7291E-06	1.89432386	0.042	0.017	0.05987465
Tex10	2.9355E-06	1.06128902	0.161	0.106	0.06440163
Setbp1	2.9625E-06	1.21867398	0.051	0.022	0.06499368
Tmtc4	2.9693E-06	2.25632999	0.023	0.007	0.06514413
Zwint	2.9983E-06	1.24298742	0.124	0.076	0.06577968
Bex3	3.1163E-06	0.95192714	0.262	0.203	0.06836866
Xrn1	3.4234E-06	1.07842061	0.18	0.124	0.0751067
Rragd	3.5106E-06	1.94057482	0.024	0.007	0.07701888
Xrcc5	3.5144E-06	1.5577379	0.04	0.016	0.07710314
Arhgap39	3.5861E-06	1.60730558	0.051	0.022	0.07867493
Zfp644	3.7485E-06	0.6684283	0.277	0.213	0.08223741
Mlec	3.7615E-06	1.03309262	0.202	0.145	0.08252449
Gpbp1l1	3.7728E-06	0.76752726	0.251	0.19	0.08277245
Cir1	3.7849E-06	0.75048081	0.295	0.231	0.08303607
Zfp945	3.9529E-06	1.3610958	0.077	0.04	0.08672306
Rnf2	4.2235E-06	0.91774995	0.155	0.101	0.09265873
Mitd1	4.3202E-06	0.9189448	0.092	0.051	0.0947815
Huwe1	4.8423E-06	0.63249594	0.384	0.333	0.1062352
Cdk17	4.9974E-06	0.88429555	0.202	0.143	0.10963758
Mogat1	5.0708E-06	2.77607811	0.01	0.002	0.11124813
Rnf38	5.5968E-06	1.15859708	0.122	0.075	0.12278879
Tubb2b	5.6841E-06	1.93556189	0.031	0.011	0.12470248
Tle4	5.7293E-06	0.65964547	0.302	0.238	0.12569405
Trat1	5.7946E-06	0.55903363	0.14	0.086	0.12712698
Ppm1b	6.1153E-06	1.00077733	0.185	0.13	0.13416386
G3bp2	6.2806E-06	0.66044981	0.316	0.256	0.13779001
Gpatch8	6.4674E-06	0.75342398	0.291	0.234	0.14188846

Zfp773	6.5849E-06	1.85214151	0.031	0.011	0.14446556
Stx6	6.6017E-06	0.97435641	0.18	0.124	0.14483558
Rab11fip4	6.623E-06	1.58376091	0.049	0.022	0.14530293
Chd8	6.6636E-06	0.81110617	0.23	0.168	0.1461923
Mat2b	6.754E-06	0.59550074	0.311	0.247	0.14817647
Srsf1	6.9407E-06	0.70743727	0.251	0.188	0.15227138
Twsg1	6.9515E-06	1.04395688	0.112	0.066	0.15250877
Upf2	7.1195E-06	0.88708073	0.213	0.155	0.15619446
Tanc1	7.3712E-06	1.65323286	0.04	0.016	0.1617159
Gatad2b	7.4808E-06	0.6639761	0.316	0.256	0.16412189
Crebrf	7.5125E-06	0.5509718	0.354	0.292	0.16481743
4932422M17F	7.6219E-06	1.54695698	0.044	0.018	0.16721606
Zfp12	7.9757E-06	2.05628354	0.026	0.009	0.17497918
Ubb	7.9811E-06	0.25413047	0.852	0.821	0.17509781
Dis3l2	8.4356E-06	1.36169087	0.079	0.042	0.18506802
Parp14	8.6053E-06	0.73868189	0.234	0.175	0.18879157
Sp100	8.9335E-06	0.62638795	0.319	0.257	0.19599235
Dnaja4	9.0514E-06	1.48674088	0.075	0.039	0.19857874
Gfi1	9.9236E-06	1.09037486	0.066	0.033	0.21771277
Spry1	1.0407E-05	1.07718107	0.052	0.024	0.22832095
Nipsnap1	1.0727E-05	1.51049328	0.042	0.018	0.2353425
Cipc	1.0842E-05	1.02542792	0.148	0.099	0.23786791
Lax1	1.1656E-05	1.12409032	0.07	0.036	0.25571548
Stag2	1.1818E-05	0.72627509	0.304	0.248	0.25926871
Pde12	1.1982E-05	1.32960383	0.086	0.048	0.26287293
Tsc22d4	1.2731E-05	0.63535581	0.337	0.28	0.27929605
Lman2l	1.279E-05	1.37019024	0.077	0.041	0.28059508
Chd6	1.3132E-05	0.77178432	0.22	0.162	0.28810628
Cbx7	1.3155E-05	1.00672017	0.086	0.047	0.2886107
Zkscan7	1.3835E-05	2.47998413	0.016	0.004	0.303521
Sccpdh	1.405E-05	1.8053484	0.026	0.009	0.30823976
Ankrd11	1.4385E-05	0.36390113	0.623	0.598	0.31558857
Trim21	1.5031E-05	1.67474787	0.042	0.018	0.32976045
Inpp1	1.5449E-05	0.91878224	0.126	0.079	0.33892484
Aldh5a1	1.546E-05	2.65081256	0.01	0.002	0.33917892
Iigp1	1.6137E-05	1.22873679	0.063	0.032	0.35402337
Ilf2	1.6258E-05	0.74491299	0.239	0.179	0.35668258
Samhd1	1.6414E-05	0.62462797	0.414	0.372	0.36010657
Arhgap20	1.6681E-05	3.43999289	0.012	0.003	0.3659618
Mink1	1.6795E-05	1.17668562	0.112	0.069	0.36847188
Fam199x	1.6852E-05	1.27532305	0.058	0.028	0.36971531
Snrnp70	1.7361E-05	0.37371535	0.548	0.522	0.38087663
Fam107b	1.82E-05	0.30631553	0.609	0.564	0.39928269
Armc9	2.0188E-05	2.35540624	0.019	0.006	0.4429122
Clip3	2.0336E-05	2.19233121	0.01	0.002	0.44615172
Zbtb20	2.1552E-05	0.68642793	0.257	0.199	0.47282791
Kansl1	2.1745E-05	0.56096066	0.36	0.305	0.47705899
Tbc1d10c	2.2346E-05	0.90563403	0.112	0.068	0.49025591

Jade2	2.3043E-05	1.04838689	0.086	0.048	0.50554735
Abhd8	2.3089E-05	1.58397225	0.063	0.032	0.50654384
Txlnb	2.3174E-05	2.71157512	0.012	0.003	0.50840931
Map2k6	2.5683E-05	2.31435116	0.021	0.007	0.56346297
2610021A01R	2.5836E-05	1.54159428	0.033	0.013	0.56680665
Dtl	2.67E-05	1.24450072	0.051	0.024	0.58577018
Eif4ebp2	2.7047E-05	0.84153719	0.23	0.177	0.59339365
Pnn	2.7056E-05	0.68526741	0.307	0.252	0.59358582
4921524J17Ril	2.7227E-05	0.92606107	0.155	0.106	0.59732474
Rbm5	2.7325E-05	0.45184158	0.408	0.361	0.59947806
Ttc39b	2.7915E-05	0.80271203	0.255	0.202	0.61242113
Map2k7	2.7919E-05	0.89390141	0.173	0.122	0.61252044
Jpx	2.9781E-05	1.84650121	0.031	0.012	0.65335679
Mkrn2	2.9855E-05	1.16150619	0.082	0.046	0.6549944
Ipmk	3.08E-05	0.45602921	0.206	0.148	0.67573037
Tshz2	3.0979E-05	2.10630376	0.019	0.006	0.6796489
Ythdc1	3.1012E-05	0.45505964	0.536	0.509	0.68037514
Atp9a	3.2202E-05	1.89208877	0.021	0.007	0.70647003
Nsd1	3.3394E-05	0.73894757	0.281	0.226	0.73262036
Insr	3.3679E-05	1.2510028	0.073	0.04	0.73888973
Carmil2	3.5433E-05	1.33220191	0.051	0.024	0.77735464
Rnf167	3.6829E-05	0.78982899	0.157	0.108	0.80799391
Fam8a1	3.799E-05	1.01079104	0.08	0.045	0.83346787
Tnks2	3.885E-05	0.64006392	0.314	0.263	0.85232505
Ankrd46	3.8925E-05	1.43339233	0.042	0.019	0.85398016
Usp34	4.0104E-05	0.64129964	0.321	0.272	0.87984821
Smg6	4.0422E-05	0.8137672	0.188	0.135	0.88682636
Zufsp	4.1469E-05	0.67243132	0.283	0.226	0.90979128
Cited2	4.3019E-05	0.83584505	0.216	0.164	0.94378837
Adss	4.3171E-05	0.85088722	0.3	0.249	0.94712291
Ica1l	4.4327E-05	1.99422715	0.016	0.004	0.97248081
Phf2	4.494E-05	1.09354549	0.11	0.069	0.98594396
Ddb2	4.4942E-05	1.03435633	0.065	0.034	0.9859884
Asxl2	4.5528E-05	0.86538094	0.225	0.173	0.99884378
Thap3	4.6197E-05	0.99534013	0.101	0.062	1
Neil2	4.6472E-05	1.97790245	0.012	0.003	1
Rcor1	4.8084E-05	0.80841192	0.161	0.111	1
Wdr82	4.9374E-05	1.02006905	0.147	0.101	1
Ssbp2	4.9524E-05	1.16328678	0.086	0.05	1
Ranbp10	4.97E-05	1.16308528	0.098	0.059	1
Rufy2	4.9998E-05	1.07369403	0.091	0.053	1
Acyp1	5.145E-05	1.28630693	0.077	0.043	1
Rnaset2b	5.2738E-05	1.04266774	0.136	0.092	1
Gm47015	5.3578E-05	0.98910094	0.075	0.041	1
Lsm14a	5.3578E-05	0.8079079	0.232	0.183	1
Adipor2	5.4497E-05	0.71724479	0.253	0.201	1
Hspd1	5.4599E-05	0.73203663	0.373	0.334	1
Oas1c	5.531E-05	1.92729419	0.019	0.006	1

Ccdc69	5.5456E-05	1.47892913	0.037	0.016	1
Rbak	5.8782E-05	2.03393178	0.028	0.011	1
Tdp2	5.8863E-05	1.04177874	0.112	0.071	1
Hibadh	6.0157E-05	0.94248095	0.133	0.089	1
Heca	6.1628E-05	0.62682115	0.283	0.229	1
Nr2c2	6.265E-05	0.7367029	0.216	0.164	1
Kri1	6.4015E-05	1.11030894	0.098	0.06	1
Scd2	6.48E-05	1.27921449	0.098	0.06	1
Cox20	6.5866E-05	0.85303183	0.201	0.151	1
Tsc1	6.7177E-05	1.06502507	0.126	0.083	1
Mrpl52	7.0101E-05	0.36904671	0.539	0.512	1
Ezh2	7.1691E-05	0.81950062	0.138	0.093	1
Kif20b	7.1744E-05	1.19121633	0.045	0.021	1
St8sia6	7.2099E-05	1.87109035	0.04	0.018	1
Tesc	7.3276E-05	0.65525526	0.166	0.114	1
Rnaset2a	7.4091E-05	1.05981361	0.195	0.151	1
Cdk5r1	7.4188E-05	1.43304524	0.054	0.027	1
Pms2	7.514E-05	1.41754505	0.063	0.034	1
Zfp41	7.716E-05	2.80939745	0.01	0.002	1
Nlrc5	7.8731E-05	0.87699604	0.129	0.086	1
Ppp6r3	8.0812E-05	0.80132475	0.269	0.222	1
Cd84	8.3926E-05	0.67387909	0.255	0.205	1
Rps27rt	8.5313E-05	0.41135846	0.462	0.428	1
Chd9	8.7365E-05	0.95775388	0.131	0.088	1
Pot1a	8.7732E-05	1.09575191	0.073	0.041	1
Sash3	8.9751E-05	0.99217093	0.101	0.063	1
Kctd18	9.0371E-05	1.06813169	0.082	0.048	1
Rsbn1l	9.0727E-05	0.50389894	0.405	0.361	1
Gm26514	9.2826E-05	2.17792562	0.019	0.006	1
Gm44777	9.4809E-05	1.71498617	0.028	0.011	1
Pja1	9.5202E-05	0.9508724	0.115	0.075	1
Fhit	9.7076E-05	2.15001563	0.01	0.002	1
4932438A13R	0.0001028	0.44664454	0.429	0.386	1
Fam92a	0.00010518	0.9999619	0.073	0.041	1
Zfp169	0.00010579	1.43212724	0.042	0.019	1
Nemp2	0.0001072	1.21110858	0.073	0.041	1
Plcl1	0.00010756	1.78455795	0.044	0.021	1
Phf3	0.00010769	0.50451217	0.394	0.355	1
Baz2a	0.00011177	1.00251197	0.175	0.129	1
Agpat3	0.00011326	1.02619295	0.138	0.096	1
Casc1	0.00011515	2.72782116	0.01	0.002	1
Dlg3	0.00011583	1.80736181	0.017	0.005	1
Zfp874a	0.00011831	1.47354463	0.044	0.021	1
Zbtb37	0.00011949	1.04824832	0.065	0.035	1
Nipbl	0.0001199	0.45240405	0.447	0.411	1
D3Ertd751e	0.00012021	1.3282725	0.04	0.018	1
Setx	0.00012268	1.00770752	0.145	0.101	1
P2rx7	0.00012711	1.18411719	0.082	0.049	1

Synj2bp	0.00012843	0.79252583	0.143	0.099	1
Unkl	0.00012914	1.22106262	0.059	0.032	1
Fam193a	0.00012928	0.956955	0.159	0.114	1
Inpp4a	0.00012945	1.21383908	0.096	0.06	1
Mtf2	0.00013107	0.76495202	0.187	0.139	1
Dopey1	0.00013236	0.93928153	0.115	0.075	1
Usp6nl	0.00013262	1.14047353	0.117	0.077	1
Vmp1	0.00013816	0.48398656	0.373	0.338	1
2810454H06R	0.00013876	1.33099126	0.07	0.04	1
Bmi1	0.0001408	1.17987739	0.072	0.041	1
Pde3b	0.0001445	0.60659079	0.318	0.269	1
Hnrnpu	0.00014611	0.38803934	0.539	0.534	1
Trps1	0.00014767	0.56750666	0.353	0.307	1
Lnx2	0.00014892	1.33753588	0.075	0.044	1
Arhgap9	0.00015128	1.06488428	0.14	0.098	1
Zfp995	0.00015408	2.12213657	0.026	0.01	1
H2-Q4	0.00015545	0.57983749	0.307	0.257	1
Gm10369	0.00015638	2.64534645	0.012	0.003	1
Thumpd2	0.00016207	1.49570955	0.024	0.009	1
Srpk1	0.00016661	0.7040783	0.236	0.187	1
Zfp950	0.00016812	1.21195581	0.073	0.042	1
Klf2	0.00017443	0.59156951	0.284	0.226	1
Ggact	0.00017444	1.85062517	0.023	0.008	1
Rapgef1	0.000178	0.88481393	0.148	0.105	1
Gm26825	0.0001784	1.44700463	0.052	0.027	1
Bpgm	0.00018258	1.42702754	0.042	0.02	1
Birc6	0.00018343	0.50208683	0.398	0.362	1
Gm17767	0.00018452	2.64231402	0.01	0.003	1
Armcx6	0.0001874	2.23501896	0.017	0.006	1
Slc25a36	0.00019045	0.62847113	0.244	0.193	1
Nfrkb	0.00019396	0.89809409	0.12	0.08	1
Rab6b	0.00020092	1.68061278	0.031	0.013	1
Serinc5	0.00021379	1.41105492	0.049	0.025	1
Fam53a	0.00021484	1.25541397	0.065	0.036	1
Ylpm1	0.00022055	0.99760679	0.112	0.074	1
Xrcc4	0.00022195	1.13745954	0.058	0.031	1
Trbv2	0.00022691	2.07285427	0.017	0.006	1
Usp18	0.00023238	1.15958236	0.037	0.017	1
Mndal	0.00023407	0.69447319	0.312	0.267	1
Bhlhb9	0.00023485	1.31399255	0.031	0.014	1
Ryk	0.00024309	1.55225548	0.042	0.02	1
Ppm1d	0.00024319	1.19927367	0.087	0.054	1
Zc3h14	0.00024411	0.8670267	0.162	0.118	1
Ociad1	0.00024603	0.66041925	0.283	0.239	1
Tmem71	0.00024961	0.83593832	0.106	0.069	1
Phf14	0.00026289	0.85511845	0.183	0.139	1
Gba2	0.00026305	1.48835995	0.028	0.012	1
Ung	0.00026362	1.59537831	0.03	0.013	1

Casp8	0.0002651	0.96557408	0.155	0.113	1
Prpf39	0.00026652	0.53371316	0.271	0.221	1
Rnf43	0.0002813	1.74865264	0.028	0.012	1
Itpr3	0.0002842	0.6722669	0.112	0.073	1
Inpp5b	0.00028718	1.41873417	0.056	0.03	1
Ppp2r5c	0.00029025	0.63159003	0.318	0.277	1
Phkb	0.00029194	1.13456651	0.058	0.031	1
Zfp955b	0.0002987	2.5468487	0.014	0.004	1
Pex26	0.00029887	1.92320361	0.017	0.006	1
Gltp	0.00030326	0.84122844	0.225	0.183	1
Tecpr1	0.00031425	0.54081552	0.272	0.224	1
Mta1	0.00031783	1.11084201	0.112	0.075	1
Rgs3	0.0003224	1.42973131	0.058	0.032	1
Ubtf	0.00032826	0.70626759	0.208	0.164	1
Jakmip1	0.00033366	0.59571776	0.192	0.143	1
Fam46c	0.00034369	0.7430043	0.26	0.216	1
Gsk3b	0.00034612	0.66603929	0.293	0.25	1
Nsd3	0.00035408	0.51036205	0.445	0.411	1
Zfp1	0.00035591	1.16247906	0.068	0.04	1
Tnfrsf22	0.00035795	2.56196601	0.016	0.005	1
Mapk1ip1	0.00036065	1.29756707	0.042	0.021	1
Cbx4	0.00036263	0.70784605	0.164	0.119	1
Faah	0.00036772	0.67651467	0.15	0.106	1
Psenen	0.00037351	0.50719978	0.384	0.362	1
2700049A03R	0.00037579	1.79922561	0.026	0.011	1
Cacybp	0.00038548	0.78125359	0.223	0.18	1
Klhl24	0.00038828	0.5623426	0.339	0.299	1
Slc35b3	0.0003916	1.05130814	0.089	0.056	1
Ypel3	0.00040151	0.5535979	0.272	0.226	1
Itm2a	0.00041453	0.98035498	0.037	0.017	1
Fam172a	0.00041793	0.81085765	0.18	0.138	1
Dtd1	0.00042614	1.14615402	0.075	0.045	1
Irf2bpl	0.00042799	0.94643046	0.133	0.093	1
Galnt6	0.00043645	1.10991958	0.119	0.082	1
Aak1	0.0004367	0.67229912	0.173	0.129	1
Pds5a	0.0004588	0.5977033	0.251	0.206	1
Dzip1	0.00046052	1.64983546	0.017	0.006	1
Ahsa1	0.00047096	1.05923658	0.155	0.116	1
Ncoa3	0.00047605	0.79448187	0.194	0.15	1
Arhgap4	0.0004788	0.96506937	0.112	0.075	1
Hectd2	0.00048396	2.06718268	0.017	0.006	1
Gm2245	0.000484	1.28449407	0.033	0.015	1
Pip4k2b	0.00048873	1.39851798	0.054	0.03	1
Adamts10	0.00048967	1.48731465	0.047	0.025	1
Nespas	0.0004919	2.68404949	0.01	0.003	1
Sept6	0.00049199	0.59817793	0.25	0.203	1
Tctex1d1	0.00049306	2.45011477	0.014	0.004	1
N4bp2l2	0.00049733	0.44282831	0.435	0.402	1

mt-Nd5	0.00050223	0.34406961	0.557	0.562	1
Capn3	0.00050417	2.3095729	0.012	0.003	1
Atl3	0.00052098	0.88821903	0.152	0.112	1
Ikbke	0.00052168	0.87175483	0.089	0.057	1
Sltm	0.00055733	0.47715752	0.351	0.311	1
Abi2	0.00055819	1.3330868	0.051	0.027	1
Zfp638	0.00056435	0.73152281	0.241	0.201	1
Gimap5	0.00056525	0.2745179	0.288	0.228	1
Med14	0.00058384	1.0049567	0.126	0.088	1
Poll	0.00058842	1.83931989	0.023	0.009	1
Als2cl	0.00058897	1.43798148	0.024	0.01	1
Arid1a	0.0005899	0.65774544	0.243	0.199	1
Nlk	0.00059135	1.04096339	0.098	0.064	1
Gtf3c3	0.00059292	1.20959593	0.065	0.038	1
Foxo1	0.00059389	0.51461409	0.36	0.31	1
Phf11c	0.00059484	1.7143722	0.021	0.008	1
Ifi206	0.00060281	1.10504664	0.059	0.034	1
Ankrd13b	0.00060747	3.0708235	0.01	0.003	1
Cyth3	0.00060808	1.02460349	0.169	0.13	1
Atxn7	0.00061636	0.78275418	0.175	0.133	1
Plekhg2	0.00061757	1.48472243	0.058	0.033	1
Zfp251	0.00062335	1.77129617	0.023	0.009	1
A930024E05R	0.00062664	0.93035215	0.086	0.054	1
Gramd1b	0.00062959	0.81449866	0.12	0.083	1
Paxbp1	0.00063193	0.53361938	0.274	0.227	1
Trip12	0.00064516	0.48834501	0.344	0.306	1
Fbxo33	0.00065186	0.74199955	0.159	0.118	1
Ddx39b	0.00065974	0.64529737	0.248	0.209	1
Cnn2	0.0006681	0.60447379	0.316	0.275	1
Pnisr	0.00066854	0.40006192	0.394	0.354	1
Rcbtb1	0.00067242	1.16793581	0.061	0.035	1
Mier1	0.00067276	0.43406912	0.44	0.408	1
Tchp	0.0006824	1.67645663	0.033	0.016	1
Bod1l	0.0006976	0.62299466	0.26	0.218	1
Thrap3	0.00070518	0.36461748	0.428	0.399	1
AU020206	0.00070783	0.53001821	0.312	0.267	1
Zmym4	0.00070802	1.14897574	0.08	0.05	1
Rbm18	0.00071064	1.05163223	0.117	0.082	1
Ldhb	0.00071285	1.88167457	0.031	0.015	1
Parp3	0.00073807	1.25203144	0.068	0.041	1
Poli	0.00074163	1.89422072	0.026	0.011	1
Gm17173	0.00077088	2.01042378	0.012	0.004	1
Nemf	0.00078976	0.78099157	0.188	0.148	1
Zmynd8	0.00079079	0.77199679	0.145	0.106	1
Notch1	0.00080494	0.7485169	0.157	0.116	1
Pex1	0.00081069	1.31297104	0.038	0.019	1
Erc1	0.00081104	1.20968784	0.047	0.025	1
Hook1	0.00082572	0.89150156	0.052	0.029	1

Zmym5	0.00084647	0.70502184	0.202	0.16	1
Lta	0.00085019	1.21077035	0.03	0.013	1
Hibch	0.00085025	1.31771312	0.033	0.016	1
Prps1	0.00085263	0.95942525	0.12	0.085	1
Arhgap27	0.00086861	1.46989694	0.049	0.027	1
Nap1l4	0.00087011	0.65848213	0.227	0.188	1
Lin9	0.00087185	1.19081679	0.052	0.029	1
Rb1cc1	0.00087887	0.49970399	0.3	0.258	1
Zfp260	0.00088204	1.06093385	0.099	0.067	1
Ube2h	0.0008832	0.69746358	0.251	0.211	1
Zfp579	0.00088399	1.57874655	0.024	0.01	1
Nucks1	0.00089997	0.37471824	0.459	0.434	1
Stx11	0.00091951	0.77472436	0.22	0.182	1
5033430I15Ril	0.00096183	1.30954953	0.026	0.011	1
AI504432	0.00098038	0.94883189	0.154	0.115	1
Zfp362	0.00098253	1.1333667	0.061	0.036	1
Atf7ip	0.000992	0.60334712	0.246	0.205	1
Zfp788	0.00101063	1.38771654	0.037	0.018	1
Abhd17c	0.00102649	0.67836803	0.112	0.076	1
Zfp595	0.00103193	1.28716768	0.035	0.017	1
Mif	0.00103595	0.48428676	0.496	0.481	1
Zfp772	0.00104386	2.05047958	0.014	0.005	1
Fam193b	0.00104732	0.86768866	0.079	0.049	1
Pkd1	0.00107654	0.95244019	0.077	0.048	1
Scaf8	0.00108182	0.78671337	0.168	0.129	1
Ptpn7	0.00110342	0.98068307	0.092	0.061	1
Lbr	0.00113895	0.53707037	0.29	0.248	1
Rapgef5	0.00115421	1.53801869	0.023	0.009	1
Gm11131	0.00115863	1.42358058	0.024	0.01	1
Map4k2	0.00116049	0.77676396	0.141	0.103	1
Creb3l2	0.00117339	1.41074978	0.068	0.042	1
Upf3b	0.00117417	1.01314764	0.12	0.085	1
Samd9l	0.00118931	0.77211262	0.168	0.128	1
Dicer1	0.00120134	0.75350142	0.175	0.135	1
Ppip5k1	0.0012086	1.86573148	0.03	0.014	1
Ifi203	0.0012158	0.56797865	0.269	0.228	1
Kmt5b	0.00123378	0.6994879	0.206	0.165	1
Emg1	0.00128391	0.50809313	0.337	0.305	1
Pias2	0.00129246	0.95873889	0.077	0.049	1
Prkcz	0.00129386	1.04819221	0.047	0.026	1
Gtpbp8	0.00129627	1.51427975	0.037	0.019	1
Prkab1	0.00131405	1.18392599	0.061	0.037	1
Tbc1d22b	0.00131798	1.48756787	0.038	0.02	1
Pogz	0.00132773	0.98533638	0.073	0.046	1
Krit1	0.00133333	0.41819843	0.337	0.293	1
Slamf1	0.00135728	0.569862	0.192	0.148	1
Fam122a	0.00137422	0.87806288	0.089	0.059	1
Zmym6	0.00137655	1.44209651	0.028	0.013	1

Kdm5a	0.00139686	0.52659846	0.351	0.317	1
Sptbn1	0.00140003	0.58153267	0.243	0.202	1
Slc19a1	0.00140429	1.36120145	0.019	0.008	1
Dcp1b	0.0014149	1.5664168	0.028	0.013	1
Hjurp	0.00141529	0.85995488	0.119	0.084	1
Arhgef3	0.00143561	0.54135835	0.213	0.172	1
L3mbtl3	0.00143897	1.03719802	0.054	0.031	1
Nars2	0.00144835	1.30348609	0.026	0.012	1
Mcm4	0.00145963	0.8737209	0.133	0.097	1
BC147527	0.00146342	2.33293947	0.01	0.003	1
Vamp5	0.00146619	1.08529956	0.047	0.026	1
Btbd11	0.00149015	1.20499722	0.033	0.016	1
E130102H24R	0.00150982	0.99092439	0.038	0.02	1
Trim8	0.00151531	1.03092305	0.099	0.069	1
Pkp3	0.00153715	0.85738286	0.12	0.087	1
Elf2	0.00154342	0.68957826	0.246	0.211	1
Cul7	0.00154512	2.36335942	0.014	0.005	1
Slc50a1	0.00155607	0.77559797	0.166	0.129	1
Ube2b	0.00155633	0.29510626	0.583	0.575	1
B3gntl1	0.00155962	2.30333475	0.014	0.005	1
Mterf1a	0.00156651	1.05661715	0.061	0.037	1
Ccdc51	0.00157648	1.79903392	0.012	0.004	1
Rtn4ip1	0.00166345	1.55660709	0.026	0.012	1
Mast3	0.00167264	0.94549244	0.086	0.057	1
Uri1	0.00167553	0.93613767	0.117	0.084	1
2410089E03R	0.00169392	0.90601524	0.084	0.055	1
Sept7	0.00170641	0.65416768	0.351	0.328	1
Dzip3	0.00171617	1.34406687	0.028	0.013	1
Txk	0.00172231	0.34911915	0.281	0.227	1
F830016B08Ri	0.00173541	2.66236402	0.01	0.003	1
Smim8	0.00179249	1.02398178	0.082	0.054	1
Slc45a4	0.00179779	1.22681277	0.059	0.036	1
Psme2b	0.00181253	1.16958128	0.056	0.033	1
E230032D23R	0.00181918	0.94236579	0.035	0.018	1
Ap1ar	0.00182735	0.86094983	0.094	0.064	1
Gm26853	0.00183508	0.84105637	0.098	0.067	1
Mcm6	0.00185645	0.60896949	0.131	0.095	1
Tceal3	0.00189969	1.90346487	0.01	0.003	1
9130221H12R	0.00190419	2.10966986	0.017	0.007	1
Slc12a2	0.00194265	1.47606876	0.044	0.024	1
Slc29a1	0.00194612	1.19294397	0.075	0.049	1
Scyl2	0.00195722	0.80820713	0.129	0.095	1
Uty	0.00196585	0.99954181	0.079	0.051	1
Mdc1	0.00198319	0.92944619	0.042	0.023	1
Tcaim	0.00201386	1.09297443	0.035	0.018	1
Aes	0.00202182	0.41677505	0.422	0.407	1
Hnrnpr	0.00202384	0.66259488	0.265	0.231	1
Sgsm2	0.00210828	1.81749898	0.023	0.01	1

Meis3	0.00213977	1.31690046	0.028	0.013	1
Ddr1	0.00217952	1.91862041	0.014	0.005	1
Rpap2	0.00221333	1.0364444	0.045	0.025	1
Prpf6	0.00225173	0.78588898	0.159	0.122	1
Stambpl1	0.00229141	1.35835673	0.04	0.022	1
Deaf1	0.00229403	1.27226318	0.03	0.014	1
Syne1	0.00229998	0.7618542	0.096	0.066	1
Ptcd3	0.00236077	1.00331465	0.077	0.05	1
Rnf114	0.00238152	0.55900735	0.23	0.192	1
Thoc7	0.00239427	0.64246634	0.237	0.202	1
Csnk1a1	0.00241458	0.25835655	0.572	0.575	1
Vps26b	0.00244242	1.10164206	0.068	0.043	1
Itgb3bp	0.00245561	0.90548743	0.045	0.025	1
Plcxd2	0.00246286	0.49671346	0.213	0.17	1
Nfatc2	0.00248959	0.85566543	0.106	0.075	1
Pdk1	0.00252215	0.88487081	0.073	0.047	1
Ppp1cb	0.00252934	0.50538868	0.36	0.332	1
Farsb	0.00253641	0.74712598	0.079	0.051	1
Naa20	0.00253906	0.88525597	0.138	0.104	1
Elp2	0.00255539	1.2087285	0.056	0.034	1
Ccdc91	0.00259762	1.38279742	0.023	0.01	1
Ppil1	0.00265141	0.97015343	0.065	0.04	1
Pigh	0.00265235	1.65323663	0.023	0.01	1
Zfp182	0.00266089	1.38427618	0.04	0.022	1
Senp6	0.00268169	0.43177647	0.386	0.359	1
Zdhhc21	0.00270598	0.89426451	0.134	0.101	1
Tmx4	0.00271	0.77089197	0.166	0.131	1
Lnpep	0.00271017	0.40099526	0.41	0.39	1
Cdc37l1	0.00279869	0.61351958	0.223	0.185	1
Gm42372	0.00280804	1.80090444	0.021	0.009	1
Pank4	0.00284723	1.10725571	0.044	0.024	1
Gigyf1	0.00284742	0.7458472	0.166	0.131	1
Cnot3	0.00284848	0.68385653	0.208	0.172	1
Zcchc11	0.0028756	0.3821248	0.571	0.556	1
Etfrf1	0.00289063	0.99942483	0.056	0.034	1
Ankrd10	0.00289951	0.94186446	0.096	0.066	1
Tcp11l2	0.00290819	0.61939542	0.255	0.22	1
Tars2	0.00290824	0.92121354	0.049	0.028	1
Rbbp9	0.00292626	1.70427408	0.014	0.005	1
Mfsd14a	0.00294269	0.88248812	0.152	0.119	1
Usp15	0.0029581	0.72349412	0.265	0.236	1
Ddx42	0.0029601	0.57127961	0.283	0.249	1
Tada1	0.00296434	1.12901421	0.072	0.047	1
Pard6g	0.00297266	1.08834723	0.017	0.007	1
Ccdc117	0.00299967	0.86197434	0.087	0.059	1
Smyd4	0.0030048	1.33738367	0.024	0.011	1
Fyttd1	0.00303508	0.72097706	0.166	0.131	1
Ptger2	0.00308761	1.06831292	0.045	0.026	1

Synpo	0.00310444	2.54372592	0.012	0.004	1
Ctps2	0.00313084	1.0017638	0.068	0.044	1
Eif3e	0.00317609	0.28487584	0.56	0.575	1
Ei24	0.0032026	1.12074532	0.087	0.06	1
Zbtb11	0.00321763	0.74687806	0.155	0.121	1
Txlng	0.00329388	1.01951337	0.073	0.048	1
Ttc38	0.00330769	1.57589425	0.024	0.011	1
Scai	0.00333009	0.80551634	0.066	0.042	1
Gm16740	0.00333523	1.87172989	0.017	0.007	1
Casp2	0.00333609	1.02388719	0.068	0.044	1
Mxra8	0.00334948	2.06675022	0.014	0.005	1
Mbtps1	0.0033557	0.74426226	0.168	0.133	1
Stx16	0.0033609	0.94139352	0.157	0.125	1
Mpzl3	0.00336825	1.04302641	0.044	0.025	1
Zfp869	0.00343116	1.21947792	0.033	0.017	1
Psma3	0.00345022	0.42502684	0.464	0.465	1
Tmcc1	0.00345035	0.95791267	0.131	0.099	1
E130308A19R	0.00352294	1.23399359	0.045	0.026	1
Vgll4	0.00353906	0.38193017	0.361	0.32	1
Cdk19	0.00354252	0.77898219	0.112	0.081	1
Gm45902	0.0035465	1.50738444	0.04	0.022	1
Mboat1	0.00355499	1.65239509	0.019	0.008	1
Wdr89	0.00357222	0.43377381	0.396	0.38	1
Cog5	0.0036409	0.88250771	0.087	0.06	1
Ankrd44	0.00364712	0.48944016	0.349	0.319	1
Uchl4	0.00366597	1.28644971	0.012	0.004	1
Nisch	0.00367152	0.53471152	0.274	0.244	1
Ulk1	0.00369118	0.77649822	0.136	0.104	1
Fbxl20	0.00374167	0.72732553	0.092	0.064	1
Dnah8	0.00383	1.38389751	0.019	0.008	1
Nt5c3b	0.00383154	1.08798897	0.056	0.034	1
Eif4enif1	0.00383832	0.84598146	0.091	0.063	1
Pdrg1	0.00385047	0.92958966	0.094	0.066	1
4930518I15Ril	0.00388994	1.96456763	0.016	0.006	1
Mapk1	0.00389564	0.60621377	0.264	0.233	1
Tpp2	0.00392751	0.53786926	0.305	0.275	1
Rfx7	0.00393935	0.61798508	0.133	0.1	1
Sgsh	0.00397765	1.35021217	0.03	0.015	1
Chchd10	0.00397902	0.6017951	0.133	0.099	1
Apex1	0.00402291	0.95368679	0.087	0.061	1
Larp4	0.00402704	0.75258331	0.25	0.219	1
March7	0.00403624	0.36131176	0.457	0.45	1
Ccnd3	0.00415305	0.75368587	0.14	0.107	1
Slain1	0.00418441	0.68847252	0.099	0.07	1
Rsf1	0.004218	0.63005025	0.239	0.203	1
Gm13012	0.00424282	1.45131893	0.024	0.012	1
Etnk1	0.00424743	0.50220624	0.291	0.258	1
Ncbp1	0.00426491	0.91854861	0.106	0.077	1

Mettl21a	0.00431058	1.69348611	0.019	0.008	1
Kif5c	0.00432195	1.35356462	0.019	0.008	1
Wdr12	0.00435991	1.09671515	0.058	0.036	1
Ankib1	0.00439111	0.92046221	0.127	0.097	1
Fdxr	0.00439231	1.26365723	0.045	0.026	1
Trim12a	0.00441484	0.59383165	0.169	0.133	1
Trim24	0.00451207	0.89854513	0.115	0.085	1
Ccdc66	0.00454103	1.0239212	0.04	0.023	1
Exoc6	0.00457487	1.01406536	0.072	0.047	1
Dtwd1	0.00462027	1.17013162	0.024	0.012	1
Ifit3b	0.00464469	1.57840051	0.012	0.004	1
Focad	0.00464728	1.31136064	0.023	0.011	1
Trim30a	0.00473375	0.62015499	0.19	0.157	1
Pitpnc1	0.00475256	0.55412734	0.206	0.168	1
Slc25a32	0.00476689	1.17316869	0.045	0.027	1
Phf11b	0.00478019	0.98640933	0.094	0.067	1
Syf2	0.0047932	0.45508129	0.421	0.412	1
Bmp5	0.00483147	1.36539833	0.016	0.006	1
Fam208a	0.0048539	0.74277969	0.166	0.133	1
Hadhb	0.0048776	0.54483867	0.211	0.178	1
Mark2	0.00488398	0.5040578	0.248	0.215	1
Retreg1	0.00489215	0.2850648	0.288	0.248	1
Pecam1	0.0049014	0.45025427	0.18	0.141	1
Sbf1	0.00492788	0.71205668	0.138	0.106	1
Hacd3	0.0049331	0.68443514	0.141	0.109	1
Gm28187	0.00501662	1.75967638	0.014	0.005	1
Csnk1g2	0.00501665	0.54137098	0.192	0.156	1
Dcaf5	0.00502985	0.81008658	0.07	0.046	1
Gm20275	0.0050386	2.08827094	0.01	0.003	1
Pura	0.00507169	0.54930444	0.211	0.175	1
Impact	0.00509536	0.70504183	0.182	0.149	1
Nlrc3	0.00510775	0.89582934	0.066	0.043	1
Klhl12	0.00516564	0.93648936	0.073	0.049	1
Bdh1	0.00520025	1.34662898	0.026	0.013	1
Rbm6	0.005227	0.76679931	0.202	0.171	1
Ppp1r12b	0.00523204	1.66105485	0.03	0.015	1
Fkbp5	0.00525994	1.07925683	0.052	0.032	1
Cdc73	0.00529931	0.5555221	0.197	0.162	1
Arhgap27os3	0.00534887	1.25228125	0.019	0.008	1
Fbxl12	0.00540026	0.58103657	0.154	0.119	1
Zfp949	0.0055185	0.98468655	0.044	0.026	1
Gdi1	0.00559857	0.58874039	0.145	0.112	1
Orc5	0.00562242	0.92894227	0.021	0.01	1
Tigit	0.00566693	1.13424768	0.042	0.024	1
2510002D24R	0.00571195	0.60645417	0.108	0.079	1
Phf21a	0.00574772	0.7411055	0.112	0.082	1
Tnfrsf23	0.00575217	0.85928927	0.051	0.031	1
Tmem86b	0.00575821	1.63891311	0.014	0.005	1

Isg20	0.00576014	0.69774155	0.086	0.059	1
Sox4	0.00578686	1.38504003	0.024	0.012	1
Zgpat	0.00579127	1.02901555	0.061	0.039	1
Zfp180	0.00581566	1.0788323	0.065	0.042	1
Pfas	0.00584608	1.19205076	0.024	0.012	1
Gm2682	0.00592659	0.88755719	0.045	0.027	1
Gbp9	0.0059331	0.91068943	0.084	0.058	1
Ccm2	0.00600617	0.66728929	0.161	0.127	1
Hltf	0.00601684	0.60732257	0.126	0.094	1
Wac	0.00602658	0.39873669	0.366	0.344	1
Cracr2a	0.00608126	1.39743423	0.024	0.012	1
Vps13c	0.00608295	1.01859365	0.103	0.076	1
Ip6k2	0.00621753	0.98266658	0.061	0.039	1
Map3k5	0.00623523	0.81812669	0.087	0.061	1
B930036N10R	0.00624691	0.84210946	0.119	0.089	1
Pbx4	0.00627442	1.37977644	0.016	0.006	1
Ighv1-9	0.00628408	1.81068709	0.014	0.005	1
Gpr22	0.00636438	1.42398114	0.012	0.004	1
Fkbp3	0.00639638	0.54900956	0.29	0.26	1
H2-T24	0.00644962	1.74139402	0.017	0.008	1
Acot2	0.00646575	0.76224888	0.058	0.036	1
Hipk2	0.00650201	0.94719128	0.133	0.104	1
Rhbdd2	0.00650525	1.27080621	0.035	0.019	1
Bphl	0.00651854	1.67329674	0.026	0.013	1
Lrrtm2	0.00661125	1.8506708	0.01	0.004	1
Rad1	0.00665829	1.71332201	0.03	0.016	1
Prdm15	0.00666935	1.14606623	0.024	0.012	1
Snw1	0.00667178	0.48289776	0.339	0.319	1
Mkln1	0.00678884	0.55901347	0.265	0.238	1
Set	0.006824	0.39194014	0.499	0.511	1
Hcfc2	0.00684049	0.78018688	0.051	0.031	1
Pdcd1	0.00690769	0.53029608	0.11	0.08	1
Ndrg2	0.00695347	2.3416181	0.012	0.005	1
Tia1	0.00696285	0.70952283	0.206	0.173	1
Scrn3	0.00700248	1.7886625	0.012	0.005	1
Pitpnm1	0.00709533	0.72097849	0.124	0.094	1
Ccdc88c	0.0071377	0.44087892	0.19	0.154	1
Arel1	0.00713867	1.03902559	0.068	0.046	1
Ppic	0.00714713	1.50364023	0.021	0.01	1
Gzmk	0.00715232	0.72737342	0.028	0.014	1
Kcna3	0.00721031	2.06078829	0.012	0.005	1
Pcm1	0.00728597	0.66729353	0.187	0.155	1
Scaper	0.00747364	0.97139631	0.07	0.047	1
Glmn	0.00749939	1.14179249	0.03	0.016	1
Zbtb44	0.00751944	0.73513012	0.134	0.103	1
Hus1	0.00760656	1.11461956	0.035	0.02	1
Hipk1	0.00763651	0.51642183	0.321	0.298	1
Akap9	0.00763662	0.60653133	0.22	0.189	1

Gtf2ird2	0.00765125	1.01031954	0.021	0.01	1
Gm26510	0.00766621	1.37824807	0.017	0.008	1
Ago1	0.00770798	1.05162756	0.063	0.041	1
Dock6	0.00778918	2.35386573	0.01	0.004	1
Elavl1	0.00780635	0.57861714	0.257	0.23	1
P2ry10	0.00780838	0.26193641	0.483	0.462	1
Gbp5	0.00792925	0.27018941	0.127	0.097	1
Anapc1	0.00797209	0.75388626	0.129	0.1	1
Bcl10	0.00802724	0.48231073	0.356	0.344	1
Slc14a1	0.00803373	1.67393909	0.026	0.013	1
Trpm7	0.00816608	0.71082859	0.223	0.195	1
Sertad2	0.00828089	0.79325609	0.159	0.129	1
Hnrnpc	0.00831513	0.28735553	0.497	0.5	1
Dtx3l	0.00842437	0.72030792	0.148	0.119	1
Slc44a2	0.00842992	0.71694406	0.108	0.08	1
Mfng	0.00843941	0.87039479	0.051	0.032	1
Dnajc9	0.00866898	0.71063249	0.147	0.115	1
Frmd8	0.00867308	1.05665535	0.051	0.032	1
Ankrd55	0.00871704	1.84112432	0.021	0.01	1
Uimc1	0.00878388	0.72467341	0.173	0.143	1
Ppp3cb	0.00884235	0.59522504	0.195	0.165	1
Cdc40	0.00884651	0.58334695	0.269	0.244	1
Mcat	0.00886257	1.79127572	0.014	0.006	1
Ccdc125	0.00890166	1.70468003	0.017	0.008	1
Jade1	0.00893307	0.77138653	0.086	0.061	1
Mgmt	0.00896062	2.33133105	0.019	0.009	1
Zfp410	0.00899097	0.89618494	0.086	0.061	1
Heg1	0.0090372	0.54328063	0.12	0.09	1
Cwf19l2	0.0090465	0.56850543	0.15	0.119	1
Galnt12	0.00912765	1.0009673	0.045	0.028	1
Rab3ip	0.00919977	1.05113589	0.063	0.042	1
Yy1	0.00926782	0.43890064	0.339	0.323	1
Dtnb	0.00933977	0.81372935	0.058	0.037	1
Ppm1f	0.00942054	1.26412954	0.017	0.008	1
Aplp1	0.00945526	1.54968196	0.021	0.01	1
Hdac4	0.00947306	0.90577892	0.096	0.07	1
Pbxip1	0.00948814	0.48682103	0.225	0.194	1
Tcea1	0.00994663	0.47718188	0.346	0.326	1
Eid2	0.00997954	1.60414386	0.016	0.007	1
Pofut1	0.01020588	1.18091489	0.049	0.031	1
Gm45606	0.01026684	2.19553676	0.012	0.005	1
Zfp507	0.01027313	0.6415474	0.072	0.049	1
Max	0.01028897	0.65987329	0.175	0.145	1
Plekha7	0.01051314	1.6192305	0.01	0.004	1
Usp40	0.0106101	1.08981057	0.042	0.025	1
Atad2b	0.01061111	0.69603435	0.148	0.119	1
Platr17	0.01062907	2.06629758	0.023	0.011	1
Cep162	0.01063123	1.53618831	0.042	0.025	1

Zfp148	0.01069372	0.61530011	0.204	0.175	1
Med17	0.01079621	1.04458629	0.072	0.05	1
Zfp7	0.01080895	1.2631071	0.016	0.007	1
Ezh1	0.01086286	0.76833907	0.115	0.088	1
Rai1	0.01096075	0.84281675	0.08	0.057	1
AU040320	0.01098015	1.22718155	0.049	0.031	1
Sp4	0.01118787	0.66205034	0.157	0.126	1
Xkr6	0.01126658	1.16057817	0.019	0.009	1
Casp3	0.0112773	0.81008626	0.138	0.11	1
Pkn2	0.01131438	0.45040768	0.33	0.313	1
Zdhhc2	0.01150704	0.73753587	0.063	0.042	1
Cd96	0.01151359	0.81719247	0.098	0.072	1
Pik3ca	0.01155644	0.83838135	0.129	0.102	1
Mtfmt	0.01172782	1.26766834	0.038	0.023	1
Gm26765	0.01177809	1.43591994	0.012	0.005	1
Irf2	0.01187771	0.46989733	0.291	0.262	1
Hace1	0.0119024	0.85649668	0.054	0.035	1
Polr2g	0.01203206	0.79540104	0.15	0.123	1
Atpaf1	0.01209128	1.05672009	0.026	0.014	1
2610035D17R	0.01211584	1.56447433	0.012	0.005	1
Slfn5	0.01220363	0.33468858	0.112	0.084	1
Gm37065	0.01221733	2.27514532	0.012	0.005	1
4933434E20R	0.01225788	0.6111531	0.178	0.15	1
Top1mt	0.0122733	2.13112013	0.012	0.005	1
Srek1ip1	0.01227544	1.04754917	0.07	0.049	1
Tha1	0.01228	1.99187666	0.014	0.006	1
Snrpf	0.0124198	0.25484797	0.543	0.561	1
Stx4a	0.0125029	0.69315044	0.187	0.159	1
Erich1	0.01251802	0.8474366	0.079	0.056	1
Dcun1d1	0.0126452	0.55557552	0.143	0.114	1
Alkbh1	0.01276003	0.53734606	0.248	0.218	1
Donson	0.01287984	0.76798271	0.108	0.082	1
Cstf2	0.01306908	0.84932552	0.073	0.051	1
Znrf3	0.01312179	0.94647598	0.033	0.019	1
Ccl25	0.01323513	1.30913023	0.044	0.027	1
Polg	0.01331229	0.94256861	0.073	0.051	1
Tmem191c	0.01343105	1.10391575	0.037	0.022	1
Wdr92	0.01348135	1.43082237	0.024	0.013	1
Mier2	0.01350518	1.35695605	0.024	0.013	1
Gm26625	0.01352856	1.29543213	0.014	0.006	1
Chordc1	0.01355109	0.71425986	0.143	0.115	1
St6gal1	0.0136032	0.8027988	0.089	0.065	1
Sacs	0.01364356	0.79476551	0.084	0.06	1
Lrrc45	0.01368229	1.33211296	0.028	0.015	1
Lims1	0.01369959	0.69503897	0.197	0.172	1
Zdhhc20	0.01372027	0.60808288	0.215	0.187	1
Zbtb40	0.01382685	1.33928532	0.028	0.015	1
Rhot2	0.01396795	1.06417087	0.04	0.024	1

Ogt	0.01417685	0.41948281	0.34	0.317	1
Thumpd3	0.01432881	0.86588093	0.08	0.058	1
Terf2	0.01435318	0.69075621	0.086	0.062	1
Zfp764	0.01438583	1.69434067	0.016	0.007	1
Ncoa2	0.01440955	0.58498747	0.216	0.188	1
Acsf3	0.01451428	1.582035	0.016	0.007	1
Zfp61	0.01451479	1.99961513	0.012	0.005	1
Zfp677	0.01502072	1.15541774	0.028	0.015	1
Usp53	0.01508209	0.89360657	0.058	0.039	1
S100pbp	0.01509004	1.12507201	0.075	0.054	1
Lrrcc1	0.01520901	1.05309991	0.047	0.03	1
Idnk	0.01522733	0.56466161	0.204	0.178	1
Wasl	0.01534633	0.87232842	0.117	0.092	1
Zscan20	0.0154769	1.67176719	0.012	0.005	1
Acad9	0.01548059	1.45468062	0.031	0.018	1
Grk6	0.01550628	0.65838163	0.164	0.134	1
Hif1an	0.01554593	0.7783269	0.122	0.097	1
Ccser2	0.0155481	0.56603289	0.148	0.121	1
Zfp146	0.01563281	0.71758131	0.065	0.044	1
Bbx	0.01580072	0.61711563	0.18	0.151	1
Rab3gap1	0.01595624	0.56320032	0.199	0.17	1
Zfp346	0.01603358	1.18470822	0.023	0.012	1
Patz1	0.01614311	1.00359088	0.038	0.023	1
Dcun1d4	0.0161948	1.20445017	0.037	0.022	1
Atp1b1	0.01621209	0.78310215	0.044	0.027	1
Mrps31	0.01650034	1.14961186	0.056	0.038	1
C2cd3	0.0165352	0.8597616	0.072	0.051	1
Slc16a1	0.01655346	0.83606108	0.073	0.052	1
Tsnax	0.01655394	0.79048585	0.068	0.048	1
Sf3b3	0.01656683	0.32235917	0.351	0.327	1
Zfp874b	0.01679333	0.85784768	0.054	0.036	1
Pex3	0.01680898	0.8785996	0.07	0.049	1
Zkscan8	0.01690384	1.24182983	0.023	0.012	1
Ino80d	0.01697255	0.49861798	0.25	0.224	1
Kmt2e	0.01705011	0.26884139	0.435	0.42	1
Cdca7	0.01705557	1.20265349	0.026	0.014	1
1810041H14R	0.01707617	1.53356799	0.026	0.014	1
Parp10	0.01723773	0.6372589	0.051	0.033	1
Jarid2	0.01729717	0.38496226	0.346	0.326	1
Dcaf8	0.01751018	0.57349381	0.216	0.191	1
Rnf213	0.01761421	0.74351418	0.154	0.127	1
Adam19	0.01765294	0.95081327	0.112	0.088	1
Fbxo32	0.01771209	0.78410368	0.07	0.049	1
Jak3	0.01774907	0.73402574	0.079	0.057	1
Cacna1a	0.01780033	1.33231202	0.014	0.006	1
Tdrp	0.01788982	1.00934709	0.021	0.011	1
Lnpk	0.01790601	0.69639642	0.11	0.084	1
Ide	0.01796188	0.76222733	0.098	0.074	1

Zfp938	0.01797454	1.05311025	0.038	0.024	1
Igtp	0.01802317	0.58218072	0.155	0.127	1
Champ1	0.01803854	0.7943175	0.054	0.036	1
Rnf125	0.01807799	0.40370702	0.241	0.207	1
Vcpip1	0.01833003	0.59426816	0.164	0.137	1
Znrf2	0.01833997	0.62073235	0.225	0.204	1
Trim23	0.01852274	0.9426695	0.042	0.026	1
Ctse	0.01854753	1.36891203	0.024	0.013	1
Rbm48	0.01862094	0.71732163	0.082	0.06	1
Trim12c	0.01871392	0.59316778	0.127	0.1	1
Carns1	0.01879819	1.08335743	0.017	0.008	1
Tspyl1	0.01889299	0.67091133	0.152	0.125	1
Etaa1	0.0189673	0.72392245	0.084	0.061	1
Zmat3	0.01898238	1.08596175	0.028	0.016	1
Cd99l2	0.01905095	1.25600995	0.028	0.016	1
Ncapd3	0.01907465	0.86517278	0.079	0.057	1
Slc37a1	0.01918192	1.42430227	0.023	0.012	1
Gabpb2	0.01920353	0.65247297	0.148	0.123	1
Fut8	0.01926496	1.00650499	0.07	0.05	1
Ciz1	0.01945889	0.74461656	0.068	0.048	1
Phf12	0.01972495	0.76947842	0.091	0.068	1
Snx19	0.01980475	0.87203762	0.04	0.025	1
Nit1	0.01996087	1.0375686	0.072	0.052	1
Dpm1	0.02002239	0.70258301	0.134	0.109	1
Sco1	0.02043692	1.03945832	0.033	0.02	1
Tmem201	0.02049281	1.51530718	0.023	0.012	1
Otud7b	0.02049603	0.81070352	0.065	0.045	1
Prkd2	0.02056213	0.71215221	0.056	0.038	1
Nae1	0.02057959	0.73568641	0.124	0.099	1
Decr1	0.02072422	1.26533731	0.035	0.021	1
Cul4b	0.02081609	0.95900188	0.068	0.049	1
Rtf1	0.02093352	0.33313613	0.347	0.332	1
Tapt1	0.02094504	0.82481265	0.072	0.051	1
Wisp1	0.02101826	1.04837645	0.012	0.005	1
Cep95	0.02105426	1.30801068	0.019	0.01	1
Nfx1	0.02125901	0.76536915	0.143	0.118	1
Tceal9	0.02136469	0.72900063	0.213	0.189	1
Sirt4	0.02148119	1.47180199	0.012	0.005	1
Gcc1	0.02151433	1.13455848	0.059	0.041	1
Cnnm3	0.02160707	0.90612415	0.049	0.032	1
2410002F23Ri	0.02161031	0.73074068	0.092	0.07	1
Arl6ip1	0.02190785	0.32164693	0.515	0.53	1
Whrn	0.02205357	1.1528846	0.024	0.013	1
Pan3	0.02207394	0.44132877	0.283	0.259	1
Hsdl1	0.02223014	0.94020307	0.063	0.044	1
Foxo3	0.02229384	0.84712274	0.079	0.058	1
Smc1a	0.02263195	0.52250196	0.262	0.243	1
Slc35d1	0.02282638	1.32697733	0.059	0.042	1

Zfp451	0.02288975	0.9651653	0.14	0.116	1
Mbd6	0.02289149	0.84973264	0.077	0.056	1
Gm42670	0.02294693	1.07841429	0.03	0.017	1
Tomm34	0.02319188	0.78378473	0.12	0.097	1
Safb	0.02325192	0.64691415	0.22	0.199	1
Ppwd1	0.02353697	0.54440043	0.188	0.161	1
Paip2	0.02354271	0.29106505	0.431	0.428	1
Ddx3y	0.02384353	0.43809616	0.23	0.201	1
Slc28a2	0.02407712	0.8333561	0.065	0.046	1
Commd8	0.02434191	0.77036521	0.147	0.123	1
Cbfa2t2	0.0243616	1.11620687	0.038	0.024	1
Ndnf	0.02449581	2.101967	0.016	0.008	1
4930481A15R	0.02459443	0.56794558	0.023	0.012	1
Akap10	0.02481865	0.77088576	0.091	0.069	1
Lrmp	0.02482515	0.62774614	0.115	0.091	1
Prr12	0.02490418	1.09768476	0.026	0.015	1
Cenpo	0.02512326	1.2172268	0.019	0.01	1
Lmtk2	0.02523643	0.94089083	0.07	0.051	1
Rad9b	0.02558361	1.74131544	0.012	0.005	1
Qprt	0.02565971	1.34555433	0.01	0.004	1
Dmtf1	0.02574005	0.5701767	0.148	0.123	1
Fnbp4	0.02578135	0.57736725	0.154	0.129	1
Rnmt	0.02582661	0.58989214	0.141	0.116	1
Arfgef1	0.02599301	0.529048	0.237	0.212	1
Clec2d	0.02610613	0.29321693	0.375	0.363	1
Ptbp2	0.02640639	0.75850426	0.157	0.133	1
Ralgapa2	0.02653282	0.81255785	0.061	0.043	1
Usp3	0.02677251	0.55515102	0.208	0.184	1
Pikfyve	0.02690538	0.76787693	0.086	0.064	1
Trbv12-2	0.02703142	1.19757782	0.014	0.006	1
Nipa2	0.02709498	0.26232782	0.171	0.228	1
Setd2	0.02712754	0.52750872	0.25	0.227	1
Sdcbp2	0.02726174	0.78559707	0.058	0.04	1
Casd1	0.02726322	1.01773367	0.049	0.033	1
Rnf123	0.02737182	0.79221619	0.058	0.04	1
Pmm2	0.02742541	0.96353109	0.094	0.073	1
Uvssa	0.02745727	0.9811017	0.044	0.029	1
Elp4	0.02751412	1.55498658	0.021	0.011	1
C1qtnf4	0.02766704	0.98135022	0.017	0.009	1
Asb2	0.02778301	0.8193745	0.068	0.049	1
Mpv17l	0.0279659	0.98041293	0.042	0.027	1
Herc4	0.02798515	0.55114993	0.169	0.143	1
1110002L01Ri	0.02801683	1.61341018	0.012	0.005	1
Ercc5	0.028227	0.84989807	0.061	0.043	1
Slain2	0.02835859	0.64485936	0.159	0.135	1
Herc3	0.02849062	0.63244122	0.066	0.047	1
Ddx11	0.02858802	1.10480201	0.021	0.011	1
Snupn	0.02869119	1.22187614	0.03	0.018	1

Tank	0.02874887	0.6180742	0.298	0.283	1
Usp9x	0.02886257	0.45673085	0.298	0.283	1
Cbl	0.02905002	0.61043148	0.271	0.258	1
Wipf1	0.02968745	0.46595794	0.3	0.285	1
Helb	0.03007491	0.61208945	0.079	0.058	1
Rab11fip3	0.03028673	1.54540888	0.01	0.004	1
Exosc1	0.03049905	0.86533651	0.059	0.042	1
Hp1bp3	0.03059505	0.4874362	0.283	0.266	1
Rab27a	0.03072576	0.97356627	0.066	0.048	1
Gstt2	0.0307638	0.83921348	0.058	0.04	1
Mrps35	0.03080696	0.85085864	0.075	0.056	1
Ndufa5	0.03112528	0.5776294	0.239	0.221	1
Zfp444	0.03113687	1.01928257	0.028	0.016	1
Kmt2d	0.03129521	0.61153272	0.145	0.121	1
Gin1	0.0313202	1.08163299	0.03	0.018	1
Oaz2	0.0313559	1.14564631	0.052	0.036	1
AI987944	0.03141103	1.10466198	0.049	0.033	1
Ppp1r15a	0.03162126	0.32786614	0.414	0.411	1
Dap3	0.0316908	0.67438524	0.115	0.093	1
D130040H23F	0.03174687	1.31995497	0.019	0.01	1
Ss18	0.03217933	0.61634396	0.19	0.167	1
Plpp3	0.03257994	1.33407917	0.026	0.015	1
A430033K04R	0.03263782	1.22308351	0.014	0.007	1
Ddhd2	0.03283973	0.83395784	0.098	0.076	1
A330035P11R	0.03286154	1.6434687	0.01	0.004	1
Hsh2d	0.03301202	1.16172668	0.017	0.009	1
Hspa4l	0.03337943	0.79094243	0.091	0.07	1
Mga	0.03340141	0.46662289	0.18	0.156	1
Gmcl1	0.03347004	0.67789737	0.061	0.043	1
Cdk13	0.03347479	0.68406005	0.171	0.148	1
Dusp14	0.03359241	0.96176431	0.024	0.014	1
Urb2	0.03377903	1.24976507	0.024	0.014	1
Cc2d1a	0.03407396	1.3999425	0.026	0.015	1
Polk	0.03412676	0.69856173	0.042	0.027	1
Carm1	0.03435624	1.19491339	0.042	0.028	1
Rgs16	0.03455797	1.0524166	0.03	0.018	1
Cep120	0.03468517	0.61734643	0.134	0.111	1
Zfp748	0.03500772	0.90507461	0.038	0.025	1
9930104L06Ri	0.03535973	1.5699184	0.016	0.008	1
Klhl36	0.0355034	1.1773669	0.021	0.012	1
Tmem106b	0.03559782	0.80037288	0.098	0.077	1
6720427I07Ril	0.03592782	0.67735079	0.049	0.033	1
Tox4	0.03608659	0.47789911	0.279	0.266	1
Tatdn1	0.03627716	1.17123112	0.051	0.035	1
Rbms2	0.0366292	0.92847436	0.059	0.043	1
Ints7	0.03671484	0.72848632	0.077	0.058	1
Hlcs	0.03700035	1.02072334	0.047	0.032	1
Setd3	0.03712349	0.74135847	0.162	0.141	1

Pdpr	0.03739814	0.87087887	0.051	0.035	1
Ccdc47	0.03748985	0.76620133	0.122	0.101	1
Kidins220	0.03761255	0.72191183	0.12	0.099	1
2900093K20R	0.0378386	0.75040556	0.054	0.038	1
Abcf3	0.03786208	0.87312106	0.07	0.052	1
2610002M06F	0.03843515	0.81813311	0.045	0.031	1
Atxn7l1	0.03849765	0.93945171	0.115	0.095	1
Zfp467	0.03895416	1.34622971	0.035	0.022	1
Trp53bp1	0.03898435	0.66925353	0.068	0.05	1
Calcoco1	0.03905027	0.8608801	0.045	0.031	1
Selenoo	0.03994555	0.96969746	0.038	0.025	1
Pip4k2a	0.03999076	0.73232471	0.222	0.206	1
Pik3cd	0.04009388	0.51728976	0.173	0.148	1
Fpgt	0.040219	1.24577446	0.042	0.028	1
Lrig2	0.0402497	0.88274246	0.045	0.031	1
Rfxank	0.04042587	1.14418261	0.031	0.02	1
Tsr2	0.04045139	1.02923194	0.045	0.031	1
Pex11a	0.04081475	1.47841498	0.01	0.005	1
Gm4707	0.041485	0.98901799	0.056	0.04	1
Zfp329	0.04153153	0.76346379	0.07	0.052	1
3830406C13R	0.04198643	0.83054753	0.108	0.088	1
Ppp1r3f	0.04215007	1.36793473	0.014	0.007	1
Atp11a	0.04257829	1.1527818	0.049	0.034	1
Ick	0.04271306	1.33823356	0.017	0.009	1
Zbtb38	0.04284975	0.66812889	0.105	0.084	1
Akap12	0.04297458	0.31950991	0.028	0.017	1
Ngly1	0.04369933	0.68571898	0.073	0.055	1
Zfp212	0.04376877	0.82496336	0.056	0.04	1
4930522L14Ri	0.04389385	1.04118789	0.03	0.018	1
Rmnd5a	0.0439333	0.6650714	0.15	0.128	1
5430403G16R	0.04395539	0.97818675	0.016	0.008	1
Fopnl	0.04442787	0.86059415	0.099	0.079	1
Atp6v0a2	0.04455822	0.7382459	0.084	0.065	1
Casp4	0.04462772	0.64613873	0.246	0.233	1
Usp45	0.04467154	0.96768961	0.065	0.048	1
Ipo8	0.04492647	0.7944977	0.08	0.062	1
Zfp266	0.04496032	0.58054354	0.112	0.09	1
Zfp775	0.04545991	1.35436689	0.014	0.007	1
Virma	0.04546408	0.49210903	0.141	0.119	1
Kif16b	0.04576547	1.06056851	0.049	0.034	1
Foxj3	0.04616225	0.58635198	0.117	0.096	1
Tmem263	0.04618663	0.6832431	0.065	0.047	1
Trim65	0.04627371	1.1202084	0.021	0.012	1
Arid2	0.04637018	0.87847007	0.073	0.055	1
C87436	0.04642019	0.82714996	0.073	0.056	1
Cradd	0.04648388	1.29126926	0.028	0.017	1
Ppargc1b	0.04650745	1.14004693	0.016	0.008	1
Hectd1	0.0465281	0.40596172	0.328	0.316	1

Las1l	0.04661821	0.62996244	0.133	0.111	1
Zfp607a	0.04673615	1.25248092	0.026	0.016	1
Maml2	0.04693813	0.79840314	0.052	0.037	1
Baz1b	0.04697201	0.52545328	0.209	0.186	1
Slc35d2	0.04705882	1.22768078	0.021	0.012	1
Tarbp2	0.04714855	0.78535103	0.059	0.043	1
Il6ra	0.04726871	1.12340607	0.11	0.091	1
Acvr1b	0.04760231	1.36908696	0.023	0.013	1
Sidt1	0.04810916	0.53507806	0.042	0.028	1
Kat6a	0.04811407	0.41510609	0.232	0.21	1
Cyp4f13	0.04855676	1.13998339	0.035	0.023	1
Wdr44	0.04894071	0.6896646	0.08	0.061	1
Zfp956	0.04915824	1.02416843	0.021	0.012	1
Tfdp2	0.04925427	0.47729683	0.049	0.034	1
Zfp609	0.04945174	1.2667816	0.035	0.023	1
Elmo1	0.0494762	0.49941579	0.307	0.298	1
Cep41	0.04951729	0.94649224	0.023	0.013	1
Bbs2	0.04961393	1.1026895	0.012	0.006	1
Cic	0.0496292	0.67224747	0.12	0.1	1
Dnmt3a	0.0496428	0.8803199	0.091	0.072	1
Klhl22	0.04976423	1.41014023	0.014	0.007	1
Ptch1	0.04982265	1.19818684	0.04	0.027	1
Nup210	0.04985797	0.52959997	0.124	0.102	1
Gpr18	0.04989037	0.66603997	0.112	0.091	1
Snrk	0.04993796	0.77076958	0.099	0.079	1
Zfp395	0.05033325	0.92227365	0.028	0.017	1
Fryl	0.05063501	0.28908854	0.25	0.223	1
Dnm2	0.05069654	0.47769757	0.206	0.187	1
Rin2	0.05076939	0.31597482	0.033	0.053	1
Sprtn	0.05125908	0.72590075	0.054	0.039	1
Mrps5	0.05168911	0.74024413	0.105	0.085	1
Zfp446	0.05170618	1.35623795	0.012	0.006	1
Dhx9	0.05190202	0.44449839	0.274	0.258	1
Hist1h4a	0.05242158	1.46786577	0.017	0.01	1
Gcfc2	0.05242587	1.1548917	0.021	0.012	1
Coro1b	0.05275928	0.36296605	0.141	0.186	1
Sgpp1	0.05306912	0.71679451	0.073	0.056	1
Trim5	0.05319432	0.81728833	0.033	0.021	1
2310035C23R	0.05324822	0.54351861	0.154	0.132	1
Cars2	0.05349959	0.75615845	0.056	0.041	1
Wdr76	0.05353644	0.89520054	0.03	0.019	1
Armcx3	0.05376237	1.01919983	0.066	0.05	1
Limk2	0.05422374	0.78177854	0.035	0.023	1
Setd1b	0.05439486	0.48180624	0.094	0.074	1
Ccnj	0.05443823	0.97665253	0.01	0.005	1
Gemin2	0.05476493	1.14727923	0.033	0.022	1
Slc35g1	0.05500055	0.80738177	0.019	0.011	1
Izumo4	0.05530582	0.90241178	0.03	0.019	1

Tgif2	0.05650355	0.50917876	0.054	0.039	1
Hmgn1	0.05673961	0.41602028	0.229	0.211	1
Slc52a2	0.05677683	0.82042006	0.033	0.022	1
Fto	0.05693198	0.93041521	0.08	0.063	1
Pcnp	0.0569784	0.53103822	0.257	0.245	1
Miga1	0.05736394	1.05597523	0.026	0.016	1
Gm31597	0.05748463	0.78057186	0.033	0.022	1
Zbp1	0.05806811	0.505378	0.145	0.125	1
Ppp2r3a	0.05837444	1.30888244	0.04	0.028	1
Mtmr3	0.05838567	0.61632298	0.131	0.11	1
Nsmaf	0.0583956	0.57099051	0.143	0.123	1
Bag4	0.05846854	0.79526231	0.065	0.048	1
Dmac2	0.05889713	0.60616634	0.094	0.075	1
Nck1	0.05894253	0.49915862	0.171	0.149	1
Ttc5	0.05919123	0.65063881	0.086	0.067	1
Fra10ac1	0.05919428	0.95519899	0.028	0.018	1
Mcoln1	0.0592441	1.04201061	0.038	0.026	1
Dph7	0.05931459	1.5412626	0.021	0.012	1
Cdca7l	0.05955542	1.00772157	0.031	0.02	1
Zfp605	0.05963995	0.96675612	0.014	0.007	1
Ly96	0.06011684	0.26981701	0.026	0.043	1
Zkscan3	0.06026915	0.83994634	0.084	0.067	1
Zbtb11os1	0.06028638	1.34460349	0.024	0.015	1
1700052K11R	0.06028749	0.85059718	0.017	0.01	1
Rbm26	0.06066223	0.53391057	0.211	0.194	1
Tmem94	0.06077763	1.0410906	0.031	0.02	1
Wdr45	0.06127374	0.80928053	0.056	0.041	1
Arid4b	0.06146984	0.25563057	0.332	0.319	1
Commd3	0.0617919	0.81490486	0.122	0.104	1
1600014C10R	0.06203333	0.83665265	0.051	0.036	1
Amer1	0.06212744	0.98652251	0.014	0.007	1
Hs1bp3	0.062645	1.76406686	0.012	0.006	1
Nudt1	0.06265543	1.09352552	0.021	0.012	1
Zfp712	0.06270369	1.55182597	0.01	0.005	1
Glrx2	0.06292983	0.63354933	0.143	0.124	1
Ilf3	0.06307183	0.43121671	0.175	0.153	1
Gm3448	0.06308646	0.83394034	0.052	0.038	1
Cdc14b	0.06332208	1.37611041	0.024	0.015	1
Timm44	0.06365764	0.7913212	0.117	0.098	1
Adcy3	0.06377257	1.30448133	0.023	0.014	1
Mtif2	0.06432491	0.82461547	0.049	0.035	1
Ncbp3	0.06448723	0.72186891	0.115	0.096	1
Hyls1	0.06452769	0.82977615	0.023	0.014	1
Nudcd3	0.06471449	0.64039956	0.197	0.181	1
Tmem62	0.06494923	1.38093151	0.023	0.014	1
Mecp2	0.06509222	0.61395486	0.148	0.129	1
Tmem245	0.06542075	0.60653151	0.087	0.069	1
Ip6k1	0.0663136	0.43600618	0.187	0.166	1

Ube2g1	0.06642493	0.72143301	0.098	0.08	1
Abce1	0.06651886	0.49355883	0.171	0.151	1
Akap8l	0.06675673	0.74810998	0.113	0.095	1
Xpo1	0.06682182	0.56889782	0.124	0.104	1
Senp1	0.06697653	0.61429641	0.096	0.077	1
Pbx2	0.06713519	0.76436608	0.054	0.04	1
Mpzl2	0.06744897	2.30674373	0.01	0.005	1
Hnrnpm	0.06747461	0.30023534	0.419	0.432	1
Cntrl	0.06749887	0.4851653	0.183	0.163	1
Ppp4r3b	0.06774123	0.46910311	0.232	0.217	1
Ifit1bl1	0.06787036	1.4602223	0.014	0.007	1
Micu2	0.06792283	0.77816781	0.098	0.08	1
Sfmbt1	0.06794808	0.470977	0.052	0.075	1
Acbd5	0.06809955	0.34769956	0.188	0.166	1
Lsm14b	0.06843173	0.94536338	0.045	0.032	1
Lypla2	0.06854612	0.71001698	0.138	0.12	1
Osbpl2	0.06854879	0.71587908	0.051	0.037	1
Cbx8	0.06860475	0.863043	0.021	0.012	1
Cep76	0.06863434	0.99530161	0.023	0.014	1
Dyrk2	0.06896543	0.51041374	0.173	0.153	1
Snhg12	0.06898548	0.3362083	0.305	0.288	1
Lrp6	0.06900856	0.5286676	0.099	0.081	1
Klhl26	0.0690337	1.19820038	0.012	0.006	1
Rock1	0.06924332	0.26347614	0.398	0.399	1
Dck	0.069686	0.76478075	0.086	0.069	1
Arhgap35	0.06983529	1.43333978	0.019	0.011	1
Ttll3	0.0705637	1.25243034	0.019	0.011	1
Gnpnat1	0.07065075	0.82330572	0.059	0.045	1
Cd1d1	0.07077352	1.48456599	0.035	0.024	1
Cirbp	0.07084736	0.30813247	0.316	0.302	1
Gtpbp3	0.07095957	0.86649768	0.019	0.011	1
Fam120b	0.07119817	0.84049002	0.073	0.057	1
Abcb7	0.0716315	0.76039281	0.065	0.049	1
Hdac10	0.07197238	0.97982537	0.014	0.007	1
Eid1	0.07204061	0.58954119	0.209	0.196	1
Sirt5	0.0721595	1.07486816	0.012	0.006	1
Ppcdc	0.07242907	0.68248148	0.04	0.028	1
Mob2	0.07268902	0.49875829	0.14	0.12	1
Egr2	0.07269911	0.27009773	0.021	0.035	1
8030462N17R	0.07276994	0.92425766	0.035	0.024	1
Adnp	0.0727834	0.93796127	0.045	0.033	1
Agk	0.07293803	0.62410648	0.049	0.035	1
Dhx34	0.073041	1.09097729	0.031	0.021	1
Ppp2r5e	0.07313536	0.58283042	0.113	0.095	1
Cntrob	0.0731575	0.95247141	0.026	0.017	1
Fkbp1a	0.07323102	0.4924064	0.291	0.292	1
Oas3	0.07342297	0.68136699	0.037	0.025	1
Zfp213	0.07374716	1.44159077	0.012	0.006	1

Irf9	0.07401878	0.47431632	0.162	0.143	1
Xpc	0.07405696	0.7794454	0.031	0.021	1
Zfp142	0.0741138	0.74473533	0.058	0.043	1
Srsf10	0.07430519	0.44561024	0.237	0.222	1
Ubqln1	0.07431628	0.57540904	0.175	0.158	1
Nanos1	0.07473314	1.56494167	0.012	0.006	1
Btbd19	0.07487063	0.98231114	0.021	0.013	1
Tpcn1	0.07488172	0.44996888	0.019	0.033	1
Zfp160	0.07495089	0.95826366	0.047	0.034	1
Cdc23	0.07545394	0.81988373	0.04	0.028	1
Dgkq	0.07558886	1.04826387	0.026	0.017	1
Clip1	0.07565513	0.52233664	0.166	0.147	1
Rab19	0.07569853	0.92720003	0.047	0.034	1
Sdhc	0.07579036	0.29100002	0.082	0.11	1
Taf9b	0.07591234	1.06556647	0.026	0.017	1
C330018D20F	0.07627132	1.51574462	0.012	0.006	1
Tyw1	0.07711767	0.86689595	0.03	0.019	1
Paqr7	0.07717482	1.60731201	0.014	0.008	1
Ppm1a	0.07731956	0.36887844	0.204	0.185	1
Pigb	0.07751417	1.02231993	0.016	0.009	1
Zw10	0.07858395	0.92099301	0.04	0.028	1
Tmsb15b1	0.0787337	0.99003411	0.031	0.021	1
Fam222b	0.07882132	1.03798744	0.033	0.022	1
Slc10a3	0.07917842	0.99901174	0.035	0.024	1
Arf3	0.07917961	0.75833906	0.155	0.139	1
Ammecr1	0.07943142	1.02084679	0.023	0.014	1
Dnajc8	0.07945115	0.32937016	0.401	0.404	1
Pim2	0.07953016	0.28431729	0.082	0.064	1
Dynll2	0.08011499	0.65463582	0.148	0.131	1
Wdr59	0.08020145	0.92416205	0.021	0.013	1
Cuedc1	0.08094222	0.75754549	0.021	0.013	1
Ubap2l	0.08100194	0.36207235	0.344	0.346	1
Rlf	0.08129033	0.44455046	0.25	0.236	1
Aifm1	0.08129983	0.82008825	0.051	0.037	1
Zbtb49	0.08144568	1.52488787	0.021	0.013	1
Rfesd	0.08168055	0.92722378	0.028	0.018	1
Nup155	0.0818126	0.82755702	0.042	0.03	1
Parp6	0.08190471	0.65934281	0.042	0.03	1
Wapl	0.08191929	0.30148801	0.351	0.348	1
Mettl2	0.08222201	0.78310523	0.035	0.024	1
Tspyl4	0.08242141	0.9151321	0.021	0.013	1
Sit1	0.08248933	0.82129861	0.037	0.025	1
Gm17435	0.08281176	1.93353653	0.012	0.006	1
Rreb1	0.0832382	0.2744253	0.031	0.048	1
Actr6	0.08329155	0.90769927	0.031	0.021	1
Zfp738	0.08330415	0.71932143	0.045	0.033	1
B4galnt4	0.08334937	0.86261451	0.021	0.013	1
Ccni	0.08361823	0.42157232	0.262	0.249	1

Mrpl3	0.08364283	0.25573768	0.065	0.088	1
Ascc3	0.08415444	0.5024106	0.236	0.221	1
1300002E11R	0.08444414	0.69939477	0.052	0.039	1
Ythdf3	0.08447588	0.51977548	0.234	0.221	1
Nipsnap2	0.08448899	0.82306436	0.051	0.038	1
Sipa1	0.08451949	0.35809313	0.11	0.09	1
Top2b	0.08453858	0.42264981	0.311	0.301	1
Armc3	0.0848229	1.50720709	0.012	0.006	1
Zfp330	0.08487686	0.68239201	0.147	0.131	1
Zfp740	0.08497612	0.74939316	0.091	0.074	1
Cdkn2c	0.08514863	0.82467772	0.028	0.018	1
Wipi2	0.08563286	0.40453791	0.129	0.11	1
Zfp369	0.08565611	0.87707378	0.037	0.025	1
Arhgef18	0.0859773	0.60992445	0.052	0.039	1
Fubp1	0.08635697	0.36980832	0.346	0.344	1
Tmpo	0.08659253	0.42328482	0.19	0.172	1
Enpp1	0.08699076	0.93432443	0.042	0.03	1
Ccdc61	0.08739305	1.14532886	0.012	0.006	1
Mttp	0.08783468	1.15739289	0.017	0.01	1
Pigl	0.08836166	0.95506587	0.016	0.009	1
Mtx2	0.08851695	1.08474738	0.052	0.039	1
Ino80dos	0.08857031	1.66763858	0.01	0.005	1
Rbbp4	0.08863618	0.45349533	0.271	0.266	1
Tbc1d32	0.0886431	1.34032561	0.017	0.01	1
Tmem242	0.08888887	0.32814888	0.033	0.05	1
Sp3os	0.08934068	0.94820787	0.052	0.039	1
Ehmt1	0.08935219	0.73115938	0.072	0.057	1
Gm39323	0.08942689	1.37096086	0.01	0.005	1
Papd4	0.08955524	0.27813923	0.089	0.117	1
Rangrf	0.08977274	1.23485403	0.016	0.009	1
Gm21781	0.08979007	1.23265102	0.012	0.006	1
Tjp3	0.09117373	1.3355098	0.01	0.005	1
Gm26541	0.09139198	0.99717064	0.017	0.01	1
Wdr33	0.09155538	0.78021995	0.073	0.058	1
Gna11	0.09166436	0.63749146	0.031	0.021	1
Rnf219	0.09222685	0.98977146	0.03	0.02	1
Dedd2	0.09238142	0.71958745	0.08	0.065	1
Zfp81	0.09242652	0.83920419	0.028	0.018	1
Supt5	0.09246373	0.41447463	0.276	0.27	1
Ccne2	0.09256083	1.13866218	0.017	0.01	1
Pla2g6	0.09258737	1.36954525	0.012	0.007	1
Zfp839	0.09464677	1.07206846	0.016	0.009	1
Slc25a42	0.09516971	1.02330615	0.012	0.007	1
9930111J21Ril	0.09534712	1.08806935	0.01	0.005	1
Ralgapa1	0.0956609	0.54477355	0.113	0.096	1
Spopl	0.09578552	0.72283496	0.101	0.085	1
D730003I15Ri	0.09580075	0.27949926	0.023	0.036	1
Tcim	0.09607588	1.21265797	0.012	0.007	1

Tbc1d31	0.09649177	1.0854966	0.03	0.02	1
Zdhhc13	0.09705051	0.70949287	0.04	0.029	1
Nup107	0.09725553	0.88079511	0.031	0.022	1
Rpe	0.09729731	0.79050759	0.089	0.073	1
Nelfcd	0.09748561	0.94233172	0.037	0.026	1
A830010M20F	0.0975577	0.8171651	0.012	0.007	1
Rab11fip2	0.09795539	0.72289364	0.094	0.078	1
Gm48882	0.09815104	0.87375512	0.01	0.005	1
Prmt9	0.09853064	1.3486762	0.03	0.02	1
Guf1	0.09865716	0.73633327	0.04	0.029	1
Rngtt	0.09877573	0.59393383	0.099	0.083	1
Rtp4	0.09887658	0.425121	0.068	0.053	1
Amfr	0.09893578	0.29443069	0.11	0.14	1
Dcaf15	0.09975036	0.54268858	0.103	0.086	1
Gtf2ird1	0.0998687	1.62308415	0.01	0.005	1
Tmem87a	0.10015755	0.50780378	0.101	0.084	1
Zfp646	0.10035695	0.71150017	0.086	0.07	1
Ppp1r12a	0.10039774	0.26724074	0.36	0.355	1
Mau2	0.10047065	0.45930597	0.162	0.144	1
E2f5	0.10066691	0.99776768	0.049	0.037	1
Uggt1	0.10079318	0.65877906	0.12	0.104	1
Herc2	0.1008058	0.45936166	0.154	0.135	1
Caap1	0.10087421	0.72309256	0.072	0.057	1
Klhdc1	0.1011382	0.79571953	0.024	0.016	1
Exosc8	0.10126046	0.72419248	0.127	0.111	1
Rad18	0.10185418	0.70625012	0.091	0.075	1
Tmem55a	0.10187572	1.29430081	0.026	0.017	1
Clk3	0.10194764	0.52693235	0.171	0.155	1
Zbtb45	0.10209449	1.13493137	0.019	0.012	1
Mettl3	0.10243407	0.80015434	0.049	0.037	1
Mdh1	0.10319426	0.49881489	0.276	0.274	1
Mbp	0.10355712	0.54344338	0.169	0.154	1
D2hgdh	0.10463136	1.23436111	0.017	0.011	1
Zfp217	0.10468745	0.8245148	0.059	0.046	1
Magee1	0.10508139	1.69934749	0.01	0.005	1
Ptov1	0.10517813	0.45855647	0.024	0.038	1
Mta2	0.10551834	0.60440122	0.147	0.13	1
Meaf6	0.10562794	0.91139187	0.073	0.059	1
Zfp846	0.10732541	1.31969631	0.021	0.013	1
Dennd1c	0.10775061	0.58826129	0.077	0.062	1
Ogfod1	0.10814218	1.11132852	0.026	0.018	1
Jazf1	0.10825436	1.23194419	0.01	0.005	1
Rfc1	0.10826685	0.6566749	0.099	0.084	1
Golga2	0.10846821	0.27211156	0.045	0.063	1
Fundc2	0.10848392	0.51935723	0.227	0.216	1
Snap47	0.10857167	0.93727819	0.014	0.008	1
Atg10	0.10921066	1.19846882	0.03	0.02	1
Tmem186	0.10921701	1.22029005	0.019	0.012	1

Dhx15	0.10968123	0.43587157	0.353	0.36	1
Rock2	0.10973303	0.45939404	0.23	0.219	1
Mturn	0.11037415	1.18824398	0.019	0.012	1
Blvra	0.11039673	0.36009752	0.04	0.057	1
Ddx58	0.11042634	0.57830445	0.084	0.068	1
Atg2b	0.11043727	0.90463613	0.04	0.029	1
Tmem9	0.11059677	1.32067532	0.023	0.015	1
Ly6k	0.11082924	1.18125607	0.012	0.007	1
Trmt13	0.11101344	0.62334302	0.077	0.062	1
Eif2s3y	0.11129006	0.59323936	0.101	0.085	1
Gpr89	0.11131735	1.10652905	0.044	0.032	1
Ppp6r2	0.11159374	0.69938632	0.04	0.029	1
Mms19	0.11167265	0.78738662	0.077	0.063	1
Dpagt1	0.11193733	0.2638304	0.012	0.022	1
Gm42715	0.11198209	1.10233593	0.014	0.008	1
Calcrl	0.11210715	0.75572151	0.04	0.029	1
Tada2a	0.11286652	0.91737908	0.014	0.008	1
Eefsec	0.11417568	0.66809355	0.038	0.028	1
Gga3	0.11431825	0.98637219	0.04	0.029	1
Desi2	0.11475339	0.62434965	0.126	0.11	1
Asb8	0.11541599	0.90829685	0.045	0.034	1
Ipp	0.11558535	0.87938212	0.017	0.011	1
Mcu	0.11573156	0.63492662	0.044	0.032	1
Mrfap1	0.11597399	0.4047355	0.33	0.335	1
Eya3	0.11675196	0.96984292	0.049	0.037	1
Srek1	0.11692142	0.68699587	0.18	0.165	1
Scaf1	0.1176211	0.62415059	0.077	0.062	1
Msl2	0.11771553	0.37675075	0.216	0.203	1
Mgea5	0.11803733	0.25853034	0.349	0.344	1
Tango6	0.11804533	0.78196531	0.017	0.011	1
Zfp512b	0.11827566	1.03559376	0.017	0.011	1
Unc13d	0.11862851	0.89200996	0.052	0.04	1
Bcl9	0.11923559	0.83093863	0.028	0.019	1
Ski	0.11948484	0.57140125	0.18	0.165	1
Cox18	0.12009024	0.93949911	0.042	0.031	1
Dtx3	0.1201787	0.67402011	0.082	0.068	1
Fxr2	0.12031511	0.5697282	0.094	0.079	1
Ighmbp2	0.12131481	0.85670131	0.026	0.018	1
Clasrp	0.12162852	0.87770193	0.033	0.024	1
Zfp799	0.1218691	1.11663418	0.019	0.012	1
Srcap	0.12236323	0.68682423	0.113	0.098	1
Ddx50	0.12340982	0.38217189	0.314	0.312	1
Endov	0.12345058	1.31536308	0.019	0.012	1
Csnk1g1	0.12561489	0.59036959	0.098	0.082	1
Lrrc28	0.1268147	1.08083986	0.026	0.018	1
Ap1g2	0.12690997	0.86757309	0.047	0.036	1
Unc45a	0.12731674	0.49194104	0.096	0.08	1
Gtf2a2	0.1275353	0.56229515	0.159	0.145	1

Ints4	0.12794463	1.17259151	0.031	0.022	1
Fxr1	0.1279556	0.41106597	0.293	0.293	1
Mepce	0.12813195	0.51134305	0.134	0.118	1
Mov10	0.12814081	1.24034716	0.03	0.021	1
Lmbr1l	0.12829342	0.69116168	0.075	0.061	1
Med18	0.12837126	1.39063165	0.012	0.007	1
Asap2	0.12865672	1.43371715	0.03	0.021	1
AC147041.1	0.12925618	1.1181521	0.012	0.007	1
Rb1	0.12938463	0.45309456	0.164	0.148	1
Hnrnpul1	0.12946887	0.42290547	0.293	0.293	1
2900076A07R	0.13035793	1.10136256	0.021	0.014	1
Mark4	0.13056914	0.32728267	0.021	0.033	1
Pef1	0.13095622	0.80348758	0.052	0.041	1
Eipr1	0.13219161	0.78670951	0.086	0.072	1
Dctn6	0.13228772	0.75063051	0.084	0.07	1
Pwwp2a	0.13252663	0.59796089	0.098	0.082	1
Prdm10	0.13256702	0.63341575	0.045	0.034	1
Tut1	0.13261074	0.70441647	0.038	0.028	1
Rnf170	0.13263203	0.82964414	0.037	0.027	1
Tet3	0.13346198	0.61710046	0.18	0.166	1
Ostm1	0.1337766	0.94006965	0.089	0.076	1
Slc4a7	0.13425753	0.54936349	0.265	0.264	1
Slmap	0.13475363	0.68476348	0.084	0.07	1
Ubn2	0.13489039	0.49465436	0.204	0.191	1
Nupl2	0.13562725	1.70887235	0.01	0.006	1
Helq	0.13586528	0.61135158	0.016	0.01	1
Cxxc1	0.13587982	0.59186412	0.08	0.067	1
Hmgxb3	0.13602214	0.81194144	0.042	0.031	1
9930021J03Ril	0.13643097	0.46917681	0.103	0.087	1
Ppp3r1	0.13669111	0.5083505	0.15	0.137	1
Fcho1	0.13725096	0.9266712	0.045	0.035	1
Pold2	0.13747381	0.8939532	0.033	0.024	1
Ubash3a	0.13819372	0.55262473	0.063	0.05	1
Bora	0.13833608	0.60417328	0.03	0.021	1
Kantr	0.13845857	1.13688078	0.024	0.017	1
Cfap97	0.13859713	0.64306282	0.061	0.049	1
Xndc1	0.13923994	0.85685391	0.026	0.018	1
Gm16124	0.13926595	1.02377531	0.016	0.01	1
Immp1l	0.13966687	0.60525036	0.091	0.077	1
Fam58b	0.13980032	0.69486686	0.042	0.031	1
Lpin1	0.14001655	0.57348115	0.068	0.055	1
Pms1	0.14109984	0.99110292	0.017	0.011	1
E230001N04R	0.14157235	0.68370421	0.017	0.011	1
Lrrc42	0.14182062	1.15875813	0.033	0.024	1
Sacm1l	0.14226478	0.87934178	0.084	0.071	1
Zfp952	0.1429853	0.75741294	0.035	0.025	1
Rnf20	0.1430194	0.42321158	0.176	0.162	1
Fam117a	0.14354734	0.8470525	0.033	0.024	1

Myg1	0.14355829	0.69729423	0.054	0.043	1
C920006O11F	0.14426684	0.82446788	0.012	0.007	1
Tcf20	0.14455697	0.49214375	0.143	0.128	1
Shmt1	0.14459193	0.9279318	0.01	0.006	1
Emc9	0.14487595	1.54478008	0.023	0.015	1
9330160F10Ri	0.14508305	1.16695116	0.021	0.014	1
Zfhx2	0.14528863	0.7799082	0.04	0.03	1
Dpy19l4	0.14564303	1.0546965	0.04	0.03	1
Herc6	0.14587052	0.81388394	0.068	0.056	1
Ndufaf1	0.14613873	1.10622636	0.026	0.018	1
Espn	0.14665618	0.7656756	0.031	0.023	1
Zfyve27	0.14673502	0.96777914	0.061	0.049	1
Ubald1	0.14674232	0.49652846	0.14	0.124	1
Plekha5	0.14734271	0.53048397	0.077	0.063	1
Hdgfl3	0.14741147	0.49740357	0.04	0.03	1
Hist1h4n	0.14757478	1.32894755	0.01	0.006	1
Mast2	0.14816182	0.80677675	0.033	0.024	1
Ank	0.14848317	0.4401974	0.037	0.051	1
Ccnh	0.14875935	0.93220375	0.058	0.046	1
Rpusd3	0.14924121	1.52090887	0.01	0.006	1
AW549877	0.1498153	0.44766114	0.113	0.099	1
Brd8	0.15106106	0.40200474	0.222	0.211	1
Vsig10	0.1511257	1.48953737	0.012	0.007	1
Scamp3	0.15141351	0.62884689	0.124	0.111	1
Fbrsl1	0.15200776	0.85981249	0.098	0.085	1
Lca5	0.1520474	1.57361053	0.01	0.006	1
Prmt3	0.15270282	0.67918464	0.056	0.044	1
Anks3	0.15305196	0.65470438	0.049	0.038	1
Rnaseh1	0.15319256	0.87051426	0.035	0.026	1
Ptgfrn	0.15387195	1.34818203	0.016	0.01	1
Ttll12	0.15414602	1.19040654	0.023	0.015	1
Gpatch1	0.15418425	1.18631284	0.021	0.014	1
Trip11	0.15433021	0.51756279	0.234	0.227	1
Asf1a	0.1544434	0.65490076	0.077	0.064	1
Rhobtb2	0.15475011	0.43209558	0.047	0.036	1
Qrich1	0.15538487	0.61007305	0.138	0.124	1
Rev3l	0.15566006	0.61686402	0.099	0.086	1
Ccsap	0.15571455	0.65233059	0.028	0.02	1
Mmaa	0.15590638	1.35075774	0.012	0.007	1
Mylpf	0.15661913	1.39931759	0.01	0.006	1
Smim11	0.15690581	0.55970011	0.15	0.137	1
Kdm4c	0.15731012	0.88423452	0.059	0.048	1
Dpy19l3	0.157408	0.64400851	0.045	0.035	1
Crtc1	0.15799098	1.28633932	0.014	0.009	1
Nrg2	0.15854788	1.22975862	0.012	0.007	1
Myef2	0.15874032	0.55058494	0.099	0.086	1
4930402H24R	0.15950637	0.68978399	0.035	0.026	1
Prickle3	0.15951847	0.66455428	0.026	0.018	1

Ube4b	0.15962861	0.3481272	0.084	0.07	1
Atxn2l	0.15968807	0.4650282	0.23	0.223	1
Spon2	0.1597767	0.96808659	0.023	0.015	1
Slc26a2	0.15980418	0.8346919	0.12	0.108	1
Cdk12	0.16030038	0.33020243	0.286	0.281	1
Ralgapb	0.16048316	0.56176705	0.094	0.081	1
Coil	0.16164249	1.06129624	0.026	0.018	1
Tstd2	0.16224275	0.78544991	0.037	0.027	1
Myo18a	0.16233416	0.79518888	0.052	0.042	1
Mrpl1	0.1625266	0.78621079	0.054	0.043	1
Dusp8	0.16264123	1.093898	0.021	0.014	1
Mtfr1	0.16267445	0.88021492	0.038	0.029	1
Stk38	0.16304236	0.40692084	0.155	0.14	1
Prkdc	0.16354928	0.60970101	0.045	0.035	1
Ccnt1	0.16417554	0.37716037	0.283	0.281	1
Synj2	0.1649081	0.60174795	0.049	0.038	1
Mapre2	0.16493889	0.53698539	0.148	0.136	1
mt-Atp8	0.16543839	0.47911857	0.206	0.196	1
Inpp5k	0.16547902	0.51687673	0.073	0.061	1
Dpysl2	0.1656105	0.26388038	0.141	0.174	1
Zhx3	0.16631296	1.03172249	0.017	0.011	1
Frs2	0.16636518	0.66885611	0.063	0.051	1
Gdap10	0.16717995	0.48005207	0.108	0.095	1
Cdyl2	0.1671809	0.98237165	0.019	0.013	1
Mrps15	0.16767178	0.44615912	0.099	0.126	1
Ndufs4	0.16871315	0.26164237	0.19	0.232	1
Suv39h2	0.16965757	1.07826846	0.019	0.013	1
Zfp26	0.17002527	0.58689795	0.059	0.048	1
Zxdb	0.17030353	0.6894346	0.051	0.04	1
Bcl7b	0.17045364	0.49402778	0.159	0.146	1
Mbtd1	0.17056069	0.30878836	0.147	0.181	1
Aaas	0.17057008	0.86446584	0.024	0.017	1
Zfand1	0.17161197	0.79046942	0.028	0.02	1
Tbc1d22a	0.1717764	0.36923277	0.037	0.05	1
Pgm2	0.17194397	0.88606343	0.028	0.02	1
Mapk8ip3	0.17292855	0.55250513	0.089	0.076	1
Xpo4	0.17385243	0.44552024	0.106	0.092	1
Cisd1	0.17419234	0.74260692	0.094	0.082	1
Ttc37	0.17485072	0.84204879	0.051	0.04	1
Scml4	0.17506216	0.28038332	0.035	0.026	1
Tnks	0.175696	0.27955024	0.065	0.083	1
Azi2	0.17571522	0.49970361	0.188	0.179	1
Fam210b	0.17625721	0.90252257	0.037	0.028	1
Igkv1-135	0.17636248	1.49134	0.01	0.006	1
Fam168b	0.17669667	0.29674339	0.236	0.226	1
Hmgn2	0.17684391	0.40448588	0.169	0.156	1
Sfpq	0.17738317	0.28127206	0.504	0.543	1
Dnajc17	0.17790288	1.35141256	0.024	0.017	1

Casp8ap2	0.178158	0.58664623	0.161	0.149	1
Ccs	0.17820599	0.97864045	0.065	0.054	1
Gm42726	0.17854601	0.75630295	0.014	0.009	1
Ttf2	0.1786485	0.75431028	0.03	0.022	1
St3gal2	0.17873339	0.83086277	0.021	0.014	1
Nxf1	0.17925484	0.37382326	0.201	0.189	1
Abhd18	0.17925689	0.89637892	0.04	0.031	1
Fcho2	0.17954155	0.58176415	0.169	0.158	1
Pcbp3	0.1798507	1.18740796	0.01	0.006	1
Gpcpd1	0.18114525	0.44026361	0.248	0.241	1
D230025D16F	0.18148977	0.48993228	0.042	0.032	1
Chpt1	0.18152307	0.39816399	0.028	0.039	1
Cdc25b	0.18250927	1.01199651	0.035	0.026	1
Fbxl3	0.18251192	0.44695702	0.201	0.192	1
Mcur1	0.18258467	0.72406458	0.115	0.104	1
Nmnat1	0.18266679	1.10583916	0.014	0.009	1
Tg	0.18270347	0.77342589	0.012	0.007	1
Epm2aip1	0.18317443	0.43084099	0.072	0.059	1
Tmem55b	0.18324652	0.50524489	0.164	0.152	1
Hic2	0.18358945	0.66084184	0.009	0.016	1
Pinx1	0.18362219	0.27834059	0.026	0.037	1
Parn	0.18443382	0.52855062	0.04	0.031	1
Abcb9	0.18451458	0.57293379	0.038	0.029	1
Tspan14	0.18494648	0.47454817	0.112	0.098	1
Arl2	0.18496762	0.27933725	0.017	0.027	1
Ddx55	0.18499469	0.77970767	0.052	0.042	1
Gtpbp2	0.18524506	0.44051191	0.148	0.135	1
Zfp386	0.1853595	0.8503201	0.049	0.039	1
Tnks1bp1	0.18536043	1.07638064	0.017	0.012	1
Nudt16l1	0.18610712	0.63296572	0.058	0.047	1
Farp1	0.18682949	0.57634908	0.021	0.014	1
Maip1	0.18705073	0.29774603	0.056	0.072	1
Plcb2	0.18749508	0.89694914	0.065	0.054	1
Hsf1	0.18786674	0.56293383	0.077	0.065	1
Hist1h3a	0.18866119	0.62553878	0.037	0.028	1
Rnf111	0.19030002	0.476161	0.202	0.192	1
Ripk3	0.19033654	0.45638061	0.026	0.037	1
Suv39h1	0.19088858	0.68056888	0.033	0.025	1
Isca2	0.19115936	0.32060912	0.059	0.076	1
Map3k3	0.19173376	0.68032235	0.073	0.062	1
Otud4	0.1923206	0.50203417	0.136	0.124	1
Coq10b	0.19383724	0.43867707	0.265	0.266	1
Ppm1l	0.19430661	0.95517058	0.019	0.013	1
Wdr73	0.19434091	0.75655258	0.024	0.017	1
1700066M21F	0.19451217	0.74913355	0.01	0.006	1
Camsap2	0.19625829	0.89413105	0.054	0.044	1
Tmem38b	0.19696339	0.74832652	0.051	0.041	1
Cmc1	0.1969852	0.51053605	0.173	0.164	1

Sufu	0.19701839	0.7156392	0.028	0.02	1
Trrap	0.19735411	0.55492697	0.087	0.075	1
Trmt11	0.19775744	0.96362521	0.037	0.028	1
Gmip	0.19777031	0.27776445	0.075	0.093	1
Mbip	0.19787779	0.61348872	0.049	0.039	1
Llgl1	0.19816522	1.10446983	0.03	0.022	1
Rbm12b1	0.19836411	0.79865791	0.014	0.009	1
Zfp930	0.19890601	0.86828926	0.021	0.015	1
Araf	0.19897265	0.70398764	0.129	0.117	1
Cdk8	0.19910552	0.54849683	0.077	0.065	1
Shprh	0.19913777	0.67852878	0.051	0.04	1
Coa6	0.19971134	0.259755	0.042	0.055	1
Csnk1d	0.19971329	0.37218572	0.25	0.248	1
Wrnip1	0.20034304	0.66783289	0.058	0.047	1
Mthfd1l	0.2006463	0.63552553	0.033	0.025	1
Ermp1	0.20116278	0.7944111	0.052	0.043	1
Ampd2	0.20121887	1.1246216	0.023	0.016	1
Lrp10	0.20162388	0.42917781	0.234	0.229	1
Sco2	0.20164965	0.317638	0.017	0.026	1
Adar	0.20212509	0.69272281	0.082	0.07	1
Ptges3	0.2024267	0.35853503	0.368	0.386	1
Fbxo6	0.20246196	0.44432439	0.04	0.053	1
Supt16	0.20255604	0.40089558	0.222	0.214	1
Marveld2	0.20257024	1.15846758	0.012	0.008	1
Smim15	0.20263345	0.38834568	0.157	0.145	1
Zmym2	0.20269243	0.45416252	0.092	0.08	1
Letmd1	0.20274522	0.85457294	0.037	0.028	1
Naa60	0.2030128	0.81525255	0.056	0.046	1
Arsa	0.20305237	0.4269947	0.019	0.028	1
Gm17092	0.20374881	1.54882844	0.01	0.006	1
Bmp4	0.20410753	0.32368863	0.005	0.011	1
Otud5	0.20440099	0.70101824	0.134	0.124	1
Parg	0.2045828	0.49392813	0.105	0.092	1
Traf5	0.20520777	1.01512884	0.052	0.043	1
Ppp2r2a	0.20544137	0.46101083	0.243	0.239	1
Msto1	0.20658646	0.70023273	0.023	0.016	1
Bcdin3d	0.20674624	1.03442467	0.023	0.016	1
Ndufb3	0.20763404	0.50711095	0.276	0.278	1
Fbxo25	0.20764548	0.54076816	0.021	0.015	1
Gon4l	0.2077091	0.63684977	0.08	0.069	1
Oxnad1	0.20858953	0.76338432	0.014	0.009	1
Hcfc1	0.20863878	0.49863648	0.089	0.077	1
Irgm2	0.20924672	0.47422923	0.054	0.044	1
Spast	0.20959646	0.35398369	0.052	0.067	1
Brms1l	0.20978431	0.73953187	0.024	0.018	1
Rccd1	0.2098557	0.81691599	0.021	0.015	1
Cspp1	0.20990548	0.39187953	0.117	0.104	1
Ormdl1	0.21005462	0.79888365	0.045	0.036	1

Usp1	0.21081188	0.4620792	0.138	0.125	1
Sept2	0.21084147	0.38239984	0.028	0.039	1
Gcn1l1	0.21195905	0.57771935	0.105	0.093	1
Slfn8	0.21212866	0.65669874	0.075	0.064	1
Yme1l1	0.21237423	0.48852908	0.215	0.207	1
Srfbp1	0.21288351	0.72433821	0.044	0.035	1
Siah1a	0.21300596	0.26102492	0.038	0.051	1
Cog3	0.21347019	0.78057119	0.051	0.041	1
Atp6v1h	0.21362617	0.42583654	0.187	0.177	1
Nup205	0.2137981	0.73626217	0.051	0.041	1
Msh3	0.21417073	0.84346026	0.047	0.038	1
Slc27a1	0.21479412	0.6727584	0.007	0.013	1
Cdr2	0.21525141	0.7193508	0.04	0.031	1
Tmem209	0.21622779	0.47256186	0.049	0.039	1
Zfp758	0.21648659	0.63090394	0.038	0.03	1
Gtf3c1	0.21649139	0.70232438	0.079	0.068	1
Fbxw4	0.21670043	0.80920109	0.035	0.027	1
Nfyb	0.21697894	0.42929884	0.127	0.115	1
Rmnd1	0.21736879	0.66992212	0.019	0.013	1
Clpb	0.21749675	0.77381682	0.028	0.021	1
Abraxas1	0.21758898	0.80483636	0.016	0.01	1
Psen1	0.21759408	0.40822255	0.082	0.102	1
Rabgap1	0.21835108	0.72870615	0.079	0.067	1
Eya2	0.21836123	0.83834416	0.01	0.006	1
Zfp317	0.21922249	0.8526792	0.028	0.021	1
Faf2	0.2193382	0.64256814	0.099	0.088	1
Pwwp2b	0.21980322	0.5522864	0.005	0.011	1
Tmem173	0.21987296	0.25459279	0.065	0.081	1
March3	0.22005069	0.7549023	0.021	0.015	1
Srsf11	0.22034127	0.29645984	0.455	0.499	1
Crebzf	0.22159186	0.42292878	0.126	0.113	1
Cluap1	0.2222995	0.83525848	0.028	0.021	1
Anp32e	0.22231235	0.32125164	0.298	0.302	1
Capn1	0.22260161	0.52384228	0.101	0.089	1
Trmt1l	0.22273622	0.68874936	0.098	0.087	1
Cdk7	0.22295986	0.66202114	0.052	0.043	1
Runx1	0.22307092	0.28409259	0.344	0.352	1
Usp22	0.22317416	0.72935601	0.075	0.065	1
Uqcc3	0.22346442	0.40691421	0.164	0.152	1
Helz	0.22375968	0.42874379	0.14	0.127	1
Szt2	0.22387572	1.33646017	0.019	0.013	1
Tnfrsf13b	0.22398222	0.42314646	0.075	0.063	1
Hddc2	0.22398829	0.85040036	0.049	0.04	1
Zxdc	0.22423571	0.68449536	0.042	0.033	1
Zscan26	0.22503472	0.77311408	0.052	0.043	1
Tbl1xr1	0.22521008	0.5270772	0.166	0.155	1
Smim19	0.2258056	0.7023055	0.075	0.064	1
Zfp119b	0.2259249	1.31129575	0.01	0.006	1

Ice2	0.2265378	0.97539172	0.021	0.015	1
Fundc1	0.22708021	0.43554835	0.056	0.071	1
Ythdc2	0.22713459	0.56938944	0.077	0.066	1
Zkscan1	0.22735974	0.30916458	0.038	0.05	1
Zfp777	0.22798125	0.81903155	0.033	0.025	1
Dcaf17	0.22828984	0.63281643	0.042	0.033	1
Xxylt1	0.2288311	0.99456127	0.028	0.021	1
Xylt2	0.22906269	1.04316505	0.023	0.016	1
Zfyve16	0.22919361	0.39426512	0.087	0.076	1
Pcgf2	0.22993068	1.48373637	0.01	0.006	1
Ttc14	0.23038512	0.30841952	0.346	0.355	1
Gm30054	0.23086295	0.75026138	0.038	0.03	1
Ago3	0.23098779	0.61157286	0.082	0.071	1
C1ra	0.2312798	0.47423243	0.007	0.013	1
Pign	0.23196121	0.70255735	0.042	0.033	1
Dst	0.23206942	0.72135454	0.091	0.08	1
Nfatc2ip	0.23289836	0.40792631	0.054	0.044	1
mt-Nd6	0.23299401	0.52052449	0.079	0.068	1
Srp54a	0.2332311	0.62553687	0.103	0.092	1
Tubgcp4	0.23355752	0.75149732	0.073	0.063	1
Lyrm2	0.23384323	0.64656167	0.066	0.056	1
Prmt6	0.23411424	0.3018245	0.021	0.03	1
Zzz3	0.23553149	0.74199785	0.124	0.114	1
Kdm6a	0.23601216	0.48947004	0.182	0.173	1
Chd3os	0.23640132	0.96774028	0.012	0.008	1
Iqcc	0.2364806	0.85431307	0.026	0.02	1
Smim24	0.23660926	0.43983955	0.017	0.026	1
Eif2b2	0.23664153	0.31964836	0.096	0.117	1
Fancc	0.23670568	0.46905435	0.014	0.021	1
Polr1a	0.23705063	0.67180089	0.068	0.058	1
Rad17	0.23739055	0.52339224	0.075	0.064	1
Nbl1	0.23794346	0.77586034	0.014	0.009	1
AW011738	0.23798311	0.49692994	0.087	0.076	1
Vps41	0.23821762	0.73815823	0.101	0.091	1
Pum2	0.23894481	0.27411116	0.347	0.361	1
Fam214a	0.23901353	0.28377812	0.079	0.067	1
Fem1a	0.23920392	0.43726858	0.026	0.036	1
Atp1b3	0.24052546	0.30675984	0.33	0.341	1
Ccno	0.24127328	0.61310934	0.012	0.008	1
2810001G20R	0.24185945	0.62141465	0.045	0.036	1
Kpna1	0.24226874	0.34818404	0.253	0.303	1
Smarcc1	0.24228277	0.3411735	0.082	0.101	1
Pou2f1	0.24229024	0.53267253	0.103	0.092	1
Pvr	0.24229248	0.40451567	0.073	0.091	1
Cep57	0.24235651	0.27335058	0.108	0.096	1
Micu3	0.24257123	0.73574205	0.033	0.026	1
Samd1	0.2427827	0.96632535	0.031	0.024	1
Trim37	0.24278572	1.08395872	0.026	0.02	1

E130307A14R	0.2434596	0.9826599	0.019	0.014	1
Bin2	0.24418971	0.61574662	0.143	0.134	1
E4f1	0.24421044	0.56146752	0.091	0.08	1
Qtrt1	0.24560266	0.71389333	0.031	0.024	1
Kif3b	0.24567127	0.53471469	0.044	0.035	1
A530017D24R	0.24582378	1.51435187	0.014	0.009	1
Lyrm9	0.2467162	0.25376731	0.037	0.029	1
Rpp38	0.24689241	0.8870314	0.031	0.024	1
Esco1	0.24774354	0.50707471	0.143	0.133	1
Smpdl3a	0.24819589	0.61867646	0.187	0.184	1
Inpp5e	0.24819876	1.20256618	0.021	0.015	1
Pskh1	0.24874891	1.0884975	0.026	0.02	1
Tmem101	0.24896751	0.54594131	0.01	0.017	1
Rmdn1	0.24932939	0.41947129	0.01	0.017	1
Gm15417	0.24947471	1.22845489	0.021	0.015	1
Cdkl3	0.24983929	0.90532253	0.012	0.008	1
Dhcr7	0.2500218	1.26334672	0.023	0.017	1
Gvin1	0.25029565	0.52925479	0.005	0.01	1
Cnpy4	0.25060753	0.387024	0.014	0.021	1
Fntb	0.25068064	1.32622358	0.01	0.007	1
Mctp2	0.25088764	0.5489793	0.056	0.046	1
Mrpl42	0.25109352	0.66632192	0.117	0.107	1
Sf3b2	0.25128398	0.277508	0.382	0.405	1
Osgepl1	0.25133508	0.44825816	0.012	0.008	1
Dennd1a	0.25179569	0.32146991	0.058	0.072	1
Ebag9	0.25195164	0.7023361	0.056	0.047	1
Apba3	0.25227456	1.19908211	0.026	0.02	1
Ppil2	0.25305651	0.61543511	0.103	0.093	1
Slf1	0.25344712	0.74117529	0.04	0.032	1
Ubr2	0.25363943	0.61253856	0.178	0.171	1
Ccdc82	0.25403357	0.64608061	0.105	0.095	1
Cpq	0.25482891	0.81613607	0.03	0.04	1
Aip	0.25556443	0.52367712	0.126	0.116	1
Mpg	0.25598935	0.29460143	0.031	0.041	1
Kdm5d	0.25658745	0.8176141	0.035	0.027	1
Kdm1b	0.25686681	0.65264637	0.024	0.018	1
Nudt22	0.25686726	0.39130755	0.007	0.012	1
Simc1	0.25695233	0.4763977	0.028	0.021	1
Arhgap12	0.2575548	0.71604151	0.045	0.037	1
Gm5914	0.25775882	0.87427229	0.03	0.023	1
Kif3c	0.25801661	1.06750321	0.012	0.008	1
Haus2	0.25806386	0.42663243	0.031	0.041	1
Atm	0.25807781	0.70551306	0.051	0.042	1
Il1rl2	0.25901058	1.13785852	0.014	0.009	1
Smg8	0.25939391	0.34089684	0.021	0.029	1
Gpr107	0.25964875	0.5832108	0.112	0.102	1
Slc7a6	0.26064727	0.66547998	0.026	0.02	1
Arhgap1	0.26065552	0.65724151	0.08	0.071	1

Ifit1	0.26156645	0.55837349	0.038	0.031	1
Cpsf7	0.26171724	0.72337432	0.15	0.143	1
Cdkal1	0.26232002	0.60288773	0.052	0.043	1
Iws1	0.2625433	0.49693902	0.175	0.167	1
Psat1	0.26350568	0.41883795	0.03	0.039	1
Cep152	0.26362655	0.48988488	0.019	0.014	1
Nin	0.26398072	0.54413689	0.141	0.132	1
Pnkp	0.26406157	0.61892914	0.054	0.045	1
Dr1	0.26421549	0.65852989	0.092	0.082	1
Ranbp2	0.2645618	0.353279	0.373	0.388	1
1110032A03R	0.26580384	0.26674086	0.023	0.031	1
2810006K23R	0.26604049	0.75375671	0.014	0.009	1
Rad54l2	0.26653129	0.32935094	0.054	0.067	1
Aftph	0.26678439	0.51156146	0.208	0.203	1
Atg16l1	0.26782505	0.42000374	0.08	0.07	1
Tlcd1	0.26862254	1.08742143	0.023	0.017	1
Col27a1	0.26951784	0.50860531	0.005	0.01	1
Adnp2	0.27029251	0.78610147	0.044	0.036	1
Aqr	0.27039185	0.41278183	0.159	0.149	1
Polr3g	0.27063006	0.88330315	0.016	0.011	1
Zfr	0.2715733	0.55987159	0.15	0.142	1
Tmc8	0.27160682	0.51445703	0.017	0.012	1
Fam149b	0.27174933	0.85397408	0.026	0.02	1
Ppm1m	0.27222131	0.49825841	0.049	0.062	1
Ints10	0.27230314	0.96572265	0.024	0.018	1
Hdac8	0.2725206	0.86244726	0.026	0.02	1
Ankrd39	0.27340222	0.96939588	0.031	0.025	1
Plekhf2	0.2736161	0.52590313	0.11	0.1	1
Mib1	0.27459103	0.27737573	0.103	0.123	1
Nufip2	0.27492803	0.39954706	0.229	0.223	1
Chac2	0.27535724	0.76131492	0.01	0.007	1
Dip2c	0.2757218	0.67256252	0.017	0.025	1
Gm10073	0.27642398	0.35759751	0.073	0.089	1
Cwf19l1	0.27643822	1.24287451	0.016	0.011	1
Dis3	0.27652556	0.67083396	0.035	0.028	1
Gapvd1	0.27697627	0.51013982	0.164	0.157	1
Fbxw5	0.27710372	0.73195406	0.037	0.029	1
Abtb1	0.27733043	0.43331395	0.058	0.048	1
Spindoc	0.27740941	1.06823296	0.019	0.014	1
Igfbp3	0.27816524	0.63221295	0.021	0.015	1
Afap1	0.27874097	1.25492243	0.024	0.019	1
Zfp850	0.27912309	0.95911809	0.01	0.007	1
Cat	0.27922539	0.26143847	0.051	0.063	1
Snrnp27	0.28083537	0.54163317	0.197	0.196	1
Snapc4	0.28133902	1.05334742	0.019	0.014	1
Ofd1	0.28183716	0.54798381	0.038	0.031	1
Invs	0.28232044	0.60673348	0.019	0.014	1
6330418K02R	0.28254435	1.02400503	0.01	0.007	1

Mfap1a	0.28257995	0.46416335	0.096	0.085	1
C730034F03R	0.28327403	0.55453036	0.028	0.021	1
Avl9	0.28351532	0.27136668	0.239	0.232	1
Orc4	0.2835479	0.45674964	0.108	0.098	1
Mthfr	0.28369895	0.38049933	0.016	0.023	1
Zfp277	0.28413247	0.3207688	0.056	0.069	1
Fbxo9	0.28474844	0.46162635	0.066	0.057	1
Zfp868	0.28538062	0.4782521	0.063	0.054	1
Vopp1	0.28569618	0.26999981	0.063	0.077	1
Strada	0.28608689	0.65946767	0.04	0.033	1
Gm37387	0.28634021	1.305197	0.012	0.008	1
Fbxl14	0.2863915	0.57548722	0.068	0.059	1
Ang	0.28650456	0.44180664	0.009	0.014	1
Mettl23	0.28670615	0.38876044	0.232	0.228	1
Prpf3	0.28693178	0.93197759	0.054	0.046	1
Abhd14b	0.28731155	0.25744282	0.007	0.012	1
9030624J02Ril	0.28793799	0.36410955	0.054	0.067	1
Recql5	0.2887371	0.7684314	0.017	0.013	1
Epc1	0.28886155	0.3002722	0.211	0.204	1
Tubgcp3	0.28904126	0.56878748	0.073	0.064	1
Tmem87b	0.28950225	0.29277994	0.051	0.063	1
Taf1a	0.28951296	0.69795541	0.021	0.016	1
1110008L16Ri	0.2896062	1.34167158	0.014	0.01	1
E130311K13R	0.29018132	1.07792329	0.017	0.013	1
Rab43	0.29055293	0.34773329	0.044	0.055	1
Mcm3ap	0.29055579	0.46840384	0.049	0.041	1
Mical3	0.29081881	1.14255994	0.016	0.011	1
9930022D16R	0.29113557	0.34464506	0.014	0.02	1
Pip5k1a	0.29136518	0.36488298	0.178	0.17	1
Llgl2	0.29175812	0.90992996	0.012	0.008	1
Hint3	0.29269009	0.70163919	0.12	0.112	1
4930430F08Ri	0.29297708	1.22885888	0.014	0.01	1
Zfp687	0.29356501	0.40656928	0.037	0.029	1
Lsm11	0.29415312	0.257042	0.016	0.022	1
Sec22c	0.29462879	0.57578234	0.014	0.01	1
Fbn1	0.2947792	0.68767838	0.016	0.011	1
Oxr1	0.29524937	0.40634227	0.094	0.084	1
Rab23	0.29604153	0.75432847	0.014	0.01	1
Orc3	0.29684387	0.63209179	0.052	0.044	1
Spred2	0.29697522	0.58234522	0.049	0.061	1
Mrpl9	0.29698459	0.26430727	0.154	0.18	1
Sptlc2	0.29717114	0.26254059	0.087	0.105	1
Luc7l	0.29789655	0.25591887	0.138	0.127	1
Zfp65	0.29796249	0.70831207	0.03	0.023	1
Mief2	0.29827158	1.51878888	0.012	0.008	1
Trim33	0.29838351	0.52290595	0.103	0.094	1
Ndufaf3	0.29847319	0.77092906	0.058	0.05	1
Naa15	0.29877489	0.36415957	0.227	0.222	1

Gopc	0.29927273	0.58368152	0.056	0.047	1
Cabin1	0.29956216	0.64885439	0.108	0.099	1
Coq5	0.30025793	0.57733197	0.061	0.053	1
Aldh4a1	0.30055427	1.04946026	0.017	0.013	1
Ift74	0.30116016	1.34190551	0.012	0.008	1
Eif2s1	0.30138618	0.2574436	0.166	0.197	1
Cant1	0.30170383	0.68326394	0.056	0.048	1
Slc41a3	0.30177026	0.33045939	0.01	0.016	1
Cds2	0.30204028	0.43516704	0.098	0.087	1
Prrc2a	0.30230561	0.27784927	0.237	0.234	1
Fam173b	0.303155	0.84479388	0.033	0.027	1
Dhx58	0.30340997	0.27400702	0.016	0.022	1
Kcmf1	0.30345938	0.30368714	0.168	0.199	1
1110038F14Ri	0.30362167	0.68981756	0.115	0.106	1
Hist2h4	0.30381481	0.75253981	0.021	0.016	1
Zfp101	0.30384985	0.60306273	0.024	0.019	1
C1d	0.30395977	0.33249379	0.08	0.097	1
Med7	0.30398813	0.53145844	0.092	0.084	1
Tubg1	0.30435434	1.06349403	0.014	0.01	1
Nbeal2	0.30491587	0.55841634	0.035	0.028	1
Rab2b	0.30537698	0.3849426	0.051	0.042	1
Trim44	0.30557127	0.63889171	0.133	0.124	1
Cacul1	0.30567199	0.26012994	0.077	0.092	1
Gpatch4	0.30579597	0.72113286	0.033	0.027	1
Nono	0.30652823	0.28330432	0.277	0.283	1
Trim27	0.30681879	0.40062043	0.091	0.109	1
Cerkl	0.30712523	1.31734851	0.01	0.007	1
Cpsf6	0.30738657	0.4199252	0.23	0.229	1
Cep63	0.30772438	0.66560325	0.059	0.051	1
BC052040	0.30831318	0.93139057	0.012	0.008	1
Enc1	0.308597	0.93467408	0.024	0.019	1
Slc35a5	0.30889085	0.67530754	0.028	0.022	1
Haus6	0.30892632	0.70651275	0.044	0.036	1
Golga5	0.30976189	0.71247524	0.075	0.067	1
Dnajc1	0.31078515	0.4015331	0.204	0.2	1
Pbrm1	0.31099556	0.33128083	0.274	0.279	1
Nbn	0.3116845	0.55012542	0.068	0.059	1
Zfp688	0.31466418	0.65309902	0.033	0.027	1
Cand1	0.31468362	0.63274606	0.119	0.112	1
Fiz1	0.31485206	0.25431539	0.056	0.068	1
Ehmt2	0.3151917	0.71968433	0.106	0.099	1
Zfp943	0.31539628	0.35843071	0.031	0.025	1
Ate1	0.31582472	0.56957281	0.092	0.083	1
Ccdc191	0.31596815	0.53103188	0.014	0.01	1
Ube2q2	0.31597681	0.53473714	0.091	0.082	1
Dsel	0.31630148	1.09111637	0.012	0.008	1
Yars2	0.31681815	0.29352501	0.01	0.016	1
Pelp1	0.31685338	0.81816138	0.045	0.038	1

3110062M04F	0.31705839	0.49344952	0.037	0.03	1
Prr14	0.31791299	0.5423939	0.106	0.098	1
Aff1	0.3183589	0.26166137	0.284	0.338	1
Spint2	0.31864102	0.71292315	0.079	0.07	1
Gbp8	0.3189163	0.73692167	0.065	0.056	1
Strn3	0.31915309	0.33810374	0.162	0.153	1
Capn10	0.32056936	0.25198989	0.009	0.014	1
Phtf1	0.32112955	0.42341837	0.049	0.041	1
Dph6	0.3219967	0.58179364	0.047	0.04	1
Taf1b	0.32212952	0.73684377	0.045	0.038	1
Stag1	0.32272649	0.42699945	0.18	0.176	1
Synrg	0.32298392	0.4116639	0.113	0.104	1
Lsm8	0.32307007	0.25419583	0.152	0.178	1
Mrpl58	0.3236439	0.37387925	0.101	0.12	1
Ifnar1	0.32378235	0.37209076	0.339	0.354	1
H1f0	0.32387312	0.48775806	0.103	0.094	1
Rexo4	0.32398374	0.31392064	0.073	0.087	1
Cables2	0.32421766	0.27561433	0.038	0.031	1
Ddx47	0.32447372	0.53006618	0.164	0.159	1
Decr2	0.32463323	0.99988628	0.019	0.014	1
Fbxo46	0.32498151	0.80586244	0.023	0.017	1
Tnk2	0.32567225	0.4450865	0.049	0.041	1
Fbxo3	0.32567951	0.45787537	0.086	0.077	1
Otud1	0.32587848	0.30944964	0.026	0.034	1
Mysm1	0.32751961	0.3782266	0.209	0.207	1
Ube2e1	0.32778596	0.38752541	0.101	0.121	1
Vrk1	0.32915667	0.63959978	0.119	0.111	1
Dcun1d2	0.32938106	0.75451614	0.014	0.01	1
Plcb4	0.32950569	0.40252508	0.051	0.043	1
Irf2bp1	0.32977709	0.74230258	0.04	0.033	1
Ccndbp1	0.33046027	0.58289642	0.12	0.113	1
Txlna	0.33074918	0.28928749	0.082	0.097	1
Alkbh8	0.3308006	0.48792458	0.038	0.031	1
Nelfb	0.33137632	0.50990455	0.092	0.084	1
Zfp276	0.3320398	0.28787974	0.009	0.013	1
Gm20234	0.33253744	0.86524207	0.014	0.01	1
Kdm4b	0.33318444	0.73248961	0.031	0.025	1
Mettl14	0.33319879	0.37592983	0.058	0.049	1
Msh2	0.33320508	0.40519753	0.04	0.033	1
Abl1	0.33369993	0.76606623	0.056	0.049	1
1500011K16R	0.33424891	0.61117989	0.115	0.108	1
Smarca2	0.33616001	0.38584858	0.152	0.144	1
Banp	0.33663129	0.887764	0.033	0.027	1
Ttc13	0.33696503	0.56166159	0.024	0.019	1
Pde6d	0.33724939	1.02949324	0.021	0.016	1
Usp5	0.33895907	0.88265147	0.051	0.043	1
Chtop	0.33907469	0.31834736	0.208	0.204	1
Hypk	0.3401775	0.39350538	0.14	0.165	1

Cct2	0.34064484	0.32610975	0.394	0.425	1
Hars2	0.34066246	0.58982361	0.031	0.025	1
Osbpl5	0.34164418	0.77908384	0.01	0.007	1
Sf3a2	0.34215031	0.39551163	0.052	0.064	1
Paics	0.34246599	0.31714902	0.101	0.12	1
Cdkn2d	0.34249365	0.66977278	0.089	0.081	1
Abhd4	0.34341528	0.38295802	0.021	0.028	1
Ccnl2	0.34415307	0.35815284	0.291	0.3	1
Arhgap19	0.34419506	0.32301531	0.009	0.013	1
Fhl1	0.34430271	1.01225913	0.012	0.009	1
Bad	0.34431033	0.3253981	0.033	0.042	1
Eef1akmt2	0.34458246	0.81687618	0.037	0.03	1
Rae1	0.34488606	0.63188813	0.08	0.072	1
Fadd	0.34507037	0.91205012	0.014	0.01	1
Snhg20	0.34561715	0.43822605	0.056	0.048	1
Fance	0.34618436	1.33395057	0.016	0.012	1
Med1	0.3467825	0.29923499	0.127	0.148	1
Mmab	0.34757268	0.95973302	0.012	0.009	1
3110009E18R	0.34865835	0.97215791	0.01	0.007	1
Pop5	0.34905147	0.3917643	0.126	0.118	1
2610008E11R	0.34999317	0.66437445	0.035	0.029	1
Pym1	0.35009594	0.35338597	0.096	0.087	1
Lxn	0.35040286	0.27467211	0.019	0.026	1
Thoc5	0.3506168	0.71257096	0.033	0.027	1
Slc9a1	0.35071919	0.40062825	0.042	0.052	1
Rdm1	0.35075016	0.61358044	0.023	0.018	1
Pi4ka	0.35111379	0.58778527	0.089	0.081	1
Chd1	0.35120977	0.40240059	0.243	0.244	1
Bicral	0.3518731	0.6836732	0.047	0.04	1
Dpp8	0.35372774	0.47426999	0.106	0.098	1
1500011B03R	0.35417655	0.79001085	0.026	0.021	1
Bcor	0.35467427	0.54052502	0.082	0.074	1
Coq4	0.35531371	0.68095676	0.016	0.012	1
Sec23ip	0.35569044	0.34497411	0.066	0.058	1
Coq9	0.35612441	0.72843796	0.026	0.021	1
Napg	0.35654223	0.32516008	0.082	0.097	1
B3gat2	0.35707605	1.12857138	0.019	0.015	1
Ubl7	0.35749966	0.64356696	0.073	0.066	1
Kin	0.35790523	0.29381078	0.082	0.097	1
Golph3l	0.35801383	0.39669065	0.037	0.046	1
Tmem41b	0.35821732	0.30298075	0.084	0.099	1
Rhbdd1	0.35911777	0.46204297	0.019	0.026	1
Klc2	0.35927453	0.54358003	0.012	0.009	1
Ints6l	0.35943174	0.57462787	0.052	0.045	1
Hdhd2	0.35944174	0.87441936	0.028	0.022	1
Ext2	0.35981826	0.39662648	0.031	0.04	1
Ccdc77	0.36007278	0.76983649	0.017	0.013	1
Lig4	0.36130166	1.06949494	0.01	0.007	1

Stk38l	0.36136381	0.70585464	0.03	0.024	1
Kdm3b	0.36177314	0.52601452	0.124	0.117	1
Atg5	0.36212108	0.30740062	0.075	0.089	1
Gja1	0.36214598	0.28519115	0.007	0.011	1
Cep164	0.36234007	0.51632716	0.058	0.05	1
Mphosph9	0.36253815	0.87714119	0.028	0.022	1
Zcchc4	0.36310341	0.64620339	0.033	0.027	1
Thyn1	0.36389414	0.34436727	0.028	0.035	1
Psen2	0.36422225	0.67452407	0.124	0.118	1
Sfxn3	0.36486776	0.2893156	0.052	0.063	1
Fbxw7	0.36487302	0.58239703	0.086	0.078	1
Smn1	0.36502939	0.37843935	0.072	0.085	1
Unc119b	0.3654243	0.53030531	0.099	0.091	1
Ube2e2	0.36587467	1.22795342	0.01	0.007	1
Akap8	0.36622685	0.42794597	0.113	0.105	1
Atp5s	0.36834367	0.64747567	0.014	0.01	1
Gm39469	0.36877191	0.39065186	0.007	0.011	1
Nfkbil1	0.37210242	0.63703997	0.042	0.035	1
Hsd17b7	0.37283376	0.44696446	0.024	0.031	1
Ap3s1	0.37349069	0.34914058	0.185	0.213	1
Stam2	0.37368479	0.2901196	0.099	0.117	1
Slc2a9	0.37453753	1.05185306	0.014	0.01	1
Mut	0.37456582	0.39691946	0.019	0.025	1
Ube2t	0.37531254	0.62119421	0.028	0.023	1
Parvg	0.3754182	0.64632357	0.084	0.076	1
Dera	0.37582395	0.57624138	0.07	0.062	1
Rab40c	0.37592373	0.84205566	0.026	0.021	1
Sipa1l3	0.37613668	0.46408564	0.023	0.029	1
Zfat	0.37622435	0.39914097	0.007	0.011	1
Znhit3	0.3764389	0.26901039	0.026	0.033	1
Nrf1	0.37651327	0.44387707	0.089	0.081	1
Axin1	0.37727887	0.5459756	0.084	0.076	1
Pex12	0.37810401	0.63610419	0.014	0.01	1
Mcph1	0.3781225	0.8375512	0.033	0.027	1
Lrch1	0.37814298	0.45372563	0.077	0.069	1
Papola	0.37873255	0.42711599	0.283	0.292	1
Ogdh	0.37898496	0.33795892	0.187	0.183	1
Zfp707	0.37967155	0.84177988	0.019	0.015	1
Chchd6	0.37990681	0.54419037	0.01	0.015	1
Mthfs	0.38084174	0.35616693	0.058	0.069	1
Tbl2	0.38101926	0.90330205	0.019	0.015	1
2610044O15R	0.38114409	0.6625155	0.017	0.013	1
Kdm3a	0.38118988	0.65868059	0.108	0.102	1
Ddx23	0.38124477	0.43465392	0.159	0.153	1
Rpl9-ps6	0.38132757	0.33212177	0.049	0.059	1
Ncoa1	0.38288948	0.47155698	0.086	0.078	1
Ints13	0.3829264	0.58767529	0.072	0.064	1
Pgam5	0.38386175	0.72801744	0.045	0.039	1

Med13l	0.38389682	0.39789019	0.25	0.256	1
E130208F15Ri	0.3841138	0.9717777	0.017	0.013	1
Slc24a5	0.38453035	0.35979441	0.012	0.017	1
Hps4	0.38469241	0.38147548	0.052	0.063	1
Apeh	0.3850448	0.6325159	0.044	0.037	1
Fkrp	0.38592851	0.42399015	0.024	0.031	1
Ogfod3	0.38616864	0.25460696	0.01	0.015	1
Ric8b	0.38649254	0.78589075	0.024	0.02	1
Man2c1	0.38652003	0.77521437	0.028	0.023	1
Armt1	0.38686205	0.51661651	0.033	0.027	1
Snx13	0.3869112	0.93660892	0.063	0.056	1
Cep19	0.38705858	0.70108512	0.012	0.009	1
Dazap1	0.38816192	0.44353015	0.173	0.169	1
Ubr5	0.38820047	0.312814	0.222	0.223	1
Mettl7a1	0.3888035	0.94026052	0.019	0.015	1
Zmym3	0.38884386	0.74721422	0.021	0.016	1
Brpf3	0.39033871	0.68181481	0.026	0.021	1
Psmc2	0.3906166	0.33278274	0.108	0.126	1
Slc9a6	0.39155962	0.47277663	0.019	0.015	1
Kremen1	0.39195822	0.96552534	0.028	0.023	1
Pygb	0.39299064	0.69951879	0.04	0.034	1
Cyb5d2	0.39334601	0.61234602	0.021	0.016	1
Slfn9	0.3934214	0.61994711	0.016	0.012	1
Pggt1b	0.39377761	0.87412221	0.033	0.028	1
Wdr20	0.39421613	0.57741238	0.094	0.088	1
Arid1b	0.39424042	0.49506318	0.143	0.14	1
Pigp	0.39431247	0.63408891	0.079	0.072	1
Smpd4	0.3943461	0.33700721	0.024	0.031	1
Vezt	0.39463775	0.52178747	0.023	0.018	1
Rrp9	0.39482771	0.41897167	0.042	0.036	1
Iqcb1	0.39488075	0.59308297	0.037	0.031	1
Yeats2	0.39531363	0.81222867	0.047	0.041	1
Kansl1l	0.39551185	0.58631364	0.04	0.034	1
Ybey	0.39652084	0.69100398	0.014	0.01	1
Nsd2	0.396673	0.47318946	0.124	0.118	1
Strip1	0.39671368	0.34098864	0.052	0.062	1
Plpp2	0.39689107	0.64897422	0.007	0.011	1
Taf4	0.39698208	0.47813097	0.047	0.041	1
Cdk16	0.39729001	0.51713589	0.037	0.031	1
Smc3	0.39759334	0.34080229	0.22	0.22	1
Coro7	0.39763945	0.64232278	0.084	0.077	1
Polm	0.39790168	0.69020466	0.021	0.016	1
Tab1	0.39828207	0.88122668	0.01	0.007	1
Rassf2	0.39874226	0.30668189	0.112	0.13	1
Ercc6l2	0.3997499	0.65390864	0.044	0.038	1
Cep135	0.39980575	0.71131645	0.079	0.072	1
Gbp4	0.40029611	0.34067756	0.131	0.122	1
Gm27010	0.40062502	0.61600053	0.014	0.01	1

Tysnd1	0.40158503	1.20938619	0.021	0.017	1
Bnip3l	0.4018626	0.34074852	0.305	0.317	1
Lmnb2	0.40203139	0.75796966	0.012	0.009	1
Ccar2	0.40255004	0.45371096	0.017	0.013	1
Slx4	0.40281833	0.34365271	0.019	0.015	1
Bag3	0.40321073	0.69343608	0.061	0.055	1
Thumpd1	0.40332062	0.46965222	0.094	0.087	1
Ifit3	0.4033432	0.78743515	0.021	0.017	1
Cd3eap	0.40343828	0.53882205	0.028	0.023	1
Hspa14	0.40404041	0.45249225	0.133	0.127	1
Dcp1a	0.40408827	0.34255405	0.051	0.06	1
Pex2	0.40449544	0.54486993	0.044	0.038	1
Tbl3	0.40455326	0.29186111	0.019	0.025	1
Oma1	0.40488966	1.02389026	0.019	0.015	1
Xpo6	0.40520703	0.50453487	0.129	0.123	1
Fam206a	0.40526493	0.72503461	0.024	0.02	1
Rmnd5b	0.40530602	0.48305127	0.04	0.034	1
A330069E16R	0.40605536	0.5471298	0.012	0.009	1
Zfp689	0.40660123	0.8894251	0.01	0.007	1
Dcaf6	0.40720222	0.56229995	0.024	0.02	1
Bcl9l	0.40782146	0.53013004	0.035	0.029	1
D5Ertd579e	0.40799784	0.30206592	0.068	0.08	1
Ubxn2b	0.40815705	1.27532848	0.01	0.008	1
Zfp275	0.40821595	0.39758062	0.028	0.023	1
Mmgt1	0.40922216	0.88248002	0.037	0.031	1
Gm46430	0.40946854	0.84790523	0.01	0.008	1
Paxx	0.40973091	0.31286748	0.047	0.056	1
Gcat	0.40975545	1.1405464	0.014	0.011	1
Cdkn2aipnl	0.40980076	0.47383831	0.058	0.051	1
Phtf1os	0.41015275	0.50582255	0.01	0.007	1
Rabif	0.41036117	0.38017931	0.042	0.05	1
Axin2	0.41041129	0.36927341	0.042	0.036	1
Ppp5c	0.41252841	0.34907261	0.035	0.043	1
Cpox	0.41269194	0.47475205	0.049	0.043	1
Krt10	0.41286514	0.70549495	0.014	0.011	1
Zfp865	0.41296927	0.62602092	0.045	0.039	1
Tbc1d14	0.41317933	0.63704813	0.061	0.055	1
Ccdc171	0.41363105	0.64935779	0.017	0.014	1
Ncor2	0.41407752	0.59939427	0.082	0.075	1
Sssca1	0.41444562	0.4676012	0.051	0.044	1
Ap4m1	0.41453736	0.42195944	0.03	0.037	1
Prpf8	0.41491388	0.25259993	0.182	0.208	1
Vrk2	0.4154135	0.28112389	0.054	0.064	1
Ccny	0.41559613	0.63612559	0.11	0.104	1
Cog8	0.41566007	0.46545662	0.049	0.042	1
Usp11	0.41584739	0.94917001	0.016	0.012	1
Ercc6	0.41624026	0.70349551	0.063	0.056	1
Lancl1	0.41657462	0.69389438	0.037	0.031	1

Nudt6	0.41669413	0.45499671	0.012	0.009	1
Zrsr1	0.41678788	0.46433728	0.054	0.047	1
Zfp287	0.41680922	1.222794	0.014	0.011	1
Riox1	0.41693708	0.68879161	0.03	0.025	1
Rttn	0.41712933	0.31682739	0.007	0.01	1
Ldlrap1	0.4171855	0.72085692	0.051	0.045	1
Mrps2	0.41795178	0.73962178	0.021	0.017	1
Prnp	0.41832345	0.8421494	0.037	0.031	1
Dars	0.41838771	0.63436729	0.092	0.086	1
Pigyl	0.41854351	0.31059669	0.042	0.05	1
Gadd45gip1	0.41875586	0.49737355	0.15	0.146	1
Nvl	0.41929947	0.50022172	0.075	0.068	1
Lias	0.42029836	0.501519	0.068	0.061	1
Tnfrsf10b	0.42030021	0.38314193	0.031	0.026	1
Cdc7	0.42172581	0.67749664	0.023	0.018	1
Poc5	0.42200209	0.53312399	0.019	0.025	1
Ttc33	0.42219558	0.50834243	0.063	0.056	1
Dock11	0.42245379	0.29222648	0.157	0.152	1
Lonp2	0.42268648	0.51324922	0.077	0.07	1
Lockd	0.42303784	0.38752944	0.047	0.041	1
Gm4673	0.42324787	1.07820949	0.023	0.018	1
Gpr137	0.42348887	0.4781837	0.019	0.025	1
Mmgt2	0.42366271	0.67631467	0.021	0.027	1
Col4a3bp	0.42400418	0.3335114	0.169	0.164	1
Ppm1k	0.42432556	0.43730383	0.051	0.044	1
Shoc2	0.4244929	0.4374518	0.134	0.13	1
Mroh1	0.42550344	0.27348653	0.035	0.042	1
Snrpa1	0.42574906	0.39816563	0.12	0.14	1
Eno3	0.42601909	1.11736417	0.007	0.01	1
Amd2	0.4260991	0.28166422	0.01	0.015	1
Faap20	0.42635234	0.652084	0.052	0.046	1
Dguok	0.42644371	0.6141804	0.092	0.086	1
Usp19	0.42721686	0.40670539	0.194	0.192	1
Msmo1	0.4276938	0.54109054	0.12	0.115	1
Anapc13	0.42779738	0.36673799	0.157	0.183	1
Trove2	0.42785623	1.23060007	0.021	0.017	1
2610037D02R	0.42846474	0.42632201	0.07	0.063	1
Abhd6	0.42852033	0.86627516	0.014	0.011	1
Ddx59	0.42902123	1.06443882	0.01	0.008	1
Msh6	0.42933344	0.40546997	0.028	0.023	1
Rfx3	0.42998172	0.39649655	0.028	0.023	1
2410131K14R	0.43086647	0.42821941	0.019	0.025	1
Zfp219	0.43098085	1.07214922	0.028	0.023	1
Dapp1	0.4310508	0.39123599	0.195	0.193	1
Abtb2	0.43153158	0.65831567	0.066	0.06	1
Tmtc3	0.43156466	1.37012272	0.01	0.008	1
Oard1	0.43171873	0.51984287	0.075	0.068	1
Mir17hg	0.43194472	0.42343859	0.023	0.018	1

Fam3c	0.43239565	0.46431681	0.087	0.08	1
Taf1	0.43335047	0.27424221	0.14	0.16	1
Gm16973	0.4335521	0.56480638	0.017	0.014	1
Tmem120b	0.43356676	0.51762254	0.009	0.012	1
Mr1	0.4339013	1.052034	0.01	0.008	1
Zfp414	0.43611909	0.68466175	0.026	0.022	1
Afg3l2	0.43612904	0.49089405	0.058	0.051	1
Srprb	0.43679205	0.26720769	0.051	0.06	1
Fam162a	0.43681347	0.54118579	0.098	0.092	1
Utp20	0.43747082	0.4942489	0.044	0.038	1
Taf6	0.43775719	1.05842157	0.038	0.033	1
Caml	0.43784173	0.32412483	0.03	0.036	1
Nbr1	0.43847667	0.58414701	0.096	0.09	1
Mapk9	0.43888018	0.32273029	0.044	0.052	1
Phax	0.43913137	0.38760528	0.092	0.107	1
Usp42	0.43947622	0.76063503	0.049	0.043	1
Xpo7	0.4397934	0.58412075	0.072	0.065	1
Fam213b	0.44088266	0.61894343	0.035	0.042	1
Parp9	0.4422649	0.43968647	0.07	0.063	1
Elac1	0.44332681	0.30158135	0.01	0.014	1
Papolg	0.4437217	0.48174315	0.058	0.051	1
Nf2	0.44510582	0.67685577	0.042	0.036	1
Gtf2a1	0.44513649	0.30922652	0.124	0.142	1
Kctd2	0.44624311	0.40749016	0.045	0.039	1
Cops7b	0.446977	0.88618651	0.024	0.02	1
Fam104a	0.44756159	0.31708935	0.274	0.282	1
Twf2	0.44762736	0.7569611	0.094	0.09	1
Gm28809	0.45030277	0.30456525	0.012	0.009	1
Kat14	0.45070236	1.06228781	0.024	0.02	1
Pcgf3	0.45080204	0.38732571	0.028	0.034	1
Ganab	0.45100423	0.27212574	0.084	0.096	1
Gramd1c	0.45148772	0.31727519	0.01	0.008	1
Pdp1	0.45161259	0.28729849	0.021	0.026	1
B3gat3	0.4516882	0.54504532	0.035	0.042	1
Pdha1	0.45179268	0.5812394	0.148	0.146	1
Sec22a	0.45188986	0.69213697	0.031	0.027	1
Mbd5	0.45198152	0.74835798	0.037	0.032	1
Mbnl3	0.45337524	0.4514439	0.051	0.045	1
Srsf4	0.45362797	0.45587681	0.101	0.095	1
Slc12a7	0.45441312	0.75095392	0.033	0.028	1
Pcnx3	0.45441445	0.54277493	0.038	0.033	1
Spata5	0.45441827	0.29573209	0.044	0.052	1
Fermt3	0.45514918	0.25769867	0.176	0.202	1
Ccdc130	0.45517353	0.72880289	0.021	0.017	1
Fubp3	0.45631375	0.58751772	0.031	0.027	1
F8a	0.45637245	0.69544891	0.03	0.025	1
Taf1c	0.45658062	0.70751109	0.026	0.022	1
Pigk	0.45736662	0.89535473	0.066	0.061	1

Pcolce	0.45767112	0.73341052	0.012	0.009	1
Wdr55	0.4578363	0.63808521	0.026	0.022	1
Pex19	0.45796834	0.56690361	0.051	0.045	1
Btbd2	0.45804149	0.7132447	0.016	0.012	1
Hikeshi	0.45889875	0.30657208	0.063	0.073	1
Rps6kb2	0.45958864	0.62785279	0.051	0.045	1
Dhx36	0.45988997	0.46786725	0.23	0.239	1
Ctso	0.46018888	0.43368825	0.089	0.083	1
Rdh11	0.46104485	0.37360304	0.052	0.046	1
Rnf103	0.46179866	0.59556062	0.063	0.057	1
Rbm45	0.46267575	0.39384816	0.024	0.02	1
Scyl3	0.46280685	0.34357686	0.054	0.063	1
Zkscan17	0.46303858	0.29887248	0.024	0.03	1
Zdhhc17	0.46303969	0.31376233	0.051	0.045	1
Stk3	0.4632309	0.67688693	0.026	0.032	1
Tsfm	0.46477058	0.60958029	0.031	0.027	1
Smpd2	0.46524743	0.8384578	0.016	0.012	1
Tfdp1	0.46538147	0.83563943	0.035	0.03	1
Lin52	0.46607597	0.77412082	0.024	0.02	1
Ssbp1	0.46651425	0.38824136	0.138	0.133	1
Naf1	0.46684015	0.53624196	0.035	0.042	1
Lrif1	0.46801872	0.39812548	0.098	0.091	1
Cinp	0.46951268	0.34628431	0.021	0.026	1
Smarcal1	0.46993895	0.90241993	0.021	0.017	1
Elk1	0.47039331	0.27550139	0.017	0.022	1
Aste1	0.47054092	0.7451121	0.019	0.016	1
Mto1	0.47056046	0.51035554	0.021	0.017	1
C2cd2	0.47087762	0.26519047	0.01	0.014	1
Churc1	0.47154865	0.37559199	0.211	0.214	1
Zzef1	0.47270797	0.46021056	0.122	0.118	1
Mx1	0.47272228	0.4831077	0.023	0.019	1
Evi5l	0.47341032	0.50149078	0.007	0.01	1
Ndufaf4	0.47430025	0.29679489	0.07	0.079	1
Rcan3	0.47474548	0.73917251	0.021	0.017	1
Tmem260	0.4753326	0.39249714	0.037	0.032	1
Smurf1	0.47541746	0.77109118	0.045	0.04	1
9330175E14R	0.47603323	0.49862776	0.012	0.009	1
Sec16a	0.47708014	0.34585137	0.084	0.078	1
Ubash3b	0.47716939	0.63539425	0.101	0.097	1
Ap4b1	0.47720403	0.44124106	0.014	0.011	1
Ddx56	0.47857805	0.30398091	0.031	0.038	1
Tceanc2	0.47935908	0.9393979	0.037	0.032	1
Tvp23b	0.48074722	0.27793789	0.035	0.041	1
1500015A07R	0.48098549	0.3367997	0.061	0.055	1
Tmem158	0.48103388	0.6776113	0.014	0.011	1
Atat1	0.48109729	1.1921289	0.017	0.014	1
Ap3m2	0.48171017	0.48118681	0.028	0.024	1
Pbx3	0.48213979	1.03419921	0.021	0.017	1

Mthfsd	0.48282882	0.62159734	0.012	0.016	1
Klhl6	0.48326421	0.33584213	0.099	0.093	1
Zdhhc3	0.48404449	0.25532107	0.091	0.104	1
Lyrm4	0.48496029	0.61137647	0.024	0.02	1
4632415L05Ri	0.48512687	0.78713118	0.016	0.013	1
Pink1	0.48542015	0.28402877	0.066	0.076	1
Cnot11	0.48569722	0.65795964	0.065	0.059	1
Paxip1	0.48579721	0.34762944	0.035	0.041	1
Fez2	0.4861141	0.55685142	0.023	0.028	1
Csad	0.48652152	0.92046546	0.017	0.014	1
Tmem60	0.48697802	0.32043424	0.075	0.069	1
Slc39a10	0.48842206	1.0322936	0.031	0.027	1
Zcchc8	0.48871681	0.42452897	0.068	0.063	1
Rnf31	0.48977872	0.54119712	0.054	0.049	1
Hivep3	0.48993502	0.31016931	0.058	0.067	1
Ints14	0.49132053	0.67871483	0.04	0.035	1
Mypopos	0.49154029	0.33358794	0.01	0.008	1
Pld2	0.49167574	0.4211	0.007	0.01	1
Zfp942	0.49386564	0.4428805	0.056	0.05	1
Fam118b	0.49411405	0.73954423	0.017	0.014	1
Mapre3	0.49413112	1.08435241	0.019	0.016	1
Ddi2	0.49535912	0.33894239	0.066	0.076	1
Daxx	0.49538585	0.49164244	0.061	0.056	1
Smug1	0.4953983	0.64575283	0.023	0.019	1
Pvt1	0.49559834	0.61111479	0.037	0.032	1
Pglyrp2	0.49632932	0.31669222	0.009	0.012	1
Sp2	0.49637509	0.27332854	0.07	0.08	1
Snx27	0.49654829	0.34961099	0.031	0.037	1
Ints3	0.4967982	0.58081911	0.037	0.032	1
Tmem164	0.49708191	0.27584006	0.042	0.037	1
Kmt5c	0.49785356	0.82812528	0.023	0.019	1
Apool	0.49810832	0.62730552	0.038	0.034	1
Edem3	0.49855771	0.3930093	0.047	0.042	1
Prrg2	0.49887518	0.68905395	0.012	0.016	1
Scnm1	0.49895916	0.56223662	0.035	0.03	1
Def6	0.4989885	0.61294834	0.059	0.054	1
Cbx5	0.5000832	0.25189093	0.066	0.06	1
Smg5	0.5001913	0.42280029	0.108	0.103	1
Fhl3	0.50028385	0.25980969	0.01	0.014	1
Spidr	0.50079177	0.55500799	0.028	0.024	1
Wipf2	0.50080062	0.27434626	0.119	0.135	1
Washc3	0.50092107	0.40211043	0.075	0.086	1
Ppp2r3d	0.50153345	0.44420959	0.035	0.041	1
1700056E22R	0.50246219	0.9494526	0.019	0.016	1
Phf23	0.50286042	0.29385317	0.068	0.078	1
Plagl2	0.50296924	0.47660588	0.08	0.075	1
Foxn2	0.50439924	0.57330046	0.124	0.122	1
Wrn	0.50459761	0.56373526	0.068	0.063	1

Atic	0.50462225	0.70629339	0.072	0.067	1
Dnajc18	0.50463815	0.29163955	0.021	0.026	1
U2surp	0.50596625	0.4180418	0.26	0.271	1
Rps6kc1	0.50600432	0.76032479	0.014	0.011	1
Lipo3	0.50606	0.64378611	0.035	0.03	1
Eif5b	0.50606588	0.25126818	0.366	0.389	1
Mfsd4a	0.50651369	0.32357264	0.054	0.049	1
Kat2b	0.50662104	0.3861832	0.072	0.066	1
Hspb1	0.50728153	0.88609255	0.019	0.016	1
Ndufv2	0.50853709	0.36663485	0.169	0.195	1
Slc12a9	0.50899768	0.49842882	0.017	0.022	1
Sclt1	0.5090483	0.48958754	0.045	0.04	1
Zbtb24	0.50981458	0.31840286	0.038	0.045	1
Pan2	0.50982738	0.57677626	0.04	0.035	1
Dhfr	0.51001937	0.38351435	0.012	0.016	1
Mtap	0.51003851	0.68732621	0.033	0.029	1
Heatr1	0.51041008	0.5355215	0.065	0.06	1
Xrn2	0.51046543	0.26563114	0.293	0.311	1
Cpped1	0.51087879	0.59457823	0.019	0.016	1
2610524H06R	0.51089558	0.80470014	0.045	0.041	1
Ints8	0.51141019	0.70745568	0.042	0.037	1
Ap3d1	0.51177239	0.27758839	0.099	0.113	1
Pfkl	0.51186255	0.44373833	0.086	0.08	1
Ccpg1	0.51234105	0.26288117	0.051	0.058	1
Slc25a13	0.51267102	0.4149269	0.009	0.012	1
2310033P09R	0.51282631	0.39445249	0.096	0.091	1
Ola1	0.51335639	0.54598382	0.157	0.157	1
Retreg2	0.5135909	0.48969773	0.084	0.079	1
Usp7	0.51431074	0.34388501	0.232	0.269	1
Slc4a1ap	0.5155551	0.46679126	0.101	0.096	1
Arglu1	0.51569828	0.26633121	0.293	0.307	1
Itfg2	0.51576861	0.31485499	0.035	0.03	1
Wdr6	0.51612057	0.4941165	0.019	0.016	1
Suox	0.51613499	0.31000244	0.01	0.014	1
Arhgap5	0.51714308	0.79639371	0.079	0.075	1
Bicra	0.51746641	0.47398525	0.037	0.032	1
Impa1	0.51868561	0.4366478	0.12	0.116	1
Clip2	0.51869833	1.1070912	0.023	0.019	1
Polr3e	0.51931216	0.36011656	0.021	0.025	1
Xiap	0.5202645	0.46205377	0.239	0.249	1
1810024B03R	0.52030305	0.87287226	0.014	0.011	1
Gm340	0.52068084	1.30806699	0.021	0.018	1
Sos1	0.52076401	0.34740029	0.059	0.054	1
Rxrb	0.5207664	0.3262009	0.054	0.062	1
Nkrf	0.52140875	0.76865158	0.019	0.016	1
Ints11	0.52166855	0.66957625	0.038	0.034	1
Trak2	0.52173828	0.4001223	0.08	0.075	1
Clock	0.52268331	0.25258637	0.082	0.092	1

Rbm38	0.52283968	0.47442339	0.065	0.059	1
Ankhd1	0.52297003	0.55631382	0.136	0.135	1
1600002K03R	0.52387462	0.32822155	0.045	0.052	1
Mrpl44	0.52400904	0.48692292	0.045	0.041	1
Atf2	0.5240492	0.38026974	0.143	0.139	1
Epc2	0.52667131	0.27906153	0.159	0.178	1
Ssx2ip	0.52682897	0.48184945	0.016	0.02	1
Zfp143	0.52684084	0.74267069	0.033	0.029	1
Timm22	0.52820734	0.53469169	0.056	0.064	1
Tagap1	0.52831462	0.38136737	0.028	0.024	1
Arhgap25	0.52881979	0.55104913	0.086	0.081	1
Cpsf1	0.52916434	0.60896479	0.047	0.042	1
Kif18a	0.53018115	0.3757612	0.017	0.014	1
Ccdc97	0.53063186	0.27732646	0.042	0.048	1
C1qtnf12	0.53138461	0.77838883	0.014	0.018	1
Rpa3	0.53150883	0.28027839	0.059	0.054	1
Znhit6	0.53264254	0.58964711	0.045	0.041	1
Ak3	0.53300272	0.29703066	0.03	0.035	1
Crim1	0.53324864	0.41791829	0.045	0.052	1
Ubp1	0.53418317	0.44403758	0.12	0.117	1
Spop	0.53425675	0.32310255	0.19	0.218	1
Nhlrc2	0.53453361	0.34511446	0.042	0.048	1
Commd6	0.53470713	0.43755764	0.127	0.125	1
Zfp809	0.53485989	0.41022059	0.028	0.033	1
2200002D01R	0.53500914	1.49955181	0.01	0.008	1
Lrrc40	0.53522423	0.30846044	0.023	0.019	1
Sugp1	0.53556116	0.378752	0.065	0.06	1
Mrps18b	0.53580473	0.30720095	0.049	0.056	1
Tmem11	0.53822741	0.26006271	0.089	0.1	1
Ndst1	0.53859886	0.53189452	0.044	0.039	1
Ppil3	0.53870073	0.2648408	0.047	0.054	1
Abhd13	0.53918034	0.44586935	0.049	0.044	1
Nek1	0.53921909	0.78537388	0.017	0.014	1
Slc38a6	0.53955657	0.32361106	0.023	0.027	1
Wdr83	0.53981052	0.50460301	0.019	0.023	1
Atad5	0.54035764	0.64693354	0.033	0.029	1
Polr2e	0.54306753	0.34387692	0.133	0.15	1
Wrap53	0.54311267	0.59232813	0.014	0.011	1
Rnf44	0.54334473	0.416906	0.099	0.112	1
2210016L21Ri	0.54366369	0.67627689	0.072	0.067	1
0610030E20R	0.54482571	0.64493256	0.091	0.087	1
Hist2h2bb	0.54574208	0.39062265	0.028	0.024	1
Haus1	0.54643651	0.47352122	0.009	0.011	1
Gpr180	0.54650483	1.03459348	0.019	0.016	1
Zfp628	0.54681285	0.30941087	0.021	0.025	1
Exoc2	0.54694574	0.47464461	0.082	0.077	1
Ppp2r5d	0.54725016	0.36677697	0.031	0.036	1
Htt	0.54725822	0.47018488	0.108	0.104	1

Mthfsl	0.54779062	0.30087077	0.08	0.09	1
Ddx17	0.548663	0.28859495	0.321	0.343	1
Nup93	0.54915097	0.57389454	0.028	0.024	1
Dbr1	0.55000214	0.65064861	0.049	0.044	1
Aasdh	0.55048027	0.98675837	0.016	0.013	1
Eps15	0.55104267	0.27498137	0.143	0.161	1
Etaa1os	0.5510536	0.42041568	0.01	0.008	1
Zfp236	0.5516072	0.54054025	0.054	0.05	1
Psmg2	0.55178612	0.59702229	0.044	0.039	1
Psmd5	0.5519841	0.31908746	0.038	0.034	1
Leng9	0.55212578	0.45068663	0.035	0.031	1
Mrps9	0.55338643	0.27262648	0.094	0.105	1
Larp1b	0.55368311	0.46076999	0.091	0.102	1
Dgkh	0.55531889	0.52719919	0.047	0.043	1
Zswim8	0.55676609	0.47459964	0.112	0.109	1
Baz1a	0.55745174	0.35881443	0.314	0.337	1
Cry2	0.55818434	0.31066461	0.026	0.023	1
Dus1l	0.55824596	0.70566853	0.033	0.029	1
Dlat	0.55837748	0.59109714	0.045	0.041	1
Polr3c	0.5584357	0.31368281	0.073	0.082	1
Bicd2	0.55869734	0.52380693	0.051	0.058	1
Amn1	0.55895737	0.29417097	0.112	0.107	1
Fdft1	0.55908217	0.41729912	0.028	0.033	1
Uba7	0.55935282	0.49902138	0.052	0.048	1
Aamdc	0.55948953	0.76095747	0.028	0.025	1
Kdsr	0.55991987	0.53952085	0.058	0.053	1
Papd5	0.56076692	0.44229322	0.086	0.081	1
Ssbp3	0.56103958	0.42382164	0.115	0.111	1
Caprin1	0.56175695	0.346691	0.269	0.283	1
Rnpepl1	0.56187313	0.50085677	0.065	0.06	1
Zfp668	0.56188811	0.90910231	0.014	0.011	1
Tmem131l	0.56199942	0.2895285	0.105	0.117	1
D830025C05F	0.56283687	0.70605273	0.028	0.024	1
Fchsd1	0.5631312	0.32069585	0.014	0.011	1
Cnot1	0.56365484	0.34741511	0.237	0.247	1
Mrpl22	0.56420105	0.54736442	0.04	0.036	1
Kbtbd7	0.56483308	0.40047595	0.012	0.01	1
Nup98	0.56506005	0.30452393	0.363	0.392	1
Irf3	0.56537245	0.45497127	0.086	0.081	1
Tbpl1	0.56600649	0.29775332	0.077	0.086	1
Acot11	0.56636151	0.71114249	0.012	0.01	1
1810044D09R	0.56650555	0.32867975	0.014	0.017	1
Saysd1	0.5668504	0.50318275	0.028	0.033	1
Stat2	0.5670313	0.71425769	0.086	0.083	1
Sugp2	0.56717697	0.28680834	0.035	0.04	1
Arv1	0.56731206	0.35703582	0.01	0.008	1
Rdh14	0.56828428	0.80159071	0.03	0.026	1
Tmem39b	0.56872245	0.69596737	0.019	0.016	1

Bnip1	0.56934699	0.7426958	0.035	0.031	1
Zdhhc9	0.56955791	0.3271457	0.024	0.029	1
Dynlt3	0.56980165	0.53688682	0.134	0.135	1
Aptx	0.57044212	0.46371084	0.028	0.033	1
Vps16	0.57056328	0.43972214	0.052	0.06	1
Def8	0.57109994	0.89216254	0.01	0.008	1
Otud3	0.57120097	0.48553564	0.012	0.015	1
Arid3b	0.571836	0.31057954	0.028	0.032	1
Zbtb1	0.57278843	0.25721074	0.141	0.138	1
Aven	0.57290093	0.25050486	0.04	0.046	1
Men1	0.57333104	0.53207849	0.016	0.013	1
Dnaaf5	0.57492165	0.51245099	0.012	0.01	1
Zswim1	0.57501895	0.67590905	0.016	0.013	1
Nsun3	0.57537968	0.3475209	0.024	0.029	1
Slc25a40	0.57580598	0.57127304	0.045	0.041	1
Heatr5b	0.57607476	0.3618477	0.03	0.026	1
Acbd6	0.57615007	0.25733542	0.117	0.13	1
Pitpnb	0.57673427	0.30349313	0.087	0.098	1
Sdad1	0.57878147	0.38193599	0.058	0.065	1
Drg1	0.57902594	0.49723912	0.087	0.084	1
Panx1	0.579236	0.33234334	0.058	0.064	1
Ccnc	0.57974114	0.37138115	0.054	0.061	1
Nol10	0.57995556	0.49157676	0.077	0.086	1
Mfsd6	0.58014287	0.31137774	0.157	0.155	1
Kif1bp	0.58050242	0.45794498	0.023	0.027	1
Golga4	0.5810865	0.43106375	0.222	0.229	1
G2e3	0.58258191	0.31093778	0.063	0.058	1
Zbtb7b	0.58337064	0.35727554	0.028	0.032	1
Echdc1	0.58338566	0.44551829	0.014	0.017	1
Arhgap10	0.5834066	0.38128077	0.035	0.04	1
Btf3l4	0.58341971	0.68091099	0.028	0.025	1
Ncoa6	0.58343928	0.40943341	0.079	0.075	1
Nol6	0.5841763	0.5205086	0.038	0.035	1
Smurf2	0.58425435	0.34890872	0.063	0.07	1
2310057M21F	0.58435084	0.54135388	0.028	0.025	1
Tmem29	0.58484418	0.2712938	0.047	0.053	1
Chuk	0.58575937	0.31045066	0.133	0.13	1
Btbd6	0.58602336	0.55843606	0.01	0.008	1
Zcchc18	0.58654569	0.53871563	0.017	0.015	1
Zfp235	0.58755911	0.38879601	0.01	0.008	1
Ppil4	0.58777602	0.34780143	0.159	0.157	1
Tpk1	0.58903555	0.4925798	0.009	0.011	1
Atxn10	0.58940326	0.68486381	0.103	0.101	1
Trit1	0.58955119	0.58662013	0.021	0.025	1
Foxred1	0.59007233	0.29593167	0.021	0.025	1
Gm26799	0.59122015	0.3824615	0.009	0.011	1
Hira	0.5916867	0.40213523	0.035	0.031	1
Pick1	0.59175126	0.40191316	0.021	0.018	1

Dopey2	0.59224455	0.65216309	0.049	0.045	1
Clec16a	0.59283192	0.41611011	0.014	0.017	1
Exd2	0.5931253	0.72398002	0.019	0.016	1
Nemp1	0.59350551	0.91867729	0.024	0.021	1
Zscan2	0.59383552	0.49153535	0.014	0.012	1
Mtif3	0.59446313	0.48670711	0.023	0.027	1
Pdik1l	0.59490465	0.58175992	0.028	0.025	1
Twnk	0.59524601	0.39115667	0.019	0.016	1
Fam219a	0.59524772	0.99007373	0.009	0.011	1
Ercc8	0.59529495	0.62610994	0.024	0.021	1
Slc25a17	0.59539773	0.53536841	0.047	0.053	1
Gzf1	0.59541329	0.55573939	0.045	0.042	1
Rft1	0.59679763	0.49411813	0.017	0.015	1
Cyb561d1	0.59741783	0.82628535	0.028	0.025	1
Ppa1	0.59776494	0.77826399	0.08	0.077	1
Kansl3	0.59784838	0.32769852	0.136	0.134	1
Pibf1	0.59841084	0.35128104	0.059	0.055	1
Nfic	0.59854072	0.6097862	0.038	0.035	1
Usp2	0.59960521	0.63665444	0.014	0.017	1
Elac2	0.60005456	0.9699541	0.031	0.028	1
Asnsd1	0.60011736	0.70505719	0.079	0.075	1
Cep70	0.60028283	0.4178811	0.01	0.013	1
Ippk	0.60043774	0.51296864	0.012	0.01	1
Rab14	0.60062611	0.36361502	0.368	0.4	1
1810043G02R	0.60202337	0.41007104	0.016	0.019	1
Ykt6	0.60204741	0.45515733	0.091	0.101	1
Ttc4	0.60226754	0.51789731	0.056	0.052	1
Eif2b4	0.60246236	0.61219229	0.047	0.044	1
Dnajc7	0.60375806	0.41470581	0.188	0.192	1
Glcci1	0.6052196	0.42445374	0.023	0.026	1
Ldah	0.60532407	0.25647332	0.042	0.047	1
Jmy	0.6054455	0.35700326	0.159	0.157	1
Rexo1	0.60611706	0.28941397	0.129	0.144	1
Mfap1b	0.60680006	0.28345243	0.075	0.071	1
Pgm3	0.60741965	0.8387657	0.028	0.025	1
Dbt	0.60748602	0.3204822	0.04	0.036	1
Klhl9	0.60799481	0.3147695	0.082	0.092	1
Ccdc85b	0.60860844	0.57988577	0.01	0.009	1
Ptcd1	0.60868488	0.90540153	0.019	0.017	1
Erf	0.60870927	0.50828627	0.035	0.032	1
Ckap5	0.6092451	0.57134797	0.091	0.088	1
Sumo3	0.60928199	0.38172823	0.094	0.106	1
Ahsa2	0.60986658	0.66152559	0.051	0.047	1
Bola3	0.60998055	0.36815634	0.147	0.147	1
Trmt10b	0.6105866	1.31034768	0.012	0.01	1
Rrm1	0.61069891	0.25901692	0.052	0.058	1
Rhno1	0.61074023	0.56969624	0.051	0.047	1
Rnpc3	0.61162712	0.29841682	0.091	0.101	1

Fbxl17	0.61171525	0.38669769	0.033	0.038	1
Bsdc1	0.61178854	0.44815023	0.11	0.107	1
Agap3	0.61242675	0.48758404	0.044	0.04	1
Ipo11	0.61256508	0.89596632	0.024	0.022	1
Arnt	0.61271941	0.41839291	0.157	0.159	1
Mon1a	0.61308379	0.5591282	0.017	0.015	1
Slc39a11	0.6131428	0.63060894	0.023	0.02	1
Tmco6	0.61337853	0.36710376	0.021	0.018	1
Gm43848	0.61409134	0.27457386	0.024	0.021	1
Mapkap1	0.61423448	0.44436849	0.094	0.091	1
Foxk2	0.6148457	0.44964658	0.04	0.037	1
Pcnx4	0.6150141	0.26737886	0.009	0.011	1
Ccdc28b	0.61514045	0.52282968	0.023	0.02	1
Zmym1	0.61519513	0.32950843	0.031	0.036	1
Pcnx	0.61578318	0.51675689	0.072	0.068	1
Thap1	0.61601544	0.85445355	0.031	0.028	1
Usp31	0.61677302	0.81532337	0.035	0.032	1
Cept1	0.61685823	0.44169194	0.082	0.079	1
Add1	0.61686172	0.30741311	0.106	0.118	1
Smim26	0.61802722	0.30292884	0.065	0.072	1
Anks1	0.61865919	0.34366816	0.03	0.034	1
Orai2	0.61971274	0.36418166	0.054	0.05	1
Abcc5	0.61975553	0.60359118	0.033	0.03	1
Alg8	0.61979607	0.49597601	0.009	0.011	1
3110040N11R	0.62042454	0.67681693	0.033	0.03	1
Chic2	0.62044607	0.28263383	0.269	0.283	1
Rab29	0.6208474	0.63249579	0.014	0.017	1
Rab18	0.62174313	0.37927783	0.133	0.149	1
Rsrc1	0.62201084	0.62538457	0.12	0.121	1
Exoc7	0.62303321	0.27319158	0.028	0.032	1
Uba1	0.62304778	0.33273386	0.197	0.203	1
Mettl16	0.62342132	0.67708084	0.07	0.067	1
Pstk	0.62384538	0.37191086	0.04	0.037	1
Zcchc17	0.62388189	0.36256414	0.092	0.103	1
Rida	0.62390896	1.05033256	0.024	0.022	1
Usf2	0.62407573	0.28045544	0.194	0.217	1
Ercc2	0.62412771	0.80179553	0.024	0.022	1
Abcc4	0.62492946	1.03996342	0.019	0.022	1
Rab10	0.62545079	0.26770701	0.319	0.342	1
Snx21	0.62627652	0.9619252	0.01	0.013	1
Adck5	0.62633018	0.54800116	0.014	0.012	1
Wdr70	0.62642932	0.32233032	0.061	0.068	1
Tdg	0.62708803	0.34412732	0.099	0.11	1
Med22	0.62714152	0.68320987	0.021	0.018	1
Rnf126	0.62760648	0.39816034	0.044	0.04	1
Tmem70	0.62782425	0.28836269	0.042	0.047	1
Flvcr1	0.62811847	0.44122571	0.04	0.045	1
Cux1	0.62851988	0.42116639	0.199	0.207	1

Eva1b	0.62867875	0.48627626	0.086	0.095	1
Rps6ka1	0.63059178	0.51300728	0.079	0.075	1
Pkmyt1	0.63066964	0.42692083	0.01	0.013	1
Pnpla7	0.6311214	0.25312039	0.054	0.06	1
Ado	0.63160703	0.61851609	0.028	0.032	1
Scfd2	0.63187099	0.41757967	0.024	0.022	1
Clybl	0.63194175	1.08858706	0.014	0.012	1
Rbm15b	0.63323397	0.5083189	0.038	0.043	1
Zfp422	0.6333502	0.3307462	0.017	0.015	1
Ccnt2	0.63337957	0.29437885	0.194	0.198	1
Auh	0.63355391	0.33185574	0.119	0.132	1
Zfp958	0.63356968	0.53521059	0.035	0.032	1
Nrp1	0.63483599	0.81589365	0.052	0.059	1
Edrf1	0.63490285	0.34691293	0.047	0.053	1
Dglucy	0.63513343	0.80558165	0.01	0.009	1
Lpp	0.63539348	0.81890683	0.147	0.15	1
Mterf3	0.6358078	0.54316787	0.056	0.053	1
Erlec1	0.63622334	0.38917443	0.108	0.106	1
Entpd6	0.63665921	0.63752337	0.045	0.042	1
Tinf2	0.63752311	0.29492375	0.075	0.083	1
Smyd3	0.63789103	0.49570031	0.03	0.027	1
Gt(ROSA)26Sor	0.63794935	0.39451377	0.216	0.222	1
Iars2	0.63802943	0.353661	0.075	0.072	1
Tango2	0.63919984	0.50879383	0.026	0.023	1
Uspl1	0.63966618	0.28964452	0.082	0.09	1
Diexf	0.64045444	0.66060112	0.031	0.028	1
Secisbp2l	0.64120028	0.33009366	0.126	0.124	1
Ccz1	0.64126713	0.35557912	0.117	0.13	1
Pja2	0.64161997	0.35431293	0.04	0.045	1
Tyk2	0.64202148	0.44853713	0.049	0.054	1
Odf2	0.64215793	0.42349478	0.066	0.073	1
Zfyve19	0.64277763	0.73090475	0.017	0.015	1
Card11	0.64323594	0.25196077	0.075	0.082	1
Nipal1	0.64348376	0.90574075	0.012	0.01	1
Vipas39	0.64373424	0.47024371	0.03	0.034	1
Urod	0.64471144	0.46049558	0.035	0.032	1
Ube3c	0.64482415	0.3878844	0.08	0.077	1
Zdhhc12	0.64564928	0.29748292	0.01	0.013	1
Mtbp	0.64692723	1.01073441	0.017	0.015	1
Gtdc1	0.64791078	0.52630659	0.054	0.051	1
6230400D17R	0.6488798	0.27683398	0.014	0.012	1
Trappc3	0.64921383	0.43803201	0.077	0.085	1
Gdf11	0.64953626	0.83784315	0.014	0.012	1
Capn15	0.64972905	0.45260072	0.045	0.042	1
C030006K11R	0.65029288	1.09347923	0.01	0.009	1
Ssh1	0.65059913	0.43727201	0.042	0.047	1
Slc20a2	0.65095814	0.3088356	0.026	0.03	1
Pi4kb	0.65119722	0.53331417	0.021	0.024	1

Cstf3	0.6519357	0.39889989	0.07	0.078	1
Abhd10	0.65217088	0.45714539	0.045	0.042	1
Clk2	0.65269356	0.43924159	0.052	0.049	1
Stoml2	0.65276703	0.33451455	0.051	0.056	1
Dpp3	0.65290003	0.26502502	0.042	0.047	1
Cpsf4	0.65373814	0.5641186	0.038	0.035	1
Tipin	0.6538183	0.38220745	0.026	0.03	1
Mon2	0.65396083	0.4779592	0.12	0.121	1
Rbmx2	0.65403271	0.50922244	0.054	0.051	1
Pum1	0.65421013	0.28546379	0.236	0.243	1
Supt3	0.6542633	0.72535277	0.028	0.025	1
Cenpc1	0.65438921	0.57354692	0.065	0.062	1
Nup188	0.65448064	0.30939192	0.033	0.03	1
Scap	0.65453786	0.42867061	0.04	0.037	1
Golga3	0.6554658	0.41286483	0.07	0.067	1
Snapin	0.65586309	0.37875787	0.052	0.049	1
Pcbd2	0.65586445	0.51031828	0.101	0.1	1
Uckl1	0.65612195	0.39189311	0.035	0.039	1
Rbks	0.65652725	0.9990952	0.014	0.012	1
Pfkp	0.65696353	0.29735101	0.257	0.267	1
Wars2	0.65732512	0.89333639	0.014	0.012	1
Ormdl3	0.65739285	0.3945515	0.084	0.081	1
Creb1	0.65791895	0.31007246	0.162	0.163	1
Cbr4	0.65812298	0.66056996	0.014	0.012	1
Mllt1	0.6583369	0.32795585	0.019	0.017	1
Lats1	0.65939919	0.32261293	0.065	0.071	1
Mfhas1	0.65964603	0.66053723	0.052	0.058	1
Ntmt1	0.65977659	0.67533806	0.024	0.022	1
Prkce	0.66049824	0.51016003	0.058	0.054	1
Bard1	0.66049946	1.2193383	0.01	0.009	1
Mrpl41	0.66209488	0.47549206	0.103	0.102	1
Gtf2h2	0.66277825	0.59002489	0.024	0.022	1
Cox16	0.66318749	0.40760214	0.077	0.074	1
Sik3	0.66448209	0.25175853	0.175	0.193	1
Dsn1	0.66475417	1.0526273	0.01	0.009	1
Pcmtd1	0.6648784	0.29360793	0.199	0.202	1
Acad8	0.66499948	0.7127827	0.014	0.012	1
Mboat7	0.66528173	0.49939106	0.054	0.06	1
Cela1	0.66576999	0.80886118	0.014	0.012	1
Dgcr14	0.66669001	0.56894104	0.042	0.039	1
Vps9d1	0.66974615	0.26620072	0.028	0.031	1
Psmf1	0.6699487	0.40853747	0.052	0.058	1
Acp6	0.67121027	0.78948053	0.012	0.014	1
Rbck1	0.67129125	0.32653867	0.131	0.13	1
Trappc9	0.67140219	0.48703533	0.045	0.042	1
Zranb2	0.67160048	0.36387641	0.157	0.159	1
Dnajc16	0.67181652	0.60412159	0.01	0.013	1
Clns1a	0.6718997	0.47527817	0.094	0.092	1

Tbck	0.67398907	0.30780762	0.052	0.049	1
Mettl26	0.67468741	0.2577418	0.056	0.061	1
Lactb2	0.67602633	0.35811072	0.042	0.039	1
Dalrd3	0.67605377	0.61184316	0.066	0.064	1
Galnt1	0.67624951	0.27925704	0.182	0.2	1
Slf2	0.67664714	0.39567264	0.089	0.098	1
Tbc1d1	0.67717323	0.26904994	0.117	0.128	1
Ppif	0.67735006	0.34697329	0.033	0.03	1
Ctdsp2	0.67771651	0.40192982	0.147	0.148	1
Vars2	0.67781982	0.60512567	0.012	0.01	1
Srebf1	0.67799092	0.45605539	0.058	0.063	1
Apex2	0.6784233	0.56923372	0.017	0.015	1
Bub3	0.67890969	0.49021526	0.154	0.157	1
Nomo1	0.67993081	0.26598529	0.03	0.033	1
Zfp770	0.68051641	0.6547089	0.023	0.02	1
Chd1l	0.68108404	0.49083561	0.03	0.027	1
Tmem161a	0.68170979	0.39440028	0.028	0.031	1
Cd101	0.68221377	0.61307875	0.024	0.022	1
Ube2v1	0.68260573	0.419741	0.023	0.02	1
Ilvbl	0.68279616	0.45442868	0.044	0.041	1
Gatsl3	0.68282374	0.31067452	0.038	0.036	1
Topors	0.68297671	0.26240773	0.092	0.101	1
AW554918	0.68452186	0.47444287	0.021	0.024	1
Ankrd26	0.68666057	0.6645223	0.014	0.012	1
2700062C07R	0.68717912	0.3937304	0.026	0.029	1
Vps11	0.68794596	0.29761037	0.059	0.065	1
Gm20324	0.68888189	0.51490452	0.01	0.009	1
Taf6l	0.68902334	0.31747093	0.171	0.191	1
Adsl	0.68908446	0.59999677	0.042	0.039	1
Aar2	0.69078129	0.47014677	0.051	0.048	1
Mdp1	0.6914315	0.35616728	0.115	0.114	1
Oas1a	0.69214699	0.61538669	0.014	0.016	1
Pgghg	0.69227246	0.43156296	0.023	0.026	1
Pus10	0.69238677	0.51703472	0.017	0.02	1
Cnp	0.69241275	0.40452797	0.063	0.069	1
Acot13	0.6925768	0.42126256	0.051	0.048	1
Slc25a22	0.69269782	0.46761459	0.017	0.015	1
Clstn1	0.69304448	0.74781683	0.021	0.019	1
Usp28	0.69349991	0.27628787	0.012	0.014	1
Hist2h2ac	0.69368917	0.31520745	0.065	0.071	1
Primpol	0.69474225	0.79382499	0.021	0.019	1
Phkg2	0.69477875	0.35349155	0.08	0.078	1
Sf3a1	0.6955235	0.3539518	0.103	0.102	1
Naa30	0.69560933	0.37158271	0.073	0.071	1
Itih5	0.69567512	0.79286525	0.017	0.016	1
Trappc2	0.69571638	0.66533185	0.024	0.022	1
Brpf1	0.69653878	0.34241038	0.079	0.086	1
Dcaf7	0.69691543	0.32763849	0.108	0.106	1

Snx2	0.70017521	0.42043696	0.164	0.167	1
C1galt1	0.7004517	0.33490157	0.084	0.081	1
Ftsj3	0.70124423	0.41524937	0.075	0.073	1
Tef	0.70129539	0.25080862	0.023	0.025	1
Rrnad1	0.70144682	0.37733704	0.033	0.037	1
Fbxl19	0.7019414	1.03799055	0.01	0.009	1
Trmt6	0.70207253	0.32731517	0.07	0.076	1
Socs5	0.70216112	0.30433817	0.035	0.038	1
Vegfb	0.70224412	0.88979786	0.026	0.024	1
Stoml1	0.70281692	0.47248164	0.019	0.017	1
Minpp1	0.70333334	0.4561296	0.028	0.026	1
Mms22l	0.7035612	0.70599637	0.026	0.024	1
Mrgbp	0.70358976	0.41926199	0.033	0.037	1
Hnrnph3	0.70386777	0.26463126	0.133	0.131	1
Acads	0.70454698	0.59161404	0.047	0.044	1
Timm9	0.70506236	0.42875786	0.045	0.05	1
Naa10	0.70600255	0.44095929	0.099	0.11	1
Ankzf1	0.70664773	0.70798611	0.019	0.017	1
Fbxo38	0.70678606	0.26038388	0.073	0.08	1
Adamts1	0.7069258	1.02646965	0.016	0.014	1
Ncln	0.70704212	0.71996047	0.051	0.048	1
Tjap1	0.70708939	0.37362932	0.045	0.05	1
3010003L21Ri	0.70760233	0.28630145	0.012	0.014	1
Zfp626	0.70798047	0.26044119	0.023	0.025	1
Plekhj1	0.70899169	0.3509989	0.18	0.186	1
Bcas3	0.70917629	0.31587395	0.033	0.037	1
Casp7	0.70939897	0.356726	0.017	0.02	1
Tfam	0.7100155	0.26572356	0.098	0.095	1
Nsdhl	0.71049324	0.67145419	0.028	0.026	1
Slx4ip	0.71106322	0.41429597	0.024	0.022	1
Mib2	0.7114622	0.91468577	0.012	0.011	1
Gm26561	0.71271701	0.96080606	0.01	0.009	1
Mtmr4	0.71316691	0.32366608	0.024	0.027	1
Nifk	0.71316845	0.30042235	0.075	0.081	1
Kmt2c	0.71327396	0.29384426	0.204	0.211	1
Actr1b	0.71349664	0.48754544	0.086	0.084	1
Phf13	0.71412395	0.26283243	0.03	0.027	1
1190007I07Ril	0.71433407	0.30829405	0.03	0.033	1
Becn1	0.71453349	0.29442589	0.173	0.192	1
Cchcr1	0.71480745	0.28898536	0.009	0.01	1
Thra	0.71486446	0.64343972	0.023	0.021	1
Ankrd13c	0.71491651	0.26619117	0.117	0.126	1
Map2k5	0.71507347	0.3130796	0.033	0.031	1
Gnl3l	0.71509512	0.41770621	0.077	0.074	1
Slc35a1	0.71510897	0.60316376	0.019	0.022	1
Med12	0.71549805	0.27003403	0.066	0.072	1
Jmjd6	0.71564203	0.29737182	0.077	0.084	1
Mllt10	0.71625236	0.4963988	0.117	0.117	1

Stxbp5	0.71633413	0.31769205	0.054	0.051	1
Fam114a2	0.71648235	0.2796579	0.087	0.085	1
Kat8	0.71653548	0.3823662	0.016	0.018	1
Phospho2	0.71682724	0.57649804	0.051	0.048	1
Klf3	0.71705093	0.27099852	0.209	0.214	1
Trim14	0.71820482	0.80630861	0.01	0.009	1
Pde4dip	0.71821995	0.34828829	0.037	0.04	1
Kif13a	0.71860054	1.36465804	0.017	0.016	1
Alkbh3	0.7188141	0.39746614	0.035	0.038	1
Rbm41	0.71921222	0.51041189	0.024	0.022	1
Wdr75	0.71936241	0.2962626	0.049	0.053	1
Flcn	0.71956197	0.45195361	0.075	0.082	1
Gm26699	0.72049937	0.54303137	0.049	0.047	1
Cald1	0.72065312	0.66341834	0.021	0.019	1
Ring1	0.72091064	0.42392124	0.042	0.046	1
Ppp1r8	0.72147616	0.41036528	0.047	0.045	1
Chchd5	0.72165365	0.48023164	0.052	0.057	1
Faap100	0.72324162	0.49148226	0.047	0.052	1
Psmd4	0.72361878	0.33694588	0.297	0.32	1
Dolpp1	0.72382426	0.87000704	0.012	0.011	1
Ctu1	0.72384822	0.77379954	0.01	0.009	1
Bcap29	0.72436382	0.77718913	0.073	0.072	1
Dclre1a	0.72496387	1.13857791	0.012	0.011	1
Alg10b	0.72503111	0.52348322	0.045	0.043	1
Tnxb	0.72602909	0.56378861	0.01	0.009	1
Zfp729a	0.72656186	0.33450202	0.047	0.045	1
Rarres2	0.72775107	0.58575243	0.023	0.021	1
Smad1	0.72827604	0.27844432	0.037	0.04	1
Ankrd28	0.72893209	0.93178548	0.049	0.047	1
B3galt6	0.72939609	0.66352903	0.014	0.012	1
Pigx	0.73019191	0.52582541	0.079	0.077	1
Gpaa1	0.73027736	0.76356108	0.026	0.024	1
Mettl8	0.73070558	0.29605515	0.01	0.009	1
Snapc2	0.73082982	0.90417199	0.024	0.023	1
Cul5	0.73091585	0.38226313	0.092	0.101	1
Tgtp2	0.73100683	0.70421679	0.017	0.016	1
Fam20a	0.73107726	0.99223916	0.01	0.009	1
Kdm1a	0.73215142	0.34205791	0.22	0.231	1
Zfp933	0.73326316	0.82709058	0.012	0.011	1
Ibtk	0.73333168	0.34198238	0.092	0.091	1
Ubap2	0.73447024	0.29003132	0.094	0.092	1
Cnot2	0.73467413	0.4745905	0.133	0.134	1
Nup35	0.73531963	0.25917993	0.017	0.016	1
Grhpr	0.73547705	0.53045166	0.023	0.021	1
Nbas	0.73574627	0.47286584	0.031	0.029	1
Pigq	0.73576796	0.32129689	0.031	0.034	1
C330027C09F	0.73578349	0.71468197	0.01	0.009	1
Cbll1	0.73595915	0.25576154	0.105	0.114	1

Taf4b	0.7365251	0.25067056	0.086	0.083	1
Tmem185a	0.73661756	0.29293796	0.016	0.018	1
Ints5.1	0.73662532	0.75303518	0.021	0.019	1
Agfg2	0.73704853	0.44215464	0.021	0.019	1
Fbxo18	0.73760151	0.63004235	0.021	0.023	1
Nup37	0.73947997	0.52312489	0.01	0.009	1
Abcd4	0.73959844	0.36459288	0.023	0.021	1
1110004F10Ri	0.73982823	0.25947766	0.283	0.315	1
Phf20	0.74018647	0.2975456	0.073	0.079	1
Zbtb25	0.74049046	0.51792804	0.058	0.055	1
Phpt1	0.74082583	0.44384148	0.037	0.035	1
Rad52	0.74136481	0.43338147	0.016	0.018	1
Gsdme	0.74159587	1.14296811	0.01	0.009	1
5730455P16R	0.74174776	0.76250992	0.026	0.024	1
Hinfp	0.74182668	0.40364462	0.047	0.045	1
Utp23	0.7418592	0.32420529	0.063	0.068	1
Ccng1	0.74265913	0.35693994	0.12	0.12	1
Tor4a	0.74281336	0.78874369	0.01	0.009	1
Sepsecs	0.74341696	0.57411931	0.016	0.018	1
Tmem140	0.74416365	0.35828918	0.04	0.038	1
Zdhhc4	0.74450918	0.41147195	0.031	0.034	1
Rprd1b	0.74470948	0.27634907	0.092	0.09	1
Ikbkg	0.74568872	0.44773233	0.033	0.031	1
Rbbp5	0.74573142	0.6495849	0.044	0.042	1
Dpf2	0.74682872	0.31737129	0.115	0.126	1
Clcc1	0.74686598	0.48436509	0.056	0.061	1
Susd1	0.7469331	0.52074944	0.012	0.014	1
Brca2	0.74710103	0.8886884	0.01	0.009	1
Aasdhppt	0.74725237	0.40297163	0.061	0.059	1
Ccdc22	0.74727681	0.55004177	0.033	0.036	1
Exoc6b	0.74770556	0.38044004	0.045	0.043	1
Smad5	0.74778466	0.60649769	0.044	0.042	1
Lrwd1	0.74799295	0.38249862	0.033	0.031	1
Cisd2	0.74911487	0.32417534	0.269	0.289	1
Pik3r2	0.74937232	1.34782151	0.012	0.011	1
Txn2	0.749406	0.26310815	0.154	0.167	1
Pnpla6	0.7502446	0.27345396	0.016	0.018	1
Siae	0.75045749	0.66858511	0.016	0.014	1
Esf1	0.75092074	0.39557744	0.141	0.144	1
Rnf169	0.75146863	0.55627601	0.04	0.038	1
Zbtb5	0.75170992	0.57253213	0.03	0.028	1
Zfp866	0.75216752	0.36902988	0.026	0.024	1
Dnajb5	0.75232125	0.68708857	0.017	0.016	1
Setd1a	0.75246862	0.39092567	0.058	0.055	1
Tmem126b	0.75251756	0.39729516	0.023	0.025	1
Fbxw2	0.75267565	0.26994962	0.206	0.225	1
Nudcd1	0.75445809	0.59077068	0.016	0.018	1
Hpcal1	0.75476196	0.53347538	0.152	0.158	1

Cln6	0.75726484	0.67499154	0.035	0.033	1
Sf3a3	0.75728934	0.58484869	0.098	0.098	1
Ccdc84	0.75809108	0.5015973	0.023	0.021	1
Msl3	0.75809602	0.32019247	0.054	0.058	1
Snx14	0.75850902	0.61990562	0.042	0.04	1
Unk	0.75912013	0.27886088	0.03	0.028	1
Rars2	0.75926215	0.44012399	0.01	0.009	1
Ppfibp1	0.75991416	0.60449584	0.042	0.04	1
Phyh	0.75992516	0.76250967	0.063	0.062	1
Wdhd1	0.76067902	0.42023605	0.024	0.027	1
Tnpo2	0.76075085	0.31762434	0.049	0.053	1
Xpr1	0.76082756	0.35089853	0.129	0.131	1
Rab6a	0.76086044	0.34170335	0.162	0.178	1
Nans	0.76114319	0.34207161	0.087	0.095	1
Tial1	0.76127433	0.347978	0.133	0.134	1
Rarg	0.76219631	0.67141861	0.01	0.012	1
Bckdhb	0.76236339	0.49783547	0.01	0.009	1
Bdp1	0.7626749	0.26164001	0.166	0.169	1
Cd2ap	0.76313777	0.29456585	0.152	0.166	1
Pcna-ps2	0.76314301	0.85697261	0.01	0.009	1
Recql	0.76327521	0.25305224	0.024	0.023	1
Dynlrb1	0.7633436	0.30595302	0.262	0.278	1
Macrod1	0.76343513	0.46573892	0.01	0.009	1
Emilin1	0.7634547	0.50163298	0.01	0.012	1
Fbxl18	0.76359056	0.27225059	0.009	0.01	1
Brwd1	0.76371095	0.3399278	0.105	0.104	1
Snrnp48	0.76395771	0.34378914	0.182	0.198	1
Cdca4	0.76404116	0.34790999	0.052	0.056	1
Urb1	0.76405396	0.42335022	0.019	0.018	1
Tmc6	0.76449294	0.47997706	0.052	0.056	1
Ahdc1	0.76450794	0.85887083	0.016	0.014	1
Cfl2	0.76468606	0.40558241	0.098	0.098	1
Zfp931	0.76491383	0.62220212	0.019	0.018	1
Ulk2	0.76509161	0.36831703	0.042	0.045	1
Atg12	0.76534972	0.30053195	0.147	0.159	1
Rint1	0.76589226	0.3930941	0.028	0.03	1
Nudt16	0.76614416	0.6207625	0.037	0.035	1
Mterf4	0.76671793	0.46005945	0.016	0.017	1
Rnf168	0.76696033	0.62985837	0.051	0.049	1
Sfswap	0.76751612	0.37076376	0.154	0.156	1
Depdc5	0.76755285	0.65084237	0.03	0.028	1
Wwox	0.76842165	0.6122487	0.021	0.023	1
Rnf115	0.76860763	0.28675005	0.12	0.131	1
Sdha	0.76925998	0.33662931	0.18	0.198	1
Impad1	0.76947779	0.35827836	0.091	0.098	1
Nme7	0.76997332	0.57249351	0.012	0.014	1
Ep300	0.77186269	0.3474829	0.188	0.195	1
Morc2a	0.77268239	0.30543012	0.04	0.038	1

Npm3	0.7738936	0.34582972	0.166	0.182	1
Prickle1	0.77399074	0.30868449	0.014	0.016	1
Arfip1	0.77423895	0.46501292	0.037	0.04	1
Tmem126a	0.77452279	0.50113079	0.079	0.085	1
Arfgap1	0.77462021	0.26145854	0.047	0.051	1
Tram2	0.77462112	0.55816058	0.019	0.021	1
Trmt5	0.77491567	0.95034637	0.01	0.009	1
Sptlc1	0.77497715	0.40043621	0.065	0.07	1
Lsm2	0.77504379	0.45318656	0.091	0.091	1
Eif5a2	0.77567366	1.0560289	0.014	0.013	1
Gys1	0.77593455	0.60074055	0.009	0.01	1
Ncapd2	0.77639753	0.45495753	0.026	0.025	1
Gm10036	0.77672113	0.31048641	0.265	0.284	1
BC003965	0.77776792	0.39273583	0.052	0.056	1
Abcb8	0.77844702	0.64582267	0.012	0.011	1
Ccdc80	0.77864495	0.52656502	0.012	0.011	1
Hadh	0.77906748	0.41689568	0.061	0.059	1
Crip2	0.77927171	0.48967859	0.016	0.017	1
Gm32036	0.78033104	0.61358858	0.017	0.016	1
Ltn1	0.78090666	0.35687263	0.07	0.068	1
Ap4s1	0.78120675	0.2939293	0.059	0.064	1
Dyrk1a	0.78141122	0.37277932	0.178	0.186	1
Man2a2	0.78145309	0.32097207	0.115	0.115	1
Akt2	0.7817884	0.48727385	0.129	0.132	1
Oxsm	0.78286806	0.78942943	0.01	0.012	1
Nek8	0.78323731	0.2919837	0.017	0.016	1
Herpud2	0.78325266	0.40900479	0.11	0.11	1
Sppl2b	0.78361299	0.54378172	0.017	0.019	1
Nprl3	0.78420164	0.70536643	0.019	0.018	1
Zbtb41	0.78423764	0.35268869	0.035	0.033	1
Gtpbp1	0.78436503	0.32672803	0.066	0.065	1
Lsg1	0.78518141	0.4150972	0.087	0.087	1
Tmx2	0.78585179	0.5475692	0.031	0.03	1
Pafah1b2	0.78620741	0.43499485	0.103	0.104	1
Krcc1	0.78631931	0.33946818	0.11	0.11	1
Rpia	0.78652826	0.5894915	0.058	0.062	1
Cwc27	0.78692983	0.42990483	0.045	0.044	1
Borcs6	0.78784488	0.36043459	0.052	0.051	1
Mllt6	0.7886755	0.32064495	0.155	0.169	1
Tlr1	0.78900659	0.9653759	0.026	0.025	1
Ppcs	0.78901924	0.64209192	0.016	0.014	1
Klf13	0.78934415	0.2595006	0.284	0.313	1
Smim27	0.78955524	0.39739987	0.084	0.083	1
Sirt7	0.79014517	0.41936059	0.113	0.115	1
Tmem238	0.79050237	0.32823853	0.035	0.038	1
Vps45	0.7907691	0.42895371	0.017	0.019	1
Ube2o	0.79081865	0.31926397	0.04	0.043	1
Gm16286	0.79107592	0.3638145	0.239	0.26	1

AC163032.1	0.79130711	0.39332079	0.024	0.023	1
Med23	0.79203455	0.54422611	0.049	0.048	1
Arhgef11	0.79297403	0.51023393	0.024	0.027	1
Ice1	0.79392604	0.26020874	0.075	0.074	1
Gm26724	0.79434696	0.34687513	0.014	0.013	1
Wdr24	0.79480135	0.38894821	0.01	0.012	1
Sc5d	0.79516885	0.34235804	0.084	0.083	1
Smarcc2	0.7954253	0.43306885	0.073	0.078	1
Fam160b2	0.79566378	0.38312653	0.044	0.047	1
Polr3b	0.796365	0.35440461	0.03	0.028	1
Dclre1b	0.79664281	0.32671937	0.017	0.019	1
Bola1	0.79673177	0.49360297	0.044	0.042	1
Zfp407	0.79684291	0.5804209	0.063	0.062	1
Telo2	0.79742283	0.36194544	0.012	0.014	1
Susd3	0.7974814	0.75463102	0.059	0.058	1
Zcwpw1	0.79770311	0.67366184	0.014	0.013	1
Alad	0.79782435	0.33039495	0.023	0.025	1
Lypla1	0.79797687	0.43641959	0.105	0.105	1
Rabggta	0.79799106	0.47537376	0.033	0.032	1
Rc3h1	0.79808644	0.25519925	0.213	0.222	1
Nepro	0.79850385	0.91147996	0.03	0.028	1
Mrpl24	0.79958681	0.34408183	0.216	0.228	1
Gbp6	0.79993947	0.42995225	0.016	0.014	1
Zfp52	0.80158979	0.35528151	0.038	0.037	1
Usp10	0.80169233	0.28779054	0.061	0.065	1
Ing4	0.80203717	0.28331361	0.063	0.067	1
Tmem165	0.8028095	0.32075046	0.073	0.078	1
Abcg2	0.80345514	0.86167808	0.009	0.01	1
Mppe1	0.80364826	1.04173192	0.014	0.013	1
Eed	0.80377727	0.34976932	0.108	0.116	1
Fbxl8	0.80404109	0.3437357	0.01	0.012	1
Hspbap1	0.80416118	0.34886685	0.019	0.018	1
Ufl1	0.80421221	0.59026468	0.073	0.073	1
Dhx57	0.80469078	0.37679882	0.024	0.023	1
Tmx1	0.80521379	0.44768681	0.099	0.107	1
Mfn2	0.80550644	0.40960896	0.033	0.036	1
Abca3	0.80585991	0.47625205	0.044	0.047	1
Grsf1	0.8059256	0.64363814	0.061	0.06	1
Map4k3	0.80627812	0.65235422	0.019	0.021	1
Gm17477	0.80639303	0.44516511	0.012	0.011	1
Ube2v2	0.80644434	0.3401029	0.108	0.116	1
Mob4	0.80716613	0.3120477	0.197	0.206	1
Rnf146	0.80833059	0.39542956	0.127	0.13	1
Dlg1	0.8097972	0.46886577	0.092	0.093	1
Tab3	0.8099019	0.38893701	0.038	0.037	1
Reep4	0.80992014	0.37430728	0.045	0.044	1
Mlycd	0.81020096	0.48697824	0.014	0.013	1
Ap3b1	0.81099942	0.42924856	0.138	0.151	1

Epb41l4aos	0.81107529	0.41805765	0.175	0.183	1
Eogt	0.81173363	0.97362558	0.01	0.012	1
Gtpbp10	0.81176681	0.51382743	0.024	0.023	1
Polr1b	0.81201205	0.26165327	0.01	0.012	1
Strn4	0.81270033	0.34353934	0.065	0.069	1
Exosc10	0.81321685	0.33654872	0.066	0.07	1
Chchd4	0.81340617	0.29047284	0.042	0.045	1
Wdr43	0.81387303	0.28890223	0.195	0.202	1
Exoc4	0.81429174	0.40433963	0.096	0.096	1
Dclre1c	0.81449957	0.44193462	0.065	0.064	1
Gpsm2	0.81473791	0.71804865	0.01	0.01	1
Fam219b	0.81485483	0.45637014	0.021	0.02	1
Tceanc	0.81522492	0.82292244	0.01	0.01	1
Zscan29	0.81565648	0.5136455	0.026	0.025	1
AU041133	0.81660318	0.56449077	0.017	0.016	1
Brcc3	0.81676758	0.31773384	0.063	0.067	1
Pik3c3	0.81683066	0.2531577	0.056	0.055	1
Pphln1	0.81706486	0.41051554	0.058	0.061	1
Slc9a7	0.8177746	0.25148882	0.012	0.013	1
Thoc2	0.81798112	0.29520428	0.222	0.233	1
Ddx19b	0.81850936	0.3704168	0.033	0.035	1
Flywch1	0.81907322	0.4566039	0.017	0.016	1
Timm21	0.81934926	0.25345906	0.023	0.024	1
Gmpr2	0.81936358	0.37052875	0.028	0.03	1
Tsr3	0.81956668	0.45934871	0.037	0.039	1
Dffb	0.8196599	0.37932563	0.016	0.017	1
Ncoa5	0.81969322	0.44440415	0.031	0.034	1
Fam3a	0.82019502	0.70127218	0.021	0.023	1
Mrm3	0.82061092	0.31622552	0.01	0.012	1
Zmat1	0.82147332	0.34776833	0.009	0.01	1
Qtrt2	0.82173943	0.46097246	0.017	0.019	1
Atg4b	0.82259899	0.38107452	0.056	0.055	1
Tfpt	0.82425426	0.35089066	0.028	0.03	1
Sirt6	0.8252729	0.55574282	0.012	0.011	1
E530011L22Ri	0.82552828	0.4194589	0.028	0.03	1
Akr1e1	0.82602662	0.53705724	0.01	0.012	1
Dyrk1b	0.82625999	0.46844502	0.026	0.025	1
Exosc9	0.82645126	0.29021441	0.045	0.048	1
Slc25a23	0.82699215	0.39977289	0.012	0.011	1
Usp46	0.82715325	0.31477987	0.009	0.01	1
Txndc12	0.82752999	0.8904855	0.035	0.034	1
Trappc8	0.82759389	0.76344628	0.086	0.086	1
Prps1l3	0.82789119	0.53059242	0.023	0.022	1
Hmgxb4	0.82794251	0.29535159	0.044	0.046	1
Setd6	0.82884162	0.36485697	0.014	0.015	1
AI606181	0.82895165	0.47731503	0.023	0.022	1
Pitrm1	0.82918628	0.65763607	0.033	0.035	1
Snx12	0.829277	0.66752715	0.063	0.062	1

Opa1	0.82931743	0.7241609	0.051	0.05	1
Borcs8	0.82937503	0.5034318	0.126	0.129	1
Rfwd3	0.83002461	0.69775134	0.038	0.037	1
Mia2	0.83042543	0.261934	0.244	0.274	1
Psph	0.83067011	0.65376924	0.033	0.035	1
Taf3	0.83077176	0.25247251	0.11	0.109	1
Arl4a	0.83113	0.29180815	0.115	0.116	1
Dtwd2	0.83130679	0.53696221	0.01	0.01	1
1810030O07R	0.83181501	0.42415895	0.056	0.059	1
Atrn	0.8320197	0.39107252	0.035	0.034	1
Ube2r2	0.83328647	0.37194953	0.157	0.164	1
Clasp1	0.8333964	0.32804793	0.066	0.065	1
Ppp2r1b	0.83395399	0.3588279	0.03	0.032	1
Sh2b1	0.83519926	0.31679715	0.07	0.069	1
Drg2	0.83531837	0.44340993	0.028	0.03	1
Rnf128	0.8354421	0.46337648	0.012	0.013	1
Mindy2	0.83552275	0.25598546	0.098	0.103	1
Ensa	0.8358356	0.52545791	0.194	0.209	1
Bccip	0.83590404	0.3612754	0.113	0.115	1
Gm4070	0.83635347	0.41580938	0.035	0.037	1
Myo9b	0.83737007	0.41339829	0.117	0.127	1
Mbd1	0.83764827	0.28860409	0.066	0.07	1
Vps13b	0.83941249	0.34296485	0.087	0.093	1
Ruvbl2	0.83981863	0.50356586	0.024	0.026	1
Lzic	0.84042137	0.81796735	0.045	0.044	1
Wdr83os	0.84133598	0.28547136	0.197	0.209	1
Disp1	0.84178324	0.32169805	0.01	0.011	1
Gatsl2	0.84268481	0.36849834	0.017	0.019	1
Ube2w	0.8427964	0.46149771	0.063	0.063	1
Spata7	0.84309883	0.29785255	0.009	0.01	1
Ccdc58	0.84310127	0.40800775	0.031	0.033	1
Foxo4	0.84322088	0.37968382	0.021	0.02	1
Ankrd40	0.84324889	0.25846226	0.065	0.068	1
Adck1	0.84344735	0.5632252	0.017	0.019	1
Tmem156	0.84354265	0.30142437	0.044	0.042	1
Inip	0.84386285	0.50685455	0.07	0.07	1
Rcc1l	0.84412289	0.37584391	0.017	0.017	1
Kat2a	0.84412589	0.49993142	0.016	0.017	1
Srrt	0.84457398	0.36588391	0.127	0.137	1
Snx6	0.84472368	0.44835181	0.11	0.113	1
2810402E24R	0.84580292	0.36975434	0.059	0.059	1
Akap11	0.84587252	0.37146441	0.068	0.072	1
Alg3	0.84649934	0.41854151	0.014	0.015	1
Habp4	0.84678747	0.53261863	0.014	0.013	1
Fuom	0.84810428	0.713777	0.03	0.029	1
Frg1	0.84821296	0.29068006	0.215	0.236	1
Plekhn1	0.84908904	0.52335522	0.026	0.028	1
Wbp2	0.84938812	0.33657453	0.201	0.212	1

Eloc	0.85011395	0.29588605	0.34	0.381	1
Ppie	0.8507437	0.53619441	0.065	0.065	1
Bloc1s6	0.85094812	0.39085795	0.049	0.048	1
Traf4	0.85113909	0.29763238	0.089	0.093	1
Armc8	0.85149004	0.44811342	0.049	0.048	1
Eef1akmt1	0.85154035	0.42983667	0.031	0.03	1
Exosc3	0.85195816	0.34111579	0.103	0.11	1
AC165953.2	0.85292614	0.54102055	0.01	0.01	1
Zfp709	0.85310611	0.53917339	0.01	0.011	1
Dusp12	0.85397603	0.50021717	0.03	0.029	1
Atg14	0.85417089	0.49877069	0.058	0.061	1
Wdr7	0.85526252	0.25761391	0.024	0.023	1
Prpsap1	0.85591258	0.3338566	0.068	0.072	1
Zbtb6	0.85614634	0.45533197	0.044	0.043	1
Fgfr1op2	0.85644702	0.26042305	0.274	0.308	1
Desi1	0.85656289	0.50155836	0.066	0.066	1
Elp3	0.85659093	0.5819171	0.021	0.02	1
Pcbp4	0.8568838	0.44522458	0.012	0.011	1
Dlgap4	0.85704514	0.40657779	0.068	0.072	1
Dcaf13	0.85706035	0.49957299	0.127	0.132	1
Zfp825	0.85775281	0.4285181	0.01	0.011	1
Ddx19a	0.85793883	0.41113147	0.063	0.066	1
Dync1li2	0.85805041	0.56200719	0.051	0.05	1
Pdss2	0.85812261	0.52252894	0.012	0.011	1
Vti1a	0.85925589	0.31018879	0.058	0.061	1
Pgrmc2	0.85932497	0.30943308	0.077	0.081	1
Mtrf1l	0.86048211	0.76154109	0.016	0.017	1
Coa3	0.86093033	0.40593447	0.115	0.124	1
Npat	0.86107771	0.25525508	0.054	0.053	1
Tpra1	0.86215678	0.42314937	0.026	0.028	1
Epg5	0.86239633	0.4429262	0.028	0.027	1
Rpap1	0.86247025	0.27661585	0.019	0.02	1
Psmd7	0.86278767	0.31871576	0.178	0.187	1
Pcnt	0.86294769	0.30703572	0.105	0.106	1
Chfr	0.86303193	0.35080204	0.131	0.141	1
Rnf121	0.86374458	0.37016774	0.045	0.048	1
R3hcc1	0.86462658	0.54872616	0.016	0.017	1
Lrrc1	0.86472739	0.5075836	0.012	0.013	1
Akip1	0.86485015	0.55281294	0.021	0.02	1
Abhd11	0.86569406	0.49815411	0.026	0.025	1
Gid8	0.86618323	0.4418019	0.072	0.076	1
Txndc15	0.86657441	0.44722021	0.052	0.055	1
Ankfy1	0.86687502	0.43634558	0.099	0.101	1
Pla2g12a	0.86725684	0.25598723	0.021	0.022	1
Ap5m1	0.86745355	0.60311907	0.045	0.045	1
Neurl4	0.86760656	0.44619313	0.019	0.02	1
Mre11a	0.86792305	0.55395708	0.037	0.039	1
Gm9493	0.86814057	0.46262289	0.079	0.079	1

Gon7	0.86870289	0.37979489	0.051	0.05	1
Utp11	0.86873882	0.53526188	0.124	0.129	1
Ttc9c	0.86894719	0.49998258	0.058	0.061	1
Fam120a	0.86918384	0.30453708	0.169	0.176	1
Pradc1	0.86930365	0.44548043	0.012	0.013	1
Armc5	0.87003604	0.51422214	0.047	0.047	1
Ppp1r21	0.87028197	0.27561821	0.044	0.043	1
Pex5	0.87079438	0.4606485	0.037	0.036	1
Bloc1s1	0.87081575	0.9646162	0.04	0.04	1
Psmg1	0.87124775	0.44633307	0.016	0.017	1
Mpv17	0.87183231	0.50369192	0.019	0.018	1
Pyroxd1	0.87188038	0.42153888	0.045	0.047	1
Eral1	0.87214841	0.27278858	0.014	0.015	1
Zhx2	0.8727479	0.27259998	0.113	0.114	1
Fbxw8	0.87340512	0.48231293	0.028	0.029	1
Ap5z1	0.87363071	0.54699951	0.01	0.01	1
Kansl2	0.87376248	0.46087307	0.056	0.059	1
Tmem170b	0.8739934	0.38064722	0.035	0.037	1
Slc39a3	0.87426519	0.39229285	0.026	0.025	1
Rcl1	0.87429947	0.4200815	0.063	0.063	1
Cep295	0.87444591	0.39718322	0.072	0.072	1
Ano10	0.87459702	0.25259247	0.026	0.025	1
Xrcc6	0.87501365	0.61712415	0.012	0.013	1
Gclm	0.8755707	0.62093688	0.037	0.039	1
Zfp39	0.87598748	0.81697898	0.01	0.01	1
Zbtb14	0.87648708	0.61471242	0.01	0.01	1
Cd2bp2	0.87693854	0.28497406	0.119	0.12	1
Runx2os1	0.87717249	0.40178009	0.017	0.018	1
Rabl3	0.87722144	0.54848683	0.023	0.024	1
Iffo1	0.87747664	0.43593868	0.024	0.024	1
Armcx2	0.87766354	0.73747205	0.01	0.01	1
Papd7	0.8781508	0.83611821	0.031	0.031	1
Zfp280b	0.87827756	0.25882308	0.021	0.02	1
Smad2	0.87942247	0.44497775	0.094	0.096	1
Igip	0.88044219	0.44251036	0.012	0.012	1
Iffo2	0.8808227	0.25470562	0.028	0.029	1
Cygb	0.88106569	0.44575988	0.01	0.01	1
Ttc27	0.88126029	0.59962924	0.016	0.015	1
Rbm28	0.88137927	0.29538338	0.143	0.147	1
Gle1	0.88169946	0.37420379	0.063	0.062	1
mt-Nd4l	0.88170073	0.29447344	0.321	0.358	1
Hdgfl2	0.88199448	0.54838277	0.054	0.057	1
Mplkip	0.88203787	0.45791414	0.11	0.112	1
AI314180	0.88226203	0.27314803	0.11	0.112	1
Thap2	0.8829426	0.48449668	0.058	0.057	1
Rdh10	0.88366093	0.36675047	0.012	0.013	1
Ubr4	0.88403436	0.34364033	0.147	0.157	1
Dnajb4	0.8841511	0.5474772	0.092	0.094	1

Dtymk	0.88440316	0.39716767	0.056	0.058	1
Lpcat4	0.88554384	0.57479726	0.023	0.022	1
Ccdc94	0.8855786	0.41150796	0.019	0.019	1
Trp53rka	0.88626937	0.57134198	0.031	0.033	1
Glyr1	0.88654817	0.27479965	0.168	0.181	1
Dcaf4	0.88676086	0.29123423	0.016	0.015	1
Chpf2	0.88702928	0.45666095	0.021	0.022	1
Gphn	0.88742675	0.87577573	0.026	0.028	1
Crtc3	0.88786768	0.49579212	0.143	0.151	1
Park7	0.88808175	0.29580239	0.277	0.313	1
Parp4	0.88888593	0.35813875	0.094	0.096	1
Mrpl15	0.8894885	0.36947373	0.12	0.124	1
Dpp7	0.88962963	0.91505335	0.021	0.022	1
2700016F22Ri	0.89027834	0.48085987	0.012	0.012	1
Cdc26	0.89042585	0.30593164	0.115	0.122	1
Agrn	0.89057627	0.74327821	0.01	0.01	1
Sdhaf4	0.89085732	0.39401109	0.044	0.046	1
Gigyf2	0.89100839	0.26618294	0.108	0.11	1
Haus5	0.89106181	0.42026516	0.014	0.013	1
Slc17a9	0.89155222	0.42232984	0.012	0.012	1
Zfp994	0.89201152	0.5308232	0.023	0.024	1
Gmeb2	0.89216482	0.32334399	0.063	0.066	1
Fbln1	0.89229564	0.34386178	0.019	0.02	1
Trim26	0.89231737	0.25371222	0.129	0.137	1
Col5a1	0.89238617	0.48279931	0.01	0.011	1
Stxbp1	0.89365431	0.58554204	0.017	0.017	1
Mex3d	0.89404915	0.82791134	0.01	0.011	1
Isoc1	0.89412676	0.56980842	0.035	0.034	1
Mark3	0.8950249	0.31977823	0.161	0.173	1
Ndufaf6	0.89541813	0.49111843	0.014	0.015	1
Xpnpep3	0.89556256	0.40408992	0.017	0.017	1
Tnfaip6	0.89595054	0.36964572	0.035	0.034	1
Rad9a	0.89732831	0.72780554	0.016	0.015	1
Zc3h3	0.89778692	0.42573193	0.026	0.026	1
Rnf25	0.89792362	0.7633337	0.026	0.027	1
Inppl1	0.8995788	0.62524463	0.024	0.026	1
Ccdc127	0.90018588	0.29575412	0.089	0.09	1
Bcas2	0.90033749	0.25901156	0.237	0.259	1
Klhdc3	0.90178242	0.29461336	0.068	0.068	1
Rbm34	0.90226592	0.43022657	0.105	0.111	1
Enox2	0.90238356	0.49023122	0.024	0.026	1
Wdr11	0.90241042	0.34185346	0.026	0.027	1
Mnat1	0.90249529	0.62856239	0.072	0.076	1
Ambra1	0.90380931	0.50705384	0.047	0.047	1
Atg4d	0.90454914	0.49071963	0.044	0.046	1
Aacs	0.90578483	0.4658548	0.016	0.015	1
Mtg2	0.90624796	0.38997819	0.017	0.018	1
Gm8186	0.90640661	0.31553819	0.073	0.077	1

Rps6kb1	0.90677541	0.26421976	0.162	0.169	1
Gm26670	0.90680705	0.79051188	0.01	0.01	1
Ulk3	0.90710034	0.46756082	0.012	0.012	1
Washc2	0.90719738	0.31733527	0.131	0.139	1
Mlh1	0.90754968	0.44740347	0.012	0.012	1
Med25	0.90760553	0.6399964	0.058	0.058	1
Slc30a7	0.90807294	0.61835967	0.044	0.044	1
Ino80e	0.90845935	0.37476087	0.072	0.075	1
Manba	0.90851808	0.35881995	0.019	0.019	1
Zbtb2	0.90855337	0.25479492	0.037	0.036	1
Gtpbp6	0.90855612	0.40864298	0.037	0.036	1
Zfp472	0.90872768	0.25019018	0.026	0.027	1
Mcmbp	0.90899068	0.77142995	0.094	0.096	1
Acp1	0.90923111	0.52287957	0.131	0.137	1
Nmi	0.90964175	0.3812658	0.119	0.122	1
Rhbdd3	0.91011231	0.4350978	0.01	0.011	1
Twf1	0.91035895	0.5926454	0.11	0.118	1
Rfx1	0.91055675	0.27297293	0.045	0.045	1
Kbtbd2	0.91060725	0.339408	0.112	0.114	1
4732440D04R	0.91071889	0.61810058	0.04	0.042	1
Rcn3	0.91074511	0.46237908	0.019	0.02	1
Ccl11	0.91129438	0.57787922	0.012	0.013	1
Naxd	0.91147444	0.4336213	0.089	0.091	1
Col6a3	0.91224232	0.38199028	0.016	0.015	1
Sdhaf3	0.91244063	0.41174494	0.014	0.015	1
Mief1	0.91247383	0.55282749	0.042	0.042	1
Riok2	0.91276068	0.51061545	0.045	0.045	1
Gm13212	0.9130256	0.4738658	0.037	0.036	1
Trip4	0.91338178	0.4097064	0.049	0.049	1
Sap130	0.91354728	0.51842642	0.019	0.019	1
Mpst	0.91373582	0.48909295	0.049	0.051	1
Sf3b4	0.91378247	0.37100675	0.103	0.109	1
Ccnyl1	0.91429049	0.41588632	0.026	0.026	1
Rnf214	0.91435018	0.29796366	0.035	0.036	1
Map3k10	0.91506953	0.58600183	0.012	0.013	1
Zc3h11a	0.91507662	0.47589624	0.021	0.02	1
Sars2	0.91553358	0.42479911	0.014	0.014	1
Usp47	0.91560164	0.32842755	0.14	0.145	1
Tmco4	0.9160604	0.69161113	0.016	0.015	1
Taok2	0.91629099	0.31245468	0.061	0.061	1
Phf1	0.91632574	0.38130879	0.07	0.07	1
Armc7	0.91654382	0.2565549	0.033	0.033	1
Rtn4rl1	0.91658847	0.28223571	0.01	0.011	1
Zfp398	0.91708908	0.28903109	0.049	0.05	1
Fam122b	0.91709817	1.01174728	0.01	0.011	1
Fasn	0.91763974	0.60710811	0.051	0.053	1
Asb7	0.91885283	0.42953616	0.016	0.015	1
Cherp	0.91905627	0.26830325	0.07	0.07	1

Smim13	0.91995329	0.61034585	0.031	0.031	1
Tnpo1	0.92008204	0.26255387	0.171	0.178	1
Nmb	0.92049922	0.32426616	0.024	0.025	1
Ecsit	0.92056891	0.4768306	0.033	0.034	1
Pgs1	0.92132632	0.46835073	0.098	0.104	1
Dnajc24	0.92132695	0.29763558	0.038	0.04	1
Cdk6	0.92138338	0.43434338	0.122	0.127	1
Cenpq	0.92281455	0.4264452	0.084	0.088	1
Igf2bp3	0.92310455	0.26152159	0.016	0.015	1
Zfp729b	0.92311817	0.48974176	0.038	0.04	1
Ascc2	0.9231542	0.26379319	0.04	0.04	1
Mob3a	0.92333043	0.45568388	0.052	0.054	1
Med21	0.92383876	0.32224363	0.11	0.113	1
Senp5	0.92498046	0.36455754	0.08	0.084	1
Itpa	0.92502635	0.3897799	0.047	0.049	1
Slc25a16	0.9256158	0.36310057	0.03	0.029	1
Gm26890	0.92596023	0.26354708	0.01	0.01	1
Tmem8	0.92601197	0.64254964	0.024	0.024	1
Katnb1	0.92677102	0.94741667	0.014	0.015	1
Ercc3	0.92740248	0.32217593	0.061	0.063	1
Pnrc2	0.92794694	0.40740415	0.138	0.144	1
March6	0.92816444	0.37604122	0.112	0.115	1
Pdcd2	0.92829818	0.37765756	0.068	0.069	1
Cep350	0.92937824	0.48487313	0.166	0.18	1
Uba3	0.92953315	0.33222187	0.058	0.058	1
Sp1	0.93046738	0.46615833	0.15	0.158	1
Bcl2l12	0.93049999	0.28417957	0.017	0.018	1
Wdr13	0.93060618	0.47242349	0.024	0.024	1
Zfand2b	0.93107833	0.7244905	0.079	0.081	1
Cenpt	0.93166567	0.45868896	0.01	0.01	1
Nsrp1	0.93196992	0.3166642	0.176	0.189	1
Gmppa	0.93280989	0.44427766	0.045	0.046	1
Oas2	0.93357528	0.87387959	0.014	0.014	1
Extl3	0.93362611	0.32309972	0.024	0.025	1
Accs	0.93401334	0.57417166	0.014	0.014	1
Gpatch2l	0.93421619	0.42522333	0.044	0.045	1
Mcm2	0.93431091	0.25289626	0.037	0.038	1
Mkl2	0.93544404	0.79676981	0.019	0.019	1
1700096K18R	0.93594104	0.43949067	0.028	0.028	1
Stambp	0.93601162	0.42701147	0.044	0.045	1
Akr1b10	0.93610111	0.56473037	0.031	0.033	1
Cenpk	0.9377896	0.2515124	0.017	0.018	1
Cnot10	0.93836772	0.41883083	0.059	0.061	1
Gm45716	0.9393162	0.64022392	0.162	0.175	1
Arhgef10	0.93958336	0.53498535	0.021	0.021	1
A630072M18F	0.94026975	0.30036357	0.059	0.059	1
Zfp704	0.94089859	1.33635265	0.01	0.01	1
Ndc80	0.94118876	0.55101605	0.014	0.014	1

Dock8	0.94120113	0.28071587	0.162	0.172	1
Nudt14	0.94186326	0.80626472	0.04	0.041	1
Gpsm3	0.9424005	0.32696824	0.209	0.228	1
Mecr	0.94247776	1.26298581	0.017	0.017	1
Mrpl10	0.94367943	0.42136037	0.044	0.045	1
Cep290	0.94408969	0.94531554	0.023	0.022	1
Timmdc1	0.94419726	0.50036781	0.016	0.016	1
Fastk	0.94479204	0.73664249	0.033	0.034	1
D17H6S53E	0.94487067	0.44522458	0.021	0.021	1
Gramd1a	0.94556805	0.27421869	0.148	0.157	1
Rpp25l	0.94615418	0.27798537	0.098	0.1	1
Cdan1	0.9470748	0.36996536	0.019	0.02	1
Nop2	0.9483611	0.72175798	0.021	0.021	1
Cacfd1	0.94860762	0.59392103	0.037	0.037	1
Tecr	0.94882225	0.35089081	0.161	0.172	1
Eml3	0.94887134	0.36202976	0.035	0.036	1
Polr2m	0.94938697	0.32554429	0.204	0.217	1
Alg1	0.94949245	0.47745111	0.026	0.026	1
Tomm70a	0.94969835	0.36258985	0.152	0.162	1
Rtl8a	0.95015788	0.55856892	0.024	0.025	1
Atf1	0.95045473	0.32157074	0.155	0.163	1
2410004B18R	0.95068331	0.41792899	0.07	0.073	1
Ggnbp2	0.95206951	0.29808381	0.199	0.21	1
Setdb1	0.95219923	0.54982011	0.04	0.04	1
Tmem175	0.95224963	0.43341172	0.012	0.013	1
Mad1l1	0.9528305	0.8561943	0.019	0.019	1
4932438H23R	0.95362602	0.29087808	0.016	0.015	1
Ccdc137	0.95395513	0.57493533	0.019	0.019	1
Josd2	0.95401267	0.5617695	0.059	0.062	1
Tgfbrap1	0.95462554	0.27691711	0.03	0.03	1
Prr3	0.95463979	0.55163193	0.03	0.03	1
Atp11c	0.95488861	0.38565612	0.056	0.056	1
Cdkn2aip	0.95527361	0.29347878	0.082	0.083	1
Map3k2	0.95545638	0.31490875	0.169	0.18	1
Ap4e1	0.95590113	0.26872352	0.026	0.027	1
Prmt7	0.956054	0.34412025	0.023	0.023	1
Kat7	0.95617204	0.37805781	0.089	0.091	1
Brf2	0.95635411	0.50128975	0.017	0.017	1
Cul2	0.95671649	0.28557353	0.075	0.076	1
2310061I04Ril	0.95796561	0.38647705	0.026	0.027	1
Mvb12b	0.9580248	0.48419479	0.056	0.058	1
Tmem259	0.95820208	0.35719786	0.117	0.121	1
Pds5b	0.95833773	0.29019576	0.063	0.063	1
Hist4h4	0.95845597	0.47849042	0.017	0.018	1
Tmem230	0.95851611	0.55762777	0.061	0.063	1
Hectd4	0.95887792	0.42460037	0.028	0.028	1
Mvk	0.95914844	0.69104488	0.026	0.026	1
Arsk	0.95929863	0.97415364	0.014	0.014	1

Pqbp1	0.95938785	0.31535419	0.117	0.119	1
Dhx37	0.9605747	0.33874681	0.017	0.018	1
Jkamp	0.96063727	0.2903082	0.047	0.048	1
Nudt13	0.96064485	0.43225331	0.023	0.023	1
Spg20	0.96078973	0.39125751	0.024	0.025	1
1600020E01R	0.96238427	0.51147799	0.056	0.058	1
Rev1	0.9625866	0.37407444	0.047	0.048	1
Fam136a	0.96275627	0.29213054	0.035	0.035	1
Ficd	0.96321644	0.71690872	0.012	0.012	1
Lsm7	0.96379027	0.44580848	0.108	0.114	1
Gemin5	0.96386316	0.41702462	0.016	0.016	1
Cyb5b	0.96418624	0.36986944	0.11	0.113	1
Slc39a13	0.96423395	0.69502396	0.016	0.016	1
Fam192a	0.96442211	0.34642346	0.106	0.11	1
Zfp445	0.9646041	0.33383282	0.14	0.146	1
Zfp653	0.96673953	0.38890891	0.016	0.016	1
Klhl11	0.96727002	0.28893431	0.016	0.016	1
Shkbp1	0.96793779	0.38719739	0.056	0.057	1
Rexo5	0.96849889	0.36294617	0.012	0.012	1
Gstcd	0.96892347	0.95208682	0.019	0.02	1
Crkl	0.9691696	0.48869012	0.086	0.09	1
Tmem80	0.96933942	0.48551439	0.03	0.03	1
Ltbp4	0.9694211	0.3955504	0.023	0.023	1
Gmeb1	0.96999049	0.35831306	0.045	0.046	1
Cpsf3	0.97002657	0.30061881	0.049	0.049	1
Dido1	0.97088602	0.31152853	0.061	0.063	1
Abca2	0.97089569	0.32264761	0.033	0.034	1
Dedd	0.97090208	0.35237781	0.045	0.046	1
Gm26882	0.97131034	0.29865483	0.024	0.024	1
Lin7c	0.97159999	0.35205161	0.175	0.185	1
Tspyl2	0.971859	0.2749148	0.031	0.032	1
Gsk3a	0.97196422	0.27116937	0.14	0.147	1
Iqce	0.9730113	0.39622548	0.012	0.012	1
Krr1	0.97317525	0.3331166	0.072	0.074	1
Pole4	0.9743485	0.28799449	0.234	0.253	1
Gm10762	0.97441851	0.53001468	0.017	0.017	1
Tsn	0.97517576	0.26841187	0.225	0.244	1
Ypel5	0.97667613	0.31036563	0.138	0.144	1
Rbm10	0.97674792	0.28953771	0.051	0.051	1
Chmp7	0.97685678	0.51303571	0.03	0.03	1
Zgrf1	0.9772026	0.55169715	0.012	0.012	1
Zbtb8os	0.97747101	0.53541335	0.086	0.09	1
Gatd1	0.97786217	0.41624316	0.052	0.053	1
Stard10	0.97788254	0.36718413	0.023	0.023	1
Vezf1	0.97793781	0.27461122	0.159	0.166	1
Ctnnbl1	0.9788985	0.40581588	0.065	0.066	1
Meg3	0.97891283	0.34830073	0.01	0.011	1
Rgp1	0.97910307	0.32650679	0.019	0.019	1

Ttf1	0.97914793	0.33297466	0.068	0.07	1
Zfp68	0.97976414	0.5043424	0.07	0.072	1
Zc3h4	0.98043323	0.28262599	0.058	0.058	1
Sil1	0.98088115	0.53834111	0.026	0.027	1
Tdrd3	0.980999	0.42435804	0.021	0.021	1
Cactin	0.98149189	0.30740793	0.033	0.034	1
Alg12	0.9819883	0.71981291	0.012	0.012	1
Lrrc61	0.98263906	0.55042547	0.016	0.016	1
Anapc7	0.9827399	0.40705646	0.056	0.057	1
Rpl36a-ps1	0.98279019	0.40961728	0.098	0.101	1
Entpd5	0.98305171	0.35772356	0.021	0.021	1
Cxcl12	0.98394101	0.38402753	0.019	0.019	1
Plrg1	0.98396592	0.37730149	0.037	0.037	1
Cdc27	0.98443714	0.39299084	0.084	0.086	1
Aldoc	0.98445782	0.68738672	0.023	0.023	1
Zfp692	0.98455338	0.29100801	0.014	0.014	1
Gm37305	0.98495194	0.50646105	0.014	0.014	1
Msl1	0.98545455	0.36404062	0.087	0.091	1
Odf2l	0.98545588	0.54685467	0.016	0.016	1
Kiz	0.98609468	0.70395769	0.019	0.019	1
Hmgn3	0.98668447	0.43075562	0.03	0.03	1
Elf4	0.98779595	0.50320633	0.092	0.096	1
Numb	0.987807	0.42365112	0.033	0.034	1
Zfp280c	0.98810469	0.37991168	0.023	0.023	1
Npepps	0.98824038	0.43458729	0.11	0.115	1
Tmem33	0.98867144	0.35776919	0.134	0.141	1
Midn	0.98922521	0.50016909	0.108	0.114	1
Zfyve1	0.98932236	0.40535501	0.028	0.028	1
Gm26526	0.98951817	0.55156737	0.019	0.019	1
Dcps	0.98958391	0.55004933	0.051	0.052	1
Arl6ip6	0.99000126	0.4360479	0.063	0.064	1
Mrps36	0.99003738	0.38112609	0.113	0.119	1
Zfp598	0.99044446	0.30283396	0.045	0.046	1
Galm	0.99063942	0.89228506	0.01	0.011	1
Ogfod2	0.99109943	0.3416793	0.024	0.025	1
Rbbp7	0.99174211	0.40733703	0.173	0.184	1
Calhm2	0.99197408	0.53078266	0.012	0.012	1
Upf1	0.992105	0.42718921	0.099	0.103	1
Htatsf1	0.99221035	0.37866738	0.103	0.107	1
Cbx1	0.99255379	0.28559168	0.155	0.162	1
Galnt11	0.99269014	0.34151229	0.141	0.147	1
Galnt10	0.99288445	0.38438996	0.026	0.026	1
Dstyk	0.99379528	0.44048658	0.03	0.03	1
Ints2	0.99410452	0.53939128	0.019	0.019	1
Gm527	0.99426113	0.42827953	0.012	0.012	1
Mocs3	0.99430914	0.3844602	0.01	0.011	1
Map3k4	0.99450326	0.38085003	0.047	0.048	1
Dxo	0.99458891	0.42124999	0.038	0.039	1

Supt6	0.99481518	0.28101843	0.19	0.202	1
Ubqln4	0.99482113	0.74645247	0.03	0.03	1
Cntln	0.99513424	0.92506145	0.023	0.023	1
Zfp639	0.99525428	0.46113172	0.082	0.085	1
Rpf1	0.99609343	0.35995483	0.098	0.101	1
AI467606	0.99622057	0.44358579	0.08	0.082	1
Lcorl	0.99632224	0.38230127	0.061	0.062	1
Fastkd2	0.99673717	0.80831392	0.021	0.021	1
Nsun2	0.99692092	0.38755634	0.086	0.088	1
B230219D22R	0.99780567	0.2568799	0.244	0.265	1
Sh3glb2	0.99783475	0.44500431	0.042	0.042	1
Med15	0.99801106	0.52834646	0.147	0.157	1
3110002H16R	0.99802638	0.39776657	0.03	0.03	1
Scaf4	0.99884354	0.49348866	0.038	0.039	1
Alg11	0.99965744	0.47580554	0.023	0.023	1

CD4 Treg

Gene	p_val	avg_log2FC	pct.1	pct.2	p_val_adj
Ctla4	0	3.16157871	0.924	0.27	0
Tnfrsf4	0	3.54056316	0.799	0.15	0
Foxp3	0	7.99036898	0.645	0.005	0
Maf	0	3.19400539	0.903	0.309	0
Icos	0	2.14923088	0.887	0.339	0
Tnfrsf9	0	3.87718208	0.65	0.119	0
Ikzf2	0	3.06685283	0.66	0.164	0
Hopx	0	3.5119519	0.718	0.222	0
Ccr2	0	3.10555236	0.705	0.237	0
Cd2	0	2.09012354	0.731	0.273	0
Tnfrsf18	0	2.23666898	0.667	0.243	0
Itgav	0	2.66554042	0.608	0.189	0
Il10	0	3.95569118	0.436	0.052	0
Cd27	0	2.91058948	0.48	0.096	0
Gzmb	0	3.62960405	0.464	0.08	0
Areg	0	2.12437788	0.454	0.117	0
Samsn1	0	1.44924904	0.845	0.514	0
Cldnd1	0	2.41794563	0.552	0.227	0
Nt5e	0	2.86394716	0.41	0.087	0
Neb	0	6.37893037	0.326	0.007	0
Ifngr1	0	1.21070166	0.926	0.611	0
Odc1	0	1.53513574	0.909	0.612	0
Izumo1r	0	4.10454538	0.312	0.02	0
Klrg1	0	4.1079163	0.268	0.026	0
Cpm	0	2.78647505	0.29	0.057	0
Il1rl1	0	3.72841955	0.27	0.045	0
Itgb8	0	6.35080613	0.174	0.004	0
Ikzf4	0	4.55367867	0.162	0.011	0
Lrrc32	0	4.11945358	0.146	0.016	0
Nckap5	0	6.33915038	0.122	0.002	0
Sh3rf1	0	4.14302456	0.119	0.01	0
Ky	0	8.45739579	0.092	0	0
H2-K1	0	0.91625618	0.986	0.911	0
Prg4	0	5.50159767	0.061	0.002	0
Frmd5	0	7.23669285	0.06	0.001	0
Trp53inp1	9.521E-299	2.01487272	0.514	0.194	2.089E-294
Dusp4	8.8E-285	2.26171395	0.395	0.124	1.931E-280
Cd28	1.115E-282	1.4599155	0.663	0.3	2.446E-278
Flicr	2.574E-279	6.11811375	0.043	0.001	5.647E-275
Lamc1	4.605E-277	2.37890074	0.331	0.091	1.01E-272
Nrip1	8.445E-269	1.37481589	0.775	0.476	1.853E-264
Trib2	9.103E-262	2.15123401	0.377	0.118	1.997E-257
Eea1	3.639E-257	2.23799409	0.505	0.221	7.984E-253
Gm15655	2.09E-256	3.27496298	0.142	0.02	4.585E-252
Smco4	5.457E-250	2.34873476	0.339	0.103	1.197E-245
Penk	3.294E-247	4.60618864	0.102	0.011	7.226E-243

Il2ra	6.958E-245	1.78078935	0.396	0.131	1.526E-240
Trac	3.554E-244	1.54673536	0.537	0.223	7.797E-240
Arhgap31	5.59E-243	1.90481334	0.505	0.218	1.226E-238
Glrx	1.315E-240	2.22164487	0.422	0.163	2.886E-236
Il6st	7.297E-239	2.63072113	0.323	0.1	1.601E-234
Nav2	2.408E-238	2.78645805	0.212	0.045	5.284E-234
Gbp3	3.51E-236	2.40283668	0.338	0.108	7.702E-232
Nr3c1	1.133E-235	1.26417169	0.739	0.427	2.485E-231
Il2rb	2.194E-235	1.12306896	0.633	0.287	4.813E-231
Cd3e	3.022E-235	1.28445099	0.621	0.283	6.63E-231
Ets1	6.042E-233	0.89401127	0.948	0.658	1.326E-228
Slc5a3	2.37E-231	2.56214586	0.276	0.075	5.2E-227
Got1	2.721E-231	1.45129195	0.72	0.45	5.969E-227
Leprotl1	2.767E-231	1.75878632	0.581	0.308	6.07E-227
Hacd3	3.793E-230	2.36402088	0.325	0.101	8.322E-226
Cd4	5.695E-230	1.73184795	0.416	0.148	1.249E-225
Vps54	2.388E-229	2.23007782	0.367	0.126	5.239E-225
Stat5b	5.424E-227	1.64862006	0.567	0.273	1.19E-222
Hif1a	6.641E-227	1.17623769	0.814	0.564	1.457E-222
Gata3	9.452E-219	1.93433547	0.394	0.14	2.074E-214
Ccr4	9.432E-214	2.47377647	0.209	0.047	2.069E-209
Crem	6.299E-212	1.10190982	0.818	0.588	1.382E-207
Sla	3.263E-210	1.43779895	0.657	0.396	7.16E-206
Gpld1	6.336E-209	5.44910984	0.038	0.001	1.39E-204
Gm45716	4.817E-207	2.2821112	0.408	0.166	1.057E-202
Sdf4	7.621E-206	1.70702705	0.604	0.361	1.672E-201
Gimap3	6.336E-204	1.32551835	0.604	0.301	1.39E-199
Faim	7.429E-204	2.46022785	0.334	0.117	1.63E-199
Rora	2.165E-203	0.95922228	0.795	0.433	4.751E-199
Tox	9.546E-203	2.2433949	0.274	0.08	2.094E-198
Sdcbp2	1.456E-199	2.90363247	0.172	0.035	3.195E-195
Fam107b	1.426E-198	1.14735142	0.78	0.557	3.129E-194
Camk4	1.485E-197	1.85374453	0.368	0.134	3.257E-193
Arl5a	2.586E-196	2.15093331	0.402	0.17	5.673E-192
Tmem64	3.436E-195	1.50204572	0.503	0.228	7.539E-191
Dgat1	3.958E-195	0.98356723	0.763	0.45	8.683E-191
Jak2	9.194E-195	1.32460801	0.692	0.442	2.017E-190
Peli1	1.899E-194	1.42039846	0.702	0.463	4.165E-190
Gm3558	4.813E-194	7.56533179	0.022	0	1.056E-189
Gpr55	2.14E-193	2.62642554	0.251	0.072	4.695E-189
Malat1	4.29E-191	0.6274776	1	0.999	9.412E-187
Zeb1	7.237E-191	1.36378936	0.62	0.344	1.588E-186
E130308A19R	3.648E-187	3.02792023	0.131	0.022	8.004E-183
Zc3h12d	1.626E-183	2.64349694	0.191	0.045	3.567E-179
Tbc1d30	2.161E-183	3.13449345	0.109	0.016	4.741E-179
Cd247	6.182E-183	1.71923638	0.344	0.123	1.356E-178
Fam46a	9.055E-183	1.8675873	0.486	0.239	1.987E-178
AU020206	7.936E-181	1.60147056	0.511	0.258	1.741E-176

Cd3d	5.549E-179	1.02799428	0.625	0.307	1.217E-174
Igtp	1.273E-175	1.87543159	0.331	0.12	2.794E-171
Ttn	3.607E-175	2.82485261	0.116	0.019	7.914E-171
Stk4	5.595E-175	1.30300873	0.644	0.389	1.227E-170
Id2	5.522E-171	1.00548809	0.809	0.594	1.211E-166
Arnt2	7.222E-169	4.04614236	0.062	0.006	1.584E-164
H2afz	2.37E-168	1.43253472	0.862	0.721	5.199E-164
Gimap7	1.206E-167	1.51920163	0.411	0.174	2.645E-163
Inpp4b	3.138E-167	1.77426034	0.356	0.14	6.885E-163
H2-D1	1.114E-166	0.60374426	0.976	0.913	2.443E-162
Ncmap	2.811E-166	5.0933904	0.031	0.001	6.168E-162
AW112010	9.316E-166	1.20221076	0.699	0.458	2.044E-161
Mxi1	6.951E-162	1.41714249	0.535	0.293	1.525E-157
Myo3b	2.907E-160	4.93585236	0.035	0.002	6.378E-156
H2-Q7	5.839E-160	0.92499448	0.803	0.62	1.281E-155
Lag3	1.701E-159	2.97752537	0.161	0.037	3.732E-155
Aebp2	1.138E-158	0.93006209	0.752	0.519	2.497E-154
Stat1	2.007E-157	1.59065458	0.567	0.345	4.403E-153
Vgll4	1.536E-155	1.28459401	0.557	0.312	3.371E-151
Ttc39c	2.747E-155	2.28926038	0.228	0.07	6.027E-151
Khdrbs1	8.208E-153	1.39141634	0.631	0.434	1.801E-148
Fam169b	4.575E-152	1.825931	0.266	0.089	1.004E-147
Reps1	1.129E-151	2.20474251	0.26	0.09	2.477E-147
Shisa5	3.143E-151	1.00155663	0.76	0.585	6.895E-147
Ubald2	4.453E-149	0.84552928	0.773	0.553	9.77E-145
Cytip	2.711E-148	0.79004115	0.884	0.736	5.949E-144
Mrps6	3.003E-147	1.97406795	0.315	0.126	6.589E-143
Ccr3	2.971E-146	4.54780716	0.037	0.002	6.517E-142
Mbnl1	3.926E-146	0.80334179	0.881	0.763	8.614E-142
Saraf	4.24E-142	1.38924035	0.58	0.374	9.303E-138
Camk2n1	6.718E-141	1.95749555	0.257	0.089	1.474E-136
Adamts6	2.591E-140	3.01590775	0.089	0.014	5.685E-136
Wnk1	5.408E-137	0.95854086	0.799	0.663	1.186E-132
Ahnak	1.499E-136	0.90606246	0.757	0.537	3.288E-132
Mbd2	2.227E-135	1.33342984	0.572	0.368	4.886E-131
Ifi27l2a	1.061E-134	0.82196695	0.696	0.467	2.328E-130
Cd3g	5.032E-134	0.75226333	0.601	0.307	1.104E-129
Gnb4	2.144E-130	2.76820038	0.153	0.04	4.704E-126
Zfp281	6.308E-129	1.88669402	0.25	0.089	1.384E-124
Crbn	6.558E-129	1.56023448	0.362	0.165	1.439E-124
Dst	3.089E-128	2.1470675	0.222	0.075	6.776E-124
Cd6	4.661E-128	1.44014298	0.342	0.145	1.023E-123
S100a10	2.687E-127	0.98947933	0.736	0.553	5.896E-123
Zfp36l1	8.726E-127	0.81943389	0.818	0.64	1.914E-122
Fam110c	2.158E-126	6.4698982	0.018	0	4.734E-122
Stx11	5.343E-126	1.72926787	0.366	0.175	1.172E-121
Mmd	9.237E-126	2.29954601	0.249	0.093	2.026E-121
Prkch	2.652E-125	1.59272418	0.338	0.15	5.819E-121

Agfg1	7.331E-125	1.67386883	0.373	0.181	1.608E-120
Spock2	1.157E-123	2.89625353	0.086	0.014	2.538E-119
Ankrd55	1.34E-123	3.93848372	0.064	0.008	2.94E-119
Bcl11b	1.416E-123	1.4494717	0.36	0.162	3.107E-119
Gas2	2.12E-123	3.73922721	0.062	0.008	4.651E-119
Abcb1a	7.718E-122	1.45407715	0.365	0.17	1.693E-117
Matn2	2.978E-120	3.90099892	0.05	0.005	6.533E-116
Srgn	9.525E-120	0.34755622	0.98	0.932	2.09E-115
Ly6a	2.321E-119	1.44149683	0.514	0.313	5.092E-115
Foxo1	2.97E-119	1.18473586	0.519	0.303	6.515E-115
Rnf125	1.416E-117	1.32611828	0.404	0.2	3.107E-113
Actg1	1.613E-117	0.52016618	0.97	0.912	3.539E-113
Gimap1	4.343E-117	1.15426076	0.492	0.271	9.528E-113
Rbm24	2.681E-116	3.80276107	0.045	0.004	5.881E-112
Slamf1	1.105E-115	1.4653594	0.325	0.142	2.423E-111
Tmem158	7.36E-115	3.78819315	0.065	0.009	1.615E-110
Cish	3.085E-114	1.52746736	0.403	0.211	6.768E-110
Prkar1a	1.379E-112	0.83892701	0.732	0.593	3.025E-108
Gimap4	2.739E-112	1.03799415	0.501	0.278	6.008E-108
Stat3	2.676E-111	0.98665319	0.74	0.605	5.87E-107
Capg	2.015E-110	1.57591356	0.371	0.191	4.421E-106
Itga6	6.699E-110	2.15036473	0.159	0.046	1.47E-105
Trbc2	4.827E-108	0.61474265	0.655	0.379	1.059E-103
Gbp7	5.854E-108	1.64829015	0.292	0.128	1.284E-103
Gna13	6.365E-108	0.81335576	0.791	0.674	1.396E-103
Kdm2b	1.339E-107	0.90763642	0.58	0.351	2.937E-103
Cd5	2.74E-107	1.32374315	0.309	0.133	6.011E-103
Runx2	1.33E-106	1.64900697	0.335	0.161	2.917E-102
Atp2b4	1.651E-106	1.44594359	0.373	0.191	3.623E-102
Psen2	3.367E-105	1.84857671	0.265	0.113	7.386E-101
Fam129a	4.036E-105	1.4273154	0.433	0.25	8.855E-101
Neu3	1.753E-104	2.89186116	0.084	0.016	3.846E-100
Crlf3	4.114E-102	1.3076413	0.525	0.355	9.0264E-98
Gm15340	2.185E-100	2.62432561	0.071	0.012	4.7933E-96
Pard6g	2.282E-100	3.31826714	0.047	0.006	5.0065E-96
Rabgap1l	2.35E-99	1.46426074	0.355	0.181	5.1546E-95
Rpap3	1.1179E-98	2.28465717	0.138	0.039	2.4526E-94
Ptprc	1.5825E-97	0.50573583	0.937	0.893	3.4719E-93
Gm3488	3.0334E-97	7.77493156	0.01	0	6.655E-93
Hsbp1l1	3.0705E-97	3.50676014	0.047	0.006	6.7364E-93
Pqlc1	1.4922E-96	2.12212906	0.216	0.085	3.2738E-92
Ctla2a	3.2318E-96	1.21893234	0.342	0.164	7.0902E-92
Emb	4.0682E-95	0.48512744	0.847	0.614	8.9253E-91
C2cd4b	5.9833E-95	4.55376564	0.028	0.002	1.3127E-90
Creb3l2	8.3319E-94	2.41880451	0.134	0.039	1.8279E-89
Itgae	4.4427E-92	2.09606244	0.155	0.05	9.7469E-88
Socs2	4.6653E-92	1.31268095	0.216	0.082	1.0235E-87
Gm3468	9.7096E-92	5.41143495	0.017	0.001	2.1302E-87

2010111I01Ril	1.626E-90	1.9239012	0.232	0.098	3.5672E-86
Gpr68	3.5965E-90	1.92394236	0.197	0.075	7.8904E-86
Tub	3.8537E-90	4.58940939	0.022	0.001	8.4546E-86
Sntb1	5.7319E-90	2.11334612	0.129	0.037	1.2575E-85
Msrb3	1.7693E-89	3.49908335	0.048	0.006	3.8816E-85
Ptprcap	1.871E-89	1.28705087	0.371	0.199	4.1049E-85
Itpkb	1.0178E-88	1.04124292	0.555	0.383	2.2329E-84
Fgf16	2.7622E-88	4.98458681	0.019	0.001	6.0601E-84
Ubl3	4.1029E-88	1.06662815	0.618	0.49	9.0013E-84
Iigp1	3.0689E-85	2.63865959	0.109	0.029	6.7328E-81
Rgs9	4.5352E-85	2.77536606	0.091	0.022	9.9498E-81
Ddx5	7.8365E-85	0.43495958	0.972	0.952	1.7193E-80
Ptp4a2	4.5599E-83	0.66732332	0.795	0.722	1.0004E-78
Adora2a	2.6649E-82	1.42182889	0.314	0.161	5.8465E-78
Lat	3.3404E-82	0.96629104	0.409	0.224	7.3284E-78
Twsg1	4.8887E-82	1.97613282	0.172	0.063	1.0725E-77
Lpcat1	8.5408E-82	2.24269028	0.165	0.06	1.8738E-77
Tnfrsf1b	1.4303E-81	0.92666623	0.578	0.423	3.1379E-77
4930503L19Ril	7.6365E-81	1.83129506	0.195	0.077	1.6754E-76
Myl12b	8.2644E-81	0.88755251	0.66	0.546	1.8131E-76
Wisp1	9.6718E-80	3.60954791	0.036	0.004	2.1219E-75
D16Ertd472e	1.5836E-79	0.80451213	0.56	0.372	3.4743E-75
Zc3hav1	7.3229E-79	0.87254269	0.591	0.441	1.6066E-74
Atxn1	8.0783E-79	1.18684562	0.351	0.19	1.7723E-74
Tox2	2.572E-78	2.08692777	0.126	0.039	5.6427E-74
Tnik	5.3093E-78	1.53827081	0.211	0.088	1.1648E-73
Itpk1	4.0814E-77	1.83804815	0.181	0.071	8.9541E-73
Mier1	1.2768E-76	0.98306474	0.551	0.403	2.8011E-72
Phlpp1	1.6931E-76	1.78982793	0.248	0.119	3.7145E-72
Ppil1	2.0727E-76	2.13861081	0.122	0.038	4.5473E-72
Kbtbd11	5.195E-76	1.54814458	0.249	0.116	1.1397E-71
Zdhhc23	7.4771E-76	2.0260232	0.138	0.046	1.6404E-71
Prrc2c	7.6565E-76	0.67241164	0.778	0.703	1.6798E-71
Itm2b	5.5746E-74	0.67521861	0.8	0.722	1.223E-69
Zfp53	5.9637E-74	2.11634473	0.116	0.035	1.3084E-69
Osbpl3	5.0389E-73	1.7645228	0.192	0.079	1.1055E-68
Ly6e	1.1705E-72	0.57045231	0.846	0.776	2.568E-68
Serpinb6a	3.5187E-72	1.68146694	0.205	0.089	7.7197E-68
Tigit	4.0148E-72	2.28242437	0.087	0.022	8.808E-68
Lrig1	5.4234E-72	2.35728282	0.074	0.017	1.1898E-67
Il7r	1.2666E-71	0.7599093	0.573	0.392	2.7789E-67
Miat	1.8688E-71	2.84286796	0.029	0.003	4.1001E-67
Dgat2	2.2918E-71	2.3096577	0.093	0.025	5.028E-67
Gm3636	5.8084E-71	4.59959213	0.02	0.001	1.2743E-66
Tank	2.5826E-70	1.16436887	0.423	0.278	5.666E-66
Lrba	5.676E-69	1.6555167	0.211	0.095	1.2453E-64
Prdm1	6.2954E-69	1.50413926	0.318	0.18	1.3811E-64
Thap12	2.129E-68	1.63550358	0.239	0.116	4.6709E-64

Bmyc	9.8824E-68	2.37300718	0.124	0.042	2.1681E-63
Irgm2	4.4402E-67	1.85024856	0.123	0.041	9.7413E-63
Itk	1.0498E-66	0.6409571	0.503	0.313	2.3032E-62
Ipmk	1.2413E-66	1.04555001	0.283	0.143	2.7233E-62
Syt11	6.2258E-66	2.18755274	0.102	0.031	1.3659E-61
Lck	1.4195E-65	0.97303549	0.351	0.197	3.1141E-61
P2ry10	4.278E-65	0.70618787	0.613	0.456	9.3855E-61
Arhgef12	7.427E-65	1.95540468	0.134	0.048	1.6294E-60
Tmem123	7.4496E-65	0.89921746	0.576	0.459	1.6344E-60
Mdh1	7.494E-65	1.37479239	0.399	0.269	1.6441E-60
Alox8	1.5556E-64	4.11991283	0.016	0.001	3.4129E-60
Traf1	1.9269E-64	0.65721024	0.681	0.536	4.2274E-60
Pim1	2.062E-64	0.44988431	0.886	0.745	4.5238E-60
B2m	2.9961E-64	0.3009559	0.987	0.979	6.5731E-60
Cdkn1b	5.4056E-64	0.85203831	0.541	0.395	1.1859E-59
Rapsn	7.7831E-63	3.09396807	0.059	0.013	1.7075E-58
Med15	1.431E-62	1.55864264	0.276	0.153	3.1394E-58
Zcchc11	5.193E-62	0.70768553	0.666	0.552	1.1393E-57
Mcm6	5.6542E-62	1.6165146	0.201	0.092	1.2405E-57
Rab37	9.6589E-62	2.00283723	0.107	0.034	2.1191E-57
Fam46c	9.8129E-62	1.24943516	0.343	0.211	2.1529E-57
Gm527	1.1727E-61	2.70610829	0.053	0.011	2.5729E-57
Gm26740	2.1077E-61	1.05225645	0.364	0.217	4.6241E-57
Coq8a	3.0826E-61	1.6067111	0.175	0.074	6.763E-57
Laptm4b	4.9533E-61	2.87494462	0.068	0.016	1.0867E-56
Panx1	6.9499E-61	1.89675541	0.153	0.061	1.5247E-56
Pak2	1.1608E-60	0.78163877	0.614	0.526	2.5466E-56
Herc1	9.6801E-60	1.10292754	0.379	0.241	2.1237E-55
Gpr83	1.1205E-59	4.24121363	0.014	0.001	2.4583E-55
Atl3	1.4409E-59	1.53240973	0.22	0.108	3.1613E-55
Fyb	6.3527E-59	0.62362171	0.648	0.506	1.3937E-54
Gimap6	8.6508E-59	0.57712319	0.593	0.407	1.8979E-54
Arid5b	9.3385E-59	1.24169354	0.314	0.184	2.0488E-54
Emsy	2.3586E-58	1.19181936	0.324	0.192	5.1744E-54
Tex2	2.4626E-58	1.19342329	0.323	0.191	5.4027E-54
Tpbgl	3.9345E-58	3.28019815	0.042	0.007	8.6319E-54
Srpk2	4.0199E-58	1.39054218	0.327	0.198	8.8192E-54
Samd11	1.2137E-57	1.42419177	0.191	0.086	2.6627E-53
Heca	1.4167E-57	1.09640303	0.36	0.225	3.1081E-53
Spin1	2.6442E-57	1.34541056	0.273	0.151	5.8011E-53
Ltb	6.7777E-57	0.9147391	0.423	0.271	1.487E-52
Egln3	8.492E-57	1.81949104	0.113	0.039	1.8631E-52
Sh2d1a	1.6093E-56	1.52161239	0.175	0.077	3.5307E-52
Elf1	1.6851E-56	0.68402768	0.597	0.462	3.697E-52
Snx18	2.5147E-56	0.89031466	0.513	0.387	5.517E-52
Gm4841	5.5157E-56	3.69436292	0.018	0.001	1.2101E-51
Tapbpl	7.4066E-56	1.47549921	0.228	0.117	1.6249E-51
Zdhhc18	5.1352E-55	0.84500533	0.496	0.36	1.1266E-50

Ptpn13	2.6904E-53	0.59729228	0.185	0.082	5.9026E-49
S100a4	1.4721E-52	0.69904456	0.514	0.355	3.2296E-48
Ube2b	1.8809E-52	0.65593162	0.652	0.572	4.1265E-48
Msi2	3.021E-52	1.01024811	0.35	0.219	6.6278E-48
S100a6	3.2254E-52	0.37150816	0.688	0.538	7.0763E-48
Sp3	3.3941E-52	1.24358378	0.323	0.202	7.4463E-48
Irf6	4.6303E-52	3.7763408	0.027	0.004	1.0159E-47
Gpr15	5.7146E-52	3.42439176	0.027	0.004	1.2537E-47
Rac1	8.0909E-52	0.70989561	0.609	0.528	1.7751E-47
Slc25a19	1.277E-51	1.74871278	0.189	0.093	2.8015E-47
Ddit4	2.5579E-51	1.15698945	0.306	0.184	5.6118E-47
Sh3gl3	2.8867E-51	3.94725375	0.019	0.002	6.333E-47
Tnfrsf10b	1.3107E-50	1.99632933	0.08	0.024	2.8756E-46
Sec14l1	1.3788E-50	1.68065077	0.166	0.076	3.025E-46
Grhl1	1.8364E-50	3.35784257	0.034	0.006	4.0288E-46
A630023P12R	2.1356E-50	2.29761731	0.053	0.012	4.6854E-46
Tnrc6b	3.5252E-50	0.82375016	0.476	0.344	7.734E-46
Zap70	3.8474E-50	1.15995355	0.248	0.133	8.4408E-46
Cep120	1.3019E-49	1.32502675	0.211	0.108	2.8562E-45
Fancf	2.1657E-49	1.94951499	0.09	0.03	4.7512E-45
Rnf157	2.3335E-49	1.10689107	0.323	0.2	5.1194E-45
Slc9a3r1	3.291E-49	1.10595962	0.315	0.194	7.2201E-45
Pnrc1	5.5121E-49	0.43948343	0.807	0.737	1.2093E-44
Stat5a	1.2224E-48	1.35217239	0.295	0.182	2.6818E-44
Cables1	5.1658E-48	1.59169729	0.132	0.055	1.1333E-43
Ndfip1	1.8343E-47	0.86603467	0.406	0.281	4.0242E-43
Zdhhc2	2.9611E-47	1.73904719	0.107	0.04	6.4963E-43
Gm2237	4.5414E-47	3.57714154	0.02	0.002	9.9633E-43
Itgb7	2.9124E-46	1.09748608	0.36	0.246	6.3896E-42
Mxd4	2.9631E-46	1.15388673	0.29	0.177	6.5007E-42
Ikzf3	6.069E-46	0.61857971	0.44	0.293	1.3315E-41
Prkca	1.0124E-45	1.44364043	0.149	0.066	2.2212E-41
Cep85l	1.0431E-45	1.99428392	0.132	0.057	2.2884E-41
Chd3	1.3751E-45	0.93431991	0.409	0.289	3.0167E-41
Cmtm7	1.769E-45	1.26099402	0.318	0.21	3.881E-41
Syp	1.8336E-45	3.73431015	0.018	0.002	4.0228E-41
Tpr	2.6842E-45	0.78007521	0.585	0.512	5.8889E-41
Ypel4	6.9243E-45	3.16077013	0.026	0.004	1.5191E-40
Nxnl2	1.1565E-44	3.22779028	0.023	0.003	2.5372E-40
Chd2	1.7385E-44	0.71677896	0.581	0.491	3.8141E-40
Cxcr3	6.0973E-44	1.55748516	0.117	0.047	1.3377E-39
Gimap8	9.0866E-44	1.13842623	0.202	0.104	1.9935E-39
Map6	1.6155E-43	3.37133326	0.021	0.003	3.5441E-39
Zfp260	1.6911E-43	1.64818304	0.142	0.064	3.7102E-39
Gm10827	1.8513E-43	1.65980994	0.112	0.044	4.0616E-39
Elovl6	3.5265E-43	1.96291241	0.089	0.031	7.7368E-39
Ccdc88c	5.7035E-43	1.05711111	0.26	0.15	1.2513E-38
Acot11	9.6202E-43	2.70464813	0.041	0.009	2.1106E-38

Magi1	1.0731E-42	3.91364269	0.014	0.001	2.3543E-38
Tiam1	1.9874E-42	1.7457682	0.17	0.086	4.3601E-38
Sv2c	5.8118E-42	1.99458001	0.062	0.018	1.2751E-37
Trbc1	6.9474E-42	0.62947466	0.398	0.259	1.5242E-37
Zcchc18	7.4305E-42	2.23711747	0.052	0.013	1.6302E-37
Batf	1.2518E-41	1.2591174	0.282	0.178	2.7464E-37
Slain1	2.5188E-41	1.27399131	0.147	0.067	5.526E-37
Mif4gd	5.1146E-41	1.0939179	0.322	0.214	1.1221E-36
Gimap5	9.8382E-41	0.79296489	0.35	0.225	2.1584E-36
Ccl20	1.0269E-40	5.03965183	0.012	0.001	2.2529E-36
Pkp3	1.3448E-40	1.38485656	0.169	0.084	2.9503E-36
Lclat1	1.548E-40	2.3300122	0.056	0.016	3.3961E-36
Sytl2	3.0594E-40	1.39608074	0.149	0.07	6.712E-36
Vmp1	4.356E-40	0.83982247	0.436	0.335	9.5565E-36
Gbp5	4.9289E-40	1.00509918	0.183	0.094	1.0814E-35
Grap	6.4376E-40	1.00191409	0.231	0.128	1.4123E-35
Ipcef1	1.2187E-39	1.44215323	0.124	0.054	2.6738E-35
Gna15	1.5853E-39	1.37382665	0.157	0.076	3.4779E-35
Gpr174	1.7082E-39	1.12098057	0.174	0.087	3.7475E-35
Serinc1	1.9301E-39	0.86436269	0.463	0.369	4.2344E-35
Spn	2.2876E-39	0.8375418	0.326	0.209	5.0189E-35
Gpr146	3.4275E-39	1.73110292	0.138	0.065	7.5195E-35
Fryl	1.0515E-38	0.92139985	0.33	0.22	2.3068E-34
Ccr5	1.858E-38	0.79240165	0.397	0.284	4.0763E-34
Atp2b1	2.3568E-38	0.62509709	0.664	0.599	5.1705E-34
Ccr9	3.3885E-38	2.50440913	0.069	0.023	7.434E-34
Slc25a51	4.085E-38	1.24560318	0.265	0.169	8.9622E-34
Morf4l1	5.3111E-38	0.45578604	0.747	0.713	1.1652E-33
Esyt2	5.4672E-38	0.92381191	0.36	0.25	1.1995E-33
Kdm5b	1.1553E-37	1.2271088	0.189	0.102	2.5346E-33
Ncoa2	1.7902E-37	1.0556914	0.287	0.185	3.9276E-33
Nmb	3.156E-37	1.80750082	0.07	0.024	6.9239E-33
Tnfrsf8	4.2456E-37	2.97608791	0.026	0.004	9.3145E-33
Sccpdh	8.2996E-37	2.77216848	0.036	0.008	1.8209E-32
Akna	1.1121E-36	1.09222334	0.314	0.214	2.4399E-32
Lgals1	1.6471E-36	0.55773568	0.604	0.512	3.6135E-32
Zfyve16	2.2645E-36	1.60710546	0.147	0.073	4.9682E-32
Dnajb13	2.4302E-36	2.83192618	0.029	0.006	5.3316E-32
Tm2d3	4.0185E-36	1.58069379	0.136	0.065	8.8162E-32
Cdk2ap2	1.417E-35	0.59741895	0.494	0.404	3.1087E-31
Plagl1	2.1537E-35	2.27089246	0.055	0.017	4.7251E-31
Fyn	7.8489E-35	0.79325893	0.442	0.347	1.722E-30
Gprin3	8.7178E-35	1.77857483	0.072	0.025	1.9126E-30
Asb2	3.8047E-34	1.73979775	0.107	0.047	8.3471E-30
Mmp9	4.3424E-34	0.52521713	0.09	0.035	9.5268E-30
Nckap1	5.1148E-34	2.36434286	0.039	0.01	1.1221E-29
Akap13	1.4877E-33	0.29588576	0.832	0.752	3.2639E-29
Adipor2	2.3165E-33	1.0623492	0.291	0.199	5.0821E-29

Pcyt1a	3.815E-33	1.60110113	0.245	0.16	8.3697E-29
Il12rb1	4.8918E-33	2.04963334	0.058	0.019	1.0732E-28
Kat6b	4.9042E-33	1.34518873	0.186	0.106	1.0759E-28
Pon2	8.1837E-33	1.17595727	0.233	0.145	1.7954E-28
Rinl	8.3704E-33	0.88095736	0.264	0.167	1.8364E-28
Cyb5a	1.3463E-32	1.02235625	0.295	0.204	2.9536E-28
Cblb	2.9081E-32	0.71044016	0.398	0.292	6.3801E-28
Cnih2	3.7882E-32	3.93161035	0.012	0.001	8.3108E-28
Specc1l	4.1804E-32	0.98703687	0.366	0.279	9.1713E-28
Glud1	4.3727E-32	0.9492254	0.459	0.389	9.5934E-28
Havcr2	8.7063E-32	1.19536469	0.259	0.171	1.9101E-27
Sema4b	9.0376E-32	1.43231746	0.109	0.049	1.9828E-27
Aplp1	9.8627E-32	2.23825502	0.037	0.009	2.1638E-27
Ube4b	1.6336E-31	1.51680619	0.134	0.068	3.5839E-27
Selenow	1.6405E-31	0.56214939	0.575	0.521	3.5992E-27
Rnf152	2.0694E-31	3.1632378	0.02	0.003	4.54E-27
Kctd12	3.4065E-31	0.54765784	0.502	0.398	7.4736E-27
Raf1	6.2669E-31	1.11265587	0.24	0.153	1.3749E-26
Cers4	7.9567E-31	1.04232689	0.18	0.1	1.7456E-26
Gpr155	1.0943E-30	2.15240782	0.042	0.012	2.4007E-26
Col4a3bp	1.4142E-30	1.10314346	0.247	0.161	3.1025E-26
Zbtb1	1.9083E-30	1.1724643	0.219	0.135	4.1866E-26
Notch1	2.0723E-30	1.3375362	0.192	0.114	4.5464E-26
Igsf9b	7.2764E-30	2.94070835	0.019	0.003	1.5964E-25
Timp2	8.6106E-30	0.81800186	0.216	0.131	1.8891E-25
Ulk1	1.054E-29	1.11637954	0.178	0.102	2.3123E-25
Ubash3b	1.2595E-29	1.60701032	0.165	0.094	2.7632E-25
Osbpl9	2.1562E-29	1.05932066	0.306	0.222	4.7304E-25
Rhoh	2.1857E-29	0.64644087	0.379	0.274	4.7952E-25
Traf3	2.5292E-29	1.12800593	0.223	0.142	5.5488E-25
Pde4d	2.8979E-29	0.8776983	0.254	0.163	6.3576E-25
Il2rg	3.2104E-29	0.55463273	0.549	0.474	7.0433E-25
Pla2g16	4.0101E-29	1.12245029	0.269	0.185	8.7977E-25
Mrpl33	4.152E-29	0.65718787	0.497	0.437	9.1092E-25
Dgkh	5.9426E-29	1.88147142	0.092	0.041	1.3038E-24
Ppp1r16b	6.027E-29	0.57498062	0.431	0.318	1.3223E-24
Gnpnat1	6.3545E-29	1.75868115	0.096	0.043	1.3941E-24
N4bp1	7.1929E-29	0.86613579	0.314	0.228	1.578E-24
Srrm2	7.985E-29	0.38877177	0.746	0.726	1.7518E-24
Ankrd12	8.2104E-29	0.84123014	0.366	0.273	1.8013E-24
Tspan32	8.3159E-29	1.4835793	0.091	0.04	1.8244E-24
Wls	1.6105E-28	1.28937823	0.155	0.086	3.5333E-24
Acot6	1.6189E-28	3.81468282	0.012	0.001	3.5518E-24
Cpd	1.6258E-28	1.43890501	0.171	0.099	3.5667E-24
Atp6v0d2	1.699E-28	3.57999455	0.015	0.002	3.7273E-24
Reep5	2.5538E-28	0.76948979	0.466	0.403	5.6029E-24
Nfil3	2.8551E-28	0.94589648	0.372	0.293	6.2638E-24
Cirbp	3.1702E-28	0.78410353	0.386	0.299	6.9551E-24

Cpox	3.3147E-28	1.61994461	0.092	0.041	7.2721E-24
Krit1	4.2319E-28	0.8535512	0.377	0.29	9.2844E-24
Fcho2	4.5834E-28	1.16924363	0.235	0.156	1.0055E-23
Il21r	1.0218E-27	0.77027701	0.409	0.32	2.2416E-23
Fip1l1	1.4381E-27	0.88506725	0.367	0.286	3.1551E-23
Fam118a	1.5023E-27	1.64392401	0.077	0.032	3.2959E-23
Ndrg2	1.7212E-27	2.54061375	0.021	0.004	3.7762E-23
Prpf18	2.2508E-27	1.08182648	0.212	0.134	4.938E-23
Gimap9	2.4334E-27	0.89125403	0.217	0.134	5.3386E-23
Tfrc	3.024E-27	1.31386621	0.169	0.098	6.6344E-23
Fam3c	3.8447E-27	1.29474923	0.144	0.078	8.4348E-23
Pdcd1	4.0682E-27	1.0371629	0.146	0.078	8.9252E-23
Mcu	5.7471E-27	1.61990204	0.075	0.031	1.2609E-22
Hdac7	6.0561E-27	1.18937225	0.147	0.08	1.3287E-22
Cdk6	6.9404E-27	1.18406399	0.2	0.124	1.5227E-22
Cd200	9.8878E-27	1.4368998	0.113	0.056	2.1693E-22
Fam69c	1.0288E-26	4.1234502	0.01	0.001	2.2571E-22
Ddx24	1.1107E-26	0.56923125	0.558	0.506	2.4369E-22
Ly75	1.1709E-26	1.6011122	0.104	0.05	2.5689E-22
Phc3	1.3493E-26	0.94151814	0.219	0.138	2.9602E-22
Naa20	1.3958E-26	1.39493993	0.171	0.102	3.0622E-22
Rabac1	1.825E-26	0.69829941	0.497	0.436	4.004E-22
Snx9	2.3959E-26	1.19154512	0.191	0.118	5.2563E-22
Mxd1	3.1506E-26	0.29347205	0.498	0.412	6.912E-22
Bclaf3	3.5617E-26	1.52642618	0.126	0.067	7.8141E-22
Asxl1	3.7657E-26	1.35734053	0.145	0.081	8.2617E-22
4932438H23R	4.6846E-26	2.04014943	0.045	0.014	1.0278E-21
Irf2bpl	5.4485E-26	1.22277669	0.159	0.091	1.1953E-21
Ssbp3	7.4959E-26	1.08779919	0.181	0.109	1.6445E-21
Anp32a	8.8596E-26	0.75850226	0.466	0.4	1.9437E-21
Slc16a6	1.0682E-25	1.00112745	0.276	0.194	2.3436E-21
Rasa1	1.3354E-25	1.26961831	0.159	0.092	2.9297E-21
Mid2	1.3635E-25	3.2082758	0.015	0.002	2.9914E-21
Gbp10	1.6132E-25	3.51475652	0.01	0.001	3.5392E-21
Prr13	1.6764E-25	0.61103645	0.49	0.423	3.6779E-21
Skap1	1.7999E-25	0.61777928	0.32	0.224	3.9488E-21
Atp9a	1.806E-25	2.44989093	0.026	0.006	3.9623E-21
Tax1bp1	1.871E-25	0.39502578	0.685	0.648	4.1048E-21
Dnajc1	2.0621E-25	1.08856433	0.275	0.198	4.524E-21
Rasgef1a	2.2061E-25	2.35401087	0.027	0.007	4.84E-21
Setd1b	2.5169E-25	1.1564874	0.134	0.072	5.5219E-21
Ncoa3	2.6762E-25	1.08932326	0.223	0.148	5.8713E-21
Ccnd2	3.255E-25	0.72837223	0.395	0.306	7.141E-21
Src	3.4228E-25	2.20700572	0.065	0.026	7.5093E-21
Ppp1r12a	3.8339E-25	0.68027344	0.427	0.353	8.4113E-21
Sh3kbp1	3.9865E-25	0.73082922	0.378	0.297	8.746E-21
Il27ra	4.1625E-25	1.22633063	0.109	0.054	9.1321E-21
Sept1	4.2046E-25	0.50707963	0.41	0.306	9.2245E-21

Grb2	4.7687E-25	0.62835432	0.543	0.499	1.0462E-20
Lbh	5.6274E-25	0.82019179	0.266	0.181	1.2346E-20
Rab27a	6.7603E-25	1.52521688	0.097	0.046	1.4831E-20
Gm35363	9.9906E-25	3.25935432	0.014	0.002	2.1918E-20
Gm17767	1.0104E-24	3.27939445	0.014	0.002	2.2166E-20
Slc52a3	1.0129E-24	2.18340356	0.042	0.014	2.2222E-20
Zbtb38	1.6295E-24	1.26958412	0.145	0.082	3.5749E-20
Clec2d	2.1698E-24	0.68282964	0.433	0.36	4.7604E-20
Gm28884	2.7056E-24	3.00351225	0.022	0.005	5.9358E-20
Bpgm	3.37E-24	1.80729073	0.052	0.019	7.3934E-20
Myh9	3.6581E-24	0.45042434	0.624	0.582	8.0255E-20
Fam117b	4.4099E-24	0.94262869	0.328	0.258	9.6748E-20
Perp	6.2013E-24	2.25436306	0.031	0.009	1.3605E-19
Gbp4	6.6733E-24	1.08719828	0.192	0.12	1.4641E-19
Nr1d2	6.9407E-24	0.92916783	0.25	0.171	1.5227E-19
Ptpn5	7.6915E-24	3.41041285	0.014	0.002	1.6874E-19
Paxbp1	8.3075E-24	0.78631434	0.306	0.224	1.8226E-19
Ccm2	8.4171E-24	1.04696872	0.196	0.125	1.8466E-19
Anapc1	1.9064E-23	1.16403673	0.164	0.098	4.1825E-19
Socs5	1.9432E-23	1.47875702	0.081	0.037	4.2633E-19
Tbc1d10c	2.0247E-23	1.13000289	0.124	0.066	4.442E-19
Serpina3g	3.0048E-23	1.41207233	0.141	0.081	6.5921E-19
Ldha	3.3458E-23	0.52765915	0.536	0.491	7.3402E-19
Ypel2	3.5191E-23	1.91595295	0.046	0.016	7.7205E-19
H1f0	3.5268E-23	1.44554075	0.155	0.092	7.7374E-19
Cast	4.364E-23	0.78009259	0.356	0.283	9.5743E-19
Aplp2	7.0488E-23	0.70023903	0.402	0.331	1.5464E-18
Etaa1	7.7562E-23	1.23829307	0.114	0.06	1.7016E-18
Adarb1	8.5172E-23	2.26679281	0.03	0.008	1.8686E-18
Ass1	1.1584E-22	0.98293808	0.208	0.137	2.5414E-18
Pik3ca	1.4379E-22	1.20786866	0.164	0.1	3.1545E-18
Klhl28	1.5337E-22	1.32319707	0.123	0.068	3.3648E-18
Snx13	3.153E-22	1.47378913	0.105	0.054	6.9174E-18
Ica1	3.2249E-22	3.00044009	0.014	0.002	7.0751E-18
Smad7	4.1304E-22	0.62274412	0.395	0.314	9.0616E-18
Crip1	6.6037E-22	0.40580732	0.731	0.649	1.4488E-17
Mllt3	8.0915E-22	0.94309829	0.179	0.111	1.7752E-17
Sh2d2a	9.0644E-22	0.33442024	0.41	0.298	1.9886E-17
Stk24	9.2925E-22	0.58527841	0.412	0.333	2.0387E-17
Ezr	9.4094E-22	0.5673455	0.501	0.439	2.0643E-17
Abtb1	1.1147E-21	1.32585284	0.094	0.047	2.4455E-17
Smc4	1.1225E-21	0.80586173	0.259	0.182	2.4627E-17
Ets2	1.2674E-21	0.40260243	0.38	0.299	2.7806E-17
Sacs	1.8355E-21	1.25595495	0.11	0.059	4.0268E-17
Bnip3l	1.9831E-21	0.71127335	0.382	0.314	4.3507E-17
Ywhaz	2.0982E-21	0.37099485	0.722	0.711	4.6032E-17
Rab6b	2.1839E-21	1.95614869	0.038	0.013	4.7913E-17
Rab8b	2.4496E-21	0.35257289	0.642	0.586	5.3742E-17

Gbp6	3.0355E-21	1.89612622	0.04	0.014	6.6596E-17
Tff1	3.4886E-21	2.0285385	0.039	0.013	7.6537E-17
Cir1	3.6364E-21	0.7756669	0.306	0.229	7.978E-17
Msl3l2	5.0269E-21	2.54294505	0.025	0.007	1.1028E-16
March3	5.1383E-21	1.93234495	0.041	0.014	1.1273E-16
Rexo5	6.1627E-21	1.98120887	0.035	0.011	1.352E-16
Grik5	7.7696E-21	2.17314526	0.025	0.007	1.7046E-16
Nfkb1	8.0234E-21	0.3467585	0.696	0.642	1.7602E-16
Ypel3	8.7683E-21	0.71108383	0.3	0.224	1.9237E-16
Sgip1	1.2757E-20	1.68976231	0.055	0.022	2.7988E-16
Donson	1.3852E-20	1.19238565	0.137	0.081	3.0391E-16
Ctdspl2	1.4165E-20	1.0500227	0.201	0.134	3.1076E-16
Rhof	1.6435E-20	1.1006912	0.15	0.091	3.6057E-16
Ldlrad4	1.7384E-20	1.1761011	0.094	0.048	3.8138E-16
6330403K07R	2.063E-20	2.69265706	0.021	0.005	4.526E-16
Cbx4	2.5764E-20	0.94155272	0.183	0.117	5.6523E-16
Bcl2l1	3.0928E-20	1.08085498	0.177	0.114	6.7852E-16
Mat2b	4.3863E-20	0.67666544	0.32	0.245	9.623E-16
March7	6.1346E-20	0.5880103	0.491	0.449	1.3459E-15
Pbxip1	6.7565E-20	0.86450653	0.262	0.192	1.4823E-15
Xbp1	8.5675E-20	0.65489127	0.438	0.385	1.8796E-15
Man1a	9.3247E-20	0.7682577	0.337	0.27	2.0457E-15
Ppme1	1.1647E-19	1.14829517	0.162	0.102	2.5552E-15
Dsel	1.2086E-19	2.07833355	0.027	0.008	2.6515E-15
L1cam	1.2095E-19	1.65045805	0.058	0.024	2.6535E-15
Dclk2	1.8846E-19	1.66610076	0.033	0.011	4.1347E-15
Ski	2.0969E-19	0.89743151	0.231	0.163	4.6003E-15
Rbfa	2.1415E-19	1.16091367	0.153	0.096	4.6982E-15
Acsbg1	2.3494E-19	1.24538866	0.079	0.038	5.1543E-15
Peak1	2.4808E-19	0.95669432	0.215	0.149	5.4427E-15
Sesn1	2.5218E-19	1.21265755	0.098	0.052	5.5326E-15
Spint2	2.8491E-19	1.33613062	0.118	0.068	6.2506E-15
Ccr6	3.0252E-19	1.05293169	0.085	0.042	6.6369E-15
Cd99l2	3.8213E-19	1.90233806	0.041	0.015	8.3835E-15
Pear1	3.9588E-19	1.42206004	0.051	0.02	8.6852E-15
Ythdc1	4.5748E-19	0.54121616	0.542	0.509	1.0037E-14
Mtmr3	5.1125E-19	1.08687284	0.169	0.108	1.1216E-14
Sbno1	5.2268E-19	0.64474278	0.411	0.352	1.1467E-14
Ptpn18	5.5578E-19	0.4154136	0.623	0.615	1.2193E-14
Birc2	6.1294E-19	0.65911397	0.395	0.33	1.3447E-14
Ttbk2	6.1323E-19	1.7647089	0.047	0.019	1.3454E-14
Ralbp1	6.5869E-19	0.8692735	0.294	0.23	1.4451E-14
Slc26a2	6.8567E-19	1.15918759	0.164	0.106	1.5043E-14
Grap2	7.0982E-19	0.67339105	0.253	0.179	1.5573E-14
Fgfr1op2	8.4298E-19	0.75074311	0.363	0.306	1.8494E-14
Zfp292	8.8761E-19	0.65279448	0.319	0.246	1.9473E-14
Tsc22d4	9.8433E-19	0.66547817	0.347	0.278	2.1595E-14
Retreg1	9.9866E-19	0.56770521	0.321	0.246	2.191E-14

Cap2	1.2254E-18	3.3740213	0.01	0.001	2.6884E-14
Tctex1d1	1.2288E-18	2.58070637	0.018	0.004	2.6959E-14
Cipc	1.3429E-18	1.05458537	0.155	0.097	2.9461E-14
Asxl2	1.4856E-18	0.8997625	0.237	0.172	3.2593E-14
Klf13	1.6037E-18	0.70765797	0.371	0.311	3.5184E-14
Smap2	1.6532E-18	0.64117504	0.411	0.35	3.627E-14
Mgea5	1.8364E-18	0.64719889	0.403	0.342	4.0289E-14
Csnk1g3	1.8574E-18	0.78711522	0.269	0.2	4.0749E-14
Slamf6	1.9799E-18	0.89753289	0.146	0.089	4.3438E-14
Epas1	2.4851E-18	1.15839794	0.154	0.096	5.4521E-14
Rab11a	2.5324E-18	0.95087449	0.274	0.215	5.5558E-14
Tasp1	2.9857E-18	1.42226812	0.061	0.028	6.5504E-14
Utrn	3.2012E-18	0.57725374	0.318	0.241	7.0232E-14
Il4ra	3.4056E-18	0.66305894	0.448	0.398	7.4715E-14
Atp11b	4.2955E-18	0.71257887	0.367	0.305	9.4239E-14
Plcb4	6.5537E-18	1.31797058	0.082	0.041	1.4378E-13
Plekha7	6.6848E-18	2.42857409	0.016	0.003	1.4666E-13
Bod1l	7.3414E-18	0.77875039	0.283	0.216	1.6106E-13
Stat4	7.6638E-18	0.49009728	0.455	0.382	1.6814E-13
Vopp1	7.7856E-18	1.31042757	0.124	0.075	1.7081E-13
Gpcpd1	9.1677E-18	0.80694204	0.3	0.239	2.0113E-13
Stk10	1.094E-17	0.73897597	0.268	0.2	2.4002E-13
Raph1	1.1602E-17	1.20607642	0.199	0.14	2.5454E-13
Ptpn22	1.1685E-17	0.26088049	0.485	0.38	2.5636E-13
Lrch3	1.3863E-17	1.12134035	0.141	0.088	3.0413E-13
Nfatc3	1.5758E-17	0.46018285	0.369	0.29	3.4572E-13
Ogt	1.5892E-17	0.59152552	0.382	0.315	3.4865E-13
Ift80	1.6813E-17	1.3610968	0.055	0.024	3.6885E-13
Rrad	2.0048E-17	0.94918453	0.146	0.091	4.3983E-13
Polg	2.0795E-17	1.08859499	0.093	0.05	4.5623E-13
Ptms	2.2086E-17	0.70728427	0.366	0.308	4.8455E-13
Hoxb4	2.2742E-17	2.34995773	0.029	0.009	4.9893E-13
Stn1	2.3261E-17	1.26599896	0.08	0.04	5.1031E-13
Galnt6	2.3367E-17	1.26422452	0.132	0.081	5.1266E-13
Sit1	2.3554E-17	1.46158944	0.055	0.024	5.1675E-13
Psip1	2.6585E-17	0.80532798	0.208	0.143	5.8325E-13
Phactr2	2.8988E-17	1.54179006	0.118	0.071	6.3596E-13
Zfp54	2.9251E-17	2.45374221	0.016	0.004	6.4175E-13
Ccr8	3.2181E-17	2.04430283	0.023	0.007	7.0601E-13
Myo1g	3.8456E-17	1.06973211	0.271	0.214	8.4369E-13
Fam96b	3.9724E-17	0.885293	0.268	0.208	8.7151E-13
Ap3b1	4.523E-17	1.20793107	0.205	0.149	9.923E-13
Cnst	4.6503E-17	1.38302025	0.067	0.032	1.0202E-12
Rsbn1l	4.6831E-17	0.57170391	0.419	0.36	1.0274E-12
Fnbp1	5.3324E-17	0.55180903	0.448	0.4	1.1699E-12
Isy1	7.6097E-17	0.55165272	0.467	0.423	1.6695E-12
Myl12a	7.7626E-17	0.41410218	0.585	0.564	1.703E-12
Ssh2	9.1206E-17	0.5336831	0.41	0.34	2.001E-12

Rap1b	9.1267E-17	0.29725523	0.67	0.64	2.0023E-12
Map1lc3b	9.5155E-17	0.37263765	0.605	0.586	2.0876E-12
Cdc42se2	9.7048E-17	0.74650572	0.346	0.293	2.1291E-12
Ankib1	1.1554E-16	1.06445401	0.148	0.095	2.5347E-12
Galnt2	1.1728E-16	2.2533554	0.022	0.006	2.5729E-12
Edem1	1.2999E-16	0.73457945	0.335	0.28	2.8519E-12
Fosl2	1.5541E-16	0.36483492	0.566	0.515	3.4095E-12
Gm16286	1.726E-16	0.749993	0.315	0.258	3.7867E-12
Cdkn2c	2.1494E-16	1.86265901	0.043	0.018	4.7155E-12
Ppp1r3f	2.2292E-16	2.24314991	0.022	0.006	4.8907E-12
Nrd1	2.3038E-16	0.77166829	0.341	0.291	5.0544E-12
Arl4a	2.3965E-16	1.10212741	0.17	0.114	5.2578E-12
Slc39a4	2.6645E-16	1.43659338	0.055	0.025	5.8455E-12
Sfmbt2	3.2974E-16	2.08727544	0.024	0.007	7.2341E-12
Itpr2	3.3946E-16	0.83002329	0.269	0.205	7.4475E-12
C1qtnf12	3.4919E-16	1.67961905	0.042	0.017	7.6608E-12
Ankrd13c	3.5109E-16	1.03423825	0.18	0.124	7.7025E-12
Dusp14	3.866E-16	1.87551313	0.036	0.013	8.4817E-12
AC149090.1	3.9608E-16	0.43842807	0.352	0.285	8.6895E-12
Lnx2	3.988E-16	1.51522921	0.081	0.043	8.7493E-12
Son	4.334E-16	0.30282958	0.718	0.721	9.5083E-12
Plagl2	4.5352E-16	1.048614	0.121	0.073	9.9498E-12
Fam83g	5.1498E-16	2.35569713	0.015	0.004	1.1298E-11
Ip6k1	5.1556E-16	0.7699284	0.226	0.164	1.1311E-11
Auh	5.4633E-16	1.08929732	0.184	0.13	1.1986E-11
Oaz2	6.0994E-16	1.5501734	0.07	0.035	1.3382E-11
Igf1r	6.8221E-16	0.90998986	0.11	0.065	1.4967E-11
Sec62	7.7378E-16	0.43202581	0.557	0.532	1.6976E-11
Rapgef6	7.795E-16	0.6498902	0.346	0.286	1.7101E-11
Scyl2	8.5375E-16	1.14363951	0.145	0.094	1.873E-11
Angptl2	9.0526E-16	2.19656383	0.02	0.005	1.9861E-11
Gxylt1	9.6991E-16	0.95234498	0.19	0.133	2.1279E-11
Fkbp1a	9.8498E-16	0.72459526	0.342	0.29	2.1609E-11
Oxr1	1.1932E-15	1.01597073	0.132	0.083	2.6178E-11
Rnf138	1.1934E-15	0.79713176	0.258	0.196	2.6181E-11
Gpx4	1.2451E-15	0.47709811	0.565	0.536	2.7316E-11
Clk1	1.264E-15	0.30547671	0.696	0.683	2.773E-11
Serpina3f	1.2723E-15	1.58587099	0.045	0.019	2.7913E-11
Ndrg1	1.32E-15	0.86681754	0.22	0.161	2.8959E-11
Fus	1.3716E-15	0.28112103	0.748	0.75	3.0092E-11
Nrn1	1.4888E-15	2.32332778	0.015	0.004	3.2662E-11
Crmp1	1.5072E-15	1.40101931	0.053	0.024	3.3067E-11
F830016B08Ri	1.5355E-15	2.39358467	0.013	0.003	3.3688E-11
Pkm	1.6175E-15	0.47408451	0.475	0.441	3.5486E-11
F2rl1	1.6401E-15	1.95985101	0.025	0.008	3.5981E-11
Tgif1	1.7183E-15	0.59578531	0.414	0.366	3.7699E-11
Jakmip1	2.8681E-15	0.67039754	0.203	0.141	6.2924E-11
Dock2	2.9352E-15	0.53104362	0.462	0.42	6.4395E-11

Epcam	2.9667E-15	1.95595901	0.057	0.027	6.5087E-11
Kif23	3.3334E-15	1.08322527	0.096	0.054	7.3132E-11
Kansl1	3.6076E-15	0.67026896	0.356	0.303	7.9146E-11
Arrdc1	4.6018E-15	1.20487517	0.132	0.084	1.0096E-10
Sigirr	4.9671E-15	1.37608157	0.059	0.029	1.0897E-10
Bmpr2	5.3805E-15	1.43317153	0.112	0.069	1.1804E-10
Faap100	5.6316E-15	1.29256305	0.089	0.05	1.2355E-10
Madd	6.2917E-15	0.72427178	0.293	0.234	1.3803E-10
Tap1	6.3758E-15	0.71200272	0.316	0.262	1.3988E-10
Mdfic	6.6846E-15	1.00685759	0.227	0.174	1.4665E-10
Kif20b	7.2466E-15	1.58644846	0.047	0.021	1.5898E-10
Pmepa1	8.1494E-15	0.59797927	0.329	0.267	1.7879E-10
Anxa6	8.3334E-15	0.72254246	0.308	0.254	1.8283E-10
Serinc5	8.383E-15	1.56002232	0.052	0.024	1.8392E-10
Rgs2	8.9123E-15	0.6067217	0.525	0.485	1.9553E-10
Sesn3	8.9505E-15	0.60919969	0.284	0.218	1.9637E-10
Dusp10	1.1215E-14	0.76907025	0.171	0.115	2.4605E-10
Pvrig	1.2609E-14	2.58274072	0.015	0.004	2.7663E-10
Mia2	1.2694E-14	0.78114034	0.321	0.272	2.785E-10
Gm10605	1.5256E-14	2.96150536	0.012	0.002	3.347E-10
Tmem154	1.6117E-14	1.3667467	0.061	0.03	3.536E-10
Vsig10	1.646E-14	2.43333217	0.022	0.007	3.6111E-10
Elovl5	1.6606E-14	0.80402205	0.289	0.236	3.6432E-10
Fam20a	1.6862E-14	2.30388204	0.026	0.009	3.6993E-10
Phtf1	1.9094E-14	1.32272718	0.075	0.04	4.189E-10
Gstp3	2.0383E-14	1.03309482	0.092	0.052	4.4718E-10
Fam210a	2.0579E-14	1.42169887	0.061	0.03	4.5147E-10
Ccdc92	2.318E-14	1.88940893	0.023	0.007	5.0854E-10
Chst2	2.6311E-14	1.39245161	0.053	0.025	5.7725E-10
Ifi27	2.6686E-14	0.80172856	0.25	0.196	5.8546E-10
Mpzl1	2.7638E-14	2.20376453	0.015	0.004	6.0634E-10
Smpdl3a	2.9178E-14	0.87619926	0.235	0.182	6.4013E-10
Ttc39b	3.0058E-14	0.69460017	0.259	0.201	6.5944E-10
Matk	3.9342E-14	1.77145964	0.072	0.039	8.6313E-10
Ppp1r3b	4.5015E-14	0.74202837	0.11	0.067	9.8758E-10
Ppm1b	5.3117E-14	0.91021487	0.181	0.128	1.1653E-09
Ncs1	5.5955E-14	2.71443417	0.018	0.005	1.2276E-09
Cdk17	1.1088E-13	0.82151511	0.195	0.141	2.4325E-09
Pycr2	1.2767E-13	1.18661569	0.081	0.045	2.801E-09
Gm19557	1.3033E-13	1.72977593	0.03	0.011	2.8592E-09
Slc4a8	1.411E-13	1.924267	0.036	0.015	3.0956E-09
Ubtd2	1.4326E-13	1.56917524	0.036	0.015	3.1429E-09
Arfgef1	1.6615E-13	0.7326731	0.264	0.21	3.6451E-09
Gm48236	2.7524E-13	1.61913592	0.031	0.012	6.0384E-09
Ccng1	3.1551E-13	0.8544827	0.168	0.118	6.9219E-09
Clcn3	3.3937E-13	0.78828493	0.233	0.179	7.4455E-09
Trim59	3.6676E-13	0.79318847	0.157	0.106	8.0464E-09
Carmil2	3.8851E-13	1.21097857	0.05	0.023	8.5236E-09

Rin2	3.9098E-13	1.7181008	0.086	0.051	8.5776E-09
Gprasp2	4.1141E-13	2.65600015	0.01	0.002	9.026E-09
Rock1	4.1778E-13	0.52287954	0.434	0.398	9.1657E-09
Fbxo33	4.24E-13	0.83614568	0.166	0.116	9.3022E-09
Mkrn2	4.3373E-13	1.32995992	0.08	0.045	9.5155E-09
Tnrc6a	4.4052E-13	0.75726016	0.254	0.201	9.6646E-09
Arrb1	4.557E-13	1.26299345	0.123	0.081	9.9975E-09
Ncapd3	4.627E-13	1.34372706	0.093	0.056	1.0151E-08
Hnrnpll	4.7036E-13	0.91166112	0.214	0.164	1.0319E-08
Xpo4	5.3243E-13	0.94570835	0.137	0.091	1.1681E-08
AI467606	5.4953E-13	0.91602309	0.125	0.08	1.2056E-08
Rragd	5.9381E-13	1.96860765	0.022	0.007	1.3028E-08
Ccser2	7.2519E-13	0.9064347	0.169	0.119	1.591E-08
Cd320	8.0647E-13	1.59648475	0.046	0.022	1.7693E-08
F2r	8.2369E-13	1.0039629	0.111	0.07	1.8071E-08
Adk	8.436E-13	1.00034459	0.102	0.062	1.8508E-08
Tmem65	9.4093E-13	1.11969645	0.116	0.075	2.0643E-08
Tnfrsf13b	9.5345E-13	1.13369826	0.101	0.062	2.0918E-08
Gm28053	1.0598E-12	1.99465668	0.02	0.006	2.3251E-08
Sri	1.0767E-12	0.58322634	0.408	0.38	2.3623E-08
D5Ertd579e	1.0798E-12	1.15084449	0.121	0.079	2.3689E-08
Samhd1	1.1294E-12	0.42722302	0.419	0.371	2.4777E-08
Adam19	1.1613E-12	1.18481283	0.129	0.087	2.5478E-08
Lgals7	1.2565E-12	1.21116385	0.048	0.023	2.7566E-08
Hey1	1.5228E-12	1.95006697	0.02	0.006	3.3408E-08
Ubxn7	1.5735E-12	1.0598497	0.128	0.085	3.4521E-08
Lztfl1	1.7834E-12	0.97621961	0.224	0.175	3.9127E-08
Tnk2	2.0223E-12	1.08465859	0.072	0.04	4.4367E-08
Polk	2.1646E-12	1.41701463	0.053	0.027	4.749E-08
Vamp1	2.2593E-12	1.25125164	0.046	0.022	4.9568E-08
Mynn	2.2829E-12	1.07268963	0.116	0.075	5.0086E-08
Gm26669	2.3146E-12	0.81613676	0.165	0.117	5.0779E-08
Spata13	2.4633E-12	0.65899286	0.178	0.127	5.4043E-08
Secisbp2l	2.7007E-12	0.92101325	0.17	0.123	5.9251E-08
Erc1	2.9479E-12	1.34778116	0.05	0.025	6.4673E-08
Itm2c	3.2583E-12	0.73284148	0.331	0.291	7.1484E-08
1500009L16Ri	3.9693E-12	1.31970742	0.024	0.009	8.7083E-08
Bmpr1a	4.0748E-12	1.93524078	0.018	0.005	8.9397E-08
Rac2	4.8729E-12	0.35316084	0.586	0.58	1.0691E-07
Pik3c2a	4.9394E-12	0.8967899	0.194	0.146	1.0836E-07
Nol10	5.0115E-12	1.11436528	0.125	0.084	1.0995E-07
Jmy	5.7671E-12	0.8138226	0.205	0.156	1.2652E-07
Spty2d1	5.9537E-12	0.6494504	0.345	0.301	1.3062E-07
Smim15	6.4903E-12	0.80945365	0.192	0.143	1.4239E-07
Fam92a	7.3244E-12	1.16052175	0.072	0.041	1.6069E-07
Arl6ip1	8.3522E-12	0.36111971	0.545	0.53	1.8324E-07
Gpm6b	8.4934E-12	1.70726236	0.021	0.007	1.8634E-07
Igsf23	9.1861E-12	1.98637555	0.012	0.003	2.0153E-07

Myo1e	1.056E-11	0.79611418	0.221	0.171	2.3168E-07
Pglyrp1	1.0699E-11	0.85350338	0.11	0.07	2.3472E-07
Arhgef1	1.0732E-11	0.47030054	0.348	0.294	2.3545E-07
Flnb	1.1371E-11	1.48353536	0.102	0.066	2.4946E-07
Clstn1	1.2983E-11	1.58383822	0.039	0.018	2.8484E-07
Elf4	1.3069E-11	1.0073799	0.137	0.095	2.8673E-07
Lsm8	1.3833E-11	0.76215591	0.225	0.176	3.0347E-07
Prrc2b	1.4207E-11	0.7022265	0.234	0.183	3.1168E-07
Dctn4	1.4412E-11	0.63043102	0.37	0.332	3.1619E-07
Il18r1	1.5258E-11	0.54227625	0.266	0.206	3.3475E-07
Igf2r	1.769E-11	0.81697509	0.155	0.109	3.8809E-07
Dock10	1.7997E-11	0.51279503	0.472	0.455	3.9484E-07
Irf1	1.8825E-11	0.5063968	0.281	0.229	4.13E-07
Npnt	2.1351E-11	1.37163419	0.051	0.026	4.6841E-07
Ppp1cc	2.2018E-11	0.40285764	0.446	0.395	4.8305E-07
Stxbp5	2.2525E-11	1.17304894	0.084	0.05	4.9418E-07
Gpbp1l1	2.8453E-11	0.7264331	0.236	0.189	6.2423E-07
Lats2	3.0539E-11	0.91241897	0.178	0.133	6.7E-07
Fam110a	3.1952E-11	0.76802337	0.188	0.14	7.01E-07
Dtnb	3.2943E-11	1.1935897	0.066	0.037	7.2273E-07
Trim46	3.3092E-11	2.37924683	0.018	0.006	7.2601E-07
Dapp1	3.5247E-11	0.72582253	0.239	0.192	7.7329E-07
Dek	4.1566E-11	0.40640282	0.511	0.49	9.1191E-07
Klc3	4.2258E-11	1.70647619	0.017	0.005	9.271E-07
Vps26b	4.3402E-11	1.16714964	0.073	0.042	9.5219E-07
Zfp945	4.5912E-11	1.04416683	0.069	0.039	1.0073E-06
Tbrg1	4.8051E-11	0.68658982	0.258	0.21	1.0542E-06
Gnpda2	5.0342E-11	0.93352059	0.097	0.061	1.1044E-06
Nfatc2	5.1958E-11	1.01210268	0.113	0.074	1.1399E-06
Tcp11l2	5.1982E-11	0.63469261	0.268	0.219	1.1404E-06
Rapgef3	5.4966E-11	2.26998433	0.013	0.003	1.2059E-06
Fbxl3	5.6788E-11	0.77587518	0.235	0.19	1.2459E-06
Cyld	5.7244E-11	0.56345368	0.355	0.315	1.2559E-06
Abracl	5.8549E-11	0.45928578	0.494	0.489	1.2845E-06
Trp53i11	5.873E-11	0.83286632	0.09	0.055	1.2885E-06
Acot2	5.8806E-11	1.10714112	0.064	0.036	1.2901E-06
Ide	5.9176E-11	0.95109215	0.111	0.073	1.2983E-06
Birc3	6.0814E-11	0.51433445	0.451	0.418	1.3342E-06
Dkkl1	6.8446E-11	1.26603195	0.055	0.03	1.5016E-06
Ppm1l	7.2097E-11	1.50227626	0.03	0.013	1.5817E-06
Bhlhe41	8.4694E-11	1.57851006	0.038	0.018	1.8581E-06
Arpp19	1.0427E-10	0.41029465	0.496	0.481	2.2875E-06
2610021A01R	1.1477E-10	1.54479284	0.03	0.013	2.518E-06
Acbd5	1.1588E-10	0.73006265	0.212	0.165	2.5424E-06
Ifnar1	1.1855E-10	0.55057892	0.385	0.353	2.6009E-06
Jak3	1.2128E-10	1.05244532	0.09	0.056	2.6608E-06
Lrrc61	1.2827E-10	1.66036526	0.034	0.015	2.8141E-06
Arntl	1.3558E-10	0.8730252	0.121	0.081	2.9744E-06

Kmt2a	1.4941E-10	0.48390492	0.372	0.328	3.278E-06
Akap12	1.5497E-10	1.24767493	0.036	0.016	3.3998E-06
Sec23a	1.6727E-10	1.46632295	0.057	0.031	3.6698E-06
Smarce1	1.7058E-10	0.64720407	0.308	0.27	3.7424E-06
Nxpe3	1.8471E-10	1.04777493	0.051	0.026	4.0524E-06
Tnfsf8	1.8708E-10	0.44268209	0.178	0.129	4.1044E-06
Apbb1	1.9148E-10	2.14365864	0.01	0.002	4.2009E-06
Cbarp	2.0804E-10	1.31248394	0.086	0.054	4.5641E-06
Pcsk1	2.2094E-10	1.03564438	0.033	0.015	4.8473E-06
Cbx7	2.2311E-10	1.00069514	0.077	0.046	4.8949E-06
Cds2	2.4402E-10	0.8045725	0.125	0.086	5.3536E-06
Amn1	3.0174E-10	0.95088245	0.147	0.106	6.6198E-06
Mphosph6	3.1865E-10	1.07890294	0.138	0.099	6.9908E-06
Trat1	3.381E-10	0.79495076	0.124	0.085	7.4175E-06
Ankrd11	3.4051E-10	0.31788912	0.605	0.599	7.4705E-06
D130040H23F	3.8558E-10	1.62259865	0.025	0.01	8.4593E-06
Ttbk1	3.8632E-10	1.83288008	0.011	0.003	8.4756E-06
Gm12840	4.2345E-10	0.43514093	0.281	0.227	9.29E-06
Arel1	4.4876E-10	1.06709221	0.075	0.045	9.8453E-06
1700020I14Ril	4.7817E-10	0.69435678	0.245	0.202	1.0491E-05
Vcpkmt	4.9944E-10	1.03674926	0.106	0.07	1.0957E-05
Slc35d1	5.6131E-10	0.9533052	0.069	0.041	1.2315E-05
Tmsb15b1	6.0261E-10	1.45845596	0.041	0.02	1.3221E-05
Cpe	6.3912E-10	1.54620898	0.018	0.006	1.4022E-05
Ubash3a	6.9018E-10	0.77912363	0.08	0.049	1.5142E-05
1810037I17Ril	7.2474E-10	0.40285534	0.471	0.46	1.59E-05
Khnyn	8.3092E-10	0.99745492	0.107	0.072	1.823E-05
Rbl2	8.5174E-10	0.65324522	0.189	0.143	1.8686E-05
Rasgrp1	8.5237E-10	0.65764689	0.203	0.159	1.87E-05
Cdc40	8.5304E-10	0.67601522	0.284	0.242	1.8715E-05
Aes	8.8815E-10	0.42622085	0.431	0.407	1.9485E-05
Sik2	8.9739E-10	0.80353176	0.146	0.106	1.9688E-05
Slc4a7	9.0581E-10	0.64978734	0.303	0.263	1.9873E-05
Tpm4	9.1262E-10	0.46834697	0.456	0.434	2.0022E-05
Tab2	9.4476E-10	0.44132068	0.447	0.418	2.0727E-05
Chrne	9.6462E-10	1.25394332	0.05	0.027	2.1163E-05
Rorc	9.864E-10	1.39767511	0.03	0.014	2.1641E-05
AW549877	9.8751E-10	0.86107102	0.137	0.097	2.1665E-05
Dhrs3	1.0966E-09	1.01535751	0.14	0.103	2.4058E-05
Arl13b	1.1859E-09	1.02707155	0.141	0.104	2.6017E-05
Prelid1	1.3609E-09	0.55994681	0.332	0.303	2.9858E-05
Pdp1	1.4309E-09	1.46400327	0.047	0.025	3.1391E-05
Abca2	1.4516E-09	1.13681267	0.058	0.033	3.1846E-05
Hipk2	1.6204E-09	1.16687212	0.14	0.103	3.555E-05
Nek7	1.7629E-09	0.68937106	0.236	0.195	3.8676E-05
Fbxo11	1.7899E-09	0.67739171	0.24	0.199	3.9268E-05
Cmas	1.8263E-09	0.81830515	0.214	0.174	4.0067E-05
Kif5c	1.8333E-09	1.63704328	0.021	0.008	4.0221E-05

Mapkbp1	1.8788E-09	1.84286758	0.025	0.01	4.1218E-05
Supt20	2.0809E-09	0.60223916	0.244	0.2	4.5653E-05
Zmym5	2.1511E-09	0.73686549	0.202	0.159	4.7192E-05
Ephx1	2.1595E-09	1.03712694	0.054	0.03	4.7377E-05
Virma	2.2247E-09	0.74885881	0.158	0.118	4.8807E-05
Fgl2	2.3796E-09	0.62852731	0.302	0.261	5.2206E-05
Chic1	2.3925E-09	1.64391835	0.021	0.008	5.2489E-05
Tecpr1	2.5275E-09	0.39304384	0.275	0.222	5.545E-05
Prkcz	2.6011E-09	1.07978254	0.047	0.025	5.7066E-05
Riok1	2.6263E-09	0.4023552	0.361	0.315	5.7618E-05
Erbb3	2.6612E-09	2.44776815	0.011	0.003	5.8384E-05
Ino80d	2.8405E-09	0.68626503	0.262	0.223	6.2317E-05
Rhod	2.8519E-09	1.71987728	0.018	0.006	6.2568E-05
Zfp160	2.9278E-09	1.34719512	0.058	0.033	6.4233E-05
Ebpl	3.6184E-09	1.13403166	0.076	0.048	7.9384E-05
Neil1	3.6412E-09	0.72969272	0.089	0.057	7.9885E-05
Pkp4	3.876E-09	1.02391897	0.068	0.041	8.5036E-05
Gnb1	4.1716E-09	0.39770679	0.504	0.503	9.152E-05
Pfkl	4.4549E-09	0.90734123	0.114	0.079	9.7736E-05
Zfand6	4.4665E-09	0.54701643	0.311	0.272	9.799E-05
Ankra2	4.4872E-09	1.02271026	0.098	0.065	9.8444E-05
Purb	4.5464E-09	0.42640261	0.514	0.513	9.9744E-05
Haus3	4.6075E-09	0.77426666	0.135	0.097	0.00010109
Map9	4.8138E-09	2.16199431	0.022	0.009	0.00010561
Dyrk1b	4.8803E-09	1.34625397	0.045	0.024	0.00010707
H2-Q4	5.1494E-09	0.55626345	0.296	0.256	0.00011297
Park7	6.125E-09	0.57404392	0.338	0.312	0.00013438
Snw1	6.2672E-09	0.53564994	0.348	0.318	0.0001375
Fbxl14	6.3609E-09	0.9680112	0.089	0.058	0.00013955
Gpr45	6.8835E-09	2.14518819	0.011	0.003	0.00015102
Nsd3	7.4348E-09	0.49107241	0.43	0.411	0.00016311
Baz1b	7.6832E-09	0.74859018	0.226	0.185	0.00016856
Rhog	8.0303E-09	0.39635244	0.435	0.416	0.00017618
Ttc14	8.082E-09	0.4262828	0.389	0.354	0.00017731
Traf4	8.3022E-09	0.81311118	0.128	0.092	0.00018214
Cpped1	8.3959E-09	1.46700249	0.032	0.015	0.0001842
BC005537	8.5484E-09	0.35314263	0.502	0.495	0.00018754
Gabpb1	8.7706E-09	0.66290019	0.21	0.169	0.00019242
H2-T22	9.1097E-09	0.63069935	0.273	0.235	0.00019986
Cdipt	9.3921E-09	0.8237857	0.163	0.126	0.00020605
P4ha1	1.0419E-08	0.80065941	0.161	0.123	0.00022859
Pitpnc1	1.0539E-08	0.62033649	0.209	0.167	0.00023121
Ppp2ca	1.0539E-08	0.37259731	0.5	0.492	0.00023122
Cbx3	1.0793E-08	0.4640933	0.324	0.283	0.00023679
Cnot6	1.1011E-08	0.53658155	0.286	0.245	0.00024156
Rgs16	1.2144E-08	1.19741951	0.035	0.017	0.00026643
Slc45a4	1.2166E-08	1.21803074	0.059	0.035	0.00026691
Hdac4	1.2851E-08	1.00083118	0.101	0.07	0.00028195

Usp9x	1.3729E-08	0.67429063	0.309	0.282	0.00030121
Podnl1	1.4038E-08	1.01666501	0.054	0.031	0.00030797
Adck5	1.4894E-08	1.59769384	0.026	0.011	0.00032677
Gbp2b	1.5046E-08	1.00744217	0.07	0.044	0.0003301
Zfp831	1.5376E-08	1.04338325	0.033	0.016	0.00033734
Whrn	1.5657E-08	1.32255526	0.028	0.013	0.00034351
Ccdc30	1.5924E-08	1.44175266	0.028	0.013	0.00034935
Rb1cc1	1.6934E-08	0.53929924	0.296	0.257	0.00037152
Clip1	1.8599E-08	0.8767192	0.183	0.146	0.00040804
Slc35e2	1.9737E-08	0.90863801	0.075	0.047	0.00043302
Dnajc8	1.9828E-08	0.43395084	0.421	0.404	0.000435
Abhd18	1.9884E-08	0.95630473	0.053	0.03	0.00043624
Casp8ap2	2.0176E-08	0.76090058	0.187	0.148	0.00044263
H2-Q6	2.0248E-08	0.25677583	0.458	0.419	0.00044423
Pnpla8	2.0788E-08	0.73087531	0.223	0.186	0.00045608
Runx2os1	2.0964E-08	1.68734914	0.035	0.018	0.00045993
Pex11a	2.1125E-08	1.9977127	0.013	0.004	0.00046347
Rassf3	2.3191E-08	0.79447438	0.239	0.202	0.00050879
Nck2	2.3233E-08	1.01269688	0.062	0.038	0.0005097
Tmem30a	2.345E-08	0.49553383	0.346	0.314	0.00051448
Picalm	2.8506E-08	0.29953653	0.546	0.526	0.00062538
Arl2bp	3.1748E-08	0.69971551	0.2	0.161	0.00069653
Itpripl1	3.3314E-08	1.34249879	0.04	0.021	0.00073087
S100a13	3.5328E-08	0.44658852	0.41	0.394	0.00077507
Smg6	3.6305E-08	0.74564086	0.172	0.134	0.0007965
Impa2	4.0319E-08	1.17222753	0.078	0.051	0.00088455
Ube2z	4.143E-08	0.81379946	0.179	0.144	0.00090893
Ranbp2	4.5519E-08	0.53006868	0.406	0.387	0.00099864
Pcmtd2	4.6157E-08	0.64393406	0.133	0.097	0.00101263
Dus2	4.7251E-08	0.99013471	0.058	0.034	0.00103664
Rcsd1	4.8856E-08	0.57932271	0.133	0.097	0.00107186
Bmp2k	5.0276E-08	0.74321007	0.181	0.145	0.00110301
Mphosph8	5.1068E-08	0.83618411	0.13	0.096	0.00112038
Exoc2	5.1679E-08	1.04620399	0.107	0.076	0.00113378
Anxa11	5.2353E-08	0.80489536	0.179	0.144	0.00114857
Erich1	5.2533E-08	1.01682432	0.083	0.055	0.00115252
Arhgap4	5.3952E-08	0.70310932	0.107	0.075	0.00118365
Tnfrsf25	5.491E-08	0.94986442	0.07	0.044	0.00120468
Rab3ip	5.5044E-08	1.04814691	0.066	0.041	0.00120762
Srp14	5.8627E-08	0.43101422	0.446	0.44	0.00128622
Art2b	6.3271E-08	1.48293284	0.031	0.015	0.0013881
Mknk2	6.4839E-08	0.47337053	0.311	0.277	0.00142249
Leng8	6.5083E-08	0.6209546	0.213	0.176	0.00142785
Fam104a	6.5278E-08	0.57297789	0.309	0.281	0.00143213
Zdhhc21	7.258E-08	0.95437018	0.133	0.101	0.00159234
Tdg	7.2722E-08	0.83443881	0.143	0.109	0.00159545
Zc3h15	7.4855E-08	0.48605985	0.401	0.384	0.00164225
Zmat3	7.537E-08	1.40070277	0.031	0.015	0.00165353

Plcg1	7.7488E-08	0.75276759	0.104	0.072	0.0017
Adap1	8.2599E-08	1.35960777	0.064	0.04	0.00181214
Ddb1	8.3921E-08	0.89781444	0.17	0.136	0.00184115
Insr	8.5849E-08	1.13051354	0.064	0.04	0.00188344
Celf2	9.0019E-08	0.31256418	0.56	0.545	0.00197493
Eif2ak1	9.004E-08	0.97704622	0.115	0.084	0.00197538
Hemk1	9.0701E-08	1.37661019	0.026	0.012	0.00198989
Ehhadh	9.4255E-08	1.73038884	0.01	0.003	0.00206785
Ltb4r1	9.5102E-08	1.11505092	0.072	0.046	0.00208644
Alg10b	1.0056E-07	1.16499243	0.067	0.042	0.00220621
Sf3b5	1.022E-07	0.56716767	0.337	0.318	0.00224218
Rab11fip1	1.03E-07	0.39015345	0.347	0.319	0.00225976
Sike1	1.0516E-07	0.79926458	0.125	0.093	0.00230719
Mtdh	1.0636E-07	0.42646837	0.452	0.449	0.00233345
Slk	1.0677E-07	0.49867371	0.358	0.333	0.00234246
Metap2	1.1235E-07	0.58828095	0.337	0.315	0.00246492
Eapp	1.2451E-07	0.59019174	0.267	0.233	0.00273169
Ppfia1	1.246E-07	0.73479028	0.171	0.135	0.00273359
Gjb2	1.2681E-07	0.89975552	0.041	0.023	0.00278216
Hic1	1.2713E-07	1.08017219	0.064	0.04	0.00278909
Hsd17b7	1.2793E-07	1.0750381	0.052	0.031	0.00280662
Gm20275	1.2904E-07	1.9356532	0.011	0.003	0.0028311
Glcci1	1.3717E-07	1.05698096	0.045	0.026	0.00300941
Trpm4	1.3752E-07	1.76811855	0.025	0.011	0.00301698
Gm42722	1.3818E-07	1.36094046	0.023	0.01	0.00303156
Amz2	1.4012E-07	0.96457383	0.123	0.091	0.00307412
H2-T23	1.4272E-07	0.43878904	0.471	0.471	0.00313107
Hnrnpl	1.4407E-07	0.54776576	0.312	0.285	0.00316067
Vamp4	1.4935E-07	0.75220038	0.223	0.19	0.00327655
Trove2	1.5318E-07	1.64959327	0.032	0.016	0.00336054
Impa1	1.5822E-07	0.77939734	0.149	0.115	0.00347108
Cited2	1.641E-07	0.46975391	0.203	0.163	0.00360026
Kif5b	1.686E-07	0.4535534	0.43	0.425	0.00369897
Thap3	1.7106E-07	0.8590388	0.09	0.061	0.00375297
Mast4	1.7733E-07	0.6250743	0.214	0.176	0.00389052
Fopnl	1.953E-07	0.75821792	0.109	0.078	0.00428461
Rbm25	1.955E-07	0.25978652	0.622	0.634	0.00428898
Kcnk7	1.9738E-07	1.59427236	0.016	0.006	0.00433033
Phc1	1.9942E-07	1.08859997	0.044	0.025	0.00437502
Zfp654	2.129E-07	0.89573408	0.085	0.058	0.00467087
1300002E11R	2.1863E-07	0.86283583	0.061	0.038	0.00479649
Rad18	2.2524E-07	0.91893933	0.104	0.074	0.00494147
Gbp8	2.3594E-07	1.14789971	0.082	0.055	0.0051762
Farp1	2.4885E-07	1.25345405	0.028	0.014	0.00545954
Izumo4	2.6985E-07	1.25096457	0.035	0.018	0.00592016
Vps13d	2.8159E-07	0.77565768	0.121	0.089	0.00617788
2310031A07R	2.8204E-07	1.7076767	0.042	0.024	0.00618773
Itih5	2.9575E-07	1.22809522	0.03	0.015	0.00648854

Rfk	2.992E-07	0.75941068	0.131	0.099	0.00656419
Map3k5	3.0753E-07	0.85755918	0.088	0.061	0.00674682
Rtn4rl1	3.1236E-07	1.68213301	0.023	0.011	0.00685287
Ier5l	3.1494E-07	0.47726026	0.179	0.14	0.00690955
Zfp874a	3.2513E-07	1.12490385	0.037	0.02	0.00713309
Nampt	3.3842E-07	0.72690124	0.23	0.198	0.0074245
Dip2b	3.4018E-07	0.58434059	0.228	0.192	0.00746325
Zfp40	3.4484E-07	1.63819397	0.015	0.006	0.00756536
Cnnm3	3.4911E-07	1.16421033	0.052	0.032	0.00765912
Ranbp9	3.5393E-07	0.83584613	0.144	0.112	0.0077648
Mturn	3.7504E-07	1.307708	0.025	0.012	0.00822792
Slc2a3	3.8222E-07	0.56351821	0.085	0.057	0.00838546
Dgka	3.9419E-07	0.51453704	0.147	0.111	0.00864807
Armcx4	3.96E-07	2.22863426	0.011	0.003	0.00868795
Rap1gds1	4.2074E-07	0.79139764	0.149	0.116	0.00923055
Fas	4.2392E-07	0.74331584	0.15	0.117	0.00930048
Capn1	4.2433E-07	0.81057284	0.119	0.088	0.00930933
Gpr160	4.2682E-07	1.62042408	0.025	0.012	0.00936398
Mapk9	4.5763E-07	0.952597	0.076	0.051	0.01003988
Atcayos	4.7257E-07	2.26538506	0.011	0.004	0.01036764
Mgmt	4.785E-07	2.29676282	0.02	0.009	0.01049784
Tstd3	5.0207E-07	1.10356272	0.052	0.031	0.01101493
Cog2	5.1416E-07	1.05895259	0.101	0.073	0.01128024
Kcnk6	5.1528E-07	1.82863389	0.034	0.018	0.01130467
AI987944	5.1732E-07	1.00255031	0.053	0.033	0.01134947
Zfp329	5.1878E-07	0.92621579	0.076	0.051	0.01138148
Krt222	5.2824E-07	2.48454041	0.01	0.003	0.01158916
Eml2	5.3208E-07	1.33000701	0.046	0.027	0.01167333
Cst7	5.364E-07	0.80032613	0.086	0.059	0.01176801
Fam53b	5.4797E-07	1.02726501	0.048	0.028	0.01202197
Hnrnpk	5.5815E-07	0.29629721	0.586	0.606	0.01224522
Arf1	5.654E-07	0.40132641	0.477	0.485	0.01240434
Wdr37	6.5598E-07	0.81775855	0.147	0.116	0.01439153
Acss2	6.6839E-07	1.02475206	0.044	0.025	0.01466391
Gm13212	6.9614E-07	1.19558697	0.057	0.036	0.01527268
Dhx36	6.9685E-07	0.55096352	0.271	0.238	0.01528813
Rbbp5	7.0406E-07	1.004331	0.064	0.041	0.01544638
Ppcs	7.2412E-07	1.39016008	0.028	0.014	0.01588643
Tg	7.4661E-07	1.88495174	0.017	0.007	0.01637991
Rad21	8.1354E-07	0.53309964	0.295	0.264	0.01784835
Pacs1	8.3374E-07	0.91643954	0.084	0.058	0.01829147
Pptc7	8.4229E-07	0.85836693	0.126	0.096	0.0184791
Klc1	8.5293E-07	1.07737593	0.091	0.065	0.01871234
Cysltr2	8.8727E-07	0.59782362	0.111	0.08	0.01946589
Kdm7a	8.8756E-07	0.59251393	0.39	0.379	0.01947207
Fam160b1	9.1881E-07	0.73542135	0.107	0.078	0.02015787
B3gnt2	9.4678E-07	1.24876907	0.036	0.02	0.02077141
Pitpnm1	9.7936E-07	0.87016596	0.123	0.094	0.02148619

Skil	9.8461E-07	0.33281252	0.502	0.492	0.02160133
Swt1	1.004E-06	0.59563348	0.183	0.149	0.02202575
Kif13a	1.0157E-06	1.73743638	0.029	0.015	0.02228315
Tigar	1.0218E-06	1.22513518	0.033	0.017	0.02241703
Rab21	1.0621E-06	0.44933521	0.333	0.308	0.0233006
Tshz2	1.0645E-06	1.80341054	0.014	0.006	0.02335479
Frmd6	1.072E-06	1.16885253	0.062	0.041	0.02351927
Pcm1	1.1078E-06	0.7317087	0.186	0.154	0.02430461
Airn	1.1218E-06	1.73298221	0.017	0.007	0.02461108
Sfr1	1.137E-06	0.6292385	0.303	0.282	0.02494365
Aldoa	1.158E-06	0.39601704	0.459	0.461	0.02540539
Cep19	1.1815E-06	1.26666083	0.019	0.009	0.02592097
Tomm7	1.2029E-06	0.25162846	0.624	0.647	0.02639101
Dcaf15	1.212E-06	0.67902921	0.114	0.085	0.02658902
Ddhd2	1.2631E-06	0.82678641	0.104	0.076	0.02771025
Tbc1d8b	1.2929E-06	1.23222908	0.037	0.021	0.02836473
Mgst2	1.2993E-06	1.20972652	0.03	0.016	0.02850513
Cdr2	1.3035E-06	1.01505812	0.05	0.031	0.02859709
F2rl2	1.3121E-06	1.88532681	0.018	0.008	0.02878637
Mms19	1.3253E-06	0.89182062	0.088	0.062	0.02907592
Bex3	1.3507E-06	0.62567749	0.234	0.203	0.02963195
Klhl2	1.3673E-06	0.92650633	0.051	0.031	0.02999644
Hectd1	1.4926E-06	0.44710263	0.338	0.315	0.03274647
Gm35082	1.5818E-06	0.86409359	0.066	0.044	0.03470298
Tsc22d3	1.6077E-06	0.40811972	0.338	0.305	0.03527024
Klhl12	1.647E-06	0.80383159	0.072	0.048	0.03613427
Sytl1	1.817E-06	0.94550038	0.037	0.021	0.03986207
Bpnt1	1.9307E-06	0.8780311	0.104	0.077	0.04235748
Samd9l	1.9554E-06	0.41581254	0.162	0.127	0.04289964
Cobll1	2.0994E-06	0.84423946	0.073	0.049	0.04605946
S1pr4	2.1179E-06	0.68445281	0.082	0.057	0.04646385
Dctn6	2.2499E-06	0.87967323	0.096	0.07	0.04936036
H2afv	2.3195E-06	0.32400351	0.316	0.284	0.05088832
Pttg1	2.4622E-06	0.67771984	0.156	0.126	0.05401854
Tex30	2.478E-06	1.06148224	0.076	0.052	0.05436462
Ptger2	2.5304E-06	1.33699351	0.043	0.026	0.05551362
Ezh1	2.5842E-06	0.69950278	0.116	0.087	0.05669395
Aggf1	2.7734E-06	0.83980071	0.144	0.114	0.06084552
Rnpepl1	2.8126E-06	0.89333497	0.084	0.059	0.06170628
Zfp874b	2.9444E-06	0.93852157	0.055	0.035	0.06459745
Sgms1	2.9487E-06	0.67073868	0.166	0.134	0.06469176
Zfp219	2.9535E-06	1.29058353	0.039	0.023	0.0647962
Setd4	2.9768E-06	1.17069457	0.022	0.011	0.06530708
Rgs10	3.0182E-06	0.54389739	0.319	0.299	0.06621542
Hccs	3.1417E-06	0.66068383	0.174	0.143	0.06892673
Gpr132	3.17E-06	0.28371584	0.513	0.513	0.0695458
Bdh1	3.1876E-06	1.15033081	0.025	0.013	0.06993332
Ncbp1	3.1879E-06	0.72283582	0.104	0.077	0.06993925

Gm42372	3.2048E-06	1.25064502	0.019	0.009	0.07030915
Ahi1	3.2136E-06	0.68265037	0.106	0.078	0.07050394
Wdr78	3.3131E-06	1.74081682	0.012	0.005	0.07268511
Snx25	3.3801E-06	0.89611802	0.037	0.021	0.0741553
Gm11131	3.748E-06	1.46590621	0.021	0.01	0.08222677
Egln1	3.8139E-06	0.68922112	0.131	0.102	0.0836722
Fam193b	3.8518E-06	0.95286756	0.072	0.049	0.08450421
Nat2	3.9614E-06	1.40223056	0.024	0.012	0.08690872
Hivep1	3.9876E-06	0.62028237	0.26	0.234	0.08748383
Tmem156	3.9957E-06	0.86687626	0.063	0.042	0.08766116
Trim21	4.0202E-06	1.20118489	0.032	0.018	0.08819942
Uty	4.0882E-06	0.86478701	0.074	0.051	0.08969129
Fbxo45	4.1315E-06	1.08829331	0.046	0.029	0.09064205
Ppp2r3a	4.209E-06	1.41608503	0.044	0.027	0.09234144
Suox	4.4954E-06	1.6016358	0.026	0.013	0.09862357
Zcrb1	4.7189E-06	0.53691502	0.319	0.3	0.10352882
Zfp677	5.0977E-06	1.20230928	0.028	0.015	0.11183949
Fuz	5.0985E-06	1.3110756	0.029	0.015	0.1118554
Gm26799	5.1571E-06	1.36275975	0.022	0.011	0.11314242
Rfx1	5.3253E-06	1.17019285	0.065	0.044	0.11683088
Foxo4	5.5073E-06	1.02431237	0.034	0.019	0.12082566
Abcb9	5.6275E-06	0.9528302	0.046	0.029	0.12346099
Traf3ip3	5.7539E-06	0.58152588	0.097	0.071	0.12623379
Pglyrp2	5.8525E-06	0.97417745	0.023	0.011	0.12839725
Casc4	5.9233E-06	1.0591685	0.031	0.017	0.1299504
Klf6	5.962E-06	0.30945254	0.566	0.566	0.13080051
Naxd	6.0347E-06	0.82822256	0.116	0.09	0.13239431
Slfn9	6.0443E-06	1.45402288	0.023	0.011	0.13260647
Rab23	6.0559E-06	1.56358179	0.02	0.009	0.13286055
Rsrc2	6.1353E-06	0.26401379	0.558	0.569	0.13460179
Paqr8	6.3452E-06	2.25971316	0.013	0.005	0.13920826
Snrk	6.658E-06	0.83337161	0.105	0.079	0.14606987
Cetn2	6.8422E-06	0.6243886	0.195	0.165	0.15011163
Ccnc	6.898E-06	0.93624696	0.083	0.06	0.15133608
Tceal9	7.0979E-06	0.74866442	0.215	0.188	0.15572
Ccdc6	7.1845E-06	0.83394715	0.108	0.082	0.15762083
Sltm	7.1899E-06	0.47508952	0.33	0.311	0.15774019
Cript	7.3585E-06	0.38117235	0.371	0.355	0.1614386
Eva1b	7.6335E-06	0.86445611	0.121	0.094	0.16747241
Tma7	7.7498E-06	0.25235682	0.564	0.58	0.170022
Ercc6	7.7572E-06	0.95773301	0.078	0.056	0.17018459
Wdr82	7.9004E-06	0.74738326	0.128	0.1	0.17332601
Gm20186	7.9308E-06	0.35763892	0.13	0.1	0.17399486
Mapre3	8.258E-06	1.37599091	0.028	0.015	0.18117162
Ywhae	8.2742E-06	0.29956691	0.501	0.501	0.18152686
Ibtk	8.7091E-06	0.81890186	0.117	0.09	0.19106961
AI480526	8.7557E-06	0.89880139	0.035	0.02	0.19209042
Cd109	8.9588E-06	2.1617211	0.011	0.004	0.19654708

Galm	8.9921E-06	1.82190763	0.021	0.01	0.19727783
R3hdm1	9.0809E-06	0.4454219	0.269	0.239	0.19922678
Azi2	9.3051E-06	0.61405304	0.206	0.178	0.20414463
Slc37a3	9.758E-06	1.00235759	0.059	0.04	0.21407984
Kcnn4	9.8037E-06	0.90782973	0.116	0.09	0.21508316
Tmem18	1.0008E-05	1.28355114	0.026	0.014	0.21955746
Fam126b	1.0066E-05	0.96332502	0.057	0.037	0.22083805
Diaph1	1.1015E-05	0.32715088	0.462	0.461	0.24166052
Zfp362	1.1126E-05	1.00259126	0.054	0.036	0.2440874
Ergic1	1.1298E-05	1.11169926	0.078	0.057	0.24786315
Inpp4a	1.1407E-05	0.88769797	0.082	0.06	0.25026355
Paip2	1.1832E-05	0.33529771	0.436	0.428	0.25957965
Slc48a1	1.2016E-05	0.96042964	0.078	0.056	0.26361448
Ccdc66	1.202E-05	1.05438781	0.037	0.022	0.26370766
Upf3b	1.2294E-05	0.79111265	0.111	0.085	0.26970891
Krtcap2	1.2463E-05	0.40325595	0.408	0.404	0.27341839
Stambpl1	1.2564E-05	0.94561124	0.036	0.021	0.27564824
4921524J17Ril	1.2891E-05	0.62209534	0.133	0.105	0.28282358
Zc3h14	1.324E-05	0.78014208	0.145	0.118	0.2904746
Alkbh5	1.3583E-05	0.54097841	0.247	0.223	0.29800597
Ube2i	1.3775E-05	0.33776525	0.456	0.46	0.30219935
Ccdc117	1.3896E-05	0.84562718	0.081	0.059	0.30485921
Sgsm2	1.4676E-05	1.58048186	0.02	0.01	0.32198589
Eid2	1.4705E-05	1.94612592	0.015	0.007	0.32261165
Nt5c	1.5118E-05	0.72764243	0.202	0.178	0.33166967
Atf2	1.5291E-05	0.81106593	0.165	0.138	0.33546816
Sash3	1.5757E-05	0.66175198	0.086	0.063	0.34568595
Acot9	1.6958E-05	0.97183531	0.092	0.069	0.37204409
Pink1	1.7268E-05	0.79333449	0.099	0.075	0.37884046
Frat1	1.7775E-05	1.03694998	0.035	0.02	0.38996959
Mypopos	1.8324E-05	1.20361118	0.017	0.008	0.40201854
Smarcd1	1.8445E-05	0.65121887	0.097	0.072	0.40466125
Cnp	1.9035E-05	0.73025698	0.092	0.068	0.41760967
Tnks2	1.9088E-05	0.52936405	0.285	0.263	0.41877399
Gatsl2	1.918E-05	1.7104477	0.031	0.018	0.4207852
Mepce	1.9247E-05	0.60001193	0.146	0.117	0.42225447
Snx14	1.9293E-05	0.87050697	0.059	0.04	0.42327801
Osbpl2	1.9511E-05	1.01633051	0.054	0.036	0.42804126
Sun1	2.0917E-05	0.97863862	0.059	0.04	0.4589078
Spry1	2.0996E-05	0.84575833	0.039	0.024	0.46063151
Fbxo25	2.101E-05	1.29802452	0.026	0.014	0.46093119
Sirt4	2.1257E-05	1.50702997	0.012	0.005	0.46635116
Gpatch8	2.1929E-05	0.53990255	0.258	0.234	0.48110226
Krba1	2.2354E-05	1.05982686	0.023	0.012	0.49043011
Sacm1l	2.2593E-05	0.91960178	0.093	0.07	0.49566172
Pigx	2.2967E-05	0.81647418	0.1	0.077	0.50387047
Cr1l	2.3764E-05	0.61544954	0.209	0.183	0.5213574
Phf1	2.3918E-05	0.70855595	0.093	0.07	0.52473491

Hnrnph1	2.4635E-05	0.27201127	0.493	0.492	0.5404768
Ssb	2.47E-05	0.42174891	0.388	0.385	0.54189761
Psd4	2.5625E-05	0.6432816	0.145	0.118	0.56219141
Idnk	2.5719E-05	0.63640182	0.203	0.177	0.56425468
Ddx55	2.675E-05	0.71924379	0.061	0.041	0.58686068
Cggbp1	2.7033E-05	0.42584907	0.383	0.377	0.59308241
Atrx	2.7248E-05	0.31877402	0.453	0.446	0.59779537
Tmem126b	2.7484E-05	1.03312539	0.039	0.024	0.60296598
Ucp2	2.7918E-05	0.28477507	0.531	0.535	0.61249016
2410089E03R	2.8827E-05	0.79164723	0.076	0.054	0.63242954
Scrt1	3.0303E-05	1.12118217	0.038	0.024	0.66480843
Crebrf	3.0601E-05	0.41459165	0.316	0.292	0.67134534
Atp9b	3.3371E-05	0.83295423	0.112	0.088	0.73211769
Zmpste24	3.606E-05	0.8638323	0.111	0.087	0.791114
Gpr107	3.7119E-05	0.71564991	0.126	0.101	0.81436237
Dennd5a	3.7902E-05	0.59898378	0.264	0.244	0.83153483
Snrnp70	3.9298E-05	0.33144141	0.511	0.523	0.86216226
Itga3	3.9766E-05	1.31639291	0.026	0.015	0.87241728
Polm	4.1388E-05	1.08404357	0.028	0.016	0.90800552
Gm37494	4.1855E-05	0.91248245	0.057	0.039	0.91826554
Tmem42	4.2846E-05	0.87093252	0.044	0.028	0.9399904
Lactb2	4.3943E-05	1.19638226	0.056	0.038	0.96405902
Zfp760	4.4266E-05	1.42047979	0.018	0.009	0.97114548
Pced1b	4.4279E-05	0.75316917	0.043	0.027	0.97142943
Zfp729a	4.4876E-05	0.78060234	0.063	0.044	0.9845406
Gpr65	4.7559E-05	0.65416055	0.279	0.264	1
Zfp119a	4.8329E-05	1.53346802	0.024	0.013	1
Col18a1	4.8433E-05	1.51081365	0.014	0.006	1
Eif4e3	4.9484E-05	0.87452198	0.082	0.061	1
Zbtb25	4.995E-05	0.7989474	0.075	0.055	1
Yipf3	5.2952E-05	0.63885149	0.181	0.156	1
Pgm2l1	5.3737E-05	0.61434479	0.134	0.108	1
Bmt2	5.4743E-05	0.60105099	0.116	0.091	1
Ppp2r5d	5.6605E-05	1.09490855	0.053	0.036	1
Tubb4b	5.6634E-05	0.69343822	0.16	0.134	1
Hsd11b1	5.8417E-05	0.48255693	0.038	0.024	1
Lyrm2	5.8535E-05	0.87743842	0.076	0.056	1
Jazf1	5.9346E-05	1.79732953	0.012	0.005	1
Coro2b	5.9597E-05	1.20939505	0.02	0.01	1
Usp42	6.2462E-05	0.91160628	0.061	0.043	1
Dnajb14	6.2552E-05	1.12616328	0.1	0.079	1
Trip11	6.3744E-05	0.47897022	0.25	0.226	1
Eno1	6.4015E-05	0.48828849	0.256	0.233	1
Wasf2	6.5708E-05	0.4797927	0.376	0.375	1
Exoc3	6.5899E-05	0.66347481	0.179	0.155	1
Ubxn8	6.6077E-05	0.94894456	0.068	0.049	1
Usp45	6.7258E-05	0.99656162	0.066	0.047	1
Rps6ka1	6.8006E-05	0.88759224	0.097	0.075	1

Rad54l2	6.8114E-05	0.80112777	0.088	0.066	1
Ccdc28a	6.8508E-05	1.12236583	0.04	0.025	1
Naa10	6.8817E-05	0.61667632	0.133	0.109	1
Pias3	6.916E-05	1.11289821	0.03	0.018	1
Tmpo	7.0328E-05	0.61464272	0.198	0.172	1
Gm9856	7.2306E-05	0.97989048	0.043	0.027	1
Zfp740	7.2398E-05	0.73133014	0.096	0.073	1
Rab2a	7.3172E-05	0.345872	0.431	0.437	1
Zfp148	7.4802E-05	0.57105886	0.2	0.175	1
Frat2	7.5186E-05	0.70024109	0.067	0.048	1
Kif3c	7.5736E-05	1.59362318	0.016	0.008	1
Ppp1r8	7.6016E-05	0.93711665	0.062	0.044	1
Lipo3	7.6075E-05	1.087043	0.045	0.03	1
Zfp217	7.7971E-05	1.0195425	0.064	0.046	1
Hbs1l	8.044E-05	0.72961215	0.109	0.086	1
Slc1a4	8.0668E-05	2.22376354	0.01	0.004	1
Blmh	8.2124E-05	0.65385057	0.149	0.125	1
2610008E11R	8.3741E-05	1.19828489	0.043	0.028	1
Lyrm9	8.6228E-05	0.82196292	0.043	0.028	1
Slc50a1	8.6481E-05	0.64957691	0.154	0.129	1
Poli	8.6798E-05	1.31797627	0.021	0.011	1
Wasl	8.7761E-05	0.75101072	0.114	0.091	1
Gcc2	9.0279E-05	0.63175169	0.148	0.123	1
Gramd1c	9.0743E-05	1.40588653	0.015	0.007	1
Xlr4c	9.183E-05	0.85180688	0.035	0.021	1
Eif4ebp2	9.326E-05	0.59230886	0.201	0.176	1
Chd8	9.3643E-05	0.60070148	0.194	0.168	1
Fbxw2	9.7637E-05	0.48737486	0.246	0.225	1
Scand1	9.8181E-05	0.38683767	0.459	0.478	1
Srp19	9.9133E-05	0.51770973	0.264	0.246	1
Nebl	9.991E-05	0.54336205	0.097	0.074	1
Vps13b	0.00010243	0.74438674	0.115	0.092	1
Zhx2	0.00010732	0.63949631	0.137	0.113	1
G2e3	0.00010766	0.74441989	0.078	0.058	1
Eif5a2	0.00010784	1.54773998	0.022	0.012	1
Cd200r1	0.00010979	0.95577191	0.064	0.045	1
Cep192	0.0001098	0.70708018	0.099	0.077	1
Lnpep	0.00011656	0.36877074	0.395	0.39	1
Ppp1cb	0.00011915	0.38530502	0.344	0.332	1
Il23r	0.00012304	0.82984608	0.03	0.018	1
Csrp2	0.00012459	1.21436608	0.044	0.03	1
Zfp579	0.00012976	1.28830294	0.019	0.01	1
4833439L19Ri	0.00013008	0.72260488	0.113	0.09	1
Exoc8	0.00013188	1.41244724	0.026	0.015	1
Tsg101	0.00013268	0.80251132	0.15	0.128	1
Cyfip1	0.00013284	0.6508464	0.219	0.2	1
Creb1	0.00013296	0.53156712	0.187	0.162	1
Lrrcc1	0.00013631	0.96243956	0.045	0.03	1

Sec23ip	0.00013666	0.86555826	0.077	0.057	1
Jade2	0.00013725	0.76906711	0.066	0.048	1
Etaa1os	0.00013733	1.45394862	0.016	0.008	1
BC004004	0.00013803	0.68121573	0.126	0.103	1
A430078G23R	0.00013951	0.99513461	0.031	0.019	1
Pigp	0.00014016	0.80430246	0.092	0.071	1
Zdhhc20	0.0001406	0.41902492	0.213	0.187	1
Rbm38	0.000145	0.79502931	0.078	0.059	1
Slc2a8	0.00014506	1.23707707	0.025	0.014	1
Calcoco1	0.0001455	0.95474948	0.045	0.03	1
Unc5cl	0.00014715	1.33960448	0.012	0.005	1
Med30	0.00014762	0.76275506	0.139	0.116	1
Rcan3	0.00014999	1.12990749	0.028	0.017	1
Kctd10	0.00015363	0.83254624	0.112	0.09	1
Fpgt	0.00015374	0.98559133	0.042	0.028	1
Fam216a	0.00015642	0.89730055	0.046	0.031	1
Rabif	0.00015739	0.68912151	0.068	0.05	1
AW146154	0.00016199	1.5439591	0.013	0.006	1
Zfp28	0.00016267	1.53281663	0.011	0.005	1
Ccdc22	0.00016336	1.27392608	0.051	0.036	1
Pgm2	0.00016817	1.0054044	0.032	0.02	1
C2cd2	0.00016955	1.20430423	0.024	0.014	1
Eprs	0.00016986	0.35584703	0.414	0.407	1
Taf1b	0.00017261	0.86064162	0.054	0.038	1
Il16	0.00018465	0.3922975	0.131	0.105	1
4833403J16Ril	0.00018584	1.18401575	0.024	0.014	1
1110008P14R	0.00019797	0.57817192	0.185	0.162	1
Ercc3	0.00020074	0.79561507	0.082	0.062	1
Cdon	0.00020177	1.03554569	0.03	0.019	1
Ing4	0.00020204	0.68555959	0.086	0.066	1
Mrps24	0.00020206	0.5692122	0.241	0.224	1
Phf6	0.00020311	0.5623562	0.121	0.097	1
Ston2	0.00020666	0.78473321	0.083	0.063	1
Rsbn1	0.00020734	0.556622	0.179	0.154	1
Nop53	0.00020958	0.27816322	0.52	0.54	1
Zfp938	0.00020967	0.97889538	0.036	0.023	1
Bola3	0.00021179	0.57549516	0.17	0.146	1
Cul3	0.00021557	0.50365278	0.259	0.242	1
Mtmr9	0.00022194	1.30125564	0.033	0.021	1
Pcbp4	0.00022724	1.23607304	0.02	0.011	1
Trnt1	0.00022808	0.62738107	0.176	0.154	1
Ralgapb	0.00023919	0.71889567	0.101	0.08	1
Elavl1	0.00023921	0.55602526	0.246	0.229	1
Aip	0.00024294	0.68335135	0.137	0.115	1
Rassf5	0.00024758	0.54714863	0.163	0.138	1
Map4k5	0.00024972	1.01881493	0.039	0.026	1
Irak1bp1	0.00025954	1.3751641	0.015	0.007	1
Pygo2	0.00026198	0.87552673	0.036	0.023	1

Sp4	0.00026341	0.50414173	0.152	0.126	1
Nr2c2	0.00026827	0.50790693	0.188	0.163	1
Zscan12	0.00026859	1.47056993	0.014	0.007	1
Rab39b	0.00027261	1.06002383	0.025	0.014	1
Sh2b1	0.00027336	0.65899108	0.089	0.068	1
Arl4c	0.0002783	0.40289608	0.336	0.319	1
Nkrf	0.00028374	1.15216519	0.026	0.016	1
Ccs	0.000286	0.92589469	0.071	0.053	1
Celsr1	0.00029231	0.96399857	0.023	0.013	1
Cdc37l1	0.00029418	0.48029325	0.209	0.185	1
A930024E05R	0.00029424	0.68755302	0.073	0.054	1
Atrn	0.00029571	0.92272257	0.048	0.033	1
Lysmd3	0.0002971	0.5505083	0.124	0.102	1
Pum2	0.00030291	0.40542328	0.361	0.361	1
Xpo6	0.0003049	0.7528185	0.144	0.123	1
Nucks1	0.00030685	0.28706492	0.435	0.434	1
Kmt5b	0.00030755	0.5109569	0.189	0.165	1
0610030E20R	0.0003096	0.6445653	0.107	0.086	1
Itsn2	0.00032166	0.47652559	0.287	0.271	1
Lin54	0.00032549	0.54331589	0.213	0.193	1
Zfp319	0.00033369	1.09552547	0.032	0.02	1
Arhgef10	0.0003394	1.06630662	0.032	0.02	1
Srebf1	0.00033993	0.86986652	0.081	0.063	1
Fnta	0.00034008	0.91956723	0.065	0.048	1
4933427D14R	0.00034536	0.91646413	0.022	0.012	1
Apaf1	0.00035354	0.61643181	0.192	0.171	1
Sik3	0.00035677	0.61095234	0.211	0.192	1
Helb	0.00036451	0.67500765	0.076	0.058	1
Zfp81	0.00036471	0.98507332	0.029	0.018	1
Ep300	0.00036564	0.54324809	0.215	0.194	1
Gdap10	0.00036806	0.68545388	0.115	0.094	1
Nr1d1	0.00037466	0.88748633	0.039	0.026	1
Mau2	0.00037879	0.62588074	0.165	0.143	1
Gmcl1	0.00037969	0.99933477	0.059	0.043	1
Bicd1	0.00038308	1.27241864	0.014	0.007	1
Ifi47	0.00038861	0.32290943	0.111	0.089	1
AI504432	0.00040744	0.64418119	0.137	0.115	1
Als2cl	0.00041893	1.3022998	0.018	0.01	1
Crebbp	0.00042237	0.43537113	0.359	0.355	1
Fam214a	0.00042513	0.57352654	0.086	0.066	1
Llgl2	0.00042992	1.24204388	0.015	0.008	1
Zdhhc15	0.00044126	1.51048079	0.011	0.005	1
Impdh1	0.00044169	0.6953821	0.073	0.055	1
Rtl8b	0.00044531	1.17170034	0.032	0.02	1
Zfp646	0.00044679	0.5504397	0.09	0.069	1
Zfp764	0.00044715	1.28356075	0.014	0.007	1
Gemin7	0.00045385	0.65589269	0.134	0.113	1
Zer1	0.00046551	0.69750691	0.061	0.045	1

Zfp266	0.00047007	0.52018843	0.112	0.09	1
Pmaip1	0.00047368	0.76444682	0.16	0.14	1
Tmem106b	0.00047685	0.68489807	0.096	0.076	1
Dhx32	0.00048113	1.10626941	0.026	0.016	1
Slc39a8	0.00048385	1.47415073	0.013	0.006	1
Zfp664	0.00048498	0.71721282	0.083	0.064	1
Spopl	0.0004879	0.67120679	0.105	0.084	1
Lockd	0.00050083	0.80509621	0.056	0.04	1
Tmem184c	0.00050211	1.1439918	0.031	0.02	1
Fchsd1	0.00050659	1.24344491	0.02	0.011	1
Tpp2	0.00051714	0.42520223	0.291	0.275	1
Dicer1	0.00051895	0.58406626	0.157	0.135	1
Tmub1	0.00053559	0.86366723	0.049	0.035	1
Ddb2	0.00054682	0.76918969	0.048	0.034	1
Hmgcr	0.00055268	0.50520314	0.229	0.21	1
Dnaja4	0.0005669	0.47830933	0.055	0.039	1
Uqcc3	0.00056839	0.55183757	0.173	0.151	1
Numa1	0.00058052	0.46918697	0.22	0.197	1
Kmt2e	0.00058056	0.26115747	0.428	0.42	1
Spcs2	0.00058189	0.3306987	0.426	0.435	1
Dubr	0.00058395	0.82265759	0.061	0.045	1
Trim12c	0.00059461	0.49359921	0.122	0.1	1
Lmtk2	0.00060605	0.90156699	0.067	0.05	1
Isg20	0.00060709	0.29303814	0.077	0.059	1
Gm15283	0.00062191	1.00639226	0.03	0.019	1
Phf3	0.0006418	0.31486941	0.362	0.356	1
Golph3	0.00064515	0.45736856	0.256	0.241	1
Fkbp3	0.00065498	0.37304363	0.279	0.26	1
Herpud2	0.00065642	0.64651138	0.13	0.11	1
Efcab14	0.00065664	0.70209997	0.1	0.08	1
Myo1d	0.0006582	1.38137699	0.01	0.004	1
Vamp5	0.0006602	0.90625882	0.038	0.026	1
Tmem140	0.0006733	0.81235411	0.052	0.038	1
Xlr4b	0.00067972	0.69363944	0.08	0.062	1
Bcl6	0.00068221	0.71366759	0.154	0.134	1
Zfp758	0.00070706	0.77981962	0.043	0.029	1
Zfp12	0.00073256	1.16690956	0.016	0.009	1
Prdm10	0.00073387	0.71641236	0.048	0.034	1
Mapk1	0.00074395	0.53081706	0.247	0.233	1
Pgghg	0.00074568	0.73779778	0.037	0.025	1
Gtf3c1	0.00074725	0.71757547	0.085	0.067	1
Fam189b	0.00075062	0.64743545	0.058	0.042	1
Nbr1	0.00076497	0.70093799	0.109	0.089	1
Fbxl20	0.00076693	0.79730249	0.081	0.063	1
Bcl3	0.00077615	0.63952977	0.199	0.181	1
Arhgap12	0.00078868	0.94547284	0.051	0.036	1
Ccl25	0.00079524	0.8479387	0.039	0.027	1
Sept9	0.0008063	0.54739959	0.186	0.166	1

Tango2	0.00081085	1.0032667	0.035	0.023	1
Msantd4	0.00085076	0.99592698	0.066	0.05	1
Stard10	0.0008917	1.12233167	0.034	0.022	1
Fam199x	0.00089779	1.00601053	0.041	0.028	1
Dcun1d4	0.00091788	1.0242814	0.033	0.022	1
Rlf	0.00092464	0.52127719	0.251	0.236	1
Trbv3	0.00094006	1.15445948	0.01	0.004	1
Zfp46	0.0009482	0.99582594	0.019	0.011	1
Zfp52	0.0009488	0.9643463	0.05	0.036	1
Fam103a1	0.00094897	0.45916851	0.211	0.191	1
Setbp1	0.00095415	0.85921189	0.033	0.022	1
Urgcp	0.00095479	0.94409103	0.052	0.038	1
Tmem175	0.0009551	1.20257169	0.021	0.012	1
Mettl17	0.00095658	0.97555091	0.029	0.018	1
Dhx57	0.00097295	0.90239436	0.034	0.023	1
Arhgap10	0.00098924	0.92706268	0.054	0.039	1
Crebl2	0.00100217	0.6629761	0.049	0.035	1
Synj2bp	0.00101053	0.64090936	0.118	0.099	1
Mfsd10	0.00101856	0.84013022	0.09	0.072	1
Gm4673	0.00102808	1.13814075	0.028	0.018	1
Traf2	0.00103039	0.75335512	0.087	0.069	1
Itga4	0.00104877	0.29505491	0.377	0.366	1
Rnf43	0.00105664	0.93385523	0.02	0.011	1
Zfp942	0.0010598	0.61711315	0.066	0.05	1
Fam8a1	0.00106173	0.68830496	0.06	0.045	1
Rpa1	0.00106197	0.71640511	0.076	0.059	1
Ttc28	0.00106964	1.03476654	0.049	0.035	1
Snapc3	0.00107146	0.80283652	0.057	0.042	1
Pgk1	0.00108049	0.37597102	0.344	0.34	1
Prpf8	0.00109328	0.50588761	0.224	0.207	1
Lonrf1	0.00109794	1.36116588	0.039	0.027	1
Zfp169	0.00110095	0.89772723	0.03	0.019	1
Atg13	0.00110335	0.87103102	0.072	0.056	1
Gas2l3	0.00111489	0.69964343	0.136	0.118	1
Ube3c	0.00115139	0.61201242	0.095	0.076	1
Fbxw17	0.00115207	1.16651283	0.035	0.024	1
Trim56	0.00115971	0.59488034	0.101	0.082	1
Arid4a	0.00116518	0.56590379	0.225	0.208	1
Prkab1	0.00116955	0.81497165	0.05	0.036	1
Abhd6	0.00117155	1.52489968	0.018	0.01	1
Pde8a	0.00117206	0.992211	0.05	0.036	1
Stk39	0.0011761	0.82429388	0.067	0.051	1
Zfp809	0.00117746	1.05334648	0.045	0.032	1
Pdcd7	0.00118091	0.91425786	0.07	0.055	1
Plekha5	0.00119112	0.59370797	0.081	0.063	1
Pdlim2	0.00119625	0.8067283	0.059	0.044	1
Ndufv3	0.001229	0.39437894	0.364	0.368	1
Mob1a	0.00123342	0.26251952	0.476	0.494	1

Pdcl	0.00124087	0.7104764	0.086	0.069	1
Zfp398	0.00124502	0.74499793	0.066	0.05	1
Pgrmc1	0.00125123	0.65848982	0.104	0.086	1
Ddr1	0.00125153	2.13930361	0.01	0.005	1
Rprd2	0.00126311	0.55936934	0.138	0.118	1
Adam12	0.00128044	1.20671841	0.016	0.009	1
Rnf103	0.00132271	0.80925639	0.073	0.057	1
Chchd10	0.0013807	0.6415634	0.118	0.099	1
Cbr1	0.00138608	0.8619264	0.041	0.029	1
Zfp445	0.00138659	0.60753513	0.164	0.146	1
Nek8	0.00140262	1.08924626	0.025	0.016	1
Rpap2	0.00140314	0.8412121	0.037	0.025	1
Hp1bp3	0.00143643	0.4319713	0.277	0.266	1
Prdm4	0.00144	0.89134225	0.061	0.046	1
Map3k1	0.00144717	0.35927762	0.348	0.341	1
Zfp58	0.00144904	1.08676809	0.013	0.007	1
Kdm1b	0.00145101	1.0722091	0.028	0.018	1
Ermp1	0.00145822	0.82871432	0.057	0.042	1
Mon1b	0.00149178	0.95589613	0.034	0.023	1
B3gnt9	0.00151821	1.37205575	0.012	0.006	1
Slc52a2	0.00151904	1.00322073	0.032	0.021	1
Kif3b	0.00153314	0.95955708	0.048	0.035	1
Gtf3c3	0.00156635	0.85722643	0.051	0.038	1
Apc	0.00158122	0.61760342	0.128	0.109	1
Ctcf	0.00158375	0.36307865	0.295	0.279	1
Ppp4r2	0.00159104	0.25457171	0.396	0.392	1
Wipf2	0.00159261	0.60704522	0.152	0.134	1
Med11	0.00161709	0.65190021	0.129	0.111	1
Rnf168	0.00166252	0.87133941	0.064	0.048	1
Rbm27	0.00167072	0.45096146	0.242	0.228	1
Tusc2	0.00168582	1.01395469	0.05	0.037	1
Zfp207	0.00169018	0.36271075	0.328	0.321	1
Gemin2	0.00173876	0.74237008	0.032	0.021	1
Sec22a	0.00174097	0.98133577	0.038	0.026	1
Pkn2	0.00176218	0.46362579	0.316	0.313	1
Pbx4	0.00176222	1.0799642	0.012	0.006	1
Cab39	0.00177963	0.37033359	0.298	0.287	1
Pfn2	0.00178649	1.35045469	0.01	0.005	1
B4galnt4	0.00179079	0.93087725	0.021	0.013	1
Wdr11	0.00183435	0.96505046	0.038	0.027	1
C1qtnf4	0.00191863	0.8521506	0.015	0.009	1
Ero1l	0.00192911	0.37016396	0.199	0.179	1
Eed	0.00193532	0.57076106	0.134	0.116	1
Nudt16	0.00194585	0.59887807	0.047	0.034	1
Strn	0.00194818	0.67235392	0.076	0.06	1
Hspb1	0.00195286	1.11933411	0.025	0.016	1
Traf3ip1	0.00200017	1.17599774	0.014	0.008	1
Cdkn2aip	0.00201626	0.5912033	0.1	0.083	1

Zfp846	0.0020515	0.98600386	0.021	0.013	1
Mark2	0.00206285	0.50476052	0.23	0.215	1
Mlf1	0.00206702	1.28040309	0.013	0.007	1
Ccdc157	0.00208782	1.36589503	0.011	0.005	1
A830010M20F	0.00209676	1.33192789	0.012	0.006	1
Prdm11	0.00210299	0.90384461	0.027	0.018	1
Rpap1	0.00215156	1.04473829	0.03	0.02	1
Extl3	0.00218966	1.13916219	0.036	0.025	1
Ddx11	0.00220125	0.95470925	0.019	0.011	1
Rab40c	0.0022078	0.83316605	0.031	0.021	1
Esco1	0.00225135	0.53941186	0.151	0.133	1
Sugp1	0.00225689	0.75182693	0.075	0.059	1
Magi3	0.00226644	0.84241471	0.028	0.019	1
Helz	0.00226726	0.50081139	0.146	0.127	1
Wbp1l	0.00227264	0.68166902	0.101	0.084	1
Txndc17	0.00229664	0.49631801	0.299	0.298	1
Slc35b3	0.00231583	0.58184191	0.072	0.056	1
Tmx4	0.00232287	0.64995907	0.148	0.13	1
E130311K13R	0.00232497	0.97360234	0.02	0.012	1
Gm20342	0.00239988	0.68346468	0.033	0.023	1
Mlec	0.00242736	0.60624447	0.162	0.145	1
Aff1	0.00247328	0.37835531	0.339	0.337	1
Cyfip2	0.00249904	0.40766765	0.241	0.225	1
Pkd1	0.002519	0.70829424	0.062	0.048	1
Tm7sf3	0.00253207	0.89091104	0.037	0.026	1
Brat1	0.00254152	1.06310345	0.021	0.013	1
Inf2	0.00255924	0.76547852	0.074	0.059	1
Camk2b	0.00255945	0.87858062	0.027	0.017	1
Fam151b	0.00264999	1.24888359	0.012	0.006	1
Wrap73	0.0026669	0.76115365	0.03	0.02	1
Xiap	0.00266789	0.49693877	0.258	0.249	1
Fubp3	0.00270471	0.9738261	0.037	0.026	1
Dync1i2	0.00271527	0.45990233	0.337	0.339	1
Zfp472	0.00276882	0.77767994	0.038	0.027	1
Wipi2	0.00278332	0.55416104	0.128	0.11	1
Cdkl2	0.00281117	1.43335662	0.011	0.006	1
Hif1an	0.0028398	0.59419512	0.114	0.097	1
Zfp949	0.00289317	0.77554705	0.036	0.025	1
Zfp85	0.00290888	1.21217512	0.011	0.005	1
Casc3	0.00297935	0.58214678	0.182	0.167	1
Kctd17	0.00301617	0.95114823	0.03	0.021	1
Gatsl3	0.00301709	0.94646172	0.047	0.035	1
Larp1b	0.00305829	0.66394138	0.118	0.101	1
Psph	0.00306182	0.78720842	0.047	0.035	1
Rtraf	0.00308275	0.4089351	0.367	0.381	1
Rnf139	0.003088	0.60326021	0.09	0.073	1
Ap5m1	0.00311784	0.81390824	0.058	0.044	1
Ptprs	0.00314351	1.07026071	0.071	0.057	1

Ubxn6	0.00315827	0.73092103	0.136	0.12	1
Pde2a	0.00316503	0.96735869	0.039	0.028	1
Jade1	0.00317546	0.66191117	0.076	0.06	1
Kbtbd4	0.00317855	0.84639624	0.027	0.018	1
Fam185a	0.0031787	1.11238707	0.017	0.01	1
Nlrx1	0.00320759	1.34154061	0.012	0.006	1
Tmf1	0.00320792	0.51021881	0.206	0.191	1
Smpd1	0.00324638	0.92126439	0.04	0.029	1
Atg4d	0.00329959	0.63204798	0.059	0.045	1
Poll	0.0033546	0.95453621	0.015	0.009	1
Vps13c	0.00338542	0.75972681	0.091	0.075	1
Ttf1	0.00346368	0.67777169	0.085	0.069	1
Cstad	0.00347657	1.22546061	0.012	0.007	1
3110040N11R	0.00350694	0.78888319	0.041	0.03	1
Esyt1	0.00351313	0.49410261	0.194	0.178	1
Supt5	0.00354821	0.43360068	0.277	0.27	1
Zfp777	0.00355855	0.78744874	0.036	0.025	1
C1qtnf6	0.0035721	0.91836467	0.021	0.013	1
9230102O04R	0.00357594	0.82957364	0.015	0.009	1
Tcaim	0.00366645	0.73515387	0.027	0.018	1
Mapkapk2	0.00368198	0.26031613	0.347	0.347	1
Usp34	0.00368204	0.45507258	0.279	0.272	1
Nlrc3	0.00369551	0.42678626	0.057	0.043	1
Lxn	0.00372033	0.92983988	0.036	0.025	1
Pmm1	0.0037354	0.90196478	0.043	0.032	1
Rtf1	0.00374875	0.33861662	0.337	0.332	1
Ghitm	0.00381285	0.29975379	0.414	0.428	1
Tada3	0.00381474	1.12157522	0.046	0.034	1
Mien1	0.00383123	0.44208438	0.267	0.259	1
Thra	0.00385951	0.99176151	0.03	0.02	1
Elmo1	0.00395623	0.42531454	0.302	0.298	1
Ptpn7	0.00397646	0.52418094	0.076	0.061	1
Zfp113	0.00408692	0.85087596	0.021	0.014	1
Pqlc3	0.00410231	1.01242185	0.05	0.038	1
Pcgf5	0.0041489	0.53832456	0.257	0.248	1
Slfn8	0.00425573	0.51080725	0.078	0.063	1
Zfp712	0.00425736	1.4342424	0.01	0.005	1
Ywhah	0.00430604	0.48283245	0.271	0.265	1
Yaf2	0.00430681	0.60594576	0.088	0.072	1
Slc35f2	0.00431125	1.19781802	0.011	0.006	1
Cyb561d1	0.00432391	1.01062191	0.035	0.025	1
Vps4a	0.00434719	0.75783492	0.093	0.078	1
Cdc14b	0.00439175	1.0993443	0.023	0.015	1
Brap	0.0044258	0.56143428	0.153	0.135	1
Zfp799	0.00445587	1.13843069	0.019	0.012	1
Zfp61	0.00448012	1.31777688	0.01	0.005	1
Kctd18	0.00451177	0.74727928	0.061	0.048	1
Dtx3	0.00456934	0.74948453	0.082	0.067	1

Gm13012	0.00475457	0.49447478	0.019	0.011	1
Cog4	0.00478137	0.62916082	0.064	0.05	1
Xpa	0.00482684	0.73160968	0.123	0.107	1
Sp3os	0.00493487	0.82827168	0.051	0.039	1
Mettl23	0.00499074	0.3907816	0.241	0.228	1
Alkbh4	0.00502921	1.01798467	0.026	0.017	1
Ndufb9	0.00510893	0.31469715	0.374	0.382	1
Coro2a	0.00513518	0.76254461	0.086	0.072	1
Bms1	0.00514122	0.62869588	0.125	0.109	1
Gm19684	0.00517997	1.51071711	0.01	0.005	1
Fbxl12	0.00519808	0.44119827	0.137	0.119	1
Mvp	0.00521436	0.27667439	0.116	0.148	1
Dok2	0.00522086	0.7206146	0.1	0.085	1
Nup35	0.00524408	1.07236977	0.023	0.015	1
Gm4951	0.00530024	1.52133923	0.011	0.006	1
Pstk	0.00534842	0.68708236	0.048	0.036	1
Iffo2	0.00541749	0.92324221	0.039	0.029	1
Zrsr1	0.00549167	0.63933255	0.06	0.047	1
Lrrfip2	0.00552792	0.92410028	0.158	0.148	1
Mex3b	0.00559744	1.07340318	0.036	0.026	1
Zfp738	0.00561442	0.77250013	0.044	0.033	1
Magee1	0.00565437	1.70236222	0.01	0.005	1
Map4k2	0.00566437	0.36228458	0.121	0.103	1
Brd1	0.00567715	0.4045026	0.293	0.287	1
Maf1	0.00575498	0.47478279	0.158	0.142	1
Sdhaf1	0.0057651	0.41812732	0.158	0.14	1
Tubgcp6	0.00579855	0.77026149	0.026	0.017	1
Usp25	0.00582737	0.46100753	0.273	0.266	1
Sept7	0.0058946	0.40527136	0.324	0.329	1
Tnfrsf22	0.0059138	0.79689913	0.01	0.005	1
Rnf19a	0.00597326	0.28120773	0.182	0.163	1
Dlg4	0.00602004	0.87598852	0.036	0.026	1
Dtx3l	0.00607714	0.57260201	0.135	0.119	1
Sept11	0.00615051	0.38477672	0.237	0.222	1
Tmem63a	0.00619103	0.89541906	0.043	0.032	1
Mrpl43	0.00630222	0.51947736	0.182	0.169	1
Med23	0.00636617	0.8968749	0.06	0.047	1
Rb1	0.00640953	0.61939198	0.162	0.148	1
Mrs2	0.00657044	0.71633048	0.053	0.041	1
Flywch2	0.00659882	1.1569266	0.011	0.006	1
Hmces	0.00664038	0.48563162	0.104	0.087	1
Pbrm1	0.00668665	0.40345385	0.284	0.279	1
Ttc5	0.0067296	0.67580484	0.081	0.067	1
Eif2ak2	0.00673088	0.53680513	0.055	0.042	1
Taf9b	0.00691189	1.1732703	0.025	0.017	1
Heg1	0.00693304	0.3000671	0.107	0.09	1
B4galt4	0.00693705	1.96519554	0.011	0.006	1
Wdr1	0.00704453	0.40545081	0.315	0.316	1

Gm11110	0.00707017	0.9126594	0.012	0.007	1
Meis3	0.00716967	1.02173738	0.02	0.013	1
Dgkq	0.00733042	0.96761411	0.025	0.017	1
Arnt	0.00733562	0.60835359	0.171	0.158	1
G3bp2	0.0074489	0.44784004	0.265	0.257	1
Plxna1	0.00747001	0.47857292	0.019	0.03	1
Rbm18	0.00761919	0.5665844	0.097	0.082	1
Pyroxd1	0.00763001	0.82801436	0.059	0.047	1
Nbeal2	0.00773857	0.67797925	0.038	0.028	1
2510009E07R	0.00784272	1.3423047	0.049	0.038	1
Dnajc9	0.00788759	0.36346537	0.132	0.115	1
Tmem165	0.00803339	0.73868376	0.092	0.078	1
Enkur	0.00815745	1.60344366	0.011	0.006	1
Gm26798	0.00815881	1.38636755	0.011	0.006	1
Tmem29	0.00817045	0.81799941	0.066	0.053	1
Zfp825	0.00818809	1.0188991	0.018	0.011	1
Ctnnb1	0.00858708	0.42795569	0.291	0.291	1
Tmem234	0.00860739	0.32255449	0.407	0.426	1
Znrf2	0.00864244	0.57478971	0.211	0.204	1
Prkd2	0.00868759	0.36209522	0.049	0.037	1
Chd6	0.00869549	0.37737468	0.179	0.162	1
Slc43a3	0.0086997	1.19197904	0.02	0.013	1
Zfp871	0.0087152	0.55295043	0.187	0.174	1
Exo5	0.00881435	1.21900684	0.016	0.01	1
Cyb561d2	0.00882049	1.04050809	0.048	0.037	1
Lmbr1l	0.00884663	0.57933987	0.074	0.061	1
Ryk	0.00888967	0.90246101	0.029	0.02	1
Hmgb3	0.00890247	0.64583977	0.034	0.025	1
Stx4a	0.00893536	0.25523667	0.13	0.16	1
Fbxo3	0.00902706	0.73358364	0.09	0.076	1
Trim23	0.00905788	0.62547262	0.036	0.026	1
Pex16	0.00910694	0.60986573	0.092	0.078	1
Xrcc5	0.00912846	0.50016864	0.023	0.016	1
Retreg2	0.00937845	0.77729028	0.092	0.078	1
Rabggta	0.00938527	0.71515222	0.042	0.031	1
Fam219b	0.00943469	0.78868098	0.028	0.019	1
Cep170	0.00949065	0.27968338	0.083	0.106	1
Zc3h13	0.00966544	0.45357531	0.292	0.292	1
Zdhhc7	0.00977212	0.62532771	0.058	0.046	1
Mrpl54	0.00980492	0.48258785	0.3	0.307	1
Zfp282	0.0098269	1.00690518	0.025	0.017	1
Mterf1a	0.00990673	0.6290952	0.047	0.036	1
D430042O09F	0.00995137	0.72392545	0.078	0.065	1
Slco2b1	0.01011796	1.20537542	0.024	0.016	1
Rab1a	0.01012598	0.49295681	0.266	0.265	1
AC139671.1	0.01021652	0.51922179	0.018	0.011	1
Fbxl15	0.01042035	0.96616724	0.031	0.022	1
4930481A15R	0.01048623	0.91781176	0.019	0.012	1

Amer1	0.01051594	1.42973353	0.012	0.007	1
Abi2	0.01054514	0.89025022	0.037	0.027	1
Smarca5	0.01054781	0.26293369	0.489	0.519	1
Zcchc9	0.01056097	0.52617492	0.184	0.171	1
Zfp35	0.01061858	0.8282512	0.023	0.016	1
Cenpa	0.01091424	0.31317451	0.393	0.395	1
2210016F16Ri	0.01091572	0.79599319	0.076	0.064	1
Sparcl1	0.0110152	1.05243554	0.012	0.007	1
1700097N02R	0.01104356	0.70972572	0.021	0.014	1
Rad51d	0.01125171	0.96603613	0.031	0.023	1
Zbtb37	0.01129153	0.58488379	0.046	0.035	1
Lysmd4	0.0113294	1.21512746	0.012	0.007	1
Dhrs7	0.01143889	0.68148297	0.093	0.08	1
Gm4070	0.01147768	0.66658167	0.047	0.037	1
Myl4	0.01148858	0.59076587	0.025	0.017	1
Rhobtb2	0.0114931	0.25690637	0.047	0.036	1
Rab33b	0.01150045	0.69080825	0.054	0.043	1
Fbxw11	0.0117582	0.75004513	0.132	0.119	1
Slc25a30	0.01176072	0.81887424	0.078	0.065	1
Slc2a9	0.01177059	1.11172597	0.016	0.01	1
Creb3l1	0.01178419	0.93320787	0.021	0.014	1
Uba2	0.01183858	0.62334397	0.122	0.108	1
Nipal1	0.01187888	1.15242757	0.016	0.01	1
Hist3h2ba	0.01207504	1.02643265	0.029	0.021	1
Adamts10	0.01207979	0.63079361	0.034	0.025	1
Trir	0.0120866	0.33835555	0.347	0.353	1
Trbv2	0.01216619	1.01943526	0.01	0.006	1
Fam207a	0.01223752	0.82529465	0.086	0.073	1
C2cd3	0.01228384	0.49245688	0.063	0.051	1
Zfp420	0.01252711	0.92557916	0.01	0.005	1
Gnptg	0.01262952	0.61946376	0.074	0.061	1
Dctn3	0.01263208	0.47528153	0.274	0.274	1
Jkamp	0.01271095	0.5953929	0.06	0.048	1
Usp30	0.01274599	0.92387091	0.019	0.013	1
Tmem237	0.01275785	1.21828901	0.018	0.012	1
Abcc1	0.0128566	0.74920221	0.105	0.092	1
Zfp952	0.01338367	0.84020959	0.034	0.025	1
Ric8b	0.01339958	0.96722091	0.027	0.019	1
Asb3	0.01345428	0.81004324	0.037	0.028	1
Zfp995	0.01348892	1.1435237	0.016	0.01	1
Trim14	0.01356193	0.59923539	0.014	0.009	1
Snrnp200	0.01357752	0.53619053	0.181	0.169	1
Ppfibp1	0.01363324	0.98174144	0.05	0.04	1
Tspyl3	0.0136635	1.14992623	0.012	0.007	1
Ubxn2a	0.01373028	0.45251517	0.195	0.184	1
Lsm14a	0.01379473	0.42348842	0.195	0.183	1
Slc41a1	0.01386438	0.67858863	0.09	0.077	1
Ddx42	0.01391357	0.40238282	0.255	0.249	1

Mmgt2	0.01395707	0.88686326	0.035	0.026	1
Ccng2	0.01402903	0.34412446	0.112	0.097	1
Tprgl	0.01404613	0.40472666	0.225	0.216	1
Osbpl7	0.01408525	0.82131749	0.031	0.023	1
Snapin	0.01409766	0.74878767	0.06	0.049	1
Cep89	0.01410881	1.15657828	0.014	0.009	1
Stim1	0.01412639	0.35812391	0.163	0.147	1
Tmem191c	0.01418972	0.78079184	0.03	0.022	1
Faah	0.01439413	0.37041764	0.122	0.106	1
2810405F17Ri	0.01439785	0.70133078	0.022	0.015	1
Setd2	0.01440319	0.35655053	0.237	0.227	1
Casp3	0.01451753	0.56280652	0.123	0.109	1
Snx33	0.01452221	0.94627817	0.013	0.008	1
Dnah8	0.01460682	0.73600437	0.013	0.008	1
Sirt5	0.01461162	1.37301353	0.011	0.006	1
Rnf24	0.01464143	0.2571164	0.031	0.044	1
Tmx1	0.01468256	0.78131591	0.119	0.107	1
Gzma	0.01468753	0.41692516	0.05	0.039	1
Zfp689	0.01479892	1.076641	0.012	0.007	1
Cnot2	0.01493179	0.45928616	0.148	0.134	1
Setd3	0.01494238	0.50236844	0.153	0.14	1
Pex26	0.0149545	1.50223023	0.01	0.006	1
Rara	0.0149689	0.66201038	0.107	0.094	1
Rab35	0.015015	0.56626234	0.134	0.121	1
Ttc3	0.0152456	0.51859478	0.121	0.107	1
Hsf2	0.01525341	1.06504478	0.05	0.04	1
Cdk2ap1	0.01532208	0.67219976	0.125	0.113	1
Mtbp	0.01532825	0.77939652	0.022	0.015	1
Klk8	0.01554113	0.26846587	0.115	0.099	1
Zfp382	0.01562491	1.18790254	0.023	0.016	1
Abca7	0.015748	0.62165102	0.074	0.061	1
Limd2	0.01591104	0.30851777	0.324	0.319	1
Prpf4b	0.01595081	0.2976614	0.394	0.403	1
Tes	0.01595599	0.27827412	0.344	0.342	1
Unkl	0.01596322	0.82382169	0.041	0.032	1
Tmem50a	0.0160265	0.29738323	0.355	0.364	1
Stam2	0.01616344	0.65178586	0.128	0.116	1
Zmym2	0.01650847	0.62278659	0.092	0.08	1
Usp19	0.01655268	0.43267594	0.202	0.192	1
Herc2	0.01693414	0.56543512	0.147	0.135	1
Grk6	0.017049	0.44065411	0.148	0.134	1
Bcdin3d	0.01714775	0.97105965	0.023	0.016	1
Sh3bp5l	0.01726057	0.8188626	0.027	0.02	1
Endod1	0.01731129	1.01035445	0.062	0.052	1
Tpd52l2	0.01736241	0.56590074	0.159	0.148	1
2310011J03Ril	0.01740263	0.68148442	0.098	0.085	1
Rab11fip2	0.01751762	0.63818086	0.091	0.078	1
Rab10	0.01752227	0.35267065	0.335	0.342	1

Fam98b	0.01762601	0.54379207	0.169	0.157	1
Rbm33	0.01772968	0.43847958	0.216	0.204	1
Plekhb2	0.01782593	0.60350914	0.146	0.134	1
Dcaf1	0.01783708	0.63228323	0.092	0.079	1
Exosc8	0.01784692	0.51147673	0.124	0.111	1
4833420G17R	0.01791265	0.4096009	0.177	0.164	1
Gm4924	0.01791953	1.14879913	0.01	0.005	1
Arf5	0.01796586	0.26742262	0.451	0.483	1
Plekha3	0.01801624	0.65578377	0.097	0.084	1
Ppp2r2d	0.01801763	0.43124993	0.224	0.216	1
Kantr	0.0181675	0.89726611	0.023	0.016	1
Tmem87a	0.01817848	0.55488256	0.097	0.084	1
Ttc33	0.01835673	0.70826707	0.067	0.056	1
Sap30l	0.01848062	0.51349229	0.115	0.101	1
Mrpl44	0.01869496	0.66015885	0.051	0.04	1
Frs2	0.01880756	0.71810733	0.062	0.051	1
Pms2	0.01892967	0.55980693	0.043	0.034	1
Med17	0.01905598	0.68254484	0.061	0.05	1
Midn	0.01923657	0.25996918	0.092	0.114	1
Rnase4	0.019253	0.41377428	0.051	0.041	1
Grb7	0.01941964	0.56036766	0.037	0.028	1
Med25	0.01963647	0.65016057	0.069	0.058	1
Rgp1	0.01973396	0.73768448	0.027	0.019	1
Atg12	0.01975436	0.4912832	0.17	0.159	1
Tmem238	0.01996039	0.87232828	0.047	0.037	1
Ppp2cb	0.01997212	0.58618825	0.108	0.095	1
Lig4	0.02000464	0.91375203	0.012	0.007	1
Zfp953	0.02002816	1.040891	0.017	0.011	1
Tmem206	0.02006186	0.32104469	0.007	0.014	1
Tbc1d9b	0.02017772	0.76553893	0.058	0.047	1
Cdc25b	0.02037241	0.64528267	0.035	0.026	1
Trpc4ap	0.02038591	0.48952479	0.204	0.198	1
Klhl9	0.02041576	0.7909515	0.103	0.091	1
Capn15	0.02041889	0.59501884	0.052	0.042	1
Naxe	0.02070395	0.25135382	0.062	0.078	1
Tssc4	0.02070673	0.90907072	0.047	0.038	1
Lgalsl	0.0207915	0.9345275	0.023	0.016	1
Cry1	0.02079636	0.5809659	0.091	0.078	1
Uevld	0.02095133	1.23339254	0.014	0.009	1
Pcnp	0.02103786	0.4065176	0.248	0.245	1
Btd	0.02154035	1.09109203	0.025	0.018	1
AU041133	0.02188564	0.51135807	0.023	0.016	1
Gphn	0.02193064	0.87667712	0.036	0.027	1
Psmd10	0.02220681	0.29144642	0.062	0.079	1
Pacsin1	0.02222455	1.04550907	0.019	0.013	1
Eif2d	0.0223589	0.61153167	0.051	0.04	1
5730455P16R	0.02247634	0.89744969	0.032	0.024	1
Glce	0.02260844	1.0186497	0.028	0.02	1

Slc35d2	0.02284313	0.92442395	0.018	0.012	1
Nipsnap1	0.0229346	0.70881245	0.025	0.018	1
Fam172a	0.0229429	0.52792215	0.149	0.138	1
Aldh18a1	0.02307926	0.88769381	0.035	0.026	1
Smim11	0.02319663	0.59376305	0.148	0.137	1
Cep104	0.02322303	0.98547646	0.028	0.02	1
Caprin2	0.02325897	0.92792333	0.012	0.007	1
Dnajc12	0.02327122	1.19751811	0.049	0.039	1
Acad8	0.02334743	1.10530708	0.018	0.012	1
Ccdc59	0.02338854	0.41820366	0.279	0.282	1
Cfap97	0.02407763	0.5511716	0.059	0.048	1
Srebf2	0.02414237	0.40065948	0.306	0.312	1
Sirt1	0.02419672	0.59702843	0.101	0.089	1
Gm26853	0.02432092	0.49112204	0.079	0.067	1
Arf6	0.02434538	0.43052816	0.277	0.281	1
Dnaaf5	0.02436744	0.86673019	0.015	0.01	1
Ggps1	0.02444608	0.46888351	0.099	0.086	1
Gas2l1	0.02448121	0.27971719	0.02	0.03	1
Chmp2a	0.02466237	0.35698017	0.353	0.366	1
Dynll2	0.02487727	0.57319121	0.143	0.131	1
Prmt2	0.02492872	1.23575228	0.015	0.01	1
Tnfaip1	0.02501847	0.98243517	0.051	0.041	1
Rfesd	0.02502038	0.84131351	0.025	0.018	1
Lnpk	0.0251049	0.56146776	0.097	0.084	1
Shprh	0.02541553	0.51931312	0.05	0.04	1
Inpp5e	0.02548606	0.69905669	0.021	0.015	1
Slc25a42	0.0262355	1.15957769	0.011	0.006	1
Ube2h	0.02627198	0.45668432	0.218	0.211	1
Tmem161b	0.02637863	0.74894009	0.047	0.038	1
Etfrf1	0.02638349	0.67255411	0.043	0.034	1
Slc25a32	0.02673918	0.71085503	0.035	0.027	1
Crebzf	0.02683109	0.41511161	0.126	0.113	1
Asb7	0.02693493	1.16337491	0.021	0.015	1
Smad4	0.02705732	0.47578445	0.252	0.25	1
Crtc3	0.0272058	0.2956006	0.125	0.152	1
Ddx17	0.02732154	0.30094523	0.334	0.343	1
Rbbp4	0.02752191	0.36724976	0.269	0.266	1
Fastkd3	0.02770683	0.83583878	0.018	0.012	1
Fkbpl	0.0278125	0.8925262	0.011	0.007	1
Tpi1	0.02840958	0.34362671	0.258	0.253	1
Klhl18	0.02846342	0.86012135	0.037	0.029	1
Zkscan3	0.02859875	0.46783467	0.078	0.066	1
Tmem138	0.02863981	1.24275733	0.015	0.01	1
Gls	0.02864403	0.25250279	0.458	0.482	1
Ext2	0.0287731	0.25481044	0.029	0.04	1
Tm9sf3	0.02895096	0.32477559	0.367	0.385	1
Cdc27	0.02899033	0.54096963	0.098	0.086	1
Nxt1	0.02908082	0.83085335	0.078	0.067	1

Zfyve27	0.02908955	0.67435529	0.059	0.049	1
Nfkb2	0.02909318	0.42603669	0.298	0.304	1
Abcd4	0.02934258	0.8538131	0.028	0.021	1
Atxn7	0.02936221	0.4962608	0.145	0.133	1
Rassf1	0.02944662	0.25425923	0.114	0.138	1
Qsox1	0.02980892	0.45466953	0.073	0.062	1
Zfp729b	0.02996525	0.86957759	0.049	0.039	1
Cops8	0.03011959	0.25135486	0.112	0.135	1
Mtif3	0.03030203	0.71819112	0.034	0.026	1
4930518I15Ril	0.03030308	0.92396541	0.01	0.006	1
Coa5	0.03037514	0.52114737	0.166	0.157	1
Ubr5	0.03037856	0.42918744	0.227	0.222	1
Zfp787	0.03076369	0.67921893	0.089	0.078	1
Tdp2	0.03077672	0.56859132	0.082	0.071	1
Fli1	0.03090142	0.37922802	0.264	0.261	1
Actr8	0.03100745	0.73612453	0.041	0.032	1
Xist	0.03162774	0.25027933	0.425	0.423	1
Ralgapa2	0.03191616	0.64164167	0.052	0.043	1
Cog6	0.0320767	0.88328223	0.042	0.033	1
Rab6a	0.03216532	0.5230651	0.185	0.178	1
E2f3	0.03224391	0.40178789	0.021	0.03	1
Mob3a	0.03232323	0.75394924	0.064	0.054	1
Gm2245	0.03240002	0.69744616	0.021	0.015	1
Mbd6	0.03260011	0.69473853	0.066	0.056	1
Crkl	0.0327707	0.50978377	0.101	0.089	1
Tmem209	0.03293508	0.71765136	0.048	0.039	1
Dcun1d1	0.03303303	0.50884557	0.125	0.114	1
Lbr	0.03312636	0.31375305	0.257	0.248	1
5830468F06Ri	0.033471	0.88661383	0.01	0.006	1
Ice1	0.03349994	0.65085087	0.084	0.073	1
Rbm28	0.03371718	0.4774579	0.157	0.146	1
Cbx6	0.03375839	0.77203026	0.025	0.018	1
Wdfy2	0.03407578	0.60208548	0.074	0.063	1
Med20	0.03425632	0.59506725	0.053	0.044	1
Lca5	0.03443791	1.20623905	0.01	0.006	1
Gm26514	0.03449025	1.04595867	0.01	0.006	1
1110051M20F	0.03478635	1.12588069	0.016	0.011	1
Bloc1s3	0.03484609	0.9123064	0.024	0.018	1
Lmf2	0.03488522	0.76610894	0.049	0.04	1
Pura	0.03494212	0.39144216	0.186	0.175	1
Trmt11	0.03504853	0.78253538	0.036	0.028	1
Tmx3	0.035055	0.50901391	0.153	0.143	1
Zfp212	0.03530233	0.60323294	0.049	0.04	1
Ubxn4	0.03566172	0.35876217	0.368	0.386	1
Bhlhb9	0.03591269	0.71978476	0.019	0.014	1
Gm12166	0.03600143	1.23385283	0.013	0.009	1
Knop1	0.036148	0.57066306	0.102	0.091	1
Syf2	0.036263	0.27396867	0.396	0.413	1

Kdsr	0.03680903	0.50497198	0.063	0.053	1
Tlcd1	0.03684785	0.81165776	0.023	0.017	1
Zfp626	0.0369616	0.80137152	0.033	0.025	1
Aebp1	0.03731123	1.05652137	0.012	0.008	1
Ocel1	0.03736397	0.89270575	0.053	0.044	1
Phldb3	0.03757591	0.85391091	0.019	0.013	1
Rbm45	0.03763101	0.82340059	0.027	0.02	1
Ngrn	0.03766698	0.66692132	0.044	0.036	1
Otud7b	0.03772368	0.31006948	0.055	0.045	1
Prkab2	0.03824375	0.33633346	0.032	0.042	1
Fam222b	0.03834461	0.84477905	0.029	0.022	1
Zfp958	0.03839637	0.84869299	0.039	0.031	1
Adss	0.03843679	0.42873599	0.25	0.25	1
Tmem109	0.03851888	0.40494696	0.059	0.074	1
5033430I15Ril	0.03861543	0.83317492	0.017	0.011	1
Whamm	0.03865116	0.49522124	0.084	0.073	1
Ap1ar	0.0387425	0.45655769	0.075	0.064	1
Zfp26	0.03883144	0.8497049	0.057	0.048	1
Axin2	0.03918482	0.47153369	0.044	0.035	1
Plpp6	0.03920391	0.53776528	0.036	0.028	1
Tbcel	0.03921694	0.41945646	0.076	0.065	1
Zscan26	0.03931008	0.58060215	0.052	0.043	1
Commd10	0.03947906	0.26335126	0.025	0.034	1
Nkap	0.03999582	0.59303595	0.121	0.11	1
Champ1	0.04001265	0.72847625	0.044	0.036	1
Nosip	0.04006428	0.63345338	0.066	0.055	1
Spryd3	0.04027539	0.78395118	0.036	0.029	1
Pot1a	0.04032922	0.56201547	0.05	0.041	1
Usp4	0.04045139	0.46917417	0.112	0.1	1
Ldb1	0.04060482	0.45816144	0.125	0.113	1
Asb6	0.0406504	0.74702897	0.039	0.031	1
Phgdh	0.04101793	0.43113436	0.064	0.054	1
Tm2d1	0.04105084	0.26162359	0.132	0.158	1
Dis3l	0.04111762	1.12676153	0.019	0.013	1
Bcr	0.04111973	0.66478448	0.038	0.03	1
Rtl8a	0.0411611	0.99134084	0.032	0.025	1
Bag4	0.04119329	0.73710828	0.058	0.048	1
Iars2	0.04122385	0.75395796	0.082	0.071	1
Uba6	0.0413096	0.60524003	0.112	0.102	1
Gm47015	0.04136212	0.48345636	0.051	0.042	1
Gna12	0.04160345	0.36656007	0.034	0.044	1
Zfp110	0.04184419	0.47423216	0.074	0.064	1
Yes1	0.04214842	0.47967215	0.03	0.023	1
Zfp574	0.0424934	0.70326894	0.037	0.029	1
Aak1	0.04265841	0.38736886	0.141	0.129	1
Mrps36	0.04285466	0.25097861	0.1	0.12	1
Ppp6r3	0.04320336	0.35769898	0.227	0.222	1
Trmt6	0.04326304	0.27680711	0.062	0.077	1

Alg13	0.04331395	0.25015714	0.021	0.029	1
Rnf20	0.04336703	0.42049411	0.172	0.162	1
Mbtps1	0.04342544	0.42531998	0.144	0.133	1
Ednrb	0.04359856	0.39863971	0.039	0.031	1
Srprb	0.04363244	0.62976087	0.069	0.059	1
Hpf1	0.04369021	0.42429708	0.197	0.19	1
Lrrc47	0.04376919	0.65696787	0.09	0.08	1
Tbc1d10b	0.04384643	0.71177227	0.083	0.073	1
Rell1	0.04387955	0.58353229	0.104	0.093	1
Gtdc1	0.04403636	0.62758353	0.06	0.051	1
Etfbkmt	0.04418847	1.05757479	0.015	0.01	1
Zfp994	0.04421753	0.83255848	0.03	0.024	1
Zfp748	0.04427972	0.72562187	0.032	0.025	1
Arfgap1	0.04449684	0.58107698	0.06	0.05	1
Prkcq	0.04479981	0.37680709	0.06	0.05	1
Ostm1	0.04551041	0.48658921	0.086	0.075	1
Naa16	0.04572257	0.54796389	0.053	0.044	1
C2cd5	0.04582302	0.46698362	0.057	0.048	1
Mov10	0.0459962	0.6567682	0.027	0.021	1
Phospho2	0.0460047	0.60352128	0.057	0.048	1
Camk2g	0.04635187	0.65917024	0.052	0.043	1
Sys1	0.04658309	0.31482031	0.318	0.326	1
Cwc22	0.04671059	0.50076541	0.113	0.103	1
Exoc6b	0.04678261	0.76861537	0.052	0.043	1
Cenpq	0.04695106	0.49815498	0.098	0.087	1
Prr7	0.0476143	0.27542712	0.177	0.164	1
Pwwp2b	0.04762361	1.06439355	0.015	0.01	1
Gm11713	0.04778131	0.91526053	0.017	0.012	1
Csrp1	0.04783279	0.54916564	0.145	0.136	1
Dpy19l3	0.04799858	0.7799209	0.043	0.035	1
Wrap53	0.04802193	1.13346465	0.016	0.011	1
Pik3c3	0.04867809	0.72783955	0.064	0.054	1
Nap1l4	0.04873074	0.42134105	0.195	0.188	1
Ormdl1	0.04901592	0.60531738	0.044	0.036	1
Gm21781	0.04902403	1.06400993	0.01	0.006	1
Ocrl	0.04977347	0.39185716	0.023	0.031	1
Kcnab2	0.04979012	0.52678535	0.063	0.053	1
Rundc3b	0.05028334	0.51116161	0.072	0.062	1
Pon3	0.05091989	1.16970538	0.022	0.016	1
Znfx1	0.05106555	0.76120418	0.06	0.051	1
Gdpd3	0.05112385	1.01860301	0.023	0.031	1
Palld	0.05116479	0.75582156	0.02	0.028	1
Fars2	0.0513238	0.25409844	0.029	0.039	1
Akap10	0.05132984	0.60079516	0.078	0.069	1
Pofut1	0.05167388	0.53669934	0.038	0.031	1
Rnf31	0.05269767	0.55357846	0.057	0.048	1
Supt16	0.05284072	0.47359845	0.217	0.214	1
Armc7	0.0537117	0.66239495	0.04	0.032	1

Cops7b	0.05446131	0.94901229	0.026	0.02	1
Marf1	0.05524113	0.42065416	0.129	0.119	1
Pou6f1	0.05579141	0.45098964	0.036	0.029	1
Cdk16	0.05587762	0.79850902	0.038	0.031	1
Tubgcp3	0.05608605	0.50163345	0.074	0.064	1
Elof1	0.05610199	0.25214399	0.088	0.106	1
Yipf2	0.05617799	0.76198451	0.021	0.016	1
Fis1	0.05619556	0.3005821	0.355	0.376	1
Tmem263	0.05624441	0.41439232	0.057	0.047	1
Arl6ip4	0.05627902	0.57433136	0.113	0.103	1
Ankrd16	0.05646182	0.51887548	0.036	0.029	1
Abhd12	0.05668968	0.34070448	0.066	0.081	1
Gpatch2	0.05721198	0.50529145	0.058	0.049	1
Ctr9	0.05737828	0.42691972	0.1	0.09	1
Apba3	0.05749795	1.13024	0.026	0.02	1
Ric8a	0.05758984	0.74295147	0.09	0.081	1
Pitpnb	0.05759827	0.68547209	0.106	0.097	1
Taf1a	0.05782151	0.73338175	0.021	0.015	1
Tomm5	0.05793059	0.45476682	0.206	0.201	1
Arhgef5	0.05829968	0.85120355	0.011	0.007	1
Lztr1	0.05876077	0.83405277	0.029	0.022	1
Gm46430	0.05900319	1.12863782	0.011	0.007	1
Ugdh	0.05910655	0.64817126	0.069	0.06	1
Nsmaf	0.05957606	0.48131624	0.132	0.123	1
Mir17hg	0.0601705	0.59803339	0.024	0.018	1
Aamdc	0.06038729	0.66084455	0.031	0.024	1
Fbxo4	0.06041883	0.69830376	0.045	0.037	1
Acin1	0.06065555	0.28695069	0.354	0.366	1
Micu2	0.06102595	0.60865774	0.09	0.08	1
Tmc8	0.06114646	0.7774584	0.017	0.012	1
Upf2	0.06120528	0.3398648	0.166	0.155	1
AC165953.2	0.06135878	0.87169031	0.014	0.01	1
Zfp235	0.06152516	0.99455548	0.012	0.008	1
C920006O11F	0.06171555	0.77988864	0.011	0.007	1
Tm9sf2	0.06189783	0.4819145	0.173	0.167	1
Hnrnpul1	0.06214601	0.36472873	0.29	0.293	1
Tbpl1	0.06218199	0.45044855	0.096	0.085	1
Uhrf1bp1	0.06223773	1.41900583	0.01	0.006	1
Zdhhc17	0.06266615	0.48882039	0.053	0.044	1
Zfp560	0.06275351	0.47051764	0.083	0.073	1
Afdn	0.06304465	0.49947141	0.028	0.037	1
Bet1l	0.06320559	0.57716224	0.121	0.113	1
A430033K04R	0.06397961	1.14231913	0.01	0.007	1
Smug1	0.06422263	0.8130442	0.025	0.019	1
Rab11fip4	0.06432617	0.74967698	0.028	0.022	1
4931406P16R	0.06436233	0.6027716	0.094	0.084	1
Golga1	0.0644001	0.96484514	0.021	0.016	1
Ei24	0.06589929	0.48109372	0.069	0.06	1

Ltn1	0.06610842	0.64688409	0.077	0.068	1
Nrde2	0.06614405	0.52401935	0.059	0.05	1
Usp38	0.06659121	0.32786956	0.147	0.137	1
Mum1	0.06663553	0.55866629	0.05	0.042	1
Syap1	0.06720455	0.29244869	0.092	0.11	1
Ube2o	0.06741651	0.59778246	0.051	0.043	1
Smim24	0.06768061	0.29171502	0.019	0.026	1
Trappc11	0.06795357	0.64063568	0.046	0.038	1
Hexim1	0.06799084	0.36566442	0.188	0.18	1
Mrpl36	0.06804632	0.25283791	0.108	0.128	1
Kxd1	0.0684021	0.26774288	0.15	0.177	1
Klhl36	0.06849836	0.89790845	0.016	0.011	1
Narf	0.06859707	0.57707249	0.066	0.057	1
Zfp213	0.06888556	1.11944157	0.01	0.006	1
Rnaset2b	0.06969625	0.59693542	0.101	0.092	1
Maml1	0.06996764	0.66700485	0.08	0.071	1
Aldh3a2	0.07008349	0.64327151	0.05	0.042	1
E4f1	0.07060784	0.40077725	0.089	0.079	1
B9d1	0.07071009	1.06577956	0.01	0.006	1
Ttc4	0.07101192	0.61817681	0.06	0.052	1
Cinp	0.0713091	0.44713622	0.019	0.026	1
Kif21b	0.07151104	0.52779042	0.091	0.081	1
Trip12	0.07151174	0.3600393	0.298	0.306	1
Pyurf	0.07223825	0.25684233	0.038	0.048	1
Decr2	0.07242538	0.85607815	0.019	0.014	1
Acad9	0.07301832	0.91066146	0.023	0.018	1
Pou5f2	0.07325266	0.64015898	0.015	0.011	1
Dynlt3	0.07335057	0.25439441	0.115	0.135	1
AU019823	0.07352683	0.29117028	0.1	0.118	1
Capn10	0.07373149	1.11887885	0.018	0.013	1
Fra10ac1	0.07384316	0.62342355	0.023	0.018	1
Rad17	0.0738628	0.51535875	0.073	0.064	1
Traf7	0.07429454	0.57900889	0.075	0.066	1
Wdyhv1	0.07452363	0.54038488	0.047	0.039	1
Aldoc	0.07488689	1.10554954	0.029	0.023	1
Gabbr1	0.07503899	0.48749806	0.07	0.062	1
Sfxn3	0.07508603	0.38638969	0.052	0.063	1
Rdx	0.07531973	0.37884604	0.277	0.282	1
Gm10093	0.07603956	0.67793553	0.031	0.025	1
Arl6ip5	0.0760723	0.4428351	0.217	0.215	1
Cisd2	0.07607809	0.41966053	0.281	0.289	1
Ring1	0.07645791	0.32851294	0.037	0.046	1
Abhd8	0.07649745	0.43915863	0.039	0.032	1
Erg28	0.07732481	0.54270804	0.084	0.075	1
D230025D16F	0.07733415	0.63107957	0.039	0.032	1
Rab26	0.07740732	0.91940167	0.02	0.015	1
Kbtbd2	0.07742018	0.52583885	0.122	0.114	1
Tap2	0.07792443	0.45703499	0.188	0.185	1

Ulk3	0.07841602	0.81732813	0.016	0.012	1
Hibadh	0.07859502	0.54045578	0.098	0.089	1
Dgcr8	0.07947347	0.98331862	0.027	0.021	1
Fam120aos	0.07961848	1.0177695	0.012	0.009	1
B9d2	0.08059424	0.27425522	0.101	0.119	1
5430403G16R	0.08077622	0.559388	0.012	0.008	1
1110004F10Ri	0.08089401	0.31553398	0.306	0.315	1
Ptbp1	0.08144141	0.29254599	0.318	0.326	1
Ipo8	0.0825473	0.55197247	0.07	0.062	1
Rbm12	0.0827156	0.44425549	0.071	0.062	1
Fkrp	0.08299852	0.80971846	0.037	0.031	1
Vezf1	0.08303667	0.35367432	0.173	0.165	1
Ddx20	0.08340807	0.59869156	0.035	0.029	1
Rbm6	0.0838174	0.45653707	0.177	0.171	1
Zfp687	0.084806	0.59818978	0.036	0.029	1
Nab1	0.08510843	0.29392332	0.312	0.321	1
Arl6ip6	0.08521164	0.55563111	0.073	0.064	1
Pcnx4	0.08545153	0.99954443	0.015	0.011	1
Ssh1	0.08547771	0.61492511	0.054	0.047	1
Rab1b	0.08601453	0.27190947	0.091	0.107	1
Frmd8	0.08606647	0.60251454	0.038	0.032	1
Cyb5d2	0.0862221	0.94539325	0.021	0.016	1
Tmem173	0.08623322	0.61424293	0.089	0.081	1
Mrpl2	0.08635916	0.33321731	0.051	0.062	1
Mthfd1l	0.08641857	0.58638881	0.031	0.025	1
Rab3gap1	0.08688943	0.49090175	0.175	0.17	1
Rbm22	0.08717216	0.42347108	0.228	0.227	1
Lrp8	0.08720111	0.29081327	0.01	0.014	1
Taok2	0.08720904	0.5827198	0.069	0.061	1
Cd59a	0.08723135	0.80689085	0.01	0.007	1
Irf2bp1	0.08724317	0.63811741	0.04	0.033	1
Paqr4	0.08739437	1.35606274	0.01	0.007	1
Ppm1j	0.08745201	0.84933743	0.021	0.016	1
Gbp9	0.08838334	0.50224104	0.067	0.058	1
Supt7l	0.08841549	0.69422968	0.035	0.029	1
Galnt4	0.08842959	0.81779464	0.011	0.007	1
2900076A07R	0.08866104	0.84656783	0.018	0.014	1
Rdh11	0.08866324	0.51547425	0.054	0.046	1
Aqr	0.08867635	0.50216279	0.156	0.149	1
Cebpzos	0.08867969	0.63379081	0.064	0.056	1
Lypla1	0.08868862	0.57779392	0.113	0.105	1
Ankrd40	0.08897097	0.68430024	0.076	0.068	1
Mapk8ip3	0.08906482	0.57756365	0.084	0.076	1
Ecsit	0.08931842	0.53203508	0.041	0.034	1
Carm1	0.08971398	0.26272721	0.021	0.028	1
Rdh14	0.09047285	0.68507737	0.032	0.026	1
Phax	0.09052111	0.54229258	0.114	0.106	1
Ormdl3	0.09060406	0.2695199	0.091	0.081	1

Snf8	0.09156335	0.35074212	0.157	0.185	1
Wtap	0.09179913	0.45945778	0.202	0.199	1
Arid3a	0.09226794	0.6391316	0.054	0.047	1
Ppp4r3a	0.09277681	0.39354769	0.225	0.222	1
Cwc15	0.09289081	0.25295639	0.377	0.396	1
Zfp780b	0.09292445	0.59900664	0.028	0.022	1
Hdhd5	0.09296682	0.55355329	0.025	0.02	1
Naf1	0.09315864	0.27756696	0.034	0.042	1
Gm26890	0.09324519	0.90520309	0.014	0.01	1
Isoc2b	0.09333246	0.95400543	0.019	0.014	1
Crim1	0.09371511	0.42293653	0.06	0.052	1
Hist1h1d	0.09397628	0.35413416	0.077	0.068	1
Zfp668	0.09453099	0.80072442	0.015	0.011	1
Ccnl2	0.09465602	0.33938472	0.292	0.3	1
Ncstn	0.09478718	0.26815347	0.091	0.107	1
Pnpla6	0.0949404	0.89914263	0.022	0.017	1
Aktip	0.09503658	0.82129225	0.03	0.024	1
Gm14154	0.09546015	1.15104427	0.01	0.006	1
Fbxo8	0.09547856	0.49080513	0.067	0.059	1
Bcorl1	0.09568478	0.88264061	0.016	0.012	1
Dgcr2	0.0959108	0.78915908	0.063	0.056	1
Bnip3	0.09591687	0.37614221	0.04	0.033	1
Traf6	0.09666386	0.27099427	0.067	0.079	1
9030025P20R	0.09691742	0.78183527	0.011	0.008	1
Epc2	0.09701261	0.40413692	0.183	0.178	1
Map2k5	0.0970206	0.81166039	0.037	0.031	1
Tpgs2	0.09711469	0.76889052	0.037	0.031	1
Krtcap3	0.09720046	0.82795645	0.015	0.011	1
Runx1	0.09730023	0.26264744	0.344	0.352	1
Med28	0.09732904	0.48654216	0.227	0.229	1
B230354K17R	0.09775762	1.08033924	0.018	0.014	1
Dnajc25	0.09822196	0.28856473	0.027	0.034	1
Zfp617	0.09835869	0.49348377	0.035	0.029	1
Nsmce2	0.0991902	0.35333429	0.067	0.08	1
Slc29a1	0.09941144	0.72880092	0.056	0.049	1
Haus2	0.09986037	0.54237239	0.048	0.041	1
Ndufaf7	0.09986325	0.77323382	0.039	0.033	1
Ptpra	0.10046463	0.27953376	0.116	0.135	1
Tmed4	0.10048486	0.25046099	0.031	0.039	1
4930430F08Ri	0.10054336	0.95251245	0.013	0.01	1
Zcchc17	0.10065369	0.5008108	0.11	0.102	1
Cox11	0.10108981	0.76284048	0.037	0.031	1
Shkbp1	0.10141197	0.56798658	0.064	0.056	1
Yipf4	0.10179351	0.48160337	0.174	0.17	1
Hars2	0.10208403	0.71854669	0.031	0.025	1
Chchd5	0.10277852	0.28699591	0.047	0.058	1
Gm45871	0.10278399	1.05086282	0.01	0.007	1
Pigb	0.1028332	1.16333755	0.012	0.009	1

Ydjc	0.10292763	1.46296321	0.015	0.011	1
Polr2b	0.10430471	0.64391775	0.077	0.069	1
Pym1	0.1043352	0.48554407	0.096	0.087	1
Platr25	0.10467396	0.74805494	0.014	0.011	1
Nek4	0.10479591	1.21122764	0.013	0.009	1
Taf12	0.10498582	0.56336042	0.068	0.06	1
Angel2	0.10511765	0.36204363	0.162	0.154	1
Slc25a14	0.10523287	1.20326706	0.01	0.007	1
Uba3	0.105739	0.31750153	0.048	0.058	1
Mogs	0.10611818	0.28657412	0.033	0.041	1
Rbm41	0.10650294	0.55511985	0.028	0.022	1
Eogt	0.10678927	1.03223483	0.015	0.011	1
1110038F14Ri	0.10698603	0.47024045	0.114	0.106	1
Dusp11	0.1070764	0.28047799	0.364	0.381	1
Gtf3c2	0.10741353	0.59171379	0.121	0.114	1
Pced1a	0.10820754	0.8841321	0.021	0.016	1
Uba1	0.1082398	0.45417578	0.203	0.203	1
Cntrob	0.10901125	0.52154782	0.021	0.017	1
Slain2	0.10931215	0.5339515	0.141	0.135	1
Fbxl8	0.10945101	0.94039959	0.015	0.011	1
Fam45a	0.11061941	0.67315857	0.053	0.046	1
Gm20300	0.11071685	0.87376977	0.017	0.012	1
Cbl	0.11072509	0.46211076	0.253	0.258	1
2700016F22Ri	0.11099878	0.85729098	0.015	0.012	1
Mphosph9	0.11127406	0.61546149	0.028	0.022	1
Cmip	0.11136627	0.33520738	0.275	0.282	1
Fancc	0.11158833	0.99329193	0.026	0.021	1
Vamp7	0.11159568	0.75327435	0.065	0.058	1
Them4	0.11184798	0.8569359	0.023	0.018	1
Tmem8	0.11210311	0.80898462	0.029	0.024	1
Spsb3	0.11235794	0.48873842	0.076	0.068	1
C1d	0.11238747	0.31141936	0.083	0.097	1
Mfge8	0.11257725	0.59642355	0.037	0.031	1
Gm26532	0.11277921	0.62932528	0.081	0.073	1
Unc50	0.11319696	0.58607988	0.093	0.086	1
Ccdc69	0.11342054	0.58081548	0.02	0.016	1
Phf11b	0.11343606	0.49201433	0.075	0.067	1
Accs	0.11350756	0.59574042	0.019	0.014	1
Zfp943	0.11357499	0.60861472	0.03	0.025	1
Nup107	0.11363778	0.67735236	0.027	0.021	1
Usp53	0.11375881	0.45302058	0.045	0.039	1
Ppp2r3c	0.11393744	0.55597141	0.069	0.062	1
Mastl	0.11418732	1.12567167	0.01	0.007	1
Nlk	0.11423382	0.42770204	0.072	0.064	1
Ccdc82	0.11467261	0.44594016	0.102	0.094	1
Mapk8	0.11485316	0.27029701	0.068	0.08	1
Cyth2	0.11530442	0.28782637	0.099	0.115	1
Dclre1a	0.11538711	1.2372351	0.014	0.011	1

Unc13d	0.11576311	0.50619513	0.047	0.04	1
Armcx3	0.11589351	0.32985015	0.042	0.051	1
Crip2	0.11615466	0.97302977	0.022	0.017	1
Gprasp1	0.1163454	0.54570937	0.034	0.028	1
Nelfb	0.11639936	0.57030439	0.091	0.083	1
Zgrf1	0.11642282	0.85077346	0.016	0.012	1
Osbpl5	0.11695537	0.81131239	0.01	0.007	1
Fasn	0.11736967	0.62924821	0.06	0.053	1
2010109A12R	0.11789819	0.4632908	0.011	0.007	1
Minpp1	0.11851241	0.71260535	0.031	0.025	1
Cog5	0.12048355	0.63984399	0.067	0.06	1
Coa6	0.1206149	0.66325508	0.062	0.055	1
Kif1bp	0.12078336	0.65255371	0.032	0.026	1
Cep290	0.12142251	0.32367712	0.017	0.023	1
Sptbn1	0.12223497	0.26245969	0.21	0.202	1
Zfp60	0.12258219	0.93305972	0.013	0.01	1
B3galt4	0.1226782	0.75014605	0.012	0.008	1
Gcat	0.12286323	0.3644282	0.007	0.011	1
3300005D01R	0.12292461	1.10222449	0.011	0.008	1
Micu1	0.1229496	0.6420059	0.059	0.052	1
Zfp667	0.12339005	0.45765125	0.037	0.046	1
Abhd14b	0.12367732	0.31850877	0.008	0.012	1
Washc1	0.12441861	0.7122091	0.057	0.05	1
Hsbp1	0.12447196	0.38283867	0.218	0.218	1
Tmem94	0.12466704	0.28854415	0.015	0.021	1
Muc1	0.12487606	0.34941708	0.037	0.031	1
Erlec1	0.12504278	0.47067271	0.114	0.106	1
Gtf3a	0.12508358	0.65758874	0.065	0.058	1
Pdp2	0.12524352	0.8998954	0.022	0.017	1
Tnpo3	0.12541848	0.48344165	0.172	0.168	1
Rexo4	0.12561727	0.29863112	0.075	0.088	1
Golgb1	0.12582699	0.44420489	0.195	0.194	1
Mob1b	0.12602486	0.56983981	0.129	0.123	1
Dnajc4	0.12623701	0.30279304	0.031	0.039	1
Git2	0.1264627	0.29482291	0.255	0.254	1
Hltf	0.12692285	0.42067892	0.102	0.094	1
Gm26870	0.12697371	0.50972899	0.027	0.022	1
Tigd2	0.12702341	0.34744947	0.115	0.106	1
Afg3l2	0.12777241	0.58890637	0.058	0.051	1
Hadhb	0.12785835	0.395752	0.182	0.178	1
Ppp1r14c	0.12860834	0.83122866	0.013	0.009	1
Tmem26	0.12875337	0.48296839	0.007	0.011	1
Vcp	0.12919164	0.28809771	0.357	0.379	1
Terf2ip	0.129499	0.58014379	0.044	0.038	1
E2f5	0.12955798	0.28608565	0.03	0.037	1
Mr1	0.12969132	0.3177083	0.011	0.008	1
Clk4	0.12985532	0.29573625	0.277	0.284	1
Fam114a2	0.1299088	0.54181379	0.092	0.085	1

Mea1	0.13008952	0.60515543	0.112	0.106	1
Anks1	0.13043465	0.30499703	0.027	0.034	1
Sptan1	0.13157398	0.41873988	0.177	0.172	1
Bola1	0.13181378	0.32120084	0.035	0.042	1
Sppl3	0.13210676	0.28803	0.074	0.087	1
Tnfaip8l1	0.1324956	0.83311567	0.02	0.016	1
Mtpap	0.13305237	0.27385227	0.075	0.087	1
Gm26526	0.13317504	0.66931666	0.024	0.019	1
Rpl39l	0.13404991	0.91239347	0.01	0.007	1
Strip1	0.13443804	0.60637872	0.069	0.062	1
Dok1	0.13494863	0.78648824	0.07	0.063	1
Ndufs7	0.13497048	0.26222534	0.177	0.204	1
Sec13	0.13502478	0.26981884	0.096	0.111	1
Tmem216	0.13508338	1.44874985	0.01	0.007	1
Umps	0.13529445	0.66949175	0.041	0.035	1
Rhbdd2	0.13559688	0.7093305	0.024	0.019	1
Itsn1	0.136172	0.29096141	0.026	0.033	1
Mrps18c	0.13624209	0.44441907	0.197	0.197	1
Nudcd1	0.13654495	0.93391813	0.022	0.017	1
Zfp444	0.13656487	0.7851406	0.021	0.016	1
Mcm3ap	0.13689599	0.50604962	0.047	0.04	1
Alg11	0.13744387	0.80178695	0.028	0.023	1
Pja1	0.1375098	0.37972931	0.083	0.075	1
Golt1b	0.13819185	0.25218067	0.086	0.099	1
Prr14	0.13877046	0.48115323	0.105	0.098	1
Cept1	0.13899038	0.2664887	0.068	0.079	1
Med12	0.13905999	0.40008589	0.079	0.071	1
Ccdc97	0.13934185	0.64211168	0.054	0.048	1
Kat7	0.13942085	0.29856978	0.079	0.092	1
Cnn2	0.14009775	0.30248345	0.271	0.275	1
Gm43848	0.14060322	0.72223698	0.026	0.021	1
Clptm1	0.14080061	0.28937175	0.093	0.108	1
Gcdh	0.14083132	0.27757184	0.01	0.014	1
Rab19	0.14085297	0.62659276	0.04	0.034	1
Zfp865	0.14089272	0.58743379	0.045	0.039	1
Suds3	0.14166669	0.43023079	0.121	0.114	1
Plp2	0.14189567	0.49212443	0.114	0.108	1
Pde12	0.14205064	0.5561342	0.054	0.048	1
Zfp605	0.14205247	0.62741351	0.01	0.007	1
Zbtb45	0.14222315	0.83782852	0.015	0.012	1
Zfp74	0.14305173	0.87827479	0.01	0.007	1
Mocs3	0.14372915	0.85920298	0.014	0.01	1
Trub2	0.14375821	0.59249248	0.027	0.022	1
Snrpa1	0.14432706	0.2654489	0.123	0.14	1
Zfp467	0.14494429	0.82409118	0.027	0.022	1
Anks3	0.14518298	0.68404192	0.044	0.038	1
Rpusd4	0.14533494	0.62278868	0.049	0.042	1
Appbp2	0.14565462	0.2704774	0.108	0.124	1

Zfp236	0.1456835	0.48218584	0.056	0.049	1
Scly	0.14634856	0.48721694	0.031	0.026	1
Dynlrb1	0.14754465	0.37853152	0.267	0.279	1
Gm39469	0.14771731	0.26264258	0.007	0.011	1
Elk4	0.14811217	0.31247594	0.185	0.18	1
Atxn1l	0.14855056	0.28159979	0.036	0.043	1
Zfp984	0.14929593	0.59259753	0.035	0.029	1
4932422M17F	0.14941794	0.68378736	0.023	0.018	1
2610002M06F	0.1497628	0.51565001	0.036	0.031	1
Chmp1b	0.15002005	0.33742614	0.16	0.154	1
Rint1	0.15052218	0.55329242	0.036	0.03	1
Dlgap5	0.15077237	0.35565414	0.01	0.014	1
Trp53i13	0.1515808	0.76025848	0.026	0.021	1
Fbxl5	0.15196014	0.29842079	0.139	0.133	1
Nucb1	0.15209551	0.44613616	0.168	0.165	1
Itfg1	0.15221236	0.73283764	0.06	0.053	1
5730480H06R	0.15227932	0.70943172	0.024	0.02	1
Mtfmt	0.15293838	0.59092146	0.028	0.023	1
Ccndbp1	0.1529663	0.45286462	0.12	0.113	1
Ids	0.15389223	0.29624724	0.062	0.073	1
Zfyve21	0.15395624	0.79877868	0.01	0.007	1
Ing2	0.15440807	0.32155535	0.157	0.15	1
Slc10a3	0.15468244	0.59124273	0.029	0.024	1
Rbms2	0.15473831	0.6413328	0.049	0.043	1
Iffo1	0.15517592	0.80854616	0.028	0.023	1
Pianp	0.15523661	0.75860846	0.007	0.011	1
Med8	0.15530298	0.53014789	0.093	0.086	1
Cbr4	0.15544706	0.80366071	0.015	0.012	1
Ube2e3	0.15591127	0.53961494	0.113	0.108	1
Ip6k2	0.15644471	0.63043049	0.045	0.039	1
Timm44	0.15674305	0.3267239	0.086	0.099	1
Tmem62	0.15695035	0.65201802	0.018	0.014	1
Vill	0.15695173	0.40500157	0.014	0.019	1
Sertad2	0.1578259	0.36150198	0.113	0.13	1
Mtfr1	0.15828984	0.611555	0.034	0.029	1
Ruvbl1	0.1583325	0.30576621	0.064	0.074	1
Cep170b	0.15840404	0.58790324	0.015	0.011	1
Itfg2	0.1588689	0.48159239	0.036	0.03	1
Arrdc3	0.15996105	0.36074987	0.162	0.157	1
Nde1	0.15996726	0.5805865	0.044	0.038	1
Ogfod2	0.16128502	0.68710763	0.029	0.025	1
Gtpbp8	0.16159867	0.71564467	0.023	0.019	1
Mad2l1	0.16167059	0.75429108	0.027	0.022	1
Zufsp	0.16201883	0.25566145	0.229	0.226	1
Ppp1r35	0.16204699	0.48552785	0.048	0.042	1
Rbm43	0.16214691	0.46190258	0.043	0.037	1
Btbd9	0.16279264	0.31601029	0.071	0.083	1
Lrp10	0.1632389	0.37496192	0.227	0.229	1

Gtpbp6	0.16348038	0.30321678	0.03	0.037	1
Wrnip1	0.16365403	0.59517317	0.053	0.047	1
Kifap3	0.16548563	0.32607289	0.015	0.02	1
Wrn	0.16549531	0.59838375	0.069	0.063	1
Mia3	0.16565786	0.26222556	0.163	0.186	1
Gpbp1	0.16613783	0.29438909	0.352	0.37	1
Nfya	0.16639722	0.26016504	0.088	0.101	1
Tubgcp4	0.1666896	0.45445901	0.07	0.063	1
Srgap2	0.16683913	0.35999351	0.088	0.102	1
BC002059	0.16688302	0.55925833	0.019	0.015	1
Atp23	0.16699267	0.8457727	0.01	0.007	1
Cep162	0.16716981	0.39906316	0.03	0.025	1
Dolk	0.1674857	1.21285548	0.017	0.013	1
Tbl2	0.16816818	0.38562798	0.019	0.015	1
Mettl14	0.16835991	0.59701007	0.055	0.049	1
Xab2	0.16862295	0.31501778	0.049	0.057	1
Snupn	0.16920756	0.68632381	0.022	0.018	1
Nprl3	0.16946929	0.52785045	0.022	0.018	1
Prr12	0.16972452	0.88686515	0.019	0.015	1
Zfp580	0.17004342	0.85640552	0.012	0.009	1
Zfp959	0.17023433	0.70027734	0.034	0.029	1
Rmnd5b	0.17063193	0.50658479	0.039	0.034	1
Trpm7	0.17077096	0.44660612	0.194	0.195	1
Igkv1-110	0.17143635	0.98730336	0.013	0.01	1
St7l	0.17212566	0.8579884	0.039	0.034	1
Ethe1	0.17257189	0.31685702	0.053	0.063	1
Acadsb	0.17328866	0.63771383	0.044	0.039	1
Cep164	0.17359403	0.54936396	0.056	0.05	1
Stard3	0.17425587	0.47633523	0.031	0.038	1
Slc17a9	0.17464923	0.47874357	0.015	0.012	1
Rab5a	0.17514319	0.41193339	0.241	0.249	1
Zfp317	0.17519012	0.57697189	0.025	0.021	1
Polr2f	0.17540037	0.27247831	0.148	0.169	1
Foxj3	0.1755865	0.51482077	0.101	0.096	1
Uqcrc1	0.17585656	0.33693513	0.173	0.2	1
Mbd1	0.17611115	0.27567513	0.061	0.07	1
Sptlc2	0.17626934	0.3008827	0.092	0.105	1
Sav1	0.17749915	0.38037037	0.056	0.065	1
Dmac2	0.17757798	0.52656594	0.082	0.075	1
Bmp7	0.17822425	0.36523412	0.01	0.007	1
Mb21d1	0.17830373	0.62557366	0.015	0.02	1
Acsl4	0.17844606	0.2720597	0.153	0.175	1
Ncaph	0.17846273	0.6579675	0.02	0.016	1
Mrpl10	0.17858133	0.54948718	0.051	0.045	1
Akap9	0.17894184	0.41438445	0.192	0.189	1
Prkcsh	0.17911605	0.33198168	0.129	0.147	1
Abcc5	0.18088166	0.31725088	0.025	0.03	1
B230219D22R	0.1812389	0.36633948	0.257	0.265	1

Ppp1r16a	0.1812424	0.83034655	0.02	0.016	1
Trdmt1	0.18124778	0.91009684	0.011	0.008	1
Dpy19l4	0.18132665	0.72501674	0.035	0.03	1
Gprc5a	0.18164893	0.62199161	0.034	0.041	1
Zfc3h1	0.18171704	0.44549623	0.103	0.097	1
Taf1c	0.1818415	0.72142297	0.026	0.022	1
Akr1c13	0.18249494	0.93339155	0.015	0.012	1
Apobec3	0.1826225	0.30204621	0.171	0.166	1
Tmem104	0.18285642	0.63910799	0.013	0.017	1
Adar	0.18289988	0.37508087	0.077	0.07	1
Icam2	0.1831245	0.4341801	0.031	0.027	1
Kdm4b	0.18365655	0.6201709	0.03	0.025	1
Hdac3	0.18408357	0.37466492	0.036	0.043	1
Lrrc40	0.18417654	0.38608507	0.015	0.019	1
A530017D24R	0.18530671	0.27621159	0.012	0.009	1
Lman1	0.18538624	0.3412919	0.094	0.108	1
Scnm1	0.18579028	0.66359189	0.035	0.03	1
Hspbp1	0.18632993	0.41885255	0.046	0.054	1
Dcaf7	0.18650951	0.29978437	0.093	0.107	1
Dcaf10	0.18656078	0.62426586	0.053	0.048	1
Gm16973	0.18666908	0.79630811	0.017	0.014	1
Timm22	0.18684113	0.5995871	0.07	0.064	1
Ankrd50	0.18696083	0.54415029	0.052	0.046	1
Clk3	0.1872999	0.39974415	0.158	0.155	1
Clpx	0.18794416	0.61602593	0.066	0.06	1
Acly	0.18855546	0.44935718	0.166	0.164	1
Aste1	0.18874821	0.73121156	0.019	0.015	1
Golga2	0.18877338	0.57314271	0.069	0.063	1
Pafah1b2	0.18877927	0.44452073	0.109	0.103	1
Usp50	0.18906611	0.33603996	0.032	0.038	1
Slc35a4	0.18909196	0.36028726	0.062	0.072	1
Sec23b	0.18976206	0.53547068	0.129	0.125	1
Rcor1	0.18976978	0.35819005	0.118	0.111	1
4732440D04R	0.18998446	0.51646116	0.047	0.041	1
Gm17018	0.19005525	0.26102281	0.056	0.065	1
Fam149b	0.1905712	0.66274675	0.024	0.02	1
Rhebl1	0.19098183	0.75807741	0.014	0.011	1
Chmp7	0.19138541	0.57412981	0.035	0.03	1
Commd7	0.19266798	0.55697211	0.115	0.11	1
Drosha	0.19293695	0.26891037	0.017	0.021	1
Ccdc84	0.19316697	0.71589243	0.025	0.021	1
Wdr48	0.19319004	0.45307526	0.099	0.093	1
Pcif1	0.19325434	0.33104554	0.231	0.234	1
Top3b	0.1936626	0.5463782	0.049	0.044	1
Trim8	0.19380523	0.63321287	0.075	0.069	1
Tada2b	0.19389578	0.62438075	0.029	0.025	1
Ccne2	0.19419692	0.89878576	0.013	0.01	1
Smarca4	0.19427714	0.48752098	0.16	0.158	1

4933434E20R	0.19472484	0.27197151	0.133	0.15	1
Ash2l	0.19486343	0.50358939	0.068	0.062	1
Dcaf5	0.19537114	0.37572723	0.052	0.046	1
Gdap2	0.19546379	0.28455122	0.038	0.045	1
Me2	0.19562614	0.51491166	0.099	0.093	1
Kctd9	0.1960405	0.48517812	0.03	0.026	1
Lsm1	0.19616496	0.53288963	0.084	0.078	1
Ptcd1	0.19642151	0.64202113	0.02	0.017	1
Prps1l3	0.19688339	0.63792063	0.026	0.021	1
Parl	0.19744755	0.29552007	0.094	0.108	1
Xndc1	0.19746384	0.52956021	0.022	0.018	1
2510002D24R	0.19767486	0.43534691	0.085	0.079	1
Setx	0.19797014	0.30647238	0.108	0.101	1
Aspscr1	0.19886268	0.64654831	0.055	0.05	1
Bloc1s1	0.19906594	0.38812764	0.034	0.04	1
Elk1	0.19947371	0.31904249	0.018	0.022	1
A230050P20R	0.20082463	0.76075784	0.02	0.016	1
Trp53bp2	0.2013047	0.35546163	0.041	0.048	1
Cwf19l2	0.2021134	0.37046433	0.125	0.119	1
Npat	0.20256436	0.47424297	0.059	0.053	1
Mvk	0.20296163	0.33112731	0.021	0.026	1
Txndc9	0.20318957	0.36318496	0.163	0.186	1
2810006K23R	0.20343902	0.71991052	0.012	0.009	1
Crnkl1	0.20420915	0.54119181	0.13	0.127	1
Ccdc181	0.20521007	0.84399574	0.011	0.008	1
Crlf2	0.20525236	0.43111104	0.226	0.234	1
Xlr4a	0.20531856	0.28631771	0.035	0.03	1
Pomc	0.20553525	0.30233729	0.009	0.012	1
Rnf141	0.20601291	0.26767756	0.038	0.045	1
Srpk1	0.20614904	0.29049897	0.192	0.187	1
Bend3	0.20617278	0.26283922	0.013	0.017	1
Phf2	0.20655149	0.42856686	0.076	0.07	1
Cers5	0.20704434	0.53559867	0.092	0.087	1
Hirip3	0.20865523	0.42661753	0.027	0.033	1
Atxn7l1	0.2087891	0.63116596	0.1	0.095	1
Trmt12	0.20954007	0.8586759	0.01	0.007	1
Sap18b	0.20985067	0.8280262	0.016	0.013	1
Mrps2	0.21080231	0.81968509	0.02	0.017	1
D17H6S53E	0.21092102	0.56449897	0.025	0.021	1
Letm2	0.21160198	0.40577166	0.054	0.048	1
Trabd	0.21184165	0.54443822	0.09	0.084	1
Akt2	0.2122821	0.50399742	0.135	0.131	1
BC003331	0.21248316	0.30957297	0.052	0.061	1
Zbtb49	0.2129702	1.05259489	0.016	0.013	1
Cant1	0.21312845	0.25546848	0.041	0.048	1
Pdpr	0.21409172	0.60169221	0.04	0.035	1
Tmem115	0.21415024	0.64787778	0.037	0.032	1
Atic	0.21432505	0.3821503	0.073	0.066	1

Cox20	0.21532735	0.36801644	0.155	0.152	1
Odf2l	0.21534025	0.38414158	0.012	0.016	1
Eya3	0.21548711	0.75956434	0.042	0.037	1
Tbc1d10a	0.21593883	0.26198131	0.101	0.114	1
Dcaf13	0.2159943	0.28301701	0.116	0.132	1
Scrib	0.21632323	0.64746119	0.039	0.034	1
Dcun1d3	0.2163559	0.53549211	0.026	0.022	1
Slmap	0.21682607	0.58303104	0.075	0.07	1
Ccpg1	0.21700981	0.43633957	0.064	0.058	1
Zbtb44	0.21814064	0.4809059	0.109	0.103	1
Krcc1	0.21840335	0.2855187	0.098	0.111	1
Ptdss1	0.2184539	0.65860423	0.059	0.054	1
Dhps	0.21851632	0.28857625	0.096	0.108	1
Prr14l	0.21877445	0.54263792	0.052	0.047	1
Ugp2	0.21918972	0.59742413	0.132	0.13	1
Rnf121	0.21998954	0.50269515	0.053	0.047	1
Edc4	0.22006359	0.59193306	0.073	0.067	1
Ptpn2	0.22054965	0.27226882	0.21	0.24	1
Zbtb7a	0.22068176	0.28255195	0.185	0.211	1
Mrpl46	0.22074852	0.33144669	0.067	0.076	1
Golga3	0.22078697	0.36784907	0.073	0.067	1
Exoc7	0.2208457	0.27195292	0.027	0.032	1
Zfp518a	0.22114031	0.53629949	0.054	0.049	1
Cisd1	0.22287576	0.51634566	0.088	0.082	1
Iars	0.22398397	0.54218978	0.091	0.086	1
Zfp182	0.22404104	0.36402088	0.026	0.022	1
Zfp568	0.22493451	0.29573123	0.009	0.012	1
Ints9	0.22533398	0.71353878	0.039	0.034	1
1810043G02R	0.22555644	0.43083096	0.015	0.019	1
Cstf2	0.22613853	0.42892618	0.057	0.052	1
Orai3	0.22624197	0.27864863	0.018	0.022	1
Zbtb21	0.22677811	0.62111121	0.109	0.105	1
Trak2	0.2274639	0.55216458	0.081	0.075	1
Ankrd17	0.22783301	0.3441475	0.233	0.236	1
Ostc	0.2281119	0.35063306	0.277	0.291	1
Ppp1r7	0.22814792	0.5889062	0.078	0.074	1
Fgd6	0.22850071	0.41815109	0.015	0.02	1
Zbtb17	0.23002851	0.37510068	0.042	0.049	1
Tut1	0.23070342	0.6770001	0.033	0.028	1
Pcgf3	0.23105038	0.56049176	0.038	0.034	1
Zfp287	0.23147757	0.44335856	0.013	0.01	1
Cdc42se1	0.23228326	0.27750289	0.175	0.199	1
Ncoa5	0.23238631	0.40819281	0.028	0.034	1
Vipas39	0.23239784	0.37631265	0.028	0.034	1
Ino80b	0.23243397	0.2553317	0.067	0.077	1
Agap2	0.23316419	0.51871834	0.023	0.02	1
Rpp21	0.23343289	0.32618136	0.136	0.154	1
Timm29	0.23390865	0.65126297	0.029	0.025	1

Itga2	0.23396021	0.25380879	0.013	0.01	1
Twf1	0.23442473	0.3439205	0.104	0.118	1
Tram2	0.23449015	0.27685939	0.017	0.021	1
Cenpt	0.23449248	0.84071646	0.013	0.01	1
Serf1	0.23537189	0.65627127	0.019	0.015	1
Rab11b	0.23551794	0.32912021	0.266	0.278	1
Mtmr12	0.23573353	0.47335233	0.093	0.088	1
Rab5b	0.2357542	0.54972476	0.087	0.082	1
Nmt2	0.23580629	0.40309431	0.117	0.112	1
Pigc	0.23609719	0.8257454	0.037	0.032	1
Fbxw8	0.23669259	0.43864709	0.025	0.03	1
Psmc1	0.23745633	0.50471531	0.122	0.118	1
Ptch1	0.23761503	0.53321151	0.031	0.027	1
Mob2	0.23801306	0.39945024	0.125	0.12	1
Ergic3	0.23826062	0.5226149	0.132	0.13	1
Wdr76	0.24298421	0.59223432	0.022	0.019	1
Lrrc28	0.24317124	0.66685371	0.021	0.018	1
Taf1	0.24330177	0.39013603	0.162	0.16	1
Ccdc186	0.24347376	0.51064112	0.099	0.094	1
Rbks	0.2439407	0.40681995	0.009	0.012	1
Krr1	0.24410891	0.47208468	0.079	0.073	1
Parp9	0.24426172	0.34333614	0.069	0.063	1
Cbx8	0.2449067	0.34319892	0.015	0.012	1
Spcs1	0.24587052	0.34802325	0.272	0.286	1
Tor4a	0.2463206	0.27200148	0.012	0.009	1
Uhrf1bp1l	0.24637545	0.34699955	0.162	0.159	1
Fut8	0.24680932	0.50433658	0.055	0.05	1
Vps33b	0.24818074	0.68293128	0.021	0.017	1
Fbxo46	0.24903625	0.5202226	0.021	0.017	1
Gas8	0.24926622	0.80002847	0.012	0.009	1
Cacfd1	0.25021904	0.35546775	0.031	0.037	1
Timmdc1	0.25125176	0.30841328	0.013	0.016	1
Vps39	0.25163694	0.7546566	0.04	0.036	1
Paxx	0.25164397	0.43839518	0.061	0.056	1
Hps6	0.25171042	1.05678708	0.018	0.015	1
Glb1	0.25253979	0.77638042	0.033	0.029	1
Rnaset2a	0.25254962	0.55141644	0.151	0.151	1
Usp39	0.25551891	0.31044445	0.053	0.061	1
Thap2	0.2556848	0.2780914	0.05	0.058	1
Dffb	0.25621641	0.52526066	0.02	0.017	1
Cyb5r4	0.25666503	0.37146139	0.128	0.145	1
Ttll5	0.25809659	0.59541945	0.021	0.017	1
Olfr60	0.25845162	0.56172827	0.023	0.02	1
Ctla2b	0.25867533	1.15255417	0.013	0.011	1
Rarg	0.25940559	0.5424862	0.009	0.012	1
Ppm1d	0.2601928	0.50872269	0.059	0.054	1
Gmppb	0.26068317	0.49911485	0.037	0.044	1
Sbf1	0.26086162	0.48640505	0.11	0.106	1

Zfp788	0.26090593	0.45936789	0.022	0.018	1
Pde6d	0.26104467	0.60656139	0.019	0.016	1
Map3k7	0.26178173	0.26787058	0.072	0.081	1
Add1	0.26193308	0.39608269	0.122	0.117	1
Tsfm	0.26278471	0.48942778	0.022	0.027	1
Zfp715	0.26321911	0.73233994	0.029	0.025	1
Nckipsd	0.26328202	0.29639914	0.01	0.013	1
Galc	0.26342233	0.30329247	0.036	0.042	1
Selenoi	0.26349499	0.61926272	0.07	0.065	1
Wdr47	0.26385688	0.67357428	0.025	0.021	1
Ube2j1	0.26390922	0.36274781	0.239	0.247	1
Mtf1	0.2639382	0.33554491	0.051	0.058	1
Fbxo6	0.26416506	0.39308802	0.046	0.054	1
Marveld1	0.26490213	0.40536328	0.018	0.022	1
Gpatch11	0.26552247	0.38970003	0.062	0.057	1
Acat2	0.26557764	0.50298083	0.022	0.019	1
Hdac2	0.2656734	0.27286788	0.057	0.065	1
Nop9	0.26588668	0.55818023	0.074	0.069	1
Gnl1	0.26599174	0.29223543	0.088	0.099	1
Spr	0.266437	0.64034254	0.049	0.045	1
Il3ra	0.26654795	0.53900318	0.044	0.039	1
Supt6	0.26655797	0.27237104	0.18	0.203	1
Med19	0.26669011	0.28821213	0.052	0.059	1
Rnf220	0.26710255	0.38426231	0.13	0.127	1
Dalrd3	0.26731216	0.30607122	0.057	0.064	1
Sec24d	0.26732752	0.30179591	0.045	0.052	1
Amfr	0.2673763	0.50427765	0.142	0.14	1
Ints8	0.26741843	0.37357124	0.042	0.037	1
Aptx	0.26752113	0.25476448	0.028	0.033	1
BC003965	0.2681816	0.2774871	0.05	0.057	1
Sucla2	0.26850368	0.28167041	0.07	0.079	1
Tradd	0.2685899	0.51224589	0.035	0.03	1
Sfn	0.26867211	0.28162657	0.029	0.025	1
Pigyl	0.26928163	0.40338414	0.044	0.051	1
Pi4kb	0.26931598	0.54481239	0.028	0.024	1
Ppa1	0.26936192	0.27024068	0.069	0.078	1
Zfp511	0.26958593	0.31579559	0.07	0.079	1
Inip	0.27028799	0.31990722	0.062	0.07	1
Mrps30	0.27041303	0.26923767	0.07	0.08	1
Trappc12	0.27162827	0.63521809	0.054	0.049	1
Ctps2	0.27277775	0.351503	0.049	0.044	1
Herc4	0.27278113	0.28771439	0.148	0.143	1
Ufl1	0.27285623	0.56649086	0.077	0.073	1
Rbak	0.27348813	0.6628107	0.013	0.011	1
Plekha8	0.27350085	0.8022997	0.012	0.009	1
Nmi	0.27356424	0.41935545	0.126	0.122	1
Uri1	0.27373019	0.4046955	0.09	0.084	1
Icmt	0.27391744	0.54353873	0.036	0.032	1

Pcmtd1	0.27404725	0.30038436	0.203	0.202	1
Aifm1	0.27411975	0.66639273	0.042	0.037	1
Cspp1	0.27441837	0.4015613	0.108	0.104	1
Borcs5	0.27554473	0.50202074	0.023	0.02	1
Dlg1	0.27561858	0.25010393	0.083	0.093	1
Scfd1	0.27593554	0.3450737	0.124	0.12	1
Txnrd2	0.27613131	0.39142434	0.032	0.037	1
Slc35a3	0.27638986	0.57643685	0.075	0.07	1
Alg2	0.27665201	0.71657631	0.02	0.017	1
Dvl3	0.27671364	0.63130918	0.044	0.04	1
Polr2d	0.27691083	0.2879308	0.046	0.053	1
Dhcr24	0.27822077	0.34682131	0.022	0.027	1
Pck2	0.27863816	0.29128127	0.031	0.037	1
Prelid3b	0.27878307	0.30720759	0.112	0.125	1
Rasgrp4	0.27890617	0.36304556	0.045	0.052	1
Vrk3	0.27982621	0.25478247	0.065	0.073	1
Fam13b	0.28034986	0.59169042	0.072	0.067	1
Aspn	0.28045919	0.63157368	0.014	0.011	1
Polr3k	0.28083575	0.27409223	0.058	0.066	1
Sec31a	0.28107955	0.32830984	0.137	0.154	1
Hectd3	0.28125749	0.56236419	0.026	0.023	1
Hcls1	0.28219811	0.28990045	0.324	0.346	1
Pdpk1	0.28270123	0.3122197	0.113	0.127	1
Phtf1os	0.28330394	0.69004555	0.01	0.007	1
Ranbp3	0.28350598	0.27237883	0.045	0.052	1
Cdkn2aipnl	0.28363666	0.4127428	0.055	0.051	1
Hipk3	0.28373058	0.29132744	0.094	0.105	1
Arfip1	0.28386299	0.56646543	0.044	0.039	1
Anapc10	0.28474183	0.29252898	0.038	0.044	1
Mettl13	0.2848026	0.59624048	0.023	0.02	1
Mkln1	0.28567971	0.33674618	0.233	0.239	1
Prpf40a	0.28581899	0.35125577	0.314	0.337	1
Pramef8	0.28586199	0.55059787	0.039	0.035	1
Cdca4	0.2859345	0.33713988	0.05	0.057	1
Atg5	0.28639446	0.37126325	0.08	0.089	1
Desi2	0.28702187	0.32093337	0.099	0.11	1
Vrk1	0.28775498	0.5249484	0.114	0.111	1
Vsir	0.28811784	0.42249518	0.145	0.142	1
Gtf2h1	0.28812184	0.31896673	0.082	0.092	1
Gpank1	0.28838253	0.49566893	0.071	0.066	1
Harbi1	0.28847444	0.63925069	0.015	0.013	1
Csnk1d	0.28850146	0.37815536	0.238	0.248	1
Rad52	0.28851834	0.39135408	0.021	0.018	1
Slc25a13	0.28898189	0.3500129	0.009	0.012	1
Pip4k2c	0.28925685	0.52171483	0.076	0.072	1
Stk16	0.29033067	0.27196002	0.054	0.062	1
Leng9	0.29033282	0.63425559	0.035	0.031	1
0610010K14R	0.2903556	0.53629648	0.053	0.049	1

Rbm10	0.2907207	0.31043666	0.045	0.052	1
Arpc1a	0.29074523	0.36380642	0.118	0.133	1
Gm33104	0.29084729	0.57847565	0.051	0.047	1
Ebna1bp2	0.2915779	0.28928146	0.124	0.138	1
Gak	0.29163218	0.33145959	0.145	0.163	1
Impad1	0.29246836	0.59498203	0.101	0.098	1
Nup160	0.29264455	0.50920067	0.041	0.036	1
Acot8	0.29427564	0.3363292	0.036	0.041	1
Edf1	0.29439466	0.29070755	0.369	0.405	1
Pigo	0.29459724	0.5623225	0.022	0.019	1
Tpm1	0.29492937	0.53079872	0.094	0.09	1
Tmco4	0.29496531	0.27021757	0.012	0.015	1
Adcy3	0.29511124	0.6158584	0.017	0.014	1
Smarcc1	0.29573262	0.43995311	0.105	0.1	1
Pigk	0.29659186	0.26808331	0.054	0.061	1
Mfsd5	0.29805909	0.39180603	0.052	0.059	1
Hcfc2	0.29869341	0.5059627	0.035	0.031	1
Pitpnm2	0.2989354	0.51280273	0.023	0.02	1
Prpf4	0.29895772	0.43668995	0.097	0.092	1
Tcf20	0.29902233	0.25902365	0.116	0.129	1
Ivns1abp	0.29922776	0.29425121	0.257	0.266	1
Mex3c	0.30024349	0.47441786	0.108	0.104	1
Atp2a2	0.30067002	0.26981082	0.224	0.254	1
Ccdc127	0.30079183	0.46079105	0.094	0.09	1
Ankrd52	0.30086696	0.6524996	0.035	0.031	1
Gbe1	0.30107369	0.53397172	0.03	0.026	1
Csnk2a2	0.30110326	0.25674594	0.219	0.247	1
Faap20	0.30159713	0.43487491	0.051	0.046	1
Taf5	0.30240263	0.52792674	0.042	0.038	1
Clock	0.30296779	0.30317023	0.083	0.092	1
Zfp65	0.30305703	0.692605	0.027	0.023	1
Phrf1	0.30306917	0.52566647	0.113	0.109	1
Kif13b	0.30362516	0.52511634	0.057	0.053	1
Dph7	0.30421575	0.60857689	0.015	0.012	1
Mbd5	0.30436506	0.28609353	0.027	0.032	1
Slc1a5	0.30470121	0.41824391	0.13	0.127	1
Ints1	0.30484147	0.62688202	0.05	0.046	1
Zfp598	0.30513632	0.25068327	0.041	0.046	1
Ighmbp2	0.30520312	0.47562939	0.021	0.018	1
Acot7	0.30539974	0.37021688	0.125	0.122	1
Armcx5	0.30570494	0.28897288	0.051	0.058	1
Ehbp1l1	0.30619769	0.60400441	0.083	0.079	1
Pld3	0.30651046	0.34061997	0.15	0.148	1
Cradd	0.3075143	0.51973423	0.02	0.017	1
Ctdsp2	0.30761381	0.27476261	0.133	0.149	1
Deaf1	0.30783999	0.38640018	0.012	0.015	1
Mfsd14b	0.3082927	0.55403265	0.108	0.105	1
Socs1	0.30928018	0.33116292	0.12	0.116	1

Dennd1a	0.30947331	0.27203097	0.064	0.072	1
Inpp5k	0.30980227	0.43796968	0.065	0.061	1
Lrrc8c	0.31008464	0.37238058	0.102	0.115	1
Leo1	0.31057145	0.45945997	0.06	0.055	1
Ints4	0.3106536	0.4571855	0.026	0.022	1
Eef1akmt1	0.31096104	0.50788467	0.034	0.03	1
Guk1	0.31131405	0.32041338	0.091	0.101	1
Ino80c	0.31140085	0.53604666	0.062	0.058	1
Smim8	0.3117806	0.38369549	0.059	0.054	1
Hist1h2br	0.31203023	0.66483943	0.01	0.008	1
Mppe1	0.31208282	0.55339707	0.015	0.013	1
L3mbtl2	0.31228732	0.31487977	0.014	0.018	1
Fam58b	0.31266065	0.47258708	0.035	0.031	1
Slc39a3	0.3127262	0.54830354	0.029	0.025	1
Slc36a4	0.31291896	0.76307542	0.046	0.043	1
Hjurp	0.31336611	0.42552649	0.089	0.085	1
Svil	0.31355213	0.26299628	0.09	0.101	1
Gm26670	0.31380542	0.63668572	0.012	0.01	1
Crtc1	0.31436995	0.7080272	0.011	0.009	1
Gnai3	0.31504037	0.32057335	0.259	0.269	1
Aftph	0.31621633	0.38251193	0.199	0.203	1
Vps16	0.3166264	0.63111476	0.064	0.059	1
Dr1	0.31718576	0.55392721	0.086	0.082	1
Mmgt1	0.31787798	0.66760777	0.035	0.031	1
Ppp6c	0.31798589	0.29629882	0.133	0.149	1
Cep57	0.318362	0.35762855	0.1	0.096	1
Slc25a46	0.3190346	0.47732485	0.056	0.052	1
Sap18	0.31963545	0.28444096	0.193	0.215	1
Kif16b	0.31969285	0.36501153	0.03	0.035	1
Psmd9	0.32033568	0.52963229	0.095	0.091	1
Ppp1r18	0.32056612	0.33472581	0.194	0.196	1
Yipf6	0.3205957	0.26101503	0.058	0.065	1
Mrpl18	0.32093317	0.39636475	0.167	0.167	1
Tceanc	0.32162699	0.5968349	0.012	0.009	1
Arhgef18	0.32182858	0.26782347	0.043	0.039	1
Trnau1ap	0.32189429	0.4062191	0.076	0.085	1
Mapk1ip1	0.32283456	0.45265578	0.024	0.021	1
Cyp20a1	0.32306405	0.81175376	0.03	0.027	1
Trp53bp1	0.32337932	0.37078995	0.054	0.05	1
Gm44777	0.3250226	0.60474478	0.013	0.011	1
Pigt	0.3251253	0.29684618	0.083	0.093	1
Acbd3	0.32515745	0.34013012	0.263	0.278	1
Cep70	0.32605516	0.61241832	0.015	0.013	1
Sh3glb2	0.32649484	0.47438251	0.046	0.042	1
Ubac1	0.32666395	0.25023281	0.03	0.035	1
Dcbld2	0.32712048	0.7254946	0.01	0.008	1
Xrcc6	0.32712226	0.65944611	0.015	0.013	1
Nt5c3b	0.32750467	0.39120451	0.038	0.034	1

Rmi2	0.32910622	0.42240441	0.009	0.011	1
Ubl4a	0.3293164	0.63298442	0.086	0.083	1
Pcnx3	0.32974465	0.54619378	0.037	0.033	1
Lyar	0.32994753	0.50261886	0.145	0.144	1
Fam35a	0.33006448	0.29362791	0.019	0.016	1
Cops7a	0.3306884	0.27135871	0.036	0.041	1
1110004E09R	0.33100962	0.44277989	0.036	0.032	1
Gatad1	0.33137749	0.46610598	0.118	0.115	1
Pop4	0.33141515	0.29603024	0.057	0.064	1
Cmtm6	0.33147555	0.29745164	0.132	0.147	1
Mnat1	0.33184895	0.47178353	0.08	0.076	1
Mrpl4	0.33293665	0.2857354	0.099	0.11	1
Scyl3	0.3331327	0.52009979	0.067	0.063	1
Atg4a	0.33387816	0.6784497	0.018	0.015	1
AI837181	0.33400985	0.49985969	0.076	0.072	1
Tmem127	0.33417298	0.3010888	0.074	0.082	1
Kdm3b	0.33447915	0.35634704	0.12	0.117	1
Rnmt	0.33547646	0.30848905	0.106	0.117	1
Zc3h3	0.33561282	0.42176263	0.029	0.025	1
Rhno1	0.33571784	0.3457945	0.051	0.047	1
Tjap1	0.33582666	0.64635869	0.053	0.05	1
H13	0.33614864	0.32396283	0.224	0.23	1
Dync1li2	0.33683675	0.45436	0.044	0.05	1
Tax1bp3	0.33688693	0.45495023	0.032	0.037	1
Pex14	0.33690598	0.32884435	0.024	0.028	1
Zfp956	0.33707007	0.37086833	0.014	0.012	1
Srp68	0.33758064	0.32652202	0.073	0.081	1
Chtf8	0.33769992	0.34785293	0.077	0.086	1
Prickle1	0.33891412	0.63795995	0.018	0.015	1
Eif2b4	0.33944903	0.41022604	0.047	0.043	1
Afg3l1	0.33955423	0.49877965	0.096	0.092	1
D2hgdh	0.33966951	0.57589841	0.013	0.011	1
Hnrnpul2	0.33973657	0.35472317	0.273	0.286	1
Rc3h1	0.34032157	0.38091952	0.216	0.222	1
4632415L05Ri	0.34049106	0.44220631	0.015	0.012	1
Zfp141	0.34062175	0.55072049	0.016	0.013	1
Slc36a3os	0.34206818	0.71021725	0.036	0.032	1
Enpp1	0.34282709	0.51737628	0.034	0.03	1
Gpatch1	0.34287356	0.58717654	0.017	0.014	1
Scoc	0.34292938	0.55027851	0.045	0.042	1
Rnf7	0.34294562	0.38091352	0.205	0.212	1
Med26	0.34348709	0.26510171	0.022	0.026	1
Ubr4	0.34363994	0.28647751	0.142	0.158	1
Usp40	0.34453894	0.61621873	0.022	0.026	1
Lyrm1	0.34587351	0.77146475	0.018	0.016	1
Cdc23	0.34600406	0.37440049	0.031	0.028	1
Kmt5c	0.34633088	0.38661442	0.016	0.019	1
Spata24	0.3468236	0.30635506	0.014	0.017	1

Cebpg	0.34694585	0.39680401	0.182	0.184	1
Mtrr	0.34775131	0.47772987	0.029	0.026	1
S1pr2	0.34790002	0.54020591	0.019	0.016	1
Zswim1	0.34830398	0.64628855	0.015	0.013	1
Usp15	0.34885882	0.3179914	0.23	0.236	1
Atg16l1	0.34943706	0.43161363	0.074	0.07	1
Mettl26	0.35005864	0.57362371	0.065	0.061	1
Tmem185a	0.35018335	0.74825642	0.02	0.018	1
Ecd	0.35029471	0.42577036	0.066	0.062	1
Rcl1	0.35099393	0.25196444	0.057	0.063	1
Ciapin1	0.35100764	0.55841514	0.097	0.094	1
Tbc1d12	0.35136328	0.33931181	0.014	0.012	1
Smarcc2	0.3520647	0.49188702	0.082	0.078	1
Relb	0.3551643	0.33695339	0.273	0.289	1
Zfyve1	0.35527694	0.6467694	0.031	0.028	1
Mcm4	0.35549939	0.44176567	0.101	0.098	1
3110002H16R	0.35551599	0.26554231	0.026	0.03	1
Fbn1	0.35721751	0.44001158	0.013	0.011	1
Slc39a9	0.35765568	0.40398402	0.038	0.034	1
Prps1	0.35882247	0.29476238	0.077	0.086	1
Med22	0.35955112	0.31451442	0.015	0.018	1
Lamtor5	0.35968275	0.48967321	0.17	0.175	1
Ndufaf8	0.35975394	0.51168131	0.186	0.191	1
Plpp5	0.36036872	0.5597513	0.03	0.034	1
Tnc	0.36085128	0.66926492	0.013	0.011	1
Ap3m1	0.36130239	0.56704962	0.056	0.052	1
Sympk	0.36376859	0.31396639	0.055	0.061	1
Abcf1	0.36393038	0.32613439	0.241	0.251	1
Eipr1	0.36439015	0.48267868	0.076	0.072	1
Rbmx2	0.36478766	0.61116283	0.054	0.051	1
Atp5s	0.36512889	0.44782475	0.012	0.01	1
Stag1	0.36521033	0.2614183	0.159	0.176	1
Mis12	0.36540163	0.52137543	0.053	0.06	1
Xpo7	0.36550651	0.52469488	0.069	0.065	1
Osbp	0.3659227	0.25530476	0.079	0.087	1
Dctpp1	0.36678201	0.6177593	0.065	0.062	1
Miip	0.36691566	0.57523048	0.03	0.027	1
Naif1	0.36693427	0.93902409	0.012	0.01	1
Alms1	0.3675709	0.56871714	0.009	0.011	1
Gm26778	0.36803932	0.60835473	0.11	0.11	1
Ripor1	0.36824858	0.72515351	0.017	0.015	1
Ldhb	0.36844125	0.99512669	0.017	0.015	1
Acads	0.36869698	0.49989255	0.048	0.044	1
Zfp950	0.36965421	0.30019404	0.046	0.043	1
Polg2	0.36975541	0.26059901	0.077	0.073	1
Mcmbp	0.37060601	0.29178417	0.088	0.097	1
Haus4	0.37129021	0.79259648	0.026	0.023	1
Pofut2	0.37138031	0.35344582	0.172	0.194	1

Rxra	0.37188548	0.26901572	0.015	0.018	1
Zfp619	0.3723651	0.5936006	0.01	0.008	1
Prpf3	0.37258782	0.46161557	0.05	0.046	1
Taf4	0.3730225	0.32866934	0.036	0.041	1
Gm11423	0.37361828	0.65038096	0.011	0.009	1
Plpp2	0.37419784	0.56913788	0.009	0.011	1
Rida	0.3742914	0.39671732	0.019	0.022	1
Manbal	0.37448713	0.46234371	0.064	0.072	1
Glmp	0.37508603	0.41659043	0.136	0.135	1
Bud23	0.37536051	0.4464533	0.047	0.053	1
Narfl	0.37543981	0.34324076	0.021	0.025	1
Tyk2	0.37561278	0.2860326	0.058	0.054	1
Rpp30	0.37577817	0.44779435	0.031	0.036	1
Mtg2	0.37601203	0.63079979	0.021	0.018	1
Snrnp27	0.37693609	0.35696259	0.193	0.196	1
March2	0.37708998	0.39118119	0.098	0.094	1
Rnf187	0.37731473	0.42445368	0.14	0.139	1
Tnfrsf14	0.3774486	0.7711493	0.017	0.015	1
Gpn2	0.37778155	0.69098325	0.022	0.019	1
Crtap	0.37817191	0.51631081	0.014	0.012	1
Bckdha	0.37843719	0.40072704	0.051	0.057	1
Adsl	0.37845426	0.60776366	0.043	0.039	1
Arfrp1	0.37894081	0.47642374	0.121	0.119	1
Shoc2	0.37921637	0.43449407	0.131	0.13	1
Tdrd3	0.37978315	0.53305408	0.024	0.021	1
Tchp	0.37986241	0.66808605	0.018	0.016	1
Brd3	0.37998649	0.376924	0.091	0.087	1
Dhx16	0.38103124	0.49133065	0.079	0.076	1
Tmem135	0.38116456	0.47243453	0.066	0.063	1
Zfp157	0.3816472	0.55773694	0.025	0.022	1
Maea	0.3820171	0.29437977	0.098	0.108	1
Haus7	0.3824377	0.40129211	0.019	0.022	1
Cpsf1	0.38280173	0.49634297	0.046	0.042	1
Hsdl1	0.38372188	0.7713985	0.047	0.044	1
Zfp595	0.38375328	0.63311459	0.02	0.017	1
Tor1b	0.38395318	0.36158878	0.075	0.083	1
Tctn3	0.38537839	0.5121117	0.008	0.01	1
Oard1	0.38618409	0.31737595	0.062	0.069	1
Depdc5	0.38638911	0.25562131	0.025	0.028	1
Thumpd1	0.38687081	0.39074995	0.08	0.088	1
Fbxl17	0.38712837	0.54183569	0.041	0.038	1
Degs1	0.38775571	0.29864587	0.195	0.218	1
Nsmce3	0.38797654	0.30655996	0.068	0.075	1
Rnf146	0.38813657	0.27491262	0.118	0.13	1
Cfap20	0.38898429	0.46554685	0.081	0.077	1
Ddx54	0.39036771	0.26730446	0.207	0.231	1
Kdm2a	0.39084402	0.26308126	0.196	0.199	1
Tbc1d13	0.39088424	0.74481704	0.028	0.025	1

Tgtp2	0.3910253	0.47241413	0.018	0.016	1
Dda1	0.39118764	0.29111461	0.144	0.159	1
Prmt9	0.39226731	0.63028507	0.023	0.02	1
Snx2	0.39265589	0.27414123	0.168	0.167	1
Rwdd2b	0.39440445	0.86302793	0.013	0.011	1
Klhdc3	0.39636426	0.28508517	0.062	0.069	1
Msh3	0.39874621	0.60370246	0.041	0.038	1
Scaf8	0.39884881	0.38632941	0.13	0.129	1
2610044O15R	0.40004	0.68526243	0.015	0.013	1
Zfp948	0.40033253	0.7500771	0.01	0.008	1
Nemf	0.40131526	0.2747682	0.136	0.149	1
Slc41a3	0.40216216	1.33935507	0.018	0.016	1
Rab18	0.40229321	0.39963681	0.148	0.149	1
Naa60	0.40261696	0.53915783	0.049	0.046	1
Uxs1	0.40274524	0.38366592	0.027	0.031	1
Ascc2	0.40279785	0.29982508	0.043	0.04	1
Lmbrd1	0.4036245	0.41783413	0.161	0.163	1
Alg6	0.40367583	0.60825191	0.008	0.01	1
Limd1	0.40391186	0.47676661	0.167	0.171	1
Zfp276	0.40443107	0.71078689	0.015	0.013	1
Yars2	0.40536765	0.36182075	0.013	0.016	1
Gm30025	0.40674168	0.59798641	0.014	0.012	1
Zfyve19	0.40679782	0.39174542	0.013	0.015	1
Trappc8	0.40722319	0.43257955	0.089	0.086	1
Ift43	0.40749631	1.24808995	0.011	0.009	1
Alkbh6	0.4086076	0.54557377	0.063	0.06	1
4930453N24R	0.40872114	0.41971164	0.099	0.096	1
Dync1h1	0.40955793	0.33347179	0.2	0.223	1
St3gal2	0.40973844	0.65538954	0.017	0.014	1
Ackr3	0.4099294	0.4621238	0.011	0.009	1
Cyhr1	0.41008757	0.41124553	0.076	0.073	1
Chpf2	0.41030219	0.53576092	0.025	0.022	1
Synj2	0.41106927	0.32150959	0.034	0.038	1
Pspc1	0.41159868	0.25199065	0.112	0.122	1
Fdxr	0.412061	0.56854526	0.029	0.027	1
Tyms	0.4121607	0.56465988	0.023	0.021	1
Fyco1	0.41216674	0.25191092	0.052	0.057	1
Gsk3b	0.4125644	0.32128128	0.244	0.251	1
Prep	0.41344156	0.29999268	0.033	0.037	1
Safb	0.41391236	0.35949414	0.195	0.199	1
Mbip	0.41431721	0.66753538	0.042	0.039	1
Lemd3	0.41443108	0.48275154	0.105	0.103	1
Wdr45	0.41521674	0.50525246	0.044	0.041	1
Diablo	0.41606252	0.65609992	0.07	0.067	1
Ap5z1	0.41649225	0.27763339	0.008	0.01	1
Reep4	0.41761719	0.3334184	0.039	0.044	1
Fam98a	0.41762872	0.42661876	0.031	0.029	1
Cars2	0.41824989	0.62224766	0.044	0.041	1

Cnot11	0.42002122	0.50017744	0.062	0.059	1
Tacc2	0.42153941	0.65882139	0.008	0.01	1
Tmem160	0.42173643	0.30777262	0.29	0.308	1
Spata2	0.42219697	0.52741273	0.077	0.075	1
Pigs	0.42330655	0.38008798	0.049	0.054	1
Bahd1	0.42371728	0.38244779	0.039	0.043	1
Ercc8	0.42404372	0.30467776	0.019	0.022	1
Agpat2	0.42406735	0.26634767	0.012	0.015	1
Abhd10	0.42430377	0.36304311	0.045	0.042	1
Ep400	0.42461223	0.3249554	0.2	0.204	1
Dmtf1	0.4247213	0.40989915	0.125	0.124	1
Ndor1	0.42522729	0.87112339	0.011	0.009	1
Exosc9	0.42537629	0.41675928	0.043	0.048	1
C330007P06R	0.42593306	0.25000999	0.073	0.08	1
Xpo1	0.4265941	0.50789151	0.106	0.104	1
Ndufaf3	0.42684101	0.3672096	0.045	0.05	1
9930022D16R	0.42689176	0.44287332	0.018	0.02	1
Mut	0.42714997	0.62091862	0.028	0.025	1
Ttc38	0.42743796	0.85244537	0.013	0.011	1
Cul4b	0.4295648	0.52414384	0.052	0.049	1
Frg1	0.43045923	0.29098962	0.227	0.236	1
Sel1l	0.43049378	0.4259755	0.14	0.156	1
Setd1a	0.43147887	0.36714421	0.059	0.055	1
Mcm3	0.43208759	0.3074987	0.064	0.07	1
Szt2	0.4327017	0.60803822	0.015	0.013	1
Uqcrc2	0.4328561	0.36415169	0.203	0.21	1
Gnl3l	0.43305817	0.26052645	0.068	0.074	1
Hook3	0.43308089	0.2742995	0.133	0.146	1
Gatd1	0.43329183	0.2748368	0.048	0.053	1
Btbd10	0.43373974	0.57597207	0.049	0.046	1
Ahctf1	0.43441896	0.36628285	0.168	0.17	1
Rfx3	0.43466136	0.50549855	0.026	0.023	1
Synrg	0.43643731	0.27017795	0.096	0.105	1
Prdx4	0.43709478	0.60664303	0.063	0.06	1
Irf4	0.43722782	0.26660227	0.145	0.156	1
2410002F23Ri	0.43754325	0.45767039	0.073	0.07	1
4930522L14Ri	0.43793068	0.50171449	0.021	0.018	1
Lypla2	0.43857695	0.37852918	0.122	0.12	1
1600014C10R	0.43957622	0.50690819	0.039	0.037	1
Platr17	0.43973659	0.31771668	0.01	0.012	1
Mettl4	0.43993871	0.64574737	0.013	0.011	1
Nudt6	0.4400031	0.40294794	0.011	0.009	1
Akap11	0.44039276	0.30453232	0.066	0.072	1
Ptpn11	0.44198952	0.30824729	0.128	0.14	1
Kctd20	0.44309036	0.26716698	0.057	0.062	1
Abcb7	0.44324512	0.28776745	0.045	0.05	1
Car5b	0.44343249	0.9391491	0.013	0.011	1
Casp2	0.4436752	0.42169276	0.047	0.044	1

Tcea1	0.44405111	0.28543079	0.306	0.327	1
Heatr6	0.44465837	0.71308962	0.047	0.044	1
Trappc6b	0.44488631	0.31362903	0.173	0.192	1
Mier3	0.44584807	0.45759184	0.045	0.042	1
Tmx2	0.44632351	0.28840505	0.027	0.03	1
Smim13	0.44642232	0.37891211	0.028	0.031	1
Stub1	0.44653455	0.40342699	0.097	0.106	1
Gpr108	0.44692823	0.38230232	0.058	0.064	1
Rab4b	0.44749524	0.82281916	0.014	0.012	1
Ercc4	0.44933815	0.66138732	0.031	0.028	1
Tinf2	0.4501209	0.36293346	0.085	0.083	1
Smim1	0.45062352	0.40364092	0.014	0.012	1
Dyrk3	0.4508311	0.4417696	0.013	0.011	1
Rela	0.45091538	0.30080539	0.243	0.273	1
Emc4	0.45109725	0.39732502	0.084	0.092	1
Cdc73	0.4514903	0.35692922	0.162	0.163	1
Psmd7	0.4520883	0.30427066	0.169	0.188	1
Asl	0.4521256	0.55734063	0.05	0.055	1
Emc7	0.45306918	0.26582181	0.247	0.28	1
Aup1	0.4536341	0.37014902	0.225	0.237	1
Tgs1	0.45380947	0.5367195	0.117	0.116	1
2510039O18R	0.45556073	0.42257964	0.088	0.085	1
R3hdm4	0.45568963	0.41784561	0.142	0.143	1
2300009A05R	0.45652378	0.62190869	0.075	0.073	1
Cox16	0.45758717	0.44094013	0.077	0.074	1
Rnf170	0.45782554	0.50369789	0.029	0.027	1
Hist2h2bb	0.45789408	0.27632571	0.021	0.024	1
Adrb2	0.45838285	0.390511	0.05	0.047	1
Actr1a	0.45853641	0.52423078	0.096	0.095	1
Mtmr2	0.45886235	0.51859942	0.06	0.057	1
Hmg20a	0.45904881	0.45404052	0.044	0.041	1
Vps37a	0.45914282	0.25530683	0.108	0.118	1
Rbl1	0.45923322	0.39284113	0.035	0.032	1
Rps6kc1	0.45946857	0.94407058	0.013	0.011	1
Uba7	0.45977354	0.30888862	0.044	0.048	1
Bag1	0.45994007	0.26682137	0.274	0.289	1
Stag2	0.45999877	0.27068963	0.24	0.249	1
Ebp	0.46025225	0.52989792	0.116	0.117	1
Dopey1	0.46040535	0.41019529	0.078	0.075	1
Wdr55	0.46093423	0.4057608	0.019	0.022	1
Cbfa2t2	0.46116491	0.68841301	0.027	0.024	1
Anapc2	0.46123616	0.69353139	0.059	0.057	1
Pibf1	0.46180275	0.34268069	0.058	0.055	1
AI597479	0.46261114	0.59009895	0.02	0.018	1
Ccnh	0.46273067	0.28698882	0.042	0.046	1
Igfbp3	0.46276863	0.31488786	0.013	0.016	1
Nid1	0.46320435	0.43325483	0.01	0.008	1
Arhgap1	0.46335516	0.54662042	0.073	0.071	1

Parg	0.46362994	0.4821032	0.094	0.093	1
Atad5	0.46363748	0.77843459	0.031	0.029	1
Dexi	0.46364067	0.25899826	0.028	0.031	1
Casp7	0.46396272	0.87130766	0.022	0.02	1
Mtx2	0.46467821	0.61628604	0.042	0.039	1
Mospd1	0.4651135	0.55774084	0.022	0.02	1
Fbxo22	0.46533656	0.46047225	0.051	0.048	1
Zfp84	0.46677658	0.51695291	0.053	0.051	1
Dis3l2	0.46775678	0.53901704	0.045	0.043	1
Mapre2	0.46834817	0.4211194	0.136	0.136	1
Epg5	0.46899201	0.81236367	0.029	0.027	1
Dnajc10	0.46936992	0.40922694	0.074	0.081	1
Lonp2	0.46958009	0.35177614	0.073	0.07	1
Nrbf2	0.46958733	0.27248375	0.099	0.108	1
Phpt1	0.46969401	0.40646065	0.037	0.035	1
Hace1	0.47047787	0.53992525	0.038	0.035	1
Nrf1	0.47146338	0.34478848	0.084	0.081	1
Aacs	0.47151627	0.91806029	0.017	0.015	1
Pex3	0.47154928	0.45155747	0.052	0.049	1
Kctd13	0.47178372	0.45407964	0.019	0.022	1
Tyw5	0.47273849	0.6034543	0.021	0.019	1
Acvr2a	0.47296786	0.32554801	0.048	0.053	1
Cdan1	0.47376938	0.38347231	0.022	0.02	1
Wdr7	0.4742452	0.31062817	0.021	0.024	1
Ube2q1	0.47434467	0.41851111	0.145	0.146	1
Csnk1g1	0.47476659	0.29155502	0.085	0.082	1
Srd5a3	0.47498356	0.59012983	0.048	0.045	1
Phf11c	0.47506742	0.39740225	0.01	0.008	1
Cox18	0.47525534	0.4902532	0.034	0.031	1
Proser1	0.47547485	0.28301712	0.082	0.09	1
Actr1b	0.47571994	0.36187661	0.077	0.085	1
Tbc1d25	0.47623921	0.67334374	0.011	0.009	1
Cramp1l	0.47730758	0.28925167	0.05	0.055	1
Ddx41	0.47765328	0.34762208	0.101	0.099	1
Derl2	0.47779815	0.35706337	0.138	0.138	1
Gtf3c5	0.47833754	0.3723351	0.021	0.024	1
3110043O21R	0.4797737	0.53980745	0.12	0.12	1
Rad9a	0.48026978	0.69633425	0.017	0.015	1
Crcp	0.48089987	0.60660947	0.046	0.043	1
Kremen1	0.48232706	0.37554968	0.02	0.023	1
AW011738	0.48233673	0.48668052	0.078	0.076	1
Zfp747	0.48265999	0.63078371	0.015	0.014	1
Bnip1	0.48275109	0.29138691	0.028	0.031	1
Nme7	0.4827764	0.68819219	0.015	0.014	1
Actl6a	0.48468632	0.2842129	0.101	0.109	1
Rmnd5a	0.48506896	0.32299174	0.129	0.128	1
Brf2	0.48538044	0.50417684	0.019	0.017	1
Slc25a25	0.48602601	0.37803735	0.057	0.054	1

Fyttd1	0.48612449	0.54695345	0.13	0.132	1
Trmt10c	0.48662283	0.30333664	0.047	0.052	1
Gpsm2	0.48672472	0.6092504	0.008	0.01	1
Zfp639	0.48679738	0.62013748	0.086	0.085	1
Mcm9	0.48685087	0.4651012	0.042	0.039	1
Pdzd8	0.4875009	0.43409565	0.098	0.096	1
Arhgef7	0.4883887	0.34927432	0.038	0.042	1
Tsn	0.48860636	0.33496358	0.234	0.245	1
Rae1	0.48953645	0.41764619	0.075	0.072	1
Sae1	0.48964965	0.51572285	0.069	0.066	1
Clptm1l	0.49084729	0.38637452	0.124	0.136	1
Malsu1	0.49157967	0.28539187	0.109	0.118	1
Nans	0.49265589	0.35010294	0.087	0.095	1
Pigh	0.49394174	0.68635297	0.012	0.01	1
Nhlrc2	0.49506039	0.52746996	0.051	0.048	1
Zfp951	0.49659949	0.49658434	0.01	0.009	1
Pmpcb	0.49697619	0.34749237	0.062	0.068	1
Smpd2	0.49729851	0.29499392	0.011	0.012	1
Tmem59	0.49746577	0.34905803	0.243	0.255	1
Nck1	0.49783494	0.31735625	0.149	0.15	1
Cluap1	0.49824143	0.55605087	0.023	0.021	1
Thoc7	0.49833695	0.40665064	0.196	0.203	1
Zranb1	0.49843038	0.36077361	0.183	0.188	1
Inpp5a	0.49900008	0.33602889	0.026	0.029	1
Pomgnt1	0.49906947	0.57587376	0.016	0.014	1
Chmp1a	0.49962945	0.26656954	0.101	0.109	1
Zfp68	0.4996583	0.36580221	0.075	0.072	1
Zfp523	0.49969454	0.69267337	0.01	0.008	1
Zfp24	0.50055502	0.46680173	0.064	0.061	1
Trappc9	0.50062165	0.54520261	0.045	0.042	1
Tuba1a	0.50066825	0.27662132	0.141	0.142	1
Scamp3	0.50072317	0.28459381	0.102	0.111	1
Cep63	0.5012426	0.44570812	0.054	0.051	1
Mysm1	0.50125763	0.32022545	0.201	0.207	1
Cstf2t	0.50153904	0.36768432	0.039	0.043	1
Hook1	0.50187424	0.45346948	0.031	0.029	1
Mical1	0.50242435	0.27486872	0.121	0.132	1
Prickle3	0.50250393	0.53482441	0.02	0.018	1
Utp11	0.50253205	0.28960167	0.119	0.13	1
Ccdc163	0.50347527	0.73222941	0.018	0.016	1
Stat2	0.50354927	0.35800035	0.076	0.083	1
Stk25	0.50407814	0.35616948	0.038	0.042	1
Ipp	0.50449869	0.49216556	0.012	0.011	1
Tmem192	0.50505357	0.37632907	0.078	0.085	1
Commd3	0.50588171	0.27373419	0.096	0.104	1
1700052K11R	0.50669781	0.78793961	0.011	0.01	1
Gm26724	0.5078072	0.79576759	0.014	0.013	1
Stk32c	0.50936897	0.46272868	0.023	0.021	1

Spg20	0.50944618	0.5603092	0.022	0.025	1
Tmem268	0.50976081	0.39382408	0.018	0.02	1
Tbcb	0.51060828	0.293673	0.189	0.209	1
Nono	0.51118521	0.28118973	0.268	0.283	1
Lrrc57	0.51129037	0.46689367	0.026	0.024	1
Edrf1	0.51139725	0.47718689	0.055	0.052	1
B230307C23R	0.51204336	0.34041161	0.046	0.05	1
Taf6l	0.51302937	0.27261338	0.175	0.192	1
Ppp2r5c	0.51371026	0.27817111	0.262	0.278	1
Sart1	0.51551275	0.32856906	0.121	0.131	1
Rtca	0.51591841	0.40097462	0.033	0.036	1
Clcn5	0.51608619	0.49454508	0.04	0.044	1
Cnep1r1	0.5167373	0.43526029	0.06	0.057	1
Elp2	0.51680624	0.29380682	0.031	0.034	1
Ptcd3	0.51714818	0.27524921	0.053	0.05	1
Thada	0.5173072	0.28593704	0.039	0.043	1
Phf11a	0.51810524	0.32968593	0.01	0.011	1
Ddx51	0.51846215	0.25373076	0.014	0.016	1
Klhl15	0.5186039	0.35469544	0.027	0.03	1
Ppp6r1	0.51924818	0.34086891	0.168	0.171	1
Abcf2	0.51932382	0.4467617	0.056	0.054	1
Dcakd	0.51949187	0.56230226	0.012	0.011	1
Wdr59	0.51966644	0.53216539	0.014	0.013	1
Chpf	0.52012507	0.58095134	0.024	0.022	1
Naa30	0.52017296	0.34858277	0.073	0.071	1
Med1	0.5204495	0.35486906	0.136	0.149	1
Aasdhppt	0.52082355	0.51189082	0.061	0.059	1
Tmem203	0.52086248	0.48590867	0.059	0.056	1
Mtif2	0.52157964	0.59551882	0.037	0.035	1
Adprhl2	0.52160023	0.33363071	0.031	0.034	1
Mrpl22	0.52202391	0.35408516	0.033	0.036	1
Poc5	0.5222926	0.5582822	0.027	0.025	1
Tarsl2	0.52419611	0.60960808	0.011	0.009	1
Denr	0.52443871	0.25990267	0.235	0.246	1
Bbip1	0.52461446	0.3087704	0.113	0.122	1
Cstf1	0.5257491	0.31541465	0.031	0.034	1
Gskip	0.52597651	0.26533816	0.065	0.071	1
Armc8	0.52598275	0.31844595	0.051	0.048	1
Zfp429	0.52632033	0.6555989	0.012	0.01	1
Nploc4	0.52668038	0.28609816	0.056	0.061	1
Arv1	0.52744692	0.40833105	0.01	0.008	1
Mfsd14a	0.52780875	0.3924846	0.12	0.119	1
Dnm1l	0.528122	0.47150734	0.125	0.126	1
Ppp6r2	0.52827024	0.45107244	0.031	0.029	1
Irf3	0.5305031	0.44914284	0.083	0.081	1
Bcl2l13	0.53068235	0.35578417	0.052	0.05	1
Mecp2	0.53100365	0.28022715	0.12	0.13	1
Isca2	0.5311428	0.48109791	0.07	0.076	1

Rnf25	0.53213824	0.54955178	0.029	0.027	1
Axin1	0.53311369	0.38477253	0.078	0.077	1
Pdxdc1	0.53353192	0.27487117	0.048	0.052	1
Bicd2	0.53425449	0.33411703	0.053	0.058	1
Mllt1	0.53442196	0.46142172	0.015	0.017	1
Abhd4	0.53536835	0.7627316	0.025	0.028	1
Tacc1	0.53828191	0.29785162	0.316	0.342	1
Hivep3	0.53900032	0.47734546	0.061	0.067	1
Kdm3a	0.54044399	0.32317015	0.104	0.102	1
Gin1	0.54173389	0.31978369	0.016	0.018	1
Ssr1	0.54175672	0.33543617	0.231	0.259	1
Klhl20	0.54180933	0.30966074	0.036	0.034	1
Wdr61	0.54393513	0.45556592	0.11	0.11	1
Fcho1	0.54517361	0.47214324	0.037	0.035	1
Cfl2	0.54622678	0.45340031	0.099	0.098	1
Mtx3	0.54628405	0.52459761	0.012	0.01	1
Mrpl32	0.54727193	0.27486676	0.21	0.231	1
Zkscan17	0.54806714	0.4584178	0.032	0.03	1
Gosr1	0.54905002	0.53293932	0.073	0.071	1
Qpctl	0.54910407	0.53952521	0.014	0.016	1
Thoc3	0.54977119	0.52127663	0.059	0.057	1
Gstcd	0.54980557	0.68142599	0.021	0.02	1
Dhcr7	0.54988998	0.26198966	0.015	0.017	1
Gm26541	0.55049464	0.32203816	0.012	0.01	1
Zdhhc9	0.55059341	0.42750531	0.026	0.029	1
Ilkap	0.55066604	0.35951238	0.114	0.114	1
Lncpint	0.55095611	0.34911614	0.164	0.167	1
Ang	0.5522399	0.68811278	0.015	0.014	1
Imp4	0.55394976	0.37064864	0.086	0.094	1
Galnt12	0.55403163	0.30533531	0.026	0.028	1
Ubiad1	0.55589938	0.32620859	0.009	0.01	1
Gpi1	0.55599171	0.25540172	0.373	0.411	1
Zbtb39	0.55707045	0.39154141	0.01	0.012	1
Gmpr2	0.55789616	0.44892165	0.027	0.03	1
Iqcc	0.55794587	0.31418022	0.021	0.02	1
Slc35g1	0.55826568	0.48248846	0.012	0.011	1
Socs6	0.55928895	0.51551204	0.049	0.053	1
Glipr1	0.55945792	0.4418555	0.125	0.127	1
Abcf3	0.55968297	0.36482478	0.054	0.052	1
Get4	0.56084196	0.33108238	0.06	0.065	1
Zcchc4	0.56137871	0.31815051	0.029	0.027	1
Scaf1	0.56175286	0.27181549	0.058	0.063	1
Ndst2	0.56235235	0.51690649	0.017	0.019	1
Rbsn	0.56251409	0.62038586	0.042	0.04	1
Trim44	0.56287266	0.26106598	0.125	0.124	1
Snx11	0.56313017	0.49776377	0.031	0.03	1
Cenpj	0.56422789	0.43982453	0.013	0.011	1
Mgp	0.56426883	0.2657024	0.02	0.022	1

Naa35	0.56480774	0.50255487	0.078	0.077	1
Zdhhc4	0.56482781	0.54830398	0.031	0.034	1
Tubgcp5	0.56484293	0.5082387	0.029	0.027	1
Pgm1	0.56585562	0.43956899	0.034	0.037	1
Gmds	0.56592589	0.2713329	0.036	0.039	1
Tprkb	0.56603559	0.39420719	0.037	0.035	1
Zfp275	0.56632033	0.2681326	0.021	0.023	1
Fbxo34	0.566412	0.34419807	0.092	0.1	1
Copg1	0.56736111	0.50896405	0.089	0.088	1
Mon1a	0.56738622	0.28193007	0.013	0.015	1
Sdr39u1	0.56745338	0.469035	0.015	0.014	1
Gtf2a2	0.56754479	0.29136106	0.134	0.145	1
Atpaf1	0.56822899	0.27706763	0.015	0.014	1
Gja1	0.56862645	0.89732604	0.012	0.011	1
Nek1	0.56929382	0.4156052	0.016	0.014	1
Mthfd2l	0.5693364	0.60700713	0.021	0.019	1
Ppp2r1a	0.56968769	0.35660435	0.193	0.202	1
Tuft1	0.57027701	0.37358119	0.011	0.009	1
Casp9	0.57073969	0.64386141	0.01	0.008	1
Tmub2	0.5713494	0.30787183	0.036	0.039	1
Brd8	0.5728796	0.27213382	0.206	0.211	1
Nras	0.5729247	0.33528574	0.207	0.216	1
Tatdn3	0.57331253	0.53187263	0.01	0.011	1
Wiz	0.57378514	0.46576508	0.029	0.032	1
Larp1	0.57541123	0.29681766	0.228	0.24	1
Nsun4	0.57551403	0.44322962	0.048	0.046	1
Dnlz	0.57682722	0.49653473	0.06	0.058	1
Gcc1	0.57701858	0.52874727	0.043	0.041	1
2900097C17R	0.57915512	0.25319286	0.219	0.24	1
Cd2bp2	0.58079973	0.38075293	0.12	0.12	1
Psmg3	0.58300475	0.61597531	0.053	0.052	1
Cd151	0.58322847	0.43802253	0.009	0.01	1
Fbxl18	0.58385392	0.42048904	0.011	0.01	1
Smarcad1	0.58386666	0.29184278	0.112	0.12	1
Rab28	0.58404508	0.59048394	0.043	0.041	1
Paip1	0.58538221	0.37018279	0.062	0.067	1
Mef2d	0.58667947	0.27990091	0.268	0.283	1
2210016L21Ri	0.58683048	0.32560207	0.062	0.067	1
Unc119b	0.58809735	0.31474477	0.085	0.092	1
S100pbp	0.58999997	0.30001273	0.056	0.054	1
Fbxl19	0.59007121	0.52846025	0.01	0.009	1
Zmynd8	0.59053568	0.3025478	0.107	0.106	1
Gbf1	0.5905598	0.38183163	0.077	0.076	1
Cln5	0.59173342	0.41096979	0.06	0.065	1
Ubr1	0.59197367	0.3444604	0.07	0.068	1
Ddx1	0.59433296	0.29067557	0.076	0.081	1
Rnf34	0.59463329	0.36239806	0.063	0.061	1
Wdr20	0.59464779	0.40685893	0.089	0.088	1

Zmym1	0.59517452	0.58421169	0.037	0.036	1
Arrdc2	0.59563524	0.30796116	0.011	0.012	1
Atat1	0.59658593	0.71469349	0.015	0.014	1
Rnf215	0.59693958	0.47002232	0.037	0.036	1
Rnf123	0.59928941	0.44803788	0.042	0.04	1
Uck1	0.60001428	0.49735671	0.026	0.028	1
Gps1	0.6002326	0.32837659	0.064	0.068	1
Fam177a	0.60115098	0.3460316	0.053	0.057	1
Setdb1	0.60157747	0.56644244	0.042	0.04	1
C1galt1c1	0.60270809	0.44725551	0.036	0.039	1
Btbd1	0.60345637	0.36501739	0.105	0.114	1
Pdzd11	0.60414894	0.53140043	0.06	0.058	1
Zbtb42	0.60457345	0.64692641	0.02	0.019	1
Mcrs1	0.60567121	0.42481304	0.088	0.087	1
Gm49359	0.60704135	0.37853151	0.02	0.019	1
Zfp746	0.60726953	0.45051888	0.084	0.083	1
Smarcd2	0.60869382	0.40972191	0.123	0.124	1
Tmem19	0.60882853	0.70028401	0.028	0.026	1
Dapk3	0.60903904	0.30925578	0.08	0.085	1
Letm1	0.60928896	0.44120673	0.096	0.096	1
Acp1	0.60934503	0.34992505	0.128	0.138	1
Ccny	0.60966499	0.49872736	0.104	0.104	1
Suz12	0.61035913	0.34105092	0.241	0.255	1
Chtop	0.6106204	0.29927295	0.187	0.205	1
Ripk3	0.6106454	0.36830537	0.034	0.037	1
Ap2a1	0.61068067	0.58283174	0.046	0.045	1
R3hcc1	0.61123511	0.49860035	0.018	0.017	1
Ndc80	0.61167476	0.7046328	0.015	0.014	1
Timm17a	0.61212421	0.32442666	0.156	0.17	1
Mtmr4	0.61222595	0.50807937	0.029	0.027	1
Tmem43	0.61240917	0.61664496	0.043	0.042	1
Asb1	0.61286107	0.40750774	0.016	0.015	1
C2cd2l	0.61289395	0.2524519	0.016	0.018	1
Plcl1	0.61324165	0.47816025	0.019	0.021	1
Zfp706	0.6143892	0.27279291	0.268	0.284	1
E2f4	0.61446725	0.46068094	0.077	0.076	1
Pdk3	0.61568851	0.41561044	0.047	0.051	1
Cenpc1	0.61572742	0.35774811	0.064	0.062	1
Rrm2b	0.61575858	0.36793181	0.067	0.066	1
Rnf11	0.61592922	0.29091453	0.103	0.11	1
Idh3a	0.61775238	0.25656542	0.098	0.096	1
Tial1	0.6182873	0.36787104	0.124	0.134	1
Adnp	0.61915082	0.31440953	0.03	0.033	1
Smim20	0.61920163	0.26313981	0.067	0.071	1
Atp6v0a2	0.61921757	0.470995	0.066	0.065	1
Arl14ep	0.61925086	0.45248543	0.044	0.043	1
Nit1	0.61998653	0.40951228	0.053	0.052	1
Zfp770	0.62018351	0.49180198	0.022	0.02	1

Taf5l	0.62041658	0.3455207	0.084	0.09	1
Lzts1	0.62221233	0.38619421	0.009	0.01	1
Zfp944	0.62276807	0.61393318	0.018	0.017	1
Rbck1	0.6228418	0.34272295	0.121	0.13	1
Zfx	0.62345972	0.26744287	0.109	0.117	1
Tsnax	0.62353639	0.26417372	0.045	0.048	1
Eif4enif1	0.62372959	0.35625556	0.065	0.063	1
Mfap1a	0.6248299	0.34394354	0.086	0.085	1
Btrc	0.62519623	0.25059024	0.025	0.027	1
Skp1a	0.62528138	0.28975287	0.208	0.228	1
Zfp1	0.62582749	0.27814319	0.037	0.04	1
Mfn2	0.62666071	0.37940479	0.033	0.036	1
Stau1	0.62673055	0.41997532	0.085	0.084	1
Ap1m1	0.62697681	0.48319107	0.074	0.073	1
Rspry1	0.62784165	0.35834006	0.085	0.091	1
Fez2	0.62794367	0.63354899	0.029	0.028	1
Becn1	0.62846691	0.27181621	0.177	0.192	1
Polr3f	0.62871843	0.27047521	0.055	0.053	1
Nfkbil1	0.62884718	0.33896411	0.033	0.036	1
Natd1	0.62925393	0.36476527	0.016	0.015	1
Dbp	0.62947926	0.459212	0.015	0.014	1
Kif1b	0.62974847	0.25412991	0.135	0.136	1
Coa4	0.62981419	0.4148834	0.021	0.023	1
Ciao1	0.62985751	0.33734281	0.047	0.051	1
Nek9	0.63051654	0.34502802	0.062	0.067	1
Oxa1l	0.63099089	0.30027008	0.102	0.109	1
Timm21	0.63120028	0.35515981	0.022	0.024	1
Ing1	0.63134221	0.25998924	0.128	0.137	1
Mindy2	0.63161155	0.27598813	0.104	0.103	1
Usp46	0.63199495	0.86000744	0.011	0.01	1
Map3k12	0.63267518	0.61378914	0.022	0.02	1
Thumpd3	0.63429555	0.40885015	0.06	0.058	1
Snx4	0.63451512	0.40235032	0.116	0.118	1
Cdk5rap2	0.63452189	0.34261726	0.049	0.047	1
Ercc5	0.63496407	0.47324117	0.045	0.043	1
Hmgxb3	0.63579603	0.40398788	0.029	0.032	1
Gmnn	0.63638135	0.31812887	0.061	0.06	1
Dgke	0.63660419	0.27462853	0.036	0.038	1
Ap3m2	0.63705602	0.28612483	0.022	0.024	1
Mrps25	0.63744046	0.35291749	0.063	0.067	1
Mtf2	0.63767852	0.31319321	0.138	0.139	1
Mga	0.63773981	0.40026934	0.153	0.156	1
Ppp2r1b	0.63784695	0.47078492	0.033	0.031	1
Otud4	0.63821909	0.41043382	0.122	0.124	1
4930524J08Ril	0.63833592	0.37052767	0.01	0.011	1
Sufu	0.63887388	0.46358459	0.022	0.02	1
Fem1c	0.64040325	0.27602596	0.245	0.261	1
Fam210b	0.64046388	0.55472863	0.029	0.028	1

Kpna3	0.64116567	0.40000772	0.106	0.107	1
Fzr1	0.64205442	0.26819443	0.049	0.052	1
Fbxw9	0.64254791	0.8225641	0.011	0.01	1
Rad1	0.64255666	0.36501565	0.017	0.016	1
Htatip2	0.6427401	0.47575321	0.058	0.057	1
Orc5	0.64286726	0.60027865	0.011	0.01	1
Ckap5	0.6429563	0.47438732	0.088	0.088	1
Casd1	0.64322762	0.28337539	0.031	0.033	1
Emc1	0.64342514	0.33746048	0.056	0.06	1
Acap3	0.64389215	0.3502072	0.014	0.015	1
Pgrmc2	0.64537661	0.4635338	0.082	0.081	1
Sec61a1	0.64600987	0.30847462	0.217	0.229	1
Zfp51	0.64622153	0.25794917	0.025	0.027	1
Sar1b	0.64658196	0.28366838	0.123	0.133	1
Adprm	0.64671344	0.2872833	0.037	0.035	1
Tab3	0.64677342	0.351087	0.038	0.037	1
Pphln1	0.64739241	0.31460052	0.058	0.062	1
Pef1	0.64869861	0.4763536	0.042	0.041	1
Gm5547	0.64910557	0.29379545	0.085	0.091	1
Prkaa1	0.65068357	0.29292315	0.035	0.037	1
Bckdk	0.65192012	0.63488731	0.035	0.034	1
Rdh10	0.65325845	0.37135625	0.012	0.013	1
Rnf181	0.65349432	0.3856514	0.081	0.08	1
Eftud2	0.65391411	0.41778025	0.096	0.096	1
Nf2	0.65394922	0.43990333	0.038	0.036	1
Pias4	0.65495189	0.41384797	0.087	0.087	1
1110012L19Ri	0.65512062	0.31363593	0.037	0.035	1
Lpin1	0.6552581	0.44758346	0.057	0.055	1
Ppcdc	0.65534621	0.25880273	0.026	0.028	1
Msh6	0.65556129	0.53074385	0.025	0.023	1
Efr3a	0.65597357	0.32639207	0.169	0.182	1
Spg11	0.65600448	0.43258176	0.018	0.02	1
Zxdc	0.65647528	0.38675535	0.035	0.033	1
Klc2	0.65685743	0.4691385	0.01	0.009	1
Ak3	0.65696233	0.40774237	0.033	0.035	1
Cops4	0.65717803	0.43249471	0.145	0.148	1
Arhgef11	0.65808331	0.65158737	0.028	0.026	1
Rc3h2	0.65815762	0.32418272	0.179	0.185	1
BC005624	0.6583312	0.29433406	0.221	0.234	1
Mmadhc	0.65887364	0.5498653	0.101	0.103	1
Sin3a	0.65907327	0.30702517	0.088	0.086	1
Tmem128	0.65953477	0.33153221	0.216	0.238	1
Pank4	0.65994862	0.32148706	0.023	0.025	1
Kdelr1	0.66053319	0.32691231	0.136	0.147	1
Snx16	0.66071084	0.77406701	0.021	0.02	1
Supt3	0.66115163	0.591361	0.027	0.025	1
H2-Ke6	0.66127228	0.45205142	0.09	0.09	1
Rabep2	0.66166299	0.35838366	0.04	0.038	1

Nkiras1	0.66231121	0.33461521	0.026	0.024	1
Zswim8	0.66291523	0.25162209	0.102	0.109	1
Crybg1	0.66293887	0.45612409	0.128	0.131	1
Glmn	0.66336641	0.34125474	0.017	0.016	1
Plec	0.66410631	0.40326237	0.114	0.115	1
Ergic2	0.66431664	0.28921675	0.146	0.156	1
Pias1	0.6652721	0.28779362	0.133	0.135	1
Zfp395	0.66533113	0.25692664	0.019	0.017	1
Jtb	0.66555085	0.27991315	0.16	0.174	1
Dedd	0.66556594	0.42764584	0.047	0.045	1
2610001J05Ril	0.66712063	0.41601677	0.108	0.116	1
Tbck	0.66838394	0.25967806	0.046	0.049	1
Tmem55a	0.66850912	0.49088005	0.019	0.017	1
Spns1	0.66907811	0.35254008	0.034	0.036	1
Ndufs1	0.66910488	0.29079415	0.084	0.09	1
Bag5	0.66937664	0.30510018	0.048	0.051	1
Sfi1	0.67119153	0.29748639	0.034	0.032	1
Yipf1	0.67148489	0.53514543	0.086	0.086	1
Nfs1	0.6715736	0.36231097	0.032	0.031	1
Dync1li1	0.67175581	0.41151477	0.141	0.146	1
Meaf6	0.67339428	0.40123765	0.056	0.06	1
Yme1l1	0.67429666	0.26201209	0.192	0.208	1
Ercc2	0.67497982	0.37952649	0.023	0.022	1
Nsun3	0.67502473	0.42898783	0.03	0.029	1
Eif1ax	0.67599265	0.26800037	0.198	0.216	1
Ap1s1	0.67626356	0.28129541	0.05	0.053	1
Tef	0.67636316	0.4828135	0.027	0.025	1
Chmp5	0.67653893	0.37781236	0.165	0.172	1
Tars2	0.67679009	0.31534063	0.03	0.028	1
Tmem248	0.6771144	0.32172226	0.152	0.165	1
Edem3	0.6778569	0.44241067	0.043	0.042	1
Mical3	0.67786122	0.48262974	0.01	0.011	1
Fbrsl1	0.67796894	0.29335943	0.081	0.086	1
Spindoc	0.67821508	0.40665355	0.013	0.014	1
Klhdc2	0.67924142	0.28816931	0.062	0.061	1
Tmem161a	0.67927862	0.56027414	0.033	0.031	1
Snapc1	0.67962831	0.40829645	0.128	0.13	1
Cdk9	0.68029979	0.37028802	0.15	0.163	1
Ints10	0.68113012	0.40404036	0.017	0.019	1
Fam168a	0.68309601	0.48936858	0.074	0.079	1
Drg1	0.68315983	0.31180919	0.079	0.084	1
Casp8	0.68355815	0.38583715	0.113	0.114	1
Rundc1	0.68410954	0.45957912	0.025	0.024	1
Ddx28	0.68415177	0.27829417	0.025	0.026	1
F8a	0.68433168	0.29917266	0.023	0.025	1
Nup153	0.68492845	0.29388818	0.156	0.16	1
Fam192a	0.68493618	0.40165002	0.109	0.11	1
Golph3l	0.68527391	0.56308364	0.043	0.046	1

Fbxo9	0.68553819	0.33499549	0.058	0.057	1
Cntln	0.68685358	0.75887618	0.021	0.023	1
Ambra1	0.68688054	0.57677467	0.044	0.047	1
Commd2	0.68690169	0.51386828	0.079	0.079	1
Mad2l1bp	0.68740839	0.4791077	0.044	0.043	1
Map3k4	0.68793803	0.56105674	0.049	0.048	1
Bbc3	0.6882258	0.31874991	0.026	0.028	1
Uba5	0.68842913	0.54909992	0.095	0.096	1
Gpn3	0.68846317	0.50980044	0.077	0.076	1
Nin	0.68947802	0.29375939	0.131	0.133	1
Arl16	0.68990505	0.36430387	0.012	0.013	1
Prpf6	0.68999922	0.37022666	0.121	0.123	1
Acta2	0.69040093	0.44078083	0.015	0.016	1
Rab29	0.69047331	0.44335021	0.015	0.017	1
Pgm3	0.69084155	0.45145356	0.026	0.025	1
Bloc1s4	0.69118088	0.47920211	0.082	0.087	1
Pkmyt1	0.69120238	0.2571923	0.012	0.013	1
Rab2b	0.69124586	0.4163071	0.044	0.042	1
Tmem218	0.69223782	0.3956089	0.012	0.013	1
Fig4	0.69232799	0.5609058	0.014	0.013	1
Cenpo	0.69275179	0.39827226	0.009	0.01	1
Ppm1a	0.69285435	0.35111681	0.179	0.186	1
Ints14	0.69332182	0.43696772	0.037	0.035	1
Wdr92	0.69388028	0.78043254	0.014	0.013	1
Gm20045	0.69451847	0.49927178	0.015	0.014	1
2310022B05R	0.69482484	0.62187871	0.021	0.02	1
Prkag1	0.69529201	0.44941896	0.056	0.055	1
Tmem181a	0.69614094	0.49672155	0.027	0.029	1
3830406C13R	0.69617546	0.41120483	0.088	0.088	1
Cox19	0.696762	0.31306355	0.045	0.048	1
Mtap	0.69697707	0.30182321	0.027	0.029	1
Tgds	0.69909067	0.48535226	0.011	0.012	1
Ube3b	0.69909604	0.40832787	0.049	0.052	1
Med6	0.69941886	0.31998462	0.038	0.041	1
Stard3nl	0.70017952	0.55505329	0.091	0.099	1
Rnf219	0.70061048	0.33330662	0.019	0.02	1
Zbtb26	0.70308639	0.69081783	0.027	0.026	1
Stradb	0.70434904	0.57504828	0.015	0.017	1
Josd2	0.70473658	0.45905008	0.062	0.062	1
Gm31597	0.70487868	0.28908185	0.023	0.022	1
Smim19	0.70508293	0.53017651	0.065	0.065	1
Nedd1	0.70511958	0.54486709	0.025	0.024	1
Slc25a24	0.70552221	0.50422767	0.064	0.067	1
Slx4	0.70605466	0.31495294	0.016	0.015	1
Caml	0.70657915	0.35932211	0.034	0.036	1
Tgfbrap1	0.70708171	0.42599626	0.028	0.03	1
Map3k11	0.70736155	0.45692547	0.043	0.042	1
Rnpc3	0.70757145	0.33634557	0.095	0.101	1

2410131K14R	0.70853003	0.56000822	0.023	0.025	1
Tbk1	0.70905805	0.36814604	0.151	0.156	1
6230400D17R	0.70909893	0.35258159	0.013	0.012	1
Cfdp1	0.70941918	0.40626554	0.139	0.143	1
Map4	0.70992275	0.38184978	0.116	0.125	1
Ksr1	0.7121911	0.30969198	0.021	0.023	1
Dhx40	0.71238286	0.30819883	0.189	0.198	1
Tfdp2	0.7130008	0.45346072	0.035	0.034	1
Fam174a	0.71301659	0.39136302	0.149	0.153	1
Skiv2l	0.71365383	0.46470505	0.076	0.076	1
Mgat1	0.71445328	0.3830332	0.043	0.045	1
Ing5	0.71468527	0.36919691	0.114	0.122	1
Ube2k	0.71478028	0.25068821	0.273	0.304	1
Cdc34	0.71492496	0.37670409	0.156	0.161	1
Utp23	0.71519912	0.36445855	0.068	0.068	1
Ppic	0.71615655	0.3473174	0.009	0.01	1
Zc3h4	0.71631171	0.43413749	0.055	0.059	1
Cnppd1	0.71677058	0.45268557	0.125	0.129	1
Tsc1	0.71738201	0.35168939	0.084	0.084	1
Chm	0.71899183	0.41376773	0.078	0.078	1
Nprl2	0.71970312	0.69682799	0.017	0.016	1
Foxo3	0.72128452	0.25726349	0.055	0.058	1
Heatr5b	0.72181653	0.42828332	0.027	0.026	1
Fem1a	0.72214632	0.47403549	0.037	0.036	1
Stambp	0.72271554	0.71438705	0.046	0.045	1
Lats1	0.72323987	0.40633567	0.067	0.071	1
Zfp101	0.72466831	0.31796792	0.02	0.019	1
Katna1	0.72485072	0.38573421	0.138	0.142	1
Tmem170b	0.72564118	0.41285376	0.035	0.037	1
Zfp810	0.72601755	0.45069196	0.012	0.011	1
Morn2	0.72647214	0.76799196	0.01	0.01	1
Upf1	0.7268811	0.40891227	0.101	0.103	1
Fbxl6	0.72710281	0.53440089	0.043	0.046	1
Rnf216	0.72739427	0.475326	0.077	0.077	1
Kif3a	0.72743884	0.45615945	0.016	0.017	1
Zkscan8	0.72799294	0.34197587	0.013	0.012	1
Mark4	0.72830406	0.40282177	0.034	0.033	1
Ube2l6	0.7286528	0.45410294	0.031	0.03	1
Tmem87b	0.72915355	0.45444431	0.063	0.063	1
Sf3a1	0.72947821	0.31375468	0.096	0.102	1
Zdhhc16	0.72985943	0.619385	0.018	0.019	1
Psme4	0.73034756	0.41674275	0.134	0.138	1
Retreg3	0.73139361	0.39342831	0.084	0.084	1
Lsm10	0.73148184	0.53988559	0.062	0.062	1
Rhot1	0.73236471	0.34864362	0.052	0.055	1
Parp4	0.73254929	0.32401695	0.091	0.096	1
Elac1	0.73258262	0.28277714	0.013	0.014	1
Zfp524	0.73304707	0.41304767	0.039	0.039	1

Zfp407	0.73338806	0.31228664	0.059	0.062	1
Zfp142	0.73341848	0.39327089	0.044	0.043	1
Ndufaf1	0.73372608	0.53064416	0.019	0.018	1
Sept2	0.73399323	0.49477013	0.039	0.038	1
Nudt15	0.73422093	0.32887903	0.012	0.013	1
Gm14305	0.73582109	0.28576446	0.011	0.012	1
Tmco6	0.73631262	0.29911707	0.017	0.018	1
Mrps23	0.73689339	0.33698805	0.129	0.137	1
Ddx47	0.73727837	0.32458971	0.148	0.159	1
Efl1	0.73808789	0.46549356	0.039	0.038	1
Pank2	0.73903776	0.41374737	0.133	0.137	1
Rnf169	0.73911903	0.38548789	0.036	0.038	1
8030462N17R	0.73923101	0.37729778	0.022	0.024	1
Zfp120	0.73929891	0.37049607	0.046	0.045	1
Stk38l	0.73946718	0.69429094	0.025	0.024	1
Eid2b	0.74073945	0.49030419	0.012	0.011	1
Ssna1	0.74076795	0.36943474	0.082	0.086	1
Rhot2	0.74095757	0.50504708	0.026	0.025	1
Lpcat4	0.7409613	0.55656635	0.023	0.022	1
Rbm12b2	0.74098834	0.37151912	0.013	0.014	1
Dyrk1a	0.74180022	0.36618059	0.177	0.186	1
Zfp930	0.74249434	0.32219666	0.015	0.015	1
Kmt2b	0.74314954	0.43665144	0.093	0.099	1
2310033P09R	0.743244	0.37614648	0.091	0.091	1
Xxylt1	0.74365584	0.50443561	0.022	0.021	1
Sirt7	0.74423651	0.35139466	0.113	0.115	1
Smpd4	0.74482014	0.37566821	0.029	0.031	1
Noc2l	0.7452406	0.35379822	0.155	0.168	1
Nipsnap2	0.74532595	0.38279518	0.036	0.038	1
Psmd5	0.74544932	0.37439897	0.035	0.034	1
Itgb3bp	0.74559075	0.28114448	0.027	0.026	1
Slc17a5	0.74575921	0.55004182	0.015	0.014	1
Rbm14	0.74588658	0.30918635	0.044	0.047	1
Zmynd11	0.74600104	0.32196939	0.174	0.18	1
Wdr44	0.74608978	0.35675861	0.062	0.062	1
2700097O09R	0.74612344	0.32010135	0.03	0.029	1
Rsf1	0.74651566	0.30517172	0.195	0.204	1
2310035C23R	0.74652522	0.25899246	0.131	0.133	1
Gigyf1	0.74655036	0.27293495	0.13	0.131	1
Cnot8	0.74700107	0.28093552	0.073	0.077	1
Tex261	0.74816025	0.46761821	0.121	0.124	1
Lig1	0.74956558	0.39800344	0.061	0.06	1
Dhx29	0.7501356	0.42713369	0.031	0.033	1
Srp54a	0.75117941	0.35287339	0.092	0.092	1
Tram1	0.75137519	0.27058229	0.233	0.254	1
Unc45a	0.75149427	0.28506475	0.077	0.081	1
Thap7	0.75161317	0.28358403	0.039	0.042	1
Gtpbp10	0.75165437	0.66161572	0.022	0.023	1

Zfp655	0.75189559	0.4612972	0.142	0.148	1
Zbtb41	0.7523008	0.42994659	0.034	0.033	1
Mpdu1	0.7532104	0.42493275	0.096	0.102	1
Calcrl	0.7534381	0.31796449	0.028	0.029	1
Tbc1d32	0.75558596	0.5623003	0.01	0.01	1
Rce1	0.75617518	0.48762745	0.051	0.054	1
Dcaf6	0.75719306	0.27656796	0.019	0.02	1
Atg14	0.75777348	0.56556147	0.058	0.061	1
Smim27	0.75801122	0.28974391	0.08	0.083	1
Sart3	0.75801587	0.4075367	0.073	0.072	1
Anxa7	0.75929405	0.3966076	0.126	0.135	1
Rngtt	0.76096207	0.44353031	0.083	0.083	1
Lrrc8a	0.76097345	0.64363178	0.025	0.026	1
Ccz1	0.76103912	0.35626417	0.122	0.13	1
Glrx5	0.76117115	0.42404699	0.128	0.132	1
4930402H24R	0.76139976	0.63405182	0.027	0.026	1
Nsfl1c	0.76149428	0.45970364	0.104	0.105	1
Snrnp25	0.76175552	0.30121565	0.056	0.059	1
Riok2	0.7618436	0.51356827	0.046	0.045	1
Zmiz2	0.76185477	0.36945443	0.145	0.155	1
C87436	0.76203515	0.26216516	0.053	0.056	1
Pex2	0.76277285	0.45584714	0.036	0.038	1
Zfp90	0.76339303	1.02365213	0.012	0.013	1
Asns	0.76391698	0.25555448	0.051	0.053	1
Clp1	0.76421218	0.35354005	0.069	0.069	1
Sbf2	0.76449511	0.74014948	0.034	0.034	1
Fam220a	0.76575052	0.47757317	0.072	0.072	1
Aida	0.76646208	0.46222771	0.075	0.075	1
Vezt	0.76662166	0.29663779	0.017	0.018	1
Capn2	0.76697095	0.53334022	0.094	0.096	1
Siae	0.76712726	0.57273511	0.013	0.014	1
Polr1e	0.76913864	0.67587336	0.012	0.012	1
Mrpl40	0.76999281	0.5872989	0.067	0.067	1
Nup155	0.77230399	0.33196536	0.031	0.03	1
Csnk2a1	0.77247849	0.29916514	0.17	0.177	1
Prdm15	0.77265698	0.52293202	0.013	0.012	1
Vps9d1	0.77273442	0.3588962	0.03	0.031	1
Fen1	0.77305833	0.47282421	0.039	0.039	1
A130010J15Ril	0.77316038	0.37112338	0.01	0.009	1
Trappc10	0.77439825	0.48872946	0.113	0.116	1
Sh3bp4	0.7745475	1.07445669	0.021	0.023	1
B3gat2	0.77492039	0.41758459	0.014	0.015	1
Akr1b10	0.77509107	0.53600892	0.031	0.033	1
Ints5.1	0.77522305	0.41126258	0.018	0.019	1
Pex11b	0.77548861	0.52114458	0.015	0.016	1
Cc2d1b	0.77571248	0.41897143	0.061	0.06	1
Ntan1	0.77696401	0.38228078	0.142	0.148	1
Mex3d	0.77730154	0.52549448	0.012	0.011	1

Mettl3	0.77745688	0.50710906	0.037	0.037	1
Nlrc5	0.77762814	0.27187435	0.086	0.086	1
Flad1	0.77780374	0.60701492	0.018	0.017	1
Mon2	0.7778538	0.27433452	0.114	0.121	1
Surf1	0.77885021	0.39453937	0.085	0.09	1
Mterf4	0.78059593	0.52595085	0.018	0.017	1
Mfap3	0.78079768	0.34279087	0.045	0.045	1
Gins4	0.78185918	0.54333321	0.052	0.052	1
Wdr53	0.7824986	0.32426354	0.023	0.025	1
Lipa	0.78295483	0.57687318	0.093	0.095	1
Dpp3	0.78363884	0.49819632	0.044	0.047	1
Ncbp3	0.78442993	0.27949678	0.096	0.096	1
Ikzf5	0.7846833	0.28260216	0.092	0.097	1
Slc25a40	0.78531341	0.42112821	0.042	0.041	1
Rnf26	0.78560519	0.2718208	0.018	0.019	1
3010003L21Ri	0.78617185	0.37305775	0.013	0.014	1
Fbxo31	0.78648353	0.49364206	0.02	0.021	1
Flot2	0.78748566	0.48078113	0.039	0.041	1
Zfp932	0.78755985	0.29181968	0.021	0.022	1
Uimc1	0.78762226	0.34593331	0.134	0.143	1
Bap1	0.78799752	0.38914123	0.053	0.055	1
Tmem39b	0.78852809	0.40015355	0.015	0.016	1
Edc3	0.78875141	0.53635634	0.026	0.025	1
Mbtps2	0.78948958	0.28289329	0.031	0.033	1
Gigyf2	0.789514	0.28011538	0.109	0.11	1
1600012H06R	0.78955252	0.57503466	0.041	0.041	1
Rnf4	0.78985068	0.29985568	0.208	0.226	1
Mis18a	0.79012097	0.34655445	0.054	0.057	1
Tbc1d22b	0.79012931	0.33475895	0.021	0.02	1
Agk	0.79048419	0.29764723	0.036	0.035	1
Tfg	0.79078258	0.47138865	0.068	0.072	1
Ank	0.79155192	0.47121353	0.051	0.051	1
Hps3	0.79197519	0.36356947	0.088	0.088	1
Hmcn2	0.79257872	0.65820857	0.01	0.01	1
Skiv2l2	0.79315543	0.29916421	0.106	0.107	1
Ddx52	0.7945409	0.35997355	0.15	0.156	1
Tom1l2	0.79501312	0.52219583	0.01	0.011	1
Sos1	0.79504869	0.37305202	0.052	0.054	1
Zfp358	0.79520704	0.66254608	0.009	0.01	1
Chmp3	0.7954361	0.39529124	0.144	0.155	1
B3galt6	0.79576813	0.42456986	0.012	0.012	1
Pank3	0.79602291	0.3539102	0.109	0.111	1
Rnf44	0.79628483	0.31168075	0.106	0.112	1
Aven	0.79653301	0.46756949	0.044	0.046	1
Ptges2	0.79661422	0.54582496	0.013	0.014	1
Bsdc1	0.7968895	0.26574333	0.102	0.107	1
Tagln	0.79738568	0.29502196	0.012	0.012	1
Tmem201	0.7976005	0.31204303	0.013	0.012	1

H60b	0.79784666	0.29814775	0.018	0.017	1
Scap	0.79805657	0.2695923	0.038	0.037	1
Stx5a	0.79834181	0.34415502	0.149	0.16	1
Nfyc	0.79909036	0.2931296	0.068	0.071	1
Rfxap	0.79972898	0.31935442	0.098	0.099	1
St14	0.79988422	0.27867024	0.011	0.011	1
Col3a1	0.79998167	0.26310154	0.084	0.088	1
Gm26982	0.80031529	0.41019317	0.025	0.026	1
Nacc1	0.80062111	0.45293302	0.062	0.062	1
Nle1	0.80202697	0.37532752	0.017	0.017	1
Keap1	0.80354402	0.28144252	0.047	0.049	1
Fmnl1	0.80442267	0.29520635	0.204	0.216	1
Usp22	0.80464798	0.47901262	0.065	0.065	1
Bmi1	0.80489179	0.27778301	0.039	0.041	1
Galt	0.80531098	0.67354859	0.027	0.026	1
Hinfp	0.80628232	0.30022556	0.043	0.045	1
Fam234a	0.80645903	0.83644558	0.034	0.035	1
Slc4a1ap	0.80682349	0.33414595	0.095	0.096	1
Utf1	0.80796192	0.47992422	0.013	0.014	1
Snx17	0.8087659	0.31577704	0.133	0.142	1
Cd1d1	0.80883021	0.78383954	0.025	0.024	1
Spata6	0.80953726	0.46627187	0.06	0.06	1
Dmap1	0.81039547	0.49823762	0.023	0.023	1
Zfp384	0.81103749	0.44912631	0.051	0.05	1
Zfp672	0.81169654	0.29033283	0.109	0.111	1
Gins2	0.81180361	0.55835734	0.016	0.015	1
Ccl11	0.81221496	0.37339804	0.013	0.013	1
Jrkl	0.81233088	0.54711022	0.043	0.043	1
Ikbkg	0.81286594	0.35161349	0.03	0.031	1
Atg4b	0.8130388	0.45211585	0.055	0.055	1
Ttc17	0.8130449	0.45970108	0.042	0.044	1
Oraov1	0.81348491	0.56682674	0.042	0.042	1
Cep57l1	0.81359402	0.47569	0.013	0.013	1
Tfpt	0.81381515	0.40245818	0.03	0.03	1
Zfp868	0.81438317	0.46947775	0.054	0.054	1
Med14	0.81443808	0.30526312	0.088	0.089	1
Sec22b	0.81470955	0.35415679	0.128	0.136	1
Taf2	0.81543746	0.4585698	0.047	0.047	1
Cacna1s	0.81547503	0.5130577	0.049	0.048	1
Gm20707	0.81604108	0.31441937	0.017	0.016	1
Dap3	0.81606839	0.49828753	0.091	0.093	1
Helq	0.81785055	0.28656287	0.01	0.01	1
Zbtb24	0.81814085	0.26144731	0.043	0.045	1
Bcs1l	0.81861901	0.37116913	0.012	0.012	1
Anapc4	0.81865669	0.42627844	0.041	0.043	1
Mul1	0.81872651	0.40399866	0.064	0.067	1
Bcas3	0.81903982	0.29847016	0.035	0.037	1
Tex264	0.82017544	0.48538374	0.052	0.055	1

Abhd13	0.82041284	0.34166855	0.043	0.044	1
Mios	0.82044726	0.512342	0.021	0.021	1
Rnps1	0.82072733	0.27506562	0.22	0.233	1
Mib2	0.82087884	0.58150013	0.011	0.011	1
Wdr33	0.82093596	0.35389429	0.056	0.058	1
Hmgxb4	0.82139484	0.52080682	0.044	0.046	1
Ptdss2	0.82239078	0.35041988	0.014	0.014	1
Bcas2	0.82249151	0.30733821	0.239	0.26	1
Taf11	0.82413165	0.4111395	0.068	0.068	1
Mlf2	0.8243615	0.37809434	0.143	0.154	1
Dbr1	0.82538367	0.47565907	0.045	0.045	1
Inppl1	0.82722961	0.69678132	0.025	0.026	1
Apeh	0.82825752	0.3850797	0.038	0.037	1
Lig3	0.82961811	0.39738902	0.041	0.043	1
Sdccag3	0.82969249	0.39594474	0.078	0.082	1
Khsrp	0.82977325	0.3507834	0.125	0.133	1
Amd2	0.8300235	0.26690443	0.014	0.015	1
Dnajc13	0.83069288	0.28335515	0.114	0.12	1
Cxxc1	0.83078209	0.33399947	0.067	0.067	1
Rarres2	0.8307834	0.4404802	0.021	0.021	1
Las1l	0.8309057	0.27379773	0.107	0.111	1
Ncaph2	0.83121358	0.48382823	0.096	0.101	1
Btaf1	0.83144113	0.47115757	0.117	0.125	1
Faap24	0.83166563	0.36837206	0.018	0.018	1
Spast	0.83180705	0.38175019	0.067	0.067	1
Pcsk7	0.83184955	0.26430547	0.064	0.066	1
Map3k3	0.8327041	0.32831788	0.062	0.062	1
Wdr90	0.83367085	0.54881867	0.014	0.013	1
Zbtb4	0.83385325	0.28838549	0.045	0.045	1
Gna11	0.83458123	0.55731604	0.022	0.021	1
Usf1	0.83525542	0.42759606	0.057	0.059	1
Dgcr14	0.83543802	0.26128892	0.038	0.039	1
Fam98c	0.83717489	0.68984023	0.047	0.048	1
Dimt1	0.83735452	0.37946495	0.022	0.023	1
Tnpo1	0.83751193	0.41644673	0.169	0.178	1
Dhx8	0.83766579	0.33330691	0.132	0.136	1
Pus10	0.83806101	0.27523955	0.019	0.02	1
Gon7	0.83862335	0.55156053	0.05	0.05	1
Tmem183a	0.83900308	0.39756743	0.101	0.106	1
Uhmk1	0.83991716	0.35599016	0.14	0.145	1
Ngly1	0.83994712	0.36663785	0.053	0.055	1
Gm29361	0.84004661	0.59310482	0.012	0.011	1
Zfp609	0.84026401	0.59769942	0.023	0.023	1
Rbm15b	0.84047372	0.29188003	0.042	0.043	1
Ptpn9	0.84231199	0.47660171	0.061	0.064	1
Yeats2	0.84279962	0.37600679	0.041	0.041	1
Gm34961	0.84298717	0.43094015	0.01	0.009	1
Trmo	0.84324519	0.32156973	0.015	0.016	1

Ctbp1	0.84329235	0.30408099	0.186	0.199	1
Itpa	0.84369444	0.33808603	0.049	0.049	1
Acbd4	0.84449643	0.46847914	0.012	0.012	1
Copb2	0.8445721	0.38461924	0.18	0.195	1
Diaph3	0.84559511	0.27967231	0.021	0.021	1
Atf7	0.84568182	0.33661211	0.07	0.073	1
Dstyk	0.84576975	0.5014707	0.03	0.03	1
Cdca7	0.84623096	0.322037	0.014	0.014	1
Rtel1	0.84709775	0.28319204	0.012	0.011	1
Dera	0.84729501	0.36221495	0.062	0.063	1
Atg2b	0.84733474	0.2848073	0.03	0.029	1
Aamp	0.84743489	0.31791882	0.264	0.29	1
Zfp628	0.84855221	0.40609751	0.026	0.025	1
Dnal4	0.84985791	0.6074193	0.01	0.011	1
Mre11a	0.85053351	0.40433384	0.037	0.039	1
Prps2	0.8511417	0.33793097	0.056	0.056	1
Ubqln4	0.85122498	0.49964844	0.03	0.03	1
Ddi2	0.85254589	0.38011831	0.073	0.076	1
Coro7	0.85265175	0.37592917	0.076	0.077	1
Ap2b1	0.8527362	0.30961321	0.191	0.206	1
Ubqln2	0.85277273	0.49774404	0.061	0.062	1
Wars	0.85279021	0.40140049	0.042	0.044	1
Psmd13	0.85282344	0.30207452	0.15	0.161	1
Yipf5	0.85283405	0.37845184	0.113	0.116	1
Exoc4	0.85379575	0.39920982	0.092	0.096	1
Hras	0.85400166	0.4134503	0.07	0.074	1
U2surp	0.85457536	0.26811996	0.248	0.271	1
Znhit3	0.85587714	0.3746851	0.032	0.033	1
Bop1	0.85654121	0.41949926	0.051	0.053	1
Tbcc	0.85658903	0.6223609	0.04	0.04	1
Mavs	0.85661626	0.33949096	0.011	0.012	1
Cryba4	0.8570919	0.25499185	0.012	0.012	1
Zfp933	0.85715076	0.48652021	0.011	0.011	1
Fam160b2	0.85727373	0.43831052	0.047	0.047	1
Zfat	0.85751845	0.65857886	0.011	0.011	1
Rhoc	0.85756117	0.50589739	0.08	0.081	1
Ints12	0.8576076	0.29608766	0.065	0.067	1
Nfyb	0.8577536	0.36150698	0.113	0.115	1
Taf3	0.85837156	0.2734406	0.105	0.11	1
Tomm40l	0.86076294	0.47899998	0.02	0.02	1
Dcaf4	0.86133602	0.40560201	0.015	0.015	1
Gm32036	0.86157455	0.38369808	0.017	0.016	1
Bod1	0.86293215	0.69975073	0.03	0.031	1
Usp20	0.86356033	0.30563865	0.013	0.012	1
Zmym4	0.86404775	0.30584382	0.049	0.051	1
Susd1	0.86420152	0.38055022	0.013	0.014	1
Ints6l	0.86515002	0.35191392	0.044	0.045	1
Ino80e	0.86583469	0.3252809	0.072	0.075	1

Rnf38	0.8671365	0.30300645	0.075	0.075	1
Gm26533	0.86733276	0.77716823	0.01	0.01	1
Dhx35	0.86741201	0.66144377	0.027	0.027	1
Gm26722	0.86767565	0.40066116	0.024	0.025	1
Rcn2	0.86771147	0.47950867	0.089	0.094	1
Coq3	0.86808017	0.54591297	0.012	0.012	1
Sdhaf2	0.86843601	0.37329382	0.081	0.082	1
Bcl2l2	0.86844301	0.58528772	0.013	0.014	1
Disp1	0.86863109	0.45076135	0.012	0.011	1
Atxn10	0.86922838	0.46639695	0.096	0.101	1
Ppil4	0.86923802	0.32395175	0.152	0.158	1
2700062C07R	0.87008408	0.63348106	0.028	0.029	1
Ttl	0.87153851	1.06358431	0.011	0.01	1
Zfp426	0.8721449	0.28548718	0.019	0.018	1
Dpm2	0.87274138	0.4339792	0.093	0.096	1
Pole3	0.87294566	0.40701999	0.066	0.068	1
Cdk19	0.87366526	0.36217561	0.078	0.082	1
Inafm2	0.87405936	0.29948182	0.02	0.021	1
Gorasp2	0.87477701	0.47528528	0.126	0.135	1
Dnpep	0.87552167	0.52312072	0.053	0.054	1
Adck1	0.87584521	0.30539121	0.018	0.019	1
Fbxo38	0.8762212	0.30107211	0.079	0.08	1
Polr2c	0.87679602	0.33760002	0.155	0.162	1
Hk1	0.87718299	0.37039523	0.073	0.073	1
Msto1	0.87732283	0.33346361	0.017	0.016	1
Haghl	0.87817075	0.41394484	0.02	0.021	1
Cul2	0.87834725	0.42842384	0.075	0.076	1
Riox1	0.87864856	0.41009133	0.025	0.025	1
Cactin	0.87865868	0.33257601	0.034	0.034	1
Actr5	0.87915781	0.47325927	0.047	0.047	1
Gm47662	0.87929387	0.35845289	0.019	0.018	1
Espn	0.87954382	0.49055272	0.023	0.023	1
Drg2	0.87976501	0.55001048	0.029	0.03	1
Zdhhc6	0.87976528	0.45631871	0.041	0.042	1
Zfp866	0.87977942	0.42652164	0.025	0.024	1
Kpna2	0.88078313	0.52912714	0.016	0.017	1
Rnf14	0.88130306	0.35131909	0.067	0.067	1
Dffa	0.88168493	0.52672977	0.019	0.019	1
Mkl2	0.88205565	0.61504433	0.019	0.019	1
Coa3	0.88247479	0.33777686	0.121	0.124	1
Pias2	0.88257859	0.37890283	0.049	0.049	1
Man1a2	0.88275656	0.31736144	0.14	0.145	1
Tmc6	0.88325822	0.4172307	0.056	0.056	1
Fance	0.88357576	0.50539608	0.011	0.012	1
Nt5dc1	0.88450454	0.32695462	0.038	0.039	1
Eaf1	0.8846324	0.53498601	0.085	0.087	1
Fxn	0.8861864	0.53221603	0.036	0.036	1
Nelfa	0.88761389	0.46879898	0.046	0.046	1

Ndc1	0.88829236	0.56206299	0.014	0.014	1
Rufy2	0.89061355	0.25507672	0.054	0.054	1
Haus6	0.89093247	0.36237429	0.036	0.036	1
Egln2	0.89149767	0.37791756	0.092	0.094	1
Cog1	0.89200603	0.60697703	0.031	0.031	1
Papd5	0.89237853	0.369709	0.08	0.081	1
Nup54	0.89330816	0.3503414	0.086	0.09	1
Agap3	0.89374853	0.41279701	0.039	0.04	1
Gtf2e1	0.89399291	0.36095831	0.023	0.024	1
Dpf2	0.89412811	0.33255995	0.122	0.126	1
Slc7a6	0.89451852	0.40657499	0.019	0.02	1
Trp53rka	0.89522528	0.4700579	0.032	0.033	1
Hist2h4	0.89523259	0.31692279	0.016	0.016	1
Polr1b	0.89526417	0.39442619	0.011	0.012	1
Clspn	0.89553001	0.3887575	0.021	0.021	1
Lzic	0.89658143	0.31486591	0.043	0.044	1
Utp6	0.89670023	0.57764819	0.055	0.058	1
Smg8	0.89716709	0.56518609	0.028	0.029	1
Gars	0.89732339	0.42165176	0.132	0.138	1
Cherp	0.89747373	0.38338935	0.069	0.07	1
Rusc1	0.89783713	0.47118801	0.057	0.058	1
Rnaseh1	0.89886612	0.47898528	0.025	0.026	1
Otud3	0.89942705	0.46103249	0.015	0.015	1
Sp2	0.89970951	0.3841711	0.078	0.08	1
Ppp2r5e	0.90055393	0.30923389	0.093	0.095	1
Wdr6	0.90210999	0.26388497	0.015	0.016	1
Micu3	0.90241884	0.28925229	0.025	0.026	1
Pick1	0.90255211	0.36074404	0.018	0.018	1
Lancl2	0.90376178	0.60940164	0.021	0.021	1
Mrpl13	0.90390446	0.39308736	0.132	0.139	1
Pcnt	0.90417785	0.34043945	0.102	0.106	1
Ptgfrn	0.90442404	0.4356822	0.01	0.01	1
Xpnpep3	0.90489807	0.62063276	0.017	0.017	1
Eci1	0.90613114	0.44909181	0.043	0.043	1
Chchd7	0.90727202	0.39970847	0.086	0.09	1
Thumpd2	0.90766544	0.32539917	0.01	0.009	1
D1Ertd622e	0.90817148	0.27029198	0.078	0.079	1
Dynlt1f	0.90858356	0.68418016	0.029	0.029	1
Fundc2	0.90868741	0.2928253	0.204	0.216	1
Med7	0.90872568	0.350027	0.081	0.084	1
Zfp62	0.90902927	0.28411262	0.144	0.149	1
Cog3	0.9092689	0.38534893	0.041	0.041	1
Scfd2	0.91025046	0.28290856	0.022	0.022	1
Ift20	0.91040053	0.33160166	0.154	0.161	1
Tecr	0.91098683	0.3713405	0.164	0.172	1
Ssbp4	0.91169712	0.46916379	0.11	0.114	1
Dpm3	0.91210509	0.2874499	0.233	0.252	1
Kti12	0.9123382	0.47844753	0.031	0.032	1

Lias	0.91300612	0.42482408	0.061	0.061	1
Bud13	0.91318781	0.43662087	0.038	0.039	1
Rmi1	0.91344584	0.41347368	0.05	0.051	1
Elp3	0.91344773	0.37579341	0.02	0.02	1
Yy1	0.91346996	0.28413202	0.296	0.324	1
Ficd	0.9144092	0.51538096	0.012	0.012	1
Chuk	0.9145245	0.41022601	0.124	0.131	1
Zfp719	0.91605108	0.36823732	0.015	0.015	1
Blzf1	0.91649972	0.36909138	0.041	0.041	1
Cdc16	0.91708301	0.37996713	0.035	0.036	1
Dcun1d5	0.91751236	0.29944945	0.258	0.281	1
Ap1g2	0.91759575	0.42878783	0.035	0.036	1
Polr3g	0.91773583	0.35561485	0.011	0.011	1
Cables2	0.91838013	0.2794387	0.031	0.031	1
Rbm4b	0.91858129	0.33621956	0.078	0.079	1
Trappc5	0.91912098	0.33576714	0.103	0.106	1
Tbcd	0.91999071	0.44146458	0.031	0.032	1
Tomm70a	0.92183566	0.36604598	0.153	0.162	1
Coil	0.92203941	0.49688511	0.018	0.019	1
Rchy1	0.92324231	0.32729735	0.185	0.196	1
Cfap36	0.92443049	0.30775521	0.086	0.089	1
Smg5	0.92498379	0.37536055	0.101	0.104	1
Usp21	0.92502796	0.39721318	0.02	0.02	1
Inpp5f	0.92613055	0.3584538	0.034	0.034	1
Dync2h1	0.92725435	0.34779561	0.011	0.011	1
1700037H04R	0.92751995	0.50457575	0.028	0.028	1
Cox15	0.92916521	0.73222231	0.018	0.018	1
Trpv2	0.92938435	0.37078003	0.074	0.076	1
Ankle2	0.92979502	0.30455115	0.068	0.069	1
Tmem164	0.93040518	0.43578923	0.037	0.037	1
Kpna6	0.93048699	0.50443643	0.05	0.05	1
Zfp280b	0.93254023	0.38254076	0.02	0.02	1
Cpsf4	0.93346375	0.4758903	0.035	0.035	1
Stxbp1	0.93372476	0.38168723	0.017	0.017	1
Uspl1	0.93384201	0.30256533	0.088	0.09	1
Tbc1d31	0.93389351	0.49026845	0.02	0.02	1
Ttf2	0.93416324	0.48326452	0.022	0.022	1
Slc35c1	0.93431861	0.35802956	0.015	0.015	1
Kat5	0.93538602	0.37608621	0.055	0.056	1
Dnajc5	0.9354855	0.25023543	0.233	0.252	1
Exosc3	0.93572003	0.34157076	0.107	0.11	1
Rps6ka4	0.93721946	0.58178476	0.044	0.044	1
Nt5c3	0.93736521	0.3718312	0.06	0.061	1
Zbtb5	0.93811519	0.38118867	0.027	0.028	1
Mpp5	0.93829202	0.489399	0.064	0.066	1
Ech1	0.93871178	0.38203418	0.1	0.103	1
Polr3a	0.93952044	0.3926422	0.038	0.038	1
Gstp1	0.93964198	0.59567136	0.022	0.022	1

Larp7	0.93968698	0.36179268	0.156	0.165	1
Rnf40	0.94091233	0.37986823	0.06	0.06	1
Cbll1	0.94102905	0.26768201	0.11	0.114	1
Zfp597	0.94201021	0.29253599	0.027	0.027	1
Aimp2	0.94244475	0.33519831	0.029	0.029	1
Ada	0.94250726	0.51831061	0.012	0.013	1
Rit1	0.94253777	0.46047675	0.042	0.042	1
Cabin1	0.94299116	0.30877165	0.096	0.099	1
Pygb	0.94336147	0.35685549	0.034	0.034	1
Rab22a	0.94387943	0.43667058	0.126	0.133	1
Cep250	0.94493403	0.36485182	0.05	0.051	1
Dnajc14	0.94624275	0.38898213	0.069	0.071	1
Zfpl1	0.94724181	0.58286861	0.036	0.036	1
Kctd5	0.94763511	0.60240759	0.042	0.042	1
Camsap1	0.94829174	0.60082169	0.028	0.028	1
Sfrp1	0.95010825	0.43994547	0.011	0.011	1
Ctu2	0.95120768	0.41464777	0.042	0.042	1
Mvb12b	0.95145803	0.46390901	0.056	0.058	1
Vamp3	0.95148463	0.3996353	0.12	0.127	1
Serhl	0.95152838	0.30824373	0.031	0.031	1
Cklf	0.9526583	0.26569851	0.114	0.117	1
Trim13	0.95421438	0.67574227	0.014	0.014	1
Sertad3	0.95511755	0.29941093	0.023	0.024	1
Tarbp2	0.9552591	0.27811528	0.043	0.043	1
Nudt1	0.95553558	0.39635185	0.012	0.012	1
Rfwd3	0.95587583	0.32459884	0.037	0.037	1
Gabpb2	0.95656684	0.27779117	0.118	0.123	1
Lcor	0.9566536	0.37093616	0.054	0.055	1
Armc1	0.95785619	0.39027223	0.072	0.073	1
Cops2	0.95833777	0.34940876	0.145	0.152	1
Per1	0.95852454	0.25261033	0.213	0.228	1
Glg1	0.95870644	0.3398199	0.184	0.196	1
Ndfip2	0.95949616	0.31282194	0.17	0.181	1
Mid1ip1	0.95970141	0.5001804	0.04	0.041	1
Nudt16l1	0.96020098	0.41512596	0.046	0.047	1
Dcaf12	0.96043217	0.27166429	0.124	0.129	1
Setd6	0.96180396	0.30028118	0.015	0.015	1
Pigv	0.96336077	0.32972773	0.016	0.016	1
Wwc1	0.96397013	0.4326626	0.013	0.013	1
Ldah	0.96411693	0.28702157	0.046	0.047	1
Zfp369	0.96415067	0.36948028	0.026	0.026	1
Esf1	0.96434358	0.33022447	0.138	0.144	1
Exoc1	0.96472987	0.43990885	0.058	0.059	1
Telo2	0.96493184	0.47580638	0.013	0.014	1
Aagab	0.96515296	0.34003763	0.067	0.068	1
Hist4h4	0.96626846	0.37669923	0.018	0.018	1
Ykt6	0.96631878	0.48267587	0.098	0.101	1
Capn7	0.96631885	0.35611562	0.08	0.082	1

Rnf10	0.96639439	0.33100197	0.205	0.22	1
Hist2h2aa1	0.96651823	0.43006832	0.035	0.036	1
Sbno2	0.96665993	0.37320475	0.229	0.249	1
2310057M21F	0.96702509	0.25184699	0.025	0.025	1
Fadd	0.96710825	0.66263854	0.01	0.01	1
Dusp12	0.9686052	0.4741047	0.028	0.029	1
Atxn3	0.96948535	0.49412724	0.044	0.045	1
Dopey2	0.96984716	0.49782974	0.044	0.045	1
Hint3	0.96994606	0.38318788	0.108	0.112	1
Slc6a8	0.97016324	0.77754419	0.01	0.01	1
Bag3	0.97286154	0.31435273	0.053	0.055	1
Fdft1	0.97291137	0.53620523	0.032	0.033	1
Ubp1	0.9738203	0.34190165	0.113	0.117	1
Rer1	0.97401558	0.35864799	0.196	0.212	1
Gtf3c4	0.97424073	0.43657253	0.019	0.019	1
Mpv17l	0.97590309	0.56199653	0.027	0.027	1
Zbtb48	0.97598681	0.60428128	0.011	0.011	1
Dolpp1	0.97608237	0.38177562	0.011	0.011	1
Slc25a38	0.97676742	0.47676507	0.03	0.03	1
Zgpat	0.97712602	0.4462296	0.039	0.04	1
Rbm48	0.97713118	0.34186951	0.059	0.06	1
Ddx19b	0.97858331	0.40626217	0.035	0.035	1
Irf9	0.98002949	0.34783779	0.138	0.144	1
Sap30bp	0.98061632	0.55506831	0.075	0.078	1
Mrgbp	0.98128216	0.31731885	0.036	0.037	1
Gtf2h2	0.98219475	0.28664523	0.022	0.022	1
Zc3h11a	0.98276822	0.52437287	0.02	0.021	1
AC129328.1	0.98323699	0.60181529	0.011	0.011	1
Zfp607a	0.98335867	0.38928032	0.016	0.016	1
Papolg	0.98377926	0.31828437	0.05	0.051	1
Med31	0.98390769	0.32900302	0.081	0.083	1
Zfp692	0.98425418	0.56140551	0.014	0.014	1
Eif2b5	0.98432335	0.58693524	0.055	0.057	1
Wdr13	0.98557015	0.31053509	0.024	0.024	1
Map3k10	0.98640278	0.28754792	0.013	0.013	1
Osbpl8	0.98656331	0.2750876	0.231	0.246	1
Snip1	0.98670167	0.44476856	0.072	0.074	1
Kdm5d	0.98721925	0.57678169	0.027	0.028	1
Gm26518	0.98807735	0.27749668	0.066	0.067	1
Ppil2	0.98853101	0.39239452	0.091	0.093	1
Nup85	0.98891639	0.64814941	0.03	0.03	1
1700123O20R	0.98900738	0.29869987	0.09	0.092	1
Ccp110	0.98955155	0.26502312	0.015	0.016	1
Phkg2	0.98988973	0.46634715	0.076	0.078	1
Ahcyl1	0.99087835	0.29912893	0.115	0.119	1
Ppp1r12b	0.99105153	0.42184922	0.015	0.016	1
Fam76b	0.9911728	0.31332738	0.118	0.123	1
Grhpr	0.99121237	0.37079975	0.021	0.021	1

Styx	0.99164054	0.59864474	0.029	0.029	1
Ints2	0.99308856	0.30398127	0.019	0.019	1
Triobp	0.99403593	0.37191751	0.052	0.053	1
Caap1	0.99469115	0.25107514	0.057	0.057	1
Snapc5	0.99651405	0.43128297	0.115	0.12	1
Slc15a2	0.99752149	0.27186839	0.024	0.024	1
Sco1	0.99779373	0.52704427	0.02	0.02	1
Bmp4	0.9978923	0.41084239	0.011	0.011	1
Snapc2	0.99865403	0.52975415	0.022	0.023	1
Polr3b	0.99999785	0.49661626	0.028	0.028	1

CD4 Trm

Gene	p_val	avg_log2FC	pct.1	pct.2	p_val_adj
Stmn1	0	5.45571865	0.919	0.061	0
Pclaf	0	6.01632606	0.816	0.021	0
Top2a	0	4.86395006	0.866	0.082	0
Mki67	0	4.65423157	0.844	0.087	0
Hist1h1b	0	5.80168164	0.782	0.05	0
Dut	0	3.44391402	0.75	0.103	0
Hmgn2	0	3.00607251	0.796	0.15	0
H2afv	0	3.03580996	0.923	0.279	0
Hist1h2ae	0	5.36261866	0.662	0.034	0
Smc2	0	4.31201472	0.665	0.04	0
Cks1b	0	4.75578429	0.658	0.034	0
Hist1h3c	0	6.62629648	0.63	0.01	0
Birc5	0	6.04529148	0.63	0.013	0
Ube2c	0	5.3730362	0.61	0.023	0
Nusap1	0	6.03489463	0.594	0.011	0
Tuba1b	0	3.09359305	0.823	0.245	0
Hist1h2ap	0	5.1242954	0.583	0.023	0
Selenoh	0	2.60896358	0.698	0.139	0
Cdca8	0	5.6648216	0.572	0.014	0
Tubb5	0	3.1370201	0.932	0.38	0
Gmnn	0	3.68220599	0.592	0.054	0
Hist1h1d	0	3.40269732	0.596	0.063	0
H2afx	0	3.63674992	0.603	0.071	0
Lig1	0	3.61970658	0.58	0.055	0
Ezh2	0	3.04228928	0.612	0.088	0
Kif11	0	5.51807877	0.535	0.013	0
Spc24	0	5.35442941	0.53	0.014	0
Lockd	0	3.97915149	0.551	0.036	0
Mcm3	0	3.25774024	0.553	0.065	0
Cenpw	0	3.02402497	0.537	0.06	0
Hist2h2ac	0	3.35593038	0.542	0.066	0
Hist1h3e	0	3.25578507	0.528	0.062	0
Ncapg2	0	3.77051738	0.506	0.046	0
Hells	0	3.00415192	0.515	0.059	0
Dnmt1	0	2.87101581	0.508	0.066	0
Ccdc34	0	3.61764414	0.481	0.041	0
Rrm1	0	3.04062146	0.478	0.054	0
Mcm7	0	3.24416441	0.463	0.044	0
Racgap1	0	4.06616332	0.44	0.024	0
Prc1	0	3.74795076	0.445	0.03	0
Cdca3	0	5.48868245	0.42	0.01	0
Plk4	0	2.88991105	0.46	0.053	0
Ccnb2	0	5.60890406	0.411	0.008	0
Hist1h2ab	0	5.91990033	0.408	0.006	0
Ncapd3	0	2.97141439	0.454	0.053	0
Mcm5	0	3.36543478	0.428	0.035	0

Ccna2	0	6.37078397	0.397	0.005	0
Clspn	0	4.65610855	0.406	0.016	0
Ncapd2	0	4.09465135	0.406	0.02	0
Diaph3	0	4.41373536	0.394	0.017	0
Cenpf	0	5.3032912	0.388	0.012	0
Incenp	0	3.57747693	0.406	0.034	0
Kif15	0	5.36318519	0.372	0.009	0
Uhrf1	0	4.85449466	0.372	0.013	0
Knl1	0	5.0366344	0.367	0.01	0
Tpx2	0	5.05448968	0.361	0.01	0
Kif4	0	5.64644453	0.352	0.006	0
Hist1h2af	0	7.08130079	0.347	0.003	0
Cenpe	0	4.50125898	0.343	0.01	0
Cdk1	0	4.93256642	0.34	0.01	0
Tacc3	0	4.35469744	0.342	0.013	0
Arhgap11a	0	3.47079382	0.358	0.031	0
Hist2h2bb	0	3.94204091	0.347	0.021	0
Hist1h4h	0	3.23562734	0.358	0.033	0
Tyms	0	4.38799447	0.342	0.018	0
Dtl	0	3.86525848	0.336	0.021	0
Asf1b	0	4.93233072	0.324	0.009	0
Hist1h1a	0	4.80173865	0.32	0.011	0
Rrm2	0	5.13374011	0.311	0.008	0
Hist1h2bj	0	4.53410817	0.313	0.012	0
Hist1h2ak	0	4.80198203	0.308	0.009	0
Hist1h3i	0	4.22875772	0.313	0.016	0
Cit	0	5.12204864	0.299	0.007	0
Fbxo5	0	4.77701761	0.293	0.01	0
Tipin	0	3.19803003	0.306	0.027	0
Cenpm	0	4.98924146	0.288	0.009	0
Ncapg	0	5.08785304	0.261	0.006	0
Mad2l1	0	3.20706598	0.274	0.019	0
Hist1h2bb	0	5.14954245	0.256	0.006	0
Esco2	0	6.09355886	0.249	0.003	0
Hist1h2bn	0	5.45909108	0.25	0.005	0
Hmmr	0	5.96979326	0.245	0.003	0
Prim1	0	3.68756882	0.256	0.016	0
Tk1	0	4.53150477	0.249	0.01	0
Hist1h3f	0	3.47602333	0.256	0.019	0
Spc25	0	4.52306013	0.245	0.008	0
Cenpk	0	3.59452095	0.249	0.016	0
Aspm	0	5.40144163	0.229	0.004	0
Shcbp1	0	4.85903815	0.22	0.006	0
Bub1	0	6.07265797	0.215	0.003	0
Ska1	0	6.26014704	0.211	0.003	0
Rad51	0	4.82111178	0.213	0.006	0
Rad51ap1	0	5.4383601	0.211	0.004	0
Sgo1	0	5.6094086	0.199	0.003	0

Dlgap5	0	3.98037095	0.206	0.011	0
Cenpp	0	5.14117033	0.199	0.005	0
Spag5	0	5.13415418	0.195	0.004	0
Aurkb	0	5.22485743	0.195	0.005	0
Ndc80	0	3.54862009	0.2	0.012	0
Hist1h2bm	0	5.034461	0.19	0.004	0
Brca1	0	4.44167286	0.191	0.006	0
Pbk	0	6.07899077	0.184	0.002	0
Neil3	0	5.882448	0.184	0.003	0
Cep55	0	5.15860012	0.181	0.004	0
Foxm1	0	4.52052762	0.17	0.005	0
Melk	0	5.22256157	0.161	0.003	0
Cdc45	0	4.10691881	0.165	0.007	0
Depdc1a	0	4.94708934	0.161	0.004	0
Kif22	0	4.81092999	0.159	0.004	0
E2f8	0	4.37092877	0.156	0.007	0
E2f7	0	5.80382804	0.15	0.002	0
Pole	0	4.45612945	0.152	0.006	0
Cdca5	0	5.71267266	0.145	0.003	0
Stil	0	5.2250169	0.143	0.003	0
Cdc6	0	4.92432465	0.136	0.005	0
Sgo2a	0	4.43895273	0.131	0.004	0
Ect2	0	4.36446994	0.132	0.005	0
Brip1	0	4.50175169	0.131	0.005	0
Cenph	0	4.33817959	0.131	0.005	0
Cdkn3	0	4.62650457	0.129	0.005	0
Nuf2	0	5.94292167	0.125	0.002	0
Fignl1	0	4.58136394	0.12	0.004	0
Mybl2	0	4.8484255	0.111	0.003	0
Cdca2	0	4.6175747	0.109	0.003	0
Hist1h3b	0	6.3574959	0.088	0.001	0
Ttk	0	5.5850131	0.086	0.002	0
Knstrn	2.219E-306	3.90553824	0.195	0.012	4.869E-302
Chaf1a	1.147E-305	3.97068306	0.168	0.008	2.516E-301
Bard1	8.743E-304	4.00825797	0.156	0.007	1.918E-299
Hmgb2	7.236E-299	2.72166931	1	0.719	1.587E-294
Bub1b	5.727E-293	3.87925473	0.195	0.012	1.257E-288
Ncaph	1.05E-291	3.4715439	0.213	0.014	2.304E-287
Hist1h3g	1.33E-286	5.92598647	0.054	0.001	2.919E-282
Ckap2l	5.314E-283	3.78675362	0.177	0.01	1.166E-278
Hist1h4d	5.252E-281	3.03785597	0.773	0.219	1.152E-276
Ticrr	2.762E-280	4.1830552	0.12	0.005	6.06E-276
Hist1h2an	6.473E-280	4.44459852	0.102	0.003	1.42E-275
Cdkn2a	1.939E-278	3.99693685	0.136	0.006	4.255E-274
Pola1	7.931E-278	3.01168716	0.343	0.039	1.74E-273
Hjurp	2.895E-275	2.35641413	0.506	0.08	6.35E-271
Kntc1	1.071E-271	5.0954085	0.084	0.002	2.35E-267
Kif2c	5.428E-270	5.69127801	0.072	0.001	1.191E-265

Hist1h2bh	2.62E-267	4.55656472	0.106	0.004	5.747E-263
Anp32e	4.571E-266	2.26360007	0.891	0.296	1.003E-261
Ckap2	5.67E-264	3.83114352	0.138	0.007	1.244E-259
Pimreg	4.312E-259	5.59529673	0.055	0.001	9.46E-255
Cenpn	8.196E-259	4.31706893	0.098	0.003	1.798E-254
Mcm10	4.846E-258	4.07080147	0.095	0.003	1.063E-253
Tmpo	7.633E-257	2.43217546	0.698	0.167	1.675E-252
C330027C09F	1.233E-256	3.85591016	0.147	0.008	2.705E-252
Hist2h2aa1	8.85E-252	3.02002983	0.302	0.033	1.942E-247
Rad54l	8.064E-251	4.6103192	0.098	0.003	1.769E-246
Mcm6	1.424E-249	2.31918179	0.519	0.091	3.124E-245
Tcf19	8.765E-248	4.19468649	0.123	0.006	1.923E-243
Hist1h2ai	1.187E-247	5.04721338	0.066	0.001	2.604E-243
Mtfr2	1.986E-247	5.05547869	0.072	0.002	4.357E-243
Rfc4	8.803E-247	3.12284172	0.254	0.024	1.931E-242
Hmgb1	1.894E-246	2.01460956	0.986	0.661	4.155E-242
Cenpi	5.292E-240	4.48031542	0.089	0.003	1.161E-235
Mxd3	3.338E-238	5.29103898	0.061	0.001	7.324E-234
Rfc3	4.975E-234	2.6197779	0.318	0.039	1.091E-229
Mcm4	6.925E-234	2.32791252	0.51	0.093	1.519E-229
Kif23	7.114E-233	2.29583782	0.376	0.052	1.561E-228
Pmf1	7.563E-233	2.3331028	0.406	0.061	1.659E-228
Mis18bp1	7.843E-231	3.8574294	0.147	0.009	1.721E-226
E2f1	5.831E-229	3.57221539	0.195	0.016	1.279E-224
Ppia	3.089E-227	1.28581056	1	0.915	6.777E-223
Smc4	8.487E-227	2.0077392	0.71	0.18	1.862E-222
Orc6	1.782E-226	2.51909111	0.363	0.051	3.909E-222
Ska3	1.497E-215	4.13817225	0.095	0.004	3.283E-211
Lgals1	1.563E-213	1.92110332	0.97	0.51	3.43E-209
Ptma	5.787E-213	1.4917309	0.998	0.942	1.27E-208
Ube2s	3.68E-211	2.10122084	0.928	0.463	8.073E-207
Nsl1	2.062E-210	4.2526724	0.104	0.005	4.524E-206
Hist1h1e	2.204E-210	2.42716266	0.751	0.238	4.835E-206
Atad5	3.298E-210	3.01666742	0.249	0.027	7.235E-206
Cbx3	1.083E-209	1.920135	0.823	0.279	2.375E-205
Cbx5	2.382E-209	2.23304306	0.376	0.057	5.226E-205
Hist1h2be	2.249E-208	2.6471326	0.286	0.035	4.935E-204
Hmgb3	1.471E-207	2.81986093	0.229	0.023	3.228E-203
Tnfrsf4	1.807E-207	1.54485945	0.691	0.167	3.964E-203
Chek1	4.242E-207	4.03804852	0.1	0.004	9.306E-203
Mns1	1.984E-206	2.76332115	0.204	0.018	4.352E-202
Cdc20	4.489E-205	3.99364379	0.111	0.005	9.847E-201
Mms22l	2.374E-204	2.90313064	0.222	0.022	5.209E-200
Actn2	2.661E-204	1.96370676	0.585	0.13	5.837E-200
Plk1	2.599E-200	4.63133212	0.077	0.003	5.703E-196
Rbl1	5.272E-200	2.68248669	0.258	0.03	1.157E-195
Dhfr	9.371E-200	3.20968781	0.174	0.014	2.056E-195
Ccnb1	4.016E-197	3.94120083	0.091	0.004	8.81E-193

Wdr76	6.3E-197	3.03906897	0.191	0.017	1.382E-192
Ermn	7.145E-197	1.94282862	0.406	0.068	1.567E-192
Anp32b	9.973E-197	1.88170228	0.921	0.456	2.188E-192
Cks2	4.217E-196	1.69613295	0.631	0.156	9.251E-192
Dek	1.63E-193	1.9112054	0.934	0.486	3.576E-189
Fam83d	4.487E-193	3.54250256	0.109	0.006	9.844E-189
Zfp367	1.874E-192	2.97155585	0.209	0.021	4.11E-188
Trip13	3.714E-192	3.81364255	0.104	0.005	8.149E-188
Hint1	4.514E-190	1.58218444	0.957	0.572	9.904E-186
Mastl	1.145E-187	3.52466321	0.107	0.006	2.513E-183
Lsm2	2.138E-185	2.11332302	0.447	0.087	4.69E-181
Alyref	4.826E-184	1.90685386	0.708	0.217	1.059E-179
Trac	3.554E-183	1.5633781	0.778	0.228	7.797E-179
H2afz	2.938E-182	1.38987119	0.991	0.724	6.445E-178
Mrpl18	3.534E-181	1.89998865	0.619	0.162	7.754E-177
Ube2t	6.558E-181	2.66585604	0.204	0.021	1.439E-176
Hirip3	1.203E-180	2.58910796	0.249	0.03	2.64E-176
Nrm	2.742E-179	2.35825021	0.297	0.043	6.016E-175
Hist1h3a	3.781E-179	2.68671289	0.227	0.026	8.295E-175
Hist1h2ag	1.07E-177	5.07598881	0.05	0.001	2.347E-173
Blm	1.749E-177	2.40926843	0.272	0.036	3.837E-173
Topbp1	7.165E-177	1.96283837	0.451	0.09	1.572E-172
Haus4	1.521E-176	2.77466287	0.202	0.021	3.338E-172
1700097N02R	1.804E-176	3.27577529	0.157	0.013	3.958E-172
Tesc	1.129E-173	1.88381009	0.506	0.11	2.477E-169
Nek2	1.668E-173	3.95142635	0.077	0.003	3.66E-169
Atad2	1.752E-173	1.96735098	0.63	0.17	3.845E-169
Smc1a	4.648E-173	1.82677056	0.732	0.238	1.02E-168
Ran	1.387E-170	1.6811361	0.887	0.422	3.042E-166
Rbm3	1.515E-170	1.20501551	0.991	0.788	3.325E-166
Zwilch	2.521E-170	3.72273762	0.089	0.004	5.53E-166
Lat	2.748E-170	1.49686335	0.755	0.225	6.029E-166
Cd5	7.731E-170	1.63802228	0.565	0.134	1.696E-165
Ctla4	1.436E-168	1.31439213	0.841	0.287	3.15E-164
Pcna	2.385E-168	2.10884818	0.521	0.127	5.233E-164
Oaz1	2.518E-167	1.08468357	0.998	0.831	5.523E-163
BC030867	1.353E-166	4.98704544	0.052	0.001	2.968E-162
Mcm2	4.809E-165	2.35357221	0.259	0.035	1.055E-160
Nsd2	8.575E-165	2.11484402	0.49	0.114	1.881E-160
Anln	9.374E-165	3.98305073	0.084	0.004	2.057E-160
Ulbp1	6.332E-163	3.43381466	0.122	0.008	1.389E-158
Cd3g	1.533E-162	1.29868834	0.893	0.312	3.363E-158
Cdc25b	4.122E-162	2.59754046	0.211	0.024	9.044E-158
Lsm5	1.723E-161	1.70422938	0.792	0.295	3.781E-157
Ska2	2.397E-161	3.42502716	0.102	0.006	5.259E-157
Kifc1	8.094E-161	4.33400244	0.064	0.002	1.776E-156
Hist1h2bk	1.669E-160	5.07818349	0.039	0.001	3.661E-156
Hist1h4i	5.053E-160	2.11444675	0.376	0.071	1.109E-155

Trbc2	2.643E-159	1.35631064	0.939	0.383	5.799E-155
Nucks1	3.977E-159	1.58280138	0.902	0.429	8.726E-155
Rpa3	5.982E-159	2.24189592	0.313	0.051	1.312E-154
Aunip	8.825E-159	6.03024553	0.032	0	1.936E-154
Kif18b	1.931E-158	4.95194765	0.05	0.001	4.237E-154
Banf1	8.476E-158	1.75325286	0.687	0.22	1.859E-153
Rfc5	2.072E-157	2.73445733	0.2	0.023	4.545E-153
Cd3d	3.398E-157	1.27600296	0.894	0.312	7.454E-153
AW112010	2.046E-155	1.53761028	0.937	0.462	4.489E-151
Dnajc9	3.385E-155	1.86459491	0.483	0.112	7.426E-151
E2f2	2.394E-154	2.97223588	0.172	0.017	5.253E-150
Kif20b	7.421E-153	2.51568995	0.184	0.02	1.628E-148
Siva1	5.793E-152	2.01880154	0.526	0.14	1.271E-147
Usp1	6.431E-152	1.82642374	0.499	0.121	1.411E-147
Elob	1.053E-151	1.3524421	0.957	0.576	2.311E-147
Prr11	8.866E-151	4.43257626	0.063	0.002	1.945E-146
Thy1	6.689E-149	1.27854082	0.766	0.242	1.467E-144
Rnaseh2b	1.464E-148	2.46961228	0.243	0.035	3.213E-144
Cdca7	1.757E-147	3.06768792	0.145	0.013	3.854E-143
Gm19585	2.211E-147	1.57195087	0.547	0.139	4.852E-143
Rangap1	2.34E-147	1.75350578	0.465	0.108	5.133E-143
Rmi2	2.039E-146	3.20132985	0.123	0.01	4.473E-142
Zdhhc2	5.882E-146	2.12908035	0.265	0.04	1.29E-141
Gins2	3.456E-145	3.14216304	0.148	0.014	7.582E-141
Cd3e	3.994E-143	1.20310542	0.841	0.289	8.762E-139
Clic1	5.289E-142	1.45072196	0.9	0.44	1.16E-137
Hist1h4k	7.775E-141	4.06052055	0.061	0.002	1.706E-136
Rpa2	1.189E-138	1.7120622	0.34	0.064	2.609E-134
Hpf1	1.457E-138	1.65059269	0.606	0.185	3.196E-134
Exosc8	1.916E-138	1.72218332	0.453	0.108	4.203E-134
Iqgap3	1.834E-137	4.60185083	0.059	0.002	4.024E-133
Snrpe	2.161E-136	1.25522791	0.957	0.609	4.74E-132
Gapdh	6.273E-135	1.05707935	0.995	0.743	1.376E-130
Cmc2	2.661E-133	2.02237843	0.358	0.075	5.837E-129
Hist3h2ba	3.07E-133	2.59438806	0.172	0.02	6.736E-129
Smc3	2.353E-132	1.53894787	0.664	0.215	5.163E-128
Hist1h4f	4.548E-132	4.43323605	0.05	0.002	9.977E-128
Phgdh	9.262E-132	1.99084316	0.292	0.052	2.032E-127
Fen1	2.37E-131	2.11452421	0.24	0.037	5.199E-127
Cd40lg	3.523E-131	1.51216831	0.417	0.094	7.73E-127
Dctpp1	1.237E-130	2.40018146	0.306	0.059	2.714E-126
Ncaph2	1.33E-130	1.72522781	0.417	0.098	2.919E-126
Calm3	1.958E-130	1.5348965	0.778	0.327	4.295E-126
Dbf4	3.05E-130	1.72498724	0.417	0.097	6.692E-126
Dscc1	3.73E-129	4.26863132	0.063	0.003	8.183E-125
Tmsb10	4.199E-129	0.9524962	1	0.892	9.213E-125
Timeless	8.103E-129	3.09097588	0.136	0.013	1.778E-124
Kif18a	2.175E-128	2.62537347	0.136	0.013	4.771E-124

Bik	6.286E-128	2.51428296	0.163	0.018	1.379E-123
Pfn1	7.566E-128	1.13492273	0.987	0.752	1.66E-123
Rad21	1.212E-127	1.51596992	0.712	0.261	2.659E-123
Tfdp1	1.674E-127	2.56094194	0.204	0.028	3.673E-123
Hmgn5	2.429E-127	1.56867481	0.453	0.112	5.328E-123
Txn1	7.443E-127	1.11770533	0.959	0.553	1.633E-122
Ppp1ca	1.557E-126	1.37264883	0.866	0.432	3.417E-122
Fam92a	1.749E-126	2.09399058	0.247	0.04	3.837E-122
Cd28	1.791E-126	1.109153	0.826	0.307	3.93E-122
S100a4	2.785E-126	1.33498278	0.828	0.355	6.111E-122
Orc1	4.112E-125	3.66001358	0.068	0.003	9.022E-121
Tubb4b	1.094E-124	1.695127	0.483	0.132	2.399E-120
Pkmyt1	4.434E-124	2.83037055	0.125	0.012	9.728E-120
Kif14	1.579E-123	4.72336879	0.036	0.001	3.465E-119
Anapc5	4.771E-123	1.70577876	0.483	0.134	1.047E-118
Lsm3	1.118E-121	1.61206049	0.528	0.155	2.454E-117
Icos	2.454E-121	1.00692352	0.88	0.353	5.383E-117
Sumo2	3.084E-121	1.27561919	0.916	0.533	6.765E-117
Syce2	3.708E-121	2.64062232	0.191	0.027	8.135E-117
Lck	9.904E-121	1.24249493	0.639	0.197	2.173E-116
Haspin	6.372E-119	3.26351141	0.091	0.007	1.398E-114
Pask	1.396E-118	4.59439761	0.039	0.001	3.063E-114
Fancd2	2.149E-118	3.91195651	0.059	0.003	4.714E-114
Chtf18	4.584E-118	4.11914705	0.055	0.002	1.006E-113
Ccne2	2.456E-117	2.96818311	0.109	0.009	5.387E-113
Psip1	5E-116	1.40543525	0.504	0.142	1.097E-111
Cd6	1.423E-115	1.27694157	0.524	0.148	3.123E-111
S100a10	2.004E-115	1.1695381	0.95	0.555	4.397E-111
Skap1	6.808E-115	1.09563448	0.691	0.222	1.494E-110
Oip5	7.031E-115	4.06762086	0.048	0.002	1.543E-110
1500009L16Ri	1.327E-114	3.02148122	0.1	0.008	2.912E-110
Carnmt1	1.677E-114	1.39387763	0.497	0.138	3.68E-110
Sh2d2a	2.692E-114	0.99546033	0.81	0.297	5.906E-110
Rnaseh2c	3.267E-113	1.55814801	0.497	0.146	7.167E-109
Rfwd3	9.372E-113	2.15209057	0.22	0.036	2.056E-108
Ssrp1	2.646E-112	1.45853878	0.567	0.182	5.805E-108
Cd4	4.981E-112	1.14595335	0.54	0.154	1.093E-107
Krtcap2	5.661E-112	1.22726206	0.852	0.399	1.242E-107
Zgrf1	5.716E-112	2.8615498	0.116	0.011	1.254E-107
4930579G24R	1.287E-111	3.19491742	0.084	0.006	2.823E-107
Med30	1.585E-111	1.60635227	0.428	0.114	3.478E-107
Mthfd2	4.087E-111	1.42813506	0.594	0.199	8.966E-107
Tube1	1.205E-110	3.29072013	0.079	0.005	2.645E-106
Cxcr6	1.225E-110	1.01195084	0.726	0.256	2.687E-106
Ranbp1	1.742E-110	1.47298369	0.685	0.273	3.821E-106
Atp5o.1	1.031E-109	1.29841497	0.805	0.362	2.262E-105
Prelid1	5.018E-109	1.34920596	0.733	0.299	1.101E-104
Spdl1	8.924E-109	3.03110526	0.118	0.012	1.958E-104

Crip1	3.312E-108	1.03780205	0.977	0.649	7.266E-104
Slbp	3.762E-108	1.58640847	0.637	0.243	8.254E-104
Hist1h2bl	4.631E-108	4.36004642	0.041	0.001	1.016E-103
Jpt1	5.218E-108	1.23791547	0.825	0.382	1.145E-103
Tagln2	6.804E-108	1.18947739	0.918	0.516	1.493E-103
BC055324	2.721E-107	3.50168738	0.059	0.003	5.97E-103
Traip	3.701E-107	3.42500711	0.066	0.004	8.119E-103
Exo1	4.463E-107	4.09790744	0.039	0.001	9.791E-103
2610318N02R	7.021E-107	3.78838728	0.066	0.004	1.54E-102
Suv39h1	8.741E-107	2.29503495	0.17	0.023	1.918E-102
Sh3bgrl3	5.015E-106	1.01614738	0.984	0.709	1.1E-101
Nudt21	2.706E-105	1.42257034	0.596	0.211	5.936E-101
Themis	3.372E-105	1.42371299	0.395	0.098	7.398E-101
Ptprcap	4.089E-105	1.2291887	0.612	0.201	8.97E-101
Efcab11	9.871E-105	4.76778085	0.041	0.001	2.166E-100
Rfc1	2.075E-104	1.71174577	0.342	0.081	4.552E-100
Maz	4.229E-104	1.80308962	0.34	0.08	9.279E-100
Rad54b	1.03E-103	4.98703612	0.038	0.001	2.259E-99
Snrpd1	1.219E-103	1.30348844	0.76	0.333	2.674E-99
Nasp	2.06E-103	1.35163964	0.567	0.187	4.518E-99
Sf3b5	2.976E-103	1.21614065	0.751	0.314	6.53E-99
Sept1	1.052E-102	1.17457342	0.764	0.305	2.3091E-98
Ckap5	1.791E-102	1.71896474	0.349	0.085	3.93E-98
Depdc1b	3.694E-102	4.88464756	0.03	0.001	8.1049E-98
Lrr1	1.001E-101	5.24925714	0.029	0.001	2.1963E-97
1810037I17Ril	1.082E-101	1.22342489	0.855	0.456	2.3737E-97
Ndufa4	1.473E-101	1.22934303	0.859	0.465	3.2307E-97
Emp3	3.189E-101	1.26639275	0.801	0.367	6.9962E-97
Cox7a2	1.082E-100	1.03945724	0.941	0.579	2.3739E-96
Polq	1.671E-100	3.8193803	0.048	0.002	3.6658E-96
Kif20a	3.611E-100	3.98237137	0.055	0.003	7.9216E-96
Tnfrsf18	2.565E-99	1.00637945	0.694	0.253	5.627E-95
Hist1h4a	1.3147E-98	2.92665226	0.097	0.009	2.8842E-94
Gins1	4.06E-98	3.70325001	0.064	0.004	8.9071E-94
Reep5	9.1302E-98	1.18177956	0.819	0.401	2.0031E-93
Hist1h4c	7.6578E-97	1.8743988	0.25	0.05	1.68E-92
Arl6ip1	7.7976E-97	1.40950636	0.871	0.527	1.7107E-92
Lsm4	2.3394E-96	1.16180655	0.753	0.323	5.1325E-92
Arhgap33	3.4579E-96	4.09096998	0.038	0.001	7.5863E-92
Fancf	1.1358E-95	1.94264466	0.188	0.03	2.4918E-91
Mbnl3	1.5092E-95	1.71314976	0.229	0.043	3.311E-91
Hist1h2bf	3.7264E-95	3.41919454	0.061	0.004	8.1754E-91
Ssna1	3.4727E-94	1.62669657	0.335	0.083	7.6188E-90
Suz12	3.7973E-94	1.26030802	0.648	0.25	8.3308E-90
Snrnp25	4.5949E-94	1.82272481	0.265	0.056	1.0081E-89
Ltb	7.8666E-94	1.32505343	0.68	0.272	1.7258E-89
Mrpl33	8.3621E-94	0.9999129	0.86	0.435	1.8346E-89
Cenpq	3.2819E-93	1.33570222	0.343	0.085	7.2003E-89

Sae1	3.4261E-93	1.69385382	0.286	0.064	7.5165E-89
Atp5j	5.9584E-93	1.18594698	0.853	0.459	1.3072E-88
S100a13	8.274E-93	1.13310684	0.807	0.391	1.8152E-88
Nmrk1	3.1661E-92	1.45017987	0.419	0.121	6.9461E-88
Lsm6	4.3439E-92	1.33999928	0.567	0.206	9.53E-88
Rif1	4.6233E-92	1.21446086	0.449	0.131	1.0143E-87
Dtymk	1.0804E-91	1.88056375	0.261	0.056	2.3702E-87
Arhgap19	3.4067E-91	2.99744884	0.111	0.012	7.4741E-87
Atp5h	4.1829E-91	0.82689427	0.984	0.739	9.1768E-87
Cdc25c	1.1008E-90	4.59480044	0.032	0.001	2.4151E-86
Cox5b	1.1668E-90	1.00787893	0.925	0.562	2.5598E-86
Ppp1cc	2.4105E-90	0.9438979	0.843	0.392	5.2885E-86
Hist1h2bp	1.2815E-89	3.84698721	0.041	0.002	2.8114E-85
Pdap1	1.852E-89	1.19464776	0.674	0.278	4.0631E-85
Tpi1	1.9044E-89	1.2527564	0.624	0.249	4.178E-85
Pold2	3.4439E-89	2.27210685	0.154	0.023	7.5556E-85
Psmb3	5.7846E-89	1.23301543	0.732	0.335	1.2691E-84
Ldha	1.1952E-88	1.09701942	0.866	0.488	2.622E-84
Csrp1	1.5716E-88	1.44980467	0.431	0.133	3.4478E-84
Nap1l1	1.6963E-88	0.95815575	0.911	0.571	3.7216E-84
Ybx1	3.1693E-88	0.85119884	0.977	0.734	6.9531E-84
Espl1	3.6215E-88	3.34117764	0.082	0.007	7.9452E-84
Ctc1	3.9155E-88	2.15723127	0.15	0.022	8.5902E-84
Tedc1	4.5092E-88	3.12072411	0.063	0.004	9.8928E-84
Ankle1	1.1674E-87	5.37599674	0.021	0	2.5612E-83
Higd1a	6.9427E-87	1.14390438	0.71	0.307	1.5232E-82
Casp8ap2	7.6699E-87	1.26613259	0.462	0.146	1.6827E-82
Tubgcp2	9.7603E-87	2.10509118	0.209	0.04	2.1413E-82
Ndufv3	1.1743E-86	1.15408379	0.767	0.364	2.5764E-82
Idh3a	2.5032E-86	1.56715679	0.347	0.094	5.4917E-82
Nebl	9.4562E-86	1.54812054	0.301	0.072	2.0746E-81
Cox6c	9.9389E-86	0.86075443	0.964	0.704	2.1805E-81
Ctcf	1.1911E-85	1.07786045	0.68	0.275	2.6132E-81
Ankrd37	1.5393E-85	1.85389068	0.208	0.04	3.3771E-81
Zap70	2.3911E-85	1.1287951	0.449	0.134	5.2458E-81
Dnajc15	2.4701E-85	1.40989002	0.522	0.191	5.4191E-81
Trat1	4.5072E-85	1.44565872	0.326	0.084	9.8884E-81
Eme1	5.7447E-85	3.98254294	0.036	0.001	1.2603E-80
Cox5a	9.612E-85	1.06705609	0.88	0.51	2.1088E-80
Rnf125	1.2651E-84	0.91224378	0.592	0.203	2.7754E-80
Bora	1.7267E-84	2.1651938	0.14	0.02	3.7883E-80
Rwdd1	2.7075E-84	1.07007305	0.716	0.312	5.9399E-80
Sapcd2	2.8421E-84	4.65934254	0.032	0.001	6.2353E-80
Hdgf	4.5758E-84	1.19157741	0.626	0.253	1.0039E-79
Ndufa11	9.6703E-84	1.21408313	0.664	0.285	2.1216E-79
Nxt1	1.4371E-83	1.75832511	0.275	0.065	3.1528E-79
Poc1a	1.8491E-83	3.31266533	0.059	0.004	4.0568E-79
Uqcr10	2.1583E-83	1.04930791	0.846	0.454	4.735E-79

Rdm1	3.4652E-83	2.49372505	0.125	0.017	7.6024E-79
Cd247	5.0352E-83	1.0578529	0.431	0.127	1.1047E-78
Slc43a3	1.1341E-82	2.46595388	0.106	0.012	2.4882E-78
Ndufb11	1.5472E-82	0.99955344	0.855	0.451	3.3944E-78
Mrpl13	2.4153E-82	1.35561244	0.429	0.136	5.2988E-78
Ddx39	2.7477E-82	1.62236897	0.315	0.083	6.0282E-78
Rcc1	3.5749E-82	2.48387005	0.123	0.016	7.843E-78
Acot7	5.9932E-82	1.40858883	0.394	0.119	1.3148E-77
Pde4d	8.2728E-82	1.06959407	0.497	0.163	1.815E-77
D16Ertd472e	4.2456E-81	0.81498237	0.819	0.374	9.3144E-77
Cox20	5.4019E-81	1.28133941	0.453	0.149	1.1851E-76
Ndufb7	5.5237E-81	1.09013613	0.767	0.373	1.2118E-76
Snrpb	5.7532E-81	1.02295325	0.805	0.415	1.2622E-76
Ppih	9.4408E-81	2.13900014	0.174	0.03	2.0712E-76
Spata24	1.1508E-80	2.30786262	0.12	0.016	2.5248E-76
Baz1b	1.4773E-80	1.10837398	0.524	0.183	3.241E-76
Eno1	1.6256E-80	1.14992237	0.592	0.23	3.5664E-76
Ndufs5	3.8451E-80	1.14602145	0.624	0.255	8.4358E-76
Csnk2b	4.7513E-80	1.14218171	0.669	0.291	1.0424E-75
Nsmce4a	4.9741E-80	1.09394881	0.497	0.169	1.0913E-75
Cox8a	6.6258E-80	0.70901204	0.986	0.809	1.4536E-75
Mif	8.8375E-80	1.11196953	0.841	0.478	1.9389E-75
Cenpx	1.0513E-79	1.42113918	0.426	0.14	2.3064E-75
Hist1h2bg	1.1494E-79	2.70704362	0.079	0.007	2.5218E-75
Aes	1.3021E-79	1.01659439	0.805	0.403	2.8567E-75
Actb	2.0038E-79	0.68628043	1	0.977	4.3961E-75
Pgk1	3.4667E-79	1.04846754	0.725	0.336	7.6055E-75
Lmnb1	3.6417E-79	0.70066977	0.866	0.439	7.9896E-75
Ubb	1.4579E-78	0.8799672	0.987	0.82	3.1986E-74
Hist1h3d	1.5645E-78	2.71419968	0.081	0.008	3.4324E-74
Tshz1	1.7601E-78	1.07610509	0.426	0.13	3.8614E-74
Lbr	1.842E-78	1.0665112	0.617	0.244	4.0412E-74
Gna15	4.4578E-78	1.50425401	0.301	0.077	9.7801E-74
Mis18a	4.6912E-78	1.60611	0.243	0.055	1.0292E-73
Tubg1	5.7871E-78	2.58087148	0.088	0.009	1.2696E-73
Hprt	6.2926E-78	1.24492699	0.546	0.211	1.3805E-73
Kif2a	6.9904E-78	1.34536889	0.395	0.123	1.5336E-73
Adgrl1	1.1312E-77	1.53870512	0.202	0.04	2.4818E-73
Rbbp7	1.1631E-77	1.19556735	0.503	0.18	2.5517E-73
Il2rb	1.3602E-77	0.57746342	0.744	0.295	2.9842E-73
Rex1bd	1.3718E-77	1.30799669	0.476	0.168	3.0096E-73
Dbi	1.692E-77	1.07295395	0.692	0.313	3.7121E-73
Dsn1	2.7653E-77	2.8577786	0.082	0.008	6.0668E-73
Olfr1369-ps1	4.7103E-77	2.29925404	0.136	0.02	1.0334E-72
Nans	5.2015E-77	1.53629343	0.327	0.092	1.1412E-72
Hnrnpa3	5.4259E-77	0.83417078	0.968	0.744	1.1904E-72
Hist1h2ac	7.0923E-77	2.03382559	0.12	0.016	1.556E-72
Cisd1	1.4801E-76	1.58965352	0.301	0.08	3.2471E-72

Haus6	2.4677E-76	1.68221575	0.184	0.035	5.4139E-72
Snrpf	3.8292E-76	0.9184853	0.909	0.557	8.401E-72
Cdk4	6.5605E-76	1.53346614	0.309	0.085	1.4393E-71
Lsm8	7.3427E-76	1.12693159	0.496	0.174	1.6109E-71
Cdc7	8.1374E-76	2.06514409	0.123	0.017	1.7853E-71
Ccne1	9.0673E-76	3.32522447	0.054	0.004	1.9893E-71
Rac2	2.2669E-75	0.85244541	0.927	0.576	4.9733E-71
Ifngr1	3.9892E-75	0.7392174	0.973	0.619	8.7518E-71
Snrpg	7.9211E-75	0.84363444	0.927	0.584	1.7378E-70
Rpa1	8.062E-75	1.60012656	0.247	0.058	1.7687E-70
Bub3	1.0533E-74	1.24276652	0.449	0.154	2.3108E-70
Pcnt	1.2657E-74	1.28373251	0.352	0.103	2.7769E-70
Mrpl58	1.3049E-74	1.46203107	0.374	0.118	2.8629E-70
Actl6a	1.5332E-74	1.3431833	0.358	0.107	3.3637E-70
Arpp19	1.9539E-74	0.93391668	0.853	0.478	4.2866E-70
Atp5f1	2.181E-74	0.94722021	0.823	0.437	4.7849E-70
Atp5k	2.5162E-74	0.99427622	0.8	0.422	5.5202E-70
Dpy30	3.5237E-74	1.60050316	0.301	0.082	7.7307E-70
Rora	5.6873E-74	0.46113358	0.927	0.44	1.2477E-69
D030056L22R	9.0297E-74	1.77518787	0.218	0.047	1.981E-69
Ostf1	1.2313E-73	0.89266133	0.862	0.523	2.7014E-69
Dap	1.8142E-73	1.53209279	0.417	0.143	3.9802E-69
Cep128	3.4206E-73	2.33321882	0.116	0.016	7.5044E-69
Crmp1	4.0353E-73	2.08348353	0.145	0.024	8.8531E-69
Camk4	6.0129E-73	0.98427296	0.436	0.139	1.3192E-68
Cyb5a	2.5325E-72	1.19873538	0.522	0.204	5.5561E-68
Nfatc2ip	2.8939E-72	1.51263309	0.204	0.043	6.3488E-68
Raly	8.2414E-72	1.02613579	0.71	0.34	1.8081E-67
Ms4a4b	1.3455E-71	0.85472779	0.623	0.248	2.9519E-67
Psmb9	1.8184E-71	0.96182366	0.691	0.312	3.9894E-67
Ttf2	2.6717E-71	2.07232158	0.132	0.021	5.8615E-67
Paxip1	3.4451E-71	1.62935476	0.193	0.04	7.5582E-67
Pole3	5.1055E-71	1.50548569	0.261	0.066	1.1201E-66
Pold3	6.6138E-71	1.64765231	0.234	0.056	1.451E-66
Mlf1	6.6713E-71	2.59728728	0.07	0.006	1.4636E-66
Aebp2	7.0214E-71	0.7425817	0.919	0.523	1.5404E-66
Pa2g4	8.8711E-71	1.21807483	0.449	0.16	1.9462E-66
Mrpl57	1.0357E-70	1.06607467	0.549	0.216	2.2723E-66
Pbrm1	2.0399E-70	1.05781903	0.631	0.275	4.4754E-66
Uchl5	4.455E-70	1.33684714	0.304	0.085	9.7737E-66
Psmc3ip	4.5806E-70	2.84856027	0.068	0.006	1.0049E-65
Fkbp3	5.308E-70	0.98918959	0.614	0.257	1.1645E-65
Lin9	9.8896E-70	1.69009474	0.157	0.028	2.1697E-65
Erh	1.6718E-69	1.4540749	0.331	0.1	3.6678E-65
Ly6g5b	4.1209E-69	2.58897115	0.098	0.012	9.0408E-65
Sf3b2	1.1542E-68	0.95395137	0.767	0.401	2.5321E-64
Snrpd2	1.5153E-68	0.91888979	0.775	0.386	3.3244E-64
Rinl	1.8003E-68	0.93194248	0.479	0.167	3.9496E-64

Snrpc	1.9371E-68	1.15522389	0.553	0.228	4.2498E-64
Set	2.3088E-68	0.88643214	0.864	0.507	5.0652E-64
Spn	2.4954E-68	0.82739887	0.558	0.209	5.4746E-64
Pfdn6	4.8601E-68	1.29021158	0.428	0.152	1.0663E-63
Gins3	7.2187E-68	2.96843982	0.07	0.007	1.5837E-63
Actn4	7.9364E-68	0.96887048	0.594	0.248	1.7412E-63
Gpc1	1.0109E-67	1.80490124	0.19	0.04	2.2178E-63
Nt5c3b	1.5644E-67	1.79862085	0.17	0.033	3.4322E-63
Cbx1	2.6026E-67	1.0762588	0.447	0.159	5.7098E-63
Pkp3	4.668E-67	1.44347218	0.299	0.085	1.0241E-62
Ccdc124	5.1412E-67	1.15211979	0.42	0.146	1.1279E-62
Tfdp2	6.0716E-67	1.75569424	0.168	0.033	1.3321E-62
Slamf1	1.431E-66	0.89962827	0.435	0.145	3.1395E-62
Cenpa	1.4621E-66	1.09613109	0.746	0.391	3.2076E-62
Anapc11	2.5874E-66	1.27452816	0.381	0.128	5.6765E-62
Myl12a	3.658E-66	0.82400378	0.911	0.561	8.0252E-62
Vars	5.1687E-66	1.11905163	0.36	0.114	1.134E-61
Samd1	5.9544E-66	2.20326545	0.136	0.023	1.3063E-61
Gins4	6.5633E-66	1.60292611	0.215	0.05	1.4399E-61
Hif1a	7.9097E-66	0.74181217	0.921	0.569	1.7353E-61
Sec11c	8.2015E-66	0.96970358	0.744	0.392	1.7993E-61
H2afy	9.9822E-66	0.82796893	0.898	0.508	2.19E-61
Lmf2	1.1865E-65	1.69145963	0.184	0.039	2.603E-61
Leprotl1	1.2186E-65	0.86006958	0.689	0.314	2.6735E-61
Sass6	1.4759E-65	2.3014112	0.102	0.014	3.238E-61
Tfrc	1.5225E-65	1.14028512	0.327	0.098	3.3402E-61
Ndufb8	1.9472E-65	1.05334416	0.662	0.314	4.2721E-61
H2-Q7	2.0112E-65	0.7950437	0.937	0.623	4.4124E-61
Dynll2	3.2745E-65	1.25706379	0.383	0.129	7.1839E-61
Cbfb	4.7445E-65	1.14072575	0.39	0.132	1.0409E-60
Nelfe	5.0224E-65	1.69355245	0.252	0.068	1.1019E-60
Mier1	5.079E-65	0.8478994	0.791	0.404	1.1143E-60
Mrps18c	5.7169E-65	1.0974505	0.496	0.194	1.2542E-60
Pla2g16	5.7228E-65	1.05354823	0.488	0.185	1.2555E-60
Smdt1	5.854E-65	0.88840114	0.841	0.467	1.2843E-60
Emg1	6.5868E-65	0.91947024	0.664	0.301	1.4451E-60
Bcl11b	6.9707E-65	0.7251254	0.476	0.165	1.5293E-60
Rnps1	8.1376E-65	1.03518644	0.555	0.229	1.7853E-60
Helb	8.4982E-65	1.45606378	0.231	0.056	1.8644E-60
Ly6a	2.1482E-64	0.89962271	0.671	0.317	4.7129E-60
Ap3s1	2.2466E-64	0.9869611	0.526	0.21	4.9288E-60
Minos1	3.1882E-64	0.84625717	0.859	0.473	6.9945E-60
Cdca7l	1.0366E-63	2.27653556	0.122	0.019	2.2743E-59
Hnrnpul1	1.3936E-63	0.91261261	0.646	0.289	3.0574E-59
Tmem97	1.6586E-63	1.91258929	0.152	0.029	3.6388E-59
Rbbp4	2.1005E-63	0.96687867	0.603	0.263	4.6082E-59
Serbp1	2.4929E-63	0.6823627	0.964	0.747	5.4692E-59
Trp53i13	2.6456E-63	2.08578005	0.125	0.021	5.8042E-59

Mrps12	3.2896E-63	1.23882684	0.336	0.107	7.2171E-59
Nkg7	3.4185E-63	0.46106819	0.565	0.221	7.4998E-59
Anp32a	4.128E-63	0.89060832	0.76	0.398	9.0564E-59
Clgn	4.3782E-63	3.56404361	0.032	0.002	9.6053E-59
Dnajc2	6.1154E-63	1.02533436	0.469	0.178	1.3417E-58
Cenps	6.2094E-63	2.07675067	0.097	0.013	1.3623E-58
Nol7	8.0118E-63	0.86994057	0.787	0.42	1.7577E-58
Supt16	8.996E-63	0.98935152	0.522	0.211	1.9736E-58
Il18rap	9.4616E-63	0.76923168	0.47	0.168	2.0758E-58
Atxn1	1.4588E-62	0.78636253	0.517	0.192	3.2005E-58
Tnfsf11	1.5168E-62	0.75231812	0.351	0.109	3.3276E-58
Nfyb	1.5609E-62	1.06778347	0.352	0.112	3.4244E-58
Gm42047	2.0065E-62	2.84217918	0.075	0.008	4.402E-58
Pgp	2.7891E-62	1.75648371	0.215	0.053	6.119E-58
Metrn	5.5669E-62	2.05588598	0.148	0.028	1.2213E-57
Rdx	6.1261E-62	0.85371589	0.628	0.278	1.344E-57
Rpl28	6.3657E-62	0.49415739	0.996	0.946	1.3966E-57
Cd2	1.0632E-61	0.65459212	0.68	0.285	2.3326E-57
Cdk2ap2	1.3734E-61	0.61023316	0.789	0.403	3.0131E-57
Ndufa5	1.663E-61	0.98403665	0.528	0.218	3.6485E-57
Mybl1	2.0331E-61	2.61105274	0.055	0.005	4.4604E-57
Stard4	3.8261E-61	1.04649507	0.331	0.103	8.3941E-57
Il18r1	4.8951E-61	0.68422177	0.533	0.204	1.0739E-56
Cox7c	8.277E-61	0.61824701	0.993	0.822	1.8159E-56
Ap2s1	1.0041E-60	0.9997049	0.589	0.266	2.2028E-56
Dgcr8	1.4167E-60	1.98406418	0.122	0.02	3.1081E-56
Ndufb6	1.677E-60	1.03678927	0.528	0.221	3.6792E-56
Magoh	1.8962E-60	1.12101681	0.544	0.239	4.16E-56
Hist2h3b	1.9485E-60	2.70453613	0.061	0.006	4.2749E-56
Txnl1	2.238E-60	0.98364974	0.547	0.232	4.9099E-56
Sytl2	2.5741E-60	1.10997707	0.263	0.071	5.6474E-56
Fdx1	2.5955E-60	1.52358921	0.349	0.118	5.6943E-56
Rfc2	4.9553E-60	1.36846678	0.268	0.077	1.0871E-55
Brca2	5.3353E-60	2.67319475	0.075	0.009	1.1705E-55
Bex3	7.0503E-60	0.93013152	0.501	0.201	1.5468E-55
Ywhah	8.4521E-60	0.87129056	0.597	0.262	1.8543E-55
Nde1	9.6424E-60	1.69200007	0.172	0.037	2.1154E-55
Atp5j2	1.2055E-59	0.80330756	0.886	0.534	2.6448E-55
Nsmce1	1.2242E-59	1.15940151	0.301	0.091	2.6858E-55
Hist1h1c	1.4394E-59	0.81050894	0.542	0.229	3.1578E-55
Atp5g3	1.5158E-59	0.83785315	0.782	0.423	3.3256E-55
H1f0	2.1243E-59	1.43156957	0.297	0.092	4.6605E-55
Srp19	2.5048E-59	1.00176896	0.556	0.243	5.4954E-55
Atp5g1	3.7532E-59	0.86905442	0.75	0.387	8.2342E-55
Eif3h	4.9494E-59	0.76566746	0.907	0.601	1.0858E-54
Cd48	6.8201E-59	1.22525579	0.383	0.138	1.4963E-54
Uqcrq	1.4969E-58	0.76174957	0.905	0.571	3.2841E-54
Rad18	2.3059E-58	1.24164303	0.261	0.073	5.059E-54

Dnajc19	3.7861E-58	1.072213	0.485	0.198	8.3063E-54
Tff1	3.8176E-58	2.03484337	0.095	0.013	8.3753E-54
Ndufa8	5.4908E-58	1.04266093	0.492	0.205	1.2046E-53
Gmfg	6.9008E-58	0.74828342	0.88	0.539	1.514E-53
Pdcd5	7.741E-58	0.92617517	0.642	0.301	1.6983E-53
Ssbp3	1.1661E-57	1.060224	0.336	0.109	2.5583E-53
Ctsw	1.3963E-57	1.05077366	0.401	0.143	3.0634E-53
Atpif1	1.5288E-57	0.79497543	0.705	0.339	3.354E-53
Psmb8	1.5894E-57	0.87680618	0.787	0.441	3.487E-53
Cox6a1	2.6368E-57	0.80681013	0.859	0.513	5.7849E-53
Smc6	2.73E-57	0.78676387	0.583	0.256	5.9893E-53
Pkm	3.0787E-57	0.84543082	0.775	0.439	6.7543E-53
Gimap7	3.8486E-57	0.93338194	0.469	0.179	8.4435E-53
Cdkn2c	5.1365E-57	2.0968866	0.109	0.017	1.1269E-52
Troap	6.7128E-57	4.3199721	0.025	0.001	1.4727E-52
Fkbp2	6.7934E-57	1.05546936	0.463	0.187	1.4904E-52
Mrps24	8.8774E-57	0.96699811	0.521	0.221	1.9476E-52
Tbc1d31	1.1591E-56	2.09918371	0.114	0.019	2.543E-52
Hist1h2br	1.6959E-56	2.66904896	0.068	0.007	3.7206E-52
Pih1d1	2.0165E-56	1.64497334	0.211	0.055	4.424E-52
A430005L14Ri	2.4963E-56	1.61558022	0.204	0.052	5.4767E-52
Tmem14c	2.5855E-56	0.97585554	0.578	0.262	5.6724E-52
Thap12	3.0705E-56	1.07506904	0.349	0.118	6.7363E-52
Snrpa1	3.9156E-56	1.04524393	0.383	0.137	8.5904E-52
H3f3b	6.1924E-56	0.51741672	0.998	0.968	1.3586E-51
Ddit4	1.0332E-55	0.9865235	0.465	0.185	2.2668E-51
1700012B07R	1.0838E-55	1.96881735	0.106	0.017	2.3778E-51
Furin	1.1048E-55	0.70831891	0.758	0.421	2.4238E-51
Runx2	1.1153E-55	0.92904689	0.435	0.165	2.4468E-51
Skp2	1.3411E-55	2.81152699	0.063	0.006	2.9422E-51
Psmb2	1.507E-55	1.01655789	0.555	0.25	3.3062E-51
Hnrnpab	2.0792E-55	0.88532822	0.796	0.476	4.5616E-51
Fundc2	2.4487E-55	0.99911635	0.504	0.213	5.3723E-51
Faf1	2.9237E-55	1.35603997	0.238	0.066	6.4142E-51
Smap1	3.5884E-55	0.84614302	0.603	0.276	7.8725E-51
Cdt1	3.6921E-55	1.09079621	0.283	0.085	8.1002E-51
Cycs	4.259E-55	0.81077101	0.694	0.338	9.3439E-51
Cchcr1	4.3362E-55	2.42401948	0.077	0.01	9.5133E-51
Coro1a	4.8159E-55	0.82132358	0.894	0.607	1.0566E-50
Ncald	4.983E-55	1.68901193	0.163	0.035	1.0932E-50
Tmsb4x	6.142E-55	0.43439374	0.998	0.997	1.3475E-50
Eloc	6.8153E-55	0.81172369	0.73	0.377	1.4952E-50
Gstp3	6.9892E-55	1.39750832	0.206	0.052	1.5334E-50
Cd96	9.1095E-55	1.12533292	0.252	0.071	1.9985E-50
Il17a	9.505E-55	1.41181235	0.199	0.05	2.0853E-50
Pop5	1.3361E-54	1.13208643	0.338	0.115	2.9312E-50
Psat1	1.3952E-54	1.64143987	0.168	0.038	3.061E-50
Brd9	1.9308E-54	0.99162917	0.429	0.166	4.2359E-50

Gng2	2.4283E-54	0.90030856	0.651	0.312	5.3275E-50
Psph	2.6772E-54	1.70774995	0.157	0.034	5.8734E-50
Cdk2	3.1746E-54	1.82989639	0.129	0.024	6.9647E-50
Psmb5	4.0756E-54	1.02905231	0.56	0.26	8.9414E-50
Yeats4	5.3609E-54	1.04343002	0.492	0.212	1.1761E-49
Hp1bp3	6.0155E-54	1.02046499	0.567	0.263	1.3197E-49
Sugt1	8.6954E-54	0.98223111	0.546	0.246	1.9077E-49
Ndfip1	8.9831E-54	0.74834625	0.619	0.282	1.9708E-49
0610010K14R	9.5788E-54	1.55125951	0.191	0.048	2.1015E-49
Gtf2i	9.7309E-54	0.86448448	0.411	0.151	2.1349E-49
Ptpn22	1.0374E-53	0.53614051	0.791	0.379	2.2758E-49
Rnaseh2a	1.2357E-53	1.75576018	0.157	0.035	2.7109E-49
Edf1	1.6558E-53	0.83497383	0.735	0.4	3.6327E-49
Uba2	1.6826E-53	1.08143608	0.32	0.107	3.6915E-49
Tonsl	1.7547E-53	2.84051546	0.05	0.004	3.8496E-49
Ostc	2.0504E-53	0.87205395	0.61	0.287	4.4985E-49
Tmem167	2.4553E-53	0.83637269	0.624	0.296	5.3867E-49
Ndufc2	2.6117E-53	0.93252051	0.617	0.301	5.7299E-49
Lyar	2.9096E-53	0.99768361	0.383	0.142	6.3833E-49
Dgat1	3.0769E-53	0.50702153	0.846	0.457	6.7505E-49
Hnrnpl	5.9249E-53	0.84107917	0.603	0.282	1.2999E-48
Abracl	6.8363E-53	0.76830392	0.821	0.485	1.4998E-48
Hdac1	8.3125E-53	1.09455549	0.327	0.111	1.8237E-48
Crot	1.067E-52	0.82546028	0.365	0.127	2.3409E-48
U2af1	1.762E-52	0.93217655	0.614	0.297	3.8657E-48
Ybx3	1.7919E-52	0.86804894	0.605	0.292	3.9313E-48
Cntrl	2.4122E-52	1.01807821	0.415	0.16	5.2922E-48
Thoc7	2.4149E-52	1.01584456	0.474	0.199	5.298E-48
Nup85	2.7895E-52	1.63915098	0.141	0.029	6.12E-48
S1pr4	3.2146E-52	1.3105511	0.213	0.056	7.0525E-48
Ndufs6	3.3205E-52	0.87219075	0.58	0.271	7.2849E-48
Mcm8	3.64E-52	2.965861	0.043	0.003	7.9859E-48
Elof1	3.8504E-52	1.30322313	0.304	0.103	8.4475E-48
Cysltr2	5.1682E-52	0.97977485	0.267	0.079	1.1339E-47
Myl6	5.2436E-52	0.65573433	0.982	0.785	1.1504E-47
Ccnf	5.6211E-52	2.65777901	0.07	0.008	1.2332E-47
Gtf2h5	5.806E-52	0.86673104	0.558	0.258	1.2738E-47
Slc25a40	6.1596E-52	1.3647105	0.172	0.04	1.3514E-47
Dcps	6.7219E-52	1.48711524	0.195	0.05	1.4747E-47
Cd47	6.947E-52	0.73652622	0.864	0.544	1.5241E-47
Sms	8.2429E-52	0.9061573	0.504	0.214	1.8084E-47
Stk26	8.3314E-52	0.99666319	0.301	0.095	1.8278E-47
Smco4	1.2101E-51	1.10704085	0.324	0.109	2.6549E-47
Cox6b1	1.3916E-51	0.68883033	0.939	0.622	3.053E-47
Gtse1	1.7449E-51	3.66732598	0.032	0.002	3.8281E-47
Mphosph9	2.0564E-51	1.59193441	0.118	0.022	4.5115E-47
Cd226	2.3575E-51	1.3431236	0.193	0.049	5.1722E-47
Eri1	3.1276E-51	1.05184018	0.377	0.143	6.8617E-47

Psmb6	3.1517E-51	0.82777321	0.699	0.364	6.9145E-47
Zfp101	3.2253E-51	1.82824485	0.106	0.018	7.076E-47
Ccsap	3.3213E-51	1.91800108	0.109	0.019	7.2865E-47
Cdkn2aipnl	3.8816E-51	1.3665005	0.193	0.049	8.5157E-47
Ppil1	3.8909E-51	1.43513909	0.168	0.039	8.5363E-47
Madd	4.0327E-51	0.77820807	0.54	0.233	8.8474E-47
Gadd45gip1	4.7889E-51	1.02851879	0.381	0.144	1.0506E-46
Arhgdib	5.8354E-51	0.78967943	0.877	0.597	1.2802E-46
Ttc39c	6.0816E-51	1.15744113	0.249	0.073	1.3342E-46
Nono	7.632E-51	0.77775929	0.605	0.279	1.6744E-46
Eif1ax	1.0542E-50	1.02611826	0.487	0.213	2.3128E-46
Reep4	1.3458E-50	1.48379826	0.175	0.043	2.9526E-46
Ube2m	1.4659E-50	1.01211944	0.444	0.186	3.2161E-46
Dcaf15	1.5409E-50	1.02017598	0.272	0.084	3.3806E-46
Ube2i	1.5809E-50	0.7485346	0.814	0.456	3.4684E-46
Swi5	2.0016E-50	1.04964679	0.512	0.234	4.3913E-46
Mbd2	2.093E-50	0.68456014	0.726	0.371	4.5919E-46
Plp2	2.9499E-50	1.16421205	0.309	0.106	6.4717E-46
Etaa1	3.512E-50	1.21266763	0.218	0.06	7.7051E-46
Mrps14	5.3145E-50	0.88208814	0.549	0.255	1.166E-45
Gm14091	6.9474E-50	4.97724523	0.013	0	1.5242E-45
Pttg1	7.1629E-50	1.07875945	0.345	0.125	1.5715E-45
Tpm3	7.83E-50	0.64154315	0.911	0.633	1.7178E-45
Fxn	9.309E-50	1.69582951	0.154	0.035	2.0423E-45
Fam96b	9.6119E-50	0.84490717	0.49	0.207	2.1088E-45
Aip	1.1145E-49	1.0440727	0.326	0.113	2.4451E-45
Fsbp	1.1521E-49	4.51766245	0.018	0.001	2.5275E-45
Ccdc18	1.1548E-49	2.85260935	0.041	0.003	2.5336E-45
Abhd8	1.1714E-49	1.5903425	0.145	0.031	2.5699E-45
Ywhaq	1.3376E-49	0.8391949	0.535	0.244	2.9346E-45
Gltp	1.3687E-49	1.0141258	0.431	0.181	3.0029E-45
Gnpda2	1.4323E-49	1.56273645	0.215	0.06	3.1423E-45
Hist1h2ad	1.4642E-49	3.23246638	0.034	0.002	3.2123E-45
Dnph1	1.5939E-49	3.75123465	0.036	0.002	3.4968E-45
Uqcrfs1	1.6269E-49	0.77573294	0.676	0.343	3.5693E-45
Bola3	1.8039E-49	0.9929588	0.379	0.144	3.9576E-45
Chst2	2.0851E-49	1.75736051	0.127	0.025	4.5746E-45
H2-T22	2.112E-49	0.77041678	0.531	0.234	4.6335E-45
Atp5b	2.8162E-49	0.68725708	0.902	0.581	6.1785E-45
Pdcd4	3.0747E-49	0.61082799	0.676	0.318	6.7457E-45
Gnai2	3.5779E-49	0.64623631	0.936	0.668	7.8496E-45
Ndufb9	3.6364E-49	0.80220489	0.703	0.378	7.9778E-45
Ndufb3	3.6733E-49	0.84065651	0.578	0.275	8.0588E-45
Snrpd3	3.8599E-49	0.92255938	0.533	0.249	8.4682E-45
Polr2j	4.7474E-49	1.26187306	0.288	0.097	1.0415E-44
Ccr4	4.9695E-49	1.02575039	0.197	0.051	1.0903E-44
Mast2	5.7448E-49	1.97496195	0.12	0.023	1.2603E-44
Sptssa	7.3207E-49	0.66655118	0.535	0.239	1.6061E-44

Arhgef39	7.9612E-49	4.19351893	0.027	0.001	1.7466E-44
Tuba1a	9.8329E-49	0.78816094	0.37	0.139	2.1572E-44
Timm17a	1.3143E-48	0.96135362	0.415	0.167	2.8834E-44
Rsl1d1	1.5419E-48	0.79853068	0.479	0.203	3.3828E-44
Haus3	2.6151E-48	0.97617351	0.293	0.096	5.7373E-44
Ddias	2.7146E-48	2.69259607	0.052	0.005	5.9555E-44
1700102H20R	2.8026E-48	3.80650803	0.025	0.001	6.1487E-44
Mnd1	3.3365E-48	3.44161323	0.038	0.003	7.3199E-44
Vax2	3.3925E-48	2.01923935	0.088	0.013	7.4428E-44
Vps36	3.6299E-48	0.98463089	0.415	0.17	7.9637E-44
Prim2	3.6784E-48	1.7715056	0.111	0.02	8.07E-44
Rbm8a	5.6668E-48	0.77467321	0.671	0.343	1.2432E-43
Upf3a	5.866E-48	0.97908943	0.347	0.128	1.2869E-43
Pcbd2	8.7633E-48	1.27331234	0.286	0.098	1.9226E-43
Ets1	1.1519E-47	0.54622118	0.991	0.665	2.5271E-43
Commd1	1.5041E-47	1.26952643	0.288	0.1	3.2999E-43
Atp10a	1.6666E-47	1.12777796	0.211	0.058	3.6564E-43
Serpinb1a	2.2803E-47	1.01465501	0.372	0.144	5.0027E-43
Tmem50a	2.2839E-47	0.75518318	0.689	0.361	5.0108E-43
Ubl4a	2.7458E-47	1.17598088	0.256	0.081	6.0241E-43
Hccs	2.9435E-47	1.05622643	0.369	0.142	6.4578E-43
Rb1	4.0593E-47	1.01283583	0.376	0.146	8.9057E-43
Blmh	4.1628E-47	1.01758306	0.336	0.124	9.1328E-43
Emb	5.321E-47	0.42339176	0.966	0.619	1.1674E-42
Pole4	6.6283E-47	0.74288353	0.546	0.25	1.4542E-42
Sh2d1a	6.665E-47	1.0598953	0.254	0.078	1.4622E-42
Trbc1	8.4339E-47	0.87954755	0.551	0.261	1.8503E-42
Park7	8.5822E-47	0.82075974	0.619	0.31	1.8829E-42
Snrnp40	9.8197E-47	1.07182904	0.286	0.097	2.1543E-42
Lysmd2	9.9197E-47	1.5321417	0.148	0.034	2.1763E-42
Psma4	1.0756E-46	0.85332843	0.556	0.266	2.3597E-42
Grpel1	1.6455E-46	1.01539303	0.392	0.159	3.61E-42
Aurkaip1	1.8433E-46	0.81182826	0.538	0.253	4.044E-42
Prdx4	1.9093E-46	1.5320706	0.204	0.059	4.1889E-42
Odc1	1.9298E-46	0.46825179	0.907	0.619	4.2338E-42
Atp5g2	3.2996E-46	0.59630915	0.932	0.691	7.2389E-42
Gpx4	3.3332E-46	0.69122794	0.86	0.533	7.3127E-42
Snu13	4.481E-46	0.75610401	0.721	0.394	9.8308E-42
Cuta	4.7243E-46	0.87349108	0.472	0.208	1.0365E-41
Gimap1	4.9129E-46	0.5347062	0.621	0.275	1.0778E-41
Sdhb	5.0451E-46	0.95846355	0.428	0.182	1.1068E-41
Ctdspl2	5.0681E-46	0.97866845	0.356	0.134	1.1119E-41
Dnajc8	5.3342E-46	0.66378793	0.75	0.401	1.1703E-41
Mrpl35	6.8218E-46	1.12152129	0.279	0.095	1.4966E-41
Gm15518	7.063E-46	3.63456496	0.029	0.002	1.5495E-41
Hmbs	8.7372E-46	1.883265	0.116	0.023	1.9168E-41
Psmc3	9.1691E-46	0.94203496	0.542	0.265	2.0116E-41
Akap13	9.3706E-46	0.52446168	0.979	0.753	2.0558E-41

Cwc15	1.0399E-45	0.73714668	0.716	0.392	2.2815E-41
Prelid3b	1.2018E-45	1.1173064	0.327	0.122	2.6366E-41
Vdac1	1.2212E-45	0.96922595	0.428	0.183	2.6793E-41
Dynlrb1	1.2268E-45	0.81839696	0.565	0.275	2.6916E-41
Ercc6l	1.2748E-45	2.5044318	0.072	0.01	2.7968E-41
Vps28	1.3168E-45	0.7856961	0.558	0.263	2.8889E-41
Ndufc1	1.384E-45	0.82662148	0.524	0.246	3.0362E-41
Larp7	2.126E-45	0.87457482	0.403	0.162	4.6642E-41
Spcs2	2.1526E-45	0.69681996	0.776	0.431	4.7226E-41
Plekha5	2.1532E-45	1.13696937	0.215	0.062	4.7239E-41
Tma7	2.5769E-45	0.66471991	0.884	0.576	5.6535E-41
Ifi27	2.7721E-45	0.91911125	0.453	0.196	6.0817E-41
Rbbp8	3.2831E-45	1.09050695	0.358	0.142	7.2027E-41
Sdf4	3.3115E-45	0.65769372	0.698	0.366	7.2651E-41
Lsp1	3.3388E-45	0.57467116	0.841	0.463	7.325E-41
Fam173a	3.6702E-45	1.00995619	0.385	0.155	8.052E-41
Sf3b6	3.9936E-45	0.66563018	0.739	0.411	8.7615E-41
Polr1d	4.0038E-45	0.67579739	0.816	0.487	8.784E-41
Anxa6	4.0307E-45	0.76769706	0.544	0.253	8.8429E-41
Impdh2	4.8659E-45	0.82783634	0.456	0.197	1.0675E-40
Hnrnpd	5.0247E-45	0.73837445	0.796	0.465	1.1024E-40
Bag1	5.22E-45	0.74875293	0.589	0.286	1.1452E-40
Gm20628	5.4758E-45	2.9024263	0.032	0.002	1.2013E-40
Ubl5	6.1699E-45	0.54818699	0.955	0.692	1.3536E-40
Nudt5	6.3932E-45	1.44111181	0.188	0.052	1.4026E-40
Itgb7	6.4432E-45	0.70458592	0.546	0.247	1.4136E-40
Trir	9.0039E-45	0.70269207	0.673	0.35	1.9754E-40
Ndc1	9.2182E-45	2.11186347	0.082	0.013	2.0224E-40
Cnot6	9.2467E-45	0.78374016	0.53	0.244	2.0286E-40
Ndufb10	1.0331E-44	0.83150654	0.624	0.33	2.2665E-40
Zfp91	1.0461E-44	0.72637933	0.694	0.378	2.2951E-40
Nudc	1.0611E-44	0.96365609	0.408	0.172	2.328E-40
Fyb	1.1971E-44	0.58082166	0.868	0.508	2.6263E-40
Rnf26	1.4128E-44	2.05381144	0.098	0.018	3.0996E-40
Psma1	1.419E-44	0.74114734	0.538	0.252	3.1132E-40
Gm10282	1.6576E-44	3.30119967	0.029	0.002	3.6367E-40
Nme1	1.6888E-44	0.80054848	0.633	0.327	3.705E-40
Cep295	2.3128E-44	1.14576663	0.229	0.07	5.074E-40
Mtbp	3.3239E-44	1.7683563	0.088	0.014	7.2924E-40
Hikeshi	3.7196E-44	1.2826107	0.227	0.071	8.1604E-40
Nedd1	4.8324E-44	1.6634151	0.114	0.023	1.0602E-39
Cdk5rap2	4.9723E-44	1.30933235	0.174	0.046	1.0909E-39
Eif3c	5.986E-44	0.67899588	0.853	0.532	1.3133E-39
Prelid2	6.8458E-44	1.51973337	0.197	0.056	1.5019E-39
Serf2	7.9227E-44	0.50035929	0.993	0.848	1.7382E-39
Atp5d	8.6963E-44	0.73566551	0.792	0.487	1.9079E-39
Cox7b	9.3E-44	0.66453294	0.839	0.511	2.0403E-39
Mis12	9.8988E-44	1.3934161	0.2	0.058	2.1717E-39

Krt83	1.0831E-43	1.57913011	0.143	0.034	2.3762E-39
Aprt	1.1017E-43	0.91673671	0.61	0.329	2.4169E-39
Mrps16	1.181E-43	0.82984653	0.49	0.224	2.5911E-39
Rpp21	1.9458E-43	0.89992245	0.376	0.151	4.2689E-39
Rad50	2.2639E-43	0.92767996	0.249	0.08	4.9668E-39
Wdr90	2.3962E-43	2.12547766	0.081	0.013	5.2569E-39
Sri	2.8839E-43	0.73557425	0.687	0.377	6.327E-39
Itk	3.321E-43	0.41331427	0.683	0.316	7.286E-39
Ndufa13	4.328E-43	0.66673368	0.834	0.522	9.4953E-39
Ube2a	6.3565E-43	0.80503382	0.506	0.235	1.3946E-38
Mrps36	6.8005E-43	1.07785267	0.311	0.117	1.492E-38
Khsrp	6.9331E-43	0.97747655	0.338	0.13	1.5211E-38
Eed	7.4422E-43	0.92436638	0.313	0.114	1.6327E-38
Mien1	1.0895E-42	0.66639283	0.544	0.256	2.3903E-38
Mrpl20	1.1326E-42	0.91669432	0.462	0.21	2.4848E-38
Chchd2	1.1352E-42	0.51166521	0.977	0.778	2.4906E-38
Sept6	1.2396E-42	0.6941994	0.47	0.201	2.7196E-38
Sept9	1.31E-42	0.86085401	0.397	0.164	2.8741E-38
Acadl	1.3965E-42	0.94432745	0.381	0.157	3.0637E-38
Cd52	1.4623E-42	0.55213057	0.973	0.818	3.2082E-38
Cxcr3	1.6368E-42	1.09025129	0.179	0.048	3.5909E-38
Itpr1	1.73E-42	0.78655514	0.451	0.195	3.7955E-38
Pam16	1.7775E-42	0.85192314	0.365	0.145	3.8996E-38
Npat	1.8071E-42	1.17954726	0.186	0.052	3.9646E-38
Pds5a	1.9989E-42	0.71100526	0.469	0.204	4.3854E-38
Chaf1b	2.4953E-42	2.18323453	0.088	0.015	5.4745E-38
Casp3	2.874E-42	1.01720137	0.297	0.108	6.3054E-38
Tmem258	3.1989E-42	0.69337188	0.705	0.382	7.018E-38
Cfl1	4.1031E-42	0.54401342	0.966	0.781	9.0018E-38
Slc25a5	4.1874E-42	0.63548679	0.825	0.51	9.1867E-38
Hnrnpa2b1	5.1262E-42	0.56304834	0.955	0.768	1.1246E-37
Ubap2	5.7661E-42	0.98009673	0.267	0.091	1.265E-37
Tcerg1	6.0287E-42	0.784159	0.395	0.163	1.3226E-37
H2-K1	6.0972E-42	0.48850694	0.998	0.913	1.3377E-37
Atp5c1	6.3405E-42	0.7021783	0.773	0.448	1.391E-37
Srrt	6.5262E-42	0.89018677	0.345	0.135	1.4318E-37
Bcl7c	6.7723E-42	1.00956871	0.336	0.132	1.4858E-37
Nubp1	7.1624E-42	1.04578549	0.309	0.117	1.5714E-37
Aurka	7.7584E-42	2.5815383	0.061	0.008	1.7021E-37
Dazap1	7.8016E-42	0.87271825	0.399	0.167	1.7116E-37
Wdhd1	8.4749E-42	1.47029396	0.12	0.026	1.8593E-37
Cenpc1	8.5419E-42	1.26686024	0.202	0.06	1.874E-37
Ramp3	1.0558E-41	0.47382564	0.719	0.382	2.3163E-37
Ifng	1.4765E-41	0.8916397	0.188	0.053	3.2392E-37
Pin1	1.5366E-41	1.00250495	0.356	0.144	3.3712E-37
Ppat	1.7002E-41	1.21367655	0.166	0.044	3.73E-37
Grb7	2.2992E-41	1.31447442	0.125	0.028	5.0443E-37
Ubald2	2.3668E-41	0.47824153	0.878	0.558	5.1925E-37

Srp9	2.523E-41	0.61513296	0.805	0.475	5.5351E-37
Nup210	2.6894E-41	0.85321365	0.284	0.1	5.9003E-37
Smarcc1	2.7597E-41	1.01205722	0.277	0.099	6.0545E-37
Osbpl3	3.7219E-41	0.95271092	0.249	0.082	8.1655E-37
Pcmt1	3.7266E-41	0.82781034	0.397	0.167	8.1757E-37
Ppib	4.4526E-41	0.64844994	0.832	0.523	9.7686E-37
Impa2	5.6685E-41	1.32694256	0.179	0.05	1.2436E-36
Ndufab1	6.2462E-41	0.83328506	0.487	0.229	1.3704E-36
Tdrkh	6.371E-41	2.10215912	0.073	0.011	1.3977E-36
Gcat	8.3878E-41	2.43933338	0.068	0.01	1.8402E-36
Tomm22	1.1747E-40	0.65597152	0.606	0.304	2.5771E-36
Pgam1	1.205E-40	0.79903089	0.51	0.245	2.6436E-36
Timm17b	1.3304E-40	0.95152491	0.308	0.117	2.9187E-36
Rbmx2	1.3668E-40	1.25502324	0.177	0.05	2.9986E-36
Abcf1	1.5634E-40	0.69904147	0.522	0.248	3.4299E-36
Polr3k	1.8564E-40	1.21357911	0.208	0.064	4.0728E-36
Taf10	1.9856E-40	0.69496681	0.712	0.392	4.3563E-36
Tbrg1	2.154E-40	0.75188136	0.465	0.209	4.7256E-36
Acp1	2.9565E-40	0.99549261	0.336	0.135	6.4863E-36
Cenpu	3.6937E-40	2.25384862	0.048	0.005	8.1036E-36
Cdk2ap1	4.2121E-40	0.92099035	0.299	0.111	9.2409E-36
Ssr2	4.7649E-40	0.99550589	0.392	0.172	1.0454E-35
Hspa14	4.8245E-40	0.97245931	0.32	0.125	1.0584E-35
Erg28	5.2656E-40	1.09491155	0.227	0.074	1.1552E-35
Myb	5.4712E-40	0.69750175	0.168	0.045	1.2003E-35
Xpo1	5.8239E-40	0.98800605	0.283	0.103	1.2777E-35
Zmynd19	6.8016E-40	1.73703405	0.113	0.024	1.4922E-35
Ech1	7.9817E-40	1.17354224	0.275	0.101	1.7511E-35
Polr2c	8.4595E-40	0.88243155	0.381	0.16	1.8559E-35
Fam162a	8.5928E-40	1.3422886	0.254	0.09	1.8852E-35
Fam102a	1.3811E-39	0.76532181	0.309	0.114	3.03E-35
Snf8	1.4828E-39	0.86135847	0.408	0.181	3.2531E-35
Get4	1.5581E-39	1.07944935	0.204	0.063	3.4183E-35
Ubac1	1.6668E-39	1.48647126	0.138	0.034	3.6568E-35
Cyc1	1.697E-39	0.91687926	0.365	0.153	3.723E-35
Stag1	1.817E-39	0.82783544	0.403	0.173	3.9864E-35
Golm1	1.8649E-39	1.40063493	0.138	0.034	4.0914E-35
Fcho1	2.2564E-39	1.31341946	0.138	0.034	4.9504E-35
Prps1	2.5242E-39	1.03117612	0.247	0.084	5.5377E-35
Mrpl28	2.5839E-39	1.16780998	0.222	0.073	5.6688E-35
Rexo2	2.6586E-39	0.89433478	0.442	0.203	5.8328E-35
Fam103a1	3.0442E-39	0.72932248	0.433	0.19	6.6787E-35
Tsn	3.1152E-39	0.71852796	0.506	0.241	6.8344E-35
Raf1	3.195E-39	0.7752338	0.376	0.154	7.0096E-35
Hlf	3.54E-39	0.56041518	0.297	0.108	7.7663E-35
Dkkl1	4.1377E-39	1.51609303	0.127	0.029	9.0778E-35
Kpnb1	4.1873E-39	0.83095928	0.342	0.137	9.1864E-35
Mxi1	6.1161E-39	0.76073629	0.569	0.299	1.3418E-34

Mphosph6	6.8793E-39	1.27540423	0.268	0.099	1.5092E-34
Psma2	7.3063E-39	0.65931061	0.721	0.411	1.6029E-34
Itm2b	7.3763E-39	0.44441999	0.95	0.722	1.6183E-34
Agfg2	7.7131E-39	1.81921155	0.095	0.018	1.6922E-34
Fkbp8	8.4804E-39	0.82213573	0.435	0.198	1.8605E-34
Brip1os	8.7289E-39	1.45949743	0.127	0.03	1.915E-34
Psmd13	9.4827E-39	0.87119421	0.372	0.158	2.0804E-34
Polr2f	9.6675E-39	0.94302862	0.379	0.166	2.121E-34
Ndufa1	1.0331E-38	0.6541626	0.737	0.419	2.2665E-34
Cep83	1.3582E-38	0.84800869	0.309	0.12	2.9798E-34
Mrpl51	1.7458E-38	0.90981882	0.329	0.132	3.83E-34
Kdm2b	2.0629E-38	0.43073851	0.691	0.355	4.5259E-34
Taf1	2.1756E-38	0.69056817	0.379	0.158	4.773E-34
Anapc15	2.3765E-38	1.1996872	0.215	0.071	5.2139E-34
Esyt1	2.5154E-38	0.80620944	0.403	0.176	5.5185E-34
Ppp2r2d	2.5671E-38	0.81147125	0.458	0.214	5.6319E-34
Znrf1	2.57E-38	0.67507256	0.338	0.132	5.6383E-34
H2-D1	2.8109E-38	0.44972954	0.993	0.914	6.1669E-34
Golt1b	3.2817E-38	1.07671017	0.267	0.097	7.1997E-34
Sesn3	3.6018E-38	0.58656847	0.485	0.218	7.902E-34
Pde5a	3.8744E-38	1.46664091	0.125	0.029	8.5001E-34
Fancb	3.9555E-38	3.12388076	0.029	0.002	8.678E-34
Atp2b4	4.3686E-38	0.64923818	0.444	0.195	9.5843E-34
Tmem256	4.6654E-38	0.86955114	0.483	0.236	1.0235E-33
Ubr7	5.2774E-38	1.14240697	0.197	0.061	1.1578E-33
Psme2	5.6422E-38	0.59819438	0.744	0.441	1.2378E-33
Nr3c1	6.2511E-38	0.44872804	0.767	0.434	1.3714E-33
Tpr	6.8339E-38	0.56993796	0.809	0.512	1.4993E-33
Rpgrip1	7.3581E-38	1.1395248	0.163	0.045	1.6143E-33
Ndufs4	9.1108E-38	0.73614726	0.479	0.228	1.9988E-33
Sept7	9.9816E-38	0.5960503	0.621	0.326	2.1899E-33
Spcs1	1.0231E-37	0.6656029	0.56	0.283	2.2445E-33
Gimap9	1.7091E-37	0.59596514	0.345	0.135	3.7495E-33
Pfdn1	1.7423E-37	0.9533663	0.345	0.145	3.8225E-33
Dctn3	1.8933E-37	0.72744989	0.537	0.271	4.1537E-33
Aaas	2.0945E-37	1.69245365	0.088	0.016	4.5951E-33
Tomm7	2.1027E-37	0.52779529	0.916	0.643	4.613E-33
Ccp110	2.1492E-37	1.89617114	0.082	0.015	4.7151E-33
Emsy	3.2311E-37	0.61660895	0.442	0.194	7.0887E-33
Cpd	3.505E-37	1.00229369	0.27	0.1	7.6897E-33
Ppp1r16b	3.5832E-37	0.42062094	0.658	0.319	7.8613E-33
Sap18	3.9934E-37	0.73165128	0.453	0.212	8.7612E-33
Metap2	4.9409E-37	0.72720945	0.587	0.313	1.084E-32
Arf5	5.2837E-37	0.60238994	0.782	0.478	1.1592E-32
Pfkp	5.367E-37	0.60400876	0.544	0.264	1.1775E-32
Tceal9	5.5017E-37	0.60963678	0.42	0.187	1.207E-32
Gtf3a	6.4957E-37	1.25442671	0.186	0.057	1.4251E-32
Stat5b	6.8446E-37	0.43973133	0.581	0.28	1.5016E-32

Rplp0	7.2062E-37	0.41076974	0.996	0.935	1.581E-32
Pecam1	7.8707E-37	0.72003514	0.345	0.139	1.7268E-32
Polg2	8.1215E-37	0.98887364	0.218	0.071	1.7818E-32
Katnb1	8.2014E-37	1.95301965	0.079	0.014	1.7993E-32
Fbxo48	9.2287E-37	3.59111247	0.021	0.001	2.0247E-32
Tusc3	1.0427E-36	1.08141139	0.213	0.07	2.2876E-32
Eif4a3	1.1786E-36	0.77853565	0.383	0.167	2.5857E-32
Paip2b	1.2282E-36	0.75390644	0.252	0.087	2.6946E-32
Psmb1	1.2363E-36	0.62407085	0.832	0.541	2.7123E-32
Limd2	1.2522E-36	0.52106013	0.621	0.316	2.7472E-32
Cfap20	1.4827E-36	1.01434778	0.224	0.076	3.2529E-32
Msh6	1.5555E-36	1.61239939	0.104	0.022	3.4126E-32
Cir1	1.6071E-36	0.52619475	0.503	0.229	3.5258E-32
Coq7	1.6878E-36	1.68644268	0.109	0.024	3.7029E-32
Psmd4	1.7003E-36	0.65617518	0.601	0.316	3.7304E-32
Eif1ad	1.7221E-36	0.98111356	0.293	0.115	3.7782E-32
Rhno1	2.2763E-36	1.09891588	0.163	0.046	4.9939E-32
Pold1	2.3572E-36	1.42586971	0.111	0.025	5.1715E-32
Phf5a	2.7641E-36	0.67214212	0.463	0.217	6.0642E-32
Hcfc1	2.8807E-36	1.04118049	0.224	0.076	6.3199E-32
Parpbp	2.9501E-36	3.87363438	0.021	0.001	6.4721E-32
Mrps30	3.4798E-36	1.04285643	0.227	0.078	7.6343E-32
Naa38	4.3689E-36	1.07265578	0.309	0.127	9.585E-32
Neurl1b	4.3877E-36	3.69467689	0.03	0.002	9.6261E-32
Dynll1	4.5146E-36	0.61718018	0.687	0.386	9.9045E-32
Psmb7	5.0042E-36	0.79702232	0.474	0.233	1.0979E-31
Inf2	5.3494E-36	1.28650741	0.186	0.058	1.1736E-31
Ndufa3	5.4612E-36	0.6732595	0.687	0.407	1.1981E-31
Srpk1	5.5005E-36	0.71303975	0.415	0.185	1.2067E-31
Ndufv2	6.1897E-36	0.80592104	0.415	0.192	1.358E-31
Trim28	6.2214E-36	0.80408929	0.306	0.121	1.3649E-31
Kmt5a	7.3395E-36	0.8687608	0.349	0.149	1.6102E-31
Dck	7.5809E-36	1.15877433	0.204	0.067	1.6632E-31
Ndufa2	8.5972E-36	0.52892312	0.919	0.632	1.8861E-31
Paics	8.743E-36	0.99642462	0.295	0.118	1.9181E-31
Rnf7	8.9938E-36	0.7328041	0.442	0.209	1.9731E-31
Hist1h4j	9.6085E-36	2.87540297	0.032	0.003	2.108E-31
Mrpl44	9.9411E-36	1.2360365	0.147	0.04	2.181E-31
Sec11a	9.9843E-36	0.68988319	0.51	0.253	2.1905E-31
Slc25a24	1.4023E-35	1.27032441	0.2	0.066	3.0765E-31
Ctla2a	1.431E-35	0.27615285	0.397	0.167	3.1395E-31
Psmb4	1.4961E-35	0.66210421	0.655	0.37	3.2824E-31
Ak2	1.7157E-35	0.75846579	0.413	0.19	3.7641E-31
Grap2	1.7555E-35	0.54174533	0.419	0.18	3.8514E-31
Denr	1.7594E-35	0.52475315	0.508	0.243	3.8599E-31
Tnfsf8	1.8295E-35	0.46825608	0.32	0.128	4.0138E-31
Cdc123	1.8965E-35	1.07724198	0.224	0.077	4.1607E-31
Ccdc107	2.0941E-35	0.94505862	0.275	0.105	4.5943E-31

Gabpb1	2.2735E-35	0.55402716	0.395	0.168	4.9877E-31
Calm2	2.2888E-35	0.52251564	0.909	0.617	5.0213E-31
Cep57	2.2898E-35	0.82719783	0.259	0.094	5.0236E-31
Sit1	2.3449E-35	1.50951828	0.109	0.025	5.1445E-31
Nedd8	2.4409E-35	0.62161317	0.699	0.401	5.355E-31
Rpsa	2.6494E-35	0.37619865	1	0.965	5.8125E-31
Ddost	3.3599E-35	0.86848388	0.397	0.183	7.3712E-31
Itgb3bp	4.2682E-35	1.37940689	0.109	0.025	9.364E-31
Mrpl40	4.301E-35	1.12746125	0.2	0.065	9.4359E-31
Kcnmb4	4.345E-35	1.14923495	0.148	0.04	9.5326E-31
Timm50	4.6333E-35	1.29581531	0.17	0.051	1.0165E-30
Zcrb1	5.8165E-35	0.6527547	0.564	0.298	1.2761E-30
Meaf6	6.7589E-35	1.25214216	0.184	0.058	1.4828E-30
Yaf2	7.3858E-35	0.95662264	0.213	0.071	1.6204E-30
Haus5	7.6612E-35	1.71340796	0.073	0.013	1.6808E-30
Rbx1	7.8794E-35	0.64534075	0.733	0.444	1.7287E-30
Hist1h2bc	8.2408E-35	1.4071213	0.236	0.087	1.8079E-30
Kars	8.5188E-35	1.01777604	0.256	0.096	1.8689E-30
Tiprl	9.4738E-35	0.42878336	0.422	0.184	2.0785E-30
Pfdn4	1.1223E-34	1.04089481	0.256	0.097	2.4621E-30
Frg1	1.2E-34	0.60433582	0.485	0.233	2.6327E-30
Rpp30	1.2669E-34	1.47667057	0.132	0.035	2.7794E-30
Sod1	1.6599E-34	0.52845282	0.821	0.519	3.6416E-30
Gnb2	1.9302E-34	0.57456381	0.714	0.403	4.2347E-30
Trp53	1.994E-34	0.69371247	0.377	0.164	4.3747E-30
Lin54	2.0637E-34	0.74569746	0.415	0.191	4.5276E-30
Ilf2	2.2269E-34	0.59413564	0.401	0.178	4.8856E-30
BC052040	2.4681E-34	2.061009	0.055	0.008	5.4147E-30
Dok2	2.524E-34	0.99382453	0.234	0.084	5.5374E-30
Iqgap2	3.0718E-34	0.82052417	0.338	0.145	6.7392E-30
Arl6ip4	3.26E-34	0.96554366	0.267	0.102	7.152E-30
Ndufb5	3.3519E-34	0.61523206	0.653	0.358	7.3537E-30
Mdh2	3.4648E-34	0.58350174	0.585	0.306	7.6013E-30
Etfb	3.5099E-34	0.90068219	0.358	0.16	7.7003E-30
Etfa	3.7491E-34	0.97342674	0.288	0.116	8.2253E-30
Insl6	4.3796E-34	1.37581165	0.161	0.048	9.6084E-30
Wdr62	4.8072E-34	2.90043218	0.045	0.005	1.0547E-29
Egln1	5.1133E-34	0.94822853	0.265	0.101	1.1218E-29
Fopnl	5.9656E-34	0.79444253	0.225	0.078	1.3088E-29
Chrac1	5.9833E-34	0.78179656	0.397	0.183	1.3127E-29
Supt4a	6.3946E-34	0.57271974	0.753	0.452	1.4029E-29
Nsmce2	6.4894E-34	1.13994905	0.22	0.078	1.4237E-29
Mrpl43	8.042E-34	0.78727276	0.374	0.168	1.7643E-29
Rer1	8.4349E-34	0.74993868	0.436	0.21	1.8505E-29
Plekhj1	8.5472E-34	0.81784332	0.395	0.184	1.8752E-29
Aars	9.8534E-34	0.69709578	0.399	0.181	2.1617E-29
Orai1	1.0023E-33	0.38137856	0.64	0.328	2.199E-29
Vcpkmt	1.2278E-33	0.91756794	0.209	0.07	2.6936E-29

Ddx39b	1.2533E-33	0.67823732	0.435	0.207	2.7497E-29
Nup107	1.3389E-33	1.33194985	0.097	0.021	2.9374E-29
Gpsm2	1.5468E-33	2.39326869	0.059	0.009	3.3936E-29
Ndufa12	1.556E-33	0.80073094	0.377	0.173	3.4136E-29
Sap30	1.6035E-33	1.24731018	0.236	0.088	3.518E-29
Naa10	1.8274E-33	1.07462139	0.272	0.108	4.009E-29
Slc2a3	1.9438E-33	0.78751406	0.182	0.057	4.2646E-29
Lnpk	2.0786E-33	0.91183168	0.233	0.083	4.5603E-29
Lsm7	2.2051E-33	0.93903966	0.279	0.112	4.8378E-29
Lsm1	2.2193E-33	0.96357558	0.22	0.077	4.8689E-29
Gimap4	2.4548E-33	0.37788408	0.585	0.283	5.3856E-29
Fbxl14	2.9846E-33	1.03627786	0.182	0.058	6.5479E-29
Gzmc	3.2208E-33	0.72724302	0.088	0.018	7.0661E-29
Slc25a4	3.6282E-33	0.71644219	0.479	0.242	7.9599E-29
Mbd4	3.9361E-33	1.40014128	0.084	0.017	8.6354E-29
Eny2	4.6526E-33	0.65933794	0.458	0.223	1.0207E-28
Cetn3	4.7046E-33	0.76902598	0.381	0.175	1.0321E-28
Rnf157	4.9369E-33	0.46437433	0.444	0.201	1.0831E-28
Mrps33	5.1111E-33	0.72400388	0.454	0.225	1.1213E-28
Memo1	5.7306E-33	1.23335109	0.15	0.043	1.2572E-28
Gabarapl2	6.0504E-33	0.6179081	0.63	0.351	1.3274E-28
AU020206	6.1341E-33	0.40588038	0.544	0.264	1.3458E-28
Plcxd2	6.8491E-33	0.51106608	0.388	0.168	1.5026E-28
Prdx2	8.1752E-33	0.65153586	0.547	0.288	1.7936E-28
Abhd17c	8.6376E-33	0.695782	0.218	0.075	1.895E-28
Zc3h15	9.8271E-33	0.50667023	0.676	0.382	2.156E-28
Recql4	9.9525E-33	3.12406011	0.029	0.002	2.1835E-28
Ahi1	1.0691E-32	0.98797367	0.22	0.077	2.3455E-28
Utp23	1.1361E-32	1.14357467	0.197	0.066	2.4924E-28
Eftud2	1.1701E-32	0.88702936	0.25	0.094	2.5671E-28
Ndufv1	1.2795E-32	0.94898321	0.268	0.107	2.807E-28
Rab2a	1.3332E-32	0.5602407	0.726	0.434	2.9249E-28
Nt5c	1.3452E-32	0.84750492	0.379	0.177	2.9512E-28
Nudt1	1.5671E-32	2.20400204	0.068	0.012	3.438E-28
Polr2h	1.5714E-32	1.08180441	0.22	0.08	3.4474E-28
Pym1	1.6421E-32	0.99576262	0.234	0.086	3.6026E-28
Wdr83os	1.9249E-32	0.72266882	0.428	0.206	4.2231E-28
Slc39a4	2.1518E-32	1.17955118	0.107	0.025	4.7209E-28
Lcp2	2.1603E-32	0.34453494	0.546	0.27	4.7395E-28
Nkap	2.9989E-32	0.78792429	0.275	0.109	6.5794E-28
Psmc5	3.1943E-32	0.77942501	0.372	0.17	7.0079E-28
Tox2	3.5711E-32	1.0376179	0.145	0.041	7.8346E-28
Hat1	3.6481E-32	1.12078957	0.272	0.111	8.0036E-28
Cript	3.9901E-32	0.48537461	0.644	0.352	8.754E-28
Desi1	4.1341E-32	1.01949849	0.193	0.065	9.0697E-28
Orc2	4.3883E-32	1.13100181	0.159	0.048	9.6276E-28
Rfxap	4.5117E-32	0.93169473	0.254	0.098	9.8982E-28
Psmg2	4.652E-32	1.28275272	0.138	0.038	1.0206E-27

Prpf4	4.8394E-32	0.70877602	0.245	0.09	1.0617E-27
Psmd14	5.1346E-32	0.70613898	0.379	0.174	1.1265E-27
Taf12	6.7359E-32	1.05884206	0.181	0.059	1.4778E-27
Cnih4	7.1721E-32	0.73264272	0.381	0.179	1.5735E-27
Rbm44	7.5393E-32	4.52434486	0.011	0	1.654E-27
Ing2	7.9724E-32	0.67733945	0.342	0.148	1.7491E-27
Taf6	8.7381E-32	1.16463201	0.123	0.032	1.9171E-27
Sarnp	9.277E-32	0.66205542	0.47	0.235	2.0353E-27
Dera	1.0476E-31	1.11640091	0.184	0.061	2.2984E-27
Hdgfl2	1.0593E-31	1.1263074	0.174	0.056	2.3241E-27
Atf1	1.0621E-31	0.71933577	0.358	0.16	2.3302E-27
Cdc25a	1.1458E-31	1.4465884	0.122	0.032	2.5138E-27
Abhd17a	1.1634E-31	0.83427011	0.284	0.116	2.5524E-27
Pank2	1.2641E-31	0.78575954	0.317	0.135	2.7732E-27
Ddt	1.5826E-31	0.72466555	0.37	0.171	3.4721E-27
Lmnb2	1.6145E-31	2.05289144	0.055	0.008	3.5419E-27
Ddx46	1.6712E-31	0.56638852	0.567	0.301	3.6664E-27
Cep192	1.7071E-31	0.78520115	0.216	0.076	3.7453E-27
Med4	1.7492E-31	0.73030456	0.258	0.098	3.8377E-27
Mrps21	2.0535E-31	0.70401064	0.463	0.236	4.5052E-27
Faah	2.1583E-31	0.5815451	0.274	0.105	4.7352E-27
Tmco1	2.3735E-31	0.72723949	0.379	0.179	5.2071E-27
Churc1	2.7973E-31	0.63992602	0.436	0.212	6.137E-27
Stk39	2.8068E-31	0.94083726	0.165	0.051	6.1578E-27
Prdx1	2.9078E-31	0.55968746	0.696	0.422	6.3795E-27
Mrpl42	3.0562E-31	1.03635748	0.261	0.106	6.705E-27
Med10	3.2905E-31	0.80699592	0.338	0.15	7.2191E-27
Naa20	3.523E-31	0.87698495	0.261	0.103	7.7291E-27
Pole2	3.7501E-31	2.59451105	0.043	0.005	8.2272E-27
Angel2	3.8688E-31	0.5664007	0.351	0.152	8.4878E-27
Tap1	4.4523E-31	0.6531713	0.497	0.262	9.768E-27
Rbpj	5.1438E-31	0.54480208	0.721	0.437	1.1285E-26
Stk24	5.2747E-31	0.44840157	0.619	0.333	1.1572E-26
Ppp1r35	5.5868E-31	1.3216088	0.141	0.041	1.2257E-26
Cdc34	5.6714E-31	0.69056797	0.352	0.158	1.2443E-26
Ints11	5.941E-31	1.3994379	0.123	0.033	1.3034E-26
Cox4i1	6.5719E-31	0.41040724	0.989	0.847	1.4418E-26
Mrpl21	6.9846E-31	0.67188807	0.374	0.172	1.5323E-26
Gars	7.1965E-31	0.75216957	0.317	0.136	1.5788E-26
Celf2	7.404E-31	0.45004841	0.825	0.542	1.6244E-26
Psma5	8.0999E-31	0.73100208	0.487	0.254	1.777E-26
Nop58	8.1615E-31	0.61905467	0.415	0.197	1.7905E-26
Slc29a1	8.7594E-31	1.17175989	0.156	0.048	1.9217E-26
Mrps26	9.2327E-31	0.87745226	0.243	0.093	2.0256E-26
Rbm43	9.7865E-31	1.3579602	0.131	0.036	2.1471E-26
Fkbp5	9.9386E-31	1.26419398	0.12	0.031	2.1804E-26
Dcun1d5	1.0421E-30	0.66437877	0.521	0.277	2.2863E-26
Itgb3	1.2121E-30	0.51757531	0.283	0.111	2.6591E-26

Tdp2	1.2788E-30	0.82684989	0.202	0.07	2.8055E-26
Enkd1	1.3416E-30	2.70104996	0.045	0.006	2.9433E-26
Sytl1	1.4185E-30	1.49918695	0.093	0.021	3.112E-26
Gm8186	1.4984E-30	0.97531624	0.209	0.075	3.2873E-26
Ntan1	1.5469E-30	0.7922848	0.329	0.146	3.3937E-26
Cep97	1.554E-30	1.17874905	0.111	0.028	3.4094E-26
Psmd9	1.6247E-30	0.90648253	0.236	0.09	3.5645E-26
Slain1	1.8168E-30	0.55355869	0.202	0.069	3.9858E-26
Rnf138	1.8413E-30	0.48081567	0.422	0.195	4.0396E-26
1110008F13Ri	1.8578E-30	0.74211782	0.417	0.207	4.0758E-26
Rbm22	2.0334E-30	0.56208844	0.458	0.224	4.4611E-26
Cdc37	2.1226E-30	0.55452849	0.619	0.346	4.6567E-26
Mrpl49	2.4958E-30	1.23698638	0.132	0.037	5.4756E-26
Strap	2.6007E-30	0.64064729	0.388	0.182	5.7057E-26
Ndufs8	2.7699E-30	0.83894664	0.363	0.171	6.0769E-26
Emc7	2.811E-30	0.57097029	0.53	0.276	6.167E-26
Anxa2	2.8893E-30	0.32418575	0.596	0.316	6.3388E-26
Ssb	3.0992E-30	0.46094221	0.674	0.382	6.7993E-26
Sept11	3.2062E-30	0.55725947	0.451	0.22	7.034E-26
Pik3r1	3.3215E-30	0.39440481	0.658	0.363	7.2869E-26
Gps2	3.4876E-30	0.78490559	0.327	0.145	7.6514E-26
Gmps	4.0444E-30	0.75671975	0.317	0.138	8.873E-26
Psma7	4.5514E-30	0.54717817	0.803	0.519	9.9854E-26
Acsbg1	5.0958E-30	1.18130307	0.136	0.039	1.118E-25
Pdcd6	5.2619E-30	0.55317519	0.515	0.268	1.1544E-25
2610524H06R	5.4228E-30	1.58211312	0.136	0.04	1.1897E-25
Got2	5.5097E-30	0.85646579	0.308	0.135	1.2088E-25
Cnih1	5.6412E-30	0.84942946	0.249	0.098	1.2376E-25
Mrpl55	5.9583E-30	1.15130245	0.17	0.056	1.3072E-25
Fam111a	6.1363E-30	0.56654512	0.297	0.123	1.3462E-25
Ufc1	6.3202E-30	0.6969497	0.462	0.237	1.3866E-25
Sla	6.3436E-30	0.44561	0.696	0.402	1.3917E-25
Akr1b3	6.7693E-30	0.69161316	0.408	0.198	1.4851E-25
Slc22a15	7.2506E-30	0.96403361	0.175	0.058	1.5907E-25
Ccng2	7.6414E-30	0.67931857	0.249	0.096	1.6765E-25
Inpp1	7.6936E-30	0.73273538	0.216	0.078	1.6879E-25
Taf15	8.1592E-30	0.59893703	0.397	0.188	1.79E-25
Ncbp1	8.1945E-30	0.87470272	0.211	0.076	1.7978E-25
Ndufs7	8.4508E-30	0.66704254	0.411	0.201	1.854E-25
Mrpl12	9.8538E-30	0.79718282	0.315	0.139	2.1618E-25
Phf20l1	9.8538E-30	0.49280884	0.578	0.309	2.1618E-25
Srp14	1.0092E-29	0.50372745	0.732	0.437	2.2141E-25
Zbtb38	1.0489E-29	0.90902578	0.222	0.083	2.3012E-25
Cacybp	1.2117E-29	0.57551411	0.383	0.179	2.6584E-25
Cldnd1	1.3782E-29	0.47845344	0.465	0.236	3.0236E-25
Edem1	1.6247E-29	0.50870682	0.526	0.28	3.5645E-25
Mrpl46	1.7009E-29	0.96835548	0.206	0.075	3.7316E-25
Actg1	1.7616E-29	0.30913122	0.996	0.913	3.8648E-25

Ero1l	1.905E-29	0.53817572	0.381	0.177	4.1793E-25
Npm3	2.0688E-29	0.61662266	0.381	0.18	4.5388E-25
Nap1l4	2.1726E-29	0.63953591	0.392	0.186	4.7665E-25
Hnrnpu	2.2666E-29	0.53207931	0.8	0.531	4.9726E-25
Lamc1	2.3291E-29	0.49761636	0.256	0.098	5.1097E-25
Ssbp4	3.0377E-29	0.87934759	0.27	0.112	6.6644E-25
Dctn4	3.0797E-29	0.53872475	0.594	0.331	6.7566E-25
Ube2j1	3.0956E-29	0.51562311	0.481	0.244	6.7915E-25
Snrnp27	3.1885E-29	0.56117429	0.404	0.194	6.9953E-25
Dnajc1	3.5576E-29	0.61013975	0.411	0.198	7.805E-25
Eif3i	3.7697E-29	0.58069011	0.578	0.321	8.2703E-25
D8Ertd738e	4.1184E-29	0.38414231	0.857	0.541	9.0353E-25
Cdc5l	4.1405E-29	0.58190218	0.481	0.248	9.0838E-25
Prmt1	4.2603E-29	0.84086915	0.272	0.114	9.3466E-25
Batf	4.5608E-29	0.66086287	0.379	0.18	1.0006E-24
Ap2m1	4.6367E-29	0.71055535	0.426	0.215	1.0173E-24
Emc4	4.8311E-29	0.96639983	0.231	0.09	1.0599E-24
Timm13	5.4984E-29	0.57246234	0.601	0.339	1.2063E-24
Arpc5l	5.5267E-29	0.68788117	0.302	0.131	1.2125E-24
Nrgn	5.779E-29	0.81538249	0.134	0.039	1.2679E-24
Ddx1	5.7931E-29	1.00456056	0.213	0.08	1.2709E-24
Vgll4	5.8202E-29	0.3820059	0.606	0.318	1.2769E-24
Leo1	5.8414E-29	1.03805024	0.166	0.054	1.2815E-24
Clint1	5.9041E-29	0.44376558	0.769	0.48	1.2953E-24
Med14	5.9671E-29	0.83346943	0.229	0.087	1.3091E-24
Stk4	6.5451E-29	0.25878029	0.723	0.395	1.4359E-24
Myl12b	6.7628E-29	0.45810899	0.821	0.547	1.4837E-24
Tnk2	6.8225E-29	0.85767978	0.138	0.04	1.4968E-24
Larp1b	7.1684E-29	0.7378924	0.25	0.1	1.5727E-24
Jakmip1	7.206E-29	0.47342177	0.333	0.141	1.5809E-24
Uqcc2	7.2694E-29	0.71199716	0.404	0.201	1.5948E-24
Dpm3	7.8476E-29	0.5354131	0.488	0.249	1.7217E-24
Psma6	7.9792E-29	0.5942225	0.458	0.235	1.7506E-24
Ebna1bp2	8.4577E-29	0.73768624	0.309	0.136	1.8555E-24
Ube2d2a	8.5118E-29	0.53420954	0.633	0.364	1.8674E-24
Trim59	8.8053E-29	0.51746221	0.268	0.106	1.9318E-24
Sytl3	9.1432E-29	0.43422986	0.315	0.133	2.0059E-24
Gatad1	9.5121E-29	0.7266662	0.274	0.114	2.0869E-24
Diaph1	1.0739E-28	0.4524252	0.753	0.458	2.3561E-24
Arl15	1.0971E-28	1.31933984	0.106	0.027	2.4069E-24
2010107E04R	1.169E-28	0.49726759	0.866	0.594	2.5646E-24
Srsf7	1.2454E-28	0.69411449	0.488	0.265	2.7323E-24
Pnn	1.3087E-28	0.46421033	0.49	0.251	2.8713E-24
Mrpl4	1.4276E-28	0.86496163	0.261	0.108	3.1321E-24
Prdx6	1.4975E-28	0.38760857	0.814	0.513	3.2854E-24
Fuom	1.5502E-28	1.59975549	0.107	0.028	3.401E-24
Zc3h13	1.566E-28	0.54712112	0.537	0.289	3.4356E-24
Gtf2f1	1.5814E-28	0.86281601	0.304	0.136	3.4695E-24

Rnf168	1.603E-28	1.11529522	0.152	0.048	3.5169E-24
Emc8	1.6611E-28	1.16719888	0.154	0.049	3.6442E-24
Gmcl1	1.6859E-28	0.9920003	0.141	0.043	3.6986E-24
Rtel1	1.7034E-28	1.81936084	0.061	0.011	3.737E-24
Sfr1	1.7207E-28	0.6293277	0.513	0.28	3.775E-24
Mdh1	1.7489E-28	0.60667068	0.504	0.271	3.8368E-24
Ndufa9	1.8673E-28	0.91683919	0.259	0.108	4.0968E-24
Uqcrc1	1.871E-28	0.79271481	0.395	0.197	4.1048E-24
Skp1a	1.892E-28	0.5250044	0.451	0.225	4.1509E-24
Nhp2	1.9003E-28	0.71527416	0.369	0.178	4.169E-24
Twistnb	1.9668E-28	0.57442479	0.413	0.202	4.3149E-24
Tbc1d10b	2.0526E-28	0.91780284	0.199	0.072	4.5032E-24
Prpf8	2.3801E-28	0.63264955	0.413	0.205	5.2217E-24
Mpc2	2.7366E-28	0.57108616	0.481	0.251	6.0038E-24
Tspo	2.7988E-28	0.48788629	0.75	0.462	6.1403E-24
Pon2	2.9446E-28	0.74667228	0.324	0.146	6.4602E-24
Rasgrp1	2.985E-28	0.50266474	0.354	0.158	6.5487E-24
Gramd3	3.007E-28	0.41011669	0.513	0.266	6.5972E-24
Tmem192	3.24E-28	0.84757946	0.218	0.083	7.1083E-24
Lsm12	3.2955E-28	0.64892033	0.549	0.309	7.2301E-24
H2afj	3.4422E-28	0.45830203	0.801	0.503	7.5518E-24
Sap30l	3.7841E-28	0.86656442	0.249	0.1	8.3018E-24
Haus1	3.8259E-28	2.06513366	0.061	0.011	8.3937E-24
Srek1ip1	3.9816E-28	1.20597896	0.15	0.048	8.7352E-24
Trib2	4.0942E-28	0.4512723	0.301	0.125	8.9822E-24
Cfap36	4.371E-28	0.81840568	0.227	0.088	9.5896E-24
Uap1	4.8668E-28	0.57781756	0.343	0.158	1.0677E-23
Ppp2r5c	5.0653E-28	0.57714529	0.508	0.275	1.1113E-23
Ints7	5.4608E-28	0.83859762	0.17	0.057	1.1981E-23
Fam221a	5.7406E-28	2.15915451	0.034	0.004	1.2594E-23
Tcp1	5.7621E-28	0.51486456	0.553	0.301	1.2642E-23
Vdac3	5.8975E-28	0.75594923	0.331	0.153	1.2939E-23
Rbm17	5.9161E-28	0.57654442	0.413	0.204	1.2979E-23
Cdkn2d	6.2903E-28	0.96762359	0.211	0.08	1.38E-23
Tmx4	6.3276E-28	0.56658433	0.302	0.129	1.3882E-23
Gm47015	6.5903E-28	1.28530344	0.136	0.041	1.4459E-23
Sgf29	6.5924E-28	1.17820769	0.122	0.034	1.4463E-23
Polr2k	6.7146E-28	0.55294459	0.488	0.259	1.4731E-23
Phb2	8.926E-28	0.60729015	0.386	0.187	1.9583E-23
Cnn2	9.1244E-28	0.50466868	0.517	0.272	2.0018E-23
Ubn1	9.272E-28	0.58106737	0.496	0.263	2.0342E-23
Zc3hav1	1.0112E-27	0.35260447	0.751	0.443	2.2184E-23
Mlec	1.0283E-27	0.61238983	0.322	0.144	2.256E-23
Aimp1	1.0726E-27	0.53284057	0.553	0.301	2.3533E-23
Faap20	1.0735E-27	1.05898516	0.145	0.045	2.3552E-23
Bcas2	1.1232E-27	0.50645287	0.494	0.256	2.4642E-23
Fasl	1.1238E-27	0.59173479	0.256	0.102	2.4655E-23
Dnajc21	1.192E-27	0.59800663	0.381	0.183	2.6152E-23

Ebp	1.199E-27	0.88886404	0.268	0.115	2.6306E-23
Atp5l	1.3043E-27	0.39998604	0.957	0.762	2.8614E-23
Polr2b	1.5253E-27	0.85033206	0.19	0.068	3.3464E-23
Cdk19	1.5981E-27	0.77765903	0.213	0.08	3.5061E-23
Cdkn2aip	1.6246E-27	0.64124539	0.218	0.082	3.5643E-23
Fis1	1.9131E-27	0.47799196	0.64	0.372	4.1973E-23
Pds5b	2.0178E-27	0.87143509	0.179	0.062	4.4268E-23
Uqcr11	2.1044E-27	0.55072628	0.546	0.299	4.617E-23
Atg101	2.4289E-27	0.8783014	0.211	0.08	5.3288E-23
Gt(ROSA)26Soı	2.449E-27	0.52413515	0.436	0.22	5.3728E-23
Ikzf1	2.4508E-27	0.44963939	0.445	0.218	5.3768E-23
Ppp3cb	2.6291E-27	0.52710299	0.354	0.163	5.768E-23
Rpn2	3.0957E-27	0.65827342	0.42	0.217	6.7917E-23
Psmc6	3.1046E-27	0.62392876	0.428	0.219	6.8111E-23
Rnf219	3.6722E-27	1.68056729	0.084	0.019	8.0564E-23
Fxyd5	3.7285E-27	0.36351436	0.927	0.658	8.1801E-23
Dpysl2	3.7298E-27	0.69001714	0.358	0.172	8.1829E-23
Mrpl54	3.7335E-27	0.54099765	0.553	0.304	8.191E-23
Pidd1	3.9756E-27	2.78339498	0.025	0.002	8.722E-23
Snapc1	4.2867E-27	0.65519377	0.295	0.129	9.4046E-23
Tnfrsf25	4.7661E-27	1.01819883	0.143	0.044	1.0456E-22
Akip1	4.8196E-27	1.53736036	0.084	0.019	1.0574E-22
Optn	4.986E-27	0.97086019	0.136	0.042	1.0939E-22
Sf3a1	5.3594E-27	0.80682412	0.245	0.1	1.1758E-22
Ppp1r8	5.4923E-27	0.94167837	0.141	0.044	1.2049E-22
Pcif1	5.7472E-27	0.4710757	0.456	0.231	1.2609E-22
Zfp511	6.3534E-27	0.92897255	0.204	0.077	1.3939E-22
Alms1	6.8753E-27	1.98545976	0.057	0.01	1.5084E-22
Clec2i	7.5223E-27	0.7116851	0.193	0.069	1.6503E-22
Atp5e	7.6167E-27	0.37051218	0.984	0.818	1.671E-22
Wbp11	7.9349E-27	0.4406147	0.569	0.312	1.7408E-22
Psmd1	8.1413E-27	0.73664873	0.32	0.148	1.7861E-22
Mpp2	8.4583E-27	2.76892592	0.027	0.003	1.8557E-22
Nup205	8.5209E-27	1.1204691	0.132	0.04	1.8694E-22
Pcnp	8.8032E-27	0.43942993	0.474	0.243	1.9313E-22
Cbx2	9.6644E-27	2.95723285	0.021	0.002	2.1203E-22
BC031181	9.6918E-27	0.44320416	0.513	0.271	2.1263E-22
Kctd17	1.053E-26	1.56003789	0.086	0.02	2.3101E-22
Ccnd3	1.0614E-26	0.76656128	0.254	0.105	2.3287E-22
Polr2d	1.0689E-26	1.09283301	0.156	0.052	2.3451E-22
Tsc22d4	1.1174E-26	0.49119298	0.524	0.278	2.4515E-22
Emc3	1.2161E-26	0.6540307	0.361	0.173	2.6679E-22
Taf5	1.2789E-26	1.05585829	0.125	0.037	2.8059E-22
Sssca1	1.367E-26	1.18321496	0.138	0.043	2.9991E-22
Sla2	1.3704E-26	1.17135901	0.134	0.041	3.0066E-22
Zfp706	1.3997E-26	0.44705397	0.526	0.281	3.0709E-22
Aida	1.416E-26	0.85192568	0.199	0.074	3.1065E-22
Impa1	1.6016E-26	0.71981629	0.268	0.115	3.5137E-22

4833420G17R	1.6915E-26	0.48456962	0.354	0.163	3.711E-22
Uchl3	1.768E-26	0.84281028	0.197	0.074	3.8789E-22
Armc1	1.8254E-26	0.71283082	0.195	0.072	4.0047E-22
Me2	1.8749E-26	0.65522849	0.231	0.092	4.1134E-22
Ola1	1.9117E-26	0.8104681	0.327	0.156	4.194E-22
Gemin2	2.0238E-26	1.42350656	0.088	0.021	4.44E-22
Klrb1f	2.4029E-26	1.31530146	0.072	0.015	5.2717E-22
Acpp	2.644E-26	1.13042492	0.12	0.035	5.8007E-22
Atp2a3	3.2078E-26	0.50797805	0.245	0.097	7.0375E-22
9430015G10R	3.369E-26	1.844351	0.047	0.007	7.3912E-22
AI467606	3.3977E-26	0.82003906	0.211	0.081	7.4542E-22
Pnkd	3.4359E-26	1.06989642	0.195	0.074	7.5381E-22
Cdon	3.8222E-26	1.4649634	0.081	0.018	8.3854E-22
Apol7e	4.5252E-26	1.38507581	0.086	0.02	9.9278E-22
Pomp	4.545E-26	0.47572139	0.716	0.432	9.9713E-22
Pcbp4	4.6851E-26	1.66444355	0.059	0.011	1.0279E-21
Senp6	4.8606E-26	0.40397728	0.631	0.356	1.0664E-21
Lyrm9	5.5795E-26	1.25646664	0.104	0.028	1.2241E-21
Fkbp1a	5.655E-26	0.58419805	0.519	0.289	1.2407E-21
Snrpa	5.6844E-26	0.83129329	0.252	0.108	1.2471E-21
Cep89	5.7122E-26	2.03421162	0.05	0.008	1.2532E-21
Arhgap31	6.3908E-26	0.37771091	0.447	0.226	1.4021E-21
Lgalsl	6.6693E-26	1.39314423	0.073	0.016	1.4632E-21
Fanca	7.2395E-26	2.29826297	0.043	0.006	1.5883E-21
1700020I14Ril	7.4383E-26	0.4778132	0.41	0.201	1.6319E-21
Nop56	7.4708E-26	0.48099894	0.411	0.203	1.639E-21
Mrps23	7.7403E-26	0.70951374	0.299	0.135	1.6982E-21
Pknox1	7.7665E-26	0.83408823	0.186	0.068	1.7039E-21
Sv2c	7.789E-26	1.39817053	0.082	0.019	1.7088E-21
Cdk17	7.8857E-26	0.45927015	0.318	0.141	1.73E-21
Stk32c	7.9094E-26	1.58583114	0.086	0.021	1.7352E-21
Bckdk	8.151E-26	1.37946743	0.114	0.033	1.7882E-21
Cct2	9.7231E-26	0.46341515	0.707	0.422	2.1331E-21
Lpxn	1.0343E-25	0.46258334	0.458	0.234	2.2691E-21
Ubash3a	1.0539E-25	0.82250838	0.15	0.049	2.3121E-21
Polr2i	1.1182E-25	0.73227844	0.284	0.128	2.4532E-21
Bnip3l	1.1727E-25	0.41253306	0.569	0.314	2.5728E-21
Sclt1	1.1979E-25	1.19910461	0.129	0.04	2.6281E-21
Setx	1.2018E-25	0.54043022	0.245	0.1	2.6367E-21
Myg1	1.2897E-25	1.29729222	0.132	0.042	2.8294E-21
Snx3	1.3045E-25	0.49549554	0.59	0.337	2.8619E-21
Gm39323	1.3379E-25	2.025442	0.038	0.005	2.9352E-21
Cmpk1	1.5477E-25	0.46063168	0.528	0.289	3.3954E-21
Nudcd2	1.6981E-25	0.96261955	0.191	0.073	3.7254E-21
Mcph1	1.806E-25	1.25093811	0.1	0.027	3.9623E-21
Mlf2	1.8374E-25	0.6964097	0.32	0.152	4.031E-21
Ing5	1.9997E-25	0.68812875	0.274	0.12	4.3872E-21
Ppme1	2.0454E-25	0.57932916	0.249	0.103	4.4873E-21

Sacs	2.0579E-25	0.81389637	0.17	0.059	4.5148E-21
Gmds	2.1096E-25	1.3179348	0.125	0.038	4.6283E-21
Spsb3	2.1399E-25	0.71841721	0.184	0.067	4.6948E-21
Dnah8	2.4751E-25	1.66384241	0.048	0.008	5.4302E-21
Wee1	2.5771E-25	1.47171691	0.079	0.018	5.6539E-21
Psma3	2.5821E-25	0.43659786	0.751	0.462	5.6648E-21
Cntrob	2.596E-25	1.53902834	0.073	0.016	5.6953E-21
Pms2	2.6656E-25	1.16287154	0.114	0.033	5.848E-21
Ctbp1	2.8426E-25	0.62421888	0.39	0.196	6.2364E-21
Psmd8	3.0931E-25	0.51961521	0.526	0.291	6.786E-21
Ccdc82	3.1453E-25	0.55813405	0.233	0.093	6.9005E-21
Tial1	3.3037E-25	0.65529928	0.292	0.132	7.2481E-21
Hras	3.315E-25	1.03231981	0.19	0.072	7.2728E-21
Eif2d	3.4302E-25	1.19844236	0.129	0.04	7.5255E-21
B2m	3.7039E-25	0.25777008	0.998	0.979	8.1259E-21
Prkrip1	3.7086E-25	0.83113652	0.209	0.083	8.1362E-21
Ywhab	3.7676E-25	0.4752636	0.596	0.349	8.2657E-21
Selplg	3.811E-25	0.33964387	0.696	0.41	8.361E-21
Trim37	3.8584E-25	1.60763198	0.081	0.019	8.465E-21
Tnks2	4.1134E-25	0.38842574	0.497	0.261	9.0243E-21
Taf1d	4.1576E-25	0.44734965	0.637	0.378	9.1214E-21
Cep72	4.2635E-25	2.76489683	0.036	0.005	9.3536E-21
Ccng1	4.3119E-25	0.81357605	0.268	0.119	9.4599E-21
Anapc16	4.3977E-25	0.73246056	0.27	0.119	9.6481E-21
Phf19	4.4077E-25	2.18291421	0.036	0.005	9.6701E-21
Ddx19a	4.417E-25	0.96800483	0.177	0.065	9.6904E-21
Mettl9	4.4824E-25	0.76346524	0.186	0.07	9.8339E-21
Hip1r	4.5234E-25	0.44098842	0.304	0.133	9.924E-21
Ccdc12	4.7064E-25	0.41751122	0.581	0.325	1.0325E-20
Mrpl9	4.9388E-25	0.53685169	0.367	0.178	1.0835E-20
Syncrip	5.064E-25	0.33165247	0.61	0.339	1.111E-20
Gabpb2	5.4813E-25	0.57181876	0.277	0.121	1.2025E-20
Nup160	5.7316E-25	0.97159021	0.12	0.036	1.2575E-20
Pradc1	6.0202E-25	1.70068535	0.063	0.013	1.3208E-20
Slfn9	6.3457E-25	2.10367388	0.059	0.011	1.3922E-20
Itgal	6.4421E-25	0.30547229	0.583	0.318	1.4133E-20
Ucp2	6.9308E-25	0.46732234	0.825	0.532	1.5205E-20
Gtf2a2	6.965E-25	0.56329586	0.311	0.143	1.5281E-20
Cd2bp2	7.2199E-25	0.64484261	0.272	0.119	1.584E-20
Ccdc25	7.2282E-25	0.68869588	0.243	0.102	1.5858E-20
Chchd3	7.5161E-25	0.68651955	0.304	0.141	1.649E-20
Ern1	7.6579E-25	0.26210687	0.492	0.255	1.6801E-20
Yipf4	8.4853E-25	0.51734391	0.351	0.168	1.8616E-20
Fzr1	8.6907E-25	1.09198736	0.15	0.051	1.9067E-20
Dph3	8.9786E-25	0.41685512	0.522	0.287	1.9698E-20
Fgfr1op2	1.0046E-24	0.4893245	0.542	0.305	2.204E-20
Bsg	1.0866E-24	0.54871575	0.503	0.279	2.3839E-20
Ier3ip1	1.0958E-24	0.50853979	0.528	0.299	2.404E-20

Gm9844	1.1204E-24	1.25094787	0.104	0.029	2.458E-20
Cyth2	1.1622E-24	0.74497103	0.259	0.113	2.5498E-20
Pdpk1	1.2346E-24	0.65958617	0.281	0.125	2.7086E-20
Arl2bp	1.2649E-24	0.47201063	0.342	0.16	2.7751E-20
Cmtm4	1.2704E-24	1.71522241	0.07	0.015	2.7871E-20
Umps	1.2798E-24	1.13849858	0.116	0.034	2.8079E-20
Fmc1	1.3915E-24	0.90720599	0.209	0.084	3.0529E-20
Lage3	1.3947E-24	1.07504978	0.161	0.058	3.0599E-20
Psmd12	1.4013E-24	0.61922655	0.361	0.18	3.0742E-20
Pycard	1.452E-24	0.74613951	0.369	0.19	3.1854E-20
Ntrk3	1.4727E-24	1.5018531	0.088	0.022	3.231E-20
Csnk1g2	1.4823E-24	0.53755943	0.329	0.155	3.2521E-20
Cbx4	2.0365E-24	0.44758815	0.275	0.118	4.4679E-20
Glrx5	2.1956E-24	0.70602895	0.286	0.131	4.8169E-20
Kcnk7	2.3665E-24	2.04604686	0.041	0.006	5.192E-20
H2-Q4	2.4636E-24	0.4366645	0.483	0.255	5.405E-20
Lgals7	2.5363E-24	1.30851044	0.089	0.023	5.5643E-20
Sh3glb2	2.5874E-24	1.05378758	0.131	0.042	5.6766E-20
Commd3	2.7228E-24	0.76065249	0.24	0.103	5.9735E-20
Seh1l	2.7432E-24	0.85643181	0.236	0.101	6.0183E-20
Xrn2	2.7642E-24	0.41641923	0.547	0.308	6.0645E-20
Mrps11	2.9016E-24	1.12750764	0.141	0.047	6.3658E-20
Gle1	2.9198E-24	0.78257542	0.17	0.061	6.4057E-20
Mllt3	3.1664E-24	0.4690956	0.265	0.112	6.9467E-20
Pigx	3.197E-24	0.83807346	0.195	0.076	7.0138E-20
1110004F10Ri	3.226E-24	0.45018991	0.551	0.313	7.0775E-20
Birc2	3.2523E-24	0.448512	0.578	0.329	7.1353E-20
Il1r1	3.3182E-24	1.03876061	0.132	0.042	7.2797E-20
Mrpl15	3.3183E-24	0.76943902	0.272	0.122	7.2801E-20
Def6	3.8706E-24	0.8735325	0.154	0.053	8.4917E-20
Eif3d	3.8882E-24	0.48759717	0.419	0.216	8.5304E-20
Pfdn2	3.934E-24	0.47350249	0.542	0.302	8.6308E-20
Adh5	4.1469E-24	0.65178612	0.29	0.134	9.0978E-20
Prkcsh	4.3823E-24	0.68337081	0.306	0.145	9.6143E-20
Cops5	4.5432E-24	0.56493097	0.333	0.16	9.9673E-20
Slc35d1	4.815E-24	1.195444	0.129	0.041	1.0564E-19
Rab39b	5.1901E-24	1.66722176	0.066	0.014	1.1386E-19
Zfpm1	5.4203E-24	1.03084461	0.118	0.036	1.1892E-19
Ccdc127	5.5317E-24	0.69449352	0.218	0.088	1.2136E-19
Zfp266	7.3126E-24	0.62426193	0.222	0.089	1.6043E-19
Knop1	7.3961E-24	0.68891624	0.22	0.09	1.6226E-19
Sub1	7.4394E-24	0.31954483	0.971	0.813	1.6321E-19
Dcp2	7.6612E-24	0.68505178	0.268	0.12	1.6808E-19
Tm2d2	8.1291E-24	0.82422964	0.277	0.128	1.7834E-19
Bcl2a1d	8.1579E-24	0.31683372	0.592	0.333	1.7898E-19
Rassf5	8.2535E-24	0.4938398	0.302	0.138	1.8107E-19
Mrpl32	8.4299E-24	0.53313225	0.431	0.228	1.8494E-19
Ndufa10	8.8844E-24	0.67380291	0.356	0.181	1.9492E-19

Htatsf1	9.0065E-24	0.7274467	0.245	0.106	1.9759E-19
Sephs2	9.5216E-24	1.07587055	0.125	0.039	2.0889E-19
Ncor1	9.6583E-24	0.35881199	0.778	0.504	2.1189E-19
Ube2n	9.797E-24	0.4622046	0.472	0.255	2.1494E-19
Mapre1	9.8547E-24	0.41430025	0.615	0.366	2.162E-19
Mrps17	1.0275E-23	0.83779604	0.25	0.111	2.2542E-19
Nol10	1.0421E-23	0.74793732	0.209	0.084	2.2864E-19
9130401M01F	1.0708E-23	0.85535338	0.188	0.073	2.3491E-19
Mta1	1.1275E-23	0.81998499	0.191	0.074	2.4737E-19
Khdrbs1	1.1349E-23	0.38737229	0.71	0.438	2.49E-19
Hddc2	1.1739E-23	1.23535441	0.123	0.039	2.5753E-19
Fam96a	1.2659E-23	0.64611513	0.327	0.16	2.7772E-19
Zcchc10	1.2864E-23	0.77725659	0.184	0.07	2.8223E-19
P2rx7	1.3089E-23	0.88199046	0.143	0.048	2.8717E-19
Gen1	1.3138E-23	2.22433911	0.03	0.004	2.8823E-19
Smarce1	1.3337E-23	0.42161612	0.497	0.269	2.9261E-19
Nmt1	1.3785E-23	0.46958682	0.544	0.308	3.0242E-19
Suds3	1.4592E-23	0.61437693	0.258	0.113	3.2013E-19
Aggf1	1.5236E-23	0.61115199	0.259	0.114	3.3427E-19
Naxe	1.6199E-23	1.06243952	0.191	0.077	3.5538E-19
Sec61b	1.6241E-23	0.40324924	0.898	0.624	3.5631E-19
Acbd6	1.665E-23	0.72744371	0.279	0.128	3.6529E-19
Ptpa	1.867E-23	0.72082298	0.265	0.12	4.0959E-19
Rbm28	1.9252E-23	0.63189177	0.306	0.145	4.2238E-19
Med11	1.9618E-23	0.71948887	0.25	0.11	4.3041E-19
Rpl15	1.9803E-23	0.3161811	0.996	0.916	4.3447E-19
Podnl1	2.021E-23	0.95662742	0.107	0.031	4.4338E-19
Cklf	2.1485E-23	0.5630281	0.261	0.116	4.7136E-19
Cenpv	2.1885E-23	1.6866659	0.072	0.016	4.8013E-19
Asf1a	2.2277E-23	0.70593306	0.172	0.063	4.8872E-19
Lancl2	2.2301E-23	1.50772813	0.081	0.02	4.8927E-19
Tomm5	2.2468E-23	0.5751962	0.386	0.199	4.9292E-19
Crbn	2.4129E-23	0.39199189	0.356	0.17	5.2936E-19
Xpo4	2.4323E-23	0.64413821	0.222	0.091	5.3363E-19
Cinp	2.4398E-23	1.39698971	0.093	0.025	5.3526E-19
Hmgcr	2.6368E-23	0.46483978	0.403	0.208	5.7848E-19
Paip2	2.7049E-23	0.37205526	0.699	0.426	5.9343E-19
Arl6ip6	2.7466E-23	0.87234371	0.17	0.063	6.0258E-19
Ptbp1	2.8872E-23	0.34822774	0.574	0.323	6.3342E-19
Sin3a	2.9081E-23	0.73695615	0.209	0.085	6.3801E-19
F2r	2.9986E-23	0.54653392	0.186	0.07	6.5786E-19
Ncl	3.3173E-23	0.31048552	0.962	0.802	7.2778E-19
Mrpl34	3.5395E-23	0.77334746	0.236	0.102	7.7652E-19
Mad2l2	3.5396E-23	1.3688337	0.106	0.031	7.7655E-19
Ralbp1	3.6784E-23	0.49686584	0.429	0.23	8.0701E-19
Prr13	3.7911E-23	0.33716812	0.696	0.423	8.3173E-19
Tex2	3.7936E-23	0.39566038	0.39	0.193	8.3227E-19
Cmss1	4.034E-23	1.04198926	0.109	0.032	8.8501E-19

Tmem59	4.1343E-23	0.51925795	0.46	0.253	9.0702E-19
Lta4h	4.4144E-23	0.83054463	0.195	0.078	9.6848E-19
Manf	4.447E-23	0.3678459	0.753	0.462	9.7563E-19
4930522L14Ri	4.5242E-23	1.42079936	0.075	0.018	9.9257E-19
Terf1	4.627E-23	0.55386394	0.197	0.077	1.0151E-18
Ndufa7	4.7644E-23	0.4558148	0.701	0.441	1.0453E-18
Cdca4	4.7907E-23	1.12165328	0.154	0.055	1.051E-18
Ryk	4.863E-23	1.22059138	0.081	0.02	1.0669E-18
Ckb	4.9132E-23	0.56678819	0.356	0.179	1.0779E-18
Fdft1	4.9561E-23	1.17056659	0.107	0.032	1.0873E-18
Smim11	4.9904E-23	0.59004315	0.292	0.135	1.0948E-18
Stard3nl	5.0256E-23	0.83640676	0.225	0.097	1.1026E-18
Ppp4c	5.3296E-23	0.43736848	0.44	0.235	1.1693E-18
Smarcd1	5.5598E-23	0.80001794	0.186	0.072	1.2198E-18
Rac1	6.2987E-23	0.41542443	0.785	0.529	1.3819E-18
Cbx8	6.8136E-23	1.54470396	0.059	0.012	1.4948E-18
Tm2d3	7.5971E-23	0.84228885	0.175	0.067	1.6667E-18
Psmf1	7.9815E-23	1.02433568	0.156	0.056	1.7511E-18
Carmil2	8.31E-23	1.11556811	0.089	0.024	1.8231E-18
Zwint	8.3576E-23	0.84259669	0.19	0.075	1.8336E-18
Cnot9	8.8046E-23	1.02776667	0.127	0.041	1.9316E-18
Haus7	9.3032E-23	1.42422092	0.082	0.021	2.041E-18
Top2b	9.3117E-23	0.4189438	0.533	0.299	2.0429E-18
Nmb	9.4053E-23	1.27707292	0.091	0.025	2.0634E-18
Hnrnpul2	9.605E-23	0.41471537	0.512	0.283	2.1072E-18
Pgm2l1	9.6225E-23	0.41561324	0.252	0.107	2.1111E-18
Polr2g	9.7137E-23	0.69501555	0.267	0.122	2.1311E-18
Ptges3	1.0083E-22	0.43077574	0.635	0.383	2.2122E-18
Psmc4	1.071E-22	0.6781085	0.274	0.127	2.3498E-18
Prkar1a	1.1001E-22	0.32328385	0.853	0.595	2.4136E-18
Ubxn11	1.1292E-22	1.10289617	0.102	0.029	2.4774E-18
Sf3a3	1.1544E-22	0.68568607	0.227	0.097	2.5325E-18
Nolc1	1.196E-22	0.53210583	0.311	0.149	2.6239E-18
Trbv31	1.2888E-22	3.42989678	0.014	0.001	2.8275E-18
Pola2	1.3132E-22	1.41925425	0.077	0.019	2.8811E-18
Slc20a1	1.3176E-22	0.4855488	0.352	0.175	2.8906E-18
Ndufaf8	1.3642E-22	0.5058947	0.37	0.189	2.9928E-18
Cmtm7	1.3805E-22	0.64403553	0.395	0.211	3.0287E-18
Lamtor5	1.4739E-22	0.5491622	0.345	0.173	3.2335E-18
Tmem107	1.7212E-22	1.8137639	0.054	0.01	3.7762E-18
Lypla1	1.7427E-22	0.58034344	0.24	0.104	3.8233E-18
Vbp1	1.9128E-22	0.42609827	0.376	0.19	4.1965E-18
Tpd52l2	1.9242E-22	0.58683606	0.306	0.146	4.2215E-18
Rgs9	2.0113E-22	1.3673912	0.088	0.023	4.4126E-18
Arf6	2.0136E-22	0.36710382	0.503	0.278	4.4177E-18
Xlr4b	2.0905E-22	0.79063897	0.166	0.061	4.5863E-18
Carhsp1	2.0949E-22	0.76473132	0.19	0.075	4.5961E-18
Crybg1	2.1914E-22	0.62811376	0.279	0.129	4.8078E-18

Cirbp	2.4444E-22	0.30163823	0.547	0.299	5.3628E-18
Stip1	2.472E-22	0.48300927	0.315	0.151	5.4233E-18
Derl2	2.8252E-22	0.61513598	0.29	0.137	6.1983E-18
Zfp524	3.2369E-22	1.16598194	0.118	0.038	7.1015E-18
Syne2	3.261E-22	1.03348192	0.109	0.033	7.1542E-18
Rtn4rl1	3.5122E-22	1.65481713	0.054	0.011	7.7055E-18
Fyn	3.6418E-22	0.27794571	0.619	0.348	7.9896E-18
Nup50	3.7894E-22	0.53304899	0.329	0.161	8.3136E-18
Nmi	3.9782E-22	0.57867249	0.265	0.121	8.7278E-18
Mgea5	4.0133E-22	0.36623029	0.592	0.342	8.8047E-18
Fh1	4.0397E-22	1.12973614	0.15	0.055	8.8628E-18
Tomm20	4.8943E-22	0.38208356	0.769	0.497	1.0738E-17
Prrc2a	4.8968E-22	0.46631771	0.431	0.232	1.0743E-17
Gclm	5.0676E-22	1.09697173	0.118	0.038	1.1118E-17
Gpr68	5.0933E-22	0.64019503	0.195	0.078	1.1174E-17
Dubr	5.1074E-22	0.91568498	0.132	0.045	1.1205E-17
Imp3	5.1442E-22	0.44236684	0.36	0.181	1.1286E-17
Med9	5.2594E-22	1.34506241	0.113	0.036	1.1539E-17
Cope	5.4366E-22	0.61425464	0.377	0.199	1.1927E-17
Sec61g	5.5119E-22	0.40153753	0.919	0.679	1.2092E-17
Babam1	5.7829E-22	0.88536253	0.181	0.072	1.2687E-17
Hist1h3h	6.3162E-22	3.77427836	0.011	0.001	1.3857E-17
Alad	6.5068E-22	1.36540367	0.088	0.024	1.4275E-17
Dnajb11	6.7597E-22	0.52449686	0.413	0.224	1.483E-17
Haus8	6.9939E-22	0.95814366	0.145	0.052	1.5344E-17
4930523C07R	7.7325E-22	0.31835739	0.699	0.424	1.6964E-17
Dad1	7.8939E-22	0.30096217	0.81	0.539	1.7318E-17
Brd8	7.993E-22	0.49438696	0.401	0.209	1.7536E-17
Hnrnpr	8.866E-22	0.48935822	0.426	0.229	1.9451E-17
Gm10827	9.3376E-22	0.73602928	0.134	0.046	2.0486E-17
Nsd3	9.8194E-22	0.31949837	0.678	0.409	2.1543E-17
Ahcy	9.8817E-22	1.767667	0.048	0.009	2.168E-17
Fam76b	9.94E-22	0.50095968	0.267	0.121	2.1807E-17
Ugp2	1.0644E-21	0.60913385	0.274	0.128	2.3351E-17
Arpc2	1.0816E-21	0.33928228	0.961	0.742	2.373E-17
Rad23a	1.0829E-21	0.7885739	0.218	0.095	2.3758E-17
Icam2	1.2127E-21	0.95840578	0.093	0.026	2.6604E-17
Mapkap1	1.2246E-21	0.79157938	0.211	0.09	2.6867E-17
1110008P14R	1.2343E-21	0.54630093	0.324	0.161	2.7079E-17
Eif2s1	1.2577E-21	0.44809356	0.377	0.195	2.7594E-17
Plcb4	1.2746E-21	0.81657684	0.127	0.042	2.7963E-17
Chchd1	1.2789E-21	0.53275647	0.381	0.201	2.8059E-17
Vps54	1.3844E-21	0.4166847	0.288	0.133	3.0371E-17
Mpv17l2	1.3896E-21	1.18825498	0.122	0.04	3.0487E-17
Neu3	1.4185E-21	1.17853807	0.073	0.018	3.112E-17
Hnrnpa0	1.529E-21	0.37162684	0.837	0.605	3.3544E-17
Mepce	1.5623E-21	0.46785201	0.261	0.117	3.4275E-17
Syt11	1.6568E-21	0.99754788	0.107	0.033	3.6348E-17

Olfr60	1.6628E-21	1.2466555	0.077	0.019	3.648E-17
Dnajb6	1.7367E-21	0.36359874	0.841	0.576	3.8101E-17
Usp50	1.7716E-21	1.03210617	0.116	0.037	3.8868E-17
Lamtor2	1.8719E-21	0.4683274	0.524	0.306	4.1067E-17
Vdac2	1.8911E-21	0.40274036	0.556	0.32	4.1488E-17
Cox14	2.1322E-21	0.37848662	0.619	0.371	4.6778E-17
Saal1	2.1664E-21	1.49463748	0.081	0.021	4.7528E-17
Wasf2	2.3465E-21	0.41718475	0.612	0.372	5.148E-17
Crip2	2.3507E-21	1.81728985	0.07	0.017	5.1572E-17
Vma21	2.3963E-21	0.54568778	0.324	0.163	5.2572E-17
Capns1	2.4927E-21	0.40983452	0.555	0.32	5.4687E-17
Prrc2b	2.6611E-21	0.29858591	0.37	0.183	5.8382E-17
Tmem208	2.9906E-21	0.67361101	0.256	0.119	6.5611E-17
Odf2	3.2298E-21	0.57193602	0.182	0.072	7.086E-17
Gm16286	3.2491E-21	0.41484251	0.467	0.258	7.1282E-17
Eri2	3.3195E-21	1.93177748	0.032	0.004	7.2827E-17
Selenof	3.3402E-21	0.46059965	0.551	0.328	7.328E-17
Cct7	3.5528E-21	0.37426804	0.522	0.297	7.7945E-17
Tinf2	3.7703E-21	0.58615524	0.199	0.081	8.2716E-17
Atp5a1	3.8699E-21	0.37275334	0.778	0.511	8.4902E-17
Zrsr2	3.9554E-21	0.49080484	0.428	0.235	8.6777E-17
Micu2	4.0251E-21	0.67159823	0.193	0.079	8.8307E-17
Cep44	4.1491E-21	0.72496578	0.157	0.058	9.1026E-17
Mat2b	4.2358E-21	0.26505145	0.465	0.246	9.2929E-17
Mad2l1bp	4.3593E-21	0.98099518	0.125	0.042	9.564E-17
Aqr	4.3958E-21	0.42762897	0.308	0.148	9.644E-17
Mrpl41	4.4199E-21	0.6326048	0.229	0.1	9.6968E-17
Ttc39b	4.6938E-21	0.38147897	0.388	0.201	1.0298E-16
Tnik	4.8355E-21	0.46901606	0.218	0.091	1.0609E-16
Itpk1	4.8495E-21	0.7982617	0.184	0.074	1.0639E-16
Abhd2	4.8631E-21	0.4181235	0.279	0.128	1.0669E-16
Xndc1	5.1333E-21	1.065269	0.072	0.018	1.1262E-16
Gnas	5.1843E-21	0.36401411	0.927	0.705	1.1374E-16
Zfp664	5.2046E-21	0.68992578	0.166	0.064	1.1418E-16
Gm33104	5.2212E-21	0.78382236	0.134	0.046	1.1455E-16
Ece1	5.6183E-21	0.76118423	0.37	0.206	1.2326E-16
Unc119b	5.8974E-21	0.71704656	0.211	0.09	1.2938E-16
Tex30	5.9139E-21	0.8262714	0.145	0.052	1.2975E-16
Med28	6.0963E-21	0.45786978	0.419	0.227	1.3375E-16
Tmem209	6.4162E-21	0.87475825	0.118	0.038	1.4076E-16
Dohh	6.5311E-21	0.84020795	0.177	0.071	1.4329E-16
Rwdd4a	6.5632E-21	0.93092729	0.166	0.065	1.4399E-16
Tecr	6.7823E-21	0.44710616	0.338	0.17	1.488E-16
Arntl	6.9581E-21	0.51148323	0.2	0.081	1.5265E-16
Cisd3	7.1839E-21	1.16434261	0.132	0.047	1.5761E-16
Tmem173	7.3925E-21	0.73839133	0.193	0.08	1.6218E-16
Malsu1	7.7995E-21	0.64986189	0.252	0.116	1.7111E-16
Ccni	8.1033E-21	0.40632706	0.449	0.247	1.7778E-16

Tnfsf10	8.1708E-21	1.08751234	0.075	0.019	1.7926E-16
Gramd1b	8.2619E-21	0.46816813	0.2	0.082	1.8126E-16
Rhebl1	8.3133E-21	1.5532405	0.052	0.01	1.8239E-16
Stag2	8.3535E-21	0.3571154	0.451	0.246	1.8327E-16
Cs	8.5721E-21	0.55774251	0.32	0.162	1.8806E-16
Kcnab2	8.6804E-21	0.90192226	0.145	0.053	1.9044E-16
Ndufb4	9.0013E-21	0.4898035	0.415	0.229	1.9748E-16
Sp3	9.7208E-21	0.37066901	0.394	0.204	2.1326E-16
Trmt6	9.7358E-21	0.72874209	0.184	0.075	2.1359E-16
Oard1	9.9434E-21	0.67074564	0.172	0.067	2.1815E-16
Cenpl	9.9554E-21	0.76780004	0.206	0.088	2.1841E-16
Fanci	1.0163E-20	3.04688236	0.018	0.002	2.2297E-16
Bag4	1.0373E-20	0.81114937	0.136	0.048	2.2757E-16
Ddx42	1.1543E-20	0.40068806	0.451	0.247	2.5325E-16
Tssc4	1.1818E-20	0.9997673	0.114	0.037	2.5929E-16
Tmem71	1.191E-20	0.60893836	0.175	0.068	2.6129E-16
Dis3	1.258E-20	1.16682855	0.093	0.027	2.7599E-16
Pbdc1	1.2611E-20	0.59931443	0.311	0.157	2.7668E-16
Arhgap10	1.2821E-20	1.01604344	0.118	0.039	2.8128E-16
Actr10	1.2891E-20	0.60113819	0.27	0.129	2.8281E-16
Tmem263	1.3359E-20	0.77138004	0.134	0.047	2.9308E-16
Ubxn1	1.537E-20	0.39530491	0.606	0.369	3.3719E-16
Numa1	1.5437E-20	0.30495278	0.386	0.196	3.3867E-16
2010111I01Ril	1.5865E-20	0.67747808	0.227	0.101	3.4806E-16
Setd3	1.6835E-20	0.49443476	0.29	0.139	3.6935E-16
Rbmx	1.8144E-20	0.71065599	0.168	0.065	3.9807E-16
Ilk	1.9302E-20	0.75709692	0.215	0.095	4.2347E-16
Adss	1.9508E-20	0.42309011	0.447	0.247	4.28E-16
Tlk2	1.9801E-20	0.45279627	0.27	0.126	4.344E-16
Rbm15	1.9874E-20	0.67264434	0.249	0.115	4.3602E-16
Cct3	2.0477E-20	0.4814558	0.401	0.218	4.4924E-16
Nup43	2.0568E-20	1.72556382	0.054	0.011	4.5125E-16
Bbip1	2.3001E-20	0.58393091	0.256	0.121	5.0462E-16
Hsd17b7	2.3211E-20	1.01767588	0.1	0.031	5.0923E-16
Gpsm3	2.4442E-20	0.49384549	0.41	0.226	5.3624E-16
Znrd1	2.5208E-20	0.39962567	0.367	0.19	5.5305E-16
Fbxw2	2.6896E-20	0.36698864	0.415	0.223	5.9007E-16
Timm22	2.7231E-20	0.73182367	0.163	0.063	5.9742E-16
Grk6	2.8035E-20	0.38394697	0.284	0.133	6.1507E-16
Eipr1	2.8267E-20	0.63364812	0.177	0.071	6.2016E-16
Kif24	3.1147E-20	3.30917983	0.014	0.001	6.8333E-16
Smap2	3.2488E-20	0.46379675	0.571	0.35	7.1276E-16
BC003965	3.3384E-20	0.79167825	0.148	0.055	7.3242E-16
Ilkap	3.6288E-20	0.59545951	0.245	0.113	7.9611E-16
Taf6l	3.7752E-20	0.43735376	0.361	0.189	8.2825E-16
Ndufs3	4.1402E-20	0.60733952	0.306	0.155	9.0832E-16
BC004004	4.4207E-20	0.64194855	0.227	0.102	9.6987E-16
Stat4	4.4414E-20	0.26806149	0.648	0.382	9.744E-16

Rps6ka5	4.5499E-20	0.78130013	0.12	0.04	9.9821E-16
Epsti1	4.9425E-20	0.39056198	0.524	0.31	1.0843E-15
Gsk3a	5.0459E-20	0.35117223	0.301	0.146	1.107E-15
Capzb	5.1165E-20	0.38094753	0.821	0.543	1.1225E-15
Il17re	5.4291E-20	1.55104678	0.064	0.015	1.1911E-15
Gcc2	5.5987E-20	0.44619324	0.263	0.123	1.2283E-15
Mphosph10	5.6614E-20	0.50375517	0.242	0.11	1.242E-15
Fam58b	5.7618E-20	1.04877765	0.1	0.031	1.2641E-15
Gsk3b	5.878E-20	0.4298853	0.444	0.248	1.2896E-15
Rbl2	5.9063E-20	0.42268967	0.299	0.143	1.2958E-15
Mpp6	5.937E-20	0.73054743	0.254	0.121	1.3025E-15
Cops6	6.0443E-20	0.67543565	0.274	0.135	1.3261E-15
Glo1	6.1033E-20	0.86351568	0.181	0.075	1.339E-15
Wdr12	6.2012E-20	1.15095019	0.109	0.035	1.3605E-15
Bzw1	6.8994E-20	0.42549402	0.673	0.433	1.5137E-15
Kifc5b	7.0979E-20	3.1481594	0.014	0.001	1.5572E-15
Smpd4	7.4903E-20	1.07694413	0.098	0.03	1.6433E-15
Arhgef6	8.0594E-20	0.51370622	0.295	0.147	1.7681E-15
Papola	8.2248E-20	0.39771581	0.499	0.29	1.8044E-15
Csnk2a2	8.229E-20	0.40651142	0.438	0.244	1.8054E-15
Asrgl1	8.336E-20	1.03715608	0.107	0.035	1.8288E-15
Rab27a	8.3574E-20	0.82901792	0.132	0.047	1.8335E-15
Capza1	9.0806E-20	0.34734362	0.58	0.345	1.9922E-15
Idh3g	9.0966E-20	0.56414939	0.293	0.147	1.9957E-15
Emc6	9.1162E-20	0.44324179	0.411	0.227	2E-15
Yy1	9.2311E-20	0.39471786	0.546	0.321	2.0252E-15
Pcbp2	9.4729E-20	0.36203761	0.911	0.687	2.0783E-15
Pebp1	9.545E-20	0.48666813	0.445	0.255	2.0941E-15
Ndufaf2	9.584E-20	1.07599868	0.129	0.046	2.1026E-15
Cyp51	9.619E-20	0.38934055	0.306	0.152	2.1103E-15
Gxylt1	1.0031E-19	0.50143093	0.277	0.134	2.2006E-15
Srsf4	1.0061E-19	0.72215411	0.213	0.094	2.2072E-15
Ndufb2	1.0069E-19	0.51438045	0.388	0.214	2.2091E-15
Ssr4	1.0229E-19	0.32852347	0.758	0.457	2.2441E-15
Lrwd1	1.0586E-19	1.07175168	0.098	0.03	2.3224E-15
Mtch1	1.1132E-19	0.73890484	0.229	0.107	2.4423E-15
Tex261	1.1318E-19	0.63322652	0.258	0.122	2.483E-15
Hdac2	1.1794E-19	0.82814368	0.161	0.064	2.5875E-15
Magohb	1.236E-19	0.84619701	0.184	0.078	2.7117E-15
Tmem248	1.2594E-19	0.46501351	0.32	0.163	2.7629E-15
Frmd8	1.2811E-19	1.02065972	0.1	0.031	2.8106E-15
Piga	1.2977E-19	1.07589603	0.123	0.043	2.847E-15
Dtnb	1.3311E-19	0.70178488	0.113	0.037	2.9203E-15
Ddx27	1.3987E-19	0.64681408	0.233	0.107	3.0685E-15
Cish	1.4338E-19	0.36737749	0.399	0.216	3.1457E-15
Sra1	1.617E-19	0.40101346	0.438	0.246	3.5476E-15
Arl1	1.6471E-19	0.51396694	0.315	0.161	3.6135E-15
Gps1	1.7202E-19	0.80201696	0.166	0.067	3.7739E-15

Thoc3	1.7419E-19	0.87665685	0.147	0.056	3.8215E-15
Morc1	1.7987E-19	2.48630119	0.018	0.002	3.9461E-15
Hmces	1.8084E-19	0.61679769	0.202	0.087	3.9673E-15
Usp37	1.8371E-19	0.77118626	0.213	0.096	4.0304E-15
Ube2o	1.8386E-19	0.95707996	0.122	0.042	4.0336E-15
Uspl1	1.8466E-19	0.55738485	0.206	0.089	4.0512E-15
Ewsr1	1.8534E-19	0.32595347	0.655	0.401	4.0661E-15
Tmem237	1.8899E-19	1.61678482	0.054	0.012	4.1463E-15
Eif2b1	1.9109E-19	1.04434615	0.095	0.029	4.1923E-15
Gm2245	1.9896E-19	1.51498073	0.063	0.015	4.365E-15
Ngdn	2.0225E-19	0.53893471	0.302	0.153	4.4371E-15
Zc3h14	2.0413E-19	0.47356041	0.252	0.117	4.4783E-15
Rbm27	2.2555E-19	0.31323378	0.419	0.226	4.9484E-15
Cmc1	2.352E-19	0.527342	0.317	0.163	5.1601E-15
Osgep	2.55E-19	0.83309683	0.236	0.113	5.5944E-15
4921524J17Ril	2.6773E-19	0.39019128	0.234	0.105	5.8738E-15
Cops3	2.701E-19	0.64141007	0.163	0.064	5.9258E-15
Aco2	2.7231E-19	0.52675272	0.343	0.182	5.9741E-15
Fkbp4	2.7833E-19	0.28141878	0.254	0.119	6.1063E-15
Hypk	2.866E-19	0.46605032	0.318	0.163	6.2878E-15
Sap30bp	3.2931E-19	0.72953787	0.182	0.077	7.2247E-15
Idi1	3.4339E-19	0.38095158	0.247	0.115	7.5337E-15
Cct5	3.5484E-19	0.47043503	0.499	0.297	7.7848E-15
Tbl1x	3.7754E-19	0.36189379	0.354	0.183	8.2829E-15
Cct4	3.777E-19	0.41020464	0.47	0.269	8.2864E-15
Tarbp2	4.0167E-19	0.93276783	0.122	0.043	8.8123E-15
Spg7	4.0663E-19	0.74412369	0.184	0.078	8.9211E-15
Hmgxb4	4.3806E-19	0.80923093	0.127	0.045	9.6105E-15
1190002N15R	4.514E-19	0.66719723	0.156	0.06	9.9032E-15
Ms4a6b	4.7673E-19	0.32933713	0.449	0.254	1.0459E-14
Coa7	4.9822E-19	1.05711803	0.116	0.04	1.093E-14
Mkrn1	5.0779E-19	0.28320869	0.667	0.411	1.114E-14
Zfp773	5.1738E-19	1.24674721	0.052	0.011	1.1351E-14
Alg5	6.368E-19	0.71242968	0.191	0.083	1.3971E-14
Agfg1	6.4403E-19	0.27391288	0.36	0.186	1.4129E-14
Ppp1r18	6.4493E-19	0.3686112	0.365	0.194	1.4149E-14
Ssx2ip	6.5471E-19	1.3828346	0.072	0.019	1.4364E-14
Rab19	6.6712E-19	1.01666367	0.104	0.034	1.4636E-14
Slc35g1	6.8144E-19	1.34533288	0.05	0.011	1.495E-14
Gas2	6.9049E-19	1.28051931	0.047	0.009	1.5149E-14
Il12rb2	6.9442E-19	0.46433995	0.175	0.071	1.5235E-14
Ly6e	7.1511E-19	0.26330114	0.964	0.776	1.5689E-14
Katna1	7.4313E-19	0.36766919	0.286	0.14	1.6303E-14
Nr1d1	7.7163E-19	1.21823129	0.088	0.026	1.6929E-14
Exosc4	7.738E-19	0.91891463	0.143	0.055	1.6976E-14
Uba1	8.0861E-19	0.4277643	0.372	0.201	1.774E-14
Rtf1	8.3297E-19	0.28456527	0.558	0.329	1.8275E-14
Fam60a	8.4804E-19	0.68427956	0.168	0.068	1.8605E-14

Srbd1	8.8955E-19	1.20639045	0.084	0.025	1.9516E-14
Fmr1	9.1728E-19	0.33099908	0.336	0.173	2.0124E-14
Mdp1	9.3032E-19	0.40168522	0.243	0.113	2.041E-14
Aak1	9.8255E-19	0.32781617	0.272	0.128	2.1556E-14
R3hdm4	1.0193E-18	0.46458802	0.286	0.142	2.2362E-14
Nfatc2	1.0246E-18	0.4453206	0.181	0.075	2.2479E-14
Sike1	1.0303E-18	0.55152906	0.209	0.093	2.2604E-14
Virma	1.0561E-18	0.48783926	0.25	0.118	2.3169E-14
Cpsf4	1.0684E-18	0.90117244	0.106	0.035	2.3441E-14
Fam120a	1.0995E-18	0.40831254	0.333	0.174	2.4121E-14
Il12rb1	1.1401E-18	1.05410909	0.073	0.02	2.5013E-14
Ociad1	1.2319E-18	0.33825028	0.429	0.237	2.7026E-14
Kin	1.2378E-18	0.39093433	0.216	0.096	2.7156E-14
Eif3b	1.244E-18	0.39242269	0.372	0.2	2.7292E-14
Zw10	1.264E-18	0.89078435	0.091	0.028	2.7731E-14
Sars	1.2771E-18	0.39312756	0.429	0.243	2.8018E-14
Fli1	1.2839E-18	0.29449797	0.46	0.259	2.8167E-14
Rpia	1.3186E-18	0.68175468	0.154	0.06	2.8928E-14
Nop10	1.3306E-18	0.31629261	0.544	0.323	2.9193E-14
Crebzf	1.3435E-18	0.53203132	0.242	0.112	2.9476E-14
Xpo6	1.3766E-18	0.4457171	0.256	0.122	3.0202E-14
Asns	1.4075E-18	0.8571579	0.138	0.052	3.0878E-14
Phf6	1.4564E-18	0.4096655	0.218	0.097	3.1953E-14
Scand1	1.464E-18	0.35294339	0.735	0.475	3.2119E-14
Mbd3	1.4658E-18	0.80997827	0.186	0.081	3.2157E-14
Mettl23	1.4881E-18	0.32413376	0.413	0.226	3.2648E-14
Nipal3	1.5081E-18	0.894741	0.122	0.043	3.3085E-14
Agk	1.5543E-18	0.83538773	0.106	0.035	3.4099E-14
Pcdhgc3	1.5666E-18	2.59336309	0.016	0.001	3.437E-14
Wbp1	1.6123E-18	0.93172017	0.134	0.05	3.5372E-14
Cramp1l	1.6762E-18	0.70088524	0.141	0.054	3.6775E-14
Ptp4a2	1.7199E-18	0.28234388	0.923	0.722	3.7732E-14
Parp1	1.7213E-18	0.3787603	0.254	0.121	3.7764E-14
Rtraf	1.75E-18	0.36088989	0.614	0.378	3.8393E-14
Abcb1b	1.799E-18	0.38287583	0.304	0.153	3.9469E-14
Ssu72	1.8021E-18	0.42689128	0.345	0.187	3.9536E-14
Hspa4l	1.8299E-18	0.53580271	0.168	0.069	4.0145E-14
Tmed2	2.0147E-18	0.31604824	0.689	0.437	4.42E-14
Nrf1	2.0223E-18	0.52097501	0.188	0.08	4.4367E-14
Aimp2	2.0551E-18	1.01404257	0.091	0.028	4.5086E-14
Pdcd10	2.1089E-18	0.30183194	0.535	0.316	4.6268E-14
Sumo1	2.1347E-18	0.31578925	0.621	0.38	4.6834E-14
Plcg1	2.1675E-18	0.54658294	0.175	0.072	4.7554E-14
Ppp1r11	2.2349E-18	0.2937527	0.472	0.27	4.9031E-14
Ube2l3	2.2589E-18	0.42216762	0.583	0.367	4.9557E-14
Chd2	2.2806E-18	0.27951016	0.751	0.491	5.0034E-14
Bloc1s4	2.3055E-18	0.55317606	0.197	0.086	5.058E-14
Actr1a	2.3079E-18	0.63329772	0.208	0.094	5.0633E-14

Ccnh	2.3818E-18	0.85006752	0.125	0.045	5.2255E-14
Hint3	2.4647E-18	0.57826445	0.234	0.111	5.4073E-14
Cggbp1	2.5557E-18	0.35336791	0.61	0.375	5.607E-14
Abt1	2.7026E-18	0.764999	0.154	0.061	5.9293E-14
Pak1ip1	2.7215E-18	0.45902952	0.345	0.186	5.9707E-14
C330007P06R	2.727E-18	0.60224534	0.184	0.079	5.9827E-14
Ier5l	2.7771E-18	0.32909746	0.286	0.14	6.0928E-14
Bin2	2.8945E-18	0.46528574	0.27	0.132	6.3502E-14
Rprd1b	2.9117E-18	0.55418942	0.202	0.089	6.3881E-14
Cep76	2.9571E-18	1.28833921	0.057	0.013	6.4876E-14
Rpp25l	3.0778E-18	0.59991407	0.216	0.099	6.7524E-14
Gatad2a	3.1465E-18	0.33705055	0.352	0.186	6.9031E-14
Erlec1	3.1942E-18	0.50111428	0.227	0.105	7.0078E-14
Snapc5	3.2225E-18	0.73523385	0.243	0.119	7.0698E-14
Prkag1	3.3133E-18	0.68230475	0.141	0.054	7.2691E-14
Mum1	3.3863E-18	0.73630093	0.118	0.041	7.4293E-14
Tcea1	3.4143E-18	0.29757958	0.54	0.324	7.4907E-14
Ankmy1	3.4375E-18	1.99562386	0.03	0.005	7.5415E-14
2810001G20R	3.4564E-18	0.77706891	0.107	0.036	7.5831E-14
Zfand6	3.4756E-18	0.34145899	0.472	0.271	7.6252E-14
Vrk1	3.4968E-18	0.64765434	0.231	0.11	7.6715E-14
Ppp4r3a	3.5597E-18	0.26749236	0.406	0.22	7.8096E-14
Iws1	3.6087E-18	0.44991259	0.32	0.165	7.917E-14
Tmx1	3.7433E-18	0.50955039	0.227	0.106	8.2124E-14
Skiv2l2	3.7438E-18	0.49143783	0.229	0.106	8.2136E-14
Gm13546	3.9123E-18	1.9932839	0.05	0.011	8.5832E-14
Acat1	3.9576E-18	0.66384383	0.209	0.096	8.6826E-14
Zdhhc12	4.0958E-18	1.67016414	0.054	0.012	8.9857E-14
Naa40	4.1263E-18	1.11382756	0.098	0.032	9.0527E-14
Eif4ebp2	4.1349E-18	0.31676972	0.338	0.175	9.0716E-14
Mrpl14	4.193E-18	0.60234963	0.268	0.136	9.199E-14
Prr29	4.402E-18	1.21891261	0.063	0.016	9.6575E-14
Zranb2	4.4987E-18	0.33815767	0.308	0.157	9.8697E-14
Supt20	4.7115E-18	0.34726377	0.372	0.199	1.0337E-13
Flna	5.1614E-18	0.47515462	0.46	0.274	1.1324E-13
Itpa	5.2881E-18	0.90797485	0.129	0.048	1.1602E-13
Syf2	5.4027E-18	0.31369452	0.657	0.409	1.1853E-13
Gnai3	5.6777E-18	0.30665392	0.467	0.267	1.2456E-13
Fam160b1	5.994E-18	0.54811572	0.182	0.077	1.315E-13
Sdhc	6.1338E-18	0.58777173	0.229	0.109	1.3457E-13
Rngtt	6.3005E-18	0.52365608	0.19	0.082	1.3823E-13
Map1a	6.3123E-18	3.41982491	0.011	0.001	1.3849E-13
Ints2	6.6538E-18	1.15008157	0.07	0.019	1.4598E-13
Xlr4a	7.08E-18	0.94667501	0.093	0.029	1.5533E-13
Anapc13	7.0982E-18	0.52526803	0.335	0.181	1.5573E-13
Amz2	7.0985E-18	0.56527759	0.204	0.091	1.5573E-13
Ppp6r3	7.1528E-18	0.26670521	0.404	0.221	1.5692E-13
Nup155	7.4629E-18	0.83768335	0.093	0.029	1.6373E-13

Smarca5	7.6105E-18	0.32781953	0.758	0.516	1.6697E-13
Sin3b	7.666E-18	0.36105595	0.542	0.33	1.6819E-13
Hspa8	7.9477E-18	0.26644454	0.979	0.823	1.7437E-13
Krit1	8.2817E-18	0.29960179	0.499	0.291	1.8169E-13
Gpaa1	8.3099E-18	1.12688906	0.081	0.024	1.8231E-13
Rpap3	9.1975E-18	0.80081785	0.118	0.042	2.0178E-13
Tpgs2	9.7615E-18	1.08973915	0.095	0.031	2.1416E-13
Cherp	9.844E-18	0.63266298	0.166	0.069	2.1597E-13
Pigf	9.9323E-18	1.23498151	0.086	0.026	2.179E-13
Mcts1	1.0053E-17	0.56906191	0.261	0.132	2.2056E-13
Plekha3	1.0122E-17	0.66076547	0.19	0.083	2.2206E-13
Nxpe3	1.0154E-17	0.70801527	0.088	0.027	2.2277E-13
Fbxo33	1.0355E-17	0.37776381	0.247	0.117	2.2719E-13
Gch1	1.0425E-17	0.29095132	0.469	0.269	2.2872E-13
Immp1l	1.0437E-17	0.60923388	0.177	0.076	2.2899E-13
Smarcc2	1.0576E-17	0.67252895	0.179	0.077	2.3203E-13
Shmt2	1.0692E-17	1.01475107	0.114	0.041	2.3458E-13
Ctr9	1.0811E-17	0.5085757	0.2	0.089	2.3718E-13
Hdac3	1.1105E-17	0.94481272	0.116	0.042	2.4363E-13
B230219D22R	1.1365E-17	0.44629491	0.447	0.263	2.4933E-13
Mapk1	1.1854E-17	0.39790675	0.406	0.231	2.6007E-13
Znhit3	1.1894E-17	1.10603818	0.098	0.032	2.6094E-13
Fancm	1.2E-17	1.06311977	0.073	0.02	2.6326E-13
Nop16	1.2709E-17	0.98517223	0.116	0.042	2.7881E-13
Rbmxl1	1.2768E-17	0.58433915	0.211	0.097	2.8011E-13
Ddrgk1	1.3766E-17	0.37966469	0.401	0.225	3.0202E-13
Sucla2	1.3791E-17	0.71382697	0.179	0.078	3.0255E-13
Zdhhc3	1.3814E-17	0.61968545	0.218	0.102	3.0306E-13
St6galnac3	1.3921E-17	0.66605894	0.152	0.06	3.0542E-13
Alg8	1.569E-17	1.99992919	0.048	0.011	3.4422E-13
Crlf2	1.5797E-17	0.51719088	0.399	0.232	3.4656E-13
Nfrkb	1.5813E-17	0.41250435	0.186	0.08	3.4693E-13
Akt1	1.5815E-17	0.66839044	0.225	0.108	3.4696E-13
Mettl26	1.5916E-17	0.86604729	0.148	0.06	3.4919E-13
Coa3	1.6246E-17	0.65611166	0.247	0.123	3.5643E-13
Lrrcc1	1.7025E-17	0.83599024	0.093	0.03	3.7352E-13
Prkcq	1.7039E-17	0.49971967	0.132	0.049	3.7383E-13
Hars	1.7626E-17	0.70818349	0.168	0.071	3.8671E-13
Washc3	1.7818E-17	0.72472982	0.19	0.085	3.9091E-13
Myef2	1.8014E-17	0.6029216	0.191	0.085	3.9521E-13
Rab8a	1.8038E-17	0.31450631	0.436	0.251	3.9573E-13
Hnrnpf	1.8856E-17	0.35319405	0.818	0.592	4.1368E-13
Glipr1	2.0121E-17	0.61834168	0.252	0.126	4.4145E-13
Taf11	2.0415E-17	0.75086374	0.161	0.067	4.4789E-13
Kat7	2.1302E-17	0.53363888	0.2	0.09	4.6735E-13
Trmt112	2.1498E-17	0.38710892	0.478	0.282	4.7165E-13
Bex2	2.1552E-17	2.32231021	0.018	0.002	4.7282E-13
Mapk14	2.3132E-17	0.66733708	0.227	0.109	5.0748E-13

Tyro3	2.3198E-17	2.92557413	0.016	0.001	5.0893E-13
Wrnip1	2.3345E-17	0.76316801	0.125	0.046	5.1217E-13
Ndufs2	2.3356E-17	0.56584842	0.281	0.146	5.1242E-13
Gstcd	2.3418E-17	0.97019597	0.07	0.019	5.1377E-13
Sar1b	2.3486E-17	0.61196603	0.259	0.131	5.1526E-13
Eef1d	2.3651E-17	0.32318611	0.814	0.576	5.1888E-13
Fam98b	2.4247E-17	0.36575328	0.304	0.156	5.3195E-13
Stx11	2.4798E-17	0.32390996	0.336	0.18	5.4404E-13
Exoc3	2.5395E-17	0.3436032	0.301	0.155	5.5714E-13
Cdk6	2.7208E-17	0.44228403	0.256	0.125	5.9692E-13
Uimc1	2.9592E-17	0.47492963	0.279	0.142	6.4922E-13
Ehmt2	3.0316E-17	0.70121419	0.209	0.098	6.6511E-13
Txnl4a	3.0793E-17	0.730213	0.206	0.096	6.7556E-13
Nr2c2	3.0948E-17	0.35587756	0.315	0.163	6.7898E-13
Brd4	3.2177E-17	0.38034089	0.567	0.354	7.0593E-13
1700123O20R	3.236E-17	0.54757488	0.2	0.091	7.0994E-13
Ruvbl1	3.3549E-17	0.80989829	0.168	0.073	7.3603E-13
Zbtb8os	3.3776E-17	0.81126616	0.193	0.089	7.4101E-13
Alkbh4	3.5475E-17	1.15547869	0.064	0.017	7.7828E-13
Mettl14	3.5531E-17	0.7371404	0.129	0.049	7.7952E-13
Nup133	3.7431E-17	0.98464195	0.084	0.026	8.2119E-13
Ppp2r5e	3.8439E-17	0.50924838	0.206	0.094	8.4332E-13
Taf3	4.2897E-17	0.33083205	0.231	0.108	9.4112E-13
Msl3	4.3462E-17	0.84252593	0.143	0.058	9.5351E-13
Drg1	4.4804E-17	0.63442883	0.186	0.083	9.8296E-13
Timm10b	4.5304E-17	0.43039888	0.354	0.197	9.9392E-13
Clns1a	4.5837E-17	0.56321655	0.2	0.091	1.0056E-12
Sf3b4	4.594E-17	0.48682963	0.225	0.107	1.0079E-12
B230354K17R	4.6486E-17	1.43102341	0.055	0.013	1.0199E-12
Mrps5	4.8159E-17	0.52741191	0.19	0.084	1.0566E-12
Gm10093	5.1146E-17	1.24387393	0.081	0.024	1.1221E-12
Rp9	5.1515E-17	0.26004356	0.465	0.267	1.1302E-12
Nfkb2	5.163E-17	0.33443431	0.501	0.301	1.1327E-12
Glod4	5.1991E-17	0.9556351	0.177	0.08	1.1406E-12
Pet100	5.3364E-17	0.34908212	0.451	0.266	1.1708E-12
Ldlrad4	5.7817E-17	0.78839042	0.129	0.049	1.2685E-12
Wbp4	5.9387E-17	0.39612116	0.342	0.185	1.3029E-12
Ankrd54	5.9569E-17	0.83994178	0.1	0.034	1.3069E-12
Nup88	6.041E-17	0.65132972	0.191	0.086	1.3253E-12
Pard6a	6.0518E-17	1.15932672	0.086	0.027	1.3277E-12
Brd7	6.074E-17	0.40907242	0.288	0.148	1.3326E-12
Rps6kb1	6.4043E-17	0.25833689	0.322	0.167	1.405E-12
Rundc3b	6.5704E-17	0.58113585	0.152	0.061	1.4415E-12
Maf1	6.6227E-17	0.36044525	0.281	0.141	1.4529E-12
Zfp850	6.8965E-17	1.79219012	0.036	0.006	1.513E-12
Psmd7	6.9877E-17	0.41427516	0.34	0.185	1.533E-12
Fam104a	7.7751E-17	0.32327227	0.472	0.28	1.7058E-12
Pdcd6ip	7.8755E-17	0.30838993	0.533	0.323	1.7278E-12

Snrnp200	7.9072E-17	0.30514171	0.32	0.168	1.7348E-12
Il17f	8.0375E-17	0.84009141	0.093	0.031	1.7634E-12
Srpr	8.4194E-17	0.35645548	0.469	0.278	1.8471E-12
Afg3l2	8.5265E-17	0.83497446	0.131	0.051	1.8706E-12
2310061I04Ril	9.0472E-17	1.04128678	0.084	0.026	1.9849E-12
Pfkl	9.1016E-17	0.71464432	0.179	0.079	1.9968E-12
Rbm10	9.1079E-17	0.83928129	0.131	0.051	1.9982E-12
Acap1	9.4389E-17	0.26564513	0.222	0.101	2.0708E-12
Prpf18	9.8918E-17	0.43925598	0.268	0.135	2.1702E-12
Smim8	1.0176E-16	0.63820247	0.136	0.053	2.2325E-12
Hist1h4b	1.0273E-16	2.21809285	0.02	0.002	2.2538E-12
Bax	1.0456E-16	0.51700078	0.444	0.268	2.2939E-12
Eif5a	1.0632E-16	0.25624433	0.782	0.508	2.3326E-12
Gm17745	1.1069E-16	1.13985417	0.089	0.029	2.4284E-12
Cops2	1.122E-16	0.44772686	0.288	0.151	2.4615E-12
Cnot11	1.1345E-16	0.70572994	0.145	0.059	2.489E-12
Alg13	1.17E-16	1.14346184	0.088	0.028	2.5668E-12
Bcap31	1.1871E-16	0.4063673	0.406	0.236	2.6043E-12
Dnlz	1.2478E-16	0.81504308	0.141	0.057	2.7376E-12
Nup62	1.3017E-16	0.71043749	0.159	0.067	2.8558E-12
St6galnac1	1.3158E-16	2.02461059	0.03	0.005	2.8866E-12
Hnrnpa1	1.3735E-16	0.29355089	0.825	0.583	3.0134E-12
Dda1	1.4231E-16	0.39186474	0.301	0.157	3.1221E-12
Nop9	1.4319E-16	0.67202719	0.161	0.068	3.1414E-12
Serpinb6a	1.4844E-16	0.41057577	0.202	0.092	3.2567E-12
Aqp3	1.5252E-16	1.06479062	0.052	0.012	3.3462E-12
2610020C07R	1.5289E-16	1.77159613	0.032	0.005	3.3543E-12
Sigirr	1.5435E-16	1.03606998	0.089	0.029	3.3863E-12
Coro1c	1.5983E-16	0.54652701	0.247	0.125	3.5065E-12
Esf1	1.6193E-16	0.34364971	0.279	0.142	3.5525E-12
0610012G03R	1.6605E-16	0.64447463	0.261	0.135	3.6429E-12
Rsrc1	1.6769E-16	0.6161369	0.24	0.119	3.679E-12
Hspbp1	1.7072E-16	0.81078203	0.134	0.053	3.7453E-12
Sdhd	1.7091E-16	0.58704172	0.275	0.144	3.7496E-12
Rlf	1.719E-16	0.30750421	0.411	0.234	3.7712E-12
Usp14	1.7263E-16	0.5153312	0.258	0.131	3.7873E-12
Zmpste24	1.7851E-16	0.54184987	0.191	0.087	3.9163E-12
Tmub1	1.7923E-16	0.88818798	0.1	0.034	3.9321E-12
March2	1.8126E-16	0.64459718	0.2	0.093	3.9767E-12
Ccm2	1.9124E-16	0.39280482	0.256	0.126	4.1956E-12
Prpf40a	2.0762E-16	0.32954021	0.54	0.334	4.555E-12
Tmem160	2.1458E-16	0.44650723	0.492	0.305	4.7077E-12
Tcrg-C2	2.1999E-16	1.59200535	0.048	0.011	4.8263E-12
Msh3	2.3643E-16	0.77540325	0.106	0.037	5.187E-12
Cd82	2.4149E-16	0.26671154	0.58	0.363	5.298E-12
Utp3	2.4333E-16	0.38097803	0.403	0.233	5.3384E-12
Bscl2	2.4576E-16	0.7098328	0.208	0.1	5.3917E-12
Primpol	2.5464E-16	1.32806189	0.066	0.018	5.5865E-12

Pcdhgc4	2.5493E-16	2.48438955	0.018	0.002	5.5928E-12
Gnb1	2.5924E-16	0.29905778	0.748	0.501	5.6874E-12
Rexo5	2.6653E-16	1.18098869	0.05	0.012	5.8473E-12
Tmed9	2.705E-16	0.34717296	0.555	0.345	5.9346E-12
Smarcd2	2.7618E-16	0.35083405	0.249	0.122	6.0592E-12
Cnot10	2.781E-16	0.68395076	0.147	0.061	6.1012E-12
Galnt12	2.812E-16	0.80557622	0.086	0.027	6.1692E-12
Anapc7	2.8191E-16	0.71591086	0.14	0.056	6.1849E-12
Rnf20	2.8313E-16	0.35568276	0.306	0.161	6.2117E-12
Stk38	2.8875E-16	0.45345184	0.272	0.139	6.3349E-12
Fam110a	2.9371E-16	0.37230659	0.275	0.141	6.4437E-12
Zcchc17	2.9727E-16	0.50571958	0.213	0.101	6.5218E-12
Mak16	3.0488E-16	0.36185435	0.292	0.152	6.6887E-12
Ddx20	3.1821E-16	0.8000483	0.088	0.028	6.9813E-12
Ipo5	3.2096E-16	0.73653091	0.191	0.089	7.0416E-12
Naa50	3.2521E-16	0.41494353	0.36	0.203	7.1348E-12
Rnf187	3.3369E-16	0.34330577	0.27	0.137	7.3209E-12
Nemp2	3.4124E-16	0.70717933	0.113	0.041	7.4865E-12
Cox19	3.6328E-16	0.84713014	0.122	0.047	7.9701E-12
Nin	3.6462E-16	0.45836528	0.259	0.131	7.9993E-12
Stub1	3.7729E-16	0.5664637	0.216	0.105	8.2775E-12
Wdr82	3.8157E-16	0.42355111	0.213	0.1	8.3712E-12
Nars	4.1921E-16	0.34428617	0.553	0.351	9.1971E-12
Vcp	4.2687E-16	0.28582518	0.596	0.376	9.3651E-12
Cdc23	4.2891E-16	0.86086231	0.086	0.027	9.4099E-12
Gm6594	4.5325E-16	2.91196567	0.013	0.001	9.9438E-12
Cmip	4.645E-16	0.34124448	0.47	0.279	1.0191E-11
Ift20	4.7396E-16	0.55159902	0.295	0.16	1.0398E-11
Mvb12a	4.7602E-16	0.59649956	0.249	0.128	1.0443E-11
Uqcc3	4.8408E-16	0.31060956	0.292	0.151	1.062E-11
Mb21d1	4.8628E-16	1.20431614	0.068	0.019	1.0669E-11
Dnajb13	4.878E-16	1.75171318	0.034	0.006	1.0702E-11
Fam204a	4.9107E-16	0.27234923	0.395	0.222	1.0774E-11
Chd4	5.0069E-16	0.26073077	0.687	0.45	1.0985E-11
Hnrnpdl	5.1096E-16	0.30192393	0.68	0.449	1.121E-11
Cotl1	5.4314E-16	0.27560518	0.737	0.485	1.1916E-11
Rpf1	5.5927E-16	0.46619108	0.211	0.1	1.227E-11
Gm14718	5.6023E-16	0.9973345	0.055	0.014	1.2291E-11
Ncoa2	5.7301E-16	0.29621797	0.349	0.187	1.2571E-11
Dkc1	6.0561E-16	0.72948057	0.129	0.05	1.3287E-11
Srrm1	6.2611E-16	0.34556651	0.698	0.466	1.3736E-11
Eif6	6.6627E-16	0.46641343	0.374	0.216	1.4617E-11
Il27ra	7.1415E-16	0.46959055	0.14	0.055	1.5668E-11
C2cd5	7.1682E-16	0.56391604	0.123	0.047	1.5726E-11
Tubgcp6	7.1861E-16	1.01220155	0.063	0.017	1.5765E-11
Nfya	7.2102E-16	0.41729246	0.209	0.099	1.5818E-11
Mzt1	7.5616E-16	0.56452583	0.191	0.088	1.6589E-11
Mpdu1	7.7077E-16	0.63517822	0.208	0.1	1.691E-11

Dcaf7	8.585E-16	0.44847875	0.218	0.105	1.8835E-11
Brix1	8.5952E-16	0.57451653	0.209	0.1	1.8857E-11
Nudt19	8.6358E-16	1.19560515	0.088	0.029	1.8946E-11
Mrps28	9.0394E-16	0.52994092	0.224	0.11	1.9832E-11
Cog6	9.1772E-16	1.07601999	0.095	0.033	2.0134E-11
Cspp1	9.2098E-16	0.45997802	0.215	0.103	2.0205E-11
Tsg101	9.3106E-16	0.52747907	0.249	0.127	2.0427E-11
Sumo3	9.6601E-16	0.50646503	0.215	0.104	2.1193E-11
Cd84	9.6697E-16	0.27389701	0.367	0.204	2.1214E-11
Fmnl1	9.9528E-16	0.26820267	0.379	0.214	2.1835E-11
Tdp1	9.9682E-16	1.46328134	0.052	0.013	2.1869E-11
Letm1	1.011E-15	0.56373868	0.2	0.095	2.2179E-11
Gemin7	1.0691E-15	0.45781796	0.229	0.112	2.3454E-11
Ptpn2	1.1074E-15	0.26120198	0.411	0.237	2.4295E-11
Cytip	1.1091E-15	0.25456246	0.964	0.739	2.4332E-11
Afg3l1	1.1176E-15	0.6386048	0.195	0.091	2.452E-11
Mcrs1	1.1324E-15	0.62949083	0.186	0.086	2.4845E-11
Golim4	1.1665E-15	0.65575674	0.141	0.058	2.5592E-11
Lman2	1.1917E-15	0.359063	0.465	0.28	2.6144E-11
Rmnd5a	1.2013E-15	0.42078412	0.252	0.127	2.6355E-11
Ptrhd1	1.2024E-15	0.77499263	0.199	0.096	2.638E-11
Safb	1.2251E-15	0.33674061	0.354	0.197	2.6876E-11
Ppp1r7	1.2418E-15	0.6582455	0.165	0.073	2.7243E-11
Ptger2	1.2837E-15	1.18221736	0.081	0.026	2.8164E-11
Snrpb2	1.3216E-15	0.28656518	0.424	0.248	2.8993E-11
Cast	1.3254E-15	0.25757821	0.474	0.283	2.9078E-11
Psmc2	1.3261E-15	0.55023961	0.243	0.124	2.9093E-11
Add1	1.3318E-15	0.48818164	0.234	0.116	2.9219E-11
Susd3	1.3352E-15	0.80891091	0.14	0.058	2.9294E-11
Rab23	1.4319E-15	1.32099708	0.043	0.009	3.1413E-11
Zmynd8	1.5339E-15	0.5370747	0.216	0.105	3.3652E-11
Nif3l1	1.6021E-15	1.15518216	0.079	0.025	3.5148E-11
Luc7l	1.6199E-15	0.47341312	0.249	0.126	3.554E-11
Rbck1	1.6275E-15	0.40845267	0.252	0.129	3.5706E-11
Mrps15	1.6515E-15	0.60457747	0.242	0.125	3.6233E-11
Adrm1	1.6994E-15	0.55880082	0.25	0.13	3.7283E-11
Cul2	1.737E-15	0.48567741	0.17	0.075	3.8108E-11
Pcdhgc5	1.743E-15	2.82180449	0.013	0.001	3.8239E-11
Ttll12	1.7557E-15	1.34469493	0.057	0.015	3.8519E-11
Luc7l3	1.7981E-15	0.35202287	0.356	0.2	3.9448E-11
Rnf5	1.8161E-15	0.98165461	0.118	0.046	3.9844E-11
Pafah1b1	1.853E-15	0.27691535	0.83	0.586	4.0653E-11
Jazf1	1.8589E-15	1.94701657	0.03	0.005	4.0782E-11
Cstf2	1.8719E-15	0.55378145	0.129	0.051	4.1067E-11
Nup37	2.0011E-15	1.66099735	0.041	0.009	4.3903E-11
Mtch2	2.0769E-15	0.37751388	0.324	0.179	4.5565E-11
Man2a2	2.0791E-15	0.37854657	0.233	0.114	4.5613E-11
Eif1b	2.1506E-15	0.3791612	0.265	0.137	4.7181E-11

Smim20	2.1904E-15	0.74868188	0.159	0.07	4.8055E-11
Ttc32	2.2698E-15	0.93616875	0.079	0.025	4.9798E-11
Txndc9	2.2907E-15	0.38059645	0.329	0.184	5.0255E-11
Entpd5	2.4385E-15	1.18532064	0.07	0.021	5.3499E-11
Ddb1	2.441E-15	0.48372626	0.261	0.136	5.3554E-11
Rnf139	2.4476E-15	0.36361662	0.166	0.073	5.3699E-11
Ripk3	2.4558E-15	1.05204516	0.1	0.036	5.3877E-11
Polr3f	2.465E-15	0.60405997	0.131	0.052	5.408E-11
Sec13	2.7584E-15	0.4430601	0.222	0.11	6.0516E-11
Mrpl27	2.7749E-15	0.82435524	0.174	0.081	6.0879E-11
Pdcd2	2.9234E-15	0.54389108	0.157	0.068	6.4138E-11
Zfand2b	3.0173E-15	0.55159644	0.175	0.08	6.6197E-11
Mrps10	3.0946E-15	0.70422139	0.123	0.049	6.7892E-11
Trav9n-4	3.1094E-15	3.46421625	0.013	0.001	6.8218E-11
Tbpl1	3.1144E-15	0.47745758	0.184	0.085	6.8328E-11
Kpna2	3.1807E-15	1.4397106	0.059	0.016	6.9781E-11
Pdik1l	3.2208E-15	1.08427993	0.077	0.024	7.0661E-11
Zfp362	3.2414E-15	0.71178758	0.1	0.036	7.1113E-11
Trappc6a	3.2856E-15	0.62703104	0.215	0.107	7.2084E-11
Pigyl	3.453E-15	0.99556076	0.123	0.05	7.5755E-11
Pxmp2	3.4548E-15	1.90607723	0.027	0.004	7.5796E-11
Pced1b	3.4619E-15	0.81740942	0.084	0.027	7.595E-11
Rbfa	3.5764E-15	0.45758403	0.202	0.096	7.8463E-11
Abhd18	3.6027E-15	1.11032017	0.089	0.03	7.904E-11
Bag6	3.8097E-15	0.46620666	0.242	0.124	8.358E-11
Dnm1l	3.8099E-15	0.41594625	0.245	0.125	8.3585E-11
St8sia1	3.8622E-15	0.70527678	0.082	0.027	8.4734E-11
Mrps9	3.9201E-15	0.48495626	0.213	0.104	8.6004E-11
Pcna-ps2	4.1494E-15	1.74386283	0.041	0.009	9.1035E-11
Zc3h18	4.1737E-15	0.38609077	0.263	0.137	9.1567E-11
Cnot1	4.1807E-15	0.28678826	0.422	0.245	9.1721E-11
Coro2a	4.2255E-15	0.65028567	0.161	0.071	9.2702E-11
Trbv3	4.235E-15	2.045407	0.027	0.004	9.2911E-11
Asxl1	4.3336E-15	0.60451411	0.179	0.082	9.5075E-11
Gpatch11	4.3522E-15	0.67330574	0.136	0.056	9.5482E-11
Rsl24d1	4.4473E-15	0.3919613	0.234	0.118	9.757E-11
Pop4	4.7077E-15	0.71881702	0.147	0.063	1.0328E-10
Zcwpw1	4.7461E-15	1.40654407	0.05	0.012	1.0412E-10
Mapkapk3	5.1025E-15	0.56252293	0.263	0.141	1.1194E-10
Mrps22	5.1055E-15	1.20129452	0.082	0.027	1.1201E-10
Rnf6	5.278E-15	0.32817239	0.309	0.169	1.1579E-10
Ino80c	5.3386E-15	0.81362499	0.136	0.057	1.1712E-10
Bet1	5.4361E-15	0.62486843	0.175	0.081	1.1926E-10
Aven	5.5784E-15	0.74650087	0.116	0.045	1.2238E-10
Mta2	5.7204E-15	0.43289435	0.252	0.129	1.255E-10
Xrcc2	5.7333E-15	1.547353	0.032	0.006	1.2578E-10
Myh9	5.9711E-15	0.26542584	0.816	0.581	1.31E-10
Exosc9	5.9826E-15	0.75396372	0.12	0.047	1.3125E-10

Brd3	6.193E-15	0.44638052	0.186	0.086	1.3587E-10
Siah1b	6.3051E-15	1.71507144	0.039	0.008	1.3833E-10
Smg5	6.3322E-15	0.39701766	0.211	0.102	1.3892E-10
Naa16	6.349E-15	0.61297536	0.114	0.044	1.3929E-10
Ccdc47	6.5074E-15	0.52775463	0.206	0.1	1.4277E-10
Rps6ka1	6.5228E-15	0.74012072	0.165	0.074	1.431E-10
Miip	6.8074E-15	0.99436571	0.081	0.026	1.4935E-10
Prr7	7.0276E-15	0.25725725	0.306	0.163	1.5418E-10
Gypc	7.2058E-15	0.51878627	0.131	0.053	1.5809E-10
Zbtb25	7.2669E-15	0.54838962	0.134	0.055	1.5943E-10
Bzw2	7.5582E-15	0.29415416	0.324	0.177	1.6582E-10
Rrad	7.8312E-15	0.35673825	0.195	0.092	1.7181E-10
Timm8a1	7.8664E-15	0.64399573	0.138	0.058	1.7258E-10
Zfp830	8.2497E-15	0.50677729	0.186	0.087	1.8099E-10
Cnep1r1	8.3151E-15	0.67668544	0.136	0.057	1.8243E-10
Tdg	8.3837E-15	0.50494496	0.218	0.109	1.8393E-10
Thumpd1	8.4553E-15	0.43137229	0.186	0.086	1.855E-10
Mphosph8	8.4972E-15	0.34194801	0.202	0.096	1.8642E-10
Dcaf1	8.5721E-15	0.44445232	0.174	0.078	1.8806E-10
Puf60	8.5905E-15	0.32620144	0.415	0.243	1.8847E-10
Mea1	8.6596E-15	0.46649679	0.213	0.105	1.8998E-10
Mthfd1l	9.0313E-15	0.84709409	0.077	0.024	1.9814E-10
Mdm1	9.0738E-15	1.19183278	0.041	0.009	1.9907E-10
Rint1	9.0778E-15	0.78645048	0.088	0.03	1.9916E-10
Rce1	9.2512E-15	0.75260774	0.129	0.053	2.0296E-10
Sys1	9.3728E-15	0.31125983	0.521	0.324	2.0563E-10
Psmb10	9.3978E-15	0.3821006	0.352	0.204	2.0618E-10
Accs	9.7035E-15	1.3089887	0.054	0.014	2.1288E-10
Rab37	9.7534E-15	0.66500592	0.1	0.036	2.1398E-10
Toe1	1.0116E-14	0.71483192	0.097	0.034	2.2193E-10
Chic2	1.0368E-14	0.26355878	0.463	0.281	2.2746E-10
Hltf	1.1039E-14	0.40770583	0.197	0.094	2.4218E-10
Mocs2	1.113E-14	0.63897402	0.175	0.082	2.4417E-10
Cnot2	1.1151E-14	0.31198676	0.258	0.133	2.4464E-10
Txlna	1.1205E-14	0.38647821	0.2	0.096	2.4583E-10
Synj2	1.1304E-14	0.83042469	0.102	0.038	2.4799E-10
Rab35	1.1777E-14	0.37773992	0.238	0.121	2.5837E-10
Sec23b	1.194E-14	0.38752298	0.242	0.124	2.6196E-10
Eif3k	1.2067E-14	0.34280124	0.805	0.568	2.6473E-10
Rap1a	1.2079E-14	0.25585686	0.585	0.37	2.65E-10
C2cd3	1.2101E-14	0.60625982	0.125	0.05	2.6548E-10
Ccser2	1.2327E-14	0.39856446	0.236	0.12	2.7045E-10
Washc1	1.2433E-14	0.80172505	0.123	0.05	2.7276E-10
Haus2	1.3015E-14	0.72711912	0.107	0.04	2.8553E-10
Gm17018	1.3333E-14	0.6727163	0.147	0.064	2.9251E-10
Med19	1.3371E-14	0.70960003	0.138	0.058	2.9335E-10
Zdhhc21	1.3624E-14	0.36361695	0.208	0.101	2.989E-10
Slfn3	1.3717E-14	1.8179527	0.025	0.004	3.0095E-10

Cfdp1	1.3768E-14	0.38901151	0.265	0.142	3.0206E-10
Rbm42	1.3922E-14	0.36927745	0.345	0.198	3.0543E-10
Pde12	1.4106E-14	0.56416511	0.12	0.047	3.0947E-10
Ctps	1.4213E-14	1.00619643	0.063	0.018	3.1182E-10
Wdr48	1.4513E-14	0.5187412	0.193	0.092	3.184E-10
E2f4	1.49E-14	0.56288899	0.166	0.075	3.2689E-10
Polr3c	1.502E-14	0.55782386	0.175	0.081	3.2953E-10
Pgrmc2	1.5284E-14	0.54887527	0.174	0.08	3.353E-10
Plekhf1	1.6052E-14	1.28637557	0.047	0.011	3.5216E-10
Ergic2	1.678E-14	0.40395326	0.284	0.155	3.6814E-10
Dhx36	1.687E-14	0.25641818	0.404	0.237	3.7012E-10
Nucb1	1.7896E-14	0.39324625	0.297	0.164	3.9263E-10
Prkacb	1.7973E-14	0.30509798	0.284	0.152	3.9431E-10
Keap1	1.8054E-14	0.72931124	0.12	0.048	3.9609E-10
Map3k7	1.8503E-14	0.57628945	0.172	0.079	4.0594E-10
Gm42743	1.8596E-14	2.55621284	0.013	0.001	4.0798E-10
Ppm1b	1.8673E-14	0.28191458	0.252	0.129	4.0966E-10
Ywhae	1.8831E-14	0.27574159	0.732	0.499	4.1314E-10
Nelfb	1.9232E-14	0.44941038	0.179	0.083	4.2193E-10
Wdyhv1	1.9506E-14	0.84669069	0.104	0.039	4.2795E-10
C1d	2.1056E-14	0.5195716	0.197	0.096	4.6194E-10
Eif2s2	2.182E-14	0.2634717	0.896	0.675	4.7871E-10
Zmym1	2.1853E-14	0.78650119	0.097	0.035	4.7944E-10
Gabpa	2.2085E-14	0.61164337	0.179	0.085	4.8453E-10
B3galt6	2.2453E-14	1.2987869	0.048	0.012	4.9259E-10
Zfp326	2.3051E-14	0.39522217	0.261	0.139	5.0571E-10
Tlk1	2.3457E-14	0.29250593	0.322	0.177	5.1462E-10
4930453N24R	2.3842E-14	0.51119865	0.197	0.095	5.2307E-10
Phax	2.5687E-14	0.50514544	0.211	0.105	5.6354E-10
Slain2	2.6428E-14	0.28228161	0.258	0.134	5.798E-10
Cep162	2.6891E-14	1.00674285	0.077	0.025	5.8995E-10
Kpna3	2.7516E-14	0.51941366	0.211	0.106	6.0367E-10
Aplf	2.8169E-14	2.06122602	0.03	0.006	6.1801E-10
Thrap3	2.8479E-14	0.25305892	0.615	0.397	6.248E-10
Marc2	3.0878E-14	0.471198	0.275	0.151	6.7742E-10
2410015M20R	3.1148E-14	0.25485725	0.499	0.303	6.8335E-10
Rdh11	3.2771E-14	0.43726583	0.116	0.046	7.1897E-10
Dhrs13	3.3903E-14	1.89142256	0.023	0.004	7.438E-10
Bud31	3.4797E-14	0.28499837	0.37	0.215	7.6342E-10
Tpgs1	3.5191E-14	0.96004911	0.116	0.047	7.7206E-10
Tsfm	3.6172E-14	1.06784823	0.079	0.026	7.9358E-10
Ptpn7	3.6279E-14	0.58530629	0.141	0.061	7.9593E-10
Parp2	3.6727E-14	0.58870267	0.147	0.065	8.0575E-10
Yif1b	3.8223E-14	0.76573851	0.172	0.082	8.3857E-10
Pdxp	3.9146E-14	1.29969678	0.036	0.007	8.5883E-10
Zfp941	4.0199E-14	2.69050468	0.02	0.003	8.8193E-10
Msh2	4.1165E-14	0.85473921	0.091	0.033	9.0312E-10
Gbp3	4.1533E-14	0.44496257	0.224	0.115	9.1119E-10

Ncbp3	4.2004E-14	0.37611575	0.197	0.095	9.2153E-10
Zcchc9	4.4218E-14	0.38485321	0.306	0.17	9.7009E-10
Smn1	4.487E-14	0.60440024	0.177	0.084	9.8441E-10
G2e3	4.5008E-14	0.59250868	0.136	0.058	9.8743E-10
Stk11	4.5325E-14	0.26228975	0.386	0.224	9.9439E-10
Immt	4.8016E-14	0.30155406	0.261	0.139	1.0534E-09
Creld2	4.8158E-14	0.51826262	0.242	0.127	1.0565E-09
Mpst	4.94E-14	0.95569992	0.122	0.05	1.0838E-09
Med31	5.0504E-14	0.40028834	0.175	0.082	1.108E-09
Utp18	5.2266E-14	0.53116713	0.208	0.105	1.1467E-09
Pelp1	5.3516E-14	0.69564532	0.1	0.037	1.1741E-09
Slf1	5.4769E-14	0.75632188	0.089	0.032	1.2016E-09
Hinfp	5.4788E-14	0.58044634	0.113	0.044	1.202E-09
Rtca	5.6451E-14	1.04731886	0.095	0.035	1.2385E-09
Arpc4	5.6486E-14	0.25360197	0.608	0.393	1.2392E-09
Ing1	6.176E-14	0.29794736	0.258	0.136	1.355E-09
Brcc3	6.5815E-14	0.56811462	0.148	0.066	1.4439E-09
Traf7	6.584E-14	0.60204395	0.148	0.066	1.4445E-09
Mapk3	6.7347E-14	0.54992792	0.193	0.096	1.4775E-09
Coa6	6.7608E-14	0.70593739	0.129	0.054	1.4833E-09
Strn	6.7618E-14	0.53764446	0.14	0.06	1.4835E-09
Ube2j2	6.776E-14	0.31442702	0.377	0.222	1.4866E-09
Ide	6.8078E-14	0.52779797	0.161	0.073	1.4936E-09
Armcx5	6.8506E-14	0.48710115	0.134	0.057	1.503E-09
Susd2	7.0866E-14	1.96272381	0.029	0.005	1.5547E-09
Sart3	7.5119E-14	0.5579911	0.157	0.071	1.648E-09
Gpi1	7.6307E-14	0.27250589	0.621	0.407	1.6741E-09
Spag7	7.7084E-14	0.50628107	0.216	0.111	1.6911E-09
2310033P09R	8.0216E-14	0.42208537	0.188	0.09	1.7599E-09
Cct8	8.0778E-14	0.32995725	0.445	0.277	1.7722E-09
Exoc4	8.5688E-14	0.41095191	0.195	0.095	1.8799E-09
Hsd11b1	8.6739E-14	0.9319882	0.073	0.024	1.903E-09
Mff	9.1209E-14	0.33999205	0.306	0.173	2.001E-09
Lin37	9.3163E-14	0.89545149	0.093	0.034	2.0439E-09
Phtf1	9.6078E-14	0.54873496	0.106	0.04	2.1079E-09
Prpf38a	9.9108E-14	0.37292172	0.224	0.115	2.1743E-09
Ufd1	9.98E-14	0.51447609	0.193	0.095	2.1895E-09
Dnaja4	9.9965E-14	0.39704812	0.104	0.039	2.1931E-09
Psmc1	1.0262E-13	0.46578178	0.225	0.117	2.2515E-09
Aup1	1.0306E-13	0.26100574	0.395	0.235	2.261E-09
Eif3l	1.0733E-13	0.48437507	0.268	0.149	2.3547E-09
Cdadc1	1.0745E-13	0.55991389	0.136	0.058	2.3574E-09
Ddx11	1.0835E-13	1.2771928	0.045	0.011	2.3771E-09
Cbll1	1.0945E-13	0.30777386	0.222	0.112	2.4013E-09
Csnk2a1	1.1008E-13	0.34221039	0.311	0.176	2.4151E-09
Pkp4	1.1326E-13	0.76545023	0.106	0.041	2.4849E-09
Cops4	1.1436E-13	0.32146118	0.27	0.147	2.5089E-09
Suv39h2	1.1685E-13	1.16818912	0.048	0.012	2.5636E-09

Isca1	1.1909E-13	0.30018081	0.202	0.099	2.6128E-09
Arsb	1.1978E-13	0.81456447	0.109	0.043	2.6278E-09
Rab10os	1.2265E-13	0.4278475	0.286	0.16	2.6908E-09
Cdc42	1.2382E-13	0.25268018	0.943	0.743	2.7165E-09
Synj2bp	1.274E-13	0.33755328	0.2	0.098	2.7951E-09
Chd1l	1.2841E-13	0.78712606	0.079	0.027	2.8171E-09
Tspan5	1.2841E-13	0.8158164	0.102	0.039	2.8172E-09
Tbcb	1.2983E-13	0.4795737	0.345	0.207	2.8484E-09
Ciapin1	1.3038E-13	0.37469058	0.191	0.093	2.8603E-09
Chmp2a	1.3142E-13	0.28505983	0.564	0.363	2.8831E-09
Agbl5	1.3352E-13	1.87590193	0.03	0.006	2.9293E-09
Commd2	1.3387E-13	0.54738599	0.166	0.078	2.9369E-09
Pik3ca	1.4112E-13	0.45225134	0.204	0.101	3.0959E-09
Tnfaip8l1	1.4422E-13	1.43164747	0.055	0.016	3.1642E-09
Ash2l	1.4569E-13	0.62285196	0.14	0.061	3.1963E-09
Herc3	1.4745E-13	0.57085103	0.116	0.047	3.2349E-09
Pom121	1.4794E-13	0.3174819	0.225	0.115	3.2456E-09
Rad52	1.4815E-13	1.25779841	0.059	0.017	3.2503E-09
Acap3	1.503E-13	1.15728728	0.054	0.015	3.2974E-09
Exosc3	1.5141E-13	0.36302644	0.215	0.109	3.3218E-09
Cenpj	1.5447E-13	1.27743719	0.045	0.011	3.3888E-09
Tuba4a	1.5574E-13	0.38943663	0.165	0.076	3.4169E-09
Rnf126	1.6001E-13	0.91718278	0.102	0.04	3.5105E-09
A630023P12R	1.6269E-13	0.99279887	0.05	0.013	3.5693E-09
Clp1	1.6703E-13	0.40500049	0.152	0.068	3.6644E-09
Oxct1	1.6904E-13	0.41807483	0.2	0.101	3.7086E-09
Mosmo	1.7366E-13	0.45530204	0.152	0.068	3.81E-09
Polr2e	1.7899E-13	0.41637432	0.27	0.149	3.9268E-09
Trappc1	1.8043E-13	0.94588141	0.148	0.069	3.9584E-09
Aasdhppt	1.8496E-13	0.72908326	0.134	0.058	4.0578E-09
Srcap	1.8974E-13	0.4544947	0.197	0.097	4.1628E-09
Ino80e	1.8988E-13	0.42737132	0.161	0.074	4.1658E-09
Cox18	1.9067E-13	0.71739497	0.086	0.03	4.1832E-09
Cenpb	1.9739E-13	0.6295874	0.2	0.102	4.3305E-09
Nmral1	2.0071E-13	1.74982477	0.054	0.015	4.4033E-09
Map4	2.0198E-13	0.40349989	0.234	0.123	4.4313E-09
Cpsf2	2.0355E-13	0.27903659	0.209	0.105	4.4658E-09
Cd160	2.0532E-13	0.98216153	0.073	0.024	4.5046E-09
Lamtor4	2.0832E-13	0.40267596	0.299	0.17	4.5703E-09
Ints1	2.1364E-13	0.61249221	0.113	0.045	4.6871E-09
Zranb3	2.1582E-13	1.87967322	0.032	0.007	4.7349E-09
Nol11	2.1773E-13	0.37504176	0.199	0.098	4.7768E-09
Crkl	2.1897E-13	0.46286039	0.184	0.089	4.804E-09
Akirin1	2.2059E-13	0.27342827	0.379	0.226	4.8395E-09
Cnot3	2.2164E-13	0.3032893	0.304	0.171	4.8625E-09
Trappc4	2.3991E-13	0.34790746	0.322	0.186	5.2633E-09
Uba6	2.4061E-13	0.36723842	0.202	0.101	5.2788E-09
Tigit	2.4288E-13	0.73011623	0.073	0.024	5.3284E-09

Atxn2l	2.4541E-13	0.27283591	0.374	0.221	5.384E-09
Prdm1	2.4931E-13	0.33009042	0.32	0.183	5.4695E-09
Dhrs7	2.5131E-13	0.65348045	0.166	0.08	5.5134E-09
Slc7a5	2.585E-13	0.60603877	0.138	0.06	5.6713E-09
Mical1	2.5968E-13	0.36995721	0.243	0.13	5.6971E-09
Ptrh2	2.615E-13	0.75885297	0.114	0.047	5.7371E-09
Zfp560	2.6649E-13	0.34961903	0.159	0.073	5.8465E-09
U2af2	2.667E-13	0.26129801	0.369	0.217	5.8512E-09
Dhrs4	2.8429E-13	0.88812661	0.098	0.038	6.237E-09
Pdha1	2.9754E-13	0.31775904	0.267	0.145	6.5278E-09
Apip	2.9867E-13	0.84194304	0.107	0.043	6.5525E-09
Mtmr9	3.0112E-13	0.96037645	0.066	0.021	6.6062E-09
Ift27	3.0294E-13	0.93701057	0.081	0.028	6.6462E-09
Anapc1	3.0798E-13	0.34301027	0.2	0.099	6.7568E-09
Mrto4	3.1588E-13	0.55535438	0.199	0.101	6.9302E-09
Dnajc4	3.2362E-13	0.96300334	0.098	0.038	7.0999E-09
Trappc6b	3.5161E-13	0.29105448	0.329	0.19	7.714E-09
Ecm2	3.6674E-13	2.20771572	0.016	0.002	8.0458E-09
Thoc6	3.6742E-13	0.86121629	0.111	0.045	8.0608E-09
E2f3	3.6965E-13	1.059505	0.082	0.029	8.1097E-09
Rbm48	3.8878E-13	0.50732	0.136	0.059	8.5294E-09
Adsl	3.9244E-13	0.66087525	0.1	0.039	8.6097E-09
Eif4e2	3.972E-13	0.36650191	0.386	0.236	8.7141E-09
Net1	3.9998E-13	0.57429396	0.123	0.052	8.7751E-09
Mtf2	4.0793E-13	0.25368504	0.259	0.138	8.9497E-09
Phf11b	4.0945E-13	0.65807691	0.147	0.067	8.9828E-09
March5	4.1864E-13	0.27523045	0.315	0.179	9.1845E-09
Utp11	4.2673E-13	0.33464951	0.242	0.128	9.3621E-09
Mrps25	4.2845E-13	0.69997944	0.145	0.066	9.3998E-09
Ston2	4.3295E-13	0.78935367	0.14	0.063	9.4984E-09
Sc5d	4.3665E-13	0.43527351	0.172	0.082	9.5796E-09
Ap1s2	4.3706E-13	0.45209666	0.233	0.125	9.5888E-09
Klhl25	4.5453E-13	0.98780628	0.064	0.02	9.972E-09
Capn2	4.5552E-13	0.53038162	0.19	0.095	9.9936E-09
Ndufs1	4.5572E-13	0.39483991	0.182	0.089	9.9981E-09
Gm47283	4.5729E-13	0.36511957	0.292	0.165	1.0033E-08
Nrd1	4.8041E-13	0.26385999	0.463	0.291	1.054E-08
Spice1	4.8427E-13	1.20973001	0.055	0.016	1.0624E-08
Cdan1	4.9844E-13	0.97215277	0.063	0.019	1.0935E-08
Selenos	4.9971E-13	0.26975801	0.624	0.419	1.0963E-08
Nlk	4.9994E-13	0.42719794	0.143	0.063	1.0968E-08
Gm17092	5.0294E-13	1.85246682	0.03	0.006	1.1034E-08
Usp39	5.0525E-13	0.42091497	0.136	0.059	1.1085E-08
Fam78a	5.3112E-13	0.92022845	0.068	0.022	1.1652E-08
Tmem11	5.3516E-13	0.40440826	0.197	0.099	1.1741E-08
Nelfcd	5.4188E-13	0.94329706	0.075	0.026	1.1888E-08
Med15	5.4872E-13	0.27079118	0.281	0.156	1.2038E-08
Psrc1	5.5409E-13	2.92113482	0.021	0.003	1.2156E-08

2610002M06F	5.9605E-13	0.88456375	0.084	0.03	1.3077E-08
Parp16	6.1065E-13	1.92882667	0.02	0.003	1.3397E-08
Got1	6.178E-13	0.31086468	0.658	0.458	1.3554E-08
4632415L05Ri	6.3301E-13	1.20795603	0.047	0.012	1.3888E-08
Pigt	6.4003E-13	0.46206324	0.184	0.092	1.4042E-08
Pmm1	6.4728E-13	1.07408728	0.086	0.032	1.4201E-08
1110038F14Ri	6.587E-13	0.46131129	0.206	0.105	1.4451E-08
Ccz1	6.8606E-13	0.33364166	0.242	0.129	1.5051E-08
Tbl2	7.0051E-13	1.02206079	0.052	0.015	1.5368E-08
Nifk	7.1134E-13	0.39596867	0.168	0.08	1.5606E-08
B3galt2	7.451E-13	0.79962744	0.061	0.019	1.6347E-08
Ing3	7.484E-13	0.29899208	0.265	0.145	1.6419E-08
Btg3	8.1168E-13	0.42570818	0.132	0.057	1.7807E-08
Rell1	8.2445E-13	0.40912905	0.186	0.093	1.8088E-08
Gfer	8.3641E-13	0.77111362	0.143	0.066	1.835E-08
Pdzd8	8.5103E-13	0.334053	0.191	0.095	1.8671E-08
Selenoi	8.833E-13	0.58018352	0.143	0.065	1.9379E-08
Nsmaf	8.8377E-13	0.38339607	0.231	0.122	1.9389E-08
Cep135	8.8652E-13	0.42639672	0.154	0.071	1.9449E-08
Mtx1	8.9097E-13	0.71548362	0.125	0.055	1.9547E-08
Tmigd1	8.9377E-13	2.57567479	0.011	0.001	1.9608E-08
Ythdc2	8.9534E-13	0.52081774	0.145	0.065	1.9643E-08
Spata2	9.1774E-13	0.49249007	0.157	0.074	2.0134E-08
Mbp	9.3944E-13	0.30356652	0.275	0.153	2.061E-08
Pstpip1	9.6057E-13	0.38199466	0.227	0.12	2.1074E-08
Ranbp9	9.6904E-13	0.44987663	0.215	0.112	2.126E-08
Gnptg	9.9785E-13	0.47869666	0.138	0.061	2.1892E-08
Eif3g	1.002E-12	0.29896971	0.349	0.207	2.1982E-08
Ptpn18	1.0533E-12	0.25112175	0.83	0.613	2.3108E-08
Nfx1	1.1464E-12	0.31130493	0.224	0.117	2.5151E-08
Lrrc49	1.1658E-12	2.20496031	0.029	0.006	2.5576E-08
Maip1	1.2067E-12	0.54691799	0.152	0.071	2.6474E-08
Sh2b1	1.2167E-12	0.33743227	0.15	0.068	2.6692E-08
Paxbp1	1.2273E-12	0.2585173	0.376	0.226	2.6925E-08
Trbv2	1.2349E-12	1.76477363	0.029	0.006	2.7093E-08
Cntln	1.2413E-12	1.0825358	0.068	0.022	2.7232E-08
Tmem60	1.2505E-12	0.47474575	0.148	0.068	2.7435E-08
Lcn4	1.2507E-12	1.09249449	0.05	0.014	2.744E-08
Itpripl1	1.2814E-12	0.96822014	0.066	0.021	2.8112E-08
Cep57l1	1.2909E-12	1.22367409	0.047	0.012	2.8321E-08
Setd6	1.2957E-12	1.2243263	0.052	0.015	2.8426E-08
Gramd1c	1.3172E-12	1.47967226	0.034	0.007	2.8898E-08
Kif5c	1.3385E-12	1.40274053	0.036	0.008	2.9365E-08
Rgs3	1.3487E-12	0.71270859	0.086	0.032	2.9589E-08
Glipr2	1.3977E-12	0.51928692	0.156	0.073	3.0664E-08
Ccdc66	1.4017E-12	0.81255653	0.068	0.022	3.0752E-08
Cetn2	1.4069E-12	0.2788589	0.292	0.165	3.0866E-08
Ttc9c	1.4223E-12	0.58226302	0.134	0.06	3.1204E-08

Noc4l	1.454E-12	0.90527532	0.097	0.038	3.1899E-08
Rab11a	1.4624E-12	0.31561772	0.356	0.216	3.2084E-08
Rabif	1.5093E-12	0.4762253	0.118	0.05	3.3113E-08
Zfp82	1.5455E-12	2.45159312	0.013	0.001	3.3907E-08
CAAA0111838	1.5859E-12	0.50825409	0.188	0.095	3.4792E-08
Srgap2	1.6078E-12	0.48958424	0.195	0.1	3.5273E-08
Nudcd3	1.615E-12	0.33717593	0.309	0.18	3.5431E-08
Zfpl1	1.6771E-12	0.81855549	0.093	0.036	3.6794E-08
Mturn	1.6817E-12	1.06207797	0.045	0.012	3.6895E-08
Rab24	1.6967E-12	0.44704351	0.202	0.105	3.7225E-08
Mbtps1	1.7085E-12	0.26105455	0.247	0.132	3.7482E-08
Pop7	1.7235E-12	0.58566436	0.19	0.097	3.7811E-08
Tomm70a	1.7376E-12	0.25594103	0.286	0.161	3.8122E-08
Dlg4	1.7561E-12	0.78558895	0.075	0.026	3.8527E-08
Bak1	1.7733E-12	0.64570972	0.182	0.093	3.8905E-08
Rnf166	1.8519E-12	0.41490742	0.222	0.117	4.0629E-08
Cebpg	1.8846E-12	0.25680616	0.317	0.183	4.1347E-08
Usf1	1.8949E-12	0.68701944	0.131	0.058	4.1573E-08
4833439L19Ri	1.9123E-12	0.50846948	0.181	0.09	4.1954E-08
Scrib	1.9137E-12	0.68826962	0.089	0.034	4.1984E-08
Pdcl3	1.9395E-12	0.45296665	0.224	0.12	4.2551E-08
Senp1	1.963E-12	0.44712106	0.161	0.077	4.3067E-08
Rbm14	1.9729E-12	0.5716893	0.111	0.046	4.3284E-08
Itgb1bp1	2.0049E-12	0.69220939	0.147	0.069	4.3986E-08
Smarcad1	2.1197E-12	0.29260191	0.225	0.119	4.6505E-08
Bicd2	2.1375E-12	0.59518645	0.129	0.057	4.6895E-08
Zhx3	2.2758E-12	1.41825322	0.043	0.011	4.9929E-08
Xpo7	2.3301E-12	0.4967428	0.141	0.064	5.112E-08
Ubr5	2.3624E-12	0.26124036	0.374	0.221	5.1828E-08
Agpat3	2.3879E-12	0.36131763	0.19	0.095	5.2389E-08
Aga	2.4995E-12	1.00356101	0.098	0.039	5.4835E-08
Asnsd1	2.5637E-12	0.49910791	0.156	0.074	5.6246E-08
Taf4	2.5836E-12	0.76450048	0.1	0.04	5.6681E-08
Ltn1	2.6008E-12	0.42022095	0.147	0.068	5.7059E-08
Atg16l2	2.7E-12	0.58993627	0.118	0.05	5.9234E-08
Lrrc8c	2.75E-12	0.37606402	0.215	0.113	6.0333E-08
Fxr2	3E-12	0.38454501	0.163	0.078	6.5817E-08
Tmem222	3.0257E-12	0.45873076	0.143	0.066	6.638E-08
Akap8	3.0524E-12	0.25137074	0.204	0.104	6.6966E-08
Auh	3.071E-12	0.47452484	0.236	0.13	6.7375E-08
Kxd1	3.2742E-12	0.25747702	0.302	0.174	7.1832E-08
Ipp	3.3774E-12	1.22383438	0.041	0.01	7.4097E-08
Chfr	3.3833E-12	0.31718629	0.254	0.14	7.4227E-08
Lamtor1	3.4735E-12	0.38582746	0.275	0.159	7.6205E-08
Yrdc	3.4872E-12	0.35392022	0.215	0.113	7.6506E-08
Cmas	3.677E-12	0.2543638	0.302	0.174	8.0671E-08
Sart1	3.7349E-12	0.29991853	0.24	0.13	8.194E-08
Ipo7	3.766E-12	0.26953331	0.306	0.176	8.2622E-08

Mpg	3.9027E-12	0.86625684	0.1	0.041	8.562E-08
Cebpzos	3.9894E-12	0.68982533	0.125	0.056	8.7522E-08
Cryl1	4.0683E-12	1.21052228	0.061	0.02	8.9254E-08
Aktip	4.3662E-12	0.91961637	0.07	0.024	9.5791E-08
Ift80	4.4716E-12	0.54182188	0.072	0.024	9.8101E-08
Eef1e1	4.4835E-12	0.42760786	0.197	0.102	9.8363E-08
Smu1	4.4909E-12	0.40501176	0.233	0.127	9.8525E-08
Rwdd2b	4.707E-12	1.41936106	0.041	0.011	1.0327E-07
Mrps7	4.8026E-12	0.29484052	0.247	0.136	1.0536E-07
Ddx47	5.0943E-12	0.29011005	0.277	0.158	1.1176E-07
Magi3	5.1477E-12	0.9340177	0.059	0.019	1.1293E-07
Slc9a5	5.176E-12	2.82006145	0.013	0.001	1.1356E-07
Setdb1	5.3711E-12	0.79532612	0.098	0.04	1.1784E-07
Unc50	5.3712E-12	0.49735949	0.172	0.085	1.1784E-07
Tmem238	5.4014E-12	1.02815475	0.093	0.037	1.185E-07
Wdr18	5.4281E-12	0.57238341	0.127	0.057	1.1909E-07
Zfp414	5.4581E-12	1.03096451	0.064	0.021	1.1974E-07
Itga2	5.6E-12	0.98477718	0.039	0.01	1.2286E-07
Herc6	5.6152E-12	0.35685823	0.125	0.055	1.2319E-07
Ttc5	5.7742E-12	0.32674385	0.145	0.067	1.2668E-07
Rps6ka3	5.8158E-12	0.32873103	0.245	0.135	1.2759E-07
Vps16	5.931E-12	0.52727589	0.131	0.059	1.3012E-07
Narf	5.9514E-12	0.50944879	0.127	0.056	1.3057E-07
1110004E09R	6.0097E-12	0.82962215	0.084	0.032	1.3185E-07
2510039O18R	6.1091E-12	0.43454285	0.172	0.085	1.3403E-07
Ppa1	6.1616E-12	0.74409895	0.156	0.076	1.3518E-07
Kansl3	6.2038E-12	0.26493057	0.243	0.133	1.3611E-07
Zc3hc1	6.2151E-12	0.63862333	0.109	0.046	1.3635E-07
Cst7	6.222E-12	0.42272856	0.132	0.059	1.365E-07
Mri1	6.3282E-12	0.48511496	0.159	0.077	1.3883E-07
Nup188	6.478E-12	0.83493194	0.081	0.03	1.4212E-07
Polh	6.4998E-12	2.08756878	0.023	0.004	1.426E-07
Ccdc77	6.5131E-12	0.97698563	0.047	0.013	1.4289E-07
Adprh	6.6111E-12	0.41178845	0.22	0.121	1.4504E-07
Mettl16	6.8675E-12	0.35080673	0.143	0.066	1.5067E-07
Fam126b	6.8967E-12	0.64485929	0.095	0.037	1.5131E-07
Capg	7.0385E-12	0.44330876	0.324	0.196	1.5442E-07
4930503L19Ri	7.0573E-12	0.26307987	0.166	0.081	1.5483E-07
Tmem9b	7.1203E-12	0.45102717	0.191	0.099	1.5621E-07
Wipi2	7.14E-12	0.27557129	0.209	0.109	1.5664E-07
B3galt5	7.1872E-12	0.76648888	0.072	0.025	1.5768E-07
Trgv2	7.1961E-12	1.40902032	0.045	0.012	1.5788E-07
Prps2	7.2823E-12	0.46873433	0.125	0.055	1.5977E-07
Ddx55	7.4582E-12	0.53656733	0.102	0.042	1.6363E-07
Pdzd11	7.759E-12	0.77940563	0.127	0.058	1.7023E-07
Ipo9	7.8099E-12	0.97380813	0.079	0.029	1.7134E-07
Paxx	8.0856E-12	0.49766052	0.125	0.055	1.7739E-07
Atl3	8.2505E-12	0.29391245	0.211	0.111	1.8101E-07

Tprgl	8.5457E-12	0.2555071	0.358	0.215	1.8748E-07
Ppp2r5d	8.5821E-12	0.76753893	0.091	0.036	1.8828E-07
Il23r	8.5987E-12	1.03456873	0.057	0.018	1.8865E-07
Rae1	9.0189E-12	0.39378779	0.15	0.071	1.9787E-07
Lxn	9.0328E-12	0.9249822	0.072	0.025	1.9817E-07
Nup35	9.2106E-12	0.97329053	0.052	0.015	2.0207E-07
Anapc2	9.313E-12	0.56504156	0.125	0.056	2.0432E-07
Psmd6	9.4155E-12	0.46721381	0.242	0.136	2.0657E-07
Topors	9.908E-12	0.36670239	0.193	0.1	2.1737E-07
Ptpn3	1.0084E-11	1.06644217	0.061	0.02	2.2122E-07
Gspt1	1.0165E-11	0.27172696	0.345	0.21	2.2301E-07
Naxd	1.0492E-11	0.3216158	0.179	0.09	2.3018E-07
Atxn10	1.0522E-11	0.49347534	0.191	0.1	2.3084E-07
Rrp8	1.0544E-11	0.39160966	0.122	0.053	2.3132E-07
Mrps34	1.0674E-11	0.44260445	0.188	0.097	2.3418E-07
Nsdhl	1.0766E-11	0.99451831	0.072	0.025	2.3619E-07
Arrdc1	1.0791E-11	0.31083341	0.172	0.085	2.3674E-07
L3mbtl2	1.1265E-11	0.93285811	0.055	0.017	2.4714E-07
Commd4	1.1494E-11	0.38271028	0.249	0.14	2.5216E-07
Ecsit	1.1562E-11	0.72264759	0.088	0.034	2.5366E-07
Hace1	1.1636E-11	0.58791355	0.089	0.035	2.5529E-07
Arpc1a	1.165E-11	0.27896688	0.238	0.131	2.5558E-07
Slc25a39	1.2083E-11	0.51950278	0.17	0.086	2.6509E-07
Stn1	1.2219E-11	0.77784885	0.1	0.041	2.6808E-07
Nrbf2	1.2463E-11	0.41299797	0.202	0.107	2.7343E-07
Fgf13	1.2496E-11	1.88372751	0.029	0.006	2.7414E-07
Prpf19	1.2545E-11	0.46695179	0.15	0.072	2.7522E-07
Zfp944	1.2728E-11	1.2861046	0.054	0.016	2.7924E-07
Cd7	1.2806E-11	0.60671829	0.147	0.07	2.8094E-07
Mob2	1.2828E-11	0.28708426	0.224	0.119	2.8144E-07
Wdr61	1.3069E-11	0.50916987	0.204	0.109	2.8672E-07
Med8	1.3685E-11	0.50261114	0.17	0.086	3.0024E-07
Traf3ip3	1.379E-11	0.28931222	0.15	0.071	3.0255E-07
Mrps35	1.4048E-11	0.5295519	0.123	0.055	3.0819E-07
Atg5	1.4138E-11	0.31207604	0.175	0.088	3.1018E-07
Vps51	1.4293E-11	0.77677166	0.129	0.059	3.1357E-07
Cib1	1.4509E-11	0.36153518	0.256	0.146	3.1831E-07
Slc35e1	1.4547E-11	0.39991863	0.159	0.078	3.1915E-07
Notch1	1.4986E-11	0.30732106	0.216	0.116	3.2878E-07
Tor1b	1.6006E-11	0.43847405	0.165	0.082	3.5116E-07
Zfp788	1.6068E-11	0.88414393	0.057	0.018	3.5253E-07
Rnf181	1.6158E-11	0.28191197	0.163	0.079	3.5449E-07
Mcmbp	1.6323E-11	0.31644498	0.186	0.095	3.5811E-07
Sec61a1	1.6443E-11	0.26797786	0.367	0.227	3.6075E-07
Vta1	1.6872E-11	0.47757927	0.179	0.092	3.7017E-07
Rasal3	1.6944E-11	0.27749093	0.147	0.068	3.7174E-07
Zfp410	1.7516E-11	0.42148478	0.132	0.06	3.8427E-07
Agpat5	1.793E-11	0.49544713	0.172	0.087	3.9336E-07

Adat2	1.9479E-11	1.15459982	0.066	0.023	4.2734E-07
Dxo	2.0308E-11	0.7257283	0.095	0.038	4.4554E-07
Pfas	2.0533E-11	1.21849259	0.043	0.012	4.5048E-07
Ruvbl2	2.0652E-11	0.90233715	0.072	0.026	4.5309E-07
Slc24a5	2.0928E-11	1.18800424	0.054	0.017	4.5913E-07
Ocel1	2.2383E-11	0.71484155	0.104	0.044	4.9106E-07
Dlat	2.3207E-11	0.67063576	0.098	0.041	5.0913E-07
Mex3b	2.3805E-11	0.78019541	0.072	0.026	5.2226E-07
Lrrc8b	2.4885E-11	0.77487407	0.089	0.036	5.4596E-07
Rabggtb	2.5187E-11	0.43915532	0.243	0.138	5.5258E-07
Kdm1a	2.5301E-11	0.25509642	0.37	0.229	5.5507E-07
2010015M23F	2.5319E-11	1.82846526	0.029	0.006	5.5548E-07
1700037H04R	2.663E-11	0.90007536	0.075	0.028	5.8424E-07
Ankra2	2.716E-11	0.56699176	0.14	0.066	5.9587E-07
Mmadhc	2.7553E-11	0.48589469	0.191	0.102	6.0449E-07
Ccdc115	2.7961E-11	0.58053457	0.12	0.054	6.1344E-07
Dgcr14	2.9523E-11	0.6442654	0.095	0.039	6.477E-07
Gpank1	2.9648E-11	0.37305363	0.14	0.066	6.5044E-07
Med21	2.9807E-11	0.35563624	0.208	0.112	6.5393E-07
Noa1	3.0826E-11	0.9948327	0.061	0.02	6.763E-07
Map3k4	3.1459E-11	0.62815973	0.109	0.047	6.9019E-07
Mrpl11	3.2108E-11	0.51316785	0.186	0.098	7.0443E-07
Rbm24	3.2486E-11	1.23972462	0.027	0.005	7.1271E-07
Bhlhb9	3.2561E-11	1.03577839	0.047	0.013	7.1437E-07
St6galnac2	3.2885E-11	1.62327758	0.025	0.005	7.2147E-07
Eif4e3	3.3421E-11	0.59836792	0.131	0.061	7.3323E-07
Capn1	3.3804E-11	0.41625411	0.174	0.088	7.4163E-07
Pygo2	3.3812E-11	0.87485585	0.066	0.023	7.4181E-07
Arhgap1	3.4237E-11	0.57015102	0.145	0.07	7.5112E-07
Nt5dc1	3.4795E-11	0.41688533	0.095	0.038	7.6337E-07
Scrt1	3.5196E-11	0.70728237	0.068	0.024	7.7217E-07
Klhdc4	3.5727E-11	0.55441894	0.118	0.053	7.8382E-07
Eif4enif1	3.5934E-11	0.45980321	0.134	0.063	7.8836E-07
Phrf1	3.6216E-11	0.2723454	0.204	0.108	7.9455E-07
Acot9	3.6783E-11	0.57682194	0.143	0.069	8.0699E-07
Cwc27	3.694E-11	0.67611686	0.102	0.043	8.1042E-07
Zfp235	3.9851E-11	1.11037362	0.034	0.008	8.7429E-07
Zfp64	4.0286E-11	0.92565725	0.07	0.025	8.8383E-07
Gid8	4.032E-11	0.48873867	0.152	0.075	8.8458E-07
Gosr1	4.0938E-11	0.55083088	0.145	0.07	8.9814E-07
Cep152	4.2469E-11	0.90494705	0.047	0.013	9.3174E-07
Yipf5	4.3858E-11	0.33587151	0.211	0.115	9.622E-07
Prkca	4.4526E-11	0.35614439	0.145	0.068	9.7685E-07
Panx1	4.5131E-11	0.37002923	0.136	0.064	9.9014E-07
Flt3l	4.5796E-11	0.56925201	0.102	0.043	1.0047E-06
Nsmce3	4.7069E-11	0.5633054	0.15	0.074	1.0327E-06
Ddb2	4.8252E-11	0.50557622	0.086	0.034	1.0586E-06
Kmt5c	4.8386E-11	1.05117779	0.057	0.019	1.0616E-06

Fancl	4.947E-11	0.84440239	0.077	0.029	1.0853E-06
Cyb5b	5.2489E-11	0.34894266	0.208	0.112	1.1516E-06
Clptm1	5.3523E-11	0.42900977	0.197	0.106	1.1742E-06
Ddhd2	5.4231E-11	0.43227996	0.154	0.076	1.1898E-06
Sirt7	5.4647E-11	0.30857693	0.211	0.114	1.1989E-06
Mettl4	5.4656E-11	1.39715642	0.041	0.011	1.1991E-06
Mtif3	5.4876E-11	0.74419604	0.072	0.026	1.2039E-06
Egln3	5.5217E-11	0.45910573	0.098	0.041	1.2114E-06
Zfp330	5.6273E-11	0.41955681	0.231	0.13	1.2346E-06
Dmap1	5.6344E-11	1.042195	0.064	0.022	1.2361E-06
Dnajc30	5.8493E-11	0.69045975	0.104	0.045	1.2833E-06
Slc4a2	5.9343E-11	0.70782393	0.098	0.041	1.3019E-06
Mrpl19	6.0316E-11	0.41166464	0.17	0.087	1.3233E-06
Rccd1	6.1139E-11	0.87768152	0.048	0.014	1.3413E-06
Parg	6.1445E-11	0.29282353	0.179	0.092	1.348E-06
Krcc1	6.1596E-11	0.39637693	0.2	0.109	1.3514E-06
Slc35b3	6.1767E-11	0.41665086	0.123	0.056	1.3551E-06
Taf2	6.2322E-11	0.4821039	0.107	0.046	1.3673E-06
Gtf3c1	6.3436E-11	0.36163857	0.141	0.067	1.3917E-06
Soat2	6.8317E-11	1.52739158	0.036	0.009	1.4988E-06
Gm26722	7.4097E-11	0.81429111	0.068	0.024	1.6256E-06
Jmjd6	7.4995E-11	0.48865478	0.163	0.083	1.6453E-06
Ccl20	7.5366E-11	1.49145239	0.011	0.001	1.6535E-06
Smim26	7.5567E-11	0.50695447	0.145	0.071	1.6579E-06
Ctdsp2	7.7164E-11	0.25188147	0.258	0.147	1.6929E-06
Ap1s1	7.7775E-11	0.77772598	0.114	0.052	1.7063E-06
Yars2	8.0993E-11	1.13153581	0.05	0.015	1.7769E-06
Sbf1	8.1996E-11	0.2721859	0.199	0.105	1.7989E-06
Akt2	8.9711E-11	0.27869487	0.233	0.13	1.9682E-06
Tmem101	9.0497E-11	1.17594129	0.052	0.016	1.9854E-06
Fam192a	9.0754E-11	0.31518601	0.202	0.109	1.991E-06
Sntb1	9.1136E-11	0.44120176	0.097	0.04	1.9994E-06
Ptpn11	9.2432E-11	0.30556827	0.243	0.139	2.0279E-06
Apoo	9.4492E-11	1.84231989	0.038	0.01	2.0731E-06
Idh3b	9.7772E-11	0.25853973	0.293	0.176	2.145E-06
Rab1b	9.8712E-11	0.43938415	0.195	0.106	2.1656E-06
Sdcbp2	9.8806E-11	0.75161684	0.095	0.04	2.1677E-06
Mpzl3	9.9123E-11	0.85466343	0.068	0.024	2.1747E-06
Usp42	1.0009E-10	0.53429844	0.1	0.043	2.1959E-06
Wdr5	1.0065E-10	0.65357598	0.113	0.05	2.2081E-06
Eefsec	1.012E-10	0.64522999	0.073	0.027	2.2202E-06
Mrpl2	1.0527E-10	0.59654228	0.129	0.061	2.3095E-06
Cxxc1	1.064E-10	0.45820489	0.138	0.066	2.3344E-06
Kctd9	1.1002E-10	0.75897709	0.07	0.025	2.4137E-06
Ercc1	1.1554E-10	1.00376973	0.073	0.028	2.5348E-06
Abca2	1.1667E-10	0.70809249	0.084	0.033	2.5595E-06
Llgl1	1.1983E-10	0.75015702	0.063	0.022	2.629E-06
Gon7	1.2204E-10	0.58157187	0.111	0.05	2.6775E-06

Bola2	1.2482E-10	0.2597594	0.585	0.407	2.7384E-06
Yipf1	1.3121E-10	0.47901246	0.165	0.085	2.8786E-06
Emc10	1.3162E-10	0.25658734	0.272	0.16	2.8876E-06
Nlrc5	1.3339E-10	0.2567786	0.168	0.085	2.9265E-06
Sephs1	1.4721E-10	1.19886909	0.061	0.021	3.2297E-06
Katnbl1	1.4946E-10	0.41974204	0.152	0.076	3.2789E-06
Zmat5	1.5314E-10	0.71789618	0.14	0.069	3.3597E-06
Tusc2	1.5385E-10	0.74991959	0.089	0.037	3.3753E-06
Dennd4c	1.5602E-10	0.30188782	0.143	0.069	3.423E-06
Nacc1	1.5706E-10	0.55404134	0.129	0.061	3.4457E-06
Ergic3	1.5716E-10	0.42905458	0.225	0.129	3.4478E-06
Ccnk	1.5738E-10	0.3236723	0.208	0.113	3.4528E-06
Atpaf2	1.5756E-10	0.70669364	0.073	0.028	3.4568E-06
Hadha	1.5799E-10	0.26155481	0.322	0.197	3.4662E-06
Samm50	1.6136E-10	0.3718906	0.222	0.126	3.54E-06
Tada1	1.672E-10	0.50745624	0.106	0.046	3.6681E-06
Dbr1	1.695E-10	0.46010058	0.102	0.044	3.7187E-06
Mospd3	1.6995E-10	0.68259514	0.106	0.047	3.7285E-06
Faf2	1.7048E-10	0.3872427	0.168	0.087	3.7402E-06
Lcmt1	1.72E-10	0.71591138	0.095	0.04	3.7734E-06
Cwf19l1	1.7344E-10	1.13791427	0.039	0.011	3.805E-06
Timm21	1.7417E-10	0.86866995	0.066	0.024	3.8211E-06
Inip	1.7811E-10	0.41141074	0.141	0.069	3.9075E-06
Mrpl50	1.8157E-10	0.47686395	0.131	0.062	3.9835E-06
Oxsr1	1.8264E-10	0.5055244	0.127	0.06	4.0069E-06
Pglyrp2	1.834E-10	0.97736636	0.041	0.012	4.0236E-06
Sf3a2	1.8863E-10	0.32368595	0.132	0.063	4.1384E-06
Mmgt1	1.8946E-10	0.55611529	0.079	0.031	4.1565E-06
Ltb4r1	1.9713E-10	0.59609706	0.106	0.047	4.3249E-06
Ccdc32	1.9773E-10	0.77416214	0.089	0.037	4.338E-06
Tars	2.0196E-10	0.26683761	0.211	0.116	4.4309E-06
Cpox	2.0342E-10	0.47929882	0.098	0.042	4.4628E-06
Gtf3c5	2.0498E-10	0.89736489	0.064	0.023	4.497E-06
Krt18	2.0887E-10	1.66911258	0.02	0.003	4.5823E-06
Nfkbil1	2.0984E-10	0.68875489	0.086	0.035	4.6036E-06
Etfdh	2.1115E-10	0.5295797	0.114	0.052	4.6324E-06
Usp48	2.1264E-10	0.43217277	0.179	0.094	4.6651E-06
Acot8	2.1598E-10	0.83087351	0.095	0.04	4.7384E-06
Gm20939	2.1834E-10	2.18140396	0.018	0.003	4.7901E-06
Hint2	2.2333E-10	0.47598049	0.209	0.118	4.8996E-06
Ppp1r14b	2.2862E-10	0.37056215	0.283	0.171	5.0156E-06
BC018473	2.2929E-10	1.8128354	0.016	0.002	5.0303E-06
Mrpl48	2.2965E-10	0.3674173	0.215	0.121	5.0384E-06
Lyrm2	2.3145E-10	0.35905618	0.12	0.056	5.0778E-06
Nabp2	2.3217E-10	0.52493164	0.132	0.064	5.0936E-06
1700048O20R	2.3666E-10	1.74323459	0.025	0.005	5.1922E-06
Gm3550	2.3933E-10	2.74359555	0.013	0.002	5.2506E-06
Gtdc1	2.5131E-10	0.32322396	0.113	0.051	5.5136E-06

Padi2	2.5177E-10	0.55609653	0.111	0.05	5.5235E-06
Trappc8	2.5245E-10	0.27433095	0.166	0.085	5.5385E-06
Mre11a	2.5736E-10	0.6596502	0.091	0.038	5.6463E-06
Clasp1	2.5912E-10	0.42221836	0.134	0.065	5.6848E-06
Klk8	2.6107E-10	0.3080045	0.188	0.099	5.7277E-06
Fam216a	2.6187E-10	0.60469863	0.079	0.031	5.7451E-06
Smarcb1	2.6398E-10	0.55877721	0.154	0.079	5.7915E-06
Appbp2	2.6517E-10	0.25034063	0.22	0.123	5.8177E-06
Ppcs	2.7051E-10	0.88938249	0.047	0.014	5.9346E-06
Stoml2	2.8154E-10	0.707644	0.118	0.055	6.1767E-06
Mpnd	2.8209E-10	0.72463532	0.131	0.064	6.1888E-06
Clpb	2.8797E-10	0.90801064	0.059	0.02	6.3177E-06
Ppp3cc	2.9227E-10	0.37844902	0.12	0.055	6.4121E-06
Fam3c	3.0439E-10	0.33564667	0.157	0.08	6.678E-06
Hk1	3.1463E-10	0.43370663	0.145	0.072	6.9028E-06
Zfp760	3.1843E-10	1.29074278	0.034	0.009	6.9861E-06
E2f6	3.2168E-10	1.09470929	0.039	0.011	7.0574E-06
Grhpr	3.2401E-10	0.97214935	0.059	0.02	7.1085E-06
Sh3gl1	3.266E-10	0.47005375	0.111	0.05	7.1652E-06
Prdx3	3.426E-10	0.46993406	0.154	0.079	7.5162E-06
Klhl12	3.4734E-10	0.35929723	0.109	0.048	7.6202E-06
Nup214	3.4981E-10	0.3147946	0.175	0.093	7.6745E-06
Tmem154	3.5368E-10	0.56268437	0.079	0.031	7.7594E-06
Apex2	3.7958E-10	0.79422158	0.048	0.015	8.3277E-06
Ppp2cb	3.8004E-10	0.45685781	0.177	0.095	8.3376E-06
Cd200r1	3.9498E-10	0.38446194	0.104	0.045	8.6655E-06
Nubp2	3.9817E-10	0.49920445	0.132	0.065	8.7355E-06
Rnf121	3.9984E-10	0.50105946	0.106	0.047	8.7721E-06
Lzic	4.0375E-10	0.57423298	0.1	0.044	8.8578E-06
Qrsl1	4.079E-10	0.64783866	0.077	0.03	8.9489E-06
Fam118a	4.0841E-10	0.62547336	0.082	0.033	8.9601E-06
Cdc27	4.1944E-10	0.37264867	0.165	0.085	9.202E-06
Slc35c1	4.3439E-10	1.45322014	0.047	0.014	9.53E-06
Lrrc40	4.4686E-10	1.03675296	0.055	0.019	9.8036E-06
Ubp1	4.6138E-10	0.28644574	0.209	0.116	1.0122E-05
Insig2	4.6374E-10	0.39456266	0.116	0.054	1.0174E-05
Vrk3	4.715E-10	0.5221628	0.143	0.072	1.0344E-05
Trib3	4.7268E-10	1.19427673	0.047	0.014	1.037E-05
Vegfb	4.7675E-10	1.15878075	0.064	0.024	1.0459E-05
Erich1	4.8314E-10	0.63503792	0.118	0.056	1.06E-05
Lonp1	4.8578E-10	0.68618617	0.102	0.045	1.0658E-05
Nfyc	4.9197E-10	0.4383912	0.141	0.07	1.0793E-05
Mrps6	4.9247E-10	0.3532172	0.231	0.132	1.0804E-05
Triobp	5.3223E-10	0.46998268	0.113	0.052	1.1677E-05
Gabarapl1	5.4574E-10	0.37784765	0.277	0.17	1.1973E-05
Adprhl2	5.5895E-10	0.56498791	0.082	0.033	1.2263E-05
Rbm4b	5.7428E-10	0.30836152	0.154	0.078	1.2599E-05
Sdf2l1	5.9191E-10	0.3785557	0.247	0.146	1.2986E-05

Eif2a	6.0341E-10	0.25444528	0.25	0.147	1.3238E-05
Tmem109	6.0389E-10	0.52879133	0.143	0.073	1.3249E-05
Mta3	6.1078E-10	0.48817016	0.157	0.082	1.34E-05
Gstt2	6.1846E-10	0.71357739	0.093	0.04	1.3568E-05
Fcf1	6.2157E-10	0.33987409	0.208	0.117	1.3637E-05
Alg6	6.383E-10	1.2890983	0.036	0.01	1.4004E-05
Ykt6	6.4384E-10	0.35986909	0.184	0.1	1.4125E-05
Gemin6	6.4502E-10	1.22769096	0.034	0.009	1.4151E-05
Ppp6c	6.5271E-10	0.3243219	0.249	0.147	1.432E-05
Smpd1	6.5561E-10	0.91975156	0.073	0.029	1.4383E-05
Tcrg-C1	6.5567E-10	0.66376899	0.064	0.023	1.4385E-05
Elk3	6.6292E-10	0.50539734	0.165	0.087	1.4544E-05
Ncbp2	6.6361E-10	0.47389792	0.136	0.068	1.4559E-05
Zfp280b	6.8362E-10	0.62385261	0.057	0.02	1.4998E-05
Smim19	7.1095E-10	0.47615992	0.131	0.064	1.5597E-05
Qsox1	7.3637E-10	0.55303702	0.127	0.062	1.6155E-05
Ccdc71	7.3972E-10	0.56991691	0.084	0.035	1.6229E-05
Nelfa	7.4402E-10	0.51527795	0.102	0.045	1.6323E-05
Upb1	7.4546E-10	1.73060944	0.027	0.006	1.6355E-05
Cep170b	7.5216E-10	1.27831114	0.039	0.011	1.6502E-05
Avpi1	7.681E-10	0.42512004	0.233	0.137	1.6851E-05
Pfkfb1	7.6962E-10	2.7906511	0.013	0.002	1.6885E-05
Exosc2	7.9451E-10	0.35259626	0.129	0.062	1.7431E-05
Irak1bp1	8.2736E-10	1.38647545	0.03	0.007	1.8152E-05
Xpa	8.3273E-10	0.25918221	0.195	0.107	1.8269E-05
Gar1	8.3748E-10	0.37343275	0.15	0.077	1.8373E-05
Tmem29	8.4767E-10	0.6069892	0.113	0.053	1.8597E-05
Taok2	8.8199E-10	0.48008291	0.125	0.06	1.935E-05
Eef1akmt1	9.1681E-10	0.8351735	0.075	0.03	2.0114E-05
Fbxo45	9.3067E-10	0.6855493	0.073	0.029	2.0418E-05
Impad1	9.4595E-10	0.25085016	0.181	0.097	2.0753E-05
Sugp1	9.573E-10	0.41290667	0.123	0.059	2.1002E-05
Nup93	9.8167E-10	0.78336819	0.064	0.024	2.1537E-05
Wrap73	9.845E-10	0.74544753	0.057	0.02	2.1599E-05
Zdhhc23	9.8976E-10	0.59437817	0.107	0.049	2.1714E-05
Hsd17b10	9.9112E-10	0.42752413	0.195	0.11	2.1744E-05
Asb6	9.9908E-10	0.61203535	0.077	0.031	2.1919E-05
Tctex1d1	1.013E-09	1.67146154	0.021	0.004	2.2225E-05
Zfp712	1.0144E-09	1.44122907	0.023	0.005	2.2255E-05
Dpp9	1.0174E-09	0.57424625	0.104	0.047	2.2321E-05
Brms1l	1.0432E-09	0.87683086	0.052	0.017	2.2887E-05
Scnm1	1.0684E-09	0.72458814	0.075	0.03	2.3441E-05
B9d2	1.085E-09	0.41825726	0.206	0.117	2.3804E-05
Rad51b	1.1006E-09	1.43860366	0.034	0.009	2.4146E-05
Zfp580	1.1536E-09	1.46125262	0.034	0.009	2.5309E-05
Stxbp5	1.2049E-09	0.32132223	0.111	0.051	2.6434E-05
Slc16a1	1.2203E-09	0.46962837	0.111	0.051	2.6773E-05
Exosc5	1.2238E-09	0.63973345	0.163	0.088	2.6848E-05

Ei24	1.24E-09	0.51121057	0.123	0.06	2.7204E-05
Tarsl2	1.28E-09	1.21139985	0.034	0.009	2.8082E-05
Gid4	1.2892E-09	0.5878779	0.113	0.053	2.8283E-05
Camk2g	1.295E-09	0.5000458	0.097	0.043	2.841E-05
Usp5	1.3152E-09	0.59313786	0.097	0.043	2.8854E-05
Fam72a	1.3472E-09	2.32457713	0.013	0.002	2.9557E-05
Fbxw7	1.3606E-09	0.30221121	0.15	0.077	2.9851E-05
Car12	1.3659E-09	2.19044922	0.013	0.002	2.9966E-05
CAAA0114733	1.4069E-09	1.93856836	0.025	0.006	3.0867E-05
Flii	1.4168E-09	0.26820873	0.245	0.146	3.1083E-05
Mrps31	1.4564E-09	0.5521307	0.088	0.037	3.1953E-05
Mesd	1.5082E-09	0.62573752	0.113	0.053	3.3089E-05
Snapc3	1.5332E-09	0.58590922	0.095	0.042	3.3638E-05
Gm1976	1.5485E-09	2.03377793	0.016	0.003	3.3973E-05
Bclaf3	1.5783E-09	0.39772673	0.136	0.068	3.4627E-05
Rcbtb2	1.5958E-09	0.31046303	0.177	0.097	3.501E-05
Tubgcp4	1.5959E-09	0.29910057	0.129	0.063	3.5012E-05
Hus1	1.6129E-09	0.72289899	0.055	0.019	3.5385E-05
Ccdc14	1.6743E-09	1.73298918	0.018	0.003	3.6733E-05
Exosc10	1.6845E-09	0.57151948	0.138	0.07	3.6956E-05
Gin1	1.7258E-09	1.03260497	0.052	0.018	3.7862E-05
Abhd10	1.7278E-09	0.42568189	0.095	0.042	3.7906E-05
Fbxo8	1.7773E-09	0.38430663	0.122	0.058	3.8992E-05
Ctnnbl1	1.8187E-09	0.51495399	0.131	0.065	3.99E-05
Ythdf2	1.8461E-09	0.34366167	0.211	0.122	4.0501E-05
Hist4h4	1.8528E-09	0.59807345	0.052	0.017	4.0649E-05
Xrcc5	1.8567E-09	0.61748448	0.048	0.016	4.0735E-05
Ubl7	1.86E-09	0.49793619	0.131	0.065	4.0806E-05
Mfap3	1.8759E-09	0.49427507	0.098	0.044	4.1155E-05
Poc5	1.8878E-09	0.79100084	0.064	0.024	4.1416E-05
Nr1h2	1.9336E-09	0.26976963	0.216	0.124	4.2422E-05
Kif3a	1.9808E-09	1.14103561	0.05	0.017	4.3456E-05
Iars	1.9874E-09	0.32704129	0.161	0.085	4.3602E-05
Rabl6	2.0418E-09	0.36224504	0.131	0.065	4.4796E-05
Zfp260	2.0876E-09	0.29095837	0.134	0.066	4.5799E-05
Frat2	2.0996E-09	0.28432172	0.106	0.048	4.6062E-05
Iltifb	2.134E-09	2.68995189	0.016	0.003	4.6819E-05
Cbwd1	2.1907E-09	0.58000002	0.061	0.022	4.8062E-05
Gm20069	2.199E-09	0.72939646	0.063	0.023	4.8245E-05
Golga3	2.2021E-09	0.45887986	0.132	0.066	4.8311E-05
Rpusd4	2.2174E-09	0.60446144	0.095	0.042	4.8647E-05
Arhgap20	2.2495E-09	2.10015322	0.016	0.003	4.9352E-05
Zfp740	2.2654E-09	0.30080173	0.145	0.073	4.9701E-05
Ikzf4	2.2718E-09	0.83807209	0.048	0.016	4.984E-05
Riox2	2.2961E-09	0.62806655	0.1	0.046	5.0375E-05
Wrn	2.3153E-09	0.32874723	0.127	0.062	5.0796E-05
Ube4b	2.3385E-09	0.30285076	0.138	0.069	5.1304E-05
Tnpo2	2.3428E-09	0.33774033	0.111	0.052	5.1398E-05

Sh3bp5l	2.3879E-09	0.68976962	0.055	0.019	5.2389E-05
Spidr	2.3889E-09	0.73484642	0.063	0.023	5.241E-05
L1cam	2.4063E-09	0.61124858	0.066	0.025	5.2791E-05
Jpt2	2.4906E-09	1.20089944	0.061	0.023	5.4641E-05
Dhx32	2.5702E-09	0.96188943	0.048	0.016	5.6389E-05
Maea	2.5847E-09	0.26189917	0.191	0.107	5.6706E-05
Hdac6	2.6257E-09	1.15640345	0.043	0.013	5.7605E-05
3110040N11R	2.697E-09	0.88697991	0.073	0.03	5.917E-05
Ss18l2	2.7787E-09	0.35978434	0.118	0.057	6.0962E-05
Coq10a	2.7875E-09	0.79321838	0.068	0.027	6.1156E-05
Plaa	2.9116E-09	0.28046769	0.215	0.124	6.3877E-05
Pgm3	2.9471E-09	0.78199278	0.064	0.025	6.4657E-05
Magt1	3.0352E-09	0.29052545	0.283	0.179	6.6588E-05
Zfp54	3.1244E-09	1.57202006	0.02	0.004	6.8547E-05
Tor1aip2	3.17E-09	0.30166077	0.216	0.125	6.9547E-05
Rad54l2	3.2109E-09	0.45251503	0.132	0.066	7.0445E-05
Slc35a3	3.2344E-09	0.28636186	0.138	0.07	7.096E-05
Tcf3	3.2585E-09	0.42335478	0.116	0.056	7.1488E-05
Srebf1	3.3087E-09	0.33178196	0.127	0.063	7.2589E-05
Trnau1ap	3.3614E-09	0.41594153	0.157	0.084	7.3747E-05
Zpr1	3.4849E-09	0.44623621	0.141	0.073	7.6456E-05
AU041133	3.494E-09	0.85079297	0.048	0.016	7.6655E-05
Hdgfl3	3.512E-09	0.7054689	0.073	0.03	7.705E-05
Yipf3	3.5374E-09	0.29971204	0.258	0.156	7.7608E-05
Isca2	3.7042E-09	0.42760775	0.145	0.075	8.1267E-05
Aldh9a1	3.7859E-09	0.56952749	0.114	0.056	8.3059E-05
Syngr3	3.8033E-09	2.0099102	0.013	0.002	8.3441E-05
Nip7	3.8278E-09	0.31911715	0.19	0.107	8.3978E-05
Tufm	3.9091E-09	0.52883076	0.113	0.054	8.5761E-05
Cox11	3.9601E-09	0.78615295	0.075	0.031	8.688E-05
Lzts1	3.9744E-09	1.43209293	0.034	0.009	8.7193E-05
B4galnt4	3.9754E-09	0.82466459	0.041	0.013	8.7217E-05
Cox16	4.1701E-09	0.25313307	0.143	0.074	9.1487E-05
Nqo2	4.1961E-09	1.05005483	0.05	0.017	9.2058E-05
Zfp69	4.3768E-09	1.90832921	0.02	0.004	9.6022E-05
Triap1	4.386E-09	0.40845073	0.12	0.059	9.6224E-05
Selenom	4.3889E-09	0.62608578	0.122	0.061	9.6289E-05
Pex16	4.4531E-09	0.3917152	0.148	0.078	9.7696E-05
Prdm10	4.4879E-09	0.6204918	0.081	0.034	9.846E-05
Kat5	4.539E-09	0.52912687	0.114	0.056	9.9582E-05
Eif2b5	4.5802E-09	0.38456441	0.116	0.056	0.00010048
Xrcc1	4.6478E-09	0.83425995	0.064	0.025	0.00010197
Cep70	4.6552E-09	0.93588009	0.041	0.013	0.00010213
Dennd2d	4.7157E-09	0.28492262	0.098	0.044	0.00010346
Wrap53	4.7177E-09	1.17871198	0.038	0.011	0.0001035
Nudt22	4.8044E-09	1.28533864	0.039	0.012	0.0001054
Nadk2	4.8493E-09	0.58663689	0.079	0.033	0.00010639
Trmt2b	4.8794E-09	0.6700262	0.064	0.025	0.00010705

Zfp382	4.8923E-09	1.31420987	0.048	0.016	0.00010733
Pnp	4.9506E-09	0.30065955	0.229	0.137	0.00010861
Map3k3	4.9922E-09	0.44315283	0.123	0.061	0.00010952
Aldh16a1	5.1099E-09	0.83544184	0.077	0.032	0.00011211
Adarb1	5.256E-09	1.26651682	0.032	0.009	0.00011531
Pdk1	5.2732E-09	0.45749916	0.102	0.047	0.00011569
Tsr3	5.3463E-09	0.69882949	0.088	0.039	0.00011729
Dna2	5.4622E-09	1.34202309	0.052	0.018	0.00011984
Gpr34	5.4973E-09	0.7909125	0.081	0.034	0.00012061
Pitpnb	5.6132E-09	0.29097993	0.175	0.097	0.00012315
Trim27	5.6241E-09	0.30662697	0.191	0.108	0.00012339
Eif3j2	6.2974E-09	0.3646339	0.175	0.098	0.00013816
Cep63	6.3141E-09	0.31572184	0.107	0.051	0.00013852
Thap7	6.3253E-09	0.5948772	0.091	0.041	0.00013877
Nosip	6.3513E-09	0.33481837	0.114	0.055	0.00013934
Polr3a	6.3984E-09	0.56233725	0.086	0.037	0.00014037
Crls1	6.423E-09	0.75888564	0.077	0.032	0.00014091
Ift52	6.4513E-09	0.4951909	0.116	0.057	0.00014153
Stk16	6.6718E-09	0.49648693	0.122	0.061	0.00014637
Etaa1os	6.7614E-09	1.27446087	0.03	0.008	0.00014834
Ppif	6.909E-09	0.4313452	0.073	0.03	0.00015158
Tmem87a	7.1698E-09	0.27957485	0.157	0.084	0.0001573
Suclg1	7.2717E-09	0.41975309	0.172	0.096	0.00015953
Slc39a3	7.278E-09	0.63681373	0.064	0.025	0.00015967
Capn7	7.4228E-09	0.28392147	0.154	0.081	0.00016285
Odf2l	7.4778E-09	0.94895866	0.047	0.016	0.00016405
Bcat1	7.6955E-09	2.15277	0.013	0.002	0.00016883
Ccdc69	7.7295E-09	0.81299039	0.047	0.016	0.00016958
Btbd10	7.7456E-09	0.55241564	0.098	0.046	0.00016993
Mapk9	7.8244E-09	0.41316797	0.107	0.051	0.00017166
Tulp3	7.8465E-09	1.86192806	0.027	0.007	0.00017214
Usp10	7.8684E-09	0.44927088	0.127	0.064	0.00017263
Cers5	7.9179E-09	0.2697603	0.159	0.086	0.00017371
Zfp958	7.933E-09	0.59747474	0.075	0.031	0.00017404
Crocc	8.0562E-09	1.78785705	0.02	0.004	0.00017674
Ube2g1	8.0938E-09	0.25260855	0.15	0.079	0.00017757
Ngly1	8.4414E-09	0.43338856	0.113	0.055	0.0001852
Mrpl22	8.4478E-09	0.67096123	0.082	0.036	0.00018534
Plcl2	8.6257E-09	0.28002656	0.156	0.083	0.00018924
Cd320	8.6639E-09	0.80074905	0.059	0.022	0.00019008
Cep85	8.728E-09	0.67976207	0.066	0.026	0.00019148
Dgke	8.9209E-09	0.4186746	0.086	0.038	0.00019571
Nefh	8.9915E-09	1.26906349	0.021	0.005	0.00019726
Ercc3	9.2202E-09	0.2666223	0.125	0.063	0.00020228
Chek2	9.3203E-09	1.58763027	0.021	0.005	0.00020448
Zfp873	9.3422E-09	1.27325504	0.025	0.006	0.00020496
Hspb6	9.3794E-09	1.63390848	0.02	0.004	0.00020577
Tbp	9.6695E-09	0.6610914	0.081	0.035	0.00021214

Dyrk3	1.0025E-08	0.71682825	0.038	0.011	0.00021993
Scaf1	1.0162E-08	0.45464263	0.123	0.062	0.00022295
Trmt10c	1.0227E-08	0.412953	0.107	0.051	0.00022437
Tmem165	1.0383E-08	0.33556269	0.147	0.078	0.00022778
3830406C13R	1.0533E-08	0.29984651	0.161	0.087	0.00023109
Dctn6	1.0569E-08	0.55405302	0.134	0.07	0.00023187
Mydgf	1.0659E-08	0.30119046	0.259	0.162	0.00023385
Prmt5	1.0711E-08	0.45079868	0.089	0.04	0.00023499
2210011C24R	1.0967E-08	1.55069741	0.02	0.004	0.0002406
Ggt1	1.1052E-08	1.65421663	0.018	0.003	0.00024247
Rorc	1.1115E-08	0.70591621	0.043	0.014	0.00024385
Axin2	1.1118E-08	0.28585209	0.082	0.035	0.00024392
Psmd10	1.1129E-08	0.52145131	0.145	0.078	0.00024416
Ccdc138	1.1162E-08	1.42875344	0.03	0.008	0.00024488
Mlx	1.1176E-08	0.47919397	0.113	0.055	0.00024519
Recql	1.1568E-08	0.72372682	0.059	0.022	0.0002538
Lman1	1.1679E-08	0.40893895	0.184	0.106	0.00025623
Kctd18	1.1854E-08	0.27123916	0.102	0.048	0.00026006
Nrarp	1.2188E-08	1.52678291	0.021	0.005	0.00026739
Pycrl	1.2414E-08	1.2291154	0.038	0.011	0.00027235
Sertad3	1.2425E-08	0.73365029	0.061	0.023	0.00027259
Brf1	1.2647E-08	0.451234	0.104	0.049	0.00027746
Paqr4	1.2718E-08	1.71845629	0.027	0.007	0.00027903
Psmd5	1.2736E-08	0.54511447	0.079	0.034	0.00027942
AC163354.1	1.2963E-08	0.50219583	0.1	0.047	0.00028439
Spast	1.3095E-08	0.41211511	0.129	0.066	0.00028728
Vopp1	1.3311E-08	0.3809586	0.143	0.076	0.00029204
Iah1	1.3329E-08	0.73044755	0.132	0.07	0.00029243
Tor4a	1.3802E-08	1.05845711	0.032	0.009	0.00030281
Eml2	1.3899E-08	0.80929269	0.068	0.028	0.00030493
Bysl	1.4621E-08	0.80855744	0.061	0.024	0.00032078
Lancl1	1.463E-08	0.73697304	0.073	0.031	0.00032097
Abcf2	1.5478E-08	0.50472293	0.109	0.053	0.00033957
Bcl2l13	1.6105E-08	0.39518056	0.104	0.049	0.00035332
Gnl1	1.6164E-08	0.39041661	0.174	0.098	0.00035463
Asb3	1.6197E-08	0.5413483	0.068	0.027	0.00035534
Slc2a8	1.6329E-08	0.91713999	0.043	0.014	0.00035824
A630089N07R	1.6473E-08	1.56575259	0.023	0.005	0.00036141
Elf4	1.6504E-08	0.27450241	0.172	0.095	0.00036208
Serpina3g	1.6535E-08	0.40505642	0.152	0.082	0.00036276
Tubgcp3	1.7076E-08	0.34229561	0.125	0.064	0.00037463
Med18	1.7245E-08	1.3244968	0.027	0.007	0.00037833
Med12	1.7472E-08	0.31541639	0.136	0.071	0.00038332
Cdk5	1.7562E-08	0.71472461	0.084	0.038	0.00038529
Cenpt	1.7723E-08	1.30509018	0.034	0.01	0.00038882
Foxk2	1.7743E-08	0.55261706	0.082	0.036	0.00038926
Cpsf1	1.8002E-08	0.58522543	0.091	0.042	0.00039495
Ints14	1.8264E-08	0.35863444	0.081	0.035	0.00040069

Cry1	1.8569E-08	0.29407085	0.147	0.078	0.00040739
Psmg3	1.8919E-08	0.52914782	0.106	0.051	0.00041507
Dtd1	1.8941E-08	0.49279322	0.097	0.045	0.00041554
Inpp5k	1.9649E-08	0.28732598	0.12	0.06	0.00043108
Gm26765	2.0223E-08	1.53400703	0.021	0.005	0.00044368
Palb2	2.039E-08	1.35534124	0.043	0.014	0.00044733
Hook1	2.0845E-08	0.53285569	0.07	0.029	0.00045732
Ddx49	2.1351E-08	0.40988073	0.129	0.067	0.00046841
Zbtb45	2.1756E-08	1.32845632	0.038	0.012	0.00047731
Fbxw17	2.1969E-08	0.93884323	0.061	0.024	0.00048198
Ptcd3	2.218E-08	0.3516073	0.104	0.05	0.0004866
Uevld	2.2305E-08	1.27147396	0.032	0.009	0.00048935
Efl1	2.297E-08	0.56148022	0.084	0.038	0.00050395
Rhbdl3	2.3132E-08	2.27647871	0.013	0.002	0.0005075
Rnaset2b	2.323E-08	0.29171607	0.165	0.092	0.00050963
Clpp	2.3425E-08	0.58306106	0.097	0.046	0.00051393
Hs2st1	2.3476E-08	0.531093	0.123	0.063	0.00051504
Wdr70	2.377E-08	0.40933664	0.129	0.067	0.00052148
Ap3b1	2.4053E-08	0.25622639	0.242	0.15	0.0005277
Prcc	2.4165E-08	0.54158457	0.095	0.044	0.00053016
Rbm45	2.4237E-08	0.73945175	0.054	0.02	0.00053174
Prkcz	2.4247E-08	0.5952917	0.064	0.026	0.00053195
N6amt1	2.4505E-08	0.41338358	0.118	0.06	0.00053762
4933427D14R	2.4631E-08	0.98887198	0.039	0.013	0.00054037
Shkbp1	2.5036E-08	0.41934098	0.113	0.056	0.00054926
Eci2	2.5519E-08	0.54598613	0.129	0.068	0.00055987
Pms1	2.5587E-08	1.16625971	0.036	0.011	0.00056135
Chst12	2.5703E-08	0.42716633	0.093	0.043	0.00056389
Perp	2.5872E-08	1.15000059	0.032	0.009	0.0005676
Gm21988	2.6005E-08	1.53608124	0.021	0.005	0.00057052
Acsl5	2.6057E-08	0.3277802	0.179	0.103	0.00057167
Dcun1d4	2.6972E-08	0.77671657	0.057	0.022	0.00059173
Mfsd10	2.7351E-08	0.46401364	0.136	0.072	0.00060005
Htatip2	2.7543E-08	0.49856184	0.113	0.056	0.00060427
Tomm40	2.7662E-08	0.39590448	0.129	0.067	0.00060688
Zfp930	2.8289E-08	0.90448207	0.043	0.014	0.00062062
Gm27162	2.8504E-08	1.85599693	0.016	0.003	0.00062535
1110065P20R	2.8623E-08	0.58979681	0.089	0.041	0.00062795
Slc35a4	2.8842E-08	0.3621274	0.134	0.071	0.00063277
Dus1l	2.9486E-08	0.44642975	0.07	0.029	0.00064689
Gemin5	2.9682E-08	0.79008878	0.045	0.015	0.0006512
Galnt7	3.0487E-08	0.29036793	0.166	0.094	0.00066885
Taf1b	3.0533E-08	0.41998642	0.084	0.038	0.00066986
Rps6kb2	3.0697E-08	0.46527692	0.095	0.045	0.00067346
Anapc4	3.088E-08	0.52924749	0.091	0.042	0.00067748
Sac3d1	3.1295E-08	1.27179042	0.03	0.008	0.00068658
Gabbr1	3.1346E-08	0.27426109	0.122	0.061	0.00068771
Ints10	3.1819E-08	0.79184515	0.05	0.018	0.00069808

Tars2	3.2219E-08	0.58764117	0.068	0.028	0.00070685
Dap3	3.2297E-08	0.28241699	0.165	0.092	0.00070856
Zfp212	3.2578E-08	0.3469331	0.088	0.04	0.00071472
Adnp	3.2965E-08	0.50314766	0.075	0.032	0.00072321
Slc10a7	3.3222E-08	0.71015533	0.064	0.026	0.00072885
Kdelr2	3.3695E-08	0.31748748	0.209	0.126	0.00073923
Rnf214	3.4072E-08	0.63276023	0.081	0.036	0.00074751
Gm4673	3.4889E-08	0.67901733	0.05	0.018	0.00076544
Pdhx	3.5545E-08	0.64010194	0.066	0.027	0.00077983
Gnpat	3.5993E-08	0.30936629	0.084	0.038	0.00078965
Sash3	3.6039E-08	0.25031983	0.123	0.063	0.00079065
Pno1	3.6095E-08	0.35164561	0.134	0.071	0.00079188
Nemp1	3.6119E-08	0.6109787	0.055	0.021	0.00079242
Glrx3	3.6326E-08	0.25677862	0.254	0.16	0.00079695
A830005F24Ri	3.7148E-08	2.35835236	0.011	0.001	0.00081499
Ak6	3.7275E-08	0.67775126	0.095	0.045	0.00081778
B3gat2	3.77E-08	0.75129891	0.043	0.014	0.0008271
Dnajc24	3.8402E-08	0.53263613	0.086	0.039	0.00084251
Aspscr1	3.9225E-08	0.42314119	0.102	0.05	0.00086055
Zdhhc15	3.9324E-08	1.5358792	0.021	0.005	0.00086272
Xrcc6	3.977E-08	1.07940427	0.039	0.013	0.00087252
Osgepl1	3.9793E-08	1.0972396	0.029	0.008	0.00087302
Wdcp	4.0467E-08	1.1575243	0.045	0.015	0.00088781
Ppm1d	4.0885E-08	0.3619478	0.109	0.054	0.00089698
Mrpl39	4.1555E-08	0.62383096	0.106	0.053	0.00091167
Hist2h4	4.1739E-08	0.75762016	0.045	0.015	0.00091571
Gm29243	4.2647E-08	0.48312354	0.066	0.027	0.00093563
Gm19557	4.4958E-08	0.84174586	0.038	0.012	0.00098633
Pcgf2	4.6981E-08	1.23492313	0.025	0.006	0.00103073
Zfp384	4.7072E-08	0.42784383	0.102	0.05	0.00103272
Mtap	4.7331E-08	0.78688526	0.068	0.029	0.00103839
Nup54	4.7952E-08	0.25839586	0.159	0.089	0.00105203
Zfp593	4.8655E-08	0.30360068	0.184	0.108	0.00106744
Mars	4.9472E-08	0.52867549	0.084	0.038	0.00108537
Ddx43	4.9786E-08	1.77067962	0.016	0.003	0.00109226
Pear1	4.9799E-08	0.46946723	0.055	0.021	0.00109254
Lsm10	5.1437E-08	0.49418476	0.118	0.061	0.00112848
Axin1	5.1771E-08	0.33881005	0.141	0.076	0.00113581
Sacm1l	5.2732E-08	0.358175	0.132	0.07	0.00115688
Sdr39u1	5.28E-08	0.81783257	0.041	0.014	0.00115838
Nipsnap1	5.3318E-08	1.0123197	0.048	0.017	0.00116974
Fbxo3	5.3841E-08	0.2847713	0.141	0.076	0.00118122
Gm14029	5.6144E-08	0.83042501	0.045	0.016	0.00123174
Ccdc92	5.621E-08	0.91626527	0.029	0.008	0.00123319
Fam207a	5.6228E-08	0.33837539	0.136	0.073	0.00123359
Ccdc58	5.7632E-08	0.54002367	0.075	0.033	0.00126438
Sgip1	6.0869E-08	0.5493286	0.059	0.023	0.00133541
Gm36445	6.2187E-08	1.14056876	0.023	0.006	0.00136432

Unc119	6.3857E-08	0.39536764	0.141	0.077	0.00140096
Zcchc8	6.4648E-08	0.43751862	0.12	0.062	0.00141831
Camta1	6.6567E-08	0.27769195	0.143	0.078	0.0014604
Mkrn2	6.7675E-08	0.2795248	0.097	0.046	0.00148472
Rsph1	6.8966E-08	1.90324691	0.014	0.003	0.00151304
Lias	6.9293E-08	0.44663803	0.118	0.061	0.00152022
Slc38a10	7.0201E-08	0.26645255	0.166	0.094	0.00154013
Ciao1	7.0477E-08	0.46879255	0.102	0.05	0.00154619
Adap1	7.1668E-08	0.73144071	0.086	0.04	0.00157232
Pan2	7.2633E-08	0.37698786	0.079	0.035	0.0015935
Nop14	7.2989E-08	0.26621012	0.213	0.129	0.00160131
Gnl2	7.3033E-08	0.44820376	0.154	0.087	0.00160228
Tmem106c	7.6004E-08	0.99373775	0.052	0.02	0.00166745
Nol6	7.6034E-08	0.55691536	0.077	0.034	0.00166811
Vps26b	7.722E-08	0.41973371	0.091	0.043	0.00169414
Pgrmc1	7.9819E-08	0.34948871	0.154	0.086	0.00175114
Zfp180	8.1628E-08	0.37779736	0.089	0.042	0.00179083
Vti1a	8.306E-08	0.37190512	0.116	0.06	0.00182224
Tnfaip1	8.3064E-08	0.36923021	0.088	0.041	0.00182234
Sugp2	8.3928E-08	0.44594804	0.086	0.04	0.00184129
Cltb	8.6909E-08	0.36080175	0.175	0.103	0.00190669
Timmdc1	8.7547E-08	1.00245445	0.045	0.016	0.00192069
Setdb2	9.2106E-08	0.35705614	0.073	0.032	0.00202071
Pacs1	9.2413E-08	0.27047567	0.114	0.058	0.00202744
Thyn1	9.5361E-08	0.98177545	0.077	0.035	0.00209213
Fbxl8	9.6685E-08	1.2720091	0.036	0.011	0.00212117
Ddx28	9.6891E-08	0.70088208	0.063	0.026	0.00212569
Gmeb2	9.8683E-08	0.43184413	0.123	0.065	0.002165
Cep250	9.9716E-08	0.25910778	0.102	0.05	0.00218768
Psmd3	9.9803E-08	0.39504347	0.17	0.099	0.00218958
2610044O15R	1.0046E-07	0.7273904	0.039	0.013	0.00220407
Gemin8	1.0132E-07	1.37305232	0.025	0.007	0.00222294
Leng1	1.0296E-07	0.28581073	0.113	0.057	0.0022589
F8a	1.0581E-07	0.57137703	0.061	0.025	0.00232146
Ctdnep1	1.0712E-07	0.25129126	0.254	0.162	0.00235015
Polr3h	1.0778E-07	0.8542379	0.041	0.014	0.00236453
Tmem184c	1.0789E-07	0.62660467	0.052	0.02	0.002367
Tm9sf1	1.079E-07	0.6627956	0.052	0.02	0.00236715
Josd2	1.0833E-07	0.63193797	0.116	0.061	0.00237667
Il21	1.0853E-07	2.06663866	0.013	0.002	0.00238098
Eef1akmt2	1.0898E-07	0.43657695	0.07	0.03	0.00239089
Pdcd2l	1.0919E-07	0.58099882	0.1	0.05	0.00239547
Mycbp	1.1048E-07	0.44557769	0.091	0.044	0.00242392
Rit1	1.1143E-07	0.53429621	0.088	0.041	0.00244466
Wisp1	1.1263E-07	1.63229039	0.021	0.005	0.00247098
Polr3gl	1.1301E-07	0.63235868	0.072	0.031	0.00247938
Dcakd	1.1408E-07	1.1352784	0.034	0.011	0.00250291
Wdr83	1.1409E-07	0.86919242	0.057	0.023	0.00250305

Tmsb15b1	1.1435E-07	0.73158552	0.054	0.021	0.00250882
Rad17	1.1529E-07	0.38372585	0.122	0.064	0.00252933
Bcdin3d	1.1672E-07	0.76813603	0.045	0.016	0.00256079
Elovl1	1.172E-07	0.32402507	0.14	0.077	0.00257122
Rcan3	1.2469E-07	0.73531584	0.047	0.017	0.00273553
Prdm4	1.2601E-07	0.52865171	0.095	0.046	0.00276444
Grwd1	1.3178E-07	0.59344998	0.05	0.019	0.00289101
Mthfd1	1.3435E-07	0.61589966	0.057	0.023	0.00294743
5730455P16R	1.3671E-07	0.46877879	0.059	0.024	0.00299918
Cnpy2	1.3772E-07	0.6043831	0.136	0.076	0.00302136
Cox10	1.3985E-07	0.55120963	0.102	0.051	0.00306808
Nudt2	1.4386E-07	1.11634763	0.055	0.022	0.00315604
Fdps	1.4914E-07	0.66046988	0.113	0.059	0.00327187
Exoc2	1.514E-07	0.33985637	0.14	0.077	0.00332159
B3gat3	1.5289E-07	0.33099444	0.088	0.042	0.00335436
Trmt10a	1.5349E-07	0.43087807	0.07	0.03	0.00336734
Phc1	1.5368E-07	0.55113621	0.061	0.025	0.00337162
Gna11	1.5569E-07	0.72923805	0.054	0.021	0.00341562
Yars	1.5786E-07	0.25945293	0.134	0.073	0.00346337
Jagn1	1.5826E-07	0.5233308	0.081	0.037	0.00347214
Zdhhc4	1.588E-07	0.57605116	0.075	0.034	0.00348385
Tmem147	1.6113E-07	0.44827643	0.156	0.09	0.00353498
Mtmr2	1.6669E-07	0.35537883	0.111	0.057	0.00365696
Atxn3	1.6716E-07	0.49899456	0.091	0.044	0.00366732
Erc1	1.6877E-07	0.61686566	0.061	0.025	0.00370261
Gnb4	1.7319E-07	0.47540325	0.089	0.043	0.00379955
C1qtnf12	1.7361E-07	1.18115625	0.047	0.017	0.0038088
B3gnt2	1.7561E-07	0.57519608	0.052	0.02	0.00385267
Zfp770	1.7665E-07	0.79272988	0.052	0.02	0.00387546
Slc48a1	1.7927E-07	0.54303633	0.109	0.057	0.00393305
Chmp7	1.7949E-07	0.57912155	0.068	0.029	0.00393784
Nrde2	1.9788E-07	0.25040449	0.1	0.05	0.00434138
Ppp5c	1.9792E-07	0.59140624	0.088	0.042	0.00434224
Gnpda1	1.9895E-07	0.53287088	0.098	0.049	0.00436474
Tomm6	2.0086E-07	0.48522141	0.1	0.05	0.00440668
Zbtb5	2.0786E-07	0.52378593	0.064	0.027	0.0045603
Tbck	2.0801E-07	0.29252192	0.098	0.049	0.00456361
Ogfod2	2.082E-07	0.67819754	0.059	0.024	0.0045677
Tjap1	2.1495E-07	0.48330107	0.098	0.049	0.00471585
Zadh2	2.2389E-07	0.35049271	0.088	0.042	0.00491182
Uckl1os	2.3579E-07	2.18364641	0.011	0.002	0.00517291
Zdhhc7	2.3975E-07	0.45596911	0.093	0.046	0.00525981
Ermp1	2.4353E-07	0.51386802	0.088	0.042	0.0053427
Slc4a8	2.4404E-07	0.83688186	0.043	0.015	0.005354
Tmem80	2.5466E-07	0.64072164	0.068	0.03	0.00558705
Mettl3	2.5608E-07	0.35514382	0.079	0.036	0.00561819
Casp7	2.6018E-07	0.83686036	0.05	0.019	0.00570804
Med25	2.6429E-07	0.284682	0.111	0.058	0.00579831

Apopt1	2.6884E-07	0.4069482	0.148	0.085	0.00589817
Ankrd50	2.7925E-07	0.39014391	0.093	0.046	0.00612643
Irf2bp1	2.8921E-07	0.53313945	0.073	0.033	0.00634493
Cnnm3	2.9886E-07	0.51942494	0.072	0.032	0.00655678
Zfp949	2.9938E-07	0.30960497	0.061	0.025	0.00656813
Slc7a6	3.0399E-07	0.83054959	0.05	0.019	0.00666918
Adamts10	3.1343E-07	0.52217795	0.059	0.025	0.00687637
Mtr	3.1438E-07	0.34090564	0.098	0.049	0.00689723
Zdhhc16	3.228E-07	0.71688747	0.048	0.018	0.00708194
Gm45509	3.3152E-07	0.99941184	0.027	0.008	0.00727332
Fibp	3.3654E-07	0.40369661	0.111	0.059	0.00738326
Zfp574	3.3777E-07	0.64882836	0.066	0.029	0.00741044
Endod1	3.4325E-07	0.62341856	0.1	0.051	0.00753053
Sco1	3.4703E-07	0.8796779	0.05	0.02	0.00761342
Kctd5	3.4805E-07	0.55931017	0.086	0.042	0.00763594
1300002E11R	3.5083E-07	0.29055979	0.082	0.039	0.00769684
Dhrs7b	3.5699E-07	0.66775524	0.066	0.029	0.00783193
Cactin	3.5882E-07	0.54081655	0.073	0.033	0.00787206
Bud13	3.5984E-07	0.35608269	0.082	0.039	0.00789451
Mettl2	3.6137E-07	0.59432841	0.057	0.024	0.00792802
Tamm41	3.6196E-07	1.22251054	0.029	0.008	0.00794108
Ppp2r1b	3.6475E-07	0.48307877	0.07	0.031	0.00800231
Coq6	3.7114E-07	0.98341998	0.039	0.014	0.00814249
D730045B01R	3.7724E-07	1.51425347	0.018	0.004	0.00827638
Cep164	3.773E-07	0.36305822	0.098	0.05	0.00827755
Ppid	3.7761E-07	0.27170324	0.123	0.067	0.00828441
Gm45871	3.8003E-07	0.96152218	0.025	0.007	0.0083374
Sirt6	3.8262E-07	1.04342237	0.034	0.011	0.00839426
Zfp825	3.9107E-07	1.25490986	0.034	0.011	0.00857967
Dusp14	3.9148E-07	1.16059722	0.039	0.014	0.00858877
Men1	3.9193E-07	0.88081875	0.038	0.013	0.0085986
Fbxl6	3.9246E-07	0.53417716	0.091	0.045	0.0086101
Fut11	3.9684E-07	0.63454626	0.055	0.023	0.00870628
Ppp2r3c	4.1035E-07	0.52408639	0.114	0.062	0.00900276
Gm27010	4.1583E-07	1.14940435	0.032	0.01	0.00912292
Gm527	4.1668E-07	1.08284096	0.036	0.012	0.0091416
Hist2h2ab	4.2161E-07	1.57619957	0.02	0.005	0.00924966
A430090L17Ri	4.3604E-07	2.0871483	0.011	0.002	0.00956629
Acaa2	4.3716E-07	0.48668179	0.093	0.047	0.0095909
Urod	4.3727E-07	0.61215441	0.07	0.032	0.00959326
Snx25	4.4431E-07	0.61051408	0.054	0.022	0.00974777
Ccno	4.4963E-07	0.86684742	0.027	0.008	0.0098645
2210016F16Ri	4.5174E-07	0.30292968	0.118	0.063	0.00991063
Mrpl16	4.5655E-07	0.36216388	0.123	0.067	0.01001614
Pdlim2	4.6315E-07	0.31843412	0.089	0.044	0.0101611
Zfp553	4.7401E-07	0.48599627	0.057	0.024	0.01039929
Dpy19l3	4.7939E-07	0.31213457	0.075	0.035	0.01051739
Npepl1	5.0174E-07	0.51335854	0.12	0.066	0.01100757

Ranbp6	5.0326E-07	0.56388795	0.063	0.027	0.01104095
Ormdl2	5.056E-07	0.49219225	0.15	0.088	0.01109244
Tprn	5.0923E-07	1.14086523	0.036	0.012	0.0111721
Rrs1	5.2169E-07	0.35978968	0.088	0.043	0.01144525
Ccdc43	5.3061E-07	0.37366091	0.1	0.051	0.01164111
Tnnt1	5.3085E-07	1.79543982	0.018	0.004	0.01164643
Kbtbd3	5.3369E-07	1.11844703	0.034	0.011	0.0117087
Nlrx1	5.4064E-07	1.89994481	0.023	0.006	0.01186112
Uba3	5.5917E-07	0.34525417	0.109	0.057	0.01226763
Apoa2	5.5993E-07	1.63372518	0.016	0.003	0.01228431
Wdr78	5.612E-07	1.31976754	0.02	0.005	0.01231207
Shc1	5.6619E-07	0.38938997	0.127	0.071	0.01242155
Ciz1	5.6929E-07	0.34358447	0.095	0.048	0.01248965
Csrp2	5.7441E-07	0.76768217	0.066	0.03	0.01260205
Tkfc	5.7499E-07	1.39353851	0.027	0.008	0.01261463
Wasl	5.7776E-07	0.26515538	0.157	0.091	0.01267537
Dars	5.7914E-07	0.26650663	0.148	0.086	0.01270577
Nipsnap3b	5.995E-07	0.28376536	0.161	0.095	0.01315239
Ints5.1	6.2013E-07	0.66886885	0.048	0.019	0.01360494
Ctu2	6.2324E-07	0.41895744	0.086	0.042	0.01367316
Usp20	6.2439E-07	0.92456336	0.036	0.012	0.01369847
Dhx29	6.2927E-07	0.44307446	0.072	0.033	0.01380558
Brpf3	6.2983E-07	0.60937675	0.052	0.021	0.01381788
Trbv13-3	6.3675E-07	2.34453111	0.011	0.002	0.01396974
Zfp442	6.4387E-07	1.89568926	0.011	0.002	0.01412593
Sntb2	6.5072E-07	0.36068356	0.095	0.048	0.01427615
Srprb	6.5341E-07	0.30789719	0.111	0.059	0.01433526
Ino80b	6.6186E-07	0.36651273	0.134	0.076	0.01452044
Myl4	6.8704E-07	0.33054865	0.045	0.017	0.01507289
Pmpca	7.388E-07	0.30535548	0.123	0.068	0.01620854
Rbm41	7.5104E-07	0.70765411	0.054	0.022	0.01647712
Zfp52	7.6281E-07	0.48355586	0.077	0.036	0.01673533
Tmem110	7.6572E-07	0.83340108	0.057	0.024	0.01679918
Zfp426	7.7369E-07	0.59451526	0.047	0.018	0.01697407
Pitpnm2	7.7585E-07	0.49818285	0.05	0.02	0.01702143
Wdr45	7.7689E-07	0.35646778	0.084	0.041	0.01704422
Cep95	7.8106E-07	0.92273365	0.03	0.01	0.01713567
Gdap2	7.8304E-07	0.41742111	0.089	0.045	0.01717904
D230025D16F	7.9445E-07	0.51112041	0.07	0.032	0.01742952
Gm27166	7.9566E-07	1.07567845	0.021	0.006	0.01745598
Tmem203	7.9665E-07	0.4824283	0.106	0.056	0.01747762
Cog2	7.9898E-07	0.30670479	0.131	0.073	0.01752893
Platr25	8.0254E-07	0.6961197	0.032	0.01	0.01760699
Zfx	8.1776E-07	0.25049958	0.188	0.116	0.01794086
Sfxn1	8.2235E-07	0.27969195	0.113	0.06	0.01804145
Ttc21b	8.4235E-07	1.0276781	0.025	0.007	0.01848025
Zfp329	8.4559E-07	0.27605767	0.1	0.051	0.01855136
Sun1	8.4763E-07	0.42067474	0.082	0.04	0.01859612

Ccdc167	8.5217E-07	0.83322557	0.068	0.031	0.01869586
Pex11b	8.6223E-07	0.79700281	0.043	0.016	0.0189164
Hira	8.6688E-07	0.59611966	0.068	0.031	0.01901843
Zfp955a	8.6974E-07	1.89791196	0.02	0.005	0.01908122
Phpt1	8.7499E-07	0.46191769	0.073	0.034	0.0191964
Lyrm4	8.7702E-07	0.80128147	0.05	0.02	0.01924088
Runx2os1	9.1412E-07	0.7623591	0.047	0.018	0.02005482
Rexo4	9.3356E-07	0.27496688	0.148	0.087	0.02048138
Ddah2	9.4262E-07	1.58075774	0.023	0.006	0.02068023
Clasrp	9.4386E-07	0.62055026	0.055	0.023	0.02070725
Mllt11	9.5892E-07	0.85286638	0.036	0.012	0.02103769
Lcmt2	9.8159E-07	0.78098762	0.039	0.014	0.02153509
Capn10	9.9445E-07	0.87925312	0.038	0.013	0.0218172
Tbcd	1.0019E-06	0.76746804	0.068	0.031	0.02198168
Fbxo21	1.0281E-06	0.43430613	0.073	0.034	0.02255582
Pigu	1.0729E-06	0.4866939	0.066	0.03	0.02353852
Sdhaf4	1.1044E-06	0.53053959	0.089	0.045	0.02422969
Celsr2	1.1111E-06	2.32361322	0.011	0.002	0.02437625
Atg13	1.1163E-06	0.36794937	0.106	0.056	0.0244902
Gtf3c3	1.122E-06	0.25291773	0.079	0.038	0.02461627
Ttll5	1.1282E-06	0.66400194	0.045	0.017	0.02475064
Mrpl1	1.1401E-06	0.35948053	0.086	0.043	0.02501363
Gmeb1	1.1716E-06	0.49039854	0.089	0.045	0.02570311
C2cd2l	1.1798E-06	0.85215626	0.045	0.017	0.0258839
Oxsm	1.1976E-06	0.8544641	0.034	0.012	0.02627463
Arrb1	1.2413E-06	0.30340062	0.141	0.082	0.02723253
Ate1	1.2471E-06	0.27544515	0.143	0.083	0.02736081
Slc45a4	1.2557E-06	0.29363095	0.075	0.036	0.02754898
Slc52a2	1.2692E-06	0.68799583	0.052	0.021	0.02784508
Gm37387	1.2848E-06	0.91514036	0.027	0.008	0.02818718
Nme7	1.301E-06	0.87931704	0.038	0.013	0.02854194
A830010M20F	1.3016E-06	0.824469	0.023	0.006	0.02855616
Uxt	1.3212E-06	0.38976641	0.118	0.065	0.02898504
Ap2a1	1.325E-06	0.51104287	0.088	0.044	0.02906999
Rab11fip4	1.3312E-06	0.71102338	0.052	0.022	0.02920437
Mrpl47	1.3529E-06	0.77535811	0.055	0.024	0.02968169
Msantd2	1.3652E-06	0.32991564	0.077	0.037	0.029951
Map3k10	1.4483E-06	0.87772705	0.036	0.013	0.03177476
Abca7	1.4534E-06	0.26464816	0.113	0.061	0.0318861
2700049A03R	1.4547E-06	0.58415482	0.032	0.011	0.0319141
2310009B15R	1.4586E-06	0.55633568	0.111	0.061	0.0320012
Rab3ip	1.4646E-06	0.35314711	0.084	0.042	0.03213261
Sap18b	1.553E-06	1.00707632	0.036	0.013	0.03407039
Gm35082	1.5552E-06	0.50003809	0.088	0.044	0.03412039
Dhcr7	1.5829E-06	0.74108989	0.043	0.016	0.03472668
Cln6	1.5932E-06	0.64972092	0.07	0.033	0.03495388
Glb1	1.6054E-06	0.74246058	0.063	0.028	0.03522011
Nudt14	1.6298E-06	0.73097716	0.081	0.04	0.03575568

Zfp386	1.6603E-06	0.48843362	0.079	0.039	0.03642621
Nomo1	1.7106E-06	0.50841696	0.07	0.033	0.0375278
Nudt9	1.7273E-06	0.52649423	0.123	0.071	0.03789568
Capn15	1.7336E-06	0.39047876	0.084	0.042	0.03803286
Xpc	1.7353E-06	0.70676154	0.05	0.021	0.03807109
Rbks	1.7532E-06	1.0606744	0.034	0.012	0.03846439
Mylpf	1.7553E-06	1.91869341	0.021	0.006	0.03850896
Itfg1	1.789E-06	0.31827339	0.1	0.053	0.03924863
Dexi	1.8131E-06	0.6261244	0.066	0.03	0.03977707
Ccdc84	1.8271E-06	0.85391715	0.05	0.021	0.04008389
Agap3	1.8305E-06	0.4928952	0.081	0.04	0.04015974
Agap2	1.875E-06	0.37660813	0.048	0.019	0.04113581
Serhl	1.88E-06	0.62365048	0.066	0.031	0.04124639
Rassf7	1.8913E-06	1.2160604	0.021	0.006	0.04149276
Atg14	1.9422E-06	0.31980508	0.111	0.06	0.04260908
Alkbh7	1.9431E-06	0.60831867	0.081	0.04	0.04262994
Tfpt	1.9472E-06	0.52418915	0.064	0.029	0.04272033
Tmem62	1.9807E-06	0.76079793	0.038	0.014	0.04345494
Dusp12	1.9961E-06	0.67372217	0.063	0.028	0.04379179
Atg4b	2.0384E-06	0.28499502	0.102	0.054	0.04472029
Lrrc28	2.0522E-06	0.72601638	0.045	0.018	0.04502359
Ecd	2.0785E-06	0.27693952	0.113	0.062	0.04559948
Dgcr2	2.1133E-06	0.2743942	0.104	0.055	0.04636397
Trove2	2.1244E-06	0.4785684	0.043	0.017	0.04660718
Exosc7	2.161E-06	0.50596104	0.081	0.04	0.04741038
Lsm11	2.1679E-06	0.77402903	0.052	0.022	0.04756224
Hspb11	2.177E-06	0.35762492	0.136	0.079	0.04776156
Bod1	2.2119E-06	0.46983227	0.066	0.031	0.04852792
Ofd1	2.2324E-06	0.41596966	0.066	0.031	0.04897768
Ppt2	2.2785E-06	0.37645338	0.107	0.058	0.04998788
Prrg1	2.3178E-06	1.30291448	0.016	0.004	0.05084983
Spryd3	2.3229E-06	0.58722934	0.063	0.029	0.05096238
Manbal	2.3259E-06	0.5107516	0.123	0.071	0.051027
3110002H16R	2.3358E-06	0.68574984	0.064	0.03	0.0512459
Mthfsd	2.3978E-06	0.72637476	0.041	0.016	0.05260439
Ndufaf7	2.4178E-06	0.35982716	0.07	0.033	0.05304368
Clpx	2.4531E-06	0.33937488	0.109	0.06	0.05381763
Klhl11	2.4603E-06	0.64506041	0.041	0.016	0.05397548
Cby1	2.4744E-06	1.20971651	0.025	0.008	0.05428572
Lrig2	2.5418E-06	0.40503273	0.066	0.031	0.05576355
9130221H12R	2.6368E-06	0.90574672	0.023	0.007	0.05784931
Utf1	2.665E-06	0.72595898	0.038	0.014	0.05846739
Sec22a	2.7631E-06	0.6186679	0.059	0.026	0.0606194
Arnt2	2.8323E-06	0.6806327	0.025	0.008	0.06213773
Kcnmb4os2	2.8448E-06	0.64490176	0.041	0.016	0.06241118
Sh3rf1	2.8475E-06	0.72943918	0.038	0.014	0.06247143
Spock2	2.8752E-06	0.32107266	0.043	0.017	0.06307821
Gm32036	2.9015E-06	0.73565907	0.041	0.016	0.0636564

Atg4d	2.9018E-06	0.46140678	0.088	0.045	0.06366196
Ufsp2	2.9298E-06	0.38993096	0.136	0.08	0.06427767
Tada3	2.9738E-06	0.31096969	0.072	0.034	0.0652423
Rom1	2.9786E-06	1.65339987	0.021	0.006	0.06534828
Isoc2b	3.0215E-06	1.0687865	0.038	0.014	0.06628804
Ccdc15	3.0463E-06	1.11176289	0.023	0.007	0.0668321
Ncdn	3.0482E-06	0.33783288	0.059	0.026	0.06687358
Tsta3	3.0575E-06	0.49794646	0.093	0.049	0.06707903
Gpn2	3.0976E-06	0.76727922	0.047	0.019	0.0679593
Pdcl	3.1371E-06	0.27761515	0.122	0.069	0.06882442
Pdss1	3.1396E-06	1.17771451	0.023	0.007	0.06887877
Mtg2	3.1842E-06	0.66952334	0.045	0.018	0.06985907
Zfp7	3.2201E-06	1.10775762	0.023	0.007	0.07064678
Cic	3.256E-06	0.31795457	0.163	0.1	0.07143251
Ppp1r14c	3.347E-06	0.50472528	0.029	0.009	0.07343021
Hadh	3.3543E-06	0.34586773	0.107	0.059	0.07358893
Zfp84	3.4234E-06	0.47727633	0.095	0.05	0.07510559
Trap1	3.4575E-06	0.48788895	0.082	0.042	0.07585396
AW146154	3.4607E-06	0.99735363	0.021	0.006	0.0759235
Aifm1	3.5318E-06	0.62353288	0.075	0.037	0.07748435
Tmem70	3.5326E-06	0.34795645	0.089	0.047	0.07750178
Klc1	3.6299E-06	0.28498587	0.116	0.065	0.07963667
Chchd4	3.7515E-06	0.35763204	0.086	0.044	0.08230398
Cbx6	3.7713E-06	0.3568349	0.045	0.018	0.08273748
Zfp983	3.9256E-06	1.62970909	0.013	0.003	0.08612474
Elp6	3.9827E-06	0.88636863	0.025	0.008	0.08737728
Ankrd52	4.017E-06	0.36855548	0.066	0.031	0.08812818
AC147041.1	4.0282E-06	0.94754017	0.023	0.007	0.08837377
Isyna1	4.0318E-06	0.3683457	0.12	0.069	0.0884536
Hcfc2	4.0643E-06	0.25568942	0.066	0.031	0.08916586
Fchsd1	4.0658E-06	0.81356302	0.032	0.011	0.08920066
Zfp846	4.1159E-06	0.89301325	0.036	0.013	0.09029828
Socs7	4.1168E-06	0.38551856	0.091	0.048	0.09031937
Zfp236	4.2179E-06	0.39189937	0.093	0.049	0.09253588
Abraxas1	4.3635E-06	0.7722656	0.03	0.01	0.09573064
Thap1	4.4412E-06	0.74014126	0.061	0.028	0.09743476
Ica1	4.4573E-06	1.53217599	0.013	0.003	0.09778793
Tmem94	4.5605E-06	0.57746825	0.048	0.02	0.10005268
Cutc	4.5616E-06	0.72474749	0.047	0.019	0.100077
Chd3os	4.5796E-06	0.8831012	0.025	0.008	0.10047148
Tmem126b	4.631E-06	0.5132602	0.055	0.025	0.1016001
Nf2	4.7747E-06	0.45989387	0.073	0.036	0.10475105
Pitrm1	4.8099E-06	0.44184674	0.072	0.035	0.10552521
Gm16062	4.8916E-06	0.59218186	0.061	0.028	0.10731615
Ksr1	5.0048E-06	0.65575061	0.052	0.022	0.10980021
Ado	5.0799E-06	0.52705765	0.066	0.032	0.1114479
Slc35b1	5.1408E-06	0.26860444	0.197	0.128	0.11278421
St6galnac6	5.1701E-06	0.48456794	0.072	0.035	0.11342643

Pnkp	5.185E-06	0.51914092	0.086	0.045	0.11375447
Mrpl10	5.2761E-06	0.34652035	0.086	0.045	0.11575308
Dusp19	5.2793E-06	0.64349342	0.045	0.018	0.11582324
Pard6g	5.289E-06	0.77969535	0.023	0.007	0.11603519
Tmem129	5.416E-06	0.69834271	0.027	0.009	0.1188216
Rtl8b	5.4447E-06	0.94190553	0.048	0.021	0.11945085
Egfl7	5.5287E-06	1.7976992	0.011	0.002	0.12129475
Gbp6	5.531E-06	0.8089397	0.038	0.014	0.12134495
Nvl	5.5718E-06	0.32745267	0.118	0.068	0.12224068
Rgs14	5.6554E-06	0.30052107	0.059	0.027	0.12407461
Cc2d1a	5.6624E-06	0.62565931	0.039	0.015	0.12422669
Mogs	5.6723E-06	0.4377768	0.079	0.04	0.12444399
Polr1e	5.7333E-06	0.84074151	0.032	0.011	0.12578306
Gm47469	5.7729E-06	1.30538629	0.018	0.005	0.12665197
Xlr4c	5.8577E-06	0.69599912	0.05	0.021	0.12851316
Hsbp1l1	5.8719E-06	0.85457016	0.023	0.007	0.12882461
Supt3	6.0284E-06	0.74935684	0.055	0.025	0.13225796
Slc39a9	6.1194E-06	0.42334884	0.07	0.034	0.13425261
Cep19	6.1355E-06	0.81636262	0.027	0.009	0.13460757
Pex10	6.1857E-06	0.97841679	0.021	0.006	0.13570753
2810455O05R	6.2202E-06	1.1654916	0.016	0.004	0.13646425
Yod1	6.315E-06	0.42508842	0.082	0.042	0.138545
Trub1	6.3479E-06	0.57647682	0.032	0.011	0.13926557
Ntmt1	6.4627E-06	0.81816785	0.05	0.022	0.14178564
Mief1	6.5768E-06	0.43317856	0.081	0.041	0.14428895
Leprot	6.5829E-06	0.27763284	0.152	0.094	0.14442186
Bop1	6.6751E-06	0.26580439	0.097	0.052	0.14644611
Trav16n	6.7435E-06	1.18080021	0.016	0.004	0.14794528
Nphp4	7.0446E-06	1.76751943	0.013	0.003	0.15455156
Fbxo25	7.4903E-06	0.68924895	0.038	0.014	0.16433
Ap1g2	7.8821E-06	0.57330191	0.072	0.036	0.17292532
Ubiad1	8.049E-06	0.78934156	0.029	0.01	0.17658805
Tbc1d9b	8.0576E-06	0.30658339	0.089	0.047	0.17677543
Tmx2	8.1939E-06	0.30777274	0.063	0.03	0.1797651
Pef1	8.3221E-06	0.45052662	0.079	0.04	0.18257867
Hars2	8.3298E-06	0.45417812	0.055	0.025	0.18274818
Nkiras1	8.4783E-06	0.33615905	0.054	0.024	0.18600574
Wdr36	8.5742E-06	0.44673667	0.093	0.05	0.18811024
Ptdss2	8.8252E-06	0.97946032	0.036	0.014	0.1936167
Gpr89	8.8766E-06	0.535614	0.066	0.032	0.19474481
Eps15l1	8.8776E-06	0.28894763	0.118	0.068	0.19476634
Mrps18a	8.881E-06	0.33308466	0.175	0.113	0.19483927
Nsun2	8.9788E-06	0.35323695	0.143	0.088	0.19698655
Taf9b	9.034E-06	0.68239483	0.041	0.017	0.19819645
Aptx	9.0665E-06	0.51884501	0.066	0.032	0.1989093
Ephb6	9.0837E-06	1.37979726	0.013	0.003	0.19928723
AI413582	9.3371E-06	0.2735131	0.134	0.081	0.20484694
Cdc16	9.392E-06	0.36564233	0.072	0.036	0.20605187

Mrnip	9.5832E-06	1.48150447	0.014	0.003	0.21024524
Wdr47	9.5952E-06	0.42178545	0.048	0.021	0.21050837
Thoc1	9.9152E-06	0.33313753	0.079	0.04	0.21752989
Atm	9.9906E-06	0.39732944	0.081	0.042	0.21918396
Uba5	1.0088E-05	0.26561968	0.154	0.096	0.221314
Thop1	1.0361E-05	0.93957568	0.027	0.009	0.22729978
Fnta	1.0385E-05	0.31216297	0.089	0.048	0.22783945
Supt7l	1.0665E-05	0.37716469	0.061	0.029	0.23398601
Tigar	1.0682E-05	0.52088105	0.043	0.018	0.23435027
Galk2	1.0901E-05	0.58369555	0.072	0.036	0.23914761
Smim12	1.0916E-05	0.26635093	0.098	0.054	0.23948606
Cops7b	1.1094E-05	0.58001958	0.047	0.02	0.24338792
Zfp300	1.1192E-05	1.57079909	0.013	0.003	0.24553081
Edaradd	1.1397E-05	0.84506835	0.025	0.008	0.25003591
Ldah	1.1403E-05	0.27742983	0.088	0.047	0.25017611
Fam136a	1.1417E-05	0.28625528	0.07	0.035	0.25048723
Tfg	1.1568E-05	0.31544634	0.122	0.071	0.25379342
Tor2a	1.1739E-05	0.32483234	0.089	0.048	0.25755235
Gprin3	1.1944E-05	0.45264046	0.057	0.026	0.26203985
Cad	1.2125E-05	0.66225484	0.05	0.022	0.2660079
Exo5	1.2258E-05	1.07711472	0.029	0.01	0.26892607
Blvra	1.235E-05	0.45786946	0.1	0.057	0.27093881
Pbx4	1.2494E-05	0.99726214	0.021	0.006	0.27411098
Styx	1.2533E-05	0.25785736	0.061	0.029	0.27495145
Fbxl15	1.2588E-05	0.62697309	0.05	0.022	0.27617365
Tmem126a	1.2708E-05	0.32114663	0.138	0.084	0.27880193
Gm20139	1.2876E-05	1.04907956	0.018	0.005	0.28248367
Vkorc1	1.2902E-05	0.5158676	0.116	0.069	0.28305903
Ethe1	1.2946E-05	0.40119358	0.107	0.062	0.28401613
Acd	1.319E-05	0.25885037	0.106	0.06	0.28936699
Tbl3	1.3382E-05	0.66089149	0.054	0.025	0.29359009
Klc3	1.3523E-05	1.01451359	0.02	0.006	0.29668514
Nbeal2	1.3651E-05	0.41452376	0.059	0.028	0.29948517
Bace1	1.37E-05	1.29624511	0.014	0.003	0.30056721
Sorbs1	1.3873E-05	0.29244865	0.045	0.019	0.30435344
Acyp1	1.4332E-05	0.32839012	0.082	0.043	0.31443811
Slc25a16	1.4391E-05	0.29976254	0.061	0.029	0.31572107
Tradd	1.4581E-05	0.41780582	0.063	0.03	0.31990305
Ppip5k1	1.4626E-05	0.74157602	0.036	0.014	0.32087949
Zfp763	1.5216E-05	1.66511013	0.013	0.003	0.33383126
Ptdss1	1.5229E-05	0.32374647	0.097	0.054	0.33410833
AI847159	1.5246E-05	1.70512058	0.013	0.003	0.33447268
Dis3l2	1.5299E-05	0.31654379	0.081	0.042	0.335645
Mettl15	1.5679E-05	1.26788789	0.016	0.004	0.34397729
Rapsn	1.589E-05	0.91762148	0.036	0.014	0.34861854
Fancg	1.6121E-05	1.31478581	0.021	0.007	0.3536895
Lztr1	1.6157E-05	0.50685833	0.05	0.022	0.35446265
Tfap4	1.6235E-05	1.25382858	0.018	0.005	0.35618804

Syne3	1.6466E-05	0.56185987	0.055	0.026	0.36124099
Pygb	1.6645E-05	0.35983278	0.068	0.034	0.3651699
Inpp5e	1.7164E-05	0.60387694	0.038	0.015	0.37656056
Serp2	1.7212E-05	1.0371569	0.023	0.007	0.37760857
Rpf2	1.7383E-05	0.32546924	0.088	0.048	0.38136899
Atp8a2	1.774E-05	0.89893025	0.034	0.013	0.38920111
Dnajc17	1.8122E-05	0.62538202	0.041	0.017	0.39757572
Tgds	1.8321E-05	0.65032232	0.032	0.012	0.40195003
Snx11	1.8457E-05	0.31727965	0.061	0.029	0.40491962
Gfod2	1.8466E-05	2.08470238	0.011	0.002	0.40511611
Igf2bp3	1.8523E-05	0.72439685	0.038	0.015	0.40637089
A530017D24R	1.8791E-05	0.8313629	0.027	0.009	0.41224644
Heatr5b	1.9122E-05	0.3824108	0.055	0.026	0.41950815
Gm1673	1.9138E-05	1.16523399	0.021	0.007	0.419863
Cyb5d1	1.9564E-05	1.15318991	0.018	0.005	0.42922128
Sp3os	1.9576E-05	0.37393458	0.075	0.039	0.42947795
Ubtd2	2.0266E-05	0.54074901	0.038	0.015	0.44461097
Cep104	2.0338E-05	0.40261172	0.047	0.02	0.44619415
Micu1	2.0804E-05	0.25448961	0.093	0.052	0.45641162
Chn2	2.0804E-05	0.33322768	0.077	0.04	0.45642408
Sgcb	2.0863E-05	0.77046288	0.047	0.02	0.45770595
Zfp748	2.1154E-05	0.29012846	0.054	0.025	0.4641074
Sec23a	2.118E-05	0.45460476	0.064	0.032	0.46467894
Emc9	2.1217E-05	0.81386711	0.038	0.015	0.46546993
Trim36	2.1316E-05	0.58682364	0.063	0.031	0.4676576
Sumf2	2.133E-05	1.39407393	0.021	0.007	0.46796023
Gm30025	2.1613E-05	1.07432203	0.032	0.012	0.47417228
Tmem18	2.1681E-05	0.70672633	0.036	0.014	0.47566451
Rpusd3	2.1725E-05	0.90503537	0.02	0.006	0.47661413
Abcb4	2.2467E-05	0.84708582	0.021	0.007	0.49289605
Tpmt	2.2945E-05	2.13548216	0.016	0.004	0.50338094
Coil	2.3166E-05	0.42688508	0.043	0.018	0.50823148
Meis3	2.3344E-05	0.72813247	0.034	0.013	0.51213494
Mr1	2.3526E-05	0.96798572	0.023	0.008	0.51614613
Rttn	2.4091E-05	0.81089175	0.029	0.01	0.52853298
2810006K23R	2.4123E-05	0.72452345	0.027	0.009	0.52922616
Trp53rkb	2.4281E-05	0.38835892	0.045	0.019	0.53269762
Pdrg1	2.4302E-05	0.32187629	0.113	0.066	0.53316115
Zc3h8	2.445E-05	0.5481495	0.029	0.01	0.53639815
Cdk5rap3	2.4462E-05	0.25390251	0.098	0.056	0.53667931
Zfp282	2.5095E-05	0.4964941	0.041	0.017	0.55056261
Aarsd1	2.5117E-05	1.34261682	0.014	0.004	0.55104967
Cyb561d2	2.5535E-05	0.49911599	0.072	0.037	0.56020977
Apmap	2.6256E-05	0.28604826	0.073	0.038	0.57602694
Apool	2.6447E-05	0.43883605	0.066	0.033	0.58022663
Mrpl37	2.6714E-05	0.60131487	0.075	0.04	0.58608137
Dph7	2.6846E-05	0.49122248	0.032	0.012	0.5889797
Ppox	2.7042E-05	0.78164861	0.045	0.02	0.59328018

Pdss2	2.74E-05	0.71277214	0.03	0.011	0.6011297
Ddx56	2.7684E-05	0.43939738	0.072	0.037	0.607359
Ankrd6	2.8515E-05	1.16079615	0.016	0.004	0.6255924
Pnpt1	3.0069E-05	0.39967141	0.064	0.032	0.65968956
Fam210a	3.1119E-05	0.3019835	0.063	0.031	0.68271541
Gm6225	3.1554E-05	1.46359303	0.014	0.004	0.69226525
Deaf1	3.2027E-05	0.53933018	0.036	0.014	0.70263949
Tax1bp3	3.2642E-05	0.55298554	0.07	0.036	0.71612376
Plpp5	3.2874E-05	0.53002792	0.066	0.034	0.72121427
AI837181	3.3646E-05	0.27801063	0.12	0.072	0.73817054
Cuedc2	3.4532E-05	0.30748157	0.148	0.095	0.7576068
Helq	3.467E-05	0.5498421	0.027	0.009	0.76062809
Ankrd46	3.5294E-05	0.2953003	0.043	0.019	0.77432033
Elp2	3.5477E-05	0.40131631	0.066	0.034	0.77832472
Mad1l1	3.6217E-05	0.73955911	0.043	0.019	0.79455596
Smug1	3.6556E-05	0.62909466	0.043	0.019	0.80200579
Cant1	3.6733E-05	0.35200871	0.086	0.047	0.80588553
Ddx19b	3.6772E-05	0.39475022	0.068	0.035	0.80674649
Thg1l	3.7365E-05	0.90342166	0.045	0.02	0.8197443
Echdc2	3.8449E-05	1.29939078	0.011	0.002	0.84354113
Sharpin	3.8872E-05	0.29732643	0.122	0.074	0.85280592
Sec22c	3.9112E-05	0.45469283	0.027	0.01	0.85807685
Trmt11	3.9958E-05	0.50359644	0.057	0.028	0.87664351
Snx12	4.0644E-05	0.27555267	0.106	0.062	0.89168337
Gstz1	4.1085E-05	0.93706003	0.027	0.01	0.90136494
Actr6	4.1388E-05	0.44525758	0.047	0.021	0.9080056
Zscan21	4.1721E-05	0.49410493	0.045	0.02	0.91531919
Usp21	4.1861E-05	0.38864579	0.045	0.02	0.91838523
Mtmr7	4.2366E-05	1.75725053	0.014	0.004	0.9294743
Prpf31	4.3006E-05	0.32928458	0.097	0.056	0.94350294
Slc25a46	4.389E-05	0.26864408	0.091	0.051	0.96289258
Acat2	4.4244E-05	0.46042976	0.043	0.019	0.97067241
Trbv15	4.431E-05	1.55269299	0.013	0.003	0.97212706
Cpped1	4.4602E-05	0.66965831	0.038	0.016	0.97852936
Nsun5	4.4771E-05	0.60238763	0.038	0.016	0.98223969
Ankrd39	4.4909E-05	0.45434009	0.052	0.024	0.98526689
Rrnad1	4.5654E-05	0.27850686	0.07	0.036	1
Wdr34	4.6944E-05	1.72577881	0.013	0.003	1
Hsd11b2	4.706E-05	1.64806912	0.011	0.002	1
Rft1	4.7767E-05	0.49845036	0.036	0.015	1
Pias3	4.7924E-05	0.41918652	0.041	0.018	1
Gm5141	4.8734E-05	1.19535165	0.013	0.003	1
Mbip	4.9531E-05	0.30858607	0.073	0.039	1
Carf	5.0485E-05	0.69651239	0.036	0.015	1
Pinx1	5.1236E-05	0.52998403	0.07	0.037	1
Pithd1	5.1257E-05	0.2719006	0.075	0.04	1
Dusp28	5.1915E-05	0.82508384	0.021	0.007	1
Papd7	5.2014E-05	0.48879438	0.061	0.031	1

1700066M21F	5.3195E-05	0.86585142	0.02	0.006	1
Zbtb14	5.3254E-05	0.56010347	0.027	0.01	1
March3	5.4339E-05	0.35177922	0.036	0.015	1
Dync2h1	5.4544E-05	0.9341139	0.029	0.011	1
Adck2	5.4711E-05	0.57457081	0.039	0.017	1
A130010J15Ril	5.8201E-05	0.66359153	0.025	0.009	1
6330403K07R	5.8398E-05	1.05632957	0.018	0.005	1
Zfp607a	5.8463E-05	0.38581751	0.038	0.016	1
Map3k12	5.8678E-05	0.60362452	0.045	0.02	1
Mlst8	5.8745E-05	0.41994758	0.038	0.016	1
Gmpr2	5.9724E-05	0.44779353	0.059	0.03	1
Wdr92	5.983E-05	0.60539505	0.032	0.013	1
Ugdh	6.0511E-05	0.25689089	0.102	0.06	1
Ndufaf1	6.0907E-05	0.60271948	0.041	0.018	1
Slc35a1	6.1525E-05	0.52472363	0.047	0.021	1
Kctd2	6.3006E-05	0.26021472	0.073	0.039	1
E430024I08Ril	6.321E-05	1.05534191	0.014	0.004	1
Ipo11	6.3342E-05	0.34691927	0.047	0.021	1
Gm15283	6.364E-05	0.56807023	0.043	0.019	1
Tsen15	6.3957E-05	0.42187535	0.043	0.019	1
Zc3hav1l	6.412E-05	1.91033513	0.011	0.002	1
Naa60	6.4375E-05	0.28206167	0.082	0.046	1
Polr3g	6.5528E-05	0.83872846	0.029	0.011	1
Zfp628	6.6413E-05	0.38053762	0.052	0.025	1
Mipep	6.7433E-05	0.63485577	0.038	0.016	1
Abi2	6.7562E-05	0.5368388	0.055	0.027	1
Zfp598	6.8958E-05	0.35953536	0.082	0.046	1
Trmt12	6.8964E-05	0.76472935	0.021	0.007	1
Mtor	6.9348E-05	0.29485849	0.102	0.06	1
Ppa2	7.0637E-05	0.4571555	0.059	0.03	1
Otud3	7.1032E-05	0.79950384	0.036	0.015	1
3010003L21Ri	7.2155E-05	0.87137324	0.034	0.014	1
Dhrs1	7.3336E-05	0.52337379	0.095	0.056	1
Msh5	7.3399E-05	1.23369833	0.013	0.003	1
Zbtb17	7.3614E-05	0.34142433	0.086	0.049	1
Git1	7.3724E-05	0.43179586	0.072	0.039	1
Adam12	7.463E-05	0.98704527	0.025	0.009	1
Mzt2	7.5818E-05	0.68830215	0.052	0.025	1
Mob3a	8.0548E-05	0.31461385	0.093	0.054	1
Ginm1	8.127E-05	0.31806298	0.129	0.081	1
Zfp961	8.1469E-05	0.50411905	0.029	0.011	1
Pi4k2b	8.4181E-05	0.30756992	0.081	0.045	1
Gm14296	8.5137E-05	0.98612355	0.018	0.005	1
Dnajb12	8.5451E-05	0.28648041	0.093	0.054	1
Kcnn4	8.5761E-05	0.25135215	0.141	0.091	1
Bloc1s6	8.7087E-05	0.34090471	0.084	0.048	1
Fam173b	8.8113E-05	0.3684507	0.054	0.026	1
Npas2	8.8293E-05	1.63003122	0.011	0.002	1

Guf1	8.9356E-05	0.27776258	0.057	0.029	1
Xkr6	8.9572E-05	0.68230648	0.025	0.009	1
Mboat1	9.0134E-05	0.96688369	0.023	0.008	1
Gcfc2	9.2117E-05	0.47938444	0.03	0.012	1
Nr2c2ap	9.233E-05	0.30154393	0.082	0.046	1
Gm4316	9.3147E-05	1.5738332	0.014	0.004	1
Map9	9.4587E-05	0.92342849	0.025	0.009	1
Prep	9.5136E-05	0.48048466	0.068	0.036	1
Fam219b	9.5732E-05	0.31813718	0.043	0.019	1
Fdxacb1	9.6781E-05	0.30016073	0.059	0.03	1
Cyb5d2	9.7058E-05	0.74134236	0.038	0.016	1
Gtpbp10	9.7692E-05	0.25191951	0.048	0.023	1
Mettl6	9.8931E-05	0.33807337	0.093	0.054	1
Bspry	9.8947E-05	0.73268716	0.021	0.007	1
Dnajc18	0.00010048	0.40563066	0.052	0.025	1
Miga2	0.00010079	1.58566637	0.013	0.003	1
Twnk	0.00010375	0.33757773	0.038	0.016	1
Ap4m1	0.00010423	0.34270925	0.068	0.036	1
Sqle	0.00010503	0.26150685	0.089	0.052	1
Rcc1l	0.00010556	0.43088807	0.038	0.016	1
Arl16	0.00010572	0.87305991	0.032	0.013	1
Gtpbp8	0.00010589	0.65052286	0.041	0.019	1
Smtn	0.00010602	1.55693374	0.014	0.004	1
A430046D13R	0.00010731	0.71234617	0.018	0.005	1
Ascc1	0.00010813	0.46363075	0.075	0.042	1
Tedc2	0.00010906	1.57433855	0.013	0.003	1
Hemk1	0.00011136	0.44381323	0.03	0.012	1
Nipal1	0.00011185	0.60996833	0.027	0.01	1
2310057M21R	0.00011431	0.40114096	0.05	0.024	1
Tbc1d7	0.00011443	1.04454526	0.023	0.008	1
Copg2	0.0001153	0.38967143	0.07	0.038	1
Cnnm4	0.00011621	0.32965144	0.063	0.033	1
Ppil3	0.00011632	0.36414032	0.091	0.053	1
Tg	0.00011637	0.96745793	0.021	0.007	1
Dis3l	0.00011701	0.73096431	0.032	0.013	1
March9	0.00011818	0.96310032	0.014	0.004	1
Gm14154	0.00012165	1.11199195	0.02	0.006	1
Fam185a	0.0001247	0.62931948	0.027	0.01	1
Dynlt1a	0.00012476	1.24849957	0.027	0.01	1
Exoc7	0.00012487	0.3112343	0.061	0.032	1
Pdk3	0.00012631	0.27949204	0.088	0.051	1
Gale	0.00012863	0.83170286	0.03	0.012	1
Nepro	0.00012887	0.47914111	0.055	0.028	1
Zfp157	0.00012945	0.36312082	0.047	0.022	1
Dclk2	0.00013004	0.58080449	0.029	0.011	1
Scap	0.00013087	0.44816802	0.068	0.037	1
Uxs1	0.0001343	0.4081583	0.059	0.031	1
Tbc1d22b	0.00013985	0.31186159	0.043	0.02	1

Snupn	0.00014169	0.45577492	0.039	0.018	1
Zdhhc8	0.00014303	0.52088297	0.048	0.023	1
Mcat	0.00014914	0.8996061	0.018	0.006	1
Lmtk3	0.0001501	0.62414806	0.025	0.009	1
Hic1	0.00015195	0.29190627	0.073	0.041	1
Zfp141	0.00015711	0.43692081	0.032	0.013	1
Msra	0.00015905	0.30506328	0.073	0.041	1
Tchp	0.00015968	0.3931829	0.036	0.016	1
Zfp341	0.00015988	1.04828331	0.02	0.007	1
Drg2	0.00016245	0.2910135	0.057	0.029	1
AC163720.3	0.00016437	1.25320899	0.016	0.005	1
E530011L22Ri	0.00016502	0.42230013	0.057	0.029	1
Cmc4	0.00016683	0.66204446	0.036	0.016	1
Paqr7	0.00016878	0.93821541	0.021	0.007	1
Paqr3	0.0001716	1.22111992	0.014	0.004	1
Trbv20	0.00017201	1.23977981	0.011	0.003	1
Kptn	0.00017213	0.3612597	0.055	0.028	1
Tstd2	0.00017406	0.45077675	0.054	0.027	1
Med24	0.00017497	0.28490669	0.061	0.032	1
Tceanc	0.00017774	0.63023692	0.025	0.009	1
Mrps18b	0.00017959	0.32480222	0.093	0.055	1
Stk35	0.00018462	0.56644402	0.047	0.023	1
Erbb3	0.00018818	1.16791731	0.013	0.003	1
Poldip2	0.00019203	0.71197218	0.057	0.03	1
Slc25a17	0.00019474	0.28060212	0.089	0.053	1
AC174678.1	0.00019643	0.94365241	0.014	0.004	1
Gm11131	0.00020248	0.47042522	0.027	0.01	1
Gm6710	0.00020256	1.26681146	0.016	0.005	1
Gpn1	0.00020752	0.29013633	0.07	0.038	1
Ctnnbip1	0.00020894	0.58997117	0.079	0.046	1
Cetn4	0.00020925	1.18164035	0.029	0.012	1
Orc5	0.00020926	0.6341671	0.025	0.01	1
Kremen1	0.00021698	0.36155507	0.047	0.023	1
Pigc	0.00022111	0.43748887	0.061	0.032	1
Uqcc1	0.00022458	0.33618678	0.052	0.026	1
Fam160a2	0.00023279	0.35960896	0.041	0.019	1
Zfp955b	0.00023328	1.14594691	0.014	0.004	1
Kbtbd4	0.00023576	0.55627806	0.039	0.018	1
Nutf2	0.00024121	1.33250591	0.014	0.004	1
Zfp934	0.00024777	1.02747286	0.018	0.006	1
B4galt4	0.0002495	1.17545781	0.018	0.006	1
Iqcc	0.00024971	0.69638902	0.041	0.019	1
Golga1	0.00025112	0.48204698	0.036	0.016	1
Tyw3	0.0002517	1.0547033	0.016	0.005	1
Jmjd7	0.00025695	0.89815302	0.018	0.006	1
Il3ra	0.00025821	0.4406677	0.07	0.039	1
Mtfmt	0.00025866	0.32187659	0.047	0.023	1
Coa4	0.00026221	0.49991673	0.047	0.023	1

Fam124b	0.00026321	0.76743549	0.027	0.011	1
Gtf2h2	0.00026358	0.44700345	0.045	0.022	1
Nxn	0.00026791	0.54577968	0.039	0.018	1
Atpaf1	0.00027616	0.36211319	0.032	0.014	1
Mtcp1	0.00027881	0.62169495	0.03	0.013	1
Zfp526	0.00028357	1.45545627	0.011	0.003	1
Mettl5	0.00028411	0.38856356	0.055	0.029	1
Gm26625	0.00028981	0.63346678	0.018	0.006	1
Dimt1	0.00029636	0.45485948	0.047	0.023	1
Coq9	0.00029861	0.33999867	0.043	0.021	1
Calcrl	0.00029928	0.30759114	0.055	0.029	1
Ccdc94	0.00029985	0.34127375	0.039	0.018	1
Zfp81	0.0003154	0.30278318	0.039	0.018	1
Fastk	0.00032614	0.27063303	0.063	0.034	1
Cd101	0.00032727	0.33977865	0.045	0.022	1
Slc36a3os	0.00032776	0.60955568	0.059	0.032	1
H60b	0.00033264	0.48615131	0.038	0.017	1
0610009B22R	0.00033419	0.64257696	0.036	0.016	1
Tmco4	0.00033522	0.43058176	0.034	0.015	1
4833403J16Ril	0.00033536	0.35995758	0.032	0.014	1
Bdh1	0.00035532	0.36442013	0.03	0.013	1
Letmd1	0.00036014	0.27925261	0.054	0.028	1
Ak3	0.00036556	0.47832111	0.063	0.035	1
D11Wsu47e	0.00036666	0.93926483	0.014	0.004	1
Gm20234	0.00036666	0.52368666	0.025	0.01	1
Gm14305	0.00036805	0.48718226	0.029	0.012	1
Gm28875	0.00037057	0.57611507	0.045	0.022	1
Heatr3	0.00038181	0.29633488	0.063	0.034	1
Rgs12	0.00038338	0.85941159	0.027	0.011	1
Oxnad1	0.00038397	0.81524459	0.023	0.009	1
Kif3c	0.00039024	0.68792983	0.021	0.008	1
Yif1a	0.00039702	0.34362976	0.077	0.045	1
Tada2a	0.00040034	1.02638472	0.021	0.008	1
Clstn1	0.00040861	0.45992923	0.039	0.019	1
Gpr15	0.00041175	1.14024758	0.014	0.004	1
Ankrd26	0.00041232	0.59756897	0.029	0.012	1
Trim14	0.00042114	0.72808448	0.023	0.009	1
Gfm1	0.00042432	0.31688847	0.052	0.027	1
Zfp335os	0.00043337	0.93946462	0.018	0.006	1
Poc1b	0.00043458	0.25445315	0.041	0.02	1
Dctn5	0.00044059	0.25538091	0.093	0.057	1
Zfp764	0.00045665	0.88253434	0.02	0.007	1
Adal	0.00046269	0.52993844	0.023	0.009	1
Pop1	0.00046638	0.9953657	0.025	0.01	1
Rdh14	0.00047166	0.30856021	0.05	0.026	1
Pramef8	0.00047603	0.26848806	0.063	0.035	1
Ankrd13b	0.00048962	1.24181853	0.011	0.003	1
Atxn7l2	0.00049392	0.32027737	0.034	0.015	1

Cacnb3	0.0004967	1.24147135	0.018	0.006	1
Vps45	0.00049856	0.52564806	0.039	0.019	1
AW554918	0.00050294	0.26663096	0.047	0.024	1
Dnaaf2	0.00051211	1.04029573	0.014	0.004	1
Cog1	0.00052753	0.40250352	0.057	0.031	1
Hk1os	0.00052962	1.02489597	0.011	0.003	1
Rpl39l	0.00053086	1.31242767	0.02	0.007	1
Gtpbp3	0.0005361	0.45973939	0.027	0.011	1
Snai3	0.00053822	1.09393597	0.014	0.004	1
1190007I07Ril	0.00053943	0.3871098	0.059	0.032	1
Rpain	0.00054213	0.27325291	0.054	0.029	1
Pex12	0.00055723	0.62787819	0.025	0.01	1
B3galnt2	0.00055914	0.2635877	0.055	0.03	1
Ano10	0.00056443	0.58555134	0.048	0.025	1
Zfp119a	0.00056762	0.40683329	0.03	0.013	1
Zfp40	0.00057034	0.64080261	0.018	0.006	1
Gm26514	0.00057866	0.99807696	0.018	0.006	1
Mus81	0.00058003	0.62065663	0.027	0.011	1
Slc25a44	0.000599	0.44436416	0.061	0.034	1
1810055G02R	0.00060449	0.62663004	0.027	0.011	1
Celsr1	0.00060731	0.3255413	0.03	0.013	1
Mtx2	0.00064882	0.31291799	0.068	0.039	1
Slc35b4	0.00065325	0.45877601	0.041	0.02	1
Zfp809	0.00067156	0.27034713	0.059	0.033	1
Eya2	0.00067374	0.68212998	0.018	0.006	1
Cep78	0.00067768	0.80136681	0.02	0.007	1
Rrp36	0.00067953	0.25603075	0.068	0.039	1
Igkv8-30	0.00068321	0.98167021	0.013	0.004	1
Ptprk	0.00068583	1.06377489	0.014	0.005	1
Trim21	0.00068878	0.27814147	0.038	0.018	1
Glmn	0.00068897	0.37868034	0.034	0.016	1
Mecr	0.00070818	0.81436485	0.036	0.017	1
Ermard	0.00071096	0.31570725	0.041	0.02	1
Krtcap3	0.00071363	0.51203824	0.027	0.011	1
Lipo3	0.00071376	0.32141635	0.055	0.03	1
Ap4e1	0.00071949	0.41747715	0.05	0.026	1
Magee1	0.0007236	0.74131689	0.016	0.005	1
Gm31597	0.00072761	0.25940928	0.043	0.022	1
Ndrg2	0.00072873	0.93313862	0.014	0.005	1
Mrm2	0.00075656	1.12873521	0.011	0.003	1
Dsel	0.00076442	0.82537235	0.021	0.008	1
Rmdn2	0.00076487	0.59182626	0.021	0.008	1
Zbtb22	0.0007743	0.33515871	0.055	0.03	1
Eif5a2	0.00077532	0.84735991	0.029	0.013	1
Xist	0.00079584	0.60340989	0.435	0.423	1
Commd10	0.00080986	0.28069585	0.059	0.033	1
Gm6277	0.00081519	1.1899167	0.013	0.004	1
Ascc2	0.00081892	0.29672779	0.068	0.04	1

Tnks1bp1	0.00082568	0.50864844	0.027	0.011	1
Gm26847	0.00084038	0.58376344	0.018	0.006	1
Tyw1	0.00084479	0.33873738	0.039	0.019	1
Bcl2l15	0.00085374	1.43396682	0.011	0.003	1
Prr12	0.00085918	0.35437525	0.032	0.015	1
B3galt4	0.00085983	1.09285621	0.021	0.008	1
Dcaf4	0.000917	0.51717704	0.032	0.015	1
Nit2	0.00093479	0.45761061	0.047	0.025	1
Fam206a	0.00095423	0.39610868	0.039	0.02	1
Gm17655	0.0009703	0.90498003	0.014	0.005	1
Dcun1d3	0.00097169	0.41551101	0.043	0.022	1
Trpm4	0.00097489	0.69982444	0.027	0.012	1
Papss1	0.00099273	0.28354739	0.057	0.032	1
Arfip2	0.00099677	0.48130414	0.032	0.015	1
Hddc3	0.00100543	0.94903962	0.023	0.01	1
Fam120aos	0.00102897	0.80184675	0.021	0.008	1
Lrrc8a	0.00104343	0.33008882	0.048	0.026	1
Tmem39b	0.0010551	0.40362131	0.034	0.016	1
Pced1a	0.00109081	0.32538087	0.034	0.016	1
Gm47551	0.00110972	1.49356501	0.011	0.003	1
Magi2	0.00114301	0.90068218	0.018	0.007	1
Vps9d1	0.00115496	0.35414489	0.055	0.031	1
Them6	0.00117103	0.32292226	0.029	0.013	1
BC147527	0.00118957	1.18941549	0.011	0.003	1
Cpe	0.00119851	0.69913553	0.018	0.007	1
Dyrk1b	0.00120322	0.50560231	0.047	0.025	1
Gss	0.00122254	0.54344307	0.036	0.018	1
Tppp	0.00122297	0.71651785	0.016	0.006	1
Wdr74	0.00123789	0.40571804	0.043	0.022	1
Ammecr1	0.00123826	0.33653618	0.03	0.014	1
Ccdc51	0.00124796	1.17646605	0.013	0.004	1
Tmem240	0.00125562	1.09046675	0.013	0.004	1
Serf1	0.00125886	0.77419859	0.032	0.015	1
Cbr4	0.00127595	0.77257569	0.027	0.012	1
Gm5106	0.00127876	1.05243262	0.011	0.003	1
Med6	0.00130343	0.25580499	0.068	0.04	1
B3glct	0.001351	0.78619069	0.025	0.011	1
Tmem267	0.00135513	2.14067018	0.016	0.006	1
Btd	0.00139663	0.54119973	0.036	0.018	1
Atcayos	0.00141014	0.73335849	0.013	0.004	1
Map6	0.00143715	1.17782541	0.011	0.003	1
Atp6v1g2	0.00144866	0.87951617	0.014	0.005	1
Lrch4	0.00147915	0.83906507	0.018	0.007	1
Umad1	0.00149216	0.30766813	0.036	0.018	1
Znhit2	0.00149603	0.25756777	0.054	0.03	1
Lyrm1	0.00152321	0.64608583	0.032	0.015	1
Gm12258	0.00152773	0.82918321	0.014	0.005	1
Rpap1	0.00156324	0.35515815	0.039	0.02	1

Bbs4	0.00159236	0.81942347	0.016	0.006	1
Hist1h4n	0.00160213	0.78431023	0.016	0.006	1
Fubp3	0.00160758	0.5365748	0.048	0.027	1
Fastkd1	0.00161267	0.36137489	0.034	0.017	1
Bloc1s1	0.00161913	0.2761771	0.066	0.04	1
R3hcc1	0.00162192	0.35290214	0.034	0.017	1
Gm44777	0.0016459	0.30638309	0.025	0.011	1
Rpusd1	0.00164622	0.71016598	0.016	0.006	1
Txndc15	0.00165193	0.34651382	0.086	0.055	1
Top3a	0.00168112	0.35072228	0.034	0.017	1
5830454E08R	0.00168917	0.32726806	0.014	0.005	1
Kif1bp	0.00168928	0.30801715	0.048	0.026	1
Farp1	0.00169061	0.4395745	0.03	0.014	1
Gstp1	0.00169415	0.67223075	0.041	0.022	1
Ube2v1	0.00169627	0.27097015	0.039	0.02	1
Tmed4	0.00172023	0.34750087	0.064	0.038	1
Nprl2	0.00172755	0.38680542	0.032	0.015	1
Coasy	0.0017456	0.54864991	0.036	0.018	1
Paox	0.00178417	0.78400125	0.038	0.019	1
Rfx2	0.00179434	1.13539445	0.016	0.006	1
Fadd	0.00183832	0.62700742	0.023	0.01	1
Sdhaf3	0.001843	0.51503537	0.03	0.014	1
Cdpf1	0.00189544	0.31303138	0.032	0.016	1
Zfp61	0.00194407	0.87476546	0.014	0.005	1
Rab3d	0.00199224	0.77189543	0.032	0.016	1
Dhx35	0.00200926	0.35135555	0.048	0.027	1
Rbm19	0.00203695	0.27401925	0.073	0.045	1
Zfp319	0.00208513	0.30744753	0.039	0.02	1
Ttll4	0.00209516	0.25467461	0.036	0.018	1
Gatc	0.00209814	0.9794612	0.016	0.006	1
Rad1	0.00210198	0.25404305	0.032	0.016	1
Slc30a6	0.00210936	0.73174674	0.029	0.013	1
Zscan20	0.00212209	0.69743999	0.014	0.005	1
1700021F05Ri	0.00214134	0.27491175	0.039	0.021	1
2810021J22Ril	0.00214404	0.53954972	0.014	0.005	1
Zfp775	0.00215624	0.77574544	0.018	0.007	1
Bloc1s5	0.00215837	0.76735803	0.021	0.009	1
Ccdc130	0.00217441	0.29547591	0.034	0.017	1
Aplp1	0.00218113	0.46413205	0.023	0.01	1
Ccdc30	0.00219008	0.37143544	0.029	0.013	1
Gm20707	0.00226751	0.3792219	0.032	0.016	1
4930524O07R	0.00234439	1.0870718	0.013	0.004	1
Grk4	0.00235659	1.00291222	0.013	0.004	1
BC049715	0.00240968	1.2873403	0.014	0.005	1
Disp1	0.00244241	0.45648916	0.025	0.011	1
Hsdl2	0.00246412	0.39515238	0.064	0.039	1
Tmem143	0.00250206	0.94768692	0.016	0.006	1
Dnaja3	0.0025258	0.34886605	0.034	0.017	1

Smyd4	0.00254679	0.32403093	0.025	0.011	1
Ankzf1	0.00257905	0.37653475	0.034	0.017	1
Rad51c	0.00258099	0.56867423	0.013	0.004	1
Rcor3	0.00258571	0.31329705	0.038	0.02	1
Acsf3	0.00261897	0.92744547	0.018	0.007	1
Gm48236	0.00264579	0.34746881	0.027	0.012	1
Phf7	0.002661	0.35653559	0.032	0.016	1
Chchd6	0.00266224	0.59831336	0.03	0.015	1
Akr1e1	0.00273313	0.61804585	0.025	0.011	1
Gm16740	0.00275882	0.74102315	0.018	0.007	1
Apba3	0.00277866	0.39186045	0.038	0.02	1
Mrm3	0.00278292	0.51393422	0.025	0.011	1
Slx4ip	0.00278593	0.40664107	0.041	0.022	1
Tmem161a	0.00278872	0.33393055	0.054	0.031	1
Gm26836	0.00282379	0.91704154	0.013	0.004	1
Wdr5b	0.00285626	0.51959264	0.016	0.006	1
Mvk	0.0028711	0.35272323	0.047	0.026	1
Taf8	0.00290978	0.46511092	0.043	0.023	1
Cdk5rap1	0.00295888	0.50474129	0.025	0.011	1
Rasd2	0.00298098	0.62654981	0.011	0.003	1
Mterf4	0.00299043	0.47208649	0.034	0.017	1
Ick	0.00299576	0.90762006	0.021	0.009	1
Zfp579	0.00302604	0.35520996	0.023	0.01	1
Fance	0.00306108	0.51420535	0.025	0.011	1
Cep290	0.00311836	0.40274261	0.041	0.022	1
Rtl8a	0.00312332	0.4325318	0.045	0.025	1
Fbxo46	0.00316528	0.30542255	0.034	0.017	1
Ltk	0.0031799	0.64072171	0.011	0.003	1
Jade3	0.0031884	0.80869197	0.018	0.007	1
BC025920	0.00319172	1.07067727	0.013	0.004	1
Mocs3	0.00324964	0.63345219	0.023	0.01	1
Exoc8	0.00326434	0.41646476	0.03	0.015	1
Trp53rka	0.00329157	0.29886039	0.055	0.033	1
Snapc2	0.00333269	0.38002368	0.041	0.022	1
Zfyve19	0.00333745	0.51734805	0.03	0.015	1
Urb1	0.00343327	0.60742039	0.034	0.017	1
Phf10	0.00344543	0.2745604	0.073	0.047	1
Kdelc1	0.0034491	0.95954438	0.016	0.006	1
Caprin2	0.00345316	0.84487349	0.018	0.007	1
Mettl21a	0.00352198	0.89028958	0.02	0.008	1
Gpatch1	0.00356725	0.49572285	0.029	0.014	1
Tjp3	0.00359444	1.37363781	0.014	0.005	1
Paqr8	0.00370997	0.80039417	0.014	0.005	1
Acyp2	0.00376263	0.43347123	0.029	0.014	1
Trpc1	0.00378585	1.1674156	0.011	0.003	1
Cdh24	0.00378646	0.57894952	0.014	0.005	1
Gstm5	0.00379198	0.2784406	0.047	0.027	1
Praf2	0.00379316	0.33906436	0.043	0.024	1

Zfp867	0.00382094	0.81973355	0.013	0.004	1
Trim32	0.00383584	0.76853048	0.013	0.004	1
Myo1f	0.00387347	0.29280098	0.114	0.081	1
C920021L13R	0.00390136	0.69050863	0.016	0.006	1
1110034G24R	0.00396592	0.3317165	0.041	0.023	1
Acbd4	0.00398657	0.67984446	0.025	0.012	1
Zfp606	0.00399643	0.7086658	0.016	0.006	1
Sgtb	0.00400238	0.3223606	0.039	0.021	1
BC051226	0.00402035	0.49557895	0.02	0.008	1
Pqlc2	0.00414433	0.81960116	0.02	0.008	1
Atp23	0.0041524	0.56853323	0.018	0.007	1
Unc5cl	0.00424961	0.49401306	0.014	0.005	1
Arhgap27os3	0.00439716	0.51865531	0.02	0.008	1
0610009O20R	0.00444263	0.40429607	0.034	0.018	1
Tysnd1	0.00447235	0.26306921	0.032	0.017	1
Olfr655	0.00448784	0.85691345	0.014	0.005	1
Esm1	0.00456886	1.02814803	0.011	0.004	1
Exog	0.00457463	1.23007092	0.011	0.004	1
Mtus2	0.00459679	0.84680127	0.016	0.006	1
Ccdc136	0.00461248	1.31509811	0.011	0.004	1
Cox15	0.00467472	0.59062533	0.034	0.018	1
4930430F08Ri	0.00476601	0.42774284	0.021	0.01	1
Rfng	0.00479878	0.41766572	0.03	0.015	1
Snx15	0.0048519	0.26524037	0.043	0.024	1
Ccdc61	0.00489164	0.43656394	0.016	0.006	1
Kat2a	0.00498325	0.51219514	0.032	0.017	1
Gpx7	0.0050548	0.81870594	0.013	0.004	1
Cd151	0.00509021	0.63600935	0.021	0.01	1
Zfp956	0.00512904	0.37631029	0.025	0.012	1
Thoc5	0.005183	0.39464972	0.047	0.027	1
Mcts2	0.0052732	1.14578593	0.011	0.004	1
Pla2g6	0.00537074	0.84834564	0.016	0.006	1
Gm15232	0.00546429	0.38047298	0.021	0.01	1
Ccr9	0.00549927	0.2691632	0.043	0.024	1
Slc52a3	0.00563137	0.45059461	0.029	0.014	1
Igsf9b	0.00564629	0.40263869	0.011	0.004	1
Smpd5	0.00579631	0.36717658	0.018	0.008	1
Arl3	0.00582681	0.35473915	0.088	0.06	1
Zfp790	0.00600852	0.92046066	0.016	0.007	1
Zfp799	0.00603538	0.25481849	0.025	0.012	1
E130208F15Ri	0.00604759	0.38047355	0.027	0.013	1
Kcna3	0.00605975	0.8954911	0.013	0.005	1
Zfp85	0.00606721	0.61534923	0.014	0.006	1
Gm6598	0.00611683	0.59230988	0.011	0.004	1
Abcd4	0.00614402	0.29549921	0.038	0.021	1
BC024063	0.0061613	0.49991801	0.011	0.004	1
Ric8b	0.00619924	0.34077943	0.036	0.019	1
Gm26610	0.0062035	0.66432308	0.014	0.006	1

Drosha	0.00620482	0.49956072	0.038	0.021	1
Cnpy4	0.00624175	0.4924206	0.038	0.021	1
Ptpn21	0.00634246	0.55236616	0.013	0.005	1
Epop	0.00636985	0.91231004	0.018	0.008	1
Ntng2	0.00637246	0.44673043	0.013	0.005	1
Sars2	0.0065287	0.47867637	0.027	0.013	1
Hmgxb3	0.00654742	0.33927277	0.052	0.031	1
Rfxank	0.00673646	0.5063643	0.036	0.02	1
A430033K04R	0.00677664	0.48588949	0.016	0.007	1
Gm26533	0.00684689	0.3224335	0.021	0.01	1
Armc6	0.00695306	0.677744	0.016	0.007	1
Gm16845	0.0070419	0.75992078	0.013	0.005	1
Ano8	0.00718935	0.61099156	0.02	0.009	1
Galk1	0.00725986	0.26222167	0.039	0.022	1
Tmem177	0.00728015	0.69070319	0.014	0.006	1
Ppp1r3e	0.00733335	0.99341705	0.014	0.006	1
Pde6d	0.00733823	0.40444822	0.03	0.016	1
Hist2h2be	0.00737263	0.78915292	0.016	0.007	1
Zfp566	0.00737373	0.44589906	0.014	0.006	1
Tfb1m	0.00737884	0.9107735	0.014	0.006	1
Zbtb34	0.0073931	0.52308007	0.016	0.007	1
Myo10	0.00741412	0.52131131	0.027	0.014	1
Slc25a23	0.00758935	0.51682991	0.023	0.011	1
Mrps27	0.00768216	0.43868767	0.032	0.017	1
Fbxl19	0.00771119	0.44491481	0.02	0.009	1
Nupl2	0.00778841	0.97332647	0.014	0.006	1
Zfp709	0.00788199	0.518554	0.023	0.011	1
Tyw5	0.00790611	0.3678066	0.034	0.019	1
Cstad	0.00798115	0.61525673	0.016	0.007	1
Alg3	0.00800086	0.5029326	0.029	0.015	1
Sap130	0.00810491	0.37599107	0.034	0.019	1
Mypopos	0.00819327	0.32006058	0.018	0.008	1
Trdmt1	0.00822889	1.00779355	0.018	0.008	1
AW209491	0.00831428	0.86683355	0.013	0.005	1
Trmu	0.008323	0.35238216	0.016	0.007	1
Zfp189	0.0084075	0.73062835	0.013	0.005	1
Ctu1	0.00851995	0.56156906	0.02	0.009	1
Hps6	0.00858759	0.56034777	0.029	0.015	1
Rsph3b	0.00860013	0.95091215	0.013	0.005	1
Znrd1as	0.00872088	0.51121033	0.013	0.005	1
Naif1	0.00883152	0.37155175	0.021	0.01	1
Aars2	0.00913147	0.71619061	0.016	0.007	1
Elac2	0.00915202	0.39112202	0.047	0.028	1
Zcchc3	0.00916591	0.79117896	0.011	0.004	1
Tsen54	0.00925499	0.33721717	0.025	0.013	1
P4ha2	0.00928798	0.64121144	0.014	0.006	1
Car5b	0.00940515	0.79757916	0.023	0.011	1
Gm32856	0.00942728	0.39170091	0.014	0.006	1

Ttl	0.00954824	0.61825505	0.021	0.01	1
1810009A15R	0.00957122	0.45413595	0.02	0.009	1
Ccdc181	0.00966865	1.04177348	0.018	0.008	1
Gm26601	0.00976551	1.14764927	0.011	0.004	1
Lca5	0.00979784	0.77100731	0.014	0.006	1
AC113595.1	0.00998725	0.41120264	0.013	0.005	1
Gpt	0.01001868	0.59329621	0.02	0.009	1
Dtd2	0.01002291	0.54433277	0.021	0.01	1
Flywch2	0.01002604	0.62971168	0.014	0.006	1
Lrrc42	0.01010312	0.25767839	0.041	0.024	1
Gm5914	0.01011039	0.2920263	0.039	0.023	1
Swsap1	0.01031678	0.69751046	0.014	0.006	1
Asb7	0.01035935	0.45904796	0.029	0.015	1
Phf11c	0.01062683	0.60974135	0.018	0.008	1
Wdr59	0.01064079	0.26843978	0.025	0.013	1
Klhl36	0.01072495	0.268379	0.023	0.012	1
Tmem138	0.01075065	0.3871296	0.021	0.01	1
5430403G16R	0.01093993	0.448863	0.018	0.008	1
Wdr55	0.01120068	0.33162991	0.038	0.022	1
Chid1	0.01146624	0.33631282	0.038	0.022	1
Gm26798	0.01176125	0.42438097	0.014	0.006	1
Gldc	0.01192517	1.12808506	0.016	0.007	1
Golph3l	0.01203795	0.26789874	0.068	0.045	1
St14	0.01241021	0.61043404	0.021	0.011	1
Atg4a	0.01277401	0.29092866	0.029	0.015	1
Thnsl1	0.01282402	1.11624868	0.011	0.004	1
3830403N18R	0.01287441	0.6634249	0.014	0.006	1
Fan1	0.01301324	0.31012983	0.016	0.007	1
Mief2	0.01303694	0.37674071	0.018	0.008	1
Pcbp3	0.01310703	0.81495122	0.014	0.006	1
Stx18	0.01328334	0.29204026	0.05	0.031	1
Tmco6	0.01340004	0.36015342	0.032	0.018	1
Coq3	0.01363805	0.48882568	0.023	0.012	1
Mllt1	0.01375619	0.28526441	0.03	0.017	1
Gls2	0.01379532	0.76422928	0.016	0.007	1
Ptrh1	0.01387783	0.45278802	0.023	0.012	1
B230317F23Ri	0.0139191	0.62782545	0.011	0.004	1
Ube2e2	0.01424149	1.22617805	0.016	0.007	1
Zfp605	0.01467474	0.46650921	0.016	0.007	1
Ddx51	0.01524331	0.27283712	0.029	0.016	1
Zfp35	0.01545836	0.30488748	0.029	0.016	1
Faap24	0.01578047	0.26223077	0.032	0.018	1
Fads1	0.01597409	0.57319652	0.034	0.02	1
Cysltr1	0.01616011	0.36822341	0.073	0.051	1
Wdr77	0.01622417	0.26896643	0.038	0.022	1
Zfp46	0.01656244	0.37376575	0.021	0.011	1
Tatdn3	0.0169509	0.63015466	0.021	0.011	1
Rab40c	0.0172313	0.25464208	0.036	0.021	1

Fastkd3	0.01735265	0.47890413	0.023	0.012	1
G630030J09Ri	0.01751022	0.84984844	0.011	0.004	1
Nudt13	0.01752005	0.37441461	0.038	0.022	1
4931428F04Ri	0.01757256	0.83884366	0.011	0.004	1
Ramp1	0.01761257	0.259805	0.111	0.083	1
Zfp213	0.017738	0.51482934	0.014	0.006	1
Endov	0.01777743	0.33220673	0.023	0.012	1
1110051M20F	0.01852591	0.5417527	0.021	0.011	1
Vps25	0.01897285	0.51902302	0.018	0.009	1
Rpusd2	0.01908177	0.95693467	0.013	0.005	1
Ubxn2b	0.01929929	0.31999442	0.016	0.007	1
Naprt	0.01932528	0.7561312	0.011	0.004	1
Pomc	0.01934695	0.28268205	0.023	0.012	1
Mex3d	0.01955587	0.45214495	0.021	0.011	1
Acoxl	0.02032999	0.7445196	0.013	0.005	1
Alg12	0.02035577	0.54569086	0.023	0.012	1
Suox	0.02061695	0.62853851	0.025	0.014	1
Adgrg1	0.0206192	0.75149615	0.016	0.008	1
Gm44066	0.02068417	0.46839502	0.014	0.006	1
Zbtb48	0.02124262	0.3385499	0.021	0.011	1
9930111J21Ril	0.02164838	0.41188265	0.013	0.005	1
Repin1	0.0219357	0.361728	0.02	0.01	1
Usp54	0.02268294	0.55486882	0.011	0.004	1
Gtf2ird1	0.02323014	0.51291745	0.013	0.005	1
Gm6169	0.02336525	0.97933875	0.013	0.005	1
Sept10	0.02375993	1.34682233	0.013	0.005	1
Elp4	0.0244275	0.25695828	0.021	0.011	1
Stard3	0.02463901	0.31525167	0.055	0.037	1
Det1	0.02480963	0.48400211	0.016	0.008	1
Gm14325	0.02554322	0.6184574	0.014	0.007	1
Gm11831	0.02610158	0.36667796	0.013	0.005	1
Pigh	0.02616734	0.41360511	0.02	0.01	1
Spindoc	0.02627497	0.47055297	0.025	0.014	1
Mt3	0.02676563	0.8749396	0.013	0.005	1
Dpagt1	0.0270615	0.28366762	0.036	0.022	1
Fah	0.0274471	0.46563497	0.013	0.005	1
Ilvbl	0.02842003	0.52081945	0.059	0.041	1
Gm48855	0.02846528	0.92043797	0.011	0.004	1
Ptges2	0.02849437	0.44492147	0.025	0.014	1
F2rl2	0.03002678	0.27337675	0.016	0.008	1
Lrp8	0.03003144	0.91154285	0.025	0.014	1
9930104L06Ri	0.03109816	0.26724278	0.016	0.008	1
Arhgef40	0.03131257	0.94799905	0.016	0.008	1
Rars2	0.03288134	0.59927402	0.018	0.009	1
Snap47	0.03293312	0.30780337	0.016	0.008	1
Sept4	0.0331701	0.49273034	0.011	0.005	1
Eid2	0.03347582	0.46095035	0.014	0.007	1
Plcb3	0.03351724	0.34542069	0.02	0.01	1

Xpo5	0.03365236	0.39803506	0.021	0.012	1
9130011E15R	0.03366883	0.80220657	0.011	0.005	1
Gm13012	0.0340668	0.38869861	0.021	0.012	1
Timm23	0.03464262	0.27098324	0.032	0.02	1
Mars2	0.03473299	0.25795041	0.02	0.01	1
Sord	0.03494335	0.32692828	0.036	0.022	1
Serpina3f	0.03507643	0.38930673	0.032	0.02	1
Adck5	0.03552357	0.29937462	0.021	0.012	1
Pus10	0.03840763	0.41217012	0.032	0.02	1
Smarcal1	0.03885669	0.31172496	0.029	0.017	1
Slc25a14	0.04159702	0.37815235	0.014	0.007	1
Rmnd1	0.04169026	0.37639858	0.023	0.013	1
Phka2	0.04215409	0.52992254	0.036	0.023	1
Gm45606	0.04381788	0.8247941	0.011	0.005	1
Fig4	0.04397034	0.29259627	0.023	0.013	1
Slc25a10	0.04424709	0.45557914	0.021	0.012	1
Ift74	0.04479655	0.30372004	0.016	0.008	1
Spef2	0.04630592	0.5240345	0.011	0.005	1
Msto1	0.04658424	0.27007842	0.027	0.016	1
Cryba4	0.04752422	0.26051362	0.021	0.012	1
4933406P04R	0.04790692	0.67309458	0.016	0.008	1
Gstm4	0.04817204	0.76801873	0.013	0.006	1
Ggact	0.04925331	0.25771592	0.016	0.008	1
Tmem170	0.04979291	0.48502936	0.011	0.005	1
Flywch1	0.05129745	0.25140655	0.027	0.016	1
Tmem41a	0.05305685	0.77630901	0.011	0.005	1
Mthfd2l	0.05367963	0.25069206	0.03	0.019	1
2900076A07R	0.05406032	0.29118309	0.023	0.014	1
Dynlt1f	0.05420013	0.31877779	0.043	0.029	1
Amer1	0.05422248	0.26480284	0.014	0.007	1
Fbxl2	0.05599554	0.6471047	0.013	0.006	1
Slc12a4	0.05644017	0.3534758	0.038	0.025	1
Dnaaf5	0.05660763	0.55163174	0.018	0.01	1
Zswim7	0.0598631	0.29693311	0.038	0.025	1
Man2c1os	0.06012678	0.35428796	0.013	0.006	1
Tab1	0.06053316	0.48400073	0.014	0.007	1
Uros	0.06100969	0.63397742	0.014	0.007	1
Uhrf1bp1	0.06118844	1.01657667	0.013	0.006	1
Fbxl18	0.06120822	0.50124044	0.018	0.01	1
Gm19684	0.06129448	0.40051823	0.011	0.005	1
Gm9774	0.06366642	0.48222087	0.011	0.005	1
Slc6a8	0.06548507	0.50950057	0.018	0.01	1
Nmnat1	0.06572277	0.36220989	0.016	0.009	1
Cmtr2	0.06927464	0.56234932	0.013	0.006	1
Tjp2	0.07046655	0.27358542	0.054	0.039	1
Gm20324	0.07087453	0.50491095	0.016	0.009	1
Slc19a2	0.07128263	0.2791849	0.016	0.009	1
Mgst3	0.07154187	0.5174443	0.039	0.027	1

U2af1l4	0.07306669	0.32965127	0.014	0.008	1
Slc39a8	0.07417772	0.64114812	0.013	0.006	1
Ogg1	0.07800213	0.54558709	0.013	0.006	1
Rpgrip1l	0.07866246	0.43736376	0.013	0.006	1
Wdr24	0.07969449	0.270464	0.02	0.012	1
Intu	0.08263579	0.35322104	0.018	0.01	1
Fntb	0.08480317	0.28084276	0.013	0.007	1
Nek1	0.08524696	0.25159057	0.023	0.014	1
Gsdme	0.08681499	0.47896062	0.016	0.009	1
Arhgap21	0.08904328	0.29537416	0.052	0.038	1
Zfp458	0.09779446	0.3940425	0.013	0.007	1
Trmt5	0.09937245	0.50447873	0.016	0.009	1
Tmem216	0.10270206	0.30798449	0.013	0.007	1
Gm4924	0.10419908	0.62419509	0.011	0.006	1
Fkbp7	0.10859301	0.34767682	0.014	0.008	1
Gtf2h3	0.10956905	0.33964461	0.016	0.009	1
Cd59a	0.11067625	0.34727746	0.013	0.007	1
Oxld1	0.11303336	0.39991303	0.014	0.008	1
Gstk1	0.11518666	0.42196487	0.018	0.011	1
Zscan12	0.11794434	0.32861416	0.013	0.007	1
Btbd2	0.11816565	0.30280778	0.02	0.012	1
Armcx6	0.11976221	0.32500252	0.011	0.006	1
Fkbpl	0.12162013	0.31136979	0.013	0.007	1
Trim16	0.12505841	0.34207431	0.014	0.008	1
Rab29	0.1275151	0.26345927	0.025	0.017	1
Phf11a	0.13024025	0.42201305	0.018	0.011	1
Gm9803	0.13797904	0.73585787	0.011	0.006	1
Ccdc85b	0.1380679	0.43029162	0.014	0.008	1
Slc25a15	0.13837949	0.41859701	0.011	0.006	1
Zfp58	0.13981027	0.4712771	0.013	0.007	1
Arhgap35	0.14237708	0.41919858	0.018	0.011	1
Tha1	0.1450905	0.38173475	0.011	0.006	1
Nhej1	0.14674521	0.40820842	0.011	0.006	1
Pomt1	0.14918196	0.36473365	0.011	0.006	1
C030006K11R	0.15695709	0.70100604	0.014	0.009	1
Gpm6b	0.16647236	0.30551715	0.013	0.007	1
Tspyl3	0.16724479	0.3025186	0.013	0.007	1
1700056N10R	0.16823125	0.47487722	0.011	0.006	1
Ankrd9	0.17322462	0.31346443	0.011	0.006	1
Abi3bp	0.18622787	0.30322733	0.021	0.015	1
Nme6	0.18768523	0.39561555	0.011	0.006	1
Pgap3	0.18804858	0.41543011	0.011	0.006	1
G430095P16R	0.18947628	0.44189339	0.011	0.006	1
Aifm2	0.18967585	0.50733329	0.014	0.009	1
B9d1	0.19173369	0.56217403	0.011	0.006	1
Fam20a	0.1991916	0.2528623	0.014	0.009	1
Enkur	0.2030797	0.47641161	0.011	0.006	1
Tmem69	0.22281323	0.31186553	0.011	0.007	1

Plekha8	0.22700877	0.27995588	0.014	0.009	1
Ptges3l	0.24149904	0.4271963	0.011	0.007	1
Zfp948	0.28589837	0.61449849	0.013	0.008	1
Ccdc191	0.2871731	0.26865533	0.014	0.01	1
Eid3	0.32593957	0.47235318	0.014	0.01	1
Wdsub1	0.32725615	0.32807409	0.016	0.012	1
Slc9a6	0.3549667	0.30413803	0.02	0.015	1
Cox7a1	0.35609744	0.46313618	0.013	0.009	1
Sema4f	0.38971327	0.40289558	0.013	0.009	1
Hmga1b	0.39441498	0.26932635	0.013	0.009	1
Sgsm3	0.39993003	0.81132902	0.011	0.008	1
5830432E09R	0.42311207	0.64762737	0.013	0.009	1
Macrod1	0.42729162	0.27928833	0.013	0.009	1
Gm20721	0.45256226	0.3349283	0.011	0.008	1
Zfp358	0.49020364	0.63584313	0.013	0.01	1
2310031A07R	0.49387472	0.36957164	0.029	0.024	1
Gstt3	0.55957573	0.42213048	0.011	0.008	1

CD8 Tc1

Gene	p_val	avg_log2FC	pct.1	pct.2	p_val_adj
Nkg7	0	3.39598472	0.861	0.171	0
Cd8a	0	5.4044636	0.679	0.025	0
Ccl5	0	4.25299757	0.863	0.209	0
Cd8b1	0	5.04481804	0.679	0.027	0
Ms4a4b	0	2.28413909	0.751	0.209	0
Cd3d	0	2.00213052	0.814	0.277	0
Sh2d2a	0	2.18594769	0.791	0.261	0
Trbc2	0	1.97069946	0.874	0.348	0
Cd3g	0	1.7796744	0.783	0.279	0
Cd3e	0	1.80763501	0.756	0.256	0
Gramd3	0	2.0401011	0.724	0.23	0
Ptpn22	0	1.59020872	0.812	0.347	0
Hcst	0	1.68728053	0.891	0.436	0
Klrc1	0	2.67101548	0.459	0.075	0
Ctsw	0	2.2817769	0.494	0.117	0
AW112010	0	1.48466545	0.806	0.438	0
Trac	0	1.46134742	0.572	0.205	0
Gimap7	0	1.93410672	0.499	0.156	0
Hopx	0	1.2243985	0.555	0.213	0
Gimap4	0	1.36285742	0.6	0.259	0
Gzmb	0	2.40344859	0.404	0.067	0
Ctla4	0	0.61908604	0.602	0.267	0
Serpinb6b	0	1.31503011	0.539	0.209	0
Ifngr1	0	0.90159757	0.922	0.597	0
Itk	0	0.94395764	0.617	0.295	0
Lck	0	1.62706316	0.498	0.177	0
Cd2	0	1.16258517	0.585	0.265	0
Lat	0	1.33244492	0.525	0.206	0
Pdcd1	0	2.99641096	0.366	0.056	0
Ctla2a	0	1.83680316	0.454	0.146	0
Serpinb9	0	0.92215527	0.549	0.248	0
Gimap3	0	0.99968442	0.589	0.288	0
Cd28	0	0.87374648	0.588	0.289	0
Fasl	0	2.39702777	0.376	0.08	0
Thy1	0	1.20257088	0.52	0.225	0
Kdm2b	0	0.89135642	0.63	0.336	0
Cxcr6	0	1.03083986	0.524	0.239	0
Gm19585	0	1.90398245	0.405	0.122	0
Ubald2	0	1.07440775	0.819	0.539	0
Ltb	0	1.14332942	0.529	0.255	0
Selplg	0	1.25298791	0.665	0.392	0
Themis	0	2.09689008	0.341	0.081	0
Ms4a6b	0	1.43046689	0.493	0.236	0
Cd27	0	1.91687058	0.345	0.089	0
Trbc1	0	1.60422218	0.496	0.245	0
H2-Q7	0	0.87304859	0.858	0.607	0

Spn	0	1.22839224	0.444	0.194	0
Klrd1	0	2.3116235	0.328	0.083	0
Ybx3	0	1.40313866	0.511	0.277	0
Dnajc15	0	1.80362093	0.408	0.177	0
Cd6	0	1.5211809	0.365	0.134	0
Prkch	0	1.54036005	0.364	0.139	0
Ostf1	0	0.89528362	0.735	0.51	0
Akap13	0	0.89781659	0.937	0.74	0
Cst7	0	2.57023546	0.233	0.045	0
Sh2d1a	0	1.9265811	0.245	0.066	0
Cd7	0	2.16482075	0.228	0.058	0
Gzma	0	3.09772531	0.196	0.026	0
Klrc2	0	1.81859368	0.224	0.055	0
Rpa2	0	2.36256269	0.22	0.054	0
Bend4	0	2.62256868	0.196	0.039	0
Gzmk	0	5.21922931	0.136	0.004	0
Mctp2	0	2.3241196	0.165	0.036	0
Eomes	0	3.65791079	0.144	0.015	0
Rgs16	0	4.17007317	0.128	0.009	0
Rpl24	0	0.46923331	0.991	0.945	0
Rps15a	0	0.67874335	0.998	0.955	0
Rpl18	0	0.53982097	0.995	0.958	0
Rps13	0	0.50099592	0.996	0.965	0
Rpl18a	0	0.41591797	0.998	0.968	0
Plcxd2	2.318E-304	1.47561691	0.377	0.153	5.085E-300
Rpl34	6.551E-303	0.37160023	0.997	0.968	1.437E-298
Cd226	1.019E-299	2.26785277	0.171	0.04	2.235E-295
Mbnl1	6.666E-296	0.69729514	0.907	0.755	1.462E-291
Ptprcap	1.645E-291	1.34567754	0.419	0.187	3.609E-287
Wdr95	5.011E-286	4.09617982	0.057	0.004	1.099E-281
Pde4d	5.893E-285	1.40565909	0.364	0.15	1.293E-280
Rnf138	1.042E-284	1.48771926	0.398	0.181	2.286E-280
Nr4a2	1.804E-281	1.41220485	0.42	0.197	3.958E-277
Rpl19	1.394E-278	0.35675827	0.998	0.968	3.058E-274
Rps3	1.237E-277	0.38666593	0.994	0.955	2.714E-273
Rps10	2.4E-273	0.35301925	0.996	0.969	5.266E-269
Skap1	2.313E-270	1.10116969	0.45	0.209	5.075E-266
Ncald	1.338E-266	2.36335928	0.135	0.029	2.935E-262
Hmgb2	1.521E-266	0.60162372	0.881	0.709	3.336E-262
Rac2	2.143E-266	0.86368335	0.738	0.567	4.702E-262
Ptpn18	9.036E-264	0.87974108	0.773	0.602	1.982E-259
Prf1	1.049E-263	1.52169498	0.146	0.033	2.301E-259
Fyb	2.597E-261	0.76626486	0.74	0.492	5.697E-257
S100a10	1.279E-260	0.7701721	0.764	0.542	2.807E-256
Gm8369	1.349E-260	0.70104227	0.502	0.237	2.96E-256
Fxyd5	1.162E-259	0.77663043	0.819	0.647	2.549E-255
Zdhhc18	5.491E-258	1.01971996	0.59	0.346	1.205E-253
Lag3	6.442E-258	2.42170846	0.143	0.033	1.413E-253

Il21r	4.006E-255	1.16903406	0.538	0.305	8.788E-251
Gtf2i	3.249E-253	1.49303207	0.332	0.139	7.129E-249
Abcb1b	3.388E-252	1.57775289	0.33	0.14	7.434E-248
Casp3	2.362E-250	1.76698672	0.262	0.097	5.183E-246
Ubb	2.777E-250	0.56868586	0.925	0.813	6.091E-246
Rpl36	6.721E-249	0.36158089	0.995	0.956	1.475E-244
Ets1	1.056E-247	0.55242493	0.939	0.645	2.316E-243
Cd160	2.453E-246	2.58057758	0.102	0.018	5.382E-242
Gimap5	3.329E-245	1.06350036	0.441	0.211	7.304E-241
Runx2	3.359E-244	1.40722136	0.345	0.153	7.37E-240
Sept1	1.012E-243	0.98903491	0.534	0.291	2.22E-239
Sla2	4.621E-243	2.1969687	0.142	0.033	1.014E-238
Crtam	1.011E-239	3.14416453	0.059	0.006	2.217E-235
Rpl11	5.229E-237	0.38199631	0.985	0.943	1.147E-232
Lgals1	4.476E-236	1.07980662	0.688	0.5	9.819E-232
Rpsa	1.087E-234	0.38714189	0.997	0.963	2.384E-230
Klk8	8.015E-233	1.59622274	0.244	0.087	1.758E-228
Epsti1	6.093E-232	1.26559346	0.497	0.296	1.337E-227
Tmsb10	2.159E-229	0.44871036	0.983	0.885	4.737E-225
Rinl	2.042E-228	1.2417294	0.35	0.155	4.48E-224
Ptprc	1.141E-227	0.48358975	0.959	0.889	2.504E-223
Uqcrh	1.043E-226	0.52995635	0.929	0.856	2.288E-222
Gm30054	1.539E-226	2.26187181	0.113	0.023	3.377E-222
Rps14	1.003E-223	0.29974418	0.999	0.975	2.201E-219
Abcb1a	2.652E-222	1.22073094	0.356	0.162	5.819E-218
Limd2	1.882E-221	1.02718142	0.521	0.302	4.13E-217
Tnfaip3	4.944E-221	0.51612004	0.893	0.654	1.085E-216
Zfp36l2	3.179E-220	0.98614764	0.667	0.46	6.974E-216
Ubl5	2.803E-217	0.67226913	0.819	0.684	6.15E-213
Crot	4.705E-216	1.40779336	0.285	0.117	1.032E-211
Rpl32	9.209E-216	0.34913987	0.993	0.956	2.02E-211
Atxn1	9.127E-215	1.14868057	0.377	0.18	2.002E-210
Gldc	1.214E-212	3.61428108	0.046	0.004	2.662E-208
Sult2b1	3.558E-212	2.6358852	0.093	0.017	7.805E-208
Tnfrsf18	3.188E-210	0.88462888	0.463	0.241	6.995E-206
H2-K1	7.414E-207	0.40579487	0.98	0.909	1.626E-202
Rpl7a	8.635E-207	0.43921141	0.963	0.896	1.894E-202
Pglyrp1	4.725E-204	1.22570427	0.191	0.062	1.037E-199
Rps18	4.403E-203	0.37089513	0.99	0.934	9.659E-199
Vps37b	8.828E-203	0.48936855	0.884	0.647	1.937E-198
Rpl27a	1.197E-200	0.30071517	0.998	0.971	2.626E-196
Icos	1.817E-200	0.43062847	0.621	0.336	3.987E-196
Itga1	2.309E-200	2.19505961	0.163	0.051	5.067E-196
Gm44175	1.135E-197	4.17906222	0.032	0.002	2.49E-193
Bcl11b	7.493E-197	1.02215551	0.34	0.154	1.644E-192
Ifng	1.347E-196	1.20169643	0.157	0.046	2.955E-192
Stat3	2.119E-195	0.834314	0.73	0.599	4.648E-191
Vgll4	7.183E-195	0.90710212	0.521	0.304	1.576E-190

Tnfrsf9	9.063E-195	0.99013753	0.292	0.125	1.988E-190
Rps11	2.224E-194	0.33412752	0.993	0.957	4.879E-190
Supt4a	2.255E-194	0.8935621	0.619	0.441	4.948E-190
Gimap1	2.457E-194	0.83925179	0.486	0.261	5.391E-190
Cd247	1.62E-193	1.17358208	0.282	0.118	3.553E-189
Igf2r	9.674E-191	1.3803146	0.247	0.099	2.122E-186
Gm47015	1.539E-185	1.93671649	0.129	0.034	3.376E-181
Fyn	2.483E-185	0.99872461	0.525	0.336	5.448E-181
Gnas	5.056E-184	0.63202932	0.821	0.697	1.109E-179
Cblb	3.012E-183	0.93183625	0.484	0.28	6.607E-179
Serbp1	3.875E-182	0.55338134	0.847	0.741	8.502E-178
Ndufa13	4.457E-182	0.80879783	0.663	0.514	9.779E-178
Chst12	2.896E-181	2.13928194	0.13	0.036	6.354E-177
Rnf125	5.352E-181	1.00897926	0.381	0.192	1.174E-176
Rpl38	8.15E-179	0.27806249	0.999	0.975	1.788E-174
Gimap6	3.269E-178	0.6887513	0.633	0.396	7.172E-174
Hdgfl3	1.231E-177	2.14060049	0.102	0.024	2.7E-173
Cox8a	4.143E-177	0.50137709	0.887	0.804	9.089E-173
Rpl17	5.343E-173	0.28120758	0.998	0.968	1.172E-168
Dut	3.248E-172	1.36227497	0.239	0.099	7.126E-168
Orai1	1.364E-171	0.8636781	0.511	0.316	2.992E-167
Il2rb	5.579E-171	0.42244022	0.52	0.281	1.224E-166
Rpl9	5.702E-171	0.30457695	0.993	0.958	1.251E-166
Rplp0	1.836E-170	0.3651534	0.978	0.932	4.028E-166
2010111I01Ril	3.559E-168	1.52762681	0.224	0.092	7.809E-164
S1pr4	1.197E-167	1.64684583	0.154	0.05	2.626E-163
Leprotl1	1.563E-167	0.93090307	0.486	0.304	3.429E-163
Rpl27	5.194E-166	0.40887385	0.944	0.877	1.14E-161
Ccdc30	1.051E-164	2.70846933	0.061	0.01	2.307E-160
H2afv	2.957E-164	0.93358226	0.451	0.272	6.487E-160
Gstp3	4.663E-164	1.67215285	0.145	0.046	1.023E-159
Ube2b	1.285E-163	0.66265605	0.71	0.563	2.82E-159
Ppp2r2c	2.912E-163	4.40971661	0.028	0.002	6.389E-159
Grap2	1.131E-161	1.08001432	0.337	0.169	2.481E-157
Cd52	1.599E-161	0.54093838	0.892	0.813	3.509E-157
Dapk2	4.205E-160	2.93858967	0.06	0.01	9.226E-156
H2-D1	5.18E-159	0.34937565	0.974	0.91	1.136E-154
Zap70	1.923E-158	1.15565242	0.276	0.125	4.218E-154
Gpr18	2.978E-157	1.41078322	0.205	0.081	6.534E-153
Ndfip1	1.305E-155	0.93824111	0.448	0.271	2.863E-151
Eif3f	5.37E-154	0.54377006	0.797	0.682	1.178E-149
Eef2	7.048E-153	0.40394338	0.941	0.872	1.546E-148
Map1lc3b	8.764E-152	0.58962717	0.723	0.575	1.923E-147
Rpl39	1.491E-151	0.26478099	0.997	0.967	3.272E-147
Saraf	2.512E-151	0.79365089	0.542	0.368	5.511E-147
Jpt1	8.77E-151	0.9259539	0.531	0.374	1.924E-146
Stat4	1.198E-148	0.72086859	0.559	0.37	2.629E-144
Trav16n	2.051E-148	3.80684877	0.029	0.002	4.5E-144

Prr13	1.493E-147	0.77384088	0.576	0.413	3.276E-143
Tiprl	8.037E-146	1.10637509	0.329	0.174	1.763E-141
Gna15	9.933E-146	1.4467953	0.182	0.071	2.179E-141
Gm20069	1.558E-145	2.16406667	0.082	0.019	3.418E-141
Cir1	7.426E-144	1.02032017	0.384	0.219	1.629E-139
Txk	1.074E-143	0.40399908	0.406	0.212	2.356E-139
Tbx21	1.075E-143	1.80569712	0.118	0.036	2.358E-139
S100a13	3.951E-143	0.83410445	0.535	0.383	8.668E-139
Abracl	5.693E-143	0.76519918	0.615	0.478	1.249E-138
Nmrk1	6.236E-143	0.9998467	0.252	0.114	1.368E-138
Crbn	6.669E-143	1.09084526	0.312	0.16	1.463E-138
Arl6ip1	6.737E-142	0.69948861	0.662	0.519	1.478E-137
D16Ertd472e	1.503E-141	0.6224441	0.564	0.363	3.297E-137
Rwdd1	4.267E-141	0.95266592	0.463	0.304	9.362E-137
Mrps16	5.561E-140	1.14613927	0.365	0.215	1.22E-135
Dgat1	9.525E-140	0.37038725	0.671	0.443	2.09E-135
Psmb1	4.036E-139	0.68294203	0.66	0.534	8.854E-135
Cd5	4.794E-139	0.94932185	0.272	0.128	1.052E-134
Gpr174	5.041E-138	1.15595456	0.199	0.081	1.106E-133
Pde7a	5.65E-138	0.91149671	0.376	0.208	1.24E-133
Ier5l	5.698E-138	1.17733362	0.27	0.131	1.25E-133
Emb	1.525E-137	0.26708763	0.831	0.605	3.346E-133
Oaz1	5.676E-136	0.40166093	0.909	0.827	1.245E-131
Ciapin1	6.471E-133	1.40471583	0.198	0.085	1.42E-128
Pnrc1	6.944E-133	0.44830203	0.853	0.73	1.523E-128
Eif1	1.655E-132	0.26662267	0.991	0.977	3.631E-128
Mat2b	1.904E-132	0.93112635	0.395	0.236	4.177E-128
Atad2	3.905E-131	1.06455963	0.31	0.163	8.568E-127
Plcg1	1.358E-130	1.44891221	0.167	0.065	2.98E-126
Gapdh	1.832E-130	0.35036353	0.863	0.736	4.02E-126
Eif3h	2.001E-130	0.58830204	0.709	0.595	4.389E-126
Gpr68	7.544E-130	1.49141129	0.175	0.071	1.655E-125
Dennd4a	3.157E-129	0.48524442	0.847	0.715	6.926E-125
Elob	3.716E-128	0.58664588	0.696	0.57	8.153E-124
Jakmip1	5.642E-128	0.98358133	0.271	0.132	1.238E-123
H2-T22	9.165E-128	1.02823629	0.372	0.225	2.011E-123
Pdcd4	1.13E-127	0.7661285	0.483	0.308	2.48E-123
Bnip3l	2.354E-127	0.86236748	0.46	0.304	5.165E-123
Rps5	2.293E-126	0.26105554	0.994	0.954	5.031E-122
Rpl13a	2.963E-126	0.60383874	0.694	0.572	6.5E-122
Cables1	5.468E-126	1.39589344	0.141	0.05	1.2E-121
Rab19	8.956E-126	1.88752056	0.099	0.029	1.965E-121
Ppia	4.032E-124	0.32653609	0.961	0.912	8.846E-120
Zeb1	4.272E-124	0.64211758	0.52	0.34	9.372E-120
Rpl12	1.595E-123	0.25357471	0.994	0.944	3.499E-119
Cdkn1b	2.112E-123	0.68281106	0.553	0.387	4.634E-119
Sumo2	2.609E-123	0.61396691	0.655	0.527	5.724E-119
Cwc15	1.2E-122	0.80612617	0.525	0.385	2.632E-118

Zcchc11	2.271E-122	0.55670073	0.703	0.544	4.983E-118
Nrn1	8.694E-122	3.82086463	0.026	0.002	1.907E-117
Gm32772	1.361E-121	5.26460774	0.016	0	2.985E-117
Ablim1	2.37E-121	0.57465678	0.462	0.276	5.199E-117
Rbm3	4.863E-120	0.41368161	0.859	0.784	1.067E-115
Myl12a	2.854E-119	0.62058171	0.665	0.556	6.262E-115
Apobec2	1.001E-118	4.03321524	0.022	0.001	2.197E-114
Cd96	1.679E-118	1.29933845	0.163	0.065	3.684E-114
Ypel3	2.049E-118	0.9634285	0.361	0.216	4.495E-114
Esyt2	8.715E-118	0.87381635	0.396	0.241	1.912E-113
Eef1d	3.012E-117	0.57657522	0.688	0.569	6.607E-113
Camk2n1	1.949E-116	1.2937745	0.194	0.087	4.277E-112
Stk26	3.044E-116	1.15149891	0.199	0.089	6.678E-112
Zc3hav1	4.357E-116	0.64545815	0.587	0.434	9.56E-112
Camk4	6.435E-115	0.91282753	0.263	0.132	1.412E-110
Btf3	1.252E-114	0.39248949	0.876	0.808	2.747E-110
Prkca	9.478E-114	1.3355199	0.155	0.062	2.079E-109
Sipa1l1	1.568E-113	0.75618457	0.462	0.306	3.439E-109
S100a6	1.892E-113	0.36553357	0.68	0.532	4.151E-109
Cxcr3	2.809E-113	1.58242083	0.123	0.043	6.163E-109
Gmfg	1.673E-112	0.50679883	0.667	0.532	3.669E-108
Serp2	2.152E-112	2.83170575	0.037	0.005	4.722E-108
Stk4	1.097E-111	0.61328174	0.55	0.386	2.406E-107
Cbx4	2.47E-110	1.2107685	0.225	0.111	5.42E-106
Dnajb6	1.247E-109	0.53341565	0.694	0.569	2.735E-105
Dgka	1.053E-108	1.09788804	0.216	0.103	2.309E-104
Syf2	2.052E-107	0.71867952	0.538	0.401	4.502E-103
Sub1	3.758E-107	0.3789946	0.885	0.808	8.245E-103
Agfg1	8.117E-107	1.033924	0.306	0.177	1.781E-102
Ptp4a2	2.379E-106	0.44456799	0.814	0.717	5.219E-102
Higd1a	7.056E-106	0.82589028	0.439	0.3	1.548E-101
Tshz1	1.147E-105	1.04275999	0.243	0.124	2.517E-101
Sytl2	6.853E-105	1.2108213	0.158	0.066	1.503E-100
Ccr5	1.159E-104	0.80005331	0.422	0.277	2.544E-100
Sept6	4.011E-104	0.90266313	0.328	0.193	8.8E-100
Myl6	1.362E-103	0.41293344	0.857	0.782	2.987E-99
Tesc	3.378E-103	1.00939544	0.219	0.106	7.412E-99
Hip1r	3.805E-103	0.92239161	0.246	0.125	8.347E-99
Ube2s	1.106E-102	0.60066018	0.587	0.458	2.4271E-98
Smap1	6.155E-102	0.93571145	0.399	0.27	1.3504E-97
Abcb9	8.222E-102	1.78592963	0.083	0.025	1.8039E-97
Lcn4	1.109E-101	2.23639561	0.052	0.011	2.433E-97
Il12rb2	1.447E-101	1.18099736	0.155	0.065	3.1747E-97
Tapbpl	3.349E-101	1.26777627	0.22	0.112	7.3475E-97
Trav16	1.216E-100	4.09305121	0.018	0.001	2.6669E-96
Pmepa1	1.749E-99	0.93355234	0.392	0.259	3.8381E-95
Atp5h	9.066E-99	0.41895721	0.822	0.735	1.989E-94
Naca	5.9904E-96	0.35980656	0.886	0.828	1.3142E-91

Mrpl9	1.0013E-94	1.00697759	0.289	0.171	2.1967E-90
Prdx6	1.7199E-94	0.47560349	0.644	0.506	3.7734E-90
Sri	6.2156E-94	0.7523054	0.489	0.371	1.3637E-89
Ppp1r16b	1.0612E-93	0.52669499	0.475	0.309	2.3281E-89
Slc25a4	1.5862E-93	0.99672501	0.355	0.235	3.4799E-89
Aebp2	1.932E-93	0.3639553	0.681	0.514	4.2386E-89
Cox7c	1.0681E-92	0.34809321	0.882	0.818	2.3433E-88
Mxd4	1.3307E-92	0.92919967	0.293	0.171	2.9193E-88
Vcpkmt	2.3599E-92	1.32269319	0.149	0.065	5.1774E-88
Bex3	2.4846E-92	1.05471035	0.315	0.194	5.4509E-88
Ubash3a	3.9873E-92	1.37127848	0.116	0.044	8.7478E-88
Prkcq	3.1088E-91	1.23035919	0.117	0.044	6.8203E-87
Mkrn1	3.9469E-91	0.65372994	0.528	0.404	8.6592E-87
Trbv29	8.7059E-91	3.82317112	0.018	0.001	1.91E-86
Cript	1.4412E-90	0.71407843	0.47	0.346	3.1618E-86
Birc2	1.4561E-90	0.67585109	0.459	0.321	3.1945E-86
Cenpw	2.1812E-90	1.36106901	0.139	0.059	4.7854E-86
Carnmt1	3.7798E-90	0.92099118	0.246	0.134	8.2925E-86
Mfsd6	4.8989E-90	0.87869509	0.262	0.146	1.0748E-85
Nsd3	9.59E-90	0.61519373	0.533	0.401	2.1039E-85
Etaa1	1.2933E-89	1.38023091	0.133	0.056	2.8375E-85
Emg1	1.6476E-89	0.79254915	0.421	0.296	3.6148E-85
Snrpe	2.7126E-89	0.4777637	0.702	0.605	5.9511E-85
Tmem163	1.0426E-88	1.79552492	0.053	0.012	2.2873E-84
Prkar1a	1.7023E-88	0.45765958	0.695	0.59	3.7346E-84
Tcrg-C2	2.294E-88	2.30537041	0.043	0.009	5.0327E-84
Dnaja1	1.2623E-87	0.31150196	0.689	0.561	2.7693E-83
Inpp1	1.7734E-87	1.1950501	0.159	0.073	3.8906E-83
Prr7	4.8397E-87	0.84665268	0.273	0.155	1.0618E-82
Mier1	5.4613E-87	0.56944044	0.533	0.398	1.1982E-82
Cox5b	6.4745E-87	0.54264308	0.658	0.558	1.4204E-82
Sec62	1.0132E-86	0.58029359	0.63	0.525	2.2228E-82
Clint1	3.5847E-86	0.51896642	0.601	0.473	7.8644E-82
P2ry10	4.2509E-86	0.45655326	0.612	0.449	9.3261E-82
Trgv2	4.5181E-86	2.16561911	0.045	0.01	9.9122E-82
Stk10	4.7334E-86	0.86201516	0.314	0.193	1.0385E-81
Stat5b	5.7601E-86	0.61589608	0.413	0.273	1.2637E-81
Tnrc6b	2.844E-85	0.59229277	0.48	0.338	6.2395E-81
Pfdn5	3.6389E-85	0.36729017	0.843	0.767	7.9834E-81
Serpinb6a	5.5208E-85	0.93742724	0.178	0.086	1.2112E-80
Mcm6	8.1649E-85	1.07011834	0.181	0.088	1.7913E-80
Rnf157	1.2413E-84	0.84703802	0.315	0.195	2.7232E-80
Ifi27	6.5354E-84	0.88527772	0.306	0.189	1.4338E-79
Dennd4c	3.292E-83	1.37480082	0.142	0.064	7.2223E-79
Lpxn	3.1018E-82	0.89213425	0.344	0.227	6.8051E-78
Gpsm3	4.8018E-82	0.88248878	0.333	0.219	1.0535E-77
Adrb1	8.0188E-82	3.79385281	0.019	0.002	1.7592E-77
Itgal	8.2925E-82	0.60290799	0.445	0.31	1.8193E-77

Ccdc12	1.2904E-80	0.75728146	0.432	0.319	2.8311E-76
Tsc22d4	2.6497E-80	0.70130466	0.396	0.271	5.8132E-76
1110008P14R	3.1894E-80	0.95489203	0.26	0.154	6.9972E-76
Cnn2	4.8253E-80	0.79180464	0.382	0.266	1.0586E-75
Polr1d	5.5519E-80	0.55687056	0.582	0.482	1.218E-75
Ehd3	8.7212E-80	1.61287577	0.082	0.028	1.9134E-75
Ugcg	3.6141E-79	0.5855782	0.415	0.284	7.929E-75
Uhrf2	5.3882E-79	0.43933381	0.469	0.336	1.1821E-74
Rnf166	7.1212E-79	1.17335609	0.205	0.111	1.5623E-74
Baiap3	3.2199E-78	2.96356564	0.025	0.003	7.064E-74
Gng2	4.2161E-78	0.73584609	0.423	0.307	9.2498E-74
Tpr	7.5144E-78	0.50031052	0.617	0.506	1.6486E-73
Chd2	7.5467E-78	0.52929456	0.6	0.485	1.6557E-73
Exph5	8.0056E-78	3.68765366	0.013	0.001	1.7563E-73
Plekha5	1.3798E-77	1.39236022	0.131	0.058	3.0272E-73
Dek	1.4989E-77	0.51495032	0.593	0.482	3.2885E-73
Mbd2	4.5264E-77	0.55948907	0.491	0.365	9.9304E-73
Pla2g16	6.8566E-77	0.9168084	0.287	0.18	1.5043E-72
Csnk2b	1.1927E-76	0.78763081	0.395	0.287	2.6167E-72
Nsmaf	1.1928E-76	1.12812975	0.209	0.116	2.6168E-72
Gzmm	1.2773E-76	3.97726046	0.017	0.001	2.8022E-72
Med10	1.3465E-76	1.10801085	0.243	0.145	2.954E-72
Rsbn1l	1.9772E-76	0.57065045	0.483	0.352	4.3377E-72
Tbrg1	2.3344E-76	0.8496699	0.315	0.203	5.1214E-72
Rbl2	2.394E-76	0.8726831	0.241	0.136	5.2522E-72
4833420G17R	4.6281E-76	0.921473	0.262	0.156	1.0154E-71
Rasgef1a	5.1711E-76	2.65199603	0.031	0.005	1.1345E-71
Nipal3	8.3816E-76	1.49753712	0.1	0.039	1.8388E-71
Utrn	3.1428E-75	0.65647098	0.358	0.234	6.8951E-71
Lax1	3.3395E-75	1.50868065	0.087	0.032	7.3266E-71
Prkcz	8.0026E-75	1.63620872	0.07	0.022	1.7557E-70
Madd	9.4447E-75	0.75749176	0.342	0.228	2.0721E-70
Gabpb1	1.9299E-74	0.80175755	0.27	0.162	4.234E-70
Gm29243	5.8255E-74	1.73695839	0.072	0.024	1.278E-69
B4galnt1	6.6584E-74	0.81738582	0.311	0.2	1.4608E-69
Dbf4	7.0525E-74	1.1944789	0.179	0.094	1.5473E-69
Aes	1.2212E-73	0.58677445	0.511	0.399	2.6791E-69
Mt3	1.5571E-73	2.77395361	0.026	0.004	3.4162E-69
Pfn1	1.6506E-73	0.41416455	0.807	0.75	3.6212E-69
Adgrg1	2.7267E-73	2.80134731	0.031	0.006	5.9822E-69
Arpp19	7.9539E-73	0.51458498	0.581	0.474	1.745E-68
Zyg11b	8.1874E-73	1.00330938	0.201	0.109	1.7962E-68
Grap	3.3983E-72	0.79323508	0.224	0.124	7.4554E-68
Fam189b	7.9457E-72	1.35931097	0.097	0.038	1.7432E-67
Tbc1d10c	1.1651E-71	1.10463836	0.136	0.063	2.5561E-67
Osbpl3	4.8945E-71	0.98084211	0.157	0.077	1.0738E-66
Mrps24	7.9082E-71	0.87047986	0.32	0.216	1.735E-66
Ipcef1	1.3042E-70	1.23559146	0.118	0.051	2.8613E-66

Ndufv3	1.5006E-70	0.65064726	0.463	0.36	3.2923E-66
Ppm1j	9.1358E-70	1.9796741	0.05	0.014	2.0043E-65
Ncl	9.9454E-70	0.27339886	0.865	0.799	2.1819E-65
Fam103a1	1.2161E-69	0.87310733	0.287	0.184	2.6681E-65
Pld3	1.3968E-69	0.97933944	0.236	0.141	3.0645E-65
Ppp1cc	1.1656E-68	0.37202646	0.524	0.387	2.5571E-64
Tprgl	1.7214E-68	0.79500121	0.315	0.209	3.7766E-64
Prrc2b	2.9925E-68	0.80124626	0.282	0.177	6.5653E-64
Scand1	3.3098E-68	0.57856056	0.565	0.47	7.2615E-64
Gprin3	1.465E-67	1.50846633	0.069	0.023	3.2141E-63
Rnf19a	2.1272E-67	0.66588856	0.26	0.156	4.6668E-63
Cox4i1	4.3092E-67	0.28950984	0.892	0.845	9.454E-63
Rpl22l1	4.7904E-67	0.31845009	0.81	0.735	1.051E-62
Pex16	5.4915E-67	1.20618037	0.146	0.073	1.2048E-62
Slc3a2	8.6932E-67	0.45376653	0.704	0.607	1.9072E-62
Tma7	1.246E-66	0.45270172	0.652	0.574	2.7335E-62
Mgea5	2.1475E-66	0.60315388	0.45	0.336	4.7114E-62
Gm33104	2.4727E-66	1.25537158	0.101	0.042	5.4249E-62
Mettl23	2.5804E-66	0.76087463	0.325	0.22	5.6612E-62
Epb41	3.9416E-66	0.67158292	0.44	0.331	8.6474E-62
Sh3bgrl3	4.4274E-66	0.3935864	0.783	0.706	9.7132E-62
Sdcbp2	5.9223E-66	1.37352016	0.09	0.036	1.2993E-61
Armc7	6.4977E-66	1.57407804	0.078	0.029	1.4255E-61
H2afj	1.1027E-65	0.49692748	0.591	0.499	2.4192E-61
Itpkb	1.238E-65	0.546543	0.497	0.38	2.7161E-61
Sugt1	1.7444E-65	0.82260605	0.342	0.241	3.8271E-61
Gxylt1	2.3899E-65	0.95719663	0.219	0.128	5.2433E-61
Frg1	2.4248E-65	0.74831843	0.331	0.228	5.3198E-61
Fkbp3	4.9657E-65	0.71189024	0.36	0.252	1.0894E-60
Atp5d	5.2322E-65	0.56422027	0.566	0.484	1.1479E-60
Eif3m	1.0052E-64	0.5597633	0.517	0.423	2.2052E-60
Lmtk3	1.1201E-64	2.46635128	0.034	0.007	2.4573E-60
Frat2	1.2514E-64	1.22903184	0.103	0.044	2.7456E-60
Ndufa11	1.799E-64	0.77651363	0.378	0.282	3.9469E-60
Lfng	5.837E-64	1.15138639	0.174	0.095	1.2806E-59
Ccdc6	5.9486E-64	1.24058281	0.151	0.077	1.3051E-59
Paip2	7.6766E-64	0.51823415	0.526	0.42	1.6842E-59
Eif2s2	7.7784E-64	0.3638538	0.752	0.672	1.7065E-59
Tox	8.1539E-64	0.82081494	0.159	0.08	1.7889E-59
Rhoh	1.2157E-63	0.38676226	0.4	0.268	2.6671E-59
Mxi1	1.2312E-63	0.68192282	0.401	0.293	2.7011E-59
Ech1	2.49E-63	1.24939307	0.174	0.097	5.4629E-59
Smad7	7.2916E-63	0.56381516	0.425	0.308	1.5997E-58
Dnajc9	1.4131E-62	0.88397043	0.195	0.109	3.1001E-58
Jak1	1.6437E-62	0.41310698	0.679	0.588	3.6061E-58
Npm1	4.6019E-62	0.2518354	0.914	0.85	1.0096E-57
Chrne	9.5134E-62	1.6264721	0.068	0.024	2.0871E-57
Nmb	2.6913E-61	1.50277196	0.064	0.022	5.9045E-57

Coro1a	3.7119E-61	0.47391595	0.672	0.605	8.1436E-57
Lsp1	5.5668E-61	0.37414753	0.586	0.457	1.2213E-56
Arhgdib	7.0697E-61	0.4759491	0.657	0.595	1.551E-56
Atp5g2	8.7137E-61	0.35695056	0.756	0.689	1.9117E-56
Srp14	3.5111E-60	0.56557716	0.521	0.433	7.703E-56
Ttc39c	4.1857E-60	1.00363988	0.14	0.07	9.183E-56
Mknk2	4.7314E-60	0.6542662	0.372	0.27	1.038E-55
Ube2i	5.5322E-60	0.50294951	0.542	0.453	1.2137E-55
Tomm7	1.6579E-59	0.36836423	0.722	0.64	3.6373E-55
Trbv12-1	3.883E-59	3.8751478	0.011	0.001	8.519E-55
Psmb8	5.0951E-59	0.47524034	0.535	0.437	1.1178E-54
Ikzf3	5.4641E-59	0.27930458	0.429	0.287	1.1988E-54
Mosmo	1.3324E-58	1.21003376	0.129	0.064	2.9231E-54
Gm26740	1.4249E-58	0.47120526	0.329	0.213	3.126E-54
Tmpo	1.0331E-57	0.74469756	0.26	0.165	2.2665E-53
Il2rg	1.8315E-57	0.41222829	0.574	0.468	4.018E-53
Smc3	2.1592E-57	0.7143118	0.312	0.212	4.7372E-53
Pde3b	4.4821E-57	0.60756588	0.368	0.261	9.8333E-53
Tmem71	4.8137E-57	1.03122455	0.13	0.064	1.0561E-52
Foxo1	7.4364E-57	0.5945356	0.414	0.302	1.6315E-52
6330403K07R	1.4297E-56	2.3371672	0.023	0.004	3.1367E-52
Aplp2	1.5101E-56	0.63883939	0.425	0.326	3.313E-52
4930523C07R	1.9205E-56	0.44169691	0.534	0.417	4.2133E-52
Pttg1	2.1765E-56	0.9859637	0.202	0.121	4.775E-52
Klf13	2.4441E-56	0.64511458	0.405	0.305	5.3621E-52
5430416N02R	2.6085E-56	1.2440908	0.151	0.082	5.7229E-52
Edf1	5.7129E-56	0.56433093	0.479	0.397	1.2533E-51
4930453N24R	5.8706E-56	0.99518187	0.164	0.091	1.2879E-51
Gm14029	7.0258E-56	1.78867837	0.045	0.013	1.5414E-51
Pym1	7.7635E-56	1.10580696	0.152	0.082	1.7032E-51
Nck1	1.2723E-55	0.95048372	0.227	0.143	2.7914E-51
Ubxn11	3.312E-55	1.39496592	0.07	0.027	7.2663E-51
Fundc2	3.8615E-55	0.80784136	0.298	0.209	8.4718E-51
Lamb3	4.4003E-55	0.94483525	0.095	0.041	9.6538E-51
Dctn4	5.3168E-55	0.59715005	0.423	0.326	1.1665E-50
Arap2	1.243E-54	0.31038463	0.532	0.395	2.7271E-50
Acsbg1	2.4063E-54	1.22059543	0.085	0.036	5.2791E-50
Adora2a	6.4401E-54	0.74394223	0.249	0.16	1.4129E-49
Prrt1	1.1301E-53	1.14152007	0.097	0.043	2.4794E-49
Unc5cl	1.1692E-53	2.57563427	0.022	0.004	2.565E-49
Slc25a40	1.5021E-53	1.21152625	0.087	0.037	3.2954E-49
Fryl	2.1303E-53	0.61573472	0.317	0.216	4.6736E-49
Sec11a	2.172E-53	0.74427602	0.338	0.249	4.7652E-49
Dnajc8	3.404E-53	0.52250094	0.49	0.397	7.468E-49
Vps54	5.8011E-53	0.71331335	0.212	0.128	1.2727E-48
Cenpa	7.4707E-53	0.45433918	0.491	0.387	1.639E-48
Zdhhc2	9.2937E-53	1.20572621	0.088	0.038	2.0389E-48
Nktr	1.2876E-52	0.34636538	0.657	0.576	2.8248E-48

Bcas2	1.5501E-52	0.65983067	0.345	0.252	3.4007E-48
Pdcd2	2.4858E-52	1.18811594	0.125	0.064	5.4536E-48
AU020206	3.3575E-52	0.50531341	0.367	0.259	7.3659E-48
Sesn3	3.7492E-52	0.52772755	0.316	0.213	8.2253E-48
Sdf4	3.8047E-52	0.54351256	0.456	0.363	8.3471E-48
Myl12b	1.3043E-51	0.36936158	0.623	0.544	2.8615E-47
Rundc3b	1.8268E-51	1.12659804	0.116	0.058	4.0078E-47
Dock9	2.2796E-51	1.7487796	0.048	0.015	5.0012E-47
Fam169b	5.1746E-51	0.59806086	0.164	0.089	1.1353E-46
Gimap9	5.2711E-51	0.58996716	0.217	0.13	1.1564E-46
Cab39	5.2873E-51	0.63010079	0.373	0.28	1.16E-46
Apol7e	6.937E-51	1.64560176	0.053	0.018	1.5219E-46
Mphosph6	1.0064E-50	1.0239714	0.165	0.095	2.208E-46
Faah	1.7257E-50	0.64559293	0.178	0.101	3.7861E-46
Nlk	3.5955E-50	1.12744309	0.118	0.06	7.8882E-46
Sys1	5.2329E-50	0.6207821	0.405	0.319	1.1481E-45
Dynll1	5.7938E-50	0.52417703	0.47	0.382	1.2711E-45
Gabarapl2	5.9984E-50	0.54866912	0.434	0.347	1.316E-45
Traf4	7.9343E-50	0.88201345	0.157	0.088	1.7407E-45
Sh3kbp1	1.0289E-49	0.58355505	0.388	0.293	2.2574E-45
Gars	2.1242E-49	0.9519628	0.209	0.132	4.6603E-45
Snapc5	3.0442E-49	1.0328811	0.186	0.115	6.6787E-45
Bicdl1	4.3084E-49	1.64005562	0.047	0.016	9.4522E-45
Tigit	5.603E-49	1.38453381	0.059	0.022	1.2292E-44
Sorbs1	7.5943E-49	1.78142915	0.049	0.017	1.6661E-44
Tstd3	1.305E-48	1.4378595	0.071	0.029	2.863E-44
Gm2682	1.4999E-48	1.29133716	0.063	0.024	3.2906E-44
Stk32c	1.5289E-48	1.66205844	0.053	0.019	3.3543E-44
Mrpl18	2.6729E-48	0.79894352	0.243	0.16	5.8641E-44
Xlr4b	3.0731E-48	0.94620693	0.115	0.058	6.7421E-44
Aak1	4.6442E-48	0.77748158	0.202	0.123	1.0189E-43
Ndufb7	5.5203E-48	0.6074953	0.445	0.372	1.2111E-43
Slamf1	6.2312E-48	0.50827676	0.229	0.142	1.3671E-43
Zfp266	6.9892E-48	0.84178801	0.153	0.085	1.5334E-43
Grb7	9.7578E-48	1.24695868	0.065	0.026	2.1408E-43
Chic2	1.0325E-47	0.61852459	0.363	0.276	2.2652E-43
Rpp25l	1.142E-47	1.01593353	0.162	0.095	2.5053E-43
Stk39	2.0455E-47	1.07267883	0.1	0.048	4.4877E-43
Rpl29	2.4701E-47	0.25365792	0.905	0.853	5.4191E-43
Trbv19	3.6739E-47	2.75294529	0.016	0.002	8.0602E-43
D8Ertd738e	4.0233E-47	0.35176424	0.619	0.538	8.8267E-43
Rgs3	8.5311E-47	1.42830856	0.07	0.029	1.8716E-42
Riok1	8.9275E-47	0.53358104	0.408	0.308	1.9586E-42
B4galt1	1.3912E-46	0.58927686	0.329	0.236	3.0523E-42
Ncoa2	1.5887E-46	0.63263932	0.269	0.182	3.4855E-42
Rab8b	1.6433E-46	0.26323885	0.685	0.58	3.6053E-42
Peli1	1.9952E-46	0.28879138	0.571	0.463	4.3773E-42
Thap3	3.6836E-46	1.03593993	0.113	0.058	8.0814E-42

Lsm1	5.0176E-46	1.07175643	0.134	0.074	1.1008E-41
Mrpl57	9.2963E-46	0.74201711	0.295	0.213	2.0395E-41
Cysltr2	9.6512E-46	0.85594276	0.14	0.076	2.1174E-41
Pik3r1	1.3317E-45	0.45155919	0.457	0.359	2.9216E-41
Cox14	1.5074E-45	0.59697727	0.446	0.368	3.307E-41
Gm12840	1.7612E-45	0.26218321	0.321	0.221	3.8638E-41
Atp5j	2.1973E-45	0.47708928	0.526	0.458	4.8207E-41
Rtraf	2.2147E-45	0.56061724	0.451	0.375	4.8588E-41
Galnt3	2.4434E-45	2.18757166	0.03	0.008	5.3605E-41
Cox7b	2.6835E-45	0.40676339	0.588	0.508	5.8873E-41
Nol7	3.9692E-45	0.4897336	0.495	0.417	8.7081E-41
Myh9	4.373E-45	0.37665223	0.639	0.578	9.594E-41
Inpp4b	4.8437E-45	0.4938835	0.225	0.141	1.0627E-40
Pkp3	4.9917E-45	0.91073149	0.146	0.082	1.0951E-40
Kmt2a	1.0506E-44	0.39123324	0.427	0.321	2.3049E-40
Fam26f	1.0952E-44	1.04151697	0.103	0.052	2.4028E-40
Mrpl33	2.6484E-44	0.43069998	0.509	0.434	5.8103E-40
Krtcap2	2.8813E-44	0.50749894	0.475	0.398	6.3213E-40
Cdc42se1	3.109E-44	0.75722868	0.271	0.192	6.8209E-40
Rcn1	5.4462E-44	1.49930049	0.046	0.016	1.1948E-39
Tomm5	1.0368E-43	0.79192088	0.273	0.195	2.2746E-39
Celf2	1.2349E-43	0.30446096	0.627	0.538	2.7093E-39
Csf1	4.659E-43	0.62839185	0.076	0.034	1.0221E-38
Mri1	8.267E-43	1.04282775	0.131	0.073	1.8137E-38
Rassf5	1.4866E-42	0.7384075	0.208	0.133	3.2614E-38
Surf1	1.5465E-42	1.07131953	0.145	0.085	3.3929E-38
Ddx24	1.6181E-42	0.39921813	0.578	0.502	3.55E-38
Fam71b	1.6349E-42	3.21545504	0.01	0.001	3.5868E-38
Npat	2.2611E-42	1.10330092	0.098	0.049	4.9607E-38
Mllt3	2.3201E-42	0.72524038	0.178	0.108	5.09E-38
Hscb	2.3711E-42	1.13414268	0.135	0.077	5.2019E-38
Arhgap45	3.3532E-42	0.50954963	0.36	0.268	7.3566E-38
Pdcd5	5.5806E-42	0.60376898	0.376	0.299	1.2243E-37
Gm17745	5.9106E-42	1.2070534	0.064	0.027	1.2967E-37
Faim	8.0103E-42	0.72224048	0.189	0.119	1.7574E-37
Polr3k	9.0245E-42	1.10170039	0.114	0.061	1.9799E-37
1700097N02R	1.3188E-41	1.61308402	0.039	0.012	2.8932E-37
Rfxap	1.7109E-41	0.93424199	0.157	0.095	3.7536E-37
Zc3h15	1.733E-41	0.47518461	0.457	0.379	3.8019E-37
Serpina3g	2.0016E-41	1.0018091	0.137	0.078	4.3912E-37
Srp19	2.0275E-41	0.66615293	0.319	0.24	4.4482E-37
Sytl3	2.1045E-41	0.30317186	0.208	0.128	4.617E-37
Ddx46	2.1712E-41	0.56625521	0.379	0.298	4.7634E-37
Maf1	2.2139E-41	0.80903098	0.209	0.137	4.8572E-37
Psmb3	2.8835E-41	0.61336053	0.405	0.334	6.326E-37
Eva1b	3.1783E-41	1.14480361	0.15	0.09	6.9729E-37
Crmp1	3.6207E-41	1.1686588	0.056	0.022	7.9435E-37
Slc2a3	4.4728E-41	0.6679579	0.106	0.054	9.8128E-37

Sars	5.3103E-41	0.58416086	0.321	0.238	1.165E-36
4921524J17Ril	8.0035E-41	0.78273099	0.167	0.101	1.7559E-36
Snrpg	1.059E-40	0.34152942	0.646	0.583	2.3233E-36
Nbeal2	1.0813E-40	1.58073682	0.061	0.025	2.3723E-36
Hint1	1.3992E-40	0.34778954	0.638	0.57	3.0697E-36
Ubr2	1.5052E-40	0.76104833	0.241	0.165	3.3022E-36
Anxa6	1.7681E-40	0.62223723	0.329	0.249	3.8791E-36
Suox	1.8931E-40	1.93288532	0.037	0.012	4.1532E-36
Cntrl	1.9197E-40	0.72001686	0.232	0.157	4.2117E-36
Irf2	2.3446E-40	0.54103993	0.341	0.255	5.1437E-36
Il18r1	2.4242E-40	0.25695665	0.297	0.2	5.3184E-36
Prpf18	2.4597E-40	0.73690666	0.201	0.131	5.3963E-36
Atp5c1	3.484E-40	0.453573	0.514	0.446	7.6436E-36
Chsy1	3.5948E-40	1.10684861	0.114	0.062	7.8865E-36
Nt5c	3.6421E-40	0.82106607	0.244	0.173	7.9905E-36
Nrgn	3.6783E-40	1.34940673	0.079	0.036	8.0698E-36
Cox6c	4.4635E-40	0.29083852	0.766	0.702	9.7924E-36
Pacsin1	7.1825E-40	1.52479875	0.035	0.011	1.5758E-35
Gpr160	8.6551E-40	2.03336567	0.034	0.011	1.8988E-35
Slc25a3	1.5737E-39	0.27648798	0.83	0.772	3.4526E-35
Gnptab	1.746E-39	1.27149743	0.108	0.059	3.8306E-35
Ppp1r12a	1.7772E-39	0.4786403	0.432	0.349	3.8989E-35
Mrpl32	3.4866E-39	0.63308721	0.301	0.225	7.6492E-35
H2-Q4	3.7201E-39	0.57833416	0.334	0.251	8.1614E-35
Nedd9	3.7753E-39	0.52991833	0.211	0.136	8.2827E-35
Mkrn2	4.1874E-39	1.12092129	0.088	0.043	9.1866E-35
Nlrc5	5.6594E-39	0.96578393	0.139	0.082	1.2416E-34
Phgdh	5.7069E-39	0.96452319	0.098	0.05	1.252E-34
Ankrd11	6.2707E-39	0.36391563	0.654	0.594	1.3757E-34
Npm3	7.7314E-39	0.72456365	0.249	0.176	1.6962E-34
Psme1	8.1302E-39	0.28469759	0.685	0.603	1.7837E-34
Ogt	9.72E-39	0.42455921	0.403	0.31	2.1325E-34
Snrpd2	1.5386E-38	0.48707772	0.452	0.384	3.3756E-34
Atp5j2	1.5428E-38	0.39929031	0.591	0.533	3.3847E-34
Atp2b4	2.318E-38	0.60934981	0.27	0.192	5.0855E-34
Sf3b6	2.895E-38	0.42805317	0.48	0.409	6.3513E-34
Ubac2	2.9184E-38	0.96249238	0.174	0.112	6.4027E-34
Ntan1	3.3179E-38	0.81629201	0.21	0.143	7.2791E-34
Gm37387	3.3387E-38	1.84859047	0.026	0.007	7.3248E-34
A930024E05R	3.553E-38	0.87458728	0.099	0.051	7.7948E-34
Mdp1	3.7353E-38	0.87035708	0.172	0.109	8.1948E-34
2510002D24R	3.985E-38	0.78949829	0.131	0.075	8.7427E-34
Xrn2	4.1112E-38	0.58338558	0.381	0.304	9.0197E-34
1810037I17Ril	4.6131E-38	0.40470502	0.527	0.455	1.0121E-33
Tmem234	5.6543E-38	0.46796635	0.486	0.42	1.2405E-33
Mrpl43	6.574E-38	0.73644204	0.234	0.164	1.4423E-33
Kdm5a	8.0588E-38	0.44191136	0.397	0.311	1.768E-33
Idh3a	1.2314E-37	0.96238339	0.151	0.092	2.7015E-33

Rabggtb	1.303E-37	0.79490268	0.2	0.134	2.8588E-33
Ppic	1.3933E-37	1.74012396	0.029	0.008	3.0567E-33
Ppib	1.7499E-37	0.3977397	0.582	0.522	3.8392E-33
Tacc1	1.7577E-37	0.53179766	0.41	0.336	3.8562E-33
Cmip	1.9777E-37	0.60290826	0.349	0.276	4.3388E-33
Fancf	2.1149E-37	1.2287295	0.065	0.029	4.6399E-33
Rab8a	2.6731E-37	0.61864486	0.321	0.247	5.8644E-33
AC147041.1	4.622E-37	2.03193425	0.023	0.006	1.014E-32
Btbd11	5.9249E-37	1.60329962	0.041	0.014	1.2999E-32
Lsm8	8.2534E-37	0.69587669	0.245	0.172	1.8107E-32
Ccnc	9.9545E-37	1.11164597	0.105	0.057	2.1839E-32
H60b	1.0091E-36	1.38907481	0.042	0.015	2.2139E-32
Arid5a	1.221E-36	0.50346888	0.346	0.266	2.6788E-32
Hp1bp3	1.2778E-36	0.6142283	0.335	0.261	2.8033E-32
Ahi1	1.339E-36	0.76286924	0.13	0.075	2.9376E-32
Spata13	1.7214E-36	0.53480364	0.191	0.123	3.7767E-32
Cr1l	2.5824E-36	0.66866489	0.25	0.179	5.6656E-32
Itpk1	4.3359E-36	0.98479315	0.123	0.071	9.5124E-32
Slc20a1	7.3182E-36	0.63631514	0.244	0.171	1.6055E-31
Ndfip2	1.0688E-35	0.74419188	0.244	0.175	2.3448E-31
1500009L16Ri	1.3059E-35	1.79531321	0.027	0.008	2.865E-31
Fip1l1	1.649E-35	0.51047653	0.36	0.283	3.6178E-31
Gpr171	2.2693E-35	0.53865525	0.311	0.234	4.9785E-31
Cox6b1	2.4723E-35	0.29194861	0.678	0.621	5.424E-31
Rac1	3.5307E-35	0.3310667	0.589	0.526	7.746E-31
Actn2	3.6546E-35	0.32358187	0.203	0.129	8.0178E-31
Acap1	4.2382E-35	0.61930519	0.16	0.098	9.2981E-31
Fam58b	4.2769E-35	1.27406338	0.064	0.029	9.383E-31
Bcl2l1	4.4271E-35	0.66356449	0.174	0.111	9.7125E-31
Taf10	5.8527E-35	0.46375641	0.454	0.39	1.284E-30
Zfp260	6.4529E-35	0.9966587	0.113	0.063	1.4157E-30
Brd9	8.3503E-35	0.72707069	0.231	0.163	1.832E-30
Ndufb11	9.3725E-35	0.38461475	0.516	0.451	2.0562E-30
Gadd45gip1	1.3456E-34	0.77603597	0.206	0.141	2.9521E-30
Tnfsf10	3.1908E-34	1.32039297	0.045	0.017	7.0003E-30
Adh5	3.3512E-34	0.88158145	0.191	0.131	7.3522E-30
Gm17018	3.5072E-34	1.06121268	0.108	0.061	7.6944E-30
Khdrbs1	4.8439E-34	0.35540864	0.507	0.436	1.0627E-29
Ift20	5.1272E-34	0.80457313	0.219	0.156	1.1249E-29
0610012G03R	5.4128E-34	0.87336345	0.193	0.132	1.1875E-29
Snu13	5.9361E-34	0.42369364	0.46	0.392	1.3023E-29
Cnot6l	6.1046E-34	0.42000322	0.464	0.384	1.3393E-29
Nudt21	6.5374E-34	0.66281187	0.277	0.21	1.4342E-29
Rad21	8.0096E-34	0.48853238	0.337	0.259	1.7572E-29
S100a4	8.4679E-34	0.25591889	0.45	0.353	1.8578E-29
Fis1	9.8146E-34	0.45828504	0.436	0.37	2.1532E-29
Ndufs4	1.0768E-33	0.66408779	0.293	0.226	2.3623E-29
Tcf7	1.262E-33	0.38368867	0.25	0.173	2.7686E-29

Gm28935	1.4928E-33	1.32450517	0.035	0.012	3.275E-29
Hopxos	1.7176E-33	2.29960077	0.015	0.003	3.7683E-29
Fam92a	1.9956E-33	1.06499031	0.078	0.039	4.3782E-29
Ube2c	2.2961E-33	0.90801029	0.06	0.027	5.0375E-29
Axin2	2.6122E-33	1.17869473	0.069	0.033	5.7308E-29
Trir	2.7113E-33	0.46171903	0.416	0.348	5.9484E-29
Gch1	2.9957E-33	0.5627206	0.337	0.265	6.5723E-29
Uqcc3	3.152E-33	0.68525833	0.212	0.147	6.9151E-29
Ccdc59	3.707E-33	0.58132262	0.346	0.277	8.1327E-29
Selenoi	3.9134E-33	0.98290553	0.109	0.062	8.5855E-29
Perp	4.7473E-33	1.71879858	0.027	0.008	1.0415E-28
Panx1	6.8833E-33	0.98174192	0.107	0.061	1.5101E-28
Brd1	6.9682E-33	0.53027388	0.354	0.282	1.5287E-28
Dcun1d5	7.4386E-33	0.53281698	0.344	0.274	1.632E-28
1700020I14Ril	8.7125E-33	0.55409308	0.271	0.198	1.9114E-28
Ppp1ca	8.9434E-33	0.45748942	0.487	0.432	1.9621E-28
Psmb9	1.0809E-32	0.41466282	0.384	0.31	2.3713E-28
Zadh2	1.2773E-32	1.07904625	0.078	0.039	2.8023E-28
Fam117b	1.4647E-32	0.57458231	0.325	0.255	3.2134E-28
Cd82	1.8478E-32	0.35854592	0.436	0.359	4.0538E-28
Eif3e	1.8634E-32	0.31644571	0.627	0.57	4.0881E-28
Nelfb	2.1978E-32	0.88299722	0.131	0.08	4.8218E-28
Gatad2a	3.1123E-32	0.63671722	0.249	0.182	6.828E-28
Gnptg	3.1507E-32	0.96535886	0.104	0.058	6.9124E-28
Gmnn	3.3593E-32	0.88886551	0.101	0.056	7.3699E-28
Padi2	3.3992E-32	1.05584856	0.089	0.047	7.4576E-28
Psma2	4.3654E-32	0.44505944	0.466	0.41	9.5772E-28
4932438A13R	5.4517E-32	0.35680139	0.462	0.381	1.196E-27
Celsr1	6.5497E-32	1.56007211	0.034	0.012	1.4369E-27
Sms	8.0602E-32	0.55271462	0.283	0.212	1.7683E-27
Gnpda2	8.7744E-32	0.8547653	0.104	0.058	1.925E-27
Pitpnc1	8.9126E-32	0.57069974	0.232	0.163	1.9553E-27
Arhgap9	1.0797E-31	0.86348186	0.148	0.094	2.3687E-27
Cox5a	1.1392E-31	0.31499823	0.574	0.509	2.4993E-27
Cggbp1	1.5371E-31	0.43938036	0.437	0.372	3.3723E-27
Sap18	2.1891E-31	0.62900439	0.275	0.21	4.8027E-27
Fdx1	2.5163E-31	0.59453658	0.176	0.116	5.5206E-27
Esyt1	2.9837E-31	0.68248	0.238	0.173	6.546E-27
Cdc25b	3.1881E-31	1.11801538	0.054	0.024	6.9943E-27
Arf5	3.2844E-31	0.38461414	0.529	0.478	7.2056E-27
Fam96b	3.4897E-31	0.66222982	0.27	0.205	7.656E-27
Sin3b	3.8053E-31	0.50536765	0.388	0.327	8.3484E-27
Gemin7	4.4924E-31	0.7845988	0.166	0.109	9.8559E-27
Ddhd2	4.6407E-31	0.88064158	0.121	0.073	1.0181E-26
Hes6	6.4597E-31	1.32323187	0.057	0.026	1.4172E-26
Sv2c	7.5709E-31	1.0900422	0.044	0.018	1.661E-26
Magohb	8.9546E-31	1.12795288	0.123	0.075	1.9646E-26
Mrpl40	9.2064E-31	1.02012197	0.108	0.063	2.0198E-26

Pdcd6	1.0911E-30	0.57311849	0.328	0.266	2.3938E-26
Itgad	1.3177E-30	3.1246407	0.01	0.001	2.8908E-26
Rtf1	1.6169E-30	0.42067272	0.398	0.326	3.5473E-26
Kmt2e	2.4795E-30	0.32349311	0.494	0.414	5.4398E-26
Wdyhv1	2.5102E-30	1.16970762	0.072	0.037	5.5072E-26
Mrpl58	3.7387E-30	0.82329838	0.171	0.116	8.2023E-26
Mrps23	3.9968E-30	0.72868376	0.191	0.133	8.7685E-26
Lsm5	4.4114E-30	0.50484405	0.363	0.295	9.6782E-26
Park7	5.0316E-30	0.5556254	0.368	0.308	1.1039E-25
Sp100	5.8996E-30	0.36176013	0.329	0.252	1.2943E-25
Timm17a	6.0194E-30	0.65214065	0.227	0.165	1.3206E-25
Cdk17	6.2269E-30	0.61197723	0.201	0.138	1.3661E-25
Atp5b	6.9632E-30	0.31179948	0.624	0.581	1.5277E-25
Tmem9b	8.5959E-30	0.8396727	0.148	0.096	1.8859E-25
Chfr	9.2204E-30	0.6754813	0.196	0.136	2.0229E-25
Ppp2r2d	9.7684E-30	0.65299618	0.275	0.211	2.1431E-25
Atp10a	9.948E-30	0.78239388	0.1	0.057	2.1825E-25
Nab1	1.0956E-29	0.47222281	0.381	0.315	2.4035E-25
Lgalsl	1.2165E-29	1.38854594	0.038	0.015	2.6688E-25
Gm12216	1.3533E-29	1.33815648	0.05	0.022	2.9689E-25
Mob2	1.5565E-29	0.7134197	0.173	0.116	3.4147E-25
Usp48	1.6128E-29	0.79328959	0.143	0.091	3.5384E-25
Tnik	1.9044E-29	0.57845251	0.142	0.088	4.1781E-25
Rpp21	2.9291E-29	0.7032885	0.208	0.148	6.4261E-25
Cbx3	2.9401E-29	0.43572146	0.351	0.279	6.4503E-25
Pstpip1	2.9471E-29	0.87708695	0.17	0.117	6.4655E-25
Prelid1	4.0923E-29	0.54354053	0.358	0.299	8.978E-25
Vps28	4.6282E-29	0.53443771	0.325	0.261	1.0154E-24
Traf3ip3	4.7511E-29	0.69490496	0.115	0.068	1.0423E-24
Rbx1	5.3036E-29	0.38782644	0.499	0.443	1.1636E-24
Pja1	6.0298E-29	0.81145628	0.118	0.071	1.3229E-24
Acadl	6.205E-29	0.74237543	0.214	0.155	1.3613E-24
Itgae	7.7414E-29	1.27832528	0.09	0.051	1.6984E-24
2900026A02R	9.8083E-29	1.78255219	0.042	0.017	2.1519E-24
Arl14ep	9.9643E-29	1.08238094	0.076	0.04	2.1861E-24
Grk6	1.1379E-28	0.56080213	0.19	0.13	2.4965E-24
Ube2a	1.1922E-28	0.60982309	0.295	0.233	2.6155E-24
Adprm	1.2015E-28	1.22584002	0.065	0.033	2.636E-24
Magoh	1.6791E-28	0.54843808	0.3	0.237	3.6838E-24
Atp5g1	1.6941E-28	0.43507159	0.444	0.387	3.7166E-24
Ctdspl2	1.8742E-28	0.67203492	0.19	0.132	4.1118E-24
Atp5o.1	1.9049E-28	0.44735226	0.42	0.363	4.1792E-24
Stat5a	1.9582E-28	0.61397508	0.244	0.181	4.2961E-24
Ndufb9	2.1501E-28	0.42273963	0.435	0.377	4.7171E-24
Gpr55	2.3274E-28	0.88837059	0.121	0.075	5.1061E-24
Hccs	2.391E-28	0.68777958	0.198	0.14	5.2457E-24
Klc3	3.5448E-28	2.03826837	0.018	0.005	7.7769E-24
Il18rap	4.9654E-28	0.28331902	0.236	0.166	1.0894E-23

Nmi	5.0066E-28	0.77223979	0.172	0.118	1.0984E-23
Mrps36	5.2358E-28	0.89903089	0.167	0.115	1.1487E-23
Pot1a	7.0231E-28	0.88721216	0.074	0.039	1.5408E-23
Ttc39b	8.7882E-28	0.51600416	0.263	0.198	1.928E-23
Fli1	9.2066E-28	0.50733385	0.321	0.256	2.0198E-23
Zbtb25	9.81E-28	0.8700636	0.093	0.052	2.1522E-23
Cbx8	1.4456E-27	1.60526312	0.031	0.011	3.1715E-23
Selenoh	1.5157E-27	0.77343958	0.197	0.141	3.3253E-23
Slc9a3r1	1.8703E-27	0.36460485	0.261	0.193	4.1032E-23
Ankrd44	2.1866E-27	0.3960748	0.385	0.314	4.7971E-23
Tnks2	2.4478E-27	0.49515565	0.324	0.258	5.3702E-23
Snw1	2.4673E-27	0.43896233	0.38	0.314	5.4131E-23
Pnn	3.1418E-27	0.43558595	0.316	0.248	6.8928E-23
Gls	3.2045E-27	0.34011797	0.536	0.476	7.0304E-23
Ubn1	3.5714E-27	0.55173845	0.322	0.261	7.8352E-23
Tmem167	5.7078E-27	0.52925277	0.352	0.296	1.2522E-22
Zfp945	5.8114E-27	1.02837154	0.072	0.037	1.275E-22
Dph3	6.3356E-27	0.43631638	0.348	0.285	1.39E-22
Rabif	6.4275E-27	1.02782696	0.085	0.047	1.4101E-22
Rab39b	6.5198E-27	1.25433203	0.034	0.013	1.4304E-22
Nop53	6.7408E-27	0.27850798	0.587	0.536	1.4789E-22
Arl6ip5	7.2694E-27	0.59091536	0.271	0.21	1.5948E-22
Tmem160	9.3615E-27	0.56046433	0.356	0.303	2.0538E-22
Samd3	9.5414E-27	2.05251406	0.019	0.005	2.0933E-22
Casp8ap2	1.0267E-26	0.5509889	0.204	0.144	2.2525E-22
Spsb3	1.2308E-26	0.8378103	0.108	0.065	2.7003E-22
Unc50	1.4986E-26	0.80110682	0.129	0.082	3.2877E-22
Mrps18c	1.802E-26	0.65027555	0.25	0.192	3.9534E-22
Tspyl1	1.8551E-26	0.71124315	0.175	0.121	4.0698E-22
Peak1	2.0104E-26	0.66478763	0.204	0.147	4.4106E-22
Mad2l1bp	2.1101E-26	1.11130256	0.075	0.04	4.6294E-22
Pced1b	2.6712E-26	1.06547516	0.054	0.026	5.8604E-22
Clic1	2.8395E-26	0.3897716	0.491	0.441	6.2297E-22
Borcs8	2.9854E-26	0.8160483	0.177	0.125	6.5496E-22
Cox7a2l	3.4475E-26	0.26431671	0.645	0.594	7.5635E-22
Top2b	3.7016E-26	0.397826	0.364	0.296	8.1209E-22
Gm20139	3.8714E-26	2.23509796	0.016	0.004	8.4935E-22
Puf60	4.1283E-26	0.51757178	0.302	0.24	9.0572E-22
Ralbp1	4.9592E-26	0.530995	0.287	0.228	1.088E-21
Fam78a	5.5902E-26	1.09473486	0.046	0.02	1.2264E-21
Arntl	6.095E-26	0.64258934	0.126	0.079	1.3372E-21
Srp9	7.2875E-26	0.34830653	0.522	0.475	1.5988E-21
Aars	8.1887E-26	0.68079159	0.236	0.179	1.7965E-21
Fam241a	1.0725E-25	1.15364983	0.115	0.073	2.353E-21
Ndufa4	1.0783E-25	0.38529998	0.513	0.466	2.3657E-21
Dtx4	1.0784E-25	1.14615597	0.108	0.067	2.3658E-21
Smarcd1	1.2277E-25	0.81841953	0.113	0.07	2.6935E-21
Sidt2	1.3378E-25	0.6034524	0.188	0.132	2.9351E-21

Churc1	1.4717E-25	0.5590115	0.269	0.21	3.2288E-21
Sap30l	1.6367E-25	0.74977911	0.147	0.098	3.5908E-21
Ap3s1	1.6629E-25	0.56263491	0.267	0.209	3.6482E-21
Fam53c	1.6903E-25	1.02278908	0.097	0.058	3.7084E-21
Prps1	1.9021E-25	0.81677978	0.128	0.082	4.173E-21
Sod1	3.085E-25	0.27847274	0.577	0.518	6.7681E-21
Lin54	5.4196E-25	0.6995513	0.245	0.189	1.189E-20
Dnaja4	5.8073E-25	0.92458375	0.07	0.037	1.2741E-20
Pam16	6.0207E-25	0.6555384	0.197	0.143	1.3209E-20
Gpatch8	6.305E-25	0.50192547	0.292	0.23	1.3832E-20
Med28	6.3876E-25	0.53453187	0.283	0.225	1.4014E-20
Alkbh4	9.6433E-25	1.38936807	0.037	0.016	2.1157E-20
Itga3	9.8657E-25	1.46178752	0.034	0.013	2.1644E-20
Selenow	1.1527E-24	0.30889766	0.566	0.519	2.5288E-20
Ddit3	1.2792E-24	0.60416475	0.279	0.222	2.8063E-20
Fam160b1	1.2959E-24	0.74652207	0.119	0.075	2.8431E-20
Vps13a	1.8384E-24	0.52128063	0.227	0.168	4.0334E-20
Tomm20	1.8536E-24	0.31909406	0.542	0.496	4.0666E-20
Pdlim2	1.9824E-24	0.96395065	0.075	0.042	4.3492E-20
Dctn3	2.004E-24	0.50442624	0.324	0.27	4.3966E-20
Sumo1	2.164E-24	0.37893433	0.431	0.379	4.7477E-20
Dnajc1	2.8648E-24	0.58424828	0.253	0.196	6.2851E-20
Gtdc1	3.6665E-24	0.88837293	0.084	0.048	8.0439E-20
Rab3ip	4.1632E-24	1.00849016	0.072	0.04	9.1337E-20
Kif13b	4.2292E-24	1.01758823	0.086	0.05	9.2784E-20
Zcchc17	4.313E-24	0.734436	0.146	0.099	9.4623E-20
Ss18	5.4361E-24	0.5222286	0.221	0.163	1.1926E-19
Crnkl1	5.6354E-24	0.70435447	0.173	0.123	1.2364E-19
Erg28	6.1589E-24	0.78868387	0.114	0.072	1.3512E-19
Ufc1	6.3246E-24	0.62115379	0.287	0.235	1.3876E-19
Nxt1	6.5492E-24	0.87029991	0.104	0.065	1.4368E-19
Gpatch11	6.8573E-24	0.86381945	0.091	0.054	1.5044E-19
Uqcr10	6.9147E-24	0.35989449	0.502	0.455	1.517E-19
Tigd2	7.2028E-24	0.57123625	0.153	0.103	1.5802E-19
Gm16286	7.6637E-24	0.49688394	0.314	0.256	1.6813E-19
Gm34794	7.9877E-24	2.21102152	0.011	0.002	1.7524E-19
Cmc1	9.2876E-24	0.77396382	0.211	0.16	2.0376E-19
Snai3	9.3291E-24	2.24718061	0.015	0.004	2.0467E-19
Zfp706	1.0048E-23	0.45899959	0.338	0.279	2.2044E-19
Izumo4	1.046E-23	1.24607573	0.039	0.017	2.2949E-19
Zbtb1	1.0519E-23	0.48673461	0.189	0.134	2.3079E-19
Trim59	1.2058E-23	0.37074532	0.156	0.104	2.6453E-19
Lockd	1.4582E-23	0.77829292	0.071	0.038	3.1991E-19
Trmt112	1.5168E-23	0.50844057	0.333	0.28	3.3278E-19
Ssna1	1.5404E-23	0.77312719	0.126	0.083	3.3795E-19
Prkacb	1.5681E-23	0.61049149	0.203	0.149	3.4403E-19
Slain1	1.7498E-23	0.65073637	0.108	0.067	3.8388E-19
Psma7	1.8667E-23	0.28809036	0.571	0.518	4.0953E-19

Gas2	1.8923E-23	1.3203663	0.024	0.008	4.1514E-19
Mdh1	2.1907E-23	0.46661293	0.326	0.269	4.8063E-19
Paip2b	2.5402E-23	0.7144265	0.131	0.086	5.573E-19
Rsl1d1	2.8545E-23	0.55340532	0.258	0.202	6.2624E-19
Eif3k	3.0756E-23	0.27365752	0.618	0.567	6.7476E-19
Capza1	3.2089E-23	0.43579602	0.393	0.343	7.04E-19
Xpa	3.5306E-23	0.80648951	0.151	0.104	7.7457E-19
Def6	4.146E-23	0.887456	0.087	0.051	9.096E-19
Rbm22	4.3434E-23	0.51126229	0.279	0.222	9.5289E-19
Cd48	4.6552E-23	0.75962995	0.185	0.136	1.0213E-18
C2cd4b	5.0796E-23	2.11996657	0.011	0.002	1.1144E-18
Sytl1	5.4655E-23	1.07116612	0.043	0.02	1.1991E-18
Hipk1	5.5245E-23	0.4967831	0.349	0.294	1.212E-18
Ran	7.1312E-23	0.34118207	0.472	0.424	1.5645E-18
Smdt1	7.5989E-23	0.34440124	0.515	0.467	1.6671E-18
Usp3	8.2496E-23	0.58432022	0.235	0.18	1.8099E-18
Psph	8.5714E-23	0.99818156	0.062	0.033	1.8805E-18
Atp8a2	8.7781E-23	1.51583912	0.03	0.012	1.9258E-18
Terf1	9.5661E-23	0.72855949	0.117	0.075	2.0987E-18
Mov10	9.694E-23	1.17613374	0.042	0.019	2.1268E-18
Nlrc3	1.0574E-22	0.72817744	0.073	0.041	2.3199E-18
Abca2	1.0594E-22	0.98321445	0.06	0.032	2.3243E-18
Nfrkb	1.2521E-22	0.69241046	0.12	0.078	2.7469E-18
F8a	1.3713E-22	1.09185196	0.048	0.023	3.0086E-18
Csnk1d	1.375E-22	0.48525542	0.298	0.243	3.0166E-18
Ezh2	1.618E-22	0.86283306	0.133	0.09	3.5497E-18
Pfkp	1.677E-22	0.4824992	0.319	0.263	3.6792E-18
Cirbp	1.8177E-22	0.36243684	0.362	0.297	3.9879E-18
Map4k2	1.8685E-22	0.47040866	0.148	0.099	4.0994E-18
Cds2	1.9769E-22	0.60377081	0.128	0.084	4.3372E-18
Eipr1	2.6356E-22	0.83846597	0.108	0.069	5.7822E-18
Rp9	2.6894E-22	0.4554061	0.322	0.265	5.9003E-18
Herc1	2.8267E-22	0.32304917	0.305	0.241	6.2015E-18
Malsu1	3.0841E-22	0.67744435	0.161	0.114	6.7661E-18
Mrps26	3.6554E-22	0.76035365	0.135	0.091	8.0196E-18
Utf1	3.7204E-22	1.34472325	0.031	0.013	8.1621E-18
Chd1	3.7643E-22	0.48019769	0.296	0.24	8.2584E-18
Fam204a	3.933E-22	0.4543232	0.277	0.219	8.6286E-18
Ndufa8	5.3604E-22	0.56380699	0.256	0.204	1.176E-17
Tsr3	5.5323E-22	1.107666	0.067	0.037	1.2137E-17
Kcnc1	5.5693E-22	1.63606902	0.026	0.01	1.2218E-17
Rif1	6.0795E-22	0.52623823	0.182	0.13	1.3338E-17
Bspry	6.0937E-22	1.71917168	0.02	0.006	1.3369E-17
Ube2h	6.1831E-22	0.47757685	0.263	0.207	1.3565E-17
Il27ra	6.2001E-22	0.6881807	0.09	0.054	1.3602E-17
Kif2a	6.2792E-22	0.69011903	0.17	0.122	1.3776E-17
Aimp1	6.2818E-22	0.50473043	0.349	0.299	1.3782E-17
Arhgef1	6.5254E-22	0.29755433	0.359	0.291	1.4316E-17

Psmb5	7.392E-22	0.52578063	0.309	0.259	1.6217E-17
Dhx36	7.4802E-22	0.44320059	0.293	0.234	1.6411E-17
2810001G20R	8.3017E-22	0.87647929	0.063	0.034	1.8213E-17
Arl2bp	8.4869E-22	0.53399308	0.212	0.158	1.8619E-17
Rab27a	9.685E-22	0.86029184	0.078	0.045	2.1248E-17
Mien1	9.8779E-22	0.46841985	0.308	0.255	2.1671E-17
Eif3c	1.0339E-21	0.28963263	0.569	0.533	2.2682E-17
Fbxo8	1.0631E-21	0.79394087	0.092	0.056	2.3323E-17
Sf3b5	1.1194E-21	0.42569018	0.366	0.315	2.4558E-17
1810041H14R	1.1263E-21	1.17487	0.032	0.013	2.4711E-17
1110012L19Ri	1.1351E-21	0.99823215	0.061	0.033	2.4902E-17
Pop5	1.1384E-21	0.60300809	0.162	0.114	2.4974E-17
Vps26b	1.2859E-21	1.08531244	0.072	0.041	2.8211E-17
Chd4	1.3738E-21	0.32210293	0.498	0.449	3.014E-17
Lzts1	1.3955E-21	1.96198504	0.024	0.009	3.0615E-17
Raly	1.4003E-21	0.39723446	0.39	0.34	3.0721E-17
Atp5g3	1.4335E-21	0.34706059	0.467	0.423	3.1449E-17
Psme2	1.6431E-21	0.26861317	0.491	0.44	3.6049E-17
Chst2	1.7636E-21	1.01073561	0.049	0.024	3.8691E-17
Hdac7	1.9143E-21	0.49826997	0.122	0.079	4.1998E-17
Smarca5	2.1956E-21	0.28371814	0.557	0.515	4.817E-17
Chd3	2.7796E-21	0.38622628	0.349	0.289	6.0982E-17
Ostc	2.9275E-21	0.45122703	0.338	0.287	6.4227E-17
Sit1	2.9915E-21	0.85916593	0.048	0.024	6.5631E-17
Raf1	3.3135E-21	0.53264534	0.205	0.152	7.2694E-17
Ppp1r8	3.3776E-21	0.91921957	0.074	0.042	7.4102E-17
Bud31	4.6268E-21	0.52248509	0.265	0.213	1.0151E-16
Tab2	5.4219E-21	0.25580997	0.473	0.415	1.1895E-16
Phax	5.6897E-21	0.76055493	0.146	0.103	1.2483E-16
Rnf14	6.4302E-21	0.88323383	0.101	0.064	1.4107E-16
Mrpl21	8.2483E-21	0.6053072	0.22	0.17	1.8096E-16
Kif5b	1.1206E-20	0.31389903	0.468	0.422	2.4584E-16
Emsy	1.1797E-20	0.43567726	0.25	0.192	2.5881E-16
Nop56	1.1994E-20	0.49461998	0.256	0.201	2.6314E-16
Upf3a	1.363E-20	0.69444218	0.173	0.127	2.9903E-16
Prex1	1.4596E-20	0.55484267	0.281	0.23	3.2023E-16
Commd2	1.4867E-20	0.782256	0.115	0.076	3.2616E-16
Pcsk7	1.6613E-20	0.79675676	0.099	0.063	3.6447E-16
Adh4	1.6749E-20	1.80616951	0.011	0.002	3.6746E-16
Acin1	1.7293E-20	0.35381194	0.413	0.362	3.7939E-16
Smpd1	1.7621E-20	1.0619282	0.052	0.027	3.866E-16
Snapc3	2.2945E-20	0.87406131	0.07	0.04	5.0338E-16
Ifitm10	2.4444E-20	2.27705321	0.01	0.002	5.3628E-16
Ryk	2.4784E-20	1.28671907	0.04	0.019	5.4375E-16
Slc35b3	2.9344E-20	0.77203301	0.088	0.054	6.4378E-16
Bola2	2.9412E-20	0.38690528	0.449	0.406	6.4527E-16
Ar	3.161E-20	0.28482494	0.152	0.103	6.9348E-16
Dock2	3.2118E-20	0.29009285	0.473	0.418	7.0463E-16

Sike1	3.3146E-20	0.63493412	0.133	0.09	7.2719E-16
Cep97	3.831E-20	0.88515212	0.052	0.027	8.4048E-16
Uba52	3.8727E-20	0.25686589	0.556	0.517	8.4964E-16
Cdc40	4.1478E-20	0.39373556	0.296	0.239	9.0999E-16
Polr2j	4.3031E-20	0.75199717	0.137	0.095	9.4407E-16
G3bp2	4.6942E-20	0.42687349	0.308	0.253	1.0299E-15
Yy1	4.7417E-20	0.41658818	0.369	0.319	1.0403E-15
Phf6	5.2638E-20	0.61805284	0.138	0.095	1.1548E-15
Aurkaip1	5.4581E-20	0.53146583	0.3	0.252	1.1975E-15
Asns	5.6804E-20	0.82015006	0.083	0.05	1.2462E-15
Utp23	6.0015E-20	0.78638431	0.101	0.065	1.3167E-15
Lpin1	7.3837E-20	0.84109829	0.086	0.053	1.6199E-15
Idnk	7.4469E-20	0.51738338	0.225	0.174	1.6338E-15
Ormdl1	7.7802E-20	0.85950597	0.062	0.034	1.7069E-15
Soat2	7.8652E-20	1.81281813	0.022	0.008	1.7255E-15
Diaph1	8.3112E-20	0.28454785	0.507	0.458	1.8234E-15
Zc3h13	8.5351E-20	0.41672435	0.342	0.287	1.8725E-15
Usp1	8.8762E-20	0.6106847	0.168	0.122	1.9473E-15
Cenpq	9.634E-20	0.65244702	0.125	0.085	2.1136E-15
Zfp773	1.0025E-19	1.29899323	0.026	0.01	2.1994E-15
Trp53	1.0427E-19	0.54643961	0.212	0.162	2.2875E-15
Mrpl41	1.1186E-19	0.61429437	0.141	0.099	2.4542E-15
Card11	1.2046E-19	0.68395738	0.118	0.079	2.6428E-15
Slc30a9	1.2596E-19	0.74710228	0.121	0.082	2.7635E-15
Srpk1	1.2933E-19	0.45339394	0.237	0.183	2.8373E-15
Akirin2	1.3051E-19	0.55605614	0.214	0.165	2.8632E-15
Hacd3	1.3517E-19	0.46273993	0.152	0.106	2.9655E-15
Gm2245	1.5181E-19	1.21896971	0.032	0.014	3.3305E-15
Dok2	1.6629E-19	0.83147816	0.121	0.082	3.6483E-15
Supt20	1.682E-19	0.40421428	0.252	0.197	3.6901E-15
Blmh	1.7163E-19	0.58070294	0.168	0.122	3.7653E-15
Esm1	1.7794E-19	1.77737191	0.012	0.003	3.9039E-15
Pin1	1.9882E-19	0.67306201	0.188	0.143	4.3618E-15
Pfdn2	2.1605E-19	0.41675155	0.349	0.3	4.7399E-15
Exosc4	2.2488E-19	0.87357026	0.086	0.053	4.9337E-15
Trbv15	2.2689E-19	2.44033575	0.01	0.002	4.9777E-15
Med11	2.3298E-19	0.68333602	0.151	0.108	5.1113E-15
Sltm	2.423E-19	0.37163621	0.362	0.307	5.3158E-15
Ostm1	2.7605E-19	0.75672963	0.11	0.073	6.0562E-15
Nasp	2.8051E-19	0.49750236	0.239	0.187	6.1542E-15
Gsk3b	3.4971E-19	0.45125785	0.297	0.246	7.6722E-15
Mrpl20	4.4008E-19	0.57701381	0.255	0.21	9.6549E-15
Rab2a	4.4725E-19	0.31524737	0.474	0.434	9.8122E-15
Unc119b	4.5165E-19	0.71112002	0.128	0.088	9.9089E-15
Ndufa6	4.9355E-19	0.2743539	0.538	0.49	1.0828E-14
Hprt	5.0577E-19	0.53645463	0.258	0.211	1.1096E-14
Gm39323	5.4064E-19	1.52233369	0.015	0.005	1.1861E-14
Ikzf1	5.6367E-19	0.37119749	0.273	0.216	1.2366E-14

Kin	6.3466E-19	0.70379136	0.135	0.094	1.3924E-14
Lysmd2	6.6256E-19	0.78680078	0.06	0.033	1.4536E-14
Impa2	7.3424E-19	0.88832085	0.081	0.049	1.6109E-14
Trbv12-2	7.8285E-19	1.53214123	0.017	0.006	1.7175E-14
Rmnd5b	8.3766E-19	0.84111322	0.058	0.032	1.8377E-14
Hip1	8.3833E-19	0.85590993	0.091	0.058	1.8392E-14
Zfp707	1.0213E-18	1.24922065	0.031	0.014	2.2407E-14
Aip	1.0956E-18	0.63164805	0.156	0.112	2.4036E-14
Tmem263	1.1116E-18	0.79353938	0.076	0.045	2.4388E-14
Abtb1	1.1733E-18	0.86310432	0.076	0.046	2.574E-14
Ndufs6	1.2024E-18	0.44982117	0.316	0.27	2.638E-14
Pstk	1.2278E-18	0.79834759	0.061	0.034	2.6937E-14
Snrnp48	1.2566E-18	0.44991501	0.245	0.194	2.7569E-14
Bag1	1.3084E-18	0.41809826	0.332	0.285	2.8706E-14
Adgrg5	1.3135E-18	1.18328931	0.079	0.049	2.8817E-14
Tmem191c	1.3309E-18	1.15288804	0.041	0.02	2.9198E-14
Zfp874b	1.5353E-18	0.8647735	0.06	0.034	3.3684E-14
Tbc1d10b	1.6786E-18	0.84324248	0.106	0.07	3.6827E-14
Slc39a4	1.7318E-18	0.81169066	0.047	0.024	3.7993E-14
Col4a3bp	1.7486E-18	0.41929602	0.21	0.16	3.8363E-14
Nt5c3b	1.799E-18	0.9426689	0.058	0.033	3.9467E-14
Fgfr1op2	1.9958E-18	0.43727685	0.351	0.304	4.3785E-14
Atp8b4	2.3233E-18	0.87352535	0.077	0.047	5.0972E-14
Crebrf	2.4985E-18	0.38836516	0.342	0.288	5.4814E-14
Cyld	2.7557E-18	0.32903042	0.367	0.312	6.0457E-14
Trip11	3.3309E-18	0.44811485	0.273	0.223	7.3076E-14
Kansl1	3.4651E-18	0.32087939	0.357	0.301	7.602E-14
Paxx	3.5664E-18	0.88474867	0.085	0.054	7.8244E-14
Cdadc1	3.8352E-18	0.87561512	0.089	0.057	8.4141E-14
Ptpn11	4.065E-18	0.60318208	0.18	0.136	8.9183E-14
Tspyl4	5.1122E-18	1.12252562	0.027	0.012	1.1216E-13
Ip6k1	5.205E-18	0.4364417	0.211	0.162	1.1419E-13
Ppp3cc	5.4637E-18	0.59042735	0.086	0.053	1.1987E-13
Ifnar1	5.7314E-18	0.36279435	0.396	0.351	1.2574E-13
Imp3	6.3218E-18	0.53475413	0.225	0.179	1.3869E-13
Klhl12	6.5119E-18	0.64376028	0.077	0.047	1.4286E-13
Ddx42	6.6038E-18	0.45187753	0.295	0.245	1.4488E-13
Slbp	8.1381E-18	0.42851463	0.294	0.243	1.7854E-13
R3hdm4	8.4532E-18	0.55712923	0.184	0.14	1.8545E-13
Phf11b	9.1584E-18	0.81642682	0.099	0.065	2.0093E-13
Tmem50a	9.27E-18	0.34251961	0.406	0.361	2.0338E-13
Ing2	9.3159E-18	0.50754624	0.192	0.147	2.0438E-13
Hdhd5	9.3392E-18	1.2912338	0.038	0.018	2.0489E-13
Ndufb8	9.9851E-18	0.41225955	0.358	0.314	2.1906E-13
Psmd4	1.0764E-17	0.37035019	0.361	0.316	2.3616E-13
Klre1	1.2122E-17	0.97559625	0.052	0.028	2.6593E-13
Gpi1	1.2309E-17	0.31298608	0.446	0.406	2.7004E-13
Aldoa	1.2662E-17	0.2598149	0.5	0.458	2.778E-13

Atg12	1.3055E-17	0.55602774	0.201	0.156	2.8642E-13
Ptpn3	1.5185E-17	1.37476024	0.038	0.019	3.3314E-13
Hpf1	1.578E-17	0.48689543	0.234	0.186	3.462E-13
Dnajc18	1.6629E-17	1.15963739	0.045	0.024	3.6482E-13
Zer1	1.6883E-17	0.81203577	0.072	0.043	3.7039E-13
Kcnk7	1.7668E-17	1.49964819	0.017	0.006	3.8762E-13
Zfp329	2.0636E-17	0.69548075	0.08	0.05	4.5273E-13
Ncbp1	2.328E-17	0.70571233	0.11	0.075	5.1074E-13
Etaa1os	2.3878E-17	1.45137647	0.02	0.007	5.2387E-13
Arhgap31	2.462E-17	0.36491337	0.276	0.224	5.4014E-13
Wdr45	2.471E-17	0.870285	0.066	0.039	5.4211E-13
Zfp622	2.5214E-17	0.38388643	0.224	0.176	5.5316E-13
Zgpat	2.6428E-17	0.77556265	0.064	0.038	5.798E-13
Ankrd12	2.7832E-17	0.3237418	0.329	0.272	6.1061E-13
Fbxo21	2.8055E-17	0.88166541	0.058	0.033	6.155E-13
Reep5	3.1322E-17	0.36991814	0.437	0.403	6.8718E-13
Acbd6	3.3297E-17	0.63297277	0.168	0.127	7.305E-13
Exosc8	3.4365E-17	0.5955487	0.149	0.108	7.5394E-13
Cep250	3.4545E-17	0.83753739	0.078	0.048	7.5787E-13
Racgap1	3.6532E-17	1.20246723	0.049	0.027	8.0147E-13
Tmem33	3.7796E-17	0.57313619	0.18	0.137	8.2922E-13
Letm1	3.8711E-17	0.73473544	0.13	0.093	8.4928E-13
Ndufa5	4.3538E-17	0.54804317	0.262	0.218	9.5517E-13
Hmgb3	4.401E-17	0.91643907	0.045	0.023	9.6553E-13
Mms22l	4.9768E-17	1.18747689	0.043	0.022	1.0919E-12
D030056L22R	5.6249E-17	0.80560297	0.076	0.047	1.2341E-12
Arl6ip4	5.7811E-17	0.6829747	0.139	0.101	1.2683E-12
Agpat3	6.2058E-17	0.70219786	0.131	0.093	1.3615E-12
Ttc14	7.6608E-17	0.27364452	0.406	0.35	1.6807E-12
Pcmtd2	7.7183E-17	0.54925757	0.135	0.096	1.6933E-12
Cast	7.8309E-17	0.30452054	0.333	0.281	1.718E-12
Etfdh	7.9227E-17	0.81664932	0.08	0.05	1.7382E-12
Cd2bp2	8.155E-17	0.55343783	0.159	0.117	1.7891E-12
Baiap2	9.4252E-17	0.63228603	0.179	0.137	2.0678E-12
Gm35082	9.4703E-17	0.70696876	0.07	0.042	2.0777E-12
Atg101	1.1603E-16	0.68787694	0.114	0.079	2.5456E-12
Tmem126a	1.1884E-16	0.75958837	0.117	0.082	2.6072E-12
Mepce	1.2165E-16	0.60283149	0.156	0.115	2.6689E-12
Sidt1	1.2272E-16	0.78777784	0.049	0.027	2.6924E-12
Lsm12	1.2307E-16	0.42119036	0.349	0.308	2.7001E-12
Ubxn1	1.3492E-16	0.30341075	0.412	0.368	2.9601E-12
Ebpl	1.4624E-16	0.94232592	0.074	0.046	3.2085E-12
Syt11	1.6138E-16	0.80194572	0.056	0.032	3.5406E-12
Sh2b1	1.6313E-16	0.59396066	0.1	0.066	3.5789E-12
Gm6277	1.656E-16	1.9104203	0.011	0.003	3.6331E-12
Farp1	1.7021E-16	0.92716687	0.029	0.013	3.7343E-12
Nup50	1.8419E-16	0.5535033	0.203	0.16	4.0409E-12
Rbm18	1.8792E-16	0.69172914	0.115	0.079	4.1227E-12

Gm31597	1.949E-16	1.16893409	0.04	0.02	4.2759E-12
Ccnd2	2.1246E-16	0.43683418	0.355	0.305	4.6611E-12
Wasf2	2.2659E-16	0.33699928	0.413	0.371	4.9712E-12
Pdcd10	2.5033E-16	0.35367625	0.359	0.315	5.4921E-12
Tmem240	2.5478E-16	1.94904048	0.012	0.003	5.5896E-12
Itm2a	2.6133E-16	0.81336021	0.034	0.016	5.7332E-12
Trim27	3.0316E-16	0.57529421	0.145	0.106	6.651E-12
Ubr5	3.3178E-16	0.49984013	0.263	0.219	7.2789E-12
Lyrm9	3.5707E-16	0.93274834	0.049	0.027	7.8337E-12
Ezh1	3.6882E-16	0.64097289	0.122	0.085	8.0916E-12
Flt3l	3.7648E-16	0.78099746	0.068	0.041	8.2595E-12
Tomm22	3.8254E-16	0.42495174	0.343	0.304	8.3925E-12
Fmnl1	3.836E-16	0.44240505	0.257	0.212	8.4158E-12
Csnk2a2	4.0231E-16	0.46842139	0.286	0.243	8.8263E-12
Tdg	4.0572E-16	0.67356129	0.145	0.107	8.901E-12
Rchy1	4.1898E-16	0.38486055	0.239	0.192	9.192E-12
1110059G10R	4.2531E-16	0.65716968	0.128	0.092	9.331E-12
St3gal6	4.4389E-16	0.62533577	0.082	0.052	9.7386E-12
Slc35d1	4.9108E-16	0.71651882	0.066	0.04	1.0774E-11
Uqcrc2	5.2266E-16	0.52228341	0.249	0.207	1.1467E-11
Klhl42	6.3006E-16	1.39027848	0.018	0.007	1.3823E-11
Larp7	6.4289E-16	0.53239708	0.204	0.162	1.4104E-11
Sf3b2	7.3161E-16	0.28844789	0.442	0.401	1.6051E-11
Nsmce4a	7.3493E-16	0.41668707	0.215	0.169	1.6124E-11
Dennd1c	7.5611E-16	0.65651	0.09	0.059	1.6588E-11
Ost4	8.7689E-16	0.27235814	0.505	0.475	1.9238E-11
Mis18a	8.9781E-16	0.88259325	0.084	0.054	1.9697E-11
Naa20	9.4739E-16	0.62389751	0.139	0.102	2.0785E-11
Fopnl	9.5005E-16	0.57057825	0.111	0.077	2.0843E-11
Cd101	9.7274E-16	1.04541293	0.04	0.021	2.1341E-11
Cd200	1.0111E-15	0.50675847	0.086	0.056	2.2182E-11
Mgst2	1.03E-15	0.93040143	0.032	0.015	2.2597E-11
Snapc1	1.0732E-15	0.4915329	0.169	0.127	2.3545E-11
Mrpl44	1.1511E-15	0.73586435	0.064	0.039	2.5255E-11
Slc37a3	1.1824E-15	0.79384929	0.064	0.038	2.594E-11
Stim1	1.4914E-15	0.4332482	0.189	0.144	3.272E-11
Haus3	1.5052E-15	0.50417103	0.133	0.095	3.3023E-11
Helz	1.5579E-15	0.43329485	0.166	0.124	3.4179E-11
Isca1	1.6486E-15	0.53339175	0.135	0.097	3.6169E-11
Rab35	1.7146E-15	0.55112223	0.158	0.119	3.7617E-11
Hnrnpd	1.7189E-15	0.26806891	0.497	0.466	3.771E-11
Ociad1	1.7317E-15	0.4201529	0.28	0.236	3.7991E-11
Taf6l	1.7744E-15	0.49829928	0.23	0.188	3.8929E-11
Hist2h2ac	1.9536E-15	0.52658096	0.101	0.068	4.286E-11
Rnf20	2.0511E-15	0.48058513	0.202	0.159	4.4998E-11
Eloc	2.1086E-15	0.30945534	0.414	0.377	4.6261E-11
Spcs2	2.1896E-15	0.2740128	0.469	0.431	4.8037E-11
Acot7	2.1931E-15	0.62403357	0.157	0.119	4.8114E-11

Ciao1	2.2034E-15	0.80538078	0.076	0.049	4.8341E-11
Gm5914	2.2163E-15	1.04763549	0.041	0.021	4.8624E-11
Phtf1	2.2186E-15	0.79990925	0.064	0.039	4.8673E-11
Mllt11	2.2367E-15	1.34082167	0.026	0.011	4.9071E-11
Slc16a1	2.27E-15	0.79014142	0.078	0.05	4.9802E-11
Phf5a	2.3093E-15	0.52565082	0.257	0.217	5.0665E-11
Cdkn2aip	2.4066E-15	0.56689433	0.116	0.08	5.2799E-11
Sbf1	2.4865E-15	0.63800032	0.14	0.103	5.4552E-11
Zfp560	2.595E-15	0.65201509	0.104	0.071	5.6931E-11
Nop9	2.5963E-15	0.86759016	0.098	0.067	5.696E-11
Nudt19	2.6139E-15	0.87342738	0.05	0.028	5.7346E-11
Tmem165	2.6933E-15	0.7053543	0.109	0.076	5.9088E-11
Smpd5	2.7166E-15	1.31083197	0.018	0.007	5.9599E-11
F2r	2.9853E-15	0.4017773	0.102	0.069	6.5495E-11
Agap2	3.0674E-15	0.90300167	0.036	0.018	6.7296E-11
Fhl2	3.132E-15	0.84557481	0.03	0.014	6.8713E-11
Lzic	3.147E-15	0.76275405	0.068	0.042	6.9043E-11
Higd2a	3.2966E-15	0.45437229	0.289	0.25	7.2323E-11
Zfp382	3.4133E-15	1.2177305	0.032	0.015	7.4885E-11
Ttc5	3.8793E-15	0.63217213	0.097	0.065	8.5107E-11
Tln1	3.8897E-15	0.26417055	0.538	0.504	8.5335E-11
Pknox1	4.1401E-15	0.75131088	0.098	0.067	9.083E-11
Sirt7	4.162E-15	0.53710686	0.15	0.112	9.131E-11
Naxd	4.2251E-15	0.63369087	0.123	0.088	9.2694E-11
Dubr	4.7163E-15	0.76426708	0.07	0.043	1.0347E-10
Ube2o	4.7167E-15	0.93383872	0.066	0.041	1.0348E-10
Pkn2	4.9693E-15	0.32009653	0.355	0.309	1.0902E-10
Lonp2	5.4508E-15	0.6757326	0.099	0.068	1.1959E-10
Mrpl13	5.8054E-15	0.56094806	0.176	0.136	1.2737E-10
Runx2os1	6.6393E-15	1.12810648	0.034	0.017	1.4566E-10
Cdc5l	6.8164E-15	0.40140792	0.29	0.247	1.4955E-10
Cct2	7.6467E-15	0.28548866	0.455	0.423	1.6776E-10
Ide	8.1578E-15	0.74079738	0.104	0.072	1.7898E-10
2410015M20F	8.2645E-15	0.40524456	0.339	0.302	1.8132E-10
Mcm4	8.6767E-15	0.56715661	0.131	0.095	1.9036E-10
Mrps30	1.0231E-14	0.69062455	0.109	0.077	2.2446E-10
Derl2	1.0468E-14	0.57221349	0.174	0.135	2.2965E-10
Lppos	1.0659E-14	1.02229396	0.039	0.021	2.3386E-10
Polr2c	1.0791E-14	0.52579905	0.199	0.159	2.3674E-10
Sdf2	1.1368E-14	0.48571216	0.259	0.218	2.4939E-10
1810058I24Ril	1.2373E-14	0.39635856	0.327	0.288	2.7146E-10
Ndufa1	1.2649E-14	0.33181344	0.448	0.42	2.7752E-10
Dcun1d4	1.3032E-14	1.04959941	0.039	0.021	2.8592E-10
Uqcrfs1	1.369E-14	0.37777123	0.379	0.344	3.0034E-10
Zdhhc3	1.4641E-14	0.63477775	0.136	0.101	3.2122E-10
Ndufb6	1.613E-14	0.5208381	0.259	0.221	3.5387E-10
Cers4	1.6693E-14	0.33206268	0.139	0.1	3.6622E-10
Katna1	1.6731E-14	0.58466215	0.177	0.139	3.6705E-10

Ccdc136	1.7343E-14	1.78364631	0.01	0.003	3.8048E-10
Xrn1	1.8174E-14	0.54159484	0.16	0.122	3.9872E-10
Nras	1.8358E-14	0.43516499	0.253	0.212	4.0275E-10
Fam102a	1.8559E-14	0.35030024	0.153	0.112	4.0716E-10
Hadha	2.0494E-14	0.51722707	0.234	0.195	4.4962E-10
Snrk	2.1289E-14	0.59203561	0.11	0.077	4.6705E-10
Bin2	2.3522E-14	0.50848611	0.17	0.131	5.1605E-10
Gmps	2.3718E-14	0.53515496	0.175	0.136	5.2036E-10
Rmnd5a	2.4144E-14	0.61215637	0.163	0.125	5.2969E-10
Eif4ebp2	2.4688E-14	0.47328316	0.216	0.174	5.4163E-10
Ccdc92	2.5289E-14	1.35961125	0.018	0.007	5.5483E-10
Rbm42	2.5737E-14	0.54410663	0.234	0.196	5.6464E-10
Pigf	2.6505E-14	0.94056347	0.045	0.025	5.8149E-10
Zfpm1	2.7742E-14	0.80415169	0.058	0.035	6.0862E-10
Arsb	3.1687E-14	0.90674521	0.067	0.042	6.9519E-10
Gpank1	3.3485E-14	0.69064647	0.094	0.064	7.3462E-10
Isg20	3.3625E-14	0.43892433	0.086	0.057	7.3771E-10
Nfyb	3.4686E-14	0.50784314	0.149	0.112	7.6097E-10
Eapp	3.5204E-14	0.38049564	0.274	0.231	7.7233E-10
Smg6	3.6155E-14	0.49096685	0.172	0.132	7.9321E-10
Il16	3.9124E-14	0.39864918	0.142	0.103	8.5833E-10
Adcy3	3.9496E-14	1.28978295	0.027	0.013	8.665E-10
Rab10os	4.6508E-14	0.67052917	0.194	0.158	1.0203E-09
Sept9	5.0529E-14	0.50723686	0.204	0.164	1.1086E-09
Wapl	5.0537E-14	0.34596577	0.382	0.345	1.1087E-09
Incenp	5.122E-14	0.84529544	0.059	0.036	1.1237E-09
Proser1	5.196E-14	0.64206902	0.12	0.087	1.1399E-09
Unkl	5.2609E-14	0.89343614	0.052	0.03	1.1542E-09
Ccnl2	5.3586E-14	0.35468956	0.338	0.297	1.1756E-09
Mak16	5.5105E-14	0.53233591	0.189	0.15	1.209E-09
Ube2m	5.6203E-14	0.51530352	0.224	0.186	1.233E-09
Slc35g1	6.123E-14	1.04401298	0.023	0.01	1.3433E-09
Minos1	6.4447E-14	0.2815455	0.499	0.476	1.4139E-09
Rb1cc1	6.7395E-14	0.31243464	0.301	0.255	1.4786E-09
Lnpep	6.9152E-14	0.28596613	0.427	0.387	1.5171E-09
Ctr9	8.1742E-14	0.57835915	0.121	0.088	1.7933E-09
Rhno1	8.1827E-14	0.71163781	0.071	0.045	1.7952E-09
Tmem128	8.1851E-14	0.43111562	0.273	0.235	1.7957E-09
Pnkd	8.4885E-14	0.74649495	0.103	0.073	1.8623E-09
Chrac1	8.5147E-14	0.51562828	0.222	0.182	1.8681E-09
Ccdc25	8.9765E-14	0.60855846	0.135	0.101	1.9694E-09
5830432E09R	9.2892E-14	1.61075722	0.02	0.008	2.038E-09
Lbr	9.7892E-14	0.2831728	0.294	0.244	2.1477E-09
Plekhj1	9.842E-14	0.49316323	0.221	0.183	2.1592E-09
Pank2	1.0043E-13	0.52315614	0.171	0.134	2.2033E-09
Hint3	1.0057E-13	0.62871712	0.144	0.109	2.2064E-09
Rab11fip4	1.0189E-13	1.16005639	0.038	0.02	2.2355E-09
Batf	1.0887E-13	0.3729511	0.221	0.178	2.3885E-09

Scml4	1.094E-13	0.84193671	0.044	0.024	2.4002E-09
Zfp949	1.0989E-13	0.81148621	0.043	0.024	2.4108E-09
Iars	1.2078E-13	0.74806122	0.115	0.083	2.6497E-09
Nefh	1.2254E-13	1.44841563	0.012	0.004	2.6884E-09
Ndufaf1	1.2524E-13	1.10070393	0.033	0.017	2.7477E-09
Ccdc88c	1.2921E-13	0.27858593	0.196	0.151	2.8347E-09
Psma4	1.4883E-13	0.43353523	0.302	0.266	3.2652E-09
Hadhb	1.4942E-13	0.45144812	0.216	0.175	3.278E-09
Dtd1	1.5352E-13	0.6702059	0.069	0.044	3.3682E-09
Exosc2	1.5905E-13	0.94550524	0.089	0.061	3.4895E-09
Parg	1.6802E-13	0.55655825	0.123	0.09	3.6861E-09
Gm42372	1.7336E-13	1.08351293	0.02	0.008	3.8034E-09
Jade2	1.7578E-13	0.43475535	0.072	0.046	3.8565E-09
Ppp4r3a	1.7871E-13	0.38335275	0.26	0.219	3.9207E-09
Fbxw2	2.0343E-13	0.34732851	0.265	0.222	4.4632E-09
Cdc34	2.04E-13	0.49252007	0.195	0.158	4.4755E-09
Heca	2.1654E-13	0.30646858	0.272	0.226	4.7508E-09
Nars	2.2466E-13	0.39468863	0.381	0.351	4.9288E-09
Zcchc2	2.2633E-13	0.6015435	0.098	0.068	4.9654E-09
Gtf2b	2.2649E-13	0.45923042	0.232	0.193	4.969E-09
Mrps6	2.4409E-13	0.41829247	0.168	0.13	5.355E-09
Ubap2	2.5707E-13	0.58719687	0.123	0.09	5.64E-09
Tnfsf4	2.8645E-13	1.30702016	0.025	0.011	6.2844E-09
Fkbp1a	3.2521E-13	0.39144952	0.325	0.289	7.1348E-09
Rpa1	3.3749E-13	0.59455343	0.085	0.058	7.4041E-09
Virma	3.6419E-13	0.53344137	0.152	0.116	7.99E-09
Ric1	3.7448E-13	0.36592923	0.264	0.222	8.2157E-09
Smg7	3.8839E-13	0.51439178	0.172	0.135	8.5208E-09
Lypla1	4.033E-13	0.63305656	0.136	0.103	8.848E-09
1110004F10Ri	4.0515E-13	0.37088524	0.348	0.312	8.8886E-09
Plekha3	4.1343E-13	0.64245624	0.113	0.082	9.0702E-09
Gnai3	4.3398E-13	0.33143338	0.306	0.266	9.5211E-09
Oxsr1	4.5547E-13	0.7599155	0.086	0.059	9.9926E-09
Bag4	4.6527E-13	0.68999709	0.072	0.047	1.0208E-08
Zc3h14	5.0921E-13	0.47390284	0.152	0.116	1.1172E-08
Ap2s1	5.1056E-13	0.4440848	0.299	0.267	1.1201E-08
Nsmce3	5.1147E-13	0.62968246	0.102	0.072	1.1221E-08
Setbp1	5.1362E-13	0.63993444	0.038	0.021	1.1268E-08
Nde1	5.214E-13	0.84321408	0.059	0.037	1.1439E-08
Cetn2	5.4129E-13	0.39703902	0.203	0.163	1.1875E-08
Mrps31	5.5721E-13	0.7897646	0.058	0.036	1.2225E-08
Acpp	5.6103E-13	0.62812535	0.055	0.034	1.2309E-08
Ncaph	5.7038E-13	1.13307349	0.03	0.015	1.2513E-08
Cep57	5.9222E-13	0.48828928	0.127	0.093	1.2993E-08
3830406C13R	5.938E-13	0.67844991	0.117	0.086	1.3027E-08
Pms2	6.1912E-13	0.71864334	0.053	0.032	1.3583E-08
Eny2	6.3867E-13	0.42879893	0.261	0.222	1.4012E-08
Asf1a	6.6495E-13	0.60179561	0.09	0.062	1.4588E-08

Gm36756	7.5647E-13	1.47413727	0.01	0.003	1.6596E-08
Mta1	7.6055E-13	0.68166199	0.102	0.073	1.6686E-08
Asxl1	8.8143E-13	0.54080953	0.112	0.081	1.9338E-08
Tprkb	8.9703E-13	0.71228267	0.054	0.033	1.968E-08
Prdm10	9.368E-13	0.9597883	0.054	0.033	2.0553E-08
1700123O20R	9.3866E-13	0.6137129	0.121	0.089	2.0593E-08
Slc4a1ap	9.5306E-13	0.45130642	0.127	0.093	2.0909E-08
A430046D13R	9.6173E-13	1.47071445	0.014	0.005	2.1099E-08
Ebp	9.6432E-13	0.66396304	0.146	0.114	2.1156E-08
Grpel1	1.0204E-12	0.60978923	0.192	0.159	2.2386E-08
Slc16a6	1.0348E-12	0.36809505	0.235	0.194	2.2702E-08
Pik3c3	1.0573E-12	0.68312336	0.078	0.053	2.3195E-08
Narf	1.0595E-12	0.65189644	0.081	0.055	2.3245E-08
Atp5f1	1.1205E-12	0.28051961	0.465	0.439	2.4583E-08
Scyl2	1.191E-12	0.61523517	0.125	0.093	2.613E-08
Gdf11	1.1993E-12	0.90308489	0.024	0.011	2.6312E-08
Ddb2	1.236E-12	0.81574295	0.053	0.032	2.7117E-08
Pglyrp2	1.2385E-12	0.9887201	0.023	0.011	2.7171E-08
Cracr2a	1.3254E-12	1.27389299	0.024	0.011	2.9079E-08
AL590144.2	1.5899E-12	0.70322391	0.077	0.052	3.4881E-08
Cox20	1.6377E-12	0.48024589	0.186	0.149	3.5929E-08
Mrpl4	1.6458E-12	0.58623631	0.14	0.107	3.6107E-08
Hltf	1.8908E-12	0.54199039	0.124	0.092	4.1482E-08
Gtpbp1	2.2931E-12	0.69037288	0.09	0.063	5.0309E-08
Vps36	2.3575E-12	0.46364703	0.206	0.169	5.1721E-08
Fam160a2	2.4541E-12	0.9239615	0.034	0.018	5.3839E-08
Krit1	2.5194E-12	0.27264009	0.335	0.29	5.5274E-08
Cd70	2.5325E-12	1.76663361	0.012	0.004	5.5559E-08
Thap12	2.5411E-12	0.42787076	0.153	0.118	5.5749E-08
Lmbr1l	2.6341E-12	0.54683779	0.086	0.059	5.779E-08
Nudt16	2.7396E-12	0.89671024	0.054	0.033	6.0104E-08
Zfp87	2.7835E-12	0.67300987	0.087	0.06	6.1067E-08
Abcb4	2.815E-12	1.51860542	0.015	0.006	6.1759E-08
Slc35e1	2.963E-12	0.58828268	0.105	0.076	6.5005E-08
Strn	3.1547E-12	0.65976221	0.085	0.059	6.9211E-08
Tubb4b	3.1547E-12	0.51695	0.168	0.133	6.9211E-08
Mrpl30	3.1744E-12	0.46129115	0.311	0.282	6.9643E-08
4931406P16R	3.2311E-12	0.6870182	0.112	0.082	7.0886E-08
Tdp2	3.2366E-12	0.5234883	0.098	0.069	7.1007E-08
Gm28053	3.2763E-12	1.23328431	0.016	0.006	7.1879E-08
Mindy3	3.4096E-12	0.55481115	0.128	0.097	7.4803E-08
Klhl28	3.5118E-12	0.58293651	0.096	0.067	7.7045E-08
Zfand6	3.7373E-12	0.36124148	0.309	0.271	8.1993E-08
Zfp654	3.8175E-12	0.63714251	0.083	0.057	8.3751E-08
Triap1	4.0463E-12	0.71750822	0.083	0.057	8.8772E-08
Gtf2h5	4.0689E-12	0.33253694	0.296	0.258	8.9269E-08
Zmym3	4.0975E-12	1.14206901	0.03	0.015	8.9896E-08
Sra1	4.2595E-12	0.36001363	0.28	0.245	9.3448E-08

2310035C23R	4.2843E-12	0.4503703	0.166	0.13	9.3993E-08
Nup210	4.3969E-12	0.58633718	0.132	0.1	9.6463E-08
Eif3i	4.6912E-12	0.35258026	0.353	0.321	1.0292E-07
Cmtm7	5.0309E-12	0.53729717	0.244	0.211	1.1037E-07
Ppp1r18	5.1259E-12	0.30653949	0.232	0.193	1.1246E-07
Zfp930	5.3056E-12	1.04934699	0.027	0.014	1.164E-07
4930570N18R	5.4954E-12	1.11457416	0.015	0.006	1.2056E-07
Cstad	6.1513E-12	1.40449431	0.015	0.006	1.3495E-07
Psmb4	6.18E-12	0.31561456	0.398	0.371	1.3558E-07
Bola3	6.2346E-12	0.44894943	0.18	0.144	1.3678E-07
Dera	6.3285E-12	0.74014592	0.086	0.061	1.3884E-07
Trim21	6.3364E-12	0.92482737	0.032	0.017	1.3902E-07
Tigar	6.5396E-12	0.87386993	0.032	0.017	1.4347E-07
Ankra2	6.5934E-12	0.47866085	0.092	0.064	1.4465E-07
Hif1an	6.8758E-12	0.4663995	0.127	0.095	1.5085E-07
Rsbn1	6.9076E-12	0.42258659	0.19	0.152	1.5155E-07
Ash2l	7.0455E-12	0.61710067	0.086	0.06	1.5457E-07
Itga6	7.2322E-12	0.51194803	0.073	0.049	1.5867E-07
Alkbh5	7.2478E-12	0.3324037	0.259	0.221	1.5901E-07
Plcl2	7.2676E-12	0.52088371	0.111	0.081	1.5944E-07
Rom1	7.605E-12	1.50564118	0.014	0.005	1.6685E-07
Kpna1	8.5289E-12	0.28892221	0.339	0.299	1.8712E-07
H2afx	8.9371E-12	0.47734433	0.103	0.074	1.9607E-07
Ercc3	9.437E-12	0.69205532	0.087	0.061	2.0704E-07
Nsd2	9.8713E-12	0.53394752	0.148	0.116	2.1657E-07
Mea1	1.1016E-11	0.56144451	0.135	0.104	2.4169E-07
Wipf1	1.1114E-11	0.30006704	0.319	0.282	2.4383E-07
Zfp69	1.1129E-11	1.61146196	0.01	0.003	2.4416E-07
Herc3	1.1153E-11	0.56662873	0.069	0.046	2.4468E-07
Bub3	1.1494E-11	0.47181062	0.19	0.154	2.5217E-07
Mrpl54	1.2876E-11	0.41163641	0.334	0.304	2.8249E-07
Slc7a5	1.3092E-11	0.6966815	0.085	0.059	2.8723E-07
Ssb	1.3269E-11	0.3007354	0.413	0.383	2.9111E-07
Ctcf	1.398E-11	0.26879017	0.32	0.276	3.067E-07
Fbxl14	1.406E-11	0.67519711	0.082	0.057	3.0846E-07
Orc6	1.4073E-11	0.79081739	0.076	0.052	3.0875E-07
Zufsp	1.4681E-11	0.25494141	0.265	0.223	3.2208E-07
Gcc2	1.5042E-11	0.4723409	0.156	0.121	3.3002E-07
Vamp5	1.5371E-11	0.96840614	0.042	0.025	3.3723E-07
Ndufs7	1.688E-11	0.44004864	0.234	0.201	3.7033E-07
Parp4	1.7879E-11	0.60479723	0.124	0.093	3.9224E-07
Fbxl12	1.798E-11	0.33178183	0.153	0.117	3.9447E-07
Cebpg	1.8601E-11	0.47216447	0.216	0.182	4.0809E-07
Wrnip1	1.9257E-11	0.61798658	0.068	0.045	4.2247E-07
Rps6ka1	1.9829E-11	0.65184377	0.1	0.073	4.3504E-07
Txndc17	2.1203E-11	0.34315156	0.326	0.296	4.6516E-07
Man1a2	2.2141E-11	0.38148211	0.178	0.142	4.8574E-07
Zfp251	2.3314E-11	1.11325009	0.019	0.008	5.1148E-07

2610524H06R	2.4822E-11	0.83915854	0.06	0.039	5.4458E-07
Yipf4	2.4823E-11	0.43880768	0.202	0.167	5.4459E-07
Nfkb2	2.5761E-11	0.32020944	0.335	0.301	5.6516E-07
Knop1	2.676E-11	0.5947566	0.119	0.089	5.8708E-07
Cdc37	2.7983E-11	0.33415492	0.373	0.346	6.1392E-07
Ankrd39	2.8719E-11	0.84351191	0.04	0.023	6.3007E-07
Polg2	2.9114E-11	0.35039169	0.099	0.071	6.3873E-07
Ilkap	2.9147E-11	0.56746303	0.143	0.112	6.3945E-07
Znrd1	3.0944E-11	0.41173564	0.225	0.19	6.7888E-07
Pcif1	3.2148E-11	0.41145374	0.264	0.231	7.0529E-07
Rbbp5	3.2376E-11	0.65489829	0.062	0.04	7.103E-07
Ntrk3	3.3682E-11	0.5884323	0.038	0.022	7.3896E-07
2310061I04Ril	3.8675E-11	0.99331532	0.043	0.025	8.485E-07
Kxd1	4.0602E-11	0.51024695	0.205	0.173	8.9076E-07
Eif2d	4.0797E-11	0.8008451	0.06	0.039	8.9506E-07
Yaf2	4.281E-11	0.50963589	0.098	0.07	9.392E-07
Sntb1	4.4033E-11	0.52678245	0.06	0.039	9.6603E-07
Crim1	4.5144E-11	0.68176646	0.073	0.05	9.904E-07
Was	4.5343E-11	0.46520584	0.193	0.16	9.9477E-07
Ppp2r5c	4.7181E-11	0.384421	0.307	0.275	1.0351E-06
Pcnp	4.7912E-11	0.33691472	0.278	0.242	1.0511E-06
Gimap8	4.8925E-11	0.32415695	0.139	0.105	1.0734E-06
Clec2i	5.5514E-11	0.37309906	0.096	0.068	1.2179E-06
Aggf1	5.6607E-11	0.56754419	0.144	0.113	1.2419E-06
Nmnat2	5.8162E-11	1.45500312	0.011	0.004	1.276E-06
Ndufs3	5.8903E-11	0.47476897	0.186	0.154	1.2923E-06
Crebzf	6.1515E-11	0.44274141	0.143	0.111	1.3496E-06
Card6	6.4295E-11	0.47201186	0.072	0.048	1.4106E-06
Gramd1b	6.4828E-11	0.40472655	0.11	0.081	1.4223E-06
Smarcd2	6.5641E-11	0.47461018	0.153	0.121	1.4401E-06
March2	6.9112E-11	0.52199269	0.121	0.092	1.5162E-06
Ube2t	7.6581E-11	0.9639331	0.037	0.021	1.6801E-06
Cyhr1	8.1093E-11	0.71514449	0.097	0.071	1.7791E-06
Ubl4a	8.2902E-11	0.64727666	0.108	0.081	1.8188E-06
Desi1	8.3671E-11	0.61151393	0.089	0.064	1.8357E-06
Zcchc9	8.4832E-11	0.46864198	0.202	0.169	1.8611E-06
Glrx5	9.2828E-11	0.51124243	0.161	0.13	2.0365E-06
Kansl3	9.3666E-11	0.47163285	0.164	0.131	2.0549E-06
Rdx	9.4062E-11	0.26916133	0.315	0.279	2.0636E-06
Itpripl1	9.42E-11	1.01587056	0.036	0.021	2.0667E-06
Mthfd2	9.5519E-11	0.43378015	0.235	0.201	2.0956E-06
Twistnb	1.0128E-10	0.39461402	0.236	0.202	2.2219E-06
Eif4e2	1.0297E-10	0.45878154	0.265	0.236	2.259E-06
Psma5	1.0696E-10	0.40788904	0.283	0.254	2.3467E-06
Pdpk1	1.1209E-10	0.51575203	0.155	0.124	2.4592E-06
Fam98a	1.1402E-10	0.72445491	0.045	0.027	2.5014E-06
Yipf3	1.1993E-10	0.49637672	0.186	0.155	2.6312E-06
Btg3	1.26E-10	0.61107153	0.08	0.056	2.7643E-06

Sh3glb2	1.2687E-10	0.71226712	0.062	0.041	2.7834E-06
Trip4	1.2861E-10	0.6546692	0.069	0.047	2.8215E-06
Rpusd4	1.3211E-10	0.691202	0.062	0.041	2.8985E-06
Osgin1	1.3449E-10	0.53294233	0.067	0.045	2.9505E-06
Copb2	1.3496E-10	0.42943784	0.225	0.192	2.9608E-06
Fmr1	1.3632E-10	0.33586962	0.208	0.172	2.9906E-06
Abcf1	1.3674E-10	0.41090064	0.28	0.248	3E-06
Rab37	1.4574E-10	0.51332584	0.055	0.035	3.1975E-06
Ubac1	1.4887E-10	0.72855896	0.052	0.033	3.266E-06
Ccndbp1	1.5182E-10	0.52120477	0.141	0.111	3.3308E-06
Phf11c	1.5465E-10	1.21908614	0.017	0.007	3.3928E-06
Vamp2	1.5469E-10	0.41271941	0.177	0.144	3.3938E-06
Nr2c2	1.5473E-10	0.38739689	0.197	0.161	3.3946E-06
Ccnk	1.611E-10	0.5647205	0.141	0.112	3.5343E-06
Azi2	1.6653E-10	0.37452678	0.211	0.176	3.6535E-06
Htatip2	1.8125E-10	0.76830135	0.078	0.055	3.9764E-06
Thoc7	1.9039E-10	0.41883937	0.233	0.2	4.1771E-06
Rpl27-ps3	1.9958E-10	0.5574578	0.124	0.096	4.3786E-06
Phf3	2.0232E-10	0.26488556	0.386	0.353	4.4387E-06
Umps	2.0385E-10	0.81125878	0.053	0.034	4.4723E-06
Nosip	2.053E-10	0.69287434	0.077	0.054	4.504E-06
Ngdn	2.1373E-10	0.51828656	0.183	0.152	4.6891E-06
Xlr4c	2.143E-10	0.60993791	0.036	0.021	4.7016E-06
Thumpd1	2.1501E-10	0.50276933	0.113	0.085	4.717E-06
Tmem29	2.306E-10	0.69250687	0.074	0.052	5.0592E-06
Mfap1a	2.4363E-10	0.54728632	0.111	0.083	5.3449E-06
Mark2	2.4432E-10	0.3507158	0.248	0.213	5.3602E-06
Psmb6	2.4572E-10	0.2500925	0.397	0.365	5.3909E-06
Ldb1	2.8669E-10	0.4292454	0.142	0.111	6.2896E-06
Vps51	2.9061E-10	0.73449672	0.081	0.058	6.3756E-06
Paqr7	3.0885E-10	1.41030666	0.016	0.007	6.7759E-06
Nkap	3.0961E-10	0.4790325	0.138	0.108	6.7925E-06
Adrm1	3.2919E-10	0.58190751	0.158	0.129	7.222E-06
Ccsap	3.5257E-10	0.9788728	0.033	0.019	7.7351E-06
Jak3	3.554E-10	0.64415623	0.078	0.055	7.7971E-06
Ndufv2	3.6233E-10	0.49586668	0.221	0.192	7.9492E-06
Map2k2	3.7708E-10	0.4393724	0.253	0.223	8.2727E-06
Abhd8	4.1378E-10	0.60114628	0.049	0.031	9.0779E-06
Mettl26	4.1867E-10	0.61499344	0.082	0.059	9.1852E-06
Topbp1	4.4872E-10	0.41030784	0.121	0.092	9.8444E-06
Tinf2	4.5042E-10	0.64452401	0.107	0.081	9.8817E-06
Ddx10	4.5315E-10	0.4847092	0.124	0.095	9.9416E-06
Kif21b	4.6127E-10	0.61114938	0.106	0.08	1.012E-05
Taf1b	4.721E-10	0.79251656	0.056	0.037	1.0357E-05
Neu3	4.7852E-10	0.50746001	0.031	0.017	1.0498E-05
Axin1	4.8416E-10	0.65145429	0.1	0.075	1.0622E-05
E2f4	4.89E-10	0.61181926	0.1	0.074	1.0728E-05
Smim26	4.9244E-10	0.69702462	0.094	0.07	1.0804E-05

Sec31a	4.9319E-10	0.48987778	0.182	0.151	1.082E-05
Mapk1	5.0795E-10	0.33746839	0.263	0.231	1.1144E-05
Mical1	5.1805E-10	0.5513642	0.158	0.129	1.1365E-05
Fnta	5.4305E-10	0.60394018	0.068	0.047	1.1914E-05
Aim2	5.8726E-10	0.52438953	0.132	0.104	1.2884E-05
Vdac2	5.9468E-10	0.29147022	0.349	0.32	1.3047E-05
Nkiras1	5.9731E-10	0.75312196	0.038	0.023	1.3104E-05
Trappc4	6.0038E-10	0.40069259	0.217	0.185	1.3172E-05
Tmem18	6.1992E-10	1.18598274	0.025	0.013	1.36E-05
Ndufb5	6.8034E-10	0.29953723	0.383	0.359	1.4926E-05
Tbce	7.4449E-10	0.59072684	0.102	0.077	1.6333E-05
4930522L14Ri	7.5633E-10	0.94599612	0.031	0.017	1.6593E-05
Prpf4	7.671E-10	0.43565937	0.118	0.09	1.6829E-05
Mrpl15	7.6937E-10	0.5025275	0.15	0.122	1.6879E-05
Fbxo9	8.2397E-10	0.61075996	0.078	0.055	1.8077E-05
Rhof	8.4378E-10	0.42131446	0.119	0.091	1.8512E-05
Chpt1	8.4401E-10	1.01611434	0.056	0.038	1.8517E-05
Kmt5b	8.4627E-10	0.38145006	0.196	0.163	1.8566E-05
Slu7	8.4875E-10	0.44982368	0.21	0.179	1.8621E-05
Rnf219	8.5735E-10	0.77933129	0.033	0.019	1.8809E-05
Rprd2	8.8409E-10	0.37304486	0.147	0.116	1.9396E-05
Lrba	8.8469E-10	0.42889751	0.125	0.096	1.9409E-05
Cenpl	9.7035E-10	0.64649029	0.113	0.087	2.1289E-05
Cnot11	1.0044E-09	0.57285871	0.081	0.058	2.2035E-05
Ankrd10	1.0289E-09	0.54504285	0.089	0.065	2.2572E-05
Strip1	1.0359E-09	0.61467576	0.083	0.06	2.2726E-05
Tpgs2	1.0458E-09	0.68085164	0.047	0.03	2.2944E-05
Rngtt	1.089E-09	0.51553608	0.107	0.081	2.3891E-05
Ppp1r35	1.0947E-09	0.70560804	0.06	0.041	2.4018E-05
Bhlhb9	1.1409E-09	0.95805171	0.024	0.013	2.503E-05
Exosc7	1.1595E-09	0.74561313	0.058	0.039	2.5438E-05
1300002E11R	1.1789E-09	0.55132243	0.057	0.038	2.5865E-05
Cdv3	1.2008E-09	0.26887065	0.433	0.411	2.6345E-05
Limk2	1.2329E-09	0.74485865	0.037	0.022	2.7048E-05
Pitpnm2	1.2664E-09	0.63564359	0.033	0.019	2.7783E-05
Abt1	1.27E-09	0.64546399	0.083	0.06	2.7862E-05
Rsf1	1.2703E-09	0.35898433	0.235	0.201	2.7869E-05
Gm9856	1.2737E-09	0.70300738	0.043	0.027	2.7943E-05
Retreg3	1.2853E-09	0.49519539	0.109	0.082	2.8198E-05
Avl9	1.3056E-09	0.28529371	0.267	0.229	2.8643E-05
Slc50a1	1.3328E-09	0.40421866	0.158	0.127	2.924E-05
Psmb10	1.3535E-09	0.48890084	0.231	0.204	2.9695E-05
Tgif2	1.3579E-09	0.63646332	0.056	0.038	2.9791E-05
Usp38	1.3713E-09	0.3143406	0.168	0.135	3.0084E-05
Ppp4r3b	1.4378E-09	0.2947357	0.249	0.215	3.1544E-05
Itgb2	1.4912E-09	0.28153151	0.279	0.247	3.2715E-05
Rbm4b	1.4986E-09	0.64115351	0.102	0.077	3.2877E-05
Smim15	1.5102E-09	0.38768338	0.174	0.143	3.3132E-05

2310033P09R	1.5525E-09	0.53660882	0.116	0.089	3.406E-05
Ddt	1.6147E-09	0.42227428	0.2	0.171	3.5424E-05
Dcp2	1.6777E-09	0.53393622	0.147	0.119	3.6807E-05
Supt5	1.7639E-09	0.32256537	0.299	0.268	3.8699E-05
Dnajc2	1.8599E-09	0.36168418	0.21	0.178	4.0805E-05
Ndufa3	1.9095E-09	0.26281812	0.428	0.409	4.1893E-05
Fam207a	1.9203E-09	0.67644334	0.096	0.072	4.213E-05
Sp4	1.9444E-09	0.32796761	0.157	0.124	4.2659E-05
Snip1	1.9743E-09	0.63231952	0.096	0.072	4.3315E-05
Zfp142	1.99E-09	0.65733746	0.061	0.042	4.366E-05
Rasal3	2.0281E-09	0.38509344	0.092	0.067	4.4494E-05
Sdhb	2.0453E-09	0.44370572	0.211	0.183	4.4872E-05
Gak	2.0612E-09	0.42352078	0.19	0.16	4.5221E-05
Ecsit	2.2156E-09	0.65878606	0.051	0.033	4.8607E-05
Ccng1	2.2328E-09	0.47115802	0.146	0.118	4.8985E-05
Fchsd1	2.2695E-09	1.04096946	0.021	0.011	4.9791E-05
Tars	2.4482E-09	0.46579604	0.144	0.115	5.3711E-05
Nudt16l1	2.469E-09	0.59991916	0.065	0.045	5.4167E-05
Hdac1	2.4691E-09	0.41872455	0.14	0.111	5.417E-05
Dennd2d	2.4979E-09	0.38977526	0.063	0.043	5.4801E-05
Sash3	2.5033E-09	0.44306646	0.085	0.062	5.492E-05
Sgcb	2.6934E-09	0.91122917	0.033	0.02	5.9091E-05
Ccser2	2.7108E-09	0.4286365	0.148	0.119	5.9473E-05
Bloc1s4	2.7527E-09	0.6426433	0.11	0.085	6.0391E-05
Fam193b	2.8586E-09	0.60048838	0.069	0.048	6.2715E-05
Cep192	2.9545E-09	0.4795265	0.101	0.076	6.4819E-05
Nif3l1	3.1766E-09	0.79657322	0.039	0.024	6.9691E-05
Ppil1	3.2224E-09	0.58517652	0.058	0.039	7.0696E-05
Zfp91	3.4146E-09	0.29455412	0.4	0.379	7.4913E-05
Lypla2	3.4941E-09	0.42353341	0.146	0.118	7.6656E-05
Wipi2	3.5525E-09	0.47765024	0.136	0.108	7.7939E-05
Fbxo32	3.6629E-09	0.63382693	0.068	0.048	8.036E-05
U2af1	3.7664E-09	0.34968801	0.323	0.298	8.2631E-05
Snrpd1	3.8773E-09	0.28416879	0.36	0.335	8.5065E-05
Dhx8	3.946E-09	0.47807278	0.163	0.134	8.6572E-05
Nono	3.9598E-09	0.32288436	0.308	0.28	8.6874E-05
Mturn	4.1588E-09	0.7669468	0.022	0.011	9.1241E-05
Ccdc174	4.1595E-09	0.37113334	0.214	0.184	9.1254E-05
Ep400	4.1723E-09	0.42787656	0.232	0.201	9.1536E-05
Rpap3	4.1846E-09	0.55472681	0.061	0.041	9.1805E-05
Sp3	4.283E-09	0.26652832	0.238	0.204	9.3965E-05
Mterf2	4.3464E-09	1.01801674	0.011	0.004	9.5355E-05
Akr1b3	4.3544E-09	0.39814213	0.226	0.198	9.553E-05
Dcaf15	4.4002E-09	0.45259802	0.11	0.084	9.6536E-05
Pdcd6ip	4.5058E-09	0.30518142	0.35	0.323	9.8852E-05
Dpm3	4.926E-09	0.33772519	0.279	0.249	0.00010807
Nt5dc1	5.0956E-09	0.62305602	0.056	0.038	0.00011179
Fam53b	5.2999E-09	0.64347956	0.044	0.028	0.00011627

Fdxr	5.3747E-09	0.70944798	0.041	0.025	0.00011792
Lsm4	5.4303E-09	0.28512253	0.351	0.326	0.00011913
Dffb	6.0668E-09	0.99422511	0.028	0.016	0.0001331
Accs	6.1481E-09	0.93097257	0.025	0.014	0.00013488
Metrn	6.5385E-09	0.75721061	0.044	0.028	0.00014345
Cnep1r1	7.1897E-09	0.64683346	0.077	0.056	0.00015773
Gm26799	7.3169E-09	0.97158307	0.02	0.01	0.00016053
Snrnp40	7.3228E-09	0.50883134	0.122	0.096	0.00016066
Eif3d	7.6274E-09	0.38216814	0.244	0.216	0.00016734
Tex30	7.8296E-09	0.57220373	0.072	0.052	0.00017177
Terf2ip	7.905E-09	0.59862629	0.055	0.037	0.00017343
Osbpl7	8.3299E-09	0.60594596	0.036	0.022	0.00018275
Nin	8.3347E-09	0.45746838	0.159	0.13	0.00018285
Tor1a	8.3944E-09	0.59828182	0.127	0.102	0.00018417
Acot2	8.4796E-09	0.54628086	0.053	0.035	0.00018603
Rab29	8.6355E-09	0.99276696	0.028	0.016	0.00018946
Pink1	8.9026E-09	0.57251869	0.097	0.074	0.00019531
Sptan1	9.2226E-09	0.32492339	0.201	0.17	0.00020233
Styk1	9.3738E-09	1.15520688	0.018	0.009	0.00020565
Zfp760	9.4448E-09	0.96188377	0.017	0.008	0.00020721
Ngrn	9.6398E-09	0.69511781	0.052	0.035	0.00021149
Kctd9	9.7579E-09	0.86033001	0.039	0.025	0.00021408
Os9	9.7923E-09	0.42380647	0.234	0.207	0.00021483
Ly6k	9.8511E-09	0.97225396	0.014	0.006	0.00021612
1190007I07Ril	1.0083E-08	0.79621737	0.048	0.031	0.0002212
Actr5	1.0143E-08	0.68239311	0.065	0.046	0.00022252
Taf12	1.0419E-08	0.57590653	0.08	0.058	0.00022859
Psmc5	1.0867E-08	0.48224723	0.197	0.17	0.00023842
Dctn6	1.1154E-08	0.48408087	0.092	0.069	0.00024472
L3mbtl2	1.1992E-08	0.90903506	0.029	0.017	0.00026309
Sap30bp	1.2766E-08	0.55238342	0.099	0.076	0.00028007
Slc4a7	1.2857E-08	0.37839448	0.288	0.262	0.00028206
Cdipt	1.3204E-08	0.46546988	0.153	0.125	0.00028969
Zdhhc7	1.3531E-08	0.76195593	0.063	0.045	0.00029686
Zfp472	1.3848E-08	0.66271918	0.041	0.026	0.0003038
Emc3	1.4316E-08	0.44423375	0.201	0.173	0.00031407
Zfp398	1.4445E-08	0.54808416	0.069	0.049	0.0003169
Uimc1	1.5315E-08	0.45801492	0.168	0.141	0.000336
Nap1l4	1.5798E-08	0.32507939	0.217	0.186	0.00034659
Cdc37l1	1.6128E-08	0.26010592	0.216	0.183	0.00035384
Ino80b	1.6131E-08	0.5367696	0.098	0.074	0.0003539
Snrpc	1.7362E-08	0.35398482	0.256	0.23	0.00038091
Gpbp1l1	1.7612E-08	0.33259953	0.219	0.188	0.00038639
Crebbp	1.7868E-08	0.27543796	0.378	0.353	0.000392
Polr2h	1.7875E-08	0.60418419	0.102	0.079	0.00039216
Dusp11	1.8252E-08	0.25672904	0.4	0.379	0.00040044
Ppat	1.8352E-08	0.60536942	0.063	0.044	0.00040262
Dcaf12	1.8497E-08	0.54539003	0.153	0.127	0.00040581

Mau2	1.9251E-08	0.38306068	0.171	0.142	0.00042236
Uqcrc1	1.9416E-08	0.47081862	0.222	0.197	0.00042596
Med4	1.9499E-08	0.40799064	0.124	0.098	0.00042778
Fkbp8	1.9552E-08	0.38026891	0.225	0.198	0.00042895
Swt1	2.0618E-08	0.25527766	0.18	0.148	0.00045233
4833439L19Ri	2.2601E-08	0.54089648	0.114	0.089	0.00049585
Snhg6	2.2714E-08	0.41784483	0.227	0.2	0.00049831
Zfp672	2.3811E-08	0.47670495	0.135	0.109	0.00052239
Mrps35	2.384E-08	0.60881947	0.074	0.054	0.00052303
Timm17b	2.3986E-08	0.46352493	0.143	0.117	0.00052623
Txnl4a	2.4024E-08	0.55768508	0.119	0.095	0.00052705
Bod1l	2.4035E-08	0.27835904	0.248	0.216	0.0005273
Edaradd	2.4429E-08	0.8089604	0.016	0.008	0.00053595
Ubash3b	2.4612E-08	0.62420866	0.118	0.095	0.00053996
Acot9	2.5017E-08	0.58914783	0.09	0.068	0.00054884
Mtf1	2.5542E-08	0.66737252	0.076	0.056	0.00056037
Dnmt1	2.7132E-08	0.43915348	0.091	0.069	0.00059524
Rnf187	2.7674E-08	0.42431045	0.164	0.137	0.00060714
Mrpl46	2.8663E-08	0.60812372	0.097	0.074	0.00062884
E130311K13R	2.8804E-08	0.99003953	0.022	0.012	0.00063193
AI467606	2.8929E-08	0.3112091	0.105	0.08	0.00063468
Smn1	2.923E-08	0.5628488	0.107	0.083	0.00064128
Cipc	2.9377E-08	0.46085157	0.123	0.097	0.00064451
Rce1	3.2868E-08	0.60609651	0.072	0.052	0.0007211
Dbr1	3.4803E-08	0.7242515	0.061	0.043	0.00076354
Zfp846	3.5214E-08	0.718914	0.023	0.013	0.00077256
Zmat2	3.5633E-08	0.25499776	0.326	0.301	0.00078175
Nbdy	3.6E-08	0.73823147	0.091	0.07	0.00078981
Crybg1	3.658E-08	0.47627083	0.155	0.129	0.00080253
Brcc3	3.8799E-08	0.58246259	0.086	0.065	0.0008512
Cdkn2aipnl	3.8956E-08	0.57038907	0.069	0.049	0.00085466
Zrsr2	3.9184E-08	0.38631814	0.26	0.235	0.00085967
Cops5	3.9388E-08	0.43647389	0.187	0.159	0.00086413
Acbd5	4.0297E-08	0.33762207	0.193	0.164	0.00088408
Mrps11	4.0702E-08	0.74269375	0.065	0.047	0.00089296
Hnrnpul1	4.1748E-08	0.28332459	0.318	0.291	0.00091592
Nxf1	4.2405E-08	0.30422582	0.216	0.186	0.00093032
Zfp942	4.3325E-08	0.58873247	0.068	0.049	0.00095051
Calm3	4.3781E-08	0.26001289	0.352	0.33	0.00096051
Nans	4.3908E-08	0.55673514	0.116	0.093	0.0009633
Dpf2	4.4615E-08	0.41360687	0.15	0.124	0.0009788
Rnf220	4.4842E-08	0.35523568	0.152	0.125	0.00098378
C1qtnf4	4.6711E-08	1.20552922	0.017	0.008	0.00102479
Zfp53	4.674E-08	0.47326063	0.054	0.037	0.00102543
Psmd14	4.7562E-08	0.49302761	0.199	0.175	0.00104345
Unc45a	4.9087E-08	0.50049334	0.102	0.079	0.00107691
Gm26532	5.0217E-08	0.55048911	0.094	0.072	0.00110172
G2e3	5.0619E-08	0.58266616	0.077	0.057	0.00111052

Capn7	5.0997E-08	0.52781134	0.103	0.08	0.00111883
Itgb1bp1	5.1436E-08	0.6679043	0.089	0.068	0.00112845
Uqcc2	5.1675E-08	0.49657765	0.225	0.201	0.0011337
Kdm1a	5.2883E-08	0.35091497	0.256	0.229	0.0011602
Snrnp27	5.3132E-08	0.3185367	0.223	0.194	0.00116567
Lig1	5.6498E-08	0.4293188	0.079	0.059	0.0012395
Tpd52l2	5.6675E-08	0.43269826	0.173	0.146	0.00124339
Ebna1bp2	5.7949E-08	0.41336384	0.162	0.136	0.00127134
Commd7	5.8291E-08	0.52847778	0.133	0.109	0.00127884
Crkl	5.8675E-08	0.50723806	0.111	0.088	0.00128727
Dmac2	5.8699E-08	0.43383036	0.096	0.074	0.0012878
0610010K14R	5.9625E-08	0.61359687	0.066	0.048	0.00130811
2210016L21Ri	6.0889E-08	0.63691087	0.086	0.065	0.00133585
Pxmp2	6.3471E-08	1.35893512	0.01	0.004	0.00139249
Smim20	6.5138E-08	0.59819046	0.09	0.069	0.00142907
Txndc9	6.6088E-08	0.4194878	0.209	0.184	0.0014499
Gm16062	6.8045E-08	0.86235474	0.042	0.027	0.00149284
Zdhhc12	6.8736E-08	1.15761764	0.022	0.012	0.001508
Mtif3	6.9586E-08	0.67477079	0.039	0.025	0.00152664
Gtf3a	7.0124E-08	0.69777128	0.076	0.057	0.00153844
Me2	7.3189E-08	0.60192655	0.114	0.092	0.0016057
Rnpc3	7.3274E-08	0.44661794	0.123	0.099	0.00160755
Afg3l1	7.5348E-08	0.48376901	0.114	0.091	0.00165306
Lamtor5	7.6645E-08	0.42613491	0.197	0.173	0.00168152
Camk2g	7.7336E-08	0.65392487	0.059	0.042	0.00169666
AC163354.1	7.8719E-08	0.56181507	0.064	0.046	0.00172701
Otud5	7.8988E-08	0.50450344	0.147	0.122	0.00173292
Cdan1	7.9608E-08	0.68929722	0.031	0.019	0.00174651
Pbxip1	8.0028E-08	0.27044345	0.222	0.192	0.00175573
Rrp8	8.271E-08	0.52806722	0.072	0.052	0.00181458
Acss2	8.6791E-08	0.77973032	0.039	0.025	0.00190411
Thap11	8.8878E-08	1.02400588	0.035	0.022	0.00194989
Mrps34	9.3809E-08	0.58825905	0.119	0.096	0.00205808
Zwint	9.8745E-08	0.57885269	0.096	0.074	0.00216636
Numa1	9.9663E-08	0.26039292	0.227	0.196	0.0021865
Zfp646	1.0047E-07	0.41792523	0.09	0.068	0.0022043
Hras	1.0048E-07	0.62377308	0.093	0.072	0.00220436
Nudt22	1.0173E-07	1.13396996	0.021	0.011	0.00223181
Gm10036	1.057E-07	0.30814627	0.31	0.281	0.00231901
Ufd1	1.0746E-07	0.48650102	0.117	0.094	0.00235766
Zfp746	1.1175E-07	0.4587258	0.104	0.081	0.0024516
Rapgef1	1.1821E-07	0.49744337	0.128	0.104	0.00259339
Cabin1	1.2367E-07	0.49240611	0.121	0.097	0.00271314
Rnps1	1.2707E-07	0.32473975	0.258	0.23	0.00278781
Ing1	1.2883E-07	0.36201498	0.162	0.135	0.0028264
Pin4	1.2901E-07	0.42830759	0.211	0.187	0.00283043
Tsn	1.2974E-07	0.3171148	0.268	0.242	0.00284646
Gabbr1	1.3018E-07	0.41493143	0.081	0.06	0.00285604

Ncapd3	1.3031E-07	0.60125689	0.075	0.056	0.00285878
2900076A07R	1.3462E-07	0.83754585	0.023	0.013	0.0029535
Rnf10	1.3477E-07	0.40250239	0.241	0.218	0.00295682
Hspa14	1.4017E-07	0.45580273	0.15	0.125	0.00307521
Mef2d	1.4095E-07	0.27761544	0.306	0.281	0.0030923
Asb3	1.4396E-07	0.6043735	0.041	0.027	0.00315826
Frat1	1.4799E-07	0.75029215	0.032	0.02	0.00324666
Fam162a	1.4807E-07	0.59413393	0.112	0.09	0.00324845
Airn	1.4921E-07	1.22257782	0.014	0.007	0.00327346
Bms1	1.5001E-07	0.33745576	0.133	0.107	0.00329114
Pcnt	1.5066E-07	0.43492586	0.128	0.104	0.00330541
Fam172a	1.5198E-07	0.45067522	0.162	0.136	0.00333439
Nemp2	1.5917E-07	0.46247765	0.057	0.04	0.00349203
Trnt1	1.6114E-07	0.43397964	0.178	0.153	0.00353528
Ccpg1	1.6472E-07	0.52875445	0.076	0.056	0.00361385
Cep41	1.7116E-07	0.71704068	0.022	0.013	0.0037551
Pag1	1.7553E-07	0.32933574	0.15	0.123	0.00385086
Nr1h2	1.778E-07	0.49299644	0.147	0.123	0.0039007
Wdr44	1.7782E-07	0.43811289	0.08	0.06	0.00390115
Ppil4	1.855E-07	0.3910473	0.181	0.155	0.00406977
Gmfb	1.9334E-07	0.35372612	0.215	0.189	0.00424176
Dcps	2.0745E-07	0.70958399	0.068	0.05	0.00455121
Taf11	2.119E-07	0.54768172	0.087	0.067	0.0046488
Coa5	2.134E-07	0.38482041	0.18	0.156	0.00468174
Ddx52	2.1634E-07	0.37338285	0.18	0.154	0.00474619
Fbxo45	2.2058E-07	0.74058183	0.042	0.028	0.00483934
Trmt1l	2.2319E-07	0.36244373	0.108	0.085	0.00489667
Zdhhc20	2.3016E-07	0.27899882	0.214	0.185	0.00504941
Brd3	2.3884E-07	0.41695885	0.108	0.085	0.00523991
Dda1	2.4509E-07	0.40054977	0.182	0.157	0.00537692
Prmt9	2.5106E-07	0.74943216	0.031	0.019	0.00550801
Rasgrp1	2.5957E-07	0.26955017	0.188	0.158	0.00569465
Cdca7l	2.6076E-07	0.9293115	0.031	0.02	0.00572078
Ndufaf8	2.6381E-07	0.41794848	0.212	0.189	0.00578766
Tbpl1	2.6945E-07	0.45478062	0.106	0.084	0.00591136
Clp1	2.7412E-07	0.46468814	0.088	0.067	0.00601386
Mta3	2.8237E-07	0.5145069	0.102	0.081	0.00619486
Fam118a	2.9617E-07	0.5028729	0.047	0.032	0.00649767
Mrpl51	2.9911E-07	0.46782922	0.156	0.132	0.00656207
Dnajc5	3.0228E-07	0.33238643	0.273	0.249	0.00663181
Polk	3.0546E-07	0.53279115	0.04	0.027	0.00670151
Zcrb1	3.078E-07	0.2755697	0.32	0.299	0.00675273
Zfp874a	3.1339E-07	0.71709603	0.032	0.02	0.00687548
Kdm1b	3.2881E-07	0.72439002	0.028	0.017	0.00721376
Ralgapa2	3.379E-07	0.52714417	0.058	0.042	0.00741329
Tarbp2	3.4861E-07	0.56941266	0.059	0.042	0.00764807
Jazf1	3.553E-07	1.15566903	0.011	0.005	0.00779489
Ankrd17	3.59E-07	0.25418821	0.262	0.234	0.00787601

Impa1	3.6941E-07	0.41665219	0.138	0.114	0.00810456
Sh3bp5l	3.7122E-07	0.79034438	0.03	0.019	0.00814415
Hyls1	3.8363E-07	0.80671474	0.023	0.013	0.00841642
Kif5c	3.8941E-07	1.16466831	0.015	0.008	0.00854336
Tm9sf1	3.9095E-07	0.77973888	0.031	0.019	0.00857713
Golga4	3.9926E-07	0.30555883	0.253	0.227	0.00875929
Cnnm3	4.0556E-07	0.61160444	0.046	0.031	0.00889768
Kctd18	4.2109E-07	0.50302888	0.064	0.047	0.00923828
Yars	4.2197E-07	0.57248786	0.092	0.072	0.00925758
C1d	4.2407E-07	0.47703068	0.117	0.095	0.00930362
Slc52a3	4.4146E-07	0.64242916	0.024	0.014	0.00968517
Gm11808	4.4286E-07	0.29808852	0.288	0.264	0.0097159
Heatr3	4.4963E-07	0.7533641	0.048	0.034	0.00986437
Cox18	4.6122E-07	0.59725461	0.044	0.03	0.01011877
Esco1	4.7316E-07	0.30620598	0.157	0.131	0.01038071
Apopt1	4.7517E-07	0.58418577	0.104	0.084	0.01042467
Tfpt	4.7605E-07	0.75248295	0.042	0.029	0.01044404
Arhgap1	4.7754E-07	0.5651552	0.089	0.069	0.01047678
Armcx5	4.9222E-07	0.43280032	0.075	0.056	0.01079878
Zfc3h1	4.9696E-07	0.46705287	0.118	0.096	0.01090273
Tecr	4.9881E-07	0.41536434	0.194	0.17	0.0109433
Rfc3	5.0137E-07	0.59837979	0.056	0.04	0.01099952
Tnfaip8l1	5.101E-07	0.84310933	0.026	0.015	0.01119101
Micu2	5.2455E-07	0.51235166	0.099	0.079	0.01150804
Pop7	5.2718E-07	0.56863255	0.118	0.097	0.01156578
Topors	5.3055E-07	0.44119999	0.122	0.099	0.01163964
Bdh1	5.4325E-07	0.65464076	0.022	0.012	0.01191841
Zfp574	5.6524E-07	0.67542759	0.042	0.028	0.01240073
Zfp758	5.9651E-07	0.58650873	0.043	0.029	0.01308673
Dnttip2	6.1417E-07	0.29346417	0.228	0.203	0.01347419
Leng1	6.4298E-07	0.55769396	0.075	0.056	0.01410643
Map1lc3a	6.4308E-07	0.46815191	0.152	0.13	0.01410863
Fam216a	6.6592E-07	0.6305673	0.044	0.03	0.01460957
Upf2	6.7321E-07	0.27319019	0.181	0.154	0.01476952
Rhoc	6.7375E-07	0.38377083	0.1	0.079	0.01478134
Ggps1	6.8528E-07	0.36160015	0.107	0.085	0.01503436
Dusp14	7.0189E-07	0.63104291	0.023	0.013	0.01539884
Zfp212	7.1349E-07	0.63980068	0.054	0.039	0.01565328
Dtx3	7.1369E-07	0.56070235	0.085	0.066	0.01565764
Gm11110	7.3771E-07	1.01520644	0.013	0.007	0.01618469
Trappc5	7.3793E-07	0.41375252	0.126	0.104	0.01618939
Prorsd1	7.3936E-07	0.31740908	0.115	0.093	0.01622088
Fbxw17	7.74E-07	0.77141115	0.036	0.023	0.01698089
Usp15	7.751E-07	0.28140645	0.259	0.234	0.01700499
Ormdl3	7.7605E-07	0.27157323	0.101	0.079	0.01702583
Pip4k2c	7.8098E-07	0.45946959	0.09	0.07	0.01713397
Timm13	7.9805E-07	0.26483867	0.358	0.34	0.01750845
Dnajb13	8.1214E-07	1.02745866	0.012	0.006	0.01781755

Phf1	8.8535E-07	0.40258218	0.089	0.069	0.01942361
Tmem127	8.9985E-07	0.43349299	0.101	0.081	0.01974186
Eif1ad	9.3581E-07	0.48928157	0.137	0.115	0.02053084
1810022K09R	9.3728E-07	0.48657966	0.185	0.164	0.020563
Vps72	9.3815E-07	0.57531247	0.069	0.052	0.02058201
Simc1	9.7341E-07	0.86596584	0.032	0.02	0.02135554
Pcmt1	9.8146E-07	0.43225256	0.191	0.167	0.02153217
Spock2	9.888E-07	0.49641661	0.027	0.016	0.02169326
1110008F13Ri	9.9915E-07	0.33833176	0.229	0.207	0.02192031
Tex261	1.0029E-06	0.44672589	0.145	0.122	0.02200316
Stk11	1.0224E-06	0.32675854	0.248	0.224	0.02243076
Leo1	1.0692E-06	0.57406075	0.072	0.054	0.02345697
Lrwd1	1.083E-06	0.76326023	0.044	0.03	0.0237608
Herc4	1.1161E-06	0.28000082	0.168	0.141	0.0244871
Khnyn	1.1217E-06	0.39429073	0.092	0.072	0.02460884
Med30	1.1407E-06	0.46045689	0.138	0.115	0.02502641
Actl6a	1.1579E-06	0.37875407	0.13	0.107	0.02540269
Ralgapb	1.1935E-06	0.46048392	0.1	0.079	0.02618376
Pigx	1.2011E-06	0.55404301	0.095	0.076	0.02634985
Alg10b	1.2137E-06	0.63026056	0.057	0.042	0.02662694
Gps2	1.2148E-06	0.41710488	0.168	0.145	0.02665242
Dgkz	1.2209E-06	0.45942251	0.1	0.08	0.02678461
Pfdn1	1.2665E-06	0.4364692	0.168	0.146	0.02778574
Zfp511	1.282E-06	0.55859711	0.097	0.077	0.0281265
Angel2	1.3017E-06	0.3471096	0.177	0.152	0.02855856
Hells	1.3191E-06	0.40329337	0.082	0.063	0.02894045
Rbm27	1.3313E-06	0.28665744	0.251	0.226	0.02920631
Rnf167	1.3707E-06	0.32467981	0.129	0.106	0.03007212
Ube2j2	1.3731E-06	0.3569977	0.243	0.222	0.03012396
Capn10	1.3782E-06	0.84362491	0.022	0.013	0.0302358
Cep120	1.432E-06	0.29703996	0.134	0.11	0.03141771
Dusp19	1.4777E-06	0.81605656	0.028	0.018	0.03241951
Lnpk	1.4999E-06	0.43408515	0.104	0.083	0.03290566
Cenpv	1.5111E-06	1.04181936	0.026	0.016	0.03315277
Ccdc127	1.5225E-06	0.52240633	0.109	0.088	0.03340231
Socs7	1.6245E-06	0.61192163	0.063	0.047	0.03563999
Pole4	1.6326E-06	0.27993729	0.275	0.251	0.03581864
Prcc	1.6602E-06	0.59736976	0.059	0.044	0.03642228
Akirin1	1.7208E-06	0.31149153	0.249	0.225	0.03775262
Rexo1	1.8128E-06	0.36543982	0.165	0.142	0.03977021
Ube2l3	1.8149E-06	0.25600482	0.382	0.369	0.03981652
Cpsf4	1.8462E-06	0.62881207	0.048	0.034	0.04050329
Coa6	1.8594E-06	0.64800282	0.071	0.054	0.04079262
Rbck1	1.87E-06	0.42700737	0.151	0.128	0.04102561
Asnsd1	1.8797E-06	0.49066242	0.093	0.074	0.04123878
Caml	1.883E-06	0.76208645	0.049	0.035	0.04131166
Pak1ip1	1.8874E-06	0.43043896	0.207	0.186	0.04140848
Zfp51	2.0304E-06	0.51625238	0.038	0.026	0.04454438

Zkscan14	2.0629E-06	0.49997751	0.063	0.047	0.04525702
Med19	2.0629E-06	0.59668963	0.075	0.058	0.045258
1110038F14Ri	2.1088E-06	0.33593481	0.127	0.105	0.04626606
Nxn	2.1238E-06	0.91859975	0.028	0.018	0.04659299
Helb	2.1334E-06	0.43296863	0.075	0.057	0.04680384
Akip1	2.1692E-06	0.81333815	0.03	0.019	0.04759111
Cacybp	2.1921E-06	0.29873553	0.204	0.179	0.04809244
Cdr2	2.196E-06	0.68093411	0.044	0.03	0.04817837
Zfp426	2.3357E-06	0.65122328	0.028	0.017	0.051244
Mcph1	2.3781E-06	0.65646433	0.039	0.027	0.05217281
Apex2	2.41E-06	0.86266501	0.024	0.015	0.05287358
Klc1	2.5051E-06	0.46539809	0.082	0.064	0.05495997
Aktip	2.5712E-06	0.71996601	0.035	0.023	0.05640981
Papolg	2.6142E-06	0.42331618	0.066	0.05	0.05735224
Phc3	2.6161E-06	0.25598077	0.164	0.139	0.05739512
2210016F16Ri	2.6648E-06	0.60531973	0.08	0.063	0.05846226
Rpa3	2.8186E-06	0.48193581	0.07	0.053	0.06183725
Cyth2	2.9E-06	0.47523041	0.134	0.113	0.06362208
Nrbf2	2.9108E-06	0.4996012	0.127	0.106	0.06385954
Zfp955a	2.922E-06	1.23703334	0.01	0.005	0.06410515
Magi2	2.959E-06	0.73573184	0.012	0.006	0.06491701
Washc1	2.9947E-06	0.62181868	0.065	0.049	0.06569982
Enpp1	2.9984E-06	0.55620881	0.042	0.029	0.06578273
Rnf31	3.0044E-06	0.43116344	0.063	0.047	0.06591324
Hectd3	3.0067E-06	0.44990792	0.033	0.022	0.06596484
Usp25	3.1693E-06	0.25377439	0.288	0.264	0.06953229
Actr10	3.2134E-06	0.4551872	0.15	0.129	0.07049925
Zfp141	3.2783E-06	0.63296341	0.022	0.013	0.07192335
Slc45a4	3.282E-06	0.65579387	0.049	0.035	0.07200382
Igbp1	3.3069E-06	0.45336252	0.187	0.167	0.07254943
Golga7	3.3183E-06	0.42365714	0.171	0.15	0.07279999
Hexim1	3.3548E-06	0.28847533	0.203	0.178	0.07360011
Dnajc19	3.3886E-06	0.3936747	0.22	0.2	0.07434154
Ube2d1	3.399E-06	0.3873023	0.104	0.084	0.07457103
Pbx4	3.4208E-06	1.12333637	0.012	0.006	0.07504901
Scamp3	3.4533E-06	0.42225191	0.13	0.109	0.07576246
Rnf113a2	3.5633E-06	0.499427	0.072	0.055	0.07817622
Kcnab2	3.564E-06	0.51031578	0.069	0.052	0.07819138
Hars	3.6075E-06	0.54961436	0.089	0.071	0.07914594
Gm11131	3.6606E-06	0.7326765	0.018	0.01	0.08031024
Faap100	3.7743E-06	0.49370777	0.066	0.05	0.08280348
Dnaja2	3.8188E-06	0.25473635	0.343	0.325	0.08378123
Slc5a3	3.9134E-06	0.25146426	0.101	0.08	0.08585567
Thada	3.976E-06	0.7174459	0.056	0.041	0.08722892
Psmd9	4.0377E-06	0.50453267	0.109	0.09	0.08858334
Polr2d	4.044E-06	0.60211965	0.068	0.052	0.08872201
Rangap1	4.1086E-06	0.42019095	0.131	0.11	0.09013925
Slc7a6os	4.2175E-06	0.32805855	0.099	0.079	0.09252792

Fbxl6	4.2548E-06	0.67663359	0.059	0.044	0.09334674
Mlf2	4.2628E-06	0.47091601	0.171	0.152	0.09352238
Whamm	4.3267E-06	0.50526888	0.09	0.072	0.09492238
Dynlrb1	4.4396E-06	0.31520505	0.292	0.277	0.09740098
Psmc4	4.4711E-06	0.55511923	0.146	0.127	0.09809245
Emc6	4.5891E-06	0.32420628	0.246	0.227	0.10068014
Nprl2	4.6101E-06	0.93343384	0.024	0.015	0.10114205
Rbm43	4.8351E-06	0.40869891	0.05	0.036	0.10607729
Slc52a2	4.8422E-06	0.81625572	0.032	0.021	0.10623296
Zfp664	4.8518E-06	0.49858866	0.081	0.063	0.10644359
Ocel1	4.8565E-06	0.70269921	0.058	0.043	0.10654711
Mrps14	4.9567E-06	0.31017042	0.275	0.256	0.10874439
Ccdc124	4.9854E-06	0.4035505	0.169	0.148	0.10937407
Hist3h2ba	5.0465E-06	0.67096825	0.031	0.02	0.11071598
Zfp280b	5.128E-06	0.70885138	0.03	0.019	0.11250326
Ap5m1	5.1559E-06	0.52679842	0.059	0.044	0.1131151
Med12l	5.1893E-06	1.0043295	0.016	0.009	0.11384738
Phf13	5.2737E-06	0.87737753	0.038	0.026	0.11569925
Ptrh2	5.2874E-06	0.59241924	0.062	0.046	0.11599954
Myg1	5.304E-06	0.67399953	0.056	0.042	0.11636508
Smug1	5.3325E-06	0.60727396	0.028	0.018	0.11698884
Mzt1	5.3871E-06	0.50168484	0.107	0.088	0.11818671
Abhd10	5.4125E-06	0.56201649	0.055	0.041	0.11874397
Cep44	5.5573E-06	0.36694513	0.075	0.058	0.12192111
Atg5	5.6171E-06	0.44004871	0.107	0.088	0.12323404
Rab3gap1	5.7966E-06	0.30101425	0.193	0.169	0.12717215
Ccne2	6.0336E-06	0.99845484	0.017	0.01	0.13237024
Mettl4	6.0901E-06	0.92521282	0.019	0.011	0.13361003
Casc4	6.0945E-06	0.36955815	0.027	0.017	0.13370752
Dpm1	6.0985E-06	0.34689754	0.129	0.108	0.133795
Ccdc66	6.1305E-06	0.6288109	0.033	0.022	0.13449645
Abhd17a	6.2404E-06	0.50493613	0.136	0.117	0.13690887
Hdac8	6.3121E-06	0.62285336	0.029	0.019	0.13848159
Cdkn2a	6.3914E-06	1.16203851	0.013	0.007	0.14022096
Orc4	6.4241E-06	0.3537432	0.117	0.096	0.1409385
Dhx32	6.4313E-06	0.63374556	0.025	0.016	0.14109724
Fbxo34	6.5083E-06	0.54479401	0.117	0.098	0.1427861
Pkp4	6.6778E-06	0.49918833	0.055	0.041	0.14650354
Rnaset2b	6.7477E-06	0.58248739	0.109	0.091	0.14803673
Med31	6.7862E-06	0.34218564	0.1	0.081	0.14888139
4732440D04R	6.9738E-06	0.39810218	0.055	0.04	0.15299921
2510039O18R	7.0129E-06	0.48180585	0.103	0.084	0.1538565
Ubxn2a	7.0586E-06	0.25159954	0.206	0.182	0.15485762
Rer1	7.0872E-06	0.3507157	0.229	0.21	0.15548568
Gins4	7.1482E-06	0.65113156	0.066	0.051	0.15682416
Kptn	7.2164E-06	0.65386078	0.04	0.028	0.15832122
Atf2	7.2789E-06	0.3663866	0.159	0.137	0.15969259
H13	7.3969E-06	0.25606196	0.25	0.228	0.16228015

Sun1	7.4271E-06	0.58059219	0.053	0.039	0.16294361
Klf16	7.4534E-06	0.74693446	0.036	0.025	0.16352104
Zfp788	7.4747E-06	0.73127619	0.027	0.018	0.16398758
Psmd13	7.5672E-06	0.35169913	0.179	0.159	0.1660175
Capn1	7.6956E-06	0.39437241	0.107	0.088	0.16883381
Smim4	7.7619E-06	0.55312637	0.104	0.086	0.17028914
S100pbp	7.9466E-06	0.42263986	0.069	0.053	0.17433947
Jmy	7.9772E-06	0.4277302	0.177	0.156	0.17501167
Glipr2	8.1383E-06	0.40732096	0.09	0.072	0.17854544
Sart1	8.2266E-06	0.34634351	0.151	0.129	0.18048292
Abhd18	8.698E-06	0.43170661	0.043	0.03	0.1908263
Mrpl12	8.8174E-06	0.38759494	0.16	0.14	0.19344403
E4f1	8.8514E-06	0.42007843	0.097	0.078	0.19419037
Thoc3	9.0403E-06	0.58136538	0.071	0.055	0.19833587
Zbtb38	9.0535E-06	0.32685341	0.102	0.083	0.19862485
Cdh1	9.1885E-06	1.25420873	0.021	0.013	0.20158737
Capn15	9.2888E-06	0.69004634	0.055	0.041	0.20378604
Irf2bpl	9.4992E-06	0.51806743	0.111	0.092	0.20840319
Dgcr14	9.7919E-06	0.5673732	0.052	0.038	0.21482468
Ing5	9.8239E-06	0.40706591	0.14	0.12	0.21552608
Ing4	1.0247E-05	0.40536935	0.083	0.065	0.22479859
Cenpj	1.0323E-05	0.59843402	0.018	0.011	0.22646647
Slc35e2	1.034E-05	0.46431253	0.062	0.047	0.22684812
Armc1	1.0375E-05	0.4101781	0.089	0.072	0.22761501
Tnk2	1.0408E-05	0.36309856	0.054	0.04	0.22834668
Lrig1	1.0492E-05	0.3114309	0.028	0.018	0.23017561
Thap4	1.0562E-05	0.83802338	0.027	0.017	0.23172109
Gm20234	1.0664E-05	0.85196	0.017	0.009	0.23395575
Zfp330	1.0854E-05	0.43094121	0.15	0.13	0.23813347
Gm27166	1.1328E-05	0.44129708	0.011	0.005	0.24851445
Pard6a	1.1474E-05	0.70342636	0.038	0.027	0.25173393
Dtx3l	1.1695E-05	0.30902148	0.138	0.118	0.25658055
Gm26982	1.1846E-05	0.63354545	0.036	0.025	0.25987965
Dynll2	1.2457E-05	0.39025671	0.15	0.13	0.2733011
Gba2	1.2733E-05	0.7459907	0.019	0.011	0.27935985
Rnf6	1.2944E-05	0.36348093	0.189	0.169	0.2839826
Zbtb44	1.2946E-05	0.34636087	0.122	0.102	0.28402615
Ppm1b	1.3139E-05	0.292262	0.151	0.129	0.28824751
Adss	1.3252E-05	0.26706824	0.269	0.248	0.2907308
Med14	1.3255E-05	0.38953827	0.106	0.087	0.29080226
Zfp809	1.3259E-05	0.62307223	0.044	0.032	0.29089896
March5	1.3436E-05	0.3467071	0.2	0.179	0.29477493
Carmil2	1.3457E-05	0.3592018	0.035	0.024	0.29523431
Tmem183a	1.348E-05	0.36291259	0.124	0.104	0.29573669
Hid1	1.3703E-05	1.10094549	0.017	0.01	0.30063854
Ppil2	1.3728E-05	0.35768342	0.111	0.092	0.30117774
Ppip5k2	1.39E-05	0.30505263	0.07	0.054	0.30495302
Znrf2	1.4523E-05	0.36149931	0.222	0.202	0.31862984

Rbm10	1.4609E-05	0.55932475	0.065	0.05	0.32050099
Rnf146	1.4861E-05	0.36483406	0.149	0.128	0.32602481
Mrpl16	1.4902E-05	0.43893239	0.083	0.067	0.3269395
Tmem59	1.5138E-05	0.27415865	0.272	0.254	0.33212114
Clk3	1.551E-05	0.39085876	0.174	0.154	0.34028044
5033430I15Ril	1.5573E-05	0.69977624	0.018	0.011	0.34165955
Slc24a5	1.604E-05	0.59881766	0.025	0.016	0.35190471
Uty	1.6262E-05	0.39068695	0.066	0.05	0.35677171
Pdap1	1.6407E-05	0.29017308	0.298	0.281	0.35995982
Ppif	1.6488E-05	0.62399747	0.041	0.03	0.36173654
Lpcat4	1.656E-05	0.73859115	0.031	0.021	0.36331769
Ndufb10	1.6588E-05	0.26692731	0.345	0.332	0.36392459
3110002H16R	1.6767E-05	0.59302782	0.041	0.029	0.3678448
Jmjd7	1.6834E-05	1.16643791	0.011	0.006	0.36933168
Akap8	1.6845E-05	0.31939483	0.124	0.104	0.36955689
Gm30025	1.7136E-05	0.85982489	0.019	0.012	0.37595193
Ly6g5b	1.7353E-05	0.54685289	0.021	0.013	0.38070305
Impdh1	1.7604E-05	0.45569322	0.07	0.055	0.38621923
Gabarapl1	1.7776E-05	0.4449888	0.187	0.169	0.3899886
Insl6	1.7971E-05	0.57626785	0.062	0.048	0.39427518
Spcs1	1.85E-05	0.28543493	0.299	0.285	0.40586743
Smyd3	1.8547E-05	0.70947012	0.037	0.026	0.40691277
Gfi1	1.9818E-05	0.28235659	0.045	0.033	0.43477728
Auh	1.9903E-05	0.46581678	0.148	0.13	0.43664626
Pms1	2.0046E-05	0.86274374	0.018	0.011	0.43977985
Fbxo46	2.06E-05	0.72024213	0.026	0.017	0.45194384
Asrgl1	2.0622E-05	0.4682518	0.047	0.034	0.45241555
Calcoco1	2.0677E-05	0.49146598	0.042	0.03	0.45363807
Lrrc41	2.0773E-05	0.49723208	0.08	0.064	0.4557439
Ap1ar	2.0778E-05	0.46399755	0.079	0.063	0.4558386
Vps4a	2.0823E-05	0.51058952	0.095	0.078	0.45683078
Ubp1	2.1081E-05	0.43955959	0.135	0.116	0.46248685
Rasa1	2.1548E-05	0.38634258	0.112	0.093	0.47273935
Mrpl35	2.201E-05	0.49785639	0.113	0.095	0.48288618
Fgf13	2.2288E-05	0.72907442	0.011	0.006	0.4889689
Rsl24d1	2.272E-05	0.39100893	0.137	0.117	0.49845845
Foxo4	2.2764E-05	0.59579326	0.029	0.019	0.49941305
Zmat5	2.2828E-05	0.58610715	0.084	0.068	0.50081821
Usp42	2.3176E-05	0.38698454	0.056	0.042	0.50845889
Kbtbd4	2.4E-05	0.6856298	0.027	0.018	0.52654144
Ttc4	2.4387E-05	0.4186767	0.066	0.051	0.53501986
Rexo5	2.4915E-05	0.65484673	0.019	0.011	0.54661363
Insig2	2.4984E-05	0.28336848	0.068	0.053	0.54812716
Pik3cd	2.5022E-05	0.26978962	0.169	0.147	0.54895424
Tmem115	2.587E-05	0.74107527	0.043	0.031	0.56755975
Klhdc1	2.6638E-05	0.60472121	0.024	0.015	0.58440135
Rps6kb1	2.6893E-05	0.28514054	0.189	0.167	0.59000251
Mbtps1	2.733E-05	0.31802664	0.153	0.132	0.59958955

Ak3	2.7783E-05	0.76643119	0.046	0.034	0.60953122
Maml2	2.8212E-05	0.49442391	0.049	0.036	0.61894649
Pgrmc2	2.8577E-05	0.50333189	0.097	0.08	0.6269483
Rbak	2.8752E-05	0.85318454	0.017	0.01	0.6307795
A230050P20R	2.9281E-05	0.68407177	0.024	0.016	0.64239187
Cct5	2.9321E-05	0.27677773	0.312	0.298	0.64328085
Zfand2b	3.0033E-05	0.46738714	0.096	0.079	0.6588841
Rpusd3	3.0131E-05	0.97070062	0.011	0.006	0.66105005
Trp53i13	3.0552E-05	0.63238862	0.031	0.021	0.67028224
Mcm9	3.0616E-05	0.46538996	0.052	0.039	0.67168573
Txnl1	3.0701E-05	0.30496972	0.251	0.234	0.67353948
Qsox1	3.1306E-05	0.42512318	0.077	0.061	0.68681144
Stn1	3.137E-05	0.53868045	0.054	0.041	0.68823508
Pdcd7	3.2218E-05	0.43986002	0.069	0.054	0.70683588
Lxn	3.2491E-05	0.6489671	0.035	0.025	0.71282689
Tpp2	3.2857E-05	0.2712725	0.292	0.274	0.72085163
2410089E03R	3.3309E-05	0.40209493	0.069	0.054	0.73077396
Suds3	3.3379E-05	0.41589009	0.131	0.113	0.73230043
Zmym1	3.3747E-05	0.58810611	0.047	0.035	0.74037342
Bscl2	3.3859E-05	0.5635828	0.116	0.1	0.74284237
Egln1	3.3937E-05	0.40758104	0.12	0.102	0.74454199
Mrpl50	3.4005E-05	0.51187245	0.077	0.062	0.7460354
Idh3b	3.5577E-05	0.36604132	0.194	0.176	0.78053173
Frmd8	3.5591E-05	0.69688689	0.043	0.031	0.78082385
Rrp36	3.6028E-05	0.62456205	0.051	0.038	0.79042207
Gna11	3.778E-05	0.7870399	0.03	0.021	0.82885544
Rab33b	3.7851E-05	0.5949803	0.056	0.042	0.83041417
Glmn	3.8481E-05	0.58399351	0.024	0.015	0.84424386
Lin37	3.9404E-05	0.64159764	0.046	0.034	0.86448438
B3gat2	4.0276E-05	0.58650447	0.022	0.014	0.88362015
Smarcad1	4.0804E-05	0.32421087	0.138	0.118	0.89520451
Zscan21	4.0866E-05	0.63040126	0.029	0.019	0.89656273
Coil	4.1212E-05	0.69194421	0.027	0.018	0.90415985
Pafah1b2	4.1215E-05	0.35251213	0.121	0.102	0.90422525
Slc1a5	4.1227E-05	0.38052636	0.144	0.125	0.9044888
Unc13d	4.1648E-05	0.41059845	0.052	0.039	0.91370713
Wrap73	4.2637E-05	0.73036271	0.029	0.02	0.93540239
Ppid	4.3513E-05	0.46444242	0.082	0.066	0.95462546
Uba6	4.4454E-05	0.3896591	0.119	0.101	0.97526626
Eml4	4.4795E-05	0.25900158	0.228	0.206	0.9827533
Tmsb15b1	4.6103E-05	0.5003086	0.03	0.02	1
Zfp677	4.6606E-05	0.5525072	0.023	0.015	1
Wdcp	4.6695E-05	0.66752118	0.023	0.015	1
Spopl	4.7366E-05	0.27430107	0.101	0.084	1
Tomm34	4.7756E-05	0.41451362	0.113	0.096	1
Prkrip1	4.7767E-05	0.48631672	0.099	0.083	1
Rbm17	4.786E-05	0.32203138	0.222	0.205	1
Cd200r4	4.9365E-05	0.84457818	0.032	0.022	1

Ube2g1	4.9522E-05	0.48512979	0.095	0.078	1
Gm15283	5.0391E-05	0.60735864	0.028	0.019	1
B3galt4	5.0666E-05	0.93072864	0.014	0.008	1
Smim19	5.1881E-05	0.55694965	0.078	0.063	1
Itih5	5.2848E-05	0.45410475	0.023	0.015	1
Ddx20	5.3294E-05	0.56605819	0.039	0.028	1
Zfp287	5.3617E-05	0.62592652	0.017	0.01	1
Fbxw4	5.3662E-05	0.77059628	0.036	0.026	1
Cpped1	5.4088E-05	0.83290746	0.023	0.015	1
Aasdhppt	5.5095E-05	0.47174869	0.073	0.058	1
Ankib1	5.601E-05	0.33392396	0.114	0.096	1
Mbip	5.6846E-05	0.57199903	0.051	0.038	1
Surf2	5.9916E-05	0.64434366	0.043	0.031	1
Rint1	6.1778E-05	0.56401442	0.041	0.03	1
Nudcd2	6.2228E-05	0.52785399	0.088	0.073	1
Hmces	6.3984E-05	0.30378136	0.105	0.087	1
Las1l	6.4215E-05	0.27018657	0.129	0.11	1
Fbxo3	6.5045E-05	0.41156864	0.092	0.075	1
Isg20l2	6.6208E-05	0.54052134	0.089	0.073	1
Msh2	6.7073E-05	0.39979699	0.044	0.032	1
Mphosph8	6.7812E-05	0.27901125	0.114	0.096	1
C730034F03R	7.1037E-05	0.61096654	0.03	0.021	1
Dkkl1	7.1353E-05	0.41057619	0.041	0.03	1
Ola1	7.7958E-05	0.45450014	0.172	0.156	1
Tbc1d8b	8.0307E-05	0.48292106	0.03	0.02	1
Gm20721	8.09E-05	0.82629907	0.013	0.007	1
Eif4a3	8.0989E-05	0.33122958	0.186	0.168	1
6030458C11R	8.2017E-05	0.7033782	0.032	0.023	1
Zfpl1	8.2213E-05	0.59705154	0.047	0.036	1
Atp8b2	8.2914E-05	0.54100701	0.046	0.035	1
Hmgxb4	8.4234E-05	0.53891205	0.058	0.045	1
Rnf181	8.6629E-05	0.42133153	0.095	0.079	1
Zfp84	8.7751E-05	0.43152553	0.063	0.05	1
Mbp	8.8104E-05	0.33377522	0.171	0.152	1
Ptpn7	8.9608E-05	0.29015768	0.075	0.06	1
Cers5	8.9911E-05	0.44392254	0.102	0.086	1
Prr14	9.479E-05	0.36193334	0.114	0.097	1
Zfp24	9.6104E-05	0.51536133	0.075	0.06	1
Gm26699	9.7006E-05	0.37799464	0.059	0.046	1
Ubr7	9.7189E-05	0.49030306	0.075	0.061	1
Ifi35	9.7488E-05	0.56339933	0.103	0.088	1
Cuta	9.7885E-05	0.36224999	0.224	0.209	1
Shprh	9.9001E-05	0.32862228	0.052	0.04	1
Mmadhc	0.00010149	0.45231281	0.117	0.101	1
Urod	0.00010172	0.69559773	0.042	0.031	1
Ksr1	0.00010185	0.57250821	0.031	0.022	1
Zw10	0.00010383	0.51034791	0.038	0.028	1
Zfp68	0.00010501	0.34997008	0.087	0.071	1

2610008E11R	0.00010562	0.50371658	0.038	0.028	1
Gdpd5	0.00010606	0.5401514	0.022	0.014	1
Txn2	0.00010659	0.37072937	0.182	0.166	1
Mid1ip1	0.00010928	0.32320344	0.052	0.04	1
Hsdl2	0.00010996	0.70404049	0.05	0.038	1
Ptch1	0.0001103	0.48915865	0.037	0.027	1
Wtap	0.00011233	0.31958361	0.214	0.197	1
Cbll1	0.00012062	0.38011584	0.129	0.112	1
Tomm70a	0.00012091	0.34345689	0.178	0.161	1
Lamtor1	0.00012305	0.35453155	0.175	0.159	1
Aimp2	0.00012452	0.53291695	0.038	0.028	1
Gm27003	0.00012491	0.69038264	0.012	0.007	1
Plaa	0.00013095	0.45151456	0.14	0.123	1
Atg4d	0.00013098	0.50025903	0.057	0.045	1
Elavl1	0.00013382	0.25581206	0.246	0.229	1
Ddx47	0.00013552	0.33410828	0.175	0.157	1
Polr3a	0.00013864	0.55453583	0.049	0.037	1
Atxn7l2	0.000139	0.66389441	0.023	0.015	1
Mtbp	0.00013999	0.78143929	0.022	0.015	1
Dguok	0.00014095	0.37086907	0.101	0.085	1
Fancm	0.00014104	0.46314187	0.029	0.02	1
Gm20342	0.00014239	0.55895312	0.031	0.022	1
Gon4l	0.00014297	0.41444111	0.083	0.068	1
Nsdhl	0.00014358	0.92656832	0.035	0.025	1
Actr8	0.0001441	0.50982029	0.043	0.032	1
Naa35	0.00014452	0.39077355	0.09	0.075	1
Gosr1	0.00014471	0.40796939	0.085	0.07	1
Exo5	0.00014606	0.73619965	0.016	0.01	1
Pfdn6	0.00014618	0.46283785	0.169	0.154	1
Btd	0.00014622	0.75083183	0.025	0.017	1
Dcp1b	0.00014721	0.77281399	0.019	0.012	1
Gdap2	0.00014734	0.55039815	0.056	0.044	1
Med12	0.00014819	0.3412221	0.086	0.071	1
Cox11	0.00014834	0.48102085	0.041	0.031	1
Glrx2	0.00014987	0.44092575	0.138	0.123	1
Ddx39	0.00015026	0.34319218	0.1	0.084	1
Arl1	0.0001506	0.39524664	0.177	0.162	1
Gtf2h2	0.00015061	0.61581642	0.03	0.021	1
Ctu2	0.00015276	0.55506498	0.054	0.041	1
2300009A05R	0.00015278	0.4698988	0.087	0.072	1
Fam173a	0.00015303	0.37501517	0.172	0.156	1
Zfp865	0.00015413	0.41713581	0.05	0.038	1
Exoc3	0.00015478	0.36762047	0.172	0.155	1
Luc7l	0.00015531	0.32109605	0.144	0.126	1
Tmem2	0.00015675	0.52399592	0.069	0.055	1
Herc2	0.00015825	0.28386989	0.152	0.134	1
Tmx4	0.00015901	0.32574853	0.147	0.13	1
Hmbs	0.0001647	0.7110204	0.033	0.023	1

9330175E14R	0.00016516	0.47480291	0.015	0.009	1
Mtfr1	0.00016611	0.57892747	0.038	0.028	1
B4galnt4	0.00016771	0.65444252	0.019	0.012	1
Ipp	0.00017102	0.6878958	0.017	0.01	1
Xpo4	0.0001727	0.32169986	0.108	0.091	1
Nsun4	0.00017293	0.40209423	0.058	0.045	1
Gm26522	0.00017566	0.74752751	0.025	0.017	1
Zbtb49	0.0001757	0.74137579	0.019	0.012	1
Ier3ip1	0.00017695	0.26407152	0.312	0.301	1
Dolpp1	0.00017819	0.7678407	0.017	0.01	1
Ppp3cb	0.00017964	0.26438465	0.182	0.163	1
Nrf1	0.00018225	0.36691446	0.096	0.08	1
Cep76	0.00018297	0.75200973	0.02	0.013	1
Klhl15	0.00018887	0.75101023	0.039	0.029	1
Eif1ax	0.00018945	0.34694561	0.228	0.214	1
Aqr	0.00019017	0.31860337	0.166	0.148	1
Med22	0.00019219	0.82377391	0.026	0.018	1
Coq10a	0.00020261	0.70684273	0.036	0.026	1
Tatdn2	0.00020662	0.36231957	0.19	0.173	1
Dnajc17	0.00020967	0.65147522	0.025	0.017	1
Gopc	0.0002098	0.36855771	0.059	0.046	1
Polr2k	0.00021243	0.29705207	0.273	0.26	1
Dpy19l3	0.00021276	0.44401337	0.045	0.034	1
Pacs1	0.00021313	0.33070259	0.072	0.058	1
Ccdc85b	0.00021919	0.82152221	0.014	0.008	1
Nelfa	0.00022538	0.44658227	0.057	0.045	1
Snhg20	0.00022547	0.36094021	0.06	0.047	1
Zfp275	0.00022759	0.44200065	0.031	0.022	1
AW146154	0.00022788	0.67223941	0.01	0.006	1
Nubp1	0.00022933	0.41167195	0.134	0.118	1
Ccl25	0.00023396	0.46018746	0.036	0.027	1
Acat2	0.00023422	0.70615082	0.027	0.018	1
Sugp1	0.00023664	0.3122793	0.072	0.059	1
Ubxn8	0.00024039	0.53141089	0.061	0.049	1
Rdh14	0.00024201	0.54059691	0.035	0.025	1
Gid8	0.0002442	0.43095831	0.089	0.074	1
Thap7	0.00024481	0.68642583	0.052	0.041	1
Cog2	0.00024791	0.45850728	0.087	0.073	1
Ppp6r1	0.00025293	0.34468891	0.186	0.169	1
Zfp738	0.00025489	0.48202774	0.043	0.032	1
Csnk1g1	0.00025885	0.25779823	0.097	0.081	1
Rbbp7	0.00025936	0.27712155	0.2	0.182	1
Fbxo33	0.00025938	0.27257657	0.134	0.117	1
Zfp827	0.00026161	0.69545841	0.01	0.006	1
Riox1	0.00026634	0.56472917	0.033	0.024	1
Hist1h1d	0.0002682	0.25746931	0.082	0.067	1
P2rx7	0.0002711	0.55701166	0.06	0.048	1
Cmc4	0.00027129	0.68572992	0.023	0.015	1

Fbxw7	0.00027205	0.34040805	0.091	0.076	1
2610021A01R	0.00027741	0.62549029	0.019	0.013	1
Snrpa1	0.00027886	0.31478322	0.155	0.138	1
Dbt	0.00028023	0.49533531	0.046	0.035	1
Cry1	0.0002823	0.35528488	0.093	0.078	1
Dock8	0.00028432	0.25506626	0.189	0.171	1
Xlr4a	0.00028524	0.40909565	0.039	0.029	1
Yeats4	0.00028549	0.25996872	0.23	0.214	1
Pdcl	0.00028766	0.4051326	0.082	0.068	1
Brms1	0.00029158	0.57866248	0.078	0.065	1
Mitd1	0.00029517	0.34781682	0.063	0.05	1
Zfp825	0.00030057	0.82945066	0.017	0.011	1
Acads	0.00030468	0.48885919	0.055	0.044	1
Kars	0.00031429	0.45775095	0.111	0.096	1
Tob1	0.00031514	0.49487404	0.091	0.077	1
Dnm2	0.00031743	0.29705239	0.202	0.186	1
Tprn	0.00031879	0.79622974	0.018	0.012	1
Hmgn5	0.00032174	0.29057842	0.132	0.115	1
Dgcr2	0.00032449	0.4580836	0.068	0.055	1
Josd1	0.00033178	0.40658198	0.096	0.081	1
Cdk2ap1	0.0003322	0.38010056	0.127	0.112	1
Scoc	0.00033228	0.48679419	0.052	0.041	1
Cog1	0.00034636	0.71322644	0.04	0.031	1
Zfp180	0.00034771	0.39419753	0.053	0.042	1
Zfp12	0.00034814	0.83435061	0.014	0.008	1
Cdkn3	0.0003583	0.8862784	0.01	0.006	1
Rnf4	0.00036205	0.26980975	0.239	0.224	1
Mars	0.00036356	0.64059299	0.049	0.038	1
Maea	0.00036446	0.38801853	0.122	0.106	1
Bcr	0.00036924	0.4881944	0.04	0.03	1
Clpp	0.00037422	0.57331144	0.057	0.045	1
Srpr	0.00038238	0.26708052	0.29	0.28	1
Mrps9	0.00038241	0.4726787	0.119	0.104	1
Trmt13	0.00038535	0.35883415	0.075	0.061	1
Casp2	0.00039196	0.31994534	0.055	0.043	1
Trabd	0.00039774	0.37391035	0.098	0.083	1
Xpo7	0.00040285	0.3795707	0.078	0.064	1
A430078G23R	0.00040294	0.43455673	0.027	0.019	1
Irf3	0.0004069	0.29237758	0.095	0.08	1
Rit1	0.00040852	0.56557165	0.052	0.041	1
Prdm11	0.00041098	0.61143829	0.025	0.017	1
Fbxl8	0.00041599	0.74850432	0.017	0.011	1
Fcho1	0.00041899	0.33287077	0.044	0.034	1
Slc25a30	0.00041914	0.5087425	0.078	0.065	1
Wdr20	0.00042106	0.31283801	0.102	0.087	1
Rnf44	0.00042187	0.30213465	0.127	0.111	1
Guk1	0.00042226	0.36314801	0.115	0.099	1
Mtfmt	0.00042873	0.49754341	0.031	0.022	1

Strap	0.00043371	0.33254984	0.197	0.183	1
Timm8b	0.00043563	0.30228182	0.201	0.187	1
Nmd3	0.00044157	0.41541951	0.106	0.091	1
Fkbpl	0.00044278	0.97883248	0.011	0.007	1
Pdik1l	0.00044357	0.55196687	0.033	0.024	1
Hjurp	0.00044416	0.44441004	0.098	0.084	1
Eif4enif1	0.00045255	0.25988511	0.076	0.062	1
Ift27	0.00046377	0.54211527	0.037	0.028	1
Polr3c	0.0004696	0.39198545	0.095	0.081	1
Knstrn	0.00047822	0.62451426	0.019	0.013	1
Susd1	0.0004837	0.64758186	0.02	0.013	1
Zfp322a	0.00048467	0.38216335	0.081	0.068	1
Acd	0.00048598	0.41332713	0.072	0.059	1
Cars	0.00048742	0.37604456	0.103	0.088	1
Atf1	0.00048954	0.27922372	0.177	0.161	1
Armc3	0.00049188	0.53892514	0.011	0.006	1
Tmem60	0.00049459	0.28906057	0.082	0.068	1
Haus2	0.00049866	0.45065056	0.051	0.04	1
Eef1e1	0.00051133	0.4017231	0.117	0.102	1
Tm2d3	0.00051998	0.3960004	0.08	0.067	1
Zfp770	0.00053021	0.55504065	0.028	0.02	1
Ccdc82	0.00053221	0.27690513	0.109	0.093	1
Mcm3ap	0.00053367	0.36590012	0.051	0.04	1
Ifit1bl1	0.00053949	0.81062049	0.012	0.007	1
Faap20	0.00054316	0.41999923	0.057	0.045	1
Tg	0.00055077	0.55079361	0.012	0.007	1
Rnf139	0.00055321	0.40051567	0.086	0.073	1
Erlec1	0.00055928	0.33124352	0.12	0.105	1
Synj2	0.00056056	0.50574985	0.048	0.037	1
Mms19	0.0005637	0.35958214	0.075	0.062	1
Hsd17b10	0.00056936	0.43768577	0.123	0.109	1
Herpud2	0.00058848	0.33444807	0.125	0.109	1
Ighmbp2	0.00058887	0.62335918	0.025	0.017	1
Uba2	0.00059063	0.27283475	0.123	0.108	1
Dhrs1	0.00060963	0.70654783	0.067	0.056	1
Zfp362	0.00061317	0.56032837	0.046	0.036	1
Sos2	0.00062473	0.49538305	0.07	0.058	1
Zfp7	0.00062652	0.72236347	0.011	0.007	1
Rnaseh2c	0.00063033	0.3129532	0.164	0.149	1
Adnp	0.00063507	0.5575442	0.042	0.032	1
Plpp6	0.00063743	0.57833649	0.036	0.027	1
Xpot	0.00064587	0.37748022	0.068	0.056	1
Gm26625	0.00064715	0.93341047	0.01	0.006	1
Vopp1	0.00065691	0.48850578	0.089	0.075	1
Ppil3	0.00065856	0.42543788	0.065	0.053	1
Hinfp	0.00066308	0.44184914	0.055	0.044	1
Mrpl19	0.00066616	0.38331303	0.101	0.087	1
Adat2	0.00067895	0.60970002	0.031	0.023	1

Trappc8	0.00068229	0.41444156	0.099	0.085	1
Llgl2	0.00068469	0.57881601	0.013	0.008	1
Chchd3	0.00069334	0.3405741	0.156	0.142	1
Kcnk5	0.00069795	0.6445953	0.018	0.012	1
Ube4b	0.00069801	0.33032117	0.082	0.069	1
Ckap2	0.00070393	1.01097513	0.012	0.008	1
Tpgs1	0.0007263	0.48476696	0.058	0.047	1
Orc2	0.0007331	0.44082723	0.06	0.048	1
Msantd2	0.00074192	0.56077921	0.046	0.036	1
Zfp850	0.00074927	0.8320432	0.011	0.006	1
Adgrl1	0.00075183	0.37195366	0.051	0.04	1
Il15ra	0.00075543	0.6270107	0.027	0.02	1
Gtpbp2	0.00075672	0.31050106	0.15	0.134	1
Mcm5	0.00075762	0.41425582	0.049	0.038	1
Nudt1	0.00075822	0.79591298	0.018	0.012	1
Ccs	0.00076938	0.56444442	0.064	0.053	1
Mypopos	0.00077387	0.65246147	0.012	0.008	1
Ercc1	0.00078669	0.59160686	0.036	0.027	1
Phf8	0.00079435	0.33424399	0.096	0.082	1
Zfp101	0.00079525	0.46625147	0.026	0.018	1
Gm9844	0.0007983	0.55688793	0.038	0.029	1
Inpp5k	0.00079994	0.41128816	0.072	0.06	1
Gm26782	0.00080421	0.509793	0.023	0.016	1
Sf3a1	0.00080556	0.33309941	0.115	0.1	1
Ankrd54	0.00081454	0.60341739	0.043	0.034	1
Pcbp4	0.00081546	0.68017554	0.017	0.011	1
Bod1	0.00081751	0.64325603	0.039	0.03	1
Prelid3b	0.00082703	0.45504078	0.137	0.124	1
Cdk6	0.00083579	0.321945	0.141	0.126	1
Mettl14	0.00084156	0.34002563	0.06	0.049	1
Gskip	0.00084493	0.42856154	0.082	0.069	1
Pycard	0.00085642	0.37766715	0.202	0.191	1
Dopey2	0.00085901	0.41998524	0.055	0.044	1
Fut11	0.0008713	0.56445239	0.03	0.022	1
Katnb1	0.0008866	0.71603851	0.02	0.014	1
Ugdh	0.00090027	0.35289131	0.071	0.059	1
Ttll12	0.00091466	0.60345722	0.022	0.015	1
Sec23b	0.00091987	0.31989868	0.139	0.124	1
Zmynd19	0.00092399	0.61105465	0.033	0.024	1
Ret	0.00093181	0.37310157	0.017	0.011	1
Casc3	0.00093358	0.25216491	0.182	0.166	1
Lyar	0.00093404	0.29563414	0.158	0.143	1
Eef1akmt1	0.00094727	0.59152747	0.039	0.03	1
Wrap53	0.00095725	0.80469963	0.017	0.011	1
Tgs1	0.00096592	0.34675506	0.13	0.115	1
Rprd1b	0.00097024	0.30978534	0.104	0.089	1
Zfp341	0.00097223	0.83416537	0.011	0.006	1
Cep170b	0.00097672	0.79311742	0.017	0.011	1

Ulk2	0.00098047	0.58573761	0.055	0.044	1
Mterf4	0.00099167	0.58911395	0.024	0.017	1
AU041133	0.00099618	0.44396778	0.023	0.016	1
Skiv2l	0.00100095	0.33450669	0.088	0.075	1
Ip6k2	0.00100396	0.39496627	0.049	0.039	1
2700097O09R	0.00100622	0.29290697	0.038	0.029	1
Zfp369	0.00102812	0.42958573	0.033	0.025	1
Deaf1	0.00103684	0.61787059	0.02	0.014	1
Heatr5b	0.00104286	0.6551639	0.034	0.026	1
Cdc23	0.00104903	0.28130195	0.036	0.027	1
Arl16	0.0010502	0.81907786	0.019	0.013	1
Zfp524	0.00105174	0.68599032	0.047	0.038	1
Naa16	0.00105891	0.5095413	0.054	0.044	1
Ilvbl	0.00106087	0.52481083	0.05	0.04	1
Rad1	0.00107324	0.61100996	0.022	0.015	1
Fcf1	0.00107394	0.38048264	0.13	0.117	1
Cfap97	0.00107713	0.29672183	0.059	0.048	1
Med26	0.00108169	0.47257647	0.034	0.025	1
Tbcb	0.00109108	0.27139236	0.219	0.207	1
Phkg2	0.00109137	0.39564223	0.09	0.077	1
Rpia	0.00109502	0.3528641	0.073	0.061	1
Elf4	0.00109822	0.3520475	0.109	0.095	1
Mrpl22	0.00110211	0.62109305	0.045	0.036	1
Oxld1	0.00110538	0.97120751	0.013	0.008	1
Med21	0.00111539	0.26651577	0.126	0.112	1
Zfp787	0.0011156	0.38937922	0.09	0.078	1
Tspyl3	0.00112656	0.85838494	0.012	0.007	1
Papd5	0.00112723	0.36177053	0.093	0.08	1
Rab2b	0.00113658	0.43217319	0.052	0.042	1
Zfp868	0.00114021	0.38121985	0.064	0.053	1
Parl	0.00114268	0.39272704	0.12	0.107	1
Zfp799	0.00115098	0.76379292	0.018	0.012	1
Impad1	0.00116261	0.35073076	0.111	0.097	1
Tysnd1	0.0011639	0.56371215	0.023	0.016	1
1700056N10R	0.00116616	1.00152177	0.01	0.006	1
Etfb	0.00116719	0.42461233	0.172	0.161	1
Sec23a	0.00117779	0.46717078	0.041	0.032	1
Tmem42	0.00120071	0.49798972	0.037	0.028	1
Zbtb33	0.00120948	0.50275007	0.062	0.051	1
2010300C02R	0.00122776	0.36175759	0.011	0.006	1
Erc1	0.0012309	0.38437891	0.033	0.025	1
Tbc1d22b	0.00123327	0.62200202	0.027	0.019	1
Itgb3bp	0.00123739	0.31282617	0.033	0.025	1
Trappc13	0.00124852	0.37557742	0.06	0.049	1
Sprtn	0.0012616	0.48839139	0.048	0.038	1
Gsk3a	0.00130704	0.27337598	0.161	0.146	1
Tlcd1	0.0013102	0.52581285	0.023	0.016	1
Inpp5b	0.0013156	0.58373087	0.039	0.03	1

Psmd12	0.00132689	0.32899675	0.193	0.181	1
Caap1	0.00133423	0.38388037	0.068	0.056	1
Vdac3	0.00133591	0.39382552	0.166	0.154	1
Rhebl1	0.00134231	0.56914333	0.016	0.01	1
Cnp	0.00135337	0.31464656	0.08	0.068	1
Dhps	0.00136073	0.41566168	0.12	0.107	1
Zfp952	0.00136698	0.53025428	0.033	0.025	1
Ptar1	0.00136866	0.52680556	0.023	0.016	1
3110040N11R	0.00137437	0.39603659	0.038	0.029	1
Fermt3	0.00137568	0.27411828	0.212	0.201	1
Ddx28	0.00138584	0.4030828	0.034	0.026	1
Atxn2l	0.00140623	0.29309351	0.234	0.222	1
Arel1	0.00141064	0.34492621	0.056	0.045	1
Hs3st3b1	0.00141218	0.67045813	0.033	0.025	1
Dnlz	0.00141672	0.42488197	0.069	0.057	1
2310011J03Ril	0.00142305	0.44369488	0.097	0.084	1
Vax2	0.00144383	0.40136159	0.02	0.014	1
Oraov1	0.00144595	0.5722704	0.051	0.041	1
Ddx27	0.00145329	0.33643146	0.121	0.107	1
Zfp580	0.00147556	0.81113189	0.014	0.009	1
Rab11fip2	0.00152125	0.31864852	0.09	0.077	1
Hddc2	0.0015218	0.47016588	0.049	0.039	1
Prpsap1	0.0015548	0.44373499	0.083	0.071	1
Bora	0.00155694	0.42338776	0.028	0.02	1
Snrnp200	0.00157	0.27034985	0.184	0.169	1
Rnf114	0.00160173	0.26456852	0.205	0.191	1
Usp39	0.00162431	0.41470858	0.071	0.059	1
Ndufaf5	0.00163724	0.81523445	0.019	0.013	1
Zfp830	0.00173384	0.32992346	0.1	0.087	1
Gbf1	0.00174108	0.3447926	0.087	0.075	1
Setd3	0.0017496	0.28460643	0.154	0.14	1
Tmem238	0.00175259	0.72939838	0.046	0.037	1
Cyb561d1	0.00181259	0.49268454	0.032	0.024	1
Nabp2	0.00183668	0.49440053	0.075	0.064	1
Pdpr	0.00184312	0.41127836	0.044	0.035	1
Rex1bd	0.00185556	0.32468349	0.182	0.17	1
Wdtc1	0.00187463	0.39527539	0.063	0.052	1
Ndufaf3	0.00188226	0.6348678	0.059	0.049	1
Sigirr	0.00188959	0.37592631	0.037	0.029	1
Mrpl34	0.00189623	0.43805614	0.115	0.103	1
Ssr2	0.00189723	0.34247211	0.185	0.173	1
9130221H12R	0.00190005	0.50011307	0.011	0.007	1
Bcas3	0.00193149	0.4923241	0.045	0.036	1
Dpy30	0.00194444	0.34623244	0.096	0.084	1
Alg5	0.00194966	0.47755467	0.095	0.083	1
Rnf7	0.00195373	0.27761963	0.221	0.211	1
Ppm1h	0.00196671	0.44429534	0.098	0.086	1
Immp1l	0.00198924	0.32577827	0.088	0.076	1

B3galt5	0.00199881	0.30757469	0.033	0.025	1
AC163032.1	0.00201124	0.42034611	0.03	0.022	1
Ppp2r5e	0.0020237	0.35030377	0.107	0.094	1
Tmem97	0.0020276	0.63373714	0.038	0.029	1
Fam173b	0.00204214	0.8150571	0.034	0.026	1
Pcnx	0.00206782	0.39346055	0.079	0.067	1
Mettl17	0.00206856	0.38796476	0.025	0.018	1
Gm8186	0.0021024	0.42811493	0.088	0.076	1
Zscan2	0.00214961	0.50715409	0.017	0.011	1
Ahsa2	0.00217431	0.31474465	0.057	0.046	1
Arl15	0.0021752	0.51419113	0.035	0.027	1
Gtf3c6	0.00219445	0.41277814	0.112	0.1	1
Nipsnap1	0.00222397	0.45722534	0.024	0.017	1
Chchd7	0.00230173	0.41203973	0.101	0.089	1
Pou6f1	0.00230485	0.45017834	0.037	0.028	1
Mpc2	0.00233814	0.31727197	0.26	0.253	1
Zfp668	0.00236038	0.78063997	0.016	0.011	1
Eif2s1	0.00242487	0.30575211	0.207	0.196	1
Ubald1	0.0024875	0.39902624	0.136	0.123	1
Donson	0.00252423	0.34643926	0.094	0.082	1
Ccl27a	0.00252562	0.97743984	0.013	0.008	1
Polr2b	0.00254937	0.38169386	0.08	0.068	1
Anapc7	0.00255799	0.37642236	0.067	0.056	1
Eed	0.00260129	0.25446463	0.129	0.115	1
Fxn	0.00262244	0.55144961	0.044	0.035	1
Cops6	0.00263288	0.36919421	0.147	0.135	1
Hira	0.00265511	0.50755664	0.039	0.031	1
E130308A19R	0.00265892	0.27048373	0.034	0.026	1
Snrnp25	0.00269918	0.37798763	0.069	0.058	1
2610044O15R	0.00270171	0.5935385	0.018	0.013	1
Wdr47	0.00275647	0.42172421	0.027	0.021	1
Ckap5	0.00276351	0.3540658	0.099	0.087	1
Lias	0.0028798	0.33667401	0.072	0.06	1
Mpg	0.00289883	0.47221564	0.05	0.041	1
Ppp2r5d	0.00291815	0.4428998	0.045	0.036	1
Arpc1a	0.00293011	0.41010424	0.143	0.132	1
Hps6	0.00293305	0.84531315	0.02	0.015	1
Tapt1	0.0029413	0.34911007	0.061	0.051	1
Tmem87a	0.00296663	0.34921977	0.096	0.084	1
Nr1d2	0.00301416	0.29617332	0.186	0.173	1
Zbtb8os	0.00303176	0.40261948	0.1	0.089	1
Haspin	0.00311127	0.75873251	0.011	0.007	1
Exosc3	0.00311931	0.27537268	0.122	0.109	1
Dcun1d2	0.00313867	0.69292075	0.014	0.01	1
Zfp729b	0.00317296	0.30730301	0.048	0.039	1
Timm10b	0.00320692	0.35876771	0.207	0.198	1
Ubtd2	0.00322596	0.51978147	0.021	0.015	1
Erf	0.00322656	0.48466283	0.039	0.031	1

Nup37	0.00322995	0.78182095	0.013	0.009	1
R3hcc1	0.00332826	0.39782763	0.022	0.016	1
Snf8	0.00335481	0.29753948	0.193	0.183	1
Dcakd	0.00338333	0.76558078	0.015	0.01	1
Polr3g	0.00339575	0.50903999	0.016	0.011	1
4932422M17F	0.00339613	0.45025556	0.025	0.018	1
Rbm15b	0.00340198	0.38138677	0.052	0.042	1
Tmem126b	0.00341008	0.51950565	0.032	0.024	1
Lcmt1	0.00341503	0.69115053	0.049	0.04	1
Phb2	0.00342056	0.3117755	0.198	0.188	1
Pithd1	0.00342801	0.4411363	0.049	0.04	1
Dhx16	0.00343779	0.39492594	0.086	0.075	1
Toe1	0.0034803	0.58760457	0.043	0.034	1
Tbcc	0.00348222	0.52695242	0.048	0.039	1
Selenoo	0.00350304	0.42878842	0.032	0.025	1
Hook1	0.00359119	0.27060365	0.036	0.029	1
Tsen54	0.00361436	0.68507459	0.018	0.012	1
Zfp944	0.00361502	0.55871813	0.022	0.016	1
Tor4a	0.00361843	0.59934294	0.013	0.009	1
Ttbk2	0.0036741	0.47332421	0.026	0.019	1
Snx15	0.00372452	0.54218806	0.031	0.024	1
Jaml	0.00374179	0.42487118	0.096	0.085	1
Eid2	0.00377262	0.6379935	0.01	0.007	1
Cyb561d2	0.00377464	0.63577007	0.045	0.037	1
Stard3nl	0.00383818	0.40855191	0.108	0.097	1
Gm14305	0.00389356	0.70572861	0.017	0.012	1
Chd1l	0.00391052	0.41819568	0.034	0.027	1
Yipf6	0.00391573	0.37097185	0.074	0.064	1
Gm47662	0.00391933	0.65587717	0.024	0.018	1
Tufm	0.0039516	0.30500344	0.064	0.054	1
Amd1	0.00396152	0.40152736	0.076	0.066	1
4833403J16Ril	0.00402413	0.40392746	0.019	0.014	1
Mlst8	0.00403295	0.56766402	0.021	0.016	1
Ikzf5	0.00404827	0.281202	0.108	0.095	1
Rab26	0.00409495	0.50110863	0.02	0.015	1
Dr1	0.00411661	0.27369156	0.093	0.081	1
Zfp961	0.00417771	0.65605845	0.016	0.011	1
Zfp40	0.00419829	0.64779154	0.01	0.006	1
Icmt	0.00419934	0.38048292	0.04	0.032	1
Tmem173	0.00421964	0.41184446	0.091	0.08	1
Sephs2	0.00423078	0.29502573	0.049	0.04	1
Cdk7	0.00425313	0.4354331	0.051	0.042	1
Gatd1	0.00427959	0.36317848	0.062	0.052	1
Akt2	0.00439132	0.33314313	0.142	0.131	1
Plrg1	0.00439735	0.42836042	0.045	0.036	1
Anks3	0.00440532	0.38717259	0.046	0.037	1
Xrcc5	0.00441586	0.37664651	0.021	0.016	1
D5Ertd579e	0.00442569	0.32025055	0.091	0.079	1

Eif3g	0.00445371	0.29349801	0.217	0.208	1
Hk1	0.00446653	0.30922704	0.083	0.072	1
Ccdc125	0.0045072	0.43842629	0.012	0.008	1
Tmx3	0.00461002	0.29082207	0.154	0.143	1
Tmem106b	0.00465966	0.31751116	0.087	0.076	1
Dnajc24	0.00466616	0.40064552	0.048	0.039	1
Ndufs8	0.00467998	0.3346922	0.181	0.172	1
Bcl2l13	0.00468659	0.46402949	0.059	0.049	1
Adamts10	0.00470226	0.49193373	0.031	0.024	1
Ppcs	0.00482065	0.6797913	0.019	0.014	1
Igf2bp3	0.00482733	0.37500669	0.02	0.015	1
Pdhb	0.0048395	0.33448547	0.171	0.16	1
Slc25a16	0.0048543	0.47346291	0.036	0.029	1
Cops2	0.00488729	0.31210619	0.163	0.151	1
Tanc1	0.00493622	0.63905408	0.022	0.016	1
Actr6	0.00496334	0.54183768	0.027	0.021	1
Ttc9c	0.00496721	0.40637117	0.07	0.06	1
Cog8	0.00497781	0.31473497	0.051	0.042	1
Atg16l2	0.00501463	0.27825386	0.06	0.05	1
Pgm3	0.00504026	0.51340373	0.031	0.024	1
Kdm6a	0.00508248	0.25734473	0.184	0.172	1
Hsd17b7	0.00508439	0.44005782	0.038	0.031	1
Zcchc8	0.00521877	0.41524736	0.072	0.062	1
Zfp444	0.00523684	0.59482036	0.022	0.016	1
Med18	0.00532358	0.67956796	0.01	0.007	1
Tmtc4	0.00537563	0.85629504	0.01	0.007	1
Zfp414	0.00538128	0.41221416	0.028	0.021	1
Ly75	0.00538487	0.27904227	0.061	0.051	1
Tars2	0.0054144	0.56248812	0.035	0.028	1
Abcf3	0.00542553	0.37557918	0.061	0.051	1
Ppp2r3c	0.00549749	0.46189402	0.071	0.061	1
Ncapd2	0.00553228	0.51550118	0.031	0.024	1
Ttc1	0.00557845	0.36989389	0.126	0.115	1
Ssbp4	0.00563973	0.37148159	0.124	0.113	1
Anapc4	0.00567028	0.42723165	0.051	0.042	1
Reep4	0.00569122	0.5493873	0.052	0.043	1
Mrpl49	0.00569438	0.55183998	0.046	0.038	1
2010109A12R	0.00574029	0.58104928	0.011	0.007	1
Klhl18	0.00577758	0.29832217	0.036	0.028	1
Ints8	0.00578526	0.50215629	0.045	0.037	1
Rptor	0.00583013	0.43482281	0.082	0.072	1
Zfp607a	0.0059321	0.52042821	0.021	0.016	1
Mpst	0.00594893	0.49265119	0.059	0.05	1
Tubgcp4	0.0061439	0.27140272	0.073	0.063	1
Ngly1	0.00629023	0.27075071	0.064	0.055	1
1810030O07R	0.00631774	0.42374725	0.068	0.058	1
Arhgap10	0.00633599	0.69911498	0.047	0.039	1
Pgrmc1	0.00636227	0.4120731	0.097	0.086	1

Ttc38	0.00653515	0.53218087	0.016	0.011	1
Zbtb45	0.00659939	0.68392858	0.016	0.012	1
Tspan5	0.00660657	0.44981383	0.047	0.039	1
Zbtb41	0.00660957	0.32797299	0.04	0.033	1
Bloc1s6	0.00662754	0.40995301	0.056	0.047	1
Psmc2	0.00666327	0.333452	0.136	0.125	1
2700062C07R	0.00668326	0.50090099	0.036	0.029	1
Tyms	0.00673214	0.35013991	0.027	0.02	1
Riok2	0.00685698	0.35109089	0.053	0.044	1
Cxxc1	0.00689134	0.33926844	0.076	0.066	1
Rcbtb1	0.00690478	0.32189207	0.043	0.035	1
Coq9	0.00690693	0.63519371	0.027	0.02	1
Oxr1	0.00692163	0.38517193	0.094	0.083	1
Cenpc1	0.00704175	0.42169522	0.071	0.061	1
Car5b	0.00708055	0.86762169	0.016	0.011	1
Prkx	0.00710783	0.29830451	0.202	0.192	1
D3Ertd751e	0.00722021	0.43390608	0.024	0.018	1
Mvb12a	0.00725162	0.41924065	0.138	0.128	1
Cops8	0.00726649	0.30994146	0.144	0.133	1
Dhx29	0.00737331	0.39352928	0.04	0.033	1
Pcnx4	0.00737521	0.70220851	0.015	0.011	1
Gtf2f1	0.00741789	0.39509233	0.147	0.137	1
Dnttip1	0.00759851	0.37572445	0.085	0.075	1
Bet1l	0.00762382	0.29043477	0.123	0.112	1
Rpap2	0.00763369	0.33426409	0.032	0.025	1
Ccdc94	0.0076344	0.55128937	0.024	0.018	1
Scaf1	0.0076429	0.42791927	0.071	0.062	1
Timm29	0.00771127	0.53203234	0.031	0.024	1
Eci1	0.00776047	0.57150489	0.051	0.043	1
Fam60a	0.00777291	0.3072468	0.079	0.069	1
Slc19a2	0.0078091	0.61814837	0.013	0.009	1
Oxa1l	0.00791113	0.32675345	0.118	0.108	1
Zfp689	0.00800782	0.62378182	0.011	0.007	1
Brap	0.00801291	0.30004643	0.146	0.135	1
A430033K04R	0.00801384	0.64917791	0.01	0.006	1
Dapk3	0.00812612	0.34129755	0.094	0.084	1
Scyl3	0.00815852	0.34919098	0.072	0.062	1
Zfp64	0.00830091	0.37163405	0.032	0.025	1
Tmem222	0.00831603	0.39641452	0.075	0.066	1
Napg	0.00844299	0.37185445	0.107	0.096	1
Tnks1bp1	0.00861173	0.59451024	0.016	0.011	1
Rpl9-ps6	0.00862111	0.44139779	0.067	0.058	1
Myef2	0.00863754	0.25891769	0.096	0.085	1
Maz	0.00866327	0.25508773	0.093	0.082	1
Apeh	0.00885001	0.41880406	0.045	0.037	1
Rbm48	0.00899918	0.2976856	0.069	0.059	1
Lrrc28	0.00911727	0.36499232	0.023	0.018	1
Pygb	0.00919107	0.47332088	0.041	0.034	1

Zscan29	0.00920626	0.38799835	0.031	0.024	1
Mrps22	0.00928099	0.53226353	0.034	0.027	1
1600014C10R	0.00928709	0.3106652	0.044	0.036	1
D230025D16F	0.00929463	0.4241318	0.039	0.032	1
Ankrd52	0.009418	0.33809078	0.038	0.031	1
Ginm1	0.00952737	0.28839486	0.09	0.08	1
Mapk9	0.00953023	0.45617148	0.06	0.051	1
Arrdc1	0.00953496	0.30605957	0.096	0.085	1
Rae1	0.00960005	0.38519349	0.081	0.072	1
Ybey	0.00966708	0.56464395	0.014	0.01	1
Zdhhc8	0.00971233	0.42384752	0.029	0.023	1
Nub1	0.00971344	0.26781008	0.205	0.196	1
Tatdn1	0.00976245	0.4804367	0.042	0.035	1
A330069E16R	0.00976328	0.43425365	0.013	0.009	1
Dtl	0.00980851	0.50823183	0.03	0.024	1
Mlx	0.01002905	0.46617044	0.064	0.055	1
Gstp1	0.01022056	0.67378616	0.027	0.021	1
D17H6S53E	0.01023531	0.47166737	0.026	0.02	1
Sar1b	0.01033018	0.38663196	0.141	0.132	1
Lsm2	0.01036166	0.29747184	0.1	0.09	1
Nek8	0.01039662	0.2923016	0.021	0.016	1
Gemin2	0.01039774	0.62773213	0.027	0.021	1
Pef1	0.01044162	0.34745177	0.048	0.04	1
Ndufa9	0.01046957	0.4804456	0.117	0.109	1
Ranbp10	0.01048135	0.41536645	0.068	0.059	1
Hist1h4c	0.01062226	0.38901427	0.06	0.051	1
Zxdb	0.01100687	0.41643029	0.047	0.039	1
Elp6	0.01103817	0.72438821	0.011	0.008	1
4632415L05Ri	0.01104319	0.50347187	0.017	0.012	1
Tbl2	0.01117663	0.7332336	0.019	0.015	1
Taok2	0.01133739	0.2711731	0.07	0.06	1
Cluap1	0.01139843	0.37221812	0.026	0.02	1
Emc4	0.01141527	0.34220273	0.101	0.091	1
Ehmt2	0.0114416	0.41673129	0.108	0.098	1
Zfyve27	0.01144452	0.30394184	0.057	0.049	1
Mfap3	0.01147527	0.41897085	0.052	0.044	1
Galt	0.01152976	0.72992076	0.032	0.026	1
Btbd10	0.01157645	0.33512423	0.054	0.046	1
Adsl	0.01160564	0.36174406	0.046	0.039	1
Taf9b	0.01161465	0.52927794	0.022	0.016	1
Ttc33	0.01169277	0.36193552	0.064	0.056	1
Sp3os	0.01177124	0.32981196	0.046	0.039	1
Acot8	0.01180101	0.64691462	0.048	0.04	1
Haus6	0.01188546	0.39263178	0.043	0.036	1
Trim39	0.01191366	0.27329006	0.061	0.052	1
Eif2s3y	0.01213182	0.2968898	0.095	0.085	1
Cdk8	0.01223863	0.28714693	0.074	0.064	1
Zbtb5	0.01232109	0.3495517	0.034	0.027	1

Tmem201	0.01242896	0.51762315	0.016	0.012	1
Ctla2b	0.01246535	0.77713258	0.015	0.01	1
Cog4	0.01246652	0.32199824	0.059	0.05	1
Ddx11	0.01248513	0.39378428	0.015	0.011	1
Fam53a	0.01266658	0.25841364	0.044	0.036	1
Ptges2	0.01274167	0.42948149	0.018	0.014	1
Commd3	0.01277614	0.31456077	0.113	0.103	1
Chpf	0.01297701	0.33408513	0.027	0.022	1
Mterf3	0.01304415	0.45171041	0.06	0.052	1
Saysd1	0.01304745	0.59027819	0.039	0.032	1
Diaph3	0.01307472	0.49716238	0.026	0.02	1
Zfp282	0.01310893	0.48441995	0.022	0.017	1
Siva1	0.0134736	0.43128577	0.151	0.143	1
Zfp688	0.01350317	0.4155272	0.033	0.026	1
4930579G24R	0.01352449	1.15701259	0.01	0.006	1
Timm21	0.01357842	0.43559838	0.03	0.024	1
BC002059	0.0136452	0.37011397	0.02	0.015	1
Golm1	0.01375336	0.32122365	0.041	0.034	1
Dnah8	0.01376423	0.45355551	0.012	0.008	1
Med17	0.01379426	0.40856696	0.058	0.049	1
Acap3	0.01381223	0.62703952	0.02	0.015	1
Asb13	0.01391785	0.29941626	0.037	0.031	1
Olfr1369-ps1	0.01448033	0.47765587	0.027	0.021	1
Haus4	0.01452884	0.5570976	0.028	0.022	1
Traf3ip1	0.01453724	0.41226155	0.011	0.008	1
Lrrc47	0.01455789	0.30639385	0.089	0.08	1
Atpaf1	0.01473672	0.48935542	0.018	0.014	1
Hist4h4	0.01483641	0.56579602	0.023	0.017	1
Nit1	0.01507962	0.26381841	0.06	0.051	1
Surf6	0.01534934	0.29423177	0.062	0.053	1
Arhgap27os3	0.01537525	0.30370754	0.012	0.008	1
Ecd	0.01539002	0.26252197	0.071	0.062	1
Cog6	0.01543384	0.45962184	0.04	0.033	1
Armc10	0.01543638	0.40420892	0.063	0.054	1
Slc37a1	0.01561067	0.45358394	0.016	0.012	1
Hacd2	0.01563845	0.41410286	0.066	0.058	1
Rdm1	0.01565028	0.70543228	0.022	0.017	1
Nup35	0.01594314	0.5561347	0.02	0.015	1
Nsfl1c	0.01596048	0.29170212	0.114	0.105	1
Mrps7	0.01618147	0.32550175	0.145	0.136	1
Cep78	0.01644629	0.50860853	0.01	0.007	1
Atg13	0.01645348	0.32074842	0.064	0.056	1
Krtcap3	0.01647258	0.53113747	0.015	0.011	1
Ccnb2	0.01659143	0.46625916	0.016	0.012	1
Apool	0.01730206	0.46364657	0.04	0.033	1
Btbd6	0.01752969	0.68069753	0.012	0.008	1
4933427D14R	0.01758872	0.4591119	0.017	0.012	1
2410004B18R	0.0175932	0.36579745	0.081	0.072	1

Mospd1	0.01788277	0.56041557	0.025	0.019	1
Gon7	0.0179052	0.40937871	0.057	0.05	1
Otud3	0.0179239	0.75167103	0.019	0.015	1
Ulk3	0.01803155	0.42454093	0.016	0.011	1
Gmcl1	0.01809218	0.28053996	0.051	0.043	1
Zfp58	0.01812936	0.99860115	0.01	0.007	1
1110004E09R	0.01819786	0.48326377	0.038	0.032	1
Glg1	0.01830186	0.27610463	0.202	0.195	1
Mmgt2	0.01842178	0.48760424	0.032	0.026	1
Rbm14	0.01862443	0.37505259	0.054	0.046	1
Pcgf1	0.01874341	0.59584437	0.025	0.02	1
Smim27	0.01880509	0.34906708	0.092	0.083	1
Nudt18	0.01883406	0.27242417	0.042	0.035	1
Slc28a2	0.01884662	0.27325279	0.053	0.045	1
Pus3	0.01910272	0.39048897	0.036	0.03	1
Suv39h2	0.01916664	0.45644173	0.017	0.013	1
Rbm15	0.01966799	0.31030471	0.125	0.116	1
Mtrr	0.02008213	0.32126326	0.032	0.026	1
Lin9	0.0201412	0.43557464	0.035	0.029	1
Lancl1	0.02027949	0.35215753	0.037	0.031	1
Asb8	0.02033112	0.30547126	0.04	0.034	1
Trim23	0.02043422	0.40965902	0.032	0.026	1
Srbd1	0.02072082	0.5889915	0.03	0.025	1
Mtrf1l	0.02072863	0.54682483	0.021	0.016	1
Sertad3	0.02086199	0.46908148	0.029	0.023	1
Acbd4	0.02116985	0.46793233	0.016	0.012	1
Cnih1	0.02121372	0.28368815	0.108	0.099	1
Iqcc	0.02128378	0.43492759	0.024	0.019	1
Zfp956	0.02131002	0.42899942	0.016	0.012	1
Ipo8	0.02141129	0.26527114	0.07	0.061	1
Als2cl	0.02158944	0.52131706	0.014	0.01	1
Cherp	0.0216759	0.34390987	0.078	0.07	1
Sec22a	0.02187663	0.43051511	0.032	0.026	1
Zfp598	0.02188003	0.49056314	0.053	0.046	1
Elk3	0.02194101	0.36627554	0.096	0.087	1
Nom1	0.02194297	0.29494785	0.102	0.093	1
Coa3	0.02225589	0.33230391	0.132	0.124	1
Wdr92	0.0223437	0.63858161	0.017	0.013	1
Krr1	0.02236921	0.27096355	0.082	0.073	1
Pdp2	0.02237565	0.51758471	0.022	0.017	1
Txlng	0.02260107	0.33818169	0.055	0.048	1
Gramd1c	0.02296784	0.61173608	0.011	0.007	1
Sdr39u1	0.02303308	0.4000401	0.018	0.014	1
Commd4	0.02321337	0.31545873	0.148	0.141	1
Tmem175	0.02328376	0.54709088	0.016	0.012	1
Wrn	0.02330123	0.27479993	0.071	0.062	1
Dcaf13	0.02351594	0.32415901	0.14	0.131	1
Ric8a	0.02386722	0.36214506	0.089	0.08	1

Inip	0.02387125	0.28973293	0.078	0.069	1
Carhsp1	0.0239554	0.26242558	0.085	0.076	1
Ankrd16	0.02397452	0.44818813	0.035	0.029	1
Rwdd2b	0.02405688	0.52816517	0.014	0.011	1
Pigp	0.02423907	0.33009314	0.079	0.071	1
Klhl21	0.02426953	0.52454058	0.037	0.031	1
Spr	0.02429109	0.60935707	0.051	0.044	1
Taf5	0.02434972	0.4304172	0.044	0.037	1
Rab6b	0.02437121	0.32193517	0.018	0.013	1
Dyrk1b	0.02451983	0.39081634	0.03	0.025	1
Ptrhd1	0.0246371	0.4577903	0.104	0.097	1
Atxn3	0.02476069	0.34838268	0.051	0.044	1
Cops4	0.02497155	0.26876909	0.156	0.147	1
Gtf2f2	0.02513512	0.37406586	0.109	0.101	1
Isca2	0.02579784	0.36694574	0.084	0.076	1
Atg4b	0.02617184	0.31901501	0.062	0.054	1
C1qtnf12	0.02632413	0.33359275	0.022	0.017	1
Klhdc3	0.02641496	0.28818177	0.076	0.068	1
Stim2	0.02667754	0.35017958	0.1	0.092	1
Zfp692	0.02700201	0.45210066	0.018	0.014	1
Dusp12	0.02711095	0.47410212	0.034	0.028	1
Irf2bp1	0.02717248	0.29657243	0.039	0.033	1
Tmem39b	0.02749323	0.75484027	0.02	0.016	1
Gab3	0.02773044	0.40884823	0.021	0.016	1
Pex19	0.02782831	0.37362309	0.052	0.045	1
1810044D09R	0.0281821	0.35088014	0.021	0.017	1
Coa4	0.02819472	0.52592764	0.028	0.023	1
Atxn10	0.02834648	0.25369216	0.109	0.1	1
Alg13	0.02843648	0.4693917	0.034	0.028	1
Spata2	0.02914183	0.32872732	0.082	0.074	1
Ric8b	0.02935242	0.40013679	0.024	0.019	1
Ptpn4	0.02967777	0.4387569	0.043	0.036	1
Rpap1	0.03066702	0.36443905	0.025	0.02	1
Klhl36	0.03079429	0.58492467	0.015	0.011	1
Pramef8	0.03127563	0.41232788	0.041	0.034	1
Tmem218	0.03155977	0.61808929	0.017	0.013	1
Imp4	0.03219131	0.33554689	0.101	0.093	1
Mplkip	0.03227901	0.26744249	0.12	0.112	1
Thyn1	0.03292522	0.65010622	0.041	0.035	1
Eef1akmt2	0.03317965	0.38746711	0.036	0.03	1
Rpl39l	0.03336336	0.65724224	0.01	0.007	1
Eif2ak1	0.03348267	0.25984662	0.092	0.084	1
Ints11	0.03351623	0.38694093	0.04	0.034	1
Gpatch2	0.03361965	0.34622816	0.055	0.048	1
Lig4	0.03367705	0.55487255	0.01	0.007	1
Dmap1	0.03381565	0.48817243	0.027	0.022	1
Gm9493	0.03387075	0.33484477	0.086	0.078	1
Rbbp8	0.03397542	0.33090877	0.15	0.143	1

Wbp1	0.03405024	0.33138977	0.058	0.051	1
Letmd1	0.03429331	0.51369662	0.033	0.028	1
Mtcp1	0.03442101	0.34302088	0.017	0.013	1
Cebpzos	0.03541305	0.30839121	0.063	0.056	1
Rrnad1	0.03580377	0.31321249	0.042	0.036	1
Zfp777	0.03645459	0.36840128	0.03	0.025	1
Nfkbil1	0.03707297	0.27418549	0.041	0.035	1
Rbm41	0.03716852	0.38534084	0.027	0.022	1
Ints10	0.03723404	0.43428923	0.023	0.018	1
Mtmr1	0.03744805	0.30480799	0.083	0.075	1
Srfbp1	0.03786855	0.50801158	0.04	0.034	1
Nol11	0.03822289	0.28358191	0.107	0.099	1
Atm	0.03868003	0.26171279	0.048	0.041	1
Usp20	0.03910573	0.5406768	0.016	0.012	1
E130309D02R	0.03944635	0.43054516	0.057	0.05	1
Tor2a	0.03948323	0.41877011	0.055	0.048	1
Zmpste24	0.0395729	0.38228302	0.095	0.087	1
Nudt6	0.03963926	0.79930924	0.012	0.009	1
Gm11423	0.03968136	0.42030096	0.012	0.009	1
Alad	0.0397991	0.47434344	0.029	0.024	1
Mycbp	0.04012836	0.34593626	0.05	0.044	1
Smg8	0.04039806	0.62694414	0.034	0.029	1
Tbc1d7	0.04048959	0.64164218	0.011	0.008	1
Prkdc	0.04059118	0.32679847	0.041	0.035	1
Blm	0.04087832	0.32684682	0.044	0.038	1
Mpdu1	0.04093329	0.40170944	0.108	0.101	1
Zfp687	0.04106775	0.29850447	0.035	0.029	1
Minpp1	0.04140429	0.3511581	0.03	0.025	1
S1pr2	0.04218903	0.40011461	0.02	0.016	1
N4bp3	0.04227793	0.42075589	0.012	0.009	1
Usp21	0.04236111	0.34963392	0.024	0.02	1
Lsm7	0.04259863	0.27928005	0.12	0.113	1
Tubgcp5	0.04303642	0.37687795	0.032	0.027	1
Crcp	0.04341996	0.37785793	0.049	0.043	1
Trrap	0.04343672	0.2650174	0.082	0.075	1
Rnf170	0.04344634	0.37806118	0.032	0.026	1
Gpkow	0.04369905	0.27381934	0.058	0.051	1
Dedd	0.04381921	0.29681681	0.052	0.045	1
Prkab1	0.04395958	0.32156	0.042	0.036	1
Fam35a	0.04410594	0.25729078	0.02	0.016	1
Habp4	0.04430913	0.44517568	0.017	0.013	1
Prickle3	0.04438006	0.32405675	0.022	0.018	1
Acp1	0.04446915	0.28011926	0.144	0.137	1
Gpn3	0.04455264	0.2752492	0.083	0.076	1
Zswim1	0.04462426	0.37955089	0.017	0.013	1
Tmem158	0.04476212	0.2845117	0.014	0.011	1
Atrn	0.04538136	0.34033565	0.039	0.033	1
Hist1h3i	0.04553589	0.37825228	0.023	0.018	1

Klhl11	0.04565928	0.43487016	0.02	0.016	1
Tmem110	0.0456876	0.40755937	0.029	0.024	1
E530011L22Ri	0.04576468	0.27977747	0.035	0.029	1
Yars2	0.045917	0.46834061	0.019	0.015	1
Kti12	0.04596789	0.29259561	0.037	0.032	1
Zbtb11os1	0.04607767	0.46861481	0.019	0.015	1
Fam177a	0.04615513	0.40150025	0.063	0.056	1
Bop1	0.04664759	0.35271889	0.059	0.052	1
8030462N17R	0.04691322	0.42681152	0.028	0.023	1
Nup43	0.04719453	0.50022073	0.015	0.011	1
Iba57	0.04773148	0.60069579	0.022	0.017	1
Mettl6	0.04774033	0.32788348	0.061	0.054	1
Rrm1	0.04781626	0.26801443	0.065	0.058	1
Tef	0.04825429	0.49855636	0.03	0.025	1
Fam149b	0.04835775	0.43406154	0.024	0.02	1
Gm26724	0.04846994	0.62777523	0.016	0.013	1
Stard10	0.0484947	0.34698476	0.027	0.022	1
Tor1b	0.0488258	0.41227163	0.089	0.082	1
Usp50	0.04896759	0.43210162	0.044	0.038	1
Cox19	0.04933668	0.33706859	0.053	0.047	1
Ahcy	0.04941821	0.65169498	0.012	0.009	1
Spata24	0.04943621	0.39768517	0.02	0.016	1
Kctd2	0.05017718	0.32904535	0.045	0.039	1
Mettl18	0.05042441	0.5147549	0.01	0.008	1
Smu1	0.05047752	0.27365536	0.135	0.128	1
Med8	0.05067632	0.27608602	0.093	0.086	1
Eml3	0.05078506	0.53514101	0.041	0.035	1
Chd3os	0.05092848	0.96568734	0.01	0.008	1
Gm42047	0.05141995	0.39877315	0.012	0.009	1
Ceacam16	0.05236561	0.34883581	0.011	0.008	1
Erh	0.052516	0.30863281	0.109	0.102	1
Memo1	0.05295379	0.35209203	0.05	0.044	1
A430005L14Ri	0.05350685	0.47790248	0.059	0.053	1
Fastk	0.05378321	0.37659644	0.039	0.034	1
Emc8	0.05403958	0.43124454	0.056	0.05	1
Mad2l1	0.05409348	0.64050133	0.026	0.022	1
Zfp637	0.05418412	0.38751976	0.017	0.013	1
Iffo1	0.0545984	0.261502	0.028	0.023	1
Mink1	0.05463502	0.40545093	0.075	0.069	1
Sart3	0.05463907	0.31581497	0.079	0.072	1
Limk1	0.05513707	0.74762585	0.018	0.015	1
Actr1a	0.05537231	0.34046006	0.101	0.094	1
Dph7	0.05644509	0.3354143	0.016	0.012	1
Top3a	0.05654012	0.3366374	0.02	0.016	1
Zpr1	0.05655357	0.3576201	0.08	0.073	1
Gcc1	0.0568199	0.28483006	0.047	0.041	1
Rtl8b	0.05685381	0.63047626	0.025	0.021	1
Faap24	0.05698767	0.44181384	0.022	0.018	1

Usp46	0.05797786	0.49871329	0.012	0.009	1
Vps33b	0.05802713	0.42604653	0.021	0.017	1
Dtymk	0.05811297	0.30724699	0.064	0.058	1
Pdxp	0.05850675	0.27696028	0.01	0.008	1
Eif1b	0.05854967	0.25039862	0.145	0.138	1
Nfx1	0.05905043	0.28253899	0.125	0.117	1
Cdk5rap2	0.05936428	0.32219297	0.053	0.046	1
Zbtb42	0.05942304	0.44617974	0.023	0.019	1
Fmc1	0.05944929	0.34828713	0.092	0.085	1
Pop4	0.05971922	0.40659801	0.07	0.063	1
Ptcd1	0.05999377	0.55720115	0.02	0.016	1
Plekhb2	0.06059433	0.29856137	0.14	0.134	1
Zhx3	0.0613272	0.64034559	0.014	0.011	1
Zbtb48	0.06202087	0.4329377	0.014	0.011	1
Usf1	0.06205453	0.26030897	0.065	0.058	1
Tyw5	0.06248223	0.52183059	0.022	0.018	1
Gm10093	0.0627939	0.28999765	0.029	0.025	1
Mppe1	0.0630596	0.39107114	0.016	0.013	1
Slc10a3	0.06313559	0.41007278	0.028	0.024	1
Tgfbr3	0.06334425	0.48559507	0.021	0.017	1
9130401M01F	0.06343866	0.3538965	0.08	0.074	1
Csrnp2	0.06361207	0.50315656	0.01	0.008	1
Abl1	0.06425269	0.29560452	0.054	0.048	1
Rgs14	0.06490505	0.36072064	0.032	0.027	1
Svil	0.06681454	0.28562212	0.106	0.1	1
Thap1	0.06731584	0.45646569	0.033	0.028	1
Zfp346	0.06754022	0.32910334	0.015	0.012	1
Ppip5k1	0.06800455	0.35144894	0.017	0.014	1
Prmt2	0.06890542	0.38349469	0.013	0.01	1
Rnaseh2b	0.06913361	0.48289759	0.042	0.036	1
E2f6	0.06913911	0.4952271	0.014	0.011	1
Zfp810	0.06940805	0.46103574	0.014	0.011	1
Inpp5e	0.06965341	0.39703716	0.018	0.015	1
Mon1a	0.06980299	0.41988888	0.018	0.015	1
B3gnt2	0.06999803	0.32456164	0.024	0.02	1
Naa38	0.07003205	0.3005053	0.134	0.128	1
B230354K17R	0.07012189	0.41742943	0.017	0.014	1
Tarsl2	0.07017884	0.32486654	0.012	0.009	1
Ccnh	0.07020696	0.26517843	0.052	0.046	1
Slc35c1	0.07024228	0.40006446	0.018	0.015	1
Lamtor4	0.07028207	0.29022548	0.175	0.171	1
Ino80c	0.07032408	0.35769433	0.063	0.057	1
Dimt1	0.07039935	0.42199374	0.027	0.023	1
Sugp2	0.0706262	0.33804089	0.045	0.04	1
Ncdn	0.07092294	0.31836325	0.031	0.026	1
Tti2	0.07128837	0.43211873	0.026	0.022	1
Klc2	0.07139645	0.47228629	0.011	0.008	1
Abcf2	0.07159651	0.29660225	0.059	0.053	1

Hdgfl2	0.07291244	0.3378307	0.063	0.056	1
Gtpbp8	0.07315768	0.363007	0.022	0.018	1
Eif2b3	0.07323181	0.38729133	0.024	0.02	1
Zfp747	0.07390928	0.46010422	0.017	0.013	1
Ctnnbip1	0.07462596	0.70989315	0.051	0.046	1
Fahd2a	0.07476332	0.66918095	0.022	0.018	1
Mettl22	0.07565026	0.52784751	0.013	0.01	1
Zbtb32	0.07690124	0.71293349	0.013	0.01	1
Zfp954	0.07758795	0.49461099	0.044	0.039	1
Sntb2	0.07783975	0.41962344	0.054	0.048	1
Nudc	0.07818587	0.25612794	0.179	0.174	1
Tex264	0.07878703	0.4326616	0.06	0.054	1
Kif11	0.07886513	0.25271398	0.022	0.018	1
Gm527	0.07947248	0.53425309	0.015	0.012	1
Ppih	0.08067064	0.6280136	0.036	0.032	1
Tab1	0.0807463	0.33929659	0.01	0.007	1
B3galt6	0.08081342	0.37675803	0.015	0.012	1
Psmd6	0.0814316	0.31193648	0.142	0.137	1
Cmtm4	0.0815541	0.46728032	0.019	0.016	1
Blzf1	0.0816512	0.32708018	0.046	0.041	1
Prps1l3	0.08192299	0.30635144	0.025	0.021	1
Vrk3	0.08227763	0.38350716	0.078	0.072	1
Pdrg1	0.08276416	0.33163178	0.072	0.066	1
Cbx6	0.08342805	0.32696768	0.022	0.018	1
Ap4s1	0.08383422	0.28405807	0.069	0.063	1
Fbxo25	0.08528784	0.34105406	0.018	0.014	1
Shkbp1	0.08531992	0.37698231	0.062	0.056	1
Mapk1ip1	0.08554105	0.30370109	0.025	0.021	1
Ttc7b	0.0858468	0.33010594	0.034	0.029	1
Kat8	0.08584759	0.4503328	0.021	0.018	1
Tyk2	0.08614572	0.38886234	0.059	0.054	1
AI837181	0.08668565	0.33203318	0.078	0.072	1
Med24	0.08779875	0.40502973	0.036	0.032	1
Zfp39	0.08833897	0.34548885	0.012	0.01	1
Timm44	0.08834445	0.27960018	0.104	0.098	1
Lmf2	0.08956494	0.40076584	0.045	0.04	1
Gm14326	0.09031162	0.56533918	0.011	0.008	1
Tax1bp3	0.0903921	0.65295776	0.041	0.036	1
Lsm14b	0.09078209	0.31265884	0.037	0.032	1
Ptdss1	0.09114114	0.38663051	0.059	0.054	1
Llgl1	0.09117874	0.41996977	0.026	0.022	1
Ssbp2	0.0913832	0.25182306	0.055	0.049	1
Ttc19	0.09150151	0.35393789	0.104	0.099	1
Cep70	0.09183293	0.74663588	0.016	0.013	1
Top3b	0.0930092	0.29259221	0.049	0.043	1
Wdr83	0.09321701	0.41544626	0.027	0.023	1
Fam120aos	0.09331005	0.46140172	0.011	0.008	1
Eif2b4	0.09369497	0.26178443	0.048	0.043	1

Golga2	0.0947662	0.25766173	0.069	0.063	1
Traf3ip2	0.09517585	0.25049124	0.039	0.034	1
Bicd2	0.09522982	0.35634743	0.063	0.057	1
Exoc2	0.09532476	0.25018452	0.083	0.077	1
Pkmyt1	0.09554608	0.42432436	0.016	0.013	1
Gm16124	0.09568364	0.57102138	0.012	0.01	1
Cit	0.09628799	0.77385837	0.012	0.01	1
Zfp81	0.09662045	0.28686308	0.022	0.018	1
Mbd3	0.09786501	0.3862268	0.087	0.082	1
Pvt1	0.09839794	0.3497606	0.036	0.032	1
Ccdc117	0.09845514	0.28972338	0.065	0.059	1
Golga1	0.09902925	0.46307831	0.019	0.016	1
Msto1	0.09923892	0.44703753	0.019	0.016	1
Tmem41b	0.0995048	0.26011995	0.104	0.098	1
Cramp1l	0.09984723	0.30293353	0.06	0.054	1
Fastkd3	0.10028762	0.62295665	0.015	0.012	1
Pex13	0.10058466	0.30973191	0.057	0.052	1
Gcfc2	0.10080522	0.4361587	0.015	0.012	1
Gm45902	0.10117654	0.32165101	0.026	0.022	1
Kif18a	0.10220587	0.39443794	0.017	0.014	1
Styx	0.10568158	0.30154842	0.033	0.029	1
Inpp4a	0.10636011	0.37947998	0.065	0.06	1
Bap1	0.10701443	0.31208005	0.06	0.055	1
Setd1a	0.10709891	0.26257111	0.061	0.055	1
Zfp579	0.10734974	0.44447788	0.013	0.01	1
Dap	0.10823185	0.26438251	0.15	0.146	1
Wdr5	0.10827944	0.33589556	0.056	0.051	1
Cwc22	0.10832484	0.29247944	0.108	0.103	1
Kpna6	0.10837982	0.30819729	0.055	0.05	1
Kif20b	0.10870896	0.3495591	0.025	0.021	1
Commd6	0.10946845	0.30571835	0.129	0.125	1
Ptpn9	0.11013392	0.27004232	0.069	0.064	1
Mios	0.11084896	0.32159527	0.024	0.02	1
Trappc6a	0.11094312	0.32002355	0.112	0.107	1
Agap3	0.11134897	0.49818215	0.044	0.04	1
Znhit3	0.1119429	0.3196262	0.037	0.033	1
Ten1	0.11218473	0.34685792	0.095	0.09	1
Champ1	0.11262273	0.40572631	0.04	0.036	1
Tbcd	0.11400753	0.55094521	0.036	0.031	1
U2af1l4	0.11439978	0.9595334	0.01	0.008	1
Slc35b1	0.11627913	0.32245364	0.132	0.128	1
Rnaseh1	0.11682693	0.33530108	0.029	0.026	1
Rbm4	0.11704374	0.27309168	0.022	0.018	1
Mtfr1l	0.11796866	0.33220982	0.072	0.066	1
Zfx	0.11938325	0.31565673	0.121	0.116	1
Nrde2	0.11952569	0.34888708	0.055	0.05	1
Eci2	0.12100844	0.47652408	0.072	0.068	1
Ttf2	0.12210683	0.33027224	0.025	0.021	1

Rnf103	0.12278607	0.31833291	0.062	0.057	1
Aptx	0.12315517	0.28461838	0.036	0.032	1
Haghl	0.12379637	0.60618607	0.024	0.021	1
Terf2	0.12441708	0.31496771	0.067	0.062	1
Mfsd8	0.12479349	0.32400049	0.024	0.021	1
Psmd3	0.12589601	0.27002026	0.105	0.099	1
Tmem11	0.12595141	0.30746576	0.104	0.099	1
Abcd4	0.12679985	0.27835594	0.024	0.021	1
Nsmf	0.12762549	0.38677631	0.026	0.022	1
Kbtbd3	0.12824115	0.45843427	0.014	0.011	1
Thg1l	0.1282609	0.3011127	0.023	0.02	1
Ap3b1	0.12981164	0.27128573	0.153	0.15	1
Rcn3	0.12994831	0.37090301	0.023	0.02	1
Etfbkmt	0.13060837	0.50960704	0.013	0.01	1
Aldh18a1	0.13222413	0.41652473	0.03	0.026	1
Bcl7b	0.13524383	0.2517947	0.149	0.145	1
Rundc1	0.13656566	0.31436439	0.027	0.024	1
Ints7	0.13709799	0.25478618	0.063	0.058	1
Mrs2	0.13819488	0.32552947	0.045	0.041	1
Adck2	0.13880787	0.35175917	0.02	0.017	1
Rfwd3	0.14078758	0.41768725	0.041	0.037	1
Trak2	0.14092029	0.27241269	0.08	0.075	1
Trmt11	0.14095752	0.3892127	0.032	0.028	1
Spryd3	0.14097114	0.37978807	0.032	0.029	1
Nol9	0.14153251	0.25650975	0.043	0.038	1
Rab22a	0.14186167	0.2578644	0.137	0.133	1
Hace1	0.14198105	0.36555966	0.039	0.035	1
Focad	0.14341958	0.31701711	0.013	0.01	1
Mis12	0.14388574	0.34044175	0.064	0.059	1
Setd6	0.14459685	0.57860994	0.018	0.015	1
Acot13	0.14540252	0.35105057	0.052	0.048	1
Bloc1s3	0.14547858	0.35559869	0.021	0.018	1
Spast	0.14569075	0.34111573	0.071	0.066	1
Ubqln2	0.1459405	0.36005685	0.066	0.061	1
AU022252	0.14640011	0.35562754	0.022	0.019	1
Gm45509	0.14714069	0.31330095	0.01	0.008	1
Zfp113	0.15102764	0.25105115	0.016	0.014	1
Gm26526	0.15105562	0.35891829	0.022	0.019	1
Tbc1d25	0.15114566	0.51523483	0.011	0.009	1
Cbr1	0.15139844	0.39699455	0.033	0.029	1
Slc35d2	0.15252537	0.42874142	0.014	0.012	1
Zfp317	0.15302329	0.33326339	0.024	0.021	1
Mtmr9	0.15347089	0.40113462	0.024	0.021	1
B3glct	0.15438241	0.64028055	0.013	0.011	1
Rgs19	0.15537018	0.27512613	0.072	0.067	1
Ofd1	0.15617595	0.31581961	0.035	0.031	1
Asap2	0.15729552	0.83224212	0.024	0.021	1
Wdr74	0.15790224	0.42703989	0.026	0.022	1

Arv1	0.15933626	0.45807879	0.01	0.008	1
Bola1	0.15991553	0.37262661	0.046	0.042	1
Golga3	0.16000647	0.28395041	0.071	0.066	1
Yipf1	0.16009586	0.34808524	0.09	0.086	1
Tmem192	0.16076925	0.29590367	0.089	0.084	1
Ulbp1	0.16088464	0.34796714	0.012	0.009	1
Rab40c	0.16105433	0.28432073	0.024	0.021	1
Brf2	0.16194659	0.30965324	0.02	0.017	1
Togaram1	0.16196061	0.31030979	0.076	0.071	1
Guf1	0.16253119	0.27239213	0.032	0.029	1
Rab1b	0.16286533	0.2723913	0.111	0.106	1
Psmg3	0.16348456	0.25985286	0.056	0.051	1
Ythdc2	0.16381167	0.26291955	0.071	0.066	1
3010003L21Ri	0.16381237	0.63360704	0.017	0.014	1
Camsap1	0.16426121	0.30536454	0.031	0.028	1
Slc7a6	0.16433841	0.2918775	0.023	0.02	1
Unc119	0.16489839	0.29506607	0.082	0.078	1
Tuft1	0.16685423	0.40551411	0.011	0.009	1
Tmem62	0.16801664	0.35968626	0.016	0.014	1
Ap3m2	0.16829834	0.25590065	0.027	0.023	1
Gm16973	0.1685782	0.25020838	0.016	0.013	1
Phospho2	0.17105305	0.26214387	0.052	0.048	1
Pitpnb	0.17173049	0.28429735	0.102	0.097	1
Snapc2	0.17192599	0.30432485	0.026	0.022	1
Ndufaf2	0.17324804	0.44185585	0.051	0.047	1
Ints5.1	0.17338101	0.46717047	0.022	0.019	1
Sap130	0.17439543	0.39138852	0.021	0.018	1
Nmnat1	0.17459504	0.64141302	0.011	0.009	1
Nipsnap3b	0.17606322	0.3680776	0.099	0.095	1
Rwdd4a	0.17654533	0.32801456	0.07	0.066	1
BC052040	0.17797531	0.38357142	0.01	0.008	1
Zfp933	0.17893389	0.33493684	0.013	0.011	1
Pold2	0.17952288	0.30034959	0.027	0.024	1
Cd3eap	0.18022475	0.35602728	0.026	0.023	1
Zc3hc1	0.18031344	0.33626268	0.05	0.046	1
Sec61a2	0.18108477	0.30818915	0.031	0.027	1
Trmt44	0.18145108	0.38206469	0.021	0.018	1
Bud13	0.18210681	0.31620942	0.043	0.039	1
Mark4	0.18481707	0.48231882	0.036	0.032	1
Rnf126	0.18523713	0.27574934	0.044	0.04	1
Spindoc	0.18570195	0.29007149	0.016	0.014	1
St14	0.1861984	0.40499144	0.013	0.01	1
Zfp593	0.18632127	0.28566094	0.112	0.108	1
Vps16	0.18728614	0.2575998	0.063	0.059	1
Naxe	0.19228403	0.36646588	0.081	0.078	1
Aspscr1	0.19419811	0.30507973	0.054	0.05	1
Gm27010	0.19481946	0.45664925	0.012	0.01	1
Tubgcp2	0.19654903	0.25816796	0.045	0.042	1

Rad52	0.19688559	0.31184355	0.02	0.017	1
Rfesd	0.19763239	0.30108699	0.021	0.018	1
Rcan3	0.19803337	0.27179754	0.02	0.017	1
Flywch1	0.19862105	0.39301372	0.019	0.016	1
Rnf214	0.19934242	0.28376157	0.04	0.036	1
Fkrp	0.19981641	0.44485635	0.034	0.031	1
Washc3	0.20006808	0.25716484	0.09	0.086	1
Rpe	0.20078863	0.26282441	0.077	0.073	1
Fam13b	0.20183029	0.29149661	0.071	0.067	1
Fam210b	0.20191802	0.25333109	0.031	0.028	1
Dhcr24	0.20425925	0.31604513	0.023	0.027	1
Kif3c	0.2057801	0.46875329	0.01	0.008	1
Dhrs4	0.20598206	0.31300117	0.042	0.038	1
2410131K14R	0.20718299	0.3710535	0.027	0.024	1
Serhl	0.20824838	0.31075354	0.034	0.031	1
Myo10	0.2104164	0.56766091	0.016	0.013	1
Rab24	0.21127791	0.29100315	0.108	0.105	1
Aven	0.2119891	0.26655377	0.049	0.046	1
Zdhhc4	0.21266993	0.35206829	0.037	0.034	1
Nubp2	0.21323728	0.35156536	0.069	0.065	1
Lmo1	0.21433958	1.45348366	0.013	0.011	1
H2-M3	0.21516139	0.30654772	0.136	0.134	1
Ercc2	0.21569459	0.30169496	0.024	0.021	1
2700016F22Ri	0.21579882	0.40442258	0.014	0.011	1
Cog3	0.21891351	0.29126587	0.045	0.041	1
Endod1	0.21971444	0.31715077	0.055	0.052	1
Spg20	0.22235433	0.25711993	0.028	0.025	1
Fbxo7	0.22352707	0.29399573	0.046	0.042	1
Pycrl	0.22497512	0.57381681	0.014	0.012	1
Sos1	0.22536886	0.2927628	0.058	0.054	1
Npepl1	0.22634784	0.32345591	0.07	0.066	1
Sdhaf2	0.22646582	0.27126577	0.086	0.082	1
Mrps15	0.22652709	0.35649778	0.128	0.126	1
Coq3	0.22752014	0.31883046	0.014	0.012	1
B4galt7	0.22886756	0.40348777	0.021	0.018	1
Lsm11	0.22937001	0.35408384	0.025	0.022	1
Zxdc	0.23137318	0.35978024	0.036	0.033	1
Copg1	0.23392232	0.27928748	0.091	0.088	1
Sap18b	0.23427527	0.6704805	0.015	0.013	1
Als2	0.23490567	0.40274416	0.025	0.022	1
Vps53	0.23576809	0.32580765	0.072	0.068	1
1500011B03R	0.23745986	0.34120335	0.023	0.021	1
Pola2	0.23775773	0.36113185	0.022	0.019	1
Rmi1	0.24027759	0.27247484	0.054	0.051	1
Sucla2	0.24041709	0.25337559	0.082	0.078	1
Stau1	0.24174446	0.27746209	0.088	0.084	1
5430403G16R	0.24751267	0.56618761	0.01	0.008	1
Cdk5rap1	0.24798891	0.43970409	0.013	0.011	1

Smpdl3b	0.24817517	0.27214061	0.016	0.014	1
Fam206a	0.24998354	0.26784617	0.022	0.02	1
Agpat2	0.2505411	0.4118222	0.017	0.014	1
Ccdc32	0.25221705	0.37598715	0.041	0.037	1
Kdm4b	0.25284638	0.30168132	0.028	0.025	1
1700025G04R	0.25447904	0.58865675	0.047	0.052	1
Eri1	0.25496086	0.27976631	0.146	0.145	1
Ndufs1	0.25580194	0.25554676	0.093	0.089	1
Trim28	0.25633064	0.30910509	0.125	0.122	1
Hist1h2ak	0.25649734	0.44373484	0.014	0.012	1
Zkscan17	0.25809988	0.41271056	0.033	0.03	1
Acaa2	0.26450241	0.32542196	0.05	0.047	1
Ppp2r5b	0.26599818	0.59672249	0.011	0.009	1
Noc4l	0.26828741	0.4090643	0.041	0.038	1
Psmf1	0.27058833	0.26958387	0.061	0.057	1
Aurka	0.27073589	0.51757511	0.01	0.008	1
Apol7b	0.27353179	0.3476086	0.01	0.009	1
Xab2	0.27364471	0.25796927	0.06	0.057	1
Fancl	0.27478748	0.38524308	0.032	0.029	1
Golt1b	0.27765719	0.26715084	0.102	0.099	1
Zfp951	0.27917551	0.45912737	0.01	0.009	1
Ccdc71	0.28100883	0.26295984	0.038	0.035	1
Spag7	0.28181416	0.27357666	0.114	0.112	1
Snupn	0.28223195	0.38092168	0.02	0.018	1
Cdc123	0.28332878	0.2940864	0.082	0.079	1
Pigo	0.283885	0.39151642	0.021	0.019	1
Med25	0.28631512	0.39977504	0.061	0.058	1
Kmt2b	0.29250783	0.29111833	0.101	0.099	1
Tango2	0.29254575	0.27707571	0.026	0.023	1
Sssca1	0.29554396	0.31373526	0.047	0.044	1
Tmem80	0.29640562	0.27052822	0.033	0.03	1
Gnb4	0.29708447	0.28063832	0.046	0.043	1
Alg6	0.297983	0.504676	0.011	0.01	1
Haus7	0.301603	0.3697989	0.024	0.022	1
Ankrd26	0.30183048	0.3212015	0.014	0.012	1
Gm13212	0.30258173	0.41350421	0.039	0.036	1
Tepsin	0.3031247	0.25259013	0.01	0.009	1
Srgap3	0.30490827	0.57939958	0.036	0.033	1
Slc35b4	0.30606376	0.40286413	0.023	0.02	1
Snx16	0.30762914	0.29002665	0.022	0.019	1
Cep152	0.30844627	0.2625285	0.016	0.014	1
Zfp65	0.31693305	0.32878637	0.026	0.023	1
Clk2	0.31937686	0.25842726	0.052	0.049	1
Psmd5	0.32035664	0.31644308	0.037	0.034	1
Smim24	0.32068771	0.38341941	0.023	0.026	1
Ttc17	0.32109419	0.38702276	0.046	0.043	1
Fars2	0.32201764	0.35289305	0.041	0.038	1
Mrpl2	0.32280837	0.41111147	0.064	0.062	1

Smarcb1	0.32589692	0.33596038	0.082	0.08	1
Ino80	0.32727945	0.25185656	0.099	0.096	1
Mrpl27	0.32968189	0.34795611	0.084	0.081	1
1700052K11R	0.33122612	0.35466237	0.011	0.01	1
Stk16	0.33174302	0.27624236	0.064	0.061	1
Repin1	0.33415235	0.32541922	0.011	0.01	1
Wdr53	0.33483866	0.26555302	0.027	0.024	1
Pqlc3	0.33587504	0.33817265	0.04	0.038	1
Plekhf1	0.3376143	0.25539184	0.013	0.011	1
4930524J08Ril	0.34065141	0.31876392	0.009	0.011	1
Lrig2	0.34290892	0.30481743	0.033	0.031	1
Slc39a3	0.34837881	0.25033075	0.027	0.025	1
Ilk	0.34917454	0.27399482	0.098	0.096	1
Tfdp1	0.34997325	0.40899276	0.032	0.03	1
Pdzd11	0.35457413	0.27793128	0.061	0.058	1
Gm20324	0.35610529	0.49428735	0.01	0.009	1
Gtf2e1	0.35731195	0.32553446	0.026	0.023	1
Miip	0.35746447	0.28898052	0.029	0.027	1
Sac3d1	0.35802414	0.34556412	0.01	0.009	1
Cep85	0.35908346	0.28230026	0.029	0.026	1
Micu3	0.36267454	0.26893327	0.028	0.026	1
Pde4dip	0.36372064	0.25445181	0.043	0.04	1
Cenpk	0.36399677	0.43643027	0.02	0.018	1
Coa7	0.36436853	0.37326176	0.043	0.041	1
Dcp1a	0.36453039	0.26676296	0.063	0.06	1
Zfp429	0.36911747	0.36345077	0.012	0.01	1
Cenpm	0.37458109	0.26754567	0.013	0.012	1
Gmppb	0.37601658	0.27422	0.04	0.044	1
Nup88	0.37728574	0.25903666	0.09	0.087	1
Vrk2	0.37944195	0.34229878	0.066	0.064	1
Kdelr2	0.38337965	0.28427283	0.127	0.126	1
Endog	0.38364968	0.49919276	0.028	0.026	1
Tstd2	0.38550721	0.29714282	0.029	0.027	1
Bag5	0.38829503	0.29447132	0.053	0.051	1
Pdcl3	0.39101671	0.25646871	0.122	0.121	1
Ppp2r1b	0.39158765	0.29173585	0.034	0.031	1
Ube2v1	0.39215623	0.35560027	0.022	0.02	1
Rusc1	0.39237883	0.27375125	0.06	0.057	1
Zfp319	0.39509701	0.34928895	0.022	0.021	1
Hspbp1	0.39650976	0.26119618	0.056	0.054	1
Rnf25	0.39651535	0.33378395	0.029	0.027	1
Glo1	0.40461339	0.3111199	0.078	0.076	1
Slc2a8	0.40898909	0.49139373	0.016	0.014	1
Acad8	0.41069586	0.38670939	0.013	0.012	1
Noa1	0.41152174	0.29014935	0.022	0.021	1
Exosc9	0.41250808	0.29544825	0.05	0.048	1
Bak1	0.41363738	0.30397012	0.096	0.094	1
Zfp46	0.41553054	0.30531588	0.012	0.011	1

Dync2h1	0.41957149	0.38924803	0.012	0.011	1
Kremen1	0.42361382	0.25950335	0.025	0.023	1
Ctdp1	0.42464396	0.29604639	0.071	0.069	1
Mgat2	0.42577038	0.28938817	0.119	0.119	1
1600002K03R	0.42855558	0.39535105	0.054	0.052	1
Tmem167b	0.42997015	0.26135602	0.07	0.068	1
Elof1	0.43553771	0.30788624	0.106	0.105	1
Ranbp3	0.43573053	0.36695861	0.054	0.052	1
Thumpd2	0.44410109	0.29264673	0.01	0.009	1
Vps25	0.44503053	0.51740773	0.01	0.009	1
Uhrf1	0.44521048	0.30062029	0.018	0.017	1
Zgrf1	0.44982547	0.31456067	0.013	0.012	1
Alg3	0.45113529	0.47639566	0.016	0.015	1
Tsta3	0.45427258	0.43418823	0.051	0.05	1
Nelfe	0.46490587	0.35100313	0.071	0.069	1
Polr1c	0.46958226	0.25594704	0.115	0.114	1
Desi2	0.47105537	0.27966957	0.11	0.11	1
Cgrrf1	0.47115387	0.35644671	0.031	0.029	1
1810009A15R	0.47281722	0.2783225	0.01	0.009	1
Fbxo42	0.47700187	0.3635735	0.04	0.038	1
Hat1	0.4833776	0.30479503	0.113	0.113	1
Atp6v0a2	0.48528941	0.2647689	0.067	0.065	1
Chaf1a	0.49180912	0.39370228	0.011	0.01	1
Dcun1d3	0.49639364	0.38115181	0.024	0.022	1
1110051M20F	0.50299115	0.3693866	0.01	0.011	1
Primpol	0.50652046	0.32317004	0.02	0.019	1
Elovl1	0.51312247	0.35434399	0.078	0.077	1
Arhgef7	0.52214611	0.26875494	0.044	0.042	1
Isoc2b	0.52656293	0.28151315	0.015	0.014	1
Pola1	0.52953701	0.30086051	0.044	0.042	1
Fbxo31	0.52992934	0.27859409	0.023	0.021	1
Dip2a	0.53108479	0.29376235	0.011	0.01	1
Gbp8	0.53387856	0.26531452	0.058	0.056	1
Psmg4	0.53746475	0.27293176	0.16	0.163	1
Klf3	0.54114647	0.28229186	0.208	0.214	1
Tubg1	0.54415408	0.35152766	0.011	0.01	1
Tmco4	0.55925245	0.30395286	0.016	0.015	1
Smim12	0.56117037	0.39529971	0.056	0.055	1
Flad1	0.56141586	0.32785693	0.018	0.017	1
Lage3	0.56637542	0.25355049	0.06	0.059	1
Commd1	0.57028956	0.3387341	0.101	0.102	1
Psat1	0.57566739	0.25637537	0.041	0.039	1
Pygo2	0.57837193	0.25689522	0.025	0.023	1
AI413582	0.58025608	0.30824768	0.082	0.081	1
Ift52	0.58346705	0.270053	0.059	0.058	1
Ddx49	0.58410534	0.32310433	0.069	0.068	1
Them6	0.59799374	0.32564611	0.014	0.013	1
Hint2	0.60014733	0.3245419	0.118	0.119	1

Lrrc42	0.60030985	0.32332021	0.025	0.024	1
Dohh	0.60099886	0.29461552	0.073	0.072	1
1110065P20R	0.60162499	0.28929957	0.043	0.042	1
Alkbh7	0.60635558	0.25950201	0.042	0.04	1
Zbtb17	0.61067905	0.32041803	0.05	0.049	1
Pradc1	0.62162672	0.40433647	0.014	0.013	1
Rbsn	0.6264909	0.25040076	0.041	0.04	1
Traf7	0.63047972	0.25180583	0.068	0.067	1
Eif2b1	0.63285353	0.30779203	0.031	0.03	1
Gm20707	0.63583497	0.38721541	0.017	0.016	1
Tchp	0.64413347	0.32584453	0.017	0.016	1
Mrps25	0.64494564	0.33903781	0.068	0.067	1
Haus1	0.64623573	0.3853309	0.012	0.011	1
Ntmt1	0.64771113	0.40216409	0.023	0.022	1
Zfp276	0.65266838	0.48737124	0.014	0.013	1
Manbal	0.66068717	0.38485244	0.072	0.071	1
Slc25a13	0.66546765	0.61766136	0.011	0.012	1
Mthfsd	0.66696003	0.30854043	0.017	0.016	1
Rabggta	0.66756195	0.27022594	0.033	0.032	1
Rfng	0.66761628	0.4372827	0.016	0.016	1
Ugp2	0.66946877	0.2835129	0.128	0.13	1
Ick	0.67467873	0.36612671	0.01	0.009	1
Cdk5	0.67752165	0.33555136	0.039	0.038	1
2310031A07R	0.68642353	0.41978768	0.023	0.024	1
C330027C09F	0.68858039	0.46351296	0.01	0.009	1
Dlgap4	0.69505601	0.29132629	0.072	0.072	1
Hectd4	0.70237341	0.278255	0.027	0.028	1
Med6	0.70772415	0.31684235	0.041	0.041	1
Blvra	0.70842623	0.28457317	0.055	0.057	1
Zbed3	0.70968903	0.25787862	0.015	0.014	1
Ercc4	0.71767781	0.30022236	0.029	0.028	1
Ccdc102a	0.71826044	0.78611984	0.014	0.014	1
Intu	0.72297142	0.28252485	0.011	0.01	1
Tbl3	0.72666883	0.27606022	0.026	0.025	1
Dock5	0.73316424	0.48792688	0.039	0.038	1
Zfp771	0.73511511	0.45143102	0.022	0.022	1
Pigyl	0.73907078	0.42369231	0.051	0.05	1
Slc39a9	0.7423343	0.27557521	0.035	0.034	1
Ipo13	0.74301628	0.29368765	0.017	0.018	1
Nudt9	0.74890326	0.31161409	0.069	0.071	1
Mrpl28	0.75189948	0.26113237	0.074	0.074	1
Lta4h	0.75625883	0.26150485	0.079	0.079	1
Tab3	0.75758644	0.26607205	0.037	0.037	1
Atxn1l	0.75776523	0.30824818	0.042	0.043	1
Psen1	0.75790877	0.25788326	0.101	0.102	1
Bad	0.7625712	0.30952927	0.042	0.042	1
Cd151	0.76411397	0.50716537	0.01	0.01	1
Dhcr7	0.76574488	0.26332001	0.017	0.017	1

Timm50	0.76638804	0.26715821	0.053	0.053	1
Pus1	0.76796338	0.3002588	0.055	0.055	1
Fbxw9	0.77478396	0.35568694	0.009	0.01	1
1700021F05Ri	0.7789055	0.37048451	0.021	0.021	1
Abhd5	0.78134005	0.32392798	0.036	0.036	1
Tgds	0.78300356	0.34057032	0.013	0.012	1
Arl10	0.78302636	0.29021568	0.014	0.015	1
Zc3h3	0.78774007	0.26599594	0.026	0.026	1
Rnf5	0.78836962	0.32872742	0.045	0.047	1
Nup107	0.79160058	0.25242279	0.022	0.022	1
Gm6133	0.7916121	0.31440454	0.03	0.03	1
Vti1b	0.7942239	0.33534658	0.13	0.134	1
Chchd5	0.79570562	0.30436532	0.057	0.057	1
Stx8	0.79701546	0.35247518	0.11	0.114	1
Anapc11	0.79824437	0.27674744	0.128	0.131	1
Ubiad1	0.80051032	0.35652447	0.009	0.01	1
Med16	0.80367801	0.25984452	0.046	0.046	1
Rassf1	0.81517374	0.29475379	0.133	0.137	1
Bloc1s1	0.8195289	0.35957093	0.039	0.04	1
Ficd	0.81987682	0.27282598	0.012	0.012	1
Kif1bp	0.82124209	0.32650796	0.027	0.027	1
C2cd2l	0.82396863	0.25006592	0.018	0.018	1
Pak4	0.82577103	0.3412824	0.023	0.024	1
Rnaseh2a	0.82623581	0.3092885	0.036	0.036	1
Tpra1	0.82810093	0.32003486	0.028	0.028	1
Vamp7	0.83505784	0.29483674	0.058	0.058	1
Trib3	0.83915178	0.30645403	0.015	0.015	1
Casp1	0.84182493	0.3250024	0.056	0.058	1
Slc12a7	0.84636251	0.30760696	0.029	0.028	1
Gm11713	0.8530067	0.30672032	0.011	0.012	1
Tbrg4	0.85330064	0.28577908	0.045	0.044	1
Med27	0.85546849	0.37973728	0.037	0.037	1
Atp11a	0.85974111	0.2574086	0.034	0.034	1
Pih1d1	0.86940336	0.28399864	0.056	0.056	1
Ptrh1	0.87058099	0.32993423	0.012	0.012	1
Pla2g12a	0.87337558	0.35023105	0.022	0.022	1
N6amt1	0.88347436	0.25370963	0.06	0.06	1
Arfip2	0.88967801	0.26812669	0.015	0.015	1
Atat1	0.89666067	0.36985738	0.014	0.014	1
Dctn5	0.90318494	0.28530777	0.056	0.058	1
Stambp	0.90410204	0.25824329	0.045	0.045	1
Vti1a	0.90631325	0.28623507	0.059	0.061	1
Scyl1	0.91819455	0.28745464	0.04	0.041	1
Praf2	0.92876694	0.459696	0.024	0.024	1
Tssk6	0.93559019	0.37361429	0.011	0.012	1
Scap	0.94666131	0.26587748	0.037	0.037	1
Ccdc73	0.95383602	0.30193669	0.013	0.013	1
Trp53rka	0.95876237	0.2659204	0.033	0.033	1

Myo1f	0.97134696	0.28882775	0.079	0.081	1
Nqo2	0.97140103	0.43838766	0.018	0.018	1
Gpr180	0.97208491	0.41672012	0.016	0.016	1
Adck1	0.97217744	0.26209328	0.019	0.019	1
Dnajc25	0.98368952	0.3204529	0.034	0.034	1
Dlgap5	0.98854966	0.53371066	0.013	0.013	1

CD8 Tn

Gene	p_val	avg_log2FC	pct.1	pct.2	p_val_adj
Lef1	0	4.48376301	0.711	0.063	0
Tcf7	0	2.74252881	0.691	0.158	0
S1pr1	0	2.47896339	0.668	0.158	0
Ms4a4b	0	2.18225172	0.729	0.232	0
Txk	0	2.31734487	0.679	0.209	0
Cd3d	0	1.3424878	0.728	0.302	0
Ablim1	0	1.92870881	0.694	0.274	0
Sell	0	2.85152648	0.473	0.074	0
Ms4a6b	0	2.34284101	0.635	0.241	0
Klf2	0	1.89973954	0.595	0.212	0
Gimap3	0	1.57286765	0.665	0.297	0
Satb1	0	1.459557	0.874	0.528	0
Rapgef6	0	1.99481879	0.618	0.275	0
Gm26917	0	1.91319181	0.658	0.341	0
Actn1	0	3.27869861	0.359	0.063	0
Zfp281	0	2.82932931	0.356	0.085	0
Dapl1	0	8.32762239	0.261	0.002	0
Cmah	0	2.56164345	0.322	0.064	0
Rflnb	0	4.4635612	0.272	0.017	0
Cd8b1	0	2.26567532	0.313	0.068	0
Fam241a	0	3.10639749	0.303	0.067	0
Igfbp4	0	2.77700765	0.297	0.072	0
Nsg2	0	3.9067286	0.223	0.017	0
Myb	0	3.19288374	0.239	0.039	0
Gm2682	0	3.923606	0.217	0.019	0
Prkcq	0	2.9445272	0.237	0.042	0
Ifi213	0	3.16058253	0.224	0.035	0
Elovl6	0	3.50740032	0.2	0.027	0
Ifi206	0	3.15549274	0.192	0.028	0
Tdrp	0	5.101766	0.146	0.005	0
Sidt1	0	3.14050929	0.163	0.023	0
Scml4	0	3.17266524	0.155	0.021	0
Ifi208	0	3.71877292	0.116	0.009	0
Rps17	0	0.91901763	0.964	0.861	0
Als2cl	0	4.54377445	0.104	0.006	0
Rpl35	0	0.88054088	0.966	0.872	0
Rpl36a	0	0.9631196	0.977	0.89	0
5830468F06Ri	0	4.79009922	0.069	0.003	0
Rps6	0	0.75412685	0.984	0.929	0
Rpl12	0	1.04035122	0.995	0.946	0
Rpl5	0	1.03491609	0.991	0.943	0
Rps7	0	0.7436973	0.992	0.945	0
Rps4x	0	0.69180897	0.995	0.954	0
Rps15a	0	0.65064544	0.997	0.956	0
Rps19	0	0.93029385	0.994	0.958	0
Rps15	0	0.83984791	0.998	0.963	0

Gm43698	0	8.04676862	0.034	0	0
Rps20	0	0.91435058	0.997	0.964	0
Rps23	0	0.80919326	0.999	0.966	0
Rpl23a	0	0.82031792	0.992	0.961	0
Rpl39	0	0.65099551	0.998	0.968	0
Rps13	0	0.58011367	0.997	0.967	0
Rps26	0	0.68225343	0.995	0.965	0
Rpl19	0	0.54549645	0.999	0.97	0
Rpl30	0	0.72548377	0.997	0.969	0
Rps3a1	0	0.74631576	0.997	0.97	0
Rpl13	0	0.55015765	0.998	0.971	0
Rpl17	0	0.63692285	0.996	0.969	0
Rps10	0	0.6633224	0.996	0.97	0
Rps28	0	0.99795765	0.996	0.971	0
Rpl38	0	0.58789913	0.999	0.976	0
Rps14	0	0.66450862	0.999	0.976	0
Rps24	0	0.94783498	0.999	0.978	0
Rps21	0	0.79009164	0.999	0.979	0
Rps8	0	0.69556689	0.999	0.979	0
Rps16	0	0.8332337	0.999	0.981	0
Rpl37a	0	0.67256943	1	0.983	0
Rplp1	0	1.03940961	0.999	0.982	0
Rplp2	0	0.86367291	0.999	0.983	0
Rps27	0	0.99617557	0.998	0.983	0
Rpl23	0	0.59433623	0.998	0.983	0
Rpl35a	0	0.77373036	0.997	0.983	0
Rps29	0	1.03121997	0.998	0.986	0
Tpt1	0	0.63766825	0.999	0.995	0
Rps3	1.059E-305	0.59005453	0.989	0.957	2.324E-301
Rpl37	8.967E-304	0.55313802	0.997	0.973	1.967E-299
Rps27a	1.161E-302	0.52703659	0.996	0.979	2.548E-298
Trib2	3.96E-302	2.35166714	0.378	0.117	8.688E-298
Rpl36	1.608E-299	0.58260026	0.995	0.957	3.528E-295
Rps18	1.442E-296	0.64086423	0.99	0.937	3.163E-292
Rpsa	6E-283	0.57313786	0.996	0.965	1.316E-278
Pik3ip1	9.17E-283	2.37124733	0.228	0.046	2.012E-278
Rpl31	1.869E-282	0.8201854	0.944	0.85	4.099E-278
Rpl27a	4.376E-282	0.49878913	0.998	0.972	9.6E-278
Trbc2	1.026E-281	1.01378623	0.777	0.373	2.251E-277
Gm14085	3.118E-277	5.13449855	0.058	0.003	6.84E-273
Ssh2	4.526E-276	1.6652304	0.629	0.33	9.93E-272
Rpl9	1.302E-274	0.54505789	0.993	0.959	2.857E-270
Rpl8	8.965E-269	0.57899925	0.991	0.958	1.967E-264
Dgka	2.827E-266	2.26441019	0.337	0.103	6.203E-262
Kif1b	4.15E-263	2.3399034	0.37	0.126	9.105E-259
Rpl32	5.356E-256	0.51774898	0.994	0.957	1.175E-251
Klhdc1	2.859E-250	3.59742389	0.107	0.012	6.272E-246
Fam78a	7.977E-248	3.48932492	0.129	0.018	1.75E-243

Auts2	5.466E-247	3.86533615	0.078	0.006	1.199E-242
Ifit1bl1	8.196E-245	4.03992024	0.07	0.005	1.798E-240
Ipcef1	4.607E-244	2.55015789	0.22	0.05	1.011E-239
Eef1b2	3.168E-241	0.73032838	0.942	0.869	6.949E-237
Gimap9	4.285E-236	1.94737641	0.368	0.127	9.4E-232
Patj	6.497E-235	3.20315626	0.114	0.015	1.425E-230
Rpl22	3.957E-226	0.53981845	0.986	0.946	8.68E-222
Rps5	1.866E-225	0.51376369	0.99	0.956	4.095E-221
Rps25	2.215E-224	0.51598484	0.992	0.956	4.86E-220
Smc4	5.569E-220	1.97053973	0.421	0.176	1.222E-215
Rpl3	5.866E-216	0.57156914	0.981	0.927	1.287E-211
Ndrg3	3.114E-214	2.87056399	0.223	0.058	6.832E-210
Rpl10a	1.648E-213	0.57636199	0.983	0.92	3.615E-209
Rpl18a	8.979E-213	0.44328033	0.997	0.969	1.97E-208
Rpl21	1.017E-212	0.51518595	0.993	0.953	2.232E-208
Rpl18	3.993E-211	0.4888759	0.994	0.96	8.76E-207
Cd3e	1.932E-210	1.19316459	0.592	0.283	4.238E-206
Eef1a1	1.265E-201	0.39336262	0.998	0.976	2.775E-197
Lcn4	1.35E-200	3.4571496	0.091	0.011	2.962E-196
1700062C10R	1.336E-198	7.8388978	0.021	0	2.93E-194
Rpl4	1.704E-195	0.69274422	0.928	0.837	3.738E-191
Mbnl1	6.115E-194	0.79170434	0.893	0.762	1.342E-189
Tmsb10	6.858E-193	0.64561303	0.984	0.889	1.505E-188
Tmem71	9.668E-189	2.24401706	0.225	0.063	2.121E-184
Gimap6	1.122E-187	1.07645917	0.692	0.403	2.461E-183
mt-Atp6	3.558E-186	0.46170477	0.995	0.964	7.807E-182
Ifi214	8.759E-186	2.37131045	0.159	0.034	1.922E-181
Trac	1.805E-185	1.31234004	0.487	0.224	3.959E-181
Rpl7	4.913E-182	0.49462955	0.982	0.945	1.078E-177
Chd3	6.899E-182	1.50193435	0.526	0.284	1.514E-177
Rpl26	3.148E-178	0.44215262	0.988	0.953	6.907E-174
Rps9	1.34E-175	0.35249436	0.997	0.991	2.941E-171
Rnf213	5.577E-173	2.35727019	0.31	0.12	1.223E-168
Fam169b	4.863E-172	1.79129547	0.269	0.088	1.067E-167
Ikzf1	2.601E-169	1.4609716	0.442	0.212	5.706E-165
Rps12	1.819E-168	0.40404096	0.997	0.981	3.991E-164
Gm42418	5.365E-168	0.25824779	1	1	1.177E-163
Bcl2	1.095E-167	1.68083393	0.325	0.125	2.403E-163
Ift80	2.062E-167	2.97893836	0.117	0.021	4.523E-163
Fam102a	3.046E-167	1.93769215	0.295	0.108	6.682E-163
Gm33994	8.875E-166	6.61389834	0.019	0	1.947E-161
Vipr1	1.275E-164	4.48807694	0.052	0.004	2.798E-160
Ifit3	5.331E-163	2.98170774	0.091	0.014	1.17E-158
Kbtbd11	1.25E-161	1.74409105	0.303	0.113	2.743E-157
Ccr7	1.933E-160	0.86274235	0.548	0.272	4.241E-156
Ddx60	1.481E-159	2.87974855	0.121	0.023	3.249E-155
Gm26551	8.216E-159	3.03702464	0.091	0.014	1.802E-154
Rpl34	4.801E-156	0.36448861	0.997	0.969	1.053E-151

Ptprc	2.219E-154	0.57601625	0.948	0.892	4.868E-150
Utrn	1.738E-153	1.44489837	0.456	0.235	3.813E-149
Cd8a	1.965E-153	1.58532279	0.228	0.07	4.311E-149
Shisa5	1.788E-151	0.98154796	0.748	0.585	3.923E-147
Inpp4b	2.035E-150	1.61310513	0.337	0.14	4.465E-146
Gramd3	7.655E-149	1.236599	0.497	0.259	1.679E-144
Rpl6	1.775E-147	0.42095014	0.994	0.954	3.894E-143
Rpl11	1.841E-145	0.43289529	0.987	0.945	4.04E-141
Cep97	1.351E-144	2.74972281	0.12	0.025	2.963E-140
Pdk1	1.513E-144	2.53175101	0.162	0.043	3.32E-140
Gm34006	1.469E-143	3.93260699	0.044	0.003	3.222E-139
Gtf2i	3.26E-143	1.67762384	0.336	0.147	7.152E-139
Rras2	1.929E-142	2.08280575	0.205	0.064	4.232E-138
Rplp0	1.082E-140	0.45707751	0.98	0.934	2.373E-136
Slamf6	4.29E-139	1.93838945	0.24	0.084	9.412E-135
B930036N10R	2.527E-138	2.30094301	0.234	0.083	5.543E-134
Fam189b	3.473E-138	2.48112285	0.149	0.038	7.62E-134
Bcl11b	5.476E-137	1.49562434	0.358	0.161	1.201E-132
Gm8369	2.083E-135	0.86961245	0.492	0.248	4.571E-131
Arhgap15	1.049E-134	1.513497	0.433	0.235	2.3E-130
Gm19261	6.736E-134	5.29209683	0.023	0.001	1.478E-129
Rpl22l1	1.736E-133	0.72929932	0.832	0.737	3.809E-129
Rack1	4.118E-133	0.4358344	0.974	0.936	9.034E-129
Dennd2d	9.82E-131	2.17838853	0.152	0.04	2.154E-126
Npm1	2.034E-130	0.58373051	0.927	0.852	4.462E-126
mt-Co1	5.55E-130	0.35332431	0.997	0.971	1.218E-125
Itm2a	9.991E-130	3.14916628	0.086	0.015	2.192E-125
Itk	3.775E-129	0.98278197	0.545	0.311	8.282E-125
F2rl1	1.057E-128	3.18854025	0.055	0.006	2.32E-124
Cdkn2d	1.747E-128	2.13981324	0.216	0.076	3.832E-124
Atp11b	7.818E-128	1.33036124	0.491	0.299	1.715E-123
Itga6	3.219E-125	2.28593495	0.161	0.046	7.061E-121
Rpl15	3.717E-122	0.43886157	0.976	0.914	8.154E-118
Tmie	7.692E-119	5.95569392	0.018	0	1.688E-114
Cd247	5.42E-118	1.52497921	0.29	0.124	1.189E-113
Arhgef1	1.315E-115	1.22554354	0.479	0.289	2.886E-111
Sptbn1	5.201E-115	1.4674589	0.376	0.196	1.141E-110
St8sia6	1.088E-112	3.16526325	0.083	0.016	2.386E-108
Skap1	2.225E-112	1.1809462	0.416	0.22	4.881E-108
Dusp10	1.873E-111	1.61359002	0.266	0.111	4.11E-107
St8sia1	2.259E-111	2.53141344	0.105	0.024	4.956E-107
Rapgef4	3.001E-110	3.15630386	0.055	0.007	6.584E-106
Grap2	2.639E-109	1.32512379	0.354	0.175	5.79E-105
Tespa1	3.987E-109	1.61441971	0.239	0.094	8.747E-105
Ifi27l2a	1.375E-108	0.50227429	0.659	0.467	3.016E-104
Gimap8	7.024E-108	1.56727167	0.25	0.102	1.541E-103
Rpl41	1.686E-107	0.27723375	0.999	0.989	3.699E-103
Rps27rt	8.08E-107	0.97388197	0.587	0.422	1.773E-102

B630019A10R	7.354E-106	3.80040124	0.036	0.003	1.613E-101
Rasgrp2	1.102E-105	1.68905128	0.247	0.103	2.418E-101
Gm26771	6.195E-105	5.39740642	0.019	0.001	1.359E-100
Ifi203	1.235E-102	1.50921122	0.391	0.222	2.7103E-98
Mgst2	3.771E-102	2.63470955	0.075	0.014	8.2723E-98
Klhdc2	6.917E-102	2.1925894	0.167	0.056	1.5176E-97
Atp1b1	7.886E-102	2.30961253	0.103	0.024	1.7301E-97
Rps2	7.087E-101	0.3622573	0.983	0.947	1.5549E-96
Ripor2	1.054E-100	1.34376494	0.286	0.128	2.3114E-96
Stk38	1.814E-100	1.61860752	0.285	0.134	3.9794E-96
Setx	7.849E-100	1.81380493	0.231	0.096	1.7219E-95
mt-Nd2	1.101E-99	0.60253743	0.848	0.777	2.4146E-95
Trbc1	1.138E-99	0.95274729	0.461	0.256	2.4966E-95
Eef1g	5.6E-99	0.7232499	0.724	0.615	1.2287E-94
Znrf3	5.643E-99	2.69502337	0.081	0.017	1.2379E-94
Ifit1	1.0154E-98	1.67776175	0.109	0.028	2.2278E-94
Gpr18	1.5849E-98	1.63007329	0.217	0.086	3.4772E-94
Mitd1	3.6E-97	2.09523486	0.148	0.047	7.898E-93
Usp18	4.9136E-97	2.99285012	0.075	0.015	1.078E-92
Gbp4	1.4338E-96	1.58761282	0.261	0.117	3.1455E-92
Gm19705	1.469E-96	3.32865999	0.045	0.006	3.2229E-92
Stat5b	1.0131E-94	1.21865275	0.452	0.277	2.2227E-90
Slc6a19	1.231E-94	6.71346024	0.011	0	2.7007E-90
Frat2	2.5649E-93	2.25127936	0.142	0.045	5.6272E-89
Saraf	7.7624E-92	0.96524625	0.539	0.375	1.703E-87
Gabrr2	2.5848E-91	5.82118611	0.014	0	5.6707E-87
Galnt6	1.0308E-90	1.92483949	0.196	0.078	2.2615E-86
Add3	3.8765E-90	1.57758388	0.263	0.124	8.5046E-86
Treml2	8.0667E-90	1.54253432	0.136	0.041	1.7698E-85
Limd2	1.1324E-88	1.00547862	0.484	0.312	2.4843E-84
Tgfbr3	7.8572E-86	2.54083477	0.072	0.015	1.7238E-81
Slfn1	2.1722E-85	1.55889802	0.178	0.067	4.7656E-81
Macf1	8.9312E-85	1.09670147	0.525	0.381	1.9594E-80
Smchd1	1.3883E-84	0.95948962	0.576	0.44	3.0457E-80
Gimap4	1.2296E-83	0.81679423	0.468	0.278	2.6976E-79
Pik3cd	1.6179E-83	1.42910046	0.283	0.143	3.5495E-79
Phf11c	1.6355E-83	3.17041765	0.046	0.007	3.5881E-79
Gm9889	5.769E-83	3.66948754	0.025	0.002	1.2657E-78
Prkch	2.3774E-82	1.31023628	0.296	0.151	5.2158E-78
Rpl14	5.8949E-82	0.39848644	0.944	0.906	1.2933E-77
mt-Co3	1.0885E-81	0.28362779	0.993	0.967	2.388E-77
mt-Nd4	2.5809E-81	0.39706021	0.949	0.907	5.6622E-77
Trim56	1.7433E-80	1.73918791	0.191	0.078	3.8246E-76
Arhgap45	2.2981E-80	1.12459673	0.425	0.269	5.0418E-76
Rpl28	5.067E-80	0.31357177	0.982	0.945	1.1117E-75
Dnah8	3.1504E-79	2.99524534	0.045	0.007	6.9118E-75
Sp100	6.2307E-79	1.2060946	0.402	0.252	1.367E-74
Zfp365	1.3796E-78	4.73092501	0.016	0.001	3.0267E-74

Arhgef18	4.4642E-78	1.7824713	0.117	0.036	9.794E-74
Ly6c1	6.6572E-77	2.9899216	0.055	0.01	1.4605E-72
Rhoh	9.1458E-76	0.94223126	0.436	0.272	2.0065E-71
Themis	3.2409E-75	1.47316387	0.217	0.097	7.1103E-71
Cd3g	5.7193E-75	0.48176392	0.532	0.309	1.2547E-70
Phf20l1	3.8046E-74	1.03874009	0.451	0.306	8.3468E-70
mt-Cytb	4.4978E-74	0.34450592	0.972	0.931	9.8678E-70
Hdac7	5.9422E-74	1.65695374	0.186	0.078	1.3037E-69
Dph5	6.8263E-74	1.73929303	0.202	0.09	1.4976E-69
Crlf3	2.3419E-72	0.98670165	0.495	0.355	5.138E-68
Gpr146	9.3093E-72	2.08850778	0.159	0.064	2.0424E-67
Ifi47	1.2563E-71	1.42699267	0.195	0.085	2.7562E-67
Ly6c2	1.9099E-71	1.7643445	0.211	0.097	4.1901E-67
Fgf13	1.9753E-71	3.17994309	0.036	0.005	4.3335E-67
Trim12a	6.7533E-71	1.42903775	0.255	0.129	1.4816E-66
Igtp	1.1295E-70	1.19572759	0.25	0.123	2.4779E-66
Slc12a7	3.2415E-70	2.36281882	0.091	0.026	7.1116E-66
Trim34a	3.5337E-69	2.02895672	0.114	0.038	7.7525E-65
Hspa1b	1.3181E-67	0.53535254	0.3	0.159	2.8918E-63
Bcl9l	2.4978E-67	2.12618274	0.092	0.027	5.48E-63
Plcxd2	6.6882E-66	1.04084078	0.303	0.165	1.4673E-61
Rpl27	5.4427E-65	0.36313675	0.923	0.881	1.1941E-60
Cracr2a	7.1977E-65	2.58641841	0.052	0.01	1.5791E-60
Tbxa2r	8.1625E-65	3.44171127	0.024	0.002	1.7908E-60
Gimap5	7.0071E-64	0.90304912	0.373	0.223	1.5373E-59
Pdcd4	7.2955E-64	0.88259967	0.461	0.316	1.6006E-59
Tet1	8.7943E-64	4.59866264	0.018	0.001	1.9294E-59
Arid5a	8.2888E-63	1.21010549	0.393	0.267	1.8185E-58
Ifi209	4.8778E-62	1.46980577	0.223	0.112	1.0701E-57
Ets1	1.0363E-61	0.38056275	0.883	0.659	2.2735E-57
Sesn3	2.0222E-61	1.05633819	0.352	0.216	4.4364E-57
Plcg1	2.8726E-59	1.56152523	0.161	0.069	6.3022E-55
Vps37b	7.2311E-59	0.60135013	0.732	0.663	1.5864E-54
Ccnd2	9.5629E-58	0.7367936	0.447	0.303	2.098E-53
Faah	1.6501E-57	1.28096519	0.209	0.102	3.6202E-53
Il6st	2.2808E-57	1.55656929	0.206	0.104	5.0038E-53
Il6ra	2.62E-57	1.83476846	0.182	0.088	5.748E-53
Atp1b3	3.4446E-57	0.95812697	0.448	0.336	7.5572E-53
Cd55	2.9126E-56	0.88747906	0.196	0.091	6.3899E-52
Rasa3	2.9239E-56	1.35537729	0.209	0.105	6.4148E-52
Tgtp1	3.8799E-56	2.79473264	0.037	0.006	8.512E-52
Art2b	4.0486E-56	2.84218031	0.058	0.014	8.8822E-52
Iigp1	5.0212E-56	1.94663006	0.091	0.03	1.1016E-51
Pole2	5.5042E-56	3.26011547	0.032	0.005	1.2076E-51
Slfn8	8.0575E-56	1.77644391	0.143	0.061	1.7677E-51
Ehd3	4.3327E-55	1.87026354	0.091	0.03	9.5056E-51
Sh3bp5	6.306E-55	1.19781275	0.191	0.091	1.3835E-50
Tnik	1.8151E-54	1.35031838	0.185	0.088	3.9822E-50

Prkd2	5.5303E-54	1.77668044	0.101	0.035	1.2133E-49
Ifngas1	1.194E-53	4.67474949	0.011	0.001	2.6195E-49
Fus	1.7021E-53	0.52116312	0.772	0.75	3.7342E-49
Irf1	1.7606E-53	0.79567545	0.353	0.226	3.8625E-49
Wdr89	2.0969E-53	0.84496534	0.48	0.376	4.6003E-49
Jak1	1.4503E-51	0.58911084	0.664	0.592	3.1818E-47
Tnrc6c	3.0959E-51	1.0946317	0.314	0.198	6.7922E-47
Mctp2	1.3665E-50	1.3782131	0.114	0.044	2.9981E-46
Ppm1h	1.3715E-50	1.58041819	0.173	0.084	3.0089E-46
Lck	1.4154E-50	0.90007407	0.323	0.197	3.1053E-46
Setd7	3.5024E-50	1.76313933	0.144	0.064	7.6838E-46
Kcna2	4.1035E-50	4.15494741	0.014	0.001	9.0027E-46
Klhl6	4.1401E-50	1.37427634	0.182	0.09	9.0829E-46
Adk	1.4095E-49	1.76957935	0.139	0.061	3.0924E-45
Lypd6b	2.8729E-49	3.54134097	0.016	0.001	6.3029E-45
Nsd3	3.1004E-49	0.81000584	0.511	0.407	6.802E-45
Cblb	1.2457E-48	0.90124481	0.411	0.291	2.7328E-44
Hs3st3b1	7.8207E-48	2.00188015	0.074	0.023	1.7158E-43
Ugcg	8.8412E-48	0.67159239	0.417	0.29	1.9397E-43
Gm43352	1.6589E-47	3.27686155	0.021	0.003	3.6394E-43
1810041H14R	2.2314E-47	2.52831893	0.052	0.013	4.8956E-43
Bach2	1.1054E-46	1.12912548	0.19	0.096	2.425E-42
Pxylp1	2.9361E-46	3.86827466	0.017	0.002	6.4414E-42
Cacna2d4	3.9633E-46	4.0640012	0.011	0.001	8.6951E-42
Elf1	5.5811E-46	0.56660613	0.575	0.462	1.2244E-41
Map7	1.1587E-45	2.59123186	0.042	0.009	2.542E-41
Slfn5	1.3045E-45	1.69196634	0.164	0.081	2.862E-41
Pacsin1	2.4522E-45	2.46440188	0.047	0.012	5.3799E-41
Mndal	2.6822E-45	1.03372256	0.37	0.264	5.8845E-41
Thada	3.6487E-45	1.78540908	0.103	0.04	8.0049E-41
Igsf23	4.1042E-45	3.60752276	0.021	0.003	9.0042E-41
Atp8b4	7.0495E-45	1.83066145	0.113	0.047	1.5466E-40
Dtx3l	1.7088E-44	1.38700353	0.207	0.116	3.7488E-40
Fyb	1.7403E-44	0.44500352	0.643	0.506	3.8181E-40
Rcbtb1	2.4657E-44	1.91100035	0.09	0.033	5.4094E-40
Arid4a	5.1152E-44	1.1219043	0.308	0.205	1.1222E-39
Tubb5	5.7324E-44	0.63412822	0.484	0.382	1.2576E-39
Srpk2	1.244E-43	0.95779076	0.308	0.198	2.7293E-39
Gimap1	1.6463E-43	0.74032064	0.399	0.274	3.6118E-39
Eml5	3.2125E-43	1.94000536	0.073	0.024	7.0479E-39
Stat1	4.6258E-43	0.78986241	0.452	0.349	1.0149E-38
Sbno2	5.8444E-43	1.11499401	0.342	0.244	1.2822E-38
Gm37387	8.1213E-43	2.56718823	0.035	0.007	1.7817E-38
Slc44a2	8.4497E-43	1.2734209	0.158	0.077	1.8538E-38
Zbp1	2.7713E-42	1.41540637	0.211	0.121	6.08E-38
Trappc12	4.9424E-42	1.64047182	0.111	0.047	1.0843E-37
Herc6	5.2366E-42	1.65726042	0.121	0.053	1.1489E-37
Aff3	5.6387E-42	1.4445959	0.095	0.036	1.2371E-37

Pou6f1	6.9293E-42	1.54109087	0.078	0.027	1.5202E-37
Trim13	9.8472E-42	2.61681918	0.048	0.012	2.1604E-37
Cox7a2l	1.798E-41	0.5561525	0.656	0.595	3.9445E-37
Klk8	3.4268E-41	1.27441251	0.182	0.096	7.518E-37
Nsa2	1.0543E-40	0.45608892	0.733	0.687	2.3129E-36
Zcchc11	1.4726E-40	0.547617	0.628	0.553	3.2307E-36
Prrc2c	1.7309E-40	0.43666974	0.752	0.704	3.7975E-36
Pced1b	1.961E-40	1.86071042	0.075	0.026	4.3022E-36
Setd1b	2.0338E-40	1.3975099	0.147	0.072	4.462E-36
Pja1	2.2033E-40	1.44928066	0.147	0.072	4.8339E-36
Cd27	4.3648E-40	0.81013162	0.198	0.106	9.5758E-36
Trim12c	5.339E-40	1.32593016	0.181	0.098	1.1713E-35
Numa1	1.5384E-39	0.97182904	0.295	0.194	3.3752E-35
4932438A13R	2.4511E-39	0.76531108	0.473	0.383	5.3775E-35
Tnrc6b	2.75E-39	0.69706052	0.453	0.344	6.0333E-35
Evl	3.0134E-39	1.32777918	0.217	0.129	6.611E-35
Tgfbr2	3.7914E-39	0.95106719	0.333	0.233	8.318E-35
Lat	5.2819E-39	0.6750193	0.347	0.226	1.1588E-34
Tnfrsf26	7.9123E-39	1.64517357	0.077	0.028	1.7359E-34
Gramd4	1.6491E-38	1.47705794	0.133	0.064	3.618E-34
Casp8	2.056E-38	1.32929479	0.194	0.111	4.5107E-34
Ssbp2	3.0418E-38	1.66846732	0.109	0.048	6.6734E-34
Rnf19a	3.4391E-38	1.1188732	0.254	0.16	7.5451E-34
Il7r	3.5139E-38	0.40444123	0.53	0.393	7.7091E-34
Cd2	5.9896E-38	0.51962166	0.416	0.284	1.314E-33
Prpf38b	1.0984E-37	0.64142239	0.547	0.477	2.4097E-33
Nup210	1.2729E-37	1.39067773	0.179	0.099	2.7927E-33
Zcchc7	1.9872E-37	0.84880141	0.385	0.288	4.3598E-33
Ranbp10	2.147E-37	1.65823757	0.122	0.057	4.7103E-33
Eif4a2	3.2347E-36	0.61215986	0.519	0.442	7.0967E-32
Rpl13a	3.2767E-36	0.52336553	0.625	0.58	7.1888E-32
Srpk1	3.4078E-36	1.01333348	0.277	0.184	7.4764E-32
Mllt3	3.4086E-36	1.22375037	0.194	0.11	7.4781E-32
H2-T24	7.1338E-36	2.68147596	0.031	0.007	1.5651E-31
Il17ra	1.2216E-35	1.01369622	0.317	0.223	2.6802E-31
Atm	1.8143E-35	1.55527615	0.095	0.04	3.9804E-31
Ddx58	3.7451E-35	1.41180466	0.133	0.066	8.2165E-31
Oas3	4.587E-35	1.66290026	0.067	0.024	1.0063E-30
Mettl27	5.6226E-35	3.71145308	0.014	0.001	1.2335E-30
Samd9l	7.5177E-35	1.06759713	0.211	0.125	1.6493E-30
Ccr9	8.4672E-34	1.9595513	0.065	0.023	1.8576E-29
Hspe1	8.8524E-34	0.35952451	0.678	0.629	1.9421E-29
Lrig1	1.1172E-33	1.86800487	0.054	0.017	2.451E-29
Tmem64	1.4391E-33	0.94824632	0.334	0.234	3.1573E-29
Traf3ip3	7.1588E-33	1.41659402	0.135	0.069	1.5706E-28
Pitpnc1	1.1313E-32	0.94661893	0.253	0.165	2.4819E-28
A630023P12R	1.4334E-32	2.13664218	0.043	0.012	3.1447E-28
Rictor	2.1759E-32	0.73750267	0.405	0.319	4.7737E-28

9930111J21Ril	2.8888E-32	1.16402405	0.201	0.122	6.3378E-28
Zscan12	3.9634E-32	2.71482133	0.028	0.006	8.6953E-28
Ap1ar	1.0468E-31	1.45961485	0.124	0.062	2.2965E-27
Dopey1	2.3206E-31	1.3663315	0.139	0.073	5.0912E-27
Tecpr1	2.4473E-31	0.7291706	0.318	0.22	5.3691E-27
Gbp9	2.8187E-31	1.46369825	0.115	0.056	6.1839E-27
Tha1	3.446E-31	2.91908149	0.025	0.005	7.5601E-27
Ash1l	3.6433E-31	0.89579163	0.314	0.226	7.9931E-27
Oas2	3.6474E-31	2.11803168	0.044	0.013	8.0021E-27
Zbtb20	4.6839E-31	0.89228078	0.286	0.196	1.0276E-26
Gm44175	5.7375E-31	3.10041845	0.019	0.003	1.2588E-26
Myh9	5.8306E-31	0.55951916	0.617	0.582	1.2792E-26
BC147527	6.9035E-31	3.43472201	0.017	0.003	1.5146E-26
Trim30a	7.0947E-31	1.15479014	0.234	0.154	1.5565E-26
Gm42809	8.8439E-31	3.92716691	0.01	0.001	1.9403E-26
Eif3e	3.4545E-30	0.54280112	0.604	0.573	7.5787E-26
Bin2	4.2503E-30	1.23744681	0.206	0.131	9.3247E-26
Letm2	4.2966E-30	1.39766983	0.1	0.046	9.4262E-26
Uba52	6.9738E-30	0.5386504	0.562	0.518	1.53E-25
Nckap5l	7.6852E-30	2.9443879	0.021	0.004	1.686E-25
Pitpnm2	8.7533E-30	1.74857663	0.054	0.019	1.9204E-25
Zfp644	1.4611E-29	0.94354185	0.293	0.21	3.2054E-25
Snrnp70	7.5631E-29	0.53139234	0.566	0.521	1.6593E-24
Grk6	7.9288E-29	1.01864248	0.209	0.132	1.7395E-24
Mycbp2	9.1388E-29	0.66201332	0.525	0.473	2.005E-24
Gm12840	1.1924E-28	0.48640365	0.323	0.225	2.6159E-24
Dzip1	1.2165E-28	2.58817301	0.025	0.005	2.6688E-24
Ccdc28a	1.2384E-28	1.6344999	0.064	0.024	2.7169E-24
Psme2b	1.2417E-28	1.67154705	0.075	0.031	2.7242E-24
Parp9	1.5573E-28	1.47517168	0.119	0.061	3.4165E-24
Nxpe3	1.8526E-28	1.72632274	0.066	0.026	4.0643E-24
Foxp1	2.6692E-28	0.36451079	0.564	0.472	5.856E-24
Lars2	3.0518E-28	0.31974256	0.748	0.733	6.6954E-24
Tanc1	4.0455E-28	1.99665119	0.047	0.015	8.8753E-24
Gpr174	5.4809E-28	0.92797732	0.157	0.087	1.2025E-23
Stk17b	9.4839E-28	0.32364918	0.78	0.767	2.0807E-23
Gm43653	1.4244E-27	3.67733146	0.012	0.001	3.1249E-23
Zeb1	1.434E-27	0.54007677	0.446	0.35	3.146E-23
Pde3b	1.4837E-27	0.68312477	0.351	0.266	3.2551E-23
Ppp2r5a	1.5598E-27	0.86471833	0.356	0.285	3.4221E-23
Usp28	1.5858E-27	2.19312351	0.042	0.013	3.4792E-23
Tcof1	1.6847E-27	1.10719626	0.204	0.13	3.6961E-23
mt-Nd5	1.7647E-27	0.48636998	0.598	0.56	3.8716E-23
Bola2	1.7769E-27	0.63994541	0.465	0.407	3.8983E-23
Hist4h4	2.3104E-27	1.83848297	0.049	0.017	5.0689E-23
Kif21b	3.6523E-27	1.40568431	0.141	0.079	8.0129E-23
Rif1	8.0558E-27	1.06888927	0.204	0.131	1.7674E-22
A230050P20R	8.7027E-27	1.90759628	0.045	0.015	1.9093E-22

D930028M14F	9.7526E-27	3.9126604	0.01	0.001	2.1396E-22
Kri1	1.0613E-26	1.28846656	0.113	0.058	2.3284E-22
Ppic	2.3349E-26	2.1200705	0.033	0.009	5.1226E-22
Casc4	3.6217E-26	1.62262038	0.048	0.017	7.9457E-22
Rasal3	5.0904E-26	1.27614153	0.125	0.067	1.1168E-21
Zfp280d	1.0847E-25	1.01258092	0.18	0.111	2.3797E-21
Gbp6	1.4851E-25	1.93163238	0.041	0.013	3.2582E-21
Ptpn18	1.5666E-25	0.4237972	0.648	0.614	3.4369E-21
Rassf5	1.6331E-25	0.9711785	0.209	0.136	3.5828E-21
Sox4	1.8109E-25	2.0448787	0.036	0.011	3.9729E-21
Herc3	2.3138E-25	1.44157813	0.094	0.046	5.0763E-21
Nup153	2.3365E-25	1.07026362	0.228	0.157	5.126E-21
Smg1	2.7448E-25	0.68497745	0.388	0.314	6.0218E-21
Tcf20	4.4392E-25	1.07930853	0.194	0.126	9.7392E-21
Car12	4.6719E-25	3.21514703	0.011	0.001	1.025E-20
Arid1a	5.76E-25	0.87079741	0.272	0.196	1.2637E-20
Ttc28	5.8545E-25	1.34302563	0.076	0.034	1.2844E-20
Xaf1	2.1571E-24	1.51665947	0.104	0.054	4.7325E-20
Bicdl1	2.4177E-24	1.71760042	0.047	0.017	5.3042E-20
Usp53	3.2807E-24	1.37997675	0.08	0.037	7.1976E-20
Stx1a	3.3446E-24	2.94528259	0.013	0.002	7.3376E-20
Tbc1d10c	4.7049E-24	1.15542121	0.122	0.066	1.0322E-19
Izumo1r	9.6859E-24	1.05985678	0.067	0.029	2.125E-19
A230072E10R	1.1644E-23	3.28401606	0.01	0.001	2.5545E-19
Mdm4	1.2842E-23	0.87767244	0.257	0.185	2.8174E-19
Nlrc5	2.1017E-23	1.13959801	0.143	0.084	4.6109E-19
Ifit2	2.1185E-23	1.47883936	0.043	0.015	4.6477E-19
Cbx7	2.6771E-23	1.11434227	0.093	0.046	5.8733E-19
2810454H06R	5.0409E-23	1.42368987	0.081	0.038	1.1059E-18
Psip1	5.3418E-23	0.91659176	0.213	0.143	1.1719E-18
Il27ra	7.7444E-23	1.28567142	0.104	0.054	1.699E-18
Socs1	9.178E-23	0.8208787	0.18	0.114	2.0136E-18
Gprc5b	1.2165E-22	3.36800949	0.014	0.002	2.6688E-18
Bclaf1	1.2352E-22	0.49544414	0.536	0.497	2.71E-18
Cfap46	1.2716E-22	2.94483691	0.014	0.002	2.7899E-18
Dcaf17	1.2915E-22	1.53270304	0.071	0.032	2.8334E-18
Patz1	1.4897E-22	1.61026215	0.055	0.022	3.2683E-18
Gm6297	1.906E-22	2.68349136	0.02	0.004	4.1816E-18
Phip	2.1812E-22	0.67451668	0.345	0.271	4.7853E-18
Naca	2.2351E-22	0.25254173	0.858	0.831	4.9035E-18
Lbr	3.4759E-22	0.70862246	0.317	0.245	7.6257E-18
Top2b	5.1545E-22	0.70404407	0.366	0.298	1.1308E-17
Rtp4	6.3903E-22	0.90865552	0.099	0.052	1.402E-17
Ankrd10	8.3492E-22	1.25491299	0.116	0.065	1.8317E-17
Ddb2	9.5706E-22	1.39539004	0.071	0.033	2.0997E-17
Kmt2a	1.1204E-21	0.64467488	0.397	0.326	2.4581E-17
Ttc3	1.3752E-21	1.06300553	0.166	0.105	3.017E-17
Rab3ip	3.2534E-21	1.61386791	0.082	0.041	7.1376E-17

Gm42722	4.0741E-21	1.79826584	0.032	0.01	8.9381E-17
Tpr	4.3951E-21	0.56646752	0.538	0.514	9.6425E-17
Btbd11	4.7258E-21	1.49397158	0.042	0.015	1.0368E-16
Ggt1	8.1349E-21	2.95067209	0.016	0.003	1.7847E-16
Senp7	1.43E-20	1.25625326	0.111	0.062	3.1373E-16
Zfp652	1.477E-20	1.23266223	0.136	0.082	3.2403E-16
Ikzf4	1.6769E-20	1.66535288	0.041	0.015	3.6789E-16
Zfp827	2.0692E-20	2.2828961	0.021	0.005	4.5396E-16
Zc3h6	4.1825E-20	1.7245561	0.045	0.018	9.176E-16
Gbp8	4.9914E-20	1.39377225	0.1	0.055	1.0951E-15
Nin	5.4362E-20	1.04335758	0.191	0.13	1.1927E-15
Eef1d	7.5294E-20	0.40383756	0.603	0.578	1.6519E-15
L3mbtl3	8.0404E-20	1.48222571	0.065	0.03	1.764E-15
Hmg20a	9.0391E-20	1.47515402	0.079	0.039	1.9831E-15
Peli1	1.6467E-19	0.40719932	0.528	0.469	3.6128E-15
Hsd11b1	1.8072E-19	1.44746482	0.054	0.023	3.9647E-15
Gm26732	2.2177E-19	2.92314139	0.011	0.002	4.8653E-15
Klf13	2.39E-19	0.66598986	0.369	0.311	5.2434E-15
Samhd1	3.1651E-19	0.43847165	0.43	0.37	6.944E-15
Txnip	3.4207E-19	0.48930322	0.311	0.234	7.5047E-15
Gm26825	6.83E-19	1.5977242	0.058	0.026	1.4984E-14
Tle4	7.5852E-19	0.68073155	0.303	0.236	1.6641E-14
Slc17a9	8.1659E-19	1.88759767	0.032	0.011	1.7915E-14
Dtx1	8.9925E-19	0.87886102	0.1	0.054	1.9729E-14
Ccdc125	9.1431E-19	1.85671824	0.025	0.007	2.0059E-14
2810001G20R	1.0239E-18	1.48859755	0.071	0.035	2.2463E-14
Kmt2d	1.1028E-18	0.97517738	0.177	0.119	2.4193E-14
4932422M17F	1.1058E-18	1.60685728	0.044	0.018	2.4261E-14
Ss18	1.2003E-18	0.78609544	0.229	0.165	2.6334E-14
D830025C05F	1.2825E-18	1.45384172	0.054	0.023	2.8136E-14
Ankrd11	1.5459E-18	0.43101864	0.614	0.598	3.3915E-14
Degs2	1.6943E-18	2.71002064	0.017	0.004	3.7171E-14
Ifitm10	1.8207E-18	2.59429705	0.013	0.002	3.9945E-14
Hist2h2ab	1.9011E-18	2.52445677	0.018	0.004	4.1707E-14
Hdgfl3	2.2015E-18	1.4039222	0.062	0.029	4.83E-14
Fam208b	2.9096E-18	0.90902813	0.164	0.108	6.3833E-14
Slc37a1	2.963E-18	1.92104615	0.033	0.011	6.5005E-14
Fam208a	3.0666E-18	0.90615082	0.19	0.131	6.7277E-14
Rnf167	3.9066E-18	1.02238543	0.161	0.106	8.5708E-14
Tgtp2	4.0227E-18	1.96943215	0.039	0.015	8.8253E-14
Snrpg	5.0797E-18	0.3839812	0.604	0.587	1.1144E-13
Hsh2d	5.5977E-18	1.72742071	0.027	0.008	1.2281E-13
Mcm4	1.028E-17	1.10484751	0.147	0.096	2.2553E-13
Carns1	2.2815E-17	1.9076158	0.025	0.008	5.0055E-13
2410002F23Ri	2.3623E-17	1.03736678	0.115	0.068	5.1826E-13
Mfsd6	2.4685E-17	0.77885876	0.213	0.152	5.4156E-13
Prrg1	2.4847E-17	2.8503259	0.015	0.003	5.4513E-13
Adi1	3.5951E-17	1.48222896	0.058	0.027	7.8873E-13

Ube2h	3.6762E-17	0.84725425	0.265	0.209	8.0651E-13
Tsc22d3	4.4229E-17	0.48267524	0.371	0.303	9.7034E-13
Zfp292	6.6912E-17	0.67433568	0.306	0.246	1.468E-12
Ikbke	6.8115E-17	1.18744594	0.097	0.055	1.4944E-12
Jun	7.0879E-17	0.30589825	0.349	0.278	1.555E-12
Atf7ip	8.4027E-17	0.66013682	0.265	0.203	1.8435E-12
Gm4707	9.1242E-17	1.36892848	0.074	0.039	2.0018E-12
Rev3l	1.04E-16	1.04693661	0.133	0.084	2.2816E-12
Clk1	1.5427E-16	0.3192408	0.682	0.683	3.3845E-12
Upf2	1.7685E-16	0.84668416	0.211	0.153	3.8798E-12
Uvssa	2.0237E-16	1.46640587	0.058	0.028	4.4397E-12
Socs3	2.4671E-16	0.4666174	0.375	0.311	5.4126E-12
Bptf	2.6814E-16	0.62250046	0.352	0.297	5.8828E-12
Atrx	2.706E-16	0.5652962	0.476	0.445	5.9367E-12
Atad2	2.8369E-16	0.76646759	0.232	0.172	6.2239E-12
Snhg12	3.6083E-16	0.52576166	0.346	0.286	7.9162E-12
Dcaf1	3.9266E-16	1.01002314	0.124	0.077	8.6146E-12
Prmt3	4.2653E-16	1.24249162	0.079	0.043	9.3576E-12
Ly9	4.6708E-16	1.05885367	0.117	0.072	1.0247E-11
Adcy6	4.7574E-16	3.07754065	0.014	0.003	1.0437E-11
Ifit3b	4.9503E-16	1.88313065	0.016	0.004	1.0861E-11
Gm47438	7.0008E-16	2.19192981	0.018	0.005	1.5359E-11
Pdlim1	7.4126E-16	0.84659048	0.166	0.114	1.6263E-11
Acer2	7.5712E-16	2.07715837	0.017	0.004	1.661E-11
Sh2d1a	8.4828E-16	0.72113003	0.126	0.078	1.8611E-11
Tox	8.8648E-16	0.63916768	0.135	0.085	1.9449E-11
Nfrkb	1.2262E-15	1.04431165	0.125	0.079	2.6901E-11
Ccnd3	1.2428E-15	1.00118135	0.155	0.105	2.7265E-11
Acss2	1.7407E-15	1.53007645	0.053	0.025	3.8189E-11
Rhobtb2	2.1745E-15	0.92278389	0.068	0.035	4.7707E-11
A930029G22R	2.2642E-15	1.81510101	0.022	0.007	4.9675E-11
Fntb	2.5306E-15	2.21893577	0.02	0.006	5.552E-11
Dkc1	2.9969E-15	1.06442834	0.088	0.05	6.575E-11
Tmlhe	4.6115E-15	2.7462371	0.011	0.002	1.0117E-10
Rfx3	5.7542E-15	1.40400195	0.048	0.022	1.2624E-10
Rbl1	6.9134E-15	1.58342917	0.061	0.031	1.5167E-10
Gm15675	1.0952E-14	2.11486339	0.013	0.003	2.4028E-10
Pag1	1.1267E-14	0.78225719	0.175	0.123	2.4718E-10
Setd4	1.4254E-14	1.92756125	0.029	0.011	3.1272E-10
Camsap2	1.8737E-14	1.4105609	0.077	0.043	4.1108E-10
Gm37065	1.8773E-14	2.42996719	0.017	0.004	4.1187E-10
Tars2	1.9124E-14	1.42535664	0.055	0.027	4.1956E-10
Crim1	2.3234E-14	0.94474524	0.088	0.05	5.0973E-10
Pim2	2.4147E-14	0.80298762	0.104	0.063	5.2977E-10
Ttc13	2.5054E-14	1.66580312	0.041	0.018	5.4966E-10
Bdh1	3.0967E-14	1.73563136	0.032	0.012	6.7938E-10
Crybg1	3.1806E-14	1.09742438	0.177	0.129	6.978E-10
Yeats2	3.7947E-14	1.25110498	0.072	0.039	8.3252E-10

A430078G23R	3.9476E-14	1.07817094	0.042	0.019	8.6606E-10
1700056E22R	5.3003E-14	1.78344535	0.036	0.015	1.1628E-09
Zc3hav1	5.7701E-14	0.48947759	0.476	0.445	1.2659E-09
Sgpp1	6.0065E-14	1.16290768	0.092	0.055	1.3178E-09
Spata13	6.8508E-14	0.66493949	0.178	0.127	1.503E-09
Nlk	7.095E-14	0.9860596	0.102	0.063	1.5566E-09
Gm26640	7.211E-14	2.66328645	0.011	0.002	1.582E-09
Zc3h12d	7.5606E-14	1.0190346	0.085	0.049	1.6587E-09
Selplg	8.5945E-14	0.36834736	0.456	0.412	1.8855E-09
Ldb1	8.7821E-14	0.91755048	0.159	0.112	1.9267E-09
Raver2	8.906E-14	2.55733615	0.01	0.002	1.9539E-09
Arhgap27os3	8.9408E-14	1.93452368	0.023	0.008	1.9615E-09
Bzw2	9.2395E-14	0.71558992	0.229	0.177	2.0271E-09
Nlrc3	1.4226E-13	1.00580238	0.075	0.042	3.121E-09
Esyt2	1.5533E-13	0.53211436	0.307	0.251	3.4079E-09
Abraxas1	1.5898E-13	1.61492052	0.027	0.01	3.4879E-09
Zfp946	1.6578E-13	1.86749701	0.03	0.012	3.637E-09
Card6	2.072E-13	0.86699453	0.084	0.049	4.5457E-09
Dock11	2.634E-13	0.73665957	0.199	0.15	5.7787E-09
Sos1	2.9661E-13	1.16973721	0.088	0.053	6.5073E-09
Smad4	3.0486E-13	0.75458988	0.291	0.249	6.6883E-09
Xrn2	3.5027E-13	0.63596858	0.346	0.309	7.6847E-09
Itpr2	3.5304E-13	0.59553197	0.259	0.205	7.7454E-09
Ankrd44	3.8951E-13	0.51623613	0.364	0.317	8.5456E-09
Zfp945	3.9324E-13	1.22149287	0.07	0.039	8.6273E-09
Rnf145	3.9848E-13	0.66396883	0.187	0.136	8.7423E-09
Parp14	4.0115E-13	0.69540657	0.224	0.174	8.8009E-09
Rgs10	4.1035E-13	0.69055993	0.338	0.298	9.0028E-09
Gprasp1	4.2491E-13	1.2216583	0.054	0.027	9.322E-09
Hid1	4.3018E-13	1.86967214	0.026	0.01	9.4377E-09
Orc5	5.8137E-13	2.04742714	0.025	0.009	1.2755E-08
Slc25a40	5.8485E-13	1.1044418	0.071	0.04	1.2831E-08
Ifngr2	6.4814E-13	1.01779376	0.199	0.156	1.422E-08
Bend4	6.5097E-13	0.96320001	0.085	0.05	1.4282E-08
Pycr2	7.3431E-13	1.32522268	0.077	0.045	1.611E-08
Mettl8	7.3884E-13	1.57102881	0.024	0.009	1.6209E-08
E230001N04R	7.7048E-13	1.53685725	0.027	0.01	1.6904E-08
Dyrk2	7.9384E-13	0.8663604	0.198	0.152	1.7416E-08
Xndc1	1.2381E-12	1.27225403	0.038	0.017	2.7162E-08
Msh2	1.4236E-12	1.31136733	0.06	0.032	3.1232E-08
Slc14a1	1.456E-12	1.69092912	0.031	0.013	3.1943E-08
Kidins220	1.6517E-12	1.01118153	0.139	0.097	3.6236E-08
Fam53a	1.9293E-12	1.31231963	0.064	0.035	4.2326E-08
Atic	2.1992E-12	1.1312121	0.102	0.065	4.8249E-08
Unc5cl	2.4446E-12	1.88000432	0.017	0.005	5.3633E-08
Aak1	3.3049E-12	0.78483142	0.174	0.128	7.2507E-08
Swt1	3.5601E-12	0.72338991	0.196	0.148	7.8106E-08
Birc6	4.4849E-12	0.55152753	0.392	0.361	9.8394E-08

Ldlrap1	4.5181E-12	1.34595947	0.074	0.044	9.9123E-08
Vps13a	5.8233E-12	0.77137052	0.216	0.171	1.2776E-07
Gpatch4	6.093E-12	1.39171409	0.05	0.026	1.3367E-07
Gm37170	6.1084E-12	2.33656256	0.013	0.004	1.3401E-07
mt-Nd4l	6.6295E-12	0.54005426	0.386	0.356	1.4545E-07
Acot2	9.401E-12	1.11675799	0.064	0.035	2.0625E-07
Gm10073	9.8222E-12	1.05485108	0.126	0.088	2.1549E-07
Pura	1.3587E-11	0.73870072	0.219	0.174	2.9809E-07
Gm16158	1.4239E-11	2.29117529	0.01	0.002	3.1238E-07
Kmt2c	1.4301E-11	0.69702474	0.253	0.209	3.1374E-07
Oas1c	1.5077E-11	1.85931002	0.018	0.006	3.3078E-07
Fam3c	1.7073E-11	1.08569821	0.116	0.079	3.7457E-07
Efr3a	1.7095E-11	0.79155927	0.222	0.18	3.7505E-07
Fam118a	1.8234E-11	1.19584555	0.059	0.033	4.0004E-07
Siah1a	2.3159E-11	1.18127947	0.081	0.049	5.0809E-07
Krba1	2.5474E-11	1.47304873	0.028	0.012	5.5886E-07
Nipbl	2.6555E-11	0.46563467	0.435	0.41	5.8258E-07
Zfp592	2.6778E-11	0.89624847	0.128	0.089	5.8749E-07
Elk4	2.7473E-11	0.60379621	0.225	0.178	6.0274E-07
Mx1	3.2705E-11	1.52597277	0.038	0.018	7.1752E-07
Nisch	3.2991E-11	0.6394416	0.283	0.243	7.2379E-07
Stim1	4.3393E-11	0.74403987	0.19	0.146	9.52E-07
Ppp1r13b	4.4932E-11	1.08353254	0.073	0.044	9.8575E-07
Parp11	5.057E-11	1.26726757	0.053	0.029	1.1095E-06
Map4k2	5.5587E-11	0.82887264	0.143	0.102	1.2195E-06
Cd69	6.8677E-11	0.27549338	0.365	0.305	1.5067E-06
Fam53b	7.2226E-11	1.07670495	0.053	0.028	1.5846E-06
Dip2b	7.4416E-11	0.7380357	0.233	0.192	1.6326E-06
Cyp2d22	7.452E-11	2.10109765	0.013	0.004	1.6349E-06
Trim14	7.6389E-11	1.76644136	0.022	0.009	1.6759E-06
Hdac4	7.8318E-11	1.09587228	0.104	0.069	1.7182E-06
Leprotl1	7.9867E-11	0.46987477	0.355	0.317	1.7522E-06
Prkca	8.4232E-11	0.91652524	0.103	0.068	1.848E-06
B3gntl1	8.5108E-11	2.04606297	0.015	0.004	1.8672E-06
Phf14	8.736E-11	0.8395547	0.178	0.138	1.9166E-06
Gm28935	9.3098E-11	1.42417806	0.03	0.013	2.0425E-06
Hnrnph1	1.0618E-10	0.35306614	0.507	0.491	2.3296E-06
Sorcs2	1.2004E-10	2.22702558	0.011	0.003	2.6335E-06
Thap3	1.2176E-10	1.09078219	0.094	0.061	2.6714E-06
Slc35g1	1.4265E-10	1.49974875	0.025	0.01	3.1297E-06
Pkp4	1.8011E-10	1.35465828	0.068	0.041	3.9514E-06
Inpp1	2.0421E-10	0.81934727	0.114	0.078	4.4801E-06
Cep68	2.0878E-10	1.44141045	0.033	0.015	4.5804E-06
mt-Atp8	2.2061E-10	0.73526265	0.234	0.195	4.84E-06
Add1	2.3569E-10	0.84150659	0.156	0.116	5.1707E-06
Zbtb37	2.5799E-10	1.13521785	0.06	0.035	5.66E-06
Zfp260	2.6237E-10	1.05214434	0.099	0.066	5.7561E-06
BC051226	2.928E-10	2.15595778	0.021	0.008	6.4237E-06

Gm10036	3.1056E-10	0.54555723	0.317	0.282	6.8133E-06
Thumpd2	3.5908E-10	1.68658696	0.022	0.009	7.8778E-06
Lmbr1l	4.2181E-10	0.92575233	0.092	0.06	9.2541E-06
Dnajc9	4.5724E-10	0.73940111	0.154	0.114	1.0031E-05
Gadd45g	4.7513E-10	0.80864406	0.143	0.104	1.0424E-05
Heca	4.9615E-10	0.55169155	0.271	0.228	1.0885E-05
R3hdm1	5.0469E-10	0.64317975	0.277	0.238	1.1072E-05
Phc3	5.6282E-10	0.73356844	0.18	0.139	1.2348E-05
Gm20324	6.6938E-10	2.05643754	0.021	0.008	1.4686E-05
Rpa1	6.7061E-10	1.00177032	0.089	0.059	1.4712E-05
Trub1	7.5295E-10	1.80396707	0.026	0.011	1.6519E-05
Spef2	7.9041E-10	1.93734198	0.014	0.005	1.7341E-05
Stk4	8.0616E-10	0.25908887	0.444	0.396	1.7686E-05
Cmpk2	8.2029E-10	1.06837885	0.024	0.01	1.7996E-05
Spopl	8.767E-10	0.97250978	0.119	0.084	1.9234E-05
Frmd8	9.2646E-10	1.31321363	0.054	0.031	2.0326E-05
Spice1	9.3252E-10	1.53584723	0.033	0.016	2.0459E-05
Gm17509	1.0086E-09	2.17618704	0.012	0.003	2.2127E-05
Rcan3	1.1247E-09	1.79903125	0.034	0.017	2.4674E-05
Poli	1.2482E-09	1.4146866	0.025	0.011	2.7385E-05
Zfp398	1.2802E-09	0.98605976	0.078	0.049	2.8086E-05
Maml2	1.3804E-09	1.17131704	0.061	0.036	3.0284E-05
Tapt1	1.406E-09	0.90537767	0.079	0.05	3.0846E-05
Zfp1	1.4693E-09	1.1422088	0.065	0.039	3.2235E-05
Sigirr	1.6736E-09	1.42145535	0.051	0.029	3.6718E-05
Arhgap9	1.7411E-09	0.97874645	0.132	0.097	3.8199E-05
Tigd2	1.9854E-09	0.83651913	0.142	0.105	4.3558E-05
Mboat1	2.0651E-09	1.93094731	0.02	0.008	4.5307E-05
Gm30054	2.1804E-09	1.12742373	0.052	0.029	4.7836E-05
Hlcs	2.5457E-09	1.2063101	0.054	0.031	5.585E-05
AI480526	2.7868E-09	1.47999992	0.038	0.02	6.1139E-05
Lman2l	3.3385E-09	1.10792228	0.066	0.041	7.3243E-05
Daxx	3.6799E-09	1.1231355	0.083	0.055	8.0732E-05
Prkacb	3.689E-09	0.73164522	0.189	0.152	8.0932E-05
Gbp7	3.8906E-09	0.70819628	0.169	0.132	8.5355E-05
Rab6b	4.0389E-09	1.49061172	0.028	0.013	8.8609E-05
Arhgap29	4.4244E-09	2.50480097	0.01	0.003	9.7067E-05
Asb13	4.6467E-09	1.235974	0.053	0.03	0.00010194
Smad5	4.8607E-09	1.06208065	0.066	0.041	0.00010664
Heatr5a	4.9883E-09	1.48696483	0.06	0.036	0.00010944
Serpini1	5.0385E-09	1.39840117	0.023	0.01	0.00011054
Mdn1	5.1094E-09	0.76028179	0.156	0.12	0.00011209
Qprt	5.7791E-09	1.95892749	0.013	0.004	0.00012679
Zdhhc20	6.5253E-09	0.64591074	0.223	0.186	0.00014316
Tbcel	6.7546E-09	0.86520616	0.095	0.064	0.00014819
Ahdc1	6.8217E-09	1.35208576	0.029	0.014	0.00014966
Cbr1	6.8526E-09	1.36349029	0.05	0.028	0.00015034
9330175E14Rik	6.9906E-09	1.50315439	0.021	0.009	0.00015337

1700019D03R	7.3673E-09	1.62221257	0.015	0.005	0.00016163
Pnpt1	7.5955E-09	1.29440134	0.054	0.032	0.00016664
Fbxl12	8.7173E-09	0.66734684	0.156	0.118	0.00019125
Def6	9.0503E-09	0.93912935	0.081	0.053	0.00019855
Rinl	9.4231E-09	0.52155862	0.21	0.169	0.00020673
Etnk1	1.0077E-08	0.44251489	0.296	0.257	0.00022107
Dnajb4	1.0484E-08	0.87647001	0.126	0.093	0.00023
Tmem245	1.0499E-08	1.00616927	0.098	0.068	0.00023034
Adcy7	1.1042E-08	1.15341484	0.112	0.082	0.00024225
Ifi44	1.3432E-08	2.1361983	0.022	0.009	0.00029468
Osbpl5	1.3683E-08	1.28718112	0.018	0.007	0.00030018
Prpf4b	1.5311E-08	0.44287967	0.42	0.402	0.0003359
Ccdc88c	1.7415E-08	0.53135711	0.193	0.153	0.00038206
Pecam1	2.0223E-08	0.62234224	0.178	0.14	0.00044366
Mrpl30	2.0444E-08	0.6289023	0.304	0.283	0.00044852
Pds5a	2.212E-08	0.67063438	0.239	0.205	0.00048529
Hist2h4	2.246E-08	1.22367488	0.031	0.015	0.00049276
Ggact	2.4139E-08	1.64667075	0.019	0.008	0.00052959
Tmc6	2.8555E-08	1.01914404	0.082	0.055	0.00062647
Mks1	2.9249E-08	1.92941316	0.014	0.005	0.0006417
Tspan32	3.5331E-08	1.14945842	0.064	0.041	0.00077512
Adamts6	3.5991E-08	1.11635887	0.032	0.016	0.00078962
Ppargc1b	3.6192E-08	1.78409716	0.019	0.008	0.00079402
Gm20069	3.7807E-08	1.20963171	0.041	0.023	0.00082944
Rragd	3.9051E-08	1.60925596	0.018	0.007	0.00085675
Txlng	3.9519E-08	1.04683209	0.072	0.047	0.00086701
Rbm33	4.2269E-08	0.64184083	0.238	0.204	0.00092735
Miga1	4.3502E-08	1.4309202	0.031	0.016	0.00095438
Actr6	4.6444E-08	1.46431905	0.038	0.021	0.00101894
Hmgn1	4.738E-08	0.56855144	0.244	0.21	0.00103948
Ccm2	4.8493E-08	0.71876158	0.161	0.126	0.00106388
Slc35b3	5.4538E-08	0.99559367	0.082	0.056	0.00119651
Mlh1	5.6036E-08	1.45631602	0.024	0.011	0.00122937
Cbx3	5.6951E-08	0.56274217	0.31	0.283	0.00124944
Polg	5.9702E-08	0.95701254	0.076	0.051	0.0013098
Atn1	6.1618E-08	0.90475749	0.074	0.049	0.00135184
Limk2	6.3385E-08	1.06595112	0.04	0.022	0.0013906
Lbh	6.447E-08	0.5106214	0.22	0.183	0.0014144
3110009E18R	6.5085E-08	1.83556465	0.017	0.007	0.00142791
Zkscan3	6.7602E-08	0.94849228	0.094	0.066	0.00148311
Abhd8	7.0997E-08	1.04761796	0.053	0.032	0.0015576
Ttll4	7.363E-08	1.31391197	0.034	0.018	0.00161536
Pank4	7.9568E-08	1.29420956	0.042	0.024	0.00174564
Airn	9.5555E-08	1.55883449	0.018	0.007	0.00209639
Pstk	9.7861E-08	1.1894253	0.057	0.036	0.00214697
Commd8	9.8792E-08	0.93180108	0.153	0.123	0.0021674
Focad	1.0545E-07	1.46751091	0.022	0.01	0.00231339
Prps1l3	1.0969E-07	1.28531575	0.038	0.021	0.00240647

Zdhhc15	1.2029E-07	1.89864614	0.013	0.005	0.00263895
Pml	1.2089E-07	0.75549304	0.087	0.059	0.00265211
Huwe1	1.2787E-07	0.45339086	0.355	0.333	0.0028054
Ice2	1.3073E-07	1.36596482	0.029	0.014	0.00286817
Irf9	1.3361E-07	0.69209692	0.175	0.142	0.00293129
E130307A14R	1.3521E-07	1.43611793	0.027	0.013	0.00296638
Trrap	1.3812E-07	0.88394113	0.103	0.074	0.00303023
Hist1h4n	1.3966E-07	1.67180082	0.015	0.006	0.00306407
Ankrd46	1.5328E-07	1.20370015	0.034	0.018	0.00336282
Ogt	1.728E-07	0.34885798	0.349	0.316	0.00379109
Tbc1d30	1.9173E-07	1.39005566	0.035	0.019	0.00420638
Amigo2	1.9206E-07	1.5433063	0.012	0.004	0.00421364
Ankmy2	2.0108E-07	1.18246969	0.039	0.022	0.00441147
Tmem108	2.2088E-07	0.29171541	0.054	0.033	0.00484592
Cdc14b	2.2531E-07	1.44656381	0.029	0.015	0.00494316
Rbm48	2.3036E-07	0.82559381	0.085	0.059	0.00505385
Map3k3	2.3043E-07	0.9896567	0.087	0.061	0.0050554
Mtap	2.405E-07	1.14938443	0.047	0.028	0.0052763
Fubp1	2.5592E-07	0.46069305	0.362	0.344	0.00561467
Pias2	2.5598E-07	0.94535685	0.072	0.048	0.00561596
Gart	2.6143E-07	1.1053982	0.05	0.03	0.00573545
Tspyl3	2.8253E-07	1.37287326	0.017	0.007	0.00619835
Piga	3.0237E-07	1.11280233	0.065	0.043	0.00663363
Tnrc6a	3.0873E-07	0.55456575	0.233	0.202	0.00677317
Icam2	3.1458E-07	1.16394746	0.044	0.026	0.00690164
1110034G24R	3.1859E-07	1.16227279	0.039	0.022	0.00698965
Dock2	3.2994E-07	0.3228272	0.433	0.421	0.00723856
Nucks1	3.4101E-07	0.30618769	0.447	0.434	0.00748139
Jmjd1c	3.4683E-07	0.42710682	0.43	0.429	0.00760906
Acin1	3.5786E-07	0.42258528	0.38	0.365	0.00785117
Sec22c	3.7901E-07	1.39438496	0.02	0.009	0.00831516
Ccdc126	3.7965E-07	1.69880072	0.017	0.007	0.00832914
Adgrl1	3.9296E-07	0.80742088	0.063	0.04	0.00862116
Zfp831	3.9785E-07	0.91409272	0.031	0.016	0.00872841
Mttp	3.9818E-07	1.12912557	0.021	0.01	0.00873559
Sept1	4.0034E-07	0.31786649	0.35	0.308	0.00878299
Alkbh1	4.1304E-07	0.51476083	0.25	0.217	0.00906162
Fam120c	4.798E-07	1.56203816	0.013	0.005	0.01052641
Ap3m2	4.9201E-07	1.10467001	0.04	0.023	0.01079417
Lrrtm2	5.02E-07	2.08975553	0.01	0.003	0.01101338
Matr3	5.08E-07	0.34687885	0.434	0.422	0.01114495
Adar	5.2314E-07	0.83667258	0.096	0.069	0.01147727
Tango6	5.3733E-07	1.39389177	0.022	0.01	0.01178842
Zfp369	5.4709E-07	1.14663446	0.042	0.025	0.01200259
Irgm1	5.478E-07	0.48834585	0.101	0.074	0.01201817
Zyg11b	6.8054E-07	0.65072423	0.146	0.115	0.01493038
Tcp11l2	6.8174E-07	0.48008415	0.252	0.219	0.01495672
Mterf1a	6.892E-07	0.99474337	0.056	0.036	0.01512027

Recql	7.006E-07	1.07182083	0.038	0.022	0.01537035
Epb41	7.0716E-07	0.49190039	0.356	0.339	0.01551438
Klhl9	7.2434E-07	0.94621981	0.118	0.091	0.0158913
Celsr1	7.3476E-07	1.31835848	0.026	0.013	0.01611989
Frat1	7.4795E-07	1.11509465	0.036	0.02	0.0164092
Hsf1	8.1093E-07	0.84369712	0.089	0.064	0.01779104
Zfp141	8.6092E-07	1.25072047	0.026	0.013	0.01888765
Zfp874a	8.8319E-07	1.08231024	0.036	0.02	0.0193763
Nemp2	8.8644E-07	0.90301091	0.062	0.041	0.01944764
Sf3b3	9.0027E-07	0.42576875	0.348	0.327	0.01975096
Hspbap1	9.0427E-07	1.57519306	0.032	0.017	0.01983887
Zer1	9.21E-07	0.94287809	0.067	0.045	0.02020573
Mdc1	9.9323E-07	0.97586009	0.038	0.022	0.02179048
Acrbp	9.9769E-07	1.60586708	0.014	0.005	0.02188821
Msh3	1.0434E-06	1.06524974	0.057	0.037	0.02289138
Gm11131	1.0564E-06	1.34067143	0.021	0.01	0.02317566
Vps13c	1.0721E-06	0.96641829	0.101	0.075	0.02352036
Apol7e	1.0926E-06	1.09395663	0.036	0.02	0.02397071
Tubb2a	1.0934E-06	1.64527401	0.091	0.068	0.0239872
Snrpf	1.1894E-06	0.27636037	0.546	0.561	0.0260945
Kat2b	1.257E-06	1.03123632	0.089	0.065	0.0275776
Prps2	1.2948E-06	0.87870664	0.079	0.055	0.02840601
Prps1	1.3057E-06	0.75550999	0.112	0.084	0.02864539
Pde12	1.3229E-06	1.03528685	0.069	0.047	0.02902256
Rnf114	1.3611E-06	0.6119337	0.219	0.191	0.02986188
Gm44777	1.4382E-06	1.34376388	0.022	0.011	0.03155245
Klf3	1.4587E-06	0.38479037	0.246	0.212	0.03200186
Bst2	1.5087E-06	0.72605142	0.289	0.277	0.0330997
Il16	1.5955E-06	0.61584024	0.135	0.105	0.03500472
Adal	1.598E-06	1.25191534	0.019	0.009	0.03505795
Zscan26	1.6331E-06	0.81638695	0.063	0.042	0.03582855
4833420G17R	1.686E-06	0.59849953	0.194	0.163	0.03698955
Nr2c2	1.7591E-06	0.60861801	0.193	0.163	0.0385936
Aldh6a1	1.7892E-06	1.87032941	0.014	0.006	0.03925399
Pcmtd2	1.7938E-06	0.72949748	0.126	0.098	0.03935332
Gm20696	1.9098E-06	0.37685144	0.083	0.058	0.04189958
Mettl17	1.9765E-06	1.26754153	0.033	0.018	0.0433621
Lyst	2.0439E-06	0.37249059	0.185	0.153	0.04484176
Nsd1	2.1639E-06	0.6184278	0.25	0.226	0.04747371
Mars2	2.2037E-06	1.25734571	0.021	0.01	0.04834753
Trim21	2.2812E-06	1.25434634	0.032	0.018	0.05004734
Dlg3	2.3186E-06	1.39388199	0.013	0.005	0.05086859
Eif4ebp2	2.3957E-06	0.57158211	0.204	0.176	0.05255973
Usp24	2.5368E-06	0.87954767	0.125	0.098	0.05565383
Stk26	2.6023E-06	0.638645	0.125	0.096	0.05709275
Syde2	2.6756E-06	1.35795208	0.01	0.004	0.0587004
Rnf123	2.7848E-06	1.05072632	0.059	0.04	0.06109678
Gm17018	3.0549E-06	1.09246949	0.087	0.064	0.06702093

Agk	3.2018E-06	1.05344645	0.054	0.035	0.07024496
Irgm2	3.2587E-06	0.92176812	0.064	0.043	0.07149214
Sfmbt2	3.6227E-06	1.58643385	0.017	0.007	0.07947945
Zfp507	3.6454E-06	0.71105175	0.07	0.048	0.07997579
Crtam	3.6467E-06	1.22053378	0.02	0.01	0.08000511
Btbd6	3.7934E-06	1.51805585	0.018	0.008	0.0832231
Rph3al	4.2069E-06	1.70267447	0.01	0.004	0.09229626
Mlh3	4.215E-06	1.62103182	0.019	0.009	0.09247292
Klhl3	4.245E-06	2.18346856	0.012	0.005	0.09313106
Rprd2	4.4264E-06	0.68825948	0.145	0.117	0.09711177
Hdhd5	4.4282E-06	1.13898143	0.034	0.019	0.09715108
Usp11	4.8326E-06	1.20741437	0.023	0.012	0.1060229
Arhgef4	5.1271E-06	1.98991822	0.01	0.004	0.11248345
Polr1a	5.1756E-06	0.78555333	0.08	0.057	0.11354814
Fam199x	5.4773E-06	1.01512119	0.044	0.028	0.12016728
Thy1	5.4822E-06	0.26705795	0.286	0.246	0.12027378
H2-T22	5.4833E-06	0.52428914	0.259	0.236	0.12029826
Znrf1	5.8303E-06	0.63051831	0.161	0.133	0.12791079
Gm46218	6.1438E-06	1.68116145	0.011	0.004	0.13478883
Calcrl	6.2438E-06	1.061348	0.045	0.029	0.13698283
Gm12216	7.2947E-06	1.08997717	0.038	0.023	0.16003739
Ndrg2	7.5105E-06	1.79858783	0.011	0.004	0.16477187
Ncapd3	7.6708E-06	0.83881497	0.078	0.056	0.16829011
Mr1	7.7111E-06	1.74234977	0.016	0.007	0.16917437
Gm26698	7.8306E-06	1.86219301	0.012	0.005	0.17179522
Mov10	7.8509E-06	0.96737165	0.035	0.02	0.17224037
Cdk19	7.9326E-06	0.73625996	0.105	0.08	0.1740323
Parn	7.9499E-06	0.94026334	0.047	0.03	0.17441344
Hltf	8.3529E-06	0.69617565	0.12	0.094	0.18325327
Rpap2	8.3794E-06	1.0889454	0.04	0.025	0.18383613
Pds5b	8.5659E-06	0.82991569	0.085	0.062	0.18792757
1600014C10R	8.631E-06	0.92087035	0.054	0.036	0.18935548
Ctse	8.744E-06	1.22903884	0.024	0.013	0.19183527
Zkscan8	8.8065E-06	1.16131403	0.022	0.012	0.19320554
Usp3	8.9504E-06	0.6589154	0.207	0.183	0.19636355
Synj2bp	8.9506E-06	0.83018617	0.124	0.098	0.19636617
Smad7	8.9962E-06	0.26988697	0.345	0.316	0.1973677
Abcc5	9.9033E-06	1.08773536	0.046	0.029	0.21726816
Trim44	1.1531E-05	0.66371594	0.15	0.123	0.25298651
Tmco4	1.1758E-05	1.2851222	0.027	0.015	0.25796176
Uqcc3	1.2649E-05	0.68848758	0.176	0.151	0.27750728
Cacna1a	1.2777E-05	1.84844229	0.014	0.006	0.28030536
Zdhhc17	1.2829E-05	0.8841343	0.063	0.044	0.28146044
Hist1h4c	1.2831E-05	0.87849207	0.071	0.051	0.28148878
Acyp1	1.3016E-05	1.15073169	0.062	0.043	0.28556247
Uri1	1.351E-05	0.79942929	0.107	0.084	0.2963969
Emc9	1.4421E-05	1.58013914	0.027	0.015	0.31638077
Ubr1	1.5217E-05	0.65580657	0.09	0.067	0.33383897

Mylpf	1.5387E-05	1.90797955	0.013	0.006	0.33757874
Lysmd1	1.623E-05	1.16186056	0.027	0.015	0.35607922
Asxl2	1.7317E-05	0.54059568	0.199	0.173	0.37991904
Zfp251	1.7445E-05	1.25672361	0.018	0.009	0.38271906
Zbtb11os1	1.7603E-05	1.40256339	0.026	0.015	0.38619616
Mfsd4a	1.7931E-05	0.84741152	0.068	0.048	0.39339088
Helz	1.9282E-05	0.65056073	0.152	0.126	0.42303672
Lin9	2.0017E-05	1.04775509	0.045	0.029	0.43916286
Zfp53	2.0865E-05	0.75453812	0.055	0.038	0.45775611
Wdr82	2.1025E-05	0.62585899	0.125	0.1	0.46126941
Thumpd1	2.1064E-05	0.68032399	0.11	0.087	0.46213055
Ppp1r3f	2.1155E-05	1.54771323	0.015	0.007	0.46412568
Helz2	2.3112E-05	0.5977733	0.119	0.094	0.50704789
Taf4b	2.3516E-05	0.53283968	0.107	0.082	0.51592296
Ier2	2.4921E-05	0.3729157	0.278	0.256	0.54673794
Tmc8	2.6452E-05	1.43705799	0.022	0.012	0.5803215
Rpap3	2.847E-05	0.8999393	0.06	0.042	0.62460358
Ylpm1	2.8522E-05	0.82379035	0.095	0.073	0.62574596
Slc25a36	2.8755E-05	0.46019153	0.219	0.193	0.63084866
Nudt6	2.951E-05	1.54322906	0.018	0.009	0.64741635
C730034F03R	3.0362E-05	0.76158187	0.035	0.021	0.66610563
Ptcd3	3.2844E-05	0.89456429	0.069	0.049	0.72057204
Cenpj	3.3452E-05	1.20972563	0.021	0.011	0.73389732
Hectd2	3.3698E-05	2.02560113	0.013	0.006	0.73930562
Pou5f2	3.4198E-05	1.37668175	0.02	0.01	0.75027715
Notch1	3.4547E-05	0.64621551	0.14	0.116	0.75791667
Rundc3b	3.5904E-05	0.54810667	0.083	0.061	0.78770655
Cyb5rl	3.6222E-05	1.73840256	0.01	0.004	0.79466649
Irf7	3.7117E-05	0.5114016	0.107	0.083	0.81430858
Zfp512b	3.751E-05	1.40410311	0.02	0.01	0.82293621
Gatad2b	3.811E-05	0.46090317	0.275	0.256	0.83610517
Jade1	3.8621E-05	0.68444922	0.081	0.06	0.84729626
Kcnq1ot1	3.9536E-05	0.4138819	0.162	0.134	0.86739001
C530050E15R	3.9987E-05	1.35911262	0.01	0.004	0.87727805
Dgkz	4.0151E-05	0.82247931	0.102	0.08	0.88086511
C2cd5	4.0624E-05	0.79650576	0.066	0.047	0.89125783
Gm26847	4.194E-05	1.64888448	0.014	0.006	0.92012077
Trmt10b	4.3289E-05	1.40763917	0.019	0.01	0.9497129
Hook1	4.4886E-05	0.72058131	0.044	0.029	0.98474533
5430403G16R	4.982E-05	1.30586312	0.016	0.008	1
Hsdl1	5.0125E-05	1.02314046	0.061	0.044	1
Tarbp1	5.163E-05	1.47275447	0.014	0.006	1
Galnt10	5.4071E-05	1.35732517	0.04	0.026	1
Znrf2	5.4234E-05	0.65039949	0.222	0.203	1
Stat2	5.4668E-05	0.89235355	0.103	0.082	1
Bod1l	5.5057E-05	0.50747004	0.239	0.218	1
Lnpep	5.5211E-05	0.34329148	0.391	0.39	1
2010109A12R	6.1804E-05	1.37578475	0.015	0.007	1

Cdh24	6.3924E-05	1.27574295	0.012	0.005	1
Dnajb1	6.503E-05	0.62459326	0.202	0.18	1
Idnk	6.7569E-05	0.61701392	0.198	0.177	1
Chd6	6.8164E-05	0.54245711	0.186	0.162	1
Disp1	6.855E-05	1.34477903	0.02	0.011	1
Tmem260	7.2231E-05	1.05337537	0.046	0.031	1
Dhx9	7.9012E-05	0.44973538	0.275	0.257	1
Taf1d	7.9798E-05	0.38576475	0.382	0.381	1
Mphosph9	8.0312E-05	0.85064079	0.035	0.022	1
Foxk1	8.0545E-05	0.9622207	0.053	0.037	1
Hp1bp3	8.4283E-05	0.47707688	0.28	0.266	1
Pms1	8.4378E-05	1.09575343	0.02	0.011	1
Slc28a2	8.4769E-05	0.81748835	0.063	0.045	1
Gtf2ird1	8.5791E-05	2.20100752	0.012	0.005	1
Pglyrp2	8.6793E-05	1.22869912	0.021	0.011	1
Tnfrsf22	8.887E-05	1.31959108	0.011	0.005	1
Uba7	9.0516E-05	1.02442693	0.065	0.047	1
Nsun4	9.4135E-05	0.84790982	0.063	0.045	1
2810004N23R	9.5038E-05	0.74087231	0.089	0.069	1
Armc9	9.9268E-05	1.66374459	0.012	0.006	1
Zbtb11	9.9302E-05	0.74484646	0.142	0.12	1
Ralgapa2	0.00010282	0.81225243	0.059	0.042	1
Arhgap4	0.00010397	0.68433408	0.096	0.075	1
Pus7	0.00010469	0.73135635	0.041	0.027	1
Atrn	0.000107	1.03396203	0.048	0.033	1
Gdpd3	0.00011095	1.72665971	0.045	0.031	1
Nat10	0.00011235	0.90363044	0.037	0.024	1
Zfp472	0.0001125	1.02726366	0.04	0.027	1
Rbm38	0.00011548	0.76417766	0.078	0.059	1
Adck2	0.00012347	1.12780783	0.028	0.017	1
Carmil2	0.00012561	0.82980581	0.037	0.024	1
Gm9856	0.00012836	0.88093408	0.041	0.027	1
Dus4l	0.00013414	1.57265224	0.012	0.005	1
Mturn	0.00013769	1.40256878	0.021	0.012	1
Kdsr	0.00013866	0.74965973	0.071	0.052	1
9130221H12R	0.00014118	1.42288088	0.014	0.007	1
Usp36	0.00014972	0.54527705	0.113	0.091	1
Gm46430	0.00015072	1.29915333	0.015	0.007	1
Gm3448	0.00015099	0.89669068	0.053	0.037	1
Ercc5	0.00015894	1.02199792	0.059	0.043	1
Fam149b	0.00016789	1.29681176	0.031	0.02	1
Xrn1	0.00018001	0.60249153	0.145	0.124	1
Dgcr8	0.00020119	0.93181993	0.033	0.021	1
Zfp280c	0.00020226	0.9968505	0.035	0.022	1
B4galnt1	0.00020538	0.47815677	0.228	0.208	1
Zdhhc8	0.00020542	0.98679649	0.036	0.023	1
Nsun5	0.00020595	1.27653114	0.026	0.015	1
Cbx6	0.000221	1.19508262	0.029	0.018	1

Ntrk3	0.00022276	0.83434747	0.035	0.022	1
Sash3	0.00022369	0.78502597	0.081	0.063	1
Gm16124	0.00022806	1.15364458	0.018	0.009	1
Ppp3cb	0.00023525	0.61749113	0.183	0.164	1
Epb41l4aos	0.00024001	0.63166853	0.2	0.182	1
Zfp773	0.00024069	1.00891941	0.02	0.011	1
Gm26561	0.00024935	1.49432117	0.017	0.009	1
Zzz3	0.00024943	0.64294033	0.134	0.114	1
Stx16	0.00025222	0.75009153	0.144	0.124	1
4833403J16Ril	0.00026903	0.86593148	0.023	0.014	1
Thra	0.00028147	1.14679765	0.032	0.02	1
Carf	0.00028705	0.9883964	0.024	0.015	1
Prr12	0.00028952	1.07272948	0.024	0.015	1
Gm15340	0.00028967	0.75440095	0.023	0.014	1
Zbtb4	0.00029067	0.74655053	0.06	0.044	1
Washc3	0.00029403	1.01554991	0.103	0.085	1
Ddx55	0.0002958	0.65585055	0.057	0.042	1
Mlec	0.00029628	0.78948336	0.163	0.145	1
Twnk	0.0002966	1.10100095	0.026	0.016	1
Apobec3	0.00030487	0.46363879	0.187	0.165	1
2510046G10R	0.00030761	1.08229671	0.029	0.018	1
S100pbp	0.00031798	0.81204491	0.07	0.053	1
Sltm	0.0003291	0.38353763	0.321	0.311	1
Sft2d3	0.00033301	0.74392906	0.062	0.046	1
Agfg1	0.00033709	0.50412061	0.205	0.187	1
D2hgdh	0.00033909	1.26439944	0.018	0.01	1
Rcsd1	0.0003398	0.53281634	0.119	0.098	1
Mllt10	0.00034478	0.66234955	0.136	0.116	1
1110002L01Ri	0.00035132	1.4794874	0.011	0.005	1
Zfp595	0.00035205	0.92875464	0.027	0.017	1
Ube3a	0.00038169	0.50619449	0.22	0.203	1
Entpd5	0.00038394	0.90944928	0.032	0.021	1
Gpbp1l1	0.00039045	0.4474155	0.21	0.19	1
Ubxn11	0.0003943	0.76038982	0.043	0.03	1
Sdr39u1	0.0004032	1.2029761	0.023	0.014	1
Hibch	0.00042429	1.28820577	0.025	0.016	1
Tubb2b	0.00042633	1.71941268	0.019	0.011	1
Hist1h4a	0.00043088	1.40239713	0.017	0.009	1
Gm47230	0.00046127	0.85878759	0.024	0.014	1
Dock9	0.00046981	0.85150724	0.028	0.017	1
Gm16973	0.0004801	0.95046116	0.022	0.013	1
Man1a2	0.000484	0.6250536	0.163	0.144	1
Fbxl20	0.00052495	0.71943499	0.081	0.063	1
Trim59	0.00055704	0.47742396	0.129	0.107	1
Zfp512	0.00057372	0.70125879	0.043	0.03	1
Smyd3	0.00057704	0.92277682	0.038	0.026	1
Tfcp2	0.00057834	1.1709685	0.011	0.005	1
Npm3	0.00058128	0.5800101	0.196	0.181	1

Rmi2	0.00058481	1.35765777	0.018	0.011	1
Timm9	0.00062263	0.71787224	0.065	0.049	1
G430095P16R	0.00063517	2.0126811	0.012	0.006	1
Scfd2	0.00063699	1.13039074	0.032	0.021	1
Sun2	0.00065048	0.38889734	0.234	0.215	1
Adat1	0.00065129	1.24799958	0.011	0.005	1
Itgb3bp	0.00065528	1.00339967	0.037	0.025	1
Adamdec1	0.00066727	0.38497203	0.099	0.08	1
Tfap4	0.00066994	1.36725289	0.01	0.005	1
C230037L18R	0.00068043	1.56444365	0.011	0.005	1
Ptch1	0.00070417	0.92735512	0.039	0.027	1
Apex1	0.00071528	0.78613135	0.076	0.06	1
Ung	0.00073295	1.01811116	0.021	0.012	1
Git2	0.00075371	0.43786919	0.265	0.254	1
Dpy19l1	0.00075751	0.29942695	0.048	0.069	1
Mcmbp	0.00077677	0.66679814	0.114	0.096	1
Eif5a	0.00078086	0.28459576	0.495	0.511	1
Rufy2	0.00081662	0.70310302	0.069	0.053	1
Syne1	0.00082434	0.69110904	0.082	0.065	1
Tspyl4	0.00084186	1.10658481	0.021	0.013	1
Mrgbp	0.0008454	0.94387824	0.05	0.036	1
Rad9b	0.00087453	1.06916894	0.011	0.005	1
Ahsa1	0.00089403	0.75632181	0.133	0.116	1
Shprh	0.0010096	0.73906764	0.054	0.04	1
Macrod1	0.00103071	1.30719031	0.016	0.009	1
Ubac2	0.0010404	0.70342497	0.133	0.116	1
Ctso	0.00108124	0.63448572	0.1	0.082	1
Grcc10	0.00110697	0.87266474	0.075	0.06	1
Smarca2	0.00112018	0.5508727	0.161	0.143	1
Zfp74	0.00117339	1.29943299	0.013	0.007	1
Chordc1	0.00117547	0.71020841	0.132	0.115	1
Eefsec	0.00117983	1.0834826	0.039	0.027	1
Ccdc117	0.00122176	0.55114184	0.075	0.059	1
Fdxr	0.0012223	0.99899836	0.037	0.026	1
Mcts1	0.00124343	0.25594382	0.102	0.134	1
Eif3m	0.0012646	0.33142828	0.41	0.431	1
Nsmce1	0.00127343	0.69666226	0.11	0.093	1
Clec2i	0.00127521	0.45593104	0.088	0.07	1
Nufip2	0.00130859	0.44454345	0.236	0.223	1
Dbt	0.00131144	0.91853369	0.049	0.036	1
Irf2bpl	0.00133528	0.46622649	0.112	0.093	1
Akap8	0.0013479	0.66503698	0.122	0.105	1
Rps6ka1	0.00135582	0.27529969	0.055	0.076	1
Ewsr1	0.00137115	0.37768143	0.388	0.405	1
A930024E05R	0.00138682	0.50056514	0.07	0.054	1
Trim65	0.00139398	0.97824872	0.019	0.012	1
Metap1d	0.00141423	0.95007314	0.038	0.027	1
Rbm6	0.00141788	0.56623612	0.187	0.171	1

Usp33	0.00142708	0.97281292	0.057	0.043	1
Slc35e2	0.00146487	0.56742387	0.062	0.048	1
Pms2	0.00153426	0.90970877	0.046	0.033	1
Mast3	0.00155698	0.56219486	0.072	0.056	1
Prkdc	0.00157376	0.78811069	0.047	0.035	1
Ehmt1	0.00158487	0.8428212	0.071	0.056	1
St6gal1	0.00160306	0.56436439	0.081	0.065	1
Zfp85	0.00164813	1.28414807	0.011	0.005	1
Phf12	0.00165588	0.71545642	0.084	0.068	1
Kdm5b	0.00169116	0.498479	0.123	0.104	1
Dhcr7	0.0017163	1.06565905	0.025	0.016	1
Lrrc28	0.00173855	1.12975533	0.027	0.018	1
Arl6ip5	0.00185204	0.52950816	0.225	0.215	1
A430033K04R	0.0020192	1.1013705	0.012	0.007	1
Pard6g	0.00204391	1.3992378	0.013	0.007	1
Hectd4	0.00204465	1.00890782	0.038	0.027	1
Tnfsf10	0.0020586	0.93227867	0.029	0.019	1
E130102H24R	0.00207814	0.8047835	0.029	0.02	1
Arhgap39	0.00209438	1.00128615	0.032	0.022	1
Unkl	0.0022055	0.6422199	0.043	0.031	1
Ngly1	0.00222973	0.7130562	0.069	0.055	1
Phf21a	0.0022323	0.53109986	0.099	0.082	1
Slc25a30	0.00227041	0.8099772	0.08	0.065	1
C920006O11F	0.00229021	1.15290066	0.013	0.007	1
Gabpb2	0.00229775	0.62921492	0.139	0.122	1
Ttc27	0.00231718	1.18270348	0.023	0.015	1
9930111J21Ri	0.00233024	1.22554247	0.01	0.005	1
Ggps1	0.00236507	0.59750403	0.103	0.086	1
Mthfd1l	0.00245598	0.83813505	0.035	0.025	1
Ddx24	0.00246447	0.26365079	0.482	0.509	1
Ncbp3	0.00248591	0.57388807	0.112	0.096	1
Trim30d	0.00248962	0.8186657	0.057	0.044	1
Minpp1	0.00258663	1.0486509	0.036	0.025	1
Ubxn7	0.00266933	0.5644864	0.102	0.086	1
Mtf2	0.00267489	0.45000724	0.156	0.139	1
Pogz	0.00272289	0.75903533	0.059	0.046	1
Dicer1	0.00279303	0.51064052	0.152	0.135	1
9330160F10Ri	0.00283959	0.9503663	0.021	0.014	1
Cep57l1	0.00287078	0.99535543	0.02	0.012	1
Sema4f	0.00309807	1.51769025	0.015	0.009	1
Slc25a26	0.00314369	1.23047919	0.015	0.008	1
Prickle3	0.00314643	0.97489028	0.027	0.018	1
Akap8l	0.00316	0.60542002	0.11	0.094	1
9930104L06Ri	0.00318415	1.08082587	0.014	0.008	1
Aqr	0.00323578	0.57923634	0.163	0.149	1
2610021A01R	0.00334268	0.82721031	0.02	0.013	1
Tcaim	0.00347097	0.84340517	0.026	0.018	1
Csnk1g1	0.00347655	0.66272009	0.097	0.082	1

Zfp7	0.00351998	0.95726845	0.012	0.007	1
Abce1	0.00357143	0.48671752	0.166	0.151	1
Dnmt3b	0.00357647	1.21106634	0.013	0.007	1
Hist1h3a	0.00358056	0.73780344	0.038	0.027	1
Smpd5	0.0036016	1.06262395	0.013	0.007	1
Gtf3c3	0.00363913	0.75317461	0.05	0.038	1
Ncdn	0.00364005	0.84236423	0.036	0.026	1
Urb1	0.00368034	0.96668096	0.026	0.017	1
Bhlhb9	0.00373717	0.95623939	0.021	0.013	1
Celf1	0.00374621	0.39283087	0.283	0.277	1
Srcap	0.0037776	0.57432145	0.113	0.098	1
Rmnd1	0.0038146	1.19828779	0.02	0.013	1
Mapk1ip1	0.00389761	0.81339286	0.03	0.021	1
Cox18	0.00397092	1.03884496	0.041	0.031	1
Rpgrip1	0.00406884	0.7254259	0.058	0.046	1
Endov	0.00409042	1.09783436	0.019	0.012	1
Kdm5d	0.00423125	0.64654729	0.037	0.027	1
Phf11b	0.00431876	0.93214891	0.081	0.067	1
Pkd1	0.00435339	0.68728289	0.061	0.048	1
Adcy3	0.00436323	1.15456473	0.021	0.014	1
Atp8b2	0.00455428	0.84916605	0.046	0.035	1
Gm26514	0.00457166	1.2645919	0.011	0.006	1
Sec63	0.00457419	0.63647618	0.173	0.162	1
Zfp426	0.00465512	0.99661065	0.026	0.018	1
Oxct1	0.00465999	0.25662429	0.079	0.103	1
Ppip5k2	0.00467128	0.68386804	0.068	0.055	1
Crebrf	0.00475702	0.2778855	0.302	0.292	1
Ccar2	0.00480416	0.98932325	0.02	0.013	1
Pus7l	0.00483565	0.93897502	0.014	0.008	1
Gm26670	0.00487974	1.1286133	0.016	0.01	1
C330018D20R	0.00500235	1.49189084	0.011	0.006	1
Zbtb44	0.00501516	0.60213075	0.118	0.103	1
Rere	0.0050996	0.65583897	0.08	0.066	1
Hist1h4h	0.00517347	0.74966763	0.047	0.036	1
Ilf2	0.00527232	0.4161482	0.194	0.179	1
Ccdc171	0.00540336	1.2642678	0.02	0.013	1
Pde4d	0.00547456	0.38877337	0.182	0.166	1
Itfg2	0.00555509	0.70533418	0.04	0.03	1
Mfng	0.00568243	0.89355745	0.042	0.031	1
Rbm5	0.00568531	0.29409277	0.354	0.361	1
Sgsh	0.00569113	0.98071398	0.022	0.015	1
A530017D24R	0.00573634	1.0532814	0.015	0.009	1
Atpaf1	0.00579708	0.94304034	0.021	0.014	1
Zcwpw1	0.00581329	0.93655747	0.019	0.013	1
Ly6k	0.00581368	1.31957775	0.012	0.007	1
Gstcd	0.00587634	0.81826659	0.028	0.019	1
Tmem42	0.00593431	1.10693095	0.038	0.029	1
Xpnpep3	0.00597338	1.01364707	0.024	0.017	1

Timeless	0.00598276	1.35497837	0.021	0.014	1
Dido1	0.00598521	0.72139834	0.075	0.062	1
Gmcl1	0.00601779	0.7179011	0.055	0.043	1
Ppm1f	0.00603166	1.40881083	0.013	0.008	1
Hba-a2	0.00606744	0.27276575	0.028	0.02	1
Zfp638	0.00608943	0.42690369	0.212	0.201	1
Fam117a	0.00621893	0.52850864	0.033	0.024	1
2700049A03R	0.00626966	0.84979838	0.017	0.011	1
Fam91a1	0.00633175	0.26900356	0.066	0.086	1
Hibadh	0.00639083	0.76327648	0.102	0.089	1
Arid2	0.00639192	0.74841145	0.068	0.055	1
Trim39	0.00656016	0.61958628	0.065	0.053	1
Ankrd17	0.00662436	0.41567959	0.243	0.236	1
Cstf2	0.00666917	0.62257219	0.064	0.051	1
Pmm2	0.0067171	0.7355778	0.087	0.073	1
Parp6	0.00673954	0.83184744	0.039	0.029	1
Ints13	0.0067932	0.71449083	0.077	0.064	1
Trmu	0.00682297	1.50985134	0.012	0.007	1
Mettl1	0.00695229	0.88350008	0.064	0.052	1
Slc35f2	0.00696375	1.1485508	0.011	0.006	1
Nle1	0.00729611	0.93167612	0.025	0.017	1
Ccdc15	0.00747085	1.30346452	0.012	0.007	1
Slc19a1	0.0075258	1.23888262	0.013	0.007	1
Pik3c2a	0.00784087	0.50082341	0.161	0.148	1
Retreg3	0.00859718	0.47459671	0.098	0.084	1
Rcn3	0.00868368	1.05432705	0.028	0.02	1
Haus6	0.00901823	0.65055076	0.046	0.036	1
A830010M20F	0.00901985	1.2100829	0.011	0.006	1
Fam196b	0.00902025	1.11324873	0.01	0.005	1
Ipo8	0.00916252	0.6517936	0.074	0.061	1
Bcs1l	0.0093739	1.20966346	0.018	0.012	1
Elmsan1	0.00942249	0.29173311	0.217	0.203	1
Nxf1	0.00945114	0.45935958	0.198	0.188	1
Fkbp3	0.00956079	0.39600472	0.266	0.26	1
Znhit6	0.00974833	0.69852459	0.051	0.04	1
Sirt5	0.00979032	1.34583675	0.011	0.006	1
AU041133	0.00980869	0.72117965	0.023	0.016	1
Tbccd1	0.00984615	0.9770238	0.018	0.012	1
Gnpat	0.01001835	0.74102404	0.048	0.038	1
Col11a2	0.01005977	1.43066556	0.011	0.006	1
Arrdc2	0.01026677	1.05594786	0.018	0.012	1
Wdr76	0.01055146	0.90564538	0.026	0.019	1
Acat1	0.01072978	0.27986174	0.077	0.098	1
Sun1	0.01083514	0.61555146	0.051	0.04	1
Snapc4	0.01095499	1.02263736	0.02	0.014	1
Rbm12b1	0.01098596	0.91460829	0.014	0.009	1
Map2k6	0.01108355	1.36586236	0.011	0.007	1
Gga3	0.01111669	0.79147463	0.038	0.029	1

Elp2	0.01113373	0.88908627	0.043	0.034	1
Slain1	0.01125186	0.34769101	0.083	0.069	1
Mbtps1	0.0113246	0.44838531	0.146	0.133	1
Zfp81	0.01134241	0.83895825	0.026	0.018	1
Eif2ak2	0.01141436	0.79908486	0.053	0.042	1
Arhgef10	0.01148692	1.03976384	0.028	0.02	1
Mga	0.01148825	0.41381232	0.168	0.155	1
Nemp1	0.01160857	0.76405609	0.029	0.021	1
Arhgap35	0.01168923	1.00651606	0.017	0.011	1
Sp3	0.01220041	0.37539314	0.215	0.206	1
Actr10	0.0126216	0.25723584	0.106	0.131	1
Rnf38	0.01273993	0.59981954	0.087	0.075	1
Tmem209	0.01275011	0.74785937	0.049	0.039	1
Ints7	0.01297739	0.62917809	0.069	0.057	1
Map2k7	0.01334355	0.59964543	0.134	0.122	1
Crebzf	0.01343404	0.52474413	0.126	0.113	1
Nop56	0.01347512	0.33612917	0.215	0.205	1
Tnfrsf10b	0.01356867	0.55620517	0.035	0.026	1
Zfp113	0.01358659	0.91995433	0.02	0.014	1
Chd8	0.01367034	0.49504267	0.179	0.169	1
Trim5	0.01380138	0.79314528	0.029	0.021	1
Smyd4	0.01380183	0.93398633	0.017	0.011	1
Zfp101	0.01396448	0.81007764	0.026	0.018	1
Noc3l	0.01402007	0.77832762	0.048	0.038	1
Tmem229b	0.01426359	1.13273431	0.025	0.018	1
Glo1	0.01436515	0.33906945	0.06	0.077	1
Poc1b	0.01469228	0.93478454	0.027	0.02	1
Gigyf1	0.01475264	0.50844213	0.143	0.131	1
Trip11	0.01478594	0.46494423	0.231	0.227	1
Elp3	0.01479861	0.88423243	0.027	0.02	1
Slc16a1	0.01491107	0.64375006	0.063	0.052	1
Ddx49	0.01492436	0.34814442	0.053	0.068	1
Armc3	0.01511558	1.03636887	0.011	0.006	1
Nrg2	0.01545483	1.63322374	0.012	0.007	1
Cxxc1	0.01553412	0.66066687	0.078	0.066	1
AC163032.1	0.01593706	0.65531357	0.031	0.023	1
Zfp952	0.01597045	0.64537363	0.034	0.025	1
Abcg1	0.01597496	0.37618273	0.119	0.105	1
Nol8	0.01612866	0.45378539	0.095	0.082	1
Fam168a	0.01640622	0.31274619	0.063	0.08	1
Adamts10	0.0164389	1.01842883	0.033	0.025	1
Zmym2	0.01669289	0.67988238	0.091	0.08	1
Tmem129	0.01669577	1.08050705	0.014	0.009	1
Btbd19	0.01670458	0.89617992	0.018	0.013	1
Jade3	0.01672024	0.92234926	0.012	0.007	1
Fam172a	0.01675465	0.47907142	0.149	0.138	1
Pcgf6	0.01704007	0.54143852	0.022	0.016	1
Gigyf2	0.01727392	0.59888025	0.122	0.11	1

Afap1	0.01750061	1.41703882	0.025	0.018	1
Pigb	0.01770357	1.10633099	0.014	0.009	1
Ago1	0.01791659	0.71584538	0.051	0.041	1
Zmym1	0.01816254	0.67511632	0.045	0.035	1
Efcab2	0.01837759	1.16068585	0.012	0.007	1
Scyl1	0.01931094	0.40089299	0.03	0.041	1
Papd5	0.02021597	0.6389552	0.092	0.081	1
Cog5	0.02047382	0.67527601	0.071	0.06	1
Nkiras1	0.02062143	0.98536454	0.032	0.024	1
Arhgap30	0.02066562	0.30456531	0.301	0.302	1
Armc8	0.02075126	0.73034413	0.058	0.048	1
Zufsp	0.02076521	0.334497	0.233	0.226	1
Pnpla7	0.02081813	0.67989545	0.07	0.06	1
Dhodh	0.02090879	0.86982957	0.014	0.009	1
Gm5914	0.02092007	0.6512802	0.03	0.023	1
Csrnp2	0.02094622	0.72621688	0.012	0.008	1
Prpf39	0.02115681	0.37854295	0.227	0.222	1
Rab3gap1	0.02183679	0.47190739	0.179	0.17	1
Kctd18	0.02201116	0.67823888	0.058	0.048	1
Nop10	0.02204748	0.33429038	0.317	0.325	1
Pip4k2a	0.02283734	0.52667746	0.208	0.206	1
Akap11	0.02297407	0.67395362	0.082	0.071	1
Usp34	0.02301357	0.4187675	0.27	0.272	1
Ulk3	0.02304201	0.58187142	0.017	0.012	1
Lsm11	0.02320982	1.02005754	0.029	0.022	1
Tubgcp6	0.02323466	0.76552654	0.024	0.017	1
Cbx4	0.0235747	0.44736302	0.132	0.119	1
Wdr6	0.02374468	0.79841471	0.022	0.016	1
Kantr	0.02396966	0.87081078	0.023	0.016	1
Lrif1	0.02397288	0.53068072	0.103	0.091	1
Dlg4	0.02409739	0.80306235	0.034	0.026	1
Mblac2	0.02413568	1.14968454	0.01	0.006	1
Il1rl2	0.02422222	1.29125875	0.014	0.009	1
Smad1	0.02493768	0.8366837	0.049	0.04	1
Tdp2	0.02497523	0.56330597	0.082	0.071	1
Pdha1	0.02528697	0.25515971	0.122	0.147	1
Orai2	0.02530047	0.70681683	0.06	0.05	1
Reps1	0.02550296	0.51415394	0.107	0.095	1
Zdhhc6	0.02553381	0.36404754	0.032	0.042	1
Cnst	0.02558663	0.64570079	0.041	0.033	1
Zbtb14	0.02572567	1.01251252	0.015	0.01	1
Fbln1	0.0257634	0.62508581	0.027	0.02	1
Hikeshi	0.02600771	0.33740694	0.058	0.073	1
Asap1	0.02600899	0.2745138	0.095	0.117	1
Wdr75	0.02629143	0.66503021	0.063	0.053	1
Zfp346	0.02645212	0.82463901	0.017	0.012	1
1700019L13Ri	0.0264748	0.2687659	0.01	0.017	1
Mum1	0.02663508	0.69158743	0.051	0.042	1

Trpm7	0.02682825	0.43499111	0.2	0.195	1
Cbl	0.02711905	0.46600189	0.254	0.258	1
Parp10	0.02769893	0.58146121	0.041	0.033	1
Zfp874b	0.02772279	0.48583569	0.044	0.035	1
Shq1	0.02776841	0.99775828	0.013	0.009	1
Fan1	0.02790342	1.07901172	0.011	0.007	1
Ccsap	0.02801514	0.75343618	0.026	0.02	1
C030006K11R	0.02812374	1.33278501	0.013	0.009	1
Izumo4	0.02836638	0.66200485	0.025	0.019	1
Zfp386	0.02876887	0.93392244	0.047	0.039	1
Fry	0.02895746	0.41090014	0.034	0.026	1
Pigw	0.02915054	1.30363145	0.01	0.006	1
Gtf2ird2	0.02942471	0.93228193	0.015	0.01	1
Dcun1d2	0.02943415	1.02943601	0.015	0.01	1
Stk10	0.02946266	0.37534704	0.21	0.202	1
Diexf	0.02957238	0.70692741	0.036	0.028	1
Paqr7	0.02982008	1.25675737	0.012	0.007	1
Brf1	0.02993242	0.74131389	0.059	0.049	1
Gm17435	0.03003618	1.21044425	0.01	0.006	1
Ptrh1	0.03041774	0.41352752	0.007	0.012	1
Gm34961	0.03044091	1.03533409	0.014	0.009	1
Igip	0.0304996	0.98452086	0.017	0.011	1
Gm2000	0.03101034	0.82880306	0.061	0.052	1
Hypk	0.03113279	0.25513975	0.138	0.166	1
Jade2	0.0313877	0.41283621	0.058	0.048	1
Entpd6	0.0314776	0.47016974	0.051	0.042	1
Rbm4	0.03171192	1.09090983	0.025	0.018	1
Dcaf5	0.0319258	0.65147121	0.055	0.046	1
Gm11696	0.03204193	1.12214236	0.011	0.007	1
Zscan2	0.03215066	0.877528	0.017	0.011	1
Lcmt2	0.0321894	0.99172866	0.02	0.014	1
Smim8	0.03264955	0.73205153	0.063	0.054	1
Rbm10	0.03312191	0.80777289	0.06	0.051	1
Rai1	0.03359341	0.56718995	0.067	0.057	1
Rars2	0.03388659	1.20388911	0.014	0.009	1
Wdr43	0.03428242	0.42268249	0.206	0.202	1
Ube2v2	0.03452067	0.52994133	0.125	0.116	1
Cdc7	0.034688	0.7852042	0.024	0.018	1
Mfhas1	0.03498956	0.29837549	0.046	0.059	1
Mcm2	0.03499456	0.54318249	0.046	0.037	1
2610044O15R	0.03514897	0.99004534	0.018	0.013	1
Tmem87a	0.03539168	0.54582944	0.094	0.084	1
Pcnx3	0.03550041	0.81616729	0.041	0.033	1
Tia1	0.03566412	0.37800702	0.182	0.173	1
Hcfc2	0.03614534	0.51057719	0.039	0.031	1
Gm26518	0.03621807	0.6057013	0.076	0.067	1
Paics	0.03631629	0.31392763	0.099	0.12	1
Lrrc45	0.03645647	0.91159142	0.021	0.015	1

AC165953.2	0.03675756	1.09018278	0.014	0.01	1
Eya3	0.03709421	0.95438731	0.045	0.037	1
Xpo1	0.03784842	0.59064068	0.114	0.104	1
Ccdc58	0.038393	0.38024257	0.025	0.034	1
Fam193b	0.03859837	0.49971274	0.059	0.049	1
Rbm45	0.03906021	0.99447131	0.026	0.02	1
Zfp287	0.03927056	1.14340331	0.015	0.01	1
Hist2h3b	0.04043447	0.97293311	0.01	0.006	1
Oasl2	0.04052188	0.41716257	0.062	0.053	1
Uimc1	0.04053124	0.61245138	0.15	0.143	1
Zhx3	0.04066773	0.97860758	0.016	0.011	1
1500015A07R	0.04099038	0.58243612	0.064	0.055	1
Gm9493	0.04103586	0.64852459	0.088	0.079	1
Herc4	0.04111741	0.33090345	0.154	0.143	1
Foxj3	0.04123554	0.57831435	0.105	0.095	1
AI606181	0.04130812	0.76968389	0.028	0.021	1
Setd6	0.04138641	0.85755147	0.02	0.015	1
Wdr77	0.04206333	0.70025186	0.029	0.022	1
Erf	0.04238435	0.70327291	0.039	0.031	1
Spsb1	0.04270866	1.09731194	0.032	0.025	1
Cdc23	0.04276384	0.64779755	0.035	0.028	1
Clybl	0.04327534	1.27896478	0.017	0.012	1
Spata6	0.04330156	0.79038124	0.068	0.059	1
Taf1a	0.04358329	0.60389069	0.021	0.015	1
Rgmb	0.04417744	1.27534281	0.016	0.011	1
Lonp2	0.04425463	0.25586783	0.057	0.071	1
Kcnn4	0.04464837	0.52537989	0.101	0.091	1
Gtpbp2	0.04470112	0.46217211	0.143	0.135	1
Foxo4	0.04538233	0.71854031	0.026	0.02	1
B130006D01R	0.04552433	0.91736607	0.015	0.01	1
Mlx	0.04555324	0.39718439	0.045	0.056	1
Pex10	0.04579221	1.25525113	0.01	0.006	1
Ercc2	0.04671733	0.76972684	0.028	0.021	1
Pex12	0.04682426	0.76312898	0.015	0.01	1
Ccnl2	0.04691322	0.30297407	0.294	0.3	1
Immp1l	0.04691762	0.29125426	0.063	0.077	1
Mtfmt	0.04730595	0.93062313	0.029	0.023	1
Smim27	0.0474076	0.60992013	0.092	0.083	1
Ptar1	0.04746876	0.78883401	0.022	0.016	1
Lpin1	0.04851793	0.5949333	0.064	0.055	1
Tnxb	0.04895613	0.93118394	0.013	0.009	1
1500011B03R	0.04967089	0.84766209	0.027	0.021	1
Gm11808	0.05065139	0.40470448	0.26	0.266	1
Dffb	0.05108572	0.62379542	0.022	0.017	1
Gm26541	0.0514211	1.08757421	0.015	0.01	1
Rrp9	0.05229732	0.71049087	0.043	0.035	1
Hist2h2be	0.05317202	0.93201579	0.01	0.007	1
Gm16740	0.05358622	0.8840426	0.011	0.007	1

Avl9	0.05372491	0.27771105	0.237	0.232	1
Tnc	0.05397098	0.48578073	0.016	0.011	1
Vmac	0.05401179	1.09670105	0.01	0.006	1
Gm20234	0.05482292	0.71079036	0.014	0.01	1
Bcdin3d	0.05495229	0.53535289	0.021	0.016	1
Phf1	0.05537079	0.43511554	0.08	0.07	1
S1pr4	0.05553101	0.39153026	0.067	0.057	1
2900076A07R	0.05557362	0.50376419	0.018	0.014	1
Hbs1l	0.05568562	0.55188371	0.095	0.087	1
Gba2	0.05629744	0.99623837	0.016	0.012	1
Acvr1b	0.05655391	0.9634254	0.018	0.013	1
Gnpnat1	0.05696917	0.56959702	0.053	0.044	1
Slc25a23	0.05699503	0.98618997	0.016	0.011	1
Adsl	0.05709477	0.72238203	0.047	0.039	1
H2-Q4	0.05719656	0.27665289	0.26	0.258	1
BC003965	0.05788017	0.27187347	0.046	0.057	1
Cntrob	0.05790068	0.68267812	0.022	0.017	1
Cep152	0.05838605	0.88582499	0.018	0.014	1
Ipo9	0.05857641	0.74303286	0.036	0.029	1
Hspg2	0.05861196	0.79596286	0.012	0.008	1
Sssca1	0.05932826	0.27423151	0.035	0.045	1
Mark2	0.05938998	0.40477693	0.217	0.216	1
Grap	0.05965909	0.28270738	0.143	0.132	1
Zfp422	0.05995715	0.82383886	0.02	0.015	1
Zfp12	0.05998276	0.87839752	0.013	0.009	1
Cntrl	0.06014778	0.41540895	0.169	0.163	1
Ankrd26	0.06142743	1.04826926	0.017	0.012	1
Fam35a	0.06170327	0.55607604	0.021	0.016	1
Xrcc6	0.06188053	0.7882757	0.018	0.013	1
Zfp637	0.06198345	0.97162959	0.018	0.013	1
4930480K23R	0.06263307	0.7281708	0.012	0.008	1
Rhot2	0.06330136	0.72395157	0.031	0.024	1
Tnfrsf14	0.06337593	1.0974238	0.019	0.015	1
Hotairm1	0.06347317	0.33711494	0.011	0.016	1
Cmc1	0.06349866	0.4168641	0.138	0.165	1
Nap1l4	0.06376892	0.46204537	0.191	0.188	1
Ubr3	0.06396436	0.25290687	0.075	0.09	1
Dpp4	0.06409812	0.78982278	0.075	0.068	1
Rccd1	0.064847	0.73366543	0.019	0.015	1
Dcp1b	0.06488581	0.61947742	0.018	0.013	1
Gcn1l1	0.06491201	0.50465499	0.101	0.092	1
Dlat	0.06553815	0.31909724	0.033	0.042	1
Neurl4	0.0658285	0.80213705	0.026	0.02	1
Dnajc2	0.06618767	0.26688082	0.155	0.182	1
Nob1	0.06626134	0.60454357	0.07	0.062	1
1700052K11R	0.06637838	0.9630644	0.014	0.01	1
Traf3ip1	0.0665918	1.1910181	0.012	0.008	1
Amer1	0.06690713	1.0833386	0.011	0.007	1

Fbxo5	0.06710578	0.75009301	0.017	0.012	1
Ppm1k	0.06819912	0.6476627	0.052	0.044	1
Lin52	0.06944334	0.64971514	0.026	0.02	1
Tsnax	0.06957382	0.82161647	0.055	0.047	1
Zfp740	0.06987451	0.50223064	0.083	0.074	1
Terf2ip	0.07022023	0.63786148	0.045	0.038	1
Nsun6	0.07033272	0.39915069	0.072	0.063	1
Rsrp1	0.07038645	0.32988784	0.286	0.294	1
Scai	0.07076597	0.52020354	0.05	0.042	1
Zfp990	0.0708646	0.62275677	0.01	0.006	1
Ttc38	0.07178846	1.06248366	0.016	0.011	1
Ubl7	0.07190767	0.25177854	0.054	0.066	1
Ptpn4	0.07339218	0.63969074	0.044	0.037	1
Cd2ap	0.07359065	0.58018666	0.168	0.166	1
Arid3b	0.07374768	0.51414391	0.039	0.032	1
Nme4	0.07401974	1.83995002	0.011	0.007	1
Alkbh8	0.07419654	0.66235925	0.038	0.031	1
Exosc7	0.07475932	0.66156375	0.047	0.04	1
BC005561	0.0750898	0.54897282	0.114	0.107	1
Bcl9	0.07511754	0.75336583	0.024	0.019	1
Klhdc3	0.07520083	0.28535611	0.057	0.069	1
Cdkn2aip	0.07590798	0.50243953	0.091	0.083	1
Slc9a6	0.07611612	0.95222088	0.019	0.015	1
Ly75	0.07624458	0.60851501	0.059	0.052	1
Dnajc24	0.07647304	0.31996474	0.032	0.04	1
Abcb9	0.07674023	0.51145359	0.036	0.029	1
Trmt10c	0.0768848	0.49737916	0.059	0.051	1
Tet3	0.07744334	0.5945342	0.169	0.166	1
Setd2	0.07786566	0.38958733	0.228	0.228	1
Gin1	0.07789699	0.79738954	0.023	0.018	1
Rab18	0.07832059	0.56851931	0.153	0.149	1
Tm9sf1	0.07854084	0.31551802	0.015	0.02	1
Slc25a32	0.0788616	0.37562364	0.02	0.027	1
Sntb1	0.07896089	0.36229546	0.048	0.04	1
Sf3a3	0.07905398	0.27334075	0.083	0.099	1
Slc30a4	0.07930551	1.03466919	0.026	0.021	1
Dmtf1	0.08067819	0.36616961	0.132	0.123	1
Deaf1	0.08104268	0.91579814	0.019	0.014	1
Hgh1	0.08195765	0.9345008	0.012	0.009	1
Stambpl1	0.08219154	0.68477426	0.027	0.022	1
C2cd3	0.08222646	0.46629625	0.058	0.051	1
Nme7	0.08227087	0.76856083	0.018	0.014	1
Pcyt2	0.0825884	0.38549985	0.031	0.039	1
Cyth3	0.08290501	0.25648051	0.111	0.131	1
Tmem186	0.08293828	0.71732448	0.016	0.012	1
Plekha1	0.08327399	0.38966973	0.083	0.099	1
Dcaf6	0.08329858	0.75241705	0.025	0.02	1
Twf2	0.08357578	0.45019461	0.075	0.091	1

Dusp28	0.08378263	1.04575048	0.01	0.007	1
Ammecr1	0.08495916	1.09699824	0.018	0.014	1
Hmbox1	0.08565542	0.67359263	0.062	0.054	1
Fam118b	0.08587739	0.79607585	0.018	0.014	1
Herc2	0.08616434	0.49348948	0.141	0.135	1
Sco1	0.08639716	0.26139467	0.015	0.02	1
Adgrg5	0.08644411	0.72814466	0.058	0.051	1
Slc25a22	0.08662471	0.98167713	0.02	0.015	1
Cep164	0.08751572	0.82383271	0.057	0.05	1
Rgl2	0.08756323	0.78910721	0.044	0.037	1
Gprin3	0.08781539	0.52236445	0.033	0.027	1
Ep400	0.08844287	0.48373218	0.204	0.204	1
Igfbp3	0.08968461	0.46430843	0.02	0.015	1
Dusp7	0.08980785	0.65770512	0.032	0.026	1
Zc3h8	0.0900677	0.80176979	0.014	0.01	1
Ss18l1	0.09038609	0.89453995	0.013	0.009	1
Prdm2	0.09068647	0.4032393	0.144	0.138	1
Ppat	0.09119157	0.60573515	0.052	0.045	1
Rttn	0.09123708	0.71235601	0.014	0.01	1
Prmt7	0.09128781	0.73033304	0.028	0.022	1
Gtf2a2	0.09150667	0.50861811	0.149	0.145	1
Rtel1	0.0920421	0.90697316	0.015	0.011	1
Sirt6	0.09241215	0.84579198	0.015	0.011	1
Abcb7	0.09244369	0.5306449	0.056	0.049	1
Tob1	0.09285629	0.41982388	0.086	0.078	1
Nkrf	0.0932764	0.6778765	0.02	0.016	1
Fto	0.09338306	0.63380678	0.071	0.063	1
Tpra1	0.09386846	0.30510337	0.021	0.028	1
Them6	0.09410236	0.72087843	0.017	0.013	1
Smim26	0.09470794	0.29595255	0.06	0.072	1
Lats1	0.09528111	0.30306114	0.06	0.072	1
Hba-a1	0.09624732	0.31036348	0.03	0.024	1
Tlk1	0.09698278	0.32871837	0.184	0.178	1
Ttll3	0.09701752	0.76377996	0.015	0.011	1
Slc46a3	0.09733681	0.26713522	0.008	0.012	1
C030034I22Ri	0.0985635	0.6332463	0.055	0.048	1
9430015G10R	0.09867328	0.72322182	0.011	0.007	1
Rnf122	0.09875823	0.61169434	0.019	0.015	1
Ube3c	0.09919342	0.43110196	0.085	0.077	1
Mipol1	0.09956068	0.89123299	0.012	0.009	1
Nphs1	0.09987108	1.22394971	0.014	0.01	1
Exd2	0.10031521	0.78399696	0.021	0.016	1
Uchl5	0.10050204	0.28557534	0.074	0.088	1
Smim15	0.10055854	0.26378944	0.125	0.146	1
Tsr2	0.10063037	0.48256968	0.037	0.031	1
Thumpd3	0.10246838	0.54537423	0.065	0.058	1
Insr	0.10257847	0.60039895	0.047	0.04	1
Cep350	0.10285082	0.51735777	0.18	0.18	1

Ep300	0.10332392	0.28464415	0.198	0.195	1
Las1l	0.10345511	0.37390541	0.119	0.111	1
Rhbdd3	0.10345946	0.35118094	0.007	0.011	1
St7l	0.10373887	0.70371566	0.04	0.034	1
D10Jhu81e	0.1037986	0.31819531	0.038	0.047	1
Fam122a	0.10527947	0.55844373	0.066	0.059	1
D930016D06F	0.10535478	0.62725341	0.024	0.019	1
Col1a2	0.1063361	0.42313013	0.046	0.039	1
Dtnb	0.10754017	0.59721361	0.044	0.037	1
Ndc1	0.10881656	0.75046794	0.018	0.013	1
Rrp12	0.10891383	0.69453121	0.033	0.028	1
Zcchc2	0.10990136	0.48479601	0.077	0.07	1
Nars2	0.11067502	0.69718339	0.016	0.012	1
Ncoa2	0.11068414	0.37416439	0.192	0.188	1
Msl1	0.1109666	0.27012498	0.078	0.091	1
Zfp157	0.11102471	0.49799434	0.027	0.022	1
Pbx2	0.1115809	0.29021726	0.033	0.04	1
Smg9	0.1120917	0.67457638	0.07	0.063	1
Ccdc85b	0.1121736	0.70840808	0.012	0.008	1
Phf3	0.11225655	0.29005281	0.339	0.356	1
Fam8a1	0.1123712	0.44215265	0.052	0.045	1
Zfp810	0.11265822	0.59732371	0.015	0.011	1
Tspyl1	0.11315813	0.27615333	0.109	0.126	1
Cd96	0.1133	0.48702222	0.08	0.072	1
Mau2	0.11331361	0.40053452	0.15	0.144	1
Atp11c	0.11487674	0.67916373	0.063	0.056	1
Myo10	0.11522113	0.98124191	0.018	0.014	1
Ddx23	0.11611317	0.47596293	0.157	0.153	1
Nudcd1	0.11618321	0.71393703	0.022	0.017	1
Mbtps2	0.11673912	0.65641582	0.038	0.033	1
Harbi1	0.11675167	0.81697961	0.017	0.013	1
Zfp961	0.11715504	0.61296824	0.015	0.011	1
Apex2	0.11757607	0.26368879	0.011	0.016	1
Abl1	0.1177986	0.6126495	0.055	0.048	1
Rsf1	0.11901804	0.30672391	0.207	0.203	1
Epg5	0.11911213	0.74338142	0.032	0.027	1
Nup107	0.1196728	0.77351202	0.026	0.021	1
Nipal1	0.11984443	1.02696815	0.014	0.01	1
Btla	0.1198698	0.29329735	0.085	0.077	1
Fbxo32	0.12033489	0.4441615	0.056	0.049	1
Kat6b	0.12099311	0.51967912	0.115	0.108	1
Zfp788	0.12139271	0.74060098	0.023	0.018	1
Gm2245	0.12203849	0.67082284	0.019	0.015	1
Suox	0.12316943	0.33816628	0.01	0.014	1
Klhl42	0.12330811	0.88991195	0.01	0.007	1
Mut	0.12356671	0.61117677	0.03	0.025	1
Wdtc1	0.12383894	0.66362231	0.059	0.052	1
Micall1	0.12470218	0.43313403	0.034	0.041	1

Cdkal1	0.12544031	0.63641789	0.05	0.043	1
Mrps26	0.12723279	0.28605122	0.082	0.095	1
Rhbdd2	0.12740468	0.83615394	0.024	0.019	1
Dglucy	0.12748163	1.06738066	0.012	0.009	1
Lrch3	0.12824277	0.29564421	0.077	0.09	1
Zfp60	0.12852735	0.82766194	0.013	0.01	1
Ascc3	0.12873299	0.45220185	0.216	0.221	1
Stx2	0.12877912	1.00563934	0.019	0.015	1
Snx27	0.12903832	0.76090732	0.043	0.037	1
Manba	0.13030153	0.36434907	0.014	0.019	1
Cfap97	0.13054404	0.52299487	0.055	0.048	1
Trim26	0.13172785	0.43063119	0.142	0.136	1
Zfp710	0.13207159	0.52542189	0.019	0.025	1
Tlcd1	0.13226872	0.39342127	0.013	0.017	1
Rasa1	0.13246943	0.49332433	0.101	0.094	1
Casd1	0.13280097	0.64492771	0.038	0.033	1
Gramd1a	0.13296121	0.5204863	0.159	0.156	1
Mtx3	0.13347096	0.85126032	0.014	0.01	1
2900093K20R	0.13432964	0.64495488	0.044	0.038	1
Washc4	0.13432981	0.45605667	0.13	0.125	1
Mtg2	0.13462435	1.01041117	0.022	0.018	1
Wdr90	0.13473558	0.90369416	0.017	0.013	1
Gpsm2	0.13534297	1.31305806	0.013	0.009	1
Dnaja2	0.13566253	0.4188414	0.306	0.327	1
Sh3bp1	0.13566484	0.64228202	0.071	0.084	1
Ctps2	0.13595464	0.72108728	0.05	0.044	1
Irf3	0.13953012	0.53029271	0.087	0.081	1
Mettl14	0.13979417	0.61995441	0.055	0.049	1
Kdm1b	0.14001588	0.80233652	0.022	0.018	1
Drosha	0.14060363	0.81040598	0.025	0.021	1
Zmym6	0.14162539	0.62654431	0.017	0.013	1
Taf8	0.14222735	0.67999995	0.028	0.023	1
Sept6	0.14312252	0.35749949	0.204	0.203	1
Gk5	0.1437986	1.14350638	0.01	0.007	1
Utp14a	0.14390189	0.60763599	0.12	0.116	1
Rnf31	0.14396335	0.51935855	0.054	0.048	1
Dcaf8	0.14421644	0.38536851	0.193	0.192	1
Bbs9	0.1443949	1.09809825	0.018	0.014	1
Orc2	0.14477778	0.66516484	0.055	0.049	1
Sp3os	0.14503101	0.25680715	0.033	0.04	1
Mypopos	0.1454211	0.83785303	0.011	0.008	1
Pradc1	0.14595084	1.1066969	0.017	0.013	1
Lrrc1	0.14607631	1.20956512	0.017	0.013	1
Lcor	0.1461536	0.48346756	0.061	0.055	1
Pole4	0.14668369	0.31003521	0.248	0.253	1
Ice1	0.146748	0.50586102	0.08	0.073	1
Trmt1	0.14722497	0.50883188	0.089	0.083	1
Zfp780b	0.14744375	0.62493444	0.027	0.022	1

Ndst2	0.14745594	0.31706503	0.014	0.019	1
Trmt1l	0.14809524	0.36461971	0.094	0.087	1
Tmem9	0.14851854	0.87255194	0.018	0.015	1
Zc3h7b	0.14968516	0.54194242	0.034	0.028	1
Ypel2	0.15015574	0.89428135	0.021	0.017	1
Slf1	0.15020479	0.52156845	0.037	0.032	1
Rpusd4	0.1516093	0.65037299	0.048	0.042	1
Zfp729b	0.15266986	0.28456669	0.033	0.04	1
Fam173b	0.15279162	0.36305713	0.021	0.027	1
Fbf1	0.15341085	0.8680623	0.011	0.008	1
Hps3	0.15353604	0.45914211	0.094	0.088	1
Ints9	0.15380975	0.82235513	0.039	0.034	1
Rae1	0.15401649	0.29153867	0.062	0.073	1
Ahsa2	0.15425097	0.30846514	0.04	0.047	1
Dvl3	0.15436012	0.26685991	0.034	0.04	1
Cdipt	0.15447401	0.27727651	0.111	0.128	1
Gpatch8	0.15470187	0.40381329	0.229	0.235	1
Fastkd3	0.1567056	0.37133965	0.009	0.012	1
Plscr3	0.15678297	0.66903952	0.014	0.018	1
Srfbp1	0.15684883	0.33605619	0.029	0.035	1
Naa16	0.15787419	0.57332061	0.05	0.044	1
Ypel5	0.15985989	0.32907397	0.125	0.145	1
Nudt3	0.16028059	0.30017643	0.1	0.115	1
Aptx	0.16112792	0.28963604	0.027	0.033	1
Gpr89	0.16151189	0.28163428	0.027	0.033	1
Polr3g	0.16258133	0.59564314	0.014	0.011	1
Abhd11	0.16322282	1.15753721	0.03	0.025	1
Zfp707	0.16373015	0.57824591	0.018	0.015	1
Primpol	0.16388164	0.71450435	0.023	0.019	1
Cul9	0.16496361	1.19810131	0.013	0.01	1
Efemp1	0.16773641	0.77310003	0.015	0.011	1
1110004E09R	0.16781138	0.70578179	0.037	0.032	1
Fnbp4	0.16806622	0.43933305	0.133	0.129	1
Ado	0.16824466	0.31733016	0.026	0.032	1
Zfp58	0.1685512	1.02471499	0.01	0.007	1
Xpo4	0.16900189	0.51888437	0.098	0.092	1
Vezt	0.16930925	0.60805464	0.022	0.018	1
Nt5c3b	0.16933118	0.71702955	0.039	0.034	1
Ttc37	0.16984344	0.54928527	0.045	0.04	1
Mrps18b	0.17027548	0.38955963	0.048	0.056	1
Pot1a	0.1710044	0.57493357	0.047	0.041	1
Klhl12	0.17115261	0.35248809	0.055	0.049	1
Pdss2	0.17150129	0.44298528	0.015	0.011	1
Rdh13	0.17181159	0.74366233	0.011	0.008	1
Haus1	0.17237072	0.28649009	0.008	0.012	1
Llgl1	0.1741538	0.60280742	0.026	0.022	1
D230025D16F	0.17447637	0.30088075	0.027	0.032	1
Tsr1	0.17481024	0.57042362	0.054	0.048	1

Hspb1	0.17600583	0.32202301	0.019	0.016	1
Kmt2b	0.17606981	0.26363817	0.087	0.099	1
Mrpl1	0.17833085	0.29650843	0.036	0.043	1
Slc35c1	0.17899791	0.32621993	0.011	0.015	1
Cep295	0.17918929	0.57127017	0.077	0.071	1
Dph7	0.17938232	0.67743501	0.016	0.012	1
Tmem126b	0.17940823	0.62948585	0.029	0.025	1
Cant1	0.18078095	0.25069531	0.041	0.048	1
Pi4ka	0.18080136	0.29620589	0.071	0.082	1
Ube2t	0.18131816	0.49830502	0.027	0.022	1
Phf13	0.18196695	0.25283346	0.022	0.028	1
Kat2a	0.18217885	0.74471693	0.02	0.017	1
Sfxn3	0.18229667	0.27181035	0.054	0.063	1
Zranb2	0.183548	0.45247259	0.161	0.159	1
Zfp605	0.18366319	1.07949721	0.01	0.007	1
Gemin5	0.18408249	0.74861354	0.019	0.015	1
Ubac1	0.18484003	0.28295652	0.029	0.035	1
Slc2a9	0.18519231	0.76157539	0.013	0.01	1
Osbpl9	0.18529638	0.41262856	0.219	0.225	1
Pinx1	0.18545198	0.824402	0.042	0.037	1
Hars2	0.18590481	0.58578646	0.03	0.025	1
Ears2	0.18653055	1.01702969	0.022	0.019	1
Armcx2	0.18724395	0.84931326	0.013	0.01	1
Tagap	0.18804065	0.3474789	0.114	0.108	1
1190007I07Ril	0.18859765	0.27983643	0.027	0.033	1
Clcc1	0.18868594	0.34628534	0.053	0.061	1
Wdr33	0.18946827	0.55792015	0.064	0.058	1
Zfp35	0.18957996	0.34679189	0.012	0.016	1
Tyw1	0.19012933	0.72642343	0.023	0.019	1
Hist1h2ak	0.19063083	0.65993559	0.016	0.012	1
Spint2	0.19101774	0.35055874	0.061	0.07	1
D3Ertd751e	0.19158507	0.68859401	0.022	0.019	1
Zfp607a	0.19266438	0.77429301	0.019	0.016	1
Usp45	0.19268608	0.70461239	0.053	0.048	1
Tbc1d22b	0.19298633	0.55030137	0.024	0.02	1
Rnaseh2a	0.19341317	0.45933696	0.03	0.036	1
Zfp39	0.19351705	0.49274603	0.013	0.01	1
B3galnt2	0.19433659	0.54395538	0.035	0.03	1
Ftsj3	0.19498454	0.48563739	0.078	0.073	1
Acss1	0.19499644	0.37092913	0.036	0.043	1
Taf1c	0.19570726	0.62963167	0.026	0.022	1
Tceanc2	0.19665174	0.77316204	0.036	0.032	1
Lgals9	0.19711403	0.57041952	0.126	0.123	1
Sh3pxd2a	0.19736171	0.68050308	0.049	0.044	1
Sparcl1	0.19778247	0.70409795	0.01	0.007	1
Thoc5	0.19872048	0.33031965	0.022	0.027	1
Trim33	0.20012322	0.52808259	0.099	0.094	1
Cwc22	0.20137939	0.26171675	0.091	0.103	1

Galnt4	0.20162035	0.85535941	0.01	0.007	1
Lrpprc	0.20189391	0.47229554	0.054	0.049	1
Bcl7a	0.20327476	0.34167681	0.033	0.028	1
Pign	0.2033858	0.67156204	0.038	0.033	1
Idua	0.20416408	0.69223259	0.025	0.021	1
Card11	0.20512221	0.38820812	0.088	0.082	1
Mettl13	0.20525217	0.64176988	0.023	0.02	1
Cd3eap	0.20612	0.58167906	0.027	0.023	1
Trappc8	0.20640737	0.32430453	0.076	0.086	1
Wdr20	0.20643067	0.49718739	0.092	0.088	1
Sprtn	0.20655892	0.39101258	0.033	0.039	1
Ttc33	0.20692895	0.36292135	0.049	0.057	1
Snrk	0.20702465	0.4027222	0.085	0.079	1
Ampd2	0.20717839	0.85954713	0.019	0.016	1
Arhgap27	0.20861037	0.48461108	0.031	0.027	1
Zfp646	0.20915377	0.46572594	0.075	0.07	1
Dgke	0.2092873	0.60256789	0.043	0.038	1
Cdpf1	0.20946308	0.88298174	0.019	0.016	1
Rdh14	0.20956971	0.60889781	0.03	0.026	1
Enpp1	0.21100536	0.4827625	0.035	0.03	1
Mrps9	0.21191555	0.26337264	0.093	0.106	1
Hnrnpr	0.21227352	0.34090591	0.227	0.231	1
Gm13889	0.21455758	0.57389803	0.013	0.01	1
Xrcc4	0.216491	0.75897161	0.036	0.031	1
Hspa4l	0.21703075	0.36598252	0.062	0.071	1
Bphl	0.21723129	0.6552628	0.01	0.014	1
Esf1	0.21798561	0.43529007	0.146	0.143	1
Hmcn2	0.21800595	0.53913434	0.012	0.009	1
Chd3os	0.2188191	0.91680311	0.01	0.008	1
Tab1	0.2204623	1.11099099	0.01	0.007	1
Cnot1	0.22149949	0.35894838	0.239	0.247	1
Dtwd2	0.22197721	0.88012538	0.012	0.01	1
Vars2	0.22327373	0.80433768	0.013	0.01	1
Ap4e1	0.22579682	0.54199362	0.031	0.027	1
Dhx58	0.22725578	0.7972928	0.026	0.022	1
Ndufaf4	0.22825742	0.37853929	0.085	0.079	1
Parp12	0.22882446	0.27944453	0.018	0.023	1
Mtfr1	0.22943434	0.65002168	0.033	0.029	1
Hist1h1a	0.22948851	0.76336285	0.018	0.014	1
Acbd4	0.22968972	0.68508603	0.015	0.012	1
Dguok	0.23005525	0.28522169	0.076	0.086	1
Mfsd8	0.23026073	0.88690873	0.024	0.021	1
Trmt11	0.23126674	0.66722837	0.032	0.028	1
Ppcdc	0.23131849	0.40242497	0.032	0.028	1
Zmat3	0.23152194	0.72156246	0.019	0.016	1
Zfp26	0.23184474	0.58931737	0.053	0.048	1
Tmem230	0.23187102	0.33904164	0.055	0.064	1
Mkln1	0.23265221	0.35880818	0.23	0.239	1

Krt10	0.23333387	0.62718984	0.013	0.01	1
Slc43a2	0.2339141	0.28747347	0.066	0.076	1
Zfp217	0.23403571	0.6764166	0.051	0.046	1
Pou2f1	0.23415136	0.41203744	0.097	0.092	1
Pan2	0.23524463	0.50280366	0.04	0.035	1
Srbd1	0.23574978	0.32539551	0.021	0.025	1
Ubr7	0.23650203	0.3683915	0.054	0.063	1
Nacc2	0.23862293	0.46049051	0.008	0.011	1
Kbtbd3	0.23942635	0.7820933	0.014	0.011	1
Rnf170	0.24040954	0.66846772	0.031	0.027	1
Zfp451	0.24081431	0.34293423	0.104	0.117	1
Wdr11	0.24089516	0.29543282	0.023	0.027	1
Prpf19	0.24098326	0.2753217	0.065	0.073	1
C1galt1	0.24153792	0.47690678	0.086	0.081	1
Ptgr2	0.24239747	0.35904587	0.016	0.02	1
Acsl3	0.24267218	0.7093822	0.042	0.038	1
Casp8ap2	0.24289422	0.38360359	0.151	0.149	1
Rab11fip4	0.24374627	0.77333485	0.025	0.022	1
Dis3	0.24379051	0.60771163	0.032	0.028	1
Mtmr2	0.24426158	0.2666324	0.051	0.058	1
Col3a1	0.24519364	0.28535924	0.093	0.088	1
Zfp180	0.24823671	0.5831722	0.047	0.042	1
Zfp148	0.2482798	0.34686806	0.176	0.176	1
Nomo1	0.25009445	0.42488871	0.028	0.033	1
Hist1h2ac	0.25037974	0.47030137	0.02	0.017	1
Scnm1	0.2513392	0.33083887	0.026	0.031	1
Mybbp1a	0.25154857	0.35595681	0.175	0.177	1
C87436	0.25273246	0.50965707	0.06	0.056	1
Xpc	0.25319479	0.53435491	0.024	0.021	1
Zfp953	0.25327085	0.77305154	0.014	0.011	1
Gcc1	0.25361859	0.40652023	0.046	0.041	1
Pnn	0.25390576	0.34499315	0.244	0.253	1
Ddx31	0.25470574	0.7436524	0.016	0.013	1
Ogn	0.25651048	0.44569011	0.017	0.014	1
Kremen1	0.25655451	0.71811715	0.026	0.023	1
Ada	0.25656307	0.30620473	0.01	0.013	1
Gstp1	0.25665721	0.32920511	0.018	0.022	1
Fastkd2	0.25764696	0.28933761	0.018	0.021	1
Nbr1	0.25805669	0.43991197	0.094	0.09	1
Atxn3	0.26004113	0.33194346	0.039	0.045	1
Capn7	0.2602263	0.44763054	0.087	0.082	1
Cep95	0.26047376	0.67942158	0.012	0.01	1
Slc39a10	0.26058727	0.37019878	0.023	0.027	1
Gm26799	0.26126251	0.59522391	0.014	0.011	1
Galnt12	0.261313	0.61309235	0.032	0.028	1
Cdca7l	0.26176753	0.61066603	0.024	0.02	1
Mrps27	0.26252697	0.49705196	0.014	0.018	1
Abcd3	0.26300952	0.74882259	0.018	0.015	1

Snhg9	0.26384029	0.49194581	0.067	0.062	1
C1qbp	0.26657434	0.42285348	0.166	0.168	1
Irf2bp1	0.266596	0.46810736	0.037	0.033	1
Lrrc8a	0.2666625	0.26942113	0.022	0.026	1
Polr1e	0.2689473	0.72894465	0.014	0.012	1
Mier2	0.269741	0.74247558	0.016	0.013	1
Zfp275	0.26982148	0.42649951	0.026	0.023	1
A130010J15Ril	0.27021797	0.99938642	0.011	0.009	1
Relt	0.27392932	0.25569515	0.019	0.023	1
D10Wsu102e	0.27413457	0.40644958	0.09	0.101	1
Pigo	0.27543145	0.63801465	0.022	0.019	1
Usp5	0.27594635	0.38947635	0.038	0.044	1
Rexo5	0.27641823	0.66068807	0.015	0.012	1
Mrps2	0.27753016	0.39430035	0.014	0.017	1
Kti12	0.27769811	0.55452454	0.036	0.032	1
Ppil2	0.27883585	0.39378162	0.083	0.094	1
Rnf139	0.27886364	0.26239129	0.066	0.074	1
Psmd5	0.27999147	0.70171715	0.038	0.034	1
Col6a3	0.2811485	0.44287996	0.018	0.015	1
Zfp395	0.2812525	0.3046835	0.02	0.017	1
Rpl36a-ps1	0.28135433	0.50789098	0.104	0.101	1
Pknox1	0.28190886	0.5024325	0.073	0.069	1
Keap1	0.28205501	0.73124054	0.053	0.049	1
Dhx34	0.28238147	0.40976984	0.018	0.021	1
Krcc1	0.2831038	0.39264519	0.098	0.111	1
Qtrt2	0.28387729	0.61937083	0.022	0.019	1
Fam3a	0.28418537	0.34310057	0.019	0.023	1
9430060I03Ril	0.28434199	0.41898315	0.016	0.013	1
Ip6k1	0.28504397	0.41225161	0.165	0.166	1
Rabggta	0.28536894	0.36013179	0.027	0.032	1
Atf7	0.28570538	0.42743402	0.077	0.073	1
Pdxp	0.2871939	0.51320571	0.01	0.008	1
Ube4a	0.28742824	0.75642	0.05	0.046	1
Ptcd1	0.28961933	0.3914858	0.014	0.017	1
4921524J17Ril	0.29060631	0.3264546	0.11	0.106	1
Tchp	0.29106197	0.72592318	0.018	0.016	1
Cables2	0.29121548	0.41114849	0.035	0.031	1
Wdr4	0.29208724	0.72680468	0.017	0.014	1
Rabl6	0.29227836	0.46551844	0.058	0.066	1
Gcc2	0.29330652	0.41133353	0.126	0.124	1
Dhx57	0.29476338	0.52604745	0.026	0.023	1
Gtf2h3	0.29499505	0.77548217	0.012	0.009	1
Bms1	0.2952578	0.37710713	0.113	0.109	1
Lsm7	0.29780901	0.50046249	0.101	0.114	1
Dstyk	0.29878825	0.25487605	0.026	0.03	1
Ddx51	0.29887946	0.50954509	0.018	0.016	1
Cdk5rap3	0.2989322	0.48164507	0.05	0.057	1
Slc39a7	0.30015172	0.38348923	0.08	0.09	1

Plekhg2	0.30137401	0.779031	0.036	0.033	1
B230354K17R	0.30194413	0.50705386	0.011	0.014	1
Mfap4	0.30296785	0.3220469	0.01	0.008	1
Gm38394	0.30425726	0.31381337	0.011	0.013	1
Ppcs	0.304411	0.61192567	0.017	0.014	1
Men1	0.30443875	0.57649931	0.016	0.013	1
Tnpo3	0.30515496	0.31175941	0.151	0.169	1
Dtwd1	0.30624183	0.75293515	0.014	0.012	1
Fam126b	0.30655266	0.50165094	0.042	0.038	1
Usp40	0.30707734	0.78756201	0.029	0.025	1
Cog8	0.30819562	0.26810184	0.037	0.043	1
Dpy19l4	0.30833668	0.35023329	0.026	0.031	1
Aldh4a1	0.30895116	1.04900406	0.015	0.013	1
BC002059	0.31082147	0.60753725	0.018	0.015	1
Pdpr	0.31102259	0.59595801	0.039	0.035	1
Rcor3	0.31161116	0.42588951	0.017	0.02	1
Brd3	0.31183615	0.33108063	0.078	0.087	1
Gm26533	0.31412034	0.65384873	0.012	0.01	1
2610008E11R	0.31457117	0.48048207	0.032	0.029	1
Gfpt2	0.31491404	0.64684924	0.013	0.011	1
Fktn	0.31567207	0.51161209	0.011	0.009	1
Tnfaip8l2	0.31720754	0.38604806	0.063	0.071	1
Cic	0.31756291	0.28963097	0.09	0.101	1
Polg2	0.31799269	0.2657166	0.077	0.073	1
Endog	0.32182826	0.73536772	0.03	0.026	1
Fam60a	0.32209471	0.28769874	0.062	0.07	1
Hspb11	0.32294894	0.50862877	0.071	0.08	1
Dnajc27	0.32458625	0.47384971	0.023	0.02	1
Nifk	0.32494008	0.26802981	0.073	0.081	1
Ip6k2	0.32509327	0.47292314	0.043	0.04	1
Ptgfrn	0.32674557	0.42301606	0.008	0.01	1
Tmem63a	0.32806351	0.62708914	0.036	0.032	1
Ttll12	0.3289333	0.98291997	0.018	0.015	1
Ube4b	0.33006039	0.30304468	0.063	0.07	1
Igf2r	0.33088765	0.25001232	0.114	0.11	1
Numb	0.33173182	0.68605894	0.037	0.034	1
Dmac1	0.33179314	0.42547996	0.101	0.114	1
Wdr83	0.33366985	0.35502612	0.02	0.023	1
Hira	0.33478719	0.63629111	0.035	0.031	1
Ap1g2	0.33510174	0.30281268	0.032	0.036	1
Strip1	0.3353598	0.2562474	0.056	0.062	1
Rbm34	0.33644109	0.27108753	0.101	0.111	1
Gm26890	0.33663823	0.45216716	0.012	0.01	1
Plcb2	0.33712251	0.60884001	0.048	0.054	1
Osbpl7	0.33725592	0.51112856	0.026	0.023	1
Atg2b	0.33881439	0.47184774	0.033	0.029	1
Bfar	0.33983173	0.29069904	0.106	0.117	1
Spout1	0.33998763	0.71754977	0.022	0.019	1

Nfyc	0.34008588	0.26052796	0.064	0.071	1
Dock8	0.34107957	0.31868397	0.155	0.173	1
Ppil4	0.3417111	0.39830927	0.157	0.158	1
Prkaa1	0.34197486	0.40557791	0.033	0.037	1
1110008L16Ri	0.34226967	0.99982767	0.012	0.01	1
Thoc2	0.34263707	0.41533788	0.223	0.233	1
Rpl9-ps6	0.34280555	0.34201626	0.053	0.059	1
Mboat7	0.34290938	0.33737849	0.054	0.06	1
Mms19	0.34322151	0.50382113	0.066	0.063	1
Gm9844	0.34365003	0.42422952	0.026	0.03	1
2510002D24R	0.34366713	0.3660144	0.083	0.079	1
E4f1	0.34440223	0.40533461	0.083	0.079	1
Heatr3	0.34460228	0.54365309	0.038	0.035	1
Pdrg1	0.34579633	0.39988514	0.06	0.067	1
Arhgef7	0.34646838	0.26207279	0.037	0.042	1
Xylt2	0.34658196	0.87865371	0.019	0.016	1
Pithd1	0.34675245	0.28095967	0.036	0.041	1
Trp53	0.34753754	0.38409783	0.164	0.166	1
Yod1	0.34784154	0.32222253	0.038	0.043	1
Trmt61a	0.34849178	0.569445	0.011	0.009	1
Iqce	0.34963304	0.69767837	0.015	0.012	1
Ldhb	0.34973796	0.97050201	0.017	0.015	1
Pcolce	0.35110789	0.7263503	0.011	0.009	1
Hmgxb3	0.35176424	0.25538586	0.028	0.032	1
Nudt1	0.35191626	0.86967353	0.015	0.012	1
Elf2	0.3520889	0.38373024	0.204	0.211	1
Polr3b	0.35257234	0.39829019	0.031	0.028	1
Lta4h	0.35295555	0.4144072	0.071	0.079	1
Mrs2	0.35366789	0.33435294	0.036	0.041	1
Ankrd27	0.35430126	0.67879677	0.023	0.021	1
Clip1	0.35490718	0.27555267	0.133	0.148	1
Ipo11	0.35506429	0.72878771	0.024	0.022	1
Zfp950	0.35578455	0.39793584	0.046	0.043	1
Ctr9	0.35647853	0.26371683	0.082	0.09	1
Enc1	0.35650515	0.62323409	0.021	0.019	1
Tarsl2	0.35725719	0.66383528	0.011	0.009	1
Gm42670	0.35796374	0.64037488	0.02	0.017	1
Bicra	0.35861181	0.30646297	0.036	0.032	1
Angel2	0.3609085	0.34428043	0.153	0.154	1
Mindy2	0.36195768	0.47366639	0.106	0.103	1
Zbtb45	0.36339917	0.37564343	0.01	0.012	1
Fam84b	0.36416824	0.54008523	0.016	0.013	1
Wrn	0.36470936	0.48145008	0.066	0.063	1
Oma1	0.3651879	0.45521443	0.013	0.015	1
Qrich1	0.36555108	0.29972429	0.113	0.125	1
Wdr44	0.36619691	0.3569149	0.065	0.062	1
Smim4	0.36644197	0.65400704	0.089	0.087	1
Gm7160	0.36657016	0.59686119	0.011	0.009	1

Tk2	0.36662227	0.26986174	0.048	0.044	1
Nup205	0.36666675	0.63033649	0.044	0.041	1
Ddx18	0.36666927	0.36180846	0.184	0.188	1
Sars2	0.36671987	0.46370364	0.011	0.014	1
Trmt2a	0.36762845	0.46882274	0.041	0.038	1
Ankib1	0.36794302	0.51335951	0.099	0.097	1
Smurf1	0.36900987	0.70274114	0.043	0.04	1
Fez2	0.37013556	0.48545067	0.024	0.028	1
Mios	0.37073511	0.43102155	0.023	0.021	1
Fance	0.37111817	0.86114831	0.014	0.012	1
Mppe1	0.37134931	0.42941918	0.015	0.013	1
Hace1	0.37152862	0.44662288	0.038	0.035	1
Ap5m1	0.37227407	0.51397283	0.048	0.045	1
Ago3	0.37270683	0.45924982	0.064	0.072	1
E130208F15Ri	0.3745173	0.49992595	0.016	0.013	1
Cwf19l2	0.3750141	0.36724442	0.121	0.119	1
Zfp956	0.3757464	0.47956043	0.014	0.012	1
Cramp1l	0.37621351	0.35222491	0.049	0.055	1
Gm20045	0.37746871	0.2777828	0.012	0.014	1
Gm8186	0.37852895	0.28584424	0.07	0.077	1
Klhl36	0.3786488	0.63016614	0.014	0.012	1
Btbd2	0.37916246	0.27268394	0.01	0.012	1
Senp1	0.38009681	0.35174843	0.071	0.078	1
Foxj2	0.38072842	0.31261372	0.058	0.065	1
Erc1	0.38087206	0.58563846	0.028	0.025	1
Zfp738	0.3823561	0.37399318	0.029	0.033	1
Ddx19a	0.38236754	0.32489493	0.06	0.066	1
Kiz	0.38298864	0.41328632	0.017	0.019	1
Slc35a5	0.38310764	0.30886588	0.019	0.022	1
Dars	0.38323786	0.63994216	0.088	0.086	1
Aven	0.38368524	0.75781035	0.049	0.046	1
Slc7a6	0.38427295	0.27557336	0.017	0.02	1
Ccdc191	0.38617491	0.66369184	0.012	0.01	1
Gpatch2	0.38677076	0.53729492	0.052	0.049	1
Mphosph10	0.38682394	0.27755501	0.101	0.111	1
Armc10	0.38707603	0.34796824	0.05	0.055	1
Rpp21	0.38860491	0.30363313	0.138	0.154	1
Wdr24	0.38888813	0.72271307	0.014	0.012	1
Sec16a	0.38963873	0.33203917	0.071	0.078	1
Rnf103	0.38973499	0.33218281	0.052	0.057	1
Brcc3	0.39009116	0.6225313	0.069	0.067	1
Hist2h2bb	0.39022948	0.53547468	0.027	0.024	1
Aar2	0.39030762	0.25499055	0.043	0.048	1
Snhg20	0.39261759	0.37145228	0.052	0.048	1
Rgp1	0.39455258	0.44048925	0.022	0.019	1
Clpb	0.39491575	0.41159907	0.018	0.021	1
Ring1	0.39578983	0.25478198	0.041	0.046	1
Atad2b	0.39602406	0.29280935	0.109	0.12	1

Tnpo2	0.39638845	0.64427352	0.055	0.052	1
Ppwd1	0.39671898	0.30220905	0.161	0.162	1
Unc13d	0.39675652	0.61128939	0.043	0.04	1
Mapk9	0.39700801	0.58984927	0.054	0.052	1
Pam	0.39778793	0.54131719	0.019	0.017	1
A330069E16R	0.39791068	0.30716794	0.011	0.009	1
Fastkd1	0.39899683	0.80040391	0.019	0.017	1
Cops3	0.40091601	0.37399607	0.059	0.066	1
Gcfc2	0.40134777	0.54912386	0.014	0.012	1
Acat2	0.40317402	0.47684984	0.021	0.019	1
Hdac8	0.40416163	0.40027385	0.022	0.02	1
Synrg	0.40438436	0.42426546	0.106	0.104	1
Top3a	0.40579264	0.54047913	0.019	0.017	1
Fam210a	0.40612111	0.54010088	0.034	0.031	1
Parp4	0.40649207	0.25068827	0.088	0.096	1
Zmynd8	0.40666366	0.32875159	0.097	0.106	1
Cspp1	0.40693992	0.39947896	0.106	0.104	1
Ubr4	0.40695663	0.30963049	0.142	0.158	1
Zfp266	0.40807019	0.42454648	0.093	0.09	1
Echdc1	0.40933104	0.53806822	0.015	0.017	1
Slc36a1	0.40977061	0.74743021	0.013	0.011	1
Aaed1	0.40993135	0.39587611	0.05	0.055	1
Lpcat3	0.41020087	0.4311823	0.028	0.032	1
Gtpbp1	0.41063661	0.36522344	0.068	0.065	1
Zfp825	0.41073441	0.78469733	0.013	0.011	1
Ccdc69	0.41202364	0.58993839	0.018	0.016	1
Rangrf	0.41219503	0.82695507	0.011	0.009	1
Faap20	0.41455289	0.25568019	0.042	0.047	1
Supt7l	0.41625675	0.30249672	0.026	0.029	1
B230307C23R	0.41641522	0.59756793	0.053	0.05	1
Cbx5	0.41650776	0.49815769	0.063	0.06	1
Cox15	0.41710971	0.40117314	0.016	0.018	1
Bcl2l12	0.41760474	0.41716294	0.016	0.018	1
Zfp871	0.41814442	0.4322384	0.17	0.175	1
Zfp579	0.41875029	0.93594973	0.012	0.01	1
Banp	0.41883837	0.58373758	0.03	0.027	1
Srsf10	0.41955109	0.27387604	0.215	0.222	1
Otud5	0.42333419	0.26448633	0.113	0.125	1
Tdp1	0.42361913	0.64238534	0.015	0.013	1
Pprc1	0.42440584	0.35006899	0.027	0.031	1
Wdr12	0.42446342	0.54532294	0.039	0.036	1
Golm1	0.42517568	0.4103392	0.037	0.035	1
Zfand1	0.42519951	0.48354972	0.018	0.02	1
Cc2d1b	0.42578247	0.45264333	0.063	0.06	1
Pofut1	0.42602183	0.64387075	0.034	0.031	1
Sec11a	0.42615687	0.36318184	0.239	0.257	1
Nipsnap1	0.42713458	0.69724736	0.02	0.018	1
Nono	0.42769706	0.34985254	0.265	0.283	1

Fem1a	0.43055587	0.31739164	0.032	0.036	1
Slc12a9	0.43068697	0.73298494	0.019	0.022	1
Zfp688	0.43105894	0.66338542	0.029	0.027	1
Eipr1	0.43229878	0.61471689	0.074	0.072	1
Ttc5	0.43241729	0.41821833	0.07	0.067	1
Ighmbp2	0.43430734	0.50486811	0.02	0.018	1
1600020E01R	0.43454096	0.5673124	0.06	0.058	1
Nprl3	0.43454647	0.60667369	0.02	0.018	1
Prkag1	0.4355093	0.32816189	0.05	0.055	1
Pip4k2b	0.4356049	0.3445251	0.027	0.03	1
Usp22	0.43614538	0.49551912	0.067	0.065	1
Ppp1r12b	0.43754968	0.60759645	0.018	0.015	1
Gemin6	0.43824812	0.78810651	0.011	0.009	1
Mettl3	0.43846947	0.42075673	0.039	0.037	1
Nup214	0.43918582	0.41230444	0.085	0.094	1
Scyl3	0.43934245	0.29449503	0.057	0.063	1
Slc25a12	0.44000228	0.40004496	0.03	0.034	1
Wdcp	0.44061396	0.27844474	0.014	0.016	1
Pptc7	0.44077228	0.45131936	0.099	0.097	1
Ofd1	0.44123779	0.32712307	0.028	0.031	1
Gvin1	0.44149655	0.30763733	0.012	0.01	1
4930481A15R	0.44408961	0.61658643	0.014	0.012	1
Mcm9	0.44448568	0.41215785	0.042	0.039	1
Letmd1	0.44589854	0.64457153	0.031	0.028	1
Xxylt1	0.4470209	0.27521484	0.018	0.021	1
Gsk3b	0.44716855	0.38107181	0.237	0.251	1
Gm26853	0.44860706	0.29741718	0.062	0.068	1
Gm42372	0.44887926	0.4589799	0.011	0.009	1
Tmem87b	0.44949792	0.39755597	0.065	0.062	1
Kdm4c	0.44977639	0.25554859	0.044	0.048	1
Phpt1	0.45107955	0.4649792	0.031	0.035	1
C330011M18R	0.45205635	0.60297088	0.011	0.01	1
Xrcc5	0.452082	0.47549158	0.018	0.016	1
Nop2	0.45227831	0.64496967	0.023	0.021	1
Cd84	0.45286594	0.33930509	0.199	0.206	1
Lpcat4	0.45373767	0.35300624	0.019	0.022	1
Cdkn2aipnl	0.45418924	0.38221893	0.054	0.051	1
Gm47015	0.4544966	0.25668405	0.045	0.042	1
Nom1	0.45741191	0.26198585	0.096	0.093	1
Foxo3	0.45814492	0.45828602	0.06	0.058	1
Gpn1	0.46018581	0.34814419	0.035	0.039	1
Ppp4r3a	0.46029608	0.26418767	0.2	0.223	1
Wdr5	0.46067582	0.3240695	0.047	0.051	1
Arv1	0.46074304	0.72131864	0.01	0.008	1
Mnat1	0.46080266	0.52079527	0.069	0.076	1
Helq	0.4608912	0.55349073	0.011	0.01	1
Pi4kb	0.46202228	0.36348363	0.021	0.024	1
Ankrd16	0.4623193	0.28079011	0.026	0.029	1

Atxn7l1	0.4642065	0.330208	0.087	0.096	1
Gm45902	0.46423734	0.3081513	0.02	0.023	1
Pgs1	0.46804368	0.41253538	0.094	0.104	1
Zfp609	0.46813607	0.41582991	0.02	0.023	1
Dhx30	0.46956752	0.26211513	0.089	0.097	1
Yae1d1	0.46977279	0.3683132	0.028	0.031	1
Ube2d1	0.47019508	0.4731835	0.088	0.086	1
Taf15	0.47056012	0.29775816	0.185	0.19	1
0610040B10R	0.47098236	0.29398158	0.043	0.04	1
Ppp1r8	0.47157309	0.30688021	0.041	0.045	1
Trpc4ap	0.47159715	0.4180322	0.189	0.198	1
Nol11	0.47207101	0.25974739	0.091	0.099	1
Ncbp1	0.47293552	0.42183596	0.08	0.078	1
Wdr45	0.47367957	0.35566782	0.044	0.041	1
Ggt5	0.47478605	0.77926468	0.026	0.024	1
B3galt6	0.47507597	0.45446895	0.014	0.012	1
Magi3	0.47559737	0.60208115	0.021	0.019	1
Flt3l	0.47623121	0.47113689	0.046	0.043	1
Scaf4	0.48089523	0.5622068	0.041	0.039	1
Rreb1	0.48113692	0.43383029	0.043	0.048	1
Wdr92	0.48130711	0.57272801	0.015	0.013	1
Taok2	0.48139539	0.48289023	0.063	0.061	1
Cog6	0.4820504	0.31340834	0.03	0.033	1
Shmt2	0.4830116	0.253287	0.038	0.042	1
2310009B15R	0.48355322	0.64460623	0.056	0.062	1
Dcaf4	0.48480089	0.37727367	0.013	0.015	1
Dnm2	0.48564393	0.37129584	0.18	0.188	1
Col5a1	0.48747251	0.39554974	0.013	0.011	1
Gstz1	0.48801337	0.3212069	0.008	0.01	1
Fbxo8	0.48981011	0.41108841	0.061	0.059	1
Pfas	0.4907784	0.27191902	0.014	0.012	1
Zzef1	0.49155247	0.39784056	0.118	0.118	1
Armc7	0.49184465	0.28724921	0.035	0.033	1
Sfxn1	0.49222427	0.43322946	0.056	0.061	1
Nek9	0.49307772	0.34627622	0.061	0.067	1
Scaf8	0.49347245	0.40436586	0.128	0.129	1
Mettl21a	0.49545073	0.98657552	0.01	0.008	1
Ankrd39	0.49651839	0.61699958	0.027	0.025	1
Dap3	0.4984881	0.43656118	0.085	0.093	1
Dclre1a	0.5017346	0.40716259	0.009	0.011	1
Zfp692	0.50186306	0.28696014	0.012	0.014	1
Ppif	0.50274357	0.59670753	0.033	0.03	1
2410089E03R	0.50316742	0.51621799	0.057	0.055	1
Nt5m	0.50423167	0.67391115	0.012	0.014	1
Phb	0.50430954	0.54943686	0.028	0.026	1
Apc	0.50468785	0.32386974	0.101	0.11	1
Tbc1d31	0.50761998	0.5381925	0.018	0.02	1
Rad1	0.5088418	0.52740561	0.018	0.016	1

Abhd18	0.50890728	0.61971332	0.033	0.031	1
Pan3	0.50909193	0.28873625	0.247	0.26	1
Kat6a	0.50991842	0.32001557	0.203	0.211	1
Szt2	0.51064309	0.37705317	0.015	0.013	1
Gatd1	0.51086957	0.40999962	0.049	0.053	1
Ipp	0.51141824	0.59449475	0.012	0.011	1
Saysd1	0.51145428	0.47870743	0.03	0.033	1
Telo2	0.51173958	0.71283227	0.015	0.013	1
Chd1l	0.51263122	0.48205756	0.029	0.027	1
Fbxw4	0.5132904	0.44957468	0.024	0.027	1
Gmip	0.51333192	0.32936256	0.086	0.094	1
Lss	0.51375775	0.57871331	0.01	0.008	1
Rbmx	0.51424246	0.33973701	0.061	0.066	1
Ccdc82	0.51533813	0.38239182	0.096	0.095	1
Nsun2	0.51747747	0.34684669	0.081	0.088	1
Gpn3	0.51779984	0.32568995	0.078	0.076	1
Kdm4b	0.51801308	0.6421529	0.027	0.025	1
Accs	0.51895481	0.46097907	0.016	0.014	1
Opa1	0.52039354	0.27615654	0.046	0.05	1
Prr14l	0.52099944	0.51661972	0.049	0.047	1
Tmsb15b1	0.52196878	0.5396926	0.019	0.021	1
Alkbh2	0.52288872	0.51268703	0.01	0.012	1
Nol9	0.52479333	0.47784497	0.041	0.039	1
H6pd	0.52579521	0.83575507	0.01	0.009	1
Hist2h2aa1	0.52587703	0.42234595	0.038	0.036	1
Ddx39b	0.52622335	0.4229308	0.198	0.21	1
Atxn7	0.52631756	0.25023022	0.123	0.134	1
Btf3l4	0.52707745	0.30389881	0.022	0.025	1
Wdhd1	0.52868582	0.55035064	0.029	0.027	1
Ccdc94	0.52911088	0.39020107	0.017	0.019	1
Ccpg1	0.5291564	0.54897264	0.06	0.058	1
Max	0.52953	0.36430555	0.143	0.145	1
Cenpt	0.53233289	0.43987509	0.009	0.01	1
Mettl9	0.53305247	0.55789699	0.072	0.071	1
Stag2	0.53370082	0.29898179	0.235	0.249	1
Hddc2	0.53430962	0.58420167	0.042	0.04	1
Cnot6	0.53487621	0.32028702	0.235	0.247	1
Rbak	0.53571278	0.3813739	0.012	0.011	1
Casp2	0.53625091	0.31320002	0.046	0.044	1
Fam222b	0.53825262	0.294539	0.02	0.023	1
Cdc16	0.5386424	0.27694632	0.033	0.036	1
Zfp408	0.53884932	0.80230162	0.017	0.015	1
Rnmt	0.54034003	0.33839088	0.107	0.117	1
Atad3a	0.54178425	0.27034777	0.03	0.032	1
Mcm7	0.54201633	0.2912463	0.045	0.049	1
AL590144.2	0.54259726	0.50563752	0.055	0.054	1
Lsm14b	0.54316617	0.36638185	0.03	0.033	1
Lyrm4	0.54323997	0.36353868	0.018	0.021	1

Mavs	0.54356114	0.38907286	0.01	0.012	1
Dvl1	0.54360163	0.44260373	0.039	0.037	1
Fam193a	0.54444418	0.34537868	0.106	0.115	1
2700016F22Ri	0.54459709	0.26589169	0.01	0.012	1
Trim23	0.54701108	0.5231624	0.028	0.026	1
Dennd6a	0.54729021	0.28125431	0.061	0.066	1
Dzip3	0.54841371	0.54167845	0.015	0.013	1
Pigl	0.54972571	0.7899546	0.01	0.009	1
Sugp1	0.54981573	0.27017628	0.055	0.06	1
Slc20a2	0.54985734	0.25773635	0.027	0.03	1
BC037034	0.55002639	0.27462129	0.025	0.027	1
Smc1a	0.55070251	0.29719932	0.23	0.243	1
Surf2	0.55134493	0.55204813	0.034	0.032	1
Mapk8	0.55227265	0.40013462	0.081	0.08	1
Mcm3ap	0.55243445	0.42358817	0.037	0.041	1
Nfatc2ip	0.55337197	0.45460417	0.046	0.044	1
Bcap29	0.55441766	0.48290924	0.067	0.072	1
Zcchc4	0.55464564	0.46197103	0.029	0.027	1
Etfdh	0.55526161	0.37043259	0.054	0.052	1
Tbl1x	0.55559379	0.25535652	0.17	0.186	1
Nvl	0.55567169	0.32127476	0.063	0.068	1
Alkbh4	0.55696824	0.5442158	0.019	0.017	1
Gon4l	0.55900935	0.45267005	0.071	0.069	1
Zfp677	0.5603647	0.43622058	0.017	0.015	1
Utp4	0.56158938	0.34931593	0.032	0.034	1
Pxmp4	0.56223522	0.46407968	0.027	0.029	1
Mettl16	0.56319585	0.48213225	0.068	0.067	1
Slc41a1	0.56327446	0.4697434	0.078	0.077	1
Ruvbl2	0.56515323	0.64751952	0.024	0.026	1
Snrpd3	0.56664664	0.43275635	0.232	0.252	1
Pcgf3	0.56796922	0.66488512	0.031	0.034	1
Mark3	0.56868327	0.35341352	0.157	0.173	1
Arhgap5	0.57015126	0.46372841	0.069	0.075	1
Rcn1	0.57219539	0.55308441	0.02	0.018	1
Cenpo	0.57233706	0.31412865	0.009	0.01	1
Ccl11	0.57298858	0.40372459	0.014	0.013	1
Rmnd5a	0.57313912	0.28445629	0.119	0.129	1
Pdxdc1	0.57536953	0.40585165	0.048	0.052	1
Camk2g	0.57562963	0.46763628	0.04	0.043	1
Gm26526	0.5760306	0.76261505	0.021	0.019	1
Rtn4rl1	0.5765516	0.79382509	0.012	0.011	1
Zfp639	0.57671931	0.3204366	0.079	0.085	1
Snx19	0.57800099	0.43056267	0.027	0.025	1
Phf2	0.57928279	0.44450072	0.071	0.07	1
Tsen15	0.57930279	0.37285713	0.021	0.019	1
Xpo7	0.57947799	0.41304193	0.067	0.065	1
Mb21d1	0.57978006	0.54810089	0.021	0.02	1
Lrp12	0.58022081	0.5788937	0.012	0.013	1

Fchsd1	0.58116456	0.5196454	0.013	0.011	1
Tceanc	0.58317765	0.6255563	0.011	0.01	1
Rrn3	0.58323213	0.45908968	0.054	0.052	1
Armt1	0.58374501	0.49617929	0.029	0.027	1
Eid2b	0.58516127	0.29160897	0.01	0.012	1
Cd226	0.58532273	0.28071077	0.052	0.05	1
Kbtbd2	0.58536778	0.27672031	0.106	0.114	1
Luc7l	0.58657393	0.35803996	0.126	0.128	1
Aatf	0.58678063	0.62192257	0.055	0.054	1
Jpx	0.5880029	0.48313052	0.014	0.012	1
Cd1d1	0.58827454	0.55170523	0.022	0.024	1
Zmym4	0.58831435	0.34243808	0.047	0.051	1
Mbd5	0.58995999	0.43407706	0.029	0.032	1
Arfip2	0.59075142	0.50185352	0.014	0.015	1
Rint1	0.59290201	0.49250938	0.032	0.03	1
Zfp597	0.59358238	0.43655639	0.028	0.027	1
Ccdc71	0.59462055	0.46871053	0.033	0.035	1
Ccdc47	0.59689639	0.41299381	0.093	0.102	1
Snrnp48	0.59696241	0.27016826	0.192	0.198	1
Pola2	0.59740633	0.63897933	0.021	0.019	1
Usp20	0.59769023	0.49788187	0.014	0.012	1
Farsa	0.59769067	0.49278518	0.057	0.062	1
Ephx1	0.59781703	0.2625369	0.033	0.031	1
Ranbp6	0.59786395	0.3826189	0.025	0.027	1
Ascc2	0.5979521	0.46433703	0.037	0.04	1
Slc35d1	0.59826692	0.45620476	0.043	0.042	1
2210016L21Ri	0.60091111	0.45989043	0.062	0.067	1
Gstk1	0.60134158	0.7310187	0.01	0.011	1
Micu3	0.6019347	0.4640064	0.027	0.026	1
Zfp668	0.601936	0.60539987	0.013	0.011	1
Appl2	0.60308841	0.55417715	0.024	0.026	1
Mccc1	0.60387936	0.42928033	0.01	0.012	1
Mto1	0.60399727	0.3194248	0.016	0.017	1
Cbx1	0.60440709	0.41726435	0.157	0.162	1
Cep192	0.60662976	0.38377247	0.079	0.078	1
Rida	0.60685359	0.50002606	0.02	0.022	1
Chfr	0.60705934	0.26827489	0.131	0.141	1
Spred2	0.60767601	0.34998827	0.057	0.061	1
Tpcn1	0.60869269	0.77727947	0.03	0.033	1
Cops7b	0.60951135	0.35522332	0.018	0.02	1
Ppil3	0.61042561	0.36298411	0.05	0.054	1
Brms1l	0.61102272	0.88387953	0.019	0.018	1
1700021F05Ri	0.61129927	0.48653336	0.019	0.021	1
6720427I07Ril	0.61273809	0.38716326	0.035	0.033	1
Akap9	0.61544476	0.40394644	0.181	0.19	1
Vps45	0.6159001	0.56076218	0.018	0.019	1
8030462N17R	0.6167523	0.46702234	0.022	0.024	1
Riok2	0.61771054	0.25468088	0.042	0.045	1

Rps6ka3	0.61896724	0.43119398	0.133	0.136	1
Dhx32	0.61960355	0.57885787	0.018	0.016	1
2700062C07R	0.62056239	0.29148089	0.027	0.029	1
Pcnt	0.62111135	0.39311904	0.105	0.106	1
Agfg2	0.62127212	0.88392152	0.02	0.019	1
Phospho2	0.62130849	0.27439386	0.045	0.048	1
Smyd5	0.62217883	0.39660994	0.021	0.023	1
Dnajc7	0.62249389	0.35400519	0.183	0.192	1
Nae1	0.62250184	0.32172082	0.093	0.1	1
Klhl20	0.62250866	0.40343882	0.036	0.034	1
Pygo2	0.62278931	0.52601578	0.025	0.023	1
Ikbkg	0.62357344	0.53437003	0.033	0.031	1
Ext1	0.6238501	0.56174463	0.078	0.085	1
Skp1a	0.62568936	0.33450726	0.214	0.228	1
Tcf3	0.62592426	0.37302972	0.058	0.056	1
Ikbkap	0.62709389	0.45405901	0.034	0.032	1
Slc30a7	0.62720812	0.71303775	0.045	0.044	1
Ivd	0.62766481	0.66074828	0.03	0.028	1
R3hcc1	0.62981876	0.38661055	0.018	0.017	1
Ikzf5	0.62996831	0.25859449	0.09	0.097	1
Strn3	0.63097477	0.36121922	0.149	0.154	1
Aasdh	0.63100052	0.57360092	0.014	0.013	1
Atp6v0a2	0.63153208	0.53468972	0.066	0.065	1
Zfp407	0.63261015	0.42199313	0.063	0.062	1
Ubtf	0.63356918	0.32948922	0.152	0.165	1
Vamp1	0.63429704	0.47675453	0.024	0.022	1
Mepce	0.63491619	0.3374888	0.117	0.118	1
Spg11	0.6353113	0.47940557	0.021	0.02	1
Gm10138	0.6358837	0.34419367	0.041	0.039	1
Hectd3	0.63700714	0.52457984	0.021	0.023	1
Mta1	0.63750623	0.423628	0.071	0.076	1
Rbm28	0.63784461	0.40466877	0.143	0.147	1
Bag4	0.63799913	0.35475818	0.05	0.048	1
Usp47	0.63828457	0.28859506	0.134	0.145	1
Zfpm1	0.63975381	0.41561205	0.038	0.036	1
Abcg2	0.64033006	0.53095579	0.009	0.01	1
Ddx10	0.64247016	0.41462625	0.097	0.097	1
Srsf1	0.64439566	0.25262646	0.185	0.189	1
Aamdc	0.64518314	0.70100246	0.026	0.024	1
Zfp518a	0.64528457	0.43826701	0.05	0.049	1
Qser1	0.64542994	0.48907375	0.018	0.017	1
Dhx16	0.64572697	0.55765349	0.071	0.076	1
Ermard	0.64743411	0.46033465	0.022	0.021	1
Odf2l	0.64772685	0.37068942	0.017	0.016	1
Fam120b	0.64781308	0.35992724	0.059	0.058	1
Zfp146	0.64860874	0.25244408	0.042	0.045	1
Slc10a3	0.64865379	0.26758907	0.022	0.024	1
Kctd2	0.64900038	0.37576919	0.037	0.04	1

Nup188	0.64969772	0.32369425	0.028	0.03	1
Vps39	0.6501799	0.52950127	0.037	0.036	1
Kat8	0.65042794	0.64681787	0.019	0.018	1
Cul4b	0.65063542	0.37372847	0.05	0.049	1
Invs	0.65142401	0.51890928	0.015	0.014	1
Mical3	0.65145704	0.66863035	0.012	0.011	1
Ccnh	0.65592318	0.44888815	0.043	0.046	1
Helb	0.65641274	0.40858845	0.059	0.058	1
Vps26b	0.65904536	0.28123624	0.041	0.044	1
Ppm1b	0.65965617	0.36739687	0.128	0.13	1
Churc1	0.66294454	0.39572494	0.201	0.215	1
Mbd1	0.66339327	0.48306806	0.071	0.07	1
Apbb1ip	0.66348078	0.28651071	0.183	0.2	1
Upf1	0.66363479	0.35647339	0.096	0.103	1
Nek1	0.66402078	0.48924418	0.016	0.014	1
Cab39l	0.6640784	0.33694453	0.059	0.058	1
Vps13b	0.66639547	0.43476409	0.092	0.093	1
Setdb1	0.66640119	0.36874494	0.038	0.04	1
Mbd4	0.66678861	0.49760186	0.018	0.017	1
Smg6	0.66679574	0.25315973	0.127	0.136	1
Cry2	0.66767173	0.49189011	0.024	0.023	1
Klhl18	0.66930559	0.32494866	0.03	0.029	1
Lmtk2	0.6700679	0.43219035	0.052	0.051	1
Mre11a	0.67020428	0.46647865	0.04	0.039	1
Erap1	0.67103802	0.53631758	0.09	0.091	1
Zfp212	0.67184161	0.27535916	0.038	0.04	1
D730003I15Ri	0.6733361	0.72496087	0.037	0.036	1
Prmt5	0.67438712	0.38824301	0.042	0.041	1
Prr3	0.6751082	0.45316042	0.028	0.03	1
Fbxo9	0.67523442	0.40555353	0.058	0.057	1
Cdc37l1	0.67620323	0.29855235	0.18	0.186	1
Agps	0.67625774	0.3744944	0.087	0.093	1
Thrap3	0.67710864	0.25268369	0.36	0.401	1
Pex1	0.67894339	0.3971223	0.018	0.019	1
Dcakd	0.67924418	0.61667397	0.012	0.011	1
Sit1	0.67924688	0.36974912	0.027	0.025	1
Ick	0.6800994	0.68100211	0.01	0.009	1
Guf1	0.68058974	0.40281579	0.03	0.029	1
Nudt16	0.68130808	0.5183908	0.036	0.035	1
Mmgt1	0.6821136	0.46326082	0.029	0.031	1
Cdk8	0.68325772	0.38704544	0.061	0.065	1
Tmem201	0.6843132	0.31449122	0.011	0.012	1
Tcta	0.68473628	0.39669285	0.01	0.009	1
Igf2bp3	0.68552561	0.2929784	0.014	0.015	1
Phf11a	0.68603489	0.59176951	0.01	0.011	1
Dcaf15	0.68733207	0.36335496	0.086	0.086	1
Etfbkmt	0.68822097	0.76079905	0.011	0.01	1
Ptbp2	0.68835578	0.25333596	0.125	0.134	1

Rps12-ps3	0.68843831	0.44140115	0.07	0.075	1
Gm42047	0.68883949	0.27542378	0.01	0.009	1
Lsm14a	0.69183361	0.31656836	0.177	0.184	1
Foxred1	0.69345125	0.35580745	0.026	0.025	1
Gna11	0.69354981	0.7101955	0.022	0.021	1
Ankhd1	0.69424516	0.33403934	0.132	0.135	1
Ebag9	0.69426925	0.28092262	0.044	0.047	1
Aen	0.69491801	0.47294614	0.032	0.03	1
Ppa2	0.69534904	0.55621779	0.028	0.03	1
Ccdc77	0.69547822	0.60305883	0.014	0.013	1
Zfp24	0.69551448	0.26872009	0.058	0.062	1
Zgpat	0.69645423	0.30728485	0.037	0.04	1
Ndc80	0.69708885	0.3762365	0.015	0.014	1
Atxn7l2	0.69748417	0.56179018	0.017	0.016	1
Abcd4	0.69843618	0.53267336	0.022	0.021	1
Safb	0.69861376	0.32690079	0.19	0.199	1
Axin1	0.70047662	0.29744839	0.072	0.077	1
Aspn	0.70052382	0.38756113	0.012	0.011	1
Ccnt2	0.70099398	0.33852009	0.189	0.198	1
Zfp709	0.70120715	0.77376413	0.012	0.011	1
Slx4ip	0.70140611	0.57160558	0.023	0.022	1
Rpap1	0.70191125	0.29639372	0.019	0.02	1
Nipsnap2	0.7026647	0.49219741	0.036	0.038	1
2900060B14R	0.70296079	0.3563028	0.051	0.05	1
Terf2	0.70309015	0.3927213	0.059	0.062	1
Dctn6	0.70320699	0.55283742	0.071	0.071	1
Ap3m1	0.70376465	0.39982263	0.05	0.053	1
Uggt1	0.70557358	0.47889337	0.097	0.105	1
Dus1l	0.70622586	0.44002685	0.028	0.03	1
Setdb2	0.70650663	0.25482783	0.034	0.032	1
Sh3gl1	0.70719975	0.46569838	0.052	0.051	1
Rnf220	0.70758614	0.27692775	0.119	0.127	1
Kansl3	0.70869623	0.29669591	0.125	0.134	1
Zfp770	0.71007503	0.49076845	0.021	0.02	1
Inpp5b	0.7102549	0.53484027	0.032	0.031	1
Nprl2	0.71280099	0.5365876	0.017	0.016	1
Ddx11	0.71316328	0.37969198	0.012	0.011	1
Uspl1	0.71367904	0.27508008	0.09	0.09	1
Phkb	0.71479815	0.87319727	0.033	0.032	1
Fyco1	0.71576423	0.32348866	0.054	0.057	1
Zfp664	0.7164745	0.34026276	0.061	0.065	1
mt-Nd6	0.71678775	0.31573471	0.064	0.068	1
AW554918	0.71699283	0.30179523	0.025	0.024	1
Ano6	0.7172697	0.55784672	0.077	0.082	1
Pcid2	0.71945918	0.45338563	0.039	0.042	1
AU040320	0.72022342	0.56026645	0.032	0.031	1
Tomm70a	0.7213562	0.34072991	0.15	0.163	1
Pdik1l	0.72177951	0.30957671	0.023	0.025	1

Cdc25a	0.72286456	0.39317937	0.031	0.033	1
Riox2	0.72313494	0.38487128	0.044	0.046	1
Pum1	0.72477237	0.25091911	0.229	0.244	1
Ptges2	0.72735522	0.4235233	0.013	0.014	1
Coq5	0.72840669	0.44683349	0.054	0.053	1
Hirip3	0.73145127	0.42946828	0.034	0.033	1
Dpm1	0.7324848	0.29248997	0.108	0.109	1
Gpatch1	0.73249751	0.39379212	0.013	0.014	1
Fubp3	0.73270252	0.44637413	0.025	0.027	1
Gdi1	0.73402784	0.34921483	0.106	0.113	1
Cabin1	0.73433507	0.40796454	0.098	0.099	1
Acad8	0.73543811	0.62539731	0.011	0.012	1
Bbx	0.73559639	0.26855131	0.142	0.152	1
Alg12	0.73611936	0.6974576	0.013	0.012	1
Smim1	0.73758673	0.33404547	0.012	0.013	1
Col12a1	0.73821776	0.33477728	0.01	0.011	1
Pgm2	0.73922965	0.42559379	0.019	0.02	1
Nosip	0.73928477	0.29388823	0.053	0.056	1
Actr5	0.74041228	0.2871127	0.045	0.047	1
Suclg2	0.74073418	0.44757046	0.061	0.061	1
Rpain	0.7416007	0.54612617	0.03	0.029	1
Rmi1	0.74209963	0.2728829	0.049	0.051	1
Lppos	0.74249508	0.49367508	0.021	0.022	1
6230400D17R	0.74282235	0.39328782	0.013	0.012	1
Pcca	0.74282866	0.53807196	0.021	0.021	1
Hist1h2be	0.74319467	0.25618517	0.038	0.037	1
Kmt5b	0.74395713	0.32616445	0.16	0.166	1
Nit2	0.7444314	0.69447954	0.023	0.025	1
Secisbp2	0.74444257	0.44897353	0.077	0.078	1
Vcpip1	0.74457965	0.29620828	0.129	0.138	1
Ttf1	0.74492066	0.3625109	0.066	0.07	1
Mlycd	0.7453987	0.53967078	0.014	0.013	1
Eral1	0.74640195	0.53843947	0.016	0.015	1
Suv39h1	0.74784351	0.38029284	0.026	0.025	1
Asxl1	0.74897374	0.33454086	0.079	0.083	1
Gtpbp6	0.74978826	0.50947645	0.037	0.036	1
Ddx27	0.75038697	0.36333946	0.102	0.108	1
Ippk	0.75106602	0.71913377	0.011	0.01	1
Dhx36	0.75163314	0.31484377	0.225	0.239	1
Ric8b	0.75197324	0.3635897	0.02	0.02	1
Polr3gl	0.75248604	0.44451131	0.033	0.032	1
Anapc5	0.75340565	0.36844774	0.127	0.138	1
Mesd	0.75354746	0.42342228	0.051	0.054	1
Zfp397	0.7548751	0.30707469	0.08	0.084	1
Cep162	0.75507841	0.52761831	0.024	0.026	1
Azi2	0.7556495	0.28837629	0.166	0.179	1
F8a	0.7558272	0.34918678	0.024	0.025	1
Vps33b	0.75771425	0.26424149	0.017	0.018	1

Lrp6	0.75799865	0.43873556	0.08	0.081	1
Mmp2	0.75977758	0.30710271	0.02	0.022	1
March6	0.7609917	0.33388776	0.108	0.115	1
Mtg1	0.76255151	0.88385785	0.011	0.011	1
Skiv2l	0.76259158	0.5054335	0.075	0.076	1
Srrt	0.76350582	0.50835449	0.132	0.138	1
Rab27a	0.76387834	0.33415769	0.046	0.048	1
Dnaja3	0.76508436	0.48289139	0.018	0.017	1
Parp3	0.76564284	0.60523188	0.042	0.041	1
Rnf219	0.76587761	0.3755268	0.019	0.02	1
Rad51d	0.76602569	0.5161512	0.024	0.023	1
Tasp1	0.76682759	0.30151787	0.03	0.029	1
Mtcp1	0.76828659	0.42095682	0.014	0.013	1
Zw10	0.7693721	0.29324624	0.029	0.028	1
Ankrd55	0.76941213	0.84864982	0.011	0.01	1
Rft1	0.7695359	0.30568029	0.016	0.015	1
Mrrf	0.76971226	0.41276035	0.023	0.025	1
Dph6	0.77010794	0.35428673	0.038	0.04	1
Mrps35	0.77114596	0.53943896	0.056	0.056	1
Smpd2	0.77499243	0.44319721	0.012	0.012	1
Ifrd2	0.77700877	0.44583662	0.011	0.011	1
Cnnm3	0.77715299	0.48716633	0.033	0.032	1
Depdc5	0.77732068	0.29840342	0.027	0.028	1
Nedd1	0.77855016	0.30093969	0.023	0.024	1
Slfn9	0.77865809	0.31118171	0.011	0.012	1
Arhgap1	0.78028628	0.59155361	0.07	0.071	1
Tfb2m	0.78081578	0.36353275	0.045	0.047	1
Ercc6l2	0.78094473	0.32394297	0.036	0.038	1
Slc30a9	0.78106471	0.39475682	0.084	0.085	1
Sag	0.78434817	0.34495065	0.011	0.011	1
Naa30	0.78489283	0.37005403	0.068	0.071	1
Slc25a53	0.78532657	0.30759634	0.018	0.017	1
Ecsit	0.78654269	0.4395471	0.035	0.034	1
Setd1a	0.78697801	0.4762347	0.053	0.056	1
Tacc2	0.78701701	0.44392387	0.009	0.01	1
Rwdd2b	0.78745271	0.34386052	0.01	0.011	1
Zfp169	0.78755308	0.34324806	0.02	0.02	1
Edc4	0.78874043	0.25336084	0.065	0.068	1
Mgme1	0.78921155	0.57870782	0.012	0.013	1
Cars2	0.79034024	0.45469165	0.039	0.041	1
Ltn1	0.79113125	0.41752512	0.068	0.068	1
Scoc	0.79258784	0.44804139	0.04	0.042	1
Slc10a7	0.79273473	0.38260627	0.027	0.027	1
Fam13b	0.79386934	0.41805172	0.064	0.067	1
Tex9	0.79547068	0.34271742	0.015	0.014	1
Slco3a1	0.79671038	0.45674683	0.144	0.151	1
Trp53bp1	0.79702095	0.39252821	0.048	0.05	1
Zfat	0.79795763	0.4702927	0.01	0.011	1

Ppid	0.79804573	0.47662695	0.067	0.067	1
Nr2c2ap	0.79824328	0.60002678	0.047	0.047	1
Sacs	0.80058294	0.28083297	0.058	0.061	1
Gpt	0.80065732	0.82752102	0.01	0.009	1
Ldah	0.80094388	0.39733716	0.045	0.047	1
4933433G15R	0.80238096	0.31518306	0.016	0.015	1
Fam114a2	0.80451789	0.38046212	0.081	0.085	1
Trmt13	0.80488032	0.33109372	0.062	0.062	1
Ripk1	0.80616465	0.4999207	0.146	0.155	1
Arhgef11	0.80633557	0.83263636	0.025	0.027	1
Mgp	0.80860198	0.37821781	0.021	0.022	1
Tom1l2	0.81130529	0.60292713	0.01	0.011	1
Prdm15	0.81420343	0.45441639	0.013	0.012	1
Nup43	0.81424877	0.5382554	0.012	0.012	1
Ctnnbl1	0.81495639	0.61132614	0.065	0.066	1
Ncoa4	0.8149988	0.48771609	0.072	0.076	1
Rarres2	0.81527768	0.32032499	0.021	0.021	1
Nt5dc1	0.81528389	0.4365954	0.039	0.039	1
Nop58	0.81547362	0.27511654	0.19	0.2	1
Fkbp5	0.81562481	0.26658174	0.033	0.032	1
Rrp15	0.81579911	0.30963984	0.052	0.054	1
Cnp	0.8165672	0.47587281	0.068	0.069	1
Hist1h3f	0.81659329	0.35899943	0.02	0.021	1
Heatr1	0.81703057	0.27881258	0.057	0.06	1
Lrig2	0.81752188	0.37813216	0.032	0.031	1
Zkscan14	0.81812354	0.26551876	0.046	0.048	1
Fbxo46	0.81926736	0.39018897	0.018	0.017	1
Miip	0.82565376	0.46072955	0.026	0.027	1
Tmem268	0.82573997	0.5989602	0.019	0.02	1
Gnl3l	0.82841067	0.38220801	0.071	0.074	1
Gm26722	0.82923701	0.30524597	0.024	0.025	1
Wdr46	0.82969511	0.51187218	0.025	0.025	1
Epm2aip1	0.83043495	0.3105159	0.059	0.059	1
Pskh1	0.83044816	0.42484369	0.019	0.02	1
Abtb2	0.8319507	0.35030069	0.058	0.061	1
Rnaseh1	0.83197424	0.47642428	0.025	0.026	1
Pwp2	0.83347713	0.27392999	0.02	0.019	1
Mta2	0.83425372	0.30340159	0.126	0.13	1
Prrc2b	0.83430849	0.2510489	0.178	0.185	1
Ftsj1	0.83458182	0.33509703	0.013	0.013	1
Ppp6r2	0.83535876	0.34627865	0.028	0.029	1
Timm21	0.83765043	0.67624135	0.023	0.024	1
Dbp	0.83789013	0.64265401	0.014	0.014	1
Clns1a	0.83793313	0.37066894	0.088	0.093	1
Trak2	0.83966218	0.36655747	0.072	0.075	1
Ttll5	0.84081724	0.59351445	0.018	0.018	1
Ogfod1	0.8417354	0.86098438	0.018	0.018	1
Riox1	0.84362287	0.33862412	0.024	0.025	1

Myo6	0.84472689	0.38569676	0.019	0.02	1
Brpf3	0.84549492	0.40465896	0.02	0.021	1
Zbtb48	0.84621914	0.28853688	0.011	0.011	1
Rgs14	0.84693462	0.39927621	0.028	0.027	1
Cdk16	0.84768544	0.43768316	0.03	0.031	1
Ncln	0.84780115	0.65578578	0.048	0.049	1
Gpr180	0.84838599	0.45488486	0.016	0.016	1
Acad9	0.8490856	0.54259173	0.018	0.018	1
Tmem80	0.84946752	0.48706044	0.031	0.03	1
Rrm2b	0.85072817	0.2641334	0.064	0.066	1
Mrpl24	0.85121225	0.28589511	0.209	0.228	1
B4galt7	0.8516978	0.56676379	0.019	0.019	1
Kpnb1	0.85239317	0.37051392	0.13	0.139	1
Zgrf1	0.85290461	0.30541346	0.012	0.012	1
1300002E11R	0.85693041	0.29957582	0.039	0.039	1
Lrrc14	0.85739876	0.50397828	0.018	0.018	1
Gm340	0.8575326	0.47525202	0.017	0.018	1
Pick1	0.85824654	0.46488205	0.018	0.018	1
Gm29361	0.8583351	0.40512525	0.012	0.011	1
C1s1	0.85949608	0.3244773	0.019	0.02	1
Cyp4f13	0.85990913	0.62570591	0.023	0.023	1
Tspyl2	0.86035711	0.66333414	0.032	0.032	1
Vps13d	0.86151966	0.39744408	0.087	0.091	1
Lrrc61	0.86268328	0.63541152	0.016	0.016	1
Prdm11	0.86567553	0.27202613	0.018	0.018	1
Exo5	0.86582088	0.40594965	0.01	0.01	1
Aldh18a1	0.86601713	0.57042504	0.026	0.027	1
Csad	0.86810919	0.86200029	0.014	0.014	1
Mapk8ip3	0.86821352	0.44897926	0.075	0.076	1
Rnpepl1	0.86826364	0.48531361	0.058	0.06	1
Ptcd2	0.86915517	0.65607945	0.063	0.064	1
Trappc13	0.87184821	0.46296187	0.05	0.05	1
Rnf169	0.87187034	0.4011174	0.037	0.038	1
Zfp865	0.87228911	0.47773195	0.039	0.039	1
Borcs5	0.87231702	0.42911991	0.019	0.02	1
Stradb	0.87254739	0.47209954	0.017	0.017	1
Wdr47	0.87305724	0.37042575	0.02	0.021	1
Med26	0.87353295	0.38678511	0.025	0.026	1
Akap10	0.87417193	0.26480148	0.069	0.069	1
Exoc6	0.87473254	0.38205345	0.048	0.048	1
Surf6	0.87509002	0.35988511	0.054	0.054	1
Dnaaf5	0.87802561	0.53861884	0.01	0.01	1
Zc3h4	0.87810272	0.37142591	0.056	0.059	1
Uty	0.879759	0.28335316	0.052	0.052	1
Nup35	0.87990221	0.45025531	0.016	0.016	1
Sp110	0.88055617	0.31182074	0.178	0.191	1
Ankrd50	0.88190579	0.58514131	0.046	0.046	1
Polr1b	0.88191012	0.32000434	0.011	0.012	1

Dag1	0.88527473	0.59834341	0.03	0.03	1
Heatr6	0.88697671	0.36667842	0.044	0.044	1
Rarg	0.8872949	0.73164266	0.012	0.012	1
Glce	0.88797539	0.33480414	0.021	0.021	1
Rngtt	0.88883498	0.45092909	0.081	0.083	1
Mrm1	0.88898257	0.5394573	0.012	0.012	1
Trp53rkb	0.88926386	0.45125851	0.02	0.02	1
Arhgap12	0.88952875	0.37734255	0.036	0.037	1
Cwf19l1	0.89014577	0.58150371	0.011	0.011	1
Ppp2r5e	0.89260343	0.32548752	0.091	0.095	1
4632415L05Ri	0.89290464	0.67364802	0.012	0.013	1
Cbx8	0.893372	0.53745249	0.012	0.013	1
P4ha1	0.89381978	0.3029528	0.12	0.125	1
Meis3	0.89426502	0.43946048	0.014	0.013	1
Ercc4	0.89445962	0.39002905	0.028	0.029	1
Pcsk7	0.89450237	0.30704087	0.064	0.066	1
Dbf4	0.89454227	0.3737925	0.096	0.1	1
Ptk2	0.89512189	0.47505206	0.011	0.011	1
Bpgm	0.89544141	0.52902342	0.02	0.02	1
Zfp687	0.89674189	0.36798827	0.029	0.029	1
Lnpk	0.89688425	0.29770756	0.082	0.085	1
Osbpl11	0.89713491	0.39908787	0.059	0.061	1
Cox10	0.89802525	0.36417506	0.05	0.052	1
Elac1	0.89881138	0.50041294	0.015	0.014	1
1700097N02R	0.90087275	0.40229422	0.014	0.015	1
Mocs3	0.90148694	0.72625304	0.01	0.011	1
Gm26759	0.90157759	0.44809926	0.025	0.025	1
Cep41	0.90221939	0.41948807	0.014	0.013	1
Psmg2	0.90236652	0.42531385	0.038	0.04	1
Zcchc17	0.90304249	0.50978389	0.097	0.103	1
Impdh1	0.90505658	0.40270878	0.055	0.056	1
Gtpbp8	0.90514413	0.62492726	0.019	0.019	1
Tagap1	0.9059886	0.40799094	0.023	0.024	1
Nck1	0.90670837	0.30886149	0.142	0.15	1
Zfp758	0.90675708	0.32966685	0.029	0.03	1
Cacybp	0.90736514	0.39870954	0.17	0.181	1
Tmem181a	0.90766444	0.52865168	0.028	0.029	1
Ncoa6	0.90955091	0.53498284	0.073	0.075	1
Zfp182	0.90983317	0.41217218	0.022	0.022	1
Mrps5	0.91038829	0.41609744	0.082	0.085	1
Dlg1	0.91130839	0.33518776	0.09	0.093	1
Platr17	0.91158614	0.81212817	0.011	0.012	1
Srm	0.91199808	0.3423438	0.037	0.039	1
Ints3	0.91247517	0.52998724	0.032	0.032	1
Rev1	0.91265381	0.36363321	0.047	0.048	1
Ppan	0.91279326	0.3313428	0.051	0.052	1
Kdm3a	0.91288855	0.35188229	0.098	0.102	1
Mettl22	0.91290234	0.62382508	0.01	0.01	1

Zfp110	0.91405434	0.27256503	0.062	0.064	1
Gm10076	0.91470456	0.38565645	0.16	0.17	1
Med12	0.91619174	0.31455708	0.071	0.072	1
Ddi2	0.91653394	0.40824761	0.073	0.076	1
Fam105a	0.91654474	0.51427091	0.102	0.108	1
Dnajc16	0.91916248	0.67619687	0.013	0.012	1
Rpl27-ps3	0.91966127	0.37065189	0.095	0.098	1
Anks3	0.92064189	0.39893939	0.038	0.038	1
Zscan29	0.92070723	0.44629963	0.024	0.025	1
Acp6	0.92209401	0.39927708	0.014	0.014	1
Zfp329	0.92365757	0.38532149	0.051	0.052	1
Racgap1	0.92393764	0.39142715	0.028	0.028	1
Nat2	0.92439818	0.61106525	0.013	0.012	1
Trappc11	0.92499748	0.48880941	0.037	0.039	1
Ust	0.92593442	0.66939673	0.018	0.018	1
Kif3b	0.92636882	0.60348522	0.035	0.035	1
Gm6133	0.92691953	0.50228187	0.03	0.03	1
Dtymk	0.92701981	0.5201213	0.057	0.058	1
Zfp626	0.92723734	0.44752469	0.025	0.025	1
Marf1	0.93101493	0.26421239	0.114	0.119	1
Osbpl2	0.93146311	0.44713147	0.036	0.037	1
Ganc	0.93178466	0.37364088	0.032	0.032	1
Coq3	0.93286723	0.32837556	0.012	0.012	1
Zfp866	0.93312994	0.44806393	0.024	0.024	1
Gne	0.93519653	0.29720688	0.01	0.01	1
Cradd	0.93631684	0.30460377	0.017	0.017	1
Sec23ip	0.93660855	0.34925437	0.056	0.058	1
Gapvd1	0.93698257	0.29696895	0.15	0.157	1
Rpp38	0.93941341	0.40230117	0.024	0.024	1
Cnot3	0.93973645	0.27608686	0.162	0.172	1
Rbmx2	0.93999576	0.43228457	0.05	0.051	1
Mthfd1	0.94047976	0.46724463	0.023	0.023	1
Kcnab2	0.94159454	0.43047123	0.053	0.054	1
Frmd6	0.94268196	0.61156957	0.041	0.041	1
Klhl11	0.94337213	0.6923253	0.016	0.016	1
Coq9	0.94338232	0.52386786	0.02	0.021	1
Rtn4ip1	0.94348214	0.44344577	0.012	0.012	1
Zbtb40	0.94356964	0.46718421	0.016	0.015	1
Nedd4l	0.94389098	0.6515544	0.038	0.038	1
Rxrb	0.94405321	0.49219804	0.06	0.062	1
Eef1akmt1	0.94532436	0.63093511	0.03	0.03	1
Acbd5	0.94587018	0.32862612	0.157	0.167	1
Rhno1	0.9476507	0.3218272	0.047	0.047	1
Otud4	0.94772818	0.25338617	0.12	0.124	1
Tbck	0.94838823	0.42995478	0.049	0.049	1
Strada	0.94882519	0.46758644	0.033	0.033	1
Tmem18	0.94894482	0.3452653	0.014	0.014	1
Bmi1	0.94996777	0.31489003	0.041	0.041	1

Amd2	0.95021929	0.48067074	0.015	0.015	1
Nepro	0.95052301	0.59872608	0.028	0.028	1
Zfp938	0.95056375	0.37631295	0.023	0.024	1
Gid4	0.95110859	0.51238008	0.053	0.054	1
Tmem70	0.9516461	0.593666	0.046	0.047	1
Adpgk	0.95434608	0.37141118	0.055	0.056	1
Tmem161a	0.95512611	0.44782615	0.031	0.031	1
Kat14	0.95516604	0.31757665	0.02	0.02	1
Hist1h2bj	0.95699984	0.5314103	0.016	0.016	1
Atp9b	0.95731666	0.53055216	0.085	0.089	1
Eogt	0.95763918	0.82623632	0.012	0.012	1
Wdr73	0.95956484	0.48848363	0.018	0.017	1
Cdyl2	0.95961659	0.27831658	0.013	0.013	1
Pwwp2a	0.96081767	0.33058381	0.081	0.083	1
Rhebl1	0.9610621	0.28547557	0.011	0.011	1
Orc3	0.96189089	0.30492046	0.044	0.044	1
Rfxank	0.96260726	0.57340136	0.019	0.02	1
Tmem55a	0.96295892	0.5667282	0.018	0.018	1
Alg11	0.96356968	0.56776937	0.023	0.023	1
Limk1	0.9660349	0.60858826	0.015	0.015	1
Klhl21	0.9673282	0.32259942	0.032	0.032	1
Klhl7	0.96802184	0.54343259	0.026	0.026	1
Prr14	0.96976086	0.30321978	0.095	0.098	1
Hdac2	0.96996352	0.40167475	0.063	0.065	1
Araf	0.97011176	0.33558057	0.113	0.117	1
Fbxo22	0.97057753	0.40862889	0.048	0.048	1
Atg16l1	0.97121435	0.32096504	0.069	0.07	1
Ltbp4	0.97205484	0.3444751	0.023	0.023	1
Tmem65	0.97298205	0.66005619	0.074	0.076	1
Mettl23	0.9737521	0.32650206	0.213	0.229	1
Map3k12	0.97380024	0.58008218	0.02	0.021	1
Fra10ac1	0.97417833	0.28474535	0.018	0.018	1
Lztr1	0.9753848	0.49493186	0.022	0.023	1
Wwox	0.97798915	0.48425325	0.023	0.023	1
Ccdc130	0.97836796	0.45009206	0.017	0.017	1
Cad	0.9788875	0.38565752	0.022	0.022	1
Pibf1	0.97955293	0.37082022	0.054	0.055	1
Zfp654	0.97992641	0.25446532	0.058	0.059	1
Ndufaf5	0.98025309	0.54517231	0.014	0.014	1
Serping1	0.9807711	0.40427199	0.021	0.021	1
Tubg1	0.98310704	0.79605445	0.01	0.01	1
Ttyh3	0.98313989	0.61618071	0.07	0.072	1
Sidt2	0.98321958	0.31629389	0.131	0.137	1
Nmi	0.98357731	0.47213125	0.117	0.123	1
Tango2	0.98441152	0.30674778	0.023	0.023	1
2610002M06F	0.98485378	0.34806441	0.031	0.031	1
2810402E24R	0.9854072	0.3371592	0.057	0.059	1
Ppa1	0.98791753	0.59888442	0.074	0.077	1

Nop14	0.98827966	0.34650695	0.125	0.13	1
Sbf1	0.98847574	0.44583152	0.102	0.106	1
Trim28	0.98976113	0.44851053	0.117	0.123	1
Bmp4	0.99124836	0.29569247	0.011	0.011	1
Phf7	0.99154698	0.53217803	0.016	0.016	1
Hjurp	0.99287367	0.36156379	0.082	0.085	1
Rab23	0.99330188	0.4273269	0.01	0.01	1
Phf6	0.99396677	0.29078649	0.095	0.098	1
Dolpp1	0.99404712	0.51357057	0.011	0.011	1
Urb2	0.99478949	0.53564198	0.014	0.014	1
Rabgap1	0.99534656	0.39430522	0.066	0.068	1
Syt11	0.99556205	0.63911768	0.033	0.034	1
Oxr1	0.99594801	0.3137754	0.082	0.084	1
Slc25a25	0.99646669	0.31168894	0.054	0.054	1
Zfp943	0.99718627	0.30222047	0.025	0.025	1
Psmg1	0.99848833	0.72992963	0.017	0.017	1
Mob3a	0.99881904	0.58673381	0.053	0.054	1
Mettl2	0.99937962	0.29018819	0.024	0.024	1
Tex10	0.99968735	0.32059332	0.103	0.107	1

CD8 Trm

Gene	p_val	avg_log2FC	pct.1	pct.2	p_val_adj
Stmn1	0	5.16151833	0.974	0.067	0
Pclaf	0	5.89034915	0.922	0.027	0
Hist1h1b	0	5.78374188	0.941	0.055	0
Top2a	0	4.5867892	0.967	0.088	0
Hist1h3c	0	6.28736176	0.869	0.014	0
Cd8b1	0	3.50885735	0.922	0.075	0
Hist1h2ae	0	5.80491504	0.843	0.038	0
Birc5	0	5.5859176	0.778	0.017	0
Hist1h2ap	0	5.21621842	0.778	0.027	0
Nusap1	0	5.48820593	0.758	0.015	0
Cks1b	0	4.74516319	0.765	0.038	0
Smc2	0	4.35485214	0.765	0.045	0
Spc24	0	5.54961343	0.719	0.017	0
Ube2c	0	5.15889026	0.699	0.027	0
Cdca8	0	5.26460202	0.686	0.018	0
Kif11	0	5.08386879	0.667	0.017	0
Ccna2	0	6.21346898	0.569	0.008	0
Ncapd2	0	4.22615611	0.556	0.023	0
Hist1h2ab	0	5.81986262	0.542	0.009	0
Clspn	0	4.34900221	0.542	0.019	0
Cdca3	0	5.1366471	0.523	0.013	0
Tyms	0	4.69766399	0.497	0.02	0
Kif15	0	5.36466718	0.484	0.011	0
Hist1h3i	0	4.48224872	0.49	0.017	0
Tacc3	0	4.6951139	0.477	0.015	0
Diaph3	0	4.23908465	0.477	0.019	0
Hist1h2bj	0	4.87245818	0.458	0.014	0
Hist1h1a	0	4.70892312	0.438	0.013	0
Cdk1	0	4.91742895	0.431	0.012	0
Knl1	0	4.60060845	0.418	0.013	0
Tpx2	0	4.93755309	0.412	0.013	0
Ccnb2	0	5.00912539	0.405	0.011	0
Hist1h2af	0	6.18643973	0.386	0.005	0
Cit	0	5.19038599	0.386	0.009	0
Fbxo5	0	4.88992502	0.386	0.012	0
Hist1h2ak	0	4.56985282	0.373	0.011	0
Rad51ap1	0	5.76019509	0.366	0.005	0
Kif4	0	4.96455067	0.359	0.009	0
Rrm2	0	4.64022572	0.346	0.01	0
Cenpm	0	4.72075482	0.346	0.011	0
Aurkb	0	5.43666952	0.327	0.006	0
Hist1h2bb	0	4.92754294	0.307	0.007	0
Esco2	0	5.50203164	0.275	0.005	0
Hist1h2bm	0	5.09087686	0.261	0.005	0
Cep55	0	5.18604212	0.248	0.005	0
Neil3	0	6.04562714	0.242	0.004	0

Hmmr	0	5.30187735	0.242	0.005	0
Mcm10	0	5.21567382	0.203	0.003	0
Cdca5	0	5.43065208	0.203	0.004	0
Hist1h3b	0	6.18292685	0.163	0.002	0
Cenpf	1.299E-307	4.48982953	0.392	0.015	2.85E-303
Sgo1	3.143E-306	5.10609392	0.229	0.005	6.896E-302
Ttk	1.078E-301	5.65693037	0.15	0.002	2.365E-297
Cd8a	2.193E-296	3.18487887	0.876	0.074	4.81E-292
Mki67	6.337E-295	4.27802664	0.922	0.093	1.39E-290
Shcbp1	1.205E-292	4.88565149	0.275	0.007	2.643E-288
Racgap1	1.989E-286	3.8738057	0.516	0.027	4.363E-282
Hist1h2bn	4.245E-283	4.89582023	0.261	0.007	9.313E-279
Gmnn	3.806E-279	3.87499661	0.739	0.058	8.351E-275
Hist2h2bb	1.635E-278	3.85966427	0.471	0.023	3.588E-274
Ska1	4.032E-277	5.16461935	0.203	0.004	8.845E-273
Brip1	1.792E-275	4.85002687	0.229	0.005	3.932E-271
Nuf2	7.455E-266	5.28112961	0.163	0.003	1.635E-261
H2afx	5.016E-265	3.68949091	0.817	0.074	1.1E-260
Mybl2	8.282E-264	5.24583592	0.19	0.004	1.817E-259
Lockd	2.284E-252	3.67709162	0.588	0.039	5.011E-248
Kif2c	1.082E-251	5.8818016	0.131	0.002	2.375E-247
Ncapg	1.838E-249	4.67369854	0.268	0.008	4.033E-245
Kif18b	1.241E-246	6.26551546	0.118	0.001	2.722E-242
Uhrf1	2.082E-243	4.23098815	0.366	0.016	4.568E-239
Asf1b	3.379E-243	4.42134636	0.314	0.012	7.413E-239
Mcm5	3.773E-242	3.74012104	0.562	0.038	8.278E-238
Fignl1	2.311E-238	4.93949743	0.196	0.005	5.07E-234
Cdkn3	9.838E-237	4.83024877	0.209	0.005	2.158E-232
Cdc45	1.383E-235	4.45606025	0.255	0.008	3.034E-231
Hist1h3e	5.464E-233	3.8551861	0.719	0.065	1.199E-228
Bard1	1.528E-231	4.36557918	0.255	0.008	3.352E-227
Mtfr2	1.942E-231	5.66929822	0.131	0.002	4.261E-227
Dlgap5	5.983E-228	4.24173935	0.314	0.013	1.313E-223
Spag5	3.149E-227	4.64502928	0.216	0.006	6.908E-223
Hist1h3f	5.77E-227	3.87118341	0.399	0.02	1.266E-222
Rad51	3.615E-226	4.70790974	0.235	0.007	7.93E-222
Hist1h1d	1.385E-225	3.51977285	0.725	0.066	3.038E-221
Cenpe	2.855E-224	4.4652454	0.314	0.013	6.264E-220
Dut	2.707E-223	3.91959458	0.876	0.108	5.939E-219
Cenpw	2.789E-221	3.23582636	0.706	0.064	6.119E-217
Incenp	2.504E-220	3.69105746	0.529	0.036	5.493E-216
Cenpp	2.222E-213	5.11001761	0.216	0.006	4.874E-209
Trip13	5.216E-211	4.56803207	0.203	0.006	1.144E-206
Hist2h2ac	2.099E-208	3.7691357	0.699	0.069	4.605E-204
Dtl	3.835E-205	3.74051258	0.405	0.023	8.414E-201
Depdc1a	6.009E-204	4.77746816	0.183	0.005	1.318E-199
Lig1	4.975E-201	3.44888931	0.641	0.059	1.091E-196
Sgo2a	5.455E-200	4.55072308	0.183	0.005	1.197E-195

Bub1	2.196E-198	5.20213602	0.183	0.005	4.818E-194
Aspm	3.422E-195	4.90146169	0.196	0.006	7.507E-191
Ccdc34	1.811E-192	3.53170118	0.542	0.044	3.973E-188
Gzmk	3.996E-191	3.95521609	0.301	0.014	8.767E-187
Cdc6	8.573E-188	5.08095996	0.19	0.006	1.881E-183
Cenph	5.672E-187	4.79656908	0.19	0.006	1.244E-182
Kif22	1.513E-186	4.88285648	0.183	0.005	3.318E-182
Ndc80	2.598E-185	3.77376572	0.288	0.013	5.7E-181
Hist1h2bh	4.274E-180	4.61423536	0.163	0.004	9.376E-176
Pbk	2.381E-179	5.40062092	0.15	0.004	5.223E-175
Chaf1a	6.671E-179	4.22274319	0.242	0.01	1.464E-174
Prc1	2.751E-178	3.218679	0.458	0.033	6.036E-174
Foxm1	6.946E-176	4.55123384	0.196	0.006	1.524E-171
Stil	7.514E-176	4.7303718	0.163	0.004	1.648E-171
Ncaph	1.468E-174	3.92066469	0.307	0.016	3.221E-170
E2f8	2.392E-174	4.59964232	0.216	0.008	5.248E-170
Ncapg2	3.397E-173	3.49961177	0.549	0.05	7.452E-169
Ckap2	8.36E-173	4.22044082	0.209	0.007	1.834E-168
Hist1h2ai	1.613E-172	5.48450601	0.105	0.002	3.538E-168
Ulbp1	1.262E-168	4.02192019	0.229	0.009	2.768E-164
Hist1h4h	1.534E-168	3.4652622	0.458	0.035	3.365E-164
Cenpk	1.025E-165	3.67829376	0.314	0.017	2.249E-161
Prim1	2.008E-164	3.62449782	0.32	0.018	4.406E-160
Mcm7	1.197E-161	3.3094083	0.523	0.047	2.626E-157
Spc25	2.184E-161	4.01699481	0.229	0.009	4.793E-157
Mastl	6.428E-157	4.29133425	0.183	0.006	1.41E-152
Cenpn	2.375E-155	4.87810637	0.144	0.004	5.212E-151
Gzmb	7.895E-152	2.48461286	0.719	0.092	1.732E-147
Mcm3	1.06E-151	3.2858025	0.608	0.069	2.325E-147
Rgs16	3.274E-151	3.80829647	0.301	0.017	7.183E-147
Nkg7	1.811E-150	3.00599369	0.993	0.223	3.972E-146
Lrr1	1.107E-148	6.09002858	0.065	0.001	2.429E-144
Kntc1	3.142E-148	4.95032987	0.118	0.003	6.892E-144
Ncapd3	7.421E-146	2.86073233	0.549	0.056	1.628E-141
Rfc3	8.166E-144	3.06543451	0.458	0.04	1.791E-139
Pimreg	8.751E-144	5.27320882	0.078	0.001	1.92E-139
Pdcd1	1.179E-143	2.77403755	0.647	0.079	2.586E-139
Hist1h2an	8.281E-142	4.6720655	0.137	0.004	1.817E-137
Hells	2.963E-141	2.92642544	0.575	0.063	6.5E-137
Kif14	5.219E-140	5.81898121	0.072	0.001	1.145E-135
Knstrn	6.156E-140	3.88212419	0.248	0.013	1.35E-135
Rad54l	5.05E-137	4.98495635	0.137	0.004	1.108E-132
Mcm6	5.962E-137	2.60963125	0.699	0.094	1.308E-132
Hmgn2	1.843E-136	2.99190902	0.843	0.155	4.043E-132
Tuba1b	5.793E-135	3.28422499	0.961	0.249	1.271E-130
Ticrr	2.376E-134	4.23529848	0.157	0.005	5.213E-130
Tk1	2.922E-134	3.99202175	0.235	0.012	6.41E-130
Chek1	5.097E-132	4.29744343	0.15	0.005	1.118E-127

Nrm	7.657E-131	2.91081245	0.458	0.044	1.68E-126
Cdc25c	1.002E-127	5.29484778	0.072	0.001	2.199E-123
Ska3	2.499E-127	4.34733002	0.137	0.004	5.482E-123
H2afv	8.137E-127	3.08720917	0.961	0.284	1.785E-122
Ckap2l	2.835E-125	3.83166815	0.222	0.011	6.22E-121
E2f7	3.241E-125	4.63643255	0.118	0.003	7.111E-121
Brca1	5.241E-125	4.1398197	0.183	0.008	1.15E-120
Pola1	2.307E-124	2.89949345	0.431	0.041	5.061E-120
Ezh2	3.285E-124	2.91888384	0.647	0.092	7.206E-120
Tcf19	4.661E-122	4.21366872	0.163	0.006	1.023E-117
Rpa2	3.788E-121	2.7702335	0.549	0.065	8.31E-117
Hist1h4d	3.932E-121	3.15501734	0.922	0.222	8.627E-117
Bub1b	7.753E-119	3.51004822	0.235	0.013	1.701E-114
Tubb5	6.661E-117	3.25825601	0.993	0.384	1.461E-112
Hist1h1e	4.182E-116	2.94844482	0.922	0.241	9.175E-112
Arhgap11a	1.117E-115	3.02580739	0.373	0.033	2.45E-111
Cd160	8.574E-115	3.00603755	0.314	0.024	1.881E-110
Ccnb1	1.884E-114	4.47706198	0.131	0.004	4.133E-110
Nsl1	3.354E-114	4.59373144	0.144	0.005	7.357E-110
Zwilch	3.358E-114	4.05518337	0.137	0.005	7.367E-110
1500009L16Ri	1.42E-113	3.87619458	0.183	0.009	3.116E-109
Dnmt1	4.609E-113	2.7928263	0.542	0.069	1.011E-108
Dnajc9	2.757E-112	2.28358417	0.719	0.114	6.048E-108
Selenoh	3.709E-112	2.69914828	0.765	0.143	8.137E-108
Hist1h2bl	3.422E-111	4.83840602	0.078	0.002	7.508E-107
Lsm2	6.442E-111	2.55916755	0.614	0.089	1.413E-106
Serp2	8.854E-110	3.99454641	0.163	0.007	1.942E-105
Ctsw	5.425E-108	2.21763976	0.797	0.144	1.19E-103
Depdc1b	2.679E-107	5.38350272	0.059	0.001	5.877E-103
Topbp1	6.153E-107	2.4506133	0.621	0.093	1.35E-102
Kifc1	3.608E-105	4.54068686	0.098	0.003	7.916E-101
Haus4	1.137E-104	3.01033645	0.288	0.022	2.493E-100
Gtse1	1.234E-104	5.0129402	0.085	0.002	2.706E-100
Mcm4	1.478E-104	2.49983904	0.627	0.096	3.242E-100
Pmf1	3.31E-104	2.50903098	0.503	0.063	7.262E-100
Plk1	4.802E-104	4.91004735	0.105	0.003	1.053E-99
Rnaseh2b	3.8E-103	3.04537201	0.366	0.036	8.337E-99
Kif20a	1.633E-102	4.48694463	0.105	0.003	3.582E-98
Hist1h2ag	2.543E-102	5.22991607	0.072	0.001	5.5784E-98
Rrm1	2.666E-102	2.8635078	0.471	0.057	5.8494E-98
Hist1h2be	1.794E-99	2.81316688	0.366	0.036	3.9361E-95
Fancb	3.394E-99	4.25876581	0.085	0.002	7.4456E-95
Gzma	5.2441E-98	3.25033295	0.373	0.039	1.1505E-93
Pole	5.9646E-98	3.79332769	0.15	0.007	1.3086E-93
Cdc25b	7.1281E-98	3.11830108	0.301	0.026	1.5638E-93
Plk4	1.1716E-97	2.54529716	0.458	0.056	2.5704E-93
Rfc5	4.7138E-97	3.42879177	0.288	0.024	1.0342E-92
Phgdh	1.6849E-96	2.49933803	0.444	0.053	3.6965E-92

Pkmyt1	2.365E-96	3.32402741	0.203	0.012	5.1885E-92
Atad5	7.7422E-96	2.97576579	0.314	0.028	1.6986E-91
Melk	2.7306E-94	4.35599944	0.124	0.005	5.9907E-90
Rpa3	1.1062E-93	2.39160771	0.438	0.053	2.427E-89
Hist2h2aa1	5.6757E-93	2.81720041	0.346	0.035	1.2452E-88
Sla2	1.9277E-92	2.62827121	0.379	0.041	4.2291E-88
Mxd3	1.9572E-92	5.13211128	0.072	0.002	4.294E-88
Hmgb2	1.2173E-91	2.55698772	1	0.721	2.6706E-87
Hjurp	1.7299E-91	2.48431275	0.549	0.083	3.7953E-87
Orc6	3.5325E-91	2.89580934	0.425	0.053	7.7499E-87
Tipin	1.6343E-90	3.12453393	0.307	0.029	3.5855E-86
Tmpo	3.3471E-90	2.28351504	0.784	0.171	7.3431E-86
Dscc1	3.8253E-90	4.67465362	0.098	0.003	8.3923E-86
Hmgb3	1.6863E-89	2.90067504	0.281	0.024	3.6996E-85
Aunip	3.375E-89	6.0293311	0.046	0.001	7.4044E-85
Mad2l1	2.0926E-88	2.98991681	0.261	0.021	4.591E-84
Cdc20	3.5373E-88	3.8425574	0.137	0.006	7.7604E-84
Brca2	6.6425E-88	3.45479573	0.163	0.009	1.4573E-83
Traip	3.7549E-87	4.53540352	0.111	0.004	8.2378E-83
Ms4a4b	6.7327E-87	2.09082714	0.922	0.25	1.4771E-82
4930579G24R	1.6219E-86	3.65663364	0.137	0.006	3.5584E-82
Cdkn2a	2.1471E-86	3.79026256	0.144	0.007	4.7106E-82
Ptprcap	4.4228E-85	2.03895064	0.85	0.203	9.7033E-81
Sult2b1	5.6845E-85	3.18880352	0.261	0.022	1.2471E-80
Wdr76	9.9568E-85	3.00412849	0.235	0.018	2.1844E-80
Cd226	1.4469E-84	2.28773429	0.405	0.049	3.1744E-80
Ccne1	2.8158E-84	4.16051694	0.105	0.004	6.1775E-80
Mrpl18	3.48E-84	2.11553223	0.758	0.165	7.6347E-80
1700097N02R	1.4507E-83	3.34825065	0.203	0.014	3.1827E-79
Ncald	4.6097E-82	2.62668768	0.333	0.036	1.0113E-77
Cmc2	1.2364E-81	2.18694203	0.503	0.077	2.7126E-77
Fen1	6.1719E-81	2.62643523	0.34	0.038	1.3541E-76
Cd3d	6.83E-81	1.91645986	0.98	0.317	1.4984E-76
Cbx5	2.2123E-80	2.49209162	0.431	0.059	4.8536E-76
Espl1	6.4582E-80	3.80875183	0.144	0.008	1.4169E-75
Exosc8	9.5043E-80	2.07590068	0.608	0.11	2.0851E-75
E2f1	1.2962E-78	3.45861185	0.216	0.017	2.8438E-74
Dctpp1	2.1926E-78	2.73191937	0.425	0.061	4.8103E-74
Hist3h2ba	6.1379E-78	2.74215524	0.242	0.021	1.3466E-73
Klrc1	1.2755E-76	1.91238582	0.582	0.103	2.7984E-72
Gm19585	2.1318E-76	1.96350578	0.693	0.142	4.6769E-72
Rfc4	2.9284E-76	2.97176118	0.268	0.026	6.4246E-72
Hmgb1	1.7923E-75	1.93845268	0.987	0.663	3.9321E-71
Pask	1.5022E-74	4.91352285	0.059	0.001	3.2956E-70
AW112010	1.6327E-74	2.11676272	0.98	0.465	3.5819E-70
Alyref	1.2531E-73	2.02516153	0.81	0.22	2.7493E-69
Rbl1	1.2567E-73	2.48601935	0.294	0.031	2.7572E-69
Mis18bp1	1.3531E-73	3.68713426	0.157	0.01	2.9685E-69

Ube2s	1.625E-73	2.15939229	0.961	0.467	3.5651E-69
Smc4	1.91E-73	2.07246308	0.752	0.184	4.1903E-69
Ccl5	1.0145E-72	1.57085754	0.882	0.258	2.2258E-68
Cd3e	1.3024E-72	1.69661449	0.961	0.293	2.8573E-68
Dbf4	4.1149E-72	2.07653557	0.549	0.099	9.0278E-68
Cbx3	1.2573E-71	1.98909858	0.889	0.283	2.7583E-67
Rdm1	1.6444E-71	2.98678229	0.209	0.017	3.6076E-67
Banf1	6.3917E-71	1.97741549	0.81	0.223	1.4023E-66
Sh2d2a	7.3626E-71	1.76820587	0.935	0.3	1.6153E-66
Haspin	1.3662E-70	3.58682493	0.131	0.007	2.9973E-66
Ran	1.5042E-70	1.96245119	0.954	0.426	3.3001E-66
Mcm2	5.9627E-70	2.37726677	0.314	0.037	1.3082E-65
Fancd2	9.7298E-70	4.23286602	0.085	0.003	2.1346E-65
Siva1	7.2826E-69	2.17112278	0.634	0.142	1.5977E-64
Lck	1.1972E-68	1.60499466	0.824	0.2	2.6266E-64
Trbc2	2.6839E-68	1.79953179	0.98	0.387	5.8882E-64
Ube2t	3.0212E-68	2.54592425	0.235	0.022	6.6282E-64
Exo1	1.5044E-67	4.3133115	0.059	0.001	3.3005E-63
2610318N02R	2.1352E-67	4.17928913	0.098	0.004	4.6844E-63
Dek	8.0685E-67	1.97797106	0.967	0.489	1.7702E-62
Lin9	6.7365E-66	2.36877661	0.268	0.029	1.4779E-61
Tubb4b	8.3287E-66	2.07374023	0.614	0.134	1.8272E-61
Anp32b	6.2681E-65	1.83322834	0.974	0.459	1.3752E-60
Lsm3	1.3466E-64	1.78461369	0.68	0.158	2.9543E-60
Mms22l	2.8796E-64	2.75054143	0.235	0.023	6.3176E-60
Ppia	6.6985E-64	1.3078249	0.993	0.916	1.4696E-59
Kif20b	7.386E-64	2.56935113	0.222	0.021	1.6204E-59
Ptma	1.0306E-63	1.34600886	1	0.942	2.2609E-59
Cd3g	3.7817E-63	1.58650391	0.954	0.316	8.2966E-59
Ddx39	3.8352E-62	2.25445695	0.464	0.084	8.4141E-58
Parpbp	4.6142E-62	4.60617127	0.052	0.001	1.0123E-57
C330027C09F	5.41E-62	3.71719557	0.137	0.009	1.1869E-57
Dhfr	6.786E-62	2.95417844	0.183	0.015	1.4888E-57
Lgals1	2.8445E-61	2.00615827	0.974	0.513	6.2406E-57
Ska2	9.4463E-61	3.6802643	0.118	0.007	2.0724E-56
Anp32e	1.4179E-60	1.87035105	0.869	0.3	3.1106E-56
Cks2	2.0105E-60	1.34840595	0.68	0.16	4.4109E-56
Metrn	9.2004E-60	2.51214314	0.255	0.029	2.0185E-55
Dtymk	1.6235E-59	2.22578466	0.373	0.058	3.5619E-55
Smc1a	2.3126E-59	1.88046324	0.791	0.241	5.0737E-55
Blm	2.0972E-58	2.26659145	0.294	0.038	4.601E-54
Lsm5	3.1521E-58	1.78464345	0.863	0.299	6.9155E-54
Nelfe	3.5484E-58	2.12188619	0.405	0.069	7.7849E-54
Cst7	4.4506E-58	1.93437209	0.379	0.059	9.7643E-54
Atad2	4.5464E-58	1.8833549	0.68	0.173	9.9743E-54
Kif18a	5.9063E-58	2.72193071	0.17	0.014	1.2958E-53
Hist1h3g	1.1269E-57	5.6844307	0.046	0.001	2.4724E-53
Trac	5.2276E-57	1.55013656	0.817	0.232	1.1469E-52

Hpf1	5.8913E-57	1.75157669	0.706	0.188	1.2925E-52
Pcna	8.2726E-57	1.98165886	0.569	0.129	1.8149E-52
Nsd2	1.0897E-56	1.94228648	0.542	0.117	2.3908E-52
Cd7	6.0722E-56	2.09775139	0.405	0.07	1.3322E-51
Prr11	7.3999E-56	4.49240014	0.072	0.003	1.6235E-51
Cdca2	8.5754E-56	3.98202104	0.085	0.004	1.8814E-51
Trav16	1.0683E-55	4.11497534	0.065	0.002	2.3437E-51
Hist1h4k	2.0482E-55	4.09441281	0.072	0.003	4.4936E-51
Hist1h3d	2.3537E-55	3.0428276	0.124	0.008	5.1638E-51
Gstp3	2.9983E-55	2.10712353	0.346	0.053	6.578E-51
Usp1	4.7744E-55	1.86246957	0.556	0.124	1.0475E-50
Nans	1.3764E-54	1.98937966	0.471	0.093	3.0197E-50
Anapc5	1.4171E-54	1.91641533	0.575	0.136	3.109E-50
Jpt1	2.3632E-54	1.66632651	0.928	0.385	5.1847E-50
Gins2	3.5516E-54	3.12007853	0.17	0.015	7.7918E-50
Zdhhc2	4.2142E-54	2.1161055	0.301	0.041	9.2454E-50
Sh2d1a	4.5524E-54	1.75331357	0.438	0.079	9.9874E-50
Serpinb6b	6.0161E-54	1.24332706	0.804	0.233	1.3199E-49
Gins4	8.6716E-54	2.15318575	0.333	0.051	1.9025E-49
Hist1h3a	2.1197E-53	2.35625414	0.235	0.027	4.6505E-49
E2f2	3.8791E-53	2.7177123	0.19	0.019	8.5104E-49
Rad21	4.1357E-53	1.44588253	0.843	0.264	9.0732E-49
Pfn1	5.8995E-53	1.41053849	1	0.753	1.2943E-48
Fam92a	1.3146E-52	2.15455269	0.294	0.041	2.8841E-48
Rnaseh2c	2.7086E-52	1.80784571	0.601	0.148	5.9424E-48
Hist1h2bp	3.5741E-52	4.06945868	0.059	0.002	7.8413E-48
Dsn1	4.0686E-52	2.97737827	0.124	0.009	8.9261E-48
Mnd1	4.8836E-52	4.23967944	0.072	0.003	1.0714E-47
Etfb	7.0827E-52	1.76662138	0.621	0.161	1.5539E-47
Clic1	9.4777E-52	1.62618579	0.928	0.443	2.0793E-47
Nasp	1.2325E-51	1.66988156	0.686	0.19	2.7039E-47
Hist1h4c	2.2813E-51	2.2236096	0.327	0.051	5.0049E-47
4930415O20R	2.306E-51	6.59519869	0.013	0	5.0591E-47
H2afz	2.7727E-51	1.27589491	1	0.726	6.0829E-47
Nxt1	3.4962E-51	2.10801444	0.379	0.067	7.6704E-47
Klrd1	5.6828E-51	1.89881753	0.477	0.101	1.2468E-46
Hist1h4i	6.2773E-51	1.90230399	0.399	0.073	1.3772E-46
Cenpi	6.7224E-51	3.73790136	0.078	0.004	1.4748E-46
Recql4	6.7816E-51	4.33218315	0.065	0.003	1.4878E-46
Zbtb45	1.5457E-50	2.8134956	0.144	0.012	3.3911E-46
Hint1	5.0111E-50	1.40834999	0.967	0.575	1.0994E-45
Dnajc15	6.6666E-50	1.92794542	0.667	0.193	1.4626E-45
Rbm3	1.1389E-49	1.16775301	1	0.789	2.4986E-45
Nudt21	1.1907E-49	1.62722808	0.719	0.214	2.6122E-45
Hirip3	1.4053E-49	2.17565825	0.248	0.032	3.083E-45
Lsm6	2.7075E-49	1.60946183	0.712	0.209	5.9399E-45
Snrpe	6.8491E-49	1.37874695	0.974	0.611	1.5026E-44
Casp3	1.6605E-48	1.85913183	0.49	0.109	3.643E-44

Cox5b	2.0934E-48	1.37028109	0.967	0.565	4.5927E-44
Snrnp25	4.7772E-48	2.07329752	0.34	0.058	1.0481E-43
Chaf1b	4.8687E-48	2.73249171	0.163	0.015	1.0681E-43
Elob	6.6516E-48	1.32492072	0.967	0.579	1.4593E-43
Slbp	7.4737E-48	1.71746402	0.745	0.246	1.6397E-43
Clgn	8.0943E-48	4.67642348	0.052	0.002	1.7758E-43
Gimap7	1.0358E-47	1.47249634	0.673	0.181	2.2724E-43
Mis18a	2.1785E-47	2.07677892	0.333	0.056	4.7794E-43
Tnfrsf9	3.2908E-47	1.28433887	0.562	0.137	7.2198E-43
Mlf1	3.3631E-47	3.36494715	0.105	0.007	7.3783E-43
Calm3	3.4614E-47	1.45800588	0.869	0.33	7.5939E-43
Fsbp	6.0556E-47	5.50273554	0.033	0.001	1.3285E-42
Tubg1	1.8683E-46	3.09573202	0.124	0.009	4.0988E-42
Lsm1	3.4093E-46	1.78140875	0.399	0.077	7.4797E-42
Rfc1	4.075E-46	1.78509046	0.412	0.083	8.9401E-42
Snrpd1	1.221E-45	1.50150615	0.856	0.336	2.6787E-41
Skap1	2.7373E-45	1.21480842	0.778	0.226	6.0053E-41
Gins3	2.8803E-45	3.52407618	0.105	0.007	6.3192E-41
Ndufb7	3.3141E-45	1.53311324	0.895	0.376	7.2708E-41
Thy1	4.2961E-45	1.2765616	0.791	0.246	9.4252E-41
Txn1	6.8745E-45	1.21989105	0.961	0.557	1.5082E-40
Med30	7.2439E-45	1.83109537	0.49	0.116	1.5892E-40
Etaa1	8.7541E-45	1.67672668	0.346	0.061	1.9206E-40
Rfc2	8.873E-45	1.84084352	0.392	0.078	1.9467E-40
Pole3	9.9837E-45	1.95568862	0.359	0.067	2.1903E-40
Anapc11	1.0576E-44	1.74738561	0.523	0.13	2.3202E-40
Rangap1	1.0579E-44	1.83348478	0.477	0.111	2.3209E-40
Themis	1.1254E-44	1.39107999	0.471	0.1	2.469E-40
Cep72	1.6021E-44	3.33141629	0.085	0.005	3.5149E-40
Wdr95	2.7458E-44	3.17163085	0.111	0.008	6.024E-40
Erfe	3.7049E-44	6.4456939	0.013	0	8.1282E-40
Kif24	7.265E-44	4.7064599	0.039	0.001	1.5939E-39
Coq7	9.3006E-44	2.47092593	0.203	0.025	2.0405E-39
Kif23	1.7956E-43	1.71539305	0.32	0.055	3.9394E-39
Tmem97	2.1144E-43	2.64342816	0.222	0.03	4.6387E-39
Cdc7	2.2421E-43	2.25703788	0.17	0.018	4.919E-39
Sh3bgrl3	2.2537E-43	1.18095861	0.993	0.711	4.9443E-39
Myb	3.1719E-43	1.60889459	0.288	0.046	6.9587E-39
Arl6ip1	3.936E-43	1.52201314	0.961	0.529	8.6352E-39
Hist1h2ac	4.1178E-43	2.37421922	0.163	0.017	9.034E-39
Tfdp1	5.0568E-43	2.3170223	0.222	0.03	1.1094E-38
Ect2	5.3935E-43	3.39533929	0.092	0.006	1.1833E-38
Ppp1ca	5.4635E-43	1.41967608	0.915	0.435	1.1986E-38
Lsm8	6.8608E-43	1.30867366	0.641	0.177	1.5052E-38
Bex3	7.9403E-43	1.36766513	0.686	0.202	1.742E-38
Ncaph2	1.107E-42	1.72358944	0.444	0.1	2.4287E-38
Cenps	1.2866E-42	2.46710944	0.144	0.013	2.8227E-38
Nup85	1.3126E-42	2.21228043	0.222	0.03	2.8797E-38

Dbi	2.0395E-42	1.48573024	0.817	0.316	4.4744E-38
Fancf	2.2216E-42	2.19337942	0.229	0.031	4.874E-38
Nek2	2.4236E-42	3.91068244	0.072	0.004	5.3171E-38
Gramd3	2.8431E-42	1.26852305	0.797	0.267	6.2374E-38
Psmb9	3.7752E-42	1.3798004	0.824	0.315	8.2825E-38
Smc3	4.0414E-42	1.56416452	0.68	0.218	8.8663E-38
Cox8a	5.0031E-42	1.00312236	0.987	0.81	1.0976E-37
Hmgn5	7.5062E-42	1.50607328	0.484	0.115	1.6468E-37
Mns1	8.2404E-42	2.30216118	0.176	0.02	1.8079E-37
S100a10	9.626E-42	1.34603277	0.974	0.558	2.1118E-37
Gins1	1.2468E-41	3.66865623	0.078	0.004	2.7355E-37
Coro1a	1.7845E-41	1.27380575	0.993	0.609	3.915E-37
Atp5j	2.3711E-41	1.41424183	0.889	0.462	5.2019E-37
Hspa14	2.7388E-41	1.51497235	0.51	0.126	6.0086E-37
Ndufa11	3.1493E-41	1.49797681	0.784	0.288	6.9093E-37
Mthfd2	3.3526E-41	1.415801	0.667	0.202	7.3553E-37
Abcb9	6.6419E-41	1.98090715	0.216	0.029	1.4572E-36
Mdrl	9.3493E-41	4.9479555	0.033	0.001	2.0511E-36
Mrpl12	1.183E-40	1.45659002	0.536	0.14	2.5954E-36
Cox7a2	1.2564E-40	1.22557852	0.961	0.582	2.7565E-36
Mrpl58	1.7651E-40	1.5854318	0.484	0.119	3.8724E-36
Abracl	1.768E-40	1.34788372	0.941	0.487	3.8788E-36
Cdca7	1.8084E-40	2.65167621	0.144	0.014	3.9674E-36
Gldc	2.655E-40	3.04198958	0.098	0.007	5.8249E-36
Hist1h2br	2.6758E-40	3.12545221	0.105	0.008	5.8704E-36
Cenpx	3.9532E-40	1.7130769	0.523	0.142	8.6729E-36
Lsm4	4.7088E-40	1.38532514	0.824	0.326	1.0331E-35
Anln	5.5031E-40	3.63343341	0.078	0.005	1.2073E-35
Supt16	5.9164E-40	1.31962887	0.686	0.213	1.298E-35
Ranbp1	7.2858E-40	1.55999816	0.752	0.276	1.5984E-35
Lat	9.3988E-40	1.06111661	0.758	0.229	2.062E-35
Zfp367	9.4563E-40	2.46925198	0.183	0.022	2.0746E-35
Ccdc30	1.093E-39	2.72444984	0.137	0.013	2.3979E-35
Sumo2	1.412E-39	1.39479376	0.922	0.536	3.0978E-35
Psat1	1.8749E-39	2.16313695	0.248	0.039	4.1134E-35
Gpr160	1.9931E-39	2.85657402	0.131	0.012	4.3727E-35
Cenpq	3.607E-39	1.39751519	0.405	0.087	7.9134E-35
Cdk4	4.2248E-39	1.79758334	0.392	0.087	9.2687E-35
Syce2	1.6846E-38	2.48314925	0.203	0.028	3.6959E-34
Iqgap3	2.3269E-38	4.09937534	0.059	0.003	5.1051E-34
Perp	2.4527E-38	2.65072757	0.111	0.009	5.3809E-34
Reep5	2.5524E-38	1.27344703	0.902	0.404	5.5998E-34
Trav13n-4	2.6104E-38	4.99189886	0.02	0	5.7271E-34
Trbv19	3.3869E-38	3.60325145	0.065	0.003	7.4305E-34
Dpy30	3.7641E-38	1.94024057	0.379	0.084	8.2581E-34
Nucks1	3.8382E-38	1.41339769	0.882	0.433	8.4207E-34
Pglyrp1	4.1379E-38	1.26649568	0.353	0.071	9.078E-34
Klrc2	4.2729E-38	1.51896661	0.34	0.067	9.3744E-34

Ybx3	4.3768E-38	1.32015811	0.791	0.294	9.6022E-34
Atp5o.1	4.6973E-38	1.33401839	0.856	0.366	1.0305E-33
Nsmaf	8.4851E-38	1.6317142	0.477	0.122	1.8615E-33
Cdk2	1.1643E-37	2.34679176	0.19	0.025	2.5543E-33
Psmb3	2.0072E-37	1.33156732	0.83	0.338	4.4037E-33
Gapdh	5.01E-37	0.89817931	0.993	0.745	1.0991E-32
Cenpv	5.1351E-37	2.80541524	0.15	0.017	1.1266E-32
Psip1	5.2176E-37	1.46732851	0.529	0.145	1.1447E-32
Nubp1	5.3785E-37	1.74622953	0.458	0.118	1.18E-32
Bend4	8.7085E-37	1.90477742	0.281	0.051	1.9105E-32
Tceal9	2.5183E-36	1.22484959	0.614	0.188	5.5249E-32
Actb	2.8E-36	0.88572972	1	0.977	6.143E-32
Fasl	3.801E-36	1.22137775	0.438	0.102	8.3391E-32
Mrpl40	5.3271E-36	1.70722396	0.327	0.066	1.1687E-31
Nt5c3b	5.7325E-36	2.05409765	0.222	0.034	1.2577E-31
Rab19	6.3103E-36	1.88297659	0.222	0.034	1.3844E-31
Oaz1	6.8371E-36	0.87477363	0.98	0.833	1.5E-31
Crip1	6.8714E-36	1.00796211	0.993	0.651	1.5075E-31
Mrps36	8.1376E-36	1.49738009	0.458	0.118	1.7853E-31
Ctla2a	1.3602E-35	1.15564248	0.575	0.169	2.9842E-31
Commd1	1.4037E-35	1.95486742	0.405	0.101	3.0795E-31
Rbbp7	1.9846E-35	1.41623723	0.588	0.182	4.3541E-31
Naa38	2.3576E-35	1.50582057	0.477	0.128	5.1724E-31
Pla2g16	2.3836E-35	1.23210353	0.614	0.187	5.2295E-31
Trbv29	2.463E-35	4.02205464	0.052	0.002	5.4035E-31
Casp8ap2	3.2512E-35	1.39249908	0.529	0.148	7.1328E-31
Zmynd19	3.6095E-35	2.20816947	0.183	0.025	7.9189E-31
Mrps18c	4.3414E-35	1.28433458	0.621	0.196	9.5245E-31
Prelid1	5.8895E-35	1.29450852	0.771	0.302	1.2921E-30
Nsmce4a	6.286E-35	1.24413613	0.582	0.172	1.3791E-30
Ssrp1	6.5492E-35	1.3154599	0.601	0.185	1.4368E-30
1810037I17Ril	1.1891E-34	1.23450657	0.908	0.459	2.6087E-30
Cyc1	1.4495E-34	1.412648	0.529	0.154	3.1801E-30
Reep4	1.6432E-34	2.21901922	0.248	0.043	3.6049E-30
Fxn	2.3518E-34	2.18687021	0.222	0.036	5.1597E-30
Pold2	3.098E-34	2.23844301	0.176	0.024	6.7966E-30
Ndufa4	3.1275E-34	1.28979148	0.882	0.468	6.8614E-30
Atpif1	4.6938E-34	1.14726492	0.83	0.341	1.0298E-29
Bub3	6.3346E-34	1.47324148	0.529	0.156	1.3897E-29
Lbr	7.0237E-34	1.1815301	0.712	0.247	1.5409E-29
Higd1a	7.5816E-34	1.16459804	0.791	0.31	1.6633E-29
Pttg1	1.053E-33	1.42982254	0.471	0.126	2.3102E-29
Mt3	1.1104E-33	3.22973628	0.078	0.005	2.4362E-29
Rac2	1.2269E-33	1.06141722	0.948	0.579	2.6916E-29
Serpinb9	1.7232E-33	0.793826	0.745	0.27	3.7806E-29
Pcnt	1.8631E-33	1.48813637	0.418	0.105	4.0875E-29
Slc43a3	2.1918E-33	2.46134458	0.124	0.013	4.8086E-29
Cmtm7	2.3491E-33	1.61275839	0.608	0.212	5.1538E-29

Emc8	2.7889E-33	1.72324118	0.268	0.05	6.1185E-29
Ms4a6b	3.6524E-33	1.07645687	0.719	0.255	8.0129E-29
Nudt5	6.5373E-33	1.75285178	0.275	0.053	1.4342E-28
Trav16n	6.8225E-33	3.47565507	0.065	0.004	1.4968E-28
Trbc1	7.3694E-33	1.70301795	0.654	0.263	1.6168E-28
Eomes	9.7216E-33	1.81006818	0.176	0.024	2.1328E-28
Dcps	1.2297E-32	1.91895221	0.268	0.051	2.6979E-28
Tonsl	1.2307E-32	3.44114201	0.072	0.005	2.6999E-28
Maz	1.29E-32	1.48522692	0.359	0.082	2.8302E-28
Rnf168	1.4225E-32	1.73934496	0.261	0.048	3.1208E-28
Rwdd1	1.5575E-32	1.06700056	0.81	0.315	3.417E-28
Orc1	1.5949E-32	3.08083672	0.065	0.004	3.499E-28
Psmd13	1.8504E-32	1.33415199	0.529	0.159	4.0595E-28
Dynll2	2.1428E-32	1.42713001	0.471	0.13	4.7011E-28
Hip1r	2.2582E-32	1.11733668	0.497	0.134	4.9543E-28
Psmb5	2.6694E-32	1.32006273	0.686	0.262	5.8565E-28
Pa2g4	2.925E-32	1.4445749	0.529	0.162	6.4172E-28
Mrpl15	2.955E-32	1.43919779	0.451	0.123	6.483E-28
Wdhd1	3.1175E-32	1.9155852	0.183	0.026	6.8396E-28
Wdr62	3.281E-32	3.39270495	0.078	0.006	7.1982E-28
Sf3b5	3.5147E-32	1.24403911	0.771	0.317	7.7108E-28
H2-Q7	4.0476E-32	1.03441489	0.98	0.625	8.8799E-28
Pgp	4.1236E-32	1.84815802	0.275	0.054	9.0467E-28
Fam83d	4.4983E-32	2.77993361	0.085	0.007	9.8689E-28
Rad54b	4.9955E-32	4.38119634	0.039	0.001	1.096E-27
Atp5d	5.084E-32	1.15376238	0.922	0.489	1.1154E-27
Arsb	5.4321E-32	1.89428393	0.242	0.043	1.1918E-27
Ubb	5.7E-32	0.97223191	0.993	0.821	1.2505E-27
Mphosph6	5.8322E-32	1.48187024	0.399	0.099	1.2795E-27
Ubl4a	8.0427E-32	1.62317389	0.353	0.082	1.7645E-27
Csrp1	9.3454E-32	1.27088708	0.484	0.135	2.0503E-27
Lyar	1.0373E-31	1.26860321	0.497	0.143	2.2757E-27
Nde1	1.5215E-31	2.00082697	0.222	0.038	3.338E-27
Acadl	2.4084E-31	1.17689707	0.529	0.158	5.2838E-27
Cntrl	2.9522E-31	1.24519057	0.536	0.162	6.4769E-27
Mylpf	2.9634E-31	3.02932548	0.078	0.006	6.5014E-27
Cbx1	3.2394E-31	1.30308741	0.529	0.16	7.1069E-27
Hist1h2bg	3.5066E-31	2.64588286	0.092	0.008	7.693E-27
Cox5a	3.8138E-31	1.1071677	0.928	0.513	8.3672E-27
C230024C17F	3.9587E-31	4.02339991	0.033	0.001	8.6849E-27
Pold1	4.3336E-31	1.87060222	0.176	0.025	9.5075E-27
Spata24	4.6026E-31	2.55519798	0.137	0.016	1.0098E-26
Rpsa	5.4887E-31	0.64832124	1	0.966	1.2042E-26
Rbmx2	6.7029E-31	1.65019	0.261	0.05	1.4705E-26
Ckap5	7.6517E-31	1.6087523	0.359	0.087	1.6787E-26
Ndufc2	9.1889E-31	1.23030931	0.745	0.303	2.0159E-26
Mis12	9.508E-31	1.49900867	0.288	0.059	2.086E-26
Dgcr8	1.2587E-30	2.00867668	0.157	0.021	2.7614E-26

Ptrh2	1.4019E-30	1.81194561	0.248	0.047	3.0757E-26
Ggt1	1.5474E-30	3.50467497	0.059	0.003	3.3949E-26
Snu13	1.587E-30	1.185714	0.837	0.396	3.4818E-26
Ap2s1	1.7151E-30	1.28719025	0.693	0.268	3.7627E-26
Rfwd3	1.7956E-30	2.08180403	0.216	0.037	3.9393E-26
Ifi27	1.9103E-30	1.28943848	0.595	0.197	4.191E-26
Prim2	1.9172E-30	2.03380916	0.157	0.021	4.2062E-26
Rpa1	1.9193E-30	1.56074535	0.288	0.059	4.2107E-26
Ppih	2.5004E-30	2.12556343	0.196	0.032	5.4855E-26
Stk26	3.0534E-30	1.23335268	0.392	0.096	6.6988E-26
Brd9	3.0934E-30	1.27100131	0.536	0.168	6.7867E-26
Ywhaq	3.593E-30	1.02584232	0.686	0.246	7.8826E-26
Esm1	3.6573E-30	2.75191578	0.059	0.003	8.0238E-26
Haus8	3.715E-30	1.86587515	0.261	0.053	8.1504E-26
Prkch	4.9884E-30	1.11036052	0.523	0.155	1.0944E-25
Snrpd2	5.6611E-30	1.10681126	0.843	0.388	1.242E-25
Gars	5.6631E-30	1.34998689	0.471	0.137	1.2424E-25
Gna15	6.2758E-30	1.79390632	0.333	0.078	1.3769E-25
Ptpn22	9.2348E-30	0.90176078	0.882	0.382	2.026E-25
Gtf2i	1.1348E-29	1.17059022	0.516	0.153	2.4896E-25
Chtf18	1.2838E-29	3.66037981	0.052	0.003	2.8165E-25
Inpp1	1.3167E-29	1.28341552	0.34	0.079	2.8886E-25
Pold3	1.3861E-29	1.71135308	0.275	0.057	3.041E-25
Ctcf	1.3889E-29	1.08575479	0.732	0.278	3.0471E-25
Il2rb	1.6533E-29	0.85169188	0.778	0.298	3.6273E-25
Ndufaf2	1.8634E-29	1.86445062	0.242	0.046	4.088E-25
A430005L14Ri	1.8908E-29	1.80415807	0.261	0.053	4.1482E-25
Lzic	2.0743E-29	1.83639881	0.235	0.044	4.5509E-25
Psmc3	2.1367E-29	1.18484038	0.693	0.266	4.6877E-25
S100a4	2.586E-29	1.19756638	0.797	0.359	5.6733E-25
Baz1b	3.2099E-29	1.29693401	0.562	0.185	7.0422E-25
Selplg	3.4814E-29	1.05440327	0.869	0.412	7.6378E-25
Haus6	3.5524E-29	1.69629021	0.209	0.036	7.7937E-25
Tcof1	3.6643E-29	1.02389472	0.471	0.132	8.039E-25
Oip5	3.6993E-29	3.69042592	0.046	0.002	8.1159E-25
Csnk2b	4.3682E-29	1.23552208	0.712	0.294	9.5834E-25
Atp5j2	4.9015E-29	1.01175132	0.948	0.536	1.0753E-24
Bspry	4.9096E-29	2.65392185	0.085	0.007	1.0771E-24
S100a13	5.0278E-29	1.03970433	0.856	0.394	1.103E-24
Rinl	6.2922E-29	1.12753884	0.542	0.169	1.3805E-24
Rmi2	6.3253E-29	2.72760373	0.105	0.011	1.3877E-24
Xrcc3	7.1981E-29	4.73983927	0.026	0.001	1.5792E-24
Magohb	7.9817E-29	1.74313	0.327	0.078	1.7511E-24
Nmnat2	9.8084E-29	2.97269772	0.065	0.004	2.1519E-24
Camk4	1.0378E-28	0.98070106	0.497	0.141	2.2768E-24
Acot7	1.0705E-28	1.34041991	0.431	0.121	2.3485E-24
Gnptg	1.1379E-28	1.3687048	0.288	0.061	2.4963E-24
Emp3	1.5349E-28	1.26069858	0.797	0.37	3.3674E-24

Idh3a	1.9662E-28	1.37826284	0.373	0.096	4.3137E-24
Pcbd2	2.0431E-28	1.63657965	0.373	0.099	4.4823E-24
Sars	2.2566E-28	1.16561071	0.654	0.243	4.9508E-24
Alad	2.6064E-28	2.24714004	0.163	0.024	5.7182E-24
Ifng	2.8324E-28	0.30035134	0.268	0.054	6.2139E-24
Leprotl1	3.0289E-28	0.99108705	0.778	0.317	6.6452E-24
Wrnip1	3.3028E-28	1.49985144	0.242	0.046	7.246E-24
Dapk2	3.6319E-28	2.12765895	0.118	0.013	7.9681E-24
Ddah2	3.8971E-28	3.14813093	0.078	0.006	8.5499E-24
Sdhb	3.944E-28	1.44403493	0.536	0.184	8.6527E-24
Sept1	4.3399E-28	0.96774101	0.791	0.308	9.5213E-24
Gltp	4.4681E-28	1.10640004	0.556	0.182	9.8026E-24
Ebp	4.6925E-28	1.44364234	0.412	0.116	1.0295E-23
S1pr4	4.6951E-28	1.42253382	0.275	0.057	1.0301E-23
Lag3	4.9165E-28	1.66334086	0.222	0.041	1.0786E-23
Cisd1	5.6353E-28	1.48899782	0.333	0.081	1.2363E-23
Troap	7.3642E-28	4.24537585	0.033	0.001	1.6156E-23
Eif1ax	7.6023E-28	1.19661566	0.601	0.214	1.6679E-23
Mtbp	9.3881E-28	2.12043508	0.124	0.015	2.0597E-23
Magoh	9.4322E-28	1.21547169	0.641	0.241	2.0693E-23
Cd247	1.5317E-27	1.09543542	0.458	0.13	3.3605E-23
Polr2c	1.5448E-27	1.13014679	0.51	0.161	3.389E-23
Pkp3	1.7205E-27	1.40633818	0.346	0.086	3.7746E-23
Trp53i13	1.801E-27	2.38152577	0.15	0.021	3.9512E-23
Prf1	1.923E-27	1.15827837	0.222	0.041	4.2189E-23
Sae1	1.9994E-27	1.41296405	0.294	0.066	4.3864E-23
Nfyb	2.2803E-27	1.25050661	0.412	0.114	5.0028E-23
Gadd45gip1	2.3784E-27	1.20973077	0.477	0.145	5.218E-23
Krtcap2	2.9285E-27	1.04285931	0.843	0.403	6.4248E-23
Gen1	2.966E-27	2.84575061	0.059	0.004	6.507E-23
Pfdn6	3.5495E-27	1.23564088	0.49	0.154	7.7873E-23
Tmem163	3.7874E-27	2.22477237	0.124	0.015	8.3091E-23
Vps36	4.1155E-27	1.31743909	0.516	0.171	9.0289E-23
Zbtb8os	4.2274E-27	1.42904742	0.346	0.089	9.2745E-23
Cep128	4.4243E-27	2.27452601	0.131	0.017	9.7065E-23
2610020C07R	4.5731E-27	3.06976605	0.072	0.006	1.0033E-22
H3f3b	4.8033E-27	0.64316077	1	0.968	1.0538E-22
Impa2	4.8195E-27	1.71099157	0.248	0.051	1.0574E-22
Snrpb	4.9864E-27	1.10744233	0.817	0.418	1.094E-22
Tuba1a	5.0617E-27	1.04074977	0.464	0.141	1.1105E-22
Spdl1	5.1148E-27	2.6525423	0.111	0.013	1.1221E-22
Mrps16	5.1709E-27	1.06710603	0.627	0.225	1.1344E-22
Ttf2	5.2694E-27	1.87522681	0.15	0.021	1.1561E-22
Cdt1	5.9242E-27	1.22038715	0.346	0.087	1.2997E-22
Pbrm1	5.975E-27	0.92872798	0.719	0.277	1.3109E-22
BC030867	9.1954E-27	4.53086203	0.039	0.002	2.0174E-22
Atp5f1	9.5348E-27	1.04913149	0.863	0.44	2.0918E-22
Esyt1	9.9614E-27	1.22910747	0.529	0.177	2.1854E-22

Psmb8	1.0479E-26	1.01570368	0.876	0.443	2.2989E-22
D030056L22R	1.0654E-26	1.57352887	0.242	0.049	2.3374E-22
Erh	1.1726E-26	1.46015277	0.373	0.102	2.5725E-22
Atp5g2	1.3216E-26	0.86395152	0.98	0.693	2.8994E-22
Mrpl28	1.3739E-26	1.45202562	0.307	0.074	3.0143E-22
Pdap1	1.4093E-26	1.09930728	0.686	0.281	3.0918E-22
Cnn2	1.4514E-26	1.03490509	0.699	0.274	3.1842E-22
Sf3b2	1.9301E-26	1.0509779	0.824	0.403	4.2344E-22
Ccne2	2.0181E-26	2.60272152	0.098	0.01	4.4274E-22
Cbx2	2.0405E-26	3.71987635	0.039	0.002	4.4766E-22
Hprt	2.2297E-26	1.18745054	0.588	0.213	4.8918E-22
Gm14029	2.3104E-26	2.13337505	0.124	0.016	5.0688E-22
Mdh1	2.6218E-26	1.06641854	0.686	0.272	5.752E-22
Gzmm	2.7852E-26	4.22882955	0.046	0.002	6.1104E-22
Asrgl1	2.8438E-26	1.81493845	0.196	0.035	6.2391E-22
Emg1	3.1009E-26	1.01461939	0.745	0.304	6.8031E-22
Agfg2	3.2838E-26	2.20852487	0.137	0.019	7.2043E-22
Syngr4	4.1774E-26	5.94148411	0.013	0	9.1647E-22
Snrpf	5.0398E-26	0.93025286	0.954	0.56	1.1057E-21
Phf11b	5.5923E-26	1.53912358	0.288	0.067	1.2269E-21
Mif	5.7152E-26	0.92540339	0.889	0.48	1.2539E-21
Arhgap19	6.0458E-26	2.53357805	0.111	0.013	1.3264E-21
Nrgn	6.317E-26	1.79760486	0.209	0.039	1.3859E-21
Suz12	7.9036E-26	1.13482267	0.66	0.253	1.734E-21
Ntan1	1.266E-25	1.23426941	0.464	0.147	2.7776E-21
Dnajc2	1.2739E-25	1.07760501	0.529	0.18	2.7949E-21
Gtf2f1	1.2992E-25	1.22687225	0.444	0.136	2.8504E-21
Hdgf	1.4946E-25	1.21650743	0.641	0.255	3.2791E-21
Eri1	1.6E-25	1.24307588	0.458	0.144	3.5102E-21
Hdac1	1.6032E-25	1.14993101	0.399	0.113	3.5173E-21
Prkca	1.6134E-25	1.34103701	0.294	0.068	3.5396E-21
Chsy1	1.7838E-25	1.44425873	0.281	0.065	3.9136E-21
Smc6	1.9282E-25	0.69688738	0.693	0.258	4.2302E-21
Atp5k	2.3596E-25	0.97374438	0.869	0.424	5.1768E-21
Hist1h2bf	2.7435E-25	2.8509229	0.059	0.004	6.0189E-21
Zgrf1	3.0877E-25	2.42160351	0.105	0.012	6.7742E-21
Suv39h1	3.4087E-25	1.98516333	0.157	0.025	7.4783E-21
Crmp1	4.3249E-25	2.1103956	0.157	0.025	9.4883E-21
Cox6c	4.3737E-25	0.83686844	0.974	0.706	9.5956E-21
Eif4a3	4.6476E-25	1.24486782	0.497	0.169	1.0196E-20
Ndufa8	5.0171E-25	1.24884449	0.569	0.207	1.1007E-20
Runx2	5.1344E-25	0.95808825	0.51	0.167	1.1264E-20
Hist1h4a	5.3577E-25	2.61622046	0.092	0.009	1.1754E-20
Cep57	5.9496E-25	1.09175563	0.359	0.095	1.3053E-20
Hist1h1c	6.0268E-25	0.76121576	0.627	0.231	1.3222E-20
Hcfc1	6.4575E-25	1.4689525	0.307	0.077	1.4167E-20
Ccsap	7.187E-25	2.03847581	0.137	0.019	1.5768E-20
Npm3	7.3248E-25	1.09938585	0.529	0.181	1.607E-20

Osbpl3	7.4403E-25	1.20789331	0.327	0.083	1.6323E-20
Tuba4a	8.0282E-25	1.41503124	0.307	0.076	1.7613E-20
Pde4d	8.3558E-25	0.94146611	0.516	0.165	1.8332E-20
Atp5g3	1.1884E-24	0.97397125	0.837	0.425	2.6072E-20
Mrps11	1.2138E-24	1.68259753	0.229	0.048	2.663E-20
Serbp1	1.2532E-24	0.74991235	0.987	0.749	2.7494E-20
Snrpg	1.6021E-24	0.82904615	0.974	0.587	3.5149E-20
Myl6	1.7692E-24	0.74912203	0.993	0.787	3.8814E-20
Ube2m	1.8476E-24	1.36973707	0.516	0.188	4.0534E-20
Rnf138	1.8951E-24	0.93960801	0.569	0.197	4.1576E-20
Rbbp8	2.0444E-24	1.29805426	0.444	0.143	4.4853E-20
Park7	2.0489E-24	1.16859153	0.693	0.312	4.495E-20
Trim27	2.1238E-24	1.27721585	0.379	0.108	4.6595E-20
Rbx1	2.1338E-24	0.96415105	0.856	0.446	4.6814E-20
Msh2	2.17E-24	1.81004896	0.183	0.033	4.7607E-20
Gng3	2.2136E-24	3.5236189	0.039	0.002	4.8564E-20
Arhgdib	2.2221E-24	0.97857319	0.922	0.599	4.8751E-20
Edf1	2.4019E-24	1.0465738	0.797	0.402	5.2696E-20
Rabif	2.4845E-24	1.48723753	0.235	0.05	5.4507E-20
Psmc4	2.667E-24	1.1678772	0.418	0.128	5.8511E-20
Rpp21	3.1866E-24	1.07521723	0.471	0.152	6.9912E-20
Sec11a	3.5805E-24	1.07519505	0.641	0.255	7.8553E-20
Slc9a5	3.696E-24	3.59909445	0.033	0.001	8.1087E-20
Cox6b1	4.2285E-24	0.879877	0.967	0.624	9.2768E-20
Nol7	4.2459E-24	0.89349249	0.856	0.422	9.3151E-20
Smap1	4.4473E-24	1.00278164	0.68	0.278	9.757E-20
Arl6ip4	4.5435E-24	1.24006864	0.366	0.103	9.968E-20
Atp5h	4.8543E-24	0.78570124	0.967	0.741	1.065E-19
Actn4	4.9683E-24	1.00074002	0.641	0.251	1.09E-19
Ndufb11	5.3064E-24	0.98649053	0.856	0.455	1.1642E-19
Spn	5.4859E-24	0.68691724	0.614	0.212	1.2035E-19
0610010K14R	5.5381E-24	1.66018468	0.229	0.049	1.215E-19
Aurkaip1	5.5926E-24	1.14439323	0.627	0.255	1.227E-19
Ech1	5.6074E-24	1.42644874	0.359	0.102	1.2302E-19
Grk6	6.2594E-24	0.90544081	0.444	0.134	1.3732E-19
Golt1b	7.0145E-24	1.24225401	0.353	0.098	1.5389E-19
Swi5	7.2973E-24	0.9983433	0.614	0.235	1.601E-19
Atp5g1	7.6964E-24	1.01215506	0.81	0.39	1.6885E-19
Poc1a	1.0092E-23	3.04898886	0.059	0.004	2.214E-19
Raly	1.0453E-23	1.0179596	0.758	0.343	2.2932E-19
Pdcd5	1.1324E-23	0.83889263	0.739	0.304	2.4843E-19
Cd2	1.1987E-23	0.77313968	0.732	0.288	2.6298E-19
Psmb4	1.2089E-23	1.05868388	0.771	0.372	2.6522E-19
Nfatc2ip	1.2445E-23	1.46602838	0.216	0.044	2.7302E-19
AA467197	1.2527E-23	0.31420978	0.379	0.109	2.7483E-19
Mrpl57	1.277E-23	1.14011837	0.575	0.218	2.8016E-19
Haus1	1.3068E-23	2.37362636	0.098	0.011	2.8671E-19
Timm17a	1.4155E-23	1.00759215	0.503	0.169	3.1055E-19

Cd52	1.4211E-23	0.76116951	1	0.819	3.1179E-19
Slc7a5	1.4676E-23	1.39985925	0.261	0.061	3.2197E-19
Mrps12	1.4693E-23	1.26110652	0.373	0.108	3.2235E-19
Arpp19	1.704E-23	0.92074623	0.895	0.481	3.7384E-19
Kars	1.7109E-23	1.31367252	0.346	0.097	3.7536E-19
Mrpl42	1.7208E-23	1.30260842	0.366	0.107	3.7753E-19
Supt4a	1.8121E-23	0.98183098	0.863	0.454	3.9755E-19
Ndufb8	2.0025E-23	1.04461582	0.725	0.316	4.3934E-19
Aip	2.0913E-23	1.29769672	0.386	0.115	4.588E-19
Nup210	2.285E-23	1.24329946	0.359	0.101	5.0131E-19
Pfdn1	2.4043E-23	1.13456647	0.451	0.146	5.2747E-19
Ift27	3.1839E-23	2.10400181	0.163	0.028	6.9851E-19
Eif3c	3.3533E-23	0.85562747	0.935	0.535	7.3569E-19
Cenpa	3.7355E-23	1.14860388	0.817	0.394	8.1953E-19
Txnl1	3.7565E-23	1.02747054	0.608	0.235	8.2415E-19
Rif1	4.6512E-23	1.03191193	0.431	0.133	1.0204E-18
Nap1l1	4.735E-23	0.85131665	0.948	0.574	1.0388E-18
Rnf7	4.9009E-23	1.02807648	0.569	0.21	1.0752E-18
Naa40	5.554E-23	1.77428244	0.176	0.032	1.2185E-18
Nup205	5.9376E-23	1.69668948	0.203	0.041	1.3026E-18
Blmh	6.0889E-23	1.16949804	0.405	0.125	1.3358E-18
Fcho1	6.1855E-23	1.72254745	0.183	0.034	1.357E-18
Ino80c	6.3633E-23	1.48127721	0.248	0.057	1.396E-18
Cd96	6.5167E-23	1.06658762	0.294	0.072	1.4297E-18
Uchl1	7.1326E-23	4.80847138	0.026	0.001	1.5648E-18
Hist1h4f	7.262E-23	3.47685521	0.039	0.002	1.5932E-18
Chst12	7.3007E-23	1.66199247	0.209	0.043	1.6017E-18
Tnfrsf18	8.2617E-23	0.72969088	0.673	0.257	1.8125E-18
Lrwd1	9.5855E-23	2.01449009	0.17	0.031	2.103E-18
Faf1	1.0375E-22	1.36341593	0.275	0.068	2.2761E-18
Hnrnpab	1.1073E-22	0.97919113	0.869	0.478	2.4293E-18
Mss51	1.1576E-22	3.59855328	0.033	0.001	2.5397E-18
Psph	1.1949E-22	1.98706864	0.183	0.035	2.6215E-18
Cox20	1.3203E-22	1.09608648	0.458	0.151	2.8965E-18
Nudc	1.3952E-22	1.13382655	0.497	0.174	3.0609E-18
Bag1	1.4275E-22	0.9774064	0.68	0.288	3.1317E-18
Pih1d1	1.5188E-22	1.5948013	0.242	0.056	3.332E-18
Pin1	1.5719E-22	1.27801898	0.438	0.146	3.4487E-18
Ccdc124	1.6292E-22	1.17058271	0.444	0.148	3.5742E-18
Helb	1.688E-22	1.62725682	0.248	0.058	3.7033E-18
Naa10	1.8106E-22	1.263798	0.366	0.109	3.9723E-18
Tmem14c	1.8258E-22	1.02714394	0.647	0.264	4.0055E-18
Ssna1	2.0085E-22	1.458824	0.314	0.085	4.4064E-18
Ilk	2.1446E-22	1.42827639	0.333	0.096	4.705E-18
Dynll1	2.1988E-22	1.03595899	0.784	0.388	4.8239E-18
Ndufa5	2.3154E-22	0.81919668	0.595	0.22	5.0798E-18
Pcmt1	2.3471E-22	0.98938871	0.49	0.168	5.1493E-18
Sms	2.6166E-22	1.07436281	0.569	0.216	5.7405E-18

Akip1	2.6683E-22	2.17291339	0.131	0.02	5.8539E-18
Mrps24	2.68E-22	1.10533725	0.569	0.223	5.8796E-18
Psmb1	2.8569E-22	0.87792877	0.922	0.542	6.2677E-18
Ndufv1	2.909E-22	1.33446638	0.359	0.108	6.3821E-18
Actg1	2.9437E-22	0.56496577	1	0.914	6.4581E-18
Mrpl20	2.9676E-22	1.07638068	0.556	0.212	6.5107E-18
Cnot9	2.9858E-22	1.82522479	0.203	0.042	6.5505E-18
Alms1	3.3745E-22	2.33498882	0.092	0.01	7.4032E-18
Rbbp4	3.3983E-22	0.95995481	0.647	0.265	7.4556E-18
Anxa2	3.435E-22	0.75191428	0.732	0.317	7.536E-18
Ywhah	3.4491E-22	0.92724745	0.641	0.265	7.567E-18
Ube2i	3.5644E-22	0.87154404	0.876	0.459	7.82E-18
Tedc1	3.5784E-22	2.82756582	0.059	0.005	7.8506E-18
Prpf4	3.946E-22	1.04774847	0.333	0.091	8.657E-18
Gimap5	4.2906E-22	0.619338	0.634	0.228	9.4131E-18
Tmsb10	4.3353E-22	0.6831753	0.993	0.892	9.5111E-18
Ube2a	4.9851E-22	1.10567469	0.588	0.237	1.0937E-17
Ipp	5.0825E-22	2.40252378	0.092	0.011	1.1151E-17
Tap1	5.1563E-22	0.78772139	0.66	0.263	1.1312E-17
Ndufs5	5.5509E-22	1.02865365	0.627	0.258	1.2178E-17
Rnf26	5.9204E-22	2.18673963	0.124	0.018	1.2989E-17
2010111I01Ril	6.9751E-22	1.03585737	0.353	0.102	1.5303E-17
Cdc123	7.1508E-22	1.42422551	0.294	0.078	1.5688E-17
Slc25a4	7.8199E-22	0.90675087	0.621	0.243	1.7156E-17
Gimap4	8.1031E-22	0.61728635	0.732	0.284	1.7777E-17
1110008F13Ri	8.7183E-22	1.0399197	0.542	0.208	1.9127E-17
4930522L14Ri	8.8421E-22	1.86399373	0.124	0.018	1.9399E-17
Mvb12a	9.2678E-22	1.215119	0.399	0.128	2.0333E-17
Taf10	9.5371E-22	1.00198183	0.797	0.394	2.0923E-17
Ddx46	9.8768E-22	0.9372893	0.686	0.303	2.1669E-17
Ppib	9.9645E-22	0.84579595	0.928	0.525	2.1861E-17
Nedd1	1.0135E-21	2.00100431	0.144	0.024	2.2235E-17
Pif1	1.0144E-21	4.89600014	0.02	0.001	2.2255E-17
Sept6	1.0604E-21	0.8165455	0.569	0.202	2.3265E-17
Acp1	1.0897E-21	1.14229796	0.418	0.136	2.3907E-17
Snrpc	1.1306E-21	1.00192815	0.588	0.231	2.4804E-17
Snrnp40	1.1386E-21	1.27415495	0.34	0.098	2.498E-17
Ndufb10	1.1422E-21	0.99843697	0.719	0.332	2.5058E-17
Grap2	1.1467E-21	0.70699488	0.529	0.181	2.5157E-17
Dck	1.3422E-21	1.44396579	0.268	0.068	2.9447E-17
Tsc22d4	1.3689E-21	0.81560385	0.686	0.279	3.0031E-17
Ptpa	1.4519E-21	1.12943991	0.386	0.12	3.1852E-17
Mbnl3	1.5182E-21	1.26614045	0.209	0.044	3.3308E-17
Hist1h4j	1.6057E-21	3.27766017	0.046	0.003	3.5227E-17
Nudt1	1.7171E-21	2.50456634	0.098	0.012	3.7672E-17
Utp23	1.7597E-21	1.32638809	0.268	0.067	3.8605E-17
Mdh2	1.7704E-21	0.85062454	0.712	0.308	3.8841E-17
Rplp0	2.1178E-21	0.57363769	0.993	0.935	4.6461E-17

Nup93	2.1413E-21	1.8408037	0.144	0.024	4.6978E-17
Cd5	2.198E-21	0.62983498	0.444	0.138	4.8221E-17
Tube1	2.2622E-21	2.59267257	0.065	0.006	4.963E-17
Gm16062	2.3722E-21	1.97791771	0.157	0.028	5.2043E-17
Phf5a	2.4174E-21	1.01253268	0.562	0.219	5.3036E-17
U2af1	2.5972E-21	1.00984872	0.68	0.299	5.6979E-17
Uqcr10	2.6002E-21	0.88874617	0.863	0.457	5.7047E-17
Maea	2.7536E-21	1.16560098	0.359	0.107	6.0412E-17
Aars	2.9571E-21	1.14972455	0.497	0.183	6.4875E-17
Elof1	3.2802E-21	1.40712126	0.346	0.105	7.1965E-17
Lmnb1	3.3346E-21	0.53736811	0.882	0.443	7.3158E-17
Psma4	3.3508E-21	1.01813933	0.634	0.268	7.3512E-17
Epsti1	3.3597E-21	0.98251313	0.686	0.311	7.3708E-17
Trp53	3.7839E-21	0.88384181	0.484	0.165	8.3015E-17
Srp19	3.8936E-21	0.95116349	0.608	0.245	8.5422E-17
Gpr18	4.2805E-21	0.93813482	0.327	0.09	9.391E-17
Trim28	4.2815E-21	1.17346254	0.386	0.122	9.3932E-17
Ankle1	4.5744E-21	4.52222679	0.02	0.001	1.0036E-16
Cxcr6	4.657E-21	0.53570452	0.673	0.26	1.0217E-16
Tpm3	4.7975E-21	0.79312611	0.954	0.635	1.0525E-16
Padi2	5.1545E-21	1.38209521	0.222	0.05	1.1308E-16
Ifngr1	5.3498E-21	0.63421988	0.98	0.621	1.1737E-16
Psmb6	5.5841E-21	0.98654058	0.745	0.366	1.2251E-16
Pigx	6.154E-21	1.23558694	0.288	0.077	1.3501E-16
Prmt1	7.2505E-21	1.29582808	0.366	0.115	1.5907E-16
Cdc25a	7.3992E-21	1.65989746	0.17	0.032	1.6233E-16
Cd48	7.5722E-21	1.28617556	0.412	0.139	1.6613E-16
Jpt2	7.7913E-21	2.121994	0.137	0.023	1.7093E-16
Myl12a	8.332E-21	0.85600389	0.915	0.564	1.828E-16
Sugt1	8.3449E-21	0.99881093	0.601	0.248	1.8308E-16
Rnps1	8.5064E-21	0.973716	0.582	0.231	1.8662E-16
Mrpl13	9.4982E-21	1.05655836	0.418	0.138	2.0838E-16
Actl6a	9.873E-21	1.07199454	0.359	0.108	2.166E-16
Ndufb6	9.9263E-21	1.02281664	0.562	0.223	2.1777E-16
Eloc	1.0206E-20	0.81919344	0.804	0.379	2.2392E-16
Fundc2	1.0444E-20	1.14045656	0.542	0.215	2.2914E-16
Lsm7	1.0449E-20	1.10818792	0.366	0.113	2.2923E-16
Tfam	1.0475E-20	0.90490197	0.333	0.095	2.2982E-16
Xpo1	1.2301E-20	1.11727637	0.346	0.104	2.6987E-16
Lamtor4	1.2505E-20	1.12456233	0.471	0.171	2.7436E-16
Ndufs6	1.2903E-20	0.9169301	0.647	0.273	2.8308E-16
Suv39h2	1.2981E-20	2.25163682	0.098	0.013	2.8478E-16
Fanci	1.3545E-20	3.54376855	0.033	0.002	2.9717E-16
Zap70	1.357E-20	0.88380798	0.431	0.136	2.9771E-16
Cenpc1	1.378E-20	1.47715562	0.248	0.061	3.0232E-16
Timeless	1.3825E-20	2.32979217	0.105	0.014	3.0331E-16
Itga1	1.417E-20	1.3261245	0.242	0.059	3.1088E-16
Set	1.4308E-20	0.80018383	0.889	0.51	3.1389E-16

Dph3	1.4482E-20	0.81928228	0.68	0.289	3.1772E-16
Umps	1.5558E-20	1.59540713	0.176	0.035	3.4132E-16
Farp1	1.5772E-20	2.20614377	0.105	0.014	3.4601E-16
Zcchc17	1.6106E-20	1.17869005	0.34	0.102	3.5336E-16
Spin4	1.6119E-20	4.03224912	0.026	0.001	3.5363E-16
Gpr174	1.627E-20	0.66349015	0.327	0.089	3.5694E-16
Gtf2h5	1.8372E-20	0.97201169	0.614	0.26	4.0306E-16
Cdk5rap2	1.8645E-20	1.44980817	0.209	0.046	4.0906E-16
Mrpl33	1.9443E-20	0.83751988	0.837	0.438	4.2657E-16
Cramp1l	2.0075E-20	1.53024359	0.229	0.054	4.4042E-16
Ablim1	2.0458E-20	0.4743931	0.758	0.289	4.4882E-16
Rnaseh2a	2.0704E-20	1.82782973	0.176	0.036	4.5423E-16
Eme1	2.1283E-20	4.09086774	0.033	0.002	4.6692E-16
Mettl23	2.1979E-20	0.80187444	0.588	0.227	4.8221E-16
Myg1	2.3761E-20	1.59057647	0.196	0.042	5.2129E-16
Prdx1	2.4038E-20	0.7616172	0.856	0.423	5.2737E-16
Hist1h4b	2.6455E-20	3.73620996	0.039	0.002	5.8039E-16
Polr2j	2.9149E-20	1.38582171	0.327	0.098	6.395E-16
Rex1bd	3.1485E-20	1.09135319	0.471	0.17	6.9074E-16
Ncbp1	3.3313E-20	1.08342726	0.288	0.077	7.3086E-16
Hist1h2bk	3.4095E-20	4.03303605	0.026	0.001	7.4801E-16
Ndufa9	4.1923E-20	1.32787248	0.346	0.109	9.1974E-16
Psma2	4.2508E-20	0.85860534	0.804	0.413	9.3259E-16
Mrpl54	4.3254E-20	0.90723357	0.68	0.305	9.4894E-16
Cd28	4.4143E-20	0.62974728	0.725	0.311	9.6844E-16
Ddt	4.4745E-20	0.87090014	0.477	0.172	9.8166E-16
Ostf1	4.804E-20	0.66710149	0.895	0.526	1.054E-15
Ppwd1	4.8822E-20	0.58482618	0.477	0.161	1.0711E-15
Thoc7	6.0784E-20	0.79261556	0.542	0.201	1.3336E-15
Tmem167	6.2759E-20	0.93507073	0.667	0.299	1.3769E-15
Fkbp3	6.5973E-20	0.81857533	0.634	0.259	1.4474E-15
Sncb	7.1541E-20	4.33678557	0.02	0.001	1.5695E-15
Cep192	7.6582E-20	0.970883	0.288	0.077	1.6801E-15
Psmb10	8.3125E-20	0.95177515	0.529	0.205	1.8237E-15
Med10	8.4225E-20	1.08788115	0.431	0.151	1.8478E-15
Paics	8.5642E-20	1.29721984	0.366	0.119	1.8789E-15
Sec11c	8.7911E-20	0.80219808	0.797	0.394	1.9287E-15
Ppil1	9.3266E-20	1.35651162	0.19	0.04	2.0462E-15
Ccdc12	9.7089E-20	0.85850001	0.725	0.327	2.13E-15
Taf15	1.0418E-19	0.87668312	0.51	0.189	2.2855E-15
Atxn1	1.0628E-19	0.67053892	0.542	0.194	2.3317E-15
Polh	1.337E-19	3.01620283	0.052	0.004	2.9333E-15
Rpl28	1.3511E-19	0.50353308	0.993	0.946	2.9642E-15
Psma7	1.3586E-19	0.80019756	0.889	0.521	2.9807E-15
Gimap1	1.3671E-19	0.54972487	0.699	0.277	2.9992E-15
Mrpl47	1.4883E-19	1.78004111	0.137	0.024	3.2651E-15
Malsu1	1.5627E-19	1.10092474	0.366	0.117	3.4285E-15
Gm33104	1.6412E-19	0.98981059	0.209	0.047	3.6006E-15

Hdgfl3	1.7292E-19	1.61414284	0.157	0.03	3.7936E-15
Eif1ad	1.7559E-19	1.04601697	0.366	0.116	3.8523E-15
Ccnf	1.7942E-19	2.65369718	0.078	0.009	3.9364E-15
Paxip1	1.843E-19	1.49997016	0.19	0.041	4.0433E-15
Npat	1.8926E-19	1.28604068	0.222	0.053	4.1521E-15
Gmcl1	1.9861E-19	1.43607212	0.196	0.043	4.3574E-15
H2-T22	2.1871E-19	0.97598041	0.575	0.236	4.7983E-15
Hnrnpl	2.3451E-19	0.78447072	0.667	0.285	5.145E-15
Serpinb1b	2.3962E-19	2.8930768	0.052	0.004	5.2571E-15
Nme1	2.4012E-19	0.91663567	0.712	0.33	5.2679E-15
Tinf2	2.4918E-19	1.06413454	0.294	0.082	5.4668E-15
Gzmc	2.6455E-19	0.97994287	0.118	0.018	5.8041E-15
Mrps15	2.7292E-19	1.12112195	0.379	0.125	5.9876E-15
Ndufs7	2.7304E-19	0.82975215	0.529	0.202	5.9903E-15
Cdkn2d	2.7352E-19	1.22223565	0.288	0.081	6.0008E-15
Cptp	2.8057E-19	2.90819297	0.059	0.005	6.1555E-15
Sf3b6	3.1097E-19	0.79321199	0.81	0.414	6.8225E-15
Plekhj1	3.1729E-19	0.89192039	0.497	0.185	6.961E-15
Cysltr2	3.3126E-19	0.8980649	0.294	0.081	7.2674E-15
Rbm8a	3.3606E-19	0.75826823	0.758	0.346	7.3727E-15
B4galnt1	3.6338E-19	0.90223099	0.536	0.208	7.9722E-15
Snrpd3	4.0902E-19	0.90335394	0.595	0.251	8.9736E-15
Hsd11b1	4.1906E-19	1.62194547	0.137	0.024	9.1938E-15
Nsmce1	4.2225E-19	1.10407235	0.314	0.093	9.2637E-15
Wdyhv1	6.2894E-19	1.4466594	0.183	0.039	1.3798E-14
Crot	6.344E-19	0.67912162	0.399	0.129	1.3918E-14
Uqcrc1	6.3556E-19	0.96800412	0.51	0.198	1.3943E-14
Gle1	7.7533E-19	1.22733223	0.242	0.062	1.701E-14
Aes	8.8421E-19	0.72552998	0.824	0.406	1.9399E-14
Dcaf15	9.0402E-19	0.89797773	0.301	0.085	1.9833E-14
Cript	9.1031E-19	0.78025405	0.752	0.354	1.9971E-14
Larp7	9.3208E-19	0.97883101	0.458	0.164	2.0449E-14
Bin2	9.4307E-19	1.14450586	0.392	0.133	2.069E-14
Ttc39c	9.7137E-19	1.02787726	0.275	0.075	2.1311E-14
Lmf2	9.8894E-19	1.65882764	0.183	0.04	2.1696E-14
Bcl11b	1.0814E-18	0.67910988	0.477	0.168	2.3724E-14
Prelid3b	1.1264E-18	1.25426916	0.366	0.124	2.4713E-14
Psmc3ip	1.1861E-18	2.55177047	0.065	0.007	2.6021E-14
Acap1	1.2655E-18	0.81528479	0.34	0.102	2.7763E-14
Ndufs3	1.3298E-18	0.91940282	0.438	0.156	2.9174E-14
Msh6	1.4805E-18	1.94807261	0.131	0.023	3.248E-14
Rabggtb	1.561E-18	0.89801916	0.405	0.138	3.4247E-14
Pycard	1.676E-18	0.96055517	0.497	0.191	3.6771E-14
Slc9a3r1	1.7388E-18	0.54474459	0.529	0.197	3.8149E-14
Igf2r	1.788E-18	0.85192962	0.353	0.11	3.9228E-14
Tesc	1.8334E-18	0.78487847	0.366	0.114	4.0224E-14
Dctn3	1.9803E-18	0.83842672	0.627	0.273	4.3446E-14
Asnsd1	2.0597E-18	1.1590226	0.268	0.075	4.5187E-14

Gpsm2	2.1068E-18	2.81466177	0.078	0.009	4.6222E-14
Fam111a	2.2393E-18	0.57322142	0.386	0.124	4.9127E-14
Pds5b	2.3844E-18	1.31086306	0.242	0.063	5.2312E-14
Ipo9	2.54E-18	1.70391416	0.15	0.029	5.5726E-14
Rnf125	2.8615E-18	0.5702791	0.556	0.206	6.2778E-14
Eif3h	3.1136E-18	0.72169052	0.948	0.603	6.831E-14
Pola2	3.1944E-18	1.90577061	0.118	0.019	7.0082E-14
Pam16	3.6385E-18	1.03209251	0.412	0.146	7.9825E-14
Lsm12	3.7954E-18	0.83904292	0.686	0.31	8.3268E-14
Bcas2	4.0281E-18	0.750448	0.614	0.258	8.8372E-14
Nkap	4.1258E-18	0.92453941	0.346	0.11	9.0516E-14
Snrpa1	4.1526E-18	0.973934	0.399	0.139	9.1105E-14
Mxi1	4.1792E-18	0.67290551	0.68	0.301	9.1688E-14
Cops6	4.2308E-18	1.02992509	0.392	0.135	9.2818E-14
Rad18	6.0689E-18	1.14762938	0.268	0.075	1.3315E-13
Cdc34	6.1001E-18	0.89865301	0.438	0.16	1.3383E-13
S100a6	6.1754E-18	0.53056474	0.895	0.542	1.3548E-13
Pole2	6.2459E-18	2.80532084	0.059	0.006	1.3703E-13
Rab27a	6.3048E-18	1.13281854	0.203	0.048	1.3832E-13
9130401M01F	6.3365E-18	1.37482318	0.261	0.073	1.3902E-13
Vdac2	6.4084E-18	0.89782444	0.68	0.321	1.4059E-13
Cwc15	7.2738E-18	0.77918573	0.778	0.395	1.5958E-13
Atp5b	7.461E-18	0.73782714	0.928	0.583	1.6369E-13
Ndufv2	7.5705E-18	1.02693274	0.484	0.194	1.6609E-13
Ctdspl2	8.0068E-18	0.8835426	0.399	0.135	1.7566E-13
Polr3k	8.5397E-18	1.42640695	0.242	0.065	1.8735E-13
Kremen2	8.7624E-18	3.86743131	0.033	0.002	1.9224E-13
Ptpn7	9.4628E-18	1.0736885	0.235	0.061	2.0761E-13
Ndufb9	9.9902E-18	1.00630005	0.725	0.381	2.1917E-13
Srsf7	1.0514E-17	0.95978956	0.601	0.266	2.3067E-13
Nt5c	1.1093E-17	1.0921382	0.458	0.178	2.4337E-13
Gnptab	1.1509E-17	1.22228221	0.235	0.062	2.5249E-13
Bola3	1.1542E-17	0.90591303	0.412	0.146	2.5322E-13
Itpripl1	1.1961E-17	1.8395178	0.124	0.022	2.6242E-13
Cfl1	1.1982E-17	0.60759993	0.98	0.782	2.6287E-13
Nop58	1.261E-17	0.71719258	0.516	0.199	2.7665E-13
Rnf187	1.4561E-17	1.01158908	0.392	0.138	3.1945E-13
Dnajc19	1.5259E-17	0.79365149	0.51	0.2	3.3478E-13
Grpel1	1.7151E-17	0.90383917	0.438	0.161	3.7627E-13
Mrpl41	1.8558E-17	1.19108482	0.32	0.101	4.0714E-13
Zfp511	1.8648E-17	1.35314186	0.268	0.078	4.0912E-13
Minos1	1.9168E-17	0.82433042	0.863	0.476	4.2053E-13
Rer1	2.0051E-17	0.9205765	0.516	0.211	4.399E-13
Fkbp2	2.0159E-17	1.00252474	0.477	0.189	4.4227E-13
Rbm15	2.0403E-17	0.91984826	0.353	0.116	4.4762E-13
Fxyd5	2.0423E-17	0.66846561	0.961	0.66	4.4805E-13
Ndufs2	2.0968E-17	0.97940795	0.405	0.147	4.6002E-13
Mcm8	2.1635E-17	2.78676128	0.046	0.004	4.7466E-13

Atp5c1	2.3373E-17	0.80463585	0.817	0.45	5.1278E-13
Itk	2.4216E-17	0.37923633	0.765	0.319	5.3128E-13
Adh5	2.4234E-17	0.90802191	0.386	0.135	5.3167E-13
Mpp2	2.4937E-17	3.36701704	0.039	0.003	5.471E-13
Ppp1r35	2.5296E-17	1.35660685	0.183	0.042	5.5496E-13
Ostc	2.5588E-17	0.79135036	0.641	0.289	5.6137E-13
Hmces	2.6692E-17	0.96939816	0.294	0.087	5.856E-13
Hnrnpd	2.6976E-17	0.73126061	0.85	0.467	5.9182E-13
Prkcz	2.7536E-17	1.54035842	0.137	0.026	6.0411E-13
Psmb2	2.8813E-17	0.98822815	0.569	0.252	6.3214E-13
Cdkn2c	2.8954E-17	1.93464364	0.111	0.018	6.3522E-13
Dpm3	2.9098E-17	0.78885358	0.588	0.251	6.3839E-13
Uchl5	2.9496E-17	1.21677365	0.288	0.087	6.4711E-13
Uchl3	3.0749E-17	1.09127274	0.261	0.074	6.7461E-13
Stard3nl	3.5852E-17	1.24931942	0.307	0.098	7.8656E-13
Zw10	3.7336E-17	1.39879953	0.144	0.028	8.1912E-13
Trappc4	3.7379E-17	0.83629251	0.484	0.187	8.2007E-13
Ufc1	3.882E-17	0.86208857	0.562	0.238	8.5167E-13
Fbxl8	4.0094E-17	2.09183675	0.085	0.011	8.7961E-13
Brip1os	4.0966E-17	1.57014967	0.15	0.031	8.9876E-13
Cpsf4	4.1283E-17	1.55701524	0.163	0.035	9.057E-13
Pop5	4.282E-17	0.92397788	0.353	0.117	9.3942E-13
Sin3a	4.2824E-17	1.0072435	0.288	0.086	9.3952E-13
Nono	4.9833E-17	0.81403788	0.621	0.282	1.0933E-12
Cdca7l	5.0544E-17	1.91411238	0.118	0.02	1.1089E-12
Sfr1	5.0848E-17	0.85535954	0.627	0.282	1.1156E-12
Hltf	5.0991E-17	0.85249185	0.307	0.094	1.1187E-12
Gabpb2	5.2771E-17	0.77546565	0.366	0.122	1.1578E-12
Ubac2	5.43E-17	1.06881476	0.346	0.116	1.1913E-12
Pop1	5.5306E-17	2.40290724	0.078	0.01	1.2134E-12
Atp10a	5.6063E-17	1.07490386	0.229	0.06	1.23E-12
Apoo	5.7438E-17	2.31867189	0.078	0.01	1.2601E-12
Atf1	6.2582E-17	0.95211969	0.431	0.162	1.373E-12
Ndufa13	6.6517E-17	0.79227847	0.876	0.524	1.4593E-12
Vrk3	6.7732E-17	1.10205195	0.255	0.072	1.486E-12
Tdp2	6.8268E-17	0.94493256	0.255	0.071	1.4977E-12
Cep78	6.9148E-17	2.260634	0.065	0.007	1.517E-12
Apobec3	7.1448E-17	0.71416325	0.444	0.165	1.5675E-12
Snapc5	7.3803E-17	1.05600008	0.353	0.12	1.6192E-12
Ccdc25	7.4607E-17	1.0258519	0.32	0.103	1.6368E-12
Coa7	7.6868E-17	1.54011086	0.176	0.041	1.6864E-12
Taf1	7.7693E-17	0.85046442	0.431	0.159	1.7045E-12
Cpsf2	7.7882E-17	0.89846891	0.327	0.106	1.7087E-12
Ndufa1	7.8707E-17	0.74833154	0.791	0.421	1.7267E-12
Hinfp	8.0372E-17	1.01706569	0.19	0.045	1.7633E-12
Cdc42se1	8.1383E-17	0.71250942	0.503	0.197	1.7855E-12
Get4	8.6981E-17	1.12360513	0.235	0.064	1.9083E-12
Sub1	9.0473E-17	0.62134884	1	0.814	1.9849E-12

Tbc1d10c	9.1898E-17	0.96982977	0.248	0.068	2.0162E-12
Gng2	9.3667E-17	0.81604629	0.667	0.315	2.055E-12
Hat1	9.369E-17	1.13590414	0.333	0.112	2.0555E-12
Faxc	9.3728E-17	4.34497149	0.013	0	2.0563E-12
Mrpl49	9.6809E-17	1.37944294	0.17	0.038	2.1239E-12
Ppm1g	1.0343E-16	0.66069715	0.516	0.203	2.269E-12
Gnai2	1.0379E-16	0.65014125	0.967	0.67	2.2771E-12
Serf2	1.0537E-16	0.53544577	0.98	0.849	2.3117E-12
Hnrnpul1	1.0689E-16	0.75821928	0.641	0.292	2.3451E-12
Frg1	1.0841E-16	0.76154726	0.562	0.235	2.3783E-12
Zbtb25	1.2328E-16	1.0759998	0.216	0.055	2.7047E-12
Vps28	1.2731E-16	0.73368753	0.608	0.265	2.793E-12
Epdr1	1.3234E-16	3.65898392	0.02	0.001	2.9034E-12
Pcdhgc4	1.3656E-16	3.22405773	0.033	0.002	2.996E-12
Gm42047	1.423E-16	2.58163245	0.072	0.009	3.122E-12
Srbd1	1.4244E-16	1.80997537	0.131	0.025	3.125E-12
Myo1f	1.4525E-16	1.212784	0.268	0.081	3.1866E-12
Mapk3	1.4555E-16	1.18322789	0.301	0.096	3.1933E-12
Aaas	1.4863E-16	1.92564489	0.105	0.017	3.2609E-12
Trbv1	1.4889E-16	3.46311447	0.026	0.001	3.2666E-12
Suox	1.4969E-16	2.19519876	0.092	0.013	3.284E-12
Mrps14	1.5802E-16	0.8126366	0.588	0.257	3.4669E-12
Klhl28	1.5881E-16	0.94049717	0.248	0.069	3.4841E-12
Xrcc2	1.593E-16	2.53392584	0.059	0.006	3.4949E-12
Atp8b4	1.6873E-16	1.79076783	0.196	0.049	3.7018E-12
D16Ertd472e	1.7764E-16	0.4005753	0.817	0.377	3.8973E-12
Anapc15	1.788E-16	1.23956126	0.248	0.072	3.9227E-12
Psma5	1.8371E-16	0.96330043	0.575	0.256	4.0304E-12
Ciapin1	1.8559E-16	0.89888165	0.301	0.094	4.0716E-12
Trbv13-1	1.9617E-16	3.10750181	0.033	0.002	4.3037E-12
Hdgfl2	1.9935E-16	1.19543695	0.216	0.056	4.3736E-12
Apex2	2.0151E-16	1.84478677	0.098	0.015	4.421E-12
Ap1s1	2.0186E-16	1.71565653	0.203	0.052	4.4287E-12
Crip2	2.0316E-16	2.13279613	0.105	0.017	4.4572E-12
Arrdc1	2.1566E-16	1.08425577	0.281	0.086	4.7313E-12
Gatad2a	2.1797E-16	0.76950006	0.477	0.187	4.7821E-12
Ybx1	2.2139E-16	0.59267658	1	0.735	4.857E-12
Ccp110	2.295E-16	1.90745664	0.098	0.015	5.0349E-12
Ndufab1	2.3117E-16	0.84630291	0.536	0.231	5.0717E-12
Chchd2	2.484E-16	0.53580575	0.987	0.78	5.4496E-12
Mrpl43	2.5542E-16	0.89534912	0.438	0.169	5.6037E-12
Tbcb	2.5766E-16	0.955534	0.49	0.207	5.6529E-12
Nup160	2.612E-16	1.34550618	0.163	0.036	5.7306E-12
Trappc1	2.6572E-16	1.27335714	0.242	0.069	5.8296E-12
Ilf2	2.6835E-16	0.63039143	0.471	0.179	5.8874E-12
Enkd1	2.7087E-16	2.82060534	0.059	0.006	5.9426E-12
Eif4e2	2.7683E-16	0.77246363	0.562	0.237	6.0734E-12
Tfrc	2.9068E-16	0.80871191	0.314	0.1	6.3773E-12

Fam173a	2.9252E-16	0.74267766	0.425	0.157	6.4175E-12
Uqcrq	3.0434E-16	0.68120809	0.928	0.574	6.677E-12
Tiprl	3.0627E-16	0.76023273	0.477	0.186	6.7193E-12
Pms2	3.0715E-16	1.10108677	0.157	0.033	6.7386E-12
Tma7	3.2103E-16	0.71033549	0.928	0.579	7.043E-12
Nmb	3.3083E-16	1.59087069	0.131	0.025	7.2581E-12
Zc3h18	3.4534E-16	0.85157174	0.386	0.137	7.5764E-12
Sytl2	3.4984E-16	1.0544962	0.255	0.072	7.6752E-12
Tmem9b	3.5417E-16	1.03914191	0.307	0.1	7.7701E-12
Ndufv3	3.5528E-16	0.80420209	0.732	0.367	7.7945E-12
Nrn1	3.8246E-16	2.61736671	0.046	0.004	8.3909E-12
Fbxo48	3.92E-16	3.9741471	0.026	0.001	8.6001E-12
Sapcd2	4.0106E-16	3.85579903	0.026	0.001	8.7988E-12
Tmem160	4.1454E-16	0.7191659	0.66	0.306	9.0947E-12
Clec2i	4.2119E-16	0.97243208	0.248	0.07	9.2405E-12
Ndufs4	4.214E-16	0.7402372	0.549	0.23	9.2451E-12
Hp1bp3	4.3499E-16	0.78091393	0.601	0.265	9.5433E-12
Sp3os	4.3606E-16	1.33522121	0.17	0.039	9.5666E-12
Akt2	4.371E-16	0.79913035	0.373	0.131	9.5896E-12
Klre1	4.5097E-16	1.60571218	0.144	0.03	9.8938E-12
Nipal3	4.8386E-16	1.10710084	0.183	0.043	1.0615E-11
Hscb	4.8607E-16	1.18395073	0.268	0.081	1.0664E-11
Smdt1	4.8684E-16	0.74259803	0.837	0.47	1.0681E-11
Ssr2	5.0712E-16	1.01128031	0.438	0.173	1.1126E-11
Ndufc1	5.1589E-16	0.84444599	0.556	0.248	1.1318E-11
Lsp1	5.3671E-16	0.61420649	0.869	0.466	1.1775E-11
Fkbp5	5.3738E-16	1.61678896	0.15	0.032	1.179E-11
Exosc3	5.4477E-16	1.02255808	0.327	0.109	1.1952E-11
Fam60a	5.9396E-16	1.14196825	0.242	0.069	1.3031E-11
Wdr31	5.9959E-16	4.82775583	0.013	0	1.3154E-11
Gm48796	5.9959E-16	5.00251557	0.013	0	1.3154E-11
Ppp1r3g	6.0269E-16	4.41124736	0.013	0	1.3222E-11
Utf1	6.1163E-16	2.09228564	0.092	0.014	1.3419E-11
Cox7b	6.2083E-16	0.70331351	0.876	0.513	1.362E-11
Slc25a5	6.8756E-16	0.72219686	0.869	0.513	1.5084E-11
Tomm5	6.9324E-16	0.91657122	0.484	0.201	1.5209E-11
Carnmt1	7.0403E-16	1.00941484	0.386	0.142	1.5446E-11
Rsl1d1	7.3985E-16	0.74982078	0.503	0.205	1.6232E-11
Cnot3	7.5089E-16	0.65205248	0.451	0.171	1.6474E-11
Exosc4	8.0876E-16	1.27207159	0.209	0.055	1.7743E-11
Snrnp27	8.213E-16	0.88643101	0.471	0.195	1.8019E-11
Pank2	8.2789E-16	0.83256014	0.379	0.136	1.8163E-11
Sart3	8.3477E-16	1.0611394	0.248	0.072	1.8314E-11
Psmd8	8.6442E-16	0.80098094	0.627	0.293	1.8964E-11
Mb21d1	9.3992E-16	1.91948262	0.111	0.02	2.0621E-11
Tarsl2	9.6423E-16	2.35405064	0.072	0.009	2.1154E-11
Trmt112	9.9725E-16	0.76928803	0.614	0.283	2.1879E-11
Pym1	1.1164E-15	0.98612224	0.281	0.087	2.4494E-11

Gspt1	1.1494E-15	0.69061535	0.51	0.21	2.5217E-11
Saraf	1.2227E-15	0.54519161	0.771	0.38	2.6825E-11
Psmd12	1.277E-15	0.92911087	0.451	0.181	2.8016E-11
Abhd17a	1.3541E-15	1.11029823	0.333	0.118	2.9708E-11
Mrps21	1.5415E-15	0.90417435	0.529	0.237	3.3819E-11
Med14	1.592E-15	1.05449677	0.281	0.088	3.4926E-11
Bnip3l	1.6396E-15	0.52115328	0.699	0.315	3.5971E-11
Sri	1.6547E-15	0.7799922	0.732	0.38	3.6302E-11
Ubap2	1.6794E-15	0.81661747	0.294	0.092	3.6843E-11
Alg13	1.7259E-15	1.88083333	0.137	0.028	3.7864E-11
Nedd8	1.8086E-15	0.6704184	0.797	0.403	3.968E-11
Acot8	1.9175E-15	1.588387	0.17	0.041	4.2067E-11
Dazap1	1.984E-15	0.85243525	0.431	0.168	4.3527E-11
Lmtk3	2.0911E-15	2.16214213	0.072	0.009	4.5878E-11
Hnrnpa3	2.1947E-15	0.65478987	0.98	0.746	4.8151E-11
Cables1	2.2023E-15	0.66237132	0.216	0.057	4.8317E-11
Aimp2	2.2243E-15	1.57520687	0.137	0.028	4.8798E-11
Panx1	2.2328E-15	1.02930203	0.229	0.064	4.8985E-11
Uxt	2.3488E-15	1.22558441	0.229	0.066	5.153E-11
Ddx43	2.3783E-15	2.86975045	0.039	0.003	5.2178E-11
Sept7	2.4262E-15	0.72087056	0.673	0.328	5.3228E-11
Hmgxb4	2.4529E-15	1.42247594	0.183	0.046	5.3814E-11
Mad2l1bp	2.4788E-15	1.19684497	0.176	0.043	5.4383E-11
Hcst	2.5529E-15	0.37858011	0.902	0.47	5.6008E-11
Ubxn1	2.5591E-15	0.7310794	0.725	0.371	5.6145E-11
Tmem192	2.672E-15	1.18227759	0.268	0.084	5.8622E-11
Ldha	2.7455E-15	0.59772514	0.863	0.491	6.0234E-11
Thoc3	2.8227E-15	1.12369643	0.209	0.056	6.1926E-11
Tbrg1	2.9429E-15	0.72876125	0.51	0.211	6.4564E-11
Fanca	3.1139E-15	2.78949599	0.059	0.007	6.8316E-11
E2f4	3.1743E-15	0.91541592	0.255	0.076	6.9642E-11
Ddx27	3.235E-15	0.86259568	0.32	0.107	7.0973E-11
BC003965	3.2514E-15	0.99607073	0.209	0.056	7.1332E-11
Bckdk	3.3742E-15	1.55612887	0.15	0.034	7.4028E-11
Ndfip1	3.6175E-15	0.69293928	0.621	0.284	7.9364E-11
Ppp2r5d	3.7318E-15	1.59654121	0.157	0.036	8.1872E-11
Pigf	3.7692E-15	1.71585392	0.131	0.027	8.2693E-11
Nars	3.8438E-15	0.64339588	0.719	0.352	8.4329E-11
Cdk2ap2	3.9625E-15	0.48606656	0.771	0.406	8.6932E-11
Tecr	4.0825E-15	0.96146595	0.425	0.171	8.9565E-11
Snf8	4.234E-15	0.93112443	0.444	0.183	9.289E-11
Thap7	4.2515E-15	1.4318567	0.17	0.041	9.3273E-11
Tsn	4.4593E-15	0.78492668	0.549	0.243	9.7832E-11
Sass6	4.4636E-15	2.31702156	0.092	0.015	9.7926E-11
Wrap73	4.5538E-15	1.51784923	0.111	0.02	9.9905E-11
Stard4	4.7759E-15	0.92864276	0.314	0.105	1.0478E-10
Polr2f	4.8194E-15	0.91607258	0.425	0.167	1.0573E-10
Tedc2	5.0908E-15	3.31165545	0.039	0.003	1.1169E-10

Gas2	5.4408E-15	1.92193657	0.072	0.009	1.1937E-10
Hist1h2bc	5.5423E-15	1.31861246	0.275	0.088	1.2159E-10
Fzr1	5.6026E-15	1.27061059	0.196	0.052	1.2291E-10
Anxa6	5.9628E-15	0.96100408	0.542	0.255	1.3082E-10
Plcg1	6.0209E-15	0.79074138	0.248	0.072	1.3209E-10
Lpxn	6.0784E-15	0.73426946	0.542	0.235	1.3335E-10
Ccr5	6.5174E-15	0.45979394	0.634	0.287	1.4298E-10
Jade2	6.5351E-15	0.93866707	0.19	0.048	1.4337E-10
Yipf1	6.6219E-15	1.18011065	0.268	0.085	1.4528E-10
Tagln2	6.7847E-15	0.60769559	0.902	0.52	1.4885E-10
Orc2	7.266E-15	1.13222304	0.19	0.049	1.5941E-10
Ank3	7.2855E-15	3.21863803	0.02	0.001	1.5984E-10
Rpp25l	7.3605E-15	0.90979631	0.301	0.099	1.6148E-10
Chchd7	7.4015E-15	0.88535904	0.281	0.09	1.6238E-10
Fam162a	7.4036E-15	1.02323805	0.281	0.091	1.6243E-10
Eif1b	7.577E-15	0.81228914	0.373	0.138	1.6623E-10
Ryk	8.2506E-15	1.45324784	0.111	0.02	1.8101E-10
Tacc1	8.6526E-15	0.62568866	0.699	0.34	1.8983E-10
Stk39	8.8066E-15	1.21784895	0.196	0.051	1.9321E-10
Tfdp2	9.0515E-15	1.3078628	0.15	0.034	1.9858E-10
Ndufa3	9.6525E-15	0.70714451	0.752	0.409	2.1177E-10
Kpnb1	9.8009E-15	0.64763362	0.379	0.138	2.1502E-10
Gps2	1.0197E-14	0.84265613	0.386	0.146	2.237E-10
Cox4i1	1.0236E-14	0.47552351	0.993	0.848	2.2457E-10
Mdp1	1.042E-14	0.88113083	0.327	0.113	2.286E-10
Leo1	1.0547E-14	1.28439499	0.203	0.055	2.3139E-10
Tmem256	1.1001E-14	0.88347988	0.529	0.238	2.4135E-10
Casp2	1.2237E-14	0.97110392	0.176	0.044	2.6846E-10
Ppic	1.3304E-14	2.13366096	0.072	0.01	2.9188E-10
Ndufs8	1.3449E-14	0.90112721	0.425	0.172	2.9506E-10
Arpc4	1.3661E-14	0.6214523	0.765	0.394	2.997E-10
Glis1	1.4751E-14	4.60623274	0.013	0	3.2362E-10
Rnf165	1.4751E-14	4.51136593	0.013	0	3.2362E-10
Nup62	1.5587E-14	1.28356633	0.229	0.068	3.4197E-10
Eef1d	1.6145E-14	0.58850317	0.935	0.577	3.5421E-10
Mov10	1.6231E-14	1.41509441	0.111	0.021	3.561E-10
Psme2	1.7372E-14	0.60565599	0.797	0.443	3.8113E-10
Ddias	1.7554E-14	2.43527987	0.052	0.006	3.8513E-10
Pycrl	1.7572E-14	2.37287746	0.078	0.012	3.8552E-10
Ilkap	1.8079E-14	0.77920375	0.327	0.113	3.9663E-10
Rtn3	1.8089E-14	0.70922379	0.516	0.222	3.9685E-10
Kif2a	1.8453E-14	0.82108523	0.346	0.125	4.0484E-10
Ubac1	1.85E-14	1.46321619	0.15	0.035	4.0586E-10
Rgs3	1.9214E-14	1.18631148	0.144	0.032	4.2154E-10
Tbc1d10b	1.9872E-14	0.9894807	0.242	0.073	4.3598E-10
Mthfd1	2.0611E-14	1.566465	0.118	0.023	4.5217E-10
Sh2b1	2.1009E-14	0.86870276	0.235	0.068	4.6092E-10
Sp100	2.1042E-14	0.55954389	0.575	0.257	4.6165E-10

Ppp2r5c	2.131E-14	0.75327068	0.588	0.277	4.6751E-10
Gmfg	2.1538E-14	0.53780096	0.889	0.541	4.7253E-10
Commd3	2.1783E-14	1.05486512	0.301	0.103	4.779E-10
Wdr90	2.232E-14	1.69388957	0.085	0.013	4.8967E-10
Tmem50a	2.2726E-14	0.71785042	0.699	0.363	4.9859E-10
Prps1	2.4205E-14	0.93029129	0.268	0.085	5.3103E-10
Abhd8	2.5755E-14	1.45796057	0.144	0.032	5.6505E-10
Arf5	2.6244E-14	0.72119605	0.843	0.481	5.7576E-10
Rbm17	2.8277E-14	0.78141749	0.484	0.205	6.2037E-10
Exosc7	2.8323E-14	1.48175691	0.163	0.04	6.2137E-10
Timm10b	2.8831E-14	0.82805717	0.464	0.198	6.3253E-10
Cdkn2aipnl	2.917E-14	1.24625168	0.19	0.05	6.3995E-10
Metap2	2.9383E-14	0.58186747	0.68	0.315	6.4464E-10
Sys1	2.9658E-14	0.6741877	0.667	0.325	6.5066E-10
Trir	2.9658E-14	0.61442852	0.706	0.352	6.5067E-10
Nmrk1	2.9826E-14	0.40713577	0.353	0.124	6.5435E-10
Nhp2	3.0495E-14	0.88728761	0.431	0.179	6.6903E-10
Tmem263	3.1356E-14	1.14334797	0.183	0.047	6.8792E-10
Mlf2	3.2371E-14	0.80641272	0.392	0.153	7.1018E-10
Prkcq	3.2528E-14	1.07212542	0.19	0.05	7.1363E-10
Ndc1	3.28E-14	2.07269221	0.085	0.013	7.1959E-10
Rpp30	3.3791E-14	1.66820656	0.15	0.035	7.4135E-10
Gnb2	3.3892E-14	0.67046018	0.758	0.405	7.4357E-10
Skp1a	3.5431E-14	0.56520947	0.529	0.227	7.7732E-10
Ndufb3	3.7036E-14	0.68318547	0.588	0.277	8.1253E-10
Bbip1	3.8798E-14	0.91246018	0.333	0.122	8.5118E-10
Fkbp8	3.9467E-14	0.67147268	0.477	0.2	8.6586E-10
Cenpu	4.0032E-14	2.3854798	0.052	0.006	8.7826E-10
Erg28	4.1053E-14	1.15095033	0.242	0.075	9.0065E-10
Glipr2	4.1211E-14	0.89523637	0.242	0.073	9.0413E-10
Prkcsh	4.1953E-14	0.90754366	0.379	0.146	9.204E-10
Itpa	4.1953E-14	1.33828372	0.183	0.048	9.2041E-10
Hadha	4.3038E-14	0.75908145	0.471	0.197	9.4421E-10
Gm36756	4.3148E-14	2.71570604	0.039	0.003	9.4662E-10
Nfkbil1	4.3313E-14	1.56908464	0.15	0.035	9.5024E-10
Tshz1	4.4088E-14	0.61429625	0.366	0.132	9.6725E-10
Hras	4.6242E-14	1.32583701	0.235	0.073	1.0145E-09
Cir1	4.6754E-14	0.4842877	0.542	0.231	1.0257E-09
Ndufb5	5.0908E-14	0.69739334	0.706	0.36	1.1169E-09
Fmc1	5.0919E-14	1.17003571	0.261	0.085	1.1171E-09
Stat3	5.1007E-14	0.65042176	0.876	0.609	1.119E-09
Def6	5.1509E-14	1.23132511	0.196	0.054	1.1301E-09
Ppp4c	5.4293E-14	0.67351787	0.536	0.236	1.1911E-09
Pfkp	5.5213E-14	0.74211189	0.575	0.266	1.2113E-09
Mlec	5.6852E-14	0.94520282	0.373	0.145	1.2473E-09
Snhg6	5.7359E-14	0.68893738	0.477	0.201	1.2584E-09
Ttc9c	5.9003E-14	1.19833286	0.209	0.06	1.2945E-09
Nop9	5.9228E-14	1.1947316	0.229	0.069	1.2994E-09

Virma	6.4941E-14	0.75172959	0.333	0.118	1.4247E-09
Emc3	6.5582E-14	0.73543351	0.431	0.174	1.4388E-09
Nsmce2	6.65E-14	1.08304911	0.248	0.079	1.4589E-09
Ywhab	6.7205E-14	0.68293739	0.68	0.35	1.4744E-09
Polr1d	6.816E-14	0.64146599	0.837	0.489	1.4954E-09
Ahi1	6.8409E-14	1.11637698	0.248	0.078	1.5008E-09
Saal1	6.9079E-14	1.59048412	0.111	0.022	1.5155E-09
Cep152	7.0176E-14	1.79839763	0.085	0.014	1.5396E-09
Gm29666	7.0877E-14	2.94075218	0.033	0.002	1.555E-09
Mrpl51	7.9776E-14	0.9451486	0.353	0.133	1.7502E-09
Nmi	8.0851E-14	0.94565761	0.333	0.122	1.7738E-09
2210016F16Ri	8.1149E-14	1.15071247	0.216	0.064	1.7803E-09
Tmco1	8.2085E-14	0.76530019	0.431	0.18	1.8009E-09
Abcb1b	8.3679E-14	0.9480194	0.386	0.154	1.8358E-09
Susd1	8.489E-14	1.59769291	0.085	0.014	1.8624E-09
Mtx1	8.6827E-14	1.26655394	0.196	0.055	1.9049E-09
Zwint	9.5012E-14	1.16640491	0.242	0.076	2.0845E-09
Prrc2b	9.8431E-14	0.69082089	0.451	0.184	2.1595E-09
Sytl3	1.0485E-13	0.35810557	0.373	0.134	2.3002E-09
Syt11	1.0494E-13	1.36853386	0.144	0.033	2.3024E-09
Gnas	1.0604E-13	0.52932333	0.935	0.706	2.3264E-09
Ndufa12	1.0657E-13	0.85420598	0.418	0.174	2.3381E-09
Tcerg1	1.1086E-13	0.69085478	0.412	0.165	2.4323E-09
Syf2	1.1098E-13	0.58151665	0.784	0.411	2.4348E-09
Cnih1	1.1122E-13	0.99024892	0.288	0.099	2.44E-09
Akt1	1.1276E-13	0.87709222	0.307	0.109	2.4737E-09
Bsg	1.1462E-13	0.63187112	0.595	0.281	2.5147E-09
Uqcc3	1.1983E-13	0.69306623	0.392	0.151	2.629E-09
Dda1	1.2467E-13	0.73503216	0.399	0.158	2.7351E-09
Ercc6l	1.2814E-13	2.19543553	0.072	0.01	2.8113E-09
Nr2c2	1.317E-13	0.55931244	0.418	0.163	2.8894E-09
Dcun1d5	1.3183E-13	0.75434435	0.582	0.279	2.8921E-09
Shmt2	1.3225E-13	1.29838346	0.163	0.041	2.9014E-09
Psmd14	1.3483E-13	0.83238259	0.425	0.176	2.9581E-09
Tubgcp2	1.358E-13	1.46744159	0.163	0.042	2.9793E-09
Rpl15	1.449E-13	0.44220719	0.993	0.917	3.1791E-09
Arhgap33	1.4731E-13	3.13712585	0.026	0.002	3.2318E-09
Pomp	1.4755E-13	0.59900408	0.81	0.434	3.2372E-09
Xcl1	1.4937E-13	0.26058868	0.222	0.066	3.277E-09
Tbc1d31	1.5859E-13	1.71036962	0.105	0.02	3.4793E-09
Aimp1	1.6178E-13	0.71151207	0.614	0.302	3.5493E-09
Gpsm3	1.7054E-13	0.61223621	0.516	0.227	3.7415E-09
Mad2l2	1.7077E-13	1.64729496	0.137	0.032	3.7466E-09
Serpinb6a	1.708E-13	0.62683958	0.281	0.093	3.7471E-09
Sdf2l1	1.726E-13	0.92118219	0.373	0.147	3.7866E-09
Exog	1.7724E-13	2.74253035	0.039	0.003	3.8885E-09
Vars	1.8398E-13	0.79809992	0.32	0.116	4.0364E-09
Mctp2	1.8542E-13	1.12212406	0.176	0.046	4.0679E-09

Psmd2	1.9237E-13	0.57963594	0.484	0.206	4.2205E-09
Zyg11b	1.9353E-13	0.58013053	0.327	0.115	4.2457E-09
Tab1	1.9659E-13	2.20633029	0.059	0.007	4.313E-09
Fam76b	1.9763E-13	0.53912986	0.34	0.122	4.3358E-09
Srrt	2.0309E-13	0.90700487	0.353	0.137	4.4555E-09
Clns1a	2.0794E-13	0.88550216	0.275	0.092	4.562E-09
Chic2	2.0795E-13	0.63931929	0.595	0.282	4.5623E-09
Hspd1	2.1127E-13	0.44110499	0.667	0.333	4.635E-09
Olfr1238	2.1287E-13	4.44626089	0.013	0	4.6701E-09
Gm44174	2.147E-13	3.65823796	0.013	0	4.7104E-09
Lin54	2.1913E-13	0.59864904	0.458	0.193	4.8075E-09
Gmds	2.2165E-13	1.26283179	0.157	0.039	4.8628E-09
Dcp2	2.2245E-13	0.96870333	0.327	0.121	4.8804E-09
Rac1	2.2699E-13	0.62750317	0.882	0.53	4.98E-09
Ppp1cc	2.2969E-13	0.41647968	0.778	0.396	5.0391E-09
Ppp1r18	2.3033E-13	0.72599885	0.464	0.196	5.0532E-09
Coa3	2.4408E-13	0.78168684	0.333	0.124	5.3549E-09
Polr2h	2.4492E-13	1.04333771	0.248	0.081	5.3732E-09
Zfpm1	2.4561E-13	1.2203639	0.15	0.036	5.3883E-09
Tmem2	2.4671E-13	1.19354164	0.196	0.056	5.4125E-09
Ugcg	2.5203E-13	0.31468921	0.634	0.294	5.5292E-09
Dera	2.5895E-13	1.22478957	0.209	0.062	5.6812E-09
Anapc16	2.6053E-13	0.81785913	0.327	0.12	5.7157E-09
Eif3i	2.6129E-13	0.69625566	0.647	0.323	5.7325E-09
Eno1	2.6299E-13	0.62819127	0.523	0.233	5.7697E-09
Med19	2.6604E-13	1.12066441	0.203	0.059	5.8367E-09
Mrpl9	2.7129E-13	0.67033593	0.438	0.179	5.9518E-09
Polq	2.8314E-13	3.35042252	0.033	0.003	6.2118E-09
Vdac3	2.8881E-13	0.79585931	0.386	0.155	6.3362E-09
Cd2bp2	2.9633E-13	0.80546177	0.327	0.12	6.5013E-09
Fis1	3.0078E-13	0.59989719	0.719	0.374	6.5987E-09
Prpf18	3.0231E-13	0.67152667	0.359	0.136	6.6324E-09
Batf	3.1405E-13	0.6455299	0.438	0.181	6.89E-09
Cct6b	3.1898E-13	4.04853173	0.02	0.001	6.9981E-09
Pdcd4	3.1992E-13	0.44394897	0.68	0.321	7.0188E-09
Mrpl21	3.2399E-13	0.73163456	0.418	0.173	7.1081E-09
Taf12	3.4058E-13	1.31448876	0.203	0.06	7.4719E-09
Wbp11	3.6493E-13	0.61071414	0.641	0.314	8.0062E-09
Nup37	3.6889E-13	2.1714462	0.065	0.009	8.0932E-09
Ube2l3	3.7659E-13	0.64158912	0.706	0.369	8.2621E-09
Rpia	3.8771E-13	0.80549694	0.209	0.061	8.506E-09
Ubr7	3.9053E-13	1.04051075	0.209	0.062	8.5679E-09
Cycs	4.0398E-13	0.63578892	0.673	0.341	8.863E-09
Polr2k	4.0538E-13	0.67575389	0.556	0.261	8.8936E-09
Tmem107	4.311E-13	2.11390808	0.072	0.011	9.4578E-09
Csrp2	4.3523E-13	1.60021158	0.131	0.03	9.5486E-09
Csf1	4.7806E-13	0.62867177	0.15	0.037	1.0488E-08
1110004F10Ri	4.8656E-13	0.59459702	0.641	0.314	1.0675E-08

Ctr9	5.1132E-13	0.83139293	0.268	0.09	1.1218E-08
Fam96b	5.1861E-13	0.80173113	0.471	0.209	1.1378E-08
Coro1c	5.2638E-13	0.79447147	0.333	0.125	1.1548E-08
Dnaja1	5.6016E-13	0.36462628	0.915	0.57	1.2289E-08
Pdcd6	5.6062E-13	0.62017579	0.575	0.27	1.2299E-08
Taf6l	5.7341E-13	0.67223764	0.451	0.19	1.258E-08
Vps16	5.9225E-13	0.95633689	0.203	0.059	1.2993E-08
Sh3glb2	5.9252E-13	1.05806379	0.163	0.042	1.2999E-08
Sp4	5.9365E-13	0.50120471	0.346	0.126	1.3024E-08
Clta	6.0941E-13	0.57323227	0.882	0.503	1.337E-08
Cep295	6.1354E-13	0.95671903	0.229	0.071	1.346E-08
Zmynd8	6.2685E-13	0.67266109	0.301	0.105	1.3752E-08
Cep83	6.4057E-13	0.68129265	0.327	0.121	1.4054E-08
Xrn2	6.7084E-13	0.64697777	0.627	0.309	1.4718E-08
Fbxl14	6.7587E-13	0.85852632	0.203	0.059	1.4828E-08
Atg101	6.9101E-13	0.9060098	0.248	0.081	1.516E-08
Limd2	6.9199E-13	0.4495807	0.673	0.318	1.5182E-08
Hypk	7.0067E-13	0.65443883	0.405	0.164	1.5372E-08
Rhof	7.0348E-13	0.7192084	0.275	0.093	1.5434E-08
Zcwpw1	7.1003E-13	1.89323203	0.078	0.013	1.5577E-08
Pknox1	7.2725E-13	1.15210723	0.222	0.069	1.5955E-08
Irf2	7.4082E-13	0.54452423	0.569	0.261	1.6253E-08
Cd27	7.6472E-13	0.60314255	0.307	0.109	1.6777E-08
Pip4k2c	7.6593E-13	0.85863315	0.229	0.071	1.6804E-08
C2cd5	8.3603E-13	0.90577245	0.176	0.048	1.8342E-08
Timm13	8.4004E-13	0.76652693	0.647	0.341	1.843E-08
U2af2	8.6552E-13	0.4575491	0.503	0.218	1.8989E-08
Zfp524	8.7897E-13	1.64721321	0.15	0.038	1.9284E-08
Ikzf1	8.8433E-13	0.53168197	0.503	0.22	1.9401E-08
Abcf1	9.0394E-13	0.63082846	0.542	0.25	1.9831E-08
Rnf31	9.0867E-13	1.13601054	0.176	0.048	1.9935E-08
Ctc1	9.2264E-13	1.45375214	0.111	0.023	2.0242E-08
Timm17b	9.3985E-13	0.96584214	0.314	0.118	2.0619E-08
Rfxap	9.4139E-13	0.93842316	0.281	0.099	2.0653E-08
Bora	9.4545E-13	1.63395008	0.105	0.021	2.0742E-08
Oard1	9.4915E-13	0.70506335	0.222	0.068	2.0823E-08
Sap18	9.7195E-13	0.62809636	0.484	0.214	2.1324E-08
Cggbp1	1.0028E-12	0.50981607	0.732	0.376	2.2E-08
Tmem80	1.0198E-12	1.26842954	0.131	0.03	2.2374E-08
Cox6a1	1.0241E-12	0.7101795	0.804	0.516	2.2467E-08
Mdm1	1.0301E-12	1.84895394	0.065	0.009	2.26E-08
Atp2b4	1.0464E-12	0.31111618	0.484	0.197	2.2957E-08
Mrpl4	1.0475E-12	0.84857588	0.301	0.109	2.298E-08
Lage3	1.0563E-12	1.29882543	0.196	0.058	2.3174E-08
Agpat3	1.0688E-12	0.97230022	0.275	0.096	2.3448E-08
Cntrob	1.0882E-12	1.34594293	0.092	0.017	2.3874E-08
Mien1	1.096E-12	0.6703482	0.549	0.258	2.4046E-08
Rbm4b	1.1381E-12	0.89747831	0.242	0.078	2.4968E-08

Eif3d	1.1568E-12	0.69154278	0.484	0.217	2.5378E-08
Crlf2	1.1718E-12	0.8088699	0.497	0.233	2.5707E-08
Itgb3bp	1.1723E-12	1.73069365	0.118	0.025	2.5719E-08
Vcpkmt	1.1777E-12	0.83647198	0.229	0.071	2.5839E-08
Prkag1	1.252E-12	1.11895466	0.19	0.055	2.7467E-08
Pop4	1.3056E-12	1.00582042	0.209	0.063	2.8643E-08
Gm6277	1.3359E-12	2.50880921	0.039	0.004	2.9308E-08
Ing2	1.343E-12	0.79198206	0.373	0.15	2.9465E-08
Dynlrb1	1.3601E-12	0.59921869	0.582	0.277	2.9838E-08
Upf3a	1.3767E-12	0.9505011	0.333	0.13	3.0204E-08
Rrp1	1.3832E-12	0.55007325	0.549	0.251	3.0345E-08
Naxe	1.4831E-12	1.07482599	0.235	0.077	3.2538E-08
Pop7	1.5465E-12	1.03507834	0.275	0.098	3.3928E-08
Jakmip1	1.6198E-12	0.60991646	0.373	0.142	3.5536E-08
Phf14	1.6321E-12	0.58291039	0.359	0.138	3.5807E-08
Bak1	1.649E-12	0.97691171	0.268	0.094	3.6176E-08
Slc25a11	1.6533E-12	0.91174468	0.255	0.086	3.6273E-08
Timm22	1.6811E-12	1.02035125	0.209	0.064	3.6881E-08
Cct8	1.6907E-12	0.68599219	0.569	0.278	3.7093E-08
Rpl18	1.698E-12	0.34326482	1	0.961	3.7252E-08
Zcrb1	1.7024E-12	0.64126601	0.601	0.3	3.7349E-08
Hmgn1	1.7051E-12	0.52417257	0.477	0.21	3.7408E-08
Ltb	1.7058E-12	0.44298703	0.601	0.275	3.7423E-08
Ptpn11	1.7599E-12	0.77379935	0.353	0.139	3.8611E-08
Nudcd2	1.7695E-12	0.99356161	0.229	0.073	3.8821E-08
Eif2d	1.8025E-12	1.14507417	0.157	0.04	3.9545E-08
Cog6	1.8309E-12	1.36107048	0.137	0.033	4.0168E-08
Zcchc9	1.8327E-12	0.66424549	0.412	0.171	4.0208E-08
Stk32c	1.8991E-12	1.68275499	0.105	0.021	4.1664E-08
Rab11a	1.9637E-12	0.52989914	0.49	0.216	4.3083E-08
Med9	2.0089E-12	1.49908458	0.144	0.036	4.4073E-08
Hnrnpr	2.0282E-12	0.61503742	0.51	0.23	4.4497E-08
Cdk2ap1	2.0547E-12	0.76776081	0.307	0.113	4.5077E-08
Atp5e	2.0702E-12	0.45188395	0.993	0.819	4.5417E-08
Znhit3	2.1276E-12	1.09319898	0.137	0.033	4.6677E-08
Ralbp1	2.2134E-12	0.64836713	0.503	0.231	4.856E-08
Ppp3cb	2.3444E-12	0.72353926	0.399	0.164	5.1434E-08
Tcrg-C2	2.4595E-12	1.89664241	0.072	0.011	5.396E-08
Gm44175	2.5035E-12	2.26375791	0.039	0.004	5.4924E-08
Elovl1	2.5183E-12	0.91182616	0.235	0.077	5.5249E-08
Fdx1	2.535E-12	0.72977414	0.32	0.12	5.5616E-08
Map2k3	2.6389E-12	0.51229872	0.562	0.26	5.7895E-08
2410015M20F	2.6581E-12	0.50165824	0.634	0.304	5.8317E-08
Agfg1	2.6699E-12	0.48791909	0.444	0.187	5.8575E-08
Borcs8	2.953E-12	0.72691902	0.333	0.129	6.4785E-08
Nosip	2.9768E-12	1.07885327	0.19	0.055	6.5308E-08
Impdh2	3.1473E-12	0.82956494	0.444	0.199	6.9048E-08
Stk19	3.1572E-12	0.79069017	0.301	0.111	6.9265E-08

Ripk3	3.2097E-12	1.59415633	0.144	0.037	7.0418E-08
Lyrm9	3.2448E-12	1.40480865	0.124	0.028	7.1187E-08
Mtch1	3.2915E-12	1.02599843	0.288	0.107	7.2212E-08
Hmbs	3.5466E-12	1.66312693	0.111	0.024	7.7809E-08
Rad23a	3.5607E-12	0.96948053	0.268	0.096	7.8118E-08
Haus5	3.5901E-12	1.65093485	0.078	0.013	7.8764E-08
Fxyd7	3.6652E-12	2.88059294	0.026	0.002	8.041E-08
Nabp2	3.7355E-12	0.86314233	0.209	0.064	8.1954E-08
Kin	3.9379E-12	0.78419229	0.275	0.097	8.6394E-08
Cyld	3.957E-12	0.39308724	0.66	0.316	8.6812E-08
Zfp763	4.0162E-12	2.40235153	0.033	0.003	8.8112E-08
Trav5-4	4.2E-12	4.20464141	0.02	0.001	9.2143E-08
Ubash3a	4.2514E-12	1.11386295	0.176	0.049	9.3272E-08
Pdpk1	4.2999E-12	0.82042264	0.327	0.126	9.4336E-08
Rab24	4.434E-12	0.84032458	0.288	0.105	9.7277E-08
Cox16	4.4432E-12	0.80821417	0.229	0.074	9.748E-08
Ntmt1	4.5035E-12	1.73387286	0.105	0.022	9.8801E-08
Rbl2	4.5862E-12	0.6351428	0.366	0.144	1.0062E-07
Grb7	4.7194E-12	1.13476053	0.124	0.028	1.0354E-07
Hdac3	4.7273E-12	1.33326468	0.157	0.042	1.0371E-07
Mrps23	4.7826E-12	0.70264793	0.346	0.136	1.0493E-07
Iah1	4.8606E-12	1.24655949	0.216	0.071	1.0664E-07
Suds3	4.8747E-12	0.66635148	0.307	0.114	1.0695E-07
Emsy	4.9573E-12	0.42509692	0.464	0.196	1.0876E-07
Atp5a1	5.0464E-12	0.53476107	0.843	0.513	1.1071E-07
Arhgap9	5.0931E-12	0.88251773	0.275	0.098	1.1174E-07
Pole4	5.2105E-12	0.56824245	0.542	0.252	1.1431E-07
Psmd6	5.2578E-12	0.71210585	0.346	0.137	1.1535E-07
Prpf19	5.4407E-12	1.20367119	0.222	0.073	1.1936E-07
Slc30a9	5.5502E-12	0.80788931	0.248	0.084	1.2177E-07
Ddx20	5.5821E-12	1.15172498	0.124	0.029	1.2246E-07
C230035I16Ri	5.5943E-12	4.35770673	0.013	0	1.2273E-07
Slc2a3	5.597E-12	0.61593425	0.196	0.058	1.2279E-07
Higd2a	5.6035E-12	0.64107577	0.536	0.252	1.2294E-07
Zfp683	5.6424E-12	4.06638388	0.013	0	1.2379E-07
Mapre1	5.6487E-12	0.56733568	0.699	0.368	1.2393E-07
Gramd1b	5.6516E-12	0.6803073	0.248	0.083	1.2399E-07
Rnf121	5.6587E-12	1.0363177	0.17	0.047	1.2415E-07
Cuta	5.6696E-12	0.76801608	0.458	0.21	1.2438E-07
Mta1	6.0473E-12	0.95373622	0.229	0.075	1.3267E-07
Ppat	6.3434E-12	1.25370991	0.163	0.045	1.3917E-07
Derl2	6.3727E-12	0.71517763	0.346	0.138	1.3981E-07
Hnrnpf	6.4069E-12	0.54693283	0.915	0.593	1.4056E-07
Psmg2	6.428E-12	1.0770115	0.15	0.039	1.4102E-07
Psmc5	6.4692E-12	0.7875569	0.399	0.171	1.4193E-07
A430033K04R	6.5565E-12	1.97953508	0.052	0.007	1.4384E-07
Cyfip2	6.8104E-12	0.4918478	0.503	0.224	1.4941E-07
Cmip	7.0612E-12	0.67738623	0.569	0.281	1.5492E-07

Cyba	7.0629E-12	0.42998525	0.941	0.627	1.5495E-07
Mepce	7.2473E-12	0.46176064	0.32	0.118	1.59E-07
Capzb	7.2875E-12	0.5582455	0.856	0.545	1.5988E-07
Urod	7.3706E-12	1.30952853	0.131	0.032	1.617E-07
Miip	7.372E-12	1.32191974	0.118	0.027	1.6174E-07
Atg3	7.4582E-12	0.76933732	0.373	0.156	1.6362E-07
Ppp2r2d	7.5679E-12	0.59130008	0.477	0.215	1.6603E-07
Marc2	7.6893E-12	0.74728921	0.366	0.152	1.687E-07
Mrpl46	7.7112E-12	0.94004679	0.229	0.076	1.6918E-07
Gmps	8.0461E-12	0.55448482	0.353	0.139	1.7652E-07
Ctnnbip1	8.0789E-12	1.37655988	0.163	0.046	1.7724E-07
Sec61b	8.2706E-12	0.46933241	0.928	0.626	1.8145E-07
Lnpk	8.3316E-12	0.74540183	0.248	0.084	1.8279E-07
Madd	8.3597E-12	0.30416017	0.536	0.236	1.834E-07
Cops5	8.3625E-12	0.53349183	0.392	0.161	1.8347E-07
Cdc5l	8.549E-12	0.54554248	0.536	0.25	1.8756E-07
Eif6	8.5994E-12	0.6631458	0.477	0.216	1.8866E-07
Psma6	8.6329E-12	0.61818116	0.51	0.237	1.894E-07
Ap2m1	8.6355E-12	0.61318081	0.477	0.216	1.8945E-07
Glrx5	8.7053E-12	0.75259861	0.333	0.132	1.9099E-07
Cmtm4	8.8263E-12	1.79471955	0.085	0.016	1.9364E-07
Sf3a1	9.0482E-12	0.6965755	0.281	0.101	1.9851E-07
Carhsp1	9.2196E-12	0.97912121	0.229	0.076	2.0227E-07
Trappc10	9.2772E-12	0.95328023	0.301	0.115	2.0353E-07
Flna	9.4627E-12	0.6013559	0.562	0.275	2.076E-07
Mtmr1	9.4889E-12	0.83284269	0.229	0.075	2.0818E-07
Dok2	9.6402E-12	0.83244462	0.248	0.085	2.115E-07
Nup35	9.9394E-12	1.48833517	0.085	0.016	2.1806E-07
Chrac1	1.0004E-11	0.64294129	0.425	0.184	2.1949E-07
Nmt1	1.0119E-11	0.68238202	0.601	0.309	2.2199E-07
Ubn1	1.0595E-11	0.55345005	0.556	0.265	2.3245E-07
Fmnl1	1.0842E-11	0.61369056	0.471	0.215	2.3787E-07
4930412M03F	1.0843E-11	3.28459735	0.02	0.001	2.379E-07
Cenpt	1.0959E-11	2.01219444	0.065	0.01	2.4044E-07
Ss18l2	1.1017E-11	0.98608168	0.19	0.057	2.417E-07
Tm9sf1	1.1189E-11	1.39979138	0.098	0.02	2.4548E-07
Glmn	1.1355E-11	1.8204924	0.085	0.016	2.4911E-07
Lman2	1.1393E-11	0.59319652	0.575	0.281	2.4995E-07
Tmem258	1.1417E-11	0.64414538	0.686	0.384	2.5047E-07
Rbm10	1.1497E-11	1.02904405	0.176	0.051	2.5223E-07
1110020A21R	1.173E-11	2.956841	0.033	0.003	2.5734E-07
1700048O20R	1.1805E-11	2.36565422	0.046	0.005	2.5899E-07
Khsrp	1.1949E-11	0.74697931	0.333	0.132	2.6215E-07
Apip	1.2017E-11	1.40286415	0.157	0.044	2.6364E-07
Prpf8	1.2819E-11	0.56653272	0.464	0.207	2.8123E-07
Eif2s2	1.3804E-11	0.46598864	0.948	0.677	3.0285E-07
Actr1a	1.3893E-11	0.93640446	0.261	0.094	3.048E-07
Fkbp1a	1.4019E-11	0.63531488	0.588	0.291	3.0756E-07

Smarcd1	1.4056E-11	0.99418443	0.222	0.073	3.0838E-07
Ndufb2	1.4246E-11	0.86827629	0.458	0.215	3.1255E-07
Fam81a	1.4314E-11	4.31105978	0.013	0	3.1404E-07
Nrf1	1.4784E-11	0.55611483	0.242	0.081	3.2434E-07
[truncated: 390,319 more chars]
