# Supplementary material for: PINK1 deficiency rewires early immune responses in a mouse model of Parkinson’s disease triggered by intestinal infection
Source: NPJ Parkinsons Dis. 2025 May 22;11:133. doi: 10.1038/s41531-025-00945-w (PMC12098848; doi:10.1038/s41531-025-00945-w)
Supplement: Supplementary file 1 — Supplementary Figures and legends [file 41531_2025_945_MOESM1_ESM.pdf]

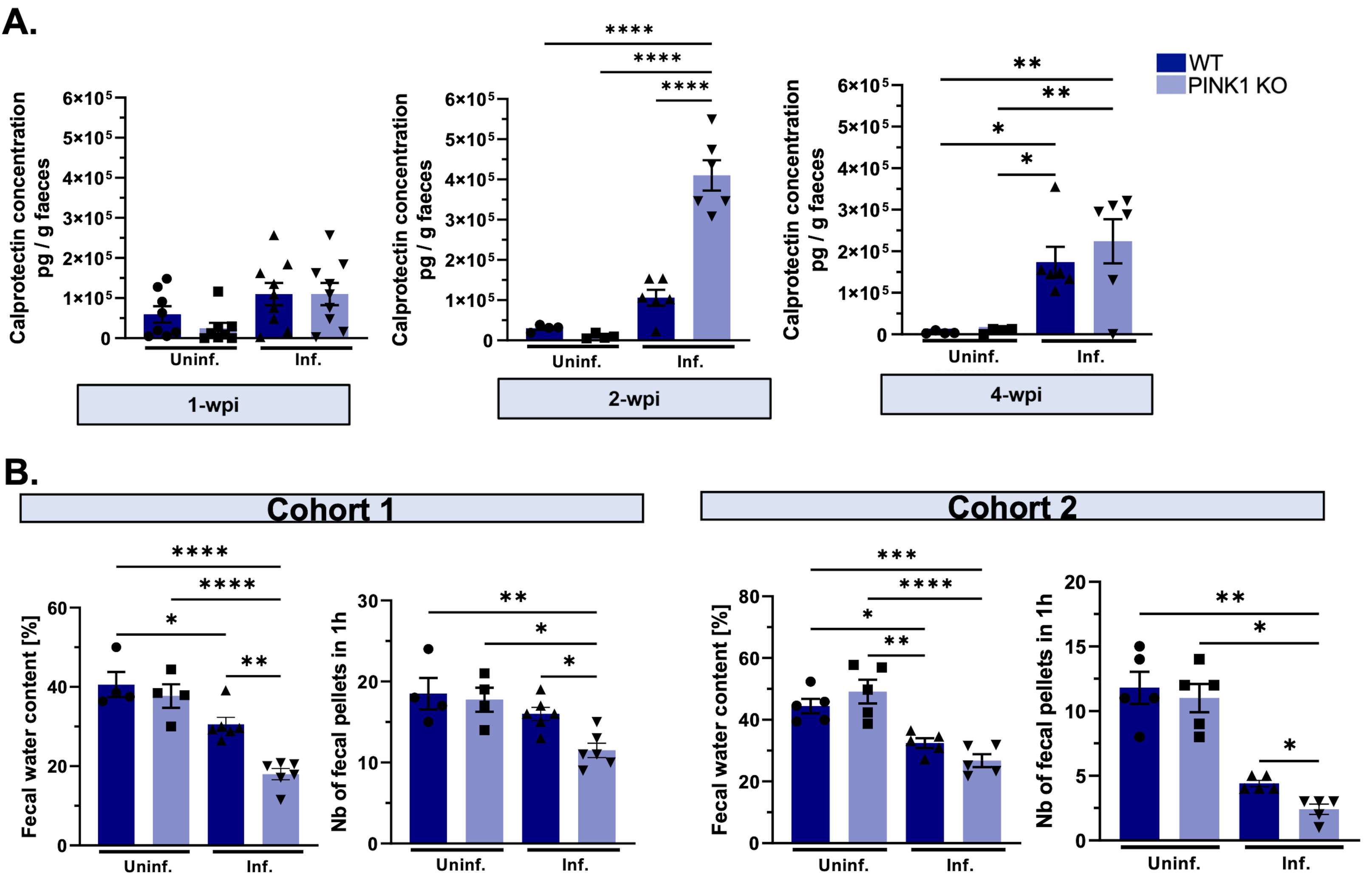

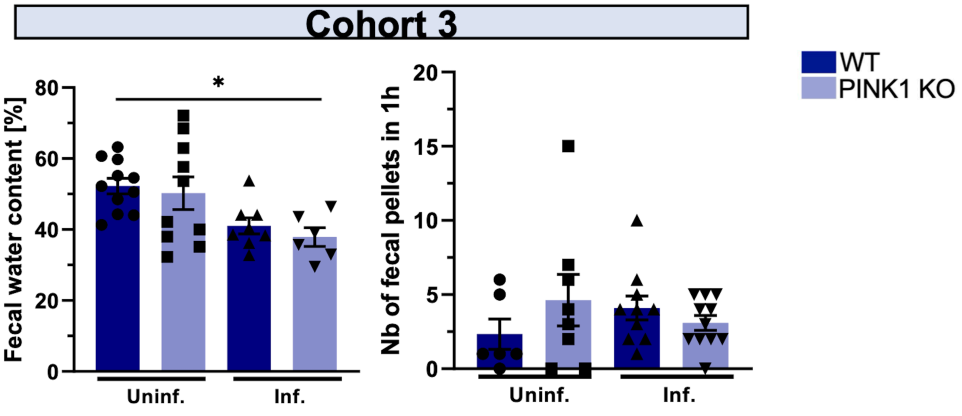

Supplementary Figure 1: PINK1 KO mice after *C. rodentium* infection show evidence of PD-like, GI-related dysfunctions

A) Calprotectin levels were measured in fecal samples of WT and PINK1 KO mice at 1-, 2- and 4- w.p.i. and uninf. controls. One-way ANOVA followed by Tukey's multiple comparison test was applied. Mean ± SEM,n=4-9 mice per group. B) Constipation phenotype following infection in WT and PINK1 KO mice was assessed by quantifying the percent of water content in stools and number of fecal pellet deposited per hour over the course of 3 hrs. Shown are measurements across three independent cohorts. One-way ANOVA followed by Tukey's multiple comparison test was applied. Mean ± SEM,n=4-11 mice per group.

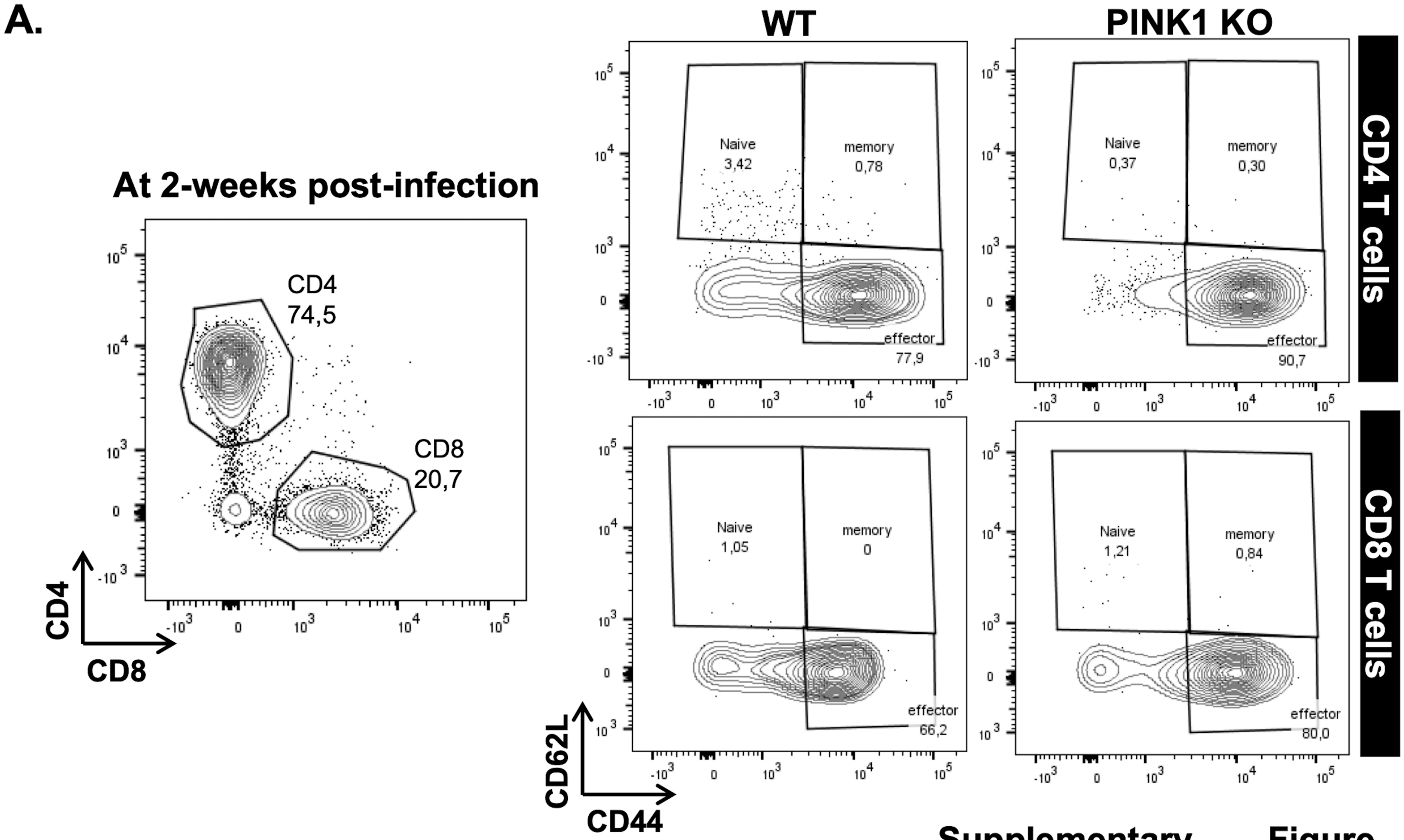

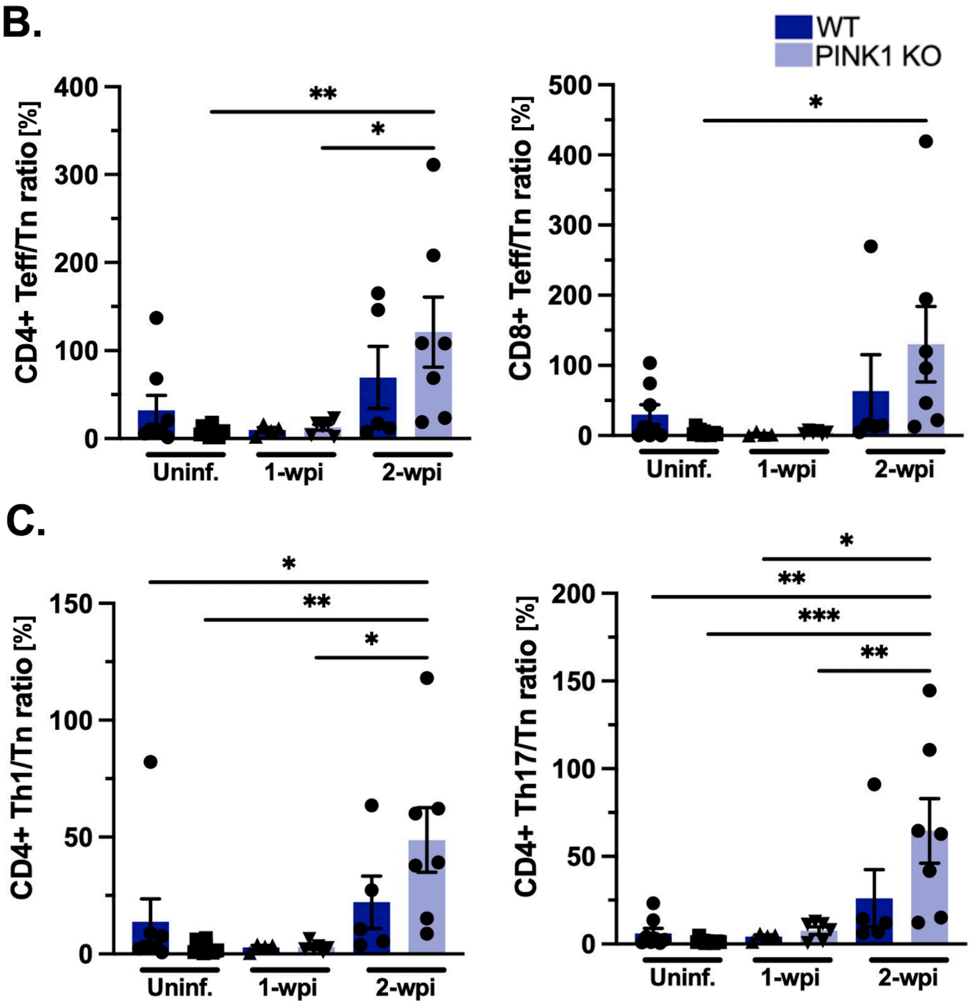

Supplementary Figure 2: Absence of PINK1 leads to higher ratios of colonic effector-to-naïve CD4 and CD8 T cells at 2-weeks-post-infection

A) Representative gating strategy for isolating naïve and effector CD4+ and CD8+ T cell subtypes in the colonic lamina propria via flow cytometry. B) The ratios of effector-to-naïve T cells (Teff/Tn) were assessed across all groups. Naïve and effector T cells are defined as $CD44^{low}CD62L^{high}$ and $CD44^{high}CD62L^{low}$, respectively. C) The frequencies of Th1 and Th17 cells were also determined, and cell subpopulations were defined as $Tbet^{+}$ and $Ror\gamma t^{+}$, respectively. Two-way ANOVA followed by Tukey's multiple comparison test was applied. Mean ± SEM, n=4-12 mice per group.

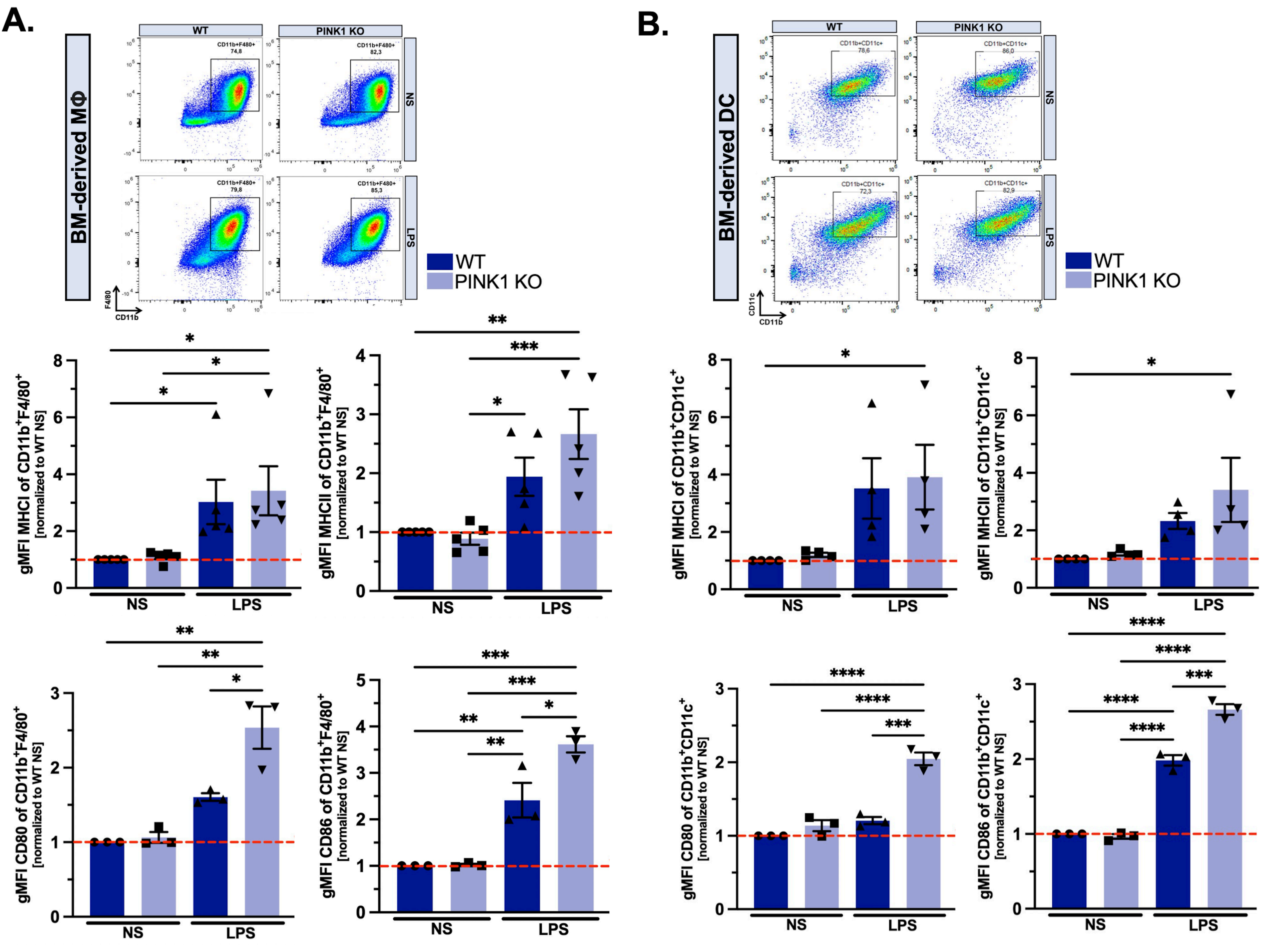

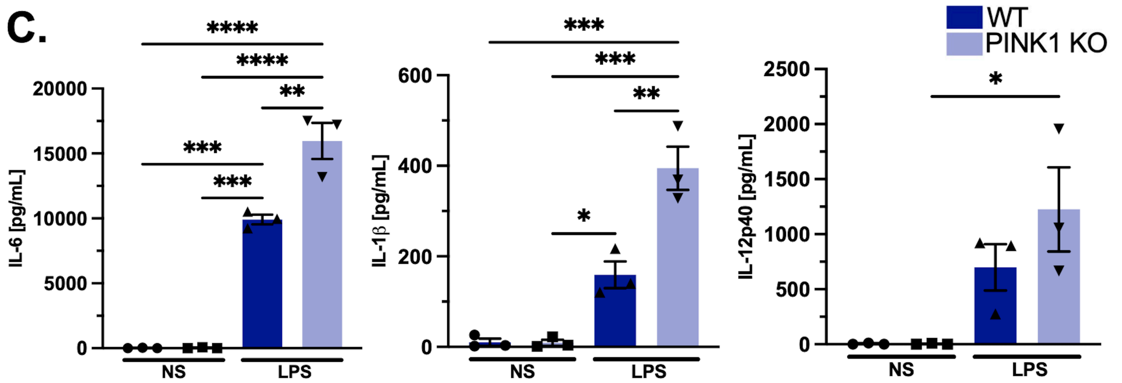

Supplementary Figure 3: LPS-treated PINK1 KO mature myeloid cells display upregulated antigen presentation markers congruent with elevated secretion of T-cells-polarizing cytokines

A, B) Subsequent to culturing of bone marrow (BM)-derived macrophages (Mϕ) and dendritic cells (DCs) from WT and PINK1 KO mice, mature myeloid cells were treated with either lipopolysaccharide (LPS) or PBS control (NS) for 24 h. Supernatant were collected, and cells harvested for immunophenotyping. Representative flow cytometric graph of BM-derived Mϕ and DCs defined as $CD11b^{+}F4/80^{+}$ and $CD11b^{+}CD11c^{+}$, respectively. Within each population, geometric mean fluorescent intensity of proteins related to antigen presentation were evaluated, including major histocompatibility markers I and II (MHCI/II) and co-stimulatory ligands (CD80 and CD86). C) Collected supernatant from stimulated BM-derived Mϕ was assessed for secreted T-cell polarizing proinflammatory cytokines using ELISA, which includes IL-6, IL-1β and IL-12p40. Two-way ANOVA followed by Tukey's multiple comparison test was applied. Mean ± SEM, n=3-4 mice per group.

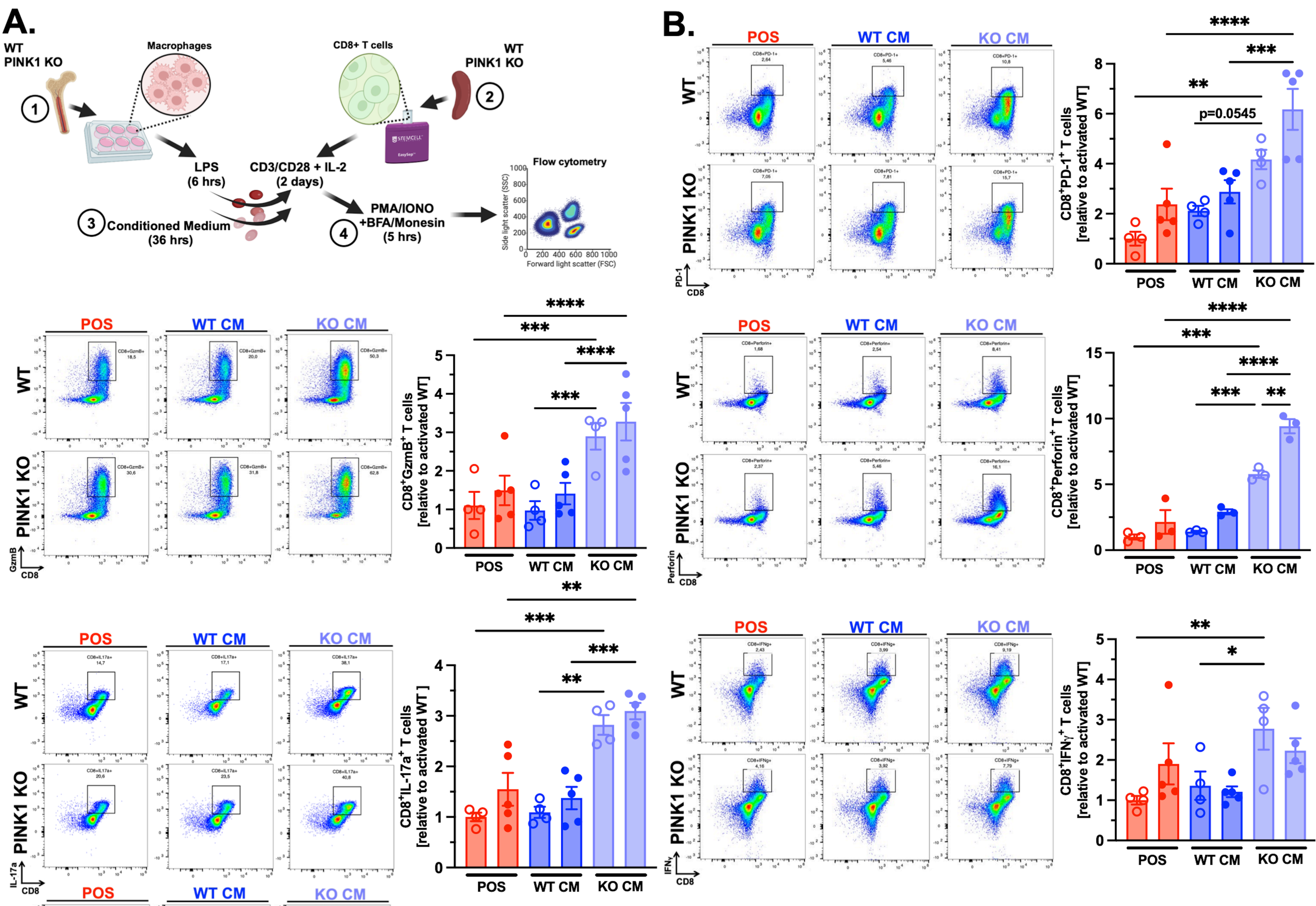

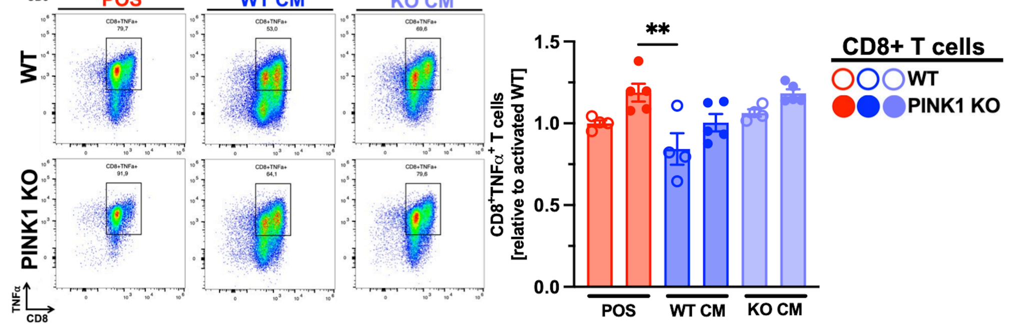

Supplementary Figure 4: Absence of PINK1 in myeloid cells following stimulation is sufficient to induce the activation of cytotoxic T cells

A) Schematic representation of experimental design: BM-derived macrophages from WT and PINK1 KO mice were cultured for 7 days in the presence of M-CSF. After maturation, cells were stimulated with LPS for 6 hrs and conditioned medium (CM) was collected. CD8+ T cells were purified from spleens of WT and PINK1 KO mice using magnetic beads. T cells were activated either with anti-CD3/CD28 alone (POS) or in combination with CM from WT or PINK1 KO BMDM for two days in the presence of IL-2. Following activation, cells were harvested and re-stimulated with PMA/ionomycin with protein transport inhibitors (Brefeldin A, BFA and monesin) for 5 hrs. CD8+ T cell activation was assessed by flow cytometry. Data is displayed as a representative flow cytometric analysis of CD8+ T cells expressing either PD-1, GzmB, Perforin, IL-17a, IFNγ or TNFα in response to following treatments. Shown is the quantification of the relative frequency of activated CD8+ T cells normalized to T cells from WT mice stimulated with anti-CD3/CD28 antibody alone. Two-way ANOVA followed by Tukey's multiple comparison test was applied. Mean ± SEM, n=4 mice per group.

Supplementary Table 1: Full list of markers for each cell type annotated in Fig. 1D.

Supplementary Table 2: Full list of GO terms and associated genes dysregulated in PINK1 KO noted in Fig. 3C, D and Fig. 4D, E
